# Multiomic Analyses Reveal Brainstem Metabolic Changes in a Mouse Model of Dravet Syndrome

**DOI:** 10.3390/cells15010067

**Published:** 2025-12-30

**Authors:** Ashwini Sri Hari, Alexandria M. Chan, Audrey Scholl, Aidan Mulligan, Janint Camacho, Ireland Rose Kearns, Gustavo Vasquez Opazo, Jenna Cheminant, Teresa Musci, Min-Jee Goh, Alessandro Venosa, Philip J. Moos, Martin Golkowski, Cameron S. Metcalf

**Affiliations:** 1 Epilepsy Therapy Screening Program (ETSP) Contract Site, University of Utah, Salt Lake City, UT 84112, USA; ashwini.srihari@pharm.utah.edu (A.S.H.); u1352171@utah.edu (J.C.); ireland.kearns@utah.edu (I.R.K.); gustavo.vasquez@hsc.utah.edu (G.V.O.); 2 Department of Pharmacology and Toxicology, University of Utah, Salt Lake City, UT 84112, USA; alexandria.carlson@pharm.utah.edu (A.M.C.); u1296125@utah.edu (A.S.); aidanmulligan@sccstudents.org (A.M.); jenna.cheminant@pharm.utah.edu (J.C.); teresa.musci@hsc.utah.edu (T.M.); min-jee.goh@pharm.utah.edu (M.-J.G.); alessandro.venosa@pharm.utah.edu (A.V.); philip.moos@pharm.utah.edu (P.J.M.); martin.golkowski@utah.edu (M.G.); 3 Huntsman Cancer Institute, University of Utah, Salt Lake City, UT 84112, USA

**Keywords:** Dravet Syndrome, sudden unexpected death in epilepsy (SUDEP), brainstem, metabolism

## Abstract

**Highlights:**

- There are widespread metabolic changes in the brainstem of *Scn1a^A1783V^^/WT^* HET mice.
- Metabolomic analyses reveal age-specific metabolic alterations in the brainstem of HET mice.
- Several druggable protein kinases are altered in the brainstem of HET mice.
- The findings of this study suggest a role of metabolic alterations in the brainstem as a plausible contributor to SUDEP pathogenesis

**Abstract:**

Dravet Syndrome (DS) is a severe genetic epileptic encephalopathy caused by mutations in the *SCN1A* gene that encodes the voltage-gated sodium channel (Na_V_1.1) subunit alpha. DS is characterized by intractable seizures, progressive developmental delay, cognitive impairment, and high mortality due to sudden unexpected death in epilepsy (SUDEP). SUDEP is mediated by respiratory dysfunction, but the exact molecular underpinnings are unclear. Though hippocampal metabolic alterations have been reported in DS mice, such changes in brain regions controlling breathing have not been studied. We used *Scn1a^A1783V^*^/*WT*^ DS mice to study temporal alterations in the brain metabolome, including analysis of brainstem and forebrain regions. Glycolytic and pentose phosphate pathway intermediates were significantly elevated in the brainstem of DS mice during the period of enhanced susceptibility to mortality (post-natal days P20–30). In older P40–P50 mice, mitochondrial aconitate and the antioxidant glutathione were significantly elevated in the brainstem. Single-nuclei RNA sequencing (snRNA seq) and proteomic analyses revealed alterations in genes associated with neurotransmission, cellular respiration, and protein translation, as well as reorganization of protein kinase-mediated pathways that are specific to the brainstem. These findings suggest that there are widespread metabolic changes in the brainstem of DS mice.

## 1. Introduction

Epilepsy is the fourth most common neurological disorder, affecting more than 65 million people worldwide [1]. Dravet Syndrome (DS) is a catastrophic childhood developmental epileptic encephalopathy that is caused mostly (~70–80%) by de novo mutations in the *SCN1A* gene [2]. This gene encodes the voltage-gated sodium channel subunit α (Na_V_1.1), which is highly expressed throughout the central nervous system [3]. Clinical manifestations include febrile and afebrile intractable seizures, developmental delay, sleep disturbances, cognitive impairment, psychomotor dysfunction, and a high mortality rate (15–20%), mainly due to sudden unexpected death in epilepsy (SUDEP) and status epilepticus (SE) [4,5,6].

Preclinical and patient data suggest respiratory distress and cardiac dysfunction preceded by post-ictal apnea as a leading cause of SUDEP [7,8,9]. The brainstem is a central regulator of critical autonomic functions like respiration, cardiac function, and arousal [10,11]. Cortical or limbic seizure activity-mediated spreading depolarization to the brainstem can suppress synaptic activity, thereby disrupting cardiorespiratory functions [12,13]. Moreover, animal studies have shown that mutations in the *SCN1A* gene lowered the threshold for spreading depolarization propagation to the brainstem [14]. Similarly, repeated seizure spread has been shown to cause metabolic/energy exhaustion, post-ictal hypoperfusion and hypoxia, disruption of ionic gradients, and impairment of neurotransmission, which in turn can beget seizures [15,16].

An overall increase in glycolysis (hypermetabolism) during the ictal phase and a drastic decrease in glycolytic rates (hypometabolism) during the inter-ictal phase, as well as diminished functionality of the tricarboxylic acid cycle (TCA cycle), impairment of mitochondrial oxidative phosphorylation, and increased formation of reactive oxygen/nitrogen species, are critical signatures of metabolic perturbations in epileptic foci [17,18,19,20]. Early evidence of metabolic perturbations in DS comes from a forebrain/hippocampal metabolomic analysis in the *Scn1a^A1783V^*^/*WT*^ DS mouse model that showed alterations in the glutamate/γ-aminobutyric acid (GABA) cycle, glycolysis, and the TCA cycle [21]. However, no study thus far has assessed metabolic alterations in subcortical regions like the brainstem in the DS population. Whether such alterations occur in the brainstem and potentially impact seizure, morbidity, and mortality outcomes in DS is largely unknown.

We utilized the *Scn1a^A1783V^*^/*WT*^ DS mouse model that carries a global, knock-in, missense, loss-of-function mutation in the *SCN1A* gene that occurs in DS patients [22,23,24,25]. Heterozygous (HET) *Scn1a^A1783V^*^/*WT*^ mice remarkably reproduce DS clinical features, including pharmacoresistant hyperthermia-induced and spontaneous seizures, behavioral/cognitive comorbidities, and a high mortality rate (~50%) during post-natal day P20–30 [26,27,28]. This suggests a critical period wherein brainstem respiratory dysfunction may contribute to an increase in mortality risk. To better understand whether changes in overall brainstem homeostasis are altered, we evaluated brainstem metabolism in *Scn1a^A1783V^*^/*WT*^ DS mice.

In this study, we performed untargeted metabolomic analysis at two developmental periods: P20–30 (critical period of enhanced susceptibility to mortality) and P40–50 (survival past the critical period). We also performed a preliminary exploration of the brainstem transcriptome in HET mice. Furthermore, to complement transcriptomic data and identify putative druggable targets to modulate epilepsy phenotypes, we performed mass spectrometry (MS)-based chemoproteomic kinome activity profiling [29,30,31]. Findings from this study will shed light on the role of the brainstem in DS etiology and pave the way forward for understanding if and how metabolic alterations in this region could potentially influence morbidity, mortality/SUDEP, and seizure outcomes in DS. Furthermore, these findings will help identify druggable targets that will have implications for the mechanistic understanding and treatment of debilitating comorbidities in DS.

## 2. Materials and Methods

### 2.1. Animals

All animal care and experimental procedures were approved by the Institutional Animal Care and Use Committee (IACUC) of the University of Utah (IACUC protocol IDs: 00002093 (approval date: 29 December 2022) and 19-12014 (approval date: 2 January 2020)). Experimental animals were generated by breeding a floxed stop male *Scn1a^A1783V^* (B6(Cg)-*Scn1a^tm1.1Dsf^*/J, Jax #026133) with a *Sox2-cre* (B6. Cg-*Edil3 ^Tg(Sox2−cre)1Amc^*/J) female mouse to produce both heterozygous (*Scn1a^A1783V^*^/*WT*^; HET) offspring maintained in a C57BL/6J background and wild-type (WT) offspring [26]. Genotypes were confirmed by obtaining tail clips under anesthesia, followed by a polymerase chain reaction using Transnetyx’s (Memphis, TN, USA) automated genotyping system. Both male and female HET mice, along with age-matched WTs, were used for experiments from ages P20–50. Mice were group-housed (5/cage) in a pathogen-free facility under a 12 h light/12 h dark light cycle and had ad libitum access to food and water except during hyperthermia testing, when they were transferred to the experimental room at least 30 min before testing. For our studies, both male and female mice (wild-type [WT] or heterozygous [HET]) were used. Since the goal of our study was to understand potential metabolic alterations in the brain of HET mice compared to wild-type mice at baseline, randomization to different groups was not required and was not performed. For the assays, the brainstem and forebrain were utilized. After euthanasia, the whole brain was dissected out on dry ice. The brainstem and forebrain regions were separated, placed in 5 mL tubes, and stored at −80 °C for use in future assays. Precautions were taken to use different collection tools to separate the brainstem and forebrain tissues for collection and storage in order to prevent cross-contamination of tissues.

### 2.2. Untargeted Metabolomic Analysis

Untargeted metabolomic analysis was performed at the Metabolomics Core Facility at the University of Utah. Two studies were performed:

*Study I*: To investigate baseline differences in the forebrain, brainstem, and plasma metabolomes of HETs compared to WTs, P20–30 mice were euthanized, blood was collected in blood collection tubes (BD Vacutainer ^TM^ with dipotassium ethylene diamine tetra acetic acid (K2 EDTA), Thermo Fisher Scientific, Waltham, MA, USA) and centrifuged at 6000 rpm for 10 min to isolate plasma. Whole brains were removed and separated into forebrain and brainstem (after removing cerebellum) portions and stored at −80 °C. Plasma and weighed (40 mg) brain tissues were transferred to 2.0 mL ceramic bead mill tubes and stored until analysis via gas chromatography–mass spectrometry (GC-MS)

➢ *Analysis of mass spectrometry data:* Data were collected using MassHunter software version 12.0 (Agilent Technologies, Santa Clara, CA, USA). Metabolites were identified, and their peak area was recorded using MassHunter Quant. This data was transferred to an Excel spreadsheet (Microsoft, Redmond, WA, USA). Metabolite identity was established using a combination of an in-house metabolite library developed using pure purchased standards, the NIST library, and the Fiehn library. Data were then analyzed using the “MetaboAnalyst” software tool (https://www.metaboanalyst.ca/). The steps involved uploading "raw input" followed by normalization and % CV analysis, which helped remove “failing compounds” by testing for individual metabolite reliability. Briefly, all the integration values were normalized relative to a labeled internal standard. This is carried out to account for minor errors caused by extraction or injection differences. Quality control (QC) samples are created by combining an equal amount of all samples in the experiment. The QC sample is injected at regular intervals between samples. During the data analysis, QC was carried out to check two factors to ensure data integrity. The first was comparing the area under the curve of the QC to the area under the curve of the process blank. Anything less than 1.5 was removed from analysis. Data were normalized to a reference value, transformed by the square root, and finally scaled with Pareto scaling. All values were adjusted for False Discovery Rate (FDR). After normalization, QC was used to evaluate the consistency of the data. This was carried out by measuring the percent coefficient of variation (%CV) (100 × standard deviation/mean). Metabolites with a %CV > 30% were removed from analysis.

The report generated was then used for Metabolite Set Enrichment Analysis (MSEA) in MetaboAnalyst. Briefly, the .csv file was uploaded, and quantitative enrichment analysis was chosen, followed by ID standardization. Next, samples were normalized to individual tissue weights. Log transformation and Pareto scaling were chosen as options for data transformation and scaling, respectively. The Kyoto Encyclopedia of Genes and Genomes (KEGG) pathway was chosen for the metabolite set library, and an overview of the top 25 enriched metabolites, along with the enrichment ratio and associated *p*-values, was generated. To ascertain changes in each individual metabolite between HETs and WTs, an unpaired, parametric Student’s *t*-test was utilized.

*Study II*: To better understand the brainstem metabolome over time, we selected two different age groups (developmental time periods). P20–30 and P40–50 mice were euthanized, and brainstem tissue was collected and stored at −80 °C. An amount of 40 mg of each tissue was weighed out, transferred to 2.0 mL ceramic bead mill tubes, and submitted on dry ice to the Metabolomics Core Facility, where metabolites were analyzed by liquid chromatography–mass spectrometry (LC-MS)

➢ *Analysis of mass spectrometry data:* Data was collected using SCIEX analyst software. Chromatogram integration was performed using SCIEX MultiQuant. Statistical analysis was performed using the Microsoft Excel Data Analysis add-in. Metabolite identity was established using a combination of an in-house metabolite library developed using pure purchased standards and the METLIN library. Normalization and QC were performed as described above for GC-MS. Values were adjusted for FDR. The report generated was then used for Metabolite Set Enrichment Analysis (MetaboAnalyst), and the steps described above were followed to generate the top 25 enriched metabolites along with the enrichment ratio, and then associated *p*-values were generated. To ascertain changes in each individual metabolite between HETs and WTs, an unpaired, parametric Student’s *t*-test was utilized.

### 2.3. Glucose Assay

Glucose levels in the forebrain and brainstem were assayed using a colorimetric assay. P20–30 HET and age-matched WT mice were euthanized using carbon dioxide (CO_2_). Forebrain and brainstem tissue were collected and stored at −80 °C. The tissues were then suspended in lysis buffer consisting of 1X ice-cold phosphate-buffered saline (PBS), 1 protease, and 1 phosphatase tablets (Pierce A32963 and A32957, Thermo Fisher Scientific, Waltham, MA, USA). The tissue was then homogenized by probe sonication (~31% amplitude, QSonica sonicator, Newtown, CT, USA), followed by centrifugation (accuSpin Micro 17R, Fisher Scientific, Hampton, NH, USA) at 13,000 revolutions per minute (rpm) for 10 min at 4 °C. The supernatant was collected, diluted (1:10 brainstem, 1:20 forebrain), and used for subsequent glucose colorimetric and protein assays. Glucose levels were assessed using the Cayman glucose colorimetric assay kit (10009582, Ann Arbor, MI, USA) and normalized to total protein measured by the Bradford assay (Pierce 23200). Briefly, glucose in samples is oxidized to delta-gluconolactone with concomitant reduction of the flavin adenine dinucleotide (FAD)-dependent enzyme glucose oxidase. Hydrogen peroxide (H_2_O_2_) is generated when the reduced form of glucose oxidase is regenerated back to its oxidized form in the presence of molecular oxygen (O_2_). H_2_O_2_ reacts with 3, 5-dichloro-2-hydroxybenzenesulfonic acid and 4-aminoantipyrine with horseradish peroxidase as a catalyst to generate a pink dye with optimal absorption at 514 nm measured using a spectrophotometer (SpectraMax 250, Molecular Devices, San Jose, CA, USA). A standard curve with R^2^ > 0.95 was generated for varying glucose concentrations from which the slope and intercept were identified. By inputting corrected optical density (O.D.) for Y, the concentration of glucose in each sample (X values) was calculated and normalized to protein concentration.

### 2.4. Glycogen Assay

Glycogen levels in forebrain and brainstem were assayed using a total glycogen colorimetric assay kit (MET-5022, Cell Biolabs Inc., San Diego, CA, USA). The forebrain and brainstem were collected and stored at −80 °C. The tissues were then suspended in lysis buffer (composition mentioned earlier). The tissue was then homogenized by probe sonication (~31% amplitude), followed by centrifugation at 13,000 rpm for 10 min at 4 °C. The supernatant was collected and used for a subsequent glycogen assay. The assay involves breaking down glycogen in samples to glucose moieties, which are then oxidized by glucose oxidase to D-gluconic acid and H_2_O_2_. HRP then catalyzes the reaction between H_2_O_2_ and a colorimetric probe, which binds in a 1:1 ratio. The resulting product is measured spectrophotometrically at 540–570 nm. A standard curve with R^2^ > 0.95 for varying glycogen concentrations was generated from which the slope and intercept were obtained. By substituting corrected O.D. values for Y, the concentration of glycogen in the sample (X values) was obtained.

### 2.5. Hexokinase (HK) Assay

HK activity was assessed in forebrain and brainstem tissues using a colorimetric assay kit (ab136957, Abcam, Cambridge, UK). P20–30 and P40–50 HETs and age-matched WTs were euthanized using CO_2_. Forebrain and brainstem tissues were collected and stored at −80 °C. The tissues were suspended in lysis buffer, homogenized by sonication, and centrifuged. The collected supernatant was used for assaying HK with the following dilutions: 1:150 (forebrain) and 1:100 (brainstem). The assay involves the oxidation of glucose to glucose-6-phosphate (G6P) by HK in the sample, which generates nicotinamide adenine dinucleotide reduced (NADH) in the process. NADH then reduces a colorless probe to a colored probe with an absorption maximum at 450 nm at room temperature. A standard curve with R^2^ > 0.95 was generated from which the NADH concentration in the samples was determined. Using the equation (B/∆T × V) × D, where B = NADH concentration, ∆T = linear phase reaction time (T_2_ − T_1_), V = sample volume, and D = sample dilution, the activity of HK was calculated. One unit of hexokinase is the amount of enzyme that will generate 1.0 μmol of NADH per min at pH 8 at room temperature.

### 2.6. Glucose-6-phosphate Dehydrogenase (G6PD) Assay

G6PD activity was assessed in forebrain and brainstem tissues using a colorimetric assay kit (ab102529, Abcam, Cambridge, UK). P20–30 and P40–50 HETs and age-matched WTs were euthanized using CO_2_. Forebrain and brainstem tissues were collected and stored at −80 °C. These tissues were suspended in lysis buffer, homogenized by sonication, and centrifuged. The collected supernatant was used for assaying G6PD with the following dilutions: 1:25 (forebrain) and 1:20 (brainstem). The assay involves the oxidation of G6P in the sample to 6-phosphoglucono-delta-lactone by G6PD, which leads to the reduction of the coenzyme nicotinamide adenine dinucleotide (NAD) to NADH. This is the first, and rate-limiting, step in the pentose phosphate pathway. NADH is formed and then converts a colorless probe into a colored product with an absorption maximum at 450 nm at 37 °C, and the color intensity is proportional to G6PD activity in the sample. A standard curve with R^2^ > 0.95 was generated from which the NADH concentration in the samples was determined. Using the equation (B/∆T × V) × D, where B = NADH concentration, ∆T = linear phase reaction time (T_2_ − T_1_), V = sample volume, and D = sample dilution, the activity of G6PD was calculated. An amount of 1 Unit of bG6PD activity = the amount of G6PD that will generate 1.0 µmol of NADH per minute at 37 °C.

### 2.7. Hyperthermia Testing

P20–30 HETs and age-matched WTs were allocated into 4 different groups: WT + sham, WT + hyperthermia, HET + sham, and HET + hyperthermia, with each group having N = 5 animals. Animals were acclimated in the experimental procedure room for at least 30 min. For the start of the hyperthermia protocol, a neonatal rectal probe (RET-4, Braintree Scientific, MA, USA) coupled to a temperature controller (TCAT-2DF, Physitemp, Clifton, NJ, USA) was rectally inserted (depth < 1 inch), and the animal was allowed to acclimate for 5 min in the experimental chamber. The core body temperature of animals in the hyperthermia group was gradually (1 °C/2 min) increased until the occurrence of a generalized seizure (only observed in HETs), or until the temperature reached 42.5 °C [26]. Seizing mice were then transferred to a granite slab to allow them to cool down to physiological core body temperature and to halt seizures. WT and HET sham animals did not receive hyperthermia but underwent all other steps as the hyperthermia group.

### 2.8. Protein Assay

Sample protein concentration was measured using the Bradford protein assay (Pierce 23200, Thermo Fisher Scientific, Waltham, MA, USA). This assay involves the binding of the acidic Coomassie dye reagent to proteins in the sample, which then induces a color shift from brown to blue that is measured at 595 nm using a spectrophotometer. A standard curve with R^2^ > 0.95 for increasing concentrations of bovine serum albumin (BSA) was obtained from which the slope and intercept were identified. By inputting corrected O.D. values for Y, the corresponding X values (protein concentration, ug/mL) were obtained.

### 2.9. Immunohistochemistry (IHC) for ΔFosB

Mice were euthanized with isoflurane and transcardially perfused with 0.1 M phosphate-buffered saline (PBS), followed by 4% paraformaldehyde (PFA) solution. Brains were removed, post-fixed in 4% PFA for 24 h, and then stored in 30% sucrose solution for cryoprotection. Tissue was briefly frozen at −20 °C before sectioning on a freezing stage microtome (Leica Biosystems, Deer Park, IL, USA) at 30 µm thickness. Sections were stored in 0.05% sodium azide in 0.1 M PBS at 4 °C.

Brainstem sections were mounted onto glass slides and washed 3 times in 0.1 M PBS, and then incubated in blocking solution (0.1 M PBS, 0.05% sodium azide, 3% Triton X-100, 5% normal donkey serum) containing the primary antibody for deltaFosB (ΔFosB) (rabbit anti-ΔFosB, Abcam ab184938, 1:1000) overnight at 4 °C. Following primary antibody incubation, sections were washed 3 times in 0.1 M PBS, and then incubated in blocking solution containing secondary antibodies (goat anti-rabbit Alexa Fluor 488, Jackson Immuno 111-545-144, 1:1000) for 2 h at room temperature. Sections were then incubated in DAPI (1 ug/mL) to stain for cell nuclei, rinsed 3 times in 0.1 M PBS, and coverslipped with Prolong Gold (Thermo Fisher P36930). Images were captured with a Zeiss FV-1000 confocal microscope using a 20×/0.75NA water objective. Regions of interest were identified using the DAPI channel. Once identified, 10 z-stack images (1 µm thick) were imaged from both sides of each section. An automated macro consisting of the following functions was created to analyze images in ImageJ (National Institutes of Health, Bethesda, MD, USA; version 1.53t, 2022): all raw images were converted to 8-bit, and the background was removed using rolling ball subtraction. All images were despeckled, and then a maximum intensity z-stack was created. Images were auto-thresholded using the Renyi Entropy algorithm. Cells that were at least 20 µm in diameter with 0.5–1 circularity within a consistent ROI containing the nuclei of interest were counted using the Analyze Particles function [32].

### 2.10. Single-Nuclei Preparation and Transcriptomic Analysis

Single-nuclei RNA sequencing (snRNA seq) was performed for brainstem nuclei isolated from P40–50 HETs and age-matched WTs. In this study, the occurrence of a spontaneous seizure prior to euthanasia/tissue collection was not considered to be a required criterion. Mice were euthanized with Euthasol ^®^ (Virbac, Westlake, TX, USA) and intracardially perfused with 1× PBS. The whole brain was collected, and the brainstem was isolated and flash-frozen using liquid nitrogen and stored at −80 °C until further processing for nuclei extraction. Upon processing, specimens were thawed in ice-cold PBS, devoid of Ca^2+^ and Mg^2+^, supplemented with 0.54 μM Necrostatin-1 (#J65341, Thermo Fisher Scientific, Waltham, MA, USA), 1 μM HPN-07 (SML2163, Sigma-Aldrich), 0.32 μM sodium hydroxybutyrate (A1161314, Thermo Fisher Scientific), 78 nM Q-VD-Oph (SML0063, Sigma-Aldrich), and 0.2 U/mL of Ribolock RNase inhibitor (EO0381, Life Technologies Corporation, Carlsbad, CA, USA). The tissue was then minced and transferred into a 2 mL Dounce homogenizer containing nuclei extraction buffer (130-128-024, Miltenyi Biotec, Bergisch Gladbach, Germany) along with 0.2 U/mL Ribolock RNase inhibitor. After homogenization, samples were centrifuged at 600× *g* for 5 min, resuspended in 2% bovine serum albumin (BSA) containing PBS, and strained sequentially with 70 μm FLOWMI and 40 μm FLOWMI. This suspension was evaluated for purity, nuclei target recovery, and submitted for snRNA-seq using the 10× Chromium 3’ (v3.1 Next GEM) gene expression platform with 600 million reads/sample.

Library construction and sample sequencing were performed by the Huntsman Cancer Institute High Throughput Genomics Core. The 10× Genomics Cell Ranger Single Cell pipeline generated alignments and counts using the recommended parameters. Cell Ranger’s default 10 mm reference dataset was used for alignment. Seurat (4.1.0) was used for quality control and downstream analysis. Cells were filtered out when having fewer than 1000 genes and more than 15% mitochondrial genes. Genes filtered out included the following: (a) mitochondrial genes; (b) hemoglobin genes; and (c) bias-producing genes, including Gm42418, AY036118, Gm47283, Rpl26, Gstp1, Rpl35a, Erh, Slc25a5, Pgk1, Eno1, Tubb2a, Emc4, Scg5, Ehd2, Espl1, Jarid1d, Pnpla4, Rps4y1, Xist, Tsix, Eif2s3y, Ddx3y, Uty, Kdm5d, Cmss1, AY036118, and Gm47283. Data were processed, including dimension reduction and clustering by SC Transformation (0.3.5) using the Gamma–Poisson generalized linear model (glmGamPoi, 1.4.0) method. Two raw data files (WMB-10Xv3-P-raw.h5ad and WMB-10Xv3-MY-raw.h5ad) were downloaded from the Allen Institute and loaded into Seurat. After merging, metadata (cluster_id, class, subclass, and supertype) from GSE246717_WMB_sample_metadata.csv was added by matching cell barcodes, and Ensembl IDs were converted to gene symbols using biomaRt version 2.66.0. The data were processed with NormalizeData, FindVariableFeatures, and ScaleData. A subset of 100,000 cells underwent SketchData for PCA, neighbor detection, and SCTransformation, followed by clustering. AzimuthReference was used to generate ref.Rds and idx.annoy files, creating a custom Allen Institute reference. Single-nuclei data from this study were similarly processed and annotated using RunAzimuth, and then projected onto the custom Allen Institute UMAP.

Pathway and Gene Ontology analyses were performed with enrichR (3.1), and then heatmaps were generated. *p*-values associated with heatmaps were obtained using Fisher’s exact test, followed by the Benjamini–Hochberg multiple testing correction for obtaining the adjusted *p*-values. The scale represents the log (adjusted *p*-value) with a cutoff of 1 × 10^−25^ for pathway heatmaps. For the top 40 globally upregulated genes, the cut-off for *p* value ranges from 2.0 × 10^−104^ to 7.578 × 10^−295^, and for the top 40 globally downregulated genes, the cut-off for *p* value ranges from 2.1 × 10^−165^ to 0 (anything < 1 × 10^−300^ is set to 0).

### 2.11. Mass Spectrometry (MS)-Based Chemoproteomics

MS-based proteomics was performed according to our in-house-developed kinobead AP-MS method as previously described [29,30,31,33]. Briefly, frozen tissue samples were ground to a fine powder using a Cryomill (Retsch GmbH, Setzingen, Germany). Native protein was extracted from 50 mg of tissue powder using 50 mM Tris-HCl, 150 mM NaCl, 1% Nonidet P40 Substitute (*v*/*v*) (Sigma), 0.25% sodium deoxycholate (*w*/*v*), 1 mM EDTA, 10 mM NaF, and 5% glycerol (*v*/*v*) (pH 7.8), containing HALT protease and phosphatase inhibitor cocktail (100×, Thermo Fisher Scientific, Waltham, MA, USA) and 1 mM phenylmethylsulfonyl fluoride (PMSF). Extraction was performed by rotating samples on an end-over-end rotator for 30 min at 4 °C. Then, lysates were vortexed five times intermittently at max speed for 5 s each while being kept on ice and lysates clarified by centrifugation (21,000× *g*, 20 min, 4 °C). Protein content was determined using the Pierce 660 nm assay (Thermo), protein concentrations were adjusted to 4 mg/mL using modified RIPA buffer, and 1 mg of total protein was used for each kinobead pulldown. All other steps, including bead cleanup, protein digestion, peptide extraction, and LC-MS analyses, were performed using the diaPASEF method on a nanoElute 2–timsTOF Pro 2 LC-MS system (Bruker, Bremen, Germany) with label-free quantification, as previously described [33,34]. MS raw files were computed using DIA-NN 2.0.1, as previously described [33,35]. Data quality control, normalization, and imputation, as well as differential expression analysis (DEA, two-tailed two-sample Student’s *t*-test, *p* < 0.1, N = 2 or 3, see Table S1), were performed in Perseus [36]. Gene set enrichment analysis was performed using the single-sample Gene Set Enrichment Analysis (ssGSEA) script in R and applying the KEGG gene set database [37,38]. Bubble graphs were plotted using OriginPro v2025. Venn diagrams were plotted using BioVenn [39]. Bruker MS raw files and DIA-NN output files were deposited in the MassIVE repository of the University of California, San Diego, and are freely available under the accession number MSV000098704.

### 2.12. Statistical Analysis

Data were analyzed using GraphPad Prism software version 10.3.0 (San Diego, CA, USA) unless otherwise noted. Data are represented as mean ± standard error of the mean (SEM), and statistical significance was defined as *p* < 0.05. Datasets were first tested for outliers using the GraphPad test for outliers. For comparisons between two groups (WT versus HET), an unpaired, parametric Student’s *t*-test was used. For comparisons among four groups, a two-way ANOVA with either Tukey’s or Sidak’s multiple comparison post hoc test was used.

## 3. Results

### 3.1. Study I: Metabolomic Analysis Reveals a Decrease in Tricarboxylic Acid (TCA) Cycle Metabolites in the Forebrain of P20–30 HETs

**Figure 1:**
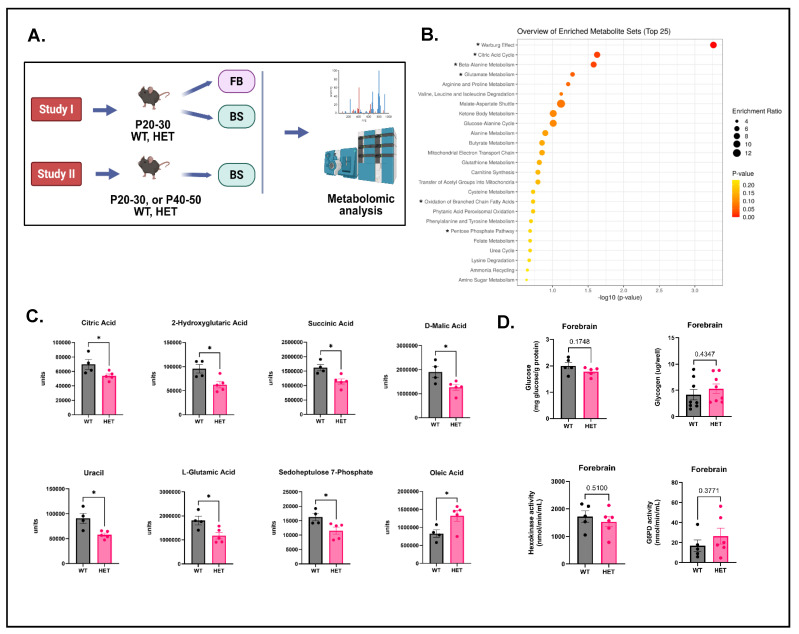
Forebrain (FB) metabolomic landscape of P20–30 HETs. Forebrain tissue was collected from P20–30 HET and age-matched WT mice for metabolomic analysis. Metabolite concentrations were normalized to tissue weight. (**A**) Schematic of the metabolomic analysis workflow. (**B**) KEGG pathway analysis showing the top 25 enriched pathways in P20–30 HET forebrain. Black-filled bars represent data from wildtype (WT) mice, and pink-filled bars represent data from *Scn1a^A1783V^*^/*WT*^ (HET) mice. (**C**) Metabolites that are significantly altered in the HET forebrain. Units on the Y-axis refer to "area under the curve" values. Tricarboxylic acid cycle cycle and pentose phosphate pathway metabolites are significantly decreased. (**D**) Levels of glucose, glycogen, and activities of HK and G6PD at baseline in the forebrain. Glucose and glycogen levels, and HK and G6PD enzyme activities, are insignificantly altered in the forebrain of HETs. Data are represented as mean ± SEM (error bars). For the KEGG pathway analysis in panel (**A**), *p*-values and enrichment ratios are represented within the image. The asterisk (*) in panel (**B**) highlights the pathways in which metabolites are significantly altered and shown in panel (**C**). For metabolites, * *p* < 0.05 versus WT by Student’s two-tailed unpaired, parametric *t*-test. N = 4–5/group (metabolomics), 5/group (glucose assay), 8/group (glycogen assay), and 5–6/group (HK and G6PD assays).

A previous study identified significant metabolic alterations in the hippocampi of *Scn1a^A1783V^*^/*WT*^ HET mice [21]. To determine whether metabolic alterations occurred in the forebrain of HET mice, this brain region was evaluated using untargeted metabolomic analysis (Figure 1A). The experimental design for all subsequent metabolomic analyses is also shown in Figure 1A. Interestingly, the Warburg effect and changes in the citric acid cycle emerged as the top two hits in the KEGG pathway analysis (Figure 1B). This was supported by changes observed in several key mitochondrial TCA cycle intermediates, including citric, 2-hydroxyglutaric, succinic, and malic acids that were significantly decreased in HETs compared to WTs (Figure 1C). The next set of hits revealed changes in beta-alanine, glutamate metabolism, and pentose phosphate pathways (Figure 1B). This was supported by a significant decrease in metabolites like uracil, L-glutamic acid, and sedoheptulose-7-phosphate in HETs (Figure 1C). Of note, the only metabolite that was significantly elevated in HETs was oleic acid. OA is involved in the transport of long-chain fatty acids and their oxidation, and can modulate mitochondrial function, and anti-inflammatory and antioxidant effects (Figure 1C). This was reflected in KEGG pathway analysis as oxidation of branched-chain fatty acids that emerged as one of the top hits (Figure 1B). These data suggest that mitochondrial energy metabolism in the forebrain of HETs may be potentially impaired.

To understand whether changes in basic cellular fuels accompanied the observed metabolic alterations in the forebrain, we evaluated baseline glucose and glycogen levels. No significant changes in glucose or glycogen levels were observed in HETs compared to WTs (Figure 1D). Since there were no changes in baseline glucose (primary fuel) levels, we determined if evoked seizures by hyperthermia treatment would alter glucose levels in the HET forebrain, as metabolic demand from seizures can deplete energy substrates like glucose [40]. Surprisingly, hyperthermia-induced seizures did not lead to increased glucose levels in the HET forebrain (Figure S1A). We further determined whether key enzyme activities were altered. Specifically, we measured the activities of HK and G6PD, which are the rate-limiting enzymes of glycolysis and PPP, respectively. Baseline HK and G6PD activities were unaltered in the HET forebrain compared to WTs (Figure 1D). To gain a preliminary understanding of potential brain-region-specific (forebrain versus brainstem) differences in the metabolic landscape in P20–30 HETs and WTs, we compared metabolites between these brain regions (Figure S2A). 2-Hydroxyglutaric acid, malic acid, sedoheptulose-7-phosphate, and inosine were significantly elevated in the HET brainstem compared to the HET forebrain. Oleic acid was significantly decreased in the HET brainstem compared to the HET forebrain. In WTs, stearic acid was significantly decreased in the brainstem compared to the forebrain (Figure S2A). These data suggest that brain-region-specific metabolic alterations could be more pronounced in HETs compared to WTs.

Circulating metabolites in plasma have been used as minimally invasive biomarkers to understand the pathogenesis of both acquired and genetic epilepsies, and response to anti-seizure medications (ASMs) [41]. In this study, we assessed the plasma metabolome to gain a better understanding of potential genotype-associated differences in metabolites. Briefly, we collected blood from P20–30 WTs and HETs, and then isolated the plasma and performed metabolomic analysis. Top enriched metabolites revealed by KEGG pathway analysis belonged to aspartate and glutamate metabolism, nicotinate and nicotinamide metabolism, citrate/TCA cycle, glutathione metabolism, and purine/pyrimidine metabolism (Figure S3A). This was supported by significant decreases observed in metabolites like L-glutamic and aspartic acids, nicotinamide 2, citric, fumaric, and D-malic acids, pyroglutamic acid, inosine, hypoxanthine, uracil, and orotic acid (Figure S3B) in HETs compared to WTs. Other significantly decreased metabolites in HETs were 4-hydroxyproline, L-methionine, lauric acid, β-glycerophosphoric acid, 1-octadecene, and myoinositol (Figure S3B). Taken together, these data suggest a potential depletion of certain metabolic intermediates involved in neurotransmission, energy production, and antioxidant replenishment.

### 3.2. Respiratory Nuclei Are Chronically Active in Several Brainstem Nuclei of HETs

**Figure 2:**
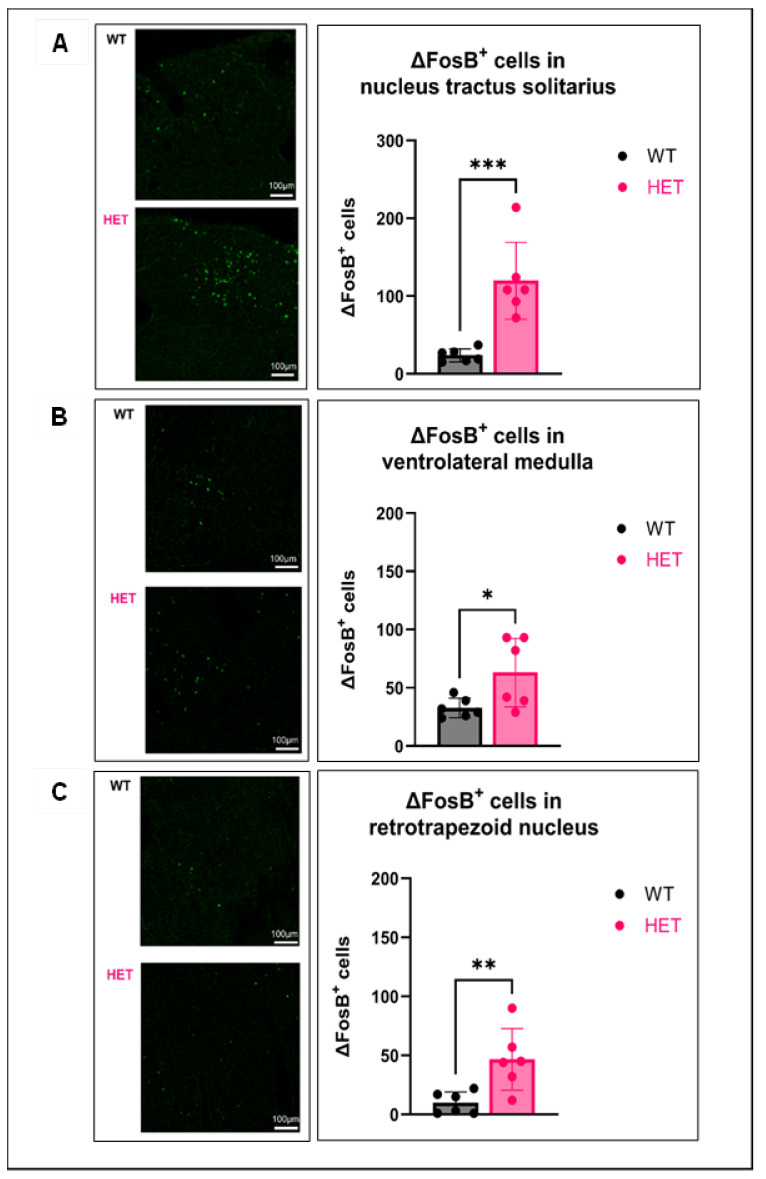
Key brainstem respiratory nuclei are chronically activated in P20–30 HETs. Brainstem tissue was collected from P20–30 HET and age-matched WT mice. ΔFosB levels were assessed by immunohistochemistry. ΔFosB staining in HET and WT brainstem tissue along with their corresponding quantification plots for the (**A**) NTS, (**B**) VLM, and (**C**) RTN. Data are represented as mean ± SEM (error bars). * *p* < 0.05, ** *p* < 0.01, and *** *p* < 0.001 versus WT by Student’s two-tailed unpaired *t*-test. N = 6/group.

Brainstem nuclei that are vital for regulating autonomic functions include the nucleus tractus solitarius (NTS), ventrolateral medulla (VLM), and the retrotrapezoid nucleus (RTN) [42,43,44]. The activity-dependent transcription factor ΔFosB is known to accumulate in regions of repeated neuronal activation and has been used as a marker of chronic neuronal activity [45,46,47]. Therefore, we used ΔFosB to determine whether key brainstem respiratory centers are chronically active in P20–30 HET mice using IHC. There was a significant increase in ΔFosB^+^ cells in all three brainstem nuclei of HETs compared to WTs (Figure 2A–C). This suggests that key brainstem nuclei are more frequently activated in HETs compared to WTs.

### 3.3. Metabolomic Analyses (Studies I and II) Reveal an Overall Increase in Glycolytic and Energy Metabolism in the Brainstem of P20–30 HETs

**Figure 3:**
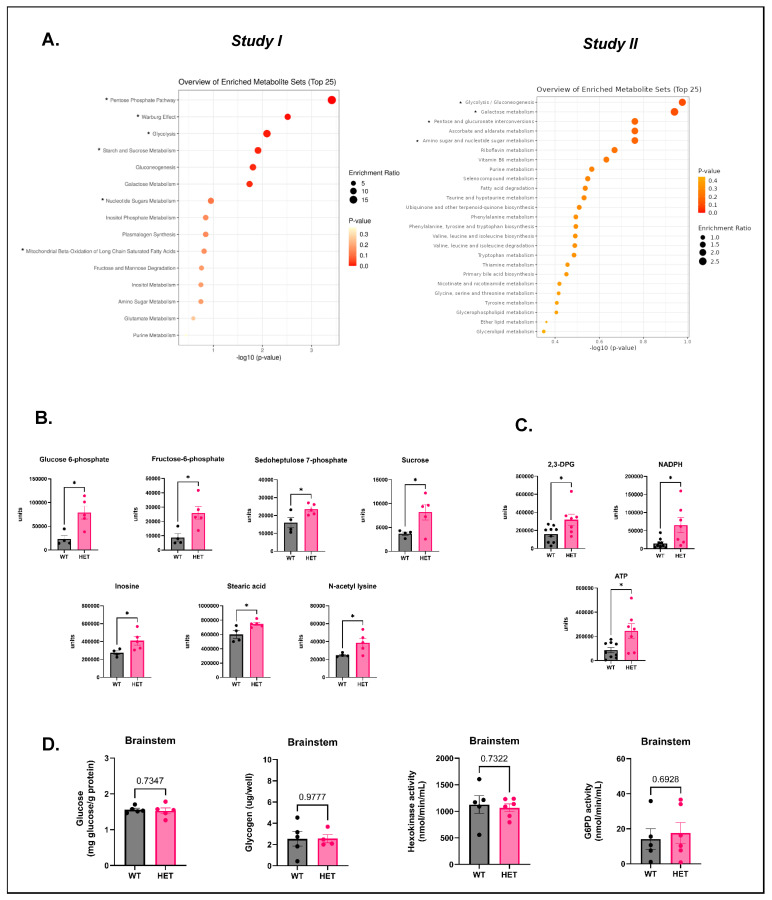
Brainstem (BS) metabolomic landscape of P20–30 HETs. Brainstem tissue was collected from P20–30 HET and age-matched WT mice for metabolomic analysis. Metabolite concentrations were normalized to tissue weight. (**A**) KEGG pathway analyses showing the top 25 enriched pathways in P20–30 HET brainstem, from studies I (left image) and II (right image). (**B**) Metabolites that are significantly altered in the HET brainstem from Study I (left panel) and (**C**) Study II (right panel). Units on the Y-axis refer to “area under the curve” values. Glycolytic intermediates and energy substrates are significantly increased. (**D**) Levels of glucose, glycogen, and activities of HK and G6PD in the brainstem at baseline. Glucose and glycogen levels, and HK and G6PD enzyme activities, are unaltered in the brainstem of HETs compared to WTs. Data are represented as mean ± SEM (error bars). For the KEGG pathway analyses in panel (**A**), *p*-values and enrichment ratios are represented within the image. The asterisk (*) in panel (**A**) highlights the pathways in which metabolites are significantly altered and shown in panel (**C**). For metabolites, * *p* < 0.05 versus WT by Student’s two-tailed unpaired *t*-test. N = 4–5/group (metabolomics, Study I), 7–9/group (metabolomics, Study II), 5/group (glucose assay), 4–5/group (glycogen assay), and 5–6/group (HK and G6PD assays).

Many mouse models of DS exhibit disordered breathing [10,48]. We investigated whether the brainstem metabolome was altered in the HET mice since the brainstem is involved in the control of critical autonomic functions like respiration. Brainstem tissue was collected from P20–30 HETs (Study I) along with age-matched WTs, and then weighed and submitted for untargeted metabolomic analysis. The pentose phosphate pathway, Warburg effect, and glycolysis emerged as the top three hits in KEGG pathway analysis (Figure 3A, left panel)). This was supported by significant increases in PPP, and glycolytic intermediates such as sedoheptulose-7-phosphate (pentose phosphate pathway), G6P, and fructose-6-phosphate (glycolysis), respectively (Figure 3B)). Other top hits included starch and sucrose metabolism, nucleotide sugar metabolism, and mitochondrial beta oxidation of long-chain saturated fatty acids (Figure 3B). This corresponded with increases observed in metabolites like sucrose, inosine, and stearic acid in HETs compared to WTs (Figure 3B)). A significant increase in N-acetyl lysine was also observed in the brainstem of HETs (Figure 3B)).

To verify the findings from our initial study, we conducted a secondary metabolomic analysis (Study II) to ascertain differences in the brainstem metabolome of HETs compared to WTs. Brainstem tissue was collected from P20–30 HETs and age-matched WTs, and then weighed and submitted for metabolomic analysis. The top hits in the KEGG analysis pathway were glycolysis/gluconeogenesis, galactose metabolism, pentose and glucuronate interconversions, and amino sugar and nucleotide sugar metabolism (Figure 3A, right panel)). This was supported by significant increases in metabolites like 2,3-diphosphoglyceric acid (2,3-DPG) (glycolysis), NADPH (pentose phosphate pathway), and adenosine triphosphate (ATP) in HETs compared to WTs (Figure 3C)). Data from studies I and II indicate a possible shift towards increased aerobic glycolysis, plausibly for energy production and enhanced reductive capacity for maintaining redox homeostasis in the HET brainstem.

Since we observed alterations in glycolytic and energy metabolism in the brainstem of P20–30 mice, we evaluated changes in basal fuel substrates such as glucose and glycogen. Baseline glucose and glycogen levels were unaltered in the HET brainstem compared to WTs (Figure 3D). Also, baseline HK and G6PD activities in the HET brainstem were insignificantly altered compared to the WT brainstem (Figure 3D). Next, we determined if hyperthermia-induced seizures would alter levels of glucose in the brainstem of HETs. Hyperthermia-induced seizures did not alter glucose levels in the brainstem of HETs compared to WTs that received hyperthermia treatment (Figure S4A). We evaluated brain-region (forebrain and brainstem) specific differences in baseline and post-seizure glucose levels and found no significant differences in HETs and WTs. Glucose levels remained unaltered in the WT brainstem both at baseline and post-hyperthermia treatment (Figure S4B). Next, we investigated whether there were brain-region-specific differences in baseline glycogen levels and observed no significant alterations in either HETs or WTs (Figure S4C). Since there were no changes in fuel substrates, we measured basal activities of HK and G6PD. There were no brain-region-specific changes in baseline activities of HK and G6PD in HETs and WTs (Figure S4D,E).

### 3.4. Glutathione (GSH) and Aconitate Are Increased in the Brainstem of P40–50 HETs

**Figure 4:**
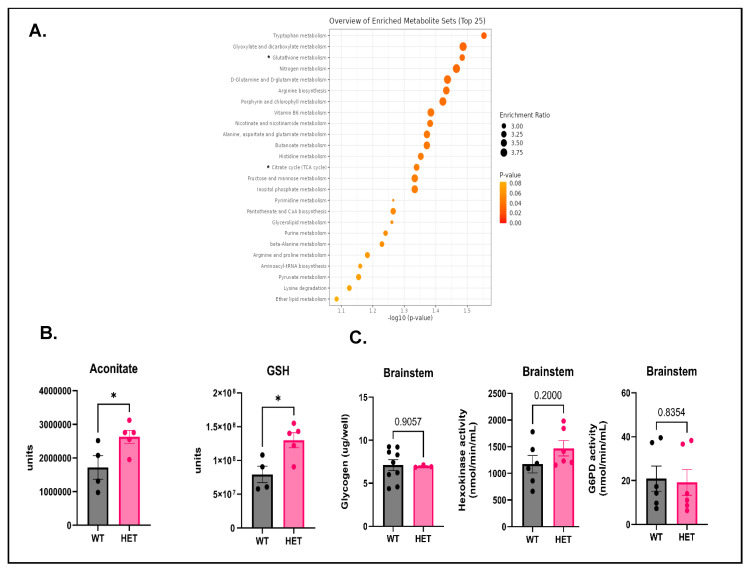
Brainstem (BS) metabolomic landscape of P40–50 HETs. Brainstem tissue was collected from P40–50 HET and age-matched WT mice for metabolomic analysis. Metabolite concentrations were normalized to tissue weight. (**A**) KEGG pathway analysis showing the top 25 enriched pathways in P40–50 HET brainstem. (**B**) Metabolites that are significantly altered in the HET brainstem. Units on the Y-axis refer to “area under the curve” values. TCA cycle intermediate (aconitate) and cellular antioxidant glutathione (reduced) are significantly elevated. (**C**) Glycogen levels and activities of HK and G6PD. Glycogen levels and HK and G6PD enzyme activities are unaltered at baseline in the brainstem of P40–50 HETs. Data are represented as mean ± SEM (error bars). For the KEGG pathway analysis in panel (**A**), *p*-values and enrichment ratios are represented within the image. The asterisk (*) in panel (**A**) highlights the pathways in which metabolites are significantly altered and shown in panel (**C**). For metabolites, * *p* < 0.05 versus WT by Student’s two-tailed unpaired *t*-test. N = 3–5/group (metabolomics), 3–9/group (glycogen assay), and 6/group (HK and G6PD assays).

To gain a better understanding of the brainstem metabolome in HETs that survived past the critical period or the period of enhanced susceptibility to mortality (P20–30), we performed untargeted metabolomics in P40–50 HETs. Brainstem samples were collected from P40–50 HETs along with age-matched WTs, weighed, and then submitted for metabolomic analysis. KEGG pathway analysis revealed GSH metabolism and the TCA cycle as some of the top hits (Figure 4A). This was corroborated by a significant increase observed in the TCA cycle intermediate, aconitate (Figure 4B), and the most abundant cellular antioxidant thiol reduced glutathione (GSH) (Figure 4B) in the brainstem of HETs compared to WTs. Glycogen levels and the activities of HK and G6PD at baseline were evaluated in the brainstem of P40–50 HETs, and no significant changes were observed in the HET brainstem compared to WTs (Figure 4C). Also, there were no brain-region (forebrain and brainstem) specific differences in glycogen, and HK and G6PD activities in P40–50 HETs (Figure S5A–C). Interestingly, the activity of HK was significantly lower in the brainstem compared to the forebrain in P40–50 WTs (Figure S5B). One explanation for this observation could be that the activity of HK has been shown to decrease with age in various tissue types, including different brain regions [49,50]. Taken together, these data suggest that there is preferential elevation of the non-protein antioxidant thiol GSH at P40–50, plausibly to maintain cellular redox homeostasis. In addition, this data suggests that there could be a possible increase in mitochondrial metabolism in the HET brainstem at this age.

### 3.5. Exploratory Single-Nuclei Sequencing (snRNA-seq) Unravels the Transcriptomes of P40–50 HETs and WTs

**Figure 5:**
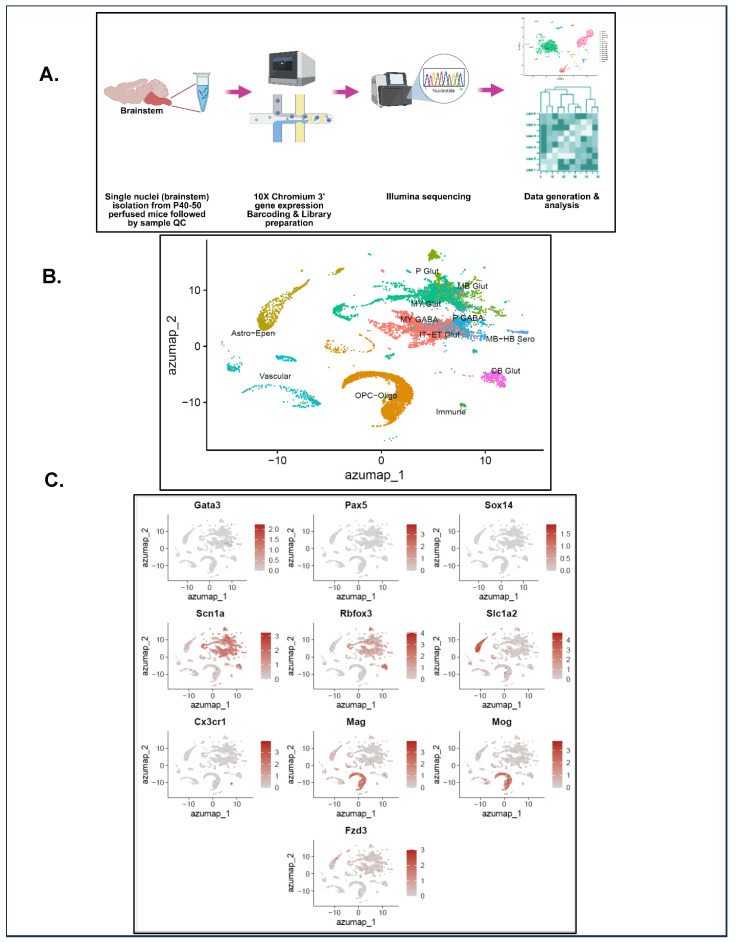
The brainstem transcriptomic landscape of P40–50 HETs and WTs. Brainstem tissue was collected from P40–50 HET and age-matched WT mice. Single-nuclei suspension of brainstem tissue was obtained by gradient physical dissociation and filtration. Exploratory snRNA-seq was performed using the 10X Chromium 3’ gene expression platform. Single-nuclei data were processed and annotated using RunAzimuth and then projected onto the custom Allen Institute Uniform Manifold Approximation and Projection (UMAP). (**A**) snRNA sequencing workflow. QC stands for quality control. (**B**) Azumap of annotated brainstem cell clusters. A total of 12 clusters were identified and include various (i) neuronal subtypes: mylencephalic glutamatergic (MY-Glut) and GABAergic (MY-GABA) neurons, pons glutamatergic (P-Glut), and GABAergic (P-GABA) neurons, midbrain glutamatergic neurons (MB-Glut), cerebellar glutamatergic neurons (CB-Glut), intratelencephalic and extratelencephalic projecting excitatory neurons (IT-ET Glut), and midbrain–hindbrain serotonergic neurons (MB-HB Sero); (ii) astrocytes–ependymal cells (Astro-Epen); (iii) oligodendrocyte precursor cells–oligodendrocytes (OPC-Oligo); (iv) immune cells; and (v) vascular cells. (**C**) Distribution of cell-type-specific markers across different cell clusters. These include markers that helped identify the brainstem (*Gata3*, *Pax5*, and *Sox14*), voltage-gated sodium channel (*Scn1a*), neurons (*Rbfox3*/*NeuN*), astrocytes (*Slc1a2*), oligodendrocytes (*Mag*/*Mog*), immune cells (*Cx3cr1*), and endothelial cells (*Fzd3*). N = 1/group.

HET mice exhibit a high rate of mortality during P20–30 [26]. HETs that survived past this "critical period" have a distinctly different brainstem metabolome compared to the P20–30 HETs (see results above). This led us to explore whether there were any changes in the brainstem transcriptome of HETs during P40–50. The workflow followed for snRNA seq is shown in Figure 5A.

Azumaps or Azimuth maps were generated using the Azimuth tool from the Allen Institute that projects single-cell or single-nuclei RNA-seq data onto a reference atlas (Allen Mouse Brain Atlas) to annotate cell types in that dataset. By utilizing this approach, 12 clusters (various cell types in the brainstem) were identified and include various (i) neuronal subtypes: mylencephalic glutamatergic (MY-Glut) and GABAergic (MY-GABA) neurons, pons glutamatergic (P-Glut), and GABAergic (P-GABA) neurons, midbrain glutamatergic neurons (MB-Glut), cerebellar glutamatergic neurons (CB-Glut), intratelencephalic and extratelencephalic projecting excitatory neurons (IT-ET Glut), and midbrain–hindbrain serotonergic neurons (MB-HB Sero); (ii) astrocytes–ependymal cells (Astro-Epen); (iii) oligodendrocyte precursor cells–oligodendrocytes (OPC-Oligo); (iv) immune cells (including microglia and lymphoid cells revealed by subclass annotation); and (v) vascular cells (Figure 5B). Comparison of brainstem cell clusters between HETs and WTs is represented in Supplementary Materials, Figure S6A. Individual cell-type-specific markers, along with their expression levels that helped in identifying the clusters, are represented in the Supplementary Materials, Figure S7A. The distribution of major cell-type-specific markers amongst the various clusters is represented in Figure 5C. These include markers that helped identify the brainstem (GATA binding protein 3 (*Gata3*), Paired Box 5 (*Pax5*), SRY-Box Transcription Factor 14 (*Sox14*)), voltage-gated sodium channel (Sodium channel alpha subunit (*Scn1a*)), neurons (RNA Binding Fox-1 Homolog-3 (*Rbfox3*)/Neuronal nuclei antigen (*NeuN*)), astrocytes (Solute carrier family 1 member 2 (*Slc1a2*)), oligodendrocytes (myelin-associated glycoprotein (*Mag*), myelin oligodendrocyte glycoprotein (*Mog*)), immune cells (C-X3-C motif chemokine receptor 1 (*Cx3cr1*)), and endothelial cells (Frizzled class receptor 3 (*Fzd3*)). Interestingly, *Scn1a* is distributed primarily in neuronal populations but also in non-neuronal populations like astrocytes, oligodendrocytes, endothelial cells, and immune cells in the brainstem (Figure 5C).

### 3.6. Genes Associated with Neurotransmission, Protein Translation, and Cellular Respiration Are Altered in the Brainstem of P40–50 HETs

**Figure 6:**
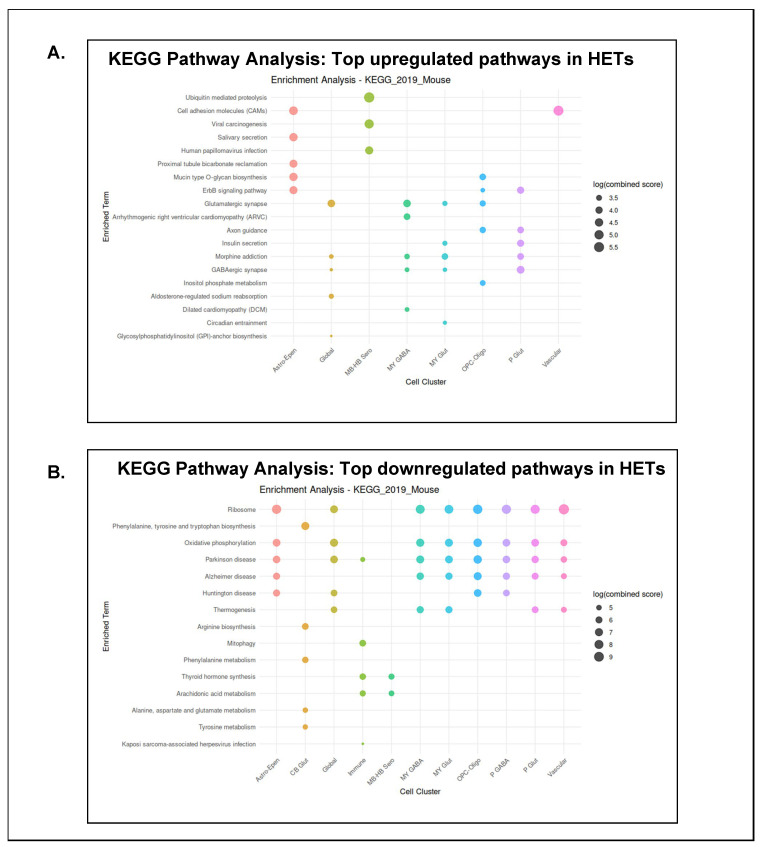
Genes involved in synaptic transmission, protein translation, and cellular respiration are altered in the brainstem of P40–50 HETs. Brainstem tissue was collected from P40–50 HET and age-matched WT mice. Single-nuclei suspension of brainstem tissue was obtained by gradient physical dissociation and filtration. snRNA-seq was performed using the 10× Chromium 3’ gene expression platform. Data was analyzed using SingleR and enrichR. (**A**,**B**) KEGG pathway analysis: (**A**) differentially expressed genes (within enriched pathways) upregulated in HETs; (**B**) differentially expressed genes (within enriched pathways) downregulated in HETs. Pathway analyses were performed with enrichR (3.1), and heatmaps were generated. *p*-values associated with heatmaps were obtained using Fisher’s exact test, followed by the Benjamini–Hochberg multiple testing correction for obtaining the adjusted *p*-values. The scale represents the log (adj *p*-value) with a cutoff of 1 × 10^−25^ for pathway heatmaps. N = 1/group.

To gain further insights about the transcriptome of HETs in comparison to WTs at P40–50, we plotted the top five significantly upregulated and downregulated genes between HETs and WTs for each cell cluster. In addition, the top five augmented and decreased genes overall (across all clusters) between HETs and WTs were plotted, and this was termed "global". To unravel the broader biological cascades wherein these differently expressed genes (DEGs) between HETs and WTs occur, we performed a KEGG pathway analysis. Genes associated with glutamatergic synaptic transmission were significantly upregulated globally, and in clusters termed MY-Glut, MY-GABA, and OPC-Oligo in HETs compared to WTs. Genes involved in GABAergic synaptic transmission were upregulated globally and in clusters termed MY-Glut, MY-GABA, and P-Glut in HETs. Genes associated with glycan biosynthesis and epidermal growth factor receptor (EGFR or ErbB) signaling were upregulated in OPC-Oligo and Astro-Epen clusters. Genes related to cell adhesion molecules (CAMs) were augmented in the Astro-Epen, MB-HB Sero, and vascular clusters. Interestingly, genes associated with ubiquitin-mediated proteolysis were significantly upregulated in the MB-HB Sero cluster (Figure 6A). Genes related to ribosomal protein translation and oxidative phosphorylation were significantly downregulated globally, and in MY-Glut, MY-GABA, P-Glut, P-GABA, Astro-Epen, OPC-Oligo, and cascular cell clusters in HETs compared to WTs. Genes associated with thermogenesis were downregulated globally, and in clusters termed MY-Glut, MY-GABA, P-Glut, and vascular. In the immune cell cluster, genes related to mitophagy and arachidonic acid metabolism were downregulated (Figure 6B).

To gain a broad overview of the genes altered in the brainstem of P40–50 HETs, the top 40 annotated genes that were significantly upregulated (Figure S8A) or downregulated (Figure S8B) globally were plotted. Genes such as acetyl-CoA synthetase short chain family, member 2 (*Acss2*), calcium/calmodulin-dependent protein kinase-1 delta (*Camk1d*), Down Syndrome cell adhesion molecule-like 1 (*DScaml1*), engulfment and cell motility 1 (*Elmo1*), gephyrin (*Gphn*), glutamate receptor metabotropic 3 (*Grm3*), inhibitory synaptic factor 2A (*Insyn2a*), leucine-rich repeat-containing protein 4 (*Lrrc4*), phospholipase C-like 1 (*Plcl1*), prickle planar cell polarity protein 2 (*Prickle2*), protocadherin 9 (*Pcdh9*), neural cell adhesion molecule 1 (*Ncam1*), solute carrier family 24 (sodium/potassium/calcium exchanger) member 2 (*Slc24a2*), slit-robo rho GTPase activating protein 3 (*Srgap3*), ST6 N-acetylgalactosaminide alpha-2,6-sialyltransferase 3 (*St6galnac3*), and tropomodulin 2 (*Tmod2*), ubiquitin (*Ubb*), and ubiquitin protein ligase 3a (*Ube 3a*) were upregulated (Figure S8A). Critical functions associated with these genes include maintenance of neural architecture and communication; complex regulation of glutamatergic and GABAergic synaptic transmission; regulation of neuronal polarity, dendritic complexity, and axonal growth; long-term potentiation/plasticity; calcium signaling; and proteasomal homeostasis [51,52,53]. Of note, genes associated with the regulation of myelin structure and function, such as myelin-associated oligodendrocyte basic protein (*Mobp*) and dishevelled-associated activator of morphogenesis 2 (*Daam2*), were upregulated in HETs [54,55]. In addition, genes like ecto-NOX disulfide-thiol exchanger 1 (*Enox1*) and oxidation resistance 1 (*Oxr1*) that are associated with regulation of redox homeostasis and resistance against oxidative stress, respectively, were upregulated [56,57].

Top downregulated genes included agrin (Agrn), Abelson helper integration site 1 (*Ahi1*), vacuolar ATPases (*Atp6v0b*, *Atpv0c*), basigin (*Bsg*), calmodulin 2 (*Calm2*), coiled-coil-helix-coiled-coil-helix domain containing protein 2 (*Chchd2*), cytochrome c oxidase subunit 4 isoform 1 (*Cox4i1*), cystatin 3 (*Cyst3*), heat shock proteins 90kDa alpha class B member 1 (*Hsp90ab1*) and 70kDa protein 8 (*Hspa8*), NEDD4 family interacting protein 1 (*Ndfip1*), NADH:Ubiquinone oxidoreductase subunit B8 (*Ndufb8*), poly(A)-binding protein, nuclear 1 (*Pabpn1*), prostaglandin D2 synthase (*Ptgds*), RNA-binding motif protein 39 (*Rbm39*), ribosomal proteins L (*Rpl6*, *9*, *13*, *17*, *27a*, and *p1*) and S (*Rps2*, *8*, *11*, *16*), solute carrier family 25 (mitochondrial phosphate carrier) member 3 (*Slc25a3*) and solute carrier family 38 (amino acid carrier) member 2 (*Slc38a2*), small nucleolar RNA host gene 11 (*Snhg11*), small nuclear ribonucleoprotein U1 subunit 70 (Snrnp70), tripartite motif containing 35 (*Trim35*), transthyretin (*Ttr*), ubiquitin (*Ubb*), X inactive specific transcript (*Xist*), and zinc finger cchc-type containing 18 (*Zcchc18*) (Figure S8B). These genes are associated with maintenance of neuromuscular junctions, serotonergic signaling, protein sorting, inflammation, calcium binding, mitochondrial electron transport chain function, thermoregulation, protein folding/trafficking/quality control, mRNA stability/translation, and synaptic plasticity/synaptogenesis, respectively [58,59,60,61]. Some of these observations are in line with data from a study that showed a significant downregulation of Trim32 protein, a multifunctional E3 ubiquitin ligase involved in synaptic protein turnover, neuronal differentiation, and immune signaling in the hippocampus of HETs [62].

Taken together, these data suggest that the brainstem transcriptomes are quite different between HETs and WTs during P40–50. Since we had only an N = 1/group for transcriptomics, the data presented are exploratory in nature. Additional biological replicates and experimental validation are required to reliably and comprehensively elucidate the link between alterations in the brainstem transcriptome of P40–50 HETs and DS pathophysiology. Nevertheless, our preliminary observations suggest potential changes in genes involved in neurotransmission, cellular respiration, redox homeostasis, and protein translation.

### 3.7. MS-Based Preliminary Proteomic Analysis Identifies Druggable, Kinase-Mediated Pathways

**Figure 7:**
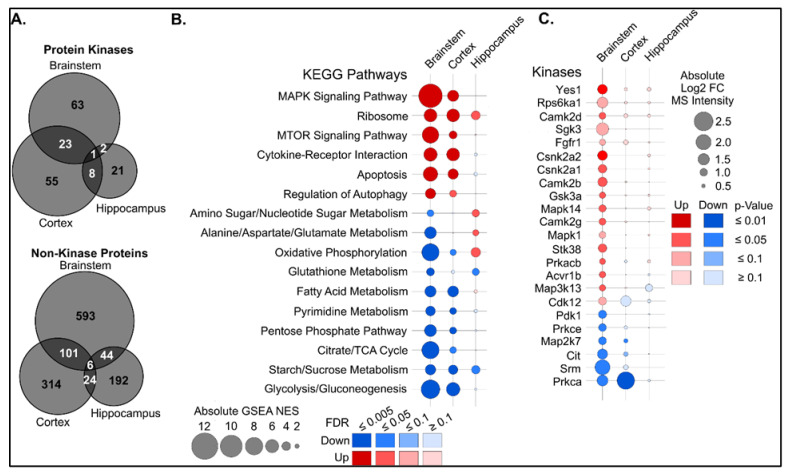
The proteome and kinome are systematically and specifically altered in the brainstem of P40–50 HETs. Brainstem, cortex, and hippocampal tissue were collected from P40–50 HET and age-matched WT mice and subjected to our MS-based kinobead AP-MS workflow. (**A**) Comparison of differentially expressed protein kinases (top) and non-kinase proteins (bottom), P40–50 HET versus WT mice, between different brain regions. Data was analyzed using a two-tailed two-sample Student’s *t*-test. *p* < 0.1, N = 2 HETs and 3 WTs, see Table S1. (**B**) GSEA analysis using KEGG gene sets, comparing pathway enrichment between the different brain regions. (**C**) Specific differentially expressed protein kinases by brain region (for statistics, see the Supplementary Materials Table S1).

Proteins are the molecular machines that control most cellular processes, and their expression and activity often correlate poorly with mRNA expression [63]. The 518 human protein kinases, i.e., the kinome, are critical cell signaling enzymes that regulate the activity, localization, and interactions of most proteins by reversible phosphorylation [64]. Kinases are frequently dysregulated in neurological disorders and highly druggable, making them priority targets for drug discovery [65,66]. To gain a preliminary understanding and quantify potential brain-region-specific alterations in the proteome and kinome, we applied our kinobead affinity purification (AP)-MS kinome profiling approach to brainstem, cortex, and hippocampus tissue from P40–50 HET and WT mice (N = 2 and 3, Table S1) [29,30,31,33]. Kinobead AP-MS identified and quantified 3771 proteins, including 315 protein kinases. Differential expression analysis revealed systematic alterations in the proteome and kinome that were brain-region-specific (Table S1, Figure 7A). Thus, 25.4%, 13.3%, and 7.2% of the brainstem, cortex, and hippocampal proteome were altered, respectively, suggesting possible systematic remodeling of the brain proteome in HET mice, particularly in the brainstem and cortex. The proteins and kinases that were altered were highly divergent, further suggesting brain-region-specific alterations (Figure 7A). Gene set enrichment analysis (GSEA) with KEGG gene sets confirmed our results with snRNA-seq analysis and showed that pathways were altered in a brain-region-specific manner (Figure 7B). Thus, metabolic pathways related to glycolysis, the TCA cycle, and fatty acid oxidation were decreased in the brainstem, and to a lesser extent in the cortex, of HET mice, while mitogen-activated protein kinase (MAPK), cytokine, and mechanistic (mammalian) target of rapamycin (MTOR) signaling, as well as oxidative stress-related autophagy were increased (Figure 7B). Next, we analyzed kinome alterations that were associated with proteome and pathway-level changes. Kinases that were highly and specifically increased in the brainstem of HET mice included the proliferation and survival kinases tyrosine-protein kinase Yes (Yes1), ribosomal protein S6 kinase alpha-1 (Rps6ka1), fibroblast growth factor receptor 1 (Fgfr1), and Mapk1; the metabolic kinases serum/glucocorticoid regulated kinase 2 (Sgk2) and casein kinase 2 alpha 1 and 2 (Csnk2a1 and 2); and the stress kinases glycogen synthase kinase 3-alpha (Gsk3a), Mapk13, and Mapk14 (Figure 7C). This suggested that in the brainstem and cortex, stress and pro-apoptotic signaling are counteracted by proliferative and pro-survival signaling, and that inhibiting stress kinases may shift the balance toward survival.

## 4. Discussion

Seizure-induced metabolic alterations in the brain can have detrimental consequences, including worsening of seizure outcomes, epilepsy comorbidities, and peripheral organ system dysfunction [40,67,68]. Disordered metabolism has been observed in DS, which has the highest mortality rate due to SUDEP compared to other childhood epileptic encephalopathies [4]. The primary focus of previous studies has been to investigate metabolic changes in cortical areas like the hippocampus [21]. Whether such metabolic alterations occur in the brainstem is unknown. Using a mouse model of DS, we observed several key findings. *First*, there is chronic neuronal activation in the brainstem of younger HET mice. *Second*, there are regional and age-based differences in the brain metabolomes of HETs. *Third*, preliminary snRNA seq and MS-based proteomic analyses have revealed concordant baseline transcriptomic and proteomic variations in the brainstem of HETs compared to WTs. Additionally, kinome profiling showed that several stress kinases were increased in expression in the brainstem. These kinases may serve as drug targets to suppress stress-induced apoptosis in the brainstem, though this warrants further investigation. To the best of our knowledge, this is the first study of its kind to (i) address the role of brainstem metabolism in DS and (ii) investigate DS metabolomic, transcriptomic, and proteomic alterations specifically in the brainstem as a plausible mechanism for SUDEP.

The brainstem contains neuronal clusters that regulate critical parameters like breathing, heart rate, blood pH, oxygen, CO_2_ levels, and gastrointestinal reflexes [69]. Key brainstem regions include the NTS, VLM, and the RTN [42,43,44]. In this study, chronic activation of the NTS, VLM, and RTN was observed during the “critical period” (P20–30) in HETs by measuring ΔFosB, a leucine-zipper activity-induced transcription factor with a long half-life, and a protein product of the truncated splice variant of the *FosB* gene [70]. Elevated ΔFosB occurs in the lateral amygdala and hippocampi in response to 6 Hz corneal stimulations [71], kainate or pilocarpine treatment in mice [72,73], and in patients with temporal lobe epilepsy (TLE) [74]. The increase in ΔFosB in the brainstem nuclei of HETs suggests repeated activation in these brain regions; however, whether this can be attributed to seizure activity or other cellular stresses needs to be validated by additional studies that assess spontaneous seizure activity and propagation to the brainstem via EEG. Additionally, further immunohistochemical or electrophysiologic studies of brainstem nuclei are needed to confirm the ΔFosB data and demonstrate chronic neuronal activation in these brain regions.

In our study, the brainstem metabolome of HETs during the critical period (~P20–30) was distinctly different compared to age-matched WTs. The brainstem metabolome was also different compared to the forebrain of HETs. Specifically, glycolysis/gluconeogenesis and PPP intermediates, such as G6P, fructose-6-phosphate, 2,3-diphosphoglyceric acid, sedoheptulose-7-phosphate, and NADPH, were significantly elevated in the brainstem. The energy substrate, ATP, was also significantly increased. This suggests an overall increase in brainstem metabolism, particularly aerobic glycolysis, which could be partially indicative of the Warburg effect (to be confirmed via metabolic flux assays), a less efficient but quicker way of meeting increased energy requirements. These results align with findings from another study that reported increases in G6P, fructose-6-phosphate, fructose 1,6-bisphosphate, dihydroxyacetone phosphate, pyruvic acid, and 6-phosphogluconic acid intermediates, respectively, [21] in the hippocampi of HET mice. Interestingly, a study using the *scn1Lab* mutant DS zebrafish larvae demonstrated that the upregulation of gluconeogenesis helped to normalize metabolic deficits and control electrographic seizures [75]. Chronic neuronal activation, particularly during seizures, is metabolically demanding and can exhaust energy provided by normal mitochondrial respiration [76]. To compensate for the rapid depletion of energy stores and overcome metabolic exhaustion, cells can start to rely on enhanced aerobic glycolysis [67,77]. While our study demonstrates chronic neuronal activation via an increase in ΔFosB in brainstem nuclei, the exact cause for enhanced glycolytic and PPP metabolism is still unclear. Other brainstem metabolites that were increased in P20–30 HETs include sucrose and stearic acid. Sucrose, a disaccharide, can be broken down into glucose and fructose, and hence could probably act as an alternative fuel source. Our metabolomic data suggest an overall increase in energy metabolism, evidenced by an increase in glycolytic and pentose phosphate pathway intermediates in the brainstem of P20–30 HETs. However, sucrose is not naturally occurring in the brain, so our observations are surprising. Two plausible explanations for the increase in sucrose could be (i) to compensate for the high metabolic demand and (ii) an early indication of a possibly compromised/leaky blood–brain barrier, which has been previously reported in a mouse model of DS [78]. Stearic acid, an 18-carbon saturated fatty acid, could play several roles in the brainstem, such as involvement in mitochondrial fatty acid oxidation, and protection against oxidative stress [79] and glutamate toxicity [80]. The increase in stearic acid has been shown to be either a compensatory adaptation in response to stress or a pathological outcome of seizure activity. An increase in stearic acid in the brainstem of HETs may be a protective/pro-survival adaptation, as stearic acid has been shown to protect against seizures [81], oxidative injury [82], and enhance mitochondrial metabolism [83]. However, we cannot rule out the possibility of an increase in stearic acid levels due to possible seizure-induced lipolysis [84]. In our study, interpretation becomes quite complex since there are no EEG studies to confirm seizure spread to the brainstem. Nevertheless, stable isotope tracing studies of select lipids could provide insights into the source and utilization of stearic acid in the brainstem of P20–30 HETs. Increases in inosine, a breakdown product of ATP, and N-acetyl lysine were also observed. Inosine levels have been shown to increase after seizures and potentially exert a benzodiazepine receptor-mediated antiseizure effect [85]. N-acetyl lysine can participate in important post-translational modifications that can exert metabolic control [86]. Whether alterations in these metabolite levels confer protection or trigger a damage response in the brainstem of HETs needs to be investigated.

When comparing metabolites between the forebrain and brainstem of HETs, 2-hydroxyglutaric acid, malic acid, sedoheptulose-7-phosphate, and inosine were significantly elevated, and oleic acid was decreased in the HET brainstem compared to the forebrain. An increase in seduheptulose-7-phosphate, an intermediate of the non-oxidative branch of the pentose phosphate pathway, could indicate an increase in flux from glycolysis. A potential downstream consequence of this could be an increase in the production of NADPH, a crucial cofactor for cellular antioxidant responses [87]. Elevated malic and 2-hydroxyglutaric acid levels could indicate a disruption in the mitochondrial tricarboxylic acid cycle, suggesting a potential impairment in mitochondrial energy metabolism, which was reported by a previous study in the hippocampus of *Scn1a^A1783V^*^/*WT*^ DS mice [17,21]. Oleic acid is a monounsaturated fatty acid that is used to synthesize myelin phospholipids. A decrease in oleic acid in the brainstem of HETs could potentially indicate underlying metabolic/oxidative stress [88]. Future studies that assess bioenergetic functions (glycolysis, mitochondrial respiration) and levels of cellular oxidants/antioxidants are warranted to delineate metabolic alterations in the HET brainstem. Baseline glucose levels in the brainstem and forebrain of P20–30 HETs were unaltered in comparison to WTs. This is consistent with observations of unaltered brain glucose levels in chronic animal models and human patients with epilepsy [89,90]. It is important to note that while absolute brain glucose concentrations remain unaltered, uptake and utilization of glucose in different brain regions to meet metabolic demands may be altered, which is often observed in DS patients. Glycogen is a reserve fuel source that is stored mainly in astrocytes, and supplements glucose via the astrocyte–neuron lactate shuttle during periods of high energy demand/metabolism (e.g., seizure activity) and stress [89,91]. Brainstem and forebrain glycogen levels remained unaltered in P20–30 HETs, plausibly indicating the absence of high/abnormal metabolic demand. Although tissue glycogen levels were not drastically altered, additional studies that assess glycogen synthesis and mobilization in HET and WT mice are needed. The activities of HK and G6PD, the rate-limiting enzymes of glycolysis and the PPP, respectively, were unaltered in the brainstem and forebrain of P20–30 HETs. While the activities of these enzymes per se may not be affected, the rate/flux through these pathways could be altered, but were not analyzed in this study. An important methodological limitation was the gross measurement of glucose, glycogen levels, and activities of HK and G6PD in the brainstem and forebrain tissue. Studies have shown clear cell-type-specific (neurons versus glia) differences in the levels and utilization of fuel substrates like glucose and glycogen [92,93]. Additionally, the expression and activities of HK and G6PD can also be cell-type specific [94,95,96]. Several factors, including the location of seizure foci, cerebral blood flow, cell-type-specific metabolic requirements, and dynamic neuronal–glial interactions, can influence the levels of fuel substrates and activities of metabolic enzymes [97]. Furthermore, for brain tissue collection in our study, the occurrence of a seizure prior to sample collection (excluding hyperthermia experiments) was not considered to be a requirement. This could also be a possible explanation for the lack of changes in baseline glucose, glycogen levels, and activities of HK and G6PD. While prolonged/repeated seizures can eventually deplete glucose/glycogen stores and alter the expression of glycolytic enzymes [67], acute seizures like hyperthermia-induced seizures typically tend to increase the metabolic rate (e.g., glycolytic rate) [20,68,98,99], but these were not assessed in our study.

To gain a better understanding of temporal changes in the brainstem metabolome, metabolomic analysis of the brainstem was performed in P40–50 HETs that survived the critical period. Surprisingly, aconitate and reduced GSH were the only two metabolites that were significantly elevated in HETs compared to WTs. Elevation in aconitate levels, a mitochondrial TCA cycle intermediate, could plausibly indicate efforts to increase cellular energy, synthesis of precursors for amino acid, and neurotransmitter production via mitochondrial oxidative phosphorylation to support brainstem metabolism. This is supported by metabolomic data from P20–30 HET brainstem from our study that showed increases in glycolytic intermediates, which are usually catabolized to feed into the mitochondrial TCA cycle. These results are incongruent with a study that reported a decrease in TCA cycle metabolites in the hippocampus of *Scn1a^A1783V^*^/*WT*^ HET mice, plausibly because older mice (>P50) were used in this study [21]. GSH, a tripeptide of glutamate, cysteine, and glycine, is a non-protein antioxidant thiol that is found in millimolar (~2mM) concentrations in the brain [100]. GSH, along with its redox couple, oxidized glutathione (GSSG), is crucial for detoxifying reactive oxygen and nitrogen species and maintaining redox homeostasis, particularly in the brain, which is highly vulnerable to oxidative stress [101,102]. Dysregulation of the glutathione antioxidant system has been observed in both genetic and acquired epilepsy models, and in human patients [103,104,105]. A significant increase in GSH in the brainstem of P40–50 HETs could indicate that this brain region is getting "primed" to actively mitigate possible cellular oxidative stress. NADPH, a cofactor that is synthesized via the oxidative arm of the PPP and required for converting GSSG to GSH by glutathione reductase to maintain the GSH pool, was elevated in the brainstem of P20–30 HETs. This suggests that the brainstem could be mounting an antioxidant response, possibly to ameliorate ongoing oxidative stress and achieve an adaptive advantage. Studies that address whether there is a disruption in brainstem redox homeostasis, and its associated triggers/causes and consequences, are warranted. Interestingly, GSH elevation using a small molecule compound, dimercaprol, attenuated both behavioral and electrographic seizure-like activity in DS zebrafish larvae [106].

The forebrain metabolome of P20–30 HETs was significantly altered compared to age-matched WTs. Specifically, there was a decrease in mitochondrial TCA cycle metabolites. The levels of glutamic acid/glutamate, an excitatory neurotransmitter, were also decreased. Decrease in glutamate levels could inevitably affect the synthesis of the inhibitory neurotransmitter GABA, thereby adversely impacting neuronal excitability [107]. These findings are consistent with a study that reported decreases in TCA and PPP intermediates in the hippocampus of *Scn1a^A1783V^*^/*WT*^ HET mice [21]. These data could indicate potential mitochondrial dysfunction and decreased dependence on mitochondrial metabolism for energy production. Interestingly, mitochondrial dysfunction has been reported in DS larval zebrafish (*scn1Lab*) and muscle biopsies of patients with DS [108,109]. Plasma metabolomic analysis of P20–30 HETs revealed significant decreases in mitochondrial TCA cycle intermediates, nucleotide/nucleoside precursors, and intermediates involved in GSH and neurotransmitter biosynthesis. These changes could be reflective of underlying metabolic stresses, including alterations in mitochondrial metabolism, biosynthesis of energy precursors, and redox balance in HETs during the period of highest susceptibility to mortality. Changes in these particular metabolites were not observed in the plasma of HETs from another study, probably owing to differences in the ages of mice (>P50) used [21].

We analyzed the brainstem of P40–50 HETs that survived the critical period to gain a preliminary understanding of potential transcriptomic changes. Twelve cell clusters were annotated using the Allen Mouse Brain Atlas as a reference. Certain clusters were annotated as midbrain, hindbrain, cerebellar, and intratelencephalic–extratelencephalic in the brainstem snRNA dataset, plausibly due to the following reasons: (i) projection of brainstem neurons to other regions; (ii) shared molecular markers/transcriptional programs; and (iii) anatomical proximity to other brain regions [110,111,112,113]. Although an N = 1 snRNA seq analysis revealed a distinct transcriptional landscape in the brainstem of HETs compared to WTs, and an upregulation of genes associated with the regulation of synaptic transmission, calcium signaling, myelin morphology/function, proteasomal/redox homeostasis, was observed in HETs. These observations are congruent with hippocampal proteomic data from HET mice that showed complex regulation of synaptic function/regulation, glutamatergic/GABAergic neurotransmission, ion channel function, and calcium and nitric oxide signaling [62]. A significant downregulation of genes associated with mitochondrial oxidative phosphorylation, protein translation, and thermogenesis was observed in the brainstem of P40–50 HETs. Mitochondrial hypometabolism and defects in the mitochondrial electron transport chain have been observed in DS zebrafish larvae and patient muscle biopsies [108,109]. Miljanovic N et al. [62] reported alterations in proteins involved in transcriptional/translational regulation and nucleic acid metabolism in the hippocampus of HET mice. Disordered thermoregulation and enhanced susceptibility to heat-induced seizures are hallmarks of DS pathophysiology and have been observed in experimental models [26] and human patients [114]. Based on our preliminary transcriptomic findings, we cautiously speculate that there could be increased neurotransmission in the brainstem of P40–50 HETs. Energy to support this would preferentially be obtained from aerobic glycolysis, leading to a decrease in mitochondrial oxidative phosphorylation, which in turn can eventually cause mitochondrial dysfunction, thereby resulting in oxidative stress. To augment cellular energy production, protein translation, which is an energy-consuming process, is often downregulated [115]. This suggests that the brainstem of HETs is altered in a way that is suggestive of being more "protective" of its functionality, given that it is a pivotal center that controls critical autonomic functions. Nevertheless, studies assessing the functionality and flux through metabolic pathways, such as glycolysis, mitochondrial oxidative phosphorylation, and the pentose phosphate pathway, are highly warranted to gain a clear understanding of plausible metabolic alterations in the HET brainstem.

MS-based proteomics and KEGG pathway analyses of brainstem, cortex, and hippocampus demonstrated that metabolic and signaling pathways were specifically altered in the brainstem versus the cortex and hippocampus. Kinome profiling revealed increases in stress and pro-apoptotic kinases like p38α and p38δ (Mapk14 and Mapk13), glycogen synthase kinase 3α (Gsk3a), and nuclear Dbf2-related kinase 1 (NDR1 or Stk38). Different p38 isoforms have been reported to be both neuroprotective in epilepsy and to increase seizure severity [116,117]. Cellular stressors, such as hypoxia, oxidative stress, and pro-inflammatory signaling, have been shown to activate stress kinases, including Stk38, Gsk3, Mapk13, and Mapk14 [118,119,120]. These kinases are involved in regulating vital cellular processes like autophagy/mitophagy, protein stability, mitochondrial energy metabolism, apoptosis, and neuroinflammation [121,122,123,124]. Dysregulated (over)expression of these proteins in the brainstem can have deleterious consequences. Whether targeting these specific druggable kinases with inhibitors would decrease spontaneous seizures and/or the incidence of SUDEP in DS requires careful evaluation in future studies. Sgk3, Gsk3a, and Stk38, as well as other stress kinases that we found to be overexpressed in the brainstem, such as Map3k13, have been less well-studied in the context of epilepsy. Whether the kinases are pro-epileptogenic or if inhibiting these kinases may prevent epilepsy has to be determined in future studies. Although our study has sample size limitations, we cautiously speculate that there is a strong trend towards alterations in processes such as energy metabolism, ribosomal protein translation, redox homeostasis, and cell survival/cell death in the brainstem of HETs, which is corroborated by our transcriptomic and metabolomic data. Our exploratory proteomics has certain caveats. The sample size per group (N = 2 HETs and 3 WTs) was small for exploratory proteomics/kinomics; hence, the threshold of *p* < 0.1 was chosen. With a limited sample size, there is an increased likelihood of finding false positives, particularly in the kinomic dataset. We interpreted the results from this study with caution, and our primary goal was to gain a broader understanding of potential alterations in the brainstem proteome/kinome of HETs. Future studies with increased sample size per group (N = 6–8) will permit more stringent statistical thresholds and increase the robustness of the analysis. Another study that performed hippocampal proteomic analysis in young HET mice (~P14–26) showed alterations in proteins involved in synaptic transmission (glutamatergic signaling), synaptic plasticity, astrogliosis, and nitric oxide signaling [62]. Hippocampal proteomic profiling in *Scn1a* knockout (KO) mice revealed changes in the von Hippel–Lindau/Hypoxia-inducible factor 1 alpha/cyclin-dependent kinase inhibitor 1A (VHL/Hif1α/p21) signaling that might contribute to neuronal apoptosis in these mice [125].

This study has certain limitations: (i) *Scn1a^A1783V^*^/*WT*^ HET mice exhibit generalized spontaneous/recurrent seizures; however, in this study, ΔFosB levels were assessed only in certain brainstem nuclei. Studies that delineate the seizure origin and potential spread to the brainstem circuitry using EEG are warranted. (ii) The brainstem is heterogeneous, consisting of diverse neuronal clusters that control different autonomic functions. However, metabolic alterations pertaining to key brainstem nuclei were not performed in this study. (iii) The N for snRNA seq was small (N = 1), and select genes were not validated by secondary techniques like RT-PCR. Increasing the sample size and verifying/validating the expression of certain genes are required. (iv) Glycolytic and mitochondrial respiration rates in different brain regions were not assessed. Analysis of these endpoints would help gain a better understanding of spatial and temporal metabolic alterations. (v) Transcriptomic and proteomic analyses of HET brainstem during the critical period (P20–30) of enhanced susceptibility to mortality are warranted to support early drug interventions. (vi) This study does not ascertain whether the observed metabolic changes were a cause or consequence of seizures. (vii) Finally, proteomic and kinomic studies were limited and lacked functional validation

Future directions include the following: (i) Assessing whether there is ongoing (younger to older) oxidative stress in the brainstem of HETs, since we observed a significant increase in GSH levels during the P40–50 developmental period; (ii) performing spatial transcriptomics, metabolomics, and proteomics with single-cell resolution to identify cell-type-specific metabolic alterations in the HET brainstem to help better understand the pathophysiology of SUDEP and identify novel druggable targets; (iii) identifying approaches for brain-region (forebrain vs. brainstem) specific correction of metabolic deficits; (iv) administering specific kinase inhibitors (e.g., BIO-acetoxime for Gsk3 inhibition) [126] and evaluating the effects on seizure outcomes, as well as SUDEP susceptibility and incidence in HET mice; (v) identifying common/overlapping pathways from the multiomics analyses, and determining putative molecular targets that can be modulated to alter seizure, morbidity, and mortality outcomes in HET mice; and (vi) determining if and how these metabolomic, transcriptomic, and proteomic changes could impact autonomic functions like respiration and cardiac functions, which can be assessed using whole-body plethysmography (WBP) and electrocardiography (ECG), respectively.

## 5. Conclusions

This study demonstrates that the brainstem is an important region that could be metabolically altered in a region-specific and age-dependent manner in DS. This study highlights alterations at the single-cell level in the brainstem of DS mice. Furthermore, findings from this study unraveled proteomic and kinomic alterations in the brainstem of DS mice. Taken together, these molecular-level changes could play a significant role in the pathogenesis of DS and could have a profound impact on seizure, mortality, and morbidity outcomes. 

## Data Availability

The single-nuclei RNA-seq data discussed in this publication have been deposited in NCBI’s Gene Expression Omnibus (GEO) and are accessible through the GEO series, accession number GSE301399. Please see the following link: https://www.ncbi.nlm.nih.gov/geo/query/acc.cgi?acc=GSE301399 (accessed on 30 June 2025). For the proteomic and kinomic analyses, Bruker MS raw files and DIA-NN output files were deposited in the MassIVE repository of the University of California, San Diego, and are freely available under the accession number MSV000098704.

## Supplementary Materials

The following supporting information can be downloaded at: https://www.mdpi.com/article/10.3390/cells15010067/s1. Figure S1. Forebrain glucose levels are unaltered by hyperthermia-induced seizures in P20–30 HETs; Figure S2. Differences between forebrain and brainstem metabolic landscapes in P20–30 HETs and WTs; Figure S3. Several plasma metabolites are decreased in P20–30 HETs; Figure S4. Brain region-specific differences in basal fuel substrates, and enzyme activities in P20–30 mice; Figure S5. Brain-region specific differences in glycogen levels and HK, G6PD activities in P40–50 mice; Figure S6. Brainstem cell clusters in P40–50 HETs and WTs; Figure S7. Different brainstem cell clusters identified using cell-type specific markers; Figure S8. Top 40 annotated genes and their expression profiles in P40–50 HET mice brainstem; Table S1. Differential expression analysis revealed systematic alterations in the kinome.
