# Supplementary material for: Multiomic Analyses Reveal Brainstem Metabolic Changes in a Mouse Model of Dravet Syndrome
Source: Cells. 2025 Dec 30;15(1):67. doi: 10.3390/cells15010067 (PMC12785442; doi:10.3390/cells15010067)
Supplement: Supplementary file 1 [file cells-15-00067-s001.zip › cells-3906916-supplementary.pdf]

Multiomic analyses reveal brainstem metabolic changes in a mouse model of Dravet Syndrome

Ashwini Sri Hari[1,2], Alexandria M. Chan[2,3], Audrey Scholl[2], Aidan Mulligan[2], Janint Camacho[1,2], Ireland Rose Kearns[1,2], Gustavo Vasquez Opazo[1,2], Jenna Cheminant[2], Teresa Musci[2], Min-Jee Goh[2], Alessandro Venosa[2], Philip J. Moos[2], Martin Golkowski[2,3], and Cameron S. Metcalf[1,2]

Author affiliations:

1. Epilepsy Therapy Screening Program (ETSP) Contract Site, University of Utah, Salt Lake City, UT, USA
2. Department of Pharmacology and Toxicology, University of Utah, Salt Lake City, UT, USA
3. Huntsman Cancer Institute, University of Utah, Salt Lake City, UT, USA

Appendix. SA **Supplementary Figures**

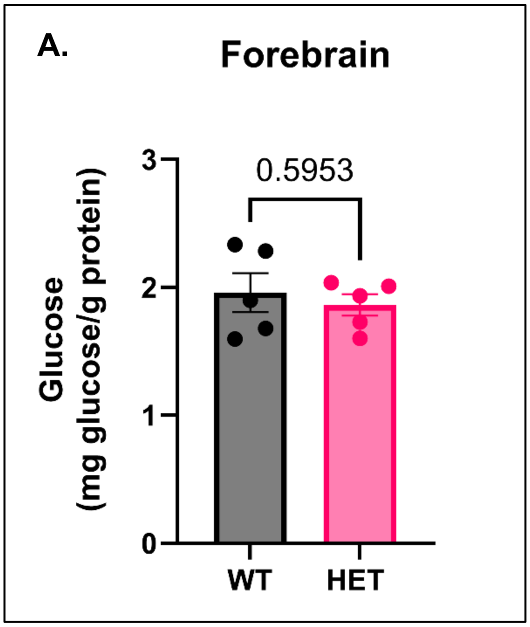

Figure S1. Forebrain glucose levels are unaltered by hyperthermia-induced seizures in P20-30 HETs. HET and WT mice received hyperthermia treatment following which forebrain tissue was collected, glucose levels were assayed and normalized to protein concentration. (**A**) Forebrain glucose levels. There were no significant changes in glucose levels in the forebrain of HETs after hyperthermia-induced seizures. Data are represented as mean ± SEM (error bars). Student's unpaired t-test was utilized to assess statistical differences between WT and HET. N= 5/group

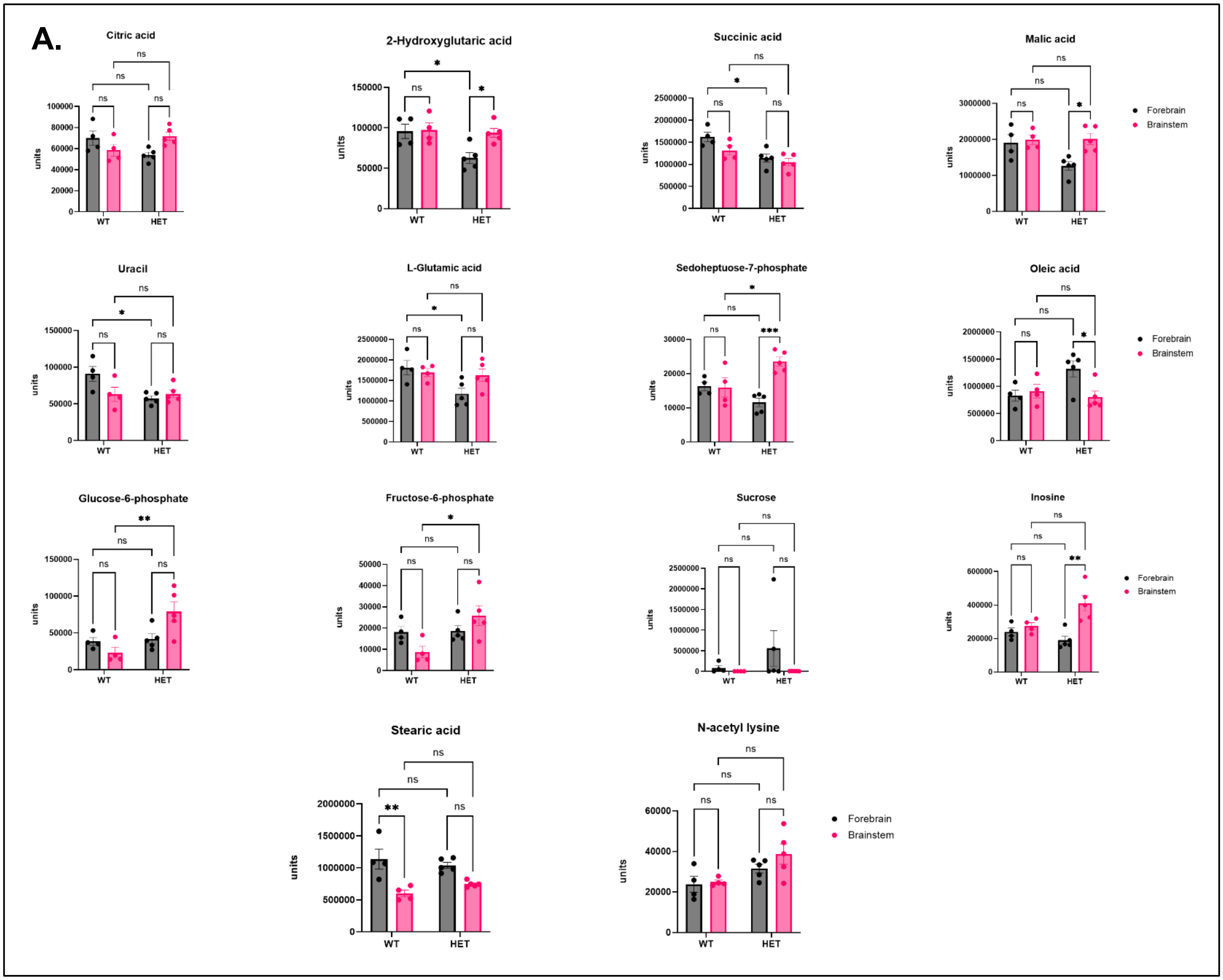

A.
Citric acid
2-Hydroxyglutaric acid
Succinic acid
Malic acid
Uracil
L-Glutamic acid
Sedoheptuose-7-phosphate
Oleic acid
Glucose-6-phosphate
Fructose-6-phosphate
Sucrose
Inosine
Stearic acid
N-acetyl lysine
units
WT
HET
Forebrain
Brainstem

Figure S2. Differences between forebrain and brainstem metabolic landscapes in P20-30 HETs and WTs. Forebrain and brainstem tissue were collected from P20-30 HET and age-matched WT mice for metabolomic analysis. Metabolite concentrations were normalized to tissue weight. (**A**) Comparison of metabolites in the forebrain *versus* brainstem regions of HETs and WTs. Units on the Y-axis refer to 'area under the curve' values. Data are represented as mean ± SEM (error bars). Two-way ANOVA with Sidak's multiple comparison's test was utilized to assess statistical differences between forebrain and brainstem in WTs and HETs. Ns: not significant, *p<0.05, **p<0.01, ***p<0.001 *versus* HET brainstem; ns: not significant, **p<0.01 *versus* WT brainstem. All other comparisons have been shown in the main manuscript in the Results section. N= 4-5/group

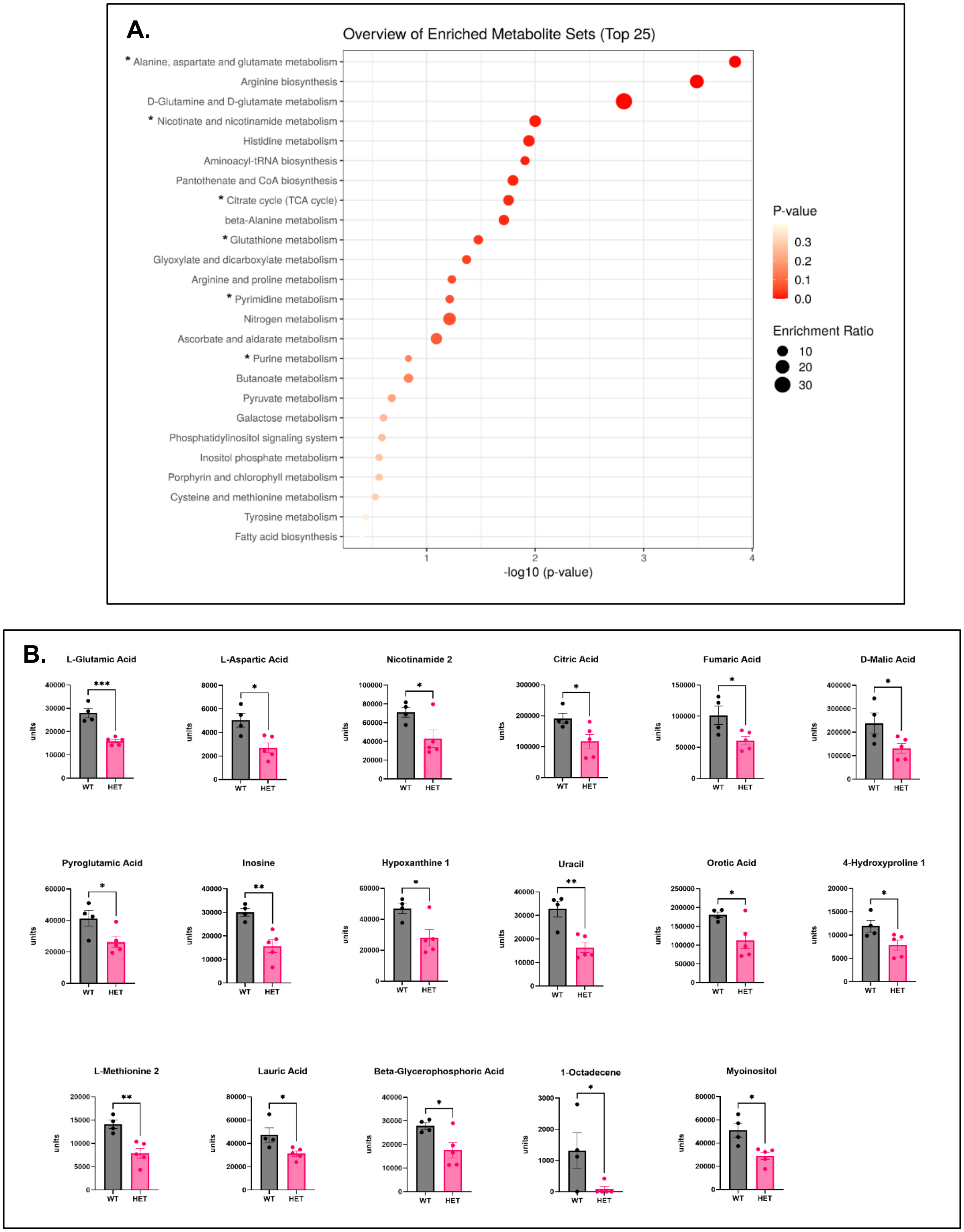
A.
Overview of Enriched Metabolite Sets (Top 25)
* Alanine, aspartate and glutamate metabolism
Arginine biosynthesis
D-Glutamine and D-glutamate metabolism
* Nicotinate and nicotinamide metabolism
Histidine metabolism
Aminoacyl-tRNA biosynthesis
Pantothenate and CoA biosynthesis
* Citrate cycle (TCA cycle)
beta-Alanine metabolism
* Glutathione metabolism
Glyoxylate and dicarboxylate metabolism
Arginine and proline metabolism
* Pyrimidine metabolism
Nitrogen metabolism
Ascorbate and aldarate metabolism
* Purine metabolism
Butanoate metabolism
Pyruvate metabolism
Galactose metabolism
Phosphatidylinositol signaling system
Inositol phosphate metabolism
Porphyrin and chlorophyll metabolism
Cysteine and methionine metabolism
Tyrosine metabolism
Fatty acid biosynthesis
-log10 (p-value)
P-value
0.3
0.2
0.1
0.0
Enrichment Ratio
10
20
30
B.
L-Glutamic Acid
L-Aspartic Acid
Nicotinamide 2
Citric Acid
Fumaric Acid
D-Malic Acid
Pyroglutamic Acid
Inosine
Hypoxanthine 1
Uracil
Orotic Acid
4-Hydroxyproline 1
L-Methionine 2
Lauric Acid
Beta-Glycerophosphoric Acid
1-Octadecene
Myoinositol
units
WT
HET

Figure S3. Several plasma metabolites are decreased in P20-30 HETs. Blood was collected from P20-30 HET and age-matched WT mice. Plasma was extracted and submitted for metabolomic analysis (**A**) KEGG pathway analysis showing top 25 enriched pathways. (**B**) Metabolites that are significantly altered in plasma. Certain metabolites possibly involved in neurotransmission, energy production, and antioxidant replenishment are depleted in HETs. Data are represented as mean ± SEM (error bars). For the KEGG pathway analysis in panel A, p-values and enrichment ratios are represented within the image. The asterix (*) in panel A highlights the pathways in which metabolites are significantly altered and shown in panel B. For metabolites in panel B, $^{*}p<0.05$, $^{***}p<0.001$ *versus* WT by Student's two-tailed unpaired t-test. N= 4-5/group

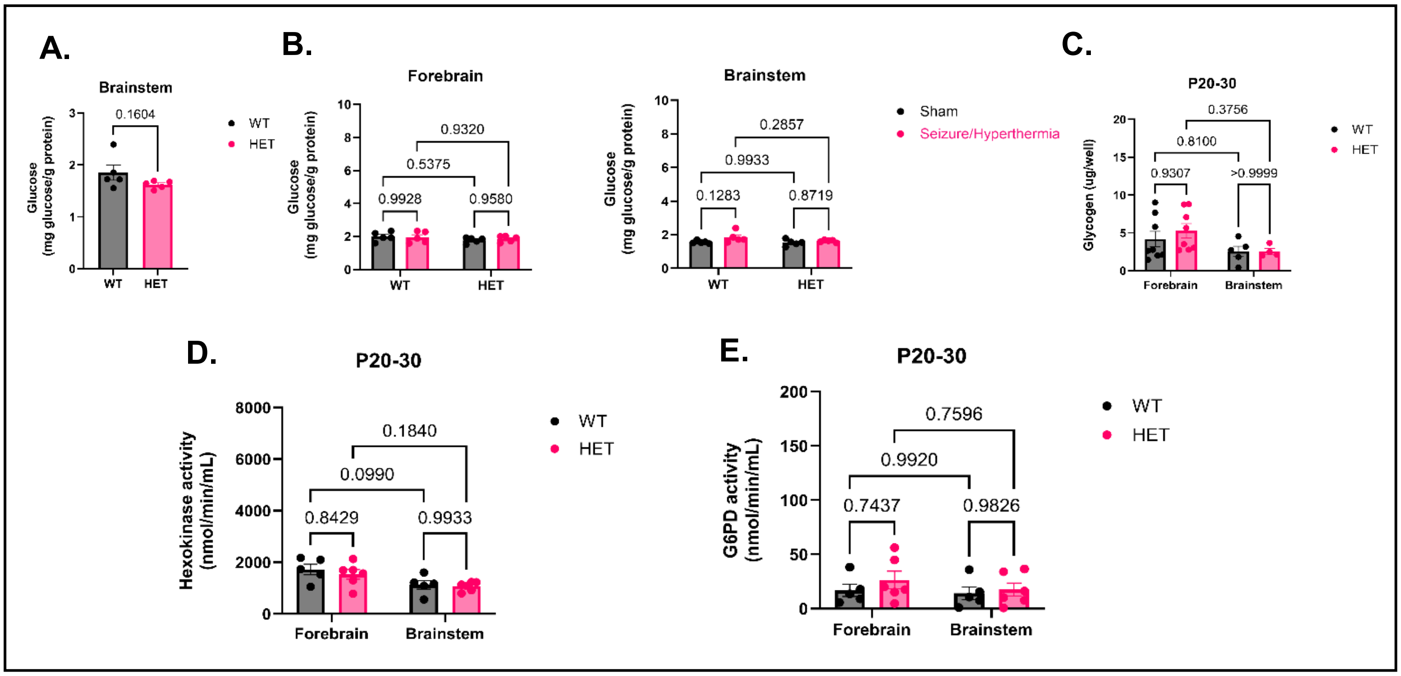

Figure S4. Brain region-specific differences in basal fuel substrates, and enzyme activities in P20-30 mice. Forebrain and brainstem tissue were collected from P20-30 HET and age-matched WT mice that were either naïve or received hyperthermia treatment. Samples were assayed for glucose, glycogen levels and hexokinase (HK), and glucose-6-phosphate dehydrogenase (G6PD) activities. (**A**) Brainstem glucose levels in WT and HET mice that received hyperthermia treatment. (**B**) Glucose levels in the forebrain and brainstem of P20-30 WTs and HETs that were either naïve (sham) or received hyperthermia treatment. (**C**) Baseline glycogen levels across different brain regions. (**D**) Baseline HK and G6PD enzyme activities across different brain regions. Data are represented as mean ± SEM (error bars). Student's two-tailed unpaired t-test was utilized to assess statistical differences between two groups. Two-way ANOVA with Tukey's multiple comparison's test was used for comparing more than 2 groups. N= 4-8/ group

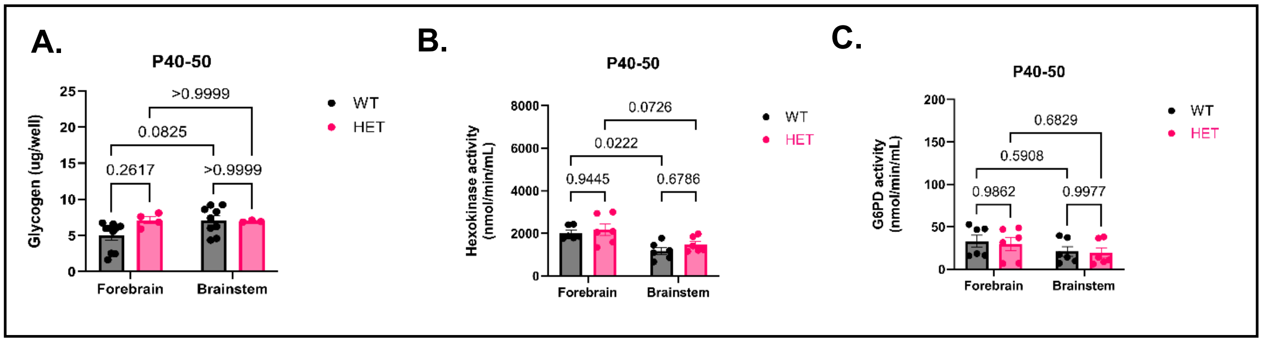

Figure S5. Brain-region specific differences in glycogen levels and HK, G6PD activities in P40-50 mice. Forebrain and brainstem tissue were collected from P40-50 mice and assayed for glycogen levels, and HK, G6PD activities. (**A**) Baseline glycogen levels across different brain regions (**B**) Basal hexokinase (HK) activity in different brain regions. (**C**) Basal glucose-6-phosphate dehydrogenase (G6PD) activity in various brain regions. Data are represented as mean ± SEM (error bars). Two-way ANOVA with Tukey's multiple comparison's test was used for comparing more than 2 groups. N= 4-9/ group

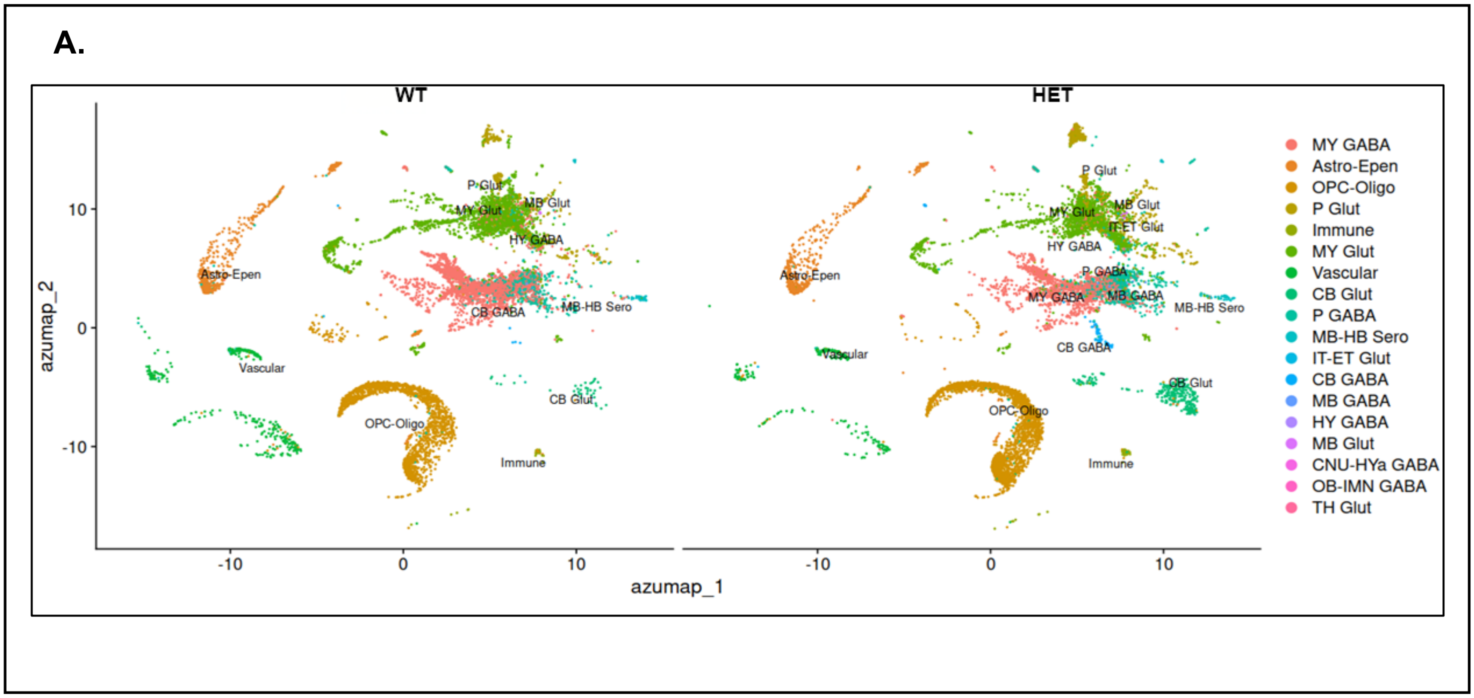

Figure S6. Brainstem cell clusters in P40-50 HETs and WTs. Brainstem tissue was collected from P40-50 HET and age-matched WT mice. Single-nuclei (sn) suspension of brainstem tissue was obtained by gradient physical dissociation and filtration. snRNA-seq was performed using the 10X Chromium 3' gene expression platform. Brainstem cell types were annotated using Azimuth *via* Seurat's reference mapping framework. (**A**) Azumap of annotated brainstem cell clusters between HETs and WTs. 12 clusters were identified and include various (i) neuronal subtypes: mylencephalic glutamatergic (MY-Glut) and GABAergic (MY-GABA) neurons, pons glutamatergic (P-Glut), and GABAergic (P-GABA) neurons, midbrain glutamatergic neurons (MB-Glut), cerebellar glutamatergic neurons (CB-Glut), intratelencephalic and extratelencephalic projecting excitatory neurons (IT-ET Glut), and midbrain-hindbrain serotonergic neurons (MB-HB Sero); (ii) astrocytes-ependymal cells (Astro-Epen); (iii) oligodendrocyte precursor cells-oligodendrocytes (OPC-Oligo); (iv) immune cells; and (v) vascular cells. N= 1/group

A.

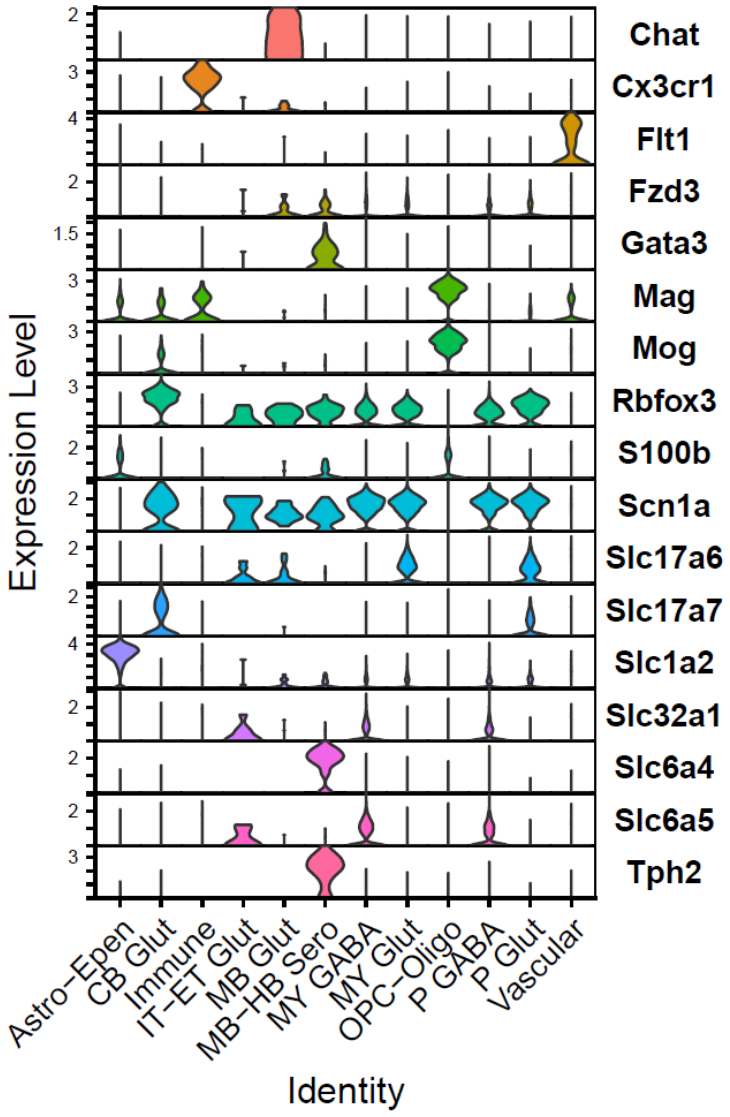
Chat
Cx3cr1
Flt1
Fzd3
Gata3
Mag
Mog
Rbfox3
S100b
Scn1a
Slc17a6
Slc17a7
Slc1a2
Slc32a1
Slc6a4
Slc6a5
Tph2
Expression Level
Astro−Epen
CB Glut
Immune
IT−ET Glut
MB Glut
MB−HB Sero
MY GABA
MY Glut
OPC−Oligo
P GABA
P Glut
Vascular
Identity

Figure S7. Different brainstem cell clusters identified using cell-type specific markers. Brainstem tissue was collected from P40-50 HET and age-matched WT mice. Single-nuclei (sn) suspension of brainstem tissue was obtained by gradient physical dissociation and filtration. snRNA-seq was performed using the 10X Chromium 3' gene expression platform. Brainstem cell types were annotated using Azimuth *via* Seurat's reference mapping framework. (**A**) Violin plots representing the gene expression levels of various cell-type specific markers that helped identify different brainstem cell clusters. Markers that helped identify the brainstem (*Gata3*), voltage-gated sodium channel (*Scn1a*), neurons (*Rbfox3/NeuN*), astrocytes (*Slc1a2*), oligodendrocytes (*Mag/Mog*), immune cells (*Cx3cr1*), vascular endothelial cells (*Flt1*, *Fzd3*), cholinergic neurons (*Chat*),

glial cells (*S100b*), glutamatergic neurons (*Slc17a6*, *Slc17a 7*), inhibitory neurons (*Slc32a1*), serotonergic neurons (*Slc6a4, Tph2*), and, glycinergic neurons (*Slc6a5*), are shown. N= 1/group

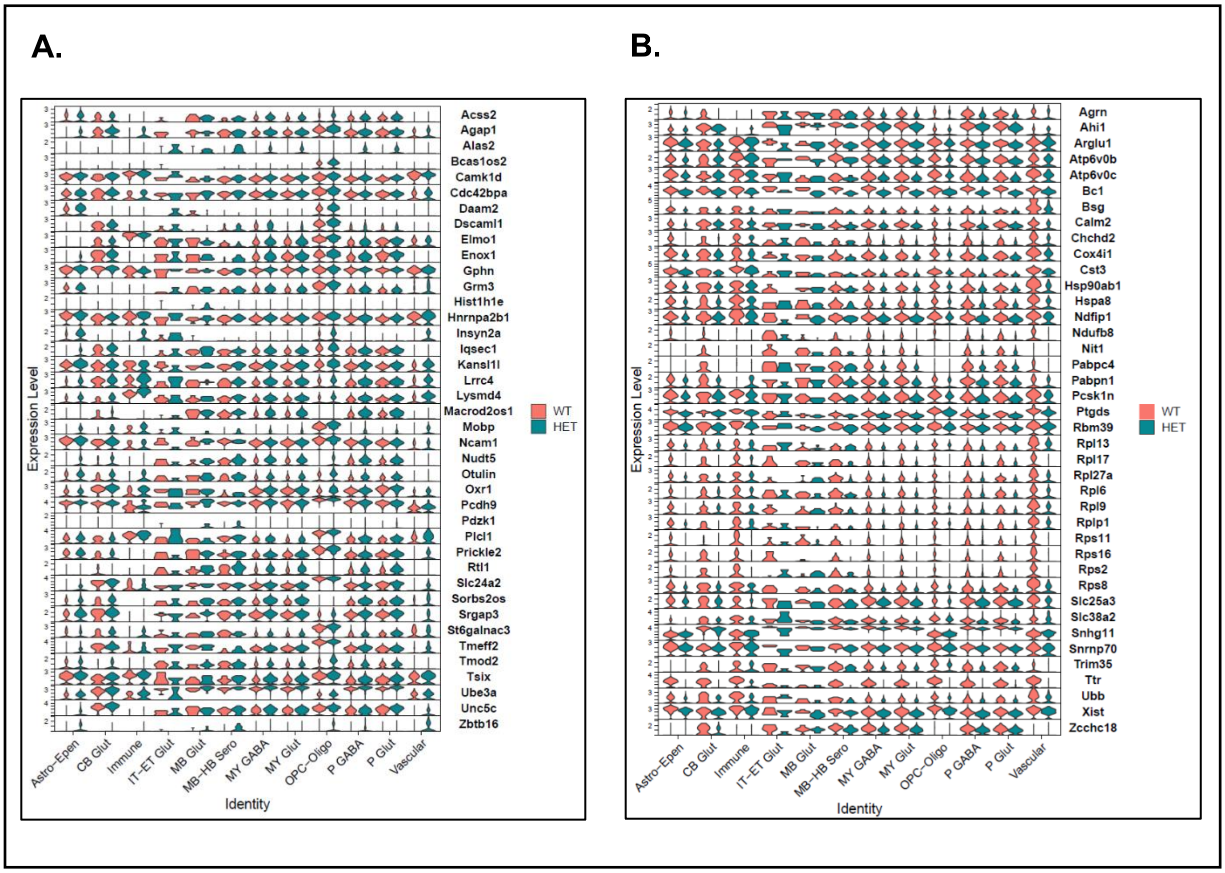

A.
B.
Expression Level
Identity
WT
HET
Acss2
Agap1
Alas2
Bcas1os2
Camk1d
Cdc42bpa
Daam2
Dscaml1
Elmo1
Enox1
Gphn
Grm3
Hist1h1e
Hnrnpa2b1
Insyn2a
Iqsec1
Kansl1l
Lrrc4
Lysmd4
Macrod2os1
Mobp
Ncam1
Nudt5
Otulin
Oxr1
Pcdh9
Pdzk1
Plcl1
Prickle2
Rtl1
Slc24a2
Sorbs2os
Srgap3
St6galnac3
Tmeff2
Tmod2
Tsix
Ube3a
Unc5c
Zbtb16
Agrn
Ahi1
Arglu1
Atp6v0b
Atp6v0c
Bc1
Bsg
Calm2
Chchd2
Cox4i1
Cst3
Hsp90ab1
Hspa8
Ndfip1
Ndufb8
Nit1
Pabpc4
Pabpn1
Pcsk1n
Ptgds
Rbm39
Rpl13
Rpl17
Rpl27a
Rpl6
Rpl9
Rplp1
Rps11
Rps16
Rps2
Rps8
Slc25a3
Slc38a2
Snhg11
Snrnp70
Trim35
Ttr
Ubb
Xist
Zcchc18
Astro-Epen
CB Glut
Immune
IT-ET Glut
MB Glut
MB-HB Sero
MY GABA
MY Glut
OPC-Oligo
P GABA
P Glut
Vascular

Figure S8. Top 40 annotated genes and their expression profiles in P40-50 HET mice brainstem. Brainstem tissue was collected from P40-50 HET and age-matched WT mice. Single-nuclei (sn) suspension of brainstem tissue was obtained by gradient physical dissociation and filtration. snRNA-seq was performed using the 10X Chromium 3' gene expression platform. Brainstem cell types were annotated using Azimuth *via* Seurat's reference mapping framework. (**A**) Top 40 annotated genes that are globally upregulated in HETs. (**B**) Top 40 annotated genes that are globally downregulated in HETs. For the top 40 globally upregulated genes, the cut-off for p value ranges from 2.0e-104 to 7.578e-295, and for the top 40 globally downregulated genes, the cut-off for p value ranges from 2.1e-165 to 0 (anything <1e-300 is set to 0). N= 1/group

Table S1. Differential expression analysis revealed systematic alterations in the kinome. Please refer to the excel tables.
