# Supplementary material for: Multiomic Analyses Reveal Brainstem Metabolic Changes in a Mouse Model of Dravet Syndrome
Source: Cells. 2025 Dec 30;15(1):67. doi: 10.3390/cells15010067 (PMC12785442; doi:10.3390/cells15010067)
Supplement: Supplementary file 1 [file Table S1 - Proteomics Data .pdf › Table S1 - Proteomics Data .pdf]

| Protein Groups Info | | | | | Results, two-tailed two-sample Student's t-test, p < 0.1 | | | | | | | | | | | | Expression columns: log2 MS intensities, median normalized (column), imputed | | | | | | | | | | | |
|---|---|---|---|---|---|---|---|---|---|---|---|---|---|---|---|---|---|---|---|---|---|---|---|---|---|---|---|---|
| Protein.Group | Protein.Ids | Protein.Names | Genes | First.Protein.Description | Student's T-test Significant HET_Brainstem_WT_Brainstem | Student's T-test Significant HET_Cortex_WT_CTX | Student's T-test Significant HET_Hippocampus_WT_HP | -Log Student's T-test p-value HET_Brainstem_WT_BS | Student's T-test Log2 FC HET_Brainstem_WT_BS | Student's T-test Test statistic HET_Brainstem_WT_BS | -Log Student's T-test p-value HET_Cortex_WT_CTX | Student's T-test Log2 FC HET_Cortex_WT_CTX | Student's T-test Test statistic HET_Cortex_WT_CTX | -Log Student's T-test p-value HET_Hippocampus_WT_HP | Student's T-test Log2 FC HET_Hippocampus_WT_HP | Student's T-test Test statistic HET_Hippocampus_WT_HP | HET_Brainstem_1 | HET_Brainstem_2 | HET_Cortex_1 | HET_Cortex_2 | HET_Hippocampus_1 | HET_Hippocampus_2 | WT_Brainstem_1 | WT_Brainstem_2 | WT_Cortex_1 | WT_Cortex_2 | WT_Hippocampus_1 | WT_Hippocampus_2 |
| Q3UHJ0 | Q3UHJ0;Q | AAK1_MOUSE | Aak1 | AP2-associated protein kinase 1 | | | | 0.71653 | 0.22787 | 1.93892 | 0.39916 | 0.14235 | 1.06377 | 0.09889 | -0.0863 | -0.29414 | 6.2573 | 6.02869 | 6.67334 | 6.66056 | 6.39557 | 6.89453 | 5.88782 | 5.94243 | 6.39093 | 6.65826 | 6.88572 | 6.57697 |
| Q80YE4 | Q80YE4 | LMTK1_MOUSE | Aatk | Serine/threonine-protein kinase LMTK1 | | | | 0.35413 | -0.43372 | -0.94981 | 0.00841 | 0.01109 | 0.02713 | 0.583 | -0.99675 | -1.55027 | -4.0007 | -4.79942 | -3.26222 | -3.77495 | -5.39312 | -4.28649 | -4.18777 | -3.74492 | -3.84795 | -3.2114 | -3.51559 | -4.17051 |
| P00520 | P00520 | ABL1_MOUSE | Abl1 | Tyrosine-protein kinase ABL1 | | | | 0.42806 | 0.23512 | 1.13764 | 0.84515 | 0.06512 | 2.35356 | 0.33926 | -0.43268 | -0.91241 | 0.06496 | -0.25236 | 0.68833 | 0.72719 | -0.31375 | 0.63465 | -0.46126 | -0.19638 | 0.62295 | 0.66234 | 0.58963 | 0.59664 |
| Q4JIM5 | Q4JIM5;P0 | ABL2_MOUSE | Abl2 | Tyrosine-protein kinase ABL2 | | + | | 0.2661 | -0.12879 | -0.72887 | 1.79092 | 0.33471 | 7.76495 | 0.29588 | -0.55844 | -0.80358 | 3.1167 | 2.79902 | 4.17156 | 4.17828 | 3.00861 | 4.39684 | 3.00923 | 3.16408 | 3.79724 | 3.88319 | 4.29501 | 4.22732 |
| P61161 | P61161 | ARP2_MOUSE | Actr2 | Actin-related protein 2 | | | | 0.63145 | -0.42292 | -1.68708 | 0.64713 | -0.64514 | -1.73232 | 0.07209 | 0.04087 | 0.218869 | -0.69168 | -0.74047 | 0.66942 | -0.01591 | 1.36321 | 1.53578 | -0.04367 | -0.54265 | 0.82604 | 1.11776 | 1.57423 | 1.24301 |
| P37172 | P37172 | ACVR1_MOUSE | Acvr1 | Activin receptor type-1 | + | + | + | 1.82555 | 0.42737 | 8.08846 | 1.27009 | 0.38824 | 4.13987 | 1.1952 | 0.71869 | 3.767123 | 2.38374 | 2.32638 | 2.41173 | 2.314 | 3.13254 | 2.86205 | 1.88332 | 1.97206 | 1.89458 | 2.05467 | 2.14405 | 2.41316 |
| Q61271 | Q61271 | ACV1B_MOUSE | Acvr1b | Activin receptor type-1B | + | | | 1.64977 | 0.80061 | 6.56891 | 0.0711 | 0.0694 | 0.21605 | 0.27858 | -0.2834 | -0.76022 | 2.53719 | 2.47162 | 4.03336 | 3.80206 | 3.60475 | 2.92113 | 1.58641 | 1.82118 | 3.54862 | 4.148 | 3.39755 | 3.69514 |
| Q8K348 | Q8K348 | ACV1C_MOUSE | Acvr1c | Activin receptor type-1C | | + | + | 0.1455 | 0.16875 | 0.41996 | 3.08954 | 1.64057 | 35.0351 | 1.09812 | 1.44743 | 3.325024 | -3.04892 | -3.83896 | -0.46517 | -0.38458 | -0.68757 | -1.33179 | -3.68633 | -3.53905 | -2.0893 | -2.04159 | -2.74992 | -2.16429 |
| P27038 | P27038;P2 | AVR2A_MOUSE | Acvr2a | Activin receptor type-2A | | | + | 0.3993 | -0.61425 | -1.06411 | 0.81127 | -0.57676 | -2.23992 | 1.0944 | -1.17383 | -3.30893 | -2.97758 | -4.00593 | -2.18668 | -1.74136 | -3.27405 | -2.56943 | -3.13987 | -2.61514 | -1.51659 | -1.25794 | -1.70637 | -1.78945 |
| Q61288 | Q61288 | ACVL1_MOUSE | Acvrl1 | Serine/threonine-protein kinase receptor R3 | | | | NaN | 0 | NaN | 0.74492 | 1.25822 | 2.02667 | NaN | 0 | NaN | -4.10886 | -5.47086 | -4.3057 | -4.07502 | -3.99038 | -4.894 | -5.10506 | -4.16789 | -6.0586 | -4.83855 | -4.6966 | -3.9788 |
| Q9D0L4 | Q9D0L4 | ADCK1_MOUSE | Adck1 | AarF domain-containing protein kinase 1 | | | | 0.04593 | 0.03346 | 0.14263 | 0.09805 | 0.06462 | 0.29184 | 0.28183 | -0.15001 | -0.76836 | 1.78277 | 1.34678 | 2.09538 | 1.97305 | 1.44536 | 1.83105 | 1.61799 | 1.44464 | 1.75678 | 2.18241 | 1.75773 | 1.8187 |
| Q80V03 | Q80V03 | ADCK5_MOUSE | Adck5 | Uncharacterized aarF domain-containing protein kinase 5 | | + | | 0.75773 | -0.92104 | -2.06695 | 1.02592 | -0.70947 | -3.02321 | 0.31219 | -0.49898 | -0.84448 | -1.8085 | -2.29961 | -2.20439 | -2.33422 | -2.48072 | -1.71224 | -1.50486 | -0.76117 | -1.33432 | -1.78535 | -1.14862 | -2.04638 |
| P31750 | P31750 | AKT1_MOUSE | Akt1 | RAC-alpha serine/threonine-protein kinase | | | | 0.14345 | 0.076 | 0.41456 | 0.47398 | -0.17192 | -1.25669 | 0.02231 | 0.01501 | 0.070898 | 0.35722 | 0.02781 | -0.18081 | 0.02135 | 0.15455 | -0.26636 | 0.19701 | 0.03603 | 0.18438 | 0 | -0.09384 | -0.04799 |
| Q60823 | Q60823 | AKT2_MOUSE | Akt2 | RAC-beta serine/threonine-protein kinase | + | + | | 1.00198 | 0.34241 | 2.92777 | 1.81643 | -0.29987 | -8.00201 | 0.1145 | 0.0636 | 0.336912 | 0.91777 | 1.03933 | 1.18499 | 1.12929 | 1.45695 | 1.08175 | 0.53623 | 0.73606 | 1.43193 | 1.48208 | 1.22681 | 1.18468 |
| Q9WUA6 | Q9WUA6 | AKT3_MOUSE | Akt3 | RAC-gamma serine/threonine-protein kinase | | | | 0.26062 | -0.32367 | -0.71509 | 0.44944 | -0.32427 | -1.19279 | 0.23858 | -0.17439 | -0.65957 | -2.73108 | -3.26295 | -2.84268 | -3.22172 | -2.37269 | -2.55577 | -3.03961 | -2.30709 | -2.90284 | -2.51301 | -2.04178 | -2.53789 |
| P97793 | P97793 | ALK_MOUSE | Alk | ALK tyrosine kinase receptor | | + | | 0.21334 | 0.2709 | 0.59558 | 1.02832 | -1.18963 | -3.0329 | 0.90603 | 0.94049 | 2.566604 | -1.85141 | -2.64288 | -4.88725 | -4.23735 | -3.49229 | -4.22185 | -2.2938 | -2.74229 | -3.15299 | -3.59235 | -4.83236 | -4.76277 |
| Q00993 | Q00993 | UFO_MOUSE | Axl | Tyrosine-protein kinase receptor UFO | | | | 0.78601 | 0.46822 | 2.15734 | 0.31897 | 0.20721 | 0.86148 | 0.231 | -0.16637 | -0.6404 | -0.00912 | -0.44291 | -0.09418 | -0.24872 | -0.85455 | -0.69524 | -0.68638 | -0.70209 | -0.60645 | -0.15088 | -0.8558 | -0.36125 |
| O55028 | O55028 | BCKD_MOUSE | Bckdk | [3-methyl-2-oxobutanoate dehydrogenase [lipoamide]] kinase, mitochondrial | | | | 0.21072 | -0.45899 | -0.58891 | 0.13384 | 0.249 | 0.389 | 0.13752 | 0.36567 | 0.398803 | -4.79906 | -4.93769 | -3.69775 | -4.8378 | -2.08098 | -3.9068 | -5.18568 | -3.63309 | -4.80797 | -4.22558 | -3.44518 | -3.27394 |
| Q6PAJ1 | Q6PAJ1 | BCR_MOUSE | Bcr | Breakpoint cluster region protein | | + | + | 0.22379 | -0.12289 | -0.62214 | 1.28362 | 0.32924 | 4.21041 | 1.2626 | -0.47963 | -4.10126 | -2.35385 | -2.67951 | -0.85783 | -0.86704 | -1.77986 | -1.69675 | -2.28196 | -2.50562 | -1.26974 | -1.11362 | -1.36799 | -1.14936 |
| Q91Z96 | Q91Z96 | BMP2K_MOUSE | Bmp2k | BMP-2-inducible protein kinase | | | + | 0.43282 | -0.16406 | -1.14989 | 0.03224 | 0.01718 | 0.10146 | 1.2243 | 0.64713 | 3.908478 | 2.03168 | 1.83078 | 0.66754 | 0.66419 | 1.70782 | 1.55163 | 1.99397 | 2.19662 | 0.47937 | 0.818 | 1.12859 | 0.8366 |
| P36895 | P36895 | BMR1A_MOUSE | Bmpr1a | Bone morphogenetic protein receptor type-1A | + | | | 1.30111 | 0.49948 | 4.30307 | 0.75711 | 0.53726 | 2.06498 | 0.23825 | 0.23485 | 0.658736 | 1.86224 | 1.64784 | 2.25076 | 2.3294 | 1.94656 | 1.47059 | 1.21103 | 1.30008 | 1.49563 | 2.01001 | 1.20826 | 1.73918 |
| P36898 | P36898 | BMR1B_MOUSE | Bmpr1b | Bone morphogenetic protein receptor type-1B | + | | | 2.39422 | 0.48447 | 15.6962 | 0.18001 | 0.05345 | 0.51013 | 0.17485 | 0.05871 | 0.496792 | 0.47531 | 0.4137 | 0.64318 | 0.66815 | 0.46702 | 0.27544 | -0.03804 | -0.0419 | 0.49819 | 0.70624 | 0.24329 | 0.38175 |
| O35607 | O35607 | BMPR2_MOUSE | Bmpr2 | Bone morphogenetic protein receptor type-2 | | | | 0.18212 | 0.11068 | 0.51559 | 0.03465 | 0.01119 | 0.10876 | 0.38726 | -0.53627 | -1.03352 | 1.19106 | 0.82807 | 1.4048 | 1.59392 | -0.15642 | 0.86801 | 1.0135 | 0.78428 | 1.44751 | 1.52882 | 0.80918 | 0.97495 |
| P97504 | P97504 | BMX_MOUSE | Bmx | Cytoplasmic tyrosine-protein kinase BMX | | | | 0.20241 | -0.11241 | -0.5677 | 0.82312 | -0.45135 | -2.27928 | 0.35822 | -0.22689 | -0.96012 | 3.77192 | 3.49062 | 3.26667 | 3.55446 | 3.32185 | 3.58972 | 3.6043 | 3.88307 | 3.72587 | 3.99796 | 3.87738 | 3.48798 |
| P28028 | P28028;P0 | BRAF_MOUSE | Braf | Serine/threonine-protein kinase B-raf | | + | | 0.60721 | 0.06518 | 1.61809 | 1.11815 | -0.22758 | -3.41269 | 0.49574 | -0.3533 | -1.31394 | 1.89108 | 1.97088 | 2.41991 | 2.51746 | 2.06583 | 2.18112 | 1.87136 | 1.86023 | 2.65078 | 2.74174 | 2.21414 | 2.73941 |
| Q7JJ13 | Q7JJ13 | BRD2_MOUSE | Brd2 | Bromodomain-containing protein 2 | + | | | 1.19594 | 0.72822 | 3.77068 | 0.32494 | -0.11446 | -0.87644 | 0.18112 | 0.2075 | 0.513013 | -0.93777 | -0.6615 | -0.50132 | -0.29848 | 0.007 | -0.80135 | -1.39289 | -1.66283 | -0.36772 | -0.20315 | -0.62 | -0.58935 |
| Q8K2F0 | Q9ESU6;Q | BRD3_MOUSE | Brd3 | Bromodomain-containing protein 3 | | | | 0.2897 | -0.26144 | -0.7881 | 0.89463 | -0.38447 | -2.5258 | 0.50807 | 0.25075 | 1.346643 | -2.6991 | -2.04489 | -2.27321 | -2.04263 | -1.86062 | -2.21687 | -2.16575 | -2.05537 | -1.87284 | -1.67406 | -2.23525 | -2.34373 |
| Q9ESU6 | Q9ESU6 | BRD4_MOUSE | Brd4 | Bromodomain-containing protein 4 | | | | 0.27695 | 0.37621 | 0.75612 | 0.9649 | -0.49654 | -2.7843 | 0.25964 | -0.67762 | -0.71263 | -4.2702 | -3.41568 | -3.60052 | -3.32452 | -3.41263 | -5.31334 | -4.47413 | -3.96417 | -2.85303 | -3.07894 | -3.71671 | -3.65402 |
| Q5RJI5 | Q5RJI5 | BRSK1_MOUSE | Brsk1 | Serine/threonine-protein kinase BRSK1 | | | | 0.14663 | 0.07232 | 0.42295 | 0.9292 | 0.58242 | 2.65084 | 0.06226 | 0.03759 | 0.1906 | 2.19378 | 1.95706 | 2.3323 | 2.7694 | 1.52644 | 1.87865 | 2.1265 | 1.8797 | 1.94582 | 1.99103 | 1.75373 | 1.57619 |
| Q69Z98 | Q69Z98 | BRSK2_MOUSE | Brsk2 | Serine/threonine-protein kinase BRSK2 | + | | | 1.18 | 0.40978 | 3.69494 | 0.78871 | 0.12834 | 2.16608 | 0.14441 | 0.02798 | 0.417106 | 4.45643 | 4.23706 | 4.36944 | 4.41473 | 3.84805 | 3.97184 | 3.95335 | 3.92058 | 4.209 | 4.3185 | 3.85611 | 3.90782 |
| P35991 | P35991 | BTK_MOUSE | Btk | Tyrosine-protein kinase BTK | | | | 0.21775 | 0.16156 | 0.60681 | 0.38145 | 0.1952 | 1.01879 | 0.88801 | 0.29612 | 2.502319 | -0.45875 | -0.76975 | -0.20386 | -0.26409 | -0.11072 | -0.3134 | -0.99193 | -0.55969 | -0.61839 | -0.23996 | -0.44707 | -0.56929 |
| Q91YS8 | Q91YS8 | KCC1A_MOUSE | Camk1 | Calcium/calmodulin-dependent protein kinase type 1 | | | | 0.08177 | 0.14371 | 0.24635 | 0.83219 | 0.742 | 2.3097 | 0.82624 | -0.7387 | -2.28972 | -1.53709 | -1.85513 | 0.26736 | 0.89576 | -1.97937 | -2.45281 | -2.4011 | -1.27855 | -0.2274 | -0.09348 | -1.69659 | -1.2582 |
| Q8BW96 | Q8BW96 | KCC1D_MOUSE | Camk1d | Calcium/calmodulin-dependent protein kinase type 1D | | | + | 0.05202 | 0.05994 | 0.16067 | 0.11883 | -0.0444 | -0.34868 | 1.35032 | -0.4149 | -4.57332 | 1.9584 | 1.2236 | 2.48159 | 2.70499 | 2.73815 | 2.58107 | 1.59576 | 1.46636 | 2.69886 | 2.57653 | 3.11993 | 3.0291 |

| Protein Groups Info | | | | | Results, two-tailed two-sample Student's t-test, p < 0.1 | | | | | | | | | | | | Expression columns: log2 MS intensities, median normalized (column), imputed | | | | | | | | | | | |
|---|---|---|---|---|---|---|---|---|---|---|---|---|---|---|---|---|---|---|---|---|---|---|---|---|---|---|---|---|
| Protein.Group | Protein.Ids | Protein.Names | Genes | First.Protein.Description | Student's T-test Significant HET_Brainstem_WT_Brainstem | Student's T-test Significant HET_Cortex_WT_CTX | Student's T-test Significant HET_Hippocampus_WT_HP | -Log Student's T-test p-value HET_Brainstem_WT_BS | Student's T-test Log2 FC HET_Brainstem_WT_BS | Student's T-test Test statistic HET_Brainstem_WT_BS | -Log Student's T-test p-value HET_Cortex_WT_CTX | Student's T-test Log2 FC HET_Cortex_WT_CTX | Student's T-test Test statistic HET_Cortex_WT_CTX | -Log Student's T-test p-value HET_Hippocampus_WT_HP | Student's T-test Log2 FC HET_Hippocampus_WT_HP | Student's T-test Test statistic HET_Hippocampus_WT_HP | HET_Brainstem_1 | HET_Brainstem_2 | HET_Cortex_1 | HET_Cortex_2 | HET_Hippocampus_1 | HET_Hippocampus_2 | WT_Brainstem_1 | WT_Brainstem_2 | WT_Cortex_1 | WT_Cortex_2 | WT_Hippocampus_1 | WT_Hippocampus_2 |
| Q9D9C0 | Q9D9C0 | Q9D9C0_I | 1700109H08Rik | RIKEN cDNA 1700109H08 gene | | | | NaN | 0 | NaN | 0.266024 | 0.262276 | 0.72868 | 0.375355 | 1.899412 | 1.003377 | -4.04985 | -3.48791 | -0.05109 | 0.43645 | 0.402654 | 0.744786 | -3.78561 | -3.75411 | -0.33441 | 0.195224 | 0.559583 | -3.21097 |
| Q8BWE1 | Q8BWE1 | Q8BWE1_I | 1810024B03Rik | RIKEN cDNA 1810024B03 gene | | | | 0.55571 | 0.214197 | 1.475081 | 0.356236 | 0.436719 | 0.955119 | 0.06478 | -0.02395 | -0.19788 | 7.097199 | 6.812057 | 6.271883 | 7.175364 | 5.540672 | 5.443407 | 6.767992 | 6.712869 | 6.357607 | 6.216201 | 5.405139 | 5.626847 |
| G5E8Z3 | G5E8Z3 | G5E8Z3_M | 2310050C09Rik | RIKEN cDNA 2310050C09 gene | | | | 0.957309 | 0.926563 | 2.755533 | 0.023301 | -0.02889 | -0.07398 | 0.615211 | 0.606322 | 1.640726 | -0.96908 | -1.21903 | -2.76247 | -3.46167 | -2.61865 | -1.96966 | -2.33278 | -1.70845 | -2.90919 | -3.25718 | -3.0773 | -2.72365 |
| A0A571BE | A0A571BE | A0A571BE | 2310057N15Rik;Gm5965;Krtap13 | Keratin-associated protein | | | | 0.577334 | 1.621428 | 1.53456 | NaN | 0 | NaN | NaN | 0 | NaN | -2.75713 | -0.76188 | -3.52135 | -3.46077 | -2.95615 | -2.90039 | -3.72901 | -3.03285 | -3.84022 | -3.71708 | -3.41901 | -3.64021 |
| B8JJ69 | B8JJ69 | B8JJ69_MC | 2310061I04Rik | RIKEN cDNA 2310061I04 gene | | | | 0.607319 | 1.406521 | 1.618387 | 0.271381 | 0.312124 | 0.742135 | 0.794082 | 0.333311 | 2.18354 | -1.04334 | -1.22156 | -1.14552 | -1.38195 | -1.52327 | -1.61957 | -1.67446 | -3.40348 | -1.17224 | -1.97948 | -2.04959 | -1.75988 |
| Q3TE80 | Q3TE80 | Q3TE80_M | 2410002F23Rik | RIKEN cDNA 2410002F23 gene | | | | 0.228223 | 0.231243 | 0.633373 | 0.498051 | 0.24846 | 1.320047 | 0.353003 | -0.34661 | -0.94698 | -0.73168 | -1.25772 | 1.114464 | 1.446963 | 0.118049 | 0.273098 | -0.97273 | -1.47916 | 0.944004 | 1.120504 | 0.18447 | 0.899903 |
| Q8R0P4 | Q8R0P4 | AAMDC_M | Aamdc | Mth938 domain-containing protein | | | | 0.707031 | -0.6078 | -1.91 | 0.622546 | -0.57784 | -1.66159 | 0.220213 | -0.48506 | -0.61306 | 0.217238 | -0.33863 | -1.42271 | -1.01582 | -0.95438 | 0.39213 | 0.392128 | 0.702094 | -0.35938 | -0.92347 | 0.619568 | -0.21169 |
| Q8BGQ7 | Q8BGQ7 | SYAC_MOI | Aars1 | Alanine--tRNA ligase, cytoplasmic | | | | 0.473595 | -0.25241 | -1.25568 | 0.068897 | -0.12356 | -0.20973 | 0.810308 | 0.291744 | 2.236754 | 0.198925 | 0.15624 | 0.579905 | -0.43207 | 0.820738 | 0.63297 | 0.230114 | 0.629879 | -0.1043 | 0.499259 | 0.525655 | 0.344566 |
| Q7TN98 | Q7TN98 | CPEB4_MC | Cpeb4 | Cytoplasmic polyadenylation element-binding protein 4 | + | | | 1.332686 | 3.215012 | 4.474838 | 0.75204 | 1.816427 | 2.049008 | 0.407692 | -1.00731 | -1.08551 | -0.79968 | 0.456834 | -0.44813 | -0.69519 | -2.77475 | -0.91975 | -3.03789 | -3.73498 | -1.51024 | -3.26593 | -0.86919 | -0.81069 |
| P06795 | P21440;P( | MDR1B_M | Abcb1b | ATP-dependent translocase ABCB1 | | + | | 0.977956 | -1.25112 | -2.83423 | 1.503097 | 1.281217 | 5.509724 | 0.184013 | 0.605925 | 0.520475 | -3.08036 | -2.26041 | -2.10863 | -2.57368 | -3.27697 | -1.40214 | -1.58292 | -1.2556 | -3.62013 | -3.62462 | -2.25515 | -3.63582 |
| Q8BNU0 | Q8BNU0 | ARMC6_M | Armc6 | Armadillo repeat-containing protein 6 | + | | | 1.46864 | 3.049317 | 5.284742 | 0.243988 | 0.252271 | 0.673217 | 0.001953 | 0.00485 | 0.006346 | -0.3185 | -1.37308 | -0.53911 | -1.07034 | 0.090512 | -1.43652 | -4.12942 | -3.6608 | -1.32132 | -0.79267 | -0.71345 | -0.64225 |
| Q9CXJ4 | Q9CXJ4 | MITOS_MC | Abcb8 | Mitochondrial potassium channel ATP-binding subunit | | | | 0.498719 | -1.70693 | -1.32182 | 0.353991 | 1.025365 | 0.949464 | 0.001494 | 0.001686 | 0.004857 | -3.0535 | -3.96666 | -1.75575 | -0.94268 | -0.88023 | -1.52322 | -0.5952 | -3.01109 | -3.37508 | -1.37408 | -1.07264 | -1.33418 |
| Q9JJ59 | Q9JJ59 | ABCB9_MC | Abcb9 | ABC-type oligopeptide transporter ABCB9 | + | | | 1.002629 | 0.787829 | 2.930335 | 0.093389 | 0.074663 | 0.278905 | 0.045807 | -0.1314 | -0.14228 | 0.260946 | -0.13623 | -1.5522 | -1.02587 | -2.15893 | -2.33824 | -0.54424 | -0.9067 | -1.31464 | -1.41275 | -3.03633 | -1.19805 |
| P97952 | P97952 | SCN1B_MC | Scn1b | Sodium channel subunit beta-1 | + | | | 1.959566 | 3.045614 | 9.466404 | 0.044712 | -0.06953 | -0.13902 | 0.027282 | -0.02736 | -0.08627 | -0.39348 | -0.64078 | -0.9785 | -1.73815 | -1.15129 | -0.52507 | -3.26573 | -3.85976 | -0.96347 | -1.61413 | -0.76041 | -0.86124 |
| P55096 | P55096 | ABCD3_MC | Abcd3 | ATP-binding cassette sub-family D member 3 | | | | 0.075764 | 0.029864 | 0.229349 | 0.253968 | 0.092385 | 0.698364 | 0.493253 | 0.110704 | 1.307359 | 1.034059 | 1.089102 | 0.132147 | 0.317738 | 0.586514 | 0.424836 | 1.158987 | 0.904446 | 0.038275 | 0.22684 | 0.420177 | 0.369765 |
| Q80ZK0 | Q80ZK0 | RT10_MOU | Mrps10 | Small ribosomal subunit protein uS10m | + | | | 2.828405 | 2.984938 | 25.92498 | 0.1044 | 0.185285 | 0.309332 | 0.040229 | 0.22403 | 0.125609 | -0.50652 | -0.64822 | -0.87922 | -0.47636 | -2.8729 | -0.7004 | -3.65306 | -3.47155 | -0.29898 | -1.42718 | -3.42529 | -0.59607 |
| Q6P542 | Q6P542 | ABCF1_MC | Abcf1 | ATP-binding cassette sub-family F member 1 | | | | 0.706942 | 1.675167 | 1.909727 | 0.329611 | 0.175237 | 0.888176 | 0.623814 | 0.279342 | 1.665213 | -0.1218 | -1.131 | -0.99084 | -1.22716 | -1.16276 | -1.10016 | -1.58406 | -3.01907 | -1.44224 | -1.12623 | -1.57561 | -1.246 |
| Q99LE6 | Q99LE6 | ABCF2_MC | Abcf2 | ATP-binding cassette sub-family F member 2 | | | | 0.061004 | -0.30378 | -0.18694 | 0.328525 | 0.350512 | 0.885449 | 0.867519 | -0.51008 | -2.43048 | -3.84621 | -1.58303 | -1.10477 | -1.28448 | -0.96126 | -1.25213 | -1.24458 | -3.5771 | -1.93066 | -1.15961 | -0.74792 | -0.4453 |
| Q99LR1 | Q99LR1 | ABD12_MC | Abhd12 | Lysophosphatidylserine lipase ABHD12 | + | | | 1.121113 | -0.7397 | -3.42579 | 0.231428 | 0.183453 | 0.641487 | 0.181838 | 0.116793 | 0.514861 | -0.19226 | -0.51974 | 0.316824 | -0.24002 | -0.04043 | 0.198143 | 0.242951 | 0.524447 | -0.07972 | -0.21038 | 0.155009 | -0.23089 |
| Q9Z1Q2 | Q9Z1Q2 | ABHGA_MC | Abhd16a | Phosphatidylserine lipase ABHD16A | | | | 0.398476 | -0.17261 | -1.06202 | 0.801342 | -0.56737 | -2.20726 | 0.354011 | -0.16125 | -0.94952 | -0.01047 | 0.027336 | -0.56114 | -0.9324 | -0.37163 | -0.06672 | 0.342467 | 0.019616 | -0.0016 | -0.35721 | 0.016887 | -0.13274 |
| Q91ZH7 | Q91ZH7 | ABHD3_MC | Abhd3 | Phospholipase ABHD3 | | + | | 0.030062 | -0.02704 | -0.09479 | 1.259879 | -0.93589 | -4.0873 | 0.061377 | -0.15666 | -0.18802 | -1.78934 | -1.22155 | -2.11851 | -2.56004 | -2.83922 | -1.42766 | -1.45036 | -1.50644 | -1.34261 | -1.46416 | -2.41958 | -1.53399 |
| Q8CBW3 | P62484;Q | ABI1_MOU | Abi1 | Abl interactor 1 | | | | 0.071013 | 0.031533 | 0.215796 | 0.534411 | -0.18009 | -1.41724 | 0.964824 | -0.24961 | -2.784 | 0.683007 | 0.637468 | 2.349554 | 2.536718 | 2.512667 | 2.691166 | 0.773042 | 0.484368 | 2.537264 | 2.709184 | 2.860062 | 2.842983 |
| P62484 | P62484 | ABI2_MOU | Abi2 | Abl interactor 2 | + | | | 1.157207 | 1.349679 | 3.58884 | 0.088779 | -0.08057 | -0.26605 | 0.419503 | -0.35874 | -1.1157 | -0.73781 | 0.007863 | 1.747917 | 1.777029 | 1.159386 | 1.79736 | -1.7639 | -1.66541 | 1.540553 | 2.145532 | 1.796686 | 1.877539 |
| Q8K4G5 | Q8K4G5 | ABLM1_MC | Ablim1 | Actin-binding LIM protein 1 | | + | | 0.083486 | -0.30096 | -0.2512 | 1.246691 | -0.55933 | -4.02025 | 0.912165 | 0.213984 | 2.58873 | -2.97463 | -0.61509 | -1.23376 | -1.18207 | -0.65794 | -0.80877 | -1.70278 | -1.28502 | -0.51188 | -0.78529 | -0.98117 | -0.91351 |
| Q5SSL4 | Q5SSL4 | ABR_MOU | Abr | Active breakpoint cluster region-related protein | | + | | 0.231243 | 0.384567 | 0.641018 | 1.417114 | 0.246235 | 4.963694 | 0.499562 | 0.582948 | 1.324049 | 0.728111 | -0.30061 | 0.899857 | 0.925332 | 1.812202 | 0.944897 | 0.137968 | -0.47961 | 0.618415 | 0.714303 | 0.871689 | 0.719514 |
| Q4KML4 | Q4KML4 | ABRAL_MC | Abracl | Costars family protein ABRACL | | | | 0.992744 | -2.53743 | -2.89156 | 0.238825 | -0.66609 | -0.66019 | 0.035097 | 0.074991 | 0.110126 | -2.76001 | -4.42789 | -2.14112 | -3.84955 | -0.67356 | -0.75199 | -1.32966 | -0.78339 | -1.79232 | -2.86616 | -0.10794 | -1.46759 |
| Q921H8 | Q8VCH0;( | THIKA_MO | Acaa1a | 3-ketoacyl-CoA thiolase A, peroxisomal | | | | 0.34001 | 0.735454 | 0.914294 | 0.624605 | 0.200549 | 1.66747 | 0.268197 | 0.221929 | 0.734139 | -1.81059 | -1.47926 | -1.76427 | -2.00093 | -1.62461 | -1.50445 | -1.59323 | -3.16753 | -2.06161 | -2.10469 | -1.49019 | -2.08273 |
| Q922C1 | Q922C1 | CS044_MC | N/A | Uncharacterized protein C19orf44 homolog | + | | | 1.932329 | 2.943286 | 9.169209 | 0.554267 | 1.800538 | 1.471139 | 0.384843 | 0.492031 | 1.027401 | -0.55053 | -0.55103 | -1.13673 | -0.10924 | -0.34877 | -0.41204 | -3.17307 | -3.81506 | -3.53438 | -1.31266 | -1.3503 | -0.39457 |
| Q5SWU9 | Q5SWU9 | ACACA_MC | Acaca | Acetyl-CoA carboxylase 1 | | | | 0.335744 | 0.068113 | 0.903575 | 0.638429 | 0.164247 | 1.707145 | 0.120717 | -0.11097 | -0.35377 | -0.88614 | -0.76117 | -0.44871 | -0.33996 | -0.65849 | -0.99177 | -0.8496 | -0.93394 | -0.63795 | -0.47921 | -0.97992 | -0.4484 |
| P63080 | P63080 | GBRB3_MC | Gabrb3 | Gamma-aminobutyric acid receptor subunit beta-3 | + | | | 1.541922 | 2.939848 | 5.773623 | 0.179723 | 0.170265 | 0.509399 | 0.044913 | 0.07444 | 0.13962 | -1.04208 | -0.20545 | -0.06598 | -0.13654 | -0.22625 | 0.828772 | -3.85393 | -3.2733 | -0.6039 | 0.060855 | 0.149441 | 0.304197 |
| Q80XL6 | Q80XL6 | ACD11_MC | Acad11 | Acyl-CoA dehydrogenase family member 11 | + | | | 1.063245 | 1.13976 | 3.176513 | 0.23372 | -0.3527 | -0.64728 | 0.698907 | -0.55567 | -1.88543 | 2.885337 | 3.454997 | -0.05059 | 0.706448 | 0.260522 | 0.012858 | 2.248617 | 1.812197 | 1.072582 | 0.288674 | 0.424921 | 0.959806 |
| Q9D7B6 | Q9D7B6 | ACAD8_MC | Acad8 | Isobutyryl-CoA dehydrogenase, mitochondrial | | | | 0.007034 | -0.00609 | -0.02272 | 0.84637 | -0.24922 | -2.35773 | 0.376446 | -0.06738 | -1.00614 | -0.24466 | 0.23808 | -0.50844 | -0.33036 | 0.524578 | 0.658512 | -0.11377 | 0.119374 | -0.11323 | -0.22715 | 0.659693 | 0.658161 |
| Q8JZN5 | Q8JZN5 | ACAD9_MC | Acad9 | Complex I assembly factor ACAD9, mitochondrial | + | + | | 1.246264 | -0.55012 | -4.01809 | 1.702025 | -0.27929 | -6.98984 | 0.41428 | -0.29488 | -1.10233 | 0.397376 | 0.657519 | 0.086442 | 0.016927 | -0.03755 | 0.38972 | 1.120297 | 1.034836 | 0.311263 | 0.350679 | 0.631973 | 0.309968 |
| P51174 | P51174 | ACADL_MC | Acadl | Long-chain specific acyl-CoA dehydrogenase, mitochondrial | | + | | 0.748094 | 0.198792 | 2.036619 | 1.141671 | -0.45405 | -3.51792 | 0.039635 | 0.057659 | 0.123822 | 3.159555 | 3.354589 | 1.410413 | 1.297196 | 1.694852 | 2.617482 | 3.06251 | 3.05405 | 1.691864 | 1.923848 | 2.162001 | 2.035014 |
| P45952 | P45952 | ACADM_M | Acadm | Medium-chain specific acyl-CoA dehydrogenase, mitochondrial | | | | 0.804291 | -0.56791 | -2.21693 | 0.430995 | -1.42675 | -1.14519 | 0.034322 | -0.05215 | -0.10778 | -0.94233 | -0.67888 | -0.68675 | -3.1759 | -1.0086 | -0.04097 | -0.02298 | -0.4624 | -0.56124 | -0.4479 | -0.4802 | -0.46507 |
| A2AR50 | A2AR50 | RGPS1_MC | Ralgps1 | Ras-specific guanine nucleotide-releasing factor RalGPS1 | + | | | 1.408343 | 2.852339 | 4.910809 | 0.077352 | -0.09823 | -0.23386 | 0.043713 | -0.0228 | -0.13604 | -0.7673 | -1.82288 | -0.39489 | -0.32135 | -0.74753 | -0.66138 | -4.38992 | -3.90495 | 0.158526 | -0.67831 | -0.5197 | -0.84361 |
| P50544 | P50544 | ACADV_MC | Acadvl | Very long-chain specific acyl-CoA dehydrogenase, mitochondrial | | | | 0.819314 | -0.88211 | -2.26661 | 0.808879 | -0.56867 | -2.23204 | 0.023415 | -0.05299 | -0.07433 | -0.56447 | 0.16196 | -0.66915 | -1.16744 | -0.81029 | 0.333112 | 0.541095 | 0.820616 | -0.29636 | -0.40289 | 0.240273 | -0.61147 |
| Q6ZQK5 | Q6ZQK5 | ACAP2_MC | Acap2 | Arf-GAP with coiled-coil, ANK repeat and PH domain-containing protein 2 | | | | 0.222445 | 0.738288 | 0.618726 | 0.312347 | -0.11443 | -0.84487 | 0.422136 | 0.444109 | 1.122445 | -1.56114 | -1.36328 | -0.2385 | -0.17393 | 0.067459 | -0.25026 | -1.01137 | -3.38963 | -0.22332 | 0.039754 | -0.89788 | -0.17314 |
| Q6NXL5 | Q6NXL5 | Q6NXL5_M | Acap3 | Arf-GAP with coiled-coil, ANK repeat and PH domain-containing protein | | | | NaN | 0 | NaN | 0.225271 | 0.489005 | 0.625894 | 0.285781 | -0.75163 | -0.77827 | -3.64977 | -3.22878 | -1.88395 | -1.31796 | -1.19799 | -3.12277 | -3.85405 | -3.50872 | -2.8182 | -1.36173 | -1.48954 | -1.32795 |
| Q9Z2V9 | Q9Z2V9 | CCNI_MOI | Ccni | Cyclin-I | + | | | 1.812744 | 2.792686 | 7.96734 | 0.888697 | 1.563956 | 2.504745 | 0.071719 | 0.1005 | 0.217816 | -0.72286 | -0.97666 | -2.4271 | -1.20136 | -2.67147 | -3.55716 | -3.96918 | -3.3157 | -3.25876 | -3.49761 | -3.08528 | -3.34435 |
| Q64519 | Q64519 | SDC3_MO | Sdc3 | Syndecan-3 | + | | + | 1.533567 | 2.725281 | 5.715884 | 0.480435 | 0.427284 | 1.273605 | 2.657253 | 2.057499 | 21.27691 | -0.42676 | -1.32594 | -1.29741 | -0.6274 | -1.38327 | -1.30082 | -3.44289 | -3.76037 | -1.40776 | -1.37162 | -3.48702 | -3.31207 |
| P21836 | P21836 | ACES_MOI | Ache | Acetylcholinesterase | | + | | 0.165158 | -0.82158 | -0.47159 | 2.047684 | 1.057837 | 10.49321 | 0.348403 | 1.708466 | 0.9354 | -3.60033 | -0.20807 | 0.891691 | 1.077033 | -0.4009 | 0.504738 | -1.48045 | -0.6848 | -0.03379 | -0.11316 | -3.42598 | 0.112884 |
| Q9JIX8 | Q9JIX8 | ACINU_MC | Acin1 | Apoptotic chromatin condensation inducer in the nucleus | | | | 0.225585 | -0.44746 | -0.62669 | 0.416838 | 0.474318 | 1.108875 | 0.852971 | 1.105559 | 2.380288 | -1.259 | -0.91056 | -1.66536 | -1.28362 | -0.47826 | -0.85287 | 0.055091 | -1.32974 | -2.3316 | -1.566 | -1.3461 | -2.19615 |
| Q91V92 | Q91V92 | ACLY_MOI | Acly | ATP-citrate synthase | | | | 0.350085 | -0.17653 | -0.93963 | 0.306377 | -0.12788 | -0.8299 | 0.685639 | 0.37722 | 1.845628 | 1.920761 | 1.801548 | 1.604462 | 1.322241 | 2.050685 | 1.642753 | 2.215854 | 1.859519 | 1.529321 | 1.653151 | 1.456402 | 1.482595 |
| P28271 | P28271 | ACOHC_M | Aco1 | Cytoplasmic aconitate hydratase | | | + | 0.715708 | -2.74441 | -1.93641 | 0.379454 | -0.55686 | -1.01375 | 1.156544 | -0.49405 | -3.58579 | -0.18668 | -3.01569 | 0.380731 | -0.60913 | 0.419354 | 0.441644 | 1.231666 | 1.054795 | 0.204374 | 0.680942 | 1.061874 | 0.787219 |
| Q99KI0 | Q99KI0 | ACON_MC | Aco2 | Aconitate hydratase, mitochondrial | | | | 0.984333 | -1.18407 | -2.85885 | 0.04433 | -0.1102 | -0.13788 | 0.015087 | -0.03143 | -0.04831 | 1.636607 | 2.340438 | 3.067193 | 1.486521 | 2.367972 | 3.054872 | 3.390995 | 2.954195 | 2.267883 | 2.50624 | 3.295466 | 2.190246 |
| O55137 | Q9QYR9;( | ACOT1_MC | Acot1 | Acyl-coenzyme A thioesterase 1 | | | | 0.913421 | -1.2755 | -2.59328 | 0.502921 | -0.57795 | -1.33296 | 0.108405 | -0.13336 | -0.32031 | -2.03454 | -1.67448 | -1.12826 | -1.99464 | -1.11022 | -0.28111 | -0.1213 | -1.03673 | -0.96492 | -1.00207 | -0.60091 | -0.5237 |
| Q8VHQ9 | Q8VHQ9 | ACO11_MC | Acot11 | Acyl-coenzyme A thioesterase 11 | | | | 0.269708 | 0.291314 | 0.737934 | 0.442655 | -0.11581 | -1.17524 | 0.725835 | -0.07964 | -1.96747 | 1.699575 | 1.79036 | 0.40669 | 0.312193 | 0.582324 | 0.516387 | 1.845805 | 1.061501 | 0.561726 | 0.388776 | 0.60551 | 0.652481 |
| Q9CQR4 | Q9CQR4 | ACO13_MC | Acot13 | Acyl-coenzyme A thioesterase 13 | | | | 0.986923 | -1.34915 | -2.86889 | 0.027551 | -0.05419 | -0.08709 | 0.108275 | -0.30282 | -0.31995 | -0.70562 | 0.107819 | 1.245647 | 0.052914 | 0.520066 | 2.172036 | 0.814174 | 1.286334 | 0.526191 | 0.880743 | 2.110958 | 1.186782 |
| Q91V12 | Q91V12 | BACH_MO | Acot7 | Cytosolic acyl coenzyme A thioester hydrolase | | | | 0.361342 | -0.16062 | -0.96799 | 0.234569 | 0.576784 | 0.649432 | 0.06454 | -0.09882 | -0.19718 | 0.618772 | 0.847426 | 1.376238 | -0.35136 | 0.44984 | 0.834305 | 0.773458 | 1.013985 | -0.27084 | 0.142147 | 1.203704 | 0.278077 |
| Q9R0X4 | Q9R0X4;Q | ACOT9_MC | Acot9 | Acyl-coenzyme A thioesterase 9, mitochondrial | | | | 0.496835 | -0.42681 | -1.31683 | 0.060857 | 0.09764 | 0.186515 | 0.948452 | 0.289173 | 2.722246 | -1.44078 | -0.85943 | -0.85938 | -1.71848 | -0.6598 | -0.83807 | -0.57989 | -0.86669 | -1.6858 | -1.08734 | -0.98032 | -1.09589 |
| Q9D358 | Q9D358 | PPAC_MO | Acp1 | Low molecular weight phosphotyrosine protein phosphatase | | | | 0.676675 | -0.24569 | -1.81896 | 0.97991 | -0.46845 | -2.84175 | 0.062164 | 0.04311 | 0.190308 | 2.620281 | 2.392711 | 2.514482 | 2.339584 | 2.917025 | 3.364548 | 2.824979 | 2.679403 | 3.035217 | 2.755744 | 3.132956 | 3.062398 |
| Q99PU5 | Q99PU5 | ACBG1_MC | Acsbg1 | Long-chain-fatty-acid--CoA ligase ACSBG1 | | | | 0.477243 | -0.34437 | -1.26523 | 0.768022 | -0.38057 | -2.0996 | 0.583736 | -0.12945 | -1.55232 | 0.97293 | 1.282805 | 0.140264 | -0.09084 | 0.290974 | 0.390988 | 1.696011 | 1.24846 | 0.265629 | 0.544927 | 0.53717 | 0.403698 |
| Q8VCW8 | Q8VCW8 | ACSF2_MC | Acsf2 | Medium-chain acyl-CoA ligase ACSF2, mitochondrial | | | | 0.233064 | -0.20637 | -0.64563 | 0.750326 | -0.67145 | -2.04362 | 0.254056 | 0.125902 | 0.698584 | -0.64731 | -0.90943 | -1.60886 | -2.26577 | -0.70943 | -0.65193 | -0.86354 | -0.28047 | -1.25772 | -1.27402 | -0.62866 | -0.9845 |
| P41216 | P41216 | ACSL1_MC | Acsl1 | Long-chain-fatty-acid--CoA ligase 1 | | | | NaN | 0 | NaN | 0.031726 | -0.07883 | -0.09988 | 0.231258 | 0.351058 | 0.641057 | -3.30847 | -3.46314 | -2.89216 | -4.07313 | -3.33743 | -4.03308 | -3.10949 | -3.44375 | -3.92757 | -2.88006 | -3.61334 | -4.45929 |
| Q9CZW4 | Q9CZW4 | ACSL3_MC | Acsl3 | Fatty acid CoA ligase Acsl3 | | | | 0.765462 | 0.942933 | 2.091447 | 0.023075 | 0.023295 | 0.073277 | 0.476663 | 0.158436 | 1.263709 | -1.49701 | -1.71443 | -1.82318 | -2.10654 | -1.84266 | -1.59218 | -2.9862 | -2.11111 | -1.70356 | -2.27274 | -1.88168 | -1.87002 |
| Q8JZR0 | Q8JZR0 | ACSL5_MC | Acsl5 | Long-chain-fatty-acid--CoA ligase 5 | | | | NaN | 0 | NaN | 0.47832 | 0.802141 | 1.268055 | 0.074037 | 0.108291 | 0.224432 | -2.81706 | -3.44667 | -1.23024 | -1.52375 | -1.40704 | -2.36854 | -4.23772 | -2.99588 | -2.79445 | -1.56382 | -1.95489 | -2.03726 |
| Q91WC3 | Q91WC3 | ACSL6_MC | Acsl6 | Long-chain-fatty-acid--CoA ligase 6 | | | | 0.071617 | 0.036279 | 0.217523 | 0.645055 | -0.21282 | -1.7263 | 0.521976 | 0.100754 | 1.383785 | 3.415088 | 3.281332 | 2.418913 | 2.269593 | 2.659481 | 2.514386 | 3.464717 | 3.159145 | 2.65517 | 2.45897 | 2.492356 | 2.480003 |
| P62737;P6 | P63268;P6 | ACTA_MOI | Acta2;Actg2 | Actin, aortic smooth muscle | | | | 0.283008 | 0.556056 | 0.771312 | 0.888556 | -0.44645 | -2.50424 | 0.083606 | 0.180383 | 0.251534 | 2.972389 | 1.624864 | 2.060259 | 2.216198 | 2.982585 | 3.549083 | 1.999035 | 1.486105 | 2.424355 | 2.745001 | 3.744275 | 2.426626 |
| P60710;P6 | P60710;P6 | ACTB_MOI | Actb;Actg1 | Actin, cytoplasmic 1 | | + | | 0.273667 | -0.1796 | -0.74787 | 1.248604 | -0.65358 | -4.02992 | 0.008456 | 0.014932 | 0.027276 | 6.218148 | 6.160781 | 6.739212 | 6.426071 | 6.831097 | 7.813028 | 6.607498 | 6.130636 | 7.278515 | 7.193927 | 7.549338 | 7.064921 |
| Q8BFZ3 | Q8BFZ3 | ACTBL_MC | Actbl2 | Beta-actin-like protein 2 | | + | | 0.043812 | -0.02391 | -0.13633 | 2.88387 | -8.81426 | -27.6382 | 0.405106 | -4.88007 | -1.07891 | 4.761962 | 4.761001 | -2.88769 | -3.17522 | 5.765705 | -3.27194 | 4.960798 | 4.609993 | 5.498137 | 6.067483 | 6.324788 | 5.929112 |
| Q99MR0 | Q99MR0 | ACL6B_MC | Actl6b | Actin-like protein 6B | | | | 0.385674 | 0.111319 | 1.029509 | 0.196048 | -0.0998 | -0.55142 | 0.282743 | -0.27285 | -0.77065 | -1.18084 | -1.11918 | -0.39269 | -0.44771 | -0.79184 | -0.86278 | -1.15769 | -1.36497 | -0.49928 | -0.14153 | -0.90673 | -0.20218 |
| Q7TPR4 | Q7TPR4 | ACTN1_MC | Actn1 | Alpha-actinin-1 | | + | | 0.055974 | 0.017414 | 0.172277 | 1.648663 | -0.24065 | -6.56024 | 0.669288 | 0.908324 | 1.797122 | -0.64994 | -0.46664 | 0.36637 | 0.300574 | 2.025052 | 1.058177 | -0.53308 | -0.61834 | 0.557893 | 0.590353 | 0.780768 | 0.485813 |
| P57780 | P57780 | ACTN4_MC | Actn4 | Alpha-actinin-4 | | + | | 0.610935 | -1.17673 | -1.62861 | 1.572346 | -0.91186 | -5.98845 | 0.447796 | 0.586985 | 1.188536 | -3.12019 | -2.62444 | -2.54604 | -2.31949 | -0.30503 | -1.25411 | -1.0169 | -2.37428 | -1.62267 | -1.41915 | -1.22973 | -1.50339 |
| Q9QZB7 | Q9QZB7 | ARP10_MC | Actr10 | Actin-related protein 10 | | | | 0.652878 | 0.215343 | 1.749026 | 0.329313 | 0.244003 | 0.887428 | 0.652765 | 0.204219 | 1.748697 | -0.90928 | -1.05678 | -0.04939 | -0.5993 | -0.69874 | -0.51966 | -1.09978 | -1.29696 | -0.56758 | -0.56912 | -0.88839 | -0.73844 |
| P61164 | P61164 | ACTZ_MOI | Actr1a | Alpha-centractin | | | | 0.006711 | -0.01897 | -0.02169 | 0.368483 | 1.213233 | 0.986009 | 0.469314 | 0.987715 | 1.244482 | -3.60775 | -1.98306 | -1.71474 | -0.80321 | -1.44347 | -1.02401 | -2.45208 | -3.10079 | -3.61513 | -1.32928 | -2.98692 | -1.45599 |
| Q8R5C5 | Q8R5C5;P | ACTY_MOI | Actr1b | Beta-centractin | | | | 0.273746 | -0.27492 | -0.74807 | 0.242822 | -0.05856 | -0.67027 | 0.205605 | 0.11335 | 0.57587 | 1.255814 | 1.533985 | 1.946412 | 1.829311 | 1.567082 | 1.817 | 1.329652 | 2.009982 | 1.881573 | 2.011276 | 1.426612 | 1.730771 |
| Q99JY9 | Q99JY9 | ARP3_MOI | Actr3 | Actin-related protein 3 | + | | | 1.113729 | 0.161729 | 3.393169 | 0.389682 | -0.3092 | -1.03968 | 0.052762 | -0.02963 | -0.16286 | 1.081074 | 1.134016 | 1.945236 | 1.565694 | 2.279746 | 2.641671 | 0.985453 | 0.906179 | 1.83568 | 2.293656 | 2.509176 | 2.4715 |
| Q641P0 | Q641P0 | ARP3B_MC | Actr3b | Actin-related protein 3B | | | | 0.332349 | 1.884336 | 0.895048 | 0.541698 | -0.42707 | -1.43695 | 0.175679 | -0.16257 | -0.49893 | -0.30563 | 0.778383 | 1.52474 | 0.938119 | 1.287582 | 1.858551 | 0.386363 | -3.68228 | 1.61054 | 1.706456 | 1.578591 | 1.892673 |
| P56375 | P56375 | ACYP2_MC | Acyp2 | Acylphosphatase-2 | | | | 0.477257 | -0.16397 | -1.26527 | 0.214246 | 0.120877 | 0.597895 | 0.801001 | 0.236375 | 2.206141 | 2.074237 | 2.117172 | 0.507934 | 0.908903 | 0.899573 | 1.052942 | 2.387477 | 2.131872 | 0.613595 | 0.561489 | 0.665053 | 0.814712 |
| Q9R1V4 | Q9R1V4 | ADA11_MC | Adam11 | Disintegrin and metalloproteinase domain-containing protein 11 | | | | 0.407698 | 1.332152 | 1.085521 | 0.37494 | 0.576458 | 1.002328 | 0.001698 | 0.003196 | 0.005518 | -3.43442 | -1.17448 | -1.19833 | -1.25151 | -1.47618 | -0.57541 | -3.15787 | -4.11533 | -2.37588 | -1.22687 | -0.66498 | -1.393 |
| Q9R1V6 | Q9R1V6 | ADA22_MC | Adam22 | Disintegrin and metalloproteinase domain-containing protein 22 | | | | 0.219919 | -0.14452 | -0.61232 | 0.499936 | -0.04398 | -1.32504 | 0.114027 | 0.132272 | 0.335638 | 0.375328 | 0.440808 | 1.274102 | 1.318566 | 1.358987 | 0.718915 | 0.786333 | 0.318846 | 1.364967 | 1.315669 | 0.676714 | 1.136644 |
| Q9R1V7 | Q9R1V7 | ADA23_MC | Adam23 | Disintegrin and metalloproteinase domain-containing protein 23 | + | | | 1.002753 | -0.73336 | -2.93082 | 0.39883 | -0.30146 | -1.06292 | 0.05358 | -0.05148 | -0.16526 | -1.41885 | -1.34841 | -0.83044 | -1.16434 | -0.50139 | -1.09977 | -0.40254 | -0.898 | -0.9252 | -0.46667 | -0.52422 | -0.88417 |
| O88444 | O88444 | ADCY1_MC | Adcy1 | Adenylate cyclase type 1 | | + | | 0.216125 | 0.830455 | 0.602674 | 1.678677 | -0.98381 | -6.79873 | 0.357728 | -0.35849 | -0.95888 | -2.9339 | -1.59648 | -1.74806 | -1.91832 | -1.44196 | -1.94891 | -4.30046 | -1.89083 | -0.73237 | -0.96639 | -1.61176 | -1.06214 |
| Q14BI2 | Q14BI2 | GRM2_MO | Grm2 | Metabotropic glutamate receptor 2 | + | + | | 1.704681 | 2.657917 | 7.011902 | 1.875731 | -0.82632 | -8.58016 | 0.210306 | 0.210107 | 0.587862 | -1.249 | -0.88899 | 0.052539 | -0.07435 | 1.061315 | 0.34652 | -4.06051 | -3.39332 | 0.742962 | 0.887869 | 0.491034 | 0.496588 |
| P84309 | P84309 | ADCY5_MC | Adcy5 | Adenylate cyclase type 5 | | | | 0.006395 | -0.02992 | -0.02067 | 0.654101 | 0.236707 | 1.752591 | 0.413105 | 0.862092 | 1.099329 | -2.9402 | -2.31654 | 0.362855 | 0.095954 | -1.18108 | -0.6177 | -1.18544 | -4.01147 | -0.0281 | 0.013492 | -2.49334 | -1.02962 |
| P97490 | P97490 | ADCY8_MC | Adcy8 | Adenylate cyclase type 8 | | + | | 0.316514 | 0.248023 | 0.855316 | 1.992809 | -0.96412 | -9.84177 | 0.236709 | 0.739538 | 0.65484 | -0.30981 | -0.10434 | -2.12891 | -2.14501 | -1.21646 | -1.78402 | -0.18393 | -0.72626 | -1.27047 | -1.07521 | -3.33289 | -1.14667 |
| P51830 | P51830 | ADCY9_MC | Adcy9 | Adenylate cyclase type 9 | | + | + | 0.007675 | 0.031957 | 0.024778 | 1.081868 | -0.39845 | -3.25516 | 1.455436 | 0.496247 | 5.200744 | -1.944 | -1.41027 | -0.60477 | -0.68158 | 0.466056 | 0.414285 | -0.44727 | -2.97091 | -0.36095 | -0.1285 | 0.035764 | -0.14792 |
| Q9QYC0 | Q9QYC0 | ADDA_MO | Add1 | Alpha-adducin | | + | | 0.407637 | -0.21367 | -1.08537 | 1.264628 | -0.70472 | -4.11168 | 0.253664 | 0.228442 | 0.697597 | 2.799896 | 2.925618 | 2.662318 | 2.719855 | 3.44797 | 2.876633 | 3.262995 | 2.889868 | 3.226842 | 3.564767 | 3.093953 | 2.773767 |
| Q9QYB8 | Q9QYB8 | ADDB_MO | Add2 | Beta-adducin | | | | 0.02899 | -0.01453 | -0.09151 | 0.882137 | -0.78402 | -2.48159 | 0.36555 | 0.406103 | 0.978604 | 1.968463 | 2.082268 | 2.096772 | 1.797126 | 2.860888 | 2.057534 | 2.188071 | 1.891712 | 2.45282 | 3.009125 | 2.157347 | 1.948868 |
| Q9QYB5 | Q9QYB5 | ADDG_MC | Add3 | Gamma-adducin | | | | 0.561685 | 0.127876 | 1.491439 | 0.247233 | -0.29872 | -0.6814 | 0.360333 | 0.494807 | 0.965447 | -0.12191 | -0.06888 | 0.16699 | -0.02598 | 1.132158 | 0.179653 | -0.3048 | -0.14173 | -0.05842 | 0.796878 | 0.350455 | -0.02826 |
| Q8C4G9 | Q8C4G9 | AGRA1_MC | Adgra1 | Adhesion G protein-coupled receptor A1 | | | | NaN | 0 | NaN | 0.333801 | -0.60843 | -0.8987 | 0.104919 | 0.486611 | 0.310756 | -3.56244 | -2.90832 | -2.92801 | -3.2784 | -0.50119 | -3.37938 | -3.3152 | -2.87893 | -1.84083 | -3.14873 | -3.04418 | -1.80961 |
| Q3UHD1 | Q3UHD1 | AGRB1_MC | Adgrb1 | Adhesion G protein-coupled receptor B1 | | | | 0.059599 | 0.14185 | 0.182858 | 0.206101 | -0.13089 | -0.57714 | 0.54227 | 0.793827 | 1.438497 | -3.93741 | -2.51339 | -0.62643 | -0.96099 | 0.004975 | -1.09018 | -3.05932 | -3.67518 | -0.5097 | -0.81595 | -1.40494 | -1.26792 |
| Q8CGM1 | Q8CGM1 | AGRB2_MC | Adgrb2 | Adhesion G protein-coupled receptor B2 | | | | NaN | 0 | NaN | 0.647913 | 0.435036 | 1.734586 | 0.221779 | 0.439518 | 0.617036 | -3.44692 | -3.62984 | -0.98791 | -0.70046 | 0.235876 | -1.03724 | -3.37975 | -3.21787 | -1.07368 | -1.48475 | -1.15984 | -0.52056 |
| Q80ZF8 | Q8CGM1;( | AGRB3_MC | Adgrb3 | Adhesion G protein-coupled receptor B3 | | | | 0.335712 | 0.55338 | 0.903494 | 0.372148 | 0.189186 | 0.995268 | 0.264822 | 0.140016 | 0.72566 | -2.04903 | -0.91222 | 0.79704 | 1.169523 | 1.007811 | 0.624071 | -1.80584 | -2.26217 | 0.832127 | 0.756064 | 0.696309 | 0.65554 |
| Q8K209 | Q8K209 | AGRG1_M | Adgrg1 | Adhesion G-protein coupled receptor G1 | | + | | 0.081391 | -0.07565 | -0.24529 | 1.114926 | 2.212385 | 3.39844 | 0.521606 | 0.329978 | 1.382793 | -1.54965 | -1.40871 | -2.26947 | -1.54701 | -1.54065 | -1.81971 | -1.70379 | -1.10326 | -4.66221 | -3.57904 | -2.20374 | -1.81657 |
| Q80TR1 | Q80TR1 | AGRL1_MC | Adgrl1 | Adhesion G protein-coupled receptor L1 | | | | 0.064014 | -0.04016 | -0.19566 | 0.769296 | 0.318378 | 2.103658 | 0.371187 | 0.18319 | 0.992842 | -0.54041 | -0.64408 | 0.681078 | 0.666674 | 0.491977 | 0.282496 | -0.75066 | -0.35351 | 0.204324 | 0.506671 | 0.355947 | 0.052147 |
| Q8JZZ7 | Q8JZZ7 | AGRL2_MC | Adgrl2 | Adhesion G protein-coupled receptor L2 | | | | NaN | 0 | NaN | 0.654732 | -0.74829 | -1.75443 | 0.039368 | -0.13098 | -0.12302 | -2.77842 | -3.0793 | -1.93865 | -2.75119 | -2.08449 | -4.19617 | -3.71145 | -3.58192 | -1.72648 | -1.46677 | -3.14676 | -2.87192 |
| Q80TS3 | Q80TS3 | AGRL3_MC | Adgrl3 | Adhesion G protein-coupled receptor L3 | | | + | 0.882455 | 0.910366 | 2.482709 | 0.317242 | 0.125442 | 0.857143 | 1.603573 | 0.521993 | 6.216581 | -1.6208 | -1.1011 | 0.233314 | 0.143628 | 0.256578 | 0.09576 | -2.53003 | -2.0126 | -0.07628 | 0.202339 | -0.37001 | -0.32164 |
| P28474 | P28474 | ADHX_MO | Adh5 | Alcohol dehydrogenase class-3 | + | | | 1.095513 | -1.49547 | -3.31372 | 0.29593 | -1.40664 | -0.80371 | 0.09102 | 0.282815 | 0.272307 | -1.75239 | -0.85285 | -0.19066 | -3.66725 | -1.1103 | 0.479864 | 0.155783 | 0.229912 | -0.31875 | -0.72589 | 0.070185 | -1.26625 |
| Q9Z0J4 | Q9Z0J4 | NOS1_MO | Nos1 | Nitric oxide synthase 1 | + | | | 1.900923 | 2.616644 | 8.837702 | 0.201488 | 0.150774 | 0.565351 | 0.22079 | -0.07054 | -0.61453 | -0.65753 | -0.15332 | -0.29717 | 0.231466 | 0.2012 | 0.148911 | -2.86681 | -3.17733 | -0.2191 | -0.14815 | 0.133824 | 0.357367 |
| P55264 | P55264 | ADK_MOU | Adk | Adenosine kinase | | | | 0.2009 | 0.3555 | 0.563847 | 0.292524 | 0.281051 | 0.795172 | 0.020219 | -0.04111 | -0.0644 | -0.98255 | 0.015821 | -0.88711 | -1.59276 | -1.63392 | -0.67287 | -0.45372 | -1.22401 | -1.54195 | -1.50002 | -0.69207 | -1.5325 |
| Q9Z103 | Q9Z103 | ADNP_MO | Adnp | Activity-dependent neuroprotector homeobox protein | | | | 0.416426 | 1.37799 | 1.10782 | 0.108226 | 0.105292 | 0.319817 | 0.018139 | 0.031851 | 0.057899 | -3.13526 | -0.68308 | -1.2339 | -0.58118 | -2.06184 | -1.11969 | -3.07758 | -3.49674 | -1.05616 | -0.9695 | -1.33853 | -1.9067 |
| Q6PDY2 | Q6PDY2 | AEDO_MO | Ado | 2-aminoethanethiol dioxygenase | | | | 0.837533 | -0.42024 | -2.32773 | 0.409972 | -1.80549 | -1.09133 | 0.253148 | 0.196472 | 0.696299 | 1.185192 | 0.830421 | -0.40113 | -3.68645 | 0.186131 | 0.091063 | 1.394457 | 1.461644 | -0.04154 | -0.43505 | -0.33601 | 0.220259 |
| Q9JKV1 | A0A0A6YV | ADRM1_M | Adrm1 | Proteasomal ubiquitin receptor ADRM1 | | | | 0.302846 | -0.4348 | -0.82105 | 0.744963 | 0.831957 | 2.026817 | 0.786346 | 0.240226 | 2.15843 | -4.08481 | -3.02637 | -2.53124 | -2.86365 | -2.90895 | -2.70577 | -3.1014 | -3.14018 | -3.15408 | -3.90472 | -3.00214 | -3.09303 |
| P46664 | P46664 | PURA2_MC | Adss2 | Adenylosuccinate synthetase isozyme 2 | | | | 0.299736 | -0.66979 | -0.81325 | 0.118329 | -0.24017 | -0.34731 | 0.473325 | -0.59814 | -1.25497 | -3.46453 | -2.79389 | -1.69601 | -2.78445 | -2.71799 | -1.78871 | -1.70719 | -3.21166 | -2.42669 | -1.57344 | -1.54903 | -1.76139 |
| Q9QZQ1 | Q9QZQ1 | AFAD_MOI | Afdn | Afadin | | | | 0.431585 | 0.398706 | 1.146708 | 0.130342 | 0.272368 | 0.379652 | 0.07971 | 0.097831 | 0.240543 | -2.07508 | -1.90568 | -1.59106 | -1.5318 | -0.94954 | -1.75696 | -2.05186 | -2.72631 | -2.5506 | -1.117 | -1.40176 | -1.50041 |
| Q9ESC8 | Q9ESC8 | AFF4_MOI | Aff4 | AF4/FMR2 family member 4 | | | | 0.633261 | 1.488377 | 1.692267 | 0.18014 | 0.21849 | 0.510476 | 0.495667 | -0.74467 | -1.31374 | -2.81608 | -1.24196 | -1.10326 | -0.71871 | -2.40737 | -1.79432 | -3.12485 | -3.90994 | -1.51187 | -0.74708 | -1.83298 | -0.87938 |
| Q3UMC0 | Q3UMC0;( | AFG2A_MC | Afg2a | ATPase family gene 2 protein homolog A | | | | 0.100504 | -0.17854 | -0.29861 | 0.035539 | 0.07507 | 0.111464 | 0.51895 | 0.689295 | 1.37568 | 0.608084 | 0.637746 | 1.977762 | 0.775868 | 1.952244 | 1.016627 | 1.399157 | 0.203744 | 0.997692 | 1.605799 | 0.974623 | 0.615658 |
| Q8JZQ2 | Q8JZQ2 | AFG32_MC | Afg3l2 | AFG3-like protein 2 | | | | 0.123628 | 0.177622 | 0.361619 | 0.104453 | 0.084882 | 0.309478 | 0.522755 | 0.784793 | 1.385874 | -0.34618 | -0.11265 | 0.254039 | -0.25366 | 1.017787 | -0.06367 | 0.070067 | -0.88414 | 0.019172 | -0.18856 | -0.13955 | -0.47592 |
| Q8BXK8 | Q8BXK8;Q | AGAP1_MC | Agap1 | Arf-GAP with GTPase, ANK repeat and PH domain-containing protein 1 | | | | 0.290694 | 0.237909 | 0.790585 | 0.134448 | -0.14891 | -0.39063 | 0.55007 | -0.66566 | -1.45969 | 1.726453 | 1.927648 | 2.454818 | 3.216998 | 1.790387 | 2.701774 | 1.872757 | 1.305527 | 2.975344 | 2.994291 | 2.929188 | 2.894294 |
| Q3UHD9 | Q3UHD9 | AGAP2_MC | Agap2 | Arf-GAP with GTPase, ANK repeat and PH domain-containing protein 2 | | + | | 0.595645 | 0.534177 | 1.585559 | 1.139792 | 0.428967 | 3.50942 | 0.453059 | -0.62619 | -1.20217 | 0.738825 | 0.065162 | 5.163805 | 5.361725 | 3.602967 | 4.600571 | -0.13905 | -0.12532 | 4.762049 | 4.905546 | 4.57792 | 4.877989 |
| Q8VHH5 | Q8VHH5 | AGAP3_MC | Agap3 | Arf-GAP with GTPase, ANK repeat and PH domain-containing protein 3 | | | | 0.030796 | -0.01322 | -0.09704 | 0.010151 | 0.006884 | 0.032681 | 0.625744 | -0.36154 | -1.67073 | 0.664481 | 0.916049 | 2.893582 | 3.119334 | 2.609237 | 2.820812 | 0.85586 | 0.751113 | 2.821716 | 3.177432 | 2.887788 | 3.265343 |
| Q09LZ8 | Q09LZ8 | CBPC6_MC | Agbl4 | Cytosolic carboxypeptidase 6 | | | | NaN | 0 | NaN | 0.326659 | 0.353271 | 0.880765 | 0.761379 | -0.88829 | -2.07849 | -3.31392 | -2.8196 | -1.10533 | -0.83554 | -2.61915 | -2.0775 | -2.78883 | -3.201 | -1.70144 | -0.94597 | -1.79064 | -1.12942 |
| Q9ESW4 | Q9ESW4 | AGK_MOU | Agk | Acylglycerol kinase, mitochondrial | | | | 0.116991 | -0.07919 | -0.34368 | 0.444576 | -0.22094 | -1.18021 | 0.505618 | 0.379953 | 1.340122 | 0.584546 | 0.958763 | 0.526042 | 0.154523 | 1.4077 | 1.097283 | 0.985321 | 0.71637 | 0.584433 | 0.538012 | 1.109803 | 0.635273 |
| F8VPN4 | F8VPN4 | F8VPN4_M | Agl | Glycogen debranching enzyme | | | + | 0.126691 | -0.09201 | -0.36986 | 0.862639 | -0.72723 | -2.41358 | 1.101239 | -1.25265 | -3.33854 | 1.560153 | 1.325046 | -0.18603 | -0.05721 | -0.41347 | -0.71536 | 1.75386 | 1.315362 | 0.899956 | 0.311269 | 0.344723 | 1.031736 |
| Q8CJG0 | Q8CJG0;Q | AGO2_MO | Ago2 | Protein argonaute-2 | | | | 0.080452 | -0.25184 | -0.24264 | 0.096571 | 0.131725 | 0.287736 | 0.737552 | 0.681351 | 2.003716 | -2.34525 | -3.24397 | -2.31302 | -1.62478 | -1.88838 | -2.30683 | -1.60717 | -3.47837 | -2.40255 | -1.7987 | -3.04701 | -2.5109 |
| O35083 | O35083 | PLCA_MOI | Agpat1 | 1-acyl-sn-glycerol-3-phosphate acyltransferase alpha | | | + | 0.669291 | 0.386957 | 1.79713 | 0.009044 | 0.009693 | 0.029153 | 1.220153 | 0.383527 | 3.888089 | 0.682818 | 0.316063 | -0.02905 | -0.28684 | 0.105302 | 0.171176 | 0.225334 | -0.00037 | 0.138845 | -0.47412 | -0.33827 | -0.15231 |
| Q9D517 | Q9D517 | PLCC_MO | Agpat3 | 1-acyl-sn-glycerol-3-phosphate acyltransferase gamma | | | | 0.154416 | 0.051675 | 0.443474 | 0.307868 | -0.16467 | -0.83364 | 0.988338 | -0.14495 | -2.87439 | 1.781716 | 1.666885 | 0.466218 | 0.440731 | 0.777287 | 0.764279 | 1.571231 | 1.774021 | 0.815275 | 0.421023 | 0.965738 | 0.865726 |
| Q8K4X7 | Q8K4X7 | PLCD_MO | Agpat4 | 1-acyl-sn-glycerol-3-phosphate acyltransferase delta | | | | 0.420051 | -0.21571 | -1.1171 | 0.436141 | 0.269808 | 1.158438 | 0.254108 | 0.259183 | 0.698715 | 0.591298 | 0.214486 | -0.20487 | -0.61153 | -0.43233 | -0.06917 | 0.660899 | 0.5763 | -0.7916 | -0.56441 | -0.18647 | -0.8334 |
| Q9D1E8 | Q9D1E8 | PLCE_MOI | Agpat5 | 1-acyl-sn-glycerol-3-phosphate acyltransferase epsilon | | | + | 0.315254 | 0.2595 | 0.852157 | 0.215659 | -0.32029 | -0.60149 | 1.230578 | 0.439144 | 3.93957 | 0.394891 | 0.290132 | -0.59388 | -1.65773 | 0.045926 | -0.15181 | -0.21697 | 0.382994 | -0.78079 | -0.83024 | -0.54357 | -0.44059 |
| A2ASQ1 | A2ASQ1 | AGRIN_MC | Agrn | Agrin | | + | | NaN | 0 | NaN | 1.352415 | -0.11842 | -4.58513 | 0.006239 | -0.00708 | -0.02017 | -2.91709 | -3.61289 | -1.52662 | -1.57808 | -1.54858 | -2.21664 | -3.38132 | -3.79931 | -1.43616 | -1.43168 | -1.7674 | -1.98365 |
| Q641K1 | Q641K1 | CBPC1_MC | Agtpbp1 | Cytosolic carboxypeptidase 1 | | | | 0.312044 | 0.549802 | 0.844107 | 0.346275 | 0.216699 | 0.930045 | 0.500979 | -0.60931 | -1.32781 | -3.44895 | -2.16669 | -1.8455 | -1.3807 | -1.74166 | -2.6547 | -3.47249 | -3.24276 | -1.81312 | -1.84648 | -1.54234 | -1.6354 |
| P50247 | P50247 | SAHH_MO | Ahcy | Adenosylhomocysteinase | | | | 0.031941 | 0.021078 | 0.100531 | 0.318893 | 0.503332 | 0.861283 | 0.176199 | 0.395703 | 0.50028 | 0.305374 | 0.154465 | -0.0702 | -0.76464 | -0.74971 | 0.667662 | 0.013224 | 0.404459 | -1.39081 | -0.45069 | -0.08547 | -0.78799 |
| Q80SW1 | Q80SW1 | SAHH2_MC | Ahcyl1 | S-adenosylhomocysteine hydrolase-like protein 1 | | | | 0.252395 | 0.286507 | 0.694402 | 0.328236 | -0.17506 | -0.88472 | 0.246569 | 0.068761 | 0.679726 | 1.787075 | 2.032773 | 1.520765 | 1.141544 | 1.523412 | 1.547851 | 2.017299 | 1.229536 | 1.449653 | 1.562768 | 1.567289 | 1.366452 |
| Q6PAL7 | Q6PAL7 | AHDC1_M | Ahdc1 | Transcription factor Gibbin | | | | NaN | 0 | NaN | 0.109222 | 0.08672 | 0.322541 | 0.150132 | -0.08961 | -0.4322 | -3.62603 | -3.69009 | -1.66992 | -1.18086 | -1.80706 | -1.64517 | -2.9167 | -3.85025 | -1.62389 | -1.40033 | -1.82739 | -1.44561 |
| Q8K3E5 | Q8K3E5 | AHI1_MOL | Ahi1 | Jouberin | + | | | 1.028134 | 0.797565 | 3.032136 | 0.009838 | 0.01997 | 0.031684 | 0.265818 | -0.35167 | -0.72816 | 0.843046 | 0.323532 | -0.23126 | 1.01033 | -0.27758 | -0.33623 | -0.17286 | -0.25569 | 0.260684 | 0.478449 | -0.4373 | 0.526836 |
| E9Q616 | E9Q616 | E9Q616_M | Ahnak | AHNAK nucleoprotein (desmoyokin) | | + | | 0.902872 | 0.350155 | 2.555255 | 1.376439 | -0.55036 | -4.72263 | 0.497477 | -0.41328 | -1.31853 | -0.56397 | -0.39935 | -0.65512 | -0.728 | -1.72619 | -1.16218 | -0.72226 | -0.94138 | -0.25189 | -0.03051 | -1.16772 | -0.89409 |
| A0A7N9VF | A0A7N9VF | A0A7N9VF | Ahnak2 | AHNAK nucleoprotein 2 | | | | 0.294376 | -0.21974 | -0.79982 | 0.898279 | 2.306409 | 2.538816 | 0.273188 | 0.542566 | 0.746671 | 1.462332 | 1.442707 | -1.07861 | -0.95138 | -1.70949 | -3.6006 | 1.946831 | 1.397696 | -2.41518 | -4.22763 | -2.65994 | -3.19528 |
| Q8BK64 | Q8BK64 | AHSA1_MC | Ahsa1 | Activator of 90 kDa heat shock protein ATPase homolog 1 | | | | 0.528675 | 0.151483 | 1.401779 | 0.339922 | 0.235205 | 0.914072 | 0.493295 | 0.501855 | 1.30747 | 0.996338 | 0.963411 | 1.434043 | 1.391886 | 2.118152 | 1.404304 | 0.935195 | 0.721589 | 0.921308 | 1.43421 | 1.118177 | 1.400568 |
| Q9Z0X1 | Q9Z0X1 | AIFM1_MC | Aifm1 | Apoptosis-inducing factor 1, mitochondrial | | + | | 0.373622 | 0.382469 | 0.998995 | 1.376403 | 0.744973 | 4.722413 | 0.746172 | 0.497586 | 2.030599 | -0.06153 | 0.001073 | 0.790505 | 0.921145 | 0.667693 | 0.208687 | -0.03113 | -0.79427 | -0.03274 | 0.254446 | -0.14527 | 0.026482 |
| Q3TY86 | Q3TY86 | AIFM3_MC | Aifm3 | Apoptosis-inducing factor 3 | | | | 0.62875 | 0.658851 | 1.679327 | 0.742624 | -0.41076 | -2.01951 | 0.516999 | -0.19134 | -1.37046 | -0.19612 | -0.33046 | -0.36458 | -0.16056 | -0.51897 | -0.24051 | -0.5356 | -1.30868 | 0.32416 | -0.02777 | -0.19878 | -0.17804 |
| Q8BY87 | Q8BY87 | UBP47_MC | Usp47 | Ubiquitin carboxyl-terminal hydrolase 47 | + | | | 1.639616 | 2.598019 | 6.489898 | 0.225704 | 0.382825 | 0.626991 | 0.097028 | -0.23644 | -0.289 | -1.60404 | -0.81155 | -1.36571 | -1.36289 | -1.36713 | -2.27854 | -3.74885 | -3.86277 | -2.3577 | -1.13655 | -2.26585 | -0.90694 |
| P31230 | P31230 | AIMP1_MC | Aimp1 | Aminoacyl tRNA synthase complex-interacting multifunctional protein 1 | | | | 0.266801 | 0.46934 | 0.730632 | 0.546468 | 0.272833 | 1.449894 | 0.385892 | -0.16815 | -1.03006 | -0.57588 | -0.71589 | 0.264286 | 0.399448 | 0.065934 | -0.13973 | -0.47668 | -1.75378 | -0.11659 | 0.234654 | 0.00447 | 0.258037 |
| Q8R010 | Q8R010 | AIMP2_MC | Aimp2 | Aminoacyl tRNA synthase complex-interacting multifunctional protein 2 | | | | 0.840096 | 1.05233 | 2.336402 | 0.053666 | 0.126604 | 0.165512 | 0.299914 | 0.502142 | 0.813698 | -1.74191 | -2.45911 | -1.73628 | -2.36269 | -1.18053 | -1.76871 | -3.42537 | -2.88031 | -2.87395 | -1.47823 | -2.51929 | -1.43423 |
| O08915 | O08915 | AIP_MOUS | Aip | AH receptor-interacting protein | | | + | 0.075041 | 0.025872 | 0.227295 | 0.351623 | 0.262731 | 0.943502 | 1.349113 | -0.77417 | -4.56651 | -0.74721 | -0.82937 | -0.15928 | -0.35703 | -1.05836 | -1.25337 | -0.92032 | -0.70801 | -0.78121 | -0.26057 | -0.243 | -0.52038 |
| A2ALI5 | A2ALI5 | AJAP1_MC | Ajap1 | Adherens junction-associated protein 1 | | | | 0.012363 | -0.01964 | -0.03971 | 0.358746 | 0.435221 | 0.961444 | 0.444657 | 0.352898 | 1.180416 | -3.43189 | -3.90819 | -1.84859 | -1.90318 | -2.79727 | -3.33149 | -3.21679 | -4.084 | -2.76295 | -1.85925 | -3.55155 | -3.28301 |
| A2AJA9 | A2AJA9 | AJM1_MOI | Ajm1 | Apical junction component 1 homolog | | | | 0.430368 | 0.644946 | 1.143578 | 0.49338 | 0.132808 | 1.307694 | 0.002937 | 0.004288 | 0.009532 | -0.25039 | -0.97858 | 0.285967 | 0.478648 | 0.421936 | -0.01749 | -1.69013 | -0.82873 | 0.281637 | 0.217362 | -0.19467 | 0.590531 |
| Q9R0Y5 | Q9R0Y5 | KAD1_MOI | Ak1 | Adenylate kinase isoenzyme 1 | | | | 0.486429 | -0.4943 | -1.28936 | 0.302256 | 0.778858 | 0.819571 | 0.331798 | 0.499872 | 0.893665 | -0.13775 | 0.495511 | 1.973427 | 0.131514 | 0.292826 | 0.623906 | 0.889328 | 0.45704 | 0.039187 | 0.508037 | 0.492787 | -0.5758 |
| Q5F2E7 | Q5F2E7 | NUFP2_MC | Nufip2 | FMR1-interacting protein NUFIP2 | + | | | 2.223622 | 2.570323 | 12.87828 | 0.593827 | 0.299332 | 1.58047 | 0.381201 | 0.051362 | 1.018173 | -0.97247 | -0.57418 | 0.856951 | 0.483205 | 0.27463 | 0.293465 | -3.33038 | -3.35691 | 0.401547 | 0.339944 | 0.183126 | 0.282244 |
| Q9WTP7 | Q9WTP7 | KAD3_MOI | Ak3 | GTP:AMP phosphotransferase AK3, mitochondrial | | | | 0.421979 | -0.5595 | -1.12204 | 0.015963 | -0.11287 | -0.05107 | 0.050275 | -0.03892 | -0.15553 | -1.45226 | -0.45991 | -0.57051 | -3.92285 | -0.57495 | -0.07546 | -0.347 | -0.44616 | -0.69319 | -3.57443 | -0.30196 | -0.27061 |
| Q9WUR9 | Q9WUR9 | KAD4_MOI | Ak4 | Adenylate kinase 4, mitochondrial | | | | 0.998525 | -0.96719 | -2.91419 | 0.037784 | -0.06379 | -0.11825 | 0.739574 | 0.719264 | 2.010004 | -1.60781 | -1.43146 | -0.89286 | -1.81956 | -0.36215 | -0.30555 | -0.87241 | -0.23248 | -1.0162 | -1.56864 | -0.69639 | -1.40983 |
| Q920P5 | Q920P5 | KAD5_MOI | Ak5 | Adenylate kinase isoenzyme 5 | | | | 0.363443 | -0.99915 | -0.97329 | 0.273519 | 0.253293 | 0.747502 | 0.254809 | 0.298398 | 0.700479 | -3.75884 | -2.69593 | 0.54482 | -0.00219 | 1.057756 | 0.205895 | -1.34994 | -3.10653 | -0.18202 | 0.21806 | 0.34058 | 0.326276 |
| O88845 | O88845 | AKA10_MC | Akap10 | A-kinase anchor protein 10, mitochondrial | | | | 0.026137 | 0.119937 | 0.082743 | 0.601796 | -0.23887 | -1.60282 | 0.12513 | 0.03091 | 0.365664 | -3.77708 | -1.40828 | -1.09106 | -1.36877 | -1.53765 | -1.47773 | -1.87697 | -3.54826 | -1.04517 | -0.93693 | -1.61764 | -1.45956 |
| Q9R078 | Q9R078 | AAKB1_MC | Prkab1 | 5'-AMP-activated protein kinase subunit beta-1 | + | | | 1.798676 | 2.503125 | 7.836304 | 0.640764 | 0.814893 | 1.713885 | 0.310997 | 0.16256 | 0.841483 | 5.584181 | 5.726467 | 5.470207 | 5.783466 | 4.924092 | 4.900216 | 2.840796 | 3.463603 | 4.363018 | 5.26087 | 4.55678 | 4.942408 |
| Q9WTQ5 | Q9WTQ5 | AKA12_MC | Akap12 | A-kinase anchor protein 12 | + | | + | 0.464588 | -0.2274 | -1.23215 | 0.940238 | 0.701069 | 2.691624 | 2.279835 | -0.30965 | -13.7468 | -0.78432 | -0.76346 | -1.5544 | -1.54537 | -2.42935 | -2.4227 | -0.73075 | -0.36224 | -1.99053 | -2.51138 | -2.09409 | -2.13865 |
| P12023 | P12023 | A4_MOUSI | App | Amyloid-beta precursor protein | + | | | 1.526851 | 2.485772 | 5.669849 | 0.411722 | 0.208977 | 1.095795 | 0.309437 | 0.349291 | 0.837571 | -1.2305 | -1.1828 | -0.85647 | -1.22055 | -0.39337 | -1.22736 | -4.13019 | -3.25465 | -1.19065 | -1.30432 | -1.15431 | -1.165 |
| E9Q9K8 | E9Q9K8 | E9Q9K8_M | Akap6 | A kinase (PRKA) anchor protein 6 | | | | NaN | 0 | NaN | 0.109257 | -0.27546 | -0.32264 | 0.117479 | 0.257867 | 0.345007 | -2.94834 | -4.27263 | -1.71082 | -3.06161 | -3.03979 | -3.9507 | -2.94999 | -2.83033 | -2.63303 | -1.58849 | -3.16048 | -4.34574 |
| Q91YI4 | Q91YI4 | ARRB2_MC | Arrb2 | Beta-arrestin-2 | + | | | 2.583659 | 2.480082 | 19.54244 | 0.660695 | -0.33073 | -1.77186 | 0.536517 | -0.41125 | -1.42293 | -0.97176 | -0.98293 | -1.19693 | -0.85859 | -1.46498 | -0.96367 | -3.58421 | -3.33064 | -0.77592 | -0.61813 | -0.94696 | -0.65919 |
| Q9DBR0 | Q9DBR0 | AKAP8_MC | Akap8 | A-kinase anchor protein 8 | | | | 0.294221 | 0.428478 | 0.799429 | 0.229779 | 0.151149 | 0.637313 | 0.213244 | -0.10738 | -0.59534 | -0.87845 | -0.5557 | -1.12402 | -1.00813 | -1.65235 | -1.42653 | -0.63444 | -1.65666 | -0.98724 | -1.4472 | -1.57272 | -1.2914 |
| Q9R0L7 | Q9R0L7 | AKP8L_MC | Akap8l | A-kinase anchor protein 8-like | | | | 0.099361 | 0.282093 | 0.295453 | 0.580648 | 0.358029 | 1.543748 | 0.043124 | -0.04678 | -0.13428 | -3.82915 | -1.92031 | 0.091221 | 0.088531 | -0.64165 | -0.85413 | -3.13064 | -3.18301 | -0.50007 | -0.03623 | -1.03287 | -0.36936 |
| Q9JII6 | Q9JII6 | AK1A1_MC | Akr1a1 | Aldo-keto reductase family 1 member A1 | + | | | 1.004986 | -1.36414 | -2.93964 | 0.071217 | -0.33629 | -0.21638 | 0.094924 | -0.15144 | -0.28317 | -2.55413 | -1.63292 | -0.15501 | -3.2291 | -1.01517 | -0.52139 | -0.78584 | -0.67294 | -1.58588 | -1.12566 | -0.14246 | -1.09123 |
| P45376 | P45376 | ALDR_MOI | Akr1b1 | Aldo-keto reductase family 1 member B1 | | | | 0.501054 | -0.37544 | -1.32801 | 0.208961 | -0.60749 | -0.58443 | 0.089914 | 0.088152 | 0.269219 | -0.68554 | -0.27834 | 0.258118 | -1.77844 | -0.35499 | -0.38762 | -0.30264 | 0.089637 | 0.056076 | -0.36141 | -0.13242 | -0.78648 |
| Q80TL1 | Q80TL1 | ADCY2_MC | Adcy2 | Adenylate cyclase type 2 | + | | + | 1.574346 | 2.476286 | 6.002829 | 0.119259 | 0.128916 | 0.349825 | 1.020407 | 0.374438 | 3.001023 | -1.31443 | -0.6907 | -1.01702 | -1.59685 | -1.17342 | -1.04111 | -3.20883 | -3.74887 | -1.20836 | -1.66335 | -1.58749 | -1.37591 |
| Q6NS52 | Q6NS52 | DGKB_MO | Dgkb | Diacylglycerol kinase beta | + | + | | 1.360762 | 2.425231 | 4.632501 | 1.209043 | 0.395901 | 3.833842 | 0.072511 | -0.03465 | -0.22008 | -0.71088 | -1.35503 | 1.204713 | 1.374382 | 1.024519 | 0.935681 | -3.04545 | -3.87092 | 0.834767 | 0.952526 | 0.863719 | 1.165772 |
| Q8CG76 | Q8CG76 | ARK72_MC | Akr7a2 | Aflatoxin B1 aldehyde reductase member 2 | | | | 0.200631 | 0.118592 | 0.563158 | 0.674885 | -0.6884 | -1.81366 | 0.555601 | -0.27992 | -1.47478 | 0.048196 | -0.21324 | -1.26509 | -1.75336 | -0.37817 | -0.00365 | -0.03601 | -0.36621 | -0.53019 | -1.11145 | 0.05803 | 0.119995 |
| P10518 | P10518 | HEM2_MO | Alad | Delta-aminolevulinic acid dehydratase | | | | 0.514046 | 0.488902 | 1.362572 | 0.053697 | 0.148515 | 0.165602 | 0.445952 | -0.82044 | -1.18377 | -1.33666 | -0.87813 | -2.01691 | -3.24632 | -2.90693 | -1.63963 | -1.32029 | -1.87231 | -3.43314 | -2.12712 | -1.17206 | -1.73362 |
| P07724 | P07724 | ALBU_MOI | Alb | Albumin | | | | 0.244684 | 0.15095 | 0.674971 | 0.026754 | 0.043514 | 0.084643 | 0.122704 | 0.100952 | 0.35913 | 2.484171 | 2.739987 | 2.272724 | 1.257183 | 1.700225 | 2.202058 | 2.277679 | 2.644579 | 1.801795 | 1.641084 | 1.976912 | 1.723467 |
| Q61490 | Q61490 | CD166_MC | Alcam | CD166 antigen | | + | | 0.672731 | -0.63261 | -1.80729 | 1.900578 | -0.67228 | -8.83412 | 0.373918 | -0.23478 | -0.99974 | -1.46179 | -0.9208 | -0.20704 | -0.24694 | -0.54491 | -0.31471 | -0.78085 | -0.33652 | 0.371852 | 0.51873 | -0.39973 | 0.009673 |
| Q9Z110 | Q9Z110 | P5CS_MOI | Aldh18a1 | Delta-1-pyrroline-5-carboxylate synthase | | | | 0.637306 | -0.5995 | -1.70391 | 0.297726 | -0.32424 | -0.80821 | 0.311639 | 0.396523 | 0.843093 | -1.78653 | -2.46718 | -1.5173 | -2.30745 | -0.90246 | -1.69484 | -1.61663 | -1.43807 | -1.65785 | -1.51841 | -1.44172 | -1.94862 |
| Q60829 | Q60829 | PPR1B_MC | Ppp1r1b | Protein phosphatase 1 regulatory subunit 1B | + | + | | 1.468064 | 2.424144 | 5.281051 | 1.16007 | 1.696543 | 3.602035 | 0.882906 | 0.31171 | 2.484297 | -1.2596 | -0.65171 | 1.749541 | 2.099825 | -0.77343 | -0.7948 | -3.72378 | -3.03582 | -0.20908 | 0.665361 | -1.22084 | -0.97081 |
| Q7TMC8 | Q7TMC8 | FCSK_MOI | Fcsk | L-fucose kinase | + | | | 1.953349 | 2.383281 | 9.397762 | 0.397203 | -0.12756 | -1.05878 | 0.940417 | 0.058049 | 2.69229 | -1.45887 | -1.28619 | -0.90785 | -0.96698 | -0.63467 | -0.60316 | -3.99426 | -3.51736 | -0.92665 | -0.69306 | -0.66224 | -0.69169 |
| Q9JHW9 | Q9JHW9 | AL1A3_MC | Aldh1a3 | Retinaldehyde dehydrogenase 3 | | | | 0.31698 | 0.4017 | 0.856484 | 0.153439 | 0.337547 | 0.440906 | 0.284834 | 0.641316 | 0.77589 | -2.54009 | -2.00454 | -1.43354 | -1.4133 | -1.45644 | -2.2614 | -3.05907 | -2.28896 | -2.52647 | -0.99546 | -3.22218 | -1.7783 |
| Q99P31 | Q99P31 | HPBP1_MC | Hspbp1 | Hsp70-binding protein 1 | + | | | 2.128132 | 2.350803 | 11.52472 | 0.299021 | 0.089107 | 0.811462 | 0.261754 | -0.31431 | -0.71795 | -0.84062 | -0.94951 | -0.67419 | -0.55867 | -1.83325 | -1.04668 | -3.44245 | -3.04929 | -0.61214 | -0.79893 | -1.31797 | -0.93334 |
| Q8CGV2 | Q8CGV2 | TPH2_MOI | Tph2 | Tryptophan 5-hydroxylase 2 | + | + | | 1.734646 | 2.33357 | 7.265355 | 1.558888 | 1.198233 | 5.892533 | 0.026085 | -0.06487 | -0.08258 | 1.801858 | 1.463595 | 0.24952 | 0.536119 | -0.16625 | -1.64797 | -0.42779 | -0.9739 | -0.66114 | -0.94969 | -1.10324 | -0.58125 |
| Q8R0Y6 | Q8K009;Q | AL1L1_MC | Aldh1l1 | Cytosolic 10-formyltetrahydrofolate dehydrogenase | | | | 0.567838 | -0.37132 | -1.50834 | 0.235889 | -0.21957 | -0.65277 | 0.462192 | 0.398216 | 1.225909 | 1.36339 | 1.515401 | 1.94065 | 1.414746 | 2.332907 | 1.797722 | 2.04487 | 1.576566 | 1.687504 | 2.107039 | 1.851248 | 1.48295 |
| P47738 | Q62148;P | ALDH2_MC | Aldh2 | Aldehyde dehydrogenase, mitochondrial | + | + | | 1.079118 | 0.233631 | 3.243455 | 1.73411 | -0.53836 | -7.26075 | 0.619575 | -0.21201 | -1.65313 | 2.857621 | 2.99319 | 2.236689 | 2.367343 | 2.001796 | 2.202994 | 2.716142 | 2.667407 | 2.875453 | 2.805305 | 2.23486 | 2.393947 |
| P47740 | P47739;P | AL3A2_MC | Aldh3a2 | Aldehyde dehydrogenase family 3 member A2 | + | | | 1.06973 | -1.47493 | -3.20374 | 0.187483 | 0.624797 | 0.529416 | 0.126874 | -0.20274 | -0.37035 | -2.72403 | -3.46507 | -2.07432 | -2.88548 | -2.99642 | -2.08598 | -1.89287 | -1.34637 | -4.21298 | -1.99641 | -2.0344 | -2.64251 |
| P62075 | P62075 | TIM13_MO | Timm13 | Mitochondrial import inner membrane translocase subunit Tim13 | + | + | | 2.123179 | 2.266885 | 11.45845 | 1.020307 | 1.074744 | 3.000624 | 0.540777 | 0.248633 | 1.434452 | 3.957635 | 3.659698 | 0.829131 | 0.43836 | 0.359061 | 0.013715 | 1.671963 | 1.411601 | -0.74119 | -0.14081 | -0.04717 | -0.07731 |
| Q8K183 | Q8K183 | PDXK_MOI | Pdxk | Pyridoxal kinase | + | | | 2.441901 | 2.261363 | 16.5871 | 0.608728 | 0.726671 | 1.622367 | 0.581047 | 0.502289 | 1.544854 | 8.959377 | 9.19887 | 8.260952 | 8.795654 | 8.406401 | 7.884071 | 6.882933 | 6.752588 | 7.442265 | 8.160999 | 7.449281 | 7.836614 |
| Q9EQ20 | Q9EQ20 | MMSA_MC | Aldh6a1 | Methylmalonate-semialdehyde dehydrogenase [acylating], mitochondrial | | | | 0.758699 | -2.02346 | -2.07001 | 0.283445 | -0.88506 | -0.77241 | 0.088211 | 0.184391 | 0.264459 | -3.20551 | -1.39584 | -0.79268 | -3.08292 | -1.70591 | -0.41598 | 0.092662 | -0.64708 | -1.01211 | -1.09338 | -0.98047 | -1.5102 |
| Q9DBF1 | Q9DBF1 | AL7A1_MC | Aldh7a1 | Alpha-aminoadipic semialdehyde dehydrogenase | + | | | 1.29451 | -0.4777 | -4.2679 | 0.001266 | -0.00161 | -0.00412 | 0.253063 | -0.2329 | -0.69609 | -0.27861 | -0.49684 | -0.07771 | -0.52945 | -0.82067 | -0.31321 | 0.065056 | 0.114901 | -0.62109 | 0.017149 | -0.11595 | -0.55213 |
| Q9JLJ2 | Q9JLJ2 | AL9A1_MC | Aldh9a1 | 4-trimethylaminobutyraldehyde dehydrogenase | + | | | 1.30099 | -1.22978 | -4.30244 | 0.553838 | -1.82203 | -1.46997 | 0.289782 | -1.14144 | -0.7883 | -1.29305 | -1.25453 | -1.52358 | -4.0007 | -3.47327 | -0.61696 | 0.241167 | -0.3292 | -0.89178 | -0.98843 | -0.66492 | -1.14243 |
| P05064 | P05064 | ALDOA_MC | Aldoa | Fructose-bisphosphate aldolase A | | | | 0.9344 | -1.13511 | -2.67 | 0.039443 | 0.130402 | 0.123246 | 0.133906 | 0.319002 | 0.389179 | 2.287241 | 2.931856 | 4.427734 | 2.319588 | 3.274294 | 4.269138 | 4.021884 | 3.467426 | 3.335069 | 3.151449 | 4.10421 | 2.801218 |
| P05063 | P05063 | ALDOC_M | Aldoc | Fructose-bisphosphate aldolase C | | | | 0.960425 | -1.66199 | -2.76731 | 0.090433 | -0.27753 | -0.27067 | 0.000108 | -0.00028 | -0.00035 | 1.318838 | 2.284401 | 2.58101 | 0.542208 | 1.119535 | 2.11538 | 3.820848 | 3.106375 | 1.949444 | 1.728835 | 2.231099 | 1.004372 |
| Q3UGP8 | Q3UGP8 | AG10B_MC | Alg10b | Putative Dol-P-Glc:Glc(2)Man(9)GlcNAc(2)-PP-Dol alpha-1,2-glucosyltransferase | | | + | 0.960066 | 0.926841 | 2.765952 | 1.101457 | 0.674389 | 3.339486 | 0.053024 | 0.038059 | 0.163624 | 1.703919 | 2.090979 | 1.922721 | 1.980475 | 2.138786 | 1.771836 | 0.697055 | 1.24416 | 1.077341 | 1.477078 | 1.774289 | 2.060216 |
| Q3TZM9 | Q3TZM9 | ALG11_MC | Alg11 | GDP-Man:Man(3)GlcNAc(2)-PP-Dol alpha-1,2-mannosyltransferase | | | | 0.381824 | 0.970263 | 1.019751 | 0.570236 | 0.377532 | 1.514951 | NaN | 0 | NaN | -1.62584 | -3.527 | -2.93713 | -3.25068 | -3.24212 | -2.50425 | -3.58784 | -3.50552 | -3.66515 | -3.27772 | -3.30183 | -2.66298 |
| Q9DBE8 | Q9DBE8 | ALG2_MOI | Alg2 | Alpha-1,3/1,6-mannosyltransferase ALG2 | | | | 0.037808 | -0.01826 | -0.11832 | 0.163768 | 0.093946 | 0.46796 | 0.325123 | -0.13697 | -0.87691 | 1.824022 | 1.781042 | 1.692501 | 1.684249 | 1.680977 | 1.930809 | 1.973602 | 1.667964 | 1.795143 | 1.393715 | 1.849092 | 2.03663 |
| Q920R0 | Q920R0 | ALS2_MOI | Als2 | Alsin | | | | 0.084235 | 0.195794 | 0.253304 | 0.171005 | 0.085659 | 0.486806 | 0.576907 | -0.39258 | -1.53338 | -1.58871 | -1.32537 | -2.2327 | -0.88804 | -1.24889 | -1.50711 | -2.63732 | -2.46835 | -1.11046 | -1.1816 | -1.20651 | -0.76434 |
| Q7TQF2 | Q7TQF2 | FBX10_MC | Fbxo10 | F-box only protein 10 | + | | | 1.689373 | 2.231375 | 6.885661 | 0.497836 | -0.24269 | -1.31948 | 0.447351 | -1.20177 | -1.18738 | -1.44691 | -1.53201 | -1.34913 | -1.02786 | -3.46232 | -1.48585 | -3.39958 | -4.04209 | -0.85622 | -1.0354 | -1.49087 | -1.05377 |
| A2AH22 | A2AH22 | AMRA1_MC | Ambra1 | Activating molecule in BECN1-regulated autophagy protein 1 | | | | 0.722781 | 0.755428 | 1.958078 | 0.034618 | 0.01306 | 0.108671 | 0.93516 | -1.38264 | -2.67281 | -3.41005 | -3.09899 | -1.00575 | -1.10544 | -3.07261 | -2.09782 | -4.36301 | -3.65689 | -1.17801 | -0.9593 | -1.37591 | -1.02925 |
| P67871 | P67871 | CSK2B_MC | Csnk2b | Casein kinase II subunit beta | + | | | 1.436417 | 2.194242 | 5.081861 | 0.506441 | 0.509401 | 1.342308 | 0.663811 | 0.334138 | 1.781003 | 7.977158 | 8.288219 | 7.931432 | 8.27142 | 8.141483 | 7.9722 | 5.535652 | 6.341241 | 7.252732 | 7.931318 | 7.55527 | 7.890138 |
| Q8CCJ4 | Q8CCJ4 | AMER2_MC | Amer2 | APC membrane recruitment protein 2 | | | | 0.398622 | -0.58666 | -1.0624 | 0.85433 | -0.36702 | -2.38495 | 0.390694 | -0.36957 | -1.04225 | -1.2261 | -2.3256 | -0.28395 | 0.003166 | -0.83899 | -0.20857 | -1.2412 | -1.13719 | 0.171194 | 0.282053 | -0.31662 | 0.008199 |
| Q9DBT5 | Q9DBT5 | AMPD2_M | Ampd2 | AMP deaminase 2 | | + | | 0.282504 | 0.209937 | 0.770048 | 1.395018 | -0.52993 | -4.83142 | 0.965066 | -0.5701 | -2.78492 | 0.589594 | 0.521666 | 0.685791 | 0.499138 | 0.727728 | 1.016887 | 0.616198 | 0.075189 | 1.064767 | 1.180016 | 1.297482 | 1.587327 |
| O08739 | O08739;Q | AMPD3_M | Ampd3 | AMP deaminase 3 | | | | 0.561381 | 0.556999 | 1.490606 | 0.103314 | -0.08997 | -0.30635 | 0.099294 | -0.13418 | -0.29527 | -0.92509 | -0.31577 | -1.44599 | -1.71361 | -0.33479 | -1.09473 | -0.96106 | -1.39379 | -1.75125 | -1.22842 | -0.82982 | -0.33135 |
| Q7TQF7 | Q7TQF7 | AMPH_MO | Amph | Amphiphysin | | | | 0.277804 | 0.094628 | 0.758255 | 0.097516 | 0.070346 | 0.290352 | 0.787027 | -0.27468 | -2.16063 | 1.196478 | 1.193017 | 2.797707 | 2.398974 | 2.261961 | 2.171149 | 1.224905 | 0.975335 | 2.390332 | 2.665658 | 2.372493 | 2.609985 |
| Q02357 | Q02357 | ANK1_MOI | Ank1 | Ankyrin-1 | | | | 0.518939 | 0.642373 | 1.37565 | 0.752164 | -0.44932 | -2.0494 | 0.099801 | -0.15501 | -0.29667 | -0.87172 | -0.66325 | -2.31393 | -2.5729 | -1.36393 | -2.40893 | -0.95468 | -1.86504 | -2.17102 | -1.81717 | -1.7273 | -1.73553 |
| Q8C8R3 | Q8C8R3 | ANK2_MOI | Ank2 | Ankyrin-2 | | | | 0.56941 | 0.484385 | 1.512675 | 0.604428 | 0.137876 | 1.610235 | 0.377127 | 0.475606 | 1.007859 | 0.862952 | 0.988641 | 1.651769 | 1.591192 | 2.321736 | 1.384857 | 0.755402 | 0.127421 | 1.403516 | 1.563694 | 1.434708 | 1.320674 |
| G5E8K5 | G5E8K5 | ANK3_MOI | Ank3 | Ankyrin-3 | | | | 0.221596 | 0.308249 | 0.616573 | 0.566266 | -0.12355 | -1.50402 | 0.079129 | -0.07098 | -0.23938 | 0.155327 | 0.326761 | 1.000277 | 0.973728 | 1.128305 | 0.958212 | 0.425331 | -0.55974 | 1.029484 | 1.191613 | 0.830194 | 1.398274 |
| Q810B6 | Q810B6 | ANFY1_MC | Ankfy1 | Rabankyrin-5 | | | | NaN | 0 | NaN | 0.088266 | -0.07571 | -0.26461 | 0.291853 | 0.190443 | 0.793492 | -3.95971 | -3.07355 | -0.95145 | -1.04129 | -0.56925 | -0.62615 | -4.00474 | -2.27279 | -1.20324 | -0.63808 | -1.02645 | -0.54983 |

| | | | | | | | | | | | | | | | | | | | | | | | | | | | | |
|---|---|---|---|---|---|---|---|---|---|---|---|---|---|---|---|---|---|---|---|---|---|---|---|---|---|---|---|---|
| Q9WUD1 | Q9WUD1 | CHIP_MOI | Stub1 | E3 ubiquitin-protein ligase CHIP | + | | | 1.486535 | 2.175676 | 5.400523 | 0.024472 | -0.02948 | -0.0776 | 0.639119 | -0.50542 | -1.70913 | -0.82572 | -1.26156 | 0.308319 | -0.07725 | -0.73596 | -0.21024 | -2.88048 | -3.55816 | -0.18227 | 0.472294 | 0.167781 | -0.10315 |
| Q505D1 | Q505D1 | ANR28_M( | Ankrd28 | Serine/threonine-protein phosphatase 6 regulatory ankyrin repeat subunit A | | | | 0.085477 | -0.47349 | -0.2568 | 0.076431 | -0.05891 | -0.23125 | 0.197064 | -0.4193 | -0.55402 | -2.52165 | -3.5225 | -0.57473 | -0.14822 | -1.669 | -1.55267 | -0.77395 | -4.32322 | -0.16318 | -0.44194 | -1.94613 | -0.43696 |
| D3YVV3 | D3YVV3 | D3YVV3_M | Ankrd29 | Ankyrin repeat domain 29 | | + | | 0.842538 | -0.92282 | -2.34469 | 2.636791 | -2.17571 | -20.7799 | 0.607404 | -0.85831 | -1.61863 | 0.245968 | 0.21507 | -3.5467 | -3.54885 | -1.72429 | -2.78307 | 1.546619 | 0.760066 | -1.47676 | -1.26737 | -1.3649 | -1.42584 |
| Q3UUF8 | Q3UUF8 | AN34B_M( | Ankrd34b | Ankyrin repeat domain-containing protein 34B | | + | | 0.216207 | 0.675974 | 0.602883 | 1.946101 | -0.80322 | -9.31833 | 0.550719 | -1.22799 | -1.46146 | -0.88523 | -1.90804 | -1.77065 | -1.79302 | -3.43149 | -1.93354 | -1.0748 | -3.07042 | -0.89314 | -1.06408 | -1.83538 | -1.07367 |
| A2ARS0 | A2ARS0 | ANR63_M( | Ankrd63 | Ankyrin repeat domain-containing protein 63 | | | | NaN | 0 | NaN | 0.834306 | 0.658337 | 2.31683 | 0.650782 | -1.3216 | -1.74292 | -3.61887 | -3.92295 | 1.244811 | 1.800537 | -1.33104 | -2.47258 | -3.02666 | -3.26232 | 0.923798 | 0.804876 | -1.0794 | -0.08102 |
| Q8BIZ1 | Q8BIZ1 | ANS1B_M( | Anks1b | Ankyrin repeat and sterile alpha motif domain-containing protein 1B | | | | 0.668585 | 0.970028 | 1.795049 | 0.463885 | -0.22688 | -1.23032 | 0.184533 | -0.20903 | -0.52182 | -1.86522 | -1.15859 | 2.114817 | 2.387508 | 2.821749 | 2.249983 | -2.07304 | -2.89082 | 2.35388 | 2.602214 | 2.464305 | 3.025477 |
| Q8K298 | Q8K298 | ANLN_MO | Anln | Anillin | | | | 0.810144 | -0.18677 | -2.23621 | 0.440796 | -0.37179 | -1.17044 | 0.100753 | 0.044555 | 0.299296 | 1.295005 | 1.280024 | -0.69883 | -0.39442 | 0.140734 | 0.205144 | 1.391098 | 1.557462 | 0.103964 | -0.45365 | 0.273726 | -0.01696 |
| O35381;Q | Q64G17;C | AN32A_M( | Anp32a;Anp32c | Acidic leucine-rich nuclear phosphoprotein 32 family member A | | | | 0.457945 | -0.88785 | -1.21486 | 0.208074 | 0.861348 | 0.582172 | 0.051751 | 0.261006 | 0.159877 | -3.40363 | -3.52418 | -0.17662 | -2.85099 | -3.15607 | -1.2661 | -3.30439 | -1.84772 | -3.00838 | -1.74193 | -1.14086 | -3.80332 |
| Q9EST5 | Q9EST5 | AN32B_M( | Anp32b | Acidic leucine-rich nuclear phosphoprotein 32 family member B | | | | 0.050949 | -0.25757 | -0.15751 | 0.176528 | 0.649842 | 0.501131 | 0.163718 | 0.765234 | 0.467828 | -3.54942 | -1.3098 | 0.514804 | -1.99749 | -1.67831 | -0.56386 | -3.3637 | -0.98037 | -1.71314 | -1.06923 | -0.34844 | -3.4242 |
| P10107 | P10107 | ANXA1_M( | Anxa1 | Annexin A1 | | | | 0.457096 | -1.98437 | -1.21265 | 0.455549 | -1.26746 | -1.20864 | 0.287108 | -0.78799 | -0.78159 | -1.69241 | -3.18148 | -0.58321 | -2.61227 | -3.56697 | -1.90689 | 1.004634 | -1.90977 | -0.06488 | -0.59569 | -1.37667 | -2.5212 |
| Q9DBZ5 | Q9DBZ5 | EIF3K_MOI | Eif3k | Eukaryotic translation initiation factor 3 subunit K | + | | + | 1.550137 | 2.162525 | 5.830925 | 0.388162 | 0.410316 | 1.035819 | 1.075886 | 0.363496 | 3.229739 | -1.5784 | -0.89704 | -0.53954 | -0.85964 | -0.94362 | -1.16776 | -3.25367 | -3.54682 | -1.47226 | -0.74755 | -1.42953 | -1.40885 |
| P07356 | P07356 | ANXA2_M( | Anxa2 | Annexin A2 | | | | 0.031987 | 0.04449 | 0.100671 | 0.337847 | -0.31308 | -0.90886 | 0.333382 | 0.116355 | 0.897643 | 1.801589 | 0.937506 | 0.135999 | 0.148002 | 0.215526 | 0.456656 | 1.23206 | 1.418054 | 0.110656 | 0.799503 | 0.172133 | 0.267339 |
| P48036 | P48036 | ANXA5_M( | Anxa5 | Annexin A5 | + | | | 1.218263 | -1.42119 | -3.87882 | 0.383573 | 0.682398 | 1.024182 | 0.29037 | 0.486449 | 0.789773 | -0.08976 | 0.29979 | 0.745138 | -0.54246 | -0.38878 | 0.356256 | 1.83655 | 1.215868 | -0.40943 | -0.75269 | -0.0122 | -0.99323 |
| Q99JB2 | Q99JB2 | STML2_M( | Stoml2 | Stomatin-like protein 2, mitochondrial | + | | | 1.850974 | 2.14613 | 8.334095 | 0.615442 | 0.603476 | 1.641381 | 0.322502 | -0.55127 | -0.87034 | 3.022642 | 3.207904 | 2.40105 | 3.06017 | 0.972682 | 1.180479 | 1.209417 | 0.728868 | 1.96414 | 2.290127 | 1.003031 | 2.252676 |
| Q07076 | Q07076 | ANXA7_M( | Anxa7 | Annexin A7 | | | | 0.311075 | -0.37162 | -0.84168 | 0.283616 | -0.22535 | -0.77284 | 0.429042 | 0.432026 | 1.14017 | -0.19005 | -1.05403 | -0.50307 | -1.07995 | 0.360668 | 0.392195 | -0.34169 | -0.15914 | -0.60883 | -0.5235 | 0.322991 | -0.43418 |
| O35643 | O35643 | AP1B1_M( | Ap1b1 | AP-1 complex subunit beta-1 | | + | | 0.488976 | 0.23397 | 1.296072 | 1.170652 | 0.277603 | 3.651132 | 0.385955 | 0.180487 | 1.03022 | 2.500576 | 2.407382 | 2.285181 | 2.372827 | 2.411144 | 2.101246 | 2.394414 | 2.045603 | 1.989268 | 2.113533 | 2.15746 | 1.993956 |
| P22892 | P22892 | AP1G1_M( | Ap1g1 | AP-1 complex subunit gamma-1 | | | | 0.299074 | -0.29862 | -0.81159 | 0.423718 | -0.28735 | -1.1265 | 0.519769 | 0.540013 | 1.377872 | -1.16731 | -0.43212 | -0.28338 | -0.68253 | 0.68189 | -0.08911 | -0.48497 | -0.5172 | -0.35446 | -0.03674 | -0.173 | -0.31425 |
| P35585 | P35585 | AP1M1_M( | Ap1m1 | AP-1 complex subunit mu-1 | | | | 0.104745 | 0.349634 | 0.310278 | 0.212909 | -0.12779 | -0.59449 | 0.589196 | 0.713558 | 1.567529 | -1.32561 | -0.36848 | 0.057221 | -0.29326 | 1.266881 | 0.364447 | -0.17651 | -2.21685 | -0.11473 | 0.134278 | 0.04193 | 0.162282 |
| P61967 | P61967 | AP1S1_M( | Ap1s1 | AP-1 complex subunit sigma-1A | | | | 0.641728 | 1.612757 | 1.716669 | 0.405154 | -0.50371 | -1.07903 | 0.30498 | 0.495226 | 0.826398 | -1.29452 | -0.541 | -0.8487 | -1.70912 | 0.135061 | -1.05883 | -1.6699 | -3.39113 | -0.59402 | -0.95639 | -0.90452 | -1.0097 |
| Q9DB50 | Q9DB50 | AP1S2_M( | Ap1s2 | AP-1 complex subunit sigma-2 | | | + | 0.061873 | 0.02435 | 0.189462 | 0.364071 | -0.38316 | -0.97487 | 1.059023 | 0.401796 | 3.158883 | 0.074604 | -0.06766 | -1.15522 | -1.88109 | -0.39652 | -0.57928 | 0.086165 | -0.12792 | -0.98417 | -1.28583 | -0.97818 | -0.80121 |
| P28654 | P28654 | PGS2_MOI | Dcn | Decorin | + | | | 1.356666 | 2.145447 | 4.609202 | 0.010967 | 0.027683 | 0.035275 | 0.56211 | 0.786367 | 1.492603 | 0.001385 | -0.81917 | -2.61917 | -1.82246 | -1.32397 | -0.42102 | -2.77419 | -2.33448 | -1.57236 | -2.92464 | -1.9304 | -1.38732 |
| A0A075B5 | A0A075B5 | A0A075B5 | Ighv4-1 | Immunoglobulin heavy variable 4-1 (Fragment) | + | | + | 1.810076 | 2.135213 | 7.94233 | 0.173635 | 0.239877 | 0.493632 | 1.012257 | 0.806989 | 2.968465 | -1.0547 | -1.54385 | -2.74706 | -2.29012 | -2.37455 | -2.22234 | -3.5461 | -3.32287 | -2.32958 | -3.18735 | -2.84445 | -3.36642 |
| Q0VAV2 | Q0VAV2 | EXPH5_M( | Exph5 | Exophilin-5 | + | | | 1.597884 | 2.133161 | 6.174433 | 0.920571 | -0.14882 | -2.61926 | 0.050356 | 0.17998 | 0.155764 | -1.49394 | -1.13745 | -1.47229 | -1.44432 | -3.16617 | -1.279 | -3.1529 | -3.7448 | -1.36455 | -1.25441 | -3.06945 | -1.73567 |
| Q01097 | Q01097 | NMDE2_M | Grin2b | Glutamate receptor ionotropic, NMDA 2B | + | | | 2.209369 | 2.127268 | 12.66677 | 0.027263 | -0.0146 | -0.08621 | 0.230329 | 0.377843 | 0.638706 | -0.8725 | -1.11477 | 1.75882 | 2.097418 | 3.217961 | 2.122289 | -3.00458 | -3.23722 | 1.945995 | 1.939438 | 2.069039 | 2.515525 |
| D3Z3N4 | D3Z3N4 | D3Z3N4_M | Hnrnph3 | Heterogeneous nuclear ribonucleoprotein H3 | + | | | 2.594289 | 2.116645 | 19.78401 | 0.046735 | 0.056417 | 0.145041 | 0.388552 | 0.512976 | 1.036808 | 2.533718 | 2.718742 | 0.617936 | 1.193484 | 2.313271 | 1.546739 | 0.455846 | 0.563323 | 0.587596 | 1.11099 | 1.10414 | 1.729918 |
| Q9Z1T1 | Q9Z1T1 | AP3B1_M( | Ap3b1 | AP-3 complex subunit beta-1 | | + | | NaN | 0 | NaN | 1.479263 | -0.14414 | -5.3532 | 0.10342 | -0.19691 | -0.30664 | -3.69015 | -2.95638 | -1.80073 | -1.83789 | -1.39261 | -2.56099 | -3.78239 | -3.15829 | -1.69466 | -1.65568 | -2.04654 | -1.51323 |
| Q9JME5 | Q9Z1T1;Q | AP3B2_M( | Ap3b2 | AP-3 complex subunit beta-2 | | + | | 0.66866 | 0.356989 | 1.795269 | 1.037824 | 0.288407 | 3.071481 | 0.446196 | 0.452004 | 1.184395 | 0.498585 | 0.81629 | 2.047699 | 2.010793 | 2.200927 | 1.443149 | 0.420061 | 0.180836 | 1.648772 | 1.832906 | 1.32435 | 1.415717 |
| O54774 | O54774 | AP3D1_M( | Ap3d1 | AP-3 complex subunit delta-1 | | | | 0.589556 | 0.31135 | 1.568535 | 0.341219 | -0.09423 | -0.91733 | 0.78397 | 0.477113 | 2.150748 | 0.641784 | 0.789256 | 1.560941 | 1.35656 | 1.988771 | 1.579892 | 0.588464 | 0.219876 | 1.54254 | 1.563423 | 1.221107 | 1.393332 |
| Q9JKC8 | Q9JKC8 | AP3M1_M( | Ap3m1 | AP-3 complex subunit mu-1 | | | | 0.741132 | 0.80012 | 2.014858 | 0.227533 | 0.327461 | 0.631625 | 0.343049 | 0.340537 | 0.921933 | -0.21666 | -1.00613 | -0.63939 | -0.51443 | 0.326204 | -0.38757 | -1.36817 | -1.45487 | -1.41903 | -0.38971 | -0.46645 | -0.276 |
| Q8R2R9 | Q9JKC8;Q | AP3M2_M( | Ap3m2 | AP-3 complex subunit mu-2 | | | | 0.71413 | 0.197598 | 1.931596 | 0.316428 | 0.240092 | 0.8551 | 0.268039 | 0.392299 | 0.733742 | 1.48156 | 1.417478 | 2.438702 | 2.351902 | 2.902815 | 1.945703 | 1.349072 | 1.15477 | 1.877809 | 2.432612 | 1.793549 | 2.270371 |
| Q9DCR2 | Q9DCR2 | AP3S1_M( | Ap3s1 | AP-3 complex subunit sigma-1 | | | | 0.62131 | 0.253644 | 1.65807 | 0.632723 | 0.390327 | 1.690722 | 0.402862 | 0.360939 | 1.073191 | -1.15686 | -1.45595 | -0.90452 | -1.28385 | -0.6715 | -0.09302 | -1.59227 | -1.52783 | -1.61614 | -1.35288 | -0.57158 | -0.91482 |
| Q8BSZ2 | Q8BSZ2 | AP3S2_M( | Ap3s2 | AP-3 complex subunit sigma-2 | | | + | 0.035917 | 0.018669 | 0.112609 | 0.324814 | 0.136317 | 0.876137 | 2.241642 | 0.415927 | 13.15066 | -0.71252 | -1.03882 | -0.88845 | -0.82303 | -0.28768 | -0.32621 | -0.92379 | -0.86489 | -1.14417 | -0.83994 | -0.74795 | -0.69778 |
| Q6A009 | Q6A009 | LTN1_MOI | Ltn1 | E3 ubiquitin-protein ligase listerin | + | | + | 2.569045 | 2.111973 | 19.21509 | 0.939508 | 1.324728 | 2.688913 | 1.255133 | -1.11945 | -4.06307 | -1.21525 | -1.30109 | -1.39195 | -0.55437 | -2.92873 | -2.44173 | -3.47133 | -3.26896 | -2.55737 | -2.0384 | -1.6947 | -1.43687 |
| P98084 | P98084 | APBA2_M( | Apba2 | Amyloid-beta A4 precursor protein-binding family A member 2 | | | | 0.097917 | 0.167778 | 0.291462 | 0.25687 | 0.399592 | 0.705666 | 0.170176 | 0.271777 | 0.48465 | -3.73023 | -2.68982 | -0.21544 | -0.45829 | -0.51586 | -1.11049 | -3.62428 | -3.13132 | -1.28955 | -0.18337 | -1.56042 | -0.60949 |
| Q9QXJ1 | Q9DBR4;C | APBB1_M( | Apbb1 | Amyloid beta precursor protein binding family B member 1 | | | | 0.204912 | -0.82208 | -0.5741 | 0.399626 | 0.776639 | 1.064951 | 0.060112 | -0.05668 | -0.18435 | -1.18504 | -3.66793 | 0.020782 | 0.310649 | -0.00202 | -0.48966 | -0.89077 | -2.31804 | -1.32565 | 0.103801 | -0.00183 | -0.37649 |
| Q61315 | Q61315 | APC_MOU | Apc | Adenomatous polyposis coli protein | | | | 0.265154 | -0.17955 | -0.72649 | 0.119286 | -0.07646 | -0.3499 | 0.39974 | -0.46035 | -1.06524 | -0.66686 | -0.8852 | 0.628191 | 0.989354 | -0.00193 | 0.547042 | -0.37475 | -0.81821 | 0.76219 | 1.008272 | 0.399117 | 1.06669 |
| Q9Z1K7 | Q9Z1K7 | APCL_MOI | Apc2 | Adenomatous polyposis coli protein 2 | | | | 0.650682 | 0.10398 | 1.742631 | 0.374849 | 0.260565 | 1.002099 | 0.262105 | -0.27112 | -0.71883 | 1.231651 | 1.16332 | 1.8865 | 2.399117 | 1.179838 | 1.339556 | 1.044586 | 1.142424 | 1.926014 | 1.838473 | 1.162202 | 1.899428 |
| Q9WVQ5 | Q9WVQ5 | MTNB_MO | Apip | Methylthioribulose-1-phosphate dehydratase | | | | 0.52289 | -1.31565 | -1.38624 | 0.570491 | -1.63553 | -1.51565 | 0.275429 | -0.73831 | -0.7523 | -2.77005 | -4.45339 | -1.51759 | -3.53481 | -2.49029 | -0.93311 | -2.73464 | -1.8575 | -0.50708 | -1.27426 | -0.37594 | -1.57085 |
| Q03157 | Q03157 | APLP1_M( | Aplp1 | Amyloid beta precursor like protein 1 | | + | | 0.40088 | -1.65038 | -1.06814 | 1.19568 | 0.620034 | 3.76942 | 0.076162 | -0.05514 | -0.23048 | -1.26922 | -4.34675 | 0.937691 | 0.610513 | 0.00326 | 0.169269 | -1.01796 | -1.29725 | 0.171264 | 0.136872 | -0.08297 | 0.365773 |
| Q00623 | Q00623 | APOA1_M( | Apoa1 | Apolipoprotein A-I | | | | 0.620031 | 1.094306 | 1.654427 | 0.270543 | 0.516695 | 0.74003 | 0.750047 | 0.508362 | 2.042747 | 0.969559 | 2.136852 | 0.197402 | -0.98401 | -0.1152 | -0.60414 | 0.147674 | 0.770125 | -0.53776 | -1.28224 | -0.82147 | -0.91459 |
| P08226 | P08226 | APOE_MO | Apoe | Apolipoprotein E | | | | 0.685626 | 0.548451 | 1.845587 | 0.459013 | 0.871989 | 1.217635 | 0.14565 | 0.191418 | 0.420373 | -0.48836 | -0.23574 | 0.668897 | -0.7374 | -0.22278 | -0.57866 | -1.17949 | -0.64151 | -0.77048 | -1.042 | -0.17299 | -1.01129 |
| Q9DCZ4 | Q9DCZ4 | MIC26_M( | Apoo | MICOS complex subunit Mic26 | | | | 0.524078 | -0.35618 | -1.38942 | 0.080776 | 0.12513 | 0.243555 | 0.26779 | 0.381072 | 0.733116 | -0.33074 | 0.145099 | 0.193111 | -0.81337 | 0.535934 | 0.146687 | 0.358796 | 0.167917 | -0.53872 | -0.3318 | 0.442225 | -0.52175 |
| Q6PD28 | Q6PD28 | 2A5B_MOI | Ppp2r5b | Serine/threonine-protein phosphatase 2A 56 kDa regulatory subunit beta isoform | + | | | 1.898848 | 2.095504 | 8.816207 | 0.439021 | 0.193364 | 1.165861 | 0.449597 | -0.31744 | -1.1932 | -0.59337 | -0.99364 | -0.43449 | -0.21592 | -0.75218 | -1.28149 | -2.76079 | -3.01723 | -0.64333 | -0.39381 | -0.72649 | -0.67232 |
| Q8K3H0 | Q8K3H0 | DP13A_M( | Appl1 | DCC-interacting protein 13-alpha | | | | 0.13141 | 0.256097 | 0.382508 | 0.3644 | 0.211671 | 0.975705 | 0.193711 | 0.393067 | 0.545426 | -0.61737 | -1.43842 | -0.4016 | -0.75563 | 0.600389 | -0.83569 | -0.7551 | -1.81288 | -0.66486 | -0.91571 | -0.57212 | -0.44932 |
| P55088 | P55088 | AQP4_MO | Aqp4 | Aquaporin-4 | + | + | | 1.10721 | -0.68144 | -3.36457 | 1.131482 | 0.135721 | 3.472021 | 0.02616 | 0.027848 | 0.082814 | 0.882188 | 1.282017 | 0.144184 | 0.120451 | 0.628107 | 1.011385 | 1.796021 | 1.731071 | -0.04065 | 0.033842 | 1.068217 | 0.515579 |
| Q8BZ05 | Q8BZ05 | ARAP2_M( | Arap2 | Arf-GAP with Rho-GAP domain, ANK repeat and PH domain-containing protein 2 | | | | 0.164035 | 0.692323 | 0.468656 | 0.007062 | -0.0103 | -0.02281 | 0.147481 | 0.342044 | 0.42521 | -3.61635 | -1.54456 | 0.07136 | 0.671245 | 0.470735 | -0.35046 | -2.21959 | -4.32596 | 0.043988 | 0.719222 | -0.97363 | 0.409821 |
| Q5XJY5 | Q5XJY5 | COPD_MC | Arcn1 | Coatomer subunit delta | | | | 0.042483 | -0.07215 | -0.13236 | 0.013803 | -0.00559 | -0.04426 | 0.305293 | 0.270467 | 0.827182 | -1.78473 | -0.87779 | -0.5804 | -0.75951 | -0.32832 | -0.81764 | -0.95663 | -1.56159 | -0.75326 | -0.57547 | -1.06036 | -0.62653 |
| P61205;P( | P61205;P( | ARF1_MOI | Arf1;Arf3 | ADP-ribosylation factor 3 | | | | 0.504289 | 0.944623 | 1.33659 | 0.006994 | 0.02029 | 0.022595 | 0.444383 | 1.12975 | 1.179708 | -1.44614 | -2.80902 | 0.249258 | -1.47984 | -1.16223 | 0.673367 | -2.88478 | -3.25962 | -0.39282 | -0.87834 | -1.64758 | -1.10079 |
| Q8BSL7 | P61205;Q | ARF2_MOI | Arf2 | ADP-ribosylation factor 2 | | | | 0.437605 | -0.14185 | -1.16221 | 0.147512 | 0.167819 | 0.425291 | 0.460527 | -0.12943 | -1.22158 | 3.351649 | 3.40577 | 4.294244 | 3.62134 | 3.965526 | 4.159159 | 3.401545 | 3.639566 | 3.583799 | 3.996147 | 4.234803 | 4.148734 |
| P61750 | P61750 | ARF4_MOI | Arf4 | ADP-ribosylation factor 4 | | | + | 0.163395 | -0.03911 | -0.46699 | 0.335994 | 0.102208 | 0.904204 | 1.420587 | 0.189721 | 4.98477 | 1.677046 | 1.804875 | 1.562838 | 1.4037 | 1.996846 | 1.931671 | 1.725943 | 1.834205 | 1.461348 | 1.300773 | 1.7942 | 1.754875 |
| Q8BH51 | Q8BH51 | COX14_M( | Cox14 | Cytochrome c oxidase assembly protein COX14 | + | | | 1.908645 | 2.063576 | 8.918128 | 0.281632 | 3.087557 | 0.76786 | 0.006092 | -0.10597 | -0.0197 | 3.409376 | 2.995021 | 3.106424 | 3.735089 | 3.990751 | -3.40682 | 1.241673 | 1.035572 | 4.341884 | -3.67548 | 4.303276 | -3.5074 |
| P62331 | P62331 | ARF6_MOI | Arf6 | ADP-ribosylation factor 6 | | | | 0.081066 | -0.09459 | -0.24437 | 0.178641 | -0.11241 | -0.5066 | 0.130701 | -0.02454 | -0.38061 | -0.09856 | 0.082008 | 0.508328 | 0.067238 | 0.635054 | 0.839706 | -0.29008 | 0.462705 | 0.375765 | 0.424623 | 0.862012 | 0.709819 |
| Q9EPJ9 | Q9EPJ9 | ARFG1_M( | Arfgap1 | ADP-ribosylation factor GTPase-activating protein 1 | | | | 0.260297 | -0.27241 | -0.71428 | 0.177559 | -0.05448 | -0.5038 | 0.037729 | 0.026875 | 0.118082 | 0.892381 | 0.762801 | 1.870357 | 1.663605 | 1.751401 | 2.170681 | 0.724169 | 1.475839 | 1.853222 | 1.789706 | 2.022768 | 1.845565 |
| Q9D8S3 | Q9D8S3 | ARFG3_M( | Arfgap3 | ADP-ribosylation factor GTPase-activating protein 3 | | | | 0.227232 | -0.60101 | -0.63086 | 0.181198 | -0.17574 | -0.51321 | 0.378546 | 1.436773 | 1.011451 | -2.5454 | -3.97776 | -1.31189 | -0.62889 | -1.79599 | -1.1603 | -2.03233 | -3.28881 | -0.81994 | -0.76935 | -4.29941 | -1.53043 |
| G3X9K3 | G3X9K3 | BIG1_MOI | Arfgef1 | Brefeldin A-inhibited guanine nucleotide-exchange protein 1 | + | | | 1.095199 | 0.207968 | 3.312365 | 0.502177 | 0.346045 | 1.330984 | 0.185143 | -0.17617 | -0.52339 | -0.64045 | -0.51588 | -0.25201 | 0.263703 | -1.04553 | -0.7351 | -0.79407 | -0.7782 | -0.37346 | -0.30694 | -1.01281 | -0.41549 |
| A2A5R2 | G3X9K3;A | BIG2_MOI | Arfgef2 | Brefeldin A-inhibited guanine nucleotide-exchange protein 2 | | + | | 0.957258 | 0.351095 | 2.75534 | 1.202534 | 0.413136 | 3.802351 | 0.303592 | -0.28671 | -0.82292 | 0.371929 | 0.305038 | 0.754217 | 0.916241 | -0.09898 | 0.004996 | 0.110344 | -0.13557 | 0.349688 | 0.494498 | -0.10479 | 0.584222 |
| Q3UGY8 | Q3UGY8 | BIG3_MOI | Arfgef3 | Brefeldin A-inhibited guanine nucleotide-exchange protein 3 | | | | 0.002424 | -0.00133 | -0.00787 | 0.691704 | 0.29479 | 1.86377 | 0.426711 | -0.31854 | -1.13418 | -1.06331 | -1.29162 | -0.16241 | -0.00137 | -1.2817 | -1.01571 | -1.05148 | -1.30079 | -0.51282 | -0.24054 | -1.07753 | -0.58279 |
| Q61176 | Q61176 | ARGI1_MC | Arg1 | Arginase-1 | | | | 0.101002 | 0.14354 | 0.299982 | 0.373874 | 0.127415 | 0.999633 | 0.843143 | -0.3629 | -2.34674 | 1.32486 | 0.543927 | -0.61257 | -0.86622 | -0.4907 | -0.419 | 0.51428 | 1.067427 | -0.87956 | -0.85407 | -0.24238 | 0.058477 |
| Q3UL36 | Q3UL36 | ARGL1_M( | Arglu1 | Arginine and glutamate-rich protein 1 | | | + | 0.72873 | 0.728576 | 1.976396 | 0.297463 | 0.231913 | 0.807555 | 1.027605 | 0.878011 | 3.029995 | 2.693665 | 3.226511 | 2.507153 | 3.061927 | 2.320452 | 2.012687 | 2.486293 | 1.97673 | 2.626981 | 2.478272 | 1.04302 | 1.534096 |
| Q5FWK3 | Q5FWK3 | RHG01_M( | Arhgap1 | Rho GTPase-activating protein 1 | | | + | 0.039423 | 0.206602 | 0.123187 | 0.042184 | -0.06015 | -0.13147 | 1.058119 | 0.785104 | 3.155121 | -0.99292 | -2.92997 | 0.42776 | -0.48569 | 0.751699 | 0.447194 | -3.53728 | -0.79882 | 0.05842 | 0.003953 | 0.011162 | -0.38248 |
| Q6Y5D8 | Q6Y5D8 | RHG10_M( | Arhgap10 | Rho GTPase-activating protein 10 | | | | 0.152333 | 0.927226 | 0.437998 | 0.358607 | -0.65452 | -0.96109 | 0.332764 | -0.87016 | -0.89609 | -4.19599 | -0.17824 | 1.022093 | -0.30943 | -2.38224 | -0.5062 | -2.44654 | -3.78214 | 0.867507 | 1.154205 | -0.8252 | -0.32291 |
| Q8C0D4 | Q8C0D4 | RHG12_M( | Arhgap12 | Rho GTPase-activating protein 12 | | | + | 0.509868 | -1.12393 | -1.35143 | 0.882847 | 0.142111 | 2.484088 | 1.240955 | -0.32839 | -3.99137 | -3.5362 | -3.40534 | -2.15589 | -2.0577 | -1.76024 | -1.61087 | -1.51776 | -3.17592 | -2.21954 | -2.27828 | -1.32265 | -1.39169 |
| Q8BRH3 | Q8BRH3 | RHG19_M( | Arhgap19 | Rho GTPase-activating protein 19 | | | + | NaN | 0 | NaN | 0.046272 | -0.06512 | -0.14366 | 1.076133 | -0.65543 | -3.23079 | -2.54084 | -3.55328 | 0.74635 | 1.59104 | 0.808879 | 0.876009 | -3.62928 | -3.22344 | 1.398352 | 1.069272 | 1.697948 | 1.2978 |
| Q6IFT4 | Q6IFT4 | RHG20_M( | Arhgap20 | Rho GTPase-activating protein 20 | | | + | NaN | 0 | NaN | 0.215107 | -0.20814 | -0.60008 | 1.307532 | -1.8832 | -4.33754 | -3.41907 | -2.45412 | -1.40031 | -1.21015 | -3.24146 | -3.98078 | -3.64218 | -3.3806 | -1.43065 | -0.76353 | -1.95563 | -1.50021 |
| Q6DFV3 | Q6DFV3 | RHG21_M( | Arhgap21 | Rho GTPase-activating protein 21 | | | | 0.910193 | 2.116065 | 2.581603 | 0.275891 | -0.23602 | -0.75345 | 0.403218 | -0.33115 | -1.0741 | -1.84098 | -0.21358 | -0.38504 | -0.31011 | -0.62235 | -0.01866 | -3.04458 | -3.24212 | -0.42256 | 0.199434 | -0.05214 | 0.073428 |
| Q8BL80 | Q8BL80 | RHG22_M( | Arhgap22 | Rho GTPase-activating protein 22 | | | | 0.535489 | -0.93033 | -1.42015 | 0.338602 | -0.68137 | -0.91076 | 0.494467 | -0.82043 | -1.31057 | -3.27804 | -2.26284 | -3.29223 | -1.79787 | -3.50829 | -2.39076 | -1.42599 | -2.25422 | -1.82582 | -1.90153 | -1.84683 | -2.41136 |
| Q69ZH9 | Q69ZH9 | RHG23_M( | Arhgap23 | Rho GTPase-activating protein 23 | | | | 0.743576 | 0.967626 | 2.022485 | 0.382002 | 0.343032 | 1.020201 | 0.162925 | 0.244058 | 0.465757 | -2.63576 | -1.71981 | -0.72712 | -0.87448 | -0.90143 | -1.22171 | -3.007 | -2.38281 | -1.4719 | -0.81576 | -1.80456 | -0.8067 |
| Q8BYW1 | Q8BYW1 | RHG25_M( | Arhgap25 | Rho GTPase-activating protein 25 | | | | NaN | 0 | NaN | 0.233204 | 0.344632 | 0.64598 | NaN | 0 | NaN | -3.79291 | -3.0238 | -3.41448 | -3.34216 | -3.66873 | -3.32317 | -3.39843 | -3.13544 | -4.25523 | -3.19068 | -3.24431 | -3.49679 |
| O88951 | O88951 | LIN7B_MO | Lin7b | Protein lin-7 homolog B | + | | | 1.826364 | 2.058452 | 8.096197 | 0.456143 | 0.437613 | 1.210177 | 0.070314 | 0.111104 | 0.213795 | -1.03149 | -1.39221 | -0.16075 | -0.74716 | -1.61796 | -1.47225 | -3.0911 | -3.4495 | -1.10322 | -0.67992 | -2.17075 | -1.14167 |
| Q8CGF1 | Q8CGF1 | RHG29_M( | Arhgap29 | Rho GTPase-activating protein 29 | | | | 0.272196 | -0.68099 | -0.74418 | 0.110103 | -0.14885 | -0.32495 | 0.32806 | -1.30152 | -0.88428 | -3.55356 | -4.0149 | -1.44316 | -0.54919 | -3.73184 | -0.93055 | -3.98878 | -2.2177 | -0.94748 | -0.74718 | -0.57745 | -1.48189 |
| A6X8Z5 | A6X8Z5 | RHG31_M( | Arhgap31 | Rho GTPase-activating protein 31 | | | | 0.204524 | -0.25325 | -0.57311 | 0.009035 | -0.07066 | -0.02912 | 0.579727 | -1.82007 | -1.54119 | -2.91861 | -3.64205 | -0.30574 | -3.59767 | -4.47878 | -2.19756 | -2.77328 | -3.28088 | -3.6637 | -0.09838 | -1.82413 | -1.21205 |
| Q811P8 | Q811P8 | RHG32_M( | Arhgap32 | Rho GTPase-activating protein 32 | | + | | 0.631089 | 1.223438 | 1.686033 | 1.053883 | -0.27757 | -3.13753 | 0.522844 | -0.90267 | -1.38611 | -0.19728 | -0.9282 | 2.314073 | 2.437769 | 0.499777 | 1.240325 | -1.1593 | -2.41306 | 2.590237 | 2.716754 | 1.237007 | 2.308443 |
| Q80YF9 | Q80YF9 | RHG33_M( | Arhgap33 | Rho GTPase-activating protein 33 | | | | NaN | 0 | NaN | 0.07956 | -0.10671 | -0.24012 | 0.036429 | 0.052935 | 0.114155 | -3.80011 | -3.58443 | -1.35491 | -1.02582 | -1.6044 | -2.15792 | -3.41643 | -3.09864 | -1.49646 | -0.67085 | -2.30616 | -1.56203 |
| O35286 | O35286;G | DHX15_M( | Dhx15 | ATP-dependent RNA helicase DHX15 | + | | | 1.324537 | 2.053464 | 4.429955 | 0.211122 | -0.14154 | -0.58994 | 0.074548 | 0.142928 | 0.225889 | -1.60893 | -1.37765 | -1.23976 | -0.76248 | -0.40033 | -1.25877 | -3.99564 | -3.09787 | -0.88446 | -0.83469 | -1.43737 | -0.50758 |
| P59281 | P59281 | RHG39_M( | Arhgap39 | Rho GTPase-activating protein 39 | | | | 0.127483 | -0.55306 | -0.37199 | 0.01389 | 0.004286 | 0.044536 | 0.488022 | -0.7545 | -1.29356 | -0.29858 | -0.53537 | 1.798552 | 1.978868 | 1.260457 | 2.153276 | -1.34597 | 1.618141 | 1.85079 | 1.918059 | 2.085964 | 2.83676 |
| B2RQE8 | B2RQE8 | RHG42_M( | Arhgap42 | Rho GTPase-activating protein 42 | | | | NaN | 0 | NaN | 0.512624 | -1.37419 | -1.35878 | 0.083807 | 0.201057 | 0.2521 | -3.05277 | -2.35304 | -1.94783 | -3.88311 | -3.29587 | -1.92465 | -3.23824 | -3.55737 | -1.2472 | -1.83536 | -2.40389 | -3.21874 |
| Q5SSM3 | Q5SSM3 | RHG44_M( | Arhgap44 | Rho GTPase-activating protein 44 | | | | 0.450063 | 0.254654 | 1.194406 | 0.739119 | -0.38453 | -2.00859 | 0.078158 | -0.06271 | -0.23615 | -0.19166 | -0.15096 | 2.064543 | 2.105891 | 1.848124 | 2.102214 | -0.21374 | -0.6382 | 2.279424 | 2.660071 | 1.804692 | 2.271063 |
| P97393 | P97393 | RHG05_M( | Arhgap5 | Rho GTPase-activating protein 5 | | | + | 0.073721 | 0.229017 | 0.223533 | 0.101907 | 0.374908 | 0.302474 | 1.294214 | 1.20772 | 4.266335 | -2.85958 | -2.26597 | -1.46693 | -1.20927 | -1.92247 | -2.48091 | -3.77239 | -1.8112 | -2.94576 | -0.48025 | -3.36279 | -3.45603 |
| Q99PT1 | Q99PT1 | GDIR1_MC | Arhgdia | Rho GDP-dissociation inhibitor 1 | | | | 0.338978 | -0.27469 | -0.9117 | 0.179984 | 0.539197 | 0.510073 | 0.235565 | 0.476033 | 0.651949 | -0.9041 | -0.33396 | 1.32963 | -0.58678 | 0.36376 | 0.830538 | -0.44187 | -0.2468 | -0.6142 | 0.278658 | 0.812981 | -0.57075 |
| Q8C033 | A2AWP8;C | ARHGA_M( | Arhgef10 | Rho guanine nucleotide exchange factor 10 | | | | 0.026586 | -0.08157 | -0.08413 | 0.315521 | 0.461466 | 0.852825 | 0.014794 | 0.022552 | 0.047391 | -3.36666 | -2.33746 | -1.87018 | -2.20763 | -2.33421 | -2.43315 | -1.9487 | -3.59228 | -3.01449 | -1.98624 | -2.87953 | -1.93294 |
| Q68FM7 | Q68FM7 | Q68FM7_M | Arhgef11 | Rho guanine nucleotide exchange factor (GEF) 11 | | | | 0.578837 | 1.510568 | 1.538725 | 0.232698 | -0.12452 | -0.6447 | 0.622244 | -0.52752 | -1.66073 | -1.4901 | -0.2491 | -0.47415 | -0.11418 | -1.07094 | -0.46573 | -1.61943 | -0.3409 | -0.23973 | -0.09955 | -0.33738 | -0.14424 |
| O35143 | O35143 | ATIF1_MOI | Atp5if1 | ATPase inhibitor, mitochondrial | + | | | 1.691092 | 2.05248 | 6.899732 | 0.09058 | 0.106806 | 0.271079 | 0.181939 | 0.307167 | 0.515124 | 1.70855 | 1.479466 | 1.30903 | 0.893308 | 1.078686 | 0.140329 | -0.18394 | -0.73301 | 1.329075 | 0.65965 | -0.06569 | 0.670365 |
| Q80U35 | Q80U35 | ARHGH_M | Arhgef17 | Rho guanine nucleotide exchange factor 17 | | | | 0.675553 | 0.692937 | 1.815636 | 0.185803 | -0.05448 | -0.52509 | 0.515621 | -0.64702 | -1.36678 | -0.85104 | -0.78685 | 0.034604 | -0.08346 | -0.08832 | 0.851811 | -1.89218 | -1.13159 | -0.05527 | 0.115369 | 0.972748 | 1.084785 |
| P24549 | P24549 | AL1A1_MC | Aldh1a1 | Aldehyde dehydrogenase 1A1 | + | + | | 1.531715 | 2.051353 | 5.703153 | 1.174638 | 1.517813 | 3.669765 | 0.096268 | 0.189457 | 0.286895 | 8.001095 | 8.715431 | 7.760316 | 8.367359 | 5.910345 | 5.187982 | 6.264414 | 6.349407 | 6.265063 | 6.826986 | 4.806865 | 5.912549 |
| Q8BW86 | Q8BW86 | ARG33_M( | Arhgef33 | Rho guanine nucleotide exchange factor 33 | | | | 0.12642 | 0.760663 | 0.36913 | NaN | 0 | NaN | NaN | 0 | NaN | -4.02441 | 0.075503 | -3.82308 | -3.96455 | -3.57619 | -3.88477 | -2.94519 | -2.52504 | -3.83329 | -2.38532 | -3.43145 | -3.55868 |
| Q8K4I3 | Q8K4I3 | ARHG6_M( | Arhgef6 | Rho guanine nucleotide exchange factor 6 | | + | | 0.678638 | -1.71656 | -1.82479 | 1.024481 | -1.31741 | -3.0174 | 0.2841 | -0.18434 | -0.77405 | -1.49969 | -3.26722 | -3.61622 | -2.92038 | -0.46409 | -0.55069 | -0.98917 | -0.34462 | -1.68712 | -2.21467 | -0.55723 | -0.08888 |
| Q9ES28 | Q9ES28 | ARHG7_M( | Arhgef7 | Rho guanine nucleotide exchange factor 7 | | | | 0.207278 | 0.21004 | 0.58014 | 0.249053 | -0.16175 | -0.68599 | 0.09966 | -0.05875 | -0.29628 | -0.64313 | -0.59773 | 1.237031 | 1.176764 | 1.180441 | 1.214394 | -0.46913 | -1.19181 | 1.134789 | 1.602509 | 1.058608 | 1.453723 |
| Q3UTH8 | Q3UTH8 | ARHG9_M( | Arhgef9 | Rho guanine nucleotide exchange factor 9 | | | | 0.871218 | 0.718633 | 2.443353 | 0.798484 | 0.659827 | 2.197905 | 0.580775 | -0.80627 | -1.5441 | -0.75324 | -1.21176 | 1.404643 | 1.91646 | 0.111262 | 0.382532 | -1.51689 | -1.88537 | 0.843769 | 1.15768 | 0.548928 | 1.557404 |
| P63082 | P63082 | VATL_MOI | Atp6v0c | V-type proton ATPase 16 kDa proteolipid subunit c | + | | | 1.504305 | 2.00863 | 5.517769 | 0.290189 | -0.86087 | -0.78932 | 0.173786 | 0.377906 | 0.494024 | 1.411849 | 2.105907 | 2.003948 | -0.05444 | 2.151171 | 3.258621 | -0.3597 | -0.1398 | 1.474684 | 2.196568 | 2.854762 | 1.799218 |
| P61211 | P61211 | ARL1_MOI | Arl1 | ADP-ribosylation factor-like protein 1 | | | | 0.061647 | -0.04563 | -0.18881 | 0.961479 | 0.343026 | 2.771304 | 0.35143 | 0.069884 | 0.943018 | 0.738126 | 0.513513 | 0.429774 | 0.333319 | 0.4349 | 0.469837 | 0.885441 | 0.457458 | 0.152516 | -0.07548 | 0.310466 | 0.454503 |
| Q8BGR6 | Q8BGR6 | ARL15_M( | Arl15 | ADP-ribosylation factor-like protein 15 | | | | 0.218034 | -0.62651 | -0.60753 | 0.512143 | 0.504347 | 1.357494 | 0.054151 | -0.04572 | -0.16694 | -3.31379 | -2.8763 | 0.068952 | 0.072472 | -0.11374 | -0.53619 | -1.46075 | -3.47632 | -0.80516 | -0.06211 | -0.1049 | -0.45359 |
| Q9WUL7 | Q9WUL7 | ARL3_MOI | Arl3 | ADP-ribosylation factor-like protein 3 | | | | 0.510677 | -0.47754 | -1.35358 | 0.37424 | -0.52013 | -1.00056 | 0.333399 | -0.54957 | -0.89769 | -0.88657 | -0.37409 | -0.06141 | -1.09946 | -1.13073 | -0.15902 | 0.0897 | -0.39529 | -0.03121 | -0.08941 | 0.277178 | -0.46778 |
| Q80ZU0 | Q80ZU0;Q | ARL5A_M( | Arl5a | ADP-ribosylation factor-like protein 5A | | | | 0.514017 | -0.59091 | -1.36249 | 0.545814 | -0.1478 | -1.44812 | 0.135988 | 0.084535 | 0.39473 | -0.57204 | -0.16074 | -0.54434 | -0.66794 | -0.35304 | -0.64426 | 0.606349 | -0.15732 | -0.53956 | -0.37711 | -0.42615 | -0.74023 |
| Q9JM93 | Q9JM93 | AR6P4_M( | Arl6ip4 | ADP-ribosylation factor-like protein 6-interacting protein 4 | + | | | 1.011361 | 0.530326 | 2.964905 | 0.183011 | 0.192858 | 0.51789 | 0.820091 | 0.570129 | 2.269195 | 1.36038 | 1.69655 | 1.176371 | 1.746863 | 1.297258 | 1.582005 | 1.059303 | 0.936975 | 1.508153 | 1.029366 | 0.662488 | 1.076517 |
| Q8R5J9 | Q8R5J9 | PRAF3_M( | Arl6ip5 | PRA1 family protein 3 | | | | 0.149681 | 0.172936 | 0.431014 | 0.043308 | 0.101543 | 0.13483 | 0.011644 | 0.020729 | 0.037425 | -1.08825 | -1.15511 | -0.37002 | -1.82728 | -1.56663 | -1.41032 | -1.69445 | -0.89478 | -1.39067 | -1.00972 | -0.96087 | -2.05754 |
| Q8VEH3 | Q8VEH3 | ARL8A_M( | Arl8a | ADP-ribosylation factor-like protein 8A | | | + | 0.16317 | -0.14926 | -0.4664 | 0.228974 | -0.49097 | -0.63528 | 1.111918 | 0.437258 | 3.385205 | -3.55402 | -3.5895 | -2.76516 | -4.30048 | -2.38636 | -2.63154 | -3.74204 | -3.10295 | -2.95243 | -3.13126 | -2.98689 | -2.90553 |
| Q9CQW2 | Q8VEH3;C | ARL8B_M( | Arl8b | ADP-ribosylation factor-like protein 8B | | | | 0.054277 | 0.064853 | 0.167306 | 0.435833 | 0.298609 | 1.157644 | 0.185463 | -0.03316 | -0.52421 | 0.389904 | 1.032735 | 1.041832 | 0.537688 | 0.63117 | 0.718453 | 0.863145 | 0.429789 | 0.545885 | 0.436417 | 0.75377 | 0.662177 |
| Q9D7A8 | Q9D7A8 | ARMC1_M | Armc1 | Armadillo repeat-containing protein 1 | | | | 0.875189 | -0.32366 | -2.45722 | 0.250996 | -0.34795 | -0.69088 | 0.380405 | 0.117294 | 1.016157 | -0.36673 | -0.11613 | -0.48194 | -0.75861 | 0.499579 | 0.269511 | 0.122841 | 0.041619 | 0.211938 | -0.75658 | 0.276784 | 0.257719 |
| Q9D0L7 | Q9D0L7 | ARM10_M( | Armc10 | Armadillo repeat-containing protein 10 | | | | NaN | 0 | NaN | NaN | 0 | NaN | 0.163743 | 0.680553 | 0.467895 | -2.88214 | -3.32749 | -3.05219 | -3.69569 | -3.68974 | -3.9197 | -3.53768 | -3.51977 | -3.15433 | -2.71726 | -5.93522 | -3.03533 |
| Q7TNV0 | Q7TNV0 | DEK_MOU | Dek | Protein DEK | + | | | 1.31697 | 1.993538 | 4.388627 | 0.106074 | 0.058551 | 0.313925 | 0.239711 | 0.300343 | 0.662424 | 1.248913 | 2.039387 | 1.365103 | 1.721568 | 1.509009 | 0.980138 | -0.57329 | -0.12549 | 1.429833 | 1.539737 | 0.57593 | 1.312531 |
| Q9DBR3 | Q9DBR3 | ARMC8_M | Armc8 | Armadillo repeat-containing protein 8 | | | | 0.321417 | 0.23248 | 0.867613 | 0.960327 | -0.26948 | -2.76694 | 0.098018 | -0.05296 | -0.29174 | -0.53715 | -0.75997 | -0.05082 | 0.107747 | -0.03645 | -0.38527 | -1.12473 | -0.63734 | 0.354512 | 0.241378 | -0.20828 | -0.10751 |
| Q9R0Q6 | Q9R0Q6 | ARC1A_M( | Arpc1a | Actin-related protein 2/3 complex subunit 1A | | + | | 0.069457 | -0.05348 | -0.21134 | 1.190864 | -0.63605 | -3.74642 | 0.317745 | -0.21498 | -0.8584 | 1.391319 | 1.826303 | 2.286608 | 1.95211 | 2.721143 | 3.160388 | 1.791642 | 1.532938 | 2.784577 | 2.726232 | 3.2761 | 3.035386 |
| Q9WV32 | Q9WV32 | ARC1B_M( | Arpc1b | Actin-related protein 2/3 complex subunit 1B | | | | 0.82563 | 1.25752 | 2.287686 | 0.161136 | 0.285081 | 0.461084 | 0.397269 | 0.413152 | 1.058955 | -2.84977 | -1.75061 | -2.61265 | -3.68822 | -1.93295 | -1.1808 | -3.56871 | -3.5467 | -3.74057 | -3.13045 | -1.86617 | -2.07388 |
| Q9CVB6 | Q9CVB6 | ARPC2_M( | Arpc2 | Actin-related protein 2/3 complex subunit 2 | | | | 0.660706 | -0.20268 | -1.7719 | 0.5111 | -0.404 | -1.35471 | 0.107545 | -0.06211 | -0.31796 | 0.894876 | 1.066714 | 2.12275 | 1.613044 | 2.411747 | 2.712036 | 1.258979 | 1.107962 | 2.11703 | 2.42676 | 2.748951 | 2.499048 |
| Q9JM76 | Q9JM76 | ARPC3_M( | Arpc3 | Actin-related protein 2/3 complex subunit 3 | | | | 0.499266 | 0.33479 | 1.323265 | 0.477556 | -0.2254 | -1.26605 | 0.368016 | 0.288395 | 0.984831 | 1.948853 | 2.358922 | 2.214489 | 1.861506 | 3.246194 | 3.633857 | 1.670871 | 1.967325 | 2.286775 | 2.240018 | 2.932124 | 3.371137 |
| P59999 | P59999 | ARPC4_M( | Arpc4 | Actin-related protein 2/3 complex subunit 4 | + | | | 1.195278 | -0.59342 | -3.76749 | 0.979621 | -0.69279 | -2.84064 | 0.152517 | -0.13636 | -0.43848 | 1.558395 | 1.831995 | 2.577504 | 2.109976 | 3.154509 | 3.439518 | 2.366686 | 2.210541 | 2.967 | 3.106062 | 3.709793 | 3.15696 |
| Q9CPW4 | Q9CPW4 | ARPC5_M( | Arpc5 | Actin-related protein 2/3 complex subunit 5 | | | | 0.181386 | -0.19325 | -0.5137 | 0.262258 | -0.29256 | -0.71922 | 0.002257 | 0.003086 | 0.007331 | -0.22848 | 0.198764 | 0.951061 | 0.235717 | 1.717385 | 2.147822 | -0.13127 | 0.488049 | 1.079701 | 0.692199 | 2.291257 | 1.567779 |
| Q9D898 | Q9D898 | ARP5L_M( | Arpc5l | Actin-related protein 2/3 complex subunit 5-like protein | | + | | 0.048123 | 0.033557 | 0.149159 | 1.038376 | -0.35312 | -3.07373 | 0.843313 | -0.51407 | -2.34732 | 0.790191 | 1.194353 | 1.761823 | 1.750543 | 1.55361 | 1.957004 | 1.057589 | 0.859842 | 1.994561 | 2.224052 | 2.354699 | 2.184052 |
| Q9DCB4 | Q9DCB4 | ARP21_M( | Arpp21 | cAMP-regulated phosphoprotein 21 | | | + | 0.426212 | 1.348323 | 1.132902 | 0.334688 | 0.242555 | 0.900922 | 1.010248 | -0.5754 | -2.96048 | -0.49465 | -1.65396 | 1.423598 | 1.858533 | 0.582329 | 0.835966 | -1.5122 | -3.78495 | 1.239788 | 1.557232 | 1.13726 | 1.431829 |
| C0HKE1;C | C0HKE3;Q | H2A1B_M( | H2ac11;H2ac12;H2ac13;H2ac15;H2ac20;H2a | Histone H2A type 1-B | + | | | 1.810785 | 1.965952 | 7.948974 | 0.23209 | 0.776528 | 0.643162 | 0.406903 | 0.376034 | 1.083491 | 5.975411 | 5.647531 | 3.89649 | 6.100325 | 4.663998 | 4.674016 | 4.030699 | 3.660339 | 4.715329 | 3.72843 | 3.945951 | 4.639995 |
| Q6PDH0 | Q6PDH0 | PHLB1_M( | Phldb1 | Pleckstrin homology-like domain family B member 1 | + | | + | 1.482001 | 1.948047 | 5.370976 | 0.127194 | -0.06332 | -0.37121 | 1.457616 | 0.319108 | 5.21453 | -0.85716 | -1.5672 | -1.49833 | -1.16031 | -1.35127 | -1.4454 | -3.08599 | -3.23446 | -1.24301 | -1.28899 | -1.75655 | -1.67833 |
| P98203 | P98203 | ARVC_MO | Arvcf | Splicing regulator ARVCF | | | + | NaN | 0 | NaN | 0.202694 | 0.196352 | 0.568433 | 1.600953 | -1.41837 | -6.19714 | -2.72886 | -3.72578 | -2.76879 | -2.30638 | -3.12885 | -3.54577 | -3.40653 | -3.59577 | -2.99058 | -2.4773 | -2.01342 | -1.82444 |
| Q9QWY8 | Q9QWY8 | ASAP1_M( | Asap1 | Arf-GAP with SH3 domain, ANK repeat and PH domain-containing protein 1 | | | + | 0.042171 | -0.04921 | -0.13143 | 0.919937 | -0.88295 | -2.61695 | 2.39113 | -0.50119 | -15.6401 | -0.53621 | -0.80584 | 0.817616 | 0.14836 | 0.203906 | 0.249907 | -0.27251 | -0.97112 | 1.322794 | 1.409089 | 0.750409 | 0.705783 |
| Q7SIG6 | Q7SIG6 | ASAP2_M( | Asap2 | Arf-GAP with SH3 domain, ANK repeat and PH domain-containing protein 2 | | | + | 0.451492 | 0.896092 | 1.19811 | 0.321386 | -0.27747 | -0.86754 | 1.827731 | -0.53624 | -8.10924 | -0.76953 | -0.54332 | -0.51473 | -0.90244 | -0.78239 | -0.80309 | -0.8132 | -0.29184 | -0.68551 | -0.17673 | -0.32181 | -0.19118 |
| Q61024 | Q61024 | ASNS_MOI | Asns | Asparagine synthetase [glutamine-hydrolyzing] | | | | 0.45315 | 0.369688 | 1.20241 | 0.46433 | -0.46874 | -1.23148 | 0.189259 | 0.274728 | 0.533987 | -1.25703 | -0.8893 | -1.59188 | -1.445 | -0.73653 | -0.48646 | -1.68927 | -1.19643 | -0.67622 | -1.42318 | -0.38717 | -1.38528 |
| Q8R3P0 | Q8R3P0 | ACY2_MOI | Aspa | Aspartoacylase | | | | 0.33348 | -0.48668 | -0.89789 | 0.201823 | -0.18717 | -0.56621 | 0.050404 | 0.062365 | 0.155905 | 0.885108 | -0.19873 | -1.53495 | -1.56976 | -1.6096 | -0.82093 | 0.81918 | 0.840553 | -1.69529 | -1.03508 | -1.21041 | -1.34485 |
| Q80VP9 | Q80VP9 | ASPH2_M( | Asphd2 | Aspartate beta-hydroxylase domain-containing protein 2 | + | | | 1.231273 | 1.570569 | 3.943019 | 0.339835 | -0.09604 | -0.91385 | 0.472061 | -0.38378 | -1.25166 | -1.8316 | -1.64728 | -0.85996 | -0.65628 | -0.33674 | 0.039295 | -2.9225 | -3.69752 | -0.68803 | -0.63613 | -0.00715 | 0.477267 |
| Q8C0M9 | Q8C0M9 | ASGL1_M( | Asrgl1 | Isoaspartyl peptidase/L-asparaginase | | | | 0.881055 | 0.314167 | 2.477785 | 0.243668 | -0.14635 | -0.67241 | 0.632512 | -0.24071 | -1.69012 | 2.704556 | 2.917344 | 2.687782 | 3.122349 | 2.612232 | 2.896986 | 2.427813 | 2.565753 | 3.038797 | 3.064033 | 2.991638 | 2.999007 |
| Q8R4H2 | Q8R4H2 | ARHGC_M | Arhgef12 | Rho guanine nucleotide exchange factor 12 | + | | | 1.713552 | 1.933725 | 7.086054 | 0.48531 | -0.29228 | -1.28642 | 0.142767 | -0.07279 | -0.41274 | -1.65392 | -1.28639 | -1.50897 | -1.22537 | -1.56229 | -1.48413 | -3.60563 | -3.20214 | -1.25242 | -0.89736 | -1.62239 | -1.27846 |
| Q61263 | Q61263 | SOAT1_M( | Soat1 | Sterol O-acyltransferase 1 | + | | + | 3.718064 | 1.888729 | 72.27189 | 0.699815 | 0.735236 | 1.888166 | 1.090108 | -0.13159 | -3.29043 | 1.243057 | 1.217002 | 1.273191 | 1.583413 | 1.216084 | 1.13613 | -0.68135 | -0.63604 | 0.335902 | 1.050231 | 1.308714 | 1.306671 |
| Q9D5T0 | Q9D5T0 | ATAD1_M( | Atad1 | Outer mitochondrial transmembrane helix translocase | + | | | 1.14088 | 0.187485 | 3.514341 | 0.161713 | -0.05576 | -0.46259 | 0.341544 | 0.405779 | 0.918149 | 0.480782 | 0.54775 | 0.12193 | -0.10796 | 1.073663 | 0.19069 | 0.368313 | 0.285249 | 0.02643 | 0.099065 | 0.246709 | 0.206085 |
| Q925I1 | Q925I1 | ATAD3_M( | Atad3 | ATPase family AAA domain-containing protein 3 | | + | | 0.290825 | 0.234095 | 0.790914 | 3.068655 | 0.429668 | 34.20184 | 0.490675 | 0.221373 | 1.300552 | 2.294997 | 2.12855 | 2.95928 | 2.936646 | 2.815739 | 2.562489 | 2.261718 | 1.693638 | 2.523749 | 2.512841 | 2.35399 | 2.581491 |
| Q8K341 | Q8K341 | ATAT_MOI | Atat1 | Alpha-tubulin N-acetyltransferase 1 | | | | 0.774647 | 0.627484 | 2.120764 | 0.892702 | -0.32286 | -2.51895 | 0.477761 | -0.25801 | -1.26659 | -0.48892 | -1.03806 | 0.110624 | 0.365686 | -0.24065 | -0.28353 | -1.50121 | -1.28073 | 0.548224 | 0.573798 | -0.20665 | 0.198486 |
| Q9Z2A5 | Q9Z2A5 | ATE1_MOI | Ate1 | Arginyl-tRNA--protein transferase 1 | | + | | 0.899319 | 1.527487 | 2.542534 | 1.373042 | 1.335154 | 4.702966 | 0.543896 | 0.829281 | 1.442908 | -2.85669 | -1.72572 | -1.86008 | -2.06312 | -2.01794 | -2.38808 | -4.02156 | -3.61583 | -3.03163 | -3.56188 | -2.48818 | -3.57641 |
| P16951 | P16951 | ATF2_MOI | Atf2 | Cyclic AMP-dependent transcription factor ATF-2 | | | | 0.04412 | 0.13611 | 0.137255 | 0.022179 | 0.044539 | 0.070496 | 0.692685 | -0.46641 | -1.86671 | -3.69469 | -1.71149 | -3.04777 | -1.80215 | -2.57192 | -3.04793 | -2.85007 | -2.82833 | -2.36331 | -2.5757 | -2.41956 | -2.26746 |
| Q9D8Z6 | Q9D8Z6 | ATGA1_M( | Atg101 | Autophagy-related protein 101 | | | | 0.371845 | 0.860194 | 0.994504 | 0.365653 | 0.650333 | 0.978865 | 0.649201 | -0.93999 | -1.73833 | -1.05126 | -2.71165 | 0.00713 | 0.467074 | -1.6384 | -1.8031 | -2.49893 | -2.98436 | -1.03653 | 0.210072 | -1.3152 | -0.24632 |
| Q91YI1 | Q91YI1 | ATG13_M( | Atg13 | Autophagy-related protein 13 | | + | | 0.461557 | 0.949443 | 1.224255 | 1.133314 | 0.629662 | 3.480238 | 0.024047 | 0.043288 | 0.076288 | -1.21355 | -1.12953 | 0.013997 | 0.343822 | -1.1591 | -1.04747 | -1.34659 | -2.89537 | -0.52517 | -0.37634 | -1.71125 | -0.5819 |
| Q8CDJ3 | Q8CDJ3 | BAKOR_M( | Atg14 | Beclin 1-associated autophagy-related key regulator | + | | | 1.145328 | 0.941995 | 3.534515 | 0.804669 | 0.860953 | 2.218175 | 0.006965 | -0.01872 | -0.0225 | 0.070081 | -0.39341 | 1.30827 | 2.066167 | -0.95429 | -0.40198 | -0.97204 | -1.23528 | 0.742318 | 0.910214 | -1.44418 | 0.125359 |
| Q6P4T0 | Q6P4T0 | ATG2A_M( | Atg2a | Autophagy-related protein 2 homolog A | | | | NaN | 0 | NaN | 0.429957 | -0.15581 | -1.14252 | 0.281572 | -1.11567 | -0.76771 | -3.57643 | -3.31723 | -1.47983 | -1.22157 | -3.87642 | -1.01535 | -3.5449 | -3.07473 | -1.23875 | -1.15103 | -1.58612 | -1.07433 |
| Q6PFE7 | Q6PFE7 | TEFF1_MC | Tmeff1 | Tomoregulin-1 | + | | | 2.737873 | 1.857996 | 23.35287 | 0.870232 | 0.597805 | 2.439918 | 0.33199 | 0.167266 | 0.894147 | -1.21985 | -1.18059 | -1.10292 | -0.62788 | -1.1979 | -1.25883 | -3.13532 | -2.98111 | -1.4031 | -1.52332 | -1.5802 | -1.21106 |
| Q9CPX6 | Q9CPX6 | ATG3_MOI | Atg3 | Ubiquitin-like-conjugating enzyme ATG3 | | | | 0.412816 | -0.56829 | -1.09859 | 0.293371 | 0.671604 | 0.797296 | 0.03518 | 0.034554 | 0.110377 | -4.01793 | -3.00023 | -2.04778 | -2.04335 | -2.33445 | -1.80199 | -3.03386 | -2.84772 | -3.55952 | -1.87482 | -1.93808 | -2.26746 |
| Q8BGE6 | Q8BGE6 | ATG4B_M( | Atg4b | Cysteine protease ATG4B | | | + | 0.2949 | 1.364424 | 0.801129 | 0.001871 | -0.00156 | -0.00608 | 1.527701 | 0.45322 | 5.675651 | -0.92953 | -1.25976 | -1.53622 | -1.07353 | -0.7789 | -0.79592 | -4.15417 | -0.76397 | -1.19428 | -1.41236 | -1.16124 | -1.32003 |
| Q99J83 | Q99J83 | ATG5_MOI | Atg5 | Autophagy protein 5 | | | | 0.06784 | 0.218178 | 0.206695 | 0.593315 | -1.5384 | -1.57904 | 0.05675 | -0.09802 | -0.17455 | -4.33621 | -2.2754 | -2.51526 | -4.23157 | -2.13583 | -1.54387 | -3.29493 | -3.75303 | -1.37377 | -2.29627 | -1.26462 | -2.21904 |
| Q9D906 | Q9D906 | ATG7_MOI | Atg7 | Ubiquitin-like modifier-activating enzyme ATG7 | | | + | 0.576845 | 1.290767 | 1.533206 | 0.404832 | -0.44959 | -1.07821 | 1.448988 | -0.39731 | -5.16017 | -0.65011 | -0.54203 | -0.57778 | -0.52501 | -0.89688 | -0.95405 | -1.0467 | -2.72698 | -0.51795 | 0.314334 | -0.59965 | -0.45667 |
| Q68FE2 | Q68FE2 | ATG9A_M( | Atg9a | Autophagy-related protein 9A | | | | 0.406842 | 0.725534 | 1.083337 | 0.293386 | 0.354258 | 0.797334 | 0.386847 | 0.297252 | 1.032484 | -1.16631 | -1.96217 | -1.17011 | -1.35415 | -1.42755 | -1.26175 | -1.75109 | -2.82846 | -2.05106 | -1.18172 | -1.91761 | -1.36619 |
| Q9CWJ9 | Q9CWJ9 | PUR9_MOI | Atic | Bifunctional purine biosynthesis protein ATIC | | | | 0.033763 | 0.260329 | 0.106075 | 0.375384 | -1.39413 | -1.00345 | 0.270381 | -0.29077 | -0.73962 | 0.12139 | -3.70597 | -3.63502 | -0.95533 | -1.40045 | -0.67647 | -3.58915 | -0.51609 | -0.53351 | -1.26858 | -0.59433 | -0.90105 |
| Q8BH66 | Q8BH66 | ATLA1_MC | Atl1 | Atlastin-1 | | | | 0.806595 | -0.42474 | -2.22451 | 0.047247 | 0.017752 | 0.14656 | 0.71885 | 0.419473 | 1.946024 | 1.176174 | 0.848373 | 1.678225 | 1.442954 | 1.814048 | 1.524314 | 1.534965 | 1.339068 | 1.513962 | 1.571712 | 1.409324 | 1.090093 |
| Q6PA06 | Q6PA06 | ATLA2_MC | Atl2 | Atlastin-2 | | | | 0.055127 | -0.04954 | -0.1698 | 0.031457 | -0.06432 | -0.09906 | 0.54935 | 0.676036 | 1.457733 | -3.18224 | -3.136 | -2.96476 | -3.31228 | -2.80555 | -3.09702 | -3.40041 | -2.81877 | -3.69988 | -2.44851 | -3.18706 | -4.06759 |
| O08997 | O08997 | ATOX1_M( | Atox1 | Copper transport protein ATOX1 | | | | 0.108545 | 0.213829 | 0.320692 | 0.240594 | 0.164964 | 0.664653 | 0.846539 | 0.283023 | 2.358303 | 0.623091 | 1.148214 | 0.727801 | 0.316192 | 0.97498 | 0.737142 | 0.05892 | 1.284727 | 0.495761 | 0.218302 | 0.589191 | 0.556886 |
| Q9EPE9 | Q9EPE9 | AT131_M( | Atp13a1 | Endoplasmic reticulum transmembrane helix translocase | | | | 0.515324 | -0.49009 | -1.36599 | 0.359799 | -0.11468 | -0.9641 | 0.253039 | 0.203583 | 0.696025 | -2.04982 | -1.33294 | -1.40423 | -1.47524 | -0.59754 | -1.09077 | -1.21699 | -1.18558 | -1.21153 | -1.43859 | -0.89047 | -1.205 |
| Q8VDN2 | Q8VDN2 | AT1A1_M( | Atp1a1 | Sodium/potassium-transporting ATPase subunit alpha-1 | | | | 0.030511 | 0.044139 | 0.096167 | 0.784293 | -0.98944 | -2.15179 | 0.660803 | 0.381395 | 1.77218 | 2.968963 | 3.736293 | 4.068868 | 3.177339 | 4.29167 | 3.978356 | 3.560415 | 3.056563 | 4.499715 | 4.725379 | 3.901181 | 3.606054 |
| Q6PIE5 | Q6PIE5 | AT1A2_M( | Atp1a2 | Sodium/potassium-transporting ATPase subunit alpha-2 | | | | 0.106038 | 0.093773 | 0.313826 | 0.35841 | 0.220436 | 0.960599 | 0.667337 | 0.61391 | 1.791372 | 4.856386 | 5.072684 | 4.255978 | 3.837097 | 4.738193 | 4.067981 | 5.149309 | 4.592214 | 3.73232 | 3.919882 | 3.860942 | 3.717411 |
| Q6PIC6 | Q6PIC6 | AT1A3_M( | Atp1a3 | Sodium/potassium-transporting ATPase subunit alpha-3 | | | | 0.071776 | -0.03541 | -0.21798 | 0.275156 | -0.28227 | -0.75161 | 0.874089 | 0.510616 | 2.453369 | 6.508166 | 6.527252 | 6.280797 | 5.533092 | 7.082117 | 6.832794 | 6.715288 | 6.390951 | 6.153483 | 6.224952 | 6.613503 | 6.280175 |
| P14094 | P14094 | AT1B1_M( | Atp1b1 | Sodium/potassium-transporting ATPase subunit beta-1 | | | | 0.324125 | -0.20439 | -0.87441 | 0.30143 | -0.32857 | -0.8175 | 0.579193 | 0.365793 | 1.539713 | 4.827173 | 4.879226 | 4.846428 | 4.043407 | 5.474115 | 5.285289 | 5.289883 | 4.825296 | 4.755318 | 4.791658 | 5.231915 | 4.795902 |
| P14231 | P14231 | AT1B2_M( | Atp1b2 | Sodium/potassium-transporting ATPase subunit beta-2 | | | | 0.129724 | 0.14838 | 0.377995 | 0.02933 | 0.025295 | 0.092553 | 0.91621 | 0.518422 | 2.603389 | 3.356783 | 3.547672 | 2.813615 | 2.267021 | 2.812671 | 2.414404 | 3.684611 | 2.923085 | 2.512664 | 2.517381 | 2.094686 | 2.095545 |
| P97370 | P97370 | AT1B3_M( | Atp1b3 | Sodium/potassium-transporting ATPase subunit beta-3 | | | | 0.709185 | 0.25947 | 1.916539 | 0.177324 | 0.284352 | 0.503191 | 0.061706 | 0.05342 | 0.18898 | 0.291003 | 0.216721 | -0.4323 | -0.61647 | -0.34019 | -0.65971 | 0.124582 | -0.1358 | -1.36628 | -0.25119 | -0.32017 | -0.78657 |
| O55143 | O55143 | AT2A2_M( | Atp2a2 | Sarcoplasmic/endoplasmic reticulum calcium ATPase 2 | | | | 0.778377 | 0.541886 | 2.132732 | 0.339224 | 0.10577 | 0.912319 | 0.297957 | 0.284976 | 0.808794 | 2.586378 | 2.862966 | 3.095018 | 3.101357 | 3.675431 | 2.99072 | 2.395933 | 1.969639 | 3.108309 | 2.876524 | 2.964788 | 3.131412 |
| Q64518 | Q64518 | AT2A3_M( | Atp2a3 | Sarcoplasmic/endoplasmic reticulum calcium ATPase 3 | | | | 0.361414 | 1.10498 | 0.968171 | NaN | 0 | NaN | NaN | 0 | NaN | -3.59226 | -1.34225 | -3.38089 | -3.8004 | -2.54844 | -3.84098 | -3.38002 | -3.76445 | -3.4155 | -2.30029 | -3.62038 | -3.8104 |
| G5E829 | G5E829;Q | AT2B1_M( | Atp2b1 | Plasma membrane calcium-transporting ATPase 1 | | | | 0.224663 | 0.126793 | 0.624353 | 0.661042 | -0.52505 | -1.77288 | 0.470384 | 0.201413 | 1.247278 | 1.585107 | 1.955606 | 3.104746 | 2.54376 | 3.878767 | 3.600066 | 1.726772 | 1.560356 | 3.254269 | 3.444328 | 3.619599 | 3.456407 |
| Q9R0K7 | Q9R0K7 | AT2B2_M( | Atp2b2 | Plasma membrane calcium-transporting ATPase 2 | | | | 0.151448 | -0.18758 | -0.43567 | 0.983836 | -0.84604 | -2.85692 | 0.437821 | 0.378806 | 1.162768 | 1.508482 | 2.329915 | 2.129259 | 1.537044 | 2.50382 | 1.965259 | 2.235929 | 1.977619 | 2.683454 | 2.674937 | 2.039092 | 1.672375 |
| Q0VF55 | Q0VF55 | Q0VF55_M | Atp2b3 | Calcium-transporting ATPase | | | | 0.474017 | 0.29138 | 1.256781 | 0.539847 | -0.38454 | -1.43193 | 0.64346 | 0.443058 | 1.721679 | 0.700675 | 0.99362 | 0.652866 | 0.226802 | 2.015421 | 1.508637 | 0.735485 | 0.37605 | 0.660867 | 0.987881 | 1.363878 | 1.274064 |
| Q6Q477 | Q6Q477 | AT2B4_M( | Atp2b4 | Plasma membrane calcium-transporting ATPase 4 | | | | 0.132484 | -0.16737 | -0.38538 | 0.469923 | -0.38819 | -1.24607 | 0.325034 | 0.312152 | 0.876689 | -0.0868 | -0.93047 | 1.33124 | 1.040907 | 1.032843 | 0.331329 | -0.23799 | -0.44454 | 1.298623 | 1.849905 | 0.431142 | 0.308726 |
| Q80XR2 | Q80XR2 | AT2C1_M( | Atp2c1 | Calcium-transporting ATPase type 2C member 1 | | | | 0.231522 | -0.22966 | -0.64173 | 0.415543 | 0.612197 | 1.105561 | 0.197612 | 0.292737 | 0.55543 | -3.35788 | -2.64244 | -3.06763 | -2.79181 | -1.85417 | -2.77509 | -2.75996 | -2.78105 | -3.00562 | -4.07821 | -2.86379 | -2.35095 |
| Q64436 | Q64436 | ATP4A_M( | Atp4a | Potassium-transporting ATPase alpha chain 1 | | | + | 0.170986 | 0.242317 | 0.486755 | 0.211336 | -0.19704 | -0.59049 | 1.000542 | 0.794627 | 2.922116 | 3.097875 | 2.776381 | 2.293276 | 1.746929 | 3.371863 | 3.089018 | 3.165966 | 2.223655 | 2.408764 | 2.025511 | 2.668082 | 2.203546 |
| Q03265 | Q03265 | ATPA_MOI | Atp5f1a | ATP synthase subunit alpha, mitochondrial | + | | | 1.229565 | 0.450116 | 3.934541 | 0.130621 | -0.09104 | -0.3804 | 0.895051 | 0.398679 | 2.527307 | 6.585764 | 6.695619 | 6.653471 | 6.320515 | 7.03648 | 6.780458 | 6.290928 | 6.090223 | 6.406081 | 6.749994 | 6.417607 | 6.601974 |
| Q9R087 | Q9R087 | GPC6_MO | Gpc6 | Glypican-6 | + | | | 2.930504 | 1.848535 | 29.16549 | 0.153984 | -0.04923 | -0.44234 | 0.410309 | 0.383265 | 1.092186 | 1.747151 | 1.857197 | 1.767341 | 1.989424 | 2.067121 | 1.374329 | -0.0149 | -0.07782 | 1.920018 | 1.935213 | 1.393599 | 1.281322 |
| Q91VR2 | Q91VR2 | ATPG_MOI | Atp5f1c | ATP synthase subunit gamma, mitochondrial | | | + | 0.138852 | 0.063328 | 0.402353 | 0.148562 | 0.061737 | 0.428063 | 1.116349 | 0.465907 | 3.404715 | 4.809698 | 4.986798 | 4.922525 | 4.635347 | 5.445917 | 5.173166 | 4.965042 | 4.704797 | 4.73073 | 4.703668 | 4.832349 | 4.854919 |
| P61809 | P61809 | CD5R1_M( | Cdk5r1 | Cyclin-dependent kinase 5 activator 1 | + | | | 1.625017 | 1.838272 | 6.377878 | 0.98933 | 0.629838 | 2.87825 | 0.09324 | -0.16473 | -0.27849 | 2.167207 | 2.507095 | 3.266102 | 3.690668 | 2.430894 | 1.841151 | 0.266084 | 0.731674 | 2.795433 | 2.901661 | 1.787983 | 2.813516 |
| P56382 | P56382 | ATP5E_M( | Atp5f1e | ATP synthase subunit epsilon, mitochondrial | | | | 0.532766 | 0.254144 | 1.4128 | 0.237801 | -0.79816 | -0.6576 | 0.242107 | 0.371511 | 0.66847 | 2.655691 | 2.684425 | 0.06721 | -0.94414 | 1.032139 | 0.508311 | 2.236601 | 2.595226 | 1.463087 | -0.7437 | 0.888891 | -0.09146 |
| Q8K274 | Q8K274 | KT3K_MOI | Fn3krp | Ketosamine-3-kinase | + | | | 1.648814 | 1.812644 | 6.561421 | 0.786296 | 0.999522 | 2.158267 | 0.28329 | 0.514987 | 0.772019 | 8.003803 | 8.519859 | 7.594913 | 8.19367 | 7.877651 | 7.05829 | 6.547878 | 6.350496 | 6.541434 | 7.248106 | 6.426545 | 7.479423 |
| P56383;P5 | P56383;Q | AT5G1_MC | Atp5mc1;Atp5mc2;Atp5mc3 | ATP synthase F(0) complex subunit C2, mitochondrial | | + | | 0.454888 | -0.46726 | -1.20692 | 1.40493 | -3.10043 | -4.89036 | 0.129674 | -0.24793 | -0.37786 | 1.684997 | 1.850566 | -2.72609 | -3.80784 | -0.24016 | 0.346653 | 1.856845 | 2.613239 | 0.16421 | -0.49728 | 0.888073 | -0.28571 |
| Q06185 | Q06185 | ATP5I_MO | Atp5me | ATP synthase subunit e, mitochondrial | | | | 0.344679 | 0.431927 | 0.926031 | 0.177252 | -0.21463 | -0.503 | 0.047045 | 0.034223 | 0.145961 | 2.855105 | 3.029513 | 2.971634 | 2.12674 | 2.834384 | 2.699848 | 2.052178 | 2.968585 | 2.823869 | 2.703763 | 2.957504 | 2.508281 |
| P56135 | P56135 | ATPK_MOI | Atp5mf | ATP synthase subunit f, mitochondrial | | | | 0.239095 | 0.260679 | 0.660867 | 0.078323 | 0.118967 | 0.236615 | 0.134991 | -0.13106 | -0.39207 | 2.888762 | 3.212313 | 3.395819 | 2.559647 | 3.071736 | 3.062845 | 3.149608 | 2.43011 | 2.579486 | 3.138046 | 3.532606 | 2.864101 |
| Q9CPQ8 | Q9CPQ8 | ATP5L_MC | Atp5mg | ATP synthase subunit g, mitochondrial | | | | 0.454189 | 0.298172 | 1.205106 | 0.104668 | -0.07712 | -0.31007 | 0.141362 | 0.075919 | 0.409018 | 3.207347 | 3.252812 | 3.083897 | 2.595631 | 2.981436 | 2.705307 | 2.685531 | 3.178285 | 2.869418 | 2.964341 | 2.891512 | 2.643393 |
| Q78IK2 | Q78IK2 | ATPMK_M( | Atp5mk | ATP synthase membrane subunit K, mitochondrial | | | | 0.325781 | 0.462099 | 0.878562 | 0.12933 | 0.061161 | 0.376939 | 0.025068 | 0.006502 | 0.079445 | 0.923368 | 1.045642 | 0.46866 | 0.205348 | 0.759041 | 0.720556 | 0 | 1.044813 | 0.181004 | 0.370681 | 0.81285 | 0.653742 |
| Q8BXV2 | Q8BXV2 | BRI3B_MO | Bri3bp | BRI3-binding protein | + | | | 1.439968 | 1.809509 | 5.103872 | 0.121586 | 0.171108 | 0.356114 | 0.737024 | 0.522943 | 2.002075 | -1.94592 | -1.32594 | -3.75174 | -3.06645 | -2.63702 | -2.84328 | -3.61749 | -3.27339 | -3.24336 | -3.91705 | -3.02311 | -3.50307 |
| Q9CQQ7 | Q9CQQ7 | AT5F1_MC | Atp5pb | ATP synthase F(0) complex subunit B1, mitochondrial | | | | 0.854266 | 0.276133 | 2.384729 | 0.160733 | 0.085077 | 0.460031 | 0.922499 | 0.465271 | 2.626295 | 4.285701 | 4.092924 | 3.85336 | 3.487169 | 4.201447 | 3.943539 | 3.977344 | 3.849016 | 3.611222 | 3.559154 | 3.728697 | 3.485748 |
| Q9DCX2 | Q9DCX2 | ATP5H_M( | Atp5pd | ATP synthase subunit d, mitochondrial | | | | 0.809027 | 0.571732 | 2.232524 | 0.114558 | -0.08221 | -0.33708 | 0.593135 | 0.302682 | 1.578534 | 4.775992 | 4.662782 | 4.154287 | 3.795714 | 4.379596 | 4.474309 | 4.397413 | 3.897897 | 4.222555 | 3.891867 | 4.31008 | 3.938462 |
| P97450 | P97450 | ATP5J_MO | Atp5pf | ATP synthase-coupling factor 6, mitochondrial | | | | 0.22554 | 0.050073 | 0.626576 | 0.253466 | 0.117061 | 0.6971 | 0.032998 | 0.020832 | 0.103751 | 3.167583 | 3.035349 | 2.708661 | 2.453584 | 2.536672 | 2.297714 | 3.096282 | 3.006505 | 2.5733 | 2.354822 | 2.557736 | 2.234984 |
| Q9DB20 | Q9DB20 | ATPO_MOI | Atp5po | ATP synthase subunit O, mitochondrial | | | | 0.231755 | 0.142005 | 0.642316 | 0.13397 | -0.0774 | -0.38935 | 0.434401 | 0.166234 | 1.153955 | 3.671043 | 3.635857 | 3.506194 | 3.113692 | 3.706279 | 3.423878 | 3.731827 | 3.291063 | 3.41903 | 3.355655 | 3.370305 | 3.427384 |
| Q9R1Q9 | Q9R1Q9 | VAS1_MOI | Atp6ap1 | V-type proton ATPase subunit S1 | | | | 0.617897 | 1.517743 | 1.648355 | 0.965009 | 0.519564 | 2.784701 | 0.270111 | -0.10164 | -0.73894 | -3.39025 | -1.6282 | -1.48549 | -1.15762 | -1.67072 | -1.44348 | -4.29455 | -3.75939 | -1.93021 | -1.75203 | -1.53299 | -1.37793 |
| Q9CYN9 | Q9CYN9 | RENR_MOI | Atp6ap2 | Renin receptor | | | | 0.383379 | 1.624635 | 1.023691 | 0.561353 | 0.692641 | 1.490528 | 0.259629 | 0.244948 | 0.712606 | -1.65794 | -0.32048 | -0.43697 | 0.192762 | -0.00794 | -0.61039 | -1.17458 | -4.05311 | -0.47298 | -1.15651 | -0.71969 | -0.38853 |
| Q9Z1G4 | Q9Z1G4 | VPP1_MOI | Atp6v0a1 | V-type proton ATPase 116 kDa subunit a 1 | | | | 0.726114 | -0.26472 | -1.96833 | 0.390072 | 0.116647 | 1.040666 | 0.00058 | -0.00053 | -0.00189 | 2.73004 | 2.995564 | 3.614004 | 3.396464 | 3.745815 | 4.038781 | 3.148994 | 3.106042 | 3.361512 | 3.415662 | 4.133481 | 3.652178 |
| Q9Z0V2 | Q9Z0V2 | KCND2_M( | Kcnd2 | Potassium voltage-gated channel subfamily D member 2 | + | | | 1.882519 | 1.795878 | 8.648834 | 0.559737 | 0.802294 | 1.486098 | 0.250942 | 0.294687 | 0.690745 | -1.55417 | -1.54742 | -1.41952 | -0.93911 | -0.64904 | -1.2233 | -3.13906 | -3.55429 | -2.46509 | -1.49812 | -1.54639 | -0.91532 |
| P51863 | P51863 | VA0D1_M( | Atp6v0d1 | V-type proton ATPase subunit d 1 | + | | | 1.119717 | -0.60748 | -3.41961 | 0.943514 | -0.38729 | -2.70381 | 0.040391 | -0.06396 | -0.1261 | 2.890304 | 3.115527 | 3.763735 | 3.498458 | 3.75342 | 4.611472 | 3.747794 | 3.473005 | 4.072472 | 3.96431 | 4.516978 | 3.97583 |
| P50516 | P50516 | VATA_MOI | Atp6v1a | V-type proton ATPase catalytic subunit A | | | | 0.269196 | -0.17941 | -0.73665 | 0.126232 | -0.12944 | -0.36862 | 0.595987 | 0.319617 | 1.586517 | 3.069995 | 2.903275 | 4.055224 | 3.357956 | 4.167233 | 4.501624 | 3.394876 | 2.937208 | 3.794212 | 3.877841 | 4.127199 | 3.902424 |
| P62814 | P62814 | VATB2_M( | Atp6v1b2 | V-type proton ATPase subunit B, brain isoform | | | | 0.765507 | -0.3854 | -2.09159 | 0.108111 | -0.12148 | -0.3195 | 0.121629 | 0.142372 | 0.356229 | 1.535202 | 1.826842 | 2.881066 | 2.34603 | 2.929124 | 3.572829 | 2.179073 | 1.953778 | 2.464839 | 3.005221 | 3.34555 | 2.871658 |
| Q9Z1G3 | Q9Z1G3 | VATC1_M( | Atp6v1c1 | V-type proton ATPase subunit C 1 | + | | | 1.022189 | -0.81282 | -3.00818 | 0.42591 | 0.249405 | 1.132125 | 0.023167 | -0.02506 | -0.07356 | -0.1456 | -0.55108 | 1.354496 | 0.962746 | 0.703999 | 1.369658 | 0.643112 | 0.285856 | 0.808399 | 1.010033 | 1.134647 | 0.989133 |
| P57746 | P57746 | VATD_MOI | Atp6v1d | V-type proton ATPase subunit D | | | | 0.192359 | 0.162358 | 0.541954 | 0.567869 | 0.42178 | 1.508429 | 0.160202 | -0.08248 | -0.45864 | 0.648942 | 0.212803 | 1.53829 | 0.994598 | 0.869896 | 1.218707 | 0.473924 | 0.063107 | 0.910122 | 0.779205 | 1.170663 | 1.082904 |
| P50518 | P50518 | VATE1_M( | Atp6v1e1 | V-type proton ATPase subunit E 1 | | | + | 0.506518 | -0.12318 | -1.34251 | 0.493943 | 0.599067 | 1.309181 | 1.094965 | -0.3051 | -3.31136 | -0.56982 | -0.40351 | 1.609007 | 0.697043 | 0.558676 | 0.695487 | -0.32471 | -0.40226 | 0.515636 | 0.59228 | 0.993901 | 0.870454 |
| Q9D1K2 | Q9D1K2 | VATF_MOI | Atp6v1f | V-type proton ATPase subunit F | | | + | 0.862406 | -0.45048 | -2.41277 | 0.533485 | 0.355756 | 1.414739 | 1.166463 | -0.25493 | -3.63163 | -0.28467 | 0.072182 | 1.539839 | 1.18204 | 1.321379 | 1.224392 | 0.399238 | 0.289246 | 0.828467 | 1.181901 | 1.578569 | 1.47706 |
| Q9CR51 | Q9CR51 | VATG1_M( | Atp6v1g1 | V-type proton ATPase subunit G 1 | | | | 0.014821 | -0.05195 | -0.04748 | 0.886246 | 0.386631 | 2.496078 | 0.225125 | 0.451444 | 0.625522 | -3.09745 | -1.44106 | -0.86137 | -1.00786 | -1.21173 | -0.22216 | -2.93238 | -1.50223 | -1.45773 | -1.18476 | -0.64299 | -1.69379 |
| Q9WTT4 | Q9WTT4 | VATG2_M( | Atp6v1g2 | V-type proton ATPase subunit G 2 | | | | 0.015845 | -0.11019 | -0.0507 | 0.277424 | 0.44255 | 0.757302 | 0.638494 | -0.65089 | -1.70733 | -0.9246 | -3.57409 | -0.09522 | -0.9807 | -1.32296 | -0.62796 | -0.41623 | -3.86209 | -1.36192 | -0.59909 | -0.1678 | -0.48136 |
| Q8BVE3 | Q8BVE3 | VATH_MOI | Atp6v1h | V-type proton ATPase subunit H | | | + | 0.049507 | -0.02303 | -0.15325 | 0.412364 | 0.153811 | 1.097433 | 1.118243 | 0.53869 | 3.413083 | 2.317029 | 2.044193 | 2.796362 | 2.55856 | 3.450239 | 3.134855 | 2.140601 | 2.266683 | 2.597853 | 2.449447 | 2.760475 | 2.747238 |
| P15655 | P15655 | FGF2_MOI | Fgf2 | Fibroblast growth factor 2 | + | | | 1.588713 | 1.777416 | 6.107037 | 0.287302 | 0.273758 | 0.78208 | 0.856143 | -0.57215 | -2.39118 | -0.46576 | 0.013739 | -0.42393 | -0.1293 | -1.39887 | -1.1407 | -1.83843 | -2.16843 | -0.23284 | -0.8679 | -0.8991 | -0.49616 |
| O70228 | O70228 | AT9A_M( | Atp9a | Probable phospholipid-transporting ATPase IIA | | | | 0.572607 | 0.744472 | 1.521491 | 0.636066 | 0.37834 | 1.700336 | 0.305163 | -0.20205 | -0.82686 | 0.560697 | 0.754494 | 0.152628 | 0.597568 | 0.435475 | 0.16048 | 0.392737 | -0.56649 | -0.00744 | 0.000957 | 0.298023 | 0.702034 |
| Q811I0 | Q811I0 | ATPF1_M( | Atpaf1 | ATP synthase mitochondrial F1 complex assembly factor 1 | | | | 0.164601 | -0.60559 | -0.47013 | 0.308414 | 0.200395 | 0.835008 | 0.227747 | 0.088588 | 0.632167 | -3.55212 | -2.15882 | -1.67336 | -1.73582 | -2.54699 | -2.61049 | -1.1664 | -3.33336 | -1.66703 | -2.14294 | -2.80382 | -2.53084 |
| P62077 | P62077 | TIM8B_MC | Timm8b | Mitochondrial import inner membrane translocase subunit Tim8 B | + | | | 2.60325 | 1.763566 | 19.98995 | 0.335018 | 0.604727 | 0.901752 | 0.092682 | -0.40679 | -0.27694 | 2.053358 | 1.876913 | -1.97819 | -3.31715 | -3.9977 | -1.53177 | 0.201435 | 0.201704 | -3.29142 | -3.21337 | -1.55955 | -3.15634 |
| P54254 | P54254 | ATX1_MOI | Atxn1 | Ataxin-1 | | + | | NaN | 0 | NaN | 1.211994 | -0.55095 | -3.84819 | 0.981548 | -0.6138 | -2.84808 | -3.1336 | -2.92442 | -1.47273 | -1.73326 | -2.40201 | -2.818 | -3.47346 | -3.27675 | -1.11144 | -0.99265 | -2.05264 | -1.93977 |

| | | | | | | | | | | | | | | | | | | | | | | | | | | | | |
|---|---|---|---|---|---|---|---|---|---|---|---|---|---|---|---|---|---|---|---|---|---|---|---|---|---|---|---|---|
| P28658 | P28658 | ATX10_MC | Atxn10 | Ataxin-10 | | + | | 0.280257 | 0.109644 | 0.76441 | 1.341117 | 0.466247 | 4.521682 | 0.323454 | 0.380771 | 0.872725 | -0.03189 | -0.19753 | 0.287697 | 0.110842 | 0.665051 | -0.17089 | -0.34146 | -0.10725 | -0.32001 | -0.21394 | -0.25883 | -0.00856 |
| O70305 | O70305 | ATX2_MOU | Atxn2 | Ataxin-2 | | + | + | 0.530748 | 1.065736 | 1.407358 | 1.609637 | 0.675276 | 6.261802 | 1.764362 | 0.80945 | 7.525238 | -0.99202 | -2.41548 | -1.41101 | -1.19804 | -1.69517 | -1.74076 | -3.0281 | -2.51088 | -1.99684 | -1.96275 | -2.63253 | -2.42229 |
| Q7TQH0 | Q7TQH0 | ATX2L_MC | Atxn2l | Ataxin-2-like protein | | | | 0.012337 | 0.105101 | 0.039622 | 0.488925 | -0.35802 | -1.29594 | 0.094839 | -0.08506 | -0.28293 | 0.032693 | -3.5248 | 0.072997 | 0.617252 | 0.151686 | 0.607851 | 0.116628 | -3.81893 | 0.655538 | 0.750742 | 0.268989 | 0.660658 |
| E9Q2E4 | E9Q2E4 | E9Q2E4_M | Hectd4 | HECT domain E3 ubiquitin protein ligase 4 | + | | | 1.600333 | 1.748652 | 6.192544 | 0.448747 | 0.213444 | 1.190997 | 0.244384 | 0.122413 | 0.674214 | -0.73211 | -1.22167 | -0.03009 | 0.097689 | -0.43607 | -0.21665 | -2.86633 | -2.58476 | -0.34708 | -0.0122 | -0.59344 | -0.3041 |
| A0A087WI | A0A087WI | AUTS2_MC | Auts2 | Autism susceptibility gene 2 protein homolog | | | | 0.122161 | 0.642978 | 0.357665 | 0.120016 | -0.13371 | -0.35187 | 0.126171 | -0.15737 | -0.36846 | -3.50122 | -0.09362 | -1.15063 | -0.42517 | -0.47041 | -1.22561 | -3.01382 | -1.86697 | -0.54096 | -0.76743 | -0.89023 | -0.49105 |
| Q80U56 | Q80U56 | AVL9_MOU | Avl9 | Late secretory pathway protein AVL9 homolog | | | | NaN | 0 | NaN | 0.100029 | -0.18477 | -0.2973 | 0.780934 | 0.956904 | 2.140958 | -3.66684 | -3.99801 | -0.76433 | -1.59389 | -0.15046 | -0.76911 | -2.66188 | -3.23002 | -1.45718 | -0.5315 | -1.09407 | -1.73931 |
| Q9CZP5 | Q9CZP5 | BCS1_MOU | Bcs1l | Mitochondrial chaperone BCS1 | + | | | 1.822416 | 1.709965 | 8.05864 | 0.614793 | 0.716538 | 1.63954 | 0.400642 | 0.57969 | 1.067536 | -1.63691 | -1.86429 | -1.33101 | -2.03601 | -0.89011 | -1.93949 | -3.2814 | -3.63973 | -2.6584 | -2.1417 | -2.13437 | -1.85461 |
| O88566 | O88566 | AXIN2_MO | Axin2 | Axin-2 | | | | 0.326652 | 1.293253 | 0.880748 | 0.199579 | -0.14622 | -0.56047 | 0.259321 | 0.226258 | 0.711831 | -3.4145 | -0.47835 | -1.08942 | -0.58696 | -1.07435 | -0.52577 | -3.2108 | -3.26856 | -0.76231 | -0.62165 | -1.18693 | -0.8657 |
| Q8CA95 | Q8CA95 | PDE10_MC | Pde10a | cAMP and cAMP-inhibited cGMP 3',5'-cyclic phosphodiesterase 10A | + | + | | 2.617501 | 1.679766 | 20.32187 | 1.246013 | 1.804097 | 4.016825 | 0.333681 | 0.271149 | 0.898393 | 4.111897 | 4.275948 | 7.305866 | 8.160803 | 4.012289 | 3.720993 | 2.524363 | 2.503951 | 5.791422 | 6.067052 | 3.331145 | 3.859838 |
| P01887 | P01887 | B2MG_MO | B2m | Beta-2-microglobulin | | | + | NaN | 0 | NaN | 0.365203 | 0.674281 | 0.977731 | 2.196212 | 2.058017 | 12.47456 | -3.45105 | -3.56732 | -1.8775 | -3.12418 | -1.37245 | -1.6827 | -4.06574 | -3.40341 | -3.47017 | -2.88007 | -3.64175 | -3.52943 |
| Q7TNS5 | Q7TNS5 | Q7TNS5_M | B630019K06Rik | RIKEN cDNA B630019K06 gene | + | | | 1.171964 | -0.98389 | -3.65726 | 0.369375 | 0.73368 | 0.988264 | 0.060477 | -0.12811 | -0.18541 | -3.67761 | -4.04409 | -3.02496 | -1.67317 | -2.31658 | -3.39073 | -2.67998 | -3.07393 | -3.38985 | -2.77564 | -3.16024 | -2.29084 |
| Q3UI43 | Q3UI43 | BABA1_MC | Babam1 | BRISC and BRCA1-A complex member 1 | | | | NaN | 0 | NaN | 0.322871 | 0.724032 | 0.871261 | 0.614553 | 0.472184 | 1.63886 | -3.53236 | -4.44468 | -1.93531 | -2.93445 | -2.24 | -1.81312 | -3.38585 | -2.82519 | -3.82301 | -2.49482 | -2.69228 | -2.30521 |
| Q8K3W0 | Q8K3W0 | BABA2_MC | Babam2 | BRISC and BRCA1-A complex member 2 | | | | 0.907055 | -2.02118 | -2.57029 | 0.088016 | 0.035145 | 0.263914 | 0.036796 | -0.02601 | -0.11527 | -2.54725 | -4.11625 | -0.75232 | -1.00721 | -0.72335 | -1.01631 | -1.36468 | -1.25646 | -0.87628 | -0.95354 | -1.01545 | -0.67219 |
| Q91YN9 | Q91YN9 | BAG2_MO | Bag2 | BAG family molecular chaperone regulator 2 | | + | | 0.970906 | 0.967884 | 2.807182 | 1.25469 | -0.14615 | -4.06081 | 0.713005 | -0.1255 | -1.92817 | 0.314284 | 0.208061 | 0.945285 | 0.933932 | 0.609467 | 0.550348 | -0.36604 | -1.04738 | 1.050221 | 1.121302 | 0.7634 | 0.64742 |
| Q9JLV1 | Q9JLV1 | BAG3_MO | Bag3 | BAG family molecular chaperone regulator 3 | | + | | 0.733638 | 0.777899 | 1.99157 | 2.087377 | -2.42683 | -10.9904 | 0.361179 | 0.25719 | 0.967577 | -0.07017 | -0.83344 | -3.60626 | -3.57373 | -1.01087 | -0.48155 | -1.14654 | -1.31287 | -1.38338 | -0.94295 | -1.02806 | -0.97874 |
| Q8CI61 | Q8CI61 | BAG4_MO | Bag4 | BAG family molecular chaperone regulator 4 | | | | 0.476654 | 1.672744 | 1.263685 | 0.717476 | 1.084957 | 1.941817 | 0.179383 | -0.14694 | -0.50852 | 0.228953 | -0.86989 | 0.263837 | 0.527499 | 0.020771 | -0.04821 | -0.19751 | -0.78892 | -1.23225 | -0.14633 | -0.15367 | 0.420104 |
| Q8CI32 | Q8CI32 | BAG5_MO | Bag5 | BAG family molecular chaperone regulator 5 | | | + | 0.389784 | 0.504378 | 1.039936 | 0.342444 | 0.247141 | 0.920413 | 1.017684 | -0.89565 | -2.99012 | 0.649172 | -0.23964 | 1.823925 | 2.113266 | 0.618128 | 0.997917 | -0.10535 | -0.49387 | 1.495249 | 1.947659 | 1.472022 | 1.935326 |
| Q9Z1R2 | Q9Z1R2 | BAG6_MO | Bag6 | Large proline-rich protein BAG6 | | | | 0.6028 | 0.439659 | 1.605651 | 0.346887 | -0.13014 | -0.93158 | 0.584137 | -0.47026 | -1.55344 | 0.662402 | 0.156765 | 1.541098 | 1.509361 | 0.239802 | 0.826622 | 0.075092 | -0.13524 | 1.516576 | 1.794167 | 0.928964 | 1.077986 |
| Q8BKX1 | Q8BKX1 | BAIP2_MO | Baiap2 | Brain-specific angiogenesis inhibitor 1-associated protein 2 | | | | 0.115642 | -0.52588 | -0.34002 | 0.153495 | -0.11493 | -0.44105 | 0.728298 | 0.712832 | 1.97506 | -3.45782 | -0.82472 | 0.036082 | -0.44612 | 1.254218 | 0.553248 | -2.42697 | -0.8038 | -0.18892 | 0.00873 | 0.277046 | 0.104756 |
| Q80TT2 | Q80TT2 | BAIP3_MO | Baiap3 | BAI1-associated protein 3 | | | | 0.41199 | 0.348101 | 1.096479 | 0.512893 | -0.60939 | -1.35949 | 0.020145 | -0.0295 | -0.06417 | -1.05815 | -0.86824 | -3.27128 | -2.44214 | -3.13172 | -3.81075 | -1.00836 | -1.61423 | -2.07685 | -2.41778 | -3.13171 | -3.75175 |
| O54962 | O54962 | BAF_MOU | Banf1 | Barrier-to-autointegration factor | | | | 0.614061 | 1.022288 | 1.637462 | 0.157449 | 0.312476 | 0.451432 | 0.174381 | -0.43053 | -0.49557 | 0.507468 | -0.73076 | 0.162405 | 1.546661 | -1.1564 | -0.62902 | -1.05354 | -1.21433 | 0.551179 | 0.532935 | -1.28996 | 0.365604 |
| Q91XV3 | Q91XV3 | BASP1_MC | Basp1 | Brain acid soluble protein 1 | | | | 0.104738 | 0.091518 | 0.310259 | 0.264424 | 1.114195 | 0.724659 | 0.148932 | 0.325606 | 0.42904 | -0.08406 | 0.247827 | 2.828313 | -0.23639 | 1.812426 | 0.653263 | -0.2535 | 0.234233 | 0.308019 | 0.055513 | 1.397179 | 0.417297 |
| A0A338P6 | A0A338P6 | A0A338P6 | BC049762 | cDNA sequence BC049762 | | | | 0.938415 | 2.432491 | 2.68486 | 0.07027 | -0.10562 | -0.21367 | 0.292891 | 0.157602 | 0.796093 | 0.397891 | 0.322548 | 0.62353 | 1.250309 | 0.781465 | 1.170892 | -1.16705 | -2.97749 | 1.424844 | 0.660243 | 0.782822 | 0.854331 |
| Q61361 | Q61361 | PGCB_MO | Bcan | Brevican core protein | | | | 0.133777 | 0.195338 | 0.388834 | 0.134499 | 0.114684 | 0.390761 | 0.442065 | 0.379192 | 1.17372 | 1.991638 | 1.654033 | 1.66346 | 1.078071 | 1.849922 | 1.267705 | 2.100657 | 1.154339 | 1.277666 | 1.234497 | 1.319726 | 1.039518 |
| Q61140 | Q61140 | BCAR1_MC | Bcar1 | Breast cancer anti-estrogen resistance protein 1 | | | | 0.552911 | 1.524836 | 1.467438 | 0.251027 | -1.3507 | -0.69096 | 0.370372 | -0.37255 | -0.99078 | -1.2504 | -0.71399 | 0.115278 | -3.55881 | -0.45551 | 0.052643 | -1.50313 | -3.51094 | -1.03933 | 0.297186 | 0.448289 | -0.10607 |
| Q80XI4 | Q80XI4;O7 | PI42B_MO | Pip4k2b | Phosphatidylinositol 5-phosphate 4-kinase type-2 beta | + | | | 1.847675 | 1.678579 | 8.301815 | 0.475218 | 0.222915 | 1.259925 | 0.03079 | 0.045606 | 0.097018 | 2.047809 | 2.180791 | 3.366354 | 3.500067 | 3.352927 | 2.492617 | 0.244773 | 0.62667 | 3.046486 | 3.374105 | 2.687592 | 3.066741 |
| Q9D287 | Q9D287 | SPF27_MC | Bcas2 | Pre-mRNA-splicing factor SPF27 | | | | 0.847477 | 0.839191 | 2.361499 | 0.880622 | 0.223868 | 2.476263 | 0.69398 | 1.10323 | 1.870602 | -2.32166 | -2.43554 | -2.33399 | -2.21788 | -1.29452 | -2.4505 | -2.86702 | -3.56857 | -2.56911 | -2.4305 | -2.85845 | -3.09303 |
| Q8CCN5 | Q8CCN5 | BCAS3_MC | Bcas3 | BCAS3 microtubule associated cell migration factor | | | + | 0.740501 | 0.2807 | 2.012893 | 0.063424 | -0.04376 | -0.19396 | 1.614864 | -0.23643 | -6.30103 | -0.29696 | -0.55123 | 0.539175 | 0.410666 | 0.151341 | 0.180584 | -0.64749 | -0.76209 | 0.302417 | 0.734938 | 0.436946 | 0.367834 |
| P24288 | P24288 | BCAT1_MC | Bcat1 | Branched-chain-amino-acid aminotransferase, cytosolic | | | | 0.129436 | 0.079592 | 0.377225 | 0.677167 | 0.161198 | 1.820421 | 0.173472 | 0.14291 | 0.49321 | 1.601242 | 1.516896 | 0.536214 | 0.449255 | 0.633276 | 0.923766 | 1.686213 | 1.272741 | 0.254396 | 0.408677 | 0.886333 | 0.384888 |
| Q6P3A8 | Q6P3A8 | ODBB_MO | Bckdhb | 2-oxoisovalerate dehydrogenase subunit beta, mitochondrial | | | + | 0.167027 | -0.20438 | -0.47646 | 0.013392 | -0.03747 | -0.04296 | 1.459469 | -0.97664 | -5.22627 | -2.65518 | -3.46388 | -2.74105 | -3.99815 | -2.4828 | -2.834 | -2.99836 | -2.71194 | -3.93657 | -2.7277 | -1.61785 | -1.74567 |
| Q8K019 | Q8K019 | BCLF1_MC | Bclaf1 | Bcl-2-associated transcription factor 1 | | | + | 0.898547 | 1.616112 | 2.539775 | 0.150001 | 0.032143 | 0.431857 | 1.070511 | 0.841689 | 3.207023 | 0.754164 | 1.427919 | 0.86908 | 0.793121 | 1.492671 | 1.120704 | 0.014761 | -1.0649 | 0.862969 | 0.734946 | 0.279819 | 0.650177 |
| Q9WVA2 | Q9WVA2;( | TIM8A_MC | Timm8a1 | Mitochondrial import inner membrane translocase subunit Tim8 A | + | | | 1.692475 | 1.641016 | 6.911076 | 0.15524 | 0.310118 | 0.445638 | 0.446544 | -0.94058 | -1.1853 | 1.837654 | 1.363697 | -1.29515 | -2.62496 | -1.91127 | -3.49681 | -0.05525 | -0.02543 | -2.06482 | -2.47553 | -1.7984 | -1.72852 |
| Q80XN0 | Q80XN0 | BDH_MOU | Bdh1 | D-beta-hydroxybutyrate dehydrogenase, mitochondrial | + | | | 1.082174 | 0.600376 | 3.256466 | 0.551709 | 0.251128 | 1.46416 | 0.292857 | 0.196426 | 0.796009 | 3.762295 | 4.052019 | 4.068118 | 4.149016 | 4.178891 | 3.880327 | 3.19274 | 3.420822 | 3.69076 | 4.024118 | 3.636696 | 4.02967 |
| P21237 | P21237 | BDNF_MO | Bdnf | Brain-derived neurotrophic factor | | + | + | 0.587437 | 2.169145 | 1.562624 | 1.314756 | 2.411617 | 4.376601 | 2.421408 | 2.693295 | 16.19821 | 0.038658 | 0.093192 | 2.106721 | 1.603398 | 2.65206 | 2.325851 | -0.71535 | -3.4911 | -1.04676 | -0.06636 | -0.17204 | -0.23664 |
| O88597 | O88597 | BECN1_M | Becn1 | Beclin-1 | + | | | 1.174822 | 1.343537 | 3.670628 | 0.924444 | 0.963768 | 2.633405 | 0.172708 | -0.1876 | -0.49123 | 1.929192 | 2.033807 | 2.843189 | 3.572434 | 1.305736 | 1.346916 | 1.00023 | 0.275696 | 2.275508 | 2.212578 | 1.132578 | 1.895279 |
| Q68EF6 | Q68EF6 | BEGIN_MC | Begain | Brain-enriched guanylate kinase-associated protein | | | | 0.001367 | -0.00381 | -0.00444 | 0.164813 | 0.385173 | 0.470685 | 0.391847 | 0.88523 | 1.045172 | -2.22687 | -3.92694 | -1.87441 | -2.90107 | -0.5229 | -2.09196 | -3.18066 | -2.96553 | -2.13562 | -3.41021 | -1.87348 | -2.51184 |
| P28653 | P28653 | PGS1_MO | Bgn | Biglycan | | | | 0.973549 | 2.014873 | 2.8173 | 0.839262 | -1.47141 | -2.33358 | 0.347658 | 0.922186 | 0.933524 | -0.69171 | -1.30979 | -4.182 | -2.92109 | -0.75249 | -1.90022 | -3.66059 | -2.37066 | -2.07001 | -2.09028 | -3.05262 | -1.44447 |
| D3YZU1 | D3YZU1 | SHAN1_M | Shank1 | SH3 and multiple ankyrin repeat domains protein 1 | + | | | 1.437275 | 1.626908 | 5.087174 | 0.660559 | -0.36781 | -1.77147 | 0.115455 | 0.192317 | 0.339517 | -0.32462 | 0.312393 | 0.56139 | 0.351277 | 2.212967 | 1.171002 | -1.6618 | -1.60425 | 0.645054 | 1.003239 | 1.277329 | 1.722006 |
| O88738 | O88738 | BIRC6_MC | Birc6 | Baculoviral IAP repeat-containing protein 6 | | | | 0.107109 | 0.534744 | 0.316761 | 0.324012 | -0.19386 | -0.87412 | 0.695413 | -0.49373 | -1.87491 | -3.87842 | -0.58997 | -1.122 | -0.74921 | -1.2169 | -1.14715 | -3.15158 | -2.38631 | -0.86192 | -0.62158 | -0.94931 | -0.42728 |
| Q8R016 | Q8R016 | BLMH_MO | Blmh | Bleomycin hydrolase | + | | | 1.164997 | 1.116292 | 3.624826 | 0.435924 | -0.64896 | -1.15788 | 0.229166 | -0.18659 | -0.63576 | 0.823236 | 0.290361 | -0.36517 | -1.02154 | -0.86987 | -0.3261 | -0.71392 | -0.40506 | -0.49874 | 0.409936 | -0.30086 | -0.52192 |
| A2AAE1 | A2AAE1 | BLTP1_MC | Bltp1 | Bridge-like lipid transfer protein family member 1 | | | | 0.075525 | 0.114392 | 0.22867 | 0.376823 | 0.218578 | 1.007091 | 0.677346 | -0.89768 | -1.82095 | -0.27724 | -0.20411 | 1.450755 | 1.834272 | -0.29428 | 0.506964 | -0.85398 | 0.143843 | 1.322277 | 1.525594 | 0.716753 | 1.291291 |
| P26450 | P26450 | P85A_MOI | Pik3r1 | Phosphatidylinositol 3-kinase regulatory subunit alpha | + | | | 1.546682 | 1.623005 | 5.80676 | 0.787646 | 0.257683 | 2.162638 | 0.107669 | 0.061439 | 0.318294 | 0.114962 | 0.01468 | -0.02172 | 0.21559 | -0.71971 | -0.34473 | -1.83315 | -1.28322 | -0.1716 | -0.1499 | -0.54778 | -0.63954 |
| Q9D8S9 | Q9D8S9 | BOLA1_MC | Bola1 | BolA-like protein 1 | | | | 0.680008 | 0.844324 | 1.828856 | 0.703703 | -0.28425 | -1.89992 | 0.592268 | -0.25599 | -1.57611 | -1.32168 | -2.1941 | -1.89808 | -1.82728 | -2.15954 | -1.90995 | -2.45103 | -2.7534 | -1.7238 | -1.43307 | -1.8827 | -1.6748 |
| Q8BGS2 | Q8BGS2 | BOLA2_MC | Bola2 | BolA-like protein 2 | | | | 0.069219 | -0.05324 | -0.21066 | 0.957273 | -0.38792 | -2.7554 | 0.134262 | 0.19271 | 0.390129 | 1.949126 | 1.660895 | 1.226674 | 1.156754 | 1.321909 | 2.259639 | 1.650634 | 2.065866 | 1.716005 | 1.443256 | 1.753521 | 1.442607 |
| P63168 | P63168;Q! | DYL1_MOI | Dynll1 | Dynein light chain 1, cytoplasmic | + | + | | 2.325948 | 1.621471 | 14.50214 | 1.540725 | 0.389691 | 5.765321 | 0.143808 | -0.0724 | -0.4155 | 3.818521 | 3.814759 | 3.976297 | 4.015301 | 3.495575 | 3.766492 | 2.306962 | 2.083375 | 3.670825 | 3.54139 | 3.593827 | 3.813041 |
| Q920P3 | Q920P3 | BRNP1_MC | Brinp1 | BMP/retinoic acid-inducible neural-specific protein 1 | | | | 0.487354 | -1.00198 | -1.2918 | 0.311952 | 0.220106 | 0.843876 | 0.875036 | 0.716429 | 2.456682 | -2.67096 | -3.95192 | -0.58322 | -0.73578 | -0.28885 | -0.78663 | -2.74697 | -1.87196 | -1.12904 | -0.63019 | -1.40615 | -1.10219 |
| Q91VR8 | Q91VR8 | BRK1_MOI | Brk1 | Protein BRICK1 | | | | 0.56364 | 0.664418 | 1.496804 | 0.975252 | -0.3332 | -2.82383 | 0.415737 | -0.49434 | -1.10606 | 0.404953 | 0.434301 | 1.706248 | 1.678085 | 1.225448 | 2.097857 | 0.198857 | -0.68844 | 1.908212 | 2.142515 | 2.058637 | 2.253345 |
| Q9Z2E9 | Q9Z2E9 | BSCL2_MC | Bscl2 | Seipin | | | | 0.994553 | 1.639423 | 2.898627 | 0.396271 | -0.27067 | -1.05642 | 0.715836 | 1.658402 | 1.936803 | -0.76888 | -1.75458 | -0.13587 | -0.57948 | -1.00834 | -0.76626 | -2.62369 | -3.17862 | 0.041259 | -0.21527 | -3.39336 | -1.69804 |
| P18572 | P18572 | BASI_MOU | Bsg | Basigin | | | | 0.697284 | 0.487901 | 1.880537 | 0.020492 | 0.046899 | 0.065247 | 0.213539 | -0.21815 | -0.5961 | 0.373188 | 0.149703 | 0.729394 | -0.68716 | -0.30285 | 0.061552 | 0.007695 | -0.46061 | 0.09666 | -0.14822 | 0.414886 | -0.21988 |
| O88737 | O88737 | BSN_MOU | Bsn | Protein bassoon | | | | 0.685987 | 0.457496 | 1.846666 | 0.101156 | 0.027106 | 0.300408 | 0.156546 | -0.18262 | -0.44906 | -0.62156 | -0.29749 | 1.405784 | 1.323822 | 1.216299 | 0.960222 | -0.72962 | -1.10443 | 1.257309 | 1.418085 | 0.884894 | 1.656867 |
| Q9DB72 | Q9DB72 | BTBDH_M | Btbd17 | BTB/POZ domain-containing protein 17 | | | | 0.781646 | 1.741069 | 2.143254 | 0.091387 | -0.06885 | -0.27333 | 0.166318 | 0.704854 | 0.474609 | -1.83644 | -0.66155 | -1.2355 | -0.85033 | -1.59166 | -2.18095 | -2.42898 | -3.55115 | -1.13645 | -0.81167 | -4.04676 | -1.13555 |
| D3YUB6 | D3YUB6 | BTBD8_MC | Btbd8 | BTB/POZ domain-containing protein 8 | | | | 0.573753 | 0.959469 | 1.524656 | 0.306517 | -0.36396 | -0.83025 | 0.197909 | 0.127896 | 0.556189 | -3.18739 | -2.16872 | -0.20073 | 0.253042 | -0.70597 | -0.24624 | -4.00712 | -3.26793 | 0.015025 | 0.765197 | -0.61023 | -0.59777 |
| Q3ULA2 | Q3ULA2 | FBW1A_M | Btrc | F-box/WD repeat-containing protein 1A | | | | 0.60011 | -0.57883 | -1.59809 | 0.104509 | 0.119372 | 0.30963 | 0.359625 | -0.33301 | -0.96366 | -3.67646 | -3.34599 | -0.47083 | -0.01989 | -1.32053 | -1.12209 | -2.61007 | -3.25471 | -0.67746 | -0.05201 | -1.21932 | -0.55727 |
| Q9WVA3 | Q9WVA3 | BUB3_MO | Bub3 | Mitotic checkpoint protein BUB3 | | | | 0.081743 | 0.268072 | 0.246286 | 0.347053 | -0.42681 | -0.932 | 0.35467 | -0.38148 | -0.95118 | -1.81007 | -3.96935 | -1.82584 | -2.59196 | -2.17021 | -1.74435 | -3.01952 | -3.29604 | -2.03306 | -1.53113 | -1.91567 | -1.23593 |
| Q69ZU6 | Q69ZU6 | THS7A_MC | Thsd7a | Thrombospondin type-1 domain-containing protein 7A | + | | | 1.677037 | 1.602587 | 6.785494 | 0.019405 | -0.02264 | -0.06186 | 0.174314 | 0.449374 | 0.495395 | -1.55404 | -1.90012 | -1.53584 | -1.22483 | -2.36171 | -0.56194 | -3.49041 | -3.16893 | -1.02646 | -1.68895 | -2.02538 | -1.79702 |
| P14106 | P14106 | C1QB_MO | C1qb | Complement C1q subcomponent subunit B | | | | 0.120764 | 0.478041 | 0.353894 | 0.040798 | -0.08451 | -0.12731 | 0.03794 | 0.040455 | 0.118719 | -3.2829 | -2.49519 | -0.75708 | -2.08275 | -1.32771 | -0.937 | -2.07498 | -4.6592 | -1.30008 | -1.37074 | -0.89361 | -1.45202 |
| O35658 | O35658 | C1QBP_M | C1qbp | Complement component 1 Q subcomponent-binding protein, mitochondrial | | | | 0.564892 | 1.083182 | 1.500242 | 0.845117 | 1.155264 | 2.353457 | 0.110241 | -0.06994 | -0.32532 | 0.183701 | 0.238382 | 2.060767 | 2.348285 | 0.735164 | 0.322199 | -1.59363 | -0.15065 | 0.579905 | 1.518619 | 0.538708 | 0.658545 |
| Q80X80 | Q80X80 | C2C2L_MC | C2cd2l | Phospholipid transfer protein C2CD2L | | | | NaN | 0 | NaN | 0.993778 | 0.411844 | 2.895594 | 0.326409 | 0.225868 | 0.880138 | -3.79558 | -3.29808 | -1.17312 | -0.97906 | -0.79074 | -1.23618 | -2.76358 | -3.14092 | -1.59193 | -1.38394 | -1.11184 | -1.36682 |
| Q5HZI2 | Q5HZI2 | C2C4C_M | C2cd4c | C2 calcium-dependent domain-containing protein 4C | | + | | 0.392711 | -0.30621 | -1.04737 | 1.510054 | -0.11965 | -5.55619 | 0.329171 | 0.516618 | 0.887071 | -3.1401 | -2.96273 | -0.52001 | -0.50022 | -0.67582 | -0.80424 | -2.46661 | -3.02379 | -0.37893 | -0.422 | -1.83549 | -0.67782 |
| Q3TJZ6 | Q3TJZ6 | FA98A_MC | Fam98a | Protein FAM98A | + | | | 2.697087 | 1.589219 | 22.27864 | 0.41897 | 0.342244 | 1.114333 | 0.205257 | -0.10529 | -0.57498 | -2.40375 | -2.53225 | -2.01975 | -2.62037 | -2.08396 | -1.97272 | -4.02622 | -4.08822 | -2.72665 | -2.59795 | -1.74859 | -2.09752 |
| Q6DFW0 | Q6DFW0 | CI072_MO | C9orf72 | Guanine nucleotide exchange factor C9orf72 homolog | | | | 0.485006 | 1.71859 | 1.285616 | 0.070807 | -0.04189 | -0.21521 | 0.048344 | 0.063298 | 0.149812 | -3.37529 | -2.23827 | -1.82888 | -1.61305 | -2.13077 | -1.67558 | -3.3155 | -5.73524 | -1.84105 | -1.51712 | -2.32245 | -1.61049 |
| P00920 | P00920 | CAH2_MO | Ca2 | Carbonic anhydrase 2 | | | | 0.658609 | -0.78969 | -1.76576 | 0.121092 | -0.23617 | -0.35478 | 0.037274 | -0.04964 | -0.11671 | 1.329367 | 1.541126 | 0.381563 | -0.91767 | -0.02561 | 0.654032 | 1.790425 | 2.659445 | 0.113481 | -0.17724 | 0.619564 | 0.108125 |
| P28651 | P28651 | CAH8_MO | Ca8 | Carbonic anhydrase-related protein | | | | 0.310685 | 1.676684 | 0.840701 | NaN | 0 | NaN | NaN | 0 | NaN | -3.47445 | 0.454497 | -3.89222 | -3.54065 | -3.93434 | -3.55695 | -3.5308 | -2.84252 | -4.08031 | -4.45141 | -4.04331 | -2.99012 |
| Q8VDY9 | Q8VDY9 | CAAP1_M | Caap1 | Caspase activity and apoptosis inhibitor 1 | | | | 0.481531 | 0.936591 | 1.276483 | 0.171108 | 0.229997 | 0.487072 | 0.598646 | 0.441671 | 1.593974 | -1.46806 | -1.53098 | -1.12248 | -0.50202 | -2.75697 | -3.25631 | -1.70306 | -3.16916 | -1.39824 | -0.68625 | -3.32813 | -3.56849 |
| Q06138 | Q06138 | CAB39_M | Cab39 | Calcium-binding protein 39 | | + | | 0.911153 | 0.348415 | 2.585071 | 1.74221 | 0.320493 | 7.330687 | 0.330133 | -0.21333 | -0.88948 | 0.718605 | 0.44953 | 0.989231 | 1.024092 | 0.291091 | -0.05924 | 0.227574 | 0.243731 | 0.646074 | 0.726263 | 0.16543 | 0.493087 |
| Q9DB16 | Q9DB16 | CB39L_M | Cab39l | Calcium-binding protein 39-like | | + | | 0.441071 | 2.391147 | 1.171153 | 2.440716 | 4.26239 | 16.56436 | NaN | 0 | NaN | -2.99394 | 1.047564 | 0.857784 | 0.569951 | -3.09179 | -3.7462 | -3.0726 | -3.65607 | -3.76184 | -3.33521 | -3.3494 | -2.75741 |
| D3YZI9 | D3YZI9 | PGBD5_M | Pgbd5 | PiggyBac transposable element-derived protein 5 | + | | | 1.699741 | 1.588574 | 6.970934 | 0.081593 | 0.044521 | 0.245863 | 0.371807 | -0.41417 | -0.99441 | -1.26556 | -1.63058 | 0.97999 | 0.81819 | 0.966856 | 0.563208 | -2.90018 | -2.17311 | 0.692566 | 1.016572 | 0.814869 | 1.543538 |
| Q8BWZ3 | Q8BWZ3 | NAA25_M | Naa25 | N-alpha-acetyltransferase 25, NatB auxiliary subunit | + | | | 1.591791 | 1.584014 | 6.12958 | 0.059683 | 0.107864 | 0.1831 | 0.033678 | -0.04244 | -0.10582 | 1.656183 | 2.012995 | 1.686433 | 2.583272 | 2.015621 | 2.053051 | 0.437531 | 0.063618 | 2.409033 | 1.644944 | 1.676167 | 2.477381 |
| P97445 | P97445 | CAC1A_M | Cacna1a | Voltage-dependent P/Q-type calcium channel subunit alpha-1A | | | | 0.573245 | 1.052249 | 1.523252 | 0.569274 | 0.354687 | 1.5123 | 0.108716 | 0.195313 | 0.321158 | -0.60424 | -0.17414 | -1.42655 | -0.98946 | -0.36237 | -1.54645 | -0.78497 | -2.0979 | -1.64782 | -1.47757 | -1.28879 | -1.01065 |
| O55017 | O55017 | CAC1B_M | Cacna1b | Voltage-dependent N-type calcium channel subunit alpha-1B | | | | 0.444335 | 0.547645 | 1.179584 | 0.125615 | 0.078093 | 0.366969 | 0.492192 | -0.32425 | -1.30456 | -2.59092 | -3.45958 | -0.46598 | -0.04177 | -0.44394 | -0.52802 | -3.73692 | -3.40887 | -0.3147 | -0.34923 | -0.4067 | 0.083246 |
| Q01815 | Q01815 | CAC1C_M | Cacna1c | Voltage-dependent L-type calcium channel subunit alpha-1C | | | | NaN | 0 | NaN | 0.678323 | -1.48524 | -1.82385 | 0.370094 | -0.59884 | -0.99008 | -3.96899 | -3.64948 | -2.13413 | -3.64219 | -2.73489 | -1.52695 | -3.47579 | -3.93612 | -1.71049 | -1.09535 | -1.56459 | -1.49956 |
| Q99246 | Q01815;Q | CAC1D_M | Cacna1d | Voltage-dependent L-type calcium channel subunit alpha-1D | | | | NaN | 0 | NaN | 0.796847 | 0.704469 | 2.192556 | 0.203837 | -0.29471 | -0.57136 | -3.77674 | -3.3908 | -2.04717 | -1.91262 | -2.5703 | -1.88152 | -3.90486 | -2.99774 | -2.99854 | -2.37019 | -2.31519 | -1.54721 |
| Q61290 | Q61290 | CAC1E_M | Cacna1e | Voltage-dependent R-type calcium channel subunit alpha-1E | | | | 0.421617 | 1.198341 | 1.121113 | 0.083894 | -0.07496 | -0.25234 | 0.81516 | -0.7945 | -2.25281 | -1.16356 | -1.19121 | 1.156741 | 1.691338 | 1.122135 | 1.255348 | -1.30693 | -3.44452 | 1.369375 | 1.628633 | 1.636918 | 2.329568 |
| Q5SUF7 | Q5SUF7 | Q5SUF7_M | Cacna1g | Voltage-dependent T-type calcium channel subunit alpha | | | | 0.463586 | 1.059701 | 1.229539 | 0.671096 | -0.74734 | -1.80245 | 0.155165 | 0.267668 | 0.44544 | -1.27486 | -0.97075 | -2.03177 | -1.63584 | -2.11419 | -1.34409 | -3.03085 | -1.33416 | -0.72216 | -1.45078 | -2.45814 | -1.53548 |
| O88427 | O88427 | CAC1H_M | Cacna1h | Voltage-dependent T-type calcium channel subunit alpha-1H | | | | 0.255568 | 0.390297 | 0.702389 | 0.397932 | -0.88613 | -1.06064 | 0.150473 | -0.24825 | -0.4331 | -2.50435 | -3.61347 | -2.85503 | -1.19385 | -1.56566 | -0.54566 | -3.41408 | -3.48433 | -1.22843 | -1.04821 | -0.54576 | -1.06905 |
| E9Q7P2 | E9Q7P2 | E9Q7P2_M | Cacna1i | Voltage-dependent T-type calcium channel subunit alpha | | | | 0.508768 | 1.357349 | 1.348501 | 0.543406 | -0.27494 | -1.44158 | 0.447368 | -0.44397 | -1.18743 | -3.45454 | -1.65745 | -0.51644 | -0.16833 | -1.82644 | -1.09518 | -3.45971 | -4.36698 | 0.010531 | -0.14542 | -1.09501 | -0.93868 |
| O08532 | O08532 | CA2D1_M | Cacna2d1 | Voltage-dependent calcium channel subunit alpha-2/delta-1 | | | | 0.889763 | -2.28084 | -2.50852 | 0.029669 | 0.039049 | 0.093591 | 0.281724 | 0.188917 | 0.768089 | -3.37761 | -1.57693 | 0.094097 | -0.39024 | -0.47008 | -0.13575 | -0.32332 | -0.06953 | -0.52689 | 0.152645 | -0.31142 | -0.67225 |
| Q8R3Z5 | Q8R3Z5 | CACB1_M | Cacnb1 | Voltage-dependent L-type calcium channel subunit beta-1 | | | + | 0.113994 | -0.14213 | -0.33555 | 0.167075 | 0.236134 | 0.476582 | 1.999106 | -0.66993 | -9.91447 | -0.40319 | -1.17104 | 0.683276 | 1.663479 | 1.013003 | 1.13415 | -0.46608 | -0.82389 | 1.010012 | 0.864474 | 1.713565 | 1.773457 |
| Q8CC27 | Q8CC27 | CACB2_M | Cacnb2 | Voltage-dependent L-type calcium channel subunit beta-2 | | | | 0.296493 | 0.283184 | 0.805124 | 0.370907 | 0.617389 | 0.992133 | 0.904073 | -0.60441 | -2.25957 | -0.86471 | -1.40977 | -0.13768 | 1.07116 | 0.798046 | 1.138645 | -1.19808 | -1.64278 | -0.00261 | -0.29869 | 1.409172 | 1.736333 |
| P54285 | P54285 | CACB3_M | Cacnb3 | Voltage-dependent L-type calcium channel subunit beta-3 | | | | 0.269515 | -1.31459 | -0.73745 | 0.197934 | 0.243431 | 0.556254 | 0.614075 | -0.55307 | -1.6375 | -3.40083 | 0.134027 | 1.597647 | 2.268211 | 1.459168 | 1.470111 | -0.08655 | -0.55107 | 1.408247 | 1.970749 | 1.68 | 2.35541 |
| Q8R0S4 | Q8R0S4 | CACB4_M | Cacnb4 | Voltage-dependent L-type calcium channel subunit beta-4 | | | + | 0.447885 | 0.319793 | 1.188766 | 0.001087 | 0.001477 | 0.003536 | 1.165611 | -0.32028 | -3.62767 | 0.250772 | 0.564474 | 2.138861 | 2.9566 | 2.071541 | 1.99212 | 0.306383 | -0.13072 | 2.460714 | 2.631793 | 2.273254 | 2.430959 |
| Q8VHW2 | Q8VHW2 | CCG8_MO | Cacng8 | Voltage-dependent calcium channel gamma-8 subunit | | | | 0.539572 | 1.707688 | 1.431189 | 0.104808 | 0.065975 | 0.310453 | 0.031249 | 0.033609 | 0.098419 | -2.98591 | -0.63327 | -1.05393 | -1.13511 | 0.138738 | 0.09543 | -3.3173 | -3.71726 | -0.9519 | -1.3691 | 0.424277 | -0.25733 |
| E9Q777 | E9Q777 | E9Q777_M | Akap11 | A kinase (PRKA) anchor protein 11 | + | + | | 3.087936 | 1.57763 | 34.97049 | 1.148317 | 0.312828 | 3.54812 | 0.563333 | -0.32591 | -1.49596 | -1.904 | -1.99247 | -0.99079 | -0.84407 | -1.98414 | -1.72478 | -3.53473 | -3.51701 | -1.27917 | -1.18135 | -1.70361 | -1.3535 |
| B2RQC6 | B2RQC6 | PYR1_MOI | Cad | CAD protein | | | | 0.836427 | 0.769498 | 2.323989 | 0.334486 | 0.259804 | 0.900414 | 0.402976 | -0.65952 | -1.07348 | -0.32434 | -0.88539 | 0.29978 | 0.827432 | -0.71752 | -0.13629 | -1.55027 | -1.19847 | 0.186965 | 0.420638 | -0.30868 | 0.773911 |
| Q8R5M8 | Q8R5M8 | CADM1_M | Cadm1 | Cell adhesion molecule 1 | | | | 0.434339 | 0.702433 | 1.153798 | 0.135684 | -0.30905 | -0.39392 | 0.946303 | 0.441387 | 2.714213 | -1.94132 | -1.51665 | -0.4133 | -1.63569 | -0.07694 | -0.37625 | -1.86085 | -3.00199 | -1.20735 | -0.22354 | -0.60435 | -0.73161 |
| Q8BLQ9 | Q8BLQ9 | CADM2_M | Cadm2 | Cell adhesion molecule 2 | | | | 0.034106 | -0.05681 | -0.10712 | 0.337166 | -0.43246 | -0.90715 | 0.644238 | -0.25278 | -1.72393 | -0.8473 | -0.58805 | 1.036382 | 0.102936 | 0.586918 | 0.779267 | -0.14665 | -1.17509 | 0.904972 | 1.099268 | 1.046556 | 0.825189 |
| Q99N28 | Q99N28 | CADM3_M | Cadm3 | Cell adhesion molecule 3 | | | | 0.038848 | 0.052911 | 0.121455 | 0.546164 | -0.54965 | -1.44907 | 0.066019 | 0.078714 | 0.20145 | 0.071535 | 0.662618 | 0.721915 | -0.01033 | 0.398842 | 1.102678 | 0.63423 | -0.0059 | 1.004588 | 0.80629 | 0.841837 | 0.502254 |
| Q8R464 | Q8R464 | CADM4_M | Cadm4 | Cell adhesion molecule 4 | | | | 0.242993 | -0.37695 | -0.67071 | 0.1647 | -0.34075 | -0.47039 | 0.506502 | 1.463897 | 1.342471 | -0.30079 | -1.03124 | -0.26128 | -1.70577 | -0.73796 | -0.71911 | 0.138115 | -0.71624 | -0.58692 | -0.69862 | -1.10202 | -3.28284 |
| Q80TJ1 | Q80TJ1 | CAPS1_M | Cadps | Calcium-dependent secretion activator 1 | | | | 0.875854 | -0.35621 | -2.45954 | 0.464317 | -0.39892 | -1.23145 | 0.568588 | 0.203001 | 1.51041 | 0.930019 | 1.071679 | 1.886812 | 1.337655 | 2.697874 | 2.471124 | 1.483391 | 1.230735 | 1.839261 | 2.18305 | 2.309319 | 2.453677 |
| Q8BYR5 | Q8BYR5 | CAPS2_M | Cadps2 | Calcium-dependent secretion activator 2 | | | + | NaN | 0 | NaN | 0.552166 | -2.40028 | -1.46541 | 1.521539 | 0.458132 | 5.633674 | -3.13614 | -3.37504 | -6.39957 | -3.27722 | -2.36781 | -2.47913 | -2.70469 | -3.9488 | -2.93373 | -1.94251 | -2.94089 | -2.82232 |
| P12658 | P12658 | CALB1_M | Calb1 | Calbindin | | | | 0.353827 | 0.839229 | 0.949053 | 0.159056 | -0.19771 | -0.45564 | 0.091158 | -0.08158 | -0.27269 | 1.268585 | 3.037117 | 1.530468 | 0.665416 | 0.915883 | 1.347331 | 1.318651 | 1.308592 | 1.33025 | 1.261047 | 1.420495 | 1.005888 |
| Q08331 | Q08331 | CALB2_M | Calb2 | Calretinin | | | | 0.075282 | -0.19576 | -0.22798 | 0.494246 | 0.588138 | 1.309983 | 0.483158 | 1.277548 | 1.280757 | -1.11166 | 0.549606 | -0.73176 | -1.62872 | -0.8541 | -1.41856 | -0.30299 | 0.132473 | -1.78935 | -1.74741 | -1.45715 | -3.37062 |
| Q8CGU1 | Q8CGU1 | CACO1_M | Calcoco1 | Calcium-binding and coiled-coil domain-containing protein 1 | | | | 0.946087 | 0.59543 | 2.713406 | 0.546966 | 0.345161 | 1.451249 | 0.515119 | -0.85909 | -1.36544 | -0.47592 | -0.86504 | 1.180103 | 1.635169 | -0.73452 | 0.309347 | -1.3674 | -1.16443 | 0.993227 | 1.131723 | 0.295165 | 0.997841 |
| P0DP26;P | P0DP26;P | CALM1_M | Calm1;Calm2;Calm3 | Calmodulin-1 | | | | 0.023535 | 0.028496 | 0.074703 | 0.336385 | 0.730293 | 0.905186 | 0.281022 | -0.22776 | -0.76633 | 3.218295 | 3.920663 | 5.176305 | 3.577665 | 3.71004 | 3.587698 | 3.392072 | 3.689895 | 3.756205 | 3.53718 | 4.167482 | 3.585782 |
| P14211 | P14211 | CALR_MOI | Calr | Calreticulin | | | | 0.963391 | -0.47542 | -2.77855 | 0.283741 | 0.630943 | 0.773149 | 0.13177 | 0.155247 | 0.383472 | -0.28424 | -0.1215 | 1.032102 | -0.59066 | -0.49794 | 0.224646 | 0.122037 | 0.42307 | -0.32286 | -0.49758 | -0.10923 | -0.47456 |
| O35887 | O35887 | CALU_MO | Calu | Calumenin | | | | NaN | 0 | NaN | 0.825546 | 0.987532 | 2.287406 | 0.681788 | 0.791043 | 1.834149 | -3.45359 | -2.97617 | -1.76923 | -2.63267 | -2.17825 | -2.12504 | -3.19683 | -2.8925 | -3.1906 | -3.18635 | -3.37315 | -2.51222 |
| Q6QWF9 | Q6QWF9;( | CK2N1_M | Camk2n1 | Calcium/calmodulin-dependent protein kinase II inhibitor 1 | | | | 0.02691 | 0.019099 | 0.085121 | 0.316205 | -0.39327 | -0.85454 | 0.332053 | -0.63198 | -0.89431 | 2.490158 | 2.532449 | 4.145349 | 5.047929 | 3.10344 | 4.318307 | 2.268823 | 2.715585 | 4.899753 | 5.080055 | 4.703979 | 3.981731 |
| Q78WH7 | Q78WH7 | CK2N2_M | Camk2n2 | Calcium/calmodulin-dependent protein kinase II inhibitor 2 | | | | 0.821825 | -0.41702 | -2.27497 | 0.240974 | -0.3231 | -0.66561 | 0.107122 | -0.22193 | -0.3168 | 2.921378 | 3.267314 | 2.706265 | 3.653893 | 2.194366 | 3.508499 | 3.572058 | 3.450674 | 3.397699 | 3.608651 | 3.316263 | 2.830451 |
| A2AHC3 | A2AHC3 | CAMP1_M | Camsap1 | Calmodulin-regulated spectrin-associated protein 1 | | | + | 0.378559 | 1.209456 | 1.011483 | 0.499454 | -0.37269 | -1.32376 | 1.534274 | -0.43646 | -5.72075 | -1.1673 | -2.00849 | -1.01837 | -1.07755 | -1.34546 | -1.29347 | -1.67804 | -3.91666 | -0.95525 | -0.39529 | -0.95474 | -0.81128 |
| Q8C1B1 | Q8C1B1 | CAMP2_M | Camsap2 | Calmodulin-regulated spectrin-associated protein 2 | | | | 0.374154 | 0.444559 | 1.00034 | 0.562511 | -0.24555 | -1.4937 | 0.288527 | -0.28175 | -0.78515 | -1.88817 | -1.39227 | -0.23969 | -0.08612 | -0.63076 | -0.17837 | -2.45359 | -1.71597 | -0.06271 | 0.227998 | -0.40139 | 0.155755 |
| Q80VC9 | Q80VC9 | CAMP3_M | Camsap3 | Calmodulin-regulated spectrin-associated protein 3 | | | | 0.490963 | 1.288547 | 1.301314 | 0.094247 | -0.03149 | -0.28129 | 0.09436 | -0.0771 | -0.2816 | -0.95906 | -0.96541 | -0.00694 | 0.096425 | -0.39357 | -0.2976 | -1.2606 | -3.24097 | -0.02307 | 0.175543 | -0.53805 | 0.001089 |
| Q80Y50 | Q80Y50;A: | CMTA2_M | Camta2 | Calmodulin-binding transcription activator 2 | | | | 0.048135 | 0.092395 | 0.149195 | 0.680889 | -0.55004 | -1.83147 | 0.531941 | -0.53338 | -1.41057 | -2.9186 | -4.09567 | -3.02496 | -2.92561 | -3.29015 | -2.63156 | -3.40679 | -3.79227 | -2.12905 | -2.72143 | -2.61334 | -2.24161 |
| Q6ZQ38 | Q6ZQ38 | CAND1_M | Cand1 | Cullin-associated NEDD8-dissociated protein 1 | | | | 0.618052 | 0.249206 | 1.648797 | 0.453566 | -0.1342 | -1.20349 | 0.182617 | 0.124199 | 0.516874 | 2.597552 | 2.455578 | 2.485584 | 2.306515 | 2.835953 | 2.486112 | 2.410796 | 2.143922 | 2.596718 | 2.463782 | 2.372087 | 2.70158 |
| P35564 | P35564 | CALX_MO | Canx | Calnexin | | | | 0.086795 | 0.088052 | 0.260493 | 0.388011 | 0.346038 | 1.035437 | 0.422208 | 0.229746 | 1.12263 | 0.394733 | 0.824767 | 1.15402 | 0.485849 | 0.657457 | 0.341529 | 0.782513 | 0.260884 | 0.482459 | 0.465335 | 0.399858 | 0.139636 |
| P40124 | Q9CYT6;P | CAP1_MO | Cap1 | Adenylyl cyclase-associated protein 1 | + | | | 1.010093 | -0.22051 | -2.95987 | 0.386804 | 0.365602 | 1.032373 | 0.639796 | 1.363212 | 1.711087 | 1.292642 | 1.3108 | 2.35546 | 1.68633 | 4.178789 | 2.616174 | 1.596182 | 1.448289 | 1.53919 | 1.771395 | 2.190088 | 1.87845 |
| Q9CYT6 | Q9CYT6 | CAP2_MO | Cap2 | Adenylyl cyclase-associated protein 2 | | | | 0.503288 | -0.28506 | -1.33393 | 0.111605 | -0.161 | -0.32905 | 0.456793 | 0.869782 | 1.211865 | -1.73072 | -1.31739 | 0.155307 | -0.77043 | 1.439049 | 0.010967 | -1.1846 | -1.29338 | -0.30515 | 0.012018 | -0.07218 | -0.21737 |
| O08529 | O08529 | CAN2_MO | Capn2 | Calpain-2 catalytic subunit | | | | 0.437294 | 0.261646 | 1.161409 | 0.164541 | 0.149461 | 0.469975 | 0.519208 | 0.603468 | 1.376371 | 0.667638 | 0.390228 | 0.229859 | -0.35939 | 1.190152 | 0.317062 | 0.444807 | 0.089768 | -0.09451 | -0.33394 | 0.109325 | 0.190952 |
| O08688 | O08688 | CAN5_MO | Capn5 | Calpain-5 | | | | 0.70242 | -1.86963 | -1.89603 | 0.244953 | -0.67099 | -0.67565 | 0.046141 | -0.04545 | -0.14327 | -1.97754 | -3.86132 | -1.42266 | -3.39016 | -1.17139 | -1.11026 | -0.75794 | -1.34166 | -1.59951 | -1.87134 | -0.77961 | -1.41114 |
| O88456 | O88456 | CPNS1_M | Capns1 | Calpain small subunit 1 | | | | 0.331863 | -0.10201 | -0.89383 | 0.020361 | 0.013682 | 0.064841 | 0.147809 | 0.025653 | 0.426078 | 0.074861 | -0.14154 | -0.39433 | -0.8018 | -0.46199 | -0.51478 | 0.104969 | 0.032372 | -0.55686 | -0.66664 | -0.56815 | -0.45992 |
| Q60865 | Q60865 | CAPR1_M | Caprin1 | Caprin-1 | | | | 0.399693 | 0.272566 | 1.065121 | 0.693372 | 0.292201 | 1.868774 | 0.680707 | -0.10204 | -1.83093 | -0.48265 | -0.39151 | 0.580497 | 0.838818 | -0.15057 | -0.05662 | -0.45783 | -0.96145 | 0.329332 | 0.505581 | -0.03155 | 0.028433 |
| P47753 | P47753 | CAZA1_M | Capza1 | F-actin-capping protein subunit alpha-1 | | | | 0.775213 | 0.978079 | 2.122577 | 0.239497 | -0.20197 | -0.66188 | 0.228881 | 0.374767 | 0.63504 | -1.68772 | -1.20911 | -1.59893 | -1.63279 | -1.71256 | -0.97026 | -2.0327 | -2.82028 | -1.71856 | -1.10923 | -2.175 | -1.25735 |
| P47754 | P47754 | CAZA2_M | Capza2 | F-actin-capping protein subunit alpha-2 | | | | 0.098149 | 0.082331 | 0.292105 | 0.848018 | -0.2829 | -2.36335 | 0.616785 | -0.31076 | -1.6452 | 2.021611 | 2.200392 | 3.074515 | 3.088623 | 3.198486 | 3.54877 | 2.295974 | 1.761367 | 3.244976 | 3.483968 | 3.75513 | 3.613647 |
| P47757 | P47757 | CAPZB_M | Capzb | F-actin-capping protein subunit beta | | | | 0.308663 | 0.13039 | 0.835632 | 0.383318 | -0.14384 | -1.02354 | 0.253497 | -0.26704 | -0.69718 | 1.461132 | 1.371998 | 2.24788 | 2.409613 | 2.284856 | 3.049221 | 1.435713 | 1.136636 | 2.357652 | 2.587514 | 2.908509 | 2.959657 |
| Q9WVG6 | Q9WVG6 | CARM1_M | Carm1 | Histone-arginine methyltransferase CARM1 | + | | | 1.175547 | 0.3931 | 3.674025 | 0.400791 | 0.178592 | 1.067915 | 0.336289 | -0.3023 | -0.90495 | -0.88799 | -0.89627 | -0.72662 | -0.76167 | -1.52917 | -0.87323 | -1.17832 | -1.39214 | -1.0891 | -0.75648 | -0.96234 | -0.83547 |
| Q6EDY6 | Q6EDY6 | CARL1_M | Carmil1 | F-actin-uncapping protein LRRC16A | | | | 0.289548 | -0.69524 | -0.78771 | 0.270795 | -0.24469 | -0.74066 | 0.152171 | -0.11467 | -0.43757 | -2.31518 | -3.8952 | -0.73161 | -1.12998 | -1.34782 | -1.68368 | -2.01641 | -2.80349 | -0.94967 | -0.42254 | -1.1999 | -1.60227 |
| Q3V3V9 | Q3V3V9 | CARL2_M | Carmil2 | Capping protein, Arp2/3 and myosin-I linker protein 2 | | | | 0.239965 | 0.423419 | 0.663065 | 0.202423 | -0.11396 | -0.56774 | 0.270591 | -0.19225 | -0.74015 | -0.75309 | -0.96966 | -0.74145 | -0.45694 | -0.25968 | 0.257504 | -1.91412 | -0.65546 | -0.62686 | -0.34361 | 0.215501 | 0.166809 |
| Q9ER72 | Q9ER72 | SYCC_MO | Cars1 | Cysteine--tRNA ligase, cytoplasmic | | | | 0.311474 | -0.82233 | -0.84268 | 0.424647 | 0.333773 | 1.128884 | 0.119439 | -0.08395 | -0.35031 | -0.89262 | -2.8292 | -0.56897 | -0.89755 | -0.80951 | -1.28735 | -1.15986 | -0.9173 | -1.31285 | -0.82121 | -0.946 | -0.98297 |
| Q6P9K8 | Q6P9K8 | CSKI1_MO | Caskin1 | Caskin-1 | | | + | 0.408954 | 0.065092 | 1.088727 | 0.243675 | -0.22298 | -0.67243 | 1.585593 | -0.53224 | -6.08426 | -0.48694 | -0.39261 | 0.253167 | -0.03782 | -0.15886 | -0.04865 | -0.46813 | -0.54161 | 0.032672 | 0.628631 | 0.360551 | 0.496433 |
| P24270 | P24270 | CATA_MO | Cat | Catalase | | | | 0.622738 | 0.208447 | 1.662142 | 0.098486 | 0.099123 | 0.293035 | 0.006336 | -0.01463 | -0.02048 | 1.134048 | 0.946147 | -0.01487 | -0.63711 | -0.45792 | 0.784396 | 0.748581 | 0.914721 | -0.29234 | -0.55788 | 0.530837 | -0.1751 |
| Q8CAL5 | Q8CAL5 | GPC5_MO | Gpc5 | Glypican-5 | + | | | 3.93345 | 1.576108 | 92.61618 | 0.270106 | -0.21519 | -0.73893 | 0.008236 | 0.009973 | 0.02657 | 0.186499 | 0.176669 | 0.081673 | 0.641447 | 0.8076 | 0.134166 | -1.41082 | -1.37823 | 0.657162 | 0.496329 | 0.295046 | 0.626774 |
| Q63918 | Q63918 | CAVN2_M | Cavin2 | Caveolae-associated protein 2 | | + | | 0.107902 | 0.714506 | 0.318933 | 1.57301 | -1.04355 | -5.99322 | 0.314378 | -0.53416 | -0.84996 | 0.140997 | -4.13676 | -0.95474 | -0.63479 | -2.01795 | -0.90145 | -3.37883 | -2.04595 | 0.180046 | 0.317531 | -1.21418 | -0.6369 |
| Q66L44 | Q66L44 | CBARP_M | Cbarp | Voltage-dependent calcium channel beta subunit-associated regulatory protein | | | | 0.469923 | -0.56505 | -1.24607 | 0.255012 | 0.463845 | 0.70099 | 0.342354 | -0.50115 | -0.92019 | 0.366024 | 0.912003 | 0.923932 | 2.217321 | 0.229472 | 1.230578 | 1.566156 | 0.841977 | 1.246898 | 0.966664 | 1.445784 | 1.016567 |
| Q8BME9 | Q8BME9 | CBLN4_M | Cbln4 | Cerebellin-4 | | + | | 0.553356 | 0.535033 | 1.468652 | 1.661262 | 0.516793 | 6.65938 | 0.091372 | 0.07502 | 0.273287 | 1.919944 | 2.129282 | 2.377086 | 2.531224 | 1.563182 | 1.418044 | 1.838522 | 1.140638 | 1.946456 | 1.928269 | 1.150848 | 1.680337 |
| P48758 | P48758 | CBR1_MO | Cbr1 | Carbonyl reductase [NADPH] 1 | | | | 0.778938 | -0.84867 | -2.13453 | 0.094561 | -0.22551 | -0.28216 | 0.051454 | -0.17946 | -0.159 | 0.085031 | -0.51474 | 0.715461 | -0.87099 | -0.47654 | 0.882572 | 0.894865 | 0.372767 | 0.049989 | 0.245501 | 1.283663 | -0.51871 |
| Q8K354 | Q8K354 | CBR3_MO | Cbr3 | Carbonyl reductase [NADPH] 3 | | | | 0.44304 | -0.24508 | -1.17624 | 0.669259 | -0.33309 | -1.79704 | 0.578749 | -0.62503 | -1.53848 | -0.10387 | -0.23183 | 0.589742 | 0.255634 | 0.334476 | 1.01314 | -0.12106 | 0.275529 | 0.836082 | 0.67547 | 1.522237 | 1.075444 |
| Q91VT4 | Q91VT4 | CBR4_MO | Cbr4 | 3-oxoacyl-[acyl-carrier-protein] reductase | | | | NaN | 0 | NaN | 0.431151 | 0.89498 | 1.145594 | 0.153755 | -0.30693 | -0.44174 | -2.89571 | -3.97778 | -2.36031 | -3.811 | -2.76676 | -1.38301 | -3.33571 | -3.54669 | -4.27081 | -3.69045 | -1.83199 | -1.70392 |
| Q91WT9 | Q91WT9 | CBS_MOU | Cbs | Cystathionine beta-synthase | | | | 0.121881 | 0.464285 | 0.356909 | 0.294058 | -0.65633 | -0.79902 | 0.005052 | -0.01944 | -0.01636 | -4.13201 | -1.56375 | -2.3726 | -4.01514 | -1.98461 | -3.20651 | -3.52007 | -3.10427 | -2.52182 | -2.55325 | -3.59542 | -1.55682 |
| P23198 | P23198 | CBX3_MO | Cbx3 | Chromobox protein homolog 3 | | | | 0.469529 | 0.594043 | 1.245044 | 0.422318 | 0.2762 | 1.12291 | 0.25719 | -0.14686 | -0.70647 | -0.89248 | -0.10598 | -0.628 | -0.21843 | -0.34183 | -0.25657 | -1.36346 | -0.82308 | -0.83567 | -0.56316 | 0.051124 | -0.3558 |
| Q8K1A6 | Q8K1A6 | C2D1A_M | Cc2d1a | Coiled-coil and C2 domain-containing protein 1A | | | | NaN | 0 | NaN | 0.502702 | -1.03596 | -1.33238 | 0.255074 | -0.31065 | -0.70115 | -4.24353 | -3.33546 | -2.21484 | -3.61596 | -2.10736 | -2.92957 | -3.6367 | -4.04932 | -2.21673 | -1.54215 | -2.37303 | -2.0426 |
| Q8CH18 | Q8CH18 | CCAR1_M | Ccar1 | Cell division cycle and apoptosis regulator protein 1 | | | | NaN | 0 | NaN | 0.222169 | -0.28526 | -0.61803 | 0.445493 | 1.123067 | 1.182578 | -3.00309 | -3.35324 | -1.38177 | -1.69629 | -0.36859 | -1.93203 | -4.00763 | -2.29882 | -1.68772 | -0.81983 | -2.81263 | -1.73412 |
| Q8VDP4 | Q8VDP4 | CCAR2_M | Ccar2 | Cell cycle and apoptosis regulator protein 2 | | | | 0.378932 | 0.619525 | 1.012428 | 0.969984 | -0.88912 | -2.80366 | 0.240416 | -0.21448 | -0.6642 | -0.55288 | -0.64032 | 0.434475 | 0.919307 | 0.787692 | 1.05593 | -0.60576 | -1.82648 | 1.361548 | 1.770472 | 0.842546 | 1.430038 |
| Q3TVA9 | Q3TVA9 | CC136_M | Ccdc136 | Coiled-coil domain-containing protein 136 | + | | | 1.025774 | 1.008288 | 3.022606 | 0.042464 | 0.051451 | 0.132306 | 0.98576 | 0.271591 | 2.864379 | -1.56923 | -1.41662 | -1.6962 | -0.95277 | -2.20544 | -2.02715 | -2.17647 | -2.82595 | -1.49018 | -1.26169 | -2.42019 | -2.35559 |
| Q3UHB8 | Q3UHB8 | CC177_M | Ccdc177 | Coiled-coil domain-containing protein 177 | | | + | 0.223263 | 1.193258 | 0.620802 | 1.802257 | -0.87448 | -7.86946 | 0.870079 | -0.86679 | -2.43939 | -0.64404 | 0.079618 | 0.139988 | 0.326321 | 0.12961 | 0.53546 | 0.412293 | -3.36323 | 1.047064 | 1.168198 | 0.907637 | 1.491011 |
| Q640L5 | Q640L5 | CCD18_M | Ccdc18 | Coiled-coil domain-containing protein 18 | | | | 0.047635 | 0.529837 | 0.147711 | 0.451681 | -2.45623 | -1.1986 | 0.05372 | -0.07669 | -0.16567 | -2.76358 | 1.546804 | -3.2493 | 0.717698 | 0.98025 | 1.330005 | -4.00555 | 1.729098 | 1.705383 | 0.675487 | 1.660454 | 0.80319 |
| Q8CEI3 | Q8CEI3 | Q8CEI3_M | Ccdc28a | Coiled-coil domain containing 28A | | | | NaN | 0 | NaN | 0.568023 | 0.664739 | 1.508853 | 0.337825 | -0.06669 | -0.9088 | -4.00268 | -4.00122 | -1.12333 | -0.52812 | -3.07947 | -1.84061 | -3.50593 | -3.20976 | -1.81531 | -1.16562 | -1.40338 | -2.18531 |
| Q8CEG5 | Q8CEG5 | CC28B_M | Ccdc28b | Coiled-coil domain-containing protein 28B | | | | 0.406939 | -0.91194 | -1.08358 | 0.244874 | 0.20021 | 0.675451 | 0.930041 | -1.66513 | -2.65394 | -4.61969 | -3.16387 | -1.30998 | -0.98693 | -3.83271 | -2.61787 | -3.40225 | -2.55742 | -1.5972 | -1.10013 | -1.71731 | -1.40302 |
| D3YZP9 | D3YZP9 | CCDC6_M | Ccdc6 | Coiled-coil domain-containing protein 6 | | | | 0.015612 | -0.04012 | -0.04997 | 0.101989 | 0.383393 | 0.3027 | 0.061344 | -0.0735 | -0.18793 | -3.16063 | -1.8044 | -1.00108 | -1.79476 | -0.57193 | -1.10105 | -2.01264 | -2.87216 | -2.98411 | -0.57851 | -0.47494 | -1.05103 |
| Q5SP85 | Q5SP85 | CC85A_M | Ccdc85a | Coiled-coil domain-containing protein 85A | | | + | 0.11281 | -0.32658 | -0.33233 | 0.462985 | -0.44328 | -1.22797 | 1.049921 | -0.64447 | -3.12113 | -2.5814 | -1.93618 | -1.34502 | -1.18629 | -1.55078 | -1.17449 | -1.00397 | -2.86045 | -1.17453 | -0.47022 | -0.6331 | -0.80324 |
| Q5SNZ0 | Q5SNZ0 | GRDN_MC | Ccdc88a | Girdin | | | + | 0.109206 | -0.33527 | -0.3225 | 0.18761 | -0.12798 | -0.52974 | 1.352134 | -0.87005 | -4.58354 | -2.71899 | -1.52541 | -1.80613 | -1.70401 | -2.84363 | -2.47254 | -0.93566 | -2.63819 | -1.86322 | -1.39096 | -1.8281 | -1.74797 |
| Q61458 | Q61458 | CCNH_MC | Ccnh | Cyclin-H | | | | 0.970386 | 0.970848 | 2.805196 | 0.347067 | 0.209022 | 0.932039 | 0.50862 | 0.40787 | 1.348107 | 1.092138 | 1.174682 | 0.491606 | 0.926708 | 1.078695 | 0.494574 | -0.18106 | 0.506181 | 0.445679 | 0.554592 | 0.29979 | 0.45774 |
| Q9QYY8 | Q9QYY8 | SPAST_MC | Spast | Spastin | + | + | | 3.458755 | 1.573946 | 53.61225 | 1.473652 | 1.98937 | 5.31694 | 0.614531 | -0.83779 | -1.6388 | 0.906042 | 0.8485 | 2.107282 | 1.849411 | -1.20954 | -0.22457 | -0.70252 | -0.69083 | -0.36226 | 0.340216 | -0.01641 | 0.257866 |
| O88874 | O88874 | CCNK_MC | Ccnk | Cyclin-K | | | | 0.370502 | 0.312805 | 0.991111 | 0.051376 | -0.07228 | -0.15877 | 0.471269 | -0.89291 | -1.24959 | 0.537373 | 1.131733 | 0.279269 | 1.126377 | -1.07962 | 0.349417 | 0.628022 | 0.415473 | 0.608271 | 0.941929 | 0.520092 | 0.535522 |
| Q8QZR8 | Q8QZR8 | CCNQ_MC | Ccnq | Cyclin-Q | + | | | 1.000389 | 1.082935 | 2.921516 | 0.260133 | -0.1829 | -0.71387 | 0.283114 | -0.32026 | -0.71158 | 0.217485 | 0.523361 | -0.34397 | -0.31502 | -0.56526 | -0.01891 | -0.37486 | -1.05017 | 0.109198 | -0.4024 | 0.340671 | -0.28433 |
| Q7TQK0 | Q7TQK0 | CCNT2_M | Ccnt2 | Cyclin-T2 | + | | | 1.12331 | 1.71416 | 3.435545 | 0.50476 | 1.086408 | 1.337841 | 0.036177 | -0.05489 | -0.11339 | -1.85683 | -1.14792 | -0.82164 | -0.68365 | -2.1125 | -1.31204 | -2.86538 | -3.56769 | -2.64818 | -1.02993 | -1.92964 | -1.38512 |
| Q8BGU5 | Q8BGU5 | CCNY_MC | Ccny | Cyclin-Y | | | | 0.151922 | 0.324292 | 0.436917 | 0.189435 | 0.333595 | 0.534439 | 0.156375 | -0.10749 | -0.44862 | -2.3408 | -1.54224 | -1.28321 | -1.53765 | -1.598 | -1.67817 | -2.89149 | -1.64013 | -2.35512 | -1.13293 | -1.76682 | -1.29437 |
| Q7TSH4 | Q7TSH4 | CP110_M | Ccp110 | Centriolar coiled-coil protein of 110 kDa | | | | 0.147549 | 0.490007 | 0.425391 | 0.21036 | -0.28312 | -0.588 | 0.769576 | -1.26281 | -2.10455 | -1.88107 | -3.71927 | -0.89127 | -0.94994 | -3.48497 | -3.58724 | -2.59583 | -3.98452 | -1.11809 | -0.15688 | -2.87115 | -1.67544 |
| Q8QZT2 | Q8QZT2 | CCSAP_M | Ccsap | Centriole, cilia and spindle-associated protein | | | | 0.016383 | -0.04993 | -0.05239 | 0.863466 | 0.321739 | 2.416434 | 0.470035 | -0.20676 | -1.24637 | -2.92591 | -1.22179 | 0.896035 | 1.16114 | 0.954911 | 0.768805 | -2.45082 | -1.59701 | 0.694291 | 0.719406 | 0.931284 | 1.205957 |
| Q3UHI0 | Q3UHI0 | CCSE2_M | Ccser2 | Serine-rich coiled-coil domain-containing protein 2 | | | | 0.993791 | 0.877303 | 2.895647 | 0.182414 | 0.204926 | 0.51635 | 0.32252 | -0.50774 | -0.87038 | -3.00327 | -2.60371 | -1.51641 | -0.75454 | -2.10255 | -1.83324 | -3.45302 | -3.90857 | -1.3195 | -1.39004 | -2.02775 | -0.89255 |
| P80314 | P80314 | TCPB_MO | Cct2 | T-complex protein 1 subunit beta | | | | 0.630622 | 0.534803 | 1.684694 | 0.829595 | 0.503502 | 2.300978 | 0.591549 | 0.225496 | 1.574098 | 1.554861 | 1.058816 | 2.531321 | 2.188944 | 1.839941 | 1.585773 | 0.970173 | 0.573898 | 1.720333 | 1.992928 | 1.421246 | 1.553477 |
| P80318 | P80318 | TCPG_MO | Cct3 | T-complex protein 1 subunit gamma | | + | | 0.76835 | 0.404431 | 2.100643 | 1.363061 | 0.387593 | 4.645619 | 0.485864 | 0.163852 | 1.287875 | 2.184057 | 1.822376 | 2.869836 | 2.706199 | 2.594615 | 2.340543 | 1.664842 | 1.53273 | 2.384099 | 2.416751 | 2.310692 | 2.296762 |
| P80315 | P80315 | TCPD_MO | Cct4 | T-complex protein 1 subunit delta | | | | 0.835601 | 0.396123 | 2.3212 | 0.929935 | 0.355435 | 2.65355 | 0.458435 | 0.232837 | 1.216133 | 2.272651 | 2.11349 | 3.188285 | 2.934279 | 2.767808 | 2.384902 | 1.94791 | 1.645984 | 2.748415 | 2.66328 | 2.342212 | 2.344824 |
| P80316 | P80316 | TCPE_MOI | Cct5 | T-complex protein 1 subunit epsilon | | + | | 0.396688 | 0.383981 | 1.057476 | 1.18594 | 0.353347 | 3.72302 | 0.435892 | 0.197858 | 1.157797 | 2.203433 | 1.867322 | 2.77569 | 2.733066 | 2.473454 | 2.152702 | 1.973277 | 1.329515 | 2.493515 | 2.308546 | 2.17424 | 2.056201 |
| P80317 | P80317 | TCPZ_MOI | Cct6a | T-complex protein 1 subunit zeta | | | | 0.917259 | 0.432331 | 2.607201 | 0.656304 | 0.351193 | 1.759019 | 0.132812 | 0.082477 | 0.386257 | 4.110214 | 3.796885 | 4.634666 | 4.410609 | 4.305059 | 3.902959 | 3.575562 | 3.466875 | 4.336705 | 4.006185 | 3.949603 | 4.093461 |
| P80313 | P80313 | TCPH_MO | Cct7 | T-complex protein 1 subunit eta | | + | | 0.90575 | 0.507899 | 2.565593 | 1.404082 | 0.356688 | 4.8853 | 0.542598 | 0.102146 | 1.439386 | 1.766741 | 1.430228 | 2.379142 | 2.28426 | 1.710112 | 1.69228 | 1.194893 | 0.986278 | 2.030512 | 1.919514 | 1.669453 | 1.528647 |
| P42932 | P42932 | TCPQ_MO | Cct8 | T-complex protein 1 subunit theta | | + | | 0.541136 | 0.540178 | 1.435425 | 1.036258 | 0.454858 | 3.065098 | 0.176033 | 0.02928 | 0.499849 | 2.070892 | 1.386907 | 2.885557 | 2.605855 | 1.854142 | 1.877649 | 1.345748 | 1.031695 | 2.340487 | 2.24121 | 1.894002 | 1.779229 |
| Q8C1Y8 | Q8C1Y8 | CCZ1_MO | Ccz1 | Vacuolar fusion protein CCZ1 homolog | | | | NaN | 0 | NaN | 0.175144 | 0.563612 | 0.497545 | 0.169886 | 0.388218 | 0.483897 | -3.05212 | -3.469 | -2.91713 | -2.36937 | -3.44467 | -2.15002 | -3.80519 | -3.21913 | -4.30604 | -2.10768 | -3.6595 | -2.71163 |
| O54901 | O54901 | OX2G_MO | Cd200 | OX-2 membrane glycoprotein | | | | 0.308422 | -0.26889 | -0.83503 | 0.436275 | -1.29906 | -1.15878 | 0.10694 | 0.21822 | 0.316298 | -1.07348 | -1.58572 | -0.26049 | -2.49797 | -0.64812 | -0.03495 | -0.86554 | -1.25589 | -0.15216 | -0.00818 | 0.058299 | -1.17781 |
| Q61735 | Q61735 | CD47_MO | Cd47 | Leukocyte surface antigen CD47 | | | | 0.467214 | -0.82355 | -1.239 | 0.62059 | -1.11729 | -1.65602 | 0.04672 | -0.09846 | -0.145 | -2.40432 | -1.29885 | -0.43306 | -1.77876 | -1.26089 | -0.26391 | -1.39722 | -0.65885 | -0.03836 | 0.061125 | -0.20279 | -1.12509 |
| P35762 | P35762 | CD81_MO | Cd81 | CD81 antigen | | | | 0.244726 | 0.880996 | 0.675078 | 0.493961 | 0.619916 | 1.30923 | 0.262632 | 0.558827 | 0.720155 | -1.10465 | -0.66538 | -1.12677 | -1.66433 | -1.59539 | -0.53043 | -3.05242 | -0.4796 | -2.40528 | -1.62565 | -1.05728 | -2.18619 |
| P56389 | P56389 | CDD_MOU | Cda | Cytidine deaminase | | | | 0.51834 | -1.12969 | -1.37405 | 0.726979 | -0.63585 | -1.97099 | 0.659365 | 1.195551 | 1.767969 | -0.27743 | -1.89189 | -3.13081 | -3.31166 | -1.89644 | -1.98732 | -0.11098 | 0.201037 | -2.89505 | -2.27571 | -2.46273 | -3.81213 |
| P62743 | P62743 | AP2S1_MC | Ap2s1 | AP-2 complex subunit sigma | + | | | 1.419615 | 1.573358 | 4.978863 | 0.489186 | 0.775939 | 1.296625 | 0.009534 | 0.01359 | 0.030714 | 5.393973 | 5.948867 | 7.352186 | 7.388016 | 5.778015 | 6.430229 | 3.946788 | 4.249336 | 5.996001 | 7.192323 | 5.79149 | 6.389575 |
| Q8BGZ4 | Q8BGZ4 | CDC23_M | Cdc23 | Cell division cycle protein 23 homolog | | | + | 0.127715 | -0.2706 | -0.37261 | 0.27009 | 0.546077 | 0.738892 | 1.51699 | 2.141993 | 5.602868 | -2.77294 | -3.98691 | -1.88775 | -0.89984 | -0.94858 | -1.48458 | -2.71057 | -3.50807 | -2.4896 | -1.39015 | -3.08594 | -3.63121 |
| Q99JP4 | Q99JP4 | CDC26_M | Cdc26 | Anaphase-promoting complex subunit CDC26 | | | | NaN | 0 | NaN | 0.319688 | 1.144238 | 0.863277 | 0.15543 | -0.39837 | -0.44614 | -3.50401 | -3.0167 | -2.53816 | -1.7734 | -2.89122 | -2.86202 | -3.25945 | -3.20971 | -4.56912 | -2.03091 | -3.37107 | -1.58543 |
| Q61081 | Q61081 | CDC37_M | Cdc37 | Hsp90 co-chaperone Cdc37 | + | | | 1.034871 | 0.914771 | 3.059452 | 0.10578 | -0.14068 | -0.31312 | 0.630928 | -0.87822 | -1.68557 | 2.307412 | 2.001998 | 3.217274 | 3.986313 | 1.86914 | 2.88177 | 0.982872 | 1.496995 | 3.510093 | 3.974852 | 3.130757 | 3.376587 |
| P60766 | P60766 | CDC42_M | Cdc42 | Cell division control protein 42 homolog | | | | 0.055422 | -0.06883 | -0.17066 | 0.100313 | -0.19939 | -0.29808 | 0.722133 | 0.341189 | 1.956089 | -0.24427 | 0.419112 | 0.598003 | -0.72469 | 0.152828 | 0.443985 | -0.0732 | 0.385697 | 0.035682 | 0.236414 | 0.053294 | -0.13886 |
| Q91W92 | Q91W92 | BORG5_M | Cdc42ep1 | Cdc42 effector protein 1 | | | | 0.347246 | 1.647247 | 0.932489 | 0.019548 | 0.034156 | 0.062305 | 0.360371 | -0.45866 | -0.96554 | -0.85564 | -0.33919 | -1.49572 | -0.48112 | -1.19567 | -1.68386 | -3.9922 | -0.49713 | -1.23035 | -0.8148 | -1.38863 | -0.57358 |
| Q8BGH7 | Q8BGH7 | C42S2_M | Cdc42se2 | CDC42 small effector protein 2 | | + | | 0.328616 | -0.34872 | -0.88568 | 1.641835 | 1.19067 | 6.507083 | 0.258749 | -0.24384 | -0.71039 | -3.56346 | -2.77657 | -2.53583 | -2.44533 | -3.31952 | -2.70303 | -2.83626 | -2.80634 | -3.85855 | -3.50395 | -2.91844 | -2.61642 |
| Q8BKE6 | Q8BKE6 | CP20A_M | Cyp20a1 | Cytochrome P450 20A1 | + | | | 1.476927 | 1.555861 | 5.338074 | 0.406841 | -3.05342 | -1.08333 | 0.32296 | -1.92566 | -0.87148 | 1.296671 | 1.725893 | -3.07677 | -3.94915 | -2.67919 | -3.48402 | 0.152637 | -0.24179 | 2.325042 | -3.24412 | 1.016743 | -3.32863 |
| P70408 | P70408 | CAD10_M | Cdh10 | Cadherin-10 | | | | 0.618608 | 1.006357 | 1.650379 | 0.858848 | 0.535964 | 2.400489 | 0.05636 | -0.10061 | -0.17341 | -3.16141 | -1.95574 | -1.06159 | -1.48482 | -1.40851 | -2.56626 | -3.65666 | -3.4732 | -1.88038 | -1.73797 | -1.92623 | -1.84731 |
| P55288 | P55288 | CAD11_M | Cdh11 | Cadherin-11 | | + | | NaN | 0 | NaN | 1.428657 | 0.46062 | 5.034057 | 0.033448 | 0.044453 | 0.105118 | -4.2326 | -4.28293 | -2.34607 | -2.36957 | -1.88899 | -2.61555 | -3.93528 | -3.53235 | -2.90919 | -2.7277 | -2.51319 | -2.08026 |
| Q9WTR5 | Q9WTR5 | CAD13_M | Cdh13 | Cadherin-13 | | | | 0.442877 | -0.88926 | -1.17582 | 0.357224 | 0.750715 | 0.957608 | 0.073702 | 0.132551 | 0.223478 | -4.11804 | -3.79014 | -1.39331 | -2.953 | -1.79519 | -2.90506 | -2.32652 | -3.80314 | -2.84374 | -3.004 | -2.69209 | -2.27327 |
| P15116 | P15116 | CADH2_M | Cdh2 | Cadherin-2 | | | | 0.434227 | 0.902143 | 1.153509 | 0.566882 | -0.09352 | -1.50571 | 0.073644 | 0.119364 | 0.223312 | -0.92489 | -1.13636 | -0.4922 | -0.38022 | -0.05018 | -1.05649 | -1.15786 | -2.70767 | -0.3158 | -0.36957 | -0.85309 | -0.49231 |
| Q9DB75 | Q9DB75 | CDIP1_M | Cdip1 | Cell death-inducing p53-target protein 1 | | | + | 0.129659 | -0.19942 | -0.37782 | 0.114986 | -0.08906 | -0.33824 | 1.126674 | 0.281885 | 3.450525 | 0.274586 | -0.57852 | -0.64586 | -0.37968 | 0.043112 | 0.116669 | -0.26344 | 0.358357 | -0.19654 | -0.65089 | -0.12905 | -0.27494 |
| P63280 | P63280 | UBC9_MO | Ube2i | SUMO-conjugating enzyme UBC9 | + | + | | 1.517383 | 1.547179 | 5.605521 | 2.394431 | 1.406289 | 15.69997 | 0.886468 | -0.80065 | -2.49686 | -1.37125 | -1.21093 | -0.40168 | -0.22373 | -2.13155 | -1.52125 | -3.10238 | -2.57416 | -1.72935 | -1.70864 | -0.92722 | -1.12428 |
| Q99PW4 | Q99PW4;( | PRPKB_M | Tp53rkb | EKC/KEOPS complex subunit Tp53rkb | + | | | 1.345783 | 1.545523 | 4.547795 | 0.148617 | -0.07873 | -0.42821 | 0.0114 | -0.01668 | -0.03665 | 1.654721 | 2.322464 | 0.885526 | 1.167177 | 1.458805 | 0.79798 | 0.506482 | 0.379655 | 1.223266 | 0.986888 | 0.832175 | 1.457968 |
| O35926 | O35926 | CD5R2_M | Cdk5r2 | Cyclin-dependent kinase 5 activator 2 | | | | 0.476234 | 1.052565 | 1.262586 | 0.296886 | -0.28135 | -0.80611 | 0.368084 | -0.18531 | -0.985 | -1.14424 | -1.28594 | -0.14813 | 0.012206 | -1.01042 | -1.37978 | -1.43702 | -3.0983 | -0.1263 | 0.553068 | -1.04565 | -0.97393 |
| P46414 | P46414 | CDN1B_M | Cdkn1b | Cyclin-dependent kinase inhibitor 1B | | | | 0.506734 | 1.241398 | 1.343088 | 0.031422 | 0.078871 | 0.098947 | 0.283366 | -0.33284 | -0.77221 | -3.5612 | -1.73287 | -2.70952 | -2.41648 | -2.38637 | -3.21853 | -3.75202 | -4.02485 | -1.85835 | -3.42539 | -2.58212 | -2.3571 |
| P98191 | P98191 | CDS1_MO | Cds1 | Phosphatide cytidylyltransferase 1 | | | + | 0.156073 | 0.245579 | 0.447823 | 0.19334 | -0.13365 | -0.54447 | 1.454591 | -0.96931 | -5.19541 | 0.929372 | 0.608987 | -0.41702 | -0.02751 | -1.06198 | -1.36452 | -0.00087 | 1.048067 | 0.060807 | -0.23804 | -0.35314 | -0.13473 |
| Q99L43 | Q99L43 | CDS2_MO | Cds2 | Phosphatide cytidylyltransferase 2 | + | | | 1.216478 | 1.134239 | 3.870075 | 0.438173 | 0.552476 | 1.163675 | 0.400271 | 0.535912 | 1.066592 | 0.766346 | 0.535068 | -0.57176 | -1.05132 | 0.331429 | -0.66421 | -0.75283 | -0.21423 | -1.77379 | -0.95425 | -0.77038 | -0.63422 |
| Q7TPC1 | Q7TPC1 | CDSN_MO | Cdsn | Corneodesmosin | | | | 0.327823 | 0.600956 | 0.883687 | 0.228902 | -0.37649 | -0.63509 | 0.155833 | 0.157659 | 0.447193 | 2.453596 | 1.356689 | 0.741611 | 0.171121 | 0.539044 | 1.124656 | 1.706273 | 0.902099 | 0.313186 | 1.352516 | 0.477828 | 0.870554 |
| D3Z1D3 | D3Z1D3 | CEFIP_MO | CEFIP | Cardiac-enriched FHL2-interacting protein | | | + | NaN | 0 | NaN | 0.410439 | 6.827441 | 1.092518 | 3.457673 | 12.58323 | 53.54548 | -3.26665 | -3.72298 | 8.800285 | 9.395189 | 8.374039 | 8.26848 | -2.92651 | -3.04503 | 8.512482 | -3.97189 | -4.49097 | -4.03297 |
| Q9Z0H4 | Q9Z0H4 | CELF2_MC | Celf2 | CUGBP Elav-like family member 2 | | | | 0.830013 | 1.419548 | 2.302383 | 0.842129 | 0.414915 | 2.343299 | 0.337394 | -0.18041 | -0.90772 | -2.65627 | -1.54416 | -0.33346 | -0.54834 | -0.38462 | -0.70377 | -3.25341 | -3.78612 | -0.99656 | -0.71507 | -0.48227 | -0.24531 |
| Q7TSY6 | Q7TSY6;A( | CELF4_MC | Celf4 | CUGBP Elav-like family member 4 | | | | 0.23255 | 0.358973 | 0.644327 | 0.428114 | 0.21999 | 1.137787 | 0.028455 | 0.034763 | 0.08987 | -2.04928 | -1.86009 | 0.508064 | 0.207857 | 0.033277 | -0.44878 | -1.76462 | -2.8627 | 0.016099 | 0.259842 | -0.54505 | 0.060021 |
| Q9JKC6 | Q9JKC6 | CEND_MO | Cend1 | Cell cycle exit and neuronal differentiation protein 1 | + | | + | 1.210513 | -0.36822 | -3.84099 | 0.036688 | -0.08083 | -0.11494 | 1.064092 | 0.442095 | 3.180059 | 0.730666 | 0.845449 | 0.258687 | -0.99628 | 0.156681 | 0.264071 | 1.079487 | 1.233062 | 0.029459 | -0.6054 | -0.35995 | -0.10349 |
| Q9CXS4 | Q9CXS4 | CENPV_M | Cenpv | Centromere protein V | | | | 0.520155 | 1.123253 | 1.378906 | 0.580877 | 1.044043 | 1.544382 | 0.054924 | 0.073859 | 0.169202 | -0.39207 | -1.08556 | -0.48444 | 0.753744 | -0.03907 | -0.69477 | -1.12496 | -2.59918 | -0.63785 | -1.18093 | -0.72898 | -0.15257 |
| Q6A065 | Q6A065 | CE170_M | Cep170 | Centrosomal protein of 170 kDa | | | | 0.830671 | 0.551725 | 2.304592 | 0.311087 | -0.15586 | -0.84171 | 0.439952 | -0.56464 | -1.16827 | 0.237472 | 0.284711 | 1.478591 | 1.564214 | 0.476027 | 1.342884 | -0.0524 | -0.52887 | 1.497108 | 1.857421 | 1.260245 | 1.687939 |
| Q80U49 | Q80U49 | C170B_M | Cep170b | Centrosomal protein of 170 kDa protein B | | | | 0.246851 | -0.57577 | -0.68043 | 0.120513 | 0.022598 | 0.353217 | 0.37456 | -0.59436 | -1.00137 | 0.355219 | 0.135441 | 2.043082 | 2.104399 | 0.479302 | 1.358503 | -0.01791 | 1.660112 | 1.994991 | 2.107295 | 1.114449 | 1.912083 |
| Q9CZ62 | Q9CZ62 | CEP97_M | Cep97 | Centrosomal protein of 97 kDa | | | | 0.223788 | -0.83877 | -0.62213 | 0.407332 | -0.25545 | -1.08459 | 0.487592 | -1.45829 | -1.29243 | -3.98223 | -1.45048 | -0.06173 | -0.47503 | -3.11187 | -1.04549 | -1.41364 | -2.34153 | -0.12593 | 0.100055 | -1.07389 | -0.16689 |
| Q64516 | Q64516 | GLPK_MOI | Gk | Glycerol kinase | + | | | 3.361495 | 1.530898 | 47.92982 | 0.872737 | 0.688391 | 2.448649 | 0.120188 | 0.229683 | 0.352338 | 2.624888 | 2.564991 | 1.599275 | 2.157804 | 2.035644 | 0.875834 | 1.075144 | 1.052939 | 1.157805 | 1.222492 | 0.928292 | 1.523819 |

| | | | | | | | | | | | | | | | | | | | | | | | | | | | | |
|---|---|---|---|---|---|---|---|---|---|---|---|---|---|---|---|---|---|---|---|---|---|---|---|---|---|---|---|---|
| Q9R1K9 | Q9R1K9 | CETN2_MC | Cetn2 | Centrin-2 | | | | 0.201155 | -1.11381 | -0.5645 | 0.250893 | 0.129004 | 0.69062 | 0.383057 | -0.45438 | -1.02287 | -0.54007 | -4.4613 | 0.249937 | 0.55136 | -0.43524 | 0.250812 | -1.60839 | -1.16536 | 0.161291 | 0.381999 | 0.079917 | 0.644424 |
| O08967 | O08967 | CYH3_MO | Cyth3 | Cytohesin-3 | + | | | 2.661799 | 1.510896 | 21.38893 | 0.1089 | -0.14447 | -0.32166 | 0.767653 | -0.91591 | -2.09842 | -1.71089 | -1.6304 | 0.292237 | 0.684619 | -0.20299 | 0.340501 | -3.12349 | -3.2396 | 0.228885 | 1.036905 | 0.643101 | 1.326222 |
| Q3UJP5 | Q3UJP5 | CF418_MC | Cfap418 | Cilia- and flagella-associated protein 418 | | | | 0.104381 | 0.233436 | 0.30928 | 0.572884 | 0.988 | 1.522255 | 0.136314 | -0.15826 | -0.3956 | -3.72102 | -2.25981 | -1.16765 | -1.229 | -1.60954 | -1.54954 | -3.03439 | -3.41332 | -2.83464 | -1.53801 | -1.02237 | -1.8202 |
| P18760 | P18760;P | COF1_MO | Cfl1 | Cofilin-1 | | | | 0.906639 | -0.33163 | -2.56879 | 0.676933 | -0.657 | -1.81973 | 0.011084 | 0.020235 | 0.035647 | 3.34758 | 3.593798 | 3.600531 | 2.904501 | 3.900124 | 4.781184 | 3.841185 | 3.763449 | 3.813408 | 4.005616 | 4.678419 | 3.962418 |
| P45591 | P45591 | COF2_MO | Cfl2 | Cofilin-2 | + | | | 1.014816 | -2.4767 | -2.97866 | 0.154036 | -0.17599 | -0.44248 | 0.055377 | -0.17703 | -0.17053 | -3.48526 | -1.95766 | -1.17481 | -1.9484 | -2.08448 | -0.49861 | 0.083828 | -0.57335 | -1.47826 | -1.29297 | -0.44451 | -1.78453 |
| Q91VZ6 | Q91VZ6 | SMAP1_M | Smap1 | Stromal membrane-associated protein 1 | + | + | | 1.644712 | 1.500807 | 6.529435 | 1.26196 | -0.76754 | -4.09797 | 0.306654 | -0.1323 | -0.83059 | 0.44141 | 0.299281 | 0.538662 | 0.478566 | 1.694702 | 1.377188 | -0.91187 | -1.34905 | 1.091283 | 1.461027 | 1.655347 | 1.681137 |
| Q8VEA4 | Q8VEA4 | MIA40_MC | Chchd4 | Mitochondrial intermembrane space import and assembly protein 40 | | | | NaN | 0 | NaN | 0.361976 | 1.698573 | 0.969589 | 0.340917 | 0.887241 | 0.916574 | -3.36918 | -3.25014 | -0.82025 | 0.406221 | -0.4704 | 0.249095 | -3.6698 | -3.83609 | -3.5466 | -0.26458 | -0.09923 | -1.89656 |
| Q91VN4 | Q91VN4 | MIC25_MC | Chchd6 | MICOS complex subunit Mic25 | + | | | 1.075259 | 0.766021 | 3.22708 | 0.607259 | 0.198858 | 1.618218 | 0.650121 | 0.273472 | 1.741001 | 1.690856 | 1.735717 | 1.188248 | 1.430218 | 1.616632 | 1.30263 | 1.183576 | 0.710954 | 1.088834 | 1.131915 | 1.181265 | 1.191053 |
| B1AR17 | B1AR17 | B1AR17_M | Chd3 | DNA helicase | | | + | NaN | 0 | NaN | 0.399096 | 0.220361 | 1.063602 | 1.789416 | -0.69952 | -7.75118 | -3.6922 | -3.55661 | -1.02872 | -0.62041 | -1.4577 | -1.27859 | -3.108 | -3.59982 | -1.0802 | -1.00965 | -0.65747 | -0.67978 |
| Q6PDQ2 | Q6PDQ2 | CHD4_MO | Chd4 | Chromodomain-helicase-DNA-binding protein 4 | + | | | 1.014404 | 0.84408 | 2.977021 | 0.027501 | 0.028594 | 0.08694 | 0.517816 | -0.29786 | -1.37264 | 0.458267 | 0.963102 | 0.90783 | 1.565572 | 0.518332 | 0.905668 | -0.26253 | -0.00426 | 1.212198 | 1.204015 | 0.91198 | 1.10774 |
| A2A8L1 | A2A8L1 | CHD5_MO | Chd5 | Chromodomain-helicase-DNA-binding protein 5 | | | | 0.171556 | 0.518856 | 0.488237 | 0.194684 | -0.22542 | -0.54792 | 0.807568 | -0.51002 | -2.22771 | -1.07714 | -2.74462 | -1.34949 | -0.53019 | -1.39915 | -1.02191 | -1.77076 | -3.08871 | -0.67639 | -0.75245 | -0.57075 | -0.83027 |
| P16014 | P16014 | SCG1_MO | Chgb | Secretogranin-1 | | + | | NaN | 0 | NaN | 1.371583 | 1.427679 | 4.694547 | 0.328103 | -1.12154 | -0.88439 | -3.24806 | -3.31084 | -1.24905 | -1.22401 | -3.53332 | -1.00554 | -4.02858 | -2.67729 | -2.36035 | -2.96807 | -1.25175 | -1.04403 |
| Q9D1P4 | Q9D1P4 | CHRD1_M | Chordc1 | Cysteine and histidine-rich domain-containing protein 1 | | | | NaN | 0 | NaN | 0.465353 | 0.669029 | 1.234146 | 0.78398 | 1.08234 | 2.150782 | -3.94841 | -3.78557 | -2.62866 | -2.89398 | -1.20545 | -1.89542 | -3.193 | -3.00685 | -3.95597 | -2.90474 | -2.2664 | -2.99915 |
| P61022 | P61022 | CHP1_MO | Chp1 | Calcineurin B homologous protein 1 | | | | 0.254614 | 0.297692 | 0.699989 | 0.694971 | 0.742135 | 1.873579 | 0.308299 | -0.2857 | -0.83472 | -0.78156 | -0.35273 | -0.17619 | -0.31711 | -0.99632 | -1.55929 | -0.49756 | -1.23211 | -1.37857 | -0.599 | -1.18682 | -0.7974 |
| Q8C025 | Q8C025 | CHPT1_MC | Chpt1 | Cholinephosphotransferase 1 | | | | 0.197788 | 0.260027 | 0.555879 | 0.368121 | -0.93593 | -0.9851 | 0.005158 | 0.008186 | 0.016697 | 0.685011 | 1.619585 | -1.26267 | -3.14585 | -0.48613 | -1.42592 | 0.870893 | 0.91365 | -1.14152 | -1.39514 | -1.10396 | -0.82446 |
| P12657 | P12657 | ACM1_MO | Chrm1 | Muscarinic acetylcholine receptor M1 | | | | NaN | 0 | NaN | 0.311437 | 0.334379 | 0.842585 | 0.366668 | 0.588672 | 0.981427 | -3.65199 | -2.71457 | -0.6706 | -0.56881 | 0.38987 | -0.78689 | -3.32409 | -3.34793 | -0.56051 | -1.34766 | -0.90374 | -0.67062 |
| Q9ERZ3 | Q9ERZ3 | ACM3_MO | Chrm3 | Muscarinic acetylcholine receptor M3 | | | | 0.067047 | 0.140926 | 0.204413 | 0.198458 | -0.37724 | -0.55759 | 0.170482 | 0.319333 | 0.485447 | -2.48937 | -3.84418 | -1.8621 | -1.57829 | -1.05786 | -2.37179 | -3.17958 | -3.43582 | -0.68144 | -2.00446 | -2.00082 | -2.0675 |
| Q8WTY4 | Q8WTY4 | CPIN1_MC | Ciapin1 | Anamorsin | | | | 0.318676 | -0.62373 | -0.86074 | 0.776736 | 0.597862 | 2.127463 | 0.560442 | 0.559772 | 1.488031 | -4.3098 | -2.87614 | -2.84287 | -2.56003 | -2.57902 | -1.90266 | -2.86306 | -3.07542 | -3.05647 | -3.54216 | -2.63584 | -2.96538 |
| Q9JKV2 | Q9JKV2 | CILK1_MO | Cilk1 | Serine/threonine-protein kinase ICK | | | | 0.778264 | 0.840811 | 2.132371 | 0.731227 | 0.959675 | 1.984108 | 0.346629 | 0.144593 | 0.930935 | 0.497341 | 0.624927 | -0.53759 | 0.314063 | -0.2424 | -0.35104 | 0.109436 | -0.66879 | -0.84205 | -1.30083 | -0.58683 | -0.2958 |
| P70213 | P70213 | FV1_MOUS | Fv1 | Friend virus susceptibility protein 1 | + | | | 1.599103 | 1.485988 | 6.183441 | 0.299041 | 0.465318 | 0.81151 | 0.054755 | -0.11728 | -0.16871 | -0.23871 | 0.238842 | -0.70375 | 0.273242 | -1.51892 | -1.3021 | -1.45874 | -1.51311 | -0.98083 | -0.38031 | -1.97991 | -0.60653 |
| Q91WS0 | Q91WS0 | CISD1_MC | Cisd1 | CDGSH iron-sulfur domain-containing protein 1 | | | | 0.057962 | 0.04663 | 0.178086 | 0.14021 | 0.258265 | 0.405961 | 0.045685 | 0.114666 | 0.141917 | 0.492963 | 0.484894 | 0.876558 | -0.29065 | 0.505662 | 1.273784 | 0.704105 | 0.180493 | 0.287929 | -0.21855 | 1.485925 | 0.064189 |
| B1AR13 | B1AR13 | CISD3_MC | Cisd3 | CDGSH iron-sulfur domain-containing protein 3, mitochondrial | | | | 0.159385 | 0.127653 | 0.456504 | 0.343985 | 0.353347 | 0.924285 | 0.273344 | 0.246002 | 0.747062 | 0.913677 | 0.413582 | 0.333968 | 0.929024 | 0.370325 | 0.305924 | 0.66116 | 0.410793 | 0.518199 | 0.038099 | -0.23559 | 0.419837 |
| A2AGT5 | A2AGT5 | CKAP5_MC | Ckap5 | Cytoskeleton-associated protein 5 | | | | 0.387574 | 0.207168 | 1.034326 | 0.857567 | -0.287 | -2.39608 | 0.324382 | 0.105847 | 0.875053 | -0.80698 | -0.574 | -0.54034 | -0.55959 | -0.41983 | -0.63066 | -0.73472 | -1.06059 | -0.38236 | -0.14358 | -0.57178 | -0.69041 |
| Q04447 | Q04447 | KCRB_MOI | Ckb | Creatine kinase B-type | | | | 0.594282 | -0.95609 | -1.58174 | 0.029978 | 0.112776 | 0.094537 | 0.023759 | -0.0537 | -0.0754 | 2.651614 | 3.783747 | 4.86196 | 2.49978 | 2.901325 | 3.79877 | 4.385734 | 3.961799 | 3.400479 | 3.735709 | 3.95682 | 2.850671 |
| P30275 | P30275 | KCRU_MO | Ckmt1 | Creatine kinase U-type, mitochondrial | + | | | 1.259292 | -1.64432 | -4.0843 | 0.042 | -0.13404 | -0.13092 | 0.041684 | -0.1342 | -0.12997 | 1.595466 | 2.387483 | 3.186707 | 1.139297 | 2.071369 | 3.148039 | 3.70832 | 3.563267 | 2.278837 | 2.315256 | 3.62498 | 1.862829 |
| Q80TV8 | Q80TV8 | CLAP1_MC | Clasp1 | CLIP-associating protein 1 | | | | 0.178533 | 0.234297 | 0.50632 | 0.050771 | 0.018262 | 0.156987 | 0.485208 | -0.42541 | -1.28615 | 0.0377 | -0.34917 | 0.499541 | 0.657167 | -0.14632 | -0.06676 | 0.03034 | -0.81041 | 0.474535 | 0.64565 | -0.00949 | 0.647232 |
| Q8BRT1 | Q8BRT1 | CLAP2_MC | Clasp2 | CLIP-associating protein 2 | | | | 0.720176 | 0.618534 | 1.950086 | 0.529929 | 0.328567 | 1.405152 | 0.3686 | -0.59416 | -0.98631 | 1.29072 | 1.245153 | 2.916738 | 3.238183 | 1.755767 | 2.289488 | 0.965766 | 0.333039 | 2.579055 | 2.918731 | 2.076712 | 3.156865 |
| Q9R0A1 | Q9R0A1 | CLCN2_MC | Clcn2 | Chloride channel protein 2 | | | | 0.311087 | -1.24151 | -0.84171 | 0.484628 | -1.02343 | -1.28462 | 0.017232 | -0.09569 | -0.05506 | -3.50587 | -0.56057 | -3.83674 | -3.43674 | -1.06072 | -3.39597 | -0.70863 | -0.87478 | -3.38447 | -1.84214 | -0.84516 | -3.42014 |
| Q9QYP6 | Q9QYP6 | AZI2_MOU | Azi2 | 5-azacytidine-induced protein 2 | + | + | | 2.763254 | 1.48431 | 24.04721 | 1.148057 | 0.869789 | 3.546937 | 0.319846 | -0.80232 | -0.86367 | 5.007951 | 5.048952 | 5.365695 | 5.848173 | 2.795365 | 4.581052 | 3.602363 | 3.48592 | 4.693122 | 4.781168 | 4.234015 | 4.74704 |
| Q80U30 | Q80U30 | CL16A_MC | Clec16a | Protein CLEC16A | | | | NaN | 0 | NaN | 0.274977 | 0.077358 | 0.751161 | 0.084253 | -0.10083 | -0.25336 | -3.82593 | -4.03777 | -0.76215 | -0.66829 | -1.2808 | -1.80997 | -4.0819 | -3.10969 | -0.88425 | -0.7009 | -1.74184 | -1.14727 |
| Q9QYB1 | Q9QYB1 | CLIC4_MC | Clic4 | Chloride intracellular channel protein 4 | | | | 0.857457 | -0.95862 | -2.3957 | 0.0159 | 0.041341 | 0.050872 | 0.178556 | 0.283028 | 0.50638 | -0.46606 | -1.18212 | -1.2384 | -2.6697 | -1.45783 | -0.66257 | -0.04416 | 0.313221 | -2.38041 | -1.61037 | -0.95043 | -1.73602 |
| Q99KN9 | Q99KN9 | EPN4_MOI | Clint1 | Clathrin interactor 1 | | | | NaN | 0 | NaN | 0.532617 | 0.433947 | 1.412395 | 0.102993 | 0.110027 | 0.305464 | -3.9889 | -2.78457 | -1.81102 | -1.30101 | -1.74853 | -1.88171 | -3.82186 | -2.99196 | -2.16134 | -1.81858 | -1.57116 | -2.27913 |
| Q922J3 | Q922J3 | CLIP1_MO | Clip1 | CAP-Gly domain-containing linker protein 1 | | | | 0.489344 | -2.69007 | -1.29704 | 0.075541 | -0.85276 | -0.22872 | 0.021765 | -0.25206 | -0.06921 | -2.66699 | 1.420991 | -4.3813 | 1.433791 | -3.67737 | 1.629192 | 2.418595 | 1.715538 | 1.713059 | -2.95504 | 1.722703 | -3.26676 |
| Q9Z0H8 | Q9Z0H8 | CLIP2_MO | Clip2 | CAP-Gly domain-containing linker protein 2 | | | | 0.956451 | 0.448861 | 2.752296 | 0.060611 | -0.05216 | -0.1858 | 0.289971 | -0.25403 | -0.78877 | -0.45578 | -0.15493 | 0.207493 | 0.27644 | -0.32083 | -0.39353 | -0.69121 | -0.81723 | 0.015513 | 0.572742 | -0.42315 | 0.216858 |
| B9EHT4 | B9EHT4 | CLIP3_MO | Clip3 | CAP-Gly domain-containing linker protein 3 | | | | 0.194412 | -0.64118 | -0.54722 | 0.337008 | 0.422717 | 0.906751 | 0.042601 | -0.05236 | -0.13272 | -3.15162 | -1.82297 | -1.62239 | -0.82502 | -1.72351 | -2.37763 | -2.81128 | -0.88094 | -1.88804 | -1.4048 | -2.21884 | -1.77759 |
| Q8C5W0 | Q8C5W0 | CLMN_MO | Clmn | Calmin | | | | NaN | 0 | NaN | 0.567095 | 0.771742 | 1.5063 | 0.039493 | -0.08178 | -0.1234 | -2.62199 | -3.08413 | -3.27511 | -3.3955 | -2.58613 | -3.72859 | -3.4612 | -3.49737 | -3.59825 | -4.61584 | -3.41156 | -2.73961 |
| Q8VBZ3 | Q8VBZ3 | CLPT1_MC | Clptm1 | Putative lipid scramblase CLPTM1 | | | | 0.757561 | 0.638651 | 2.066409 | 0.809673 | 0.22172 | 2.234657 | 0.2725 | -0.18493 | -0.74494 | -1.05468 | -1.37605 | -1.45719 | -1.65153 | -0.88172 | -1.37818 | -2.11802 | -1.59001 | -1.79615 | -1.75602 | -0.94768 | -0.94236 |
| Q9JHS4 | Q9JHS4 | CLPX_MOI | Clpx | ATP-dependent Clp protease ATP-binding subunit clpX-like, mitochondrial | + | | | 1.115028 | 1.668463 | 3.398889 | 0.565269 | 1.439941 | 1.501279 | 0.405204 | 0.682284 | 1.07916 | -1.01424 | -1.50222 | -1.80306 | -1.9326 | -1.14509 | -2.14959 | -2.50074 | -3.35265 | -2.35082 | -4.26472 | -2.71364 | -1.9456 |
| Q9EPL2 | Q9EPL2 | CSTN1_MC | Clstn1 | Calsyntenin-1 | | | | 0.150675 | 0.245581 | 0.433633 | 0.674355 | 0.671361 | 1.812089 | 0.511995 | 0.85466 | 1.357099 | -3.06995 | -1.97366 | -1.46663 | -1.23267 | -0.9556 | -1.32063 | -2.62501 | -2.90977 | -2.37255 | -1.66947 | -2.59551 | -1.39003 |
| Q99JH7 | Q99JH7 | CSTN3_MC | Clstn3 | Calsyntenin-3 | | | | NaN | 0 | NaN | 0.055071 | -0.1464 | -0.16963 | 0.130813 | 0.231913 | 0.380911 | -3.36821 | -3.2553 | -2.11506 | -3.07813 | -2.8265 | -3.53613 | -3.46318 | -2.84498 | -3.16638 | -1.73401 | -2.91846 | -3.90799 |
| O08585 | O08585 | CLCA_MO | Clta | Clathrin light chain A | | | | 0.009391 | 0.005238 | 0.03026 | 0.338653 | 0.133577 | 0.910884 | 0.013159 | 0.007146 | 0.042225 | 2.000695 | 2.227719 | 2.919651 | 2.75995 | 2.37954 | 2.593954 | 2.23967 | 1.978267 | 2.829222 | 2.583225 | 2.610538 | 2.348665 |
| Q6IRU5 | Q6IRU5 | CLCB_MO | Cltb | Clathrin light chain B | | | | 0.648256 | 0.340392 | 1.735581 | 0.771612 | 0.388775 | 2.111052 | 0.130983 | -0.09699 | -0.38137 | 1.424273 | 1.555438 | 2.237714 | 2.122817 | 1.118539 | 1.606989 | 1.334299 | 0.964628 | 1.966463 | 1.616518 | 1.38881 | 1.530697 |
| Q68FD5 | Q68FD5 | CLH1_MOI | Cltc | Clathrin heavy chain 1 | | + | | 0.773186 | 0.564903 | 2.116084 | 1.256602 | 0.427521 | 4.070555 | 0.730997 | 0.280214 | 1.983396 | 2.967657 | 3.097991 | 3.940763 | 3.798223 | 3.537607 | 3.30315 | 2.726801 | 2.209039 | 3.364827 | 3.519118 | 3.061314 | 3.219015 |
| Q6A068 | A0A1W2P( | CDC5L_M( | Cdc5l | Cell division cycle 5-like protein | + | | | 1.468137 | 1.483681 | 5.281515 | 0.290434 | 0.180061 | 0.789933 | 0.305854 | 0.622319 | 0.828589 | 1.017677 | 1.556667 | 0.71759 | 1.117695 | 2.151696 | 0.751205 | -0.27581 | -0.11721 | 0.628321 | 0.846841 | 0.55757 | 1.100693 |
| Q5SW19 | Q5SW19 | CLU_MOU | Cluh | Clustered mitochondria protein homolog | | | | 0.171654 | 0.411505 | 0.488491 | 0.402459 | 0.399247 | 1.072164 | 0.069149 | -0.12763 | -0.21045 | -1.32228 | -2.57482 | -1.99395 | -1.63918 | -2.41187 | -1.24542 | -1.79666 | -2.92346 | -2.54322 | -1.8884 | -1.86733 | -1.5347 |
| Q8R4N0 | Q8R4N0 | CLYBL_MC | Clybl | Citramalyl-CoA lyase, mitochondrial | + | | | 1.048286 | -0.53191 | -3.11439 | 0.017118 | 0.058645 | 0.0547 | 0.411096 | 0.457244 | 1.094195 | -1.72605 | -1.68417 | -1.91007 | -3.69138 | -1.85044 | -1.01575 | -1.0037 | -1.34271 | -2.26258 | -3.45616 | -1.86916 | -1.91152 |
| Q99KK2 | Q99KK2 | NEUA_MO | Cmas | N-acylneuraminate cytidylyltransferase | | + | | 0.58268 | -0.40776 | -1.54939 | 1.406382 | -0.75432 | -4.89905 | 0.203238 | -0.3511 | -0.56982 | 1.374341 | 1.081214 | 2.265252 | 2.52415 | 1.987521 | 3.03091 | 1.854126 | 1.416952 | 3.065651 | 3.232391 | 3.188157 | 2.532472 |
| Q9D486 | Q9D486 | CMIP_MOI | Cmip | C-Maf-inducing protein | | | | NaN | 0 | NaN | 0.004211 | 0.017239 | 0.013647 | 0.650971 | 1.045074 | 1.743473 | -3.11207 | -3.27912 | -3.28839 | -3.28883 | -2.01314 | -2.88578 | -3.13988 | -2.8893 | -4.56912 | -2.04258 | -3.08352 | -3.90554 |
| Q6WQJ1 | Q6WQJ1 | DGLA_MO | Dagla | Diacylglycerol lipase-alpha | + | | | 1.887396 | 1.458865 | 8.698498 | 0.801333 | -0.3352 | -2.20723 | 0.359926 | -0.16897 | -0.96442 | -0.51185 | -0.81901 | -0.68084 | -0.54057 | 0.022608 | -0.30744 | -2.19169 | -2.0569 | -0.41021 | -0.1408 | 0.085423 | -0.03231 |
| Q3U5Q7 | Q3U5Q7 | CMPK2_M | Cmpk2 | UMP-CMP kinase 2, mitochondrial | | | | 0.030193 | -0.05231 | -0.09519 | 0.435594 | -0.17763 | -1.15703 | 0.074766 | 0.051517 | 0.22651 | -0.99989 | -0.71979 | -1.03995 | -1.07102 | -1.01449 | -0.70245 | -0.27619 | -1.33888 | -0.72512 | -1.03059 | -0.74451 | -1.07547 |
| Q9D6G9 | Q9D6G9 | CKLF5_MC | Cmtm5 | CKLF-like MARVEL transmembrane domain-containing protein 5 | | | | 0.401478 | -0.22885 | -1.06967 | 0.212881 | 0.242296 | 0.594419 | 0.692964 | -0.17325 | -1.86755 | 1.71747 | 2.107428 | 0.344366 | -0.17642 | 0.510041 | 0.676206 | 2.229375 | 2.053229 | -0.47193 | 0.155281 | 0.807646 | 0.725102 |
| Q9D1A2 | Q9D1A2 | CNDP2_M | Cndp2 | Cytosolic non-specific dipeptidase | | | | 0.972737 | -0.59211 | -2.81419 | 0.159333 | 0.18814 | 0.456368 | 0.049238 | -0.06633 | -0.15246 | -0.87853 | -1.20803 | -0.48243 | -1.29496 | -0.93589 | -0.3574 | -0.58204 | -0.3203 | -1.14688 | -1.0068 | -0.25535 | -0.90528 |
| O35089 | O35089 | CNIH2_MC | Cnih2 | Protein cornichon homolog 2 | | | | NaN | 0 | NaN | 0.655212 | -1.19679 | -1.75583 | 0.533911 | -0.34094 | -1.41589 | -3.99056 | -3.39624 | -2.53126 | -3.42908 | 0.50257 | 0.436136 | -2.73443 | -2.86577 | -1.27047 | -2.29628 | 1.048783 | 0.571798 |
| Q8K245 | Q8K245 | UVRAG_M | Uvrag | UV radiation resistance-associated protein | + | | | 2.22476 | 1.451536 | 12.89532 | 0.749694 | 0.611029 | 2.041639 | 0.045991 | -0.05313 | -0.14283 | 1.235437 | 1.456269 | 1.709408 | 2.298054 | 0.670579 | 0.731487 | -0.12756 | -0.0838 | 1.446968 | 1.338437 | 0.38345 | 1.124869 |
| Q08093 | Q08093 | CNN2_MO | Cnn2 | Calponin-2 | | | + | 0.032602 | 0.076103 | 0.102545 | 0.854482 | -0.69702 | -2.38547 | 3.063508 | 1.218087 | 33.9995 | -2.1268 | -3.50682 | -2.2409 | -1.84002 | -1.9988 | -2.06683 | -3.16616 | -2.61967 | -1.13083 | -1.55604 | -3.23965 | -3.26216 |
| Q9DAW9 | Q9DAW9;( | CNN3_MO | Cnn3 | Calponin-3 | | | | 0.986956 | 0.365147 | 2.869019 | 0.291973 | 0.166987 | 0.793793 | 0.49556 | 0.216836 | 1.313455 | -0.15535 | 0.072263 | 0.582905 | 0.589239 | 0.854269 | 0.543744 | -0.46367 | -0.34972 | 0.208743 | 0.629427 | 0.426067 | 0.538274 |
| Q0GA42 | Q0GA42;Q | CNNM1_M | Cnnm1 | Metal transporter CNNM1 | | | | 0.33119 | 0.630595 | 0.892139 | 0.036317 | -0.01149 | -0.11382 | 0.633928 | 0.401783 | 1.694186 | -2.00335 | -1.66712 | -1.07602 | -1.16856 | -1.03314 | -1.47161 | -1.77927 | -3.15238 | -1.20049 | -1.02112 | -1.56372 | -1.74459 |
| Q6ZQ08 | Q6ZQ08 | CNOT1_M | Cnot1 | CCR4-NOT transcription complex subunit 1 | + | | | 1.118535 | 0.702786 | 3.414374 | 0.328282 | -0.22282 | -0.88484 | 0.893538 | -0.74224 | -2.52192 | -1.2532 | -1.3986 | -0.80627 | -0.31315 | -1.54967 | -1.10255 | -1.83612 | -2.22125 | -0.38806 | -0.28573 | -0.77529 | -0.39244 |
| Q8C5L3 | Q8C5L3 | CNOT2_M | Cnot2 | CCR4-NOT transcription complex subunit 2 | | | | 0.593023 | 0.691393 | 1.578218 | 0.265021 | -0.40255 | -0.72616 | 0.434564 | -0.31712 | -1.15438 | -1.95615 | -2.80542 | -1.56047 | -1.43077 | -1.82246 | -1.84273 | -3.1799 | -2.96445 | -0.54253 | -1.64362 | -1.24094 | -1.79 |
| Q60809 | Q60809 | CNOT7_M | Cnot7 | CCR4-NOT transcription complex subunit 7 | | | | 0.077818 | -0.10648 | -0.23518 | 0.298237 | 0.199342 | 0.809496 | 0.512743 | -0.46565 | -1.35909 | -3.98995 | -3.09327 | -2.53024 | -2.14572 | -3.00396 | -2.32856 | -3.37206 | -3.4982 | -2.38344 | -2.6912 | -2.25844 | -2.14277 |
| Q9JKY0 | Q9JKY0 | CNOT9_M | Cnot9 | CCR4-NOT transcription complex subunit 9 | | | + | 0.254919 | 0.787905 | 0.700756 | 0.914607 | -0.37777 | -2.59757 | 1.946007 | -1.02823 | -9.31731 | -1.07637 | -1.27844 | -0.82297 | -0.7258 | -1.99005 | -1.77756 | -0.8455 | -3.08513 | -0.53369 | -0.25954 | -0.88542 | -0.82574 |
| P16330 | P16330 | CN37_MO | Cnp | 2',3'-cyclic-nucleotide 3'-phosphodiesterase | | + | | 0.547575 | -0.33361 | -1.4529 | 1.04204 | 0.46119 | 3.08872 | 0.948917 | 0.176461 | 2.723988 | 5.666789 | 5.506528 | 4.75524 | 5.050958 | 4.699241 | 4.820398 | 6.135445 | 5.705087 | 4.421113 | 4.462705 | 4.606309 | 4.560408 |
| B2RUJ5 | B2RUJ5 | APBA1_M | Apba1 | Amyloid-beta A4 precursor protein-binding family A member 1 | + | | | 3.029559 | 1.443612 | 32.69452 | 0.256789 | -0.16778 | -0.70546 | 0.219956 | 0.250144 | 0.612408 | -1.74885 | -1.83644 | -1.19701 | -1.67189 | -1.23116 | -1.82292 | -3.23064 | -3.24188 | -1.28041 | -1.25292 | -2.05878 | -1.49559 |
| Q5M8N0 | Q5M8N0 | CNRP1_M | Cnrip1 | CB1 cannabinoid receptor-interacting protein 1 | | + | | 0.468811 | 0.264187 | 1.243169 | 1.096137 | -0.95266 | -3.31642 | 0.023686 | 0.02558 | 0.07517 | -0.26854 | 0.138287 | 1.182703 | 0.626235 | 2.113386 | 2.549167 | -0.2678 | -0.39083 | 1.928544 | 1.785706 | 2.567081 | 2.044312 |
| P12960 | P12960 | CNTN1_M | Cntn1 | Contactin-1 | | | | 0.280007 | -0.2492 | -0.76378 | 0.520567 | 0.478269 | 1.380009 | 0.536461 | 0.795604 | 1.422773 | 0.740912 | 1.141559 | 1.52326 | 0.832682 | 2.699462 | 1.581299 | 1.447966 | 0.932904 | 0.669935 | 0.729468 | 1.333618 | 1.355934 |
| O54991 | O54991 | CNTP1_M | Cntnap1 | Contactin-associated protein 1 | | | | 0.199134 | 0.244838 | 0.559326 | 0.015767 | -0.016 | -0.05045 | 0.459056 | 0.295324 | 1.217748 | 0.034426 | 0.353801 | 0.22171 | -0.40987 | 0.966423 | 0.513032 | 0.356847 | -0.4583 | -0.10691 | -0.04925 | 0.530563 | 0.358244 |
| Q8C0L8 | Q8C0L8 | COG5_MO | Cog5 | Conserved oligomeric Golgi complex subunit 5 | | + | | NaN | 0 | NaN | 1.043935 | 1.70206 | 3.09649 | 0.276847 | 0.891831 | 0.755855 | -2.67581 | -3.69251 | -1.11077 | -1.35278 | -3.7837 | -1.61559 | -3.11269 | -3.17187 | -3.47002 | -2.39764 | -3.12567 | -4.05729 |
| Q3UM29 | Q3UM29 | COG7_MO | Cog7 | Conserved oligomeric Golgi complex subunit 7 | | | | 0.220939 | -0.6725 | -0.6149 | 0.268535 | -0.5482 | -0.73499 | 0.193199 | 0.494488 | 0.544111 | -3.33968 | -2.06653 | -2.87591 | -1.39598 | -1.65565 | -2.63245 | -2.91992 | -1.1413 | -1.49417 | -1.68133 | -3.40495 | -1.87213 |
| E9PWQ3 | E9PWQ3 | E9PWQ3_I | Col6a3 | Collagen, type VI, alpha 3 | | | | 0.002479 | 0.016261 | 0.00805 | 0.013037 | -0.06196 | -0.04184 | 0.293917 | 0.620595 | 0.798667 | -0.72485 | -3.30615 | -3.37149 | -1.36346 | -1.36479 | -0.74546 | -0.47785 | -3.58567 | -1.21675 | -3.39427 | -2.38839 | -0.96305 |
| Q8BXC6 | Q8BXC6 | COMD2_M | Commd2 | COMM domain-containing protein 2 | | | | 0.031786 | -0.15931 | -0.10006 | 0.585118 | -0.50992 | -1.55617 | 0.24698 | 0.585653 | 0.680761 | -1.59309 | -3.81419 | -1.40486 | -1.6265 | -0.31659 | -1.14356 | -1.4035 | -3.68517 | -1.31413 | -0.69739 | -0.56132 | -2.07013 |
| Q9CQ02 | Q9CQ02 | COMD4_M | Commd4 | COMM domain-containing protein 4 | | | | 0.278522 | -0.94425 | -0.76006 | 0.43717 | 0.470579 | 1.161089 | 0.235691 | 0.16385 | 0.652267 | -0.83959 | -3.31975 | -1.99143 | -2.22738 | -1.62515 | -1.55694 | -1.06049 | -1.21034 | -2.19224 | -2.96772 | -1.50602 | -2.00377 |
| Q8BIG7 | Q8BIG7 | CMTD1_M | Comtd1 | Catechol O-methyltransferase domain-containing protein 1 | | | | 0.817339 | -1.52879 | -2.26004 | 0.491158 | 1.427563 | 1.301827 | 0.035819 | 0.084796 | 0.112312 | -2.51844 | -1.16962 | -1.25697 | -3.14015 | -1.80557 | -1.34462 | -0.36771 | -0.26276 | -4.18817 | -3.06406 | -0.94093 | -2.37886 |
| Q8CIE6 | Q8CIE6 | COPA_MO | Copa | Coatomer subunit alpha | | | | 0.079705 | 0.091738 | 0.240529 | 0.22936 | 0.090937 | 0.636252 | 0.330483 | 0.359853 | 0.890364 | 0.101423 | 0.507607 | 0.926534 | 0.687841 | 1.531792 | 0.998061 | 0.535609 | -0.11006 | 0.637609 | 0.794892 | 0.601542 | 1.208605 |
| Q9JIF7 | Q9JIF7 | COPB_MO | Copb1 | Coatomer subunit beta | | | | 0.830234 | 0.495092 | 2.303125 | 0.936525 | 0.433471 | 2.677859 | 0.596333 | 0.423033 | 1.587486 | 0.298339 | 0.377141 | 0.789493 | 0.579515 | 1.144646 | 0.634698 | -0.36868 | 0.053972 | 0.127826 | 0.374239 | 0.389181 | 0.544098 |
| O55029 | O55029 | COPB2_M | Copb2 | Coatomer subunit beta' | | | | 0.354388 | 0.324899 | 0.950464 | 0.218482 | -0.14679 | -0.60867 | 0.541293 | 0.323582 | 1.435849 | -0.36241 | -0.52922 | -0.03398 | -0.40646 | 0.357085 | 0.111394 | -0.43921 | -1.10221 | -0.22665 | 0.079797 | -0.27828 | 0.099591 |
| O89079 | O89079 | COPE_MO | Cope | Coatomer subunit epsilon | | | | 0.113941 | 0.177632 | 0.335404 | 0.321732 | -0.17158 | -0.8684 | 0.033613 | -0.04151 | -0.10562 | -3.42941 | -2.38822 | -2.09021 | -1.83776 | -1.47226 | -1.94194 | -3.18373 | -2.98917 | -1.94441 | -1.6404 | -1.98073 | -1.35045 |
| Q9QZE5 | Q9QZE5 | COPG1_M | Copg1 | Coatomer subunit gamma-1 | | | | 0.064812 | 0.060808 | 0.197969 | 0.216956 | 0.28634 | 0.604786 | 0.283132 | 0.368083 | 0.771622 | -1.99645 | -2.55372 | -1.68132 | -1.87633 | -0.65407 | -1.59626 | -2.20662 | -2.46516 | -2.52847 | -1.60186 | -1.56825 | -1.41824 |
| Q9QXK3 | Q9QXK3 | COPG2_M | Copg2 | Coatomer subunit gamma-2 | | + | | 0.738887 | -0.49678 | -2.00787 | 1.72643 | 0.670923 | 7.195017 | 0.249634 | 0.33854 | 0.68745 | -1.13051 | -0.65144 | 0.172163 | 0.160351 | 0.712191 | -0.18991 | -0.33223 | -0.45615 | -0.4116 | -0.59773 | -0.27506 | 0.120254 |
| P61202 | P61202 | CSN2_MO | Cops2 | COP9 signalosome complex subunit 2 | | | | 0.282931 | -0.09949 | -0.77112 | 0.173891 | -0.10663 | -0.4943 | 0.108446 | 0.045343 | 0.32042 | -0.10626 | -0.35885 | 0.129573 | -0.29057 | 0.228254 | 0.135749 | -0.15948 | -0.10665 | 0.075194 | -0.02293 | 0.270397 | 0.002921 |
| O88543 | O88543 | CSN3_MO | Cops3 | COP9 signalosome complex subunit 3 | | | | 0.163658 | 0.924068 | 0.467673 | 0.289809 | 0.625558 | 0.788367 | 0.252674 | -0.10495 | -0.69511 | -3.04502 | -0.20722 | 0.32515 | 0.028133 | 0.291108 | 0.429227 | -1.17511 | -3.92527 | -1.22838 | 0.330548 | 0.599381 | 0.330853 |
| O88544 | O88544 | CSN4_MO | Cops4 | COP9 signalosome complex subunit 4 | | | | 0.320216 | -0.16537 | -0.8646 | 0.130294 | -0.05971 | -0.37952 | 0.407806 | 0.223011 | 1.085795 | -0.68747 | -1.068 | -0.49439 | -0.60484 | 0.17289 | -0.19337 | -0.73195 | -0.69278 | -0.63722 | -0.3426 | -0.32625 | -0.14025 |
| O35864 | O35864 | CSN5_MO | Cops5 | COP9 signalosome complex subunit 5 | | | | 0.394547 | 0.45569 | 1.052031 | 0.502756 | -0.31524 | -1.33252 | 0.418996 | -0.16659 | -1.1144 | -0.38375 | -1.02364 | -0.45053 | -0.88391 | -0.58054 | -0.30559 | -1.45137 | -0.8674 | -0.25704 | -0.44692 | -0.21776 | -0.33519 |
| O88545 | O88545 | CSN6_MO | Cops6 | COP9 signalosome complex subunit 6 | | | | 0.616469 | -0.27794 | -1.6443 | 0.199952 | -0.133 | -0.56142 | 0.025099 | -0.00774 | -0.07954 | -0.35785 | -0.46083 | -0.07459 | 0.019133 | 0.159495 | -0.01848 | 0.029603 | -0.2924 | -0.12695 | 0.337489 | 0.038968 | 0.117522 |
| Q7TPS5 | Q7TPS5 | C2CD5_M | C2cd5 | C2 domain-containing protein 5 | + | | | 2.567336 | 1.440428 | 19.17717 | 0.497467 | 0.387972 | 1.3185 | 0.351592 | -0.47739 | -0.94342 | 5.400307 | 5.540527 | 5.11177 | 5.69788 | 3.853042 | 4.567808 | 4.003038 | 4.056941 | 5.043372 | 4.990335 | 4.329577 | 5.046062 |
| Q8VBV7 | Q8VBV7 | CSN8_MO | Cops8 | COP9 signalosome complex subunit 8 | | | | 0.263835 | -0.61921 | -0.72318 | 0.080117 | -0.14362 | -0.24169 | 0.045043 | 0.032128 | 0.140007 | -4.1278 | -2.42488 | -1.51062 | -2.69863 | -1.63018 | -2.01963 | -2.7474 | -2.56686 | -1.94552 | -1.97649 | -1.73563 | -1.97845 |
| P61924 | P61924 | COPZ1_M | Copz1 | Coatomer subunit zeta-1 | | | | 0.503466 | 0.653036 | 1.334404 | 0.466015 | 0.709738 | 1.235871 | 0.11398 | 0.292853 | 0.33551 | -3.41022 | -2.47239 | -1.35434 | -0.71189 | -1.54308 | -1.02823 | -3.73439 | -3.4543 | -1.26681 | -2.2189 | -0.74447 | -2.41254 |
| Q9CXI0 | Q9CXI0 | COQ5_MC | Coq5 | 2-methoxy-6-polyprenyl-1,4-benzoquinol methylase, mitochondrial | | + | | 0.5773 | 0.655729 | 1.534468 | 1.131352 | 1.33775 | 3.471436 | 0.456007 | 0.336206 | 1.209825 | -0.67908 | -0.97745 | -1.12602 | -0.85383 | -0.68371 | -0.16427 | -1.88444 | -1.08355 | -1.96715 | -2.6882 | -0.66134 | -0.85906 |
| Q60936 | Q60936 | COQ8A_M | Coq8a | Atypical kinase COQ8A, mitochondrial | | + | | 0.130712 | 0.097565 | 0.380642 | 1.882452 | 0.773715 | 8.648156 | 0.267306 | 0.191754 | 0.731901 | 0.260985 | 0.344132 | 0.613862 | 0.77382 | 0.883414 | 0.361831 | 0.457917 | -0.04793 | -0.03978 | -0.11997 | 0.405789 | 0.455948 |
| O89053 | O89053 | COR1A_M | Coro1a | Coronin-1A | | | | 0.025307 | -0.0334 | -0.08018 | 0.828025 | -1.45626 | -2.29571 | 0.146081 | -0.32286 | -0.42151 | -0.88511 | -0.31076 | 0.804777 | -0.45469 | 0.427004 | 1.722278 | -0.26281 | -0.86626 | 1.707625 | 1.554987 | 1.806468 | 0.988535 |
| Q9WUM3 | Q9WUM3 | COR1B_M | Coro1b | Coronin-1B | | + | | 0.436238 | -0.52808 | -1.15869 | 1.246839 | -0.53022 | -4.02099 | 0.010488 | -0.01937 | -0.03375 | -0.40679 | -1.25096 | -0.45185 | -0.30987 | -0.5225 | 0.617498 | -0.47271 | -0.12888 | 0.03824 | 0.260486 | 0.001023 | 0.13271 |
| Q8BH50 | Q8BH50 | ARK2N_M | ARK2N | Protein ARK2N | + | | + | 1.435899 | 1.440274 | 5.078659 | 0.676352 | 0.512449 | 1.818005 | 1.3597 | 0.590879 | 4.626449 | 3.82724 | 4.392504 | 3.631178 | 4.058496 | 3.967369 | 3.799812 | 2.692932 | 2.646264 | 3.148533 | 3.516244 | 3.196312 | 3.389112 |
| Q8C0P5 | Q8C0P5 | COR2A_M | Coro2a | Coronin-2A | | | + | 0.346764 | -0.64434 | -0.93128 | 0.707168 | -1.14789 | -1.91041 | 1.048669 | 0.216799 | 3.115964 | -1.74362 | -2.40061 | -1.82285 | -3.02354 | -1.23133 | -1.36287 | -0.81884 | -2.03671 | -1.30012 | -1.25049 | -1.5366 | -1.4912 |
| Q8BH44 | Q8BH44 | COR2B_M | Coro2b | Coronin-2B | | | | 0.145032 | 0.376292 | 0.418739 | 0.740682 | -0.8244 | -2.01346 | 0.14525 | 0.269534 | 0.419317 | -2.0969 | -0.30133 | -0.50562 | -1.2283 | -0.56336 | 0.673499 | -1.53637 | -1.61445 | -0.23511 | 0.149998 | -0.03917 | -0.38976 |
| Q9D2V7 | Q9D2V7 | CORO7_M | Coro7 | Coronin-7 | | + | | NaN | 0 | NaN | 1.669301 | -2.8255 | -6.72337 | 0.64887 | 2.161931 | 1.737365 | -3.43734 | -3.0996 | -3.05406 | -3.56942 | -0.03104 | -0.02095 | -3.37069 | -3.4043 | -0.81823 | -0.15426 | -0.94356 | -3.43229 |
| Q9CQI6 | Q9CQI6 | COTL1_M | Cotl1 | Coactosin-like protein | | | | 0.184012 | 0.626709 | 0.520474 | 0.243417 | 1.064898 | 0.671777 | 0.032345 | 0.079967 | 0.101762 | -1.97845 | -0.5798 | 0.399644 | -2.21513 | -0.90898 | -0.23066 | -0.92561 | -2.88605 | -2.86907 | -1.07621 | 0.059081 | -1.35865 |
| P97412 | P97412 | LYST_MOU | Lyst | Lysosomal-trafficking regulator | + | | | 1.48418 | 1.429971 | 5.385156 | 0.853446 | 0.409174 | 2.381918 | 0.128957 | -0.22051 | -0.37594 | 0.08926 | 0.174723 | 1.249013 | 1.575494 | -0.64808 | -0.04477 | -1.0359 | -1.56006 | 0.94958 | 1.056579 | -0.62897 | 0.377144 |
| P19783 | P19783 | COX41_M | Cox4i1 | Cytochrome c oxidase subunit 4 isoform 1, mitochondrial | | | | 0.08882 | -0.11923 | -0.26616 | 0.455328 | 0.471638 | 1.20806 | 0.085046 | 0.145051 | 0.255585 | 3.044873 | 3.037303 | 3.030353 | 2.251573 | 2.47569 | 2.526175 | 3.608274 | 2.712367 | 2.141129 | 2.19752 | 2.922845 | 1.788918 |
| Q9JI19 | Q9JI19 | FIBP_MOU | Fibp | Acidic fibroblast growth factor intracellular-binding protein | + | | | 1.997107 | 1.424196 | 9.891342 | 0.28036 | 0.241966 | 0.764669 | 0.474077 | -0.61939 | -1.25694 | 5.311804 | 5.404815 | 5.050642 | 5.678921 | 3.670051 | 4.263106 | 4.07038 | 3.797846 | 5.160838 | 5.084793 | 4.192393 | 4.979544 |
| P60824 | P60824 | CIRBP_MC | Cirbp | Cold-inducible RNA-binding protein | + | | | 1.497757 | 1.422024 | 5.474301 | 0.375072 | 0.478567 | 1.002662 | 0.396686 | 1.065668 | 1.057472 | -1.80175 | -1.34228 | -1.47825 | -1.64311 | -1.72079 | -1.67026 | -3.11527 | -2.8728 | -1.56912 | -2.50937 | -3.76863 | -1.75376 |
| P56391 | P56391 | CX6B1_M | Cox6b1 | Cytochrome c oxidase subunit 6B1 | | | | 0.642272 | 0.542253 | 1.718241 | 0.643994 | 0.719434 | 1.723225 | 0.069765 | 0.10096 | 0.212222 | 1.321279 | 1.313356 | 1.739341 | 0.928476 | 0.700897 | 0.665765 | 1.090626 | 0.459504 | 0.51485 | 0.714098 | 1.057777 | 0.106964 |
| O88522 | O88522 | NEMO_MC | Ikbkg | NF-kappa-B essential modulator | + | + | | 3.065853 | 1.418885 | 34.09154 | 1.107769 | 1.173177 | 3.367012 | 0.009088 | 0.011369 | 0.029294 | 3.53622 | 3.512398 | 3.951877 | 4.648221 | 2.785327 | 2.681381 | 2.145304 | 2.065546 | 3.113392 | 3.140351 | 2.337375 | 3.106595 |
| P48771 | P48771 | CX7A2_M | Cox7a2 | Cytochrome c oxidase subunit 7A2, mitochondrial | | | | 0.339946 | 0.350973 | 0.914133 | 0.295107 | 0.303739 | 0.801649 | 0.29861 | 0.254588 | 0.810431 | 2.80079 | 2.570527 | 2.526083 | 1.792838 | 2.120605 | 2.237628 | 2.700957 | 1.968414 | 1.951367 | 1.760075 | 2.233171 | 1.615888 |
| P17665 | P17665 | COX7C_M | Cox7c | Cytochrome c oxidase subunit 7C, mitochondrial | + | | | 1.130382 | 1.294633 | 3.46709 | 0.394916 | -0.60278 | -1.05297 | 0.097376 | 0.326563 | 0.289966 | 2.367703 | 2.231688 | 1.778907 | 0.912413 | 1.544559 | 2.878385 | 0.637901 | 1.372223 | 2.322614 | 1.574269 | 2.792422 | 0.977395 |
| Q00493 | Q00493 | CBPE_MOI | Cpe | Carboxypeptidase E | | + | | 0.392108 | -0.60776 | -1.04584 | 1.870199 | 1.362001 | 8.52458 | 0.79728 | 0.522958 | 2.19397 | -1.93439 | -0.79624 | 0.248782 | -0.05433 | 0.031561 | 0.260756 | -0.63981 | -0.87528 | -1.2142 | -1.31535 | -0.16779 | -0.58581 |
| Q812E0 | Q812E0 | CPEB2_M | Cpeb2 | Cytoplasmic polyadenylation element-binding protein 2 | | | | NaN | 0 | NaN | 0.873222 | 1.182941 | 2.450343 | 0.04656 | 0.107316 | 0.144521 | -3.56144 | -2.78509 | -2.57654 | -1.76531 | -3.32048 | -2.96194 | -3.42749 | -2.87617 | -3.61566 | -3.09207 | -3.96913 | -2.52792 |
| Q7TN99 | Q7TN98;Q | CPEB3_M | Cpeb3 | Cytoplasmic polyadenylation element-binding protein 3 | + | | | 1.235002 | 0.604179 | 3.961582 | 0.292577 | 0.236723 | 0.795306 | 0.342117 | -0.35832 | -0.91959 | -0.11968 | 0.171428 | 0.644289 | 0.891404 | 0.15529 | 0.3653 | -0.62384 | -0.53277 | 0.260331 | 0.801918 | 0.243377 | 0.993862 |
| Q9WVJ5 | Q9WVJ5 | CRBB1_M | Crybb1 | Beta-crystallin B1 | + | | | 2.967707 | 1.415826 | 30.44404 | 0.986875 | 0.549639 | 2.868704 | 0.650734 | 0.526356 | 1.742782 | -2.11931 | -2.08935 | -2.34385 | -2.00942 | -1.65016 | -1.47432 | -3.47613 | -3.56418 | -2.81982 | -2.63273 | -1.79965 | -2.37754 |
| P63040 | P63040 | CPLX1_M | Cplx1 | Complexin-1 | | | | 0.299441 | -0.31835 | -0.81251 | 0.195415 | -0.23467 | -0.5498 | 0.354525 | 0.466289 | 0.950811 | -0.2313 | 0.520194 | -0.21245 | -1.06501 | -0.12873 | 0.850008 | 0.573848 | 0.351753 | -0.42557 | -0.38256 | -0.13766 | -0.07364 |
| P84086 | P84086;P | CPLX2_M | Cplx2 | Complexin-2 | | | | 0.203366 | 0.171553 | 0.570152 | 0.120949 | 0.148771 | 0.354395 | 0.11079 | -0.08221 | -0.32682 | 1.211738 | 1.736542 | 2.426045 | 1.923136 | 2.503544 | 2.831846 | 1.155347 | 1.449828 | 1.689677 | 2.361963 | 2.940498 | 2.559308 |
| Q8C166 | Q8BT60;Q | CPNE1_M | Cpne1 | Copine-1 | | | | 0.640467 | -1.57171 | -1.71303 | 0.042094 | -0.09472 | -0.1312 | 0.382953 | 0.259333 | 1.022612 | -3.89522 | -3.11942 | -1.6774 | -3.04879 | -1.1125 | -1.56674 | -1.10414 | -2.76708 | -2.49417 | -2.04259 | -1.48613 | -1.71177 |
| Q8BLR2 | Q8BLR2 | CPNE4_M | Cpne4 | Copine-4 | | + | | 0.986152 | 0.559325 | 2.865901 | 1.720973 | -0.58153 | -7.14866 | 0.110637 | -0.08331 | -0.32641 | 1.879551 | 1.48953 | 1.73346 | 1.572513 | 3.455359 | 3.538656 | 1.132992 | 1.117439 | 2.246412 | 2.22262 | 3.328495 | 3.832149 |
| Q9Z140 | Q9Z140 | CPNE6_M | Cpne6 | Copine-6 | | + | | 0.720451 | -0.35922 | -1.95093 | 1.121744 | -0.67089 | -3.42859 | 0.435897 | -0.74233 | -1.15781 | 0.059045 | -0.2697 | 0.06777 | -0.3086 | 1.900167 | 2.314973 | 0.170918 | 0.336874 | 0.604095 | 0.496855 | 3.456581 | 2.243223 |
| Q0VE82 | Q0VE82 | CPNE7_M | Cpne7 | Copine-7 | | | | 0.87362 | 1.487073 | 2.451733 | 0.131677 | 0.045979 | 0.383224 | 0.196834 | 0.083222 | 0.553434 | -1.34891 | -0.36197 | -0.12417 | 0.078313 | 2.212772 | 2.134809 | -1.98984 | -2.69519 | -0.00452 | -0.13329 | 1.945336 | 2.235802 |
| Q1RLL3 | Q1RLL3 | CPNE9_M | Cpne9 | Copine-9 | + | | | 1.266762 | 1.520962 | 4.122675 | 0.221941 | 0.400757 | 0.617448 | NaN | 0 | NaN | -2.44612 | -1.71149 | -2.96477 | -2.7795 | -3.64112 | -3.54132 | -3.63422 | -3.56532 | -3.9153 | -2.63048 | -2.91334 | -3.99034 |
| Q9QXK7 | Q9QXK7 | CPSF3_M | Cpsf3 | Cleavage and polyadenylation specificity factor subunit 3 | | | | NaN | 0 | NaN | 0.174559 | 0.346116 | 0.496029 | 0.923697 | -0.51494 | -2.63067 | -3.45229 | -3.60265 | -2.28091 | -3.41605 | -3.50965 | -3.45169 | -2.90256 | -3.81727 | -3.60049 | -2.7887 | -3.15932 | -2.77215 |
| P97742 | P97742 | CPT1A_M | Cpt1a | Carnitine O-palmitoyltransferase 1, liver isoform | | | | 0.504295 | -0.36433 | -1.3366 | 0.806354 | 0.554058 | 2.223718 | 0.739279 | 0.245644 | 2.009086 | 0.341591 | 0.189072 | 0.602002 | 0.729624 | 0.799533 | 0.975289 | 0.367968 | 0.891352 | 0.352603 | -0.12909 | 0.556758 | 0.726776 |
| Q8BGD5 | Q8BGD5 | CPT1C_M | Cpt1c | Carnitine O-palmitoyltransferase 1, brain isoform | | + | | 0.467591 | 0.641587 | 1.239984 | 2.10788 | 0.264947 | 11.25609 | 0.062059 | -0.04988 | -0.19 | 0.547902 | -0.02826 | 0.51853 | 0.477571 | 0.981503 | 0.469857 | 0.048034 | -0.81157 | 0.221501 | 0.244706 | 0.83448 | 0.716639 |
| E9Q3M9 | E9Q3M9 | E9Q3M9_ | Cracdl | Capping protein inhibiting regulator of actin like | | + | | 0.320654 | -0.91368 | -0.8657 | 1.319286 | -0.93517 | -4.40124 | 0.585837 | -0.51486 | -1.55817 | -0.23436 | -2.15874 | 1.347524 | 1.753851 | 2.102906 | 2.698894 | -0.71658 | 0.150838 | 2.548077 | 2.423633 | 3.05853 | 2.772996 |
| Q8C7D2 | Q8C7D2 | CRBN_MO | Crbn | Protein cereblon | | | | 0.05373 | 0.232609 | 0.165699 | 0.199387 | -0.21952 | -0.55997 | 0.034613 | -0.10383 | -0.10866 | -3.07039 | -0.83511 | -1.20381 | -1.19046 | 0.148504 | -1.61358 | -1.33591 | -3.0348 | -1.36958 | -0.58564 | -0.99863 | -0.2588 |
| Q01147 | Q01147 | CREB1_M | Creb1 | Cyclic AMP-responsive element-binding protein 1 | | | | 0.240392 | 0.19927 | 0.664144 | 0.075072 | 0.075001 | 0.227383 | 0.27994 | -0.4447 | -0.76361 | -2.36401 | -2.09852 | -1.76381 | -1.84214 | -2.00117 | -3.16519 | -2.16146 | -2.69961 | -2.20548 | -1.55046 | -2.11827 | -2.15869 |
| Q9DCT8 | Q9DCT8 | CRIP2_MO | Crip2 | Cysteine-rich protein 2 | | | | 0.210507 | 0.652529 | 0.588372 | 0.03504 | 0.024947 | 0.109952 | 0.019444 | -0.02462 | -0.06198 | -1.55363 | -1.17333 | -0.1417 | -0.59505 | -0.95063 | -0.35693 | -3.10863 | -0.92339 | -0.4032 | -0.38344 | -0.36528 | -0.89304 |
| P56480 | P56480 | ATPB_MOI | Atp5f1b | ATP synthase subunit beta, mitochondrial | + | | | 1.5656 | 1.41523 | 5.94019 | 0.413239 | 0.352916 | 1.09967 | 0.54222 | 0.251384 | 1.438362 | 5.853739 | 6.078012 | 6.168811 | 5.698418 | 5.842613 | 5.524881 | 4.340439 | 4.760852 | 5.362347 | 5.799051 | 5.359519 | 5.505208 |
| P97427 | P97427 | DPYL1_M | Crmp1 | Dihydropyrimidinase-related protein 1 | | | | 0.162753 | 0.091791 | 0.46531 | 0.120952 | -0.13594 | -0.3544 | 0.21707 | -0.12252 | -0.60508 | 1.465291 | 1.707798 | 3.391696 | 3.018678 | 3.328032 | 3.682143 | 1.650356 | 1.33915 | 3.005951 | 3.6763 | 3.725835 | 3.529371 |
| Q8R555 | Q8R555 | CRAC1_M | Crtac1 | Cartilage acidic protein 1 | | | | 0.043678 | 0.19559 | 0.135936 | 0.644139 | 0.507051 | 1.723644 | 0.172715 | -0.1821 | -0.49125 | -2.79666 | -0.39668 | 0.476761 | 0.796227 | 1.163175 | 0.545042 | -0.99837 | -2.58615 | -0.11759 | 0.376473 | 1.240895 | 0.831529 |
| A2RSJ4 | A2RSJ4 | BLT3B_MC | Bltp3b | Bridge-like lipid transfer protein family member 3B | + | | | 1.324287 | 1.409305 | 4.428585 | 0.337624 | 0.301826 | 0.908298 | 0.004787 | 0.001323 | 0.015502 | -1.89814 | -1.44245 | -1.00801 | -1.16037 | -1.42184 | -1.39773 | -3.30176 | -2.85743 | -1.70947 | -1.06257 | -1.32659 | -1.49563 |
| P23927 | P23927 | CRYAB_M | Cryab | Alpha-crystallin B chain | | | | 0.477502 | -0.41107 | -1.26591 | 0.290542 | -0.47662 | -0.7902 | 0.519155 | -0.31878 | -1.37623 | -0.67526 | -1.14722 | -1.78369 | -2.91495 | -2.03472 | -2.46627 | -0.27711 | -0.72323 | -1.66327 | -2.08213 | -1.84749 | -2.01595 |
| Q9JHG7 | Q9JHG7 | PK3CG_M | Pik3cg | Phosphatidylinositol 4,5-bisphosphate 3-kinase catalytic subunit gamma isoform | + | | | 2.462468 | 1.407663 | 16.98669 | 0.45 | 0.202657 | 1.194243 | 0.206158 | -0.31526 | -0.57728 | -1.52655 | -1.69013 | -0.93462 | -0.59659 | -1.02664 | -2.07687 | -3.00268 | -3.02933 | -0.95307 | -0.98344 | -1.38647 | -1.08653 |
| O54983 | O54983 | CRYM_MO | Crym | Ketimine reductase mu-crystallin | | | | 0.438468 | -1.08148 | -1.16444 | 0.6834 | -0.91956 | -1.83895 | 0.097281 | -0.24434 | -0.2897 | -0.90398 | -2.75348 | 0.752002 | -0.21094 | 2.250254 | 3.72135 | -0.83342 | -0.66108 | 1.055068 | 1.325112 | 3.642848 | 2.817439 |
| Q921W4 | Q921W4 | QORL1_M | Cryzl1 | Quinone oxidoreductase-like protein 1 | | | | 0.548745 | -0.70629 | -1.45609 | 0.787092 | 0.411317 | 2.160843 | 0.183119 | -0.19544 | -0.51817 | -1.87422 | -2.80597 | -1.01048 | -0.78257 | -1.53872 | -0.91116 | -1.76887 | -1.49874 | -1.46031 | -1.15537 | -0.82022 | -1.23878 |
| Q3UNZ8 | Q3UNZ8 | QORL2_M | Cryzl2 | Quinone oxidoreductase-like protein 2 | | | | 0.008363 | 0.018044 | 0.026977 | 0.821146 | 0.76681 | 2.272709 | 0.432722 | -0.91863 | -1.14963 | -2.77705 | -1.55421 | -3.21484 | -2.56355 | -3.96016 | -2.43289 | -2.45484 | -1.9125 | -3.56772 | -3.74428 | -2.5132 | -2.04259 |
| Q60931 | Q60931 | VDAC3_M | Vdac3 | Voltage-dependent anion-selective channel protein 3 | + | | | 1.65561 | 1.406123 | 6.614736 | 0.863534 | 0.358487 | 2.416669 | 0.621625 | 0.535553 | 1.658968 | 5.430329 | 5.458057 | 4.739708 | 4.937032 | 5.453484 | 4.858678 | 4.250192 | 3.825948 | 4.369111 | 4.590655 | 4.494965 | 4.746092 |
| Q91W50 | Q91W50 | CSDE1_M | Csde1 | Cold shock domain-containing protein E1 | | | | 0.425102 | 1.124683 | 1.130053 | 0.214492 | 0.132118 | 0.598519 | 0.365985 | 0.552964 | 0.979705 | -1.34869 | -0.5017 | -0.31267 | -0.47882 | 0.133518 | -0.85969 | -1.14923 | -2.95053 | -0.73238 | -0.32335 | -1.18429 | -0.64781 |
| Q9ERK4 | Q9ERK4 | XPO2_MOI | Cse1l | Exportin-2 | | | | 0.48552 | 0.307156 | 1.28697 | 0.735621 | 0.263573 | 1.997719 | 0.312283 | 0.201116 | 0.844707 | 1.489734 | 1.353492 | 1.285419 | 1.392519 | 1.539411 | 1.085399 | 1.343195 | 0.885719 | 1.195977 | 0.954814 | 1.039491 | 1.183087 |
| V9GX34 | V9GX34 | V9GX34_M | Csmd2 | CUB and Sushi multiple domains 2 | | | | NaN | 0 | NaN | 0.229518 | 0.540204 | 0.636652 | NaN | 0 | NaN | -3.34898 | -3.17792 | -1.91897 | -3.61427 | -3.73697 | -2.98073 | -3.09218 | -3.53191 | -3.34492 | -3.26873 | -3.03189 | -3.4211 |
| Q9JKD3 | Q9JKD3 | SCAM5_M | Scamp5 | Secretory carrier-associated membrane protein 5 | + | | | 2.955723 | 1.392615 | 30.0262 | 0.153992 | 0.194957 | 0.44236 | 0.558751 | 0.246237 | 1.483398 | 1.406466 | 1.316077 | 1.806332 | 1.31778 | 1.552692 | 1.828419 | -0.02092 | -0.04176 | 1.733929 | 1.000268 | 1.351864 | 1.536775 |
| Q71M36 | Q71M36 | CSPG5_M | Cspg5 | Chondroitin sulfate proteoglycan 5 | | | | 0.496152 | 1.653921 | 1.315022 | 0.010556 | -0.00764 | -0.03397 | 0.041105 | 0.058296 | 0.128236 | -0.44908 | -0.49801 | 1.249362 | 0.800467 | 0.886864 | 0.111792 | -0.86999 | -3.38494 | 1.019747 | 1.045355 | 0.678685 | 0.203378 |
| P97315 | P97315 | CSRP1_M | Csrp1 | Cysteine and glycine-rich protein 1 | | | | 0.590414 | -0.65561 | -1.57093 | 0.134802 | 0.152168 | 0.391569 | 0.484802 | -1.34701 | -1.28508 | 0.16524 | 0.997428 | 0.059023 | -0.70643 | -0.4029 | 0.734759 | 1.269186 | 1.20471 | -0.4085 | -0.54324 | 2.393354 | 0.63252 |
| P97314 | P97314 | CSRP2_M | Csrp2 | Cysteine and glycine-rich protein 2 | | | | 0.021836 | -0.03807 | -0.06943 | 0.7727 | -1.82936 | -2.11453 | 0.986369 | 0.59292 | 2.866744 | 0.76417 | 0.052778 | -1.12707 | -2.83533 | 0.70978 | 0.573455 | 0.863865 | 0.029226 | -0.01427 | -0.28941 | -0.14657 | 0.24397 |
| P21460 | P21460 | CYTC_MOI | Cst3 | Cystatin-C | | + | | NaN | 0 | NaN | 1.475276 | 1.990753 | 5.327412 | 0.57168 | 0.627747 | 1.518934 | -2.85674 | -3.62067 | -1.18924 | -1.70581 | -1.92468 | -2.23983 | -2.7428 | -3.26651 | -3.16823 | -3.70833 | -2.32794 | -3.09206 |
| Q62426 | Q62426 | CYTB_MOI | Cstb | Cystatin-B | | | | 0.725285 | 0.462028 | 1.965778 | 0.358909 | -0.112 | -0.96186 | 0.376431 | 0.186414 | 1.0061 | 1.820118 | 1.552556 | 0.708143 | 0.82291 | 1.21686 | 1.582418 | 1.417556 | 1.031063 | 0.978858 | 0.776206 | 1.243586 | 1.182865 |
| Q8BIQ5 | Q8C7E9;Q | CSTF2_MC | Cstf2 | Cleavage stimulation factor subunit 2 | | | | 0.616541 | 0.257769 | 1.644502 | 0.116213 | 0.183408 | 0.341574 | 0.931768 | -0.52468 | -2.6603 | -1.12505 | -0.99157 | -0.04649 | -0.47993 | -0.99926 | -1.37189 | -1.17425 | -1.45791 | -0.93788 | 0.044654 | -0.72559 | -0.5962 |
| O88712 | P56546;O | CTBP1_M | Ctbp1 | C-terminal-binding protein 1 | | | | 0.294579 | -0.13728 | -0.80032 | 0.220531 | -0.26614 | -0.61387 | 0.097962 | 0.127722 | 0.291586 | -0.44889 | -0.28579 | 0.177545 | -0.67686 | 0.117878 | 0.532271 | -0.07915 | -0.38097 | -0.0574 | 0.090362 | 0.583274 | -0.18857 |
| Q61301 | Q61301;P | CTNA2_M | Ctnna2 | Catenin alpha-2 | | | | 0.116 | 0.138652 | 0.340998 | 0.35208 | 0.121187 | 0.944653 | 0.328685 | 0.119833 | 0.88585 | 0.146495 | 0.186928 | 1.407566 | 1.286386 | 1.402486 | 1.138853 | 0.434164 | -0.37804 | 1.11271 | 1.338867 | 1.181226 | 1.120447 |
| Q02248 | Q02248 | CTNB1_M | Ctnnb1 | Catenin beta-1 | | | | 0.001549 | -0.00331 | -0.00504 | 0.044597 | 0.009741 | 0.138677 | 0.269452 | 0.306438 | 0.73729 | 0.439001 | -0.10874 | 1.441862 | 1.312421 | 1.836441 | 1.01358 | 0.767067 | -0.43017 | 1.3401 | 1.394701 | 1.059658 | 1.177487 |
| P30999 | P30999 | CTND1_M | Ctnnd1 | Catenin delta-1 | | | | 0.182007 | 0.218385 | 0.515299 | 0.022766 | -0.01909 | -0.07232 | 0.107379 | 0.113922 | 0.317501 | -1.64432 | -1.69865 | -0.05949 | -0.40675 | -0.41857 | -0.88222 | -1.46694 | -2.3128 | -0.41285 | -0.0152 | -1.03817 | -0.49045 |
| O35927 | O35927 | CTND2_M | Ctnnd2 | Catenin delta-2 | | + | | 0.293369 | 0.135825 | 0.797291 | 1.215885 | 0.264033 | 3.867175 | 0.067124 | 0.039376 | 0.204636 | -0.36064 | -0.1473 | 1.270928 | 1.396076 | 0.989669 | 0.752275 | -0.52263 | -0.25697 | 1.042156 | 1.096782 | 0.680151 | 0.983042 |
| P70698 | P70303;P | PYRG1_M | Ctps1 | CTP synthase 1 | | + | | 0.042618 | 0.052925 | 0.132768 | 1.694688 | -0.56192 | -6.92925 | 0.135091 | 0.051015 | 0.392339 | -0.31069 | -0.63934 | -1.29249 | -1.36171 | -0.65844 | -0.51144 | -0.89112 | -0.16476 | -0.69184 | -0.83851 | -0.74322 | -0.5287 |
| P70303 | P70303 | PYRG2_M | Ctps2 | CTP synthase 2 | | | | 0.295148 | 0.800345 | 0.801753 | 0.000478 | 0.001496 | 0.001557 | 0.786494 | -1.13805 | -2.15891 | -1.3294 | -3.30747 | -2.55576 | -3.62287 | -2.96514 | -4.01815 | -2.9835 | -3.25405 | -3.89009 | -2.29154 | -2.37947 | -2.32773 |
| P10605 | P10605 | CATB_MOI | Ctsb | Cathepsin B | + | | | 1.171789 | -0.90964 | -3.65644 | 0.40837 | 0.432225 | 1.087236 | 0.299197 | 0.269043 | 0.811901 | -0.41283 | -0.78836 | 0.676997 | -0.04856 | 0.159911 | 0.758491 | 0.47225 | 0.145847 | -0.28059 | 0.044581 | 0.04791 | 0.332406 |
| P62889 | P62889 | RL30_MOI | Rpl30 | Large ribosomal subunit protein eL30 | + | | | 2.312592 | 1.380013 | 14.27926 | 0.935597 | 0.512686 | 2.674429 | 0.192638 | 0.254008 | 0.542672 | 2.012764 | 1.895587 | 2.174212 | 2.439297 | 1.981249 | 1.28334 | 0.651024 | 0.497303 | 1.655572 | 1.932564 | 1.066327 | 1.690247 |
| A2A9T0 | A2A9T0 | TBKB1_MC | Tbkbp1 | TANK-binding kinase 1-binding protein 1 | + | + | | 2.059761 | 1.37813 | 10.64211 | 1.055939 | 0.982898 | 3.146055 | 0.178684 | -0.45145 | -0.50671 | 2.880982 | 2.831306 | 3.267838 | 3.880814 | 1.517991 | 3.20948 | 1.605107 | 1.35092 | 2.652033 | 2.530821 | 2.535039 | 3.095325 |
| B9EJA2 | B9EJA2 | CTTB2_MC | Cttnbp2 | Cortactin-binding protein 2 | | | | 0.212745 | 0.332007 | 0.594073 | 0.3235 | -0.35862 | -0.87284 | 0.444098 | -0.72476 | -1.17897 | -0.66589 | 0.450989 | 1.762124 | 2.557519 | 0.761488 | 1.98989 | -0.41773 | -0.46119 | 2.415241 | 2.62165 | 2.126237 | 2.074668 |
| Q9WTX6 | Q9WTX6 | CUL1_MOI | Cul1 | Cullin-1 | | | | 0.409852 | 1.027107 | 1.091019 | 0.646422 | 0.296441 | 1.730258 | 0.28058 | -0.27158 | -0.76522 | -1.36055 | -1.85689 | -1.16627 | -0.82381 | -0.93309 | -1.62392 | -1.72771 | -3.54395 | -1.28564 | -1.29731 | -1.08843 | -0.92542 |
| Q9D4H8 | Q9D4H8 | CUL2_MOI | Cul2 | Cullin-2 | | | | 0.251306 | 0.132341 | 0.691661 | 0.595258 | 0.202917 | 1.584474 | 0.100696 | 0.139688 | 0.299139 | -0.16181 | -0.28233 | 0.14453 | -0.0385 | 0.426795 | -0.23694 | -0.17281 | -0.53601 | -0.23949 | -0.06032 | -0.37328 | 0.283751 |
| Q9JLV5 | Q9JLV5 | CUL3_MOI | Cul3 | Cullin-3 | | | | 0.156188 | -0.05487 | -0.44812 | 0.366447 | -0.29658 | -0.98087 | 0.518412 | 0.246826 | 1.37424 | -0.49767 | -0.72399 | -0.2495 | -0.75608 | 0.29685 | 0.080432 | -0.50918 | -0.60274 | -0.37134 | -0.04109 | -0.20154 | 0.085169 |
| Q3TCH7 | Q3TCH7 | CUL4A_M | Cul4a | Cullin-4A | | | | NaN | 0 | NaN | 0.116027 | 0.332008 | 0.34107 | 0.233832 | 0.417922 | 0.647568 | -2.66173 | -2.82902 | -3.08687 | -1.83022 | -1.5242 | -2.8094 | -3.58801 | -3.41305 | -3.53404 | -2.04707 | -2.525 | -2.64444 |
| A2A432 | A2A432 | CUL4B_M | Cul4b | Cullin-4B | | | | 0.325963 | 0.960135 | 0.879021 | 0.049805 | 0.053289 | 0.154136 | 0.065076 | -0.11679 | -0.19873 | -3.83954 | -1.69706 | -2.14234 | -2.83356 | -1.9053 | -3.08012 | -3.51507 | -3.9418 | -2.55015 | -2.53233 | -2.35759 | -2.39425 |
| Q9D5V5 | Q9D5V5 | CUL5_MOI | Cul5 | Cullin-5 | | | | 0.435577 | 0.311488 | 1.156986 | 0.843166 | 0.183078 | 2.346823 | 0.280633 | -0.13347 | -0.76535 | 0.228202 | -0.28991 | 1.302483 | 1.454581 | 0.978647 | 0.891165 | -0.26906 | -0.41563 | 1.178069 | 1.21284 | 0.899563 | 1.23718 |
| Q80TT8 | Q8VE73;Q | CUL9_MOI | Cul9 | Cullin-9 | + | | | 1.115158 | 0.861442 | 3.399461 | 0.620998 | -0.38612 | -1.65718 | 0.393676 | 0.180272 | 1.049818 | -2.48806 | -2.022 | -1.37158 | -1.72734 | -1.71689 | -1.37794 | -3.21603 | -3.01691 | -1.31382 | -1.01284 | -1.75537 | -1.7 |
| Q9D8X1 | Q9D8X1 | CUTC_MO | Cutc | Copper homeostasis protein cutC homolog | | + | | 0.374942 | 1.221308 | 1.002332 | 2.105509 | -0.40841 | -11.225 | 0.236156 | -0.18668 | -0.65344 | -1.16106 | -3.5978 | -0.15565 | -0.10861 | -0.4475 | 0.034782 | -3.5855 | -3.61597 | 0.304035 | 0.248518 | 0.133506 | -0.17287 |
| Q9CQX2 | Q9CQX2 | CYB5B_M | Cyb5b | Cytochrome b5 type B | | | | 0.427883 | 0.59411 | 1.137193 | 0.005405 | 0.008095 | 0.017494 | 0.01662 | 0.015448 | 0.053135 | -0.34118 | -0.37832 | -1.10648 | -1.80632 | -0.16419 | -0.67645 | -0.43175 | -1.47597 | -1.76726 | -1.16172 | -0.57333 | -0.29821 |
| Q9DCN2 | Q9DCN2 | NB5R3_M | Cyb5r3 | NADH-cytochrome b5 reductase 3 | | | | 0.663098 | -0.40049 | -1.77891 | 0.080544 | -0.20204 | -0.2429 | 0.464884 | 0.502877 | 1.232922 | -0.05435 | 0.39488 | 0.595756 | -1.06696 | 0.012096 | 0.443504 | 0.58596 | 0.555542 | -0.06029 | -0.00683 | 0.071093 | -0.62125 |
| Q9D0M3 | Q9D0M3 | CY1_MOU | Cyc1 | Cytochrome c1, heme protein, mitochondrial | | | | 0.873935 | -0.8805 | -2.45283 | 0.109821 | -0.27304 | -0.32418 | 0.202343 | 0.391538 | 0.567537 | 1.449248 | 1.852388 | 1.91649 | 0.232135 | 1.636827 | 2.20096 | 2.828354 | 2.234281 | 1.33563 | 1.359076 | 2.156947 | 0.897762 |
| P62897 | P62897 | CYC_MOU | Cycs | Cytochrome c, somatic | | | | 0.33441 | -0.78674 | -0.90022 | 0.207003 | 0.716305 | 0.579439 | 0.028736 | -0.0531 | -0.09073 | -0.17204 | 1.554369 | 2.48729 | 0.017493 | 0.645013 | 0.665118 | 1.341372 | 1.614434 | 0.59286 | 0.479314 | 1.293373 | 0.122967 |
| Q7TMB8 | Q7TMB8 | CYFP1_M | Cyfip1 | Cytoplasmic FMR1-interacting protein 1 | | | | 0.725738 | 0.617993 | 1.967173 | 0.26175 | -0.18017 | -0.71794 | 0.012887 | 0.011748 | 0.041365 | 0.353031 | 0.165602 | 0.712224 | 0.785907 | 1.642822 | 1.243836 | -0.65853 | -0.05883 | 0.680998 | 1.17748 | 1.229427 | 1.633735 |
| Q5SQX6 | Q5SQX6 | CYFP2_M | Cyfip2 | Cytoplasmic FMR1-interacting protein 2 | | | | 0.694914 | 0.533399 | 1.873408 | 0.85986 | -0.23382 | -2.40398 | 0.265954 | 0.085729 | 0.728504 | 1.410744 | 1.521225 | 3.090728 | 3.168153 | 3.371567 | 3.397313 | 1.211897 | 0.653275 | 3.27403 | 3.452482 | 3.181738 | 3.415683 |
| Q9CX80 | Q9CX80 | CYGB_MO | Cygb | Cytoglobin | | | | 0.126768 | 0.270299 | 0.370066 | 0.190122 | -0.13989 | -0.53621 | 0.480963 | -0.87576 | -1.27499 | 0.231126 | -1.19942 | -2.01104 | -2.43246 | -3.14632 | -1.92958 | -0.60653 | -0.90236 | -2.2357 | -1.92801 | -1.98107 | -1.34331 |
| Q80TQ2 | Q80TQ2 | CYLD_MOI | Cyld | Ubiquitin carboxyl-terminal hydrolase CYLD | | + | | 0.323254 | 0.160262 | 0.872221 | 1.043713 | 0.320236 | 3.095577 | 0.382255 | -0.43122 | -1.02084 | -1.64105 | -1.45546 | 1.024684 | 1.121649 | -0.04629 | -0.03632 | -1.54993 | -1.86711 | 0.661545 | 0.844316 | -0.03247 | 0.812296 |
| P85094 | P85094 | ISC2A_MO | Isoc2a | Isochorismatase domain-containing protein 2A | + | | | 1.674202 | 1.371988 | 6.762664 | 0.602824 | -0.55746 | -1.60572 | 0.036899 | -0.03017 | -0.11558 | 2.551508 | 2.74747 | 0.856111 | 0.664424 | 1.948483 | 2.434424 | 1.099854 | 1.455149 | 0.984047 | 1.651406 | 2.126255 | 2.316986 |
| O55071;P | P12790;E | CP2B9_M | Cyp2b19;Cyp2b9 | Cytochrome P450 2B19 | | | + | 0.483172 | 2.213827 | 1.280794 | 0.330214 | 1.554818 | 0.88969 | 2.338396 | -3.62708 | -14.7129 | 0.374156 | -3.08137 | -0.06974 | 0.436437 | -3.25701 | -3.74118 | -3.51772 | -3.61715 | -3.10064 | 0.357701 | 0.174547 | 0.081421 |
| Q9WVK8 | Q9WVK8 | CP46A_M | Cyp46a1 | Cholesterol 24-hydroxylase | + | | + | 1.065304 | 0.492459 | 3.185137 | 0.041709 | 0.003863 | 0.130047 | 1.293849 | 0.074232 | 4.264395 | 0.400146 | 0.434765 | 1.785861 | 1.727535 | 1.158052 | 1.159617 | 0.078636 | -0.22864 | 1.758466 | 1.747205 | 1.067213 | 1.101993 |
| Q8BHZ0 | Q8BHZ0 | CYRIA_MO | Cyria | CYFIP-related Rac1 interactor A | | | | 0.255766 | 0.320555 | 0.702887 | 0.926701 | 0.841034 | 2.641671 | 0.458103 | 0.150596 | 1.21527 | -1.72144 | -2.58008 | -0.29932 | -0.74882 | -0.49619 | -0.69006 | -2.31747 | -2.62517 | -1.5906 | -1.13961 | -0.66652 | -0.82092 |
| Q921M7 | Q921M7;Q | CYRIB_MO | Cyrib | CYFIP-related Rac1 interactor B | | | | 0.131666 | 0.066617 | 0.383192 | 0.243936 | 0.324516 | 0.673085 | 0.054295 | -0.01675 | -0.16736 | -0.084 | 0.077843 | 1.439619 | 0.617091 | 0.761661 | 0.935556 | -0.22356 | 0.084171 | 0.452217 | 0.955461 | 0.914941 | 0.815778 |
| Q9QX11 | Q9QX11 | CYH1_MO | Cyth1 | Cytohesin-1 | | | | 0.254338 | 0.242219 | 0.699295 | 0.213231 | -0.15694 | -0.59531 | 0.678222 | -0.93972 | -1.82355 | 0.23621 | -0.43568 | 1.618893 | 1.961756 | 0.655165 | 1.524496 | -0.42631 | -0.25759 | 1.746994 | 2.147525 | 1.752738 | 2.306355 |
| P63034 | P63034 | CYH2_MO | Cyth2 | Cytohesin-2 | | | | 0.326668 | -0.89527 | -0.88079 | 0.220323 | 0.26772 | 0.613341 | 0.791335 | -0.96418 | -2.17461 | -3.39899 | -3.80485 | 0.444671 | 0.750072 | -0.83848 | 0.01193 | -1.71067 | -3.70263 | -0.07926 | 0.738565 | 0.425253 | 0.676552 |
| Q91VN6 | Q91VN6 | DDX41_M | Ddx41 | Probable ATP-dependent RNA helicase DDX41 | + | | | 1.666037 | 1.369585 | 6.697319 | 0.23066 | 0.153043 | 0.639543 | 0.29494 | 0.217548 | 0.801229 | 0.786697 | 1.028405 | 0.925949 | 1.307295 | 0.472369 | 0.374921 | -0.627 | -0.29707 | 0.818981 | 1.108177 | -0.06101 | 0.473207 |
| A0A2R8VK | A0A2R8VK | A0A2R8VK | D430041D05Rik | RIKEN cDNA D430041D05 gene | | | | 0.33683 | -1.13734 | -0.9063 | 0.501097 | 0.182343 | 1.328119 | 0.0678 | 0.065959 | 0.20658 | -3.30832 | -4.22513 | -0.2128 | -0.27289 | -0.53559 | -0.94992 | -1.46118 | -3.79759 | -0.55915 | -0.29122 | -1.05168 | -0.56576 |
| Q8BPM0 | Q8BPM0 | DAAM1_M | Daam1 | Disheveled-associated activator of morphogenesis 1 | | | | 0.172524 | 0.226827 | 0.490749 | 0.781425 | 0.586061 | 2.142542 | 0.07232 | -0.05089 | -0.21953 | -0.76498 | -1.47969 | -0.17941 | 0.361486 | -0.3941 | -0.7699 | -1.05602 | -1.6423 | -0.536 | -0.45404 | -0.66687 | -0.39534 |
| Q80U19 | Q80U19 | DAAM2_M | Daam2 | Disheveled-associated activator of morphogenesis 2 | + | | | 1.138817 | -0.83257 | -3.50502 | 0.049877 | 0.063476 | 0.154348 | 0.162569 | 0.129486 | 0.46483 | -1.41245 | -1.05245 | -1.06861 | -0.62744 | -1.06216 | -1.60413 | -0.24489 | -0.55488 | -1.25859 | -0.56441 | -1.52719 | -1.39808 |
| Q3UHC7 | Q3UHC7 | DAB2P_M | Dab2ip | Disabled homolog 2-interacting protein | | + | | 0.283048 | -0.43644 | -0.77141 | 1.008954 | -0.41835 | -2.95535 | 0.705247 | -0.52813 | -1.90459 | -0.83906 | -1.51719 | -0.27509 | -0.29005 | -1.25725 | -1.08173 | -1.1946 | -0.28876 | -0.00558 | 0.277136 | -0.9044 | -0.37832 |
| Q0PHV7 | Q0PHV7 | DACT3_M | Dact3 | Dapper homolog 3 | | | | 0.580224 | -1.35724 | -1.54257 | 0.509406 | 0.271005 | 1.3502 | 0.263453 | -0.50517 | -0.72222 | -3.89993 | -3.10865 | -0.06392 | 0.295568 | -0.4939 | -0.61513 | -1.36117 | -2.93294 | -0.2445 | -0.06586 | -0.74618 | 0.647488 |
| P61804 | P61804 | DAD1_MO | Dad1 | Dolichyl-diphosphooligosaccharide--protein glycosyltransferase subunit DAD1 | | | | 0.145332 | 0.075521 | 0.419532 | 0.519496 | 1.149006 | 1.377142 | 0.077056 | 0.120228 | 0.233022 | -1.0832 | -1.11715 | -1.63481 | -1.27581 | -1.78288 | -1.36619 | -1.35491 | -0.99649 | -1.78951 | -3.41912 | -1.22275 | -2.16678 |
| Q62165 | Q62165 | DAG1_MO | Dag1 | Dystroglycan 1 | | | + | NaN | 0 | NaN | 0.80252 | 0.369828 | 2.211122 | 1.097652 | 1.429817 | 3.322977 | -3.45623 | -2.72769 | -2.97413 | -3.16089 | -1.57903 | -2.11379 | -3.22729 | -3.5159 | -3.5761 | -3.29857 | -3.61335 | -2.93911 |
| P83940 | P83940 | ELOC_MO | Eloc | Elongin-C | + | + | | 2.235771 | 1.365646 | 13.0613 | 1.773656 | 0.19037 | 7.608313 | 0.374885 | -0.32398 | -1.00219 | 0.354106 | 0.271266 | 1.263341 | 1.273436 | 0.503823 | 0.752777 | -0.95696 | -1.14896 | 1.053511 | 1.102525 | 0.653934 | 1.250628 |
| Q922B2 | Q922B2 | SYDC_MO | Dars1 | Aspartate--tRNA ligase, cytoplasmic | | | | 0.002668 | -0.00176 | -0.00866 | 0.063493 | -0.0296 | -0.19416 | 0.066025 | 0.036682 | 0.201469 | 2.638428 | 2.710874 | 3.051609 | 2.942229 | 2.897363 | 3.222327 | 2.876485 | 2.47634 | 3.168856 | 2.884191 | 3.105326 | 2.940999 |
| Q9JII5 | Q9JII5 | DAZP1_M | Dazap1 | DAZ-associated protein 1 | | | | 0.235432 | 0.898501 | 0.651613 | 0.248262 | -0.33668 | -0.68399 | 0.134003 | 0.305423 | 0.389436 | -0.50078 | -0.71152 | -1.71996 | -1.19047 | -1.32103 | 0.115408 | -0.12979 | -2.87951 | -1.53351 | -0.70356 | -0.59321 | -1.22326 |
| P31786 | P31786 | ACBP_MO | Dbi | Acyl-CoA-binding protein | | | | 0.434998 | 1.716685 | 1.155493 | 0.479969 | 1.941284 | 1.272381 | 0.154897 | 0.243226 | 0.444736 | -2.90714 | -0.23293 | 0.305984 | -2.68874 | -1.84562 | -2.24288 | -2.63912 | -3.93432 | -2.83991 | -3.42541 | -1.77792 | -2.79703 |
| Q9QXS6 | Q9QXS6 | DREB_MOI | Dbn1 | Drebrin | | + | | 0.899429 | -1.08851 | -2.54293 | 1.256774 | -0.2591 | -4.07143 | 0.067799 | -0.17881 | -0.20658 | -1.53254 | -0.67919 | 0.628847 | 0.522227 | 0.449659 | 1.8077 | 0.016988 | -0.0517 | 0.799884 | 0.8694 | 1.844314 | 0.770668 |
| Q62418 | Q62418 | DBNL_MO | Dbnl | Drebrin-like protein | | | | 0.464768 | -0.13394 | -1.23262 | 0.061185 | 0.013694 | 0.187468 | 0.451599 | -0.33295 | -1.19839 | -1.35592 | -1.51242 | -0.06411 | 0.042421 | -0.51078 | 0.040842 | -1.22484 | -1.37562 | -0.07453 | 0.025452 | 0.131405 | 0.064551 |

| | | | | | | | | | | | | | | | | | | | | | | | | | | | |
|---|---|---|---|---|---|---|---|---|---|---|---|---|---|---|---|---|---|---|---|---|---|---|---|---|---|---|---|
| Q80T85 | Q80T85 | DCAF5_M( | Dcaf5 | DDB1- and CUL4-associated factor 5 | | | | NaN | 0 | NaN | 0.166183 | -0.56671 | -0.47426 | 0.523281 | -0.75656 | -1.38729 | -3.25832 | -3.40788 | -1.48046 | -3.58759 | -4.08943 | -3.40154 | -3.38411 | -2.94487 | -1.40348 | -2.53115 | -3.41214 | -2.5657 |
| Q3UMY5 | Q3UMY5 | EMAL4_M( | Eml4 | Echinoderm microtubule-associated protein-like 4 | + | | | 1.63942 | 1.36325 | 6.488383 | 0.874089 | 0.628044 | 2.453371 | 0.457638 | -0.79172 | -1.21406 | 3.63989 | 3.233453 | 3.467578 | 3.978721 | 1.798403 | 2.42735 | 2.020065 | 2.126778 | 3.080429 | 3.109783 | 2.333304 | 3.475891 |
| Q8N7N5 | Q8N7N5 | DCAF8_M( | Dcaf8 | DDB1- and CUL4-associated factor 8 | | | | NaN | 0 | NaN | 0.328231 | 0.866505 | 0.88471 | 0.908252 | -1.17553 | -2.5746 | -3.95026 | -3.69344 | -1.5739 | -1.58217 | -3.43974 | -2.62254 | -2.91411 | -2.6729 | -3.42395 | -1.46513 | -1.65187 | -2.05937 |
| Q62148 | Q62148 | AL1A2_M( | Aldh1a2 | Retinal dehydrogenase 2 | + | | | 2.021417 | 1.353082 | 10.17632 | 0.529265 | -0.30843 | -1.40337 | 0.085014 | 0.098517 | 0.255494 | 0.797134 | 0.53144 | -0.50949 | -0.11697 | -0.61565 | -0.90078 | -0.69437 | -0.68322 | -0.10371 | 0.094121 | -1.215 | -0.49846 |
| O08788 | O08788 | DCTN1_M( | Dctn1 | Dynactin subunit 1 | | | | 0.581167 | 0.48673 | 1.545186 | 0.927293 | 0.559387 | 2.643842 | 0.444332 | 0.369823 | 1.179576 | 0.547607 | 0.126254 | 0.971338 | 1.025056 | 0.992682 | 0.643573 | 0.084377 | -0.38398 | 0.22894 | 0.648679 | 0.187868 | 0.70874 |
| Q99KJ8 | Q99KJ8 | DCTN2_M( | Dctn2 | Dynactin subunit 2 | | | | 0.994654 | 0.648273 | 2.899021 | 0.598325 | 0.432517 | 1.593073 | 0.45215 | -0.26601 | -1.19982 | -0.00573 | 0.149473 | 0.892499 | 0.878675 | -0.23934 | -0.23383 | -0.78612 | -0.36668 | 0.18166 | 0.724481 | -0.19227 | 0.251109 |
| Q9Z0Y1 | Q9Z0Y1 | DCTN3_M( | Dctn3 | Dynactin subunit 3 | | + | | 0.503723 | 0.147108 | 1.335087 | 1.249505 | 0.381242 | 4.034481 | 0.061373 | 0.04351 | 0.188014 | -0.18648 | 0.019625 | 0.771337 | 0.584176 | 0.080557 | -0.25071 | -0.19153 | -0.26954 | 0.309636 | 0.283394 | -0.2902 | 0.033029 |
| Q8CBY8 | Q8CBY8 | DCTN4_M( | Dctn4 | Dynactin subunit 4 | | | | 0.86199 | 0.586039 | 2.411329 | 0.123636 | -0.11602 | -0.36164 | 0.109251 | -0.06156 | -0.32262 | 1.033225 | 0.770175 | 1.18478 | 0.973278 | 0.609543 | 0.991167 | 0.11129 | 0.520032 | 0.892171 | 1.497919 | 0.863824 | 0.860011 |
| Q9QZB9 | Q9QZB9 | DCTN5_M( | Dctn5 | Dynactin subunit 5 | + | | | 1.132661 | 0.36379 | 3.477307 | 0.637761 | -0.33595 | -1.70522 | 0.304306 | -0.10816 | -0.82471 | 0.077265 | 0.212284 | -0.16815 | 0.202096 | -0.13807 | 0.104852 | -0.29894 | -0.13909 | 0.420318 | 0.28552 | 0.042072 | 0.141039 |
| Q9WUB4 | Q9WUB4 | DCTN6_M( | Dctn6 | Dynactin subunit 6 | | | | 0.005632 | 0.023533 | 0.018224 | 0.306156 | -0.32508 | -0.82935 | 0.187959 | 0.165889 | 0.530642 | -1.50014 | -4.01487 | -1.55979 | -1.60171 | -1.762 | -1.21758 | -3.07516 | -2.48691 | -0.86427 | -1.64708 | -1.50195 | -1.8094 |
| Q9D9Z5 | Q9D9Z5 | DDA1_MO | Dda1 | DET1- and DDB1-associated protein 1 | | | | 0.376646 | 1.378317 | 1.006644 | 0.247946 | 0.866432 | 0.683196 | 0.013595 | 0.078222 | 0.043604 | -3.46993 | -0.73287 | -1.58626 | -1.65243 | -1.43378 | -3.21016 | -3.43627 | -3.52317 | -3.75355 | -1.21801 | -3.95877 | -0.84161 |
| Q9CWS0 | Q9CWS0 | DDAH1_M | Ddah1 | N(G),N(G)-dimethylarginine dimethylaminohydrolase 1 | | | | 0.931555 | -1.03806 | -2.65951 | 0.587044 | -1.05851 | -1.56153 | 0.098102 | -0.29326 | -0.29197 | -0.96709 | -0.29759 | 0.886346 | -0.38789 | -0.60851 | 1.113674 | 0.606445 | 0.204994 | 1.076259 | 1.539207 | 1.062919 | 0.02877 |
| Q99LD8 | Q99LD8 | DDAH2_M | Ddah2 | N(G),N(G)-dimethylarginine dimethylaminohydrolase 2 | + | | + | 1.059563 | 0.255321 | 3.161136 | 0.158323 | 0.383687 | 0.453721 | 1.113384 | 1.058145 | 3.391649 | -0.05 | -0.04712 | -0.51923 | -1.00863 | 0.269744 | -0.18155 | -0.38464 | -0.22313 | -1.95708 | -0.33815 | -1.2295 | -0.7986 |
| Q3U1J4 | Q3U1J4 | DDB1_MO | Ddb1 | DNA damage-binding protein 1 | | | | 0.495487 | 0.30441 | 1.313263 | 0.110287 | 0.041046 | 0.32545 | 0.240507 | -0.19036 | -0.66443 | 1.062134 | 0.976325 | 2.157116 | 2.126586 | 1.302041 | 1.577343 | 0.942611 | 0.487029 | 1.975612 | 2.225998 | 1.378784 | 1.881324 |
| Q80YA3 | Q80YA3 | DDHD1_M | Ddhd1 | Phospholipase DDHD1 | | | | 0.734064 | 0.723671 | 1.992891 | 0.992926 | 0.640882 | 2.892267 | 0.165366 | 0.245044 | 0.472129 | -1.41619 | -1.62146 | -0.53111 | -0.15694 | -1.06486 | -1.18349 | -2.59082 | -1.89418 | -1.10365 | -0.86617 | -1.88483 | -0.8536 |
| Q80TS7 | Q80TS7 | DEND_MO | Ddn | Dendrin | | | + | NaN | 0 | NaN | 0.343515 | 0.293008 | 0.923104 | 1.63974 | -1.04025 | -6.49086 | -2.56593 | -2.88489 | -1.16733 | -0.58213 | -1.18141 | -0.94667 | -3.31061 | -3.29925 | -1.0447 | -1.29077 | -0.13291 | 0.08534 |
| O54734 | O54734 | OST48_M( | Ddost | Dolichyl-diphosphooligosaccharide--protein glycosyltransferase 48 kDa subunit | | | | 0.211584 | -0.28142 | -0.59112 | 0.252804 | -0.28466 | -0.69543 | 0.347483 | 0.122481 | 0.933083 | -0.2756 | -0.64886 | -0.29865 | -1.1087 | -0.01337 | -0.24886 | 0.257157 | -0.61878 | -0.47819 | -0.35984 | -0.19558 | -0.31162 |
| Q91VR5 | Q91VR5 | DDX1_MO | Ddx1 | ATP-dependent RNA helicase DDX1 | + | | | 1.292883 | -0.37062 | -4.25927 | 0.70491 | -0.32625 | -1.90357 | 0.062077 | -0.03031 | -0.19006 | -0.22879 | -0.37876 | 0.45372 | 0.198984 | 0.484187 | 0.521858 | 0.111001 | 0.022695 | 0.537922 | 0.767286 | 0.374962 | 0.691707 |
| Q501J6 | Q501J6 | DDX17_M( | Ddx17 | Probable ATP-dependent RNA helicase DDX17 | | | | 0.571473 | 0.510936 | 1.518362 | 0.000397 | -0.00034 | -0.00129 | 0.971015 | -0.10461 | -2.8076 | 0.419713 | 1.001768 | 0.886791 | 1.163117 | 1.195829 | 1.150173 | 0.368737 | 0.030872 | 0.796557 | 1.254041 | 1.307055 | 1.248162 |
| Q9JIK5 | Q9JIK5 | DDX21_M( | Ddx21 | Nucleolar RNA helicase 2 | | | | 0.061916 | 0.185558 | 0.189588 | 0.754761 | 0.937166 | 2.057574 | 0.20965 | 0.574634 | 0.586189 | -3.91845 | -2.12184 | -3.03146 | -2.13975 | -1.6223 | -3.45792 | -3.59427 | -2.81714 | -3.42966 | -3.61587 | -2.77037 | -3.45912 |
| Q9Z1N5 | Q8VDW0;( | DX39B_M( | Ddx39b | Spliceosome RNA helicase Ddx39b | | | | 0.06018 | -0.12868 | -0.18455 | 0.52649 | -0.50941 | -1.3959 | 0.126086 | -0.39728 | -0.36823 | -0.678 | -1.63155 | -1.16101 | -1.89085 | -1.2116 | -3.23621 | -0.51731 | -1.53488 | -1.01944 | -1.01359 | -1.45347 | -2.19977 |
| Q62167 | P16381;Q( | DDX3X_M( | Ddx3x | ATP-dependent RNA helicase DDX3X | | | | 0.713324 | 0.314089 | 1.929137 | 0.027201 | -0.01503 | -0.08602 | 0.057916 | -0.03071 | -0.17795 | 1.47208 | 1.401802 | 1.806693 | 1.957196 | 2.052665 | 1.804926 | 1.281828 | 0.963876 | 1.739275 | 2.054675 | 1.839338 | 2.079675 |
| Q9JME7 | Q9JME7 | TPC2L_M( | Trappc2l | Trafficking protein particle complex subunit 2-like protein | + | | | 1.562885 | 1.352932 | 5.920872 | 0.45509 | 0.454275 | 1.207442 | 0.500222 | -0.63569 | -1.3258 | -1.81537 | -1.59979 | -1.69423 | -1.5634 | -2.32563 | -2.09803 | -2.85903 | -3.26199 | -2.45359 | -1.71259 | -2.04192 | -1.11037 |
| Q810A7 | Q810A7 | DDX42_M( | Ddx42 | ATP-dependent RNA helicase DDX42 | | | | NaN | 0 | NaN | 0.091025 | -0.22588 | -0.27232 | 0.19844 | 0.946689 | 0.557548 | -3.48746 | -3.01397 | -2.4314 | -3.5433 | -0.92355 | -4.2227 | -3.22989 | -3.24866 | -3.37705 | -2.14589 | -3.11741 | -3.92222 |
| Q61656 | Q61656 | DDX5_MO | Ddx5 | Probable ATP-dependent RNA helicase DDX5 | + | | | 1.121705 | 0.63701 | 3.42842 | 0.010819 | 0.002929 | 0.034807 | 0.322737 | -0.1362 | -0.87093 | 1.708156 | 1.96091 | 2.613628 | 2.687128 | 2.964013 | 2.651281 | 1.333727 | 1.061319 | 2.571749 | 2.723149 | 2.941459 | 2.946234 |
| P54823 | P54823 | DDX6_MO | Ddx6 | Probable ATP-dependent RNA helicase DDX6 | | | | 0.692231 | 0.558354 | 1.86535 | 0.08429 | 0.08182 | 0.25346 | 0.127487 | 0.035033 | 0.371996 | -1.07978 | -1.5831 | -0.33698 | -0.13651 | -0.49606 | -0.60265 | -1.72772 | -2.05186 | -0.62542 | -0.01171 | -0.66203 | -0.50674 |
| Q9CQ62 | Q9CQ62 | DECR_MO | Decr1 | 2,4-dienoyl-CoA reductase [(3E)-enoyl-CoA-producing], mitochondrial | + | | | 1.298766 | -3.13828 | -4.29056 | NaN | 0 | NaN | NaN | 0 | NaN | -4.44616 | -2.99163 | -3.21905 | -3.46926 | -3.9011 | -3.30241 | -0.65864 | -0.50259 | -3.33911 | -2.69083 | -3.2952 | -3.10388 |
| Q9WV68 | Q9WV68 | DECR2_M( | Decr2 | Peroxisomal 2,4-dienoyl-CoA reductase [(3E)-enoyl-CoA-producing] | | + | | 0.267361 | -1.29692 | -0.73204 | 1.517232 | -2.72959 | -5.6045 | 0.753373 | -0.53447 | -2.0532 | -4.11439 | -0.58495 | -3.64933 | -3.37796 | -1.07096 | -0.59671 | -0.89617 | -1.20935 | -0.31631 | -1.25181 | -0.40676 | -0.19196 |
| Q8K3M5 | Q8K3M5 | CABL2_M( | Cables2 | CDK5 and ABL1 enzyme substrate 2 | + | | | 3.118768 | 1.351164 | 36.23566 | 0.89682 | 0.613548 | 2.533611 | 0.287717 | -0.28809 | -0.78312 | 4.119572 | 4.193921 | 3.725169 | 4.201487 | 3.048146 | 3.407377 | 2.802669 | 2.808496 | 3.393636 | 3.305924 | 3.194805 | 3.836903 |
| Q9D8N2 | Q9D8N2 | DEN10_M( | Dennd10 | DENN domain-containing protein 10 | | | | 0.074629 | -0.11264 | -0.22612 | 0.185899 | 0.190716 | 0.525337 | 0.258301 | 0.592728 | 0.709265 | -3.28934 | -3.02308 | -1.70174 | -2.41014 | -1.25567 | -2.22762 | -2.56357 | -3.52358 | -2.32626 | -2.16705 | -1.65451 | -3.01423 |
| P61460 | P61460 | DEPD5_M( | Depdc5 | GATOR complex protein DEPDC5 | | | | 0.235319 | 0.250852 | 0.651328 | 0.637339 | 1.036301 | 1.704002 | 0.134731 | -0.45086 | -0.39138 | -3.32067 | -2.58307 | -1.65719 | -1.12346 | -2.04161 | -3.38137 | -3.31372 | -3.09173 | -2.9731 | -1.88015 | -3.19781 | -1.32346 |
| Q570Y9 | Q570Y9 | DPTOR_M( | Deptor | DEP domain-containing mTOR-interacting protein | | | | 0.330833 | -1.32403 | -0.89124 | 0.317224 | -0.35977 | -0.8571 | 0.18429 | 0.15464 | 0.521188 | -1.06711 | -3.95824 | -1.04709 | -1.60154 | -1.93781 | -1.85662 | -1.53122 | -0.84607 | -1.27972 | -0.64936 | -1.75794 | -2.34577 |
| Q99J56 | Q99J56 | DERL1_M( | Derl1 | Derlin-1 | | | | 0.338636 | 0.261815 | 0.910842 | 0.138149 | 0.147209 | 0.400483 | 0.091617 | -0.04422 | -0.27397 | 1.288552 | 1.384632 | 0.001434 | 0.546742 | 0.87549 | 0.647038 | 0.791377 | 1.358177 | 0.373404 | -0.11965 | 0.691442 | 0.919531 |
| O88673 | O88673 | DGKA_MO | Dgka | Diacylglycerol kinase alpha | | | | 0.326318 | 1.011847 | 0.87991 | 0.106131 | 0.089612 | 0.314082 | 0.410511 | -0.21619 | -1.0927 | -3.23654 | -0.93685 | 1.344525 | 1.564724 | 1.152245 | 0.766296 | -3.11371 | -3.08338 | 1.101797 | 1.628228 | 1.131821 | 1.219094 |
| Q8CIQ7 | Q8CIQ7 | DOCK3_M | Dock3 | Dedicator of cytokinesis protein 3 | + | | | 2.837417 | 1.339072 | 26.19596 | 0.591975 | -0.13616 | -1.57529 | 0.564967 | -0.31573 | -1.50045 | 0.031857 | 0.089314 | 0.490937 | 0.638364 | -0.0675 | 0.155901 | -1.23621 | -1.32077 | 0.655674 | 0.74594 | 0.181602 | 0.538259 |
| E9PUQ8 | E9PUQ8 | DGKD_MO | Dgkd | Diacylglycerol kinase delta | | | + | NaN | 0 | NaN | 0.362264 | -0.55881 | -0.97031 | 1.593348 | -0.40542 | -6.14101 | -2.83657 | -3.64512 | -2.00888 | -2.87671 | -1.98932 | -1.93948 | -2.96325 | -2.95996 | -2.26265 | -1.50531 | -1.62011 | -1.49784 |
| Q9R1C6 | Q9R1C6 | DGKE_MO | Dgke | Diacylglycerol kinase epsilon | | | | 0.208579 | 0.849601 | 0.583458 | 0.36027 | 0.403517 | 0.965286 | 0.032728 | 0.061983 | 0.102927 | -1.84494 | -1.01855 | -0.78825 | -0.93567 | -0.51154 | -1.58224 | -3.67764 | -0.88505 | -1.67695 | -0.854 | -0.8331 | -1.38465 |
| Q91WG7 | Q91WG7 | DGKG_MO | Dgkg | Diacylglycerol kinase gamma | | | | 0.467058 | 1.173036 | 1.238593 | 0.616883 | 0.449554 | 1.645474 | 0.287647 | -0.21508 | -0.78294 | -3.25258 | -1.47057 | -0.30546 | 0.109205 | 0.65604 | 0.681455 | -3.85564 | -3.21359 | -0.7256 | -0.36977 | 0.609416 | 1.158233 |
| D3YXJ0 | D3YXJ0 | DGKH_MO | Dgkh | Diacylglycerol kinase eta | | | | 0.154227 | -0.20251 | -0.44298 | 0.39328 | 0.239308 | 1.048813 | 0.113714 | 0.081526 | 0.334786 | -0.87042 | -1.73974 | -0.42619 | -0.42045 | -0.75153 | -1.23001 | -1.2442 | -0.96094 | -0.89078 | -0.43448 | -1.11772 | -1.02687 |
| D3YWQ0 | D3YWQ0 | DGKI_MOI | Dgki | Diacylglycerol kinase iota | | | | 0.500005 | 2.646954 | 1.325224 | 0.810584 | 0.576914 | 2.237665 | 0.207414 | -0.12827 | -0.58049 | -0.66158 | 0.471352 | 0.982188 | 1.228646 | 0.532205 | 0.436864 | -0.82671 | -4.65742 | 0.302041 | 0.754966 | 0.397041 | 0.82856 |
| Q6P5E8 | Q6P5E8 | DGKQ_MO | Dgkq | Diacylglycerol kinase theta | | + | | 0.269743 | -0.64201 | -0.73802 | 2.214958 | 0.419097 | 12.7493 | 0.300326 | -0.42262 | -0.81473 | -2.69492 | -3.43489 | -1.61706 | -1.57442 | -1.94767 | -1.87625 | -1.63559 | -3.2102 | -2.03986 | -1.98982 | -0.97185 | -2.00683 |
| Q80UP3 | Q80UP3 | DGKZ_MOI | Dgkz | Diacylglycerol kinase zeta | | | | 0.075086 | 0.360151 | 0.227422 | 0.418779 | -0.24039 | -1.11384 | 0.663263 | -0.54298 | -1.7794 | -4.27346 | -1.11044 | -0.2522 | -0.0027 | -0.14534 | -0.24916 | -3.13394 | -2.97026 | -0.06317 | 0.289052 | 0.045028 | 0.646433 |
| Q9QX60 | Q9QX60 | DGUOK_M | Dguok | Deoxyguanosine kinase, mitochondrial | | | | 0.169765 | -0.47342 | -0.48358 | 0.371893 | -1.5528 | -0.99462 | 0.048565 | 0.132063 | 0.150468 | -1.81428 | 0.106905 | -0.95551 | -4.06602 | -1.54566 | 0.172913 | -0.5691 | -0.19145 | -1.09404 | -0.82188 | -0.99717 | -0.63971 |
| Q6PF93 | Q6PF93 | PK3C3_M( | Pik3c3 | Phosphatidylinositol 3-kinase catalytic subunit type 3 | + | | | 2.877945 | 1.337391 | 27.44991 | 0.894463 | 0.865633 | 2.525212 | 0.024222 | -0.04359 | -0.07683 | 2.805554 | 2.901959 | 3.811232 | 4.491338 | 2.339452 | 2.426577 | 1.523456 | 1.509276 | 3.242371 | 3.328933 | 1.860908 | 2.992302 |
| E9Q5C9 | E9Q5C9 | NOLC1_M( | Nolc1 | Nucleolar and coiled-body phosphoprotein 1 | + | | | 2.377544 | 1.336956 | 15.39583 | 0.104974 | 0.124356 | 0.310907 | 0.693958 | 0.81856 | 1.870534 | 2.208761 | 2.381882 | 2.469427 | 2.902471 | 3.264749 | 2.431713 | 0.951421 | 0.96531 | 2.225288 | 2.897897 | 1.895456 | 2.163884 |
| Q99LB2 | Q99LB2 | DHRS4_M( | Dhrs4 | Dehydrogenase/reductase SDR family member 4 | | | | 0.126532 | -0.15318 | -0.36943 | 0.196936 | -0.24129 | -0.5537 | 0.106645 | 0.076466 | 0.315489 | -1.54659 | -0.82478 | -1.05226 | -1.79411 | -1.28449 | -1.41362 | -1.23663 | -0.82838 | -0.95317 | -1.41063 | -1.19191 | -1.65914 |
| Q99J47 | Q99J47 | DRS7B_M( | Dhrs7b | Dehydrogenase/reductase SDR family member 7B | | | + | 0.036943 | 0.092978 | 0.115712 | 0.098524 | -0.18032 | -0.29314 | 1.395093 | 2.095787 | 4.831864 | -4.01729 | -2.54979 | -1.06432 | -1.95995 | -2.07444 | -1.48278 | -3.049 | -3.70404 | -1.75356 | -0.91007 | -3.55719 | -4.1916 |
| Q05BC3 | Q05BC3 | EMAL1_M( | Eml1 | Echinoderm microtubule-associated protein-like 1 | + | | | 2.012624 | 1.335078 | 10.07233 | 0.777615 | 0.632428 | 2.130283 | 0.372659 | -0.61872 | -0.99656 | 4.980654 | 5.023087 | 4.376194 | 4.967121 | 2.374164 | 2.777896 | 3.797632 | 3.535952 | 4.010316 | 4.068142 | 2.607629 | 3.781862 |
| Q99PU8 | Q99PU8 | DHX30_M( | Dhx30 | ATP-dependent RNA helicase DHX30 | | | | 0.014371 | 0.048747 | 0.046056 | 0.255915 | -0.28798 | -0.70326 | 0.339467 | 0.672898 | 0.912929 | -1.70763 | -2.70639 | -2.37415 | -1.55737 | -1.77019 | -2.31788 | -1.32253 | -1.18898 | -1.64768 | -1.70787 | -3.40125 | -2.03262 |
| Q8BZS9 | Q8BZS9 | DHX32_M( | Dhx32 | Putative pre-mRNA-splicing factor ATP-dependent RNA helicase DHX32 | | | | NaN | 0 | NaN | 0.011388 | 0.016226 | 0.036612 | 0.328472 | -0.2547 | -0.88532 | -4.17287 | -4.04455 | -3.01529 | -2.45186 | -2.73655 | -3.21853 | -3.155 | -2.94747 | -3.09193 | -2.40767 | -2.87998 | -2.56569 |
| Q6P5D3 | Q6P5D3 | DHX57_M( | Dhx57 | Putative ATP-dependent RNA helicase DHX57 | | | | NaN | 0 | NaN | 0.013862 | -0.06919 | -0.04445 | 0.022524 | -0.05334 | -0.07157 | -3.88603 | -3.58627 | -2.10359 | -3.09375 | -2.26192 | -3.18517 | -3.00806 | -3.34287 | -4.00536 | -1.0536 | -3.25531 | -2.08511 |
| O70133 | O70133 | DHX9_MO | Dhx9 | ATP-dependent RNA helicase A | + | | | 1.187766 | 0.989336 | 3.731683 | 0.16741 | 0.26833 | 0.477454 | 0.465342 | 0.927932 | 1.234116 | 3.08104 | 2.759433 | 1.686455 | 2.787264 | 3.47772 | 1.998774 | 2.141685 | 1.720117 | 2.082115 | 1.854944 | 1.674179 | 1.946452 |
| O08808 | O08808 | DIAP1_MO | Diaph1 | Protein diaphanous homolog 1 | | | | NaN | 0 | NaN | 0.514668 | -0.29373 | -1.36423 | 0.284128 | -0.46738 | -0.77412 | -2.75381 | -3.22002 | -2.67865 | -2.98091 | -2.65938 | -3.60369 | -3.20895 | -3.83807 | -2.6894 | -2.38269 | -3.04043 | -2.28788 |
| O70566 | O70566 | DIAP2_M( | Diaph2 | Protein diaphanous homolog 2 | | + | + | 0.125669 | -0.25098 | -0.36711 | 1.675461 | -0.6603 | -6.77279 | 1.320553 | -0.79149 | -4.40816 | -3.86401 | -2.57868 | -1.21429 | -1.02102 | -1.7724 | -1.41395 | -2.7372 | -3.20354 | -0.44444 | -0.47027 | -0.81253 | -0.79084 |
| Q8BWT5 | Q8BWT5 | DIP2A_M( | Dip2a | Disco-interacting protein 2 homolog A | | | | NaN | 0 | NaN | 0.135089 | -0.28344 | -0.39233 | NaN | 0 | NaN | -3.04532 | -3.90151 | -3.8749 | -2.52714 | -3.36361 | -3.87788 | -3.61513 | -3.11086 | -3.17799 | -2.65718 | -2.87995 | -4.16424 |
| Q3UH60 | Q3UH60 | DIP2B_M( | Dip2b | Disco-interacting protein 2 homolog B | | + | | 0.24555 | 0.269287 | 0.677155 | 1.372231 | -0.47922 | -4.69828 | 0.082861 | 0.041063 | 0.249438 | -2.44421 | -0.27988 | 0.008623 | -0.14061 | -0.5248 | -0.22941 | -0.9286 | -0.13406 | 0.482761 | 0.343681 | -0.49087 | -0.34547 |
| E9PWR4 | Q8BWT5;E | E9PWR4_N | Dip2c | Disco interacting protein 2 homolog C | | | | 0.14291 | 0.956977 | 0.413122 | 0.368425 | -0.20855 | -0.98586 | 0.107509 | 0.092707 | 0.317856 | -0.1181 | 0.180011 | 0.277897 | -0.07516 | 0.46381 | -0.07153 | 1.38563 | -3.23767 | 0.193353 | 0.426488 | -0.01242 | 0.219281 |
| P13346 | P13346 | FOSB_MO | Fosb | Protein FosB | + | | | 1.379393 | 1.330075 | 4.739779 | 0.472016 | 2.416861 | 1.251544 | 0.276601 | 0.333317 | 0.755238 | -0.90298 | -0.54636 | -0.36371 | 0.569201 | 0.072092 | 0.509898 | -2.27143 | -1.83806 | -4.18803 | -0.44019 | -0.42555 | 0.340904 |
| Q5PR73 | Q5PR73 | DIRA2_M( | Diras2 | GTP-binding protein Di-Ras2 | | | | 0.061001 | 0.096431 | 0.186934 | 0.752839 | 0.295914 | 2.051522 | 0.089315 | 0.068238 | 0.267545 | -0.41702 | 0.069388 | 1.174374 | 0.886793 | 0.986198 | 1.359005 | 0.184683 | -0.72517 | 0.723277 | 0.746063 | 1.278418 | 0.931209 |
| Q8BMF4 | Q8BMF4 | ODP2_MO | Dlat | Dihydrolipoyllysine-residue acetyltransferase component of pyruvate dehydrogenase complex, mitochondrial | | | | 0.047874 | -0.03214 | -0.14842 | 0.673818 | 0.189048 | 1.810502 | 0.306118 | 0.225111 | 0.829251 | 4.108213 | 3.998629 | 4.27315 | 4.474705 | 4.526207 | 3.984389 | 4.295047 | 3.876071 | 4.157549 | 4.212209 | 4.01285 | 4.047523 |
| Q9D394 | Q9D394 | RUFY3_M( | Rufy3 | Protein RUFY3 | + | | | 1.376044 | 1.320086 | 4.720336 | 0.699652 | 0.491264 | 1.887675 | 0.138233 | 0.182777 | 0.400707 | 3.7528 | 4.116348 | 3.083914 | 3.582502 | 2.88268 | 2.435517 | 2.827014 | 2.401961 | 2.916654 | 2.767235 | 2.07874 | 2.873903 |
| Q811D0 | Q811D0 | DLG1_MO | Dlg1 | Disks large homolog 1 | | | | 0.106054 | 0.095928 | 0.313871 | 0.093122 | -0.03435 | -0.27816 | 0.533692 | 0.348229 | 1.415297 | -0.56136 | 0.034824 | 1.323131 | 1.17092 | 0.975859 | 0.484766 | -0.42665 | -0.29175 | 1.184121 | 1.378633 | 0.366404 | 0.397762 |
| Q91XM9 | Q91XM9 | DLG2_MO | Dlg2 | Disks large homolog 2 | | | | 0.561513 | 0.563131 | 1.490966 | 0.737256 | 0.418268 | 2.002795 | 0.308596 | 0.703584 | 0.835462 | -0.90106 | -0.26334 | 1.066798 | 1.166049 | 2.969167 | 1.34294 | -0.94289 | -1.34777 | 0.495295 | 0.901016 | 1.233238 | 1.6717 |
| P70175 | P70175 | DLG3_MO | Dlg3 | Disks large homolog 3 | | | | 0.000727 | 0.005009 | 0.002367 | 0.552489 | -0.1181 | -1.46629 | 0.008375 | 0.008353 | 0.027015 | -3.71541 | -0.33836 | 1.452222 | 1.515685 | 2.131812 | 1.732349 | -0.7561 | -0.30769 | 1.528023 | 1.676077 | 1.687694 | 2.159761 |
| Q62108 | Q62108 | DLG4_MO | Dlg4 | Disks large homolog 4 | | | | 0.754421 | 0.305574 | 2.056503 | 0.73625 | 0.368513 | 1.999672 | 0.300541 | 0.829401 | 0.815271 | 0.738672 | 0.896873 | 2.393025 | 2.630539 | 4.323896 | 2.397006 | 0.637983 | 0.386414 | 2.002349 | 2.284189 | 2.204345 | 2.857756 |
| E9Q9R9 | E9Q9R9 | DLG5_MO | Dlg5 | Disks large homolog 5 | | | | 0.040946 | 0.057888 | 0.12776 | 0.975229 | -0.39753 | -2.82374 | 0.310964 | -0.58063 | -0.8414 | -0.89316 | -1.42198 | -0.58017 | -0.31831 | -1.33534 | -0.0376 | -0.8475 | -1.58341 | 2.15E-05 | -0.10345 | -0.34073 | 0.129045 |
| Q9D415 | Q9D415 | DLGP1_M( | Dlgap1 | Disks large-associated protein 1 | | + | | 0.753809 | 1.966015 | 2.054576 | 1.897071 | 0.454808 | 8.797847 | 0.040858 | -0.0572 | -0.12749 | 0.349353 | 0.286944 | 2.833319 | 2.869909 | 2.880641 | 2.565691 | -0.69148 | -2.60425 | 2.348456 | 2.445156 | 2.360295 | 3.200428 |
| Q8BJ42 | Q8BJ42 | DLGP2_M( | Dlgap2 | Disks large-associated protein 2 | | | | 0.083615 | 0.616832 | 0.25156 | 0.210377 | -0.03902 | -0.58804 | 0.046551 | -0.07937 | -0.14449 | 0.427156 | -0.26016 | 0.967251 | 1.099371 | 1.122715 | 0.471645 | 1.894488 | -2.96116 | 1.078534 | 1.066123 | 0.434134 | 1.318959 |
| Q6PFD5 | Q6PFD5 | DLGP3_M( | Dlgap3 | Disks large-associated protein 3 | | + | | 0.011987 | 0.031827 | 0.038515 | 1.773836 | -0.32663 | -7.60993 | 0.143425 | -0.13011 | -0.41449 | -1.81691 | -0.1884 | 1.453557 | 1.486164 | 1.950469 | 1.600928 | -0.89357 | -1.17539 | 1.836196 | 1.756787 | 1.64505 | 2.166572 |
| B1AZP2 | B1AZP2 | DLGP4_M( | Dlgap4 | Disks large-associated protein 4 | | | | 0.912494 | 1.836722 | 2.58992 | 0.653224 | 0.387608 | 1.750034 | 0.316561 | -0.47476 | -0.85543 | -1.19781 | -0.30771 | 1.956039 | 1.886509 | 1.181757 | 0.917755 | -2.03733 | -3.14163 | 1.314926 | 1.752407 | 0.98545 | 2.063577 |
| Q9D2G2 | Q9D2G2 | ODO2_MO | Dlst | Dihydrolipoyllysine-residue succinyltransferase component of 2-oxoglutarate dehydrogenase complex, mitochondrial | | | | 0.933069 | -0.50559 | -2.66509 | 0.68564 | -0.60763 | -1.84563 | 0.006441 | 0.010481 | 0.020823 | 2.189598 | 2.265634 | 2.197779 | 1.651144 | 2.121449 | 2.614796 | 2.919071 | 2.547348 | 2.715631 | 2.34855 | 2.7964 | 1.918882 |
| Q9WV69 | Q9WV69 | DEMA_MO | Dmtn | Dematin | | + | | 0.356132 | -0.30968 | -0.95486 | 1.214766 | -0.64835 | -3.86171 | 0.351056 | -0.36842 | -0.94208 | -0.28684 | -0.93545 | 0.244861 | -0.03816 | 0.157 | 0.900557 | -0.29825 | -0.30469 | 0.661351 | 0.842044 | 1.01852 | 0.775878 |
| Q08274 | Q08274 | DMWD_M( | Dmwd | Dystrophia myotonica WD repeat-containing protein | | | | NaN | 0 | NaN | 0.024182 | -0.02825 | -0.07671 | 0.095002 | 0.071719 | 0.283384 | -3.26453 | -3.54937 | -0.60526 | -0.67498 | -0.96929 | -0.84203 | -3.60916 | -3.71501 | -0.97848 | -0.24527 | -1.22233 | -0.73243 |
| Q6PNC0 | Q6PNC0 | DMXL1_M( | Dmxl1 | DmX-like protein 1 | | | | 0.793779 | -1.20395 | -2.18255 | 0.402524 | 0.277089 | 1.07233 | 0.1235 | -0.05724 | -0.36127 | -2.23834 | -1.83665 | -0.54615 | -0.47411 | -0.85042 | -0.56768 | -1.3473 | -0.31978 | -1.0431 | -0.53134 | -0.58027 | -0.72335 |
| Q8BPN8 | Q8BPN8 | DMXL2_M( | Dmxl2 | DmX-like protein 2 | | | | 0.70047 | 0.283578 | 1.890142 | 0.190644 | -0.08313 | -0.53755 | 0.162439 | -0.13259 | -0.46449 | 0.68688 | 0.752593 | 2.056692 | 2.181655 | 1.970021 | 2.440206 | 0.582546 | 0.289771 | 2.060839 | 2.343771 | 2.175803 | 2.499596 |
| Q6PAR5 | Q6PAR5 | GAPD1_M( | Gapvd1 | GTPase-activating protein and VPS9 domain-containing protein 1 | + | | | 1.940112 | 1.30775 | 9.253199 | 0.274456 | -0.05525 | -0.74985 | 0.295168 | 0.269604 | 0.801802 | -0.47987 | -0.21995 | -0.13699 | -0.00179 | 0.156968 | -0.51383 | -1.7132 | -1.60212 | 0.01518 | -0.04346 | -0.42417 | -0.4719 |
| Q9QYJ0 | Q9QYJ0 | DNJA2_M( | Dnaja2 | DnaJ homolog subfamily A member 2 | | | | 0.86871 | 0.566588 | 2.434624 | 0.628305 | 0.189985 | 1.678053 | 0.246999 | -0.208 | -0.68081 | 2.754986 | 2.289547 | 4.675629 | 4.536134 | 3.61423 | 3.813742 | 1.956481 | 1.954877 | 4.505079 | 4.326715 | 3.633214 | 4.210751 |
| Q99M87 | Q99M87 | DNJA3_M( | Dnaja3 | DnaJ homolog subfamily A member 3, mitochondrial | + | | | 1.088078 | 0.748085 | 3.281713 | 0.640589 | 0.909131 | 1.713377 | 0.079536 | -0.18669 | -0.24005 | -0.87564 | -1.17518 | 0.051207 | 0.848551 | -0.2215 | -0.80577 | -1.60165 | -1.94534 | -0.8094 | -0.1091 | -1.0477 | 0.393805 |
| Q9JMC3 | Q9JMC3 | DNJA4_M( | Dnaja4 | DnaJ homolog subfamily A member 4 | | | | 0.137299 | 0.095303 | 0.398221 | 0.584092 | 0.259917 | 1.553315 | 0.580241 | -0.29474 | -1.54262 | -1.61021 | -1.56844 | 0.784549 | 0.465668 | -0.22476 | -0.10028 | -1.92303 | -1.44622 | 0.415967 | 0.314417 | -0.04842 | 0.312862 |
| Q99KV1 | Q99KV1 | DJB11_M( | Dnajb11 | DnaJ homolog subfamily B member 11 | | | | 0.057498 | -0.29914 | -0.17673 | 0.70131 | 1.038343 | 1.892681 | 0.021138 | 0.030362 | 0.06726 | -3.72506 | -1.36487 | -0.58979 | -0.28361 | -0.74333 | -1.11667 | -1.03241 | -3.45924 | -2.00186 | -0.94823 | -1.37138 | -0.54935 |
| Q9QYI4 | Q9QYI4 | DJB12_M( | Dnajb12 | DnaJ homolog subfamily B member 12 | | + | | 0.475765 | 0.523295 | 1.261356 | 1.108708 | 0.994584 | 3.371124 | 0.081576 | 0.286702 | 0.245813 | -3.60994 | -3.0842 | -1.3997 | -1.07495 | -1.61471 | -3.84474 | -4.19132 | -3.54941 | -1.98558 | -2.47823 | -2.67423 | -3.35863 |
| Q149L6 | Q149L6 | DJB14_M( | Dnajb14 | DnaJ homolog subfamily B member 14 | | + | | 0.126273 | 0.082863 | 0.368735 | 2.237484 | 0.608009 | 13.08731 | 0.151807 | 0.147173 | 0.436614 | -0.72287 | -1.0292 | -0.11599 | -0.06209 | -0.9361 | -0.37225 | -0.79446 | -1.12334 | -0.65921 | -0.73489 | -0.98611 | -0.61658 |
| Q9QYI5 | O54946;Q | DNJB2_M( | Dnajb2 | DnaJ homolog subfamily B member 2 | | + | | 0.875745 | 0.482797 | 2.459161 | 2.680707 | 0.82833 | 21.86116 | 0.243997 | -0.06828 | -0.67324 | 1.007159 | 0.631956 | 1.164111 | 1.225677 | 0.608383 | 0.532422 | 0.394635 | 0.278884 | 0.344471 | 0.388656 | 0.544644 | 0.732714 |
| Q9D832 | Q9D832;Q | DNJB4_M( | Dnajb4 | DnaJ homolog subfamily B member 4 | | | | 0.280812 | 0.891647 | 0.765802 | 0.572608 | 0.31099 | 1.521494 | 0.165809 | -0.17298 | -0.47328 | -2.08768 | -1.68009 | 0.016715 | 0.067019 | -0.80189 | -1.38573 | -3.92189 | -1.62918 | -0.47197 | -0.06628 | -1.14074 | -0.70091 |
| O89114 | O89114 | DNJB5_M( | Dnajb5 | DnaJ homolog subfamily B member 5 | | | | 0.127759 | 0.10872 | 0.372727 | 0.428281 | 0.427829 | 1.138216 | 0.017142 | -0.01496 | -0.05477 | -2.98041 | -3.256 | -0.00261 | -0.09142 | -0.60041 | -0.27885 | -2.96983 | -3.48401 | -0.84809 | -0.1016 | -0.6455 | -0.20383 |
| O54946 | O54946;Q | DNJB6_M( | Dnajb6 | DnaJ homolog subfamily B member 6 | + | | | 1.055671 | 0.671216 | 3.144944 | 0.154427 | 0.163189 | 0.443502 | 0.699659 | -0.62693 | -1.88769 | -0.86407 | -0.98641 | 1.415348 | 1.537031 | 0.654319 | 0.513048 | -1.39198 | -1.80093 | 0.950109 | 1.675891 | 0.8861 | 1.535136 |
| Q9DC23 | Q9DC23 | DJC10_M( | Dnajc10 | DnaJ homolog subfamily C member 10 | | | | NaN | 0 | NaN | 0.18582 | -0.15127 | -0.52513 | 0.186911 | -0.43035 | -0.52794 | -3.86034 | -3.99951 | -2.69402 | -2.13451 | -2.88242 | -2.70156 | -2.80988 | -3.54628 | -2.19432 | -2.33167 | -3.17175 | -1.55153 |
| Q5U458 | Q5U458 | DJC11_M( | Dnajc11 | DnaJ homolog subfamily C member 11 | | | | NaN | 0 | NaN | 0.027924 | -0.05253 | -0.08824 | 0.381545 | 0.458506 | 1.019045 | -3.80275 | -3.35847 | -2.22043 | -2.42014 | -0.81403 | -1.49495 | -3.69806 | -3.77362 | -2.85462 | -1.68089 | -1.31884 | -1.90716 |
| D4AFX7 | D4AFX7 | D4AFX7_M | Dnajc13 | DnaJ heat shock protein family (Hsp40) member C13 | | | | 0.568726 | 0.717567 | 1.51079 | 0.418404 | -0.18085 | -1.11288 | 0.498235 | -0.144 | -1.32053 | -1.3692 | -1.65038 | -0.84263 | -0.51784 | -0.87065 | -0.7226 | -1.77368 | -2.68103 | -0.49329 | -0.50547 | -0.7327 | -0.57256 |
| Q6P4T2 | Q6P4T2 | U520_MOI | Snrnp200 | U5 small nuclear ribonucleoprotein 200 kDa helicase | + | | | 2.267344 | 1.305035 | 13.54897 | 0.294418 | -0.15278 | -0.79992 | 0.407131 | 0.818372 | 1.084075 | -1.06601 | -1.14334 | -1.28096 | -1.00663 | -0.41756 | -1.37004 | -2.32149 | -2.49792 | -0.85811 | -1.12392 | -2.2979 | -1.12645 |
| P00848 | P00848 | ATP6_MOI | Mtatp6 | ATP synthase subunit a | + | | | 2.753773 | 1.299784 | 23.78548 | 0.161268 | -0.29542 | -0.46143 | 0.169789 | -0.18087 | -0.48364 | 1.128532 | 1.219306 | -0.47521 | -1.2758 | -0.19565 | 0.364026 | -0.09543 | -0.1563 | -0.08041 | -1.07975 | 0.51313 | 0.016977 |
| Q9QYI3 | Q9QYI3 | DNJC7_M( | Dnajc7 | DnaJ homolog subfamily C member 7 | | | | 0.115606 | -0.09839 | -0.33993 | 0.085311 | -0.09803 | -0.25633 | 0.388363 | -0.28766 | -1.03633 | -1.65821 | -2.1488 | -1.48542 | -0.72284 | -1.60504 | -1.48319 | -1.95874 | -1.6515 | -1.03571 | -0.97649 | -1.52726 | -0.98565 |
| P39053 | P39053 | DYN1_MO | Dnm1 | Dynamin-1 | | | | 0.020055 | 0.033549 | 0.063888 | 0.035684 | -0.04486 | -0.1119 | 0.318291 | 0.509673 | 0.859774 | 2.108778 | 2.542553 | 3.701571 | 2.91813 | 4.702106 | 3.52772 | 2.770352 | 1.813881 | 3.269411 | 3.440014 | 3.523908 | 3.686572 |
| Q8K1M6 | Q8K1M6 | DNM1L_M | Dnm1l | Dynamin-1-like protein | | | | 0.52015 | -0.23023 | -1.37889 | 0.269788 | -0.18416 | -0.73814 | 0.417743 | 0.431595 | 1.11119 | 0.729876 | 0.86345 | 1.549515 | 1.060394 | 2.351316 | 1.610822 | 1.17993 | 0.873866 | 1.538478 | 1.43975 | 1.666853 | 1.432096 |
| P39054 | P39054 | DYN2_MO | Dnm2 | Dynamin-2 | | | | 0.360856 | -0.42506 | -0.96676 | 0.718384 | -1.28293 | -1.94459 | 0.474304 | 1.33215 | 1.257531 | -3.20491 | -3.28068 | -2.48917 | -3.68401 | -1.05721 | -2.84531 | -2.3797 | -3.25577 | -1.52374 | -2.08357 | -3.85162 | -2.71519 |
| Q8BZ98 | Q8BZ98;P | DYN3_MO | Dnm3 | Dynamin-3 | | | | 0.060622 | 0.092981 | 0.185832 | 0.065022 | 0.089939 | 0.198576 | 0.521095 | 0.775303 | 1.381425 | 2.316728 | 2.623832 | 3.033288 | 2.226404 | 3.561689 | 2.449229 | 2.853504 | 1.901094 | 2.334062 | 2.745751 | 2.155377 | 2.304935 |
| Q9Z2W0 | Q9Z2W0 | DNPEP_M( | Dnpep | Aspartyl aminopeptidase | | | | 0.220714 | 0.197756 | 0.614333 | 0.444577 | 0.12799 | 1.18021 | 0.4348 | 0.222467 | 1.154984 | 0.654919 | 1.117988 | 0.685448 | 0.647898 | 0.802771 | 0.882102 | 0.912334 | 0.465062 | 0.431873 | 0.645492 | 0.808455 | 0.431483 |
| Q7TNF0 | Q7TNF0 | DOC2A_M | Doc2a | Double C2-like domain-containing protein alpha | | | | 0.019417 | -0.05484 | -0.06189 | 0.74182 | 0.386921 | 2.017003 | 0.610002 | 0.473724 | 1.625969 | -2.44395 | -3.36213 | -0.16193 | -0.53825 | -1.39565 | -1.89372 | -2.09038 | -3.60602 | -0.77436 | -0.69966 | -1.96719 | -2.26963 |
| Q8BGR8 | Q8BGR8 | GSKIP_MO | Gskip | GSK3B-interacting protein | + | | | 2.156091 | 1.298788 | 11.90591 | 0.359172 | 0.401423 | 0.962519 | 0.397155 | 0.249126 | 1.058664 | 3.989933 | 4.078226 | 3.436775 | 3.78494 | 3.494236 | 3.171639 | 2.635535 | 2.835047 | 2.830449 | 3.588419 | 2.912468 | 3.255156 |
| P59764 | P59764 | DOCK4_M | Dock4 | Dedicator of cytokinesis protein 4 | | | | 0.311783 | 0.08232 | 0.843454 | 0.15932 | -0.10849 | -0.45633 | 0.406244 | -0.35622 | -1.08181 | -0.77041 | -0.63603 | 0.386343 | 0.510589 | 0.988055 | 1.149187 | -0.71476 | -0.85659 | 0.327475 | 0.786431 | 1.105568 | 1.744114 |
| Q8R1A4 | Q8R1A4 | DOCK7_M | Dock7 | Dedicator of cytokinesis protein 7 | | | | 0.015709 | 0.037244 | 0.050273 | 0.356563 | -0.20459 | -0.95594 | 0.684243 | -0.28356 | -1.84146 | -1.95648 | -1.27127 | -0.74275 | -0.40782 | -0.9543 | -0.8727 | -0.99426 | -2.30797 | -0.23743 | -0.50396 | -0.77842 | -0.48145 |
| Q8BIK4 | Q8BIK4 | DOCK9_M | Dock9 | Dedicator of cytokinesis protein 9 | | | | 0.395107 | 0.174559 | 1.053456 | 0.224353 | -0.16476 | -0.62356 | 0.389128 | -0.34151 | -1.03827 | -0.71253 | -1.0399 | -0.04863 | 0.078439 | 0.356456 | 0.485449 | -1.02502 | -1.07653 | -0.07681 | 0.436142 | 0.439926 | 1.085006 |
| O70152 | O70152 | DPM1_MO | Dpm1 | Dolichol-phosphate mannosyltransferase subunit 1 | | | | 0.176264 | 0.317297 | 0.500448 | 0.106503 | 0.086318 | 0.3151 | 0.262193 | 0.224585 | 0.719054 | -2.12349 | -0.85583 | -1.33225 | -1.70655 | -1.30302 | -1.83311 | -1.82272 | -1.7912 | -1.40567 | -1.80576 | -1.95789 | -1.62741 |
| Q9D1Q4 | Q9D1Q4 | DPM3_MO | Dpm3 | Dolichol-phosphate mannosyltransferase subunit 3 | | + | | 0.27164 | -0.22765 | -0.74278 | 1.751217 | 1.108565 | 7.409213 | 0.339745 | 0.822024 | 0.913626 | -0.63321 | -0.30312 | -2.70175 | -2.48481 | -1.88681 | -2.34462 | 0.017717 | -0.49876 | -3.8049 | -3.59879 | -3.80787 | -2.06761 |
| Q6NXK7 | Q6NXK7 | DPP10_M( | Dpp10 | Inactive dipeptidyl peptidase 10 | | | | 0.216883 | 0.155758 | 0.6046 | 0.400177 | -0.36123 | -1.06635 | 0.160976 | 0.175534 | 0.460666 | -0.85608 | -1.27199 | 0.142383 | -0.43204 | -0.21158 | -0.85187 | -1.06773 | -1.37186 | 0.036784 | 0.396015 | -0.91391 | -0.50062 |
| Q9Z218 | Q9Z218 | DPP6_MO | Dpp6 | Dipeptidyl aminopeptidase-like protein 6 | | | | 0.081334 | 0.079391 | 0.245132 | 0.404696 | -0.27211 | -1.07786 | 0.004875 | -0.00133 | -0.01579 | 0.579746 | 0.724475 | 1.159792 | 0.760181 | 1.870298 | 1.703955 | 0.888403 | 0.257036 | 1.077792 | 1.386401 | 1.774981 | 1.801933 |
| Q99LT0 | Q99LT0 | DPY30_M( | Dpy30 | Protein dpy-30 homolog | + | | | 1.13399 | 0.989831 | 3.483276 | 0.014429 | 0.015693 | 0.04624 | 0.012699 | -0.06119 | -0.04077 | -2.12681 | -2.03521 | -1.07684 | -0.56532 | -3.47136 | -1.31689 | -3.35129 | -2.79039 | -1.05986 | -0.61369 | -3.37797 | -1.2879 |
| O08553 | O08553 | DPYL2_M( | Dpysl2 | Dihydropyrimidinase-related protein 2 | | | | 0.236957 | -0.12671 | -0.65547 | 0.096386 | 0.114262 | 0.287225 | 0.33191 | -0.17677 | -0.89395 | 3.696103 | 3.993456 | 5.034269 | 4.709136 | 4.40019 | 4.738514 | 4.095039 | 3.847939 | 4.394357 | 5.120523 | 4.848509 | 4.643727 |
| Q6P1F6 | Q6P1F6 | 2ABA_MOI | Ppp2r2a | Serine/threonine-protein phosphatase 2A 55 kDa regulatory subunit B alpha isoform | + | | | 2.044641 | 1.298354 | 10.45601 | 0.429537 | 0.232778 | 1.141442 | 0.103896 | 0.141532 | 0.307948 | 2.53518 | 2.2871 | 2.83312 | 3.030139 | 3.088354 | 2.315595 | 1.11854 | 1.107032 | 2.520288 | 2.877414 | 2.311563 | 2.809321 |
| O35098 | O35098 | DPYL4_M( | Dpysl4 | Dihydropyrimidinase-related protein 4 | | | | 0.09169 | 0.07204 | 0.274173 | 0.413205 | -0.24327 | -1.09958 | 0.299625 | -0.25293 | -0.81297 | -0.4854 | -0.01745 | 1.085588 | 0.864529 | 0.779978 | 1.146556 | -0.2039 | -0.44303 | 1.026679 | 1.409985 | 1.467581 | 0.964806 |
| Q9EQF6 | Q9EQF6 | DPYL5_M( | Dpysl5 | Dihydropyrimidinase-related protein 5 | | | | 0.186849 | -0.07902 | -0.52778 | 0.494914 | -0.32831 | -1.31175 | 0.106434 | -0.05939 | -0.31491 | 0.838665 | 0.873637 | 1.769822 | 1.269994 | 1.308429 | 1.671821 | 1.083855 | 0.786479 | 1.834555 | 1.861888 | 1.600089 | 1.498947 |
| P32233 | P32233 | DRG1_MO | Drg1 | Developmentally-regulated GTP-binding protein 1 | | | | 0.15286 | 0.125658 | 0.439384 | 0.66333 | -0.4786 | -1.77959 | 0.478616 | -0.21438 | -1.26883 | -2.41988 | -2.24009 | -2.08866 | -1.60941 | -2.09318 | -1.83854 | -2.72713 | -2.18415 | -1.49253 | -1.24835 | -1.64042 | -1.86255 |
| Q9QXB9 | Q9QXB9 | DRG2_MO | Drg2 | Developmentally-regulated GTP-binding protein 2 | | | | 0.247524 | -0.7391 | -0.68213 | 0.054548 | -0.0449 | -0.1681 | 0.153395 | -0.1653 | -0.44079 | -1.8288 | -3.7457 | -1.10676 | -0.7399 | -0.9205 | -0.88393 | -1.54279 | -2.55351 | -0.6843 | -1.07257 | -1.11147 | -0.36236 |
| Q61495;Q | Q61495;Q | DSG1A_M( | Dsg1a;Dsg1b | Desmoglein-1-alpha | | | | 0.383039 | 0.809247 | 1.02283 | 0.381065 | 0.439839 | 1.017829 | 0.048495 | 0.07808 | 0.150261 | 2.51632 | 0.973817 | 0.774278 | 0.868128 | 0.370605 | 0.739645 | 0.759345 | 1.112298 | -0.04822 | 0.810943 | -0.00872 | 0.96281 |
| E9Q557 | E9Q557 | DESP_MOI | Dsp | Desmoplakin | | | | 0.435276 | 0.557331 | 1.156209 | 0.005666 | -0.01172 | -0.01833 | 0.085256 | 0.103304 | 0.256175 | 3.539331 | 2.623832 | 2.041049 | 1.985086 | 1.746299 | 2.506915 | 2.373184 | 2.675317 | 1.386278 | 2.663291 | 1.889209 | 2.157396 |
| Q60930 | Q60930 | VDAC2_M( | Vdac2 | Voltage-dependent anion-selective channel protein 2 | + | | | 2.339337 | 1.297189 | 14.729 | 0.601369 | 0.392681 | 1.601623 | 0.418694 | 0.398135 | 1.113625 | 6.534976 | 6.466941 | 5.565338 | 5.955929 | 6.335943 | 5.677015 | 5.122534 | 5.285005 | 5.219725 | 5.51618 | 5.469533 | 5.747155 |
| Q3TDD9 | Q3TDD9 | PPR21_M( | Ppp1r21 | Protein phosphatase 1 regulatory subunit 21 | + | | | 1.446735 | 1.286956 | 5.146054 | 0.511248 | -0.28679 | -1.35511 | 0.449146 | -0.5737 | -1.19203 | -2.23655 | -1.75104 | -1.01406 | -1.43272 | -0.99252 | -1.9098 | -3.22064 | -3.34086 | -0.96777 | -0.90543 | -0.73159 | -1.02334 |
| Q9D2N4 | Q9D2N4 | DTNA_MO | Dtna | Dystrobrevin alpha | | | + | 0.205718 | 0.28513 | 0.576159 | 0.320199 | -0.16966 | -0.86456 | 2.333039 | -0.31273 | -14.6219 | -1.45282 | -0.57637 | -0.64915 | -0.27147 | -0.75556 | -0.77023 | -1.52964 | -1.06981 | -0.34401 | -0.23729 | -0.43007 | -0.47025 |
| Q80V91 | Q80V91 | DTX3_MOI | Dtx3 | Probable E3 ubiquitin-protein ligase DTX3 | | | + | 0.055359 | 0.083843 | 0.170477 | 0.268016 | -0.23782 | -0.73368 | 1.198216 | -0.64795 | -3.78157 | -3.32777 | -2.35824 | -1.58912 | -0.94825 | -1.67805 | -1.34256 | -2.84389 | -3.0098 | -1.07981 | -0.98192 | -0.89727 | -0.82744 |
| P97930 | P97930 | KTHY_MOI | Dtymk | Thymidylate kinase | | | | 0.288682 | 0.414441 | 0.78554 | 0.286456 | 0.575081 | 0.779958 | 0.205298 | 0.253204 | 0.575085 | -2.09588 | -1.9082 | -3.21115 | -2.16354 | -2.74451 | -1.87385 | -2.93565 | -1.8973 | -3.78134 | -2.74351 | -2.49648 | -2.62829 |
| Q9JLY7 | Q9JLY7 | DUS14_M( | Dusp14 | Dual specificity protein phosphatase 14 | | | | NaN | 0 | NaN | 0.455752 | -0.36323 | -1.20916 | 0.173564 | 0.185988 | 0.493448 | -3.27901 | -2.89086 | -1.25413 | -0.68367 | -0.54566 | -0.56674 | -3.22852 | -2.4667 | -0.69992 | -0.5114 | -0.36542 | -1.11896 |
| Q8R4V2 | Q8R4V2 | DUS15_M( | Dusp15 | Dual specificity protein phosphatase 15 | + | + | | 1.269153 | -0.82643 | -4.13502 | 2.354647 | -0.15544 | -14.9927 | 0.603058 | 0.8623 | 1.606375 | -1.54357 | -1.15993 | -3.66096 | -3.67062 | -2.86498 | -2.68549 | -0.4692 | -0.58144 | -3.50118 | -3.51952 | -4.16678 | -3.10829 |
| Q9D7X3 | Q9D7X3 | DUS3_MO | Dusp3 | Dual specificity protein phosphatase 3 | | | | 0.240987 | 0.565828 | 0.665646 | 0.015949 | -0.01289 | -0.05103 | 0.015804 | -0.00529 | -0.05057 | -0.44415 | -1.8762 | 0.307935 | 0.12768 | -0.16347 | 0.035846 | -2.18413 | -1.26788 | -0.00525 | 0.466642 | -0.09029 | -0.02675 |
| Q9JHU4 | Q9JHU4 | DYHC1_M( | Dync1h1 | Cytoplasmic dynein 1 heavy chain 1 | | | | 0.385801 | 0.288865 | 1.02983 | 0.307095 | 0.05751 | 0.831699 | 0.137324 | -0.11754 | -0.39829 | 2.126846 | 2.127657 | 2.668523 | 2.584926 | 2.245942 | 2.368656 | 2.118884 | 1.557889 | 2.51413 | 2.624299 | 2.136181 | 2.71349 |
| O88485 | O88485 | DC1I1_M( | Dync1i1 | Cytoplasmic dynein 1 intermediate chain 1 | | | | 0.099395 | -0.09216 | -0.29555 | 0.542324 | 0.078186 | 1.438646 | 0.153975 | 0.140997 | 0.442315 | 0.064772 | 0.184877 | 0.525064 | 0.632668 | 0.754551 | 0.453483 | 0.522965 | -0.089 | 0.508355 | 0.493006 | 0.182034 | 0.744007 |
| O88487 | O88487 | DC1I2_M( | Dync1i2 | Cytoplasmic dynein 1 intermediate chain 2 | | + | | 0.676415 | 0.225582 | 1.81819 | 1.112464 | 0.609879 | 3.387606 | 0.176914 | -0.1104 | -0.50213 | -0.81144 | -0.79762 | -0.01887 | -0.23337 | -0.47412 | -0.83989 | -0.90624 | -1.15399 | -0.88059 | -0.5914 | -0.66863 | -0.42458 |
| Q8R1Q8 | Q8R1Q8 | DC1L1_M( | Dync1li1 | Cytoplasmic dynein 1 light intermediate chain 1 | | | | 0.73057 | 0.34861 | 1.982075 | 0.110312 | 0.090505 | 0.325517 | 0.105403 | -0.0472 | -0.31209 | 0.73067 | 0.953542 | 1.539976 | 1.403753 | 1.336948 | 1.315923 | 0.629571 | 0.357422 | 1.111795 | 1.650923 | 1.222751 | 1.52453 |
| Q6PDL0 | Q6PDL0 | DC1L2_M( | Dync1li2 | Cytoplasmic dynein 1 light intermediate chain 2 | | | | 0.318551 | 0.195377 | 0.860424 | 0.110176 | 0.050242 | 0.325148 | 0.121852 | 0.018336 | 0.356831 | 0.65272 | 0.424444 | 0.813146 | 0.588428 | 0.423587 | 0.455763 | 0.146905 | 0.539505 | 0.544468 | 0.756622 | 0.372537 | 0.470141 |
| Q9D1K7 | Q9D1K7 | ADSSP_M( | Adissp | Adipose-secreted signaling protein | + | | | 1.536425 | 1.28041 | 5.735577 | 0.062917 | 0.215138 | 0.192488 | 0.479244 | -0.35646 | -1.27048 | -1.60028 | -1.63909 | -1.332 | -3.46824 | -1.86496 | -2.3813 | -3.12249 | -2.6777 | -2.28617 | -2.94434 | -1.65681 | -1.87653 |
| Q9D0M5 | Q9D0M5 | DYL2_MOI | Dynll2 | Dynein light chain 2, cytoplasmic | | | | 0.361622 | 0.122895 | 0.968695 | 0.598184 | -0.23253 | -1.59268 | 0.477444 | -0.45705 | -1.26576 | 4.209527 | 4.119104 | 3.875173 | 4.031138 | 2.921412 | 3.63938 | 3.922882 | 4.159957 | 4.309114 | 4.062257 | 3.776338 | 3.698546 |
| P62627 | P62627 | DLRB1_M( | Dynlrb1 | Dynein light chain roadblock-type 1 | | | | 0.279745 | 0.167515 | 0.763126 | 0.153652 | 0.372946 | 0.441466 | 0.262869 | -0.17709 | -0.72075 | -0.23494 | -0.54111 | 0.43535 | 0.187397 | 0.000716 | 0.361097 | -0.71286 | -0.39822 | -0.89721 | 0.774069 | 0.190958 | 0.525041 |
| P56387 | P56387 | DYLT3_M( | Dynlt3 | Dynein light chain Tctex-type 3 | | | | 0.265767 | 0.262926 | 0.728036 | 0.772087 | -0.24289 | -2.11257 | 0.849477 | 0.283261 | 2.36833 | 0.393365 | 0.627296 | 0.288851 | 0.223554 | 0.714556 | 0.927382 | 0.589084 | -0.09427 | 0.609327 | 0.38885 | 0.483107 | 0.592309 |
| Q8C008 | Q8C008 | DZAN1_M( | Dzank1 | Double zinc ribbon and ankyrin repeat-containing protein 1 | | + | | 0.483344 | 1.087011 | 1.281247 | 1.595789 | 0.458972 | 6.158979 | 0.247117 | -0.26564 | -0.68111 | -0.15073 | -0.04251 | 0.881071 | 0.960252 | -0.2655 | -0.40854 | -0.33696 | -2.0303 | 0.398555 | 0.524824 | -0.45478 | 0.312025 |
| Q9D9V3 | Q9D9V3 | ECHD1_M( | Echdc1 | Ethylmalonyl-CoA decarboxylase | + | | | 1.264631 | -0.26282 | -4.1117 | 0.891338 | -0.39083 | -2.51411 | 0.331802 | -0.20447 | -0.89367 | 0.361261 | 0.307926 | -0.29281 | -0.59972 | 0.12427 | -0.0118 | 0.539322 | 0.655505 | -0.03058 | -0.08029 | 0.042257 | 0.479158 |
| Q8VC03 | Q8VC03 | EMAL3_M( | Eml3 | Echinoderm microtubule-associated protein-like 3 | + | | | 1.909958 | 1.279808 | 8.931865 | 0.406963 | 0.303053 | 1.083645 | 0.349947 | -0.75666 | -0.93928 | 1.824081 | 1.552657 | 0.295436 | 0.764612 | -1.53357 | -0.02036 | 0.454528 | 0.362593 | 0.379217 | 0.074727 | -0.29689 | 0.256284 |
| P42125 | P42125 | ECI1_MOI | Eci1 | Enoyl-CoA delta isomerase 1, mitochondrial | | | + | 0.192422 | 0.10994 | 0.542116 | 0.128096 | -0.09914 | -0.37363 | 1.633588 | 0.261394 | 6.443423 | 0.487296 | 0.66332 | -0.66956 | -1.19583 | -0.04577 | -0.06013 | 0.282664 | 0.648072 | -0.79937 | -0.86774 | -0.35427 | -0.27441 |
| Q78JN3;Q | Q9WUR2;( | ECI2_MOI | Eci2;Eci3 | Enoyl-CoA delta isomerase 3, peroxisomal | | | | 0.272438 | -1.2204 | -0.74479 | NaN | 0 | NaN | 0.029344 | -0.12746 | -0.09259 | -3.11431 | -0.03188 | -3.46333 | -4.03203 | -3.06859 | -2.78345 | -0.90914 | 0.203752 | -3.40756 | -3.08495 | -1.42946 | -4.16767 |
| Q6PDI5 | Q6PDI5 | ECM29_M( | Ecpas | Proteasome adapter and scaffold protein ECM29 | | | | 0.471614 | 0.434024 | 1.250493 | 0.867904 | 0.569439 | 2.431822 | 0.307599 | -0.32795 | -0.83296 | -0.63427 | -0.87179 | -0.22391 | -0.09297 | -0.62004 | -1.14601 | -1.51318 | -0.86092 | -0.9527 | -0.50306 | -0.84808 | -0.26206 |
| Q3UJB9 | Q3UJB9 | EDC4_MO | Edc4 | Enhancer of mRNA-decapping protein 4 | | | | 0.086376 | 0.200621 | 0.259319 | 0.807534 | 0.64066 | 2.227603 | 0.597891 | 0.419876 | 1.591854 | -1.84983 | -3.14445 | -1.10752 | -1.45785 | -1.22256 | -1.3693 | -3.12145 | -2.27407 | -2.15145 | -1.69524 | -1.96916 | -1.46245 |
| Q8BL66 | Q8BL66 | EEA1_MOI | Eea1 | Early endosome antigen 1 | | | | 0.153984 | 0.429786 | 0.442338 | 0.06814 | 0.130937 | 0.207557 | 0.081661 | -0.17657 | -0.24605 | -1.38168 | -3.2797 | 0.006905 | -1.24303 | -0.5539 | -1.35569 | -2.55207 | -2.96887 | -0.66306 | -0.83494 | -0.18305 | -1.3734 |
| P10126 | P10126 | EF1A1_M( | Eef1a1 | Elongation factor 1-alpha 1 | | | | 0.207334 | 0.101356 | 0.580283 | 0.291479 | 0.191095 | 0.792553 | 0.509387 | 0.181621 | 1.350149 | 4.686532 | 4.765521 | 5.874717 | 5.559456 | 5.561573 | 5.73624 | 4.794813 | 4.454528 | 5.343541 | 5.708442 | 5.36497 | 5.5696 |
| P62631 | P62631 | EF1A2_M( | Eef1a2 | Elongation factor 1-alpha 2 | | | | 0.011889 | 0.025889 | 0.038203 | 0.367596 | -0.46685 | -0.98377 | 0.072986 | -0.13968 | -0.22144 | 1.358748 | 1.840171 | 2.518973 | 1.580324 | 1.672114 | 2.74118 | 2.207048 | 0.940094 | 2.586777 | 2.446229 | 2.68125 | 2.011405 |
| O70251 | O70251 | EF1B_MOI | Eef1b | Elongation factor 1-beta | | | | 0.415302 | 0.212415 | 1.104946 | 0.58976 | -0.11701 | -1.5691 | 0.705507 | -0.29983 | -1.90538 | 2.005263 | 1.723599 | 2.336549 | 2.37546 | 1.797451 | 2.092446 | 1.782869 | 1.521163 | 2.401027 | 2.54501 | 2.2996 | 2.189948 |
| P57776 | P57776 | EF1D_MO | Eef1d | Elongation factor 1-delta | | + | | 0.326699 | 0.196532 | 0.880867 | 1.03583 | 0.309447 | 3.063354 | 0.195376 | 0.179699 | 0.549696 | 0.267201 | 0.527168 | 1.150136 | 1.146826 | 1.200044 | 0.547456 | 0.019315 | 0.38199 | 0.738032 | 0.940036 | 0.714042 | 0.674061 |
| Q9D8N0 | Q9D8N0 | EF1G_MOI | Eef1g | Elongation factor 1-gamma | | | | 0.148756 | 0.189783 | 0.428575 | 0.141606 | 0.035178 | 0.409665 | 0.248525 | 0.049272 | 0.684654 | 1.864865 | 1.808712 | 2.495198 | 2.636772 | 2.134674 | 2.246505 | 2.088939 | 1.205072 | 2.482195 | 2.579418 | 2.096011 | 2.186624 |
| P58252 | P58252 | EF2_MOUS | Eef2 | Elongation factor 2 | | | | 0.034535 | -0.02835 | -0.10842 | 0.550173 | -0.24468 | -1.45997 | 0.365369 | 0.21265 | 0.978149 | 2.018289 | 1.917058 | 2.937994 | 2.630696 | 3.062775 | 2.880404 | 2.252543 | 1.739501 | 2.9621 | 3.095953 | 2.561587 | 2.956292 |
| Q8BGQ6 | Q8BGQ6 | EFC14_M( | Efcab14 | EF-hand calcium-binding domain-containing protein 14 | | | | 0.968769 | 0.632484 | 2.799021 | 0.143533 | -0.15735 | -0.41477 | 0.014219 | -0.00841 | -0.04558 | 2.857555 | 3.078372 | 1.046598 | 1.798347 | 0.839401 | 1.127349 | 2.532636 | 2.138322 | 1.631153 | 1.528491 | 0.876242 | 1.107336 |
| Q9D8Y0 | Q9D8Y0 | EFHD2_M( | Efhd2 | EF-hand domain-containing protein D2 | | + | | 0.439615 | 0.78551 | 1.167395 | 2.193138 | 1.531102 | 12.43006 | 0.463696 | 0.669788 | 1.229827 | -0.19346 | -0.28197 | 1.394243 | 1.161511 | 0.183066 | -0.89391 | -0.35181 | -1.69465 | -0.29362 | -0.21283 | -1.1067 | -0.94372 |
| O35393 | O35393 | EFNB3_M( | Efnb3 | Ephrin-B3 | | | | 0.424325 | 0.186067 | 1.128059 | 0.063196 | -0.04948 | -0.1933 | 0.17686 | -0.13221 | -0.50199 | 0.369283 | 0.113804 | -1.03078 | -0.5195 | -0.50211 | -0.48412 | -0.04888 | 0.159829 | -0.7393 | -0.712 | -0.62413 | -0.09769 |
| Q8BG67 | Q8BG67 | EFR3A_M( | Efr3a | Protein EFR3 homolog A | | | | 0.24692 | -0.42312 | -0.68061 | 0.071661 | 0.038705 | 0.217649 | 0.758986 | 0.515164 | 2.070913 | -1.24038 | -0.44177 | -0.22223 | 0.116465 | 0.585413 | 0.571302 | -0.89444 | 0.058529 | -0.03731 | -0.14587 | -0.18547 | 0.311855 |
| Q6ZQ18 | Q6ZQ18 | EFR3B_M( | Efr3b | Protein EFR3 homolog B | | | | 0.668055 | 0.194914 | 1.793488 | 0.818825 | 0.45664 | 2.264981 | 0.189652 | -0.13099 | -0.535 | 0.358676 | 0.371802 | 1.700613 | 2.102887 | 0.848634 | 1.33438 | 0.278806 | 0.061845 | 1.431324 | 1.458897 | 1.253441 | 1.191546 |
| O08810 | O08810 | U5S1_MOI | Eftud2 | 116 kDa U5 small nuclear ribonucleoprotein component | | | | 0.155998 | 0.361779 | 0.447628 | 0.332934 | 0.333848 | 0.896518 | 0.56472 | 0.718915 | 1.499771 | -1.91156 | -2.15238 | -1.54702 | -1.56997 | -0.78415 | -1.64515 | -1.59456 | -3.19294 | -2.26455 | -1.52013 | -2.14439 | -1.72275 |
| Q69ZW3 | Q69ZW3 | EHBP1_M( | Ehbp1 | EH domain-binding protein 1 | | | | NaN | 0 | NaN | 0.247317 | -0.21239 | -0.68161 | 0.975103 | -0.2401 | -2.82326 | -3.34157 | -3.04741 | -1.31654 | -0.95602 | -1.38854 | -1.35084 | -3.26844 | -3.00941 | -1.17806 | -0.66971 | -1.04666 | -1.21252 |
| Q9WVK4 | Q9WVK4 | EHD1_MO | Ehd1 | EH domain-containing protein 1 | | | | 0.752623 | 0.65487 | 2.050842 | 0.24936 | 0.122413 | 0.686758 | 0.241161 | 0.405747 | 0.666085 | -1.56597 | -0.93177 | -0.81246 | -1.05206 | 0.460732 | -0.28107 | -1.86616 | -1.94132 | -1.18666 | -0.92269 | 0.167299 | -0.79913 |
| Q9QXY6 | Q9QXY6;( | EHD3_MO | Ehd3 | EH domain-containing protein 3 | | | | 0.100741 | 0.117818 | 0.299262 | 0.282401 | 0.359012 | 0.769788 | 0.956659 | 0.810677 | 2.753081 | -0.46095 | -0.46987 | 0.54565 | -0.36518 | 1.316342 | 0.78458 | -0.18956 | -0.97689 | -0.16825 | -0.3693 | 0.366334 | 0.113235 |
| P70339 | P70339 | FRAT1_M( | Frat1 | Proto-oncogene FRAT1 | + | | | 1.802884 | 1.27153 | 7.875284 | 0.760268 | 0.709458 | 2.07497 | 0.337627 | 0.322264 | 0.908305 | 0.346358 | 0.552899 | 0.499606 | 0.624328 | 0.681618 | 0.298131 | -0.69779 | -0.94601 | -0.48367 | 0.188686 | -0.13091 | 0.466133 |
| Q9QZF2 | Q9QZF2 | GPC1_MO | Gpc1 | Glypican-1 | + | + | | 1.926217 | 1.270705 | 9.103772 | 1.594376 | 1.29497 | 6.148572 | 0.55784 | 0.583254 | 1.480904 | 1.083332 | 1.327082 | 2.576887 | 2.543714 | 2.882281 | 2.099823 | 0.002539 | -0.13353 | 1.055371 | 1.475289 | 1.953156 | 1.86244 |
| P48024;Q | Q9CXU9;F | EIF1B_MO | Eif1;Eif1b | Eukaryotic translation initiation factor 1 | | | | NaN | 0 | NaN | 0.071526 | -0.18689 | -0.21726 | 0.254774 | -0.48243 | -0.70039 | -3.75934 | -3.83134 | -3.22967 | -4.37266 | -4.60049 | -3.22457 | -3.4547 | -3.36923 | -2.97138 | -4.25717 | -3.39609 | -3.46411 |
| Q99LC8 | Q99LC8 | EI2BA_MO | Eif2b1 | Translation initiation factor eIF-2B subunit alpha | | | | 0.43214 | 1.275331 | 1.148138 | 0.240815 | -0.33276 | -0.66521 | 0.362802 | 0.814742 | 0.971673 | -1.23717 | -0.25302 | -0.30729 | -1.28198 | 1.143756 | -0.47309 | -1.02459 | -3.01627 | -0.34904 | -0.57471 | -0.70195 | -0.25687 |
| B1AUN2 | B1AUN2 | B1AUN2_N | Eif2b3 | Eukaryotic translation initiation factor 2B, subunit 3 | | | | NaN | 0 | NaN | 0.10991 | -0.24718 | -0.32442 | 0.25676 | -0.10628 | -0.70539 | -2.9454 | -4.04775 | -2.79221 | -4.15132 | -2.97757 | -3.23676 | -3.07861 | -3.38244 | -3.56912 | -2.88006 | -2.92404 | -3.07773 |
| Q61749 | Q61749 | EI2BD_MO | Eif2b4 | Translation initiation factor eIF-2B subunit delta | | | | 0.256818 | -0.88716 | -0.70553 | 0.337664 | -0.29886 | -0.9084 | 0.062972 | -0.06234 | -0.19265 | -0.96265 | -3.42011 | -0.67804 | -1.33532 | -0.24285 | -0.87946 | -1.03713 | -1.57131 | -0.72305 | -0.69259 | -0.55704 | -0.44059 |
| Q9D0T1 | Q9D0T1 | NH2L1_M( | Snu13 | NHP2-like protein 1 | + | | | 1.933088 | 1.267728 | 9.177366 | 0.394324 | 0.731529 | 1.051467 | 0.360296 | 0.639899 | 0.965353 | -1.90195 | -2.02821 | -1.95112 | -0.59277 | -1.09399 | -2.06436 | -3.10994 | -3.35567 | -2.15431 | -1.85264 | -2.67073 | -1.76742 |
| Q99L45 | Q99L45 | IF2B_MOU | Eif2s2 | Eukaryotic translation initiation factor 2 subunit 2 | | | | 0.685371 | -0.68767 | -1.84483 | 0.735969 | 0.44573 | 1.998799 | 0.539831 | 0.736512 | 1.43189 | -3.0008 | -2.44992 | -2.05915 | -1.70117 | -1.00797 | -1.37319 | -1.78653 | -2.28885 | -2.4589 | -2.19288 | -1.44624 | -2.40795 |
| Q9Z0N1 | Q9Z0N1;Q | IF2G_MOL | Eif2s3x | Eukaryotic translation initiation factor 2 subunit 3, X-linked | | | | 0.27025 | 0.181353 | 0.739295 | 0.45774 | 0.244722 | 1.214327 | 0.913176 | 0.828579 | 2.592388 | -1.40603 | -1.78788 | -0.90797 | -1.2697 | -1.29789 | -0.68482 | -1.62429 | -1.93232 | -1.42246 | -1.24466 | -1.91045 | -1.72941 |
| P23116 | P23116 | EIF3A_MO | Eif3a | Eukaryotic translation initiation factor 3 subunit A | | | | 0.983141 | 0.725936 | 2.854234 | 0.251139 | 0.223155 | 0.69124 | 0.161166 | 0.183034 | 0.461161 | -0.08 | -0.27748 | 0.446272 | 0.529612 | 0.609829 | -0.18353 | -0.67029 | -1.13906 | -0.05535 | 0.584919 | 0.043313 | 0.016921 |
| Q8JZQ9 | Q8JZQ9 | EIF3B_MO | Eif3b | Eukaryotic translation initiation factor 3 subunit B | | | | 0.069895 | 0.151938 | 0.212594 | 0.935117 | 0.248727 | 2.672654 | 0.336489 | 0.450896 | 0.905447 | -0.68948 | -0.48681 | 0.430825 | 0.257637 | 0.726214 | -0.22108 | -0.03262 | -1.44755 | 0.06141 | 0.129597 | -0.35209 | -0.04457 |
| Q01063 | Q01063 | PDE4D_M( | Pde4d | cAMP-specific 3',5'-cyclic phosphodiesterase 4D | + | | | 2.24594 | 1.248547 | 13.21645 | 0.675145 | 0.554825 | 1.81443 | 0.225241 | -0.38709 | -0.62582 | 3.412111 | 3.396657 | 4.331385 | 4.882826 | 2.996256 | 4.214948 | 2.24999 | 2.061684 | 3.92006 | 4.184502 | 3.886447 | 4.098943 |
| O70194 | O70194 | EIF3D_MO | Eif3d | Eukaryotic translation initiation factor 3 subunit D | | | | 0.002472 | -0.00249 | -0.00803 | 0.029048 | 0.024569 | 0.091689 | 0.351672 | 0.188225 | 0.943626 | -0.45639 | -0.86949 | -0.08179 | -0.02329 | 0.441286 | 0.103667 | -0.89153 | -0.42937 | -0.34347 | 0.189248 | 0.190513 | -0.02201 |
| Q91XU3 | Q91XU3 | PI42C_MO | Pip4k2c | Phosphatidylinositol 5-phosphate 4-kinase type-2 gamma | + | | | 1.505668 | 1.246957 | 5.526855 | 0.57096 | 0.365344 | 1.516947 | 0.131367 | -0.08923 | -0.38239 | 3.874431 | 4.247414 | 5.43251 | 5.879108 | 5.60416 | 5.279548 | 2.940948 | 2.686985 | 5.200232 | 5.380699 | 5.363439 | 5.698726 |
| Q9DCH4 | Q9DCH4 | EIF3F_MO | Eif3f | Eukaryotic translation initiation factor 3 subunit F | | | | 0.076976 | 0.118135 | 0.232793 | 0.299562 | 0.433823 | 0.812818 | 0.329897 | 0.210088 | 0.888894 | -0.66538 | -0.31741 | 0.664548 | 0.463736 | 0.667758 | 0.195159 | -0.13281 | -1.08624 | -0.39388 | 0.654517 | 0.226185 | 0.216556 |
| Q9Z1D1 | Q9Z1D1 | EIF3G_MO | Eif3g | Eukaryotic translation initiation factor 3 subunit G | | | | 0.079859 | -0.35829 | -0.24096 | 0.747488 | 0.418943 | 2.034721 | 0.033379 | 0.016724 | 0.104908 | -1.55081 | -3.80728 | -0.73925 | -0.60294 | -0.78851 | -1.08212 | -1.35229 | -3.28922 | -0.89575 | -1.28433 | -1.01417 | -0.8899 |
| Q91WK2 | Q91WK2 | EIF3H_MO | Eif3h | Eukaryotic translation initiation factor 3 subunit H | | | | 0.000956 | 0.004248 | 0.003109 | 0.855846 | -0.3246 | -2.39016 | 0.373142 | 0.961507 | 0.997783 | -1.41082 | -4.11787 | -1.36952 | -1.50326 | -1.97291 | -1.26767 | -2.95574 | -2.58145 | -1.23 | -0.99359 | -1.68498 | -3.47861 |
| Q9QZD9 | Q9QZD9 | EIF3I_MOL | Eif3i | Eukaryotic translation initiation factor 3 subunit I | | | | 0.02913 | -0.05542 | -0.09194 | 0.276521 | 0.273219 | 0.755038 | 0.32977 | 0.450818 | 0.888574 | -1.31646 | -1.06008 | -0.24237 | -0.47514 | 0.250008 | -0.54753 | -0.54387 | -1.72183 | -0.97461 | -0.28934 | -0.91325 | -0.28592 |
| Q6ZWN5 | Q6ZWN5 | RS9_MOUS | Rps9 | Small ribosomal subunit protein uS4 | + | | | 1.353883 | 1.244645 | 4.59343 | 0.558895 | -0.24601 | -1.48379 | 0.633806 | 0.425585 | 1.693834 | 5.020486 | 5.35629 | 4.282843 | 4.593872 | 4.619079 | 4.267315 | 4.156415 | 3.731071 | 4.741845 | 4.626887 | 3.838183 | 4.197042 |
| Q68ED7 | Q68ED7 | CRTC1_M( | Crtc1 | CREB-regulated transcription coactivator 1 | + | | + | 1.764297 | 1.243022 | 7.524664 | 0.067842 | -0.08443 | -0.2067 | 1.096738 | -0.43161 | -3.31902 | -1.79614 | -1.48482 | 0.028042 | 0.344428 | 0.177739 | 0.407594 | -2.8282 | -2.93881 | -0.10592 | 0.647247 | 0.785126 | 0.663429 |
| Q8QZY1 | Q8QZY1 | EIF3L_MOI | Eif3l | Eukaryotic translation initiation factor 3 subunit L | | | | 0.067821 | 0.035455 | 0.206639 | 0.023381 | -0.03068 | -0.07422 | 0.718077 | 0.173455 | 1.943657 | -0.00779 | -0.31688 | -0.13962 | -0.25272 | 0.074642 | -0.0046 | -0.27231 | -0.12326 | -0.57497 | 0.243991 | -0.2184 | -0.05847 |
| Q99JX4 | Q99JX4 | EIF3M_M( | Eif3m | Eukaryotic translation initiation factor 3 subunit M | | | | 0.71312 | 0.536651 | 1.928516 | 0.674328 | 0.53056 | 1.81201 | 0.092874 | 0.054923 | 0.27747 | -1.39109 | -0.94398 | -0.76027 | -0.41081 | -1.24619 | -1.52227 | -1.53848 | -1.86989 | -1.35106 | -0.88115 | -1.58102 | -1.29728 |
| P60843 | P10630;P( | IF4A1_MO | Eif4a1 | Eukaryotic initiation factor 4A-I | | | | 0.708903 | -0.48425 | -1.91568 | 0.166914 | -0.24222 | -0.47616 | 0.267067 | 0.156707 | 0.7313 | -0.0664 | -0.14899 | 0.750782 | -0.03535 | 0.640995 | 0.69048 | 0.625937 | 0.127166 | 0.277029 | 0.922851 | 0.721883 | 0.296178 |
| P10630 | P10630 | IF4A2_MO | Eif4a2 | Eukaryotic initiation factor 4A-II | | | | 0.280687 | -0.38009 | -0.76549 | 0.020594 | -0.04892 | -0.06556 | 0.025858 | -0.03716 | -0.08188 | -1.45922 | -0.50918 | -0.359 | -1.64686 | -0.42769 | -0.60583 | -0.45957 | -0.74866 | -1.33102 | -0.57699 | -0.03457 | -0.92463 |
| Q91VC3 | Q91VC3;A | IF4A3_MO | Eif4a3 | Eukaryotic initiation factor 4A-III | | | | 0.278486 | 0.509488 | 0.759968 | 5.57E-05 | -0.00014 | -0.00018 | 0.108699 | 0.125276 | 0.32111 | -1.47159 | -0.1552 | -2.14645 | -0.63973 | -1.3565 | -2.12379 | -1.19553 | -1.45024 | -1.38622 | -1.39968 | -1.93628 | -1.79456 |
| Q8BGD9 | Q8BGD9 | IF4B_MOU | Eif4b | Eukaryotic translation initiation factor 4B | | | | 0.701101 | -1.8559 | -1.89205 | 0.749556 | -0.26927 | -2.04121 | 0.060492 | -0.06857 | -0.18546 | -2.72045 | -0.90115 | 0.008679 | -0.15584 | 0.043933 | 0.635147 | -0.32191 | 0.412111 | 0.298811 | 0.092559 | 0.630217 | 0.186007 |
| P63073 | P63073 | IF4E_MOU | Eif4e | Eukaryotic translation initiation factor 4E | | | | 0.506939 | 1.503701 | 1.343634 | 0.212909 | 0.177245 | 0.594491 | 0.09237 | 0.121153 | 0.276067 | -1.24925 | -0.70633 | -0.28917 | -0.54221 | -1.13603 | -0.61326 | -1.39579 | -3.5672 | -0.8629 | -0.32296 | -1.34832 | -0.64328 |
| Q6NZJ6 | Q6NZJ6 | IF4G1_MO | Eif4g1 | Eukaryotic translation initiation factor 4 gamma 1 | | | | 0.760009 | 0.641906 | 2.074151 | 0.553583 | 0.534414 | 1.469273 | 0.33189 | 0.31515 | 0.893896 | -1.45545 | -2.03175 | -1.3969 | -1.41601 | -0.9961 | -1.69794 | -2.27262 | -2.4984 | -2.30447 | -1.57727 | -1.69613 | -1.62821 |
| Q62448 | Q62448 | IF4G2_MO | Eif4g2 | Eukaryotic translation initiation factor 4 gamma 2 | | | | 0.058563 | 0.170771 | 0.179838 | 0.263247 | -0.32034 | -0.7217 | 0.350769 | 0.330982 | 0.941353 | -3.2824 | -1.63386 | -2.30514 | -2.85517 | -1.55985 | -1.97377 | -3.10037 | -2.15744 | -2.60822 | -1.9114 | -2.38204 | -1.81356 |
| Q80XI3 | Q80XI3 | IF4G3_MO | Eif4g3 | Eukaryotic translation initiation factor 4 gamma 3 | | | | 0.284704 | 0.208668 | 0.775564 | 0.063682 | 0.042773 | 0.194704 | 0.193162 | 0.121641 | 0.544016 | -0.26105 | -0.12387 | 0.825696 | 0.886736 | 0.410556 | 0.102901 | -0.14096 | -0.66129 | 0.595891 | 1.030995 | -0.02719 | 0.297362 |
| Q9WUK2 | Q9WUK2 | IF4H_MOL | Eif4h | Eukaryotic translation initiation factor 4H | | + | | 0.081218 | -0.26088 | -0.2448 | 1.57432 | 0.33987 | 6.002639 | 0.104495 | -0.07079 | -0.30959 | -2.02609 | -3.84369 | -1.02622 | -0.94828 | -1.59138 | -1.46642 | -2.11745 | -3.23057 | -1.28605 | -1.36819 | -1.23817 | -1.67805 |
| P63242 | Q8BGY2;P | IF5A1_MO | Eif5a | Eukaryotic translation initiation factor 5A-1 | | | | 0.572862 | -0.68892 | -1.5222 | 0.169247 | -0.20336 | -0.48223 | 0.196057 | 0.215118 | 0.551442 | -1.51514 | -0.61 | -0.3932 | -1.0005 | -0.961 | -0.39756 | -0.37026 | -0.37705 | -0.78612 | -0.20087 | -0.62456 | -1.16423 |
| Q8K0G5 | Q8K0G5 | EIPR1_MO | Eipr1 | EARP and GARP complex-interacting protein 1 | | | | 0.38721 | -0.71624 | -1.0334 | 0.783288 | -0.74767 | -2.14855 | 0.128399 | -0.20279 | -0.37444 | -1.99694 | -3.37637 | -0.67039 | -1.15622 | -1.49572 | -0.63079 | -1.90213 | -2.03871 | 0.083546 | -0.41481 | -1.18648 | -0.53445 |
| P70372 | P70372 | ELAV1_M( | Elavl1 | ELAV-like protein 1 | | | | 0.600165 | 1.798739 | 1.598241 | 0.506727 | 0.586172 | 1.34307 | 0.142779 | -0.18501 | -0.41277 | -0.34392 | -0.69315 | -0.55289 | 0.316767 | -0.02721 | -0.778 | -1.20545 | -3.42909 | -0.74172 | -0.66674 | 0.027302 | -0.46249 |

| | | | | | | | | | | | | | | | | | | | | | | | | | | | | |
|---|---|---|---|---|---|---|---|---|---|---|---|---|---|---|---|---|---|---|---|---|---|---|---|---|---|---|---|---|
| Q60899 | Q61701;Q | ELAV2_MC | Elavl2 | ELAV-like protein 2 | | | | 0.549825 | 1.382058 | 1.459026 | 0.366885 | 0.302337 | 0.981976 | 0.127486 | 0.232797 | 0.371993 | 0.730353 | 0.735521 | 0.180922 | 0.786015 | 0.218657 | -0.8273 | 0.298122 | -1.59636 | 0.124038 | 0.238225 | -0.88082 | -0.19341 |
| Q60900 | Q60900 | ELAV3_MC | Elavl3 | ELAV-like protein 3 | | | | 0.58024 | 1.675293 | 1.542615 | 0.370806 | 0.386417 | 0.991877 | 0.642905 | 0.636429 | 1.720072 | -0.74039 | -0.34667 | -0.94457 | -0.19264 | -0.43057 | -1.14122 | -1.15081 | -3.28684 | -0.85292 | -1.05712 | -1.31916 | -1.5255 |
| Q61701 | Q61701 | ELAV4_MC | Elavl4 | ELAV-like protein 4 | | | | 0.721882 | 0.700643 | 1.955317 | 0.556102 | 0.474209 | 1.476153 | 0.160495 | 0.130604 | 0.459408 | 0.031435 | 0.006209 | -0.2588 | 0.33486 | -0.65637 | -1.12271 | -0.32372 | -1.03993 | -0.55903 | -0.31332 | -0.85751 | -1.18279 |
| Q68FM6 | Q68FM6;C | PPR29_MC | Elfn2 | Protein phosphatase 1 regulatory subunit 29 | | | | 0.296168 | 1.599021 | 0.804308 | 0.354031 | 0.941004 | 0.949566 | 0.219243 | 0.411291 | 0.610598 | 0.362877 | -3.61298 | 0.151762 | 0.05322 | 0.880576 | -0.24886 | -3.24769 | -3.20046 | -1.82827 | 0.151245 | -0.4626 | 0.271734 |
| Q8BPU7 | Q8BPU7 | ELMO1_M | Elmo1 | Engulfment and cell motility protein 1 | | | | 0.117315 | 0.201271 | 0.344563 | 0.360068 | -0.17002 | -0.96478 | 0.082774 | 0.191326 | 0.249192 | -0.04546 | -0.93669 | -0.15512 | 0.075592 | -0.44579 | -1.62216 | -0.31467 | -1.07002 | -0.00297 | 0.263489 | -1.7188 | -0.73181 |
| Q8BHL5 | Q8BHL5 | ELMO2_M | Elmo2 | Engulfment and cell motility protein 2 | | | | 0.365315 | 0.963022 | 0.978011 | 0.035018 | -0.02679 | -0.10988 | 0.23233 | -0.13692 | -0.64377 | -0.58137 | -0.92151 | 0.353349 | 0.501161 | 1.044744 | 0.932384 | -0.74459 | -2.68434 | 0.22171 | 0.686381 | 0.920357 | 1.330603 |
| P62869 | P62869 | ELOB_MOI | Elob | Elongin-B | | | | 0.826134 | 0.473702 | 2.289375 | 0.899036 | 0.210881 | 2.541524 | 0.833163 | -0.52982 | -2.31298 | 0.794943 | 0.935703 | 1.705979 | 1.864783 | 0.82886 | 1.201615 | 0.586197 | 0.197045 | 1.550416 | 1.598584 | 1.41189 | 1.67823 |
| P60122 | P60122 | RUVB1_M | Ruvbl1 | RuvB-like 1 | + | | | 1.876576 | 1.241812 | 8.58868 | 0.54198 | 0.337694 | 1.437711 | 0.53649 | -0.31725 | -1.42285 | -0.24983 | -0.35284 | 0.618204 | 0.796343 | -0.15023 | -0.25298 | -1.67825 | -1.40805 | 0.152239 | 0.586919 | -0.10132 | 0.332607 |
| Q7TT37 | Q7TT37 | ELP1_MOI | Elp1 | Elongator complex protein 1 | | | | 0.345353 | 1.087655 | 0.927726 | 0.402584 | -0.21044 | -1.07248 | 0.181273 | -0.09902 | -0.5134 | -1.10771 | -0.79099 | -0.9623 | -1.32273 | -0.66104 | -0.28052 | -0.87536 | -3.19865 | -1.00969 | -0.85446 | -0.40341 | -0.3401 |
| Q9CZX0 | Q9CZX0 | ELP3_MOI | Elp3 | Elongator complex protein 3 | | | | 0.067999 | -0.3251 | -0.20715 | 0.397744 | -0.16662 | -1.06016 | 0.267573 | 0.53655 | 0.732572 | -3.773 | -0.9788 | -0.94859 | -0.64685 | -0.98933 | -0.14715 | -1.33594 | -2.76568 | -0.67513 | -0.58708 | -1.70406 | -0.50553 |
| Q9ER73 | Q9ER73 | ELP4_MOI | Elp4 | Elongator complex protein 4 | | | | 0.359973 | -0.86372 | -0.96454 | 0.233161 | 0.20891 | 0.645871 | 0.142117 | 0.193091 | 0.411019 | -3.65781 | -2.79268 | -2.22619 | -1.59126 | -1.59143 | -1.82053 | -3.1456 | -1.57745 | -2.17957 | -2.05569 | -2.35468 | -1.44347 |
| Q99L85 | Q99L85 | ELP5_MOI | Elp5 | Elongator complex protein 5 | | | | NaN | 0 | NaN | 0.318951 | 0.604471 | 0.861428 | 0.374179 | 0.65768 | 1.000405 | -3.2836 | -3.50768 | -2.02549 | -2.39658 | -1.49744 | -2.00092 | -3.13995 | -3.42813 | -3.49224 | -2.13878 | -3.01416 | -1.79955 |
| Q64310 | Q64310 | SURF4_M | Surf4 | Surfeit locus protein 4 | + | + | | 1.593631 | 1.231236 | 6.143097 | 1.006795 | 0.423199 | 2.94679 | 0.199192 | 0.242105 | 0.559475 | 2.887271 | 3.26353 | 2.694042 | 2.977942 | 2.839401 | 2.391825 | 1.913287 | 1.775043 | 2.434589 | 2.390996 | 2.003132 | 2.743885 |
| Q8C7X2 | Q8C7X2 | EMC1_MO | Emc1 | ER membrane protein complex subunit 1 | | | + | 0.694251 | -0.58878 | -1.87142 | 0.346408 | 0.35882 | 0.93038 | 1.898323 | 0.546235 | 8.810786 | -1.27999 | -1.09745 | -0.75285 | -1.371 | -0.44669 | -0.53758 | -0.90103 | -0.29885 | -1.65142 | -1.19007 | -0.9962 | -1.08054 |
| Q9CRD2 | Q9CRD2 | EMC2_MO | Emc2 | ER membrane protein complex subunit 2 | | | | 0.60896 | -1.3539 | -1.62302 | 0.159971 | 0.181495 | 0.458037 | 0.084834 | 0.227915 | 0.254988 | -3.92861 | -2.94931 | -2.19651 | -2.87671 | -3.49634 | -1.71213 | -2.76042 | -1.40971 | -2.92143 | -2.51478 | -2.77668 | -2.88762 |
| O08579 | O08579 | EMD_MOL | Emd | Emerin | | | | 0.203194 | 0.458619 | 0.569711 | 0.94024 | 0.95327 | 2.691634 | 0.16163 | 0.376295 | 0.462376 | -1.93684 | -3.5354 | -1.43336 | -1.71929 | -1.29618 | -2.39839 | -3.09892 | -3.29056 | -2.20557 | -2.85362 | -1.62475 | -2.82241 |
| Q3UMT1 | Q3UMT1 | PP12C_M | Ppp1r12c | Protein phosphatase 1 regulatory subunit 12C | + | | | 1.618997 | 1.227323 | 6.332209 | 0.353823 | -0.29903 | -0.94904 | 0.041382 | -0.03611 | -0.12907 | -1.58118 | -1.53736 | -1.48276 | -1.18078 | -1.51442 | -1.24314 | -2.97917 | -2.59401 | -1.30929 | -0.75619 | -1.09801 | -1.58734 |
| Q9D1G3 | Q9D1G3 | HHATL_M | Hhatl | Protein-cysteine N-palmitoyltransferase HHAT-like protein | + | | + | 1.353591 | 1.220875 | 4.591778 | 0.344316 | 0.425944 | 0.925118 | 2.908024 | 3.776385 | 28.41904 | 0.491423 | 1.011642 | -1.11278 | -1.35962 | 0.080982 | 0.305993 | -0.52445 | -0.41424 | -1.21857 | -2.10572 | -3.65361 | -3.51219 |
| P68372 | P99024;Q | TBB4B_MC | Tubb4b | Tubulin beta-4B chain | + | | | 1.727398 | 1.214749 | 7.203274 | 0.7248 | 0.68363 | 1.964284 | 0.034859 | 0.039264 | 0.109402 | 6.90762 | 6.724252 | 8.079952 | 8.210686 | 7.688969 | 7.533336 | 5.742725 | 5.45965 | 7.119853 | 7.803525 | 7.221524 | 7.922252 |
| Q91ZR1 | P56371;Q | RAB4B_MC | Rab4b | Ras-related protein Rab-4B | + | | | 1.705111 | 1.203798 | 7.015484 | 0.263454 | 0.811412 | 0.722223 | 0.012185 | 0.029992 | 0.039141 | -1.96907 | -1.73647 | -1.23153 | -2.4133 | -1.02275 | -2.45051 | -3.18274 | -2.9304 | -3.58939 | -1.67827 | -1.48818 | -2.04507 |
| Q5SQM0;( | Q5SQM0;( | EMAL5_M | Eml5;Eml6 | Echinoderm microtubule-associated protein-like 6 | | | | 0.302756 | 0.812333 | 0.820822 | 0.120349 | -0.30366 | -0.35277 | 0.946821 | -0.52092 | -2.71615 | -3.39853 | -1.62238 | -2.70433 | -1.02024 | -2.12541 | -2.30562 | -2.88604 | -3.75954 | -1.38009 | -1.73717 | -1.8639 | -1.5253 |
| Q8BMB0 | Q8BMB0 | EMSY_MOI | Emsy | BRCA2-interacting transcriptional repressor EMSY | | | | NaN | 0 | NaN | 0.282502 | -0.06393 | -0.77004 | 0.678813 | -1.27581 | -1.82531 | -2.53125 | -2.82344 | -0.9921 | -1.10581 | -2.46721 | -1.23542 | -3.39698 | -3.27562 | -1.04552 | -0.92453 | -0.90598 | -0.24503 |
| Q03173 | Q03173 | ENAH_MO | Enah | Protein enabled homolog | | | | 0.079953 | 0.293704 | 0.241228 | 0.35554 | -0.20216 | -0.95337 | 0.079568 | -0.04289 | -0.24014 | -1.32719 | -3.75635 | -0.90364 | -1.07634 | -0.76965 | -0.62604 | -2.75061 | -2.92034 | -0.59415 | -0.9815 | -0.49143 | -0.81849 |
| Q8C522 | Q8C522 | ENDD1_M | Endod1 | Endonuclease domain-containing 1 protein | | | | 0.785808 | 0.571517 | 2.156688 | 0.571361 | 0.20483 | 1.518054 | 0.164275 | 0.181066 | 0.469282 | 2.588891 | 2.108926 | 0.33137 | 0.063322 | 0.856488 | 0.441727 | 1.889785 | 1.664998 | -0.02309 | 0.008121 | 0.142676 | 0.793407 |
| P17182 | P17182 | ENOA_MO | Eno1 | Alpha-enolase | + | | | 1.031769 | -0.9932 | -3.04685 | 0.035712 | -0.12192 | -0.11199 | 0.020654 | -0.06191 | -0.06575 | 2.259194 | 2.853914 | 4.209362 | 2.033719 | 2.739316 | 3.883296 | 3.683308 | 3.416196 | 3.287547 | 3.199377 | 4.121065 | 2.625362 |
| P17183 | P17183 | ENOG_MO | Eno2 | Gamma-enolase | | | | 0.570462 | -0.75882 | -1.51557 | 0.164395 | 0.475183 | 0.469596 | 0.059406 | 0.114763 | 0.182294 | 0.84236 | 1.836868 | 3.046639 | 1.024787 | 1.526826 | 2.087298 | 2.156924 | 2.039945 | 1.516183 | 1.604876 | 2.256037 | 1.128561 |
| P21550 | P21550 | ENOB_MO | Eno3 | Beta-enolase | | | | 0.243092 | 1.734428 | 0.670957 | 0.270035 | -1.73521 | -0.73876 | 0.256446 | 1.99206 | 0.704598 | 0.774882 | 0.710817 | -2.98234 | -3.92417 | 0.495023 | 2.109276 | 1.593229 | -3.57639 | 0.583083 | -4.01918 | 2.019659 | -3.39948 |
| Q8BGB7 | Q8BGB7 | ENOPH_M | Enoph1 | Enolase-phosphatase E1 | | | | NaN | 0 | NaN | 0.289187 | 0.690042 | 0.786807 | 0.126589 | -0.17074 | -0.36958 | -3.82422 | -3.47062 | -1.70304 | -2.7673 | -2.36477 | -3.26761 | -3.36319 | -3.99469 | -2.22808 | -3.62234 | -2.54725 | -2.74365 |
| Q8BGN3 | Q8BGN3 | ENPP6_M | Enpp6 | Glycerophosphocholine cholinephosphodiesterase ENPP6 | | | | 0.563328 | -1.71913 | -1.49595 | 0.001223 | 0.004705 | 0.003977 | 0.448212 | 0.9864 | 1.189612 | -0.60631 | -2.39506 | -1.90136 | -4.19239 | -3.31098 | -1.6659 | 0.940056 | -0.50317 | -3.34665 | -2.75652 | -3.57957 | -3.37011 |
| P53810 | P53810 | PIPNA_MC | Pitpna | Phosphatidylinositol transfer protein alpha isoform | + | | | 1.505106 | 1.200447 | 5.523104 | 0.564549 | 0.627853 | 1.499299 | 0.301865 | 0.560556 | 0.81859 | -1.82725 | -1.57749 | -0.87353 | -1.69917 | -1.89717 | -1.64353 | -3.08071 | -2.72492 | -1.98452 | -1.84389 | -1.65797 | -3.00384 |
| Q9JIX0 | Q9JIX0 | ENY2_MOI | Eny2 | Transcription and mRNA export factor ENY2 | | | | 0.166909 | 0.640622 | 0.476148 | 0.065881 | -0.10867 | -0.20105 | 0.545201 | -0.29316 | -1.44645 | -3.72226 | -1.03499 | -1.52698 | -0.50194 | -1.48016 | -1.17799 | -3.08864 | -2.94985 | -1.0775 | -0.73407 | -1.17101 | -0.90082 |
| Q9Z2H5 | Q9Z2H5 | E41L1_MC | Epb41l1 | Band 4.1-like protein 1 | | | | 0.278646 | 0.352872 | 0.760368 | 0.175791 | -0.11058 | -0.49922 | 0.151138 | 0.092158 | 0.434853 | 0.645008 | 0.792634 | 2.183485 | 2.1598 | 2.476426 | 2.138595 | 0.824121 | -0.09222 | 2.061037 | 2.503402 | 2.087362 | 2.343343 |
| O70318 | O70318 | E41L2_MC | Epb41l2 | Band 4.1-like protein 2 | | | | 0.111459 | -0.0845 | -0.32865 | 0.468225 | 0.288429 | 1.24164 | 0.338223 | 0.492737 | 0.909804 | 0.088027 | 0.266133 | 0.102753 | 0.470042 | 1.173456 | 0.090349 | 0.502794 | 0.020372 | -0.14429 | 0.140224 | 0.145088 | 0.133243 |
| Q9WV92 | Q9WV92 | E41L3_MC | Epb41l3 | Band 4.1-like protein 3 | | | | 0.508114 | 0.467073 | 1.34676 | 0.33743 | 0.128921 | 0.90781 | 0.269637 | 0.285579 | 0.737755 | 2.07933 | 2.04388 | 2.343094 | 2.328238 | 2.438018 | 1.736868 | 1.940891 | 1.248173 | 2.064927 | 2.348564 | 1.637736 | 1.965991 |
| Q8VI51 | Q8VI51 | SORC3_M | Sorcs3 | VPS10 domain-containing receptor SorCS3 | + | + | | 2.328876 | 1.196943 | 14.55145 | 1.180038 | 0.810484 | 3.695132 | 0.912139 | 0.566627 | 2.588635 | -2.47446 | -2.37928 | -0.47124 | -0.77861 | -0.29667 | -0.73081 | -3.55672 | -3.6909 | -1.5919 | -1.27891 | -1.05219 | -1.10855 |
| O54724 | O54724 | CAVN1_M | Cavin1 | Caveolae-associated protein 1 | + | + | | 1.977951 | 1.195288 | 9.672254 | 1.065311 | -0.32236 | -3.18517 | 0.186861 | -0.20821 | -0.52782 | 0.390088 | 0.554355 | 0.534392 | 0.730364 | -0.04403 | 0.467258 | -0.63073 | -0.8154 | 0.980072 | 0.929409 | 0.119401 | 0.720239 |
| P34914 | P34914 | HYES_MOI | Ephx2 | Bifunctional epoxide hydrolase 2 | | | | 0.025791 | 0.09785 | 0.081674 | 0.715497 | 0.289585 | 1.935769 | 0.06922 | 0.037613 | 0.210657 | -3.68413 | -1.75008 | -0.52256 | -0.25027 | -0.70568 | -0.88375 | -2.1077 | -3.52222 | -0.614 | -0.738 | -0.9871 | -0.67756 |
| Q8VEH5 | Q8VEH5 | EPMIP_MC | Epm2aip1 | EPM2A-interacting protein 1 | | | | 0.738794 | 0.867888 | 2.007579 | 0.071643 | -0.02349 | -0.2176 | 0.534488 | 0.376433 | 1.417445 | 0.407424 | 0.998239 | 0.579067 | 0.450477 | 0.491539 | 0.193117 | 0.150575 | -0.48069 | 0.451532 | 0.624998 | -0.2538 | 0.185586 |
| Q8CHU3 | Q8CHU3 | EPN2_MOI | Epn2 | Epsin-2 | | | | NaN | 0 | NaN | 0.783478 | -0.82279 | -2.14916 | 0.009388 | -0.00668 | -0.03025 | -4.03721 | -3.79541 | -1.62071 | -1.64006 | -1.04407 | -1.3753 | -3.58967 | -2.89085 | -0.42488 | -1.19032 | -1.05688 | -1.34912 |
| Q8R0W0 | Q8R0W0 | EPIPL_MO | Eppk1 | Epiplakin | | | | 0.037367 | 0.0901 | 0.116992 | 0.605714 | -1.50105 | -1.61386 | 0.523446 | -0.43566 | -1.38773 | -3.82337 | -2.86895 | -3.90009 | -3.16859 | -3.17141 | -3.66141 | -2.83178 | -4.04073 | -2.88845 | -1.17812 | -3.17705 | -2.78445 |
| Q8K370 | Q8K370 | ACD10_M | Acad10 | Acyl-CoA dehydrogenase family member 10 | + | | | 1.590523 | 1.194712 | 6.120287 | 0.016429 | -0.02076 | -0.05254 | 0.21664 | 0.251528 | 0.603982 | 2.263937 | 2.620967 | 0.75986 | 1.482022 | 1.601563 | 1.299888 | 1.326716 | 1.168763 | 1.302116 | 0.981282 | 0.811025 | 1.587371 |
| P42567 | P42567 | EPS15_MC | Eps15 | Epidermal growth factor receptor substrate 15 | | | | 0.098603 | -0.11796 | -0.29336 | 0.029037 | 0.023633 | 0.091656 | 0.607835 | -0.54861 | -1.61984 | -1.27298 | -1.13735 | -2.03768 | -1.57149 | -1.86084 | -2.47469 | -1.48354 | -0.69087 | -1.93845 | -1.718 | -1.76234 | -1.47596 |
| Q60902 | Q60902 | EP15R_MC | Eps15l1 | Epidermal growth factor receptor substrate 15-like 1 | | | | 0.923457 | 0.699237 | 2.629795 | 0.038291 | 0.024003 | 0.119778 | 0.517945 | -0.24576 | -1.37299 | 0.112443 | 0.231787 | -0.05767 | -0.45846 | -0.792 | -0.45485 | -0.78623 | -0.26801 | -0.28056 | -0.28358 | -0.31748 | -0.43785 |
| Q80TH2 | Q80TH2 | ERBIN_MC | Erbin | Erbin | | | | 0.659537 | 4.192321 | 1.768472 | 0.085811 | -0.08064 | -0.25773 | 0.376004 | -3.27176 | -1.00502 | 2.087158 | 3.416233 | 2.599375 | 3.114804 | 2.302556 | -4.19427 | 0.834914 | -3.71617 | 3.11516 | 2.760302 | 2.112474 | 2.53933 |
| Q6PH08 | Q6PH08 | ERC2_MOI | Erc2 | ERC protein 2 | | | | 0.055262 | 0.230397 | 0.170192 | 0.360162 | 0.14239 | 0.965014 | 0.302854 | -0.281 | -0.82107 | -1.27328 | -3.24989 | 0.00137 | -0.11761 | 0.054578 | -0.37127 | -1.56683 | -3.41713 | -0.06548 | -0.33554 | -0.14528 | 0.390581 |
| O08811 | O08811 | ERCC2_M | Ercc2 | General transcription and DNA repair factor IIH helicase subunit XPD | | | | 0.013242 | -0.1111 | -0.04249 | 0.908177 | 1.17324 | 2.574331 | 0.385261 | 2.50424 | 1.028462 | -3.92406 | 0.332882 | 1.288526 | 1.311798 | 0.312628 | 1.891294 | -0.16565 | -3.20332 | 0.582519 | -0.32867 | -3.70573 | 0.901168 |
| Q9DC16 | Q9DC16 | ERGI1_MO | Ergic1 | Endoplasmic reticulum-Golgi intermediate compartment protein 1 | | | | 0.726563 | 0.18967 | 1.969713 | 0.09945 | -0.11452 | -0.2957 | 0.454146 | 0.430183 | 1.204993 | -0.13619 | -0.30893 | -0.12337 | -0.71651 | -0.60069 | -0.0228 | -0.36966 | -0.45481 | -0.05633 | -0.55451 | -0.53226 | -0.9516 |
| P84089 | P84089 | ERH_MOU | Erh | Enhancer of rudimentary homolog | | | | 0.886105 | 1.208639 | 2.495579 | 0.481741 | 0.447275 | 1.277033 | 0.952177 | 1.061345 | 2.736213 | 0.914546 | 1.486173 | 0.141593 | 0.841969 | 1.449124 | 0.681793 | -0.39926 | 0.382705 | 0.050867 | 0.038146 | 0.061192 | -0.05296 |
| Q8C460 | Q8C460 | ERI3_MOU | Eri3 | ERI1 exoribonuclease 3 | | + | | 0.078333 | 0.060299 | 0.236644 | 1.34794 | -0.28675 | -4.55991 | 0.790993 | 0.110679 | 2.173493 | 0.840299 | 0.566938 | 0.965868 | 0.854136 | 1.186508 | 1.279048 | 0.428273 | 0.858366 | 1.167879 | 1.225619 | 1.100835 | 1.143363 |
| Q91X78 | Q91X78 | ERLN1_MC | Erlin1 | Erlin-1 | | | | 0.380296 | 0.963728 | 1.01588 | 0.992707 | 0.789616 | 2.891412 | 0.121585 | -0.28894 | -0.35611 | -1.35271 | -3.19878 | -0.75337 | -0.20773 | -0.69334 | -1.85411 | -3.45847 | -3.02047 | -1.25808 | -1.28225 | -0.4178 | -1.55178 |
| Q8BFZ9 | Q8BFZ9 | ERLN2_MC | Erlin2 | Erlin-2 | | | | 0.996052 | 0.789639 | 2.904492 | 0.799457 | 0.554094 | 2.201085 | 0.002678 | -0.00183 | -0.00869 | 1.172364 | 1.661232 | 1.539632 | 1.958973 | 1.767308 | 1.383231 | 0.508146 | 0.746172 | 1.05589 | 1.334527 | 1.491662 | 1.662532 |
| Q8QZZ7 | Q8QZZ7 | TPRKB_MC | Tprkb | EKC/KEOPS complex subunit Tprkb | + | | | 1.443021 | 1.187979 | 5.122863 | 0.00847 | -0.00517 | -0.02732 | 0.061288 | 0.07931 | 0.187766 | 2.356281 | 2.774374 | 1.709185 | 2.01476 | 2.176755 | 1.41428 | 1.477727 | 1.276969 | 1.979041 | 1.755252 | 1.534356 | 1.898059 |
| P57759 | P57759 | ERP29_MC | Erp29 | Endoplasmic reticulum resident protein 29 | | | | 0.337204 | -1.46644 | -0.90724 | 0.058162 | 0.088774 | 0.178671 | 0.23307 | 0.990422 | 0.645641 | -4.06147 | -0.83376 | -0.96445 | -1.94426 | -1.25639 | -1.40891 | -0.89109 | -1.07127 | -1.62597 | -1.46029 | -0.79095 | -3.85519 |
| Q9R0P3 | Q9R0P3 | ESTD_MOI | Esd | S-formylglutathione hydrolase | | | | 0.518595 | -1.98284 | -1.37473 | 0.560803 | 0.559996 | 1.489021 | 0.021034 | -0.06406 | -0.06694 | -0.63532 | -3.50163 | 0.102971 | -0.6461 | -1.19429 | 0.186552 | 0.076955 | -0.24823 | -0.79748 | -0.86564 | 0.222821 | -1.10245 |
| Q3U7R1 | Q3U7R1 | ESYT1_MC | Esyt1 | Extended synaptotagmin-1 | | | | 0.251305 | -0.47123 | -0.69166 | 0.136415 | 0.161966 | 0.395869 | 0.029499 | -0.06841 | -0.09307 | -3.3884 | -2.71632 | -2.66601 | -1.447 | -0.37941 | -1.84562 | -1.98846 | -3.1738 | -1.34057 | -1.09637 | -0.9913 | -1.09692 |
| Q3TZZ7 | Q3TZZ7 | ESYT2_MC | Esyt2 | Extended synaptotagmin-2 | | | | 0.022563 | 0.07366 | 0.071687 | 0.266693 | 0.225394 | 0.730361 | 0.191039 | 0.355755 | 0.538563 | -3.00367 | -2.54373 | -1.38255 | -1.44888 | -0.03446 | -1.33223 | -3.8488 | -1.84591 | -1.94793 | -1.33429 | -1.16277 | -0.91543 |
| Q8BWY3 | Q8BWY3 | ERF1_MOI | Etf1 | Eukaryotic peptide chain release factor subunit 1 | | | | 0.027867 | -0.13031 | -0.08806 | 0.160441 | -0.49538 | -0.45927 | 0.547539 | 0.937366 | 1.452806 | -3.6116 | -1.71802 | -1.41045 | -3.5148 | -1.01058 | -1.6874 | -1.39734 | -3.67165 | -2.20467 | -1.72982 | -1.73701 | -2.8357 |
| P49446 | P49446 | PTPRE_MC | Ptpre | Receptor-type tyrosine-protein phosphatase epsilon | + | | | 1.491555 | 1.18148 | 5.433418 | 0.375727 | 0.277494 | 1.00432 | 0.424093 | -0.44793 | -1.12746 | -1.9674 | -2.00046 | -1.6653 | -1.42077 | -0.71911 | -1.5085 | -2.94859 | -3.38223 | -2.06831 | -1.57275 | -0.71119 | -0.62055 |
| Q9DCW4 | Q9DCW4 | ETFB_MOL | Etfb | Electron transfer flavoprotein subunit beta | | | | 0.931409 | -1.01913 | -2.65897 | 0.242747 | -0.39292 | -0.67009 | 0.017957 | 0.045794 | 0.05733 | -0.59207 | -0.07217 | 0.323295 | -0.84173 | -0.52661 | 0.994716 | 0.405355 | 0.96867 | 0.200913 | 0.0665 | 0.432056 | -0.05554 |
| Q91WM6 | Q91WM6 | EVA1A_MC | Eva1a | Protein eva-1 homolog A | | | | 0.306607 | -0.5545 | -0.83048 | 0.304865 | -0.88621 | -0.82611 | 0.598484 | -0.76584 | -1.59352 | -2.52493 | -2.98642 | -1.72127 | -3.72009 | -2.81808 | -1.97474 | -1.57463 | -2.82773 | -2.2243 | -1.44464 | -1.39999 | -1.86115 |
| H3BKQ3 | H3BKQ3 | H3BKQ3_N | Evi5l | Ecotropic viral integration site 5 like | | + | | 0.31907 | 1.038136 | 0.861728 | 1.37491 | 0.395242 | 4.713765 | 0.781874 | 0.158696 | 2.143989 | -1.03906 | -0.4128 | 0.239516 | 0.373336 | -0.42435 | -0.39156 | -0.60076 | -2.92738 | -0.13935 | -0.03828 | -0.63883 | -0.49447 |
| Q61545 | Q61545 | EWS_MOU | Ewsr1 | RNA-binding protein EWS | | | | 0.03497 | -0.09836 | -0.10974 | 0.640764 | 0.480456 | 1.713883 | 0.044927 | 0.057148 | 0.139662 | 1.726567 | 0.7003 | 1.199183 | 1.705825 | 1.036369 | 0.936505 | 0.576937 | 2.046646 | 1.092108 | 0.851987 | 0.523162 | 1.335417 |
| Q8VEG4 | Q8VEG4 | EXD2_MOI | Exd2 | Exonuclease 3'-5' domain-containing protein 2 | | | | NaN | 0 | NaN | 0.122495 | -0.09727 | -0.35857 | 0.338214 | -0.36266 | -0.90978 | -3.04693 | -3.67392 | -3.42749 | -2.93058 | -3.16754 | -3.36929 | -3.1022 | -3.35074 | -2.97286 | -3.19068 | -3.2914 | -2.52011 |
| Q8R3S6 | Q8R3S6 | EXOC1_M | Exoc1 | Exocyst complex component 1 | | | | 0.512768 | 1.364397 | 1.359162 | 0.826519 | -0.19155 | -2.29066 | 0.890838 | 0.166932 | 2.512333 | -1.03233 | -1.22686 | -0.322 | -0.48053 | -0.16626 | -0.21394 | -1.49487 | -3.49313 | -0.18308 | -0.23635 | -0.41905 | -0.29501 |
| Q9D4H1 | Q9D4H1 | EXOC2_M | Exoc2 | Exocyst complex component 2 | | | | 0.181713 | -0.74174 | -0.51454 | 0.074036 | -0.05395 | -0.22443 | 0.111568 | -0.07333 | -0.32894 | -3.38925 | -0.53332 | -0.20261 | -0.36111 | -0.10718 | -0.12294 | -1.41707 | -1.02202 | -0.45486 | -0.00096 | -0.26453 | 0.181074 |
| Q6KAR6 | Q6KAR6 | EXOC3_M | Exoc3 | Exocyst complex component 3 | | | | 0.106647 | -0.10647 | -0.3155 | 0.994063 | -0.47604 | -2.89671 | 0.047648 | 0.031945 | 0.14775 | -1.35789 | -0.69279 | -0.14171 | -0.46669 | 0.14345 | 0.044538 | -0.86152 | -0.97622 | 0.196427 | 0.147247 | -0.14843 | 0.272526 |
| O35382 | O35382 | EXOC4_M | Exoc4 | Exocyst complex component 4 | | | | 0.597169 | -1.49617 | -1.58983 | 0.453746 | -0.17288 | -1.20395 | 0.393258 | -0.23171 | -1.04876 | -0.99485 | -2.87643 | 0.16842 | 0.040586 | 0.519357 | 0.087073 | -0.41579 | -0.46314 | 0.148799 | 0.405962 | 0.489136 | 0.580716 |
| Q3TPX4 | Q3TPX4 | EXOC5_M | Exoc5 | Exocyst complex component 5 | | | | 0.014296 | 0.016152 | 0.04582 | 0.411854 | 0.148032 | 1.096131 | 0.342719 | -0.14572 | -0.9211 | -0.68805 | -0.6821 | -0.06297 | -0.01924 | -0.26567 | -0.0558 | -1.05371 | -0.34874 | -0.05587 | -0.3224 | -0.1334 | 0.103371 |
| Q8R313 | Q8R313 | EXOC6_M | Exoc6 | Exocyst complex component 6 | | | | 0.448225 | 0.823454 | 1.189647 | 0.246791 | 0.229612 | 0.680285 | 0.082648 | -0.08955 | -0.24884 | -3.18967 | -1.80627 | -0.79241 | -1.23535 | -1.41208 | -2.06295 | -3.29549 | -3.34736 | -0.98879 | -1.49819 | -1.49431 | -1.80161 |
| A6H5Z3 | A6H5Z3 | EXC6B_M | Exoc6b | Exocyst complex component 6B | | | | 0.367535 | -0.59039 | -0.98362 | 0.387484 | -0.09248 | -1.0341 | 0.195297 | -0.08694 | -0.5495 | -1.33487 | -0.85115 | -0.65827 | -0.79872 | -0.36141 | -0.67413 | -1.05196 | 0.04671 | -0.69139 | -0.58065 | -0.45497 | -0.4067 |
| O35250 | O35250 | EXOC7_M | Exoc7 | Exocyst complex component 7 | | | | 0.646118 | 0.55558 | 1.729377 | 0.125159 | 0.048764 | 0.36574 | 0.312405 | -0.2272 | -0.84501 | -0.91758 | -0.7458 | 0.060436 | -0.11365 | -0.54471 | -0.21898 | -1.69684 | -1.0777 | -0.17637 | 0.025628 | -0.36857 | 0.059287 |
| Q6PGF7 | Q6PGF7 | EXOC8_M | Exoc8 | Exocyst complex component 8 | | | | 0.30486 | -0.43039 | -0.8261 | 0.745944 | 0.377217 | 2.029885 | 0.253506 | 0.273275 | 0.697199 | -1.22759 | -2.23801 | -0.61179 | -0.80148 | -0.00573 | -0.78026 | -1.42966 | -1.17518 | -1.24366 | -0.92405 | -0.60577 | -0.72678 |
| Q91WG5 | O54950;Q | AAKG2_M | Prkag2 | 5'-AMP-activated protein kinase subunit gamma-2 | + | + | | 2.147573 | 1.181297 | 11.78849 | 1.323504 | 1.155943 | 4.424295 | 0.492965 | 0.2784 | 1.306599 | 7.197197 | 7.225339 | 8.165695 | 8.462591 | 7.7516 | 7.993845 | 6.129187 | 5.930757 | 6.943198 | 7.373203 | 7.419024 | 7.76962 |
| P26040 | P26040;P2 | EZRI_MOU | Ezr | Ezrin | | | | 0.493918 | -0.52956 | -1.30911 | 0.636712 | -0.83719 | -1.70219 | 0.222915 | -0.1382 | -0.61992 | -1.0488 | -1.61538 | -0.70039 | -1.68201 | -0.63317 | -1.04125 | -0.51378 | -1.09129 | -0.32238 | -0.38563 | -0.60923 | -0.7888 |
| Q00558 | Q00558 | HAP40_M | F8a1 | 40-kDa huntingtin-associated protein | | + | | 0.021479 | -0.05159 | -0.06832 | 1.181587 | 0.745261 | 3.702436 | 0.339441 | -0.31561 | -0.91286 | -2.19445 | -3.70452 | -0.3396 | -0.47783 | -2.0926 | -1.45518 | -2.89188 | -2.90391 | -1.34303 | -0.96493 | -1.59228 | -1.32427 |
| O08914 | O08914 | FAAH1_M | Faah | Fatty-acid amide hydrolase 1 | + | | | 1.174884 | 0.430954 | 3.67092 | 0.2986 | -0.27088 | -0.81041 | 0.71504 | -0.17654 | -1.93437 | -0.4575 | -0.43867 | 0.048471 | -0.17784 | 0.677486 | 0.495648 | -0.99605 | -0.76202 | -0.10832 | 0.520722 | 0.771038 | 0.755176 |
| Q05816 | Q05816 | FABP5_M | Fabp5 | Fatty acid-binding protein 5 | | | + | 0.272845 | -0.33637 | -0.74581 | 0.248168 | 1.654914 | 0.683756 | 1.819637 | -0.87335 | -8.03231 | -0.86961 | -0.32602 | 0.910262 | -2.86653 | -1.02979 | -1.15445 | -0.62136 | 0.098466 | -1.11913 | -4.14696 | -0.12968 | -0.30786 |
| P51880 | P51880 | FABP7_M | Fabp7 | Fatty acid-binding protein, brain | + | | | 1.29308 | -0.6813 | -4.26031 | 0.061512 | -0.2192 | -0.18842 | 0.136432 | -0.24831 | -0.39591 | -0.25187 | 0.038115 | -0.28478 | -2.59325 | -1.15293 | -0.2246 | 0.641891 | 0.50696 | -1.36541 | -1.07422 | -0.01866 | -0.86225 |
| Q920L1 | Q920L1 | FADS1_M | Fads1 | Acyl-CoA (8-3)-desaturase | | | | 0.122267 | 0.078923 | 0.357951 | 0.374438 | -0.52724 | -1.00106 | 0.638436 | 0.241775 | 1.707166 | 1.78225 | 1.447883 | 0.25436 | -0.29863 | 0.869941 | 0.587509 | 1.392393 | 1.679893 | 0.953373 | 0.056837 | 0.476211 | 0.497688 |
| Q9Z0R9 | Q9Z0R9 | FADS2_M | Fads2 | Acyl-CoA 6-desaturase | | | | 0.930577 | -0.25023 | -2.65591 | 0.163481 | -0.13187 | -0.46721 | 0.011499 | 0.011895 | 0.036964 | -0.05375 | -0.2362 | -1.23486 | -0.87877 | 0.183319 | -0.40613 | 0.081715 | 0.128795 | -1.14395 | -0.70594 | -0.25249 | 0.005884 |
| Q3TDN2 | Q3TDN2 | FAF2_MOI | Faf2 | FAS-associated factor 2 | | | | 0.097115 | 0.225491 | 0.289243 | 0.166989 | -0.28179 | -0.47636 | 0.197756 | 0.433618 | 0.555796 | -2.56809 | -4.08458 | -3.09452 | -4.13979 | -1.69166 | -3.15751 | -3.73301 | -3.37064 | -3.61245 | -3.0583 | -3.12559 | -2.59083 |
| Q8C6G1 | Q8C6G1 | CF410_MC | Cfap410 | Cilia- and flagella-associated protein 410 | + | | | 2.135704 | 1.178721 | 11.62675 | 0.449051 | 0.578288 | 1.191784 | 0.427392 | -0.75884 | -1.13593 | 0.947384 | 0.829347 | 0.618415 | 1.522227 | -1.29419 | -0.49003 | -0.37279 | -0.20793 | 0.668747 | 0.31532 | -0.66675 | 0.400209 |
| P70347 | P70347 | TANK_MOI | Tank | TRAF family member-associated NF-kappa-B activator | + | | | 1.555071 | 1.17679 | 5.86559 | 0.762507 | 0.664018 | 2.082066 | 0.348808 | -1.05703 | -0.93642 | 2.733156 | 2.845065 | 2.827226 | 3.455432 | 0.388692 | 2.639481 | 1.804985 | 1.419654 | 2.422075 | 2.532547 | 2.483422 | 2.65882 |
| Q3U3E2 | Q3U3E2 | F117B_MC | Fam117b | Protein FAM117B | | | | 0.022269 | -0.03714 | -0.07078 | 0.507929 | -0.24335 | -1.34627 | 0.086457 | -0.1046 | -0.25954 | -2.3821 | -2.13355 | -1.0134 | -0.65215 | -1.54487 | -0.73994 | -1.71091 | -2.73046 | -0.58253 | -0.59633 | -1.01694 | -1.05867 |
| Q6A0A9 | Q6A0A9 | F120A_MC | FAM120A | Constitutive coactivator of PPAR-gamma-like protein 1 | | | | 0.0484 | 0.067382 | 0.149978 | 0.541328 | 0.199175 | 1.435945 | 0.151317 | 0.140285 | 0.435325 | -1.27615 | -1.25308 | 0.176718 | 0.340269 | 0.07126 | -0.36864 | -1.78113 | -0.88287 | -0.05272 | 0.171356 | -0.52449 | -0.05345 |
| Q6RI63 | Q6RI63 | F120B_MC | Fam120b | Constitutive coactivator of peroxisome proliferator-activated receptor gamma | | + | + | 0.507793 | 1.675137 | 1.345905 | 1.283816 | -0.40986 | -4.21143 | 1.865459 | -0.49 | -8.47722 | -0.77713 | -1.20294 | -1.15209 | -1.27777 | -1.65941 | -1.63601 | -1.4389 | -3.89144 | -0.87938 | -0.73075 | -1.21432 | -1.10111 |
| Q8C3F2 | Q8C3F2 | F120C_MC | Fam120c | Constitutive coactivator of PPAR-gamma-like protein 2 | | | | 0.165513 | 0.415612 | 0.472512 | 0.835738 | 0.501192 | 2.321661 | 0.205118 | 0.289494 | 0.574627 | -1.5489 | -1.66063 | -0.60366 | -0.42648 | -0.51643 | -0.96367 | -1.14257 | -2.89818 | -1.21312 | -0.8194 | -1.48099 | -0.5781 |
| Q8BWU3 | Q8BWU3 | F131A_MC | Fam131a | Protein FAM131A | | | + | NaN | 0 | NaN | 0.277349 | -0.92549 | -0.75711 | 1.52144 | -1.1952 | -5.633 | -3.05473 | -3.14052 | -1.63653 | -3.92023 | -2.09555 | -2.51859 | -3.74456 | -3.4462 | -2.28927 | -1.4165 | -1.0952 | -1.12854 |
| Q3TY60 | Q3TY60 | F131B_MC | Fam131b | Protein FAM131B | | + | | 0.363356 | 0.743916 | 0.97307 | 1.916749 | 1.472666 | 9.003281 | 0.089764 | 0.128877 | 0.268802 | 0.423907 | -1.03611 | 0.168786 | 0.17585 | -0.23672 | -1.06433 | -0.82296 | -1.27707 | -1.13682 | -1.46388 | -1.02155 | -0.53725 |
| Q9CVI2 | Q9CVI2 | F133B_MC | Fam133b | Protein FAM133B | | | | 0.328008 | 0.440883 | 0.884151 | 0.664145 | 0.38074 | 1.781985 | 0.047126 | 0.022317 | 0.146201 | -0.1848 | -0.03877 | -0.38892 | 0 | -0.29723 | -0.40517 | -1.04595 | -0.05939 | -0.48668 | -0.66372 | -0.5163 | -0.23073 |
| Q9DBR2 | Q9DBR2 | FA13C_MC | Fam13c | Protein FAM13C | | | | NaN | 0 | NaN | 0.3724 | 0.281382 | 0.995905 | 0.339104 | -0.36034 | -0.91202 | -3.49292 | -3.15347 | -1.67486 | -2.2338 | -2.90758 | -2.11971 | -3.38405 | -3.21903 | -2.19417 | -2.27725 | -2.18369 | -2.12291 |
| Q7TN79 | Q7TN79 | AKA7G_M | Akap7 | A-kinase anchor protein 7 isoform gamma | + | | | 2.293639 | 1.172026 | 13.96876 | 0.052867 | 0.028459 | 0.163162 | 0.509694 | -0.35496 | -1.35097 | 1.820135 | 1.652331 | 1.356259 | 1.523275 | 0.743834 | 0.445691 | 0.564707 | 0.563706 | 1.564441 | 1.258174 | 1.16609 | 0.73336 |
| A2ATK9 | A2ATK9 | A2ATK9_M | Fam171a1 | Family with sequence similarity 171, member A1 | | | | 0.537244 | -1.14984 | -1.42489 | 0.851429 | -0.48197 | -2.37501 | 0.569936 | -0.53349 | -1.51412 | -3.67938 | -4.25663 | -1.67384 | -1.76723 | -2.36993 | -1.78858 | -2.06458 | -3.57175 | -1.04107 | -1.43605 | -1.74489 | -1.34662 |
| A2A699 | A2A699 | F1712_MC | Fam171a2 | Protein FAM171A2 | | | | 0.236709 | -0.41334 | -0.65484 | 0.661355 | -0.30422 | -1.7738 | 0.506776 | -0.38302 | -1.3432 | -2.3437 | -3.59307 | -0.48115 | -0.67017 | -1.4199 | -1.01821 | -2.64554 | -2.46455 | -0.41456 | -0.12832 | -1.03846 | -0.63362 |
| Q8BR63 | Q8BR63 | F177A_MC | Fam177a1 | Protein FAM177A1 | | | | 0.515965 | 0.264529 | 1.367696 | 0.286921 | 0.337213 | 0.781126 | 0.096033 | 0.086994 | 0.286246 | 1.214802 | 1.135383 | -0.03958 | -0.13316 | -0.18703 | 0.278805 | 1.099855 | 0.721271 | -0.85274 | 0.005573 | -0.23633 | 0.15412 |
| A2AJW5 | A2AJW5 | A2AJW5_M | Fam217b | Family with sequence similarity 217, member B | | | | 0.064555 | -0.27538 | -0.19723 | 0.254051 | 0.106744 | 0.698573 | 0.769572 | -0.5954 | -2.10454 | -1.30107 | -3.46268 | 0.161601 | 0.466058 | -1.04009 | -0.47451 | -1.22252 | -2.99047 | 0.220323 | 0.193847 | -0.15355 | -0.17025 |
| Q3UXZ6 | Q3UXZ6 | FA81A_MC | Fam81a | Protein FAM81A | | | | NaN | 0 | NaN | 0.7494 | 0.171306 | 2.040716 | 0.121066 | -0.25414 | -0.35471 | -3.2968 | -3.72373 | -0.59318 | -0.4364 | -0.7061 | -1.86872 | -2.85135 | -2.75158 | -0.71612 | -0.65606 | -1.45208 | -0.61447 |
| Q3URQ4 | Q3URQ4 | Q3URQ4_I | Fam8a1 | Family with sequence similarity 8, member A1 | | + | | 0.013783 | -0.04174 | -0.0442 | 1.027402 | 1.179327 | 3.029176 | 0.419053 | -1.28042 | -1.11455 | -4.23488 | -2.39524 | -1.38499 | -1.44861 | -4.0542 | -1.75744 | -3.05933 | -3.48731 | -2.98415 | -2.2081 | -1.59319 | -1.65761 |
| Q6NXH9 | Q6NXH9 | K2C73_MC | Krt73 | Keratin, type II cytoskeletal 73 | + | | | 1.566614 | 1.170601 | 5.947421 | 0.021156 | -0.02712 | -0.06732 | 0.546336 | 0.61185 | 1.449535 | 1.629426 | 1.799801 | -0.12814 | 0.238669 | 0.476591 | 0.641387 | 0.366578 | 0.721447 | 0.441015 | -0.27625 | -0.46684 | 0.361119 |
| Q7TSH8 | Q7TSH8 | TMM94_M | Tmem94 | Transmembrane protein 94 | + | | | 1.73633 | 1.153837 | 7.279858 | 0.599828 | 0.429895 | 1.597294 | 0.073358 | -0.08976 | -0.2225 | 3.84151 | 3.643561 | 3.857297 | 4.388951 | 2.959368 | 2.84408 | 2.712494 | 2.464902 | 3.735325 | 3.651134 | 2.592198 | 3.390774 |
| Q922J9 | Q922J9 | FACR1_M | Far1 | Fatty acyl-CoA reductase 1 | | + | | 0.12337 | 0.173166 | 0.360925 | 1.466828 | 0.510806 | 5.273138 | 0.648896 | 0.246845 | 1.73744 | -1.63718 | -1.11801 | -1.75209 | -1.57836 | -1.59021 | -1.6747 | -1.95426 | -1.14726 | -2.2189 | -2.13315 | -2.01495 | -1.74365 |
| Q9D1H7 | Q9D1H7 | GET4_MOI | Get4 | Golgi to ER traffic protein 4 homolog | + | | | 1.888928 | 1.152587 | 8.714164 | 0.005405 | -0.00793 | -0.01749 | 0.083074 | 0.199081 | 0.250037 | -1.52187 | -1.42332 | 0.290878 | -0.4144 | 0.361864 | -1.07864 | -2.50243 | -2.74792 | -0.33848 | 0.230811 | -0.89685 | -0.21809 |
| Q8C0C7 | Q8C0C7 | SYFA_MOI | Farsa | Phenylalanine--tRNA ligase alpha subunit | | | | 0.048674 | 0.014112 | 0.150792 | 0.081284 | -0.05421 | -0.24499 | 0.106062 | 0.110596 | 0.313892 | 0.677126 | 0.739072 | 1.458904 | 1.180132 | 1.783632 | 1.391423 | 0.782298 | 0.605677 | 1.201883 | 1.545569 | 1.18421 | 1.769652 |
| Q9WUA2 | Q9WUA2 | SYFB_MOI | Farsb | Phenylalanine--tRNA ligase beta subunit | | | | 0.033028 | -0.01456 | -0.10384 | 0.666554 | -0.43576 | -1.78907 | 0.711665 | 0.135443 | 1.924082 | 0.393535 | 0.498173 | 0.379017 | -0.06541 | 0.906843 | 0.800887 | 0.330328 | 0.5905 | 0.692301 | 0.49283 | 0.764774 | 0.672069 |
| P19096 | P19096 | FAS_MOU | Fasn | Fatty acid synthase | | | | 0.092073 | 0.070434 | 0.275242 | 0.384565 | -0.05761 | -1.0267 | 0.479646 | 0.316954 | 1.271534 | 1.18824 | 1.060965 | 1.424089 | 1.375091 | 1.858969 | 1.410361 | 1.302029 | 0.806307 | 1.406719 | 1.507679 | 1.208979 | 1.426443 |
| P62862 | P62862 | RS30_MOI | Fau | Ubiquitin-like FUBI-ribosomal protein eS30 fusion protein | | | | 0.246631 | 0.471789 | 0.679882 | 0.397606 | 0.162686 | 1.059812 | 0.114575 | 0.080012 | 0.337128 | 2.270711 | 2.848036 | 2.702649 | 2.878005 | 2.613326 | 2.430982 | 2.718623 | 1.456546 | 2.753642 | 2.501641 | 2.223018 | 2.661266 |
| P35550 | P35550 | FBRL_MOI | Fbl | rRNA 2'-O-methyltransferase fibrillarin | | | + | 0.438292 | 0.299888 | 1.163981 | 0.456071 | 0.507329 | 1.20999 | 1.667832 | 0.684914 | 6.71163 | 0.201832 | -0.18005 | -0.8262 | -0.007 | 0.288953 | 0.110097 | -0.11603 | -0.46197 | -0.83434 | -1.01352 | -0.43623 | -0.53455 |
| Q8R089 | Q8R089 | FBRS_MOI | Fbrs | Probable fibrosin-1 | | | | 0.478742 | 0.648268 | 1.26916 | 0.635932 | -0.59272 | -1.69995 | 0.713594 | 0.952346 | 1.929961 | -0.70139 | 0.319962 | -0.54047 | -0.18505 | -0.22965 | -0.51856 | -0.8496 | -0.82836 | -0.07002 | 0.529939 | -1.79828 | -0.85462 |
| A2RT62 | A2RT62 | FXL16_MC | Fbxl16 | F-box/LRR-repeat protein 16 | | | | 0.157421 | 0.043238 | 0.451359 | 0.01012 | 0.012583 | 0.032582 | 0.334546 | -0.30267 | -0.90057 | -1.04979 | -0.94725 | 1.229943 | 1.826336 | 1.294881 | 0.943669 | -1.12268 | -0.96083 | 1.270146 | 1.760967 | 1.135383 | 1.708506 |
| Q9QZN1 | Q9QZN1;C | FXL17_MC | Fbxl17 | F-box/LRR-repeat protein 17 | | + | | 0.592432 | 0.669138 | 1.576568 | 1.725979 | 0.768003 | 7.191172 | 0.143976 | 0.392691 | 0.415943 | -0.72878 | -0.24693 | -1.27416 | -1.46331 | -1.36171 | -2.35452 | -0.80758 | -1.50642 | -2.08712 | -2.18635 | -3.05386 | -1.44775 |
| Q9DBG3 | Q9DBG3;C | AP2B1_MC | Ap2b1 | AP-2 complex subunit beta | + | | | 1.455557 | 1.144513 | 5.201507 | 0.739972 | 0.636163 | 2.011244 | 0.362332 | -0.52713 | -0.97049 | 4.762234 | 4.708027 | 6.067098 | 6.393856 | 3.942829 | 4.925193 | 3.808977 | 3.372259 | 5.323473 | 5.865156 | 4.729273 | 5.193007 |
| O70172 | O70172 | PI42A_MO | Pip4k2a | Phosphatidylinositol 5-phosphate 4-kinase type-2 alpha | + | | | 2.037228 | 1.143112 | 10.36593 | 0.522877 | 0.32148 | 1.386201 | 0.158177 | 0.169557 | 0.45334 | 1.552819 | 1.344486 | 1.545889 | 2.001551 | 1.520292 | 0.837668 | 0.341736 | 0.269345 | 1.495569 | 1.408912 | 0.856466 | 1.162379 |
| Q78JE5 | Q78JE5 | FBX22_MC | Fbxo22 | F-box only protein 22 | + | | | 1.012125 | 0.864983 | 2.967943 | 0.870819 | 0.345763 | 2.441964 | 0.350144 | -0.20889 | -0.93978 | -0.95221 | -0.50978 | -0.45018 | -0.73286 | -1.27973 | -1.18733 | -1.40623 | -1.78572 | -0.94571 | -0.92885 | -1.24206 | -0.80723 |
| Q9DC63 | Q9DC63 | FBX3_MOI | Fbxo3 | F-box only protein 3 | | + | | 0.940816 | 2.575168 | 2.693773 | 1.195158 | 0.950732 | 3.766919 | 0.345864 | -0.42071 | -0.92901 | 0.001346 | 0.25111 | 0.562374 | 0.656816 | -0.11406 | -0.67603 | -1.50116 | -3.39672 | -0.58907 | -0.0932 | -0.32947 | 0.380796 |
| Q6NS60 | Q6NS60 | FBX41_MC | Fbxo41 | F-box only protein 41 | | | | 0.510855 | 1.623863 | 1.354059 | 0.240103 | 0.079881 | 0.663413 | 0.087062 | -0.1665 | -0.26124 | -0.82455 | -1.31428 | 0.328075 | 0.246648 | 0.307472 | -0.5989 | -1.51929 | -3.86727 | 0.094163 | 0.320797 | -0.42734 | 0.468908 |
| Q9QZN4 | Q9QZN4 | FBX6_MOI | Fbxo6 | F-box only protein 6 | | + | | 0.349724 | 1.640009 | 0.938722 | 1.112571 | 0.651197 | 3.388076 | 0.592177 | -0.27274 | -1.57585 | -0.37186 | -0.77489 | 0.09723 | 0.081371 | -0.89453 | -1.08351 | -0.47798 | -3.94879 | -0.36986 | -0.75394 | -0.57128 | -0.86129 |
| Q5SRY7 | Q5SRY7 | FBW1B_M | Fbxw11 | F-box/WD repeat-containing protein 11 | | | | 0.951929 | 0.476304 | 2.735279 | 0.470634 | 0.529464 | 1.247931 | 0.286095 | -0.3272 | -0.77905 | 0.224691 | -0.08989 | 0.433548 | 1.275133 | -0.48093 | 0.344071 | -0.48362 | -0.33419 | 0.270635 | 0.379117 | 0.179767 | 0.337776 |
| Q569Z6 | Q569Z6 | TR150_MC | Thrap3 | Thyroid hormone receptor-associated protein 3 | + | | | 1.473754 | 1.141734 | 5.317596 | 0.474167 | 0.217872 | 1.257173 | 0.719642 | 0.697748 | 1.94845 | 2.738363 | 2.911854 | 2.009355 | 2.355947 | 3.060001 | 2.361113 | 1.87978 | 1.486969 | 1.966362 | 1.963195 | 1.93453 | 2.091087 |
| P17427 | P17427 | AP2A2_MC | Ap2a2 | AP-2 complex subunit alpha-2 | + | | | 1.404009 | 1.141575 | 4.884863 | 0.608153 | 0.513901 | 1.620743 | 0.058196 | 0.043957 | 0.17877 | 3.98663 | 4.224017 | 5.053993 | 5.100917 | 4.330607 | 4.820087 | 3.165059 | 2.762438 | 4.247346 | 4.879762 | 4.555096 | 4.507685 |
| P22315 | P22315 | HEMH_MC | Fech | Ferrochelatase, mitochondrial | | | | 0.163117 | -0.5271 | -0.46626 | 0.703225 | 0.83024 | 1.89847 | 0.006442 | 0.01002 | 0.020824 | -3.35376 | -2.03571 | -1.1779 | -1.34563 | -1.60153 | -1.11448 | -3.08616 | -1.24911 | -2.52121 | -1.6628 | -0.95301 | -1.78304 |
| Q8CIB5 | Q8CIB5 | FERM2_M | Fermt2 | Fermitin family homolog 2 | | | | 0.589815 | -0.66729 | -1.56926 | 0.009754 | 0.019147 | 0.031417 | 0.96262 | 0.567049 | 2.775627 | -1.53162 | -1.94306 | -1.20698 | -2.31101 | -0.8261 | -1.09781 | -0.69789 | -1.4422 | -2.03641 | -1.51987 | -1.68158 | -1.37643 |
| Q8K0X8 | Q8K0X8 | FEZ1_MOL | Fez1 | Fasciculation and elongation protein zeta-1 | | | | NaN | 0 | NaN | 0.147966 | 0.130991 | 0.42649 | 0.240415 | -0.5052 | -0.6642 | -3.98436 | -3.82435 | -2.61398 | -2.41013 | -4.2003 | -2.68061 | -3.36613 | -3.70016 | -2.93278 | -2.35331 | -2.96948 | -2.90103 |
| Q99NH0 | Q99NH0 | ANR17_M | Ankrd17 | Ankyrin repeat domain-containing protein 17 | + | | | 2.066361 | 1.131895 | 10.72434 | 0.165491 | -0.13416 | -0.47245 | 0.697995 | -0.82214 | -1.88268 | -2.03285 | -1.82177 | -0.77528 | -0.70222 | -1.87606 | -1.07961 | -3.05832 | -3.0601 | -0.8862 | -0.32298 | -0.83489 | -0.4765 |
| Q69ZL1 | Q69ZL1 | FGD6_MO | Fgd6 | FYVE, RhoGEF and PH domain-containing protein 6 | | | + | 0.195437 | -0.4453 | -0.54985 | 0.772238 | -1.13113 | -2.11305 | 1.050348 | -1.41865 | -5.1229 | -2.84331 | -3.34136 | -2.56869 | -1.4988 | -2.18555 | -2.84318 | -1.87643 | -3.41765 | -0.88302 | -0.9222 | -0.78228 | -1.40916 |
| P61148 | P61148 | FGF1_MOI | Fgf1 | Fibroblast growth factor 1 | | | | 0.963741 | 0.626436 | 2.779881 | 0.590371 | 0.300316 | 1.570809 | 0.63906 | -0.28162 | -1.70896 | 4.146384 | 4.260364 | 3.566209 | 3.921036 | 2.486245 | 2.649107 | 3.794959 | 3.358918 | 3.514555 | 3.372058 | 2.706031 | 2.992558 |
| P61329;P7 | P70379;P( | FGF12_M | Fgf12;Fgf14 | Fibroblast growth factor 12 | | + | | 0.493754 | -0.419 | -1.30868 | 1.624171 | -0.41117 | -6.37144 | 0.155122 | -0.08041 | -0.44533 | 0.2918 | -0.34777 | 0.436507 | 0.481042 | 0.249978 | 0.113602 | 0.406672 | 0.375354 | 0.809374 | 0.930514 | 0.429396 | 0.095006 |
| O08583 | O08583;Q | THOC4_M | Alyref | THO complex subunit 4 | + | + | | 1.405757 | 1.121519 | 4.895311 | 1.56439 | 0.483199 | 5.931578 | 0.027511 | 0.024249 | 0.08697 | 0.609368 | 0.711537 | -0.27638 | -0.293 | 0.359711 | 0.435585 | -0.23773 | -0.6844 | -0.84893 | -0.68685 | 0.097172 | 0.649626 |
| Q9CRA9 | Q9CRA9 | FGOP2_M | Fgfr1op2 | FGFR1 oncogene partner 2 homolog | | | | 0.39385 | 1.616297 | 1.050261 | 0.018199 | -0.02446 | -0.05809 | 0.756088 | -1.276 | -2.06176 | -1.03063 | -1.10642 | 0.457664 | 1.286223 | -1.47021 | -0.30704 | -0.71689 | -3.65275 | 0.97237 | 0.820447 | 0.175764 | 0.598982 |
| P47746 | P47746 | CNR1_MO | Cnr1 | Cannabinoid receptor 1 | + | + | | 2.383162 | 1.119291 | 15.49636 | 2.130658 | 1.023379 | 11.55866 | 0.875784 | 0.563243 | 2.459297 | -1.02221 | -0.87817 | 0.234499 | 0.373002 | 0.276666 | -0.10166 | -2.07493 | -2.06403 | -0.77479 | -0.66446 | -0.34663 | -0.60485 |
| Q8CDM8 | Q8CDM8 | FHI2A_MO | Fhip2a | FHF complex subunit HOOK interacting protein 2A | | + | | 0.05541 | -0.2466 | -0.17063 | 1.930248 | -1.14864 | -9.14688 | 0.735759 | -0.2454 | -1.99815 | -1.39011 | -3.46088 | -2.12721 | -2.17485 | -1.89524 | -1.84384 | -1.17058 | -3.18721 | -1.12568 | -0.87909 | -1.74423 | -1.50405 |
| P97447 | P97447 | FHL1_MOI | Fhl1 | Four and a half LIM domains protein 1 | + | | | 1.056285 | -1.71377 | -3.14749 | 0.038922 | 0.056742 | 0.121677 | 0.181614 | -0.35121 | -0.51428 | -2.53317 | -3.62132 | -1.78557 | -2.65056 | -1.9112 | -1.80652 | -1.34222 | -1.38474 | -2.4492 | -2.10042 | -0.82675 | -2.18855 |
| O70433 | O70433 | FHL2_MOI | Fhl2 | Four and a half LIM domains protein 2 | | | | 0.4276 | 0.710949 | 1.136466 | 0.989211 | 0.087293 | 2.877785 | 0.323806 | -0.43111 | -0.87361 | -3.38566 | -2.45378 | -0.56755 | -0.54711 | -1.6885 | -0.7995 | -4.0481 | -3.21324 | -0.67319 | -0.61606 | -1.02723 | -0.59854 |
| Q76LL6 | Q76LL6 | FHOD3_M | Fhod3 | FH1/FH2 domain-containing protein 3 | | | | NaN | 0 | NaN | 0.508744 | 0.540046 | 1.348436 | 0.26965 | -0.32905 | -0.73779 | -3.23919 | -3.35405 | -2.34186 | -2.67645 | -2.85526 | -3.72895 | -3.26239 | -2.70391 | -3.41308 | -2.68532 | -3.05292 | -2.8732 |
| F7ANP4 | F6U3N7;L | F7ANP4_N | Gm16506 | Predicted gene 16506 (Fragment) | + | | | 1.497368 | 1.118501 | 5.471726 | 0.420959 | 3.679105 | 1.119429 | 0.565855 | 0.203967 | 1.50289 | 1.689813 | 1.357944 | 2.193453 | 2.688444 | 1.837776 | 1.569273 | 0.524755 | 0.286 | -4.51541 | 2.039103 | 1.479668 | 1.519447 |
| Q91WF7 | Q91WF7 | FIG4_MOI | Fig4 | Polyphosphoinositide phosphatase | | | | NaN | 0 | NaN | 0.006448 | 0.017018 | 0.020845 | 0.102157 | -0.23396 | -0.30316 | -3.42636 | -3.22694 | -2.69916 | -2.2346 | -4.06448 | -2.67231 | -2.57343 | -3.85392 | -3.26656 | -1.70123 | -3.46766 | -2.80121 |
| P26883 | P26883 | FKB1A_MC | Fkbp1a | Peptidyl-prolyl cis-trans isomerase FKBP1A | | | | 0.329698 | 0.5218 | 0.888394 | 0.472281 | 1.106333 | 1.252238 | 0.161845 | 0.28966 | 0.462936 | 0.524569 | 1.483901 | 2.453668 | 0.712351 | 1.363763 | 0.824041 | 0.143461 | 0.821409 | 0.326683 | 0.62667 | 1.368757 | 0.239726 |
| Q9Z2I2 | Q9Z2I2 | FKB1B_MC | Fkbp1b | Peptidyl-prolyl cis-trans isomerase FKBP1B | | | | 0.462113 | 0.923563 | 1.225703 | 0.221738 | 0.11641 | 0.616933 | 0.671557 | -1.2526 | -1.80382 | -2.01827 | -1.61591 | -0.26983 | 0.036285 | -2.04229 | -0.65605 | -3.4668 | -2.01451 | -0.12283 | -0.34353 | -0.05424 | -0.13891 |
| P45878 | P45878 | FKBP2_MC | Fkbp2 | Peptidyl-prolyl cis-trans isomerase FKBP2 | | | | 0.142913 | 0.706491 | 0.413131 | 0.44193 | 1.222093 | 1.17337 | 0.368215 | -0.74083 | -0.98533 | -3.11119 | -0.30578 | 0.366117 | -1.66182 | -1.37553 | -2.08322 | -1.4368 | -3.39316 | -2.10794 | -1.63195 | -0.32517 | -1.65193 |
| Q64378 | Q64378 | FKBP5_MC | Fkbp5 | Peptidyl-prolyl cis-trans isomerase FKBP5 | | | | NaN | 0 | NaN | 0.358439 | -0.22099 | -0.96067 | 0.197297 | -0.19592 | -0.55462 | -3.37908 | -3.91109 | -0.72653 | -0.43165 | -0.58526 | -0.68687 | -3.42011 | -3.41289 | -0.53468 | -0.18152 | -0.09057 | -0.78972 |
| O35465 | O35465 | FKBP8_MC | Fkbp8 | Peptidyl-prolyl cis-trans isomerase FKBP8 | | | | 0.818069 | 0.491016 | 2.262467 | 0.114699 | 0.152017 | 0.337463 | 0.32867 | 0.550494 | 0.885813 | -1.00446 | -1.43771 | -1.51028 | -1.45996 | -0.65202 | -1.83261 | -1.72537 | -1.69884 | -1.18737 | -2.08691 | -1.59849 | -1.98713 |
| Q8R123 | Q8R123 | FAD1_MOI | Flad1 | FAD synthase | + | | | 1.119769 | 0.404234 | 3.419835 | 0.686756 | -0.19164 | -1.84896 | 0.453522 | 0.157254 | 1.203374 | 1.003934 | 0.990149 | 0.611152 | 0.782343 | 0.658422 | 0.67506 | 0.474806 | 0.710809 | 0.946845 | 0.829939 | 0.379075 | 0.639899 |
| Q8QZS3 | Q8QZS3 | FLCN_MOI | Flcn | Folliculin | | | | 0.683787 | 0.960222 | 1.840105 | 0.963511 | 0.676236 | 2.779008 | 0.765516 | -0.3259 | -2.09162 | -1.90141 | -2.68194 | -0.59767 | -0.17943 | -1.90629 | -2.17794 | -3.59831 | -2.90549 | -1.18921 | -0.94036 | -1.79256 | -1.63986 |
| Q9JJ28 | Q9JJ28 | FLII_MOUS | Flii | Protein flightless-1 homolog | | | | NaN | 0 | NaN | 0.320498 | -0.28349 | -0.86531 | 0.276159 | -0.22034 | -0.75413 | -3.09408 | -3.63301 | -1.79999 | -2.39294 | -1.70821 | -2.28521 | -3.16121 | -3.30008 | -1.9524 | -1.67354 | -1.73021 | -1.82254 |
| O08917 | O08917 | FLOT1_MC | Flot1 | Flotillin-1 | + | | | 1.226769 | 0.451399 | 3.920693 | 0.670984 | 0.611952 | 1.802125 | 0.741056 | -0.56078 | -2.01462 | 1.155981 | 1.16471 | 2.810732 | 3.309378 | 1.601244 | 1.087388 | 0.593897 | 0.823996 | 2.217566 | 2.678641 | 1.798001 | 2.012185 |
| Q60634 | Q60634 | FLOT2_MC | Flot2 | Flotillin-2 | | | | 0.789556 | 0.449639 | 2.168829 | 0.499254 | 0.583453 | 1.323234 | 0.281611 | -0.3354 | -0.76781 | 0.548503 | 0.5368 | 2.132981 | 2.788709 | 1.253812 | 0.440748 | 0.300249 | -0.11422 | 1.582562 | 2.172221 | 1.022834 | 1.342525 |
| Q8BLU0 | Q8BLU0 | FLRT2_MC | Flrt2 | Leucine-rich repeat transmembrane protein FLRT2 | | | | NaN | 0 | NaN | 0.501211 | -0.21611 | -1.32842 | 0.445193 | 0.388264 | 1.181801 | -3.24117 | -3.35521 | -2.9341 | -3.20152 | -2.85211 | -3.0601 | -3.57129 | -3.37806 | -2.75903 | -2.94436 | -3.65601 | -3.03273 |
| Q9JL04 | Q9JL04 | FMN2_MO | Fmn2 | Formin-2 | | | | NaN | 0 | NaN | 0.258506 | 0.694578 | 0.70978 | 0.913828 | -1.06178 | -2.59475 | -3.30657 | -3.38854 | -2.34322 | -3.93081 | -2.30995 | -3.06295 | -2.79362 | -3.0687 | -3.00901 | -1.65418 | -1.78496 | -1.46438 |
| Q9JL26 | Q9JL26 | FMNL1_M | Fmnl1 | Formin-like protein 1 | | | + | 0.315725 | 0.66715 | 0.853338 | 0.558761 | -0.22899 | -1.48343 | 1.965906 | 0.275033 | 9.536905 | -2.19056 | -2.24515 | -1.20995 | -1.51728 | -0.79591 | -0.85186 | -3.66634 | -2.10367 | -1.14934 | -1.1199 | -1.10593 | -1.09191 |
| A2APV2 | A2APV2 | FMNL2_M | Fmnl2 | Formin-like protein 2 | | + | + | 0.338931 | 0.214961 | 0.911582 | 1.360284 | -0.46121 | -4.62977 | 1.6868 | -0.34906 | -6.86466 | -1.18952 | -0.82532 | -1.28434 | -1.48194 | -0.56612 | -0.57245 | -1.07255 | -1.37221 | -0.9347 | -0.90917 | -0.27098 | -0.16947 |
| P35922 | P35922 | FMR1_MO | Fmr1 | Fragile X messenger ribonucleoprotein 1 | | | | 0.195452 | 0.683675 | 0.549893 | 0.029443 | 0.03801 | 0.092898 | 0.302325 | 0.730769 | 0.819743 | -1.54676 | -3.9555 | -1.80636 | -1.23312 | -1.37045 | -2.23351 | -3.74344 | -3.12616 | -1.84974 | -1.26576 | -3.31281 | -1.7527 |
| Q80VD1 | Q80VD1 | FA98B_MC | Fam98b | Protein FAM98B | + | + | | 1.422491 | 1.117337 | 4.99636 | 1.183928 | -0.29061 | -3.7135 | 0.128364 | 0.061257 | 0.374351 | -0.34477 | -0.39456 | -0.67551 | -0.82921 | -0.23193 | -0.01348 | -1.70924 | -1.26476 | -0.44697 | -0.47653 | -0.06212 | -0.30581 |
| Q8BWB6 | Q8BWB6 | STEA2_MC | Steap2 | Metalloreductase STEAP2 | + | | | 1.538802 | 1.116536 | 5.752003 | 0.393858 | 0.426291 | 1.05028 | 0.139325 | -0.2927 | -0.40361 | -0.31064 | -0.02112 | -3.06431 | -3.53519 | -2.8846 | -2.81829 | -1.41173 | -1.15309 | -3.39542 | -4.05666 | -3.2832 | -1.83429 |
| Q80TY0 | Q80TY0 | FNBP1_M | Fnbp1 | Formin-binding protein 1 | | | | 0.75143 | 0.73469 | 2.047092 | 0.337788 | 0.155652 | 0.908709 | 0.180832 | -0.13637 | -0.51226 | -0.37137 | -0.91123 | -0.90483 | -1.10015 | -1.46716 | -1.10003 | -1.61252 | -1.13947 | -1.29886 | -1.01742 | -1.34003 | -0.95442 |
| Q8K012 | Q8K012 | FBP1L_MC | Fnbp1l | Formin-binding protein 1-like | | | | NaN | 0 | NaN | 0.352659 | 0.196149 | 0.946111 | 0.606117 | 0.390823 | 1.614995 | -3.47458 | -3.94185 | -0.23352 | -0.11721 | -1.02541 | -0.8641 | -2.75367 | -3.3766 | -0.57052 | -0.17252 | -1.56374 | -1.10742 |
| Q68FD7 | Q68FD7 | FNIP1_MO | Fnip1 | Folliculin-interacting protein 1 | + | + | | 1.14693 | 1.624186 | 3.541803 | 1.564073 | 0.705965 | 5.929318 | 0.193195 | -0.04778 | -0.5441 | -1.3296 | -2.16203 | -0.40908 | -0.46571 | -1.57176 | -1.58323 | -3.1775 | -3.56251 | -1.25901 | -1.02771 | -1.61735 | -1.44208 |
| Q61239 | Q61239 | FNTA_MOI | Fnta | Protein farnesyltransferase/geranylgeranyltransferase type-1 subunit alpha | | | + | 0.099729 | 0.174904 | 0.296471 | 0.007284 | -0.01056 | -0.02352 | 1.019099 | -0.66158 | -2.99578 | -0.62583 | -1.05078 | -0.56907 | -1.32715 | -1.88461 | -1.66407 | -1.56357 | -0.46285 | -1.17773 | -0.69738 | -1.3041 | -0.92142 |
| Q8K2I1 | Q8K2I1 | FNTB_MOI | Fntb | Protein farnesyltransferase subunit beta | | | | 0.586115 | 1.51555 | 1.558943 | 0.644993 | 1.106396 | 1.726117 | 0.545621 | -1.56055 | -1.44759 | -0.58266 | -1.22835 | -0.99617 | -0.99979 | -1.40746 | -3.49636 | -3.33805 | -1.50406 | -2.74535 | -1.46341 | -0.62436 | -1.15834 |
| Q9JHG6 | Q9JHG6 | RCAN1_M | Rcan1 | Calcipressin-1 | + | | | 1.319801 | 1.11646 | 4.404052 | 0.060278 | -0.02431 | -0.18483 | 0.316666 | 0.401999 | 0.855697 | 2.878039 | 2.952515 | 4.317916 | 4.340612 | 3.548759 | 3.2217 | 2.049575 | 1.54806 | 4.222543 | 4.484604 | 2.542819 | 3.423642 |
| Q60987 | Q60987 | FOXG1_M | Foxg1 | Forkhead box protein G1 | | | | NaN | 0 | NaN | 0.522322 | -0.32093 | -1.38471 | 0.635456 | -0.34235 | -1.69858 | -4.1097 | -2.9665 | -1.47493 | -1.13712 | -1.49281 | -1.59388 | -4.04555 | -2.64116 | -0.82639 | -1.1438 | -1.39611 | -1.00589 |
| P42128 | P42128 | FOXK1_M | Foxk1 | Forkhead box protein K1 | | | | 0.076947 | 0.245026 | 0.232712 | 0.960993 | -0.24377 | -2.76946 | 0.513813 | -0.87085 | -1.36195 | -1.64987 | -3.62452 | -0.21768 | -0.14744 | -0.83685 | -0.58262 | -2.5164 | -3.24804 | 0.14192 | -0.0195 | -0.46554 | 0.787756 |
| Q9D8B1 | Q9D8B1 | AIG1_MOL | Aig1 | Androgen-induced gene 1 protein | + | + | | 1.334701 | 1.112661 | 4.485994 | 1.071211 | 0.344773 | 3.209977 | 0.129777 | -0.21307 | -0.37814 | 1.634433 | 1.679791 | -0.16632 | -0.27653 | 0.522996 | -0.20427 | 0.297461 | 0.791442 | -0.65839 | -0.474 | -0.05799 | 0.802849 |
| P97376 | P97376 | FRG1_MOI | Frg1 | Protein FRG1 | | | | 0.565835 | 0.688987 | 1.502835 | 0.071572 | -0.04154 | -0.21739 | 0.741027 | -0.46755 | -2.01453 | -3.01172 | -2.10315 | -2.46391 | -2.19862 | -2.4083 | -2.7751 | -3.30813 | -3.18471 | -2.42725 | -2.15221 | -2.26638 | -1.98191 |
| Q3UFK8 | Q3UFK8 | FRMD8_M | Frmd8 | FERM domain-containing protein 8 | | | | 0.532385 | 1.45965 | 1.41177 | 0.747581 | 0.559232 | 2.035011 | 0.002009 | -0.00249 | -0.00653 | -2.05691 | -1.78527 | -2.18162 | -1.6327 | -1.67944 | -1.16854 | -2.35578 | -4.40569 | -2.45263 | -2.48015 | -1.70458 | -1.13842 |
| A0A140LI\ | A0A140LI\ | A0A140LI\ | Frmpd3 | FERM and PDZ domain containing 3 | | | | 0.245163 | 0.45458 | 0.67618 | 0.198584 | -0.09039 | -0.55792 | 0.183274 | 0.276216 | 0.518568 | -1.84506 | -2.59177 | -1.34822 | -1.1776 | -0.91976 | -1.35975 | -2.11392 | -3.23207 | -1.31026 | -1.03477 | -1.90107 | -0.93087 |
| A2AFR3 | A2AFR3 | FRPD4_M | Frmpd4 | FERM and PDZ domain-containing protein 4 | | | | NaN | 0 | NaN | 0.582125 | 0.702771 | 1.547847 | 0.810539 | -0.6579 | -2.23752 | -3.30742 | -3.60455 | -1.92154 | -1.73315 | -3.3027 | -2.7971 | -3.57347 | -3.25544 | -2.97427 | -2.08596 | -2.54216 | -2.24184 |
| E9Q8I9 | E9Q8I9 | FRY_MOU | Fry | Protein furry homolog | | | | 0.738584 | 0.535019 | 2.006924 | 0.462042 | 0.187656 | 1.225519 | 0.501484 | -0.42709 | -1.32914 | -0.00101 | -0.4654 | 0.591425 | 0.618087 | -0.35076 | -0.10221 | -0.63725 | -0.8992 | 0.264558 | 0.569643 | -0.09571 | 0.496926 |
| F8VQ05 | F8VQ05 | F8VQ05_N | Fryl | FRY like transcription coactivator | | | | 0.208786 | 0.314571 | 0.583986 | 0.429783 | 0.418324 | 1.142076 | 0.087905 | 0.162992 | 0.263601 | -2.61761 | -2.58 | -2.20477 | -1.73034 | -2.20562 | -2.62131 | -2.37504 | -3.45171 | -2.10678 | -2.66497 | -3.15881 | -1.99411 |
| Q61553 | Q61553 | FSCN1_M | Fscn1 | Fascin | + | | | 1.205353 | -0.80914 | -3.81596 | 0.789013 | -0.80048 | -2.16707 | 0.157979 | 0.219094 | 0.452822 | 0.725663 | 0.407405 | 1.138828 | 0.483545 | 0.868262 | 1.6213 | 1.515821 | 1.235536 | 1.782234 | 1.441092 | 1.329555 | 0.721821 |
| Q7TPM6 | Q7TPM6 | FSD1_MOI | Fsd1 | Fibronectin type III and SPRY domain-containing protein 1 | | + | | NaN | 0 | NaN | 1.298828 | -0.34992 | -4.29089 | 0.022354 | -0.04297 | -0.07104 | -4.38757 | -3.11145 | -1.00771 | -1.04664 | -0.53939 | -1.6953 | -2.66796 | -3.52342 | -0.59806 | -0.75645 | -0.89594 | -1.25281 |
| P09528 | P09528 | FRIH_MOL | Fth1 | Ferritin heavy chain | | + | | 0.86733 | -1.34439 | -2.42983 | 3.01278 | -1.31291 | -32.0681 | 0.043334 | 0.135801 | 0.13491 | -1.81331 | -0.73255 | -1.76019 | -1.71413 | -1.60784 | -0.17325 | 0.190288 | -0.04735 | -0.4581 | -0.3904 | -0.32012 | -1.73256 |
| Q8BGW1 | Q8BGW1 | FTO_MOU | Fto | Alpha-ketoglutarate-dependent dioxygenase FTO | | | | NaN | 0 | NaN | 0.208832 | 0.203804 | 0.584104 | 0.187331 | -0.09782 | -0.52903 | -3.0182 | -3.84191 | -3.01207 | -3.08315 | -3.30482 | -3.34461 | -3.61543 | -3.52427 | -2.90431 | -3.59851 | -3.04305 | -3.41073 |
| P56959 | P56959 | FUS_MOU | Fus | RNA-binding protein FUS | | | | 0.09349 | -0.0952 | -0.27918 | 0.200286 | -0.32079 | -0.56228 | 0.618812 | -0.32958 | -1.65096 | 2.419971 | 1.839017 | 0.808775 | 1.876467 | 2.171359 | 1.772775 | 2.403295 | 2.046093 | 1.864663 | 1.462159 | 2.31324 | 2.290052 |
| Q6PAM0 | Q9R078;Q | AAKB2_M | Prkab2 | 5'-AMP-activated protein kinase subunit beta-2 | + | + | | 1.828931 | 1.110947 | 8.120704 | 1.181626 | 0.918535 | 3.702622 | 0.352021 | 0.194939 | 0.944505 | 5.646815 | 5.508327 | 5.607904 | 6.046253 | 5.222826 | 5.366781 | 4.58461 | 4.348638 | 4.792334 | 5.024753 | 4.906428 | 5.2933 |
| Q9Z2V5 | Q9Z2V5 | HDAC6_M | Hdac6 | Histone deacetylase 6 | + | | | 2.298379 | 1.108445 | 14.04579 | 0.489294 | 0.593476 | 1.85655 | 0.270824 | 0.460906 | 0.740736 | 0.68817 | 0.781639 | 1.185524 | 1.738564 | 0.925812 | 0.048137 | -0.30995 | -0.43713 | 0.708185 | 1.028951 | -0.41505 | 0.467191 |
| Q9WVR4 | Q9WVR4 | FXR2_MOI | Fxr2 | RNA-binding protein FXR2 | | + | | 0.097188 | 0.103962 | 0.289446 | 1.285586 | 1.088071 | 4.220731 | 0.206502 | 0.628102 | 0.578159 | -2.95715 | -2.28065 | -1.67105 | -1.97157 | -1.71884 | -3.40871 | -2.84367 | -2.60206 | -2.69991 | -3.11885 | -2.50901 | -3.87475 |
| P59648 | P59648 | FXYD7_MC | Fxyd7 | FXYD domain-containing ion transport regulator 7 | | | | 0.220876 | -0.24019 | -0.61475 | 0.440424 | -0.6659 | -1.16948 | 0.015366 | 0.026781 | 0.049192 | -0.17986 | 0.567345 | 0.334316 | -0.62649 | 0.233903 | -0.07229 | 0.319568 | 0.548299 | 0.825476 | 0.214155 | 0.576471 | -0.46842 |
| P97379 | P97379 | G3BP2_M | G3bp2 | Ras GTPase-activating protein-binding protein 2 | | | | 0.199383 | -0.15313 | -0.55996 | 0.729662 | 0.305991 | 1.97927 | 0.437999 | 0.698247 | 1.163227 | 0.178484 | -0.29029 | 1.073393 | 1.381598 | 1.774204 | 0.575974 | 0.238118 | -0.04365 | 0.933873 | 0.909136 | 0.514008 | 0.439677 |
| O88343 | O88343 | S4A4_MOL | Slc4a4 | Electrogenic sodium bicarbonate cotransporter 1 | + | | | 1.610379 | 1.100261 | 6.267363 | 0.738633 | 0.543813 | 2.007078 | 0.176771 | -0.16265 | -0.50176 | 5.489161 | 5.687648 | 5.925853 | 6.435703 | 4.691878 | 4.691009 | 4.632954 | 4.343334 | 5.545174 | 5.728757 | 4.529933 | 5.178258 |
| Q9CQC6 | Q91VK1;Q | SMP2_MO | Bzw1 | eIF5-mimic protein 2 | + | | + | 1.639943 | 1.099889 | 6.49243 | 0.296012 | -0.32605 | -0.80392 | 1.787324 | -0.5115 | -7.73207 | -2.25187 | -1.97032 | -1.44002 | -2.19048 | -1.73459 | -1.86675 | -3.30523 | -3.11674 | -1.64314 | -1.33526 | -1.28603 | -1.29232 |
| Q80T41 | Q80T41 | GABR2_M | Gabbr2 | Gamma-aminobutyric acid type B receptor subunit 2 | | | | 0.51919 | 1.710529 | 1.376322 | 0.442456 | -0.18751 | -1.17473 | 0.377749 | 0.158541 | 1.009435 | -0.58289 | -0.37303 | 0.650103 | 0.374065 | 0.658249 | 0.379067 | -0.9501 | -3.42688 | 0.779774 | 0.619412 | 0.28813 | 0.432103 |
| P62812 | P62812;Q | GBRA1_M | Gabra1 | Gamma-aminobutyric acid receptor subunit alpha-1 | | | | 0.921019 | 0.239901 | 2.620891 | 0.765411 | -0.75656 | -2.09129 | 0.209006 | 0.139298 | 0.584547 | 0.494654 | 0.618737 | 1.352324 | 0.714214 | 1.465652 | 1.387988 | 0.384095 | 0.249494 | 1.960352 | 1.619301 | 1.522637 | 1.052407 |
| P26049 | P26049 | GBRA3_M | Gabra3 | Gamma-aminobutyric acid receptor subunit alpha-3 | | | | 0.236538 | -0.46642 | -0.65441 | 0.88093 | -0.39441 | -2.47735 | 0.020438 | 0.135638 | 0.06508 | -1.19069 | -2.61575 | -0.789 | -0.83789 | -0.86498 | -3.40216 | -1.42005 | -1.45355 | -0.57636 | -0.26172 | -0.61559 | -3.92282 |
| P50571 | P50571 | GBRB1_M | Gabrb1 | Gamma-aminobutyric acid receptor subunit beta-1 | | | | 0.527751 | 1.244289 | 1.399294 | 0.765195 | -0.72737 | -2.9096 | 0.920887 | 0.440881 | 2.620412 | -3.26281 | -1.57723 | -1.0449 | -1.69664 | 0.044013 | -0.02068 | -3.94792 | -3.3807 | -0.52149 | -0.76531 | -0.59433 | -0.26411 |
| P63137 | P63137 | GBRB2_M | Gabrb2 | Gamma-aminobutyric acid receptor subunit beta-2 | | | | 0.535804 | 1.018378 | 1.421 | 0.904521 | -0.60757 | -2.56118 | 0.193396 | 0.167365 | 0.544616 | -1.45639 | -0.38372 | -0.16331 | -0.37545 | -0.11125 | 0.187856 | -1.46309 | -2.41377 | 0.126006 | 0.550381 | 0.1394 | -0.39752 |
| P63017 | P63017 | HSP7C_M | Hspa8 | Heat shock cognate 71 kDa protein | + | | | 1.809292 | 1.098176 | 7.934996 | 0.727263 | 0.403696 | 1.97187 | 0.387588 | -0.34328 | -1.03436 | 7.530314 | 7.431747 | 8.096497 | 8.499687 | 7.081345 | 7.629186 | 6.512178 | 6.25353 | 7.858721 | 7.93007 | 7.51117 | 7.885922 |
| P22933 | P22933 | GBRD_MO | Gabrd | Gamma-aminobutyric acid receptor subunit delta | | + | + | 0.146419 | 0.202967 | 0.422407 | 1.20655 | -0.39438 | -3.82176 | 1.75133 | -0.80926 | -7.4102 | -2.99438 | -2.64776 | -1.94788 | -1.74212 | -3.88322 | -3.74374 | -3.47219 | -2.57588 | -1.4426 | -1.45863 | -2.92019 | -3.08826 |
| P22723 | P22723 | GBRG2_M | Gabrg2 | Gamma-aminobutyric acid receptor subunit gamma-2 | | | | NaN | 0 | NaN | 0.572917 | -0.80946 | -1.52235 | 0.422586 | 0.346693 | 1.1236 | -2.9348 | -3.65694 | -1.76176 | -2.69388 | -1.26644 | -1.74224 | -3.23679 | -3.6227 | -1.67431 | -1.1624 | -1.65454 | -2.04753 |
| P48318 | P48318 | DCE1_MO | Gad1 | Glutamate decarboxylase 1 | | | | 0.502238 | 0.548018 | 1.331145 | 0.885124 | 0.284893 | 2.492116 | 0.151306 | 0.106761 | 0.435296 | 1.341244 | 1.128149 | 1.756559 | 1.816662 | 1.90179 | 1.555715 | 1.084341 | 0.289015 | 1.39142 | 1.612014 | 1.448179 | 1.795804 |
| P48320 | P48320 | DCE2_MO | Gad2 | Glutamate decarboxylase 2 | | + | + | 0.110251 | -0.09903 | -0.32535 | 2.641735 | 0.729647 | 20.89889 | 2.590855 | 0.951787 | 19.70565 | 0.689697 | 0.117836 | 1.11253 | 1.051333 | 1.086264 | 1.172383 | 0.607198 | 0.398404 | 0.369097 | 0.335472 | 0.155655 | 0.199417 |

| | | | | | | | | | | | | | | | | | | | | | | | | | | | | |
|---|---|---|---|---|---|---|---|---|---|---|---|---|---|---|---|---|---|---|---|---|---|---|---|---|---|---|---|---|
| P54818 | P54818 | GALC_MO | Galc | Galactocerebrosidase | | | | 0.86024 | 0.580106 | 2.405286 | 0.507913 | 0.270952 | 1.346225 | 0.623012 | -0.34786 | -1.66292 | 0.324168 | 0.327012 | -0.30866 | 0.081672 | -0.72873 | -1.01393 | -0.01334 | -0.49569 | -0.43313 | -0.33556 | -0.67652 | -0.37042 |
| Q9R0N0 | Q9R0N0 | GALK1_MC | Galk1 | Galactokinase | | | | 0.192326 | 0.222776 | 0.54187 | 0.184389 | -0.39609 | -0.52144 | 0.291272 | -0.4172 | -0.79203 | -1.21228 | -1.26818 | -1.43469 | -2.34403 | -2.07181 | -1.11095 | -1.87318 | -1.05283 | -0.88476 | -2.10177 | -0.95821 | -1.39015 |
| P70419 | P70419 | GALT3_MC | Galnt3 | Polypeptide N-acetylgalactosaminyltransferase 3 | | | + | 0.607493 | 0.299092 | 1.61888 | 0.052779 | -0.04993 | -0.1629 | 1.958314 | 0.344124 | 9.452544 | 6.458077 | 6.771554 | 4.203406 | 4.708551 | 4.988123 | 5.026575 | 6.217916 | 6.413532 | 4.679577 | 4.332245 | 4.63231 | 4.694139 |
| P06837 | P06837 | NEUM_MC | Gap43 | Neuromodulin | | + | | 0.826102 | 0.593044 | 2.289265 | 1.194052 | 0.921337 | 3.761631 | 0.61066 | 0.665862 | 1.627831 | -0.03258 | -0.20351 | 0.794413 | 1.114652 | 0.793736 | 0.009415 | -0.95564 | -0.46654 | -0.15215 | 0.21854 | -0.38061 | -0.14796 |
| P16858 | A0A1D5RL | G3P_MOU | Gapdh | Glyceraldehyde-3-phosphate dehydrogenase | + | | | 1.159144 | -0.39181 | -3.59776 | 0.524294 | -0.87518 | -1.39 | 0.538284 | -0.8298 | -1.4277 | 3.832507 | 4.039017 | 4.960439 | 3.754319 | 4.48319 | 5.561355 | 4.362189 | 4.292954 | 5.05161 | 5.413501 | 6.069327 | 5.634818 |
| Q9JI75 | Q9JI75 | NQO2_MC | Nqo2 | Ribosyldihydronicotinamide dehydrogenase [quinone] | + | | + | 1.55645 | 1.09698 | 5.875311 | 0.597082 | 0.400236 | 1.589587 | 1.196108 | 0.497502 | 3.771467 | 8.561388 | 8.932409 | 7.407707 | 7.701206 | 7.695436 | 7.674798 | 7.628788 | 7.671049 | 6.749622 | 7.358819 | 7.056108 | 7.319123 |
| Q9CY66 | Q9CY66 | GAR1_MO | Gar1 | H/ACA ribonucleoprotein complex subunit 1 | | | | 0.468967 | -0.20269 | -1.24357 | 0.076388 | -0.13783 | -0.23112 | 0.369193 | 0.284762 | 0.987804 | -0.35485 | -0.30713 | -1.45737 | -2.29979 | -0.4475 | -0.44522 | -0.28953 | 0.032934 | -0.59705 | -0.88444 | -1.0194 | -0.44285 |
| Q3UFT3 | Q3UFT3 | GARE1_MC | Garem1 | GRB2-associated and regulator of MAPK protein | | | + | NaN | 0 | NaN | 0 | NaN | 0.20577 | -0.17275 | -0.57629 | 1.112997 | -0.82793 | -3.38995 | -3.02525 | -3.77241 | -1.78337 | -1.27696 | -1.16198 | -1.36599 | -1.72788 | -3.87933 | -1.19697 | -1.51787 | -1.65796 | -0.21415 |
| Q9CZD3 | Q9CZD3 | GARS_MO | Gars1 | Glycine--tRNA ligase | | | | 0.87695 | -0.67081 | -2.46338 | 0.062413 | -0.08025 | -0.19103 | 0.325332 | 0.358897 | 0.877435 | -0.65365 | -0.98644 | -0.62836 | -2.07767 | -1.72083 | -1.21342 | -1.9456 | -1.14401 | -0.2908 | 0.262641 | 0.3139 | -0.77947 | -0.43341 | -0.17391 | -0.9787 |
| Q64737 | Q64737 | PUR2_MO | Gart | Trifunctional purine biosynthetic protein adenosine-3 | | + | | 0.012157 | 0.002101 | 0.039053 | 1.184267 | -0.24261 | -3.7151 | 0.213304 | 0.046325 | 0.595495 | -0.16801 | -0.06882 | -0.2746 | -0.30587 | 0.053256 | -0.102 | -0.09965 | -0.14138 | 0.015782 | -0.11103 | -0.06565 | -0.07575 |
| Q61592 | Q61592 | GAS6_MO | Gas6 | Growth arrest-specific protein 6 | | | | 0.861293 | 0.663982 | 2.408924 | 0.92103 | 0.676605 | 2.620932 | 0.228386 | -0.14487 | -0.63379 | -0.23762 | -0.78535 | -0.98644 | -0.62836 | -2.07767 | -1.72083 | -1.21342 | -1.29803 | -1.61148 | -1.89726 |
| Q60780 | Q60780 | GAS7_MO | Gas7 | Growth arrest-specific protein 7 | | | | 0.262245 | -1.15477 | -0.71918 | 0.105975 | 0.097158 | 0.313654 | 0.452319 | -0.30189 | -1.20025 | -3.83383 | -1.61252 | 0.046443 | 0.233089 | -0.43991 | -0.31021 | -2.72799 | -0.40882 | -0.25276 | 0.533279 | 0.169857 | -0.31619 |
| Q8VHR5 | Q8VHR5 | P66B_MO | Gatad2b | Transcriptional repressor p66-beta | | | | 0.9352 | 2.065594 | 2.672959 | 0.135948 | 0.062848 | 0.394623 | 0.391862 | -0.33431 | -1.04521 | -0.50306 | -1.7527 | -0.81968 | -0.53337 | -1.33461 | -0.70148 | -1.64821 | -2.73874 | -0.66958 | -0.80917 | -0.63798 | -0.72948 |
| Q8BFQ8 | Q8BFQ8 | GALD1_MC | Gatd1 | Glutamine amidotransferase-like class 1 domain-containing protein 1 | | | | 0.177536 | 0.202034 | 0.503741 | 0.146282 | -0.20485 | -0.42204 | 0.754273 | -1.27193 | -2.05604 | -3.45019 | -2.76127 | -0.9485 | -1.57876 | -1.55231 | -2.7826 | -3.11458 | -3.50996 | -1.42795 | -0.68959 | -0.96109 | -0.82996 |
| Q8C547 | Q8C547 | HTR5B_MC | Heatr5b | HEAT repeat-containing protein 5B | + | | | 1.313351 | 1.086957 | 4.368979 | 0.08289 | -0.05154 | -0.24952 | 0.645993 | 0.322727 | 1.729016 | -2.34058 | -1.96171 | -1.18592 | -1.09727 | -1.44867 | -1.17565 | -3.07682 | -3.39938 | -0.88829 | -1.29182 | -1.50759 | -1.76218 |
| Q9D6Y9 | Q9D6Y9 | GLGB_MO | Gbe1 | 1,4-alpha-glucan-branching enzyme | | | + | 0.020937 | -0.05557 | -0.06663 | 0.578279 | -0.92768 | -1.53718 | 1.456438 | -1.24861 | -5.20707 | -0.78315 | -1.76187 | -2.99614 | -0.42439 | -4.21489 | -3.96566 | -0.54164 | -1.89225 | -2.26655 | -2.89862 | -3.04653 | -2.63679 |
| Q6DFZ1 | Q6DFZ1 | Q6DFZ1_M | Gbf1 | Golgi-specific brefeldin A-resistance factor 1 | | | + | NaN | 0 | NaN | 0.882867 | 0.851904 | 2.484162 | 1.0612 | 0.735192 | 3.167965 | -3.66295 | -3.2215 | -2.12017 | -2.00828 | -1.85278 | -1.40917 | -3.37928 | -3.14731 | -3.25447 | -2.67779 | -2.43442 | -2.29791 |
| Q60759 | Q60759 | GCDH_MC | Gcdh | Glutaryl-CoA dehydrogenase, mitochondrial | | | | 0.46553 | 0.175282 | 1.234608 | 0.390965 | -0.47024 | -1.04293 | 0.377529 | 0.285894 | 1.008878 | 2.242728 | 2.511808 | 1.252875 | 2.035379 | 2.270421 | 2.669759 | 2.156649 | 2.247324 | 1.890276 | 2.338459 | 2.385282 | 1.983111 |
| P97494 | P97494 | GSH1_MO | Gclc | Glutamate--cysteine ligase catalytic subunit | | | | 0.429099 | -0.77247 | -1.14032 | 0.589428 | -0.20616 | -1.56818 | 0.518548 | -0.25289 | -1.3746 | -3.12384 | -2.39047 | -2.32635 | -2.30908 | -2.52644 | -2.16727 | -1.41509 | -2.55428 | -1.98038 | -2.24274 | -2.05403 | -2.1339 |
| O09172 | O09172 | GSH0_MO | Gclm | Glutamate--cysteine ligase regulatory subunit | | | | 0.487997 | -0.25806 | -1.29349 | 0.029409 | 0.028373 | 0.092796 | 0.338992 | 0.263942 | 0.911734 | 1.149892 | 0.77028 | 0.583198 | 0.691455 | 0.7979 | 0.874629 | 1.279596 | 1.256996 | 0.909886 | 0.30802 | 0.285382 | 0.859263 |
| Q50H33 | Q50H33 | KCTD8_MC | Kctd8 | BTB/POZ domain-containing protein KCTD8 | + | | | 2.509052 | 1.08688 | 17.92732 | 0.01654 | 0.021833 | 0.052884 | 0.54644 | -0.65808 | -1.44982 | -1.46768 | -1.586 | -1.30053 | -1.46681 | -2.34113 | -2.46722 | -2.60048 | -2.62696 | -1.80989 | -1.00111 | -2.1956 | -1.29658 |
| Q9R111 | Q9R111 | GUAD_MO | Gda | Guanine deaminase | | + | | 0.140036 | -0.08197 | -0.4055 | 2.824759 | -0.67732 | -25.8161 | 0.236514 | -0.17899 | -0.65435 | 0.065208 | -0.30203 | 2.403928 | 2.351509 | 2.637417 | 3.054126 | -1.12098 | 0.04809 | 3.056225 | 3.053855 | 3.20201 | 2.847521 |
| O88741 | O88741 | GDAP1_M | Gdap1 | Ganglioside-induced differentiation-associated protein 1 | + | | | 1.225481 | -0.79433 | -3.91433 | 0.589162 | 0.460015 | 1.567434 | 0.750109 | 0.940819 | 2.042941 | -0.17875 | -0.05457 | 0.221448 | -0.36551 | 0.979859 | 0.219376 | 0.870687 | 0.484474 | -0.53346 | -0.53063 | -0.0814 | -0.601 |
| Q8VE33 | Q8VE33 | GD1L1_MC | Gdap1l1 | Ganglioside-induced differentiation-associated protein 1-like 1 | + | + | | 1.162221 | 0.576432 | 3.611968 | 1.759232 | 0.777948 | 7.479758 | 0.600448 | 0.861478 | 1.599034 | 0.807833 | 0.49052 | 0.884135 | 0.88589 | 1.773445 | 0.84943 | 0.089975 | 0.055513 | 0.211068 | 0.003061 | 0.172833 | 0.727086 |
| P50396 | P50396 | GDIA_MO | Gdi1 | Rab GDP dissociation inhibitor alpha | + | | | 1.251481 | -1.12488 | -4.0445 | 0.142754 | -0.38137 | -0.41271 | 0.08783 | -0.20231 | -0.26339 | 0.803747 | 1.349857 | 2.738204 | 0.985198 | 1.456309 | 2.846783 | 2.254548 | 2.148814 | 1.950424 | 2.5372 | 2.680327 | 2.027376 |
| Q8VCN9 | Q8VCN9 | TBCC_MO | Tbcc | Tubulin-specific chaperone C | + | | | 1.554629 | 1.07997 | 5.862475 | 0.524737 | 0.893906 | 1.391192 | 0.12595 | -0.20193 | -0.36787 | -1.75269 | -2.062 | -2.47712 | -1.21512 | -0.83707 | -1.92218 | -3.0874 | -2.88722 | -2.86128 | -2.61877 | -1.67077 | -1.09432 |
| Q9CRY7 | Q9CRY7 | GDPD1_M | Gdpd1 | Lysophospholipase D GDPD1 | | | | 0.800143 | -0.20412 | -2.20333 | 0.380098 | -0.31262 | -1.01538 | 0.13675 | -0.15223 | -0.39676 | 1.11306 | 1.241997 | 0.476014 | -0.13282 | 0.677141 | 1.428681 | 1.31512 | 1.448184 | 0.530312 | 0.438125 | 1.125751 | 1.282721 |
| Q8BX17 | Q8BX17 | GEMI5_M | Gemin5 | Gem-associated protein 5 | | | | 0.381038 | 1.289321 | 1.017759 | 0.548551 | 0.509756 | 1.455558 | 0.336708 | -0.96283 | -0.906 | -1.61996 | -0.96659 | 0.901385 | 0.86528 | 0.066299 | 0.799857 | -1.35843 | -3.80646 | 0.723324 | 0.023828 | 2.39333 | 0.398478 |
| O54984 | O54984 | GET3_MO | Get3 | ATPase GET3 | | | | 0.027621 | 0.111306 | 0.087308 | 0.064234 | -0.13756 | -0.1963 | 0.427184 | -0.33152 | -1.1354 | -2.62702 | -1.62352 | -0.3317 | -1.73319 | -2.53939 | -2.47808 | -1.04635 | -0.81863 | -0.08907 | -0.90185 | -0.58154 | -1.03204 |
| Q7TNG5 | Q7TNG5 | EMAL2_M | Eml2 | Echinoderm microtubule-associated protein-like 2 | + | | | 1.358921 | 1.079161 | 4.622014 | 0.87627 | 0.603291 | 2.460999 | 0.319588 | -0.61148 | -0.86303 | 6.271769 | 5.996116 | 5.060834 | 5.546997 | 3.109972 | 4.28919 | 5.243244 | 4.866319 | 4.668917 | 4.732333 | 3.918154 | 4.703968 |
| P03995 | P03995 | GFAP_MO | Gfap | Glial fibrillary acidic protein | | | + | 0.472499 | -1.18428 | -1.25281 | 0.333517 | -0.29999 | -0.89798 | 1.446623 | 1.115846 | 5.14535 | 4.318211 | 3.58926 | 3.281169 | 2.615357 | 5.042511 | 5.469507 | 6.010221 | 4.265805 | 3.276203 | 4.102097 | 4.178229 |
| Q3UHD2 | Q3UHD2 | GFOD1_M | Gfod1 | Glucose-fructose oxidoreductase domain-containing protein 1 | | | | 0.943216 | 1.564356 | 2.702698 | 0.070811 | 0.036091 | 0.215217 | 0.661915 | -1.22669 | -1.77544 | -1.02068 | -0.92117 | -0.03441 | 0.309132 | -2.00988 | -1.09483 | -1.35981 | -1.11195 | 0.056619 | 0.141916 | -0.84339 | 0.19206 |
| Q9CYH5 | Q9CYH5 | GFOD2_M | Gfod2 | Glucose-fructose oxidoreductase domain-containing protein 2 | | | | 0.354769 | 1.329077 | 0.951408 | 0.185001 | 0.220621 | 0.523024 | 0.426577 | -1.10179 | -1.13384 | -0.68965 | -3.47907 | -0.79302 | 0.028515 | -0.53346 | -1.32312 | -3.36774 | -3.4562 | -0.69879 | -0.50695 | -1.53772 | -0.61586 |
| P47856 | P47856 | GFPT1_M | Gfpt1 | Glutamine--fructose-6-phosphate aminotransferase [isomerizing] 1 | + | | | 0.166268 | 0.283789 | 0.47448 | 0.454175 | 1.417747 | 1.205069 | 1.387822 | 1.295488 | 4.789023 | -2.34704 | -3.41392 | -1.00404 | -0.38173 | -3.31111 | -1.18032 | -2.89377 | -3.43476 | -3.24523 | -0.97604 | -2.80369 | -2.27871 |
| Q8BMI3 | Q8BMI3 | GGA3_MO | Gga3 | ADP-ribosylation factor-binding protein GGA3 | | | | 0.578388 | -1.23566 | -1.53748 | 0.339152 | 0.162014 | 0.912138 | 0.021996 | 0.048797 | 0.069928 | -0.01609 | -1.43019 | -0.8755 | -1.23003 | -2.31856 | -1.05526 | -0.85651 | -1.11845 | -1.226 | -1.20357 | -0.02331 | -1.43911 |
| Q99JP7 | Q99JP7 | GGT7_MO | Ggt7 | Glutathione hydrolase 7 | | | | 0.459619 | 1.706108 | 1.16372 | 0.943651 | 0.617008 | 2.704319 | 0.21211 | 0.130688 | 0.592456 | -0.01123 | -0.18109 | 0.081686 | 0.12312 | -0.33865 | -0.26589 | 0.282707 | 0.247654 | -0.34478 | 0.043969 | | |
| Q91VC9 | Q91VC9 | GHITM_M | Ghitm | Growth hormone-inducible transmembrane protein | | | | 0.309503 | -0.18389 | -0.83774 | 0.645878 | -0.37546 | -1.72868 | 0.505381 | 0.347762 | 1.339492 | -0.22544 | -0.32307 | -1.62669 | -1.50096 | -0.81819 | -0.65186 | 0.12658 | -0.30438 | -0.98047 | -1.39626 | -0.83685 | -1.32873 |
| Q9D7M1 | Q9D7M1 | GID8_MO | Gid8 | Glucose-induced degradation protein 8 homolog | | | | 0.836858 | 0.678681 | 2.325444 | 0.022596 | 0.050193 | 0.071792 | 0.646436 | -1.56416 | -1.7303 | -2.76591 | -2.48356 | -2.60319 | -1.78095 | -4.38593 | -2.59758 | -3.55655 | -3.04029 | -2.80776 | -1.67676 | -1.79482 | -2.06037 |
| P99027 | A0A5F8M | RLA2_MO | Rplp2 | Large ribosomal subunit protein P2 | + | | | 1.657105 | 1.078971 | 6.626486 | 0.501187 | 0.638496 | 1.328357 | 0.793651 | -0.41827 | -2.16214 | 2.282259 | 2.523602 | 2.634719 | 3.050684 | 1.474803 | 1.841121 | 1.43328 | 1.214639 | 1.835825 | 2.772585 | 2.152079 | 2.171063 |
| P23242 | P23242 | CXA1_MO | Gja1 | Gap junction alpha-1 protein | + | | + | 1.227375 | 0.646996 | 3.923693 | 0.407516 | 1.314656 | 1.085057 | 1.051263 | 0.762717 | 3.126679 | -0.1868 | 0.137262 | -0.67507 | -1.1999 | 0.077725 | -0.04561 | -0.64116 | -0.70237 | -3.43498 | -1.0693 | -0.51065 | -0.98268 |
| Q8BTI8 | Q8BTI8 | SRRM2_M | Srrm2 | Serine/arginine repetitive matrix protein 2 | + | + | | 1.319703 | 1.071602 | 4.403514 | 1.287049 | 0.618045 | 4.228435 | 0.535766 | 0.233218 | 1.420895 | -0.20615 | 0.210783 | 0.403894 | 0.383526 | 0.281669 | -0.03953 | -0.94374 | -1.19483 | -0.37014 | -0.07853 | -0.07827 | -0.14603 |
| Q8K3I9 | Q8K3I9 | GLCI1_MC | Glcci1 | Glucocorticoid-induced transcript 1 protein | | | | NaN | 0 | NaN | 0.192137 | -0.11128 | -0.54138 | 0.746796 | -1.01036 | -2.03255 | -4.2047 | -2.86263 | -1.22456 | -1.24138 | -2.84835 | -1.93634 | -2.80975 | -3.33164 | -1.32706 | -0.91632 | -1.1841 | -1.57987 |
| Q91W43 | Q91W43 | GCSP_MO | Gldc | Glycine dehydrogenase (decarboxylating), mitochondrial | | | | NaN | 0 | NaN | 0.445139 | 0.333306 | 1.181663 | NaN | 0 | NaN | -3.01599 | -3.66286 | -2.89552 | -3.30673 | -3.19218 | -3.07642 | -3.67083 | -3.18162 | -3.24133 | -3.62753 | -3.46824 | -3.30978 |
| Q9CPU0 | Q9CPU0 | LGUL_MO | Glo1 | Lactoylglutathione lyase | | | | 0.528565 | -0.66526 | -1.40148 | 0.103462 | 0.238356 | 0.306755 | 0.321354 | -0.26499 | -0.86746 | -0.52047 | 0.389088 | 0.788604 | -0.75983 | 0.031307 | 0.175371 | 0.463552 | 0.735591 | -0.15799 | -0.28995 | 0.66519 | 0.071464 |
| Q8CPV4 | Q9CPV4 | GLOD4_M | Glod4 | Glyoxalase domain-containing protein 4 | | | | 0.512087 | -0.49338 | -1.35734 | 0.090237 | -0.17108 | -0.27012 | 0.100345 | -0.26126 | -0.29817 | -1.19974 | -1.01531 | -0.62591 | -1.84988 | -2.20764 | -0.54814 | -0.26255 | -0.96574 | -0.9037 | -1.22992 | -0.83506 | -1.39818 |
| Q64018 | Q64018 | GLRA1_M | Glra1 | Glycine receptor subunit alpha-1 | | | | 0.085629 | -0.56301 | -0.25722 | NaN | 0 | NaN | | | | -0.86334 | 0.313595 | -0.42809 | -3.46044 | -0.92334 | -1.26363 | -0.29446 | -0.35451 | -3.5686 | -0.24216 | -3.63017 | -3.65559 |
| Q9QUH0 | Q9QUH0 | GLRX1_M | Glrx | Glutaredoxin-1 | + | | | 1.178645 | -0.37672 | -3.68858 | 0.028075 | 0.02635 | 0.088703 | 0.472792 | -0.3116 | -1.25357 | -0.20987 | -0.31112 | -0.4888 | -1.05073 | -0.61409 | -0.11729 | 0.027525 | 0.204928 | -0.89255 | -0.69968 | -0.045 | -0.06319 |
| Q9CQM9 | Q9CQM9 | GLRX3_M | Glrx3 | Glutaredoxin-3 | | | | 0.263395 | -0.11868 | -0.72207 | 0.878699 | -0.80566 | -2.46951 | 0.045713 | 0.038074 | 0.142001 | -0.58279 | -0.63185 | -0.57416 | -1.18766 | -0.69407 | -0.18328 | -0.65116 | -0.13555 | -0.0354 | -0.39611 | -0.55839 | |
| Q7TQN3 | Q7TQN3 | WFKN2_M | Wfikkn2 | WAP, Kazal, immunoglobulin, Kunitz and NTR domain-containing protein 2 | | | | 1.699771 | 1.064877 | 6.971181 | 0.394294 | -1.13675 | -1.05139 | 0.411123 | 0.592503 | 1.094264 | 3.715013 | 4.01005 | 2.363791 | -3.59611 | 3.849753 | 2.78051 | 2.837304 | 2.758005 | 2.664811 | 2.37638 | 2.636828 | 2.808429 |
| Q571F8 | Q571F8 | GLSL_MO | Gls2 | Glutaminase liver isoform, mitochondrial | | | | 0.791699 | -1.05772 | -2.17579 | 0.177565 | -0.44376 | -0.50382 | 0.278825 | -0.38405 | 0.760837 | -0.53839 | -0.80463 | 0.742309 | -1.01182 | 0.88813 | 0.309497 | 0.853759 | -0.38018 | 0.227993 | 0.628386 | -0.19889 | |
| P61963 | P61963 | DCAF7_M | Dcaf7 | DDB1- and CUL4-associated factor 7 | | | | 2.251886 | 1.060518 | 13.30802 | 0.158053 | -0.08051 | -0.45301 | 0.003301 | 0.002397 | 0.010707 | 2.397037 | 2.522187 | 1.908937 | 2.264244 | 1.763295 | 1.893731 | 1.448439 | 1.349748 | 2.172211 | 2.161995 | 1.61193 | 2.040301 |
| P26443 | P26443 | DHE3_MO | Glud1 | Glutamate dehydrogenase 1, mitochondrial | | | | 1.088244 | -1.03866 | -3.28242 | 0.555653 | -0.86403 | -1.47493 | 0.090251 | 0.112333 | 0.27016 | 2.62002 | 3.028422 | 3.063327 | 1.905729 | 2.9896 | 3.759925 | 4.104603 | 3.621156 | 3.258169 | 3.438955 | 3.419082 | 3.105779 |
| P15105 | P15105 | GLNA_MO | Glul | Glutamine synthetase | + | | | 1.00934 | 0.535278 | 2.952912 | 0.465372 | -0.20474 | -1.2342 | 0.219359 | -0.14776 | -0.61089 | 5.266484 | 5.014951 | 4.456934 | 4.819331 | 4.621799 | 5.009971 | 4.779349 | 4.471689 | 4.752741 | 5.107989 | 4.819303 | |
| Q922P9 | Q922P9 | GLYR1_M | Glyr1 | Cytokine-like nuclear factor N-PAC | | | | 1.018 | 1.215131 | 2.991381 | 0.165844 | -0.71128 | -0.47337 | 0.224638 | -0.96008 | -0.62429 | -1.31704 | -1.1318 | -0.73774 | -3.73906 | -0.55261 | -3.61074 | -2.03494 | -2.82288 | -1.45142 | -1.60283 | -1.28601 | -0.95717 |
| A2AQX6 | A2AQX6;Q | A2AQX6_M | Gm14151 | Predicted gene 14147 | | | | 0.890945 | 2.412581 | 2.512713 | 0.265542 | 1.689651 | 0.729882 | 0.167197 | 1.199885 | 0.476898 | 3.616161 | 5.013027 | 0.057147 | 0.61727 | -2.67333 | 0.767806 | 2.56086 | 1.243166 | 0.945199 | -3.65009 | -0.3169 | -3.9684 |
| Q3TRG0 | Q3TRG0 | Q3TRG0_M | Gm1604b | Gene model 1604B, (NCBI) | | | | 0.997705 | 1.898524 | 2.910972 | 0.685029 | 1.142893 | 1.843808 | 0.127329 | 0.063699 | 0.291297 | 3.905711 | 3.840222 | 4.186999 | 4.729771 | 3.098825 | 2.748843 | 1.32307 | 2.625816 | 2.758204 | 3.872779 | 2.698292 | 2.981977 |
| P35438 | P35438 | NMDZ1_M | Grin1 | Glutamate receptor ionotropic, NMDA 1 | + | | | 1.878461 | 0.950407 | 8.607714 | 0.439215 | -0.19384 | -1.16636 | 0.166974 | 0.28687 | 0.476319 | -0.04644 | 0.04378 | 1.981001 | 2.232018 | 3.421263 | 2.401515 | -0.94623 | 1.409289 | 2.191412 | 2.303972 | 2.845065 | |
| Q60648 | Q60648 | SAP3_MO | Gm2a | Ganglioside GM2 activator | | | | 0.498168 | 0.65047 | 1.320358 | 0.388514 | 0.14149 | 1.036711 | 0.401748 | 0.235252 | 1.070352 | 0.789932 | 1.725296 | 0.761088 | 0.796114 | 0.622472 | 0.866707 | 0.761981 | 0.452306 | 0.501759 | 0.772462 | 0.69208 | 0.326595 |
| S4R1W1 | S4R1W1 | S4R1W1_M | Gm3839 | Glyceraldehyde-3-phosphate dehydrogenase | | | | 0.889129 | -0.97516 | -2.50628 | 0.020829 | -0.06814 | -0.0668 | 0.022982 | -0.14263 | -0.07299 | -3.4461 | -3.11725 | -2.57795 | -3.92297 | -3.44992 | -1.1364 | -2.65915 | -1.95387 | -3.9595 | -2.40514 | -0.5755 | -3.72556 |
| A0A2R8V | Q6F28;A0 | A0A2R8V | Gm5414;Gm5478 | Predicted pseudogene 5478 | | | | 1.105117 | 2.299993 | 3.355428 | 0.425122 | -0.72197 | -1.1301 | 0.066944 | 0.555841 | 0.204116 | 1.448147 | -0.852076 | 0.158907 | -1.05197 | -3.55739 | 1.756293 | -3.26715 | 0.479344 | 0.071534 | -0.85979 | -2.05255 | |
| Q8K0C9 | Q8K0C9 | GMDS_MC | Gmds | GDP-mannose 4,6 dehydratase | | | | 1.137904 | -2.25988 | -3.50089 | 0.17326 | 0.053016 | 0.49266 | 0.08176 | -0.12027 | -0.24633 | -2.91305 | -1.62752 | -0.1234 | -0.10625 | -0.39637 | 0.013876 | 0.049095 | -0.0699 | -0.27511 | -0.06057 | 0.37209 | -0.51404 |
| O08576 | O08576 | RUN3A_M | Rundc3a | RUN domain-containing protein 3A | | | | 1.824861 | 1.045945 | 8.018879 | 0.945097 | 0.846063 | 2.709712 | 0.220173 | -0.36893 | -0.61296 | 2.906415 | 2.680262 | 2.54173 | 2.721356 | 1.320922 | 2.416317 | 1.684442 | 1.810345 | 1.486443 | 2.084518 | 1.987977 | 2.487115 |
| Q922H4 | Q922H4 | GMPPA_M | Gmppa | Mannose-1-phosphate guanyltransferase alpha | | | | 0.508197 | 0.529495 | 1.346982 | 0.160933 | -0.54343 | -0.46055 | 0.068184 | 0.079709 | 0.207681 | -1.13054 | -1.63323 | -1.27979 | -3.61737 | -0.7697 | -1.49483 | -1.60914 | -2.21362 | -2.06699 | -1.74332 | -1.33437 | -1.09318 |
| Q9DCZ1 | Q9D27;Q | Q9L27_M | Gmpr | GMP reductase 1 | | | | 0.799073 | -1.97207 | -2.19883 | 0.756631 | -0.75268 | -1.79677 | 0.28646 | -0.4968 | -0.77997 | -1.27306 | -3.7133 | -0.5513 | -2.22002 | -2.15425 | -2.03195 | -1.38062 | -0.5258 | -2.3357 | -3.00096 | -0.9623 | -2.2903 |
| Q99L27 | Q99L27 | GMPR2_M | Gmpr2 | GMP reductase 2 | | | | NaN | 0 | NaN | 0.098922 | 0.261672 | 0.294241 | 0.247422 | -0.64712 | -0.68187 | -3.23174 | -2.36235 | -1.3905 | -1.50832 | -2.77764 | -3.40956 | -3.34152 | -2.659 | -2.23897 | -2.89995 | -1.55158 | -3.34137 |
| Q3THK7 | Q3THK7 | GUAA_MO | Gmps | GMP synthase [glutamine-hydrolyzing] | + | | | 0.723815 | 0.589672 | 1.961257 | 1.012295 | -0.27948 | -2.96862 | 0.565389 | -0.15782 | -1.50161 | 0.709254 | 0.108504 | 0.031053 | -0.07369 | 0.269659 | 0.110561 | -0.19389 | -0.1677 | 0.179925 | 0.336388 | 0.416617 | 0.279242 |
| P20029 | P20029 | BIP_MOU | Hspa5 | Endoplasmic reticulum chaperone BiP | + | | | 1.682873 | 1.045413 | 6.167165 | 0.098038 | 0.825813 | 2.573829 | 0.434447 | 0.410023 | 1.154075 | 3.341845 | 3.644747 | 4.206209 | 4.380815 | 3.947373 | 3.582173 | 2.534719 | 2.383948 | 3.158955 | 3.776443 | 3.047973 | 3.659527 |
| Q9CR57 | Q9CR57 | RL14_MO | Rpl14 | Large ribosomal subunit protein eL14 | + | + | | 1.895192 | 1.031523 | 8.778468 | 1.498931 | 0.4927 | 5.482071 | 0.107295 | 0.14627 | 0.31727 | 2.70659 | 2.913837 | 3.23052 | 3.253673 | 2.61608 | 1.979981 | 1.834097 | 1.723284 | 2.66027 | 2.838522 | 1.818012 | 2.48551 |
| B2RSH2 | P08752;Q | GNAI1_M | Gnai1 | Guanine nucleotide-binding protein G(i) subunit alpha-1 | | | | 0.848792 | -0.4094 | -2.36599 | 0.363771 | -0.30293 | -0.97412 | 0.014324 | 0.01482 | 0.045098 | 1.399245 | 1.58136 | 2.282995 | 1.684081 | 2.169953 | 2.455876 | 2.446981 | 1.752599 | 2.370348 | 2.202592 | 2.587529 | 2.00866 |
| P08752 | P08752 | GNAI2_M | Gnai2 | Guanine nucleotide-binding protein G(i) subunit alpha-2 | | | | 0.278556 | -0.22077 | -0.76014 | 0.307301 | 0.084308 | 0.224047 | 0.096115 | 0.093601 | 0.284472 | 0.204626 | 0.766166 | 0.673751 | -0.03511 | 0.990312 | 0.842013 | 0.632557 | 0.779126 | 0.108614 | 0.361409 | 1.140775 | 0.504347 |
| Q9DC51 | Q9DC51 | GNAI3_M | Gnai3 | Guanine nucleotide-binding protein G(i) subunit alpha-3 | | | | 0.275548 | -0.27545 | -0.75259 | 0.052026 | 0.035527 | 0.160687 | 0.548944 | 0.652647 | 1.456629 | -1.97375 | -2.29119 | -1.77199 | -2.13379 | -1.4576 | -0.99088 | -1.52723 | -2.18681 | -1.86131 | -2.11552 | -1.4944 | -2.25937 |
| Q8CGK7 | Q8CGK7 | GNAL_MO | Gnal | Guanine nucleotide-binding protein G(olf) subunit alpha | | | | NaN | 0 | NaN | 0.483736 | 0.399981 | 1.282277 | 0.142295 | 0.198056 | 0.411493 | -1.33975 | -2.71968 | 0.130654 | -0.30008 | -0.9496 | -1.03313 | -3.75392 | -3.59743 | -0.71034 | -0.25905 | -0.70992 | -1.66891 |
| P18872 | P18872 | GNAO_MC | Gnao1 | Guanine nucleotide-binding protein G(o) subunit alpha | | | | 0.646005 | 1.181842 | 1.72905 | 0.156315 | -0.17835 | -0.44848 | 0.212021 | 0.178474 | 0.592223 | 3.169751 | 3.373078 | 4.558464 | 3.845778 | 4.457599 | 4.736336 | 3.063824 | 3.116036 | 4.570306 | 4.685761 | 4.151525 | |
| P21279 | P21279 | GNAQ_MC | Gnaq | Guanine nucleotide-binding protein G(q) subunit alpha | | | | 1.10206 | -0.80066 | -3.52471 | 0.503881 | -0.38171 | -0.98575 | 0.027641 | 0.031091 | 0.087371 | 1.28525 | 1.759709 | 2.3148 | 1.592361 | 2.389023 | 2.674794 | 2.289747 | 2.356538 | 2.195773 | 2.474808 | 2.826728 | 2.174906 |
| Q6R0H7 | Q6R0H7 | GNAS1_M | Gnas | Guanine nucleotide-binding protein G(s) subunit alpha isoforms XLas | | | | 0.90076 | -0.52303 | -2.54769 | 0.439271 | -0.3662 | -1.16651 | 0.352436 | 0.098819 | 0.945549 | -0.18082 | 0.185228 | 0.006658 | -0.62107 | -0.0698 | -0.27532 | 0.618242 | 0.432234 | 0.065318 | 0.052688 | -0.25235 | -0.2904 |
| O70443 | O70443 | GNAZ_MC | Gnaz | Guanine nucleotide-binding protein G(z) subunit alpha | | | | 0.618097 | -0.52157 | -1.64892 | 0.10423 | -0.07348 | -0.30886 | 0.411128 | 0.340922 | 1.094277 | -0.44215 | 0.176736 | 1.456715 | 1.05216 | 1.537557 | 1.421237 | 0.454407 | 0.323318 | 1.202688 | 1.453148 | 1.444548 | 0.832401 |
| P62874 | P62874 | GBB1_MC | Gnb1 | Guanine nucleotide-binding protein G(I)/G(S)/G(T) subunit beta-1 | | | | 0.10989 | 0.162579 | 0.243365 | 0.258888 | -0.30844 | -0.71074 | 0.146596 | -0.14882 | -0.42287 | 3.113759 | 2.740215 | 4.408376 | 3.301176 | 3.818994 | 4.269404 | 3.229351 | 2.299946 | 3.813241 | 4.463412 | 3.922232 | |
| P62880 | P62874;P | GBB2_MC | Gnb2 | Guanine nucleotide-binding protein G(I)/G(S)/G(T) subunit beta-2 | | | | 0.806562 | -0.3806 | -2.2244 | 0.587662 | -0.57684 | -1.56325 | 0.017943 | 0.018521 | 0.057286 | 4.193969 | 4.158789 | 5.315282 | 4.577462 | 5.130358 | 5.480983 | 4.727222 | 4.386823 | 5.514967 | 5.531465 | 5.558801 | 5.015498 |
| P29387 | P29387 | GBB4_MC | Gnb4 | Guanine nucleotide-binding protein subunit beta-4 | | | | 0.980927 | -2.19592 | -2.84568 | 0.143597 | -0.52438 | -0.41494 | 0.171977 | -0.62117 | -0.48933 | -3.09821 | -1.8643 | -3.72783 | -3.55952 | -3.24784 | -3.42222 | 0.178175 | -0.74884 | -1.85836 | -4.38024 | -1.44743 | -3.98029 |
| P62881 | P62881 | GNB5_MO | Gnb5 | Guanine nucleotide-binding protein subunit beta-5 | | | | 0.120169 | 0.173708 | 0.352287 | 0.160663 | 0.05611 | 0.459647 | 0.543906 | -0.27381 | -1.44294 | -1.02803 | -0.32942 | 1.32907 | 1.423731 | 0.209775 | 0.585151 | -0.50441 | -1.20046 | 1.432755 | 1.207826 | 0.643312 | 0.699236 |
| Q9D6X9 | Q9D6X9 | GBG12_M | Gng12 | Guanine nucleotide-binding protein G(I)/G(S)/G(O) subunit gamma-12 | + | | | 1.207576 | -1.20346 | -3.82673 | 0.071419 | -0.11527 | -0.21896 | 0.096615 | 0.248207 | 0.287859 | 1.652323 | 2.103823 | 1.896742 | 0.906978 | 1.987371 | 3.023538 | 2.873247 | 3.323819 | 1.323823 | 1.710434 | 2.4465 | 1.567995 |
| Q9JMF3 | Q9JMF3 | GBG13_M | Gng13 | Guanine nucleotide-binding protein G(I)/G(S)/G(O) subunit gamma-13 | + | | | 0.467739 | 1.189913 | 1.240369 | 0.320817 | -1.50837 | -0.86611 | 0.580311 | -0.26124 | -1.54281 | 0.094853 | -0.63239 | -0.06358 | -3.54854 | -0.73163 | -0.41291 | -1.05795 | -2.34642 | -0.26852 | -0.32927 | -0.36827 | -0.25378 |
| P63213 | P63213 | GBG2_MO | Gng2 | Guanine nucleotide-binding protein G(I)/G(S)/G(O) subunit gamma-2 | | | | 0.975073 | 0.34313 | 2.823146 | 0.093747 | -0.16859 | -0.2799 | 0.030458 | 0.038423 | 0.096004 | 0.428308 | 0.186921 | 2.150455 | 0.946236 | 1.654445 | 2.036836 | -0.04985 | -0.02118 | 1.701008 | 1.73286 | 2.158614 | 1.455621 |
| P63216 | P63216 | GBG3_MC | Gng3 | Guanine nucleotide-binding protein G(I)/G(S)/G(O) subunit gamma-3 | | | | 0.683739 | -0.10642 | -1.68996 | 0.220087 | -0.2884 | -0.64351 | 0.09151 | 0.11183 | 0.273673 | 3.228529 | 2.498759 | 2.567559 | 2.801508 | 2.220342 | 3.413611 | 2.885785 | 2.962408 | 2.944423 | 2.783784 | | |
| Q80SZ7 | Q80SZ7 | GBG5_MO | Gng5 | Guanine nucleotide-binding protein G(I)/G(S)/G(O) subunit gamma-5 | | | | 0.383996 | -1.49172 | -1.02525 | 0.454399 | -1.46113 | -1.20565 | 0.212747 | 0.065264 | 0.594077 | -3.023 | -0.13365 | -1.27513 | -3.38146 | -0.66977 | -0.47556 | -0.25942 | 0.086195 | -0.26754 | -1.46579 | -0.58656 | -0.68929 |
| D3YVF0 | D3YVF0 | AKAP5_M | Akap5 | A-kinase anchor protein 5 | + | + | | 1.353574 | 1.02967 | 4.591683 | 1.002226 | 0.762233 | 2.928747 | 0.242951 | -0.28028 | -0.6706 | -0.48456 | -0.91707 | 2.355943 | 2.870199 | 1.126436 | 0.715756 | -1.67114 | -1.78982 | 1.810589 | 1.891087 | 0.837341 | 1.565413 |
| P36916 | P36916 | GNL1_MO | Gnl1 | Guanine nucleotide-binding protein-like 1 | + | + | | 1.166612 | 0.168116 | 3.632326 | 1.155867 | 0.632105 | 3.58268 | 0.395007 | -0.29157 | -1.0532 | -0.0479 | 0.041481 | 1.134603 | 1.371477 | 0.164006 | -0.36553 | -0.1503 | -0.18336 | 0.490162 | 0.751708 | 0.109939 | 0.271682 |
| Q6PGG6 | Q6PGG6 | GNL3L_M | Gnl3l | Guanine nucleotide-binding protein-like 3-like protein | + | | | 0.954356 | 0.117282 | 2.534512 | 0.245521 | 0.641726 | 0.385261 | 0.184583 | 0.354639 | 0.549867 | -1.48256 | -2.61154 | -2.72474 | -1.27213 | -2.62899 | -1.78338 | -2.61466 | -2.77448 | -1.16263 | -1.59082 | -1.37498 | -2.1567 |
| Q9D428 | Q9D428 | GOG7B_M | GOLGA7B | Golgin subfamily A member 7B | | | | 0.181847 | -0.73342 | -0.41382 | 0.127628 | -0.04303 | -0.37243 | 0.111766 | -0.15935 | -0.32948 | -0.37172 | -0.30721 | 0.208584 | 0.439527 | 0.366204 | -0.60075 | -0.53442 | -1.44247 | 0.363189 | 0.37098 | 0.054832 | 0.029328 |
| Q6P2L7 | Q6P2L7 | GOLM2_M | Golm2 | Protein GOLM2 | | | | NaN | 0 | NaN | 0.685475 | 0.241291 | 1.845137 | 0.152115 | -0.51568 | -0.47342 | -3.67467 | -3.04258 | -3.27394 | -3.52236 | -1.81606 | -3.50229 | -3.20377 | -3.37879 | -3.68035 | -3.59853 | -2.95998 | -1.31962 |
| Q9D0B0 | Q9D0B0 | GON4L_M | Gon4l | GON-4-like protein | | | | 0.32125 | 0.646428 | 0.867196 | 0.764876 | 0.038937 | 0.085958 | 0.247883 | 1.12917 | 0.683036 | -0.30009 | -1.86825 | 1.968787 | 1.304338 | 0.074228 | -0.02296 | -3.43823 | -4.28882 | -0.48593 | -2.85343 | 3.407979 | |
| Q8BH60 | Q8BH60 | GOPC_MO | Gopc | Golgi-associated PDZ and coiled-coil motif-containing protein | | | | 0.066996 | -0.15462 | -0.20426 | 0.925334 | 0.495339 | 2.636661 | 0.239193 | 0.526436 | 0.661116 | -2.44904 | -0.65162 | -0.44532 | -0.62788 | -1.16328 | -1.22635 | -1.20373 | -1.78873 | -1.19614 | -0.86774 | -2.50276 | -0.91274 |
| Q99JX3 | Q99JX3 | GORS2_M | Gorasp2 | Golgi reassembly-stacking protein 2 | | | | 0.505714 | -1.09275 | -1.34038 | 0.309202 | 0.922739 | 0.836868 | 0.055526 | -0.26334 | -0.17097 | -1.46994 | -4.37341 | -1.58918 | -3.19715 | -1.70712 | -4.13006 | -0.50759 | -2.15027 | -0.41655 | -2.56442 | -1.70404 | -3.60647 |
| P05201 | P05201 | AATC_MO | Got1 | Aspartate aminotransferase, cytoplasmic | | | | 1.266802 | 1.77594 | -4.12335 | 0.095787 | -0.32839 | -0.28556 | 0.021797 | 0.007773 | 0.037136 | 0.396398 | 1.106457 | 2.359149 | 0.398697 | 0.885863 | 2.345272 | 1.771205 | 2.283552 | 1.614195 | 1.465729 | 2.408945 | 0.768644 |
| P84091 | P84091 | AP2M1_M | Ap2m1 | AP-2 complex subunit mu | + | | | 1.419972 | 1.024211 | 4.981033 | 0.74401 | 0.480507 | 2.023839 | 0.166394 | -0.14416 | -0.47481 | 5.290396 | 5.118229 | 6.525657 | 6.833462 | 5.106958 | 5.469581 | 4.366838 | 3.993367 | 6.018116 | 6.379808 | 5.188901 | 5.67595 |
| Q9EQP2 | Q9EQP2 | EHD4_MO | Ehd4 | EH domain-containing protein 4 | + | | | 1.63559 | 1.020143 | 6.458825 | 1.105557 | 0.607526 | 2.961709 | 0.031702 | 0.053664 | 0.099802 | -2.22686 | -1.91833 | -1.70846 | -1.65381 | -1.30117 | -2.3765 | -3.12662 | -3.05885 | -2.49196 | -2.08536 | -1.89962 | -1.85358 |
| P51655 | P51655 | GPC4_MO | Gpc4 | Glypican-4 | | | | 0.469584 | 1.118288 | 1.245187 | 0.19776 | -0.09318 | -0.55581 | 0.387747 | -0.38736 | -1.03477 | -0.74653 | -0.74153 | 0.365182 | 0.700444 | 1.295516 | 0.70442 | -0.96423 | -2.7604 | 0.628391 | 0.623596 | 1.157578 | 1.617075 |
| O54950 | O54950 | AAKG1_M | Prkag1 | 5'-AMP-activated protein kinase subunit gamma-1 | + | | | 1.816729 | 1.004449 | 8.603438 | 0.885931 | 0.515428 | 2.494966 | 0.370148 | 0.222644 | 0.990115 | 2.582947 | 2.749001 | 2.896003 | 2.816198 | 2.675101 | 2.28619 | 2.611023 | 2.734529 | 3.196883 | 3.833226 | 3.282777 | |
| Q8VEK3 | Q8VEK3 | HNRPU_M | Hnrnpu | Heterogeneous nuclear ribonucleoprotein U | + | | | 1.429615 | 1.004247 | 5.039938 | 0.059144 | -0.0436 | -0.18153 | 0.726061 | 0.526569 | 1.968167 | 3.762825 | 4.156873 | 2.984626 | 3.454618 | 4.080016 | 3.611166 | 2.925848 | 2.985356 | 3.213482 | 3.312972 | 3.190088 | 3.447957 |
| Q8C0L9 | Q8C0L9 | GPCP1_M | Gpcpd1 | Glycerophosphocholine phosphodiesterase GPCPD1 | | | | 0.11966 | -0.38599 | -0.35091 | 0.042401 | 0.054105 | 0.132118 | 0.284374 | -0.88785 | -0.77524 | -2.49718 | -0.30113 | -1.18128 | -1.18773 | -1.5561 | -3.73307 | -1.87597 | -3.64038 | -1.64811 | -0.8291 | -1.11284 | -1.40064 |
| P13707 | P13707 | GPDA_MO | Gpd1 | Glycerol-3-phosphate dehydrogenase [NAD(+)], cytoplasmic | | | | 0.609713 | -0.62691 | -1.66922 | 0.328895 | -0.57551 | -0.88638 | 0.097936 | 0.010885 | 0.072612 | 0.456994 | 1.390183 | 0.513589 | 0.75164 | 0.793152 | 1.602831 | 2.03809 | 1.999301 | 0.266334 | 1.314368 | 1.057785 | |
| Q3ULJ0 | Q3ULJ0 | GPDL1_M | Gpd1l | Glycerol-3-phosphate dehydrogenase 1-like protein | | | | 0.690706 | -0.4659 | -1.86078 | 0.818613 | -0.57538 | -2.26428 | 0.026433 | 0.030296 | 0.085634 | 0.292909 | -0.1821 | 0.510417 | 0.017101 | 0.570632 | 1.182083 | 0.600554 | 0.442052 | 0.900237 | 0.778041 | 1.238897 | 0.72226 |
| Q64521 | Q64521 | GPDM_MC | Gpd2 | Glycerol-3-phosphate dehydrogenase, mitochondrial | | | | 0.378242 | -0.09714 | -1.01068 | 0.093997 | -0.04342 | -0.28059 | 0.525321 | -0.17748 | -1.39276 | 1.56356 | 1.751543 | 1.860944 | 1.683493 | 2.237667 | 2.266611 | 1.774777 | 1.734605 | 1.688845 | 1.94244 | 2.556224 | 2.303013 |
| Q8BUV3 | Q8BUV3 | GEPH_MO | Gphn | Gephyrin | | | | 0.269248 | 0.329916 | 0.736778 | 0.162135 | 0.094403 | 0.463694 | 0.411019 | -0.25844 | -1.094 | 0.92309 | 0.863589 | 2.554127 | 2.873264 | 2.314685 | 2.12552 | 1.010216 | 0.116631 | 2.492856 | 2.745173 | 2.262069 | 2.695019 |
| P06745 | P06745 | G6PI_MO | Gpi | Glucose-6-phosphate isomerase | | | | 1.122881 | -1.47413 | -3.43364 | 0.064748 | 0.211852 | 0.197784 | 0.023249 | 0.059548 | 0.07099 | 0.60662 | 0.036165 | 1.997001 | 0.14129 | 0.41262 | 1.592489 | 1.473547 | 0.904259 | 0.650886 | 0.781117 | 1.539327 | 0.346685 |
| P35802 | P35802 | GPM6A_M | Gpm6a | Neuronal membrane glycoprotein M6-a | | | | 0.605988 | -0.53435 | -1.61463 | 0.726341 | -1.373 | -1.96903 | 0.053034 | -0.13627 | -0.16366 | 1.218279 | 1.8617 | 3.45145 | 2.958668 | 2.844477 | 3.11095 | 2.748179 | 2.003265 | 4.163643 | 4.092478 | 4.154407 | 3.073352 |
| P35803 | P35803 | GPM6B_M | Gpm6b | Neuronal membrane glycoprotein M6-b | | | | 0.552261 | -0.53327 | -1.46567 | 0.359742 | -0.70614 | -0.96386 | 0.076568 | -0.24442 | -0.23163 | -0.26231 | 0.263556 | 0.861968 | -0.60293 | -0.0516 | 1.619664 | 0.866248 | 0.785389 | 0.847887 | 0.823427 | 1.673248 | 0.384086 |
| Q8C419 | Q8C419 | GP158_M | Gpr158 | Probable G-protein coupled receptor 158 | | | | 0.372906 | 0.316303 | 0.872249 | 0.61759 | -0.25869 | -1.64748 | 0.338722 | -0.04885 | -0.55542 | 2.293709 | 0.813341 | 1.734275 | 2.037633 | 1.497869 | 1.969761 | 0.338275 | 2.184855 | 2.104724 | 1.426585 | 1.528314 | |
| Q3UN16 | Q3UN16 | GP162_M | Gpr162 | Probable G-protein coupled receptor 162 | + | | | 0.262615 | 1.24111 | 0.720112 | 1.421142 | -0.25663 | -4.98815 | 0.448957 | 0.353935 | 1.191543 | -0.11051 | -0.52208 | 0.419499 | 0.37365 | 0.178512 | -0.18792 | 0.15376 | -0.26857 | 0.60715 | 0.699268 | 0.59244 | -0.12484 |
| Q9QY42 | Q9QY42 | GPR37_M | Gpr37 | Prosaposin receptor GPR37 | | | | 0.29564 | 0.175936 | 0.802985 | 0.809607 | -0.24549 | -2.23444 | 0.298503 | -0.24285 | -0.81016 | 1.028958 | 0.61594 | -1.14531 | -1.00514 | -0.79106 | -0.48439 | 0.573304 | 0.719227 | -0.1947 | -0.13731 | -0.65244 | |
| Q99JG2 | Q99JG2 | G37L1_M | Gpr37l1 | G-protein coupled receptor 37-like 1 | | | | 0.810766 | 0.653299 | 2.238264 | 0.190113 | 0.121832 | 0.536182 | 0.602408 | 0.323309 | 1.604547 | 4.792053 | 5.373056 | 3.961708 | 3.852359 | 4.452829 | 4.061695 | 4.400948 | 4.457663 | 4.005746 | 3.564657 | 3.885436 | 3.98247 |
| Q5U431 | Q5U431 | GPR39_M | Gpr39 | G-protein coupled receptor 39 | | | | 0.869065 | 0.771903 | 2.43586 | 0.161235 | -0.2124 | -0.46134 | 0.01526 | -0.01894 | -0.04886 | 2.345349 | 1.919545 | 0.848754 | 1.469484 | 2.214037 | 0.939249 | 1.595263 | 1.125825 | 1.711575 | 1.031463 | 0.458645 | 0.732514 |
| Q8BS95 | Q8BS95 | GPHR_MO | Gpr89 | Golgi pH regulator | | | | 0.66525 | 0.612165 | 1.785233 | 0.367865 | 0.557605 | 1.082146 | 0.219057 | 0.121305 | 0.589518 | -0.18753 | -0.68481 | -1.24038 | -0.6187 | -1.13614 | -0.96588 | -0.81219 | -1.28447 | -1.47563 | -1.09901 | -0.98498 | -1.35965 |
| Q5U4C1 | Q5U4C1 | GASP1_M | Gprasp1 | G-protein coupled receptor-associated sorting protein 1 | + | | | 0.310695 | 0.4477 | 0.84726 | 1.57653 | 0.351175 | 6.018562 | 0.742917 | -0.61666 | -2.02043 | -0.21497 | -0.44881 | 1.141391 | 0.272375 | -0.1157 | -0.30636 | -1.29911 | -0.26007 | 0.745644 | 0.720772 | 0.115779 | 0.695873 |
| Q3UNH4 | Q3UNH4 | GRIN1_M | Gprin1 | G protein-regulated inducer of neurite outgrowth 1 | + | | | 1.277054 | 0.807008 | 4.17709 | 0.665577 | 0.02824 | 0.200178 | 0.209438 | -0.06032 | -0.58565 | -0.49279 | -0.06269 | 0.807039 | 0.861538 | 1.068892 | 1.059655 | -1.5406 | -1.4289 | 0.759497 | 1.018659 | 1.227443 | 1.152208 |
| Q8BWS5 | Q8BWS5 | GRIN3_M | Gprin3 | G protein-regulated inducer of neurite outgrowth 3 | | | | NaN | 0 | NaN | 0.918887 | 0.475928 | 2.613125 | 0.17624 | -0.18758 | -0.50039 | -3.422 | -4.20771 | -2.55207 | -2.58481 | -3.24436 | -3.99304 | -3.95213 | -2.95966 | -2.86296 | -3.22576 | -3.41107 | -3.45116 |
| Q99LD4 | Q99LD4 | CSN1_MO | Gps1 | COP9 signalosome complex subunit 1 | | | | 0.954523 | -0.25914 | -2.74503 | 0.409702 | -0.09511 | -1.09084 | 0.073221 | 0.064133 | 0.222106 | -0.50478 | -0.54384 | -0.53764 | -0.54865 | 0.137286 | -0.40916 | -0.17282 | -0.35754 | -0.36099 | -0.53507 | -0.10667 | -0.29348 |
| Q6R5I6 | Q6R5I6 | GPSM1_M | Gpsm1 | G-protein-signaling modulator 1 | | | | 0.461489 | 1.399484 | 1.224078 | 0.225804 | -0.09601 | -0.62724 | 0.195921 | -0.26396 | -0.5511 | -0.05562 | -0.46838 | -0.7074 | -0.41487 | -1.16589 | -1.55503 | -2.78404 | -0.53419 | -0.41999 | -0.50126 | -1.53418 | -0.65883 |
| Q8BGT5 | Q8BGT5 | ALAT2_M | Gpt2 | Alanine aminotransferase 2 | | | | 0.359189 | -0.78645 | -0.96256 | 0.728781 | 0.960126 | 1.976552 | 0.150986 | -0.26533 | -0.43445 | -0.45961 | -0.17883 | -1.83595 | -2.75653 | -2.81 | -2.58045 | -1.50801 | -1.9993 | -1.3008 | -3.41314 | -1.80085 | -3.40095 |
| O70325 | O70325 | GPX4_MO | Gpx4 | Phospholipid hydroperoxide glutathione peroxidase | | | | 0.921711 | 0.9528 | 2.623417 | 0.985424 | 0.575657 | 2.863075 | 0.034711 | 0.027287 | 0.108955 | 3.592221 | 3.706319 | 3.232012 | 3.606896 | 2.840479 | 2.859087 | 3.055152 | 2.337788 | 2.771231 | 2.916462 | 2.572224 | 3.072767 |
| Q99LJ6 | Q99LJ6 | GPX7_MO | Gpx7 | Glutathione peroxidase 7 | | | | NaN | 0 | NaN | 0.45486 | 0.180685 | 1.206848 | 0.007683 | 0.018386 | 0.024003 | -2.26878 | -1.25917 | -3.15344 | -2.98093 | -3.5702 | -3.09869 | -3.47856 | -3.32012 | -3.1255 | -3.37024 | -4.05561 | -2.65004 |
| Q80T10 | Q80T10 | ASTRB_M | Gramd1b | Protein Aster-B | + | | | 0.566428 | 0.710182 | 1.504466 | 1.616474 | -0.76164 | -4.99138 | 0.543994 | -0.3027 | -1.44318 | -0.76967 | -0.26241 | 0.183705 | 0.481625 | -0.27909 | -0.06379 | -0.8281 | -1.62434 | 1.061225 | 1.217153 | 0.311276 | -0.24875 |
| Q60631 | Q60631 | GRB2_MO | Grb2 | Growth factor receptor-bound protein 2 | | | | 0.667397 | 0.426936 | 1.791551 | 0.563446 | 0.176335 | 1.49627 | 0.136942 | 0.060648 | 0.390761 | 0.807815 | 1.070757 | 1.367092 | 1.15837 | 1.630897 | 1.79963 | 0.330106 | 0.766954 | 1.038348 | 1.139843 | 1.781847 | 1.527383 |
| O35127 | O35127 | C10_MOU | Grcc10 | Protein C10 | | | | 0.845681 | 1.004216 | 2.355378 | 0.067168 | 0.184201 | 0.204728 | 0.163378 | -0.27963 | -0.46669 | -2.21971 | -1.07238 | -0.67491 | -0.10536 | -0.68787 | -1.32686 | -2.64694 | -2.65588 | -1.42766 | 0.278987 | -1.23424 | -0.22124 |
| Q60875 | Q60875 | ARHG2_M | Arhgef2 | Rho guanine nucleotide exchange factor 2 | | | | 1.325689 | 1.004157 | 4.436275 | 0.312081 | -0.13748 | -0.8442 | 0.341364 | -0.25334 | -0.97177 | -0.56023 | -1.01294 | 0.792178 | 0.871696 | 0.581324 | -0.79094 | -1.79054 | 0.987323 | 1.152632 | 0.74168 | 0.152095 | |
| P23818 | P23818 | GRIA1_M | Gria1 | Glutamate receptor 1 | + | | | 0.318689 | 0.453957 | 0.86077 | 1.964916 | -0.75784 | -9.75135 | 0.479221 | -0.19737 | -1.27042 | -1.59605 | -1.08798 | -0.97893 | -1.13355 | 1.559833 | 1.676479 | -2.25815 | -1.33381 | -0.30633 | -0.29048 | 1.959522 | 1.671531 |
| P21995 | P21995 | EMB_MOU | Emb | Embigin | + | | | 1.58388 | 1.00324 | 6.071795 | 0.458993 | 0.292511 | 1.217584 | 0.039885 | 0.074519 | 0.124576 | 2.674014 | 2.435833 | 1.485152 | 1.965193 | 1.799169 | 0.61318 | 3.438616 | 4.662005 | 1.422424 | 1.442899 | 1.053046 | 1.210264 |
| Q9Z2W9 | Q9Z2W9;Q | GRIA3_M | Gria3 | Glutamate receptor 3 | | | | 0.691145 | -1.41208 | -1.86209 | 0.769694 | -1.4359 | -2.04093 | 0.080986 | -0.06908 | -0.26103 | -0.40032 | -2.11686 | -1.84493 | -3.09639 | -0.74553 | -0.63111 | -1.75055 | -0.94247 | -0.76307 | -1.30643 | -0.36023 | -0.87725 |
| Q9Z2W8 | Q9Z2W8 | GRIA4_M | Gria4 | Glutamate receptor 4 | | | | 0.213496 | -0.70416 | 0.595633 | 0.166093 | -0.21127 | -0.47402 | 0.287612 | -0.45322 | -0.92196 | -2.82036 | -2.16996 | -3.09115 | -3.68653 | -3.76053 | -3.90786 | -2.60674 | -4.92787 | -2.64768 | -3.50936 | -3.18551 | -3.57644 |
| Q61625 | Q61625 | GRID2_M | Grid2 | Glutamate receptor ionotropic, delta-2 | | | | 0.304072 | 1.560466 | 0.824122 | 0.020953 | 0.051004 | 0.066686 | NaN | 0 | NaN | -3.12441 | 0.647168 | -2.11354 | -3.47643 | -2.9869 | -2.8075 | -2.62849 | -2.96969 | -2.49871 | -3.19327 | -2.97347 | -3.3013 |
| Q8VCQ3 | Q8VCQ3 | NRBF2_M | Nrbf2 | Nuclear receptor-binding factor 2 | + | | | 3.146537 | 0.997897 | 37.41416 | 0.935477 | 1.190088 | 2.673965 | 0.258875 | -0.31086 | -0.71071 | 0.99211 | 1.073913 | 2.195334 | 3.047546 | 0.280308 | 0.548482 | 0.028861 | 0.010283 | 1.301016 | 1.560089 | 0.308922 | 1.141583 |
| P35436 | P35436 | NMDE1_M | Grin2a | Glutamate receptor ionotropic, NMDA 2A | | | | 0.349545 | 1.169447 | 0.938272 | 0.828241 | -0.13502 | -2.29643 | 0.0918 | 0.185947 | 0.274483 | -0.11827 | 0.619764 | 0.164959 | 0.712593 | 2.250168 | 1.226785 | 0.320001 | -0.37072 | 0.871612 | 0.768002 | 1.108577 | 1.996488 |
| Q9QYF3 | Q9QYF3 | E9Q6Y8_M | Usp31 | ubiquitinyl hydrolase 1 | + | | | 1.975986 | 0.999344 | 9.650044 | 0.250308 | 0.219649 | 0.689148 | 0.299044 | -0.27008 | -0.81152 | 0.003173 | -0.04366 | 0.625317 | 1.180517 | 0.239958 | -0.08808 | -1.12046 | -0.91871 | 0.526668 | 0.839869 | 0.056436 | 0.635594 |
| Q925T6 | G3XA20;Q | GRIP1_M | Grip1 | Glutamate receptor-interacting protein 1 | | | | 0.017278 | 0.066035 | 0.0552 | 0.023408 | -0.02772 | -0.07431 | 0.019878 | -0.01909 | -0.06333 | -1.29747 | -2.79025 | -2.27493 | -0.43251 | -0.48242 | -0.21779 | -1.17502 | -3.04476 | 0.038651 | -0.69065 | -0.60182 | -0.06021 |
| Q8VD04 | Q8VD04 | GRAP1_M | Gripap1 | GRIP1-associated protein 1 | | | | 0.149163 | -0.32565 | -0.42965 | 0.472212 | 0.45956 | 1.252007 | 0.168434 | -0.35986 | -0.48012 | -3.20392 | -2.93157 | -2.2409 | -3.0053 | -2.54045 | -3.46816 | -3.48771 | -1.79967 | -3.09749 | -3.16553 | -2.05568 | -3.2332 |
| Q99MK8 | Q3UYH7;C | ARBK1_M | Grk2 | Beta-adrenergic receptor kinase 1 | | | | 0.297133 | -0.58626 | -0.92138 | 0.114393 | -0.08228 | -0.29319 | 0.135738 | 0.156226 | 0.313004 | -0.63142 | -0.66866 | 0.767031 | 1.135581 | -0.13464 | 0.59355 | -0.45957 | -0.41998 | 0.415195 | 0.601108 | 0.182052 | -0.28744 |
| P97772 | P97772 | GRM1_MC | Grm1 | Metabotropic glutamate receptor 1 | | | | 0.541767 | 1.222733 | 1.437134 | 0.798041 | 0.327657 | 2.196455 | 0.073571 | 0.055228 | 0.223106 | -0.64715 | 0.960582 | 0.120862 | 0.611572 | 0.661296 | 0.168186 | -0.80801 | -1.30442 | -0.30675 | -0.08193 | 0.337408 | 0.381617 |
| Q9WV18 | Q9WV18 | GABR1_M | Gabbr1 | Gamma-aminobutyric acid type B receptor subunit 1 | + | | | 1.631573 | 0.991177 | 6.427957 | 0.510604 | -0.27296 | -1.35339 | 0.03776 | -0.0509 | -0.11818 | 0.110697 | 0.041721 | 0.970908 | 1.028316 | 1.210088 | 0.46058 | -1.07458 | -0.7774 | 1.072939 | 1.47221 | 0.703889 | 1.014575 |
| Q9QYS2 | Q9QYS2 | GRM3_MO | Grm3 | Metabotropic glutamate receptor 3 | | | | 0.270401 | 0.249452 | 0.739673 | 0.579478 | -0.19765 | -1.55405 | 0.099758 | 0.119674 | 0.296655 | -0.40952 | 0.231347 | 1.779899 | 1.856809 | 1.078584 | 0.91471 | -0.1233 | 1.045174 | 1.815027 | 2.058236 | 1.241021 | 1.450023 |
| Q3UVX5 | Q3UVX5 | GRM5_MO | Grm5 | Metabotropic glutamate receptor 5 | | | | 0.200925 | 0.858995 | 0.56391 | 0.873309 | -0.40677 | -2.45065 | 0.040497 | 0.03481 | 0.126411 | -1.01507 | -1.38829 | -0.02555 | 0.249526 | 0.212758 | 0.701808 | -0.54887 | -3.57249 | 0.425838 | 0.611675 | 0.857183 | 1.006832 |
| Q68ED2 | Q68ED2 | GRM7_MO | Grm7 | Metabotropic glutamate receptor 7 | | | | 0.092738 | -0.3384 | -0.279078 | 0.810253 | -0.09889 | -2.23657 | 0.674776 | 0.785158 | 1.813335 | -3.73158 | -2.10544 | -1.47412 | -1.4638 | -0.1419 | -0.91761 | -1.66883 | -3.49138 | -1.41398 | -1.32616 | -0.11273 | -1.52709 |
| P28798 | P28798 | GRN_MOU | Grn | Progranulin | | | | 0.114529 | 0.34368 | 0.337001 | 0.3429 | 1.067733 | 0.921559 | 0.853967 | -0.66974 | -2.3837 | -1.94252 | -3.6147 | -0.84278 | -1.58837 | -4.22396 | -2.35941 | -2.53835 | -3.70623 | -3.37945 | -1.18637 | -2.00105 | -1.44284 |
| Q99LP6 | Q99LP6 | GRPE1_M | Grpel1 | GrpE protein homolog 1, mitochondrial | | | | 0.178341 | 0.60495 | 0.505824 | 0.60796 | 0.448144 | 1.627168 | -0.62745 | -1.41333 | -1.43207 | -1.43909 | -1.98907 | -1.89129 | -1.45637 | -3.60994 | -1.86161 | -1.84155 | -1.42135 | -1.9781 | | | |
| Q32M21;C | Q9ESI7;Q | GSDA2_M | Gsdma;Gsdma2;Gsdma3 | Gasdermin-A2 | + | | | 1.253539 | 2.40528 | 4.054954 | 0.08453 | -0.38586 | -0.25413 | 1.075327 | 0.351869 | 3.22737 | -0.35636 | -1.48911 | -3.149 | -2.2262 | -0.8456 | -1.02795 | -3.15175 | -3.50428 | -3.74858 | -0.8558 | -1.34842 | -1.22887 |
| Q2KHK6;C | Q8CB12;C | GSDC2_M | Gsdmc2;Gsdmc3 | Gasdermin-C2 | | | | 0.3529 | 1.417646 | 0.946719 | 0.536085 | -1.36228 | -1.42176 | NaN | 0 | NaN | -3.51906 | -0.52596 | -3.04717 | -3.47538 | -3.24977 | -3.60035 | -3.49156 | -3.38874 | -0.96506 | -2.83293 | -2.98153 | -3.80705 |
| Q8ZD03 | Q9Z2D3 | GSDME_M | Gsdme | Gasdermin-E | | | | 0.178341 | 0.147223 | 0.505825 | 0.383856 | -0.72297 | -1.27586 | 0.62735 | -0.54371 | -1.67525 | -1.24649 | -0.63716 | -0.42162 | -0.57901 | -0.09399 | -0.95363 | -1.45612 | -0.15544 | 0.290607 | -0.48398 | 0.061403 | |
| Q3Z7H4 | Q3Z7H4 | GSG1L_M | Gsg1l | Germ cell-specific gene 1-like protein | | | | NaN | 0 | NaN | 0.809196 | 0.602651 | 2.233083 | 0.354089 | 1.114662 | 0.949713 | -3.76647 | -2.80957 | -1.78763 | -0.5679 | -2.79945 | -1.03142 | -1.52021 | -3.2956 | -2.37542 | -3.32154 | -2.29546 | -3.74918 |
| Q8BGS7 | Q8BGS7 | CEPT1_M | Cept1 | Choline/ethanolaminephosphotransferase 1 | + | | | 2.036372 | 0.998008 | 10.35557 | 0.445112 | -0.16614 | -1.18109 | 0.217061 | -0.15372 | -0.60711 | 2.815918 | 2.740571 | 0.96611 | 1.23728 | 1.666881 | 1.519091 | 1.868941 | 1.691531 | 1.260164 | 1.265515 | 1.503628 | 1.989788 |
| Q9ET77 | Q9ET77 | JPH3_MO | Jph3 | Junctophilin-3 | + | | | 1.469168 | 0.991557 | 5.281822 | 0.265243 | -0.23248 | 0.719833 | 0.678862 | -0.5692 | -1.82057 | -0.48702 | -1.44745 | 1.172516 | 1.561244 | 0.415346 | 0.300103 | -1.63224 | -1.61997 | 1.609587 | 1.612083 | 0.855393 | 1.040584 |
| Q8R050 | Q149F3;Q | ERF3A_M | Gspt1 | Eukaryotic peptide chain release factor GTP-binding subunit ERF3A | | | | 0.062051 | 0.049742 | 0.18998 | 0.066306 | -0.11778 | -0.25912 | 0.214188 | 0.441449 | 0.595965 | -1.6935 | -1.22197 | -1.02931 | -1.71867 | -0.84409 | -1.0215 | -1.3936 | -1.55253 | -0.95983 | -0.91892 | -1.31515 | |
| Q06890 | Q06890 | CLUS_MO | Clu | Clusterin | + | + | | 3.163084 | 0.990795 | 38.13449 | 1.129422 | 0.634025 | 3.462796 | 0.224314 | 0.278893 | 0.623468 | 0.558355 | 0.562866 | 0.711917 | 2.448731 | 2.148743 | 1.447581 | -0.45618 | -0.40439 | 1.570784 | 1.727814 | 1.241433 | 1.797105 |
| P51855 | P51855 | GSHB_MC | Gss | Glutathione synthetase | + | + | | 0.832292 | -1.92196 | -2.31004 | 1.533273 | -1.57269 | -5.71385 | 0.100278 | 0.36838 | 0.29799 | -2.17256 | -3.58171 | -2.76249 | -3.12339 | -2.49953 | -2.01069 | -2.52723 | -1.34983 | -1.87828 | -1.45113 | -1.41172 | -2.83546 |
| Q9ESI1 | Q9ESI1 | CABL1_M | Cables1 | CDK5 and ABL1 enzyme substrate 1 | | | | 2.298041 | 0.986218 | 14.53009 | 0.646645 | -0.32163 | -1.2323 | 0.446479 | -0.36718 | -1.28561 | -0.15794 | -0.1474 | 0.718698 | 2.018167 | 1.871395 | 1.378665 | 2.199447 | 2.234885 | 3.092316 | 3.201244 | 2.011777 | 2.610944 |
| P10649 | P10649 | GSTM1_M | Gstm1 | Glutathione S-transferase Mu 1 | | | | 0.526562 | -0.49725 | -1.3961 | 0.240127 | 0.021137 | 0.248158 | 0.068265 | -0.09424 | -0.20792 | 1.422187 | 1.832583 | 3.278878 | 1.339778 | 1.491947 | 2.271241 | 2.415751 | 1.833513 | 1.666575 | 1.755487 | 2.207418 | 1.744254 |
| P48774 | P48774 | GSTM5_M | Gstm5 | Glutathione S-transferase Mu 5 | + | | | 1.145978 | -0.37487 | -3.37422 | 0.724431 | -0.38845 | -0.74477 | 0.545278 | -0.47318 | -0.92754 | 0.593927 | 0.825259 | 1.034382 | 0.206519 | 0.466303 | 1.269992 | 1.558245 | 1.265414 | 1.326232 | 0.691573 | 1.655593 | 1.027094 |
| P19157 | P19157 | GSTP1_M | Gstp1 | Glutathione S-transferase P 1 | | | | 0.270401 | 0.249452 | 0.739673 | 0.007727 | -0.00142 | -0.02353 | 0.028669 | 0.157 | 0.09514 | 1.100454 | 1.477563 | 0.957056 | 0.482214 | 1.191648 | 0.629638 | 0.806594 | 0.16424 | -0.26928 | 0.742805 | 0.813071 | |
| Q9WVL0 | Q9WVL0 | MAAI_MO | Gstz1 | Maleylacetoacetate isomerase | | | | 0.587276 | -0.53019 | -1.56218 | 0.06137 | 0.272657 | 0.188004 | 0.001095 | 0.005701 | 0.00336 | -1.45919 | -1.88409 | -1.10306 | -3.7917 | -3.17857 | -2.89737 | -0.87678 | -1.40612 | -2.17589 | -2.58188 | -0.95228 | -3.56757 |
| Q9ESZ8 | Q9ESZ8 | GTF2I_MO | Gtf2i | General transcription factor II-I | | | | 0.351332 | 0.104482 | 0.942769 | 0.777012 | -0.23647 | -2.12835 | 0.360483 | 0.38836 | 1.024894 | 0.703752 | 0.533768 | 0.234103 | 0.335721 | 0.308636 | 0.423277 | 0.442778 | 0.185549 | 0.620189 | 0.422575 | 0.313961 | 0.291363 |
| Q8K284 | Q8K284 | TF3C1_M | Gtf3c1 | General transcription factor 3C polypeptide 1 | | | | 0.051968 | -0.24202 | -0.16052 | 0.556184 | 0.5114 | 1.476377 | 0.455927 | 0.78678 | 1.209617 | -5.05994 | -2.18769 | -1.49286 | -1.46495 | -0.94721 | -1.29265 | -0.92252 | -3.84106 | -2.33641 | -1.6442 | -2.65156 | -1.79887 |
| O08582 | O08582 | GTPB1_M | Gtpbp1 | GTP-binding protein 1 | | | | 0.150335 | 0.155795 | 0.154657 | 0.288026 | 0.444107 | 0.676125 | -0.62745 | -1.61548 | -2.53492 | -1.71823 | -2.83189 | -2.08597 | -1.59325 | -3.03937 | -0.56947 | -1.67799 | -1.38907 | -1.55185 | -1.66844 | -1.54998 | -0.11604 |
| Q9ERL9 | Q9ERL9 | GCYA1_M | Gucy1a1 | Guanylate cyclase soluble subunit alpha-1 | | | | 0.003253 | 0.003087 | 0.010555 | 0.449029 | 0.445353 | 1.299613 | 0.290511 | 0.191378 | 0.863116 | -1.30809 | -1.38801 | -2.28761 | -0.96284 | -1.2006 | -2.96656 | -3.5919 | -3.01186 | -1.28768 | -3.36158 | -2.61558 | -2.24524 |
| F8VQK3 | F8VQK3 | F8VQK3_M | Gucy1a2 | guanylate cyclase | | | | 0.799315 | 0.377609 | 2.200622 | 1.062406 | 0.252118 | 3.173003 | 0.049935 | -0.03832 | -0.15452 | -0.21598 | -0.24072 | 0.91547 | 0.901022 | 0.43964 | 0.075587 | -0.43481 | -0.7771 | 0.735256 | 0.576999 | 0.127341 | 0.468533 |
| O54865 | O54865 | GCYB1_M | Gucy1b1 | Guanylate cyclase soluble subunit beta-1 | | | | 0.478266 | 0.293421 | 0.828906 | 0.136462 | 0.129845 | 0.390655 | 0.99135 | -0.42095 | -0.90583 | -0.78232 | -0.49968 | -2.26883 | -0.86302 | -1.70282 | -1.55756 | -0.86074 | -0.24207 | -2.03827 | -0.99296 | -0.90646 | -0.63444 |
| Q9Z1E4 | Q9Z1E4 | GYS1_MO | Gys1 | Glycogen [starch] synthase, muscle | + | | | 0.469055 | 0.285917 | 1.24581 | 1.324291 | -0.669 | -4.42861 | 0.991105 | -0.88596 | -2.88516 | 0.078103 | 0.901843 | 0.225027 | -0.0281 | -0.1194 | -0.3121 | 0.547174 | 0.299819 | 0.651405 | 0.586789 | 0.571177 | 0.987777 |
| P10922 | P10922 | H10_MOU | H1-0 | Histone H1.0 | | | | 0.264631 | 0.609076 | 0.72518 | 0.35155 | 0.370921 | 0.943318 | 0.794663 | 0.538907 | 2.185434 | 2.653609 | 1.953043 | 0.715061 | 1.447643 | 1.970997 | 1.483585 | 2.457616 | 0.930883 | 0.853414 | 0.567447 | 1.150777 | 1.22599 |
| P15864 | P15864 | H12_MOU | H1-2 | Histone H1.2 | | | | 0.169795 | 0.478487 | 0.48366 | 0.188304 | -0.26496 | -0.53153 | 0.611026 | 0.297044 | 1.826866 | 3.471719 | 2.539293 | 1.393513 | 2.313782 | 2.514233 | 2.203552 | 3.399583 | 1.654465 | 2.33589 | 1.910324 | 1.966321 | 2.157375 |
| P43274 | P43274 | H14_MOU | H1-4 | Histone H1.4 | | | + | 0.390707 | 0.814083 | 1.042277 | 0.038684 | -0.05236 | -0.11475 | 0.982874 | 0.692506 | 3.022654 | 3.475936 | 2.649029 | 1.384873 | 3.072906 | 3.721889 | 3.26917 | 4.181934 | 2.849557 | 2.767749 | 2.392955 | 2.619974 | |
| P43276 | P43276 | H15_MOU | H1-5 | Histone H1.5 | | | + | 0.567652 | 1.220549 | 1.587584 | 0.358349 | 0.477838 | 0.960445 | 1.475876 | 0.350182 | 5.331287 | 1.603247 | 1.676929 | 0.150825 | 0.892052 | 1.572041 | 0.717539 | 1.228172 | -0.38909 | 0.375517 | -0.28832 | 0.266881 | 0.407534 |
| Q61687 | Q61687 | ATRX_MO | Atrx | Transcriptional regulator ATRX | + | | | 1.465401 | 0.976172 | 5.264022 | 0.046689 | -0.05723 | -0.1449 | 0.162257 | -0.21632 | -0.44061 | 0.135988 | 0.472832 | 1.078711 | 1.859536 | 0.884108 | 0.27675 | -0.79437 | 1.469018 | 1.58285 | 0.222503 | 1.094081 | |
| Q64522 | Q64522;P | H2A2B_M | H2ac21 | Histone H2A type 2-B | | | | 0.453508 | 0.377105 | 1.156808 | 0.685494 | -0.8282 | -1.84519 | NaN | 0 | NaN | -0.2007 | -2.87172 | -3.11716 | -3.20839 | -3.54244 | -2.8639 | -2.82999 | -1.76026 | -4.00313 | -2.62869 | | |
| P0C0S6;Q | Q3THW5;F | H2AV_MO | H2az1;H2az2 | Histone H2A.Z | | | | 0.367952 | 0.829966 | 0.837353 | 0.120241 | 0.268978 | 0.35248 | 0.576004 | -0.34275 | -1.53088 | 3.950799 | 3.493906 | 2.580478 | 3.016718 | 3.703727 | 2.081046 | 3.237069 | 2.861921 | 3.206592 | 3.252569 | | |
| P10853;P | P10854;Q | H2B1B_M | H2bc12;H2bc14;H2bc15;H2bc3;H2bc8;H2bc6 | Histone H2B type 1-F/J/L | | | | 0.40037 | 0.846983 | 1.066844 | 0.145325 | 0.572256 | 0.419514 | 0.562604 | 0.394247 | 1.493958 | 7.320443 | 6.692824 | 5.09761 | 5.844158 | 5.804128 | 6.889519 | 5.436235 | 5.967817 | 6.678684 | 6.556407 | 5.69303 | |
| Q64524;Q | Q64524;Q | H2B2E_M | H2bc21;H2bc26;H2bu2 | Histone H2B type 2-E | | | | 0.945387 | 1.418761 | 2.710794 | 0.015476 | 0.041185 | 0.049539 | 0.096649 | -0.58658 | -0.28795 | 1.262269 | 0.978561 | -0.68823 | 0.604408 | 1.100619 | -0.07216 | -1.14594 | 0.190791 | -3.07234 | | | |
| P02301;P | P84244;P | H31_MOU | H3-3b;H3-5;H3c11;H3c15 | Histone H3.3C | | | | 0.362521 | 0.999659 | 0.970964 | 0.156971 | 0.719949 | 0.552813 | 0.265309 | 0.277859 | 0.726883 | 5.221968 | 4.341855 | 3.008868 | 5.165891 | 3.900137 | 3.499839 | 4.71302 | 2.851484 | 4.097426 | 2.637435 | 3.096454 | 3.747804 |
| P62806 | P62806 | H4_MOUS | H4c16 | Histone H4 | | | | 0.539734 | 1.056797 | 1.431626 | 0.237733 | 0.890475 | 0.657428 | 0.282131 | 0.250958 | 0.769111 | 6.825987 | 6.477286 | 4.26014 | 6.492701 | 4.911935 | 5.177166 | 6.309019 | 4.875536 | 5.253113 | 4.495461 | 5.091722 | |
| Q9JKS5 | Q9JKS5 | HABP4_M | Habp4 | Intracellular hyaluronan-binding protein 4 | | | | 0.50919 | -0.71943 | -1.49742 | 0.396067 | -0.29353 | -1.0559 | 0.387268 | -0.28797 | -1.03355 | -1.51197 | -0.44688 | -0.28235 | -0.27024 | -0.29102 | 0.1686 | -0.28357 | -0.26069 | 0.295168 | 0.383403 | 0.069221 | |
| Q8K2C9 | Q8K2C9 | HACD3_M | Hacd3 | Very-long-chain (3R)-3-hydroxyacyl-CoA dehydratase 3 | | | | 0.686859 | 0.311058 | 1.84927 | 0.860146 | 0.224672 | 2.404964 | 0.052919 | 0.065065 | 0.163315 | -0.22353 | 0.392857 | 2.0061 | 1.72096 | 1.61141 | -2.53362 | 1.480586 | 1.372475 | 2.808176 | 1.143787 | 1.42266 | 1.69598 |
| Q61425 | Q61425 | HCDH_MO | Hadh | Hydroxyacyl-coenzyme A dehydrogenase, mitochondrial | | | | 0.749687 | -0.50251 | -2.01462 | 0.21955 | -0.55322 | -0.61138 | 0.112071 | 0.177819 | 0.330314 | -0.29847 | -0.01679 | -0.45192 | -2.23562 | -0.81688 | -0.19542 | 0.637956 | -0.94353 | -0.31768 | -0.65122 | | |
| Q8BMS1 | Q8BMS1 | ECHA_MC | Hadha | Trifunctional enzyme subunit alpha, mitochondrial | | | | 0.917919 | -0.44141 | -2.6896 | 0.005607 | 0.005211 | 0.018143 | 0.351694 | 0.474947 | 0.943681 | 1.467535 | 1.743097 | 1.055468 | 0.514186 | 2.067318 | 1.115776 | 2.144855 | 1.948607 | 0.875721 | 0.683511 | 0.952454 | 1.280746 |
| Q99JY0 | Q99JY0 | ECHB_MO | Hadhb | Trifunctional enzyme subunit beta, mitochondrial | | | | 0.660414 | -0.4701 | -1.77104 | 0.400415 | -0.56773 | -1.07638 | 0.501549 | 0.289467 | 1.269379 | 0.400548 | 0.058575 | 1.716885 | 1.766236 | 2.793445 | 2.003305 | 1.301111 | 0.940427 | 1.668447 | 1.23574 | | |
| Q99KB8 | Q99KB8 | GLO2_MO | Hagh | Hydroxyacylglutathione hydrolase, mitochondrial | | | | 0.19374 | 0.064655 | 0.5025 | 0.4155 | 0.752473 | 0.792329 | 0.205037 | 0.51503 | 0.353743 | 0.524563 | 0.30771 | 0.975527 | 0.685202 | 0.708009 | 1.064042 | 0.303998 | 0.398346 | -0.32009 | 0.396166 | 0.696815 | 0.542955 |
| Q9D8B2 | Q9D8B2 | HAGHL_M | Haghl | Hydroxyacylglutathione hydrolase-like protein | | | | 0.56694 | 0.509896 | 1.505874 | 0.235809 | 0.311802 | 0.652566 | 0.646646 | -0.15653 | -1.73091 | 2.301081 | 1.948277 | 1.464212 | 1.605013 | 1.816395 | 1.291201 | 1.326386 | 1.90396 | 0.750217 | 1.695405 | 1.321626 | 1.469025 |
| P35492 | P35492 | HUTH_MO | Hal | Histidine ammonia-lyase | | | | 0.075832 | -0.172788 | 0.229544 | 0.756215 | -0.99636 | -2.059 | 0.126383 | 0.219665 | 0.362091 | -0.15445 | -3.73513 | -1.72658 | -2.0414 | -1.48747 | -1.54492 | -1.51793 | -0.48441 | -1.34521 | -0.43004 | -2.33041 | -1.1413 |
| O35668 | O35668 | HAP1_MO | Hap1 | Huntingtin-associated protein 1 | | | | 0.655003 | 0.762859 | 1.75522 | 0.031146 | -0.05079 | -0.09811 | 0.393743 | -0.74334 | -1.04999 | 0.863501 | 0.042083 | -0.22695 | 0.786352 | -0.66765 | -1.02926 | -0.16789 | -0.45225 | 0.223836 | 0.437154 | -0.78959 | 0.579363 |
| Q9QUP5 | Q9QUP5 | HPLN1_M | Hapln1 | Hyaluronan and proteoglycan link protein 1 | | | | 0.557765 | -0.44333 | -1.4807 | 0.473715 | 0.515275 | 1.255989 | 0.357053 | 0.15197 | 0.957179 | 0.722349 | 0.909526 | 2.736236 | 2.234727 | 1.202458 | 1.122874 | 1.543673 | 0.974865 | 0.197107 | 0.140736 | 1.164397 | 0.856996 |
| Q80WM4 | Q80WM4 | HPLN4_M | Hapln4 | Hyaluronan and proteoglycan link protein 4 | + | | | 0.223414 | 1.003749 | 0.621186 | 1.150521 | 1.222275 | 5.833616 | 0.426495 | 0.725161 | 1.230986 | 2.017774 | 2.070635 | 0.85135 | 0.957564 | 0.022976 | -0.16012 | -1.14317 | -1.38022 | -0.20329 | -0.06529 | | |
| Q61035 | Q99KK9;Q | HARS1_M | Hars1 | Histidine--tRNA ligase, cytoplasmic | | | | 0.976067 | -0.41592 | -2.82996 | 0.001184 | 0.000717 | 0.003851 | 0.519177 | -0.21114 | -0.49269 | -0.76621 | -0.81289 | -0.54777 | -0.89055 | -2.13577 | -0.95252 | -0.86185 | -0.96308 | -0.4774 | -0.66165 | -0.63802 | |
| F8VPU2 | F8VPU2 | FARP1_M | Farp1 | FERM, ARHGEF and pleckstrin domain-containing protein 1 | + | | | 2.039029 | 0.969127 | 10.37874 | 0.096581 | -0.07053 | -0.28776 | 0.112371 | -0.12738 | -0.33133 | 1.04127 | 1.225008 | 1.486166 | 1.914501 | 1.645734 | 1.339529 | 0.147759 | 0.180263 | 1.651662 | 1.890069 | 1.2671 | 1.972932 |
| O35387 | O35387 | HAX1_MO | Hax1 | HCLS1-associated protein X-1 | + | | | 0.338935 | -0.60758 | -0.91159 | 1.203887 | 1.428639 | 3.80888 | 0.116678 | 0.231817 | 0.342835 | -3.75075 | -3.05829 | -0.58506 | -0.57675 | -1.2237 | -2.32987 | -2.36655 | -3.67733 | -2.3846 | -1.63448 | -2.39759 | -1.61961 |

| | | | | | | | | | | | | | | | | | | | | | | | | | | | | |
|---|---|---|---|---|---|---|---|---|---|---|---|---|---|---|---|---|---|---|---|---|---|---|---|---|---|---|---|---|
| P01942 | P01942 | HBA_MOU | Hba | Hemoglobin subunit alpha | | | | 0.084624 | -0.18135 | -0.2544 | 0.111986 | -0.31943 | -0.33008 | 0.056578 | -0.15308 | -0.17404 | 4.593866 | 6.014314 | 5.029886 | 3.107865 | 3.796658 | 5.291141 | 5.424194 | 5.546688 | 4.502075 | 4.274532 | 5.160859 | 4.23309 |
| A8DUK4 | P02088;A | A8DUK4_I | Hbb-bs | Beta-globin | | | | 0.103973 | 0.156342 | 0.30816 | 0.028848 | -0.06642 | -0.09107 | 0.199304 | -0.23479 | -0.55976 | 5.632367 | 6.634235 | 5.558216 | 4.10658 | 4.705792 | 5.500162 | 6.057329 | 5.896589 | 4.969854 | 4.82778 | 5.472631 | 5.202912 |
| P53702 | P53702 | CCHL_MO | Hccs | Holocytochrome c-type synthase | | + | | 0.037001 | -0.03896 | -0.11588 | 1.152418 | 0.216835 | 3.56686 | 0.545775 | 0.291532 | 1.448011 | -0.18025 | -0.37522 | -0.70328 | -0.77229 | -0.48117 | -0.32975 | -0.56056 | 0.083015 | -0.90457 | -1.00467 | -0.51044 | -0.88355 |
| Q61191 | Q61191 | HCFC1_M | Hcfc1 | Host cell factor 1 | | + | | 0.587205 | -0.66397 | -1.56198 | 1.357084 | -0.38607 | -4.61157 | 0.133107 | 0.169945 | 0.387046 | -3.88079 | -3.21799 | -0.99019 | -0.90802 | -0.82084 | -1.15737 | -3.15164 | -2.61921 | -0.63598 | -0.4901 | -1.56462 | -0.75349 |
| O88704 | O88704;O | HCN1_MO | Hcn1 | Potassium/sodium hyperpolarization-activated cyclic nucleotide-gated channel 1 | | | | 0.672963 | 1.171162 | 1.807973 | 0.114897 | 0.120093 | 0.338001 | 0.764979 | 0.446054 | 2.089912 | -0.48076 | 0.328836 | -0.40692 | -0.54342 | 0.088407 | -0.27755 | -1.75284 | -0.74141 | -0.24657 | -0.94394 | -0.6505 | -0.43076 |
| O88703 | O88703 | HCN2_MO | Hcn2 | Potassium/sodium hyperpolarization-activated cyclic nucleotide-gated channel 2 | | | | 0.720434 | -0.90887 | -1.95088 | 0.107303 | 0.117621 | 0.317292 | 0.089695 | -0.22115 | -0.26861 | -0.69669 | 0.171737 | -0.78242 | -0.6386 | -0.24246 | -1.6913 | 0.477573 | 0.81521 | -1.19179 | -0.46447 | -1.13696 | -0.3545 |
| Q6P3E7 | Q6P3E7 | HDA10_M | Hdac10 | Polyamine deacetylase HDAC10 | | + | | 0.478774 | 0.738198 | 1.269245 | 1.023779 | 0.730571 | 3.014572 | 0.514911 | 0.457459 | 1.364882 | 0.351679 | 1.271719 | 0.621456 | 0.920758 | 0.003743 | -0.40383 | -0.28237 | 0.429373 | -0.15008 | 0.231157 | -0.9236 | -0.39141 |
| Q91WA3 | Q91WA3 | HDA11_M | Hdac11 | Histone deacetylase 11 | | | + | 0.226447 | 0.281807 | 0.628874 | 0.134751 | 0.130777 | 0.391434 | 1.231102 | -0.63285 | -3.94217 | -0.77525 | -1.28926 | -0.98493 | -0.32866 | -1.62317 | -1.31167 | -1.68115 | -0.94697 | -0.85039 | -0.72475 | -0.87347 | -0.79566 |
| P70288 | P70288 | HDAC2_M | Hdac2 | Histone deacetylase 2 | | | | 0.890684 | 1.758667 | 2.511785 | 0.049615 | 0.052867 | 0.153573 | 0.755704 | -0.41981 | -2.06055 | -0.83416 | -0.11975 | 0.937318 | 1.486086 | 0.177196 | 0.327055 | -1.63343 | -2.83782 | 0.950942 | 1.366728 | 0.482478 | 0.861397 |
| Q6NZM9 | Q6NZM9 | HDAC4_M | Hdac4 | Histone deacetylase 4 | | | + | NaN | 0 | NaN | 0.245334 | 0.909535 | 0.676612 | 1.584484 | -0.56933 | -6.07619 | -2.87525 | -3.54418 | -1.47411 | -1.81061 | -2.1935 | -2.17922 | -3.38941 | -3.52565 | -3.88557 | -1.21822 | -1.71046 | -1.52361 |
| Q9Z2V6 | Q99N13;Q | HDAC5_M | Hdac5 | Histone deacetylase 5 | | | | 0.140642 | 0.538592 | 0.407107 | 0.720116 | -1.58788 | -1.9499 | 0.294635 | -1.15236 | -0.80047 | -3.55407 | -0.90826 | -1.42405 | -3.01994 | -3.84543 | -1.01826 | -2.78348 | -2.75603 | -0.47155 | -0.79669 | -1.55198 | -1.00699 |
| Q8CD19 | Q8CD19 | LANC3_M | Lancl3 | LanC-like protein 3 | + | | | 1.782605 | 0.966805 | 7.689127 | 0.672087 | -0.58429 | -1.80538 | 0.178096 | 0.24404 | 0.505189 | 1.803922 | 1.805915 | -0.48809 | -0.08901 | 0.359838 | -0.4381 | 0.712381 | 0.963847 | 0.550539 | 0.040939 | -0.55552 | -0.01081 |
| P63166 | P63166 | SUMO1_M | Sumo1 | Small ubiquitin-related modifier 1 | + | + | + | 2.656688 | 0.950277 | 21.26303 | 1.436025 | 1.447902 | 5.079437 | 1.237992 | -0.81389 | -3.97652 | -2.32442 | -2.2387 | -1.95015 | -1.38797 | -3.29005 | -3.13653 | -3.2445 | -3.21917 | -3.16432 | -3.0696 | -2.58914 | -2.20967 |
| Q3UGR5 | Q3UGR5 | HDHD2_M | Hdhd2 | Haloacid dehalogenase-like hydrolase domain-containing protein 2 | | | | 0.822537 | -0.48719 | -2.27735 | 0.603633 | -0.46402 | -1.60799 | 0.215652 | -0.36368 | -0.60147 | -1.93673 | -1.51647 | -1.49624 | -2.07336 | -1.71615 | -0.50784 | -1.19927 | -1.27955 | -1.32312 | -1.31846 | -0.72366 | -0.77296 |
| Q8VDJ3 | Q8VDJ3 | VIGLN_MC | Hdlbp | Vigilin | | | | NaN | 0 | NaN | 0.021482 | -0.0607 | -0.06833 | 0.059579 | -0.04592 | -0.1828 | -3.41764 | -2.92934 | -1.76911 | -2.57859 | -1.65967 | -1.49159 | -3.00709 | -3.42111 | -2.9039 | -1.3224 | -1.76645 | -1.29296 |
| P38647 | P38647 | GRP75_M | Hspa9 | Stress-70 protein, mitochondrial | + | + | | 1.643769 | 0.936233 | 6.522101 | 1.08338 | 0.598378 | 3.261609 | 0.399617 | 0.29173 | 1.064929 | 3.177783 | 3.2858 | 3.640701 | 3.841773 | 3.516866 | 3.157377 | 2.428559 | 2.162559 | 2.989398 | 3.29632 | 2.838663 | 3.25212 |
| Q69ZR2 | Q69ZR2 | HECD1_M | Hectd1 | E3 ubiquitin-protein ligase HECTD1 | | | | 0.036784 | 0.093886 | 0.115229 | 0.099828 | -0.06448 | -0.29674 | 0.189417 | 0.209639 | 0.534393 | -1.51188 | -1.35218 | -1.23147 | -0.81793 | -1.12751 | -1.36376 | -0.71506 | -2.33676 | -1.02705 | -0.89339 | -1.82936 | -1.08119 |
| Q3U487 | Q3U487 | HECD3_M | Hectd3 | E3 ubiquitin-protein ligase HECTD3 | | | | 0.013687 | 0.057188 | 0.043895 | 0.318417 | -0.42858 | -0.86009 | 0.688119 | 0.238072 | 1.853036 | -4.30479 | -1.78863 | -1.74426 | -1.71692 | -1.02703 | -1.26461 | -3.44237 | -2.76542 | -1.80012 | -0.8039 | -1.43284 | -1.33494 |
| Q9ER35 | Q9ER35 | FN3K_MOI | Fn3k | Fructosamine-3-kinase | + | + | | 1.745915 | 0.933836 | 7.36289 | 1.025087 | 0.252921 | 3.019839 | 0.007172 | 0.010651 | 0.023165 | 2.105988 | 1.946979 | 1.284951 | 1.452456 | 1.241894 | 0.751327 | 0.99383 | 1.191465 | 1.115978 | 1.115588 | 0.597058 | 1.374861 |
| Q8K4P8 | Q8K4P8 | HECW1_M | Hecw1 | E3 ubiquitin-protein ligase HECW1 | | | | 0.173536 | 0.529159 | 0.493377 | 0.061741 | 0.086154 | 0.189079 | 0.676153 | -1.0556 | -1.81742 | -1.62452 | -3.31133 | -1.35867 | -0.61061 | -2.95609 | -2.59854 | -2.33453 | -3.65963 | -1.33102 | -0.81057 | -2.27434 | -1.16909 |
| Q6I6G8 | Q6I6G8 | HECW2_M | Hecw2 | E3 ubiquitin-protein ligase HECW2 | | | + | 0.507022 | 1.286363 | 1.343855 | 0.70918 | -0.64907 | -1.91652 | 1.113371 | -0.84654 | -3.39159 | -0.8555 | -2.3619 | -0.82278 | -0.66474 | -1.62769 | -1.70971 | -3.48579 | -2.30433 | -0.42401 | 0.234631 | -1.06837 | -0.57596 |
| Q640R3 | Q640R3 | HECAM_M | Hepacam | Hepatic and glial cell adhesion molecule | | | | 0.196485 | 0.127116 | 0.552539 | NaN | 0 | NaN | 0.287692 | 1.19425 | 0.783058 | -0.39663 | -0.28459 | -3.54253 | -3.64708 | -0.52242 | -3.54839 | -0.2446 | -0.69086 | -3.68559 | -2.89775 | -3.42159 | -3.03771 |
| E9PZP8 | E9PZP8 | E9PZP8_M | Herc1 | HECT and RLD domain containing E3 ubiquitin protein ligase family member 1 | | | | NaN | 0 | NaN | 0.764236 | -0.16051 | -2.08755 | 0.252039 | -0.20897 | -0.69351 | -3.01759 | -2.64467 | -1.61355 | -1.76551 | -2.01891 | -2.01532 | -3.12228 | -3.18377 | -1.54082 | -1.51721 | -2.10946 | -1.50682 |
| A6H6S0 | A6H6S0 | A6H6S0_M | Herc3 | Hect domain and RLD 3 | | | | 0.158512 | 0.304314 | 0.454217 | 0.172395 | -0.07669 | -0.49041 | 0.203026 | 0.192715 | 0.569281 | -2.49235 | -3.77591 | -1.62007 | -1.91211 | -1.56311 | -2.07774 | -3.63075 | -3.24614 | -1.74537 | -1.63343 | -1.79316 | -2.23311 |
| Q6PAV2 | Q6PAV2 | HERC4_M | Herc4 | Probable E3 ubiquitin-protein ligase HERC4 | | | | 0.073152 | -0.34307 | -0.22191 | 0.802662 | -0.3451 | -2.21159 | 0.336529 | -0.16946 | -0.90555 | -1.67022 | -4.55434 | -0.86412 | -0.95664 | -0.64305 | -0.96966 | -2.21202 | -3.3264 | -0.7104 | -0.40915 | -0.54551 | -0.72828 |
| P20060 | P20060 | HEXB_MO | Hexb | Beta-hexosaminidase subunit beta | | | + | 0.922696 | -1.19337 | -2.62701 | 0.2176 | -0.30956 | -0.60642 | 1.062208 | 0.270702 | 3.172174 | -0.98349 | -1.88995 | -0.27683 | -0.90029 | -0.49254 | -0.65937 | -0.27401 | -0.21268 | -0.68323 | 0.125236 | -0.82863 | -0.86468 |
| Q99LI8 | Q99LI8 | HGS_MOU | Hgs | Hepatocyte growth factor-regulated tyrosine kinase substrate | + | + | | 1.17109 | 0.845096 | 3.653175 | 1.990414 | 0.735197 | 9.814251 | 0.614695 | 0.415462 | 1.639261 | -0.7195 | -1.17912 | 0.241847 | 0.159433 | -0.43816 | -0.53708 | -1.82088 | -1.76793 | -0.59712 | -0.472 | -1.15165 | -0.65451 |
| Q80YA9 | Q80YA9 | CNKR2_M | Cnksr2 | Connector enhancer of kinase suppressor of ras 2 | + | | | 1.55357 | 0.93029 | 5.855024 | 0.394166 | 0.170045 | 1.051065 | 0.525301 | -0.73926 | -1.39271 | 0.276244 | -0.04056 | 3.08934 | 3.344838 | 2.183496 | 2.921401 | -0.80004 | -0.82485 | 2.947777 | 3.146311 | 2.910092 | 3.673314 |
| P60904 | P60904 | DNJC5_M | Dnajc5 | DnaJ homolog subfamily C member 5 | + | | | 1.832449 | 0.929892 | 8.154405 | 0.187953 | 0.4507 | 0.530626 | 0.323351 | 0.689515 | 0.872464 | -1.2648 | -1.12932 | 0.363389 | -1.31271 | -0.93529 | -1.06292 | -2.21869 | -2.03522 | -0.78713 | -1.06359 | -0.90089 | -2.47635 |
| Q8R1F6 | Q8R1F6 | HID1_MOI | Hid1 | Protein HID1 | | | | NaN | 0 | NaN | 0.287708 | -0.88856 | -0.7831 | 0.80156 | 0.513754 | 2.207974 | -3.58647 | -3.39755 | -3.76171 | -1.51448 | -1.08533 | -1.15368 | -3.27073 | -3.23819 | -1.59155 | -1.90752 | -1.86342 | -1.4031 |
| P70349 | P70349 | HINT1_MO | Hint1 | Adenosine 5'-monophosphoramidase HINT1 | | | | 0.642019 | -1.34505 | -1.71751 | 0.03077 | -0.11618 | -0.09696 | 0.214914 | -0.23332 | -0.59959 | -0.16389 | 0.292149 | 1.140646 | -1.25186 | 0.156313 | 0.351239 | 0.620567 | 1.197792 | 0.129749 | -0.0086 | 0.86381 | 0.110373 |
| Q9JKY5 | Q9JKY5 | HIP1R_MC | Hip1r | Huntingtin-interacting protein 1-related protein | | + | + | 0.56301 | -0.66481 | -1.49507 | 1.319841 | 0.326701 | 4.404271 | 1.545806 | 0.437993 | 5.80065 | -1.42581 | -2.08452 | -0.50521 | -0.44897 | -0.85039 | -0.94703 | -1.3891 | -0.79161 | -0.73515 | -0.87243 | -1.39473 | -1.27868 |
| Q3UHF7 | Q3UHF7 | ZEP2_MOL | Hivep2 | Transcription factor HIVEP2 | | | | 0.922246 | 0.47975 | 2.625372 | 0.335147 | -0.19447 | -0.90208 | 0.794901 | -0.86488 | -2.18621 | 0.265221 | 0.418395 | 2.125338 | 2.545959 | 1.883872 | 2.433095 | 0.02797 | -0.30385 | 2.577523 | 2.482725 | 2.738593 | 3.30813 |
| P17710 | P17710 | HXK1_MOI | Hk1 | Hexokinase-1 | + | | | 1.020828 | -0.73223 | -3.00271 | 0.237638 | 0.165664 | 0.657189 | 0.765992 | 0.496028 | 2.093135 | 2.002203 | 2.105014 | 3.439023 | 2.935342 | 3.577608 | 3.409597 | 3.024218 | 2.547464 | 3.032501 | 3.010536 | 3.219164 | 2.775985 |
| Q91W97 | Q91W97 | HKDC1_M | Hkdc1 | Hexokinase HKDC1 | | | | 0.146578 | -0.32828 | -0.42283 | 0.357521 | -1.51721 | -0.95836 | 0.057203 | 0.05618 | 0.175871 | -2.0007 | -0.5331 | -0.27172 | -3.42926 | 0.276046 | 0.166124 | -1.19225 | -0.68499 | -0.45083 | -0.21572 | 0.479581 | -0.14977 |
| D3YXG0 | D3YXG0 | HMCN1_M | Hmcn1 | Hemicentin-1 | | | | 0.207549 | 0.58707 | 0.58083 | NaN | 0 | NaN | 0.035761 | 0.180761 | 0.112135 | -3.51005 | -1.88409 | -4.06531 | -3.6229 | -1.437 | -1.09842 | -3.8847 | -2.68359 | -3.2938 | -3.00511 | -3.05156 | 0.154607 |
| P63158 | P63158 | HMGB1_M | Hmgb1 | High mobility group protein B1 | | | | 0.192661 | 1.03263 | 0.54273 | 0.004045 | 0.020221 | 0.013112 | 0.168492 | -0.48676 | -0.48027 | -3.90384 | -0.09854 | -0.93182 | -4.01573 | -1.70521 | -3.63568 | -3.02768 | -3.03995 | -2.52086 | -2.46713 | -1.87459 | -2.49277 |
| P38060 | P38060 | HMGCL_M | Hmgcl | Hydroxymethylglutaryl-CoA lyase, mitochondrial | | + | | 0.186448 | 0.146338 | 0.526752 | 1.051987 | -0.53206 | -3.12967 | 0.234855 | 0.235174 | 0.650154 | 1.919308 | 2.000687 | 0.817752 | 0.573361 | 1.844185 | 2.264905 | 1.538843 | 2.088476 | 1.345817 | 1.109424 | 2.113633 | 1.525109 |
| Q8JZK9 | Q8JZK9 | HMCS1_M | Hmgcs1 | Hydroxymethylglutaryl-CoA synthase, cytoplasmic | | | | 0.404253 | 0.644329 | 1.076736 | 0.105318 | -0.21704 | -0.31185 | 0.076157 | 0.097097 | 0.230467 | -1.51263 | -1.35242 | -2.06517 | -2.85927 | -1.64553 | -2.05386 | -2.66988 | -1.48383 | -2.81678 | -1.67358 | -1.57826 | -2.31532 |
| O70252 | O70252 | HMOX2_M | Hmox2 | Heme oxygenase 2 | | | | 0.425016 | -0.90003 | -1.12983 | 0.234892 | -0.54561 | -0.65025 | 0.091409 | 0.174343 | 0.273392 | -1.65129 | -3.2365 | -0.99221 | -2.62745 | -1.59233 | -2.0349 | -1.6236 | -1.46413 | -1.45274 | -1.0757 | -1.38988 | -2.58603 |
| P62267 | P62267 | RS23_MOI | Rps23 | Small ribosomal subunit protein uS12 | + | | | 1.499446 | 0.926843 | 5.485479 | 0.356738 | -0.17496 | -0.95638 | 0.133957 | -0.11628 | -0.38931 | 1.295142 | 1.400954 | 1.586176 | 1.915948 | 1.497182 | 1.68724 | 0.581671 | 0.260738 | 2.00527 | 1.846777 | 1.425326 | 1.991662 |
| P17426 | P17426 | AP2A1_M | Ap2a1 | AP-2 complex subunit alpha-1 | + | | | 1.632746 | 0.922605 | 6.436958 | 0.850799 | 0.552043 | 2.37285 | 0.333415 | -0.49816 | -0.89773 | 5.195467 | 5.160936 | 6.215034 | 6.492525 | 4.013597 | 5.005519 | 4.397882 | 4.113311 | 5.614987 | 5.988487 | 4.758824 | 5.256604 |
| O88569 | O88569 | ROA2_MO | Hnrnpa2b1 | Heterogeneous nuclear ribonucleoproteins A2/B1 | | | + | 0.83722 | 0.555257 | 2.326668 | 0.319556 | 0.477364 | 0.862945 | 1.625799 | 0.721473 | 6.38383 | 2.697156 | 2.947169 | 2.018007 | 3.122106 | 2.948847 | 2.726144 | 2.470196 | 2.063616 | 2.12805 | 2.057333 | 2.135347 | 2.096699 |
| Q8BG05 | Q8BG05 | ROA3_MO | Hnrnpa3 | Heterogeneous nuclear ribonucleoprotein A3 | | | | 0.463723 | 0.407206 | 1.229896 | 0.365381 | 0.432294 | 0.97818 | 0.490282 | 0.498249 | 1.299515 | 2.293536 | 2.10943 | 1.455647 | 2.335216 | 2.471658 | 2.036894 | 2.112312 | 1.476241 | 1.506705 | 1.419569 | 2.071858 | 1.440195 |
| Q99020 | Q99020 | ROAA_MO | Hnrnpab | Heterogeneous nuclear ribonucleoprotein A/B | | | | 0.179469 | 0.252252 | 0.508741 | 0.227719 | 0.187478 | 0.632097 | 0.487128 | 0.416461 | 1.291204 | 0.184847 | 1.069817 | -0.32422 | 0.064707 | 0.803385 | 0.929217 | 0.598822 | 0.151339 | -0.54119 | -0.09329 | 0.766181 | 0.133499 |
| Q9Z204 | Q9Z204 | HNRPC_M | Hnrnpc | Heterogeneous nuclear ribonucleoproteins C1/C2 | | | | 0.785889 | 1.389385 | 2.15695 | 0.271297 | 0.559107 | 0.741923 | 0.583102 | 1.395657 | 1.550561 | 0.970066 | 1.087327 | -0.07139 | 1.401392 | 1.517187 | -0.00381 | 0.280781 | -1.00216 | 0.265992 | -0.0542 | -1.12044 | -0.15749 |
| Q60668 | Q60668 | HNRPD_M | Hnrnpd | Heterogeneous nuclear ribonucleoprotein D0 | | | | 0.115984 | -0.15594 | -0.34095 | 0.01231 | -0.00383 | -0.03954 | 0.45192 | 0.455567 | 1.199219 | 2.308405 | 3.144188 | 1.584193 | 1.775142 | 2.481558 | 2.985077 | 2.696374 | 3.068098 | 1.699483 | 1.667506 | 2.562234 | 1.993268 |
| Q9Z130 | Q9Z130 | HNRDL_M | Hnrnpdl | Heterogeneous nuclear ribonucleoprotein D-like | | | | 0.030099 | 0.026558 | 0.094906 | 0.07405 | -0.05856 | -0.22447 | 0.464566 | 0.383528 | 1.232094 | 0.529909 | 0.952219 | 0.133523 | 0.557511 | 0.877632 | 0.994046 | 0.530879 | 0.898133 | 0.55611 | 0.252042 | 0.858102 | 0.246519 |
| Q9Z2X1 | Q9Z2X1 | HNRPF_M | Hnrnpf | Heterogeneous nuclear ribonucleoprotein F | | | | 0.236744 | 0.424698 | 0.65493 | 0.369506 | 0.484452 | 0.988595 | 0.061827 | -0.06833 | -0.18933 | -0.2718 | 0.890408 | 0.694488 | 1.091183 | 0.981317 | 0.447978 | 0.172398 | -0.40318 | -0.03972 | 0.856489 | 0.539775 | 1.026185 |
| O35737 | Q9Z2X1;P | HNRH1_M | Hnrnph1 | Heterogeneous nuclear ribonucleoprotein H | + | | | 1.134781 | 1.487588 | 3.486829 | 0.20697 | 0.147244 | 0.579353 | 0.843775 | 0.126028 | 2.348893 | 2.152118 | 3.003465 | 2.460001 | 2.607924 | 3.015139 | 2.923202 | 1.061645 | 1.118763 | 2.143566 | 2.629871 | 2.815471 | 2.870813 |
| P70333 | P70333 | HNRH2_M | Hnrnph2 | Heterogeneous nuclear ribonucleoprotein H2 | | | | 0.670467 | 0.952056 | 1.800601 | 0.038987 | -0.07783 | -0.12187 | 0.065007 | 0.054552 | 0.198531 | 1.135446 | 1.378997 | 1.889159 | 2.038779 | 2.163043 | 2.425451 | -0.20936 | 0.819695 | 1.407592 | 2.676004 | 1.998266 | 2.481124 |
| Q60932 | Q60932 | VDAC1_M | Vdac1 | Voltage-dependent anion-selective channel protein 1 | + | | | 1.546968 | 0.915457 | 5.808763 | 0.725188 | 0.548301 | 1.965479 | 0.724805 | 0.499051 | 1.964299 | 6.34685 | 6.21599 | 6.031314 | 6.300531 | 6.827349 | 6.321439 | 5.222588 | 5.509338 | 5.373282 | 5.861963 | 6.099021 | 6.051665 |
| P61979 | P61979 | HNRPK_M | Hnrnpk | Heterogeneous nuclear ribonucleoprotein K | + | | | 1.030806 | 0.825437 | 3.042948 | 0.960054 | 0.461385 | 2.765909 | 0.03242 | 0.019378 | 0.10199 | 1.545108 | 2.059976 | 2.392998 | 2.475533 | 2.513863 | 2.134866 | 1.06261 | 0.891601 | 1.811254 | 2.134507 | 2.291153 | 2.318819 |
| Q8R081 | Q8R081 | HNRPL_M | Hnrnpl | Heterogeneous nuclear ribonucleoprotein L | | | | 0.553155 | 1.271547 | 1.468105 | 0.405272 | 0.406187 | 1.079334 | 0.454912 | 1.005841 | 1.206982 | -0.07579 | -0.17822 | -0.54684 | 0.146535 | 1.02685 | -0.63706 | -0.53395 | -2.26315 | -0.45994 | -0.75274 | -0.85917 | -0.76273 |
| Q921F4 | Q921F4 | HNRLL_M | Hnrnpll | Heterogeneous nuclear ribonucleoprotein L-like | | | | 0.684973 | 1.650338 | 1.843639 | 0.660006 | -0.33125 | -1.76985 | 0.5273 | -0.14949 | -1.39808 | -2.84132 | -1.05104 | -1.80348 | -1.8477 | -0.96869 | -1.17643 | -3.59194 | -3.6011 | -1.6802 | -1.30849 | -0.8977 | -0.94844 |
| Q9D0E1 | Q9D0E1 | HNRPM_M | Hnrnpm | Heterogeneous nuclear ribonucleoprotein M | | | | 0.452904 | 0.564939 | 1.201771 | 0.034112 | -0.02733 | -0.10714 | 0.304516 | 0.352378 | 0.825234 | 0.662098 | 0.755484 | 1.258239 | 1.492443 | 1.731645 | 1.047569 | 0.611616 | -0.32391 | 1.17606 | 1.629279 | 0.781609 | 1.29285 |
| Q8VHM5 | Q8VHM5 | Q8VHM5_I | Hnrnpr | Heterogeneous nuclear ribonucleoprotein R | + | | | 1.117022 | 0.518696 | 3.407689 | 0.132038 | 0.137883 | 0.384187 | 0.407652 | 0.496405 | 1.085402 | -0.44718 | -0.1603 | -0.20948 | 0.4578 | 0.382115 | -0.35774 | -0.87337 | -0.7715 | -0.14597 | 0.118524 | -0.75314 | -0.21529 |
| Q3UMU9 | Q3UMU9;( | HDGR2_M | Hdgfl2 | Hepatoma-derived growth factor-related protein 2 | + | | + | 1.327037 | 0.907929 | 4.443679 | 0.157025 | -0.08952 | -0.45032 | 1.001428 | 0.491408 | 2.925606 | 2.165581 | 2.574078 | 2.010029 | 2.235404 | 2.334437 | 2.002573 | 1.456522 | 1.467279 | 2.375994 | 2.048474 | 1.703167 | 1.651028 |
| Q8VDM6 | Q8VDM6 | HNRL1_M | Hnrnpul1 | Heterogeneous nuclear ribonucleoprotein U-like protein 1 | | | | 0.569641 | 0.578192 | 1.51331 | 0.539195 | 0.403551 | 1.430169 | 0.460119 | 0.249629 | 1.220513 | 0.335201 | 0.92674 | 1.024465 | 1.588731 | 0.991978 | 0.583118 | 0.294643 | -0.18909 | 0.898442 | 0.907651 | 0.53158 | 0.544257 |
| Q00PI9 | Q00PI9 | HNRL2_M | Hnrnpul2 | Heterogeneous nuclear ribonucleoprotein U-like protein 2 | | | | 0.907079 | 1.051139 | 2.570374 | 0.367057 | 0.204645 | 0.982408 | 0.319958 | 0.529568 | 0.863954 | 1.76115 | 2.317709 | 1.706857 | 2.123298 | 2.700282 | 1.539445 | 1.28795 | 0.688632 | 1.704321 | 1.716543 | 1.393233 | 1.787357 |
| Q9Z2Y3 | Q9Z2Y3 | HOME1_M | Homer1 | Homer protein homolog 1 | | | | 0.510954 | 0.288779 | 1.354322 | 0.788165 | -0.43379 | -2.16432 | 0.225998 | -0.15357 | -0.62774 | -0.64142 | -0.26587 | 2.448995 | 2.170208 | 2.509744 | 2.170819 | -0.64139 | -0.84345 | 2.599376 | 2.887414 | 2.317406 | 2.670303 |
| Q9QWW1 | Q9QWW1 | HOME2_M | Homer2 | Homer protein homolog 2 | | + | | 0.321545 | -0.61123 | -0.86793 | 1.787453 | -1.71632 | -7.73324 | 0.004073 | -0.00138 | -0.0132 | 0.88348 | 0.833361 | -0.49093 | -0.82809 | 1.257764 | 1.143036 | 2.173431 | 0.765862 | 1.201162 | 0.912458 | 1.11412 | 1.289447 |
| Q99JP6 | Q99JP6 | HOME3_M | Homer3 | Homer protein homolog 3 | | | | 0.82163 | 2.325217 | 2.274324 | 0.089217 | 0.296036 | 0.267271 | 0.455799 | 0.348902 | 1.209283 | -1.05443 | 0.471578 | -2.4848 | -3.11747 | 0.484304 | 0.12505 | -1.93614 | -3.29714 | -4.15866 | -2.03568 | -0.27001 | 0.181558 |
| Q8BIL5 | Q8BIL5 | HOOK1_M | Hook1 | Protein Hook homolog 1 | | | | NaN | 0 | NaN | 0.089757 | 0.095888 | 0.26878 | 0.163714 | -0.08944 | -0.46782 | -3.05518 | -3.54064 | -2.97412 | -2.93354 | -3.06942 | -3.43289 | -3.67917 | -3.6049 | -3.40589 | -2.69354 | -3.10238 | -3.22106 |
| Q8BUK6 | Q8BUK6 | HOOK3_M | Hook3 | Protein Hook homolog 3 | | | | NaN | 0 | NaN | 0.320573 | 0.686829 | 0.865498 | 0.643364 | -0.44893 | -1.7214 | -2.48943 | -3.58399 | -1.81079 | -2.25826 | -2.80885 | -2.51162 | -3.581 | -3.5695 | -3.48273 | -1.95998 | -2.42561 | -1.99699 |
| Q3TEA8 | Q3TEA8 | HP1B3_M | Hp1bp3 | Heterochromatin protein 1-binding protein 3 | | | | 0.769212 | 1.186422 | 2.10339 | 0.536167 | 0.382344 | 1.421978 | 0.581405 | 0.571685 | 1.545849 | 0.974892 | 1.504799 | 1.246745 | 1.782003 | 1.722664 | 0.98336 | 0.551374 | -0.44453 | 1.157949 | 1.106112 | 0.792455 | 0.770199 |
| O35625 | O35625 | AXIN1_MO | Axin1 | Axin-1 | + | | | 1.635001 | 0.907008 | 6.454289 | 0.85824 | 0.227354 | 2.398394 | 0.122457 | -0.03891 | -0.35846 | 1.480891 | 1.553298 | 1.980761 | 2.151876 | 1.50708 | 1.490548 | 0.745871 | 0.474302 | 1.879779 | 1.79815 | 1.429495 | 1.64595 |
| P62748 | P62748 | HPCL1_M | Hpcal1 | Hippocalcin-like protein 1 | | | | 0.665092 | 0.71669 | 1.78477 | 0.17917 | -0.13676 | -0.50797 | 0.213493 | -0.12081 | -0.59598 | 2.008562 | 2.802221 | 1.27354 | 1.683693 | 1.344376 | 1.430737 | 1.627253 | 1.750149 | 1.440938 | 1.789817 | 1.706429 | 1.310308 |
| Q8BGZ1 | Q8BGZ1 | HPCL4_M | Hpcal4 | Hippocalcin-like protein 4 | | | + | 0.10591 | -0.52519 | -0.31348 | 0.225672 | 0.261943 | 0.62691 | 1.079444 | 0.760156 | 3.244841 | -0.2732 | -3.58378 | 2.176407 | 1.49409 | 2.93701 | 2.919495 | -1.66181 | -1.14479 | 1.332073 | 1.814539 | 1.933994 | 2.402199 |
| P00493 | P00493 | HPRT_MOI | Hprt1 | Hypoxanthine-guanine phosphoribosyltransferase | | | + | 0.272243 | 0.174223 | 0.744299 | 0.194615 | -0.11624 | -0.54774 | 1.004124 | -0.36354 | -2.93623 | 1.629302 | 1.542571 | 1.634871 | 1.576767 | 1.386635 | 1.579026 | 1.181689 | 1.641737 | 1.511837 | 1.93229 | 1.924311 | 1.768421 |
| Q61411 | Q61411 | RASH_MOI | Hras | GTPase HRas | | | | 0.515522 | -0.61141 | -1.36651 | 0.284898 | 0.239261 | 0.776052 | 0.228075 | 0.111279 | 0.632999 | 1.391341 | 0.844618 | 2.38444 | 1.850156 | 1.808337 | 2.091398 | 1.375184 | 2.083598 | 2.031945 | 1.724128 | 1.942865 | 1.734311 |
| Q9CQZ1 | Q9CQZ1 | HSBP1_M | Hsbp1 | Heat shock factor-binding protein 1 | + | | | 1.041384 | 1.217724 | 3.086033 | 0.505769 | 0.743086 | 1.340522 | 0.390801 | -0.98783 | -1.04252 | 0.411817 | -0.11026 | 3.9193 | 4.136207 | 0.989909 | 2.225859 | -1.36285 | -0.77103 | 2.741054 | 3.82828 | 1.877419 | 3.314008 |
| O08756 | O08756 | HCD2_MO | Hsd17b10 | 3-hydroxyacyl-CoA dehydrogenase type-2 | + | | | 1.266391 | -1.18052 | -4.12076 | 0.016344 | 0.015988 | 0.052267 | 0.27237 | 0.473746 | 0.744618 | -1.52498 | -0.984 | -0.02449 | -0.63051 | 0.14782 | 1.008201 | 0.020392 | -0.16835 | -0.38539 | -0.30159 | 0.573009 | -0.36448 |
| Q9EQ06 | Q9EQ06 | DHB11_M | Hsd17b11 | Estradiol 17-beta-dehydrogenase 11 | | | | 0.184792 | 0.185035 | 0.522484 | 0.333826 | -0.21197 | -0.89876 | 0.144058 | -0.21953 | -0.41616 | -0.93904 | -1.48609 | -1.72916 | -2.17425 | -1.9112 | -1.73139 | -1.17265 | -1.62255 | -1.8178 | -1.66167 | -1.08196 | -2.12157 |
| O70503 | O70503 | DHB12_M | Hsd17b12 | Very-long-chain 3-oxoacyl-CoA reductase | + | | | 1.261879 | 0.346501 | 4.097559 | 0.845843 | 0.224154 | 2.355929 | 0.290151 | 0.168653 | 0.789224 | 2.839525 | 2.834531 | 2.017448 | 1.830276 | 2.176708 | 1.945422 | 2.575053 | 2.406002 | 1.682559 | 1.716857 | 1.712711 | 2.072112 |
| P51660 | P51660 | DHB4_MO | Hsd17b4 | Peroxisomal multifunctional enzyme type 2 | | | | 0.038173 | -0.05312 | -0.11942 | 0.189626 | -0.16582 | -0.53493 | 0.141094 | 0.190136 | 0.408308 | 0.0948 | -0.01284 | -0.38482 | -1.00417 | -0.05249 | -0.73656 | 0.535605 | -0.34742 | -0.5151 | -0.54227 | -0.90067 | -0.26865 |
| P50171 | P50171 | DHB8_MO | Hsd17b8 | (3R)-3-hydroxyacyl-CoA dehydrogenase | + | | | 1.039541 | -2.1193 | -3.07849 | 0.397134 | -0.409 | -1.05861 | 0.313972 | -0.4004 | -0.84894 | -1.94171 | -3.31533 | -0.99818 | -0.82559 | -1.0992 | -0.16245 | -0.55635 | -0.46209 | -0.12629 | -0.87948 | -0.17503 | -0.28582 |
| Q8BTX9 | Q8BTX9 | HSDL1_M | Hsdl1 | Inactive hydroxysteroid dehydrogenase-like protein 1 | | | | 0.275131 | 0.170099 | 0.751549 | 0.318659 | 0.52438 | 0.860696 | 0.117614 | -0.0371 | -0.34537 | 0.585829 | 0.146097 | 0.281647 | -0.23811 | 0.114413 | 0.314744 | 0.142155 | 0.249573 | 0.048434 | -1.05366 | 0.290451 | 0.212898 |
| O89084 | Q01063;O | PDE4A_M | Pde4a | cAMP-specific 3',5'-cyclic phosphodiesterase 4A | + | | | 2.214262 | 0.902394 | 12.739 | 0.283025 | 0.30635 | 0.771354 | 0.07238 | -0.14348 | -0.21971 | 1.958623 | 1.837451 | 4.013517 | 4.701665 | 3.034219 | 4.254452 | 0.958939 | 1.032348 | 3.852879 | 4.249604 | 4.020637 | 3.554985 |
| Q9CXW4 | Q9CXW4 | RL11_MOL | Rpl11 | Large ribosomal subunit protein uL5 | + | | | 1.798185 | 0.899536 | 7.831765 | 0.480417 | -0.15616 | -1.27356 | 0.223696 | -0.08514 | -0.6219 | 2.239395 | 2.410679 | 2.034031 | 2.278654 | 2.251502 | 2.420609 | 1.502036 | 1.348966 | 2.321199 | 2.303813 | 2.313522 | 2.528872 |
| P08113 | P08113 | ENPL_MOI | Hsp90b1 | Endoplasmin | | | | 0.449618 | -0.30055 | -1.19325 | 0.133589 | 0.28451 | 0.388332 | 0.47393 | 0.906259 | 1.256552 | 0.092996 | 0.368422 | 0.986024 | -0.47525 | 1.872946 | 0.434278 | 0.742145 | 0.320366 | 0.025081 | -0.08333 | 0.299579 | 0.195127 |
| Q8K0U4 | Q8K0U4;Q | HS12A_M | Hspa12a | Heat shock 70 kDa protein 12A | | | + | 0.060498 | 0.044718 | 0.185471 | 0.076336 | 0.038579 | 0.230977 | 1.012796 | -0.27618 | -2.97061 | 1.604112 | 1.527344 | 2.004454 | 2.085743 | 1.995347 | 1.811825 | 1.759041 | 1.282979 | 1.844513 | 2.168525 | 2.164811 | 2.194731 |
| Q9CZJ2 | Q9CZJ2 | HS12B_M | Hspa12b | Heat shock 70 kDa protein 12B | | | | 0.575641 | 0.252029 | 1.529876 | 0.361802 | 0.150311 | 0.96915 | 0.046321 | -0.06134 | -0.14381 | -0.47141 | -0.80037 | -0.28073 | -0.31871 | -0.54711 | -0.85497 | -0.89721 | -0.87863 | -0.2961 | -0.60396 | -1.03752 | -0.24187 |
| P17879;Q | Q61696;P | HS71A_M | Hspa1a;Hspa1b | Heat shock 70 kDa protein 1B | + | | | 1.004187 | 1.210738 | 2.936483 | 0.81815 | 0.870457 | 2.262737 | 0.115731 | -0.1605 | -0.34027 | 0.650822 | 0.630264 | 2.385484 | 2.979791 | 1.464114 | 2.27111 | -0.15801 | -0.98238 | 1.567869 | 2.056491 | 1.783812 | 2.272416 |
| P17156 | P17156 | HSP72_M | Hspa2 | Heat shock-related 70 kDa protein 2 | + | | | 1.275953 | 0.788215 | 4.170308 | 0.082383 | 0.059027 | 0.248089 | 0.867885 | -0.69714 | -2.43176 | 1.694285 | 1.510742 | 3.239228 | 3.660362 | 2.933877 | 3.440893 | 0.979529 | 0.649066 | 3.279992 | 3.501544 | 3.750658 | 4.018383 |
| Q61316 | Q61316 | HSP74_M | Hspa4 | Heat shock 70 kDa protein 4 | | | | 0.912263 | -0.22113 | -2.58909 | 0.94005 | 0.682908 | 2.690925 | 0.074011 | -0.03368 | -0.22436 | 0.569523 | 0.51384 | 2.375655 | 2.40863 | 1.21323 | 1.507535 | 0.843561 | 0.682071 | 1.455988 | 1.96248 | 1.364367 | 1.42376 |
| Q8K0T0 | Q8K0T0 | RTN1_MOI | Rtn1 | Reticulon-1 | + | | | 2.356783 | 0.891008 | 15.02988 | 0.210958 | 0.063872 | 0.589523 | 0.095087 | 0.097265 | 0.283621 | 3.533732 | 3.415215 | 3.536138 | 3.446808 | 3.876533 | 3.227911 | 2.581779 | 2.585152 | 3.32889 | 3.526311 | 3.343465 | 3.566449 |
| P47941 | P47941 | CRKL_MOI | Crkl | Crk-like protein | + | | | 1.827345 | 0.886643 | 8.105554 | 0.18903 | 0.066326 | 0.533397 | 0.264688 | 0.153845 | 0.725324 | 3.073721 | 2.923104 | 1.88535 | 2.029791 | 2.459432 | 2.55646 | 2.032434 | 2.191105 | 1.79002 | 1.992468 | 2.147619 | 2.560583 |
| Q791V5 | Q791V5 | MTCH2_M | Mtch2 | Mitochondrial carrier homolog 2 | + | | | 1.776266 | 0.88448 | 7.631801 | 0.680665 | -0.42502 | -1.83081 | 0.354093 | 0.2404 | 0.949722 | 4.454089 | 4.521637 | 3.096691 | 3.260176 | 4.48308 | 4.076904 | 3.714247 | 3.49252 | 3.820735 | 3.386172 | 3.888501 | 4.190682 |
| Q68FH0 | Q68FH0 | PKP4_MOI | Pkp4 | Plakophilin-4 | + | + | | 1.968559 | 0.883917 | 9.566557 | 1.299752 | 0.517997 | 4.295825 | 0.207531 | -0.11316 | -0.58079 | -0.57711 | -0.55806 | 0.675943 | 0.595927 | 0.771351 | 0.421145 | -1.3596 | -1.5434 | 0.231689 | 0.004187 | 0.623949 | 0.794874 |
| Q9Z210 | Q9Z210 | PX11B_MC | Pex11b | Peroxisomal membrane protein 11B | + | + | | 1.852507 | 0.882912 | 8.349128 | 1.966599 | 0.846242 | 9.544643 | 0.085723 | 0.142268 | 0.257486 | 0.520558 | 0.311101 | -0.33669 | -0.4905 | 0.257058 | -0.42576 | -0.48174 | -0.45242 | -1.30396 | -1.21571 | -0.66105 | 0.207814 |
| Q6ZWV3 | Q6ZWV3 | RL10_MOL | Rpl10 | Large ribosomal subunit protein uL16 | + | | | 1.707877 | 0.875778 | 7.03854 | 0.547985 | 0.067215 | 1.454018 | 0.085579 | -0.10234 | -0.25708 | 1.504375 | 1.312625 | 2.056758 | 2.145919 | 1.79601 | 1.85047 | 0.612031 | 0.453413 | 2.046353 | 2.021894 | 1.528429 | 2.322729 |
| P63038 | P63038 | CH60_MO | Hspd1 | 60 kDa heat shock protein, mitochondrial | | + | | 0.133584 | 0.096932 | 0.388317 | 1.449286 | 0.555623 | 5.162035 | 0.215494 | 0.187767 | 0.601069 | 2.211595 | 2.517249 | 2.857698 | 2.994692 | 2.871611 | 2.246884 | 2.464858 | 2.070122 | 2.287543 | 2.4536 | 2.367572 | 2.375389 |
| Q64433 | Q64433;Q | CH10_MO | Hspe1 | 10 kDa heat shock protein, mitochondrial | | | | 0.012329 | -0.01802 | -0.0396 | 0.257099 | 0.818549 | 0.706241 | 0.102614 | -0.24702 | -0.30442 | 0.82395 | 1.230278 | 2.227737 | 0.073353 | 1.051579 | -0.40759 | 0.637917 | 1.452352 | 0.759768 | -0.09578 | 0.924152 | 0.213874 |
| Q8BH57 | Q8BH57 | WDR48_M | Wdr48 | WD repeat-containing protein 48 | + | | | 1.457283 | 0.872846 | 5.212418 | 0.053894 | -0.0307 | -0.16618 | 0.077161 | -0.05464 | -0.23332 | -0.30844 | -0.43347 | 0.942101 | 1.256389 | 0.800447 | 0.4969 | -1.08845 | -1.39915 | 1.227078 | 1.032813 | 0.52498 | 0.881642 |
| Q8BGC0 | Q8BGC0 | HTSF1_M | Htatsf1 | HIV Tat-specific factor 1 homolog | | | | 0.047632 | 0.103429 | 0.147703 | 0.004971 | -0.00734 | -0.0161 | 0.250833 | 0.322805 | 0.69047 | -2.28633 | -3.6322 | -2.48823 | -1.9268 | -1.89783 | -2.75307 | -3.25636 | -2.86903 | -2.55953 | -1.84083 | -2.83723 | -2.45928 |
| P42859 | P42859 | HD_MOUS | Htt | Huntingtin | | | | 0.880724 | 0.438421 | 2.476622 | 0.184135 | -0.04422 | -0.52079 | 0.515888 | -0.45218 | -1.36749 | 0.292074 | 0.485082 | 1.220154 | 1.353511 | 0.214761 | 0.723141 | 0.098563 | -0.19825 | 1.278477 | 1.383633 | 0.709646 | 1.132611 |
| Q7TMY8 | Q7TMY8 | HUWE1_M | Huwe1 | E3 ubiquitin-protein ligase HUWE1 | | | | 0.236446 | 0.55691 | 0.654177 | 0.297358 | -0.10726 | -0.80729 | 0.377277 | -0.13055 | -1.00824 | -0.61654 | -0.7704 | -0.04742 | -0.2318 | -0.30309 | -0.51108 | -0.40255 | -2.09821 | -0.12803 | 0.063322 | -0.35368 | -0.1994 |
| Q8C729 | Q8C729 | HYCC2_M | Hycc2 | Hyccin 2 | | | | 0.097828 | 0.057122 | 0.291217 | 0.242243 | -0.24126 | -0.66881 | 0.078442 | -0.0721 | -0.23695 | 2.143397 | 2.511075 | 3.238395 | 3.95977 | 3.304243 | 3.785516 | 2.338509 | 2.201719 | 3.845471 | 3.835208 | 3.803218 | 3.430744 |
| Q9JKR6 | Q9JKR6 | HYOU1_M | Hyou1 | Hypoxia up-regulated protein 1 | | | | 0.12571 | -0.44001 | -0.36722 | 0.341628 | 0.997801 | 0.918361 | 0.125796 | 0.212505 | 0.367455 | -3.00658 | -2.21515 | -1.50146 | -1.45627 | -0.52559 | -1.67381 | -1.03986 | -3.30184 | -3.56294 | -1.3904 | -1.38182 | -1.24259 |
| Q9DB29 | Q9DB29 | IAH1_MOL | Iah1 | Isoamyl acetate-hydrolyzing esterase 1 homolog | | + | + | 0.20501 | 0.158193 | 0.57435 | 1.134685 | -0.55381 | -3.4864 | 1.366602 | -0.42243 | -4.66589 | 0.310055 | 0.431241 | -0.74846 | -1.05997 | -0.31293 | -0.13801 | 0.481137 | -0.05623 | -0.38161 | -0.3192 | 0.173573 | 0.220349 |
| Q8BU30 | Q8BU30 | SYIC_MOL | Iars1 | Isoleucine--tRNA ligase, cytoplasmic | | | | 0.537274 | -0.1359 | -1.42497 | 0.131734 | -0.05879 | -0.38337 | 0.293848 | 0.396077 | 0.798494 | -0.04262 | 0.133316 | 0.401666 | 0.223902 | 0.833037 | -0.04826 | 0.218092 | 0.144408 | 0.496527 | 0.246616 | -0.23145 | 0.224072 |
| Q8BIJ6 | Q8BIJ6 | SYIM_MOL | Iars2 | Isoleucine--tRNA ligase, mitochondrial | | | | 0.583014 | -1.678 | -1.55032 | 0.157613 | -0.15534 | -0.45186 | 0.231835 | 0.163731 | 0.642517 | -0.68347 | -2.70646 | -0.41646 | -1.04834 | 0.280645 | 0.204821 | 0.368246 | -0.40218 | -0.71259 | -0.44152 | -0.17299 | 0.330994 |
| Q8CAK1 | Q8CAK1 | CAF17_M | Iba57 | Putative transferase CAF17 homolog, mitochondrial | | | | 0.447021 | -1.21331 | -1.18653 | 0.25277 | -0.11091 | -0.69535 | 0.767035 | -0.57699 | -2.09645 | -1.94563 | -3.49783 | -2.05923 | -1.7474 | -2.14348 | -1.61056 | -0.84259 | -2.17424 | -1.75873 | -1.82607 | -1.36892 | -1.23114 |
| P97411 | P97411;Q | ICA69_MO | Ica1 | Islet cell autoantigen 1 | | | | 0.457128 | 0.981566 | 1.212736 | 0.519154 | 0.27476 | 1.376226 | 0.212249 | -0.29319 | -0.59281 | -0.75382 | -1.15673 | -0.517 | -0.37473 | -0.55865 | -1.26585 | -1.15293 | -2.72075 | -0.90717 | -0.53408 | -0.96486 | -0.27326 |
| Q60625 | Q60625 | ICAM5_MC | Icam5 | Intercellular adhesion molecule 5 | | | | 0.380934 | -0.8414 | -1.0175 | 0.683762 | 0.552675 | 1.840028 | 0.411321 | 0.302401 | 1.09477 | -3.60138 | -3.47562 | 1.549761 | 1.559772 | 1.79233 | 1.368609 | -3.52163 | -1.87256 | 0.701771 | 1.302413 | 1.100826 | 1.45531 |
| Q9JHR7 | Q9JHR7 | IDE_MOUS | Ide | Insulin-degrading enzyme | | | | 0.770639 | 0.511045 | 2.107945 | 0.396031 | -1.24654 | -1.05581 | 0.620872 | -0.32998 | -1.65682 | 0.5459 | 0.117226 | -0.32206 | -0.61168 | -0.65148 | -0.27314 | -0.29278 | -0.06619 | -0.39207 | 1.951419 | -0.19464 | -0.07001 |
| O88844 | O88844 | IDHC_MOI | Idh1 | Isocitrate dehydrogenase [NADP] cytoplasmic | + | | | 1.13789 | -1.07219 | -3.50083 | 0.3996 | -0.78877 | -1.06488 | 0.128799 | 0.218148 | 0.375515 | -0.77361 | -0.50368 | -0.37589 | -1.8566 | -0.61208 | 0.252792 | 0.708467 | 0.158616 | -0.3505 | -0.30445 | -0.00987 | -0.78571 |
| Q9R112 | Q9R112 | SQOR_MO | Sqor | Sulfide:quinone oxidoreductase, mitochondrial | + | | | 2.263855 | 0.872613 | 13.49422 | 0.622007 | 0.676437 | 1.660057 | 0.011762 | -0.0298 | -0.0378 | -0.6161 | -0.56196 | -0.59918 | 0.212895 | -0.36726 | -1.05605 | -1.40291 | -1.52037 | -0.83535 | -0.90381 | -1.39093 | 0.02722 |
| Q9D6R2 | Q9D6R2 | IDH3A_MC | Idh3a | Isocitrate dehydrogenase [NAD] subunit alpha, mitochondrial | | | | 0.979883 | -0.89151 | -2.84165 | 0.460466 | -0.63585 | -1.22142 | 0.157132 | -0.10489 | -0.4506 | 1.849314 | 2.410264 | 2.470986 | 1.45761 | 2.454705 | 2.896163 | 3.161875 | 2.88073 | 2.480671 | 2.719621 | 2.706421 | 2.854222 |
| Q91VA7 | Q91VA7 | Q91VA7_I | Idh3b | Isocitrate dehydrogenase [NAD] subunit, mitochondrial | | | + | 0.40541 | 0.230347 | 1.079684 | 0.07113 | -0.03932 | -0.21613 | 1.196374 | 0.330333 | 3.772744 | 3.067757 | 3.147102 | 3.169797 | 2.810676 | 3.401516 | 3.559329 | 3.086708 | 2.667456 | 3.058897 | 3.000223 | 3.188039 | 3.112141 |
| P03888 | P03888 | NU1M_MC | Mtnd1 | NADH-ubiquinone oxidoreductase chain 1 | + | | | 1.36247 | 0.868063 | 4.642244 | 0.436302 | -0.90818 | -1.15885 | 0.629839 | 0.598016 | 1.682446 | 2.164828 | 2.320714 | 0.414349 | -1.00997 | 1.782564 | 2.1761 | 1.544682 | 1.204735 | 0.283259 | 0.937494 | 1.677328 | 1.085304 |
| Q8BXL9 | Q8R2V2;Q | IFFO1_MO | Iffo1 | Non-homologous end joining factor IFFO1 | | | | NaN | 0 | NaN | 0.667809 | 0.734513 | 1.792763 | 0.083006 | -0.2107 | -0.24985 | -3.59991 | -3.06587 | -1.36573 | -1.21075 | -3.08912 | -2.22963 | -3.22166 | -3.43407 | -2.42507 | -1.62044 | -3.17427 | -1.72308 |
| Q3UDP0 | Q3UDP0 | WDR41_M | Wdr41 | WD repeat-containing protein 41 | + | | | 1.775179 | 0.853821 | 7.622004 | 0.009367 | 0.015048 | 0.030184 | 0.118759 | 0.175187 | 0.348473 | -1.10492 | -0.91713 | -1.19085 | -0.95999 | -0.45035 | -0.89041 | -1.80376 | -1.92593 | -0.60546 | -1.57548 | -1.29759 | -0.39355 |
| P01867 | P01867 | IGG2B_MC | Ighg2b | Immunoglobulin heavy constant gamma 2B | | | | 0.372544 | 1.693526 | 0.99627 | 0.255719 | 1.112311 | 0.70277 | 0.189169 | 0.493551 | 0.533756 | 1.09072 | -1.11103 | -0.07209 | -1.84162 | 0.509403 | -1.26443 | -2.99891 | -0.40845 | -3.38153 | -0.75681 | -1.13261 | -0.60952 |
| P01872 | P01872 | IGHM_MO | Ighm | Immunoglobulin heavy constant mu | + | | | 1.182468 | 1.399782 | 3.706596 | 0.001283 | 0.001202 | 0.00417 | 0.479455 | 0.503619 | 1.271031 | 1.541352 | 1.006766 | -0.27508 | -0.02814 | 0.15486 | -0.35092 | 0.141056 | -0.3925 | 0.107613 | -0.41323 | -0.29662 | -0.90668 |
| A0A075B5 | P01756;P | A0A075B5 | Ighv1-31;Ighv1-42 | Immunoglobulin heavy variable 1-31 | | | | 0.461793 | 0.592145 | 1.22487 | 0.038164 | -0.10908 | -0.1194 | NaN | 0 | NaN | -2.96443 | -2.29646 | -4.372 | -3.09837 | -3.4038 | -4.12181 | -2.87307 | -3.57211 | -4.28122 | -2.97097 | -2.96651 | -3.57415 |
| Q9JIS5 | Q9JIS5 | SV2A_MOI | Sv2a | Synaptic vesicle glycoprotein 2A | + | | | 1.757395 | 0.852604 | 7.46353 | 0.752308 | 0.320769 | 2.049852 | 0.763193 | 0.232298 | 2.08424 | 2.999974 | 2.937148 | 3.416588 | 3.285104 | 3.40502 | 3.287011 | 2.225789 | 2.006125 | 3.172081 | 2.888073 | 3.208272 | 3.019163 |
| P11983 | P11983 | TCPA_MOI | Tcp1 | T-complex protein 1 subunit alpha | + | | | 1.886231 | 0.847337 | 8.686607 | 0.510542 | 0.222642 | 1.353225 | 0.375902 | 0.315384 | 1.00476 | 2.145439 | 2.022455 | 2.735677 | 2.443379 | 2.905587 | 2.321578 | 1.312331 | 1.160887 | 2.291325 | 2.442448 | 2.183047 | 2.41335 |
| P01635 | P01635;A | KV5A3_MC | Igkv12-41 | Immunoglobulin kappa chain variable 12-41 (Fragment) | | | | 0.319445 | 0.814263 | 0.862668 | 0.97444 | -0.63319 | -2.82072 | 0.14023 | 0.193574 | 0.406014 | -1.41717 | -3.23138 | -3.36644 | -3.71201 | -2.29891 | -3.25222 | -2.87761 | -3.39948 | -2.76273 | -3.04933 | -2.97959 | -2.95869 |
| P0C673 | P0C673 | IGS11_MO | Igsf11 | Immunoglobulin superfamily member 11 | | | | 0.055598 | 0.264809 | 0.171177 | 0.271709 | 0.64857 | 0.742959 | 0.459643 | 0.564529 | 1.219275 | -1.11126 | -3.00411 | -0.99139 | -0.18368 | -0.46053 | -1.31688 | -1.09879 | -3.54619 | -2.01003 | -0.46219 | -1.27706 | -1.62942 |
| Q7TNR6 | Q7TNR6 | IGS21_MO | Igsf21 | Immunoglobulin superfamily member 21 | | | | NaN | 0 | NaN | 0.62624 | -0.73632 | -1.67214 | 0.833359 | -0.88236 | -2.31364 | -2.60087 | -2.59148 | -3.58268 | -3.33779 | -2.95433 | -3.62898 | -2.99598 | -2.73549 | -3.14689 | -2.30093 | -2.23136 | -2.58722 |
| Q8R366 | Q8R366 | IGSF8_MO | Igsf8 | Immunoglobulin superfamily member 8 | | | | 0.679664 | -0.59465 | -1.82783 | 0.086961 | -0.14676 | -0.26096 | 0.495745 | 0.52662 | 1.313944 | 0.328941 | 0.915344 | 1.067257 | 0.123818 | 1.092652 | 0.859282 | 1.35777 | 1.07582 | 0.436118 | 1.04847 | 0.832779 | 0.065915 |
| O09167 | O09167 | RL21_MOL | Rpl21 | Large ribosomal subunit protein eL21 | + | + | | 1.325536 | 0.846794 | 4.435433 | 1.349217 | 0.422268 | 4.567091 | 0.389283 | 0.267368 | 1.038663 | 0.609153 | 0.901271 | 1.328526 | 1.512751 | 0.694688 | 0.916831 | 0.031363 | -0.21453 | 1.006368 | 0.990372 | 0.306172 | 0.770611 |
| O54824 | O54824 | IL16_MOU | Il16 | Pro-interleukin-16 | | | | NaN | 0 | NaN | 0.210518 | -0.12833 | -0.5884 | 0.885284 | -1.15938 | -2.49268 | -3.84287 | -3.31589 | -3.32317 | -3.72014 | -2.49192 | -1.78416 | -3.29573 | -3.15818 | -3.30292 | -3.48373 | -1.28049 | -0.67684 |
| Q61730 | Q61730 | IL1AP_MO | Il1rap | Interleukin-1 receptor accessory protein | | | | 0.476925 | -1.43965 | -1.2644 | 0.510709 | 0.174523 | 1.353671 | 0.09102 | 0.060098 | 0.272306 | -3.30641 | -1.23344 | -0.68917 | -0.47941 | -0.24625 | -0.45692 | -0.35898 | -1.30158 | -0.68383 | -0.83379 | -0.21774 | -0.60562 |
| B5TVM2 | B5TVM2 | ILDR2_MO | Ildr2 | Immunoglobulin-like domain-containing receptor 2 | | + | | NaN | 0 | NaN | 1.17103 | -1.15923 | -3.6529 | 0.075455 | -0.21975 | -0.22847 | -3.16304 | -3.19802 | -1.76247 | -2.33915 | -0.66882 | -2.51948 | -2.88879 | -4.03816 | -0.75902 | -1.02412 | -1.11191 | -1.63688 |
| Q9CXY6 | Q9CXY6 | ILF2_MOU | Ilf2 | Interleukin enhancer-binding factor 2 | | | | 0.875084 | 1.128432 | 2.456847 | 0.047857 | -0.03622 | -0.14837 | 0.639884 | 0.514075 | 1.711342 | -0.22406 | -0.02792 | -0.15165 | 0.328784 | 0.229507 | -0.36921 | -0.80571 | -1.70313 | 0.081284 | 0.16829 | -0.60883 | -0.55902 |
| Q9Z1X4 | Q9Z1X4;Q | ILF3_MOU | Ilf3 | Interleukin enhancer-binding factor 3 | | | + | 0.6126 | 1.290584 | 1.633322 | 0.908111 | 0.339505 | 2.574092 | 1.108262 | 1.199218 | 3.369171 | -0.66201 | -0.50026 | -1.43851 | -1.18948 | -0.41217 | -0.99112 | -1.08571 | -2.65773 | -1.69699 | -1.61001 | -1.69374 | -2.10798 |
| Q8CAQ8 | Q8CAQ8 | MIC60_MC | Immt | MICOS complex subunit Mic60 | + | | | 1.045586 | 0.471137 | 3.10327 | 0.224595 | 0.117619 | 0.62418 | 0.757387 | 0.292233 | 2.06586 | 3.723708 | 3.607112 | 2.901222 | 3.161821 | 3.445258 | 3.163132 | 3.334454 | 3.054093 | 3.05003 | 2.777774 | 3.022529 | 3.001396 |
| Q8VHZ7 | Q8VHZ7 | IMP4_MOL | Imp4 | U3 small nucleolar ribonucleoprotein protein IMP4 | | | | 0.408893 | 0.559398 | 1.088571 | 0.548971 | 1.289185 | 1.456702 | 0.285582 | -0.48141 | -0.77777 | 1.095402 | 0.158126 | -1.32006 | -1.10476 | -1.7208 | -0.58803 | -0.14348 | 0.278208 | -3.38002 | -1.62316 | -0.92266 | -0.42335 |
| P12970 | P12970 | RL7A_MOL | Rpl7a | Large ribosomal subunit protein eL8 | + | + | | 1.41064 | 0.824877 | 4.924609 | 1.149963 | 0.449379 | 3.555633 | 0.45891 | 0.385181 | 1.217369 | 2.210542 | 2.190355 | 2.510983 | 2.7634 | 2.274604 | 1.759995 | 1.542768 | 1.208375 | 2.194489 | 2.181137 | 1.447983 | 1.816255 |
| O55091 | O55091 | IMPCT_MC | Impact | Protein IMPACT | | | | 0.240716 | 0.525223 | 0.66496 | 0.169285 | -0.13343 | -0.48233 | 0.436777 | 0.24202 | 1.160077 | -0.05893 | -0.92952 | -0.16235 | -0.65207 | -0.58994 | -0.23245 | -1.67853 | -0.36036 | -0.14506 | -0.4025 | -0.76079 | -0.54564 |
| P24547 | P24547 | IMDH2_M | Impdh2 | Inosine-5'-monophosphate dehydrogenase 2 | | | | NaN | 0 | NaN | 0.262609 | -1.05657 | -0.7201 | 0.298205 | 0.74698 | 0.809415 | -3.07687 | -3.5362 | -1.0274 | -3.91188 | -3.48564 | -1.75746 | -3.50141 | -3.04365 | -1.14331 | -1.68284 | -3.69262 | -3.04445 |
| P46660 | P46660 | AINX_MOL | Ina | Alpha-internexin | | | + | 0.705821 | -1.43702 | -1.90633 | 0.215598 | -0.13979 | -0.60133 | 1.692687 | 0.716709 | 6.91281 | 3.311287 | 2.994947 | 2.934137 | 2.473008 | 3.740568 | 3.906578 | 5.327164 | 3.853101 | 2.872976 | 2.81374 | 3.04474 | 3.168988 |
| Q0GNC1 | Q0GNC1 | INF2_MOL | Inf2 | Inverted formin-2 | | + | | 0.128812 | 0.153817 | 0.375552 | 1.055298 | 0.539188 | 3.143394 | 0.162385 | -0.64807 | -0.46435 | -0.89504 | -1.70512 | -0.71765 | -1.05579 | -0.5873 | -3.27402 | -1.39311 | -1.51468 | -1.39694 | -1.45488 | -1.66102 | -0.90417 |
| Q04998 | Q04998 | INHBA_MC | Inhba | Inhibin beta A chain | | | | NaN | 0 | NaN | 0.652411 | 1.556866 | 1.747666 | 0.871967 | 1.060279 | 2.445965 | -3.27982 | -3.57385 | -3.05638 | -3.31443 | -2.88267 | -2.07097 | -3.17987 | -4.10585 | -3.92928 | -3.55526 | -3.68938 | -3.38481 |
| P49442 | P49442 | INPP_MOU | Inpp1 | Inositol polyphosphate 1-phosphatase | | | | 0.534086 | -2.04003 | -1.41636 | 0.2688 | -0.98923 | -0.73565 | 0.355293 | 1.016093 | 0.952745 | -1.85332 | -4.64701 | -1.20026 | -3.8884 | -1.61328 | -1.43448 | -0.85887 | -1.56139 | -1.59626 | -1.51393 | -1.47724 | -3.60271 |
| Q9EPW0 | Q9EPW0 | INP4A_MC | Inpp4a | Inositol polyphosphate-4-phosphatase type I A | + | | | 1.229738 | 0.661683 | 3.935398 | 0.145404 | -0.02429 | -0.41972 | 0.325547 | -0.1009 | -0.87798 | 0.964391 | 1.255006 | 1.675034 | 1.747182 | 1.249214 | 1.02603 | 0.532606 | 0.363426 | 1.690146 | 1.78065 | 1.211052 | 1.265992 |
| Q9JII1 | Q9JII1 | INP5E_MO | Inpp5e | Phosphatidylinositol polyphosphate 5-phosphatase type IV | | + | | NaN | 0 | NaN | 1.264346 | -0.70866 | -4.11023 | 0.114922 | -0.38102 | -0.33807 | -2.58283 | -4.05333 | -2.23343 | -2.05569 | -2.75653 | -2.33805 | -3.34791 | -2.92411 | -1.58364 | -1.28815 | -2.37373 | -1.05882 |
| Q8CDA1 | Q8CDA1 | SAC2_MOI | Inpp5f | Phosphatidylinositide phosphatase SAC2 | | + | | 0.1688 | -0.10552 | -0.48107 | 1.079166 | 0.408047 | 3.243659 | 0.239138 | 0.228736 | 0.660978 | 0.076772 | -0.24697 | 0.669738 | 0.921323 | 0.840458 | 0.353741 | 0.168433 | -0.1276 | 0.386232 | 0.388734 | 0.122332 | 0.614396 |
| P59644 | P59644 | PI5PA_MO | Inpp5j | Phosphatidylinositol 4,5-bisphosphate 5-phosphatase A | | | | 0.641842 | 1.309961 | 1.716999 | 0.372949 | -0.38663 | -0.99729 | 0.383973 | -0.59891 | -1.0252 | -1.59589 | -2.61776 | -2.30137 | -1.53129 | -1.2794 | -2.44745 | -3.98337 | -2.85021 | -1.57482 | -1.48459 | -1.25039 | -1.27862 |
| Q5DU31 | Q5DU31 | ICEF1_MO | Ipcef1 | Interactor protein for cytohesin exchange factors 1 | | | | NaN | 0 | NaN | 0.01911 | 0.02586 | 0.060936 | 0.095521 | -0.25457 | -0.28482 | -3.86642 | -3.18792 | -0.95771 | -0.81495 | -3.29543 | -1.52619 | -3.01921 | -2.755 | -1.33051 | -0.49387 | -2.28375 | -2.02874 |
| Q7TT16 | Q7TT16 | IPMK_MOL | Ipmk | Inositol polyphosphate multikinase | | | | 0.943563 | 0.662638 | 2.703994 | 0.308644 | 0.374575 | 0.835584 | 0.823808 | 0.415478 | 2.281595 | 0.509524 | 0.688233 | -0.03507 | 0.624735 | 0.636302 | 0.41252 | -0.29195 | 0.164428 | 0.223763 | -0.38325 | -0.03474 | 0.252602 |
| Q8K2V6 | Q8K2V6 | IPO11_MO | Ipo11 | Importin-11 | | | | 0.898508 | -0.76286 | -2.53964 | 0.664921 | -0.41266 | -1.78427 | 0.670882 | 1.043634 | 1.801823 | -4.08445 | -3.60258 | -4.32601 | -4.40335 | -3.35353 | -2.39147 | -2.90128 | -3.26004 | -3.724 | -4.18005 | -3.5935 | -4.23877 |
| Q8VI75 | Q8VI75 | IPO4_MOL | Ipo4 | Importin-4 | | | | 0.223564 | 0.672488 | 0.621566 | 0.020262 | 0.011131 | 0.064533 | 0.038451 | 0.023677 | 0.120258 | -0.72754 | -1.32274 | -0.55413 | -0.87687 | -0.46359 | -0.69118 | -0.65744 | -2.73782 | -0.78752 | -0.66574 | -0.76173 | -0.44039 |
| Q8BKC5 | Q8BKC5 | IPO5_MOL | Ipo5 | Importin-5 | + | | | 1.138046 | 0.481381 | 3.501534 | 0.031268 | 0.013883 | 0.098477 | 0.242646 | 0.276329 | 0.669832 | 0.272518 | 0.353436 | 0.474378 | 0.346159 | 0.787348 | 0.025692 | -0.29979 | -0.03702 | 0.270831 | 0.521941 | -0.02841 | 0.288794 |
| Q9EPL8 | Q9EPL8 | IPO7_MOL | Ipo7 | Importin-7 | | | | 0.179167 | 0.079198 | 0.507961 | 0.02603 | 0.02685 | 0.082412 | 0.664986 | 0.301856 | 1.784456 | 0.48868 | 0.721999 | 0.712889 | 0.085339 | 1.053209 | 0.722886 | 0.422704 | 0.62958 | 0.459974 | 0.284554 | 0.549637 | 0.622746 |
| Q91YE6 | Q91YE6 | IPO9_MOL | Ipo9 | Importin-9 | | | | 0.827667 | 1.135613 | 2.294509 | 0.017503 | -0.02359 | -0.05591 | 0.20431 | 0.125844 | 0.572563 | -1.2553 | -1.49036 | -1.49631 | -2.00718 | -0.80972 | -1.20435 | -2.98921 | -2.02767 | -2.06403 | -1.39229 | -1.22969 | -1.03606 |
| Q8BP00 | Q8BP00 | IQCB1_MC | Iqcb1 | IQ calmodulin-binding motif-containing protein 1 | | | | 0.877637 | 0.760937 | 2.465788 | 0.392371 | 0.50401 | 1.046505 | 0.453041 | -0.48811 | -1.20213 | -1.35179 | -1.00267 | -0.75134 | -0.39497 | -1.09844 | -1.25755 | -2.19265 | -1.68369 | -1.5246 | -0.62973 | -1.08805 | -0.29171 |
| Q3UQ44 | Q3UQ44 | IQGA2_MC | Iqgap2 | Ras GTPase-activating-like protein IQGAP2 | | | + | NaN | 0 | NaN | 0.113763 | -0.24132 | -0.33492 | 1.322167 | -0.74537 | -4.41697 | -3.16913 | -3.36504 | -3.06915 | -2.65966 | -0.76023 | -1.09557 | -3.85145 | -2.48361 | -3.31391 | -1.93224 | -0.16344 | -0.20162 |
| Q8R0S2 | Q8R0S2 | IQEC1_MC | Iqsec1 | IQ motif and SEC7 domain-containing protein 1 | + | + | | 1.04005 | 0.869984 | 3.080575 | 1.911283 | 0.250433 | 8.945758 | 0.466455 | -0.55842 | -1.23702 | 2.092102 | 1.753858 | 4.025197 | 4.076164 | 3.074325 | 3.403744 | 1.279166 | 0.826826 | 3.78866 | 3.811835 | 3.377152 | 4.217762 |
| Q5DU25 | Q5DU25 | IQEC2_MC | Iqsec2 | IQ motif and SEC7 domain-containing protein 2 | | + | | 0.525777 | 0.656815 | 1.393987 | 2.651175 | -0.39495 | -21.128 | 0.27836 | -0.40862 | -0.75965 | -0.07547 | -0.22971 | 1.406201 | 1.402601 | 2.171026 | 1.959165 | -0.34458 | -1.27423 | 1.780742 | 1.817955 | 1.946343 | 3.001098 |
| Q3TES0 | Q3TES0 | IQEC3_MC | Iqsec3 | IQ motif and SEC7 domain-containing protein 3 | + | | | 1.044697 | 0.58681 | 3.099619 | 0.906658 | 0.422539 | 2.568861 | 0.619613 | -0.49861 | -1.65324 | -0.09193 | -0.44352 | 1.282115 | 1.272935 | 0.095998 | 0.438221 | -0.92481 | -0.78426 | 0.690565 | 1.019407 | 0.517362 | 1.014071 |
| Q8VIM9 | Q8VIM9 | IRGQ_MOL | Irgq | Immunity-related GTPase family Q protein | | | + | 0.697037 | -0.36751 | -1.87979 | 0.499336 | -0.31559 | -1.32345 | 1.594437 | 0.275147 | 6.149019 | -0.78954 | -1.16961 | -0.21313 | -0.61178 | -0.71005 | -0.67609 | -0.65799 | -0.56613 | 0.034021 | -0.22776 | -0.92682 | -1.00962 |
| P81122 | P81122 | IRS2_MOU | Irs2 | Insulin receptor substrate 2 | | | + | NaN | 0 | NaN | 0.80543 | 0.705393 | 2.220679 | 3.043844 | -0.68299 | -33.2374 | -4.02369 | -3.02242 | -2.38706 | -1.78564 | -3.43055 | -3.43887 | -3.05193 | -3.14699 | -2.6894 | -2.89408 | -2.77184 | -2.7316 |
| Q9D924 | Q9D924;Q | ISCA1_MO | Isca1 | Iron-sulfur cluster assembly 1 homolog, mitochondrial | | | | 0.020333 | 0.065145 | 0.064752 | 0.523426 | -0.15472 | -1.38767 | 0.313526 | -0.11523 | -0.84782 | -0.02212 | -0.78306 | -0.33501 | -0.15752 | -0.03775 | -0.00247 | -1.39908 | 0.463606 | -0.02406 | -0.15904 | 0.229877 | -0.03964 |
| Q9DCB8 | Q9DCB8 | ISCA2_MO | Isca2 | Iron-sulfur cluster assembly 2 homolog, mitochondrial | + | | | 1.118996 | 0.474742 | 3.416415 | 0.027913 | -0.01807 | -0.0882 | 0.236036 | -0.20066 | -0.65314 | -0.11811 | -0.29205 | -0.14789 | -0.51646 | -0.50805 | -0.123 | -0.78821 | -0.57145 | -0.40358 | -0.22463 | 0.124544 | -0.35428 |
| Q9D7P6 | Q9D7P6 | ISCU_MOL | Iscu | Iron-sulfur cluster assembly enzyme ISCU | | | | 0.484358 | -0.23906 | -1.28391 | 0.236967 | 0.099265 | 0.655492 | 0.135731 | -0.09012 | -0.39405 | -0.40539 | -0.62019 | -0.43948 | -0.54226 | -0.86154 | -0.43179 | -0.12163 | -0.42583 | -0.44768 | -0.73258 | -0.4782 | -0.63489 |
| Q64339 | Q64339 | ISG15_MO | Isg15 | Ubiquitin-like protein ISG15 | | | | NaN | 0 | NaN | 0.194156 | -0.20483 | -0.54657 | 0.284207 | 0.725673 | 0.774317 | -3.47688 | -3.30724 | -4.15344 | -3.66767 | -3.48947 | -3.64635 | -3.20486 | -3.59346 | -3.99112 | -3.42032 | -3.35969 | -5.22747 |
| Q5RKR3 | Q5RKR3 | ISLR2_MO | Islr2 | Immunoglobulin superfamily containing leucine-rich repeat protein 2 | | | | NaN | 0 | NaN | 0.527852 | 0.705099 | 1.399563 | 0.316048 | 0.35376 | 0.854148 | -3.02112 | -3.86112 | -1.53586 | -2.13976 | -1.06446 | -1.84795 | -3.80293 | -3.15519 | -2.9462 | -2.13962 | -1.67554 | -1.94439 |
| P63037 | P63037 | DNJA1_MC | Dnaja1 | DnaJ homolog subfamily A member 1 | + | | | 1.375069 | 0.822065 | 4.714685 | 0.697102 | 0.302735 | 1.879991 | 0.362219 | -0.35835 | -0.9702 | 1.594495 | 1.25066 | 3.636358 | 3.329432 | 2.352854 | 2.421962 | 0.571414 | 0.629611 | 3.131376 | 3.228944 | 2.378021 | 3.113498 |
| Q9JHU9 | Q9JHU9 | INO1_MOL | Isyna1 | Inositol-3-phosphate synthase 1 | | | | 0.235387 | -1.14812 | -0.6515 | 0.44673 | 0.315051 | 1.185778 | 0.514368 | 0.343994 | 1.36343 | -3.02465 | -2.99908 | -0.28768 | -0.81468 | -0.49869 | -0.05265 | -3.62597 | -0.10154 | -0.83216 | -0.9003 | -0.5017 | -0.73763 |
| Q99KW9 | Q99KW9 | TIP_MOUS | Itfg1 | T-cell immunomodulatory protein | | | + | 0.068192 | -0.22113 | -0.20771 | 0.36723 | 0.224527 | 0.982847 | 2.131363 | 0.834439 | 11.56816 | -2.19271 | -2.79237 | -1.8362 | -1.75495 | -1.71758 | -1.58512 | -1.24986 | -3.29295 | -1.7953 | -2.2449 | -2.51437 | -2.45721 |
| P43406 | P43406 | ITAV_MOU | Itgav | Integrin alpha-V | | + | | 0.031319 | -0.08358 | -0.09863 | 1.77955 | -0.7697 | -7.66144 | 0.745637 | 0.759336 | 2.028925 | -2.91005 | -1.51382 | -1.54101 | -1.36065 | -0.80246 | -1.38707 | -2.60868 | -1.64804 | -0.7254 | -0.63685 | -1.62038 | -2.08782 |
| Q0VBD0 | Q0VBD0 | ITB8_MOU | Itgb8 | Integrin beta-8 | | + | | 0.880467 | 0.428132 | 2.475717 | 1.825678 | -0.70849 | -8.08966 | 0.86075 | 0.460921 | 2.407048 | 0.96643 | 1.258013 | -0.08215 | -0.08866 | 1.003689 | 0.655288 | 0.777098 | 0.591081 | 0.535561 | 0.710598 | 0.289057 | 0.448078 |
| O89051 | O89051 | ITM2B_MO | Itm2b | Integral membrane protein 2B | | | | 0.657979 | 0.10585 | 1.763914 | 0.90442 | 0.4535 | 2.560812 | 0.57394 | 0.383842 | 1.525173 | -0.18835 | -0.07639 | -0.03294 | -0.02547 | 0.114104 | -0.38918 | -0.25983 | -0.21662 | -0.30565 | -0.65976 | -0.52512 | -0.51764 |
| Q91VK4 | Q91VK4 | ITM2C_MC | Itm2c | Integral membrane protein 2C | | + | | 0.127593 | 0.053579 | 0.37228 | 1.309543 | 0.724779 | 4.348387 | 0.093397 | -0.07083 | -0.27893 | -0.54279 | -0.6245 | 0.982628 | 1.268738 | 0.610075 | 0.140213 | -0.77522 | -0.49922 | 0.315365 | 0.486442 | 0.349612 | 0.542328 |
| Q9D892 | Q9D892 | ITPA_MOU | Itpa | Inosine triphosphate pyrophosphatase | | | | 0.306242 | -0.68356 | -0.82956 | 0.125994 | 0.313495 | 0.367985 | 0.499403 | 0.336944 | 1.323629 | -3.13422 | -3.88272 | -2.05108 | -3.72699 | -1.90228 | -1.95199 | -2.09081 | -3.55902 | -3.35616 | -3.0489 | -2.51742 | -2.01073 |
| Q8BYN3 | Q8BYN3 | ITPK1_MO | Itpk1 | Inositol-tetrakisphosphate 1-kinase | | | | 0.028194 | -0.09384 | -0.08907 | 0.112135 | 0.070956 | 0.330489 | 0.253697 | 0.166768 | 0.697681 | -2.09588 | -3.22672 | -1.20472 | -1.54259 | -0.86174 | -1.3315 | -1.67842 | -3.45649 | -1.31211 | -1.57712 | -1.30774 | -1.21904 |
| Q8R071 | Q8R071 | IP3KA_MO | Itpka | Inositol-trisphosphate 3-kinase A | | | + | 0.215076 | 0.951992 | 0.600007 | 0.988959 | -0.74481 | -2.87681 | 1.165058 | -0.78852 | -3.62511 | -3.87515 | -0.74347 | 1.058897 | 0.606742 | 1.072705 | 1.505051 | -3.51734 | -3.00526 | 1.451458 | 1.703795 | 2.101545 | 2.053258 |
| P11881 | Q9Z329;P | ITPR1_MO | Itpr1 | Inositol 1,4,5-trisphosphate receptor type 1 | | | | 0.618126 | 1.471383 | 1.649008 | 0.684479 | -0.16141 | -1.84217 | 0.758578 | -0.55447 | -2.06962 | 0.272239 | 2.055755 | 0.986664 | 1.016499 | 0.008122 | 0.343278 | -0.27676 | -0.33801 | 1.076649 | 1.249326 | 0.521142 | 0.939196 |
| Q9Z329 | Q9Z329 | ITPR2_MO | Itpr2 | Inositol 1,4,5-trisphosphate receptor type 2 | | | | 0.914173 | -0.84335 | -2.596 | 0.593909 | -2.22259 | -1.5807 | 0.377857 | 1.302919 | 1.009707 | 1.266737 | 0.780952 | -1.04617 | -1.37018 | -0.32775 | -0.77846 | 2.082921 | 1.65146 | 0.806131 | -0.7773 | -0.58546 | -3.12659 |
| Q9Z0R6 | Q9Z0R4;Q | ITSN2_MO | Itsn2 | Intersectin-2 | | | | 0.145966 | 0.378015 | 0.421209 | 0.724145 | 0.270016 | 1.96227 | 0.249338 | -0.40189 | -0.6867 | -2.40765 | -2.49519 | -2.06763 | -1.97156 | -1.90672 | -3.06156 | -3.72582 | -1.93305 | -2.41856 | -2.16067 | -1.9869 | -2.17761 |
| Q9CWF2 | Q9CWF2 | TBB2B_MC | Tubb2b | Tubulin beta-2B chain | + | + | | 1.993158 | 0.819896 | 9.845787 | 1.233422 | 0.420987 | 3.953711 | 0.157206 | 0.137661 | 0.450796 | 3.412089 | 3.250541 | 3.987092 | 4.100435 | 4.457549 | 4.518187 | 2.531671 | 2.491168 | 3.532631 | 3.712922 | 4.046342 | 4.654072 |
| Q8BVL9 | Q8BVL9 | JKIP1_MOL | Jakmip1 | Janus kinase and microtubule-interacting protein 1 | | | | 0.532206 | -0.85 | -1.41129 | 0.314116 | 0.160342 | 0.849302 | 0.142773 | -0.10492 | -0.41276 | -1.116 | -1.47416 | -0.32702 | -0.00204 | -1.48214 | -1.04032 | -1.02013 | 0.129967 | -0.42099 | -0.22876 | -1.28207 | -1.03054 |
| D3YXK0 | D3YXK0 | D3YXK0_M | Jakmip2 | Janus kinase and microtubule interacting protein 2 | | | | 0.200946 | -0.80813 | -0.56396 | 0.25489 | 0.110884 | 0.700684 | 0.101586 | 0.048501 | 0.301592 | -1.79598 | -3.3784 | -0.73446 | -0.45656 | -1.34382 | -1.58145 | -0.58434 | -2.97377 | -0.78213 | -0.63066 | -1.61951 | -1.40276 |
| Q5DTX6 | Q5DTX6 | JCAD_MOI | Jcad | Junctional cadherin 5-associated protein | | | | 0.543481 | 1.163227 | 1.441783 | 0.694006 | 0.136079 | 1.870679 | 0.579834 | 0.401033 | 1.54149 | -0.50844 | -0.63489 | 1.088467 | 1.10513 | 0.503963 | -0.01408 | -0.93058 | -2.53921 | 0.888456 | 1.032984 | -0.13178 | -0.1804 |
| Q9CR30 | Q9CR30 | JOS2_MOL | Josd2 | Josephin-2 | | | | 0.624036 | -1.88655 | -1.66585 | 0.023783 | -0.043 | -0.07547 | 0.270067 | 0.723781 | 0.738836 | -3.11713 | -1.45526 | -3.54869 | -3.24453 | -2.01215 | -1.70888 | -1.1691 | 0.369821 | -2.80448 | -3.90273 | -1.61648 | -3.55211 |
| Q9ET80 | Q9ET80 | JPH1_MOL | Jph1 | Junctophilin-1 | | | | NaN | 0 | NaN | 0.628281 | 0.467754 | 1.677985 | 0.787573 | -0.78339 | -2.1624 | -3.53634 | -4.34348 | -0.67369 | -0.36338 | -1.21464 | -0.91056 | -3.25655 | -3.33018 | -1.21788 | -0.7547 | -0.60804 | 0.049616 |
| Q8BWG8 | Q8BWG8 | ARRB1_M | Arrb1 | Beta-arrestin-1 | + | + | | 1.449053 | 0.819533 | 5.160572 | 1.142676 | 0.497233 | 3.522475 | 0.038369 | -0.02436 | -0.12001 | 1.645376 | 1.744826 | 0.75167 | 1.031265 | -0.11744 | 0.287498 | 0.724747 | 1.026389 | 0.374668 | 0.413802 | 0.123809 | 0.094973 |
| Q80WT0 | Q80WT0 | JPH4_MOL | Jph4 | Junctophilin-4 | | | | NaN | 0 | NaN | 0.920583 | -0.42802 | -2.6193 | 0.81561 | -0.83184 | -2.2543 | -3.11692 | -3.57372 | -0.84705 | -0.52029 | -1.14553 | -0.75285 | -3.09482 | -3.30364 | -0.25242 | -0.25888 | -0.42978 | 0.195084 |
| P15066 | P15066 | JUND_MOI | Jund | Transcription factor JunD | | | | 0.024093 | 0.125744 | 0.076431 | 0.730886 | 0.65151 | 1.983054 | 0.7558 | 1.244384 | 2.06085 | -1.5062 | -4.65452 | -1.08233 | -0.57003 | -1.88473 | -1.43365 | -3.68432 | -2.72788 | -1.68342 | -1.27196 | -3.46369 | -2.34346 |
| Q02257 | Q02257 | PLAK_MOL | Jup | Junction plakoglobin | | | | 0.482269 | 0.813087 | 1.278422 | 0.022421 | 0.035362 | 0.071248 | 0.334554 | 0.247981 | 0.900586 | 3.498859 | 2.37574 | 1.96936 | 1.792631 | 1.675252 | 2.19803 | 1.82562 | 2.422804 | 1.357239 | 2.334028 | 1.602077 | 1.775242 |
| Q6P9J5 | Q6P9J5 | KANK4_M | Kank4 | KN motif and ankyrin repeat domain-containing protein 4 | | | | NaN | 0 | NaN | 0.08941 | 0.272238 | 0.267813 | NaN | 0 | NaN | -2.86956 | -3.7313 | -3.18599 | -4.32416 | -3.92221 | -3.70206 | -3.56105 | -3.21019 | -3.18502 | -4.86961 | -2.75067 | -3.18881 |
| Q99MN1 | Q99MN1 | SYK_MOUS | Kars1 | Lysine--tRNA ligase | | | | 0.122053 | 0.104937 | 0.357375 | 0.036778 | -0.00833 | -0.11521 | 0.112123 | 0.12771 | 0.330457 | -0.49257 | -0.09552 | 0.46934 | 0.360317 | 0.411428 | -0.35603 | -0.18263 | -0.61533 | 0.470618 | 0.375694 | -0.14592 | -0.0541 |
| Q8K0T4 | Q9WV86;Q | KATL1_MC | Katnal1 | Katanin p60 ATPase-containing subunit A-like 1 | | | | 0.343717 | -0.80894 | -0.92361 | 0.007899 | 0.014811 | 0.025494 | 0.17483 | 0.819862 | 0.496732 | -3.15406 | -3.50513 | -0.62376 | -0.90989 | -1.37092 | -2.31851 | -1.66258 | -3.37873 | -1.34469 | -1.21858 | -1.08353 | -4.24562 |
| Q8BG40 | Q8BG40 | KTNB1_MC | Katnb1 | Katanin p80 WD40 repeat-containing subunit B1 | | | | 0.045091 | 0.123277 | 0.140149 | 0.480956 | -0.42334 | -1.27497 | 0.014284 | -0.01968 | -0.04578 | -3.37628 | -1.66153 | -1.91884 | -1.52539 | -0.9205 | -1.22352 | -2.44563 | -2.83873 | -0.93396 | -0.6636 | -0.65002 | -1.45464 |
| Q8BNW9 | Q8BNW9 | KBTBB_MC | Kbtbd11 | Kelch repeat and BTB domain-containing protein 11 | | | + | 0.521074 | 0.257364 | 1.381367 | 0.136804 | -0.09266 | -0.3969 | 1.19888 | -0.24834 | -3.78477 | -0.31321 | 0.036462 | 0.592064 | 0.78648 | 0.953992 | 0.918287 | -0.33136 | -0.46011 | 0.569669 | 0.994202 | 1.121338 | 1.247618 |

| | | | | | | | | | | | | | | | | | | | | | | | | | | | |
|---|---|---|---|---|---|---|---|---|---|---|---|---|---|---|---|---|---|---|---|---|---|---|---|---|---|---|---|
| P16388 | P63141;Q( | KCNA1_M( | Kcna1 | Potassium voltage-gated channel subfamily A member 1 | | | | 0.064776 | 0.086034 | 0.197865 | 0.023662 | -0.00466 | -0.0751 | 0.629572 | 0.500541 | 1.681682 | 1.505929 | 1.561256 | 1.255641 | 1.286054 | 1.499975 | 0.97103 | 1.881489 | 1.013628 | 1.215401 | 1.335605 | 0.598412 | 0.871511 |
| P63141 | P63141 | KCNA2_M( | Kcna2 | Potassium voltage-gated channel subfamily A member 2 | | | | 0.086882 | -0.0366 | -0.26074 | 0.343335 | -0.35808 | -0.92265 | 0.170855 | -0.15898 | -0.48641 | -0.86342 | -0.84322 | -1.26573 | -1.82578 | -1.01387 | -1.62885 | -0.67671 | -0.95672 | -1.45639 | -0.91896 | -1.2732 | -1.05155 |
| Q61423 | Q61423 | KCNA4_M( | Kcna4 | Potassium voltage-gated channel subfamily A member 4 | | | | 0.388274 | 0.630802 | 1.036102 | 0.213424 | 0.100145 | 0.595802 | 0.03244 | 0.055592 | 0.102052 | -1.68981 | -2.88407 | -0.33103 | -0.66655 | -0.26208 | -1.33969 | -2.799 | -3.03648 | -0.60936 | -0.58851 | -0.9367 | -0.77627 |
| P62482 | P62482 | KCAB2_MC | Kcnab2 | Voltage-gated potassium channel subunit beta-2 | | | | 0.921513 | -0.40876 | -2.62269 | 0.000683 | -0.00052 | -0.00222 | 0.235295 | -0.13546 | -0.65127 | 1.932002 | 1.728812 | 1.741994 | 1.79833 | 1.593031 | 1.942675 | 2.357364 | 2.120977 | 2.003114 | 1.538251 | 2.015999 | 1.790626 |
| Q63959 | Q63959 | KCNC3_M( | Kcnc3 | Potassium voltage-gated channel subfamily C member 3 | | | | 0.085225 | -0.12257 | -0.25609 | 0.207512 | -0.23575 | -0.58074 | 0.057939 | 0.227964 | 0.17802 | 0.925178 | 0.796834 | -1.56916 | -2.37452 | -1.18242 | -3.12138 | 1.457891 | 0.509265 | -1.78751 | -1.68466 | -3.21649 | -1.54324 |
| O35988 | O35988 | SDC4_MO | Sdc4 | Syndecan-4 | + | | | 1.775167 | 0.815835 | 7.621904 | 0.213363 | -0.2496 | -0.59565 | 0.005477 | -0.00842 | -0.01772 | -0.05411 | -0.05761 | 0.609056 | 1.443987 | 0.96587 | 0.891965 | -0.97872 | -0.76467 | 1.239772 | 1.312476 | 0.463791 | 1.410881 |
| Q9Z0V1 | Q9Z0V1 | KCND3_M( | Kcnd3 | Potassium voltage-gated channel subfamily D member 3 | | + | | 0.021324 | -0.13972 | -0.06784 | 1.14743 | -0.5277 | -3.54408 | 0.4185 | -0.20727 | -1.11313 | -0.62217 | -3.24384 | -0.86428 | -0.6002 | -0.11799 | -0.36037 | -3.38194 | -0.20463 | -0.27335 | -0.13573 | -0.17328 | 0.109461 |
| Q9QXT8 | Q9QXT8 | CSEN_MO | Kcnip3 | Calsenilin | | | | NaN | 0 | NaN | 0.028745 | 0.02834 | 0.090759 | 0.220112 | 0.380484 | 0.612805 | -3.66799 | -3.28622 | -3.05107 | -2.62462 | -2.20543 | -3.18865 | -3.48201 | -3.38828 | -2.63806 | -3.09431 | -3.45676 | -2.69829 |
| Q6PHZ8 | Q9JJ57;Q6 | KCIP4_MO | Kcnip4 | Kv channel-interacting protein 4 | | | | 0.507385 | 1.102358 | 1.34482 | 0.398789 | -0.2641 | -1.06282 | 0.198247 | -0.11095 | -0.55706 | -1.37485 | -0.63886 | -1.0743 | -0.6852 | -0.33809 | -0.49723 | -1.37675 | -2.84168 | -0.46108 | -0.77023 | -0.4893 | -0.12412 |
| Q9DCM2 | Q9DCM2 | GSTK1_MC | Gstk1 | Glutathione S-transferase kappa 1 | + | | | 2.487502 | 0.807954 | 17.48594 | 0.292589 | 0.47795 | 0.795336 | 0.607702 | -0.448 | -1.61947 | 1.547842 | 1.63782 | 2.311009 | 3.502484 | 1.769114 | 2.06782 | 0.795411 | 0.774343 | 2.349887 | 2.507706 | 2.133615 | 2.59932 |
| Q08460 | Q08460 | KCMA1_M( | Kcnma1 | Calcium-activated potassium channel subunit alpha-1 | | + | | 0.930413 | 2.895074 | 2.655308 | 1.673892 | -1.06448 | -6.76017 | 0.726823 | 0.168456 | 1.970514 | -1.49517 | 0.685403 | -0.12852 | -0.42486 | 1.121746 | 0.959056 | -3.3045 | -3.29541 | 0.841069 | 0.734499 | 0.845654 | 0.898237 |
| P58390 | P58390 | KCNN2_M( | Kcnn2 | Small conductance calcium-activated potassium channel protein 2 | | | | 0.449256 | 1.181901 | 1.192317 | 0.521513 | -0.35869 | -1.38254 | 0.787801 | -0.34966 | -2.16314 | -1.91257 | -1.20559 | -1.9341 | -1.44568 | -0.39137 | -0.34127 | -1.81489 | -3.66708 | -1.24363 | -1.41878 | -0.17635 | 0.143039 |
| Q9Z351 | Q9Z351 | KCNQ2_M | Kcnq2 | Potassium voltage-gated channel subfamily KQT member 2 | | | | 0.352483 | 0.712524 | 0.945668 | 0.273545 | 0.140622 | 0.747568 | 0.256362 | -0.28672 | -0.70439 | -1.66878 | -1.50767 | 0.73889 | 1.066778 | 0.768548 | 0.526619 | -1.55161 | -3.04989 | 0.669983 | 0.854441 | 0.545643 | 1.322973 |
| Q9JK45 | Q9JK45 | KCNQ5_M | Kcnq5 | Potassium voltage-gated channel subfamily KQT member 5 | | | | NaN | 0 | NaN | 0.212261 | 1.472252 | 0.592842 | 0.360674 | -2.06137 | -0.96631 | -3.4368 | -3.63073 | 0.054476 | 0.601275 | -4.12553 | -3.82821 | -3.34899 | -2.97473 | 1.323909 | -3.61266 | 0.212567 | -4.04356 |
| Q6ZPR4 | Q6ZPR4;D | KCNT1_MC | Kcnt1 | Potassium channel subfamily T member 1 | | | | NaN | 0 | NaN | 0.084434 | 0.1114 | 0.253865 | 0.249618 | 0.914707 | 0.687408 | -2.82619 | -4.11846 | -1.32304 | -0.81153 | -1.39573 | -0.72495 | -4.01846 | -3.48038 | -0.8221 | -1.53527 | -3.26275 | -0.68735 |
| D3Z649 | D3Z649 | D3Z649_M | Kcnt2 | Potassium channel, subfamily T, member 2 | | | | NaN | 0 | NaN | 0.559704 | -1.72473 | -1.48601 | 0.907178 | -1.44233 | -2.57073 | -3.21383 | -2.95771 | -1.67407 | -3.94384 | -1.53893 | -2.63177 | -3.43382 | -3.33535 | -0.84105 | -1.3274 | -0.77034 | -0.51569 |
| Q6WVG3 | Q5DTY9;Q | KCD12_M( | Kctd12 | BTB/POZ domain-containing protein KCTD12 | | | | 0.502868 | 0.44299 | 1.332818 | 0.79656 | -0.41746 | -2.19162 | 0.299229 | -0.18852 | -0.81198 | -0.31196 | 0.308289 | 0.862554 | 1.002347 | 1.637211 | 2.047136 | -0.32527 | -0.56438 | 1.17272 | 1.52711 | 1.921638 | 2.139739 |
| Q5DTY9 | Q5DTY9 | KCD16_M( | Kctd16 | BTB/POZ domain-containing protein KCTD16 | | | | 0.113063 | -0.5174 | -0.33302 | 0.422735 | -0.44726 | -1.12398 | 0.032312 | 0.032795 | 0.101662 | -1.21542 | -3.62046 | 1.553474 | 1.328963 | 0.919313 | 0.576479 | -0.91672 | -2.88436 | 1.506715 | 2.270243 | 0.441828 | 0.988376 |
| E0CYQ0 | E0CYQ0 | E0CYQ0_I | Kctd17 | Potassium channel tetramerisation domain containing 17 | | | | NaN | 0 | NaN | 0.122979 | 0.296026 | 0.359872 | 0.677102 | -0.46354 | -1.82023 | -3.12904 | -3.22589 | -2.46611 | -3.77257 | -3.69746 | -3.19459 | -3.43392 | -2.88343 | -3.91531 | -2.91543 | -2.94207 | -3.0229 |
| Q8BFX3 | Q8BFX3 | KCTD3_MC | Kctd3 | BTB/POZ domain-containing protein KCTD3 | | | | NaN | 0 | NaN | 0.003732 | -0.00763 | -0.0121 | 0.209575 | -0.6389 | -0.586 | -3.40139 | -3.81494 | -2.65344 | -1.39441 | -3.61888 | -2.53069 | -2.9124 | -2.27529 | -2.05311 | -1.97948 | -3.38069 | -1.49108 |
| Q9D7X1 | Q9D7X1 | KCTD4_MC | Kctd4 | BTB/POZ domain-containing protein KCTD4 | | | | 0.808451 | 0.84043 | 2.230625 | 0.45503 | 0.53378 | 1.207287 | 0.861081 | -0.47499 | -2.40819 | -1.364 | -1.73714 | -0.30288 | -0.20127 | 0.03581 | 0.422847 | -2.06367 | -2.71833 | -1.22506 | -0.34666 | 0.666192 | 0.742441 |
| P49312 | P49312 | ROA1_MO | Hnrnpa1 | Heterogeneous nuclear ribonucleoprotein A1 | + | | | 2.729757 | 0.807308 | 23.13507 | 0.187055 | -0.2551 | -0.52831 | 0.643993 | 0.501673 | 1.723221 | 0.83988 | 0.827326 | -0.05803 | 0.793742 | 0.839443 | 0.276483 | -0.00803 | 0.060621 | 0.850502 | 0.395413 | -0.01803 | 0.130607 |
| Q3UXZ9 | Q3UXZ9 | KDM5A_M | Kdm5a | Lysine-specific demethylase 5A | | | | NaN | 0 | NaN | 0.650621 | 0.491402 | 1.742456 | 0.2075 | -0.16813 | -0.58071 | -3.27159 | -3.74198 | 0.892225 | 0.967021 | 0.396779 | -0.00698 | -4.15583 | -2.74308 | 0.717747 | 0.158695 | 0.155489 | 0.570583 |
| Q9Z2X8 | Q9Z2X8 | KEAP1_MC | Keap1 | Kelch-like ECH-associated protein 1 | | | | 0.044348 | -0.03897 | -0.13794 | 0.580044 | 0.1435 | 1.542073 | 0.848772 | -0.98621 | -2.36592 | -1.27363 | -1.82829 | 0.417104 | 0.519537 | -1.1564 | -1.15555 | -1.56604 | -1.45793 | 0.402515 | 0.247126 | -0.58661 | 0.24707 |
| Q60749 | Q9R226;Q | KHDR1_M( | Khdrbs1 | KH domain-containing, RNA-binding, signal transduction-associated protein 1 | | | | 0.89781 | 0.982257 | 2.537144 | 0.570888 | -0.30129 | -1.51675 | 0.179592 | 0.081795 | 0.509059 | 1.488703 | 1.776084 | 1.899232 | 2.279458 | 2.100542 | 1.845266 | 0.290639 | 1.009634 | 2.333055 | 2.448212 | 1.988712 | 1.793505 |
| Q9R226 | Q9R226 | KHDR3_M( | Khdrbs3 | KH domain-containing, RNA-binding, signal transduction-associated protein 3 | | | | 0.125402 | 0.241868 | 0.366395 | 0.313125 | 0.98466 | 0.846819 | 0.175071 | 0.53748 | 0.497356 | -3.32694 | -2.01028 | -2.14718 | -1.44079 | -1.03188 | -3.10598 | -2.95919 | -2.86177 | -3.88648 | -1.67081 | -2.30246 | -2.91037 |
| Q3U0V1 | Q3U0V1 | FUBP2_MC | Khsrp | Far upstream element-binding protein 2 | | | | 0.886186 | 0.405543 | 2.495868 | 0.768833 | 0.129131 | 2.102182 | 0.217413 | 0.152465 | 0.605947 | 0.652145 | 0.975769 | 0.775859 | 0.845251 | 0.889274 | 1.243144 | 0.423198 | 0.39363 | 0.630734 | 0.732114 | 1.09264 | 0.734847 |
| Q8R0A7 | Q8R0A7 | K0513_MC | Kiaa0513 | Uncharacterized protein KIAA0513 | | + | | 0.161662 | 0.302152 | 0.462459 | 1.445677 | 0.472297 | 5.139435 | 0.709762 | 0.44996 | 1.918294 | -0.84694 | -1.79048 | 0.090004 | -0.07321 | -0.26279 | -0.66923 | -1.16885 | -2.07287 | -0.42165 | -0.50615 | -1.0331 | -0.79883 |
| Q68FD9 | Q68FD9 | K1549_MC | Kiaa1549 | UPF0606 protein KIAA1549 | | | | 0.318021 | 0.595019 | 0.859097 | 0.435404 | 0.401531 | 1.15654 | 0.321506 | 0.50912 | 0.867837 | -1.7919 | -3.16929 | -0.45679 | -0.71912 | -0.11992 | -1.03411 | -3.14917 | -3.00206 | -0.66803 | -1.31093 | -1.45387 | -0.7184 |
| Q8BRV5 | Q8BRV5 | K1671_MC | Kiaa1671 | Uncharacterized protein KIAA1671 | | | | NaN | 0 | NaN | 0.279368 | 0.766628 | 0.762179 | 0.024561 | 0.064743 | 0.077877 | -3.232 | -2.78188 | -1.11362 | -1.28147 | -1.22052 | -0.66751 | -3.16265 | -3.18427 | -2.9665 | -0.96184 | -1.79278 | -0.22474 |
| E9Q9B7 | E9Q9B7 | E9Q9B7_M | Kidins220 | Kinase D-interacting substrate 220 | + | | | 1.233898 | -0.61079 | -3.95608 | 0.217402 | -0.15009 | -0.60592 | 0.599044 | -0.22391 | -1.59509 | -1.02512 | -1.32073 | -0.66637 | -0.2113 | -0.71245 | -0.60759 | -0.51752 | -0.60675 | -0.38665 | -0.19084 | -0.56632 | -0.3059 |
| Q61699 | Q61699 | HS105_MC | Hsph1 | Heat shock protein 105 kDa | + | + | | 1.540013 | 0.796994 | 5.760384 | 1.000796 | 0.808863 | 2.923116 | 0.062456 | -0.05347 | -0.19115 | 1.853025 | 1.582418 | 3.290483 | 3.564932 | 2.253823 | 2.712348 | 0.949638 | 0.891816 | 2.378554 | 2.859134 | 2.376258 | 2.696862 |
| Q60575 | Q60575 | KIF1B_MO | Kif1b | Kinesin-like protein KIF1B | | + | | NaN | 0 | NaN | 1.761606 | 1.156547 | 7.500774 | 0.006522 | -0.00631 | -0.02108 | -3.65781 | -3.67448 | -2.56847 | -2.28883 | -3.42416 | -3.6116 | -3.74003 | -3.40194 | -3.65019 | -3.5202 | -3.7957 | -3.22745 |
| Q9QXL2 | Q9QXL2 | KI21A_MO | Kif21a | Kinesin-like protein KIF21A | | | + | 0.189277 | 0.09217 | 0.534033 | 0.03436 | -0.01817 | -0.10789 | 1.697061 | 0.262095 | 6.9488 | 0.725267 | 0.54484 | 1.041523 | 1.158271 | 1.191606 | 1.127817 | 0.690022 | 0.395744 | 0.96012 | 1.276008 | 0.877481 | 0.91775 |
| P28740 | P28740 | KIF2A_MO | Kif2a | Kinesin-like protein KIF2A | | + | | 0.537266 | 0.470036 | 1.42495 | 1.115616 | -0.54239 | -3.40148 | 0.129187 | -0.08799 | -0.37656 | 1.082482 | 0.89721 | 1.592867 | 1.858893 | 1.876101 | 1.946523 | 0.836396 | 0.203223 | 2.180325 | 2.356207 | 2.230287 | 1.768308 |
| P28741 | P28741 | KIF3A_MO | Kif3a | Kinesin-like protein KIF3A | | | | 0.019372 | 0.082776 | 0.061755 | 0.379748 | 0.112192 | 1.014493 | 0.038662 | -0.03179 | -0.1209 | -3.38244 | -2.27432 | -1.00821 | -0.80744 | -0.89107 | -1.41428 | -4.1317 | -1.69062 | -1.06641 | -0.97362 | -1.1478 | -1.09396 |
| O35066 | O35066 | KIF3C_MO | Kif3c | Kinesin-like protein KIF3C | | | | 0.236101 | 0.564219 | 0.653304 | 0.313467 | -1.32028 | -0.84767 | 0.419027 | 0.213408 | 1.11448 | -1.76582 | -3.18501 | -1.06546 | -4.18036 | -1.47353 | -1.18502 | -2.54734 | -3.53192 | -1.28659 | -1.31867 | -1.66861 | -1.41676 |
| P33175 | P33175 | KIF5A_MO | Kif5a | Kinesin heavy chain isoform 5A | | | | 0.074254 | -0.18164 | -0.22505 | 0.311385 | 0.513685 | 0.842455 | 0.322893 | -0.81555 | -0.87132 | -3.74823 | -2.24951 | -0.77534 | -0.37035 | -0.96823 | -2.83673 | -3.11706 | -2.5174 | -0.51139 | -1.66167 | -1.02988 | -1.144 |
| Q61768 | Q61768 | KINH_MOI | Kif5b | Kinesin-1 heavy chain | | | | 0.422503 | -0.5457 | -1.12339 | 0.461641 | -0.24727 | -1.22447 | 0.744135 | -0.63204 | -2.02423 | -0.92133 | -0.50787 | 0.167758 | -0.21493 | -0.92289 | -0.80132 | 0.270683 | -0.60848 | 0.159128 | 0.288246 | 0.076194 | -0.53633 |
| P28738 | P33175;P2 | KIF5C_MO | Kif5c | Kinesin heavy chain isoform 5C | + | | | 1.159467 | 0.387667 | 3.599251 | 0.126649 | 0.078987 | 0.369747 | 0.254637 | -0.08556 | -0.70005 | 0.17669 | 0.348228 | 0.92944 | 0.809005 | 0.535101 | 0.441981 | -0.06006 | -0.19036 | 0.585272 | 0.995198 | 0.687096 | 0.461099 |
| Q921F2 | Q921F2 | TADBP_MC | Tardbp | TAR DNA-binding protein 43 | + | + | | 1.670209 | 0.790133 | 6.730631 | 1.530895 | 0.242892 | 5.697528 | 0.223637 | 0.198067 | 0.621751 | 0.490166 | 0.639305 | 0.354591 | 0.433543 | 1.025268 | 0.388173 | -0.13473 | -0.31607 | 0.16727 | 0.135081 | 0.511904 | 0.505402 |
| O88448 | Q9DBS5;C | KLC2_MOI | Klc2 | Kinesin light chain 2 | | + | | 0.360579 | 0.200078 | 0.966065 | 1.123264 | -0.08032 | -3.43534 | 0.454566 | 0.338792 | 1.206082 | 0.335822 | 0.520354 | 0.586894 | 0.569338 | 0.432443 | 0.627149 | 0.042592 | 0.413428 | 0.636767 | 0.680109 | 0.454497 | -0.07249 |
| Q9D2D9 | Q9D2D9 | KLD8B_MC | Klhdc8b | Kelch domain-containing protein 8B | | | | NaN | 0 | NaN | 0.14739 | 0.530046 | 0.424972 | 0.897284 | 1.359937 | 2.535267 | -3.52565 | -2.87674 | -2.67375 | -1.49316 | -1.36019 | -1.42592 | -3.1696 | -3.30124 | -3.71222 | -1.51478 | -2.21759 | -3.28839 |
| Q99JN2 | Q99JN2 | KLH22_MC | Klhl22 | Kelch-like protein 22 | | | | 0.155769 | -0.16981 | -0.44703 | 0.125383 | 0.107632 | 0.366342 | 0.366092 | -0.57392 | -0.97997 | -3.21634 | -2.45995 | -1.46886 | -1.56843 | -1.6111 | -1.26318 | -2.70397 | -2.63269 | -1.91583 | -1.33672 | -1.42243 | -0.304 |
| Q8BGY4 | Q8BGY4 | KLH26_MC | Klhl26 | Kelch-like protein 26 | | | | 0.14748 | 0.729595 | 0.425207 | 0.284178 | -0.20372 | -0.77425 | 0.531842 | -1.22934 | -1.41031 | -3.36169 | -0.15286 | 1.757338 | 1.231673 | 0.720821 | 1.51942 | -1.87858 | -3.09516 | 1.685927 | 1.710523 | 1.574613 | 3.124305 |
| Q0KK55;Q | Q2EG98;Q | KNDC1_M | Kndc1;Pkd1l3 | Kinase non-catalytic C-lobe domain-containing protein 1 | + | | + | 1.270775 | 0.674196 | 4.143416 | 0.943159 | -0.78774 | -2.70249 | 1.175841 | -0.82249 | -3.67541 | -2.57522 | -2.47705 | -2.01853 | -1.66372 | -3.22572 | -2.81563 | -3.35547 | -3.04519 | -0.8221 | -1.28467 | -2.28782 | -2.10854 |
| Q60960 | Q60960 | IMA5_MOI | Kpna1 | Importin subunit alpha-5 | | | | 0.589103 | 1.883136 | 1.567269 | 0.183453 | 0.193731 | 0.519031 | 0.096077 | 0.072138 | 0.286366 | 0.389179 | 0.128661 | 0.324455 | 0.66366 | 0.5424 | 0.073707 | -0.42976 | -2.81867 | -0.03217 | 0.632823 | 0.143512 | 0.328321 |
| O35344 | O35344 | IMA4_MOI | Kpna3 | Importin subunit alpha-4 | | | | 0.157971 | 0.880365 | 0.452801 | 0.251318 | 0.162192 | 0.691692 | 0.311676 | 0.395781 | 0.843185 | -0.76721 | -0.75346 | 0.11866 | 0.574448 | 0.290807 | -0.47759 | 0.303556 | -3.58495 | 0.239573 | 0.129151 | -0.75883 | -0.21952 |
| O35345 | O35345 | IMA7_MOI | Kpna6 | Importin subunit alpha-7 | | | | 0.018608 | 0.125301 | 0.059366 | 0.145189 | 0.123137 | 0.419154 | 0.580666 | 0.527659 | 1.543798 | 0.333153 | -3.76699 | -0.44699 | -1.0152 | 0.280212 | -0.18949 | -2.34425 | -1.34019 | -0.77949 | -0.92898 | -0.73063 | -0.23397 |
| P70168 | P70168 | IMB1_MOI | Kpnb1 | Importin subunit beta-1 | | + | | 0.567744 | 0.610198 | 1.508087 | 2.212197 | 0.76448 | 12.70847 | 0.134907 | 0.09264 | 0.391849 | 0.653558 | 1.209898 | 1.753439 | 1.857008 | 1.337445 | 1.009698 | 0.615361 | 0.0277 | 1.071354 | 1.010132 | 0.910525 | 1.251339 |
| P32883 | P08556;P3 | RASK_MOI | Kras | GTPase KRas | | | | 0.792509 | -1.38105 | -2.17842 | 0.175403 | 0.437791 | 0.498217 | 0.174922 | 0.265415 | 0.49697 | -2.73691 | -1.46923 | 0.203496 | -0.61547 | -0.45403 | -0.16562 | -0.73476 | -0.70928 | -1.42125 | 0.133694 | -0.06101 | -1.08947 |
| Q6S5J6 | Q6S5J6 | KRIT1_MOI | Krit1 | Krev interaction trapped protein 1 | | | | 0.019825 | -0.03371 | -0.06317 | 0.585311 | 0.342088 | 1.556705 | 0.437171 | 0.560917 | 1.161092 | 0.023884 | -0.25725 | -0.2945 | -0.67889 | -0.74452 | 0.004056 | 0.431816 | -0.59776 | -0.93532 | -0.72224 | -1.23658 | -0.62572 |
| P04104 | P04104 | K2C1_MOI | Krt1 | Keratin, type II cytoskeletal 1 | | + | | 0.266105 | 0.457079 | 0.728885 | 1.273154 | 0.140589 | 4.155756 | 0.334836 | 0.466554 | 0.901295 | 8.718473 | 7.54 | 6.434619 | 6.371941 | 6.613403 | 7.495815 | 7.457573 | 7.886743 | 6.275431 | 6.249952 | 6.317319 | 6.85879 |
| P02535 | Q9Z2K1;Q | K1C10_MC | Krt10 | Keratin, type I cytoskeletal 10 | | | | 0.239961 | 0.400111 | 0.663056 | 0.099676 | -0.03276 | -0.29632 | 0.272284 | 0.306026 | 0.744401 | 8.016676 | 7.090353 | 5.765441 | 5.986029 | 5.977394 | 6.463371 | 6.766602 | 7.540205 | 5.900853 | 5.916139 | 5.582749 | 6.245963 |
| Q64291 | Q64291 | K1C12_MC | Krt12 | Keratin, type I cytoskeletal 12 | | | | 0.119676 | -1.06876 | -0.35095 | 0.105205 | -0.28772 | -0.31154 | 0.133342 | -0.77606 | -0.38767 | 2.023899 | -1.09623 | 3.157298 | 1.347315 | -3.24458 | 0.538806 | 4.147943 | -1.08276 | 2.355921 | 2.724128 | 0.07805 | -1.23171 |
| P08730 | P08730 | K1C13_MC | Krt13 | Keratin, type I cytoskeletal 13 | | | | 0.33107 | -3.24971 | -0.89184 | 0.02812 | 0.075369 | 0.088841 | 0.07906 | 0.660851 | 0.238703 | 2.009118 | -3.93472 | 3.460399 | 1.824562 | -2.65189 | 0.807034 | 4.395272 | 0.178537 | 2.341898 | 2.792325 | 0.578572 | -3.74513 |
| Q61781 | Q61781 | K1C14_MC | Krt14 | Keratin, type I cytoskeletal 14 | | | | 0.43242 | -0.55288 | -1.14886 | 0.198665 | 0.441025 | 0.558126 | 0.004621 | 0.0072 | 0.014968 | 0.746796 | 0.681569 | 0.650987 | 1.661211 | 1.052403 | 1.870173 | 0.786925 | 1.7472 | 0.107407 | 1.322742 | 1.200655 | 1.70752 |
| Q9Z2K1 | Q9Z2K1 | K1C16_MC | Krt16 | Keratin, type I cytoskeletal 16 | | + | | 0.451614 | 0.539427 | 1.198426 | 1.072125 | 0.512599 | 3.213834 | 0.338833 | 0.617543 | 0.911337 | 4.976137 | 4.123125 | 4.410442 | 4.091729 | 4.116108 | 5.471269 | 3.866348 | 4.154059 | 3.745198 | 3.731776 | 4.183702 | 4.16859 |
| Q9QWL7 | Q9QWL7;C | K1C17_MC | Krt17 | Keratin, type I cytoskeletal 17 | | | | 0.13586 | 0.197264 | 0.39439 | 0.807034 | 0.258276 | 2.225957 | 0.138929 | 0.300761 | 0.402556 | 8.663025 | 7.971418 | 7.457607 | 7.643194 | 7.491196 | 8.781946 | 7.758575 | 8.481339 | 7.222468 | 7.361782 | 7.45939 | 8.21223 |
| Q8BIW1 | Q8BIW1 | PRUN1_M | Prune1 | Exopolyphosphatase PRUNE1 | + | | | 1.418075 | 0.787092 | 4.969517 | 0.491522 | -0.64266 | -1.30279 | 0.266329 | 0.147771 | 0.729446 | 2.215034 | 2.361939 | 3.022559 | 2.858058 | 2.518661 | 2.128438 | 1.641716 | 1.361072 | 3.096578 | 4.069365 | 2.121279 | 2.230277 |
| Q3TTY5 | Q3TTY5 | K22E_MOI | Krt2 | Keratin, type II cytoskeletal 2 epidermal | | + | | 0.476876 | 0.625648 | 1.264268 | 1.84507 | 0.628388 | 8.276421 | 0.514912 | 0.462291 | 1.364884 | 8.14699 | 7.231599 | 6.334608 | 6.465797 | 6.430294 | 6.932973 | 6.875469 | 7.251825 | 5.81005 | 5.733579 | 5.992299 | 6.446385 |
| Q99PS0 | Q99PS0 | K1C23_MC | Krt23 | Keratin, type I cytoskeletal 23 | | | | 0.871971 | 2.622506 | 2.445977 | 0.351506 | -0.43079 | -0.94321 | 0.029216 | -0.08817 | -0.0922 | 0.432756 | -0.94515 | -1.3161 | -1.50664 | -1.45973 | -1.49005 | -3.70022 | -2.05718 | -1.42726 | -0.53389 | -2.34281 | -0.43063 |
| Q8VCW2 | Q8VCW2 | K1C25_MC | Krt25 | Keratin, type I cytoskeletal 25 | | | + | 0.848846 | 1.103491 | 2.366172 | 0.076404 | 0.290579 | 0.231168 | 1.118807 | 1.12449 | 3.415577 | 1.687756 | 2.602818 | -0.13492 | 1.011908 | 1.209223 | 1.126357 | 0.951474 | 1.132119 | 1.26651 | -0.97068 | 0.369906 | -0.28331 |
| Q9Z320 | Q9Z320 | K1C27_MC | Krt27 | Keratin, type I cytoskeletal 27 | | | | 0.899258 | 1.129164 | 2.542318 | 0.223888 | -0.60856 | -0.62239 | 0.387246 | 0.783422 | 1.033496 | 0.762069 | 1.446847 | -1.32329 | 0.026079 | 0.337018 | -0.04434 | -0.30761 | 0.258202 | 0.667676 | -0.74776 | 0.096577 | -1.37074 |
| Q9D646 | Q9D646;Q | KRT34_MC | Krt34 | Keratin, type I cuticular Ha4 | | | | 0.151525 | -0.85607 | -0.43587 | 0.039104 | -0.2533 | -0.12223 | 0.31057 | -1.96936 | -0.84041 | -3.63362 | -1.60908 | -3.44199 | -0.93026 | -3.19009 | -3.63102 | -0.08217 | -3.44837 | -0.28432 | -3.58134 | -3.77413 | 0.891723 |
| Q497I4 | Q9D646;Q | KRT35_MC | Krt35 | Keratin, type I cuticular Ha5 | | | + | 0.007477 | 0.041278 | 0.024144 | 0.052137 | -0.13741 | -0.16101 | 2.122577 | -5.37682 | -11.4504 | 1.957496 | 1.652525 | 3.120828 | 1.414412 | -3.4353 | -3.55283 | 3.466588 | 0.060878 | 2.386302 | 2.423761 | 1.416872 | 2.348638 |
| B1AQ75 | B1AQ75;Q | KRT36_MC | Krt36 | Keratin, type I cuticular Ha6 | | | | 0.048534 | 0.479341 | 0.150376 | 0.587581 | -2.03628 | -1.56303 | 0.434583 | -2.29859 | -1.15442 | -3.44239 | 1.937637 | -3.25603 | -3.91728 | -3.50097 | -3.26578 | 0.478464 | -2.9419 | -0.29025 | -2.8105 | -3.07242 | 0.902845 |
| P07744 | P07744 | K2C4_MOI | Krt4 | Keratin, type II cytoskeletal 4 | | | | 0.049914 | -0.48889 | -0.15446 | 0.133922 | -0.4216 | -0.38922 | 0.330164 | -1.25676 | -0.88956 | 0.251894 | -3.16154 | 0.766171 | -1.3718 | -3.0886 | -3.33235 | 1.699752 | -3.63161 | 0.293722 | -0.05614 | -0.5462 | -3.36123 |
| Q6IFX2 | Q6IFX2 | K1C42_MC | Krt42 | Keratin, type I cytoskeletal 42 | | | | 0.009038 | 0.020067 | 0.029134 | 0.845739 | 0.506667 | 2.355576 | 0.160394 | 0.324355 | 0.459143 | 5.077063 | 4.34442 | 4.140102 | 4.138906 | 3.8557 | 5.099111 | 4.107356 | 5.273992 | 3.417745 | 3.847929 | 3.817588 | 4.488512 |
| Q922U2 | Q922U2 | K2C5_MOI | Krt5 | Keratin, type II cytoskeletal 5 | | | | 0.327097 | 0.410908 | 0.881864 | 0.412391 | 0.208414 | 1.097505 | 0.34296 | 0.393055 | 0.921709 | 8.767426 | 7.924318 | 7.077325 | 7.148451 | 7.215969 | 7.907863 | 7.736458 | 8.133471 | 6.717936 | 7.091012 | 6.919517 | 7.418206 |
| P50446 | P50446;Q! | K2C6A_MC | Krt6a | Keratin, type II cytoskeletal 6A | | + | | 0.179457 | -0.29501 | -0.50871 | 2.13856 | 0.87384 | 11.66547 | 0.074195 | 0.239675 | 0.224884 | 4.251746 | 3.909858 | 4.085295 | 4.15525 | 3.629428 | 5.509784 | 3.82166 | 4.929967 | 3.312673 | 3.180192 | 3.828005 | 4.831858 |
| Q9R0H5 | Q9R0H5;C | K2C71_MC | Krt71 | Keratin, type II cytoskeletal 71 | | | + | 0.879764 | 0.837388 | 2.473248 | 0.063777 | -0.27659 | -0.19498 | 1.814408 | 0.795832 | 7.982975 | 2.726964 | 3.334466 | 0.2272 | 2.156455 | 2.08681 | 1.987915 | 2.043757 | 2.342896 | 2.508511 | 0.428317 | 1.328094 | 1.154966 |
| Q6IME9 | Q6IME9 | K2C72_MC | Krt72 | Keratin, type II cytoskeletal 72 | | + | | 0.383227 | 0.525572 | 1.023307 | 1.507204 | 0.486548 | 5.537111 | 0.451625 | 0.271767 | 1.198455 | 3.640686 | 2.676274 | 1.667248 | 1.788338 | 1.650285 | 1.973195 | 2.456091 | 2.809724 | 1.177562 | 1.304928 | 1.380742 | 1.699203 |
| O88455 | O88455 | DHCR7_M | Dhcr7 | 7-dehydrocholesterol reductase | + | + | | 1.794517 | 0.780795 | 7.797965 | 1.525709 | 0.539698 | 5.662054 | 0.358492 | 0.416012 | 0.960806 | 3.358491 | 3.162786 | 1.331159 | 1.496586 | 2.157253 | 1.742953 | 2.501068 | 2.45862 | 0.921546 | 0.826803 | 1.153878 | 1.914305 |
| Q3UV17 | Q3UV17 | K22O_MOI | Krt76 | Keratin, type II cytoskeletal 2 oral | | + | | 0.1326 | 0.209944 | 0.38569 | 1.81278 | 0.72341 | 7.967678 | 0.565301 | 0.339388 | 1.501366 | 7.147767 | 6.205085 | 4.752353 | 4.633677 | 5.132696 | 5.566005 | 6.194201 | 6.738763 | 3.900885 | 4.038324 | 4.94546 | 5.074466 |
| Q6IFZ6 | Q6IFZ6 | K2C1B_MC | Krt77 | Keratin, type II cytoskeletal 1b | | | | 0.197059 | 0.35665 | 0.554011 | 0.645826 | -0.42159 | -1.72853 | 0.181593 | 0.299021 | 0.514231 | 1.720778 | 0.442132 | -0.57043 | -0.3793 | 0.182199 | -0.01942 | 0.800261 | 0.64935 | 0.171131 | -0.27767 | -0.79032 | 0.355055 |
| E9Q0F0 | E9Q0F0 | E9Q0F0_I | Krt78 | Keratin 78 | | | | 0.608994 | 0.404555 | 1.623118 | 0.928554 | 0.537493 | 2.648472 | 0.169187 | 0.353176 | 0.482078 | 7.530953 | 7.296478 | 6.145698 | 6.50654 | 6.228025 | 7.419366 | 6.789209 | 7.229112 | 5.695702 | 5.881549 | 6.044028 | 6.897012 |
| Q8VED5 | Q8VED5 | K2C79_MC | Krt79 | Keratin, type II cytoskeletal 79 | + | | | 1.18798 | -6.28476 | -3.7327 | NaN | 0 | NaN | NaN | 0 | NaN | -3.75174 | -3.41348 | -2.99031 | -3.09612 | -4.0404 | -3.41661 | 4.377344 | 1.026968 | -3.51946 | -2.93547 | -3.11953 | -3.68785 |
| Q91ZU6 | Q91ZU6 | DYST_MOI | Dst | Dystonin | + | | | 1.462446 | 0.776825 | 5.245185 | 0.912683 | 0.454773 | 2.590604 | 0.00955 | 0.00621 | 0.030766 | -0.13696 | -0.0355 | -1.02468 | -0.86915 | -0.90646 | -1.02163 | -1.0022 | -0.72392 | -1.2443 | -1.55907 | -0.77681 | -1.1637 |
| Q0VBK2 | Q0VBK2 | K2C80_MC | Krt80 | Keratin, type II cytoskeletal 80 | | | | 0.199542 | 0.208791 | 0.56037 | 0.070355 | 0.12315 | 0.213912 | 0.270052 | 0.259203 | 0.738798 | 0.808331 | 0.078671 | -0.71915 | -0.6045 | -0.70418 | -0.2269 | 0.310375 | 0.159046 | -1.35782 | -0.21213 | -0.98193 | -0.46756 |
| Q99M74 | Q99M74 | KRT82_MC | Krt82 | Keratin, type II cuticular Hb2 | | | + | 0.148723 | -1.01212 | -0.42849 | NaN | 0 | NaN | 1.26707 | -1.15506 | -4.12426 | -3.4713 | -0.52908 | -3.43933 | -2.8071 | -2.51448 | -3.06094 | 0.859953 | -2.83609 | -3.58283 | -4.01453 | -1.57114 | -1.69415 |
| Q99M73 | Q99M73 | KRT84_MC | Krt84 | Keratin, type II cuticular Hb4 | | | | 0.274188 | 1.743356 | 0.749182 | NaN | 0 | NaN | 0.341867 | -2.14292 | -0.91896 | -3.45534 | 1.138211 | -3.40547 | -3.75746 | -2.76134 | -3.80977 | -2.528 | -3.27584 | -3.22401 | -4.06285 | -3.41485 | 1.129573 |
| Q9Z2T6 | Q9ERE2;P | KRT85_MC | Krt85 | Keratin, type II cuticular Hb5 | | | | 0.074896 | -0.64988 | -0.22688 | 0.441608 | -2.23778 | -1.17254 | 0.457756 | -2.2007 | -1.21437 | -3.31496 | 0.470636 | -3.22395 | -3.76137 | -3.51667 | -0.50171 | 1.377645 | -2.92221 | 0.634591 | -3.14436 | -0.69304 | 0.716052 |
| E9Q1Z0 | E9Q1Z0 | E9Q1Z0_M | Krt90 | Keratin 90 | | | | 0.165846 | -1.84939 | -0.47338 | 0.347215 | -1.66181 | -0.93241 | NaN | 0 | NaN | 1.395174 | -3.50317 | -3.75961 | -3.27618 | -2.63523 | -3.37358 | -2.24838 | 3.839157 | -3.62189 | -0.09028 | -3.22648 | -3.55183 |
| Q61595 | Q61595 | KTN1_MOI | Ktn1 | Kinectin | | | | 0.028115 | -0.05814 | -0.08883 | 0.235993 | 0.045167 | 0.653031 | 0.612452 | 0.190498 | 1.632902 | -1.99903 | -1.32439 | -1.41137 | -1.31578 | -1.76896 | -1.90096 | -2.16454 | -1.04258 | -1.35875 | -1.45874 | -1.92926 | -2.12166 |
| P11627 | P11627 | L1CAM_M( | L1cam | Neural cell adhesion molecule L1 | | | | 0.101748 | 0.126683 | 0.302037 | 0.794217 | -0.69269 | -2.18398 | 0.506444 | -0.50909 | -1.34232 | -3.17451 | -3.53535 | -1.91147 | -2.50493 | -1.86497 | -2.38808 | -3.86025 | -3.10297 | -1.40348 | -1.62753 | -1.3428 | -1.89208 |
| Q9EP89 | Q9EP89 | LACTB_MC | Lactb | Serine beta-lactamase-like protein LACTB, mitochondrial | | | | 0.191751 | -0.75003 | -0.54039 | 0.088001 | 0.026578 | 0.263871 | 0.124147 | 0.10497 | 0.363019 | -3.45091 | -0.67676 | -0.0604 | 0.019151 | -0.94524 | -0.44187 | -1.36257 | -1.26504 | 0.045333 | -0.13974 | -0.65616 | -0.94089 |
| P19137 | P19137 | LAMA1_M( | Lama1 | Laminin subunit alpha-1 | | | | 0.117344 | 0.152993 | 0.34464 | 0.019654 | -0.01867 | -0.06264 | 0.311983 | 2.765858 | 0.843955 | 2.474123 | 1.597973 | 3.904223 | 3.69892 | 2.860229 | 3.013167 | 1.954859 | 1.811252 | 4.100081 | 3.540402 | -3.10552 | 3.447204 |
| P11438 | P11438 | LAMP1_M( | Lamp1 | Lysosome-associated membrane glycoprotein 1 | | | + | 0.117233 | -0.14677 | -0.34434 | 0.089185 | -0.34122 | -0.26718 | 1.466492 | 0.83194 | 5.270993 | -1.86017 | -1.14005 | -1.17445 | -3.53499 | -1.27358 | -1.15513 | -1.12525 | -1.58143 | -2.50137 | -1.52562 | -1.9 | -2.1926 |
| O89112 | O89112 | LANC1_M( | Lancl1 | Glutathione S-transferase LANCL1 | | | | 0.335209 | -0.1858 | -0.90223 | 0.995087 | -0.36812 | -2.90071 | 0.137254 | -0.17504 | -0.3981 | 1.297875 | 1.69999 | 0.918398 | 0.84863 | 1.231664 | 1.951165 | 1.729258 | 1.640199 | 1.373659 | 1.129619 | 2.019239 | 1.513666 |
| Q9CRB9 | Q9CRB9 | MIC19_MC | Chchd3 | MICOS complex subunit Mic19 | + | | | 2.996818 | 0.77543 | 31.48332 | 0.738759 | 0.343948 | 2.00747 | 0.837834 | 0.166857 | 2.328745 | 4.390886 | 4.343184 | 3.443565 | 3.785484 | 3.631204 | 3.662924 | 3.58546 | 3.59775 | 3.281897 | 3.259256 | 3.55008 | 3.410333 |
| Q9CZ04 | Q9CZ04 | CSN7A_M( | Cops7a | COP9 signalosome complex subunit 7a | + | | | 1.423052 | 0.774235 | 4.999777 | 0.115887 | -0.08552 | -0.34069 | 0.205001 | 0.217156 | 0.574327 | -1.15177 | -1.27435 | -0.94883 | -1.36924 | -0.47373 | -1.20459 | -2.12951 | -1.84509 | -1.2107 | -0.93634 | -0.95924 | -1.15339 |
| Q9CPY7 | Q9CPY7 | AMPL_MO | Lap3 | Cytosol aminopeptidase | | | | NaN | 0 | NaN | 0.589166 | -0.97444 | -1.56745 | 0.204957 | -0.43762 | -0.57421 | -3.3577 | -3.90023 | -4.45083 | -3.43473 | -5.09462 | -3.67658 | -3.00964 | -3.49779 | -3.32661 | -2.61006 | -3.66849 | -4.22748 |
| Q9Z1M7 | Q9Z1M7 | LARG1_MC | Large1 | Xylosyl- and glucuronyltransferase LARGE1 | | | | NaN | 0 | NaN | 0.778699 | 1.183391 | 2.133768 | 0.144157 | -0.587 | -0.41642 | -3.08106 | -3.36725 | -1.92979 | -1.70295 | -1.76714 | -4.37388 | -3.58151 | -3.94355 | -3.54264 | -2.45688 | -3.02039 | -1.94664 |
| Q6ZQ58 | Q6ZQ58 | LARP1_MC | Larp1 | La-related protein 1 | | | | 0.354905 | 1.170238 | 0.951766 | 0.266988 | 0.135587 | 0.731103 | 0.428098 | 0.331287 | 1.137746 | -0.97604 | -1.36026 | -0.59963 | -0.43725 | -0.75218 | -1.05074 | -1.12395 | -3.55283 | -0.82076 | -0.48729 | -1.48275 | -0.98275 |
| Q6A0A2 | Q6A0A2 | LAR4B_MC | Larp4b | La-related protein 4B | | | | NaN | 0 | NaN | 0.535303 | -0.72959 | -1.41964 | 0.50783 | -0.46175 | -1.346 | -3.09259 | -3.11769 | -2.425 | -3.09763 | -3.18908 | -3.05437 | -3.31761 | -3.37547 | -1.64313 | -2.42033 | -2.99635 | -2.3236 |
| Q8BMJ2 | Q8BMJ2 | SYLC_MOI | Lars1 | Leucine--tRNA ligase, cytoplasmic | | | | 0.154411 | -0.2286 | -0.44346 | 0.117791 | -0.07291 | -0.34585 | 0.320076 | 0.54119 | 0.86425 | -1.05087 | -1.50526 | 0.088254 | -0.12534 | 0.439605 | -0.42744 | -0.58673 | -1.5122 | -0.12738 | 0.236104 | -0.98697 | -0.08324 |
| Q8VDC0 | Q8VDC0 | SYLM_MOI | Lars2 | Probable leucine--tRNA ligase, mitochondrial | | | | 0.003101 | 0.020555 | 0.010061 | 0.086782 | -0.09728 | -0.26046 | 0.308015 | 0.71577 | 0.834007 | -3.62616 | -0.623 | -2.12606 | -1.95447 | -1.59734 | -1.04234 | -0.75973 | -3.53054 | -2.30649 | -1.57948 | -2.84774 | -1.22348 |
| A2BE28 | A2BE28 | LAS1L_MC | Las1l | Ribosomal biogenesis protein LAS1L | | | | NaN | 0 | NaN | 0.402175 | 0.293946 | 1.071441 | 0.272884 | -0.4949 | -0.74591 | -2.6954 | -3.96994 | -3.01849 | -3.54257 | -4.25566 | -4.20052 | -3.25498 | -3.83961 | -3.65573 | -3.49323 | -4.3961 | -3.07028 |
| Q61792 | Q61792 | LASP1_MC | Lasp1 | LIM and SH3 domain protein 1 | | | | 0.15625 | -0.18283 | -0.44829 | 0.038303 | 0.07754 | 0.119813 | 0.364048 | -0.35537 | -0.97482 | -0.91142 | -0.27312 | 0.685923 | -0.52791 | -0.4661 | -0.23552 | -0.66336 | -0.15551 | -0.22323 | 0.226158 | 0.350401 | -0.34128 |
| Q3U9G9 | Q3U9G9 | LBR_MOU! | Lbr | Delta(14)-sterol reductase LBR | | | | 0.075436 | 0.1742 | 0.228418 | 0.03907 | 0.060695 | 0.122123 | 0.172475 | 0.275016 | 0.490623 | -2.20711 | -3.37271 | -3.87471 | -3.31229 | -2.6745 | -2.49369 | -3.456 | -2.47221 | -3.24441 | -4.06398 | -2.30591 | -3.41232 |
| Q3UN02 | Q3UN02 | LCLT1_MC | Lclat1 | Lysocardiolipin acyltransferase 1 | | | | 0.113174 | 0.238635 | 0.333316 | 0.19842 | -0.70617 | -0.5575 | 0.178535 | -0.65557 | -0.50633 | -3.84011 | -2.62238 | -3.14624 | -3.35334 | -3.57113 | -1.00437 | -3.09325 | -3.84651 | -3.80606 | -1.2812 | -1.80342 | -1.46095 |
| A2RTH5 | A2RTH5 | A2RTH5_I | Lcmt1 | Leucine carboxyl methyltransferase 1 | | | | NaN | 0 | NaN | 0.456837 | 1.781687 | 1.21198 | 0.13602 | 0.148165 | 0.394815 | -3.93121 | -2.70589 | -1.06432 | -0.84108 | -1.77675 | -1.22611 | -3.24238 | -3.38137 | -4.20021 | -1.26857 | -1.9046 | -1.39459 |
| P06151 | P06151 | LDHA_MO | Ldha | L-lactate dehydrogenase A chain | | | | 0.347566 | -0.13259 | -0.93329 | 0.560958 | -0.43269 | -1.48945 | 0.054016 | -0.04808 | -0.16654 | 2.858414 | 3.141067 | 4.054888 | 3.54105 | 4.138613 | 4.585694 | 3.1178 | 3.146871 | 4.366256 | 4.095067 | 4.59291 | 4.227552 |
| Q9D0R8 | Q9D0R8 | LSM12_MC | Lsm12 | Protein LSM12 | + | | + | 2.472311 | 0.772284 | 17.18128 | 0.143294 | 0.383177 | 0.414139 | 1.033985 | 0.706393 | 3.055849 | -2.07212 | -1.99045 | -0.75235 | -0.86738 | -0.53417 | -0.1049 | -2.82236 | -2.78478 | -2.11648 | -0.26959 | -1.11176 | -0.94009 |
| Q9Z2I0 | Q9Z2I0 | LETM1_M( | Letm1 | Mitochondrial proton/calcium exchanger protein | | | | 0.176975 | 0.212631 | 0.502288 | 0.671923 | 0.819136 | 1.8049 | 0.142014 | 0.173152 | 0.410748 | -0.65502 | 0.191206 | 0.198526 | 0.461556 | -0.04758 | -0.57296 | -0.43117 | -0.4579 | -0.92346 | -0.05473 | -0.15372 | -0.81312 |
| Q924L1 | Q924L1 | LTMD1_M( | Letmd1 | LETM1 domain-containing protein 1 | + | | | 1.227793 | -1.0084 | -3.92576 | 0.223237 | -0.12351 | -0.62074 | 0.018832 | -0.02191 | -0.06007 | -1.12273 | -1.18475 | -0.61081 | -0.86111 | -0.69659 | -0.10331 | -0.40033 | 0.10965 | -0.76714 | -0.45775 | -0.16572 | -0.59036 |
| P16045 | P16045 | LEG1_MOI | Lgals1 | Galectin-1 | | | | 0.232253 | -0.76272 | -0.64357 | 0.149997 | 0.617853 | 0.431847 | 0.345891 | 0.521637 | 0.929079 | -2.23493 | -4.50456 | -1.42074 | -3.9881 | -2.5267 | -1.9051 | -2.26539 | -2.94866 | -3.95404 | -2.6905 | -2.26995 | -3.20512 |
| Q8VED9 | Q8VED9 | LEGL_MOI | Lgalsl | Galectin-related protein | | | | 0.064317 | 0.24161 | 0.19654 | 0.212769 | -0.51617 | -0.59413 | 0.160861 | -0.32698 | -0.46036 | -1.09333 | -2.35603 | -0.35673 | -1.99986 | -1.8265 | -0.6376 | -0.91148 | -3.0211 | -0.94463 | -0.37961 | -0.51637 | -1.29378 |
| Q9JIA1 | Q9JIA1 | LGI1_MOU | Lgi1 | Leucine-rich glioma-inactivated protein 1 | | | | 0.830584 | 0.353182 | 2.3043 | 0.207041 | -0.13301 | -0.57954 | 0.226705 | -0.15589 | -0.62953 | -0.0628 | 0.163764 | 1.493089 | 1.105417 | 1.45707 | 1.242686 | -0.40594 | -0.19946 | 1.309371 | 1.555154 | 1.282544 | 1.728983 |
| Q8K406 | Q8K406 | LGI3_MOU | Lgi3 | Leucine-rich repeat LGI family member 3 | | | | 0.086615 | 0.038774 | 0.259989 | 0.097105 | 0.102442 | 0.289215 | 0.334616 | -0.29811 | -0.90074 | 1.756732 | 1.627353 | -0.11946 | -0.0595 | -0.47802 | -0.75831 | 1.787646 | 1.518891 | 0.161016 | -0.54486 | -0.61988 | -0.02023 |
| Q99JW4 | Q99JW4 | LIMS1_MO | Lims1 | LIM and senescent cell antigen-like-containing domain protein 1 | | | | 0.4691 | 0.528166 | 1.243924 | 0.119984 | 0.065771 | 0.351787 | 0.874966 | 0.285636 | 2.456434 | -0.57485 | -1.23356 | -1.01689 | -1.00498 | -0.8605 | -0.65631 | -1.70034 | -1.16441 | -0.88984 | -1.26358 | -1.0997 | -0.98838 |
| Q8JZS0 | Q8JZS0 | LIN7A_MO | Lin7a | Protein lin-7 homolog A | | + | | 0.467218 | 0.539187 | 1.239011 | 1.54983 | -0.31933 | -5.82877 | 0.311087 | -0.09387 | -0.84171 | 1.087045 | 1.55515 | 2.257676 | 2.155063 | 2.058896 | 1.893061 | 0.415035 | 1.148785 | 2.506486 | 2.544904 | 1.995269 | 2.144428 |
| P84075 | P62748;P8 | HPCA_MO | Hpca | Neuron-specific calcium-binding protein hippocalcin | + | | | 1.882037 | 0.771703 | 8.643937 | 0.0807 | 0.020884 | 0.243341 | 0.395778 | -0.17396 | -1.05516 | 3.068158 | 2.970731 | 3.662139 | 3.580466 | 4.403795 | 4.696423 | 2.322557 | 2.172926 | 3.524936 | 3.675902 | 4.800049 | 4.648089 |
| O88952 | O88952 | LIN7C_MO | Lin7c | Protein lin-7 homolog C | | + | | 0.153432 | 0.047639 | 0.440888 | 1.182713 | -0.43761 | -3.70775 | 0.009207 | 0.004733 | 0.029673 | 1.368178 | 1.583504 | 1.324367 | 1.559805 | 1.538311 | 1.851462 | 1.419049 | 1.437356 | 1.8712 | 1.888187 | 1.720654 | 1.659653 |
| Q9D1T0 | Q9D1T0 | LIGO1_MC | Lingo1 | Leucine-rich repeat and immunoglobulin-like domain-containing nogo receptor-interacting protein 1 | | | | NaN | 0 | NaN | 0.032801 | 0.049779 | 0.10315 | 0.631008 | 0.463647 | 1.685798 | -3.44667 | -3.82777 | -0.93521 | -1.156 | -0.4233 | -0.94863 | -3.33469 | -3.43849 | -1.56518 | -0.62559 | -1.06808 | -1.23115 |
| Q80Y17 | Q80Y17 | L2GL1_MC | Llgl1 | Lethal(2) giant larvae protein homolog 1 | | | | 0.540841 | 0.252243 | 1.434624 | 0.822378 | -0.14632 | -2.27682 | 0.131239 | -0.10169 | -0.38205 | 0.57339 | 0.221836 | -0.38515 | -0.25813 | -0.36314 | -0.18901 | 0.141279 | 0.149461 | -0.1655 | -0.18514 | -0.42591 | 0.077144 |
| Q8C561 | Q8C561 | LMBD2_M( | Lmbrd2 | G-protein coupled receptor-associated protein LMBRD2 | | + | + | 0.827165 | 1.804225 | 2.292828 | 1.017156 | 0.902157 | 2.988006 | 2.902267 | 0.298882 | 28.23093 | -0.35308 | -1.26236 | -0.52498 | -0.83322 | -0.82177 | -0.80706 | -1.96968 | -3.25421 | -1.84089 | -1.32163 | -1.12091 | -1.10568 |
| P48678 | P48678 | LMNA_MO | Lmna | Prelamin-A/C | | | | 0.482637 | 0.358283 | 1.279388 | 0.128054 | -0.24678 | -0.37352 | 0.580978 | 0.667551 | 1.544662 | 0.80057 | 0.542425 | 0.09071 | 0.533296 | 0.876639 | 0.121923 | 0.561738 | 0.06469 | -0.06375 | 1.18131 | 0.042369 | -0.37891 |
| P14733 | P14733 | LMNB1_M( | Lmnb1 | Lamin-B1 | | | | 0.263368 | 0.582739 | 0.722005 | 0.194353 | 0.245566 | 0.547074 | 0.461037 | 0.443053 | 1.222901 | -0.20532 | 0.437974 | -0.96945 | -0.25291 | 0.122145 | -0.58773 | 0.27384 | -1.20666 | -0.58632 | -1.12717 | -0.6032 | -0.74849 |
| E9PYF4 | E9PYF4 | E9PYF4_M | Lmo7 | LIM domain only 7 | | | | NaN | 0 | NaN | 0.377316 | -0.58768 | -1.00834 | 0.327512 | -0.42969 | -0.88291 | -3.40476 | -4.25999 | -3.02984 | -3.10289 | -3.14819 | -3.74752 | -2.86404 | -4.10461 | -3.06036 | -1.89701 | -2.63469 | -3.40164 |
| Q8C129 | Q8C129 | LCAP_MOI | Lnpep | Leucyl-cystinyl aminopeptidase | | | | 0.501032 | 0.510884 | 1.327947 | 0.04252 | 0.034165 | 0.132474 | 0.406018 | 0.367198 | 1.081235 | -1.28991 | -1.90205 | -2.02476 | -2.49162 | -1.18215 | -1.6293 | -1.87378 | -2.33995 | -2.40199 | -2.18272 | -2.02856 | -1.51729 |
| Q7TQ95 | Q7TQ95 | LNP_MOU | Lnpk | Endoplasmic reticulum junction formation protein lunapark | | + | | 0.231432 | -0.21874 | -0.6415 | 1.148192 | 0.363311 | 3.547552 | 0.326429 | 0.381614 | 0.880189 | 0.63492 | 1.082637 | 0.682456 | 0.795076 | 0.575769 | 0.652466 | 1.33474 | 0.820304 | 0.289914 | 0.460996 | 0.664364 | -0.19936 |
| Q8CGK3 | Q8CGK3 | LONM_MC | Lonp1 | Lon protease homolog, mitochondrial | | | | 0.444282 | 0.171189 | 1.179447 | 0.262911 | 0.123554 | 0.720856 | 0.147493 | 0.271051 | 0.425243 | -0.59231 | -0.88241 | -0.5971 | -0.869 | 0.33454 | -0.93265 | -0.9033 | -0.9138 | -0.96099 | -0.75222 | -0.50056 | -0.63965 |
| Q3TFD2 | Q3TFD2 | PCAT1_MC | Lpcat1 | Lysophosphatidylcholine acyltransferase 1 | | | | 0.565793 | -0.64825 | -1.50272 | 0.078575 | -0.11641 | -0.23733 | 0.019646 | 0.035642 | 0.06261 | -3.53616 | -3.42375 | -3.09114 | -2.15026 | -2.03247 | -2.76184 | -3.25941 | -2.40399 | -2.64312 | -2.36547 | -1.99568 | -2.86991 |
| Q91V01 | Q91V01 | MBOA5_M | Lpcat3 | Lysophospholipid acyltransferase 5 | | | | 0.752163 | 0.261385 | 2.049396 | 0.748511 | 0.295885 | 2.037927 | 0.377915 | -0.24644 | -1.00985 | 0.26542 | 0.432875 | -0.62293 | -0.74613 | 0.019768 | -0.24633 | 0.183975 | -0.00845 | -1.11189 | -0.84894 | 0.337745 | -0.07142 |
| Q6NVG1 | Q6NVG1 | LPCT4_MC | Lpcat4 | Lysophospholipid acyltransferase LPCAT4 | | + | + | 0.783943 | 0.55938 | 2.150663 | 1.571922 | 0.44111 | 5.985405 | 1.005339 | 0.421795 | 2.941031 | -1.2679 | -1.03342 | -0.80908 | -0.93781 | -1.36993 | -1.60222 | -1.94221 | -1.47786 | -1.27867 | -1.35045 | -1.992 | -1.82374 |
| Q91YX5 | Q91YX5 | LGAT1_MC | Lpgat1 | Acyl-CoA:lysophosphatidylglycerol acyltransferase 1 | | | | 0.048069 | -0.05579 | -0.149 | 0.071489 | 0.023865 | 0.217159 | 0.113656 | 0.075702 | 0.334629 | 0.97685 | 1.707963 | 1.023087 | 0.897542 | 0.956752 | 0.883159 | 1.479185 | 1.317205 | 0.846248 | 1.026652 | 1.067469 | 0.621037 |
| Q99PI5 | Q99PI5 | LPIN2_MO | Lpin2 | Phosphatidate phosphatase LPIN2 | | | | 0.145862 | 0.52402 | 0.420933 | 0.136637 | -0.14343 | -0.39646 | 0.092136 | -0.08188 | -0.27542 | -1.72724 | -4.18141 | -0.79558 | -0.73042 | -0.81161 | -1.40579 | -3.26847 | -3.68822 | -0.97988 | -0.25926 | -1.03757 | -1.01607 |
| Q99N69 | Q99N69 | LPXN_MOI | Lpxn | Leupaxin | | | | NaN | 0 | NaN | 0.307984 | -0.45814 | -0.83393 | 0.463801 | -1.0133 | -1.2301 | -3.58164 | -4.05445 | -2.30035 | -3.26273 | -3.77461 | -2.23676 | -4.23571 | -3.64277 | -2.58849 | -2.05831 | -1.69687 | -2.28789 |
| P62046 | P62046 | LRCH1_M( | Lrch1 | Leucine-rich repeat and calponin homology domain-containing protein 1 | | | | 0.321774 | 0.746746 | 0.86851 | 0.138215 | -0.18413 | -0.40066 | 0.17315 | -0.8802 | -0.49237 | -1.69751 | -3.3923 | -1.40065 | -0.63848 | -1.99881 | -3.96556 | -3.43716 | -3.14614 | -1.09231 | -0.57855 | -3.59487 | -0.60909 |
| Q2WF71 | Q2WF71 | LRFN1_MC | Lrfn1 | Leucine-rich repeat and fibronectin type III domain-containing protein 1 | | | + | NaN | 0 | NaN | 0.600105 | 0.894554 | 1.598071 | 1.062488 | 1.252142 | 3.173348 | -2.82151 | -4.13216 | -2.58269 | -2.25719 | -2.39261 | -3.08471 | -3.01919 | -2.69664 | -3.85009 | -2.77891 | -4.18039 | -3.80122 |
| Q80TG9 | Q80TG9 | LRFN2_MC | Lrfn2 | Leucine-rich repeat and fibronectin type-III domain-containing protein 2 | | + | | NaN | 0 | NaN | 1.738362 | -0.46605 | -7.29738 | 0.362661 | 0.166885 | 0.971316 | -3.22353 | -3.09682 | -1.56195 | -1.47495 | -1.23865 | -1.55708 | -3.89436 | -3.96639 | -1.09916 | -1.00563 | -1.50016 | -1.62934 |
| Q91ZX7 | Q91ZX7 | LRP1_MOI | Lrp1 | Prolow-density lipoprotein receptor-related protein 1 | | + | | 0.39234 | 0.459471 | 1.046425 | 2.877024 | 0.096068 | 27.42074 | 0.090969 | 0.110346 | 0.272164 | -0.35862 | -0.34729 | 0.250312 | 0.243332 | 0.290645 | -0.27801 | -0.37337 | -1.25147 | 0.150449 | 0.151059 | -0.39306 | 0.185005 |
| Q6PB66 | Q6PB66 | LPPRC_MC | Lrpprc | Leucine-rich PPR motif-containing protein, mitochondrial | | | | 0.491997 | -0.38399 | -1.30404 | 0.227671 | 0.250497 | 0.631975 | 0.281451 | 0.520992 | 0.767407 | -1.25227 | -0.70224 | -0.57339 | -1.32226 | 0.456642 | -0.90018 | -0.69851 | -0.48802 | -1.06827 | -1.32837 | -0.71698 | -0.76853 |
| Q505F5 | Q505F5 | LRC47_MC | Lrrc47 | Leucine-rich repeat-containing protein 47 | | | + | 0.073631 | 0.088268 | 0.223277 | 0.349565 | 0.183928 | 0.938321 | 1.104248 | -0.33026 | -3.35164 | -0.90979 | -1.37957 | -0.29011 | -0.65486 | -0.89305 | -0.71831 | -0.91497 | -1.55093 | -0.72826 | -0.58457 | -0.42986 | -0.52098 |
| Q91YK0 | Q91YK0 | LRC49_MC | Lrrc49 | Leucine-rich repeat-containing protein 49 | | | | 0.202422 | 0.875265 | 0.567738 | 0.777781 | -0.27022 | -2.13082 | 0.013388 | -0.08101 | -0.04295 | -0.58163 | -3.53782 | -0.70181 | -0.89821 | -1.14276 | -3.38981 | -2.49683 | -3.37314 | -0.61004 | -0.44953 | -3.70023 | -0.67031 |
| P0C192 | P0C192 | LRC4B_MC | Lrrc4b | Leucine-rich repeat-containing protein 4B | | | | NaN | 0 | NaN | 0.326496 | 0.381838 | 0.880356 | 0.238191 | 0.414883 | 0.658586 | -3.33667 | -3.58452 | -1.06337 | -1.59856 | 0.168139 | -1.04628 | -2.83658 | -3.19351 | -2.05415 | -1.37146 | -0.68618 | -1.02172 |
| O88441 | O88441 | MTX2_MOI | Mtx2 | Metaxin-2 | + | | | 1.729533 | 0.769279 | 7.22151 | 0.638332 | 0.250835 | 1.706864 | 0.29812 | 0.19903 | 0.809201 | 1.464605 | 1.655377 | 0.883797 | 1.177495 | 1.333966 | 1.185815 | 0.838139 | 0.743285 | 0.78544 | 0.774182 | 0.826323 | 1.2954 |
| Q922Q8 | Q922Q8 | LRC59_MC | Lrrc59 | Leucine-rich repeat-containing protein 59 | | | | 0.002558 | 0.010434 | 0.008306 | 0.143375 | -0.78195 | -0.41435 | 0.189157 | -0.66535 | -0.53372 | -3.13291 | -0.62718 | -0.77821 | -4.39382 | -3.53064 | -1.13583 | -1.98299 | -1.79798 | -2.3455 | -1.26264 | -1.32106 | -2.0147 |
| Q80TE7 | Q80TE7 | LRRC7_MC | Lrrc7 | Leucine-rich repeat-containing protein 7 | | | | 0.645247 | 0.207812 | 1.726853 | 0.184946 | 0.185766 | 0.522882 | 0.565707 | -0.50305 | -1.50248 | 1.378245 | 1.138761 | 5.151094 | 5.763746 | 4.658262 | 5.32776 | 1.062689 | 1.038693 | 5.0917 | 5.451608 | 5.489573 | 5.502547 |
| B2RWC4 | B2RWC4 | B2RWC4_I | Lrrc73 | Gm88 protein | | | | NaN | 0 | NaN | 0.907975 | 0.798923 | 2.573601 | 0.401126 | 1.071968 | 1.068768 | -3.34174 | -3.91897 | -0.81114 | -0.67792 | -0.36324 | -1.82019 | -3.31454 | -3.3839 | -1.84665 | -1.24025 | -2.85312 | -1.47425 |
| Q80WG5 | Q80WG5 | LRC8A_MC | Lrrc8a | Volume-regulated anion channel subunit LRRC8A | | | | NaN | 0 | NaN | 0.090973 | 0.231037 | 0.272174 | 0.026427 | 0.10751 | 0.083634 | -3.40254 | -3.05308 | -2.01486 | -3.49144 | -1.65542 | -2.48801 | -2.80209 | -3.55922 | -3.40311 | -2.56527 | -3.39543 | -0.96302 |
| Q8K377 | Q8K377 | LRRT1_MC | Lrrtm1 | Leucine-rich repeat transmembrane neuronal protein 1 | | + | | NaN | 0 | NaN | 1.229152 | -0.33873 | -3.93249 | 0.006728 | 0.020199 | 0.021742 | -3.31924 | -2.7137 | -1.26049 | -1.16731 | 0.309854 | -1.50832 | -3.6899 | -3.45208 | -0.94762 | -0.80272 | -0.42798 | -0.81088 |
| Q8BZ81 | Q8BZ81 | LRRT3_MC | Lrrtm3 | Leucine-rich repeat transmembrane neuronal protein 3 | | | | NaN | 0 | NaN | 0.309703 | -0.44336 | -0.83824 | 0.261907 | -0.61811 | -0.71833 | -3.90651 | -2.98585 | -1.9427 | -1.83666 | -2.76383 | -3.15659 | -3.26379 | -3.69424 | -1.97257 | -0.92007 | -3.17987 | -1.50432 |
| Q80ZI6 | Q80ZI6 | LRSM1_MC | Lrsam1 | E3 ubiquitin-protein ligase LRSAM1 | | | | 0.316817 | -1.74155 | -0.85608 | 0.527222 | -1.76076 | -1.39787 | 0.334514 | 1.054189 | 0.900486 | -2.82135 | -3.29967 | -0.76248 | -3.22212 | -0.58402 | -1.16114 | -3.3392 | 0.701267 | 0.040773 | -0.50385 | -3.06134 | -0.7922 |
| Q8BLK3 | Q8BLK3 | LSAMP_M( | Lsamp | Limbic system-associated membrane protein | | | | 0.915072 | -0.82752 | -2.59926 | 0.468613 | -0.84585 | -1.24265 | 0.145359 | -0.16338 | -0.41961 | -1.04302 | -1.24608 | -1.55477 | -2.74703 | -0.83573 | -0.33157 | -0.01528 | -0.61877 | -0.97649 | -1.63361 | -0.12351 | -0.71703 |
| Q922S4 | Q922S4 | PDE2A_M( | Pde2a | cGMP-dependent 3',5'-cyclic phosphodiesterase | + | + | | 2.623899 | 0.766862 | 20.47266 | 1.023581 | 0.584196 | 3.013773 | 0.302874 | 0.29086 | 0.821118 | -0.42914 | -0.49389 | 2.268383 | 2.585769 | 2.117397 | 1.556273 | -1.20954 | -1.24721 | 1.731563 | 1.954195 | 1.329734 | 1.762216 |
| Q8K2F8 | Q8K2F8 | LS14A_MC | Lsm14a | Protein LSM14 homolog A | | | | 0.790903 | 1.086466 | 2.173203 | 0.61965 | 1.686519 | 1.653343 | 0.182754 | -0.28053 | -0.51723 | -1.79345 | -1.9493 | -1.97567 | -1.10091 | -2.30183 | -1.65996 | -2.46401 | -3.45167 | -4.14635 | -2.30327 | -2.1376 | -1.26312 |
| P62311 | P62311 | LSM3_MOI | Lsm3 | U6 snRNA-associated Sm-like protein LSm3 | | | | 0.66189 | 1.1873 | 1.775367 | 0.64144 | 0.452389 | 1.715835 | 0.001964 | -0.00508 | -0.00638 | -1.4957 | -2.14553 | -1.68249 | -1.33314 | -2.39574 | -3.26766 | -2.42339 | -3.59245 | -2.1577 | -1.76271 | -2.16048 | -3.49276 |
| Q7TSH2 | Q7TSH2 | KPBB_MOI | Phkb | Phosphorylase b kinase regulatory subunit beta | + | | | 1.30349 | 0.764272 | 4.315824 | 0.174745 | -0.07725 | -0.49651 | 0.613465 | -0.6275 | -1.63577 | 2.184372 | 1.841976 | 2.563966 | 2.450798 | 1.116203 | 1.724712 | 1.203618 | 1.294185 | 2.4397 | 2.729567 | 1.814321 | 2.281595 |
| O35945 | O35945 | AL1A7_MC | Aldh1a7 | Aldehyde dehydrogenase, cytosolic 1 | + | + | | 1.574873 | 0.761974 | 6.006623 | 1.095744 | 1.163251 | 3.314721 | 0.045685 | -0.05793 | -0.14192 | 6.415419 | 6.573583 | 7.336143 | 8.008858 | 4.998745 | 4.30178 | 5.633339 | 5.831716 | 6.409155 | 6.609344 | 4.495577 | 4.920817 |
| Q7TNC4 | Q7TNC4 | LC7L2_MC | Luc7l2 | Putative RNA-binding protein Luc7-like 2 | | | | 0.126094 | -0.15959 | -0.36826 | 0.566215 | 0.495957 | 1.50388 | 0.10109 | -0.2709 | -0.30022 | -3.83113 | -3.0352 | -3.67611 | -3.03357 | -4.48947 | -3.50914 | -3.10201 | -3.44514 | -3.77634 | -3.92525 | -2.9708 | -4.486 |
| Q9D6I9 | Q9D6I9 | LURA1_MC | Lurap1 | Leucine rich adaptor protein 1 | | | | 0.43786 | 0.420962 | 1.162869 | 0.675133 | -0.61663 | -1.81439 | 0.197533 | -0.39783 | -0.55523 | -0.24019 | 0.264192 | -0.14136 | 0.504551 | -1.52306 | -0.20372 | -0.14926 | -0.66866 | 0.904063 | 0.692391 | -0.74528 | -0.18583 |
| Q8K2P1 | Q8K2P1 | LUR1L_MC | Lurap1l | Leucine rich adaptor protein 1-like | + | | | 1.083545 | 2.006871 | 3.262315 | 0.352443 | 0.776734 | 0.945567 | 0.766092 | 0.651294 | 2.093453 | -1.84411 | -1.09516 | -1.4222 | -1.06214 | -0.64672 | -1.26877 | -2.98845 | -3.96456 | -1.21743 | -2.82038 | -1.61634 | -1.60174 |
| Q8R4U7 | Q8R4U7 | LUZP1_MC | Luzp1 | Leucine zipper protein 1 | | | | 0.269934 | 0.765681 | 0.738502 | 0.914201 | -0.51702 | -2.5961 | 0.507805 | -0.66323 | -1.34594 | -0.888 | -0.85978 | -0.35488 | -0.06176 | -0.72203 | 0.240222 | -0.60287 | -2.67628 | 0.173859 | 0.443547 | 0.315866 | 0.528782 |
| P70202 | P70202 | LXN_MOU! | Lxn | Latexin | | | | 0.341541 | -0.34198 | -0.91814 | 0.134972 | -0.3526 | -0.39202 | 0.610409 | -0.45116 | -1.62712 | -1.07026 | -0.42519 | -0.6441 | -2.35022 | -0.3485 | -0.45979 | -0.21946 | -0.59203 | -1.42969 | -0.85942 | 0.318643 | -0.22462 |
| Q8BP67 | Q8BP67 | RL24_MOI | Rpl24 | Large ribosomal subunit protein eL24 | + | | | 1.694035 | 0.760131 | 6.923882 | 0.392554 | -0.08766 | -1.04697 | 0.252364 | -0.4115 | -0.69432 | 1.70318 | 1.664142 | 2.64783 | 2.784511 | 2.217064 | 2.010259 | 1.031565 | 0.815495 | 2.852201 | 2.755458 | 1.941591 | 3.108723 |
| P97823 | P97823 | LYPA1_MC | Lypla1 | Acyl-protein thioesterase 1 | | | | 0.428351 | 0.100841 | 1.138394 | 0.016858 | -0.01784 | -0.05388 | 0.020683 | 0.010716 | 0.065844 | 1.425003 | 1.425429 | 0.275647 | 0.233473 | 0.375993 | 0.662323 | 1.412956 | 1.235793 | 0.602749 | -0.05796 | 0.585848 | 0.431035 |
| Q9WTL7 | Q9WTL7 | LYPA2_MC | Lypla2 | Acyl-protein thioesterase 2 | | | | 0.033442 | 0.00393 | 0.105101 | 0.71586 | -0.93908 | -1.93688 | 0.385898 | 0.194616 | 1.030075 | 3.05992 | 2.99158 | 1.473058 | 1.430404 | 2.871732 | 3.219042 | 3.006637 | 3.037004 | 2.87518 | 1.906436 | 2.776342 | 2.9252 |
| Q3UFF7 | Q3UFF7 | LYPL1_MC | Lyplal1 | Lysophospholipase-like protein 1 | | | | 0.261566 | 0.562365 | 0.717477 | 0.522639 | -0.48161 | -1.38556 | 0.011545 | -0.01298 | -0.03711 | -1.93741 | -2.07995 | -2.24589 | -2.37398 | -1.56711 | -0.91844 | -3.3516 | -1.79048 | -1.48668 | -2.16996 | -1.09913 | -1.36047 |
| Q8K215 | Q8K215 | LYRM4_MC | Lyrm4 | LYR motif-containing protein 4 | | | | 0.311346 | -0.33178 | -0.84236 | 0.876345 | -0.42537 | -2.46126 | 0.782496 | -0.30431 | -2.14599 | -0.80361 | -0.42665 | -0.61375 | -0.86943 | -0.49714 | -0.39471 | -0.62919 | 0.062506 | -0.19992 | -0.43253 | -0.27385 | -0.00938 |
| Q9D0E3 | Q9D0E3 | LYSM1_MC | Lysmd1 | LysM and putative peptidoglycan-binding domain-containing protein 1 | | + | | 0.055697 | -0.15242 | -0.17147 | 1.242055 | -0.63614 | -3.9969 | 0.175574 | -0.1373 | -0.49866 | -3.11193 | -1.59997 | -1.12056 | -0.91878 | -0.76995 | -0.35304 | -2.67115 | -1.73592 | -0.50663 | -0.26044 | -0.24431 | -0.60407 |
| Q9D7V2 | Q9D0E3;Q | LYSM2_MC | Lysmd2 | LysM and putative peptidoglycan-binding domain-containing protein 2 | | + | + | 0.900082 | 0.791856 | 2.545262 | 1.783472 | -0.17487 | -7.69699 | 1.395431 | -0.18691 | -4.83387 | -0.03557 | 0.04168 | -0.69661 | -0.7371 | -0.27899 | -0.20289 | -1.0975 | -0.4801 | -0.55229 | -0.53168 | -0.04715 | -0.0609 |
| Q9D3D9 | Q9D3D9 | ATPD_MOI | Atp5f1d | ATP synthase subunit delta, mitochondrial | + | | | 2.349944 | 0.759419 | 14.91122 | 0.460385 | 0.259794 | 1.221204 | 0.251286 | 0.153124 | 0.691611 | 4.128058 | 4.22244 | 3.573763 | 3.340372 | 3.463782 | 3.024144 | 3.434982 | 3.396678 | 3.375146 | 3.019401 | 3.064419 | 3.11726 |
| P17897 | P17897 | LYZ1_MOL | Lyz1 | Lysozyme C-1 | | | | 0.884335 | 0.797508 | 2.489334 | 0.876828 | -0.52335 | -2.46295 | 0.059775 | -0.11094 | -0.18337 | 0.601816 | -0.03852 | -1.2278 | -0.80319 | -0.34122 | -0.98754 | -0.52722 | -0.5045 | -0.50102 | -0.48326 | -1.06491 | -0.04198 |
| P60853 | P60853 | LZTS1_MO | Lzts1 | Leucine zipper putative tumor suppressor 1 | | | | 0.213559 | 0.880908 | 0.596145 | 0.539813 | 0.435377 | 1.431841 | 0.989544 | -0.44042 | -2.87908 | -0.90105 | -3.68835 | 0.310056 | 0.917204 | -0.67879 | -0.92417 | -2.68443 | -3.66678 | 0.16092 | 0.195586 | -0.45243 | -0.2697 |
| A2AHG0 | A2AHG0 | LZTS3_MO | Lzts3 | Leucine zipper putative tumor suppressor 3 | | + | | NaN | 0 | NaN | 1.579274 | -0.83091 | -6.03839 | 0.007734 | -0.0062 | -0.02497 | -3.78915 | -3.39255 | -0.75415 | -0.50497 | -0.49287 | -0.0102 | -3.50578 | -3.10721 | 0.25977 | 0.142927 | -0.18705 | -0.30361 |
| Q9QXZ0 | Q9QXZ0 | MACF1_M | Macf1 | Microtubule-actin cross-linking factor 1, isoforms 1/2/3/4 | | | | 0.33125 | 0.190368 | 0.892291 | 0.061127 | -0.01525 | -0.1873 | 0.409529 | -0.28061 | -1.09019 | -0.41174 | -0.76189 | -0.13454 | -0.17596 | -0.85041 | -0.58984 | -0.65526 | -0.89912 | -0.21874 | -0.06126 | -0.6615 | -0.21752 |
| Q9QZQ8 | Q9QZQ8 | H2AY_MOI | Macroh2a1 | Core histone macro-H2A.1 | | | | 0.373523 | 0.950387 | 0.998745 | 0.279955 | 0.841439 | 0.763652 | 0.374903 | 0.716856 | 1.002233 | 2.114218 | 1.555504 | 0.497742 | 2.378491 | 2.085102 | 0.678393 | 1.794127 | -0.02518 | 1.170956 | 0.022398 | 0.534942 | 0.794842 |
| Q8CCK0 | Q8CCK0 | H2AW_MC | Macroh2a2 | Core histone macro-H2A.2 | | | | 0.138205 | 0.447629 | 0.400633 | 0.44942 | 0.640422 | 1.192742 | 0.168415 | 0.312309 | 0.48007 | -3.35341 | -1.67658 | -3.37168 | -2.3064 | -2.13323 | -3.40312 | -2.22409 | -3.70116 | -3.5472 | -3.41173 | -2.93884 | -3.22212 |
| Q80U28 | Q80U28 | MADD_MC | Madd | MAP kinase-activating death domain protein | | | | 0.05392 | -0.04296 | -0.16626 | 0.637185 | 0.186727 | 1.703557 | 0.168636 | -0.14129 | -0.48064 | -0.27412 | -0.10969 | 1.761491 | 1.726526 | 1.173701 | 1.124143 | 0.096034 | -0.39392 | 1.449075 | 1.665489 | 0.997304 | 1.583111 |
| Q4VC33 | Q4VC33 | MAEA_MO | Maea | E3 ubiquitin-protein transferase MAEA | | | | 0.472893 | -0.87144 | -1.25384 | 0.758173 | 0.185495 | 2.068343 | 0.259128 | 0.406075 | 0.711346 | -3.46398 | -3.26798 | -0.98244 | -0.97211 | -1.0959 | -1.66652 | -1.80646 | -3.18261 | -1.2523 | -1.07323 | -2.28173 | -1.29285 |
| P20917 | P20917 | MAG_MOL | Mag | Myelin-associated glycoprotein | | | | 0.693486 | -0.41427 | -1.86912 | 0.033203 | -0.05946 | -0.10437 | 0.957847 | 0.604734 | 2.757565 | 3.807347 | 3.607419 | 1.762038 | 0.690929 | 2.56121 | 2.12785 | 4.319475 | 3.923838 | 1.09178 | 1.480104 | 1.773594 | 1.705998 |
| Q9QYH6 | Q9QYH6 | MAGD1_M | Maged1 | Melanoma-associated antigen D1 | | | | 0.35588 | 0.55773 | 0.954224 | 0.619019 | 0.435735 | 1.651546 | 0.09827 | -0.07785 | -0.29244 | 0.049015 | -0.68139 | 0.983764 | 0.757891 | 0.10586 | -0.15978 | -0.41757 | -1.33026 | 0.196651 | 0.673533 | -0.17982 | 0.281596 |
| Q6PCZ4 | Q6PCZ4 | MAGE1_M | Magee1 | Melanoma-associated antigen E1 | | | | NaN | 0 | NaN | 0.291591 | 0.181392 | 0.792835 | 0.809817 | -0.93446 | -2.23513 | -2.59377 | -4.04863 | -1.76113 | -1.52023 | -2.31363 | -3.07469 | -2.91352 | -3.44156 | -2.01659 | -1.62756 | -1.58654 | -1.93286 |
| Q6RHR9 | Q6RHR9 | MAGI1_M( | Magi1 | Membrane-associated guanylate kinase, WW and PDZ domain-containing protein 1 | | | | 0.575128 | -0.29409 | -1.52846 | 0.020468 | -0.01199 | -0.06517 | 0.578369 | -2.0629 | -1.53743 | -1.62331 | -2.00654 | -0.5594 | -0.22419 | -1.52002 | -4.1968 | -1.50337 | -1.53829 | -0.30386 | -0.45574 | -0.70013 | -0.8909 |
| Q9WVQ1 | Q9WVQ1 | MAGI2_M( | Magi2 | Membrane-associated guanylate kinase, WW and PDZ domain-containing protein 2 | | | | 0.268442 | 0.895886 | 0.734755 | 0.656452 | 0.238254 | 1.759451 | 0.777796 | -0.2798 | -2.13087 | -3.23961 | -1.26235 | -0.23502 | -0.01832 | -0.37832 | -0.37107 | -2.43322 | -3.86052 | -0.44614 | -0.2837 | -0.22615 | 0.036369 |
| Q9EQJ9 | Q9EQJ9 | MAGI3_M( | Magi3 | Membrane-associated guanylate kinase, WW and PDZ domain-containing protein 3 | | | | NaN | 0 | NaN | 0.026459 | 0.024184 | 0.083735 | 0.841029 | -0.65034 | -2.33957 | -3.88157 | -3.24362 | -1.23606 | -0.91953 | -2.16604 | -1.63872 | -3.17725 | -2.46182 | -1.34358 | -0.86038 | -1.34009 | -1.16401 |
| O35691 | O35691 | PININ_MO | Pnn | Pinin | + | | | 2.332167 | 0.75837 | 14.60707 | 0.783131 | 0.39343 | 2.14804 | 0.809115 | 0.466328 | 2.232814 | -0.50704 | -0.61085 | -0.63384 | -0.38164 | -0.13826 | 0.160499 | -1.31859 | -1.31604 | -1.03401 | -0.76833 | -0.30925 | -0.60117 |
| P48453 | P48453 | PP2BB_MC | Ppp3cb | Serine/threonine-protein phosphatase 2B catalytic subunit beta isoform | + | | | 1.671343 | 0.757084 | 6.739716 | 0.498115 | -0.65109 | -1.32022 | 0.382762 | -0.46963 | -1.02213 | 1.494544 | 1.702481 | 3.943872 | 3.095056 | 3.207555 | 4.119607 | 0.88396 | 0.798896 | 3.91937 | 4.421736 | 4.077082 | 4.18935 |
| Q64133 | Q64133 | AOFA_MO | Maoa | Amine oxidase [flavin-containing] A | | | | 0.852621 | -0.69814 | -2.37909 | 0.558943 | -0.47629 | -1.48392 | 0.022341 | -0.04785 | -0.071 | 0.067841 | 0.484852 | -0.56583 | -1.19658 | -0.64242 | -0.14177 | 1.180967 | 0.767996 | -0.46456 | -0.34527 | 0.281432 | -0.96993 |
| Q8BW75 | Q8BW75 | AOFB_MO | Maob | Amine oxidase [flavin-containing] B | | | | 0.729579 | -1.30252 | -1.97902 | 0.330682 | -1.07292 | -0.89086 | 0.20637 | 0.279402 | 0.577822 | -1.29254 | -0.10965 | -0.88677 | -1.29053 | -0.6559 | -0.00166 | 0.890178 | 0.312676 | -0.93843 | -1.09302 | -0.25208 | -0.96428 |
| Q9QYR6 | Q9QYR6 | MAP1A_M | Map1a | Microtubule-associated protein 1A | | | | 0.868986 | 0.325492 | 2.435584 | 0.142069 | 0.073895 | 0.410892 | 0.46757 | -0.08623 | -1.23993 | 4.15174 | 4.026159 | 4.531893 | 4.891561 | 4.194926 | 4.313126 | 3.881428 | 3.645486 | 4.636357 | 4.639307 | 4.303598 | 4.376923 |
| P14873 | P14873 | MAP1B_M | Map1b | Microtubule-associated protein 1B | | + | | 0.623747 | 0.505824 | 1.66502 | 1.228902 | 0.547525 | 3.931257 | 0.082735 | -0.08328 | -0.24908 | 2.489489 | 2.148881 | 3.437258 | 3.59001 | 2.921195 | 2.729119 | 2.064932 | 1.561791 | 2.849643 | 3.082574 | 2.588164 | 3.228719 |
| Q91VR7 | Q9CQV6;C | MLP3A_M( | Map1lc3a | Microtubule-associated proteins 1A/1B light chain 3A | | | | 0.655748 | -0.7157 | -1.7574 | 0.503224 | 0.167345 | 1.333762 | 0.683852 | -0.52713 | -1.8403 | 1.014773 | 1.563065 | 2.589529 | 2.407091 | 0.928244 | 1.397882 | 2.305777 | 1.70346 | 2.244817 | 2.417113 | 1.85422 | 1.526161 |
| Q8C052 | Q8C052 | MAP1S_M( | Map1s | Microtubule-associated protein 1S | | | | 0.83681 | 0.991444 | 2.325284 | 0.105889 | -0.09306 | -0.31342 | 0.042261 | -0.02654 | -0.1317 | 0.509013 | -0.13523 | 0.260744 | 0.589703 | 0.014356 | 0.055957 | -0.5252 | -1.0839 | 0.271091 | 0.765469 | 0.167854 | 0.354445 |
| P20357 | P20357 | MTAP2_M( | Map2 | Microtubule-associated protein 2 | | | | 0.729414 | 0.343868 | 1.978506 | 0.10712 | -0.07813 | -0.31679 | 0.418676 | -0.19947 | -1.11358 | 3.607283 | 3.357025 | 5.170105 | 5.505583 | 4.716468 | 5.074716 | 3.258908 | 3.017663 | 5.235156 | 5.596801 | 5.095708 | 5.094417 |
| P27546 | P27546 | MAP4_MO | Map4 | Microtubule-associated protein 4 | | | | 0.004946 | -0.00293 | -0.01602 | 0.278513 | 0.188713 | 0.760034 | 0.101857 | -0.0582 | -0.30234 | 1.597013 | 1.906446 | 2.438651 | 2.909446 | 2.822329 | 3.043069 | 1.656533 | 1.852794 | 2.406352 | 2.564319 | 2.833168 | 3.14864 |

| | | | | | | | | | | | | | | | | | | | | | | | | | | | | |
|---|---|---|---|---|---|---|---|---|---|---|---|---|---|---|---|---|---|---|---|---|---|---|---|---|---|---|---|---|
| Q7TSJ2 | Q7TSJ2 | MAP6_MO | Map6 | Microtubule-associated protein 6 | | | + | 0.520202 | 0.266998 | 1.379031 | 0.558307 | -0.18515 | -1.48218 | 1.283954 | -0.12979 | -4.21215 | 3.718539 | 3.432477 | 4.325658 | 4.547866 | 3.869384 | 3.929137 | 3.439001 | 3.178018 | 4.564813 | 4.679014 | 4.02151 | 4.036581 |
| Q14BB9 | Q14BB9 | MA6D1_M | Map6d1 | MAP6 domain-containing protein 1 | | | | 0.006655 | 0.011122 | 0.021508 | 0.949289 | 0.32118 | 2.72538 | 0.358111 | -0.61035 | -0.95985 | 0.160378 | 0.256444 | 1.413504 | 1.300642 | 1.059432 | 0.012111 | -0.31758 | 0.712162 | 1.139351 | 0.932435 | 0.785392 | 1.506846 |
| O88735 | O88735 | MAP7_MO | Map7 | Ensconsin | | | | 0.171115 | 0.34193 | 0.487091 | 0.172926 | 0.108007 | 0.491792 | 0.458176 | -0.71056 | -1.21546 | -0.32627 | 0.170667 | 1.978674 | 2.411182 | 0.795712 | 1.291103 | -1.07627 | 0.236811 | 2.048621 | 2.125222 | 1.224433 | 2.283497 |
| A2AJI0 | A2AJI0 | MA7D1_M | Map7d1 | MAP7 domain-containing protein 1 | | | | 0.917082 | 0.588454 | 2.606558 | 0.561886 | 0.227947 | 1.491991 | 0.192667 | 0.240274 | 0.542746 | 2.143476 | 1.798195 | 2.478373 | 2.779336 | 2.271398 | 2.083399 | 1.527855 | 1.236908 | 2.374506 | 2.42731 | 1.504519 | 2.36973 |
| A2AG50 | A2AG50 | MA7D2_M | Map7d2 | MAP7 domain-containing protein 2 | | | | 0.484215 | 0.244714 | 1.283537 | 0.557719 | 0.455026 | 1.480575 | 0.176363 | -0.11089 | -0.5007 | -0.10377 | -0.17787 | 0.407603 | 0.708011 | -0.2708 | -0.03357 | -0.57256 | -0.19852 | -0.16534 | 0.370905 | -0.22832 | 0.145728 |
| Q9WVI9 | Q9WVI9 | JIP1_MOU | Mapk8ip1 | C-Jun-amino-terminal kinase-interacting protein 1 | | | | 0.784199 | 1.917607 | 2.151488 | 0.768867 | 0.700898 | 2.10229 | 0.252013 | 0.221843 | 0.69344 | -0.95229 | -0.90707 | -0.20185 | -0.1533 | -1.15208 | -0.66385 | -3.73829 | -1.95628 | -1.21099 | -0.54596 | -0.92304 | -1.33658 |
| Q9ERE9 | Q9ERE9 | JIP2_MOU | Mapk8ip2 | C-Jun-amino-terminal kinase-interacting protein 2 | | | | NaN | 0 | NaN | 0.621236 | -0.44963 | -1.65786 | 0.961675 | -0.39591 | -2.77204 | -3.60925 | -3.1889 | -1.99614 | -1.51682 | -1.86498 | -1.85028 | -3.68742 | -2.64846 | -1.43381 | -1.17989 | -1.60435 | -1.31908 |
| Q9ESN9 | Q9ESN9 | JIP3_MOU | Mapk8ip3 | C-Jun-amino-terminal kinase-interacting protein 3 | | + | | 0.778389 | 0.673242 | 2.132771 | 1.080978 | 0.331511 | 3.251367 | 0.481904 | -0.28409 | -1.27746 | 0.716519 | 0.78819 | 1.378005 | 1.528045 | 0.402891 | 0.587378 | 0.392737 | -0.23451 | 1.052463 | 1.190564 | 0.576872 | 0.981584 |
| Q8BKH7 | Q8BKH7 | SIN1_MOU | Mapkap1 | Target of rapamycin complex 2 subunit MAPKAP1 | | | | NaN | 0 | NaN | 0.4192 | 0.736088 | 1.114921 | 0.109548 | 0.185158 | 0.323432 | -3.61461 | -3.37229 | -1.90269 | -1.01478 | -2.31063 | -2.02284 | -3.10741 | -3.30233 | -2.68349 | -1.70616 | -2.90599 | -1.7978 |
| Q61166 | Q61166 | MARE1_M | Mapre1 | Microtubule-associated protein RP/EB family member 1 | | | | 0.257555 | -0.16027 | -0.70739 | 0.822074 | -0.31609 | -2.2758 | 0.306113 | -0.22405 | -0.82924 | -0.15181 | 0.253552 | 0.938097 | 0.660965 | 0.34035 | 0.879759 | 0.109883 | 0.312401 | 1.125121 | 1.106122 | 0.850197 | 0.818007 |
| Q8R001 | Q8R001 | MARE2_M | Mapre2 | Microtubule-associated protein RP/EB family member 2 | | | | 0.930516 | -0.28274 | -2.65568 | 0.031511 | 0.030045 | 0.099219 | 0.128037 | 0.151491 | 0.373473 | -0.31609 | -0.24929 | 0.341709 | -0.26392 | 0.587214 | -0.16212 | 0.101146 | -0.10104 | 0.008641 | 0.009058 | 0.21648 | -0.09437 |
| Q6PER3 | Q6PER3 | MARE3_M | Mapre3 | Microtubule-associated protein RP/EB family member 3 | | | | 0.514127 | 0.12008 | 1.362787 | 0.877945 | -0.25734 | -2.46687 | 0.405213 | -0.13274 | -1.07918 | 2.099386 | 2.091792 | 2.461788 | 2.328471 | 2.287474 | 2.243856 | 2.06354 | 1.887477 | 2.732708 | 2.572224 | 2.519462 | 2.277353 |
| Q8R1B4 | Q8R1B4 | EIF3C_MO | Eif3c | Eukaryotic translation initiation factor 3 subunit C | + | | | 1.652157 | 0.754208 | 6.587592 | 0.157132 | 0.086312 | 0.450602 | 0.352109 | 0.492214 | 0.944725 | 0.210941 | 0.163393 | 0.494891 | 0.55523 | 1.275484 | 0.274954 | -0.45505 | -0.67903 | 0.249592 | 0.627905 | 0.137437 | 0.428573 |
| P26645 | P26645 | MARCS_M | Marcks | Myristoylated alanine-rich C-kinase substrate | | | | 0.542939 | 0.703544 | 1.440312 | 0.699176 | 0.729054 | 1.886238 | 0.263479 | 0.163071 | 0.722285 | 0.842395 | 1.789992 | 2.141946 | 1.370685 | 0.843216 | 1.276111 | 0.493845 | 0.731454 | 1.053346 | 1.001177 | 0.960803 | 0.832382 |
| P28667 | P28667 | MRP_MOU | Marcksl1 | MARCKS-related protein | | | | NaN | 0 | NaN | 0.075967 | -0.13429 | -0.22993 | 0.581409 | 0.786057 | 1.54586 | -4.45752 | -3.16689 | -2.64596 | -3.7836 | -2.98226 | -2.1623 | -2.52961 | -2.78356 | -3.21297 | -2.94801 | -3.65913 | -3.05754 |
| Q68FL6 | Q68FL6 | SYMC_MO | Mars1 | Methionine--tRNA ligase, cytoplasmic | | | | 0.295956 | 1.35129 | 0.803776 | 0.518079 | -0.20437 | -1.37335 | 0.085686 | 0.050483 | 0.257383 | -0.48115 | -1.35395 | -0.40172 | -0.21556 | -0.19765 | -0.37405 | -0.64529 | -3.89239 | -0.22038 | 0.011834 | -0.51153 | -0.16114 |
| Q3THS6 | Q3THS6 | METK2_M | Mat2a | S-adenosylmethionine synthase isoform type-2 | | | | 0.341447 | -0.2689 | -0.9179 | 0.983276 | -0.38554 | -2.85476 | 0.048173 | -0.07455 | -0.14931 | -1.63499 | -1.06408 | -1.38888 | -1.49822 | -1.45495 | -0.59352 | -1.01478 | -1.14648 | -1.1815 | -0.93452 | -0.69711 | -1.20225 |
| Q99LB6 | Q99LB6 | MAT2B_M | Mat2b | Methionine adenosyltransferase 2 subunit beta | | | | 0.453885 | -0.40866 | -1.20431 | 0.213803 | 0.075115 | 0.596766 | 0.122244 | -0.14221 | -0.35789 | -0.45363 | -1.04504 | -0.32571 | -0.12316 | -0.69715 | -0.03498 | -0.17424 | -0.50712 | -0.37429 | -0.22481 | -0.00413 | -0.44357 |
| Q8K310 | Q8K310 | MATR3_M | Matr3 | Matrin-3 | | + | | 0.650208 | 1.326552 | 1.741254 | 1.012077 | 0.903025 | 2.967749 | 0.357395 | 0.951514 | 0.958041 | -0.29099 | 0.208486 | 0.198545 | 0.768469 | 1.537582 | -0.40157 | -0.64806 | -2.08754 | -0.31283 | -0.52621 | -0.59879 | -0.16823 |
| P56671 | P56671 | MAZ_MOU | Maz | Myc-associated zinc finger protein | + | | | 1.055478 | 0.520021 | 3.144141 | 0.543938 | 2.256206 | 1.443023 | 0.387258 | -0.34838 | -1.03353 | 1.082501 | 1.36927 | 0.074987 | 0.457395 | 0.098812 | 0.772748 | 0.623426 | 0.788302 | -3.54181 | -0.43822 | 0.792631 | 0.775681 |
| Q9Z2D8 | Q9Z2D8 | MBD3_MO | Mbd3 | Methyl-CpG-binding domain protein 3 | | | | 0.128978 | 0.399555 | 0.375996 | 0.300806 | -0.37704 | -0.81593 | 0.450676 | -0.42582 | -1.19599 | -3.72294 | -1.66 | -1.44794 | -1.31211 | -1.81532 | -2.18176 | -3.34658 | -2.83547 | -1.46007 | -0.5459 | -1.26744 | -1.878 |
| Q8BL86 | Q8BL86 | MBLC2_M | Mblac2 | Acyl-coenzyme A thioesterase MBLAC2 | | | | 0.2775 | 0.881143 | 0.757494 | 0.057652 | -0.09382 | -0.17718 | 0.308183 | 0.172347 | 0.834428 | -1.18798 | -3.51358 | -2.57557 | -1.56531 | -0.83815 | -1.14356 | -3.20017 | -3.26367 | -1.8178 | -2.13544 | -1.30227 | -1.02412 |
| Q8C181;Q | Q9JKP5;Q | MBNL1_M | Mbnl1;Mbnl2 | Muscleblind-like protein 2 | | + | | 0.666784 | -1.8776 | -1.78975 | 1.535878 | -0.35483 | -5.7318 | 0.17953 | 1.065088 | 0.508898 | -2.86934 | -0.77661 | -0.33202 | -0.31748 | -0.8371 | -0.57058 | -0.02093 | 0.130176 | 0.091562 | -0.03139 | 0.319754 | -3.85761 |
| Q8R3I2 | Q8R3I2 | MBOA2_M | Mboat2 | Lysophospholipid acyltransferase 2 | | | | 0.282149 | 0.097714 | 0.769156 | 0.688658 | -0.6373 | -1.85465 | 0.073209 | 0.071814 | 0.222072 | 0.558821 | 0.306733 | -1.51155 | -0.83827 | -0.69901 | -0.43693 | 0.350939 | 0.319188 | -0.46868 | -0.60654 | -0.93543 | -0.34415 |
| Q9CZS1 | Q9CZS1 | AL1B1_M | Aldh1b1 | Aldehyde dehydrogenase X, mitochondrial | + | | | 1.387647 | 0.747945 | 4.787994 | 0.658369 | 0.428795 | 1.765056 | 0.073257 | 0.18157 | 0.222208 | 2.0526 | 1.819286 | 2.737228 | 3.123667 | 3.156907 | 2.23957 | 1.084106 | 1.29189 | 2.354397 | 2.648907 | 1.840429 | 3.192908 |
| P04370 | P04370 | MBP_MOU | Mbp | Myelin basic protein | | + | | 0.603393 | 0.651374 | 1.60732 | 1.102265 | 0.917614 | 3.342999 | 0.106691 | 0.158412 | 0.315616 | 8.796253 | 8.624126 | 6.981319 | 7.360775 | 6.537039 | 6.598505 | 8.454826 | 7.662805 | 6.451794 | 6.055072 | 5.908389 | 6.910331 |
| Q99MR8 | Q99MR8 | MCCA_M | Mccc1 | Methylcrotonoyl-CoA carboxylase subunit alpha, mitochondrial | | | | 0.286769 | -0.20278 | -0.78074 | 0.39342 | 0.495846 | 1.049168 | 0.059817 | -0.14072 | -0.18349 | -1.39581 | -1.17652 | -1.12119 | -1.44653 | -0.20755 | -1.61478 | -0.84794 | -1.31883 | -2.22343 | -1.33597 | -1.07554 | -0.46536 |
| Q3ULD5 | Q3ULD5 | MCCB_M | Mccc2 | Methylcrotonoyl-CoA carboxylase beta chain, mitochondrial | | | + | 0.002542 | -0.00432 | -0.00825 | 0.147852 | -0.11387 | -0.42619 | 1.427423 | -0.14633 | -5.02649 | -1.03287 | -0.20979 | -1.47471 | -0.94831 | -0.48471 | -0.53183 | -0.94137 | -0.29265 | -1.05166 | -1.14361 | -0.34484 | -0.37905 |
| Q3UGS4 | Q3UGS4 | MCRI1_M | Mcrip1 | Mapk-regulated corepressor-interacting protein 1 | | | | 0.822149 | -1.63615 | -2.27606 | 0.203145 | 0.180944 | 0.569586 | 0.294634 | -0.23268 | -0.80046 | -2.06748 | -3.50435 | -0.74338 | -0.90691 | -1.77671 | -1.5148 | -1.17433 | -1.12521 | -1.31306 | -0.69912 | -1.67259 | -1.15357 |
| Q3UMR5 | Q3UMR5 | MCU_MOU | Mcu | Calcium uniporter protein, mitochondrial | | | | 0.150561 | -0.05686 | -0.43333 | 0.176186 | -0.07054 | -0.50025 | 0.312771 | 0.211296 | 0.845932 | 0.391606 | 0.243764 | 0.650725 | 0.440842 | 1.515459 | 1.55784 | 0.482969 | 0.266127 | 0.710498 | 0.522142 | 1.574232 | 1.076474 |
| P14152 | P14152 | MDHC_M | Mdh1 | Malate dehydrogenase, cytoplasmic | + | | | 1.091209 | -1.69013 | -3.29517 | 0.080447 | -0.2632 | -0.24262 | 0.003322 | 0.008126 | 0.010778 | 1.808044 | 2.453057 | 3.759408 | 1.596447 | 2.158551 | 3.207912 | 4.219515 | 3.421847 | 3.02594 | 2.856313 | 3.216635 | 2.133575 |
| P08249 | P08249 | MDHM_M | Mdh2 | Malate dehydrogenase, mitochondrial | + | | | 1.229535 | -1.61529 | -3.93439 | 0.485447 | -1.27546 | -1.28678 | 0.068033 | -0.18413 | -0.20725 | 2.36851 | 3.088488 | 4.086042 | 2.115026 | 3.09028 | 4.641192 | 4.541176 | 4.146393 | 4.269851 | 4.482146 | 4.483428 | 3.616297 |
| P06801 | P06801 | MAOX_M | Me1 | NADP-dependent malic enzyme | | | | 0.710348 | -0.20813 | -1.92007 | 0.193362 | -0.47028 | -0.54453 | 0.147067 | 0.222518 | 0.424117 | -0.72275 | -0.56576 | -0.51716 | -2.24254 | -0.62581 | 0.187467 | -0.51088 | -0.36138 | -0.95002 | -0.86913 | -0.11016 | -0.77322 |
| Q8BMF3 | Q8BMF3 | MAON_M | Me3 | NADP-dependent malic enzyme, mitochondrial | | | | 0.993922 | -1.28642 | -2.89616 | 0.267112 | -0.46678 | -0.73141 | 0.22925 | 0.280526 | 0.635973 | -0.49224 | -0.53728 | -0.08631 | -1.23064 | -0.40826 | 0.419088 | 0.32805 | 1.215269 | 0.090998 | -0.47439 | -0.42822 | -0.12201 |
| Q9Z2D6 | Q9Z2D6 | MECP2_M | Mecp2 | Methyl-CpG-binding protein 2 | | | | 0.755538 | 0.434692 | 2.060024 | 0.017186 | 0.044557 | 0.054913 | 0.564337 | -0.56376 | -1.49872 | 0.285964 | 0.230819 | -0.39832 | 1.219293 | -0.79387 | -0.07236 | 0.032904 | -0.3855 | 0.300786 | 0.431076 | 0.024104 | 0.237177 |
| Q80YQ2 | Q80YQ2 | MED23_M | Med23 | Mediator of RNA polymerase II transcription subunit 23 | | | | NaN | 0 | NaN | 0.058823 | 0.179289 | 0.180596 | 0.053967 | 0.158226 | 0.166394 | -3.60805 | -3.27386 | -2.34003 | -2.77135 | -2.08448 | -2.99528 | -3.05939 | -2.87948 | -3.70404 | -1.76592 | -3.53288 | -1.86334 |
| Q8CFN5;Q | Q8CFN5;Q | MEF2C_M | Mef2c | Myocyte-specific enhancer factor 2C | | | | NaN | 0 | NaN | 0.100073 | -0.18235 | -0.29742 | 0.00968 | 0.037871 | 0.031182 | -3.97253 | -3.68707 | -0.61755 | -1.24082 | -1.31277 | -3.21546 | -2.58336 | -4.64221 | -1.27482 | -0.21885 | -3.05699 | -1.54698 |
| Q63943 | Q63943 | MEF2D_M | Mef2d | Myocyte-specific enhancer factor 2D | | | | 0.130982 | 0.402134 | 0.381363 | 0.097118 | 0.064217 | 0.289251 | 0.564906 | 0.550758 | 1.500282 | -3.38331 | -1.27477 | -0.89486 | -1.09362 | -0.98565 | -1.70483 | -2.7516 | -2.71074 | -1.25698 | -0.85993 | -1.8221 | -1.9699 |
| Q07646 | Q07646 | MEST_MO | Mest | Mesoderm-specific transcript protein | | + | | 0.124147 | 0.187757 | 0.363018 | 2.202116 | -0.46832 | -12.5605 | 0.159527 | -0.13954 | -0.45688 | -0.1242 | 0.005161 | -0.67994 | -0.74518 | -1.00954 | -1.47555 | -0.76043 | 0.265874 | -0.22619 | -0.2623 | -1.30046 | -0.90555 |
| Q8BP48 | Q8BP48 | MAP11_M | Metap1 | Methionine aminopeptidase 1 | | | | 0.734328 | 0.369993 | 1.993709 | 0.551853 | 0.296102 | 1.464553 | 0.085344 | -0.06745 | -0.25642 | -1.09427 | -1.09938 | -0.38429 | -0.45653 | -0.82433 | -0.3632 | -1.28126 | -1.65239 | -0.91544 | -0.51759 | -0.65291 | -0.39973 |
| O08663 | O08663 | MAP2_MO | Metap2 | Methionine aminopeptidase 2 | | + | | 0.47572 | -1.63193 | -1.26124 | 1.033756 | -0.4816 | -3.05492 | 0.124533 | -0.13902 | -0.36406 | -4.07493 | -1.62628 | -1.44112 | -1.69557 | -1.82039 | -1.08711 | -0.80009 | -1.63727 | -1.17984 | -0.99365 | -1.20801 | -1.42146 |
| Q9DCS2 | Q9DCS2 | MTL26_M | Mettl26 | Methyltransferase-like 26 | | | | 0.429475 | 1.719961 | 1.141284 | 0.058081 | 0.058385 | 0.178435 | 0.00535 | 0.008711 | 0.017317 | 0.20576 | -0.23158 | -0.40274 | -1.05103 | -0.542 | -0.18983 | -3.22396 | -0.24178 | -0.74061 | -0.82992 | 0.096582 | -0.84584 |
| Q8C680 | Q8C680 | Q8C680_M | Mettl7a1 | Methyltransferase-like 7A1 | | | | 0.497539 | -2.23813 | -1.31869 | NaN | 0 | NaN | NaN | 0 | NaN | -0.10228 | -3.49082 | -3.13574 | -3.86024 | -3.62439 | -3.5967 | 0.341352 | 0.541804 | -3.11088 | -3.05618 | -3.4249 | -3.24553 |
| Q6PCP5 | Q6PCP5 | MFF_MOU | Mff | Mitochondrial fission factor | | | | 0.172127 | -0.1614 | -0.48972 | 0.283564 | 0.156563 | 0.772705 | 0.071691 | -0.08274 | -0.21774 | -1.10258 | -0.47184 | -0.16839 | -0.29463 | 0.263728 | -0.27 | -0.5301 | -0.72153 | -0.58061 | -0.19554 | -0.19092 | 0.350124 |
| Q80U63 | Q80U63 | MFN2_MO | Mfn2 | Mitofusin-2 | | | | 0.6723 | 0.534257 | 1.806013 | 0.382038 | 0.116094 | 1.020294 | 0.480082 | 0.475823 | 1.272678 | 0.238014 | 0.536391 | 0.639947 | 0.414203 | 1.423352 | 0.710243 | 0.108392 | -0.4025 | 0.396601 | 0.425361 | 0.478495 | 0.703454 |
| O35678 | O35678 | MGLL_MO | Mgll | Monoglyceride lipase | | + | | 0.986922 | 0.369861 | 2.868887 | 1.458932 | -0.48952 | -5.22287 | 0.218169 | -0.2058 | -0.60787 | 0.521582 | 0.498155 | 0.505184 | 0.550269 | 0.833735 | 1.143958 | 0.01162 | 0.268396 | 1.108226 | 0.926275 | 1.495591 | 0.893706 |
| Q91VS7 | Q91VS7 | MGST1_M | Mgst1 | Microsomal glutathione S-transferase 1 | | + | | 0.349592 | -0.45072 | -0.93839 | 1.901353 | 1.015194 | 8.842158 | 0.713151 | -0.66494 | -1.92861 | -1.52629 | -1.49823 | -2.23153 | -2.01902 | -3.17924 | -3.13336 | -1.54165 | -0.58143 | -3.09698 | -3.18396 | -2.14735 | -2.83538 |
| Q9CPU4 | Q9CPU4 | MGST3_M | Mgst3 | Glutathione S-transferase 3, mitochondrial | | | | 0.927818 | 2.155522 | 2.645771 | 0.151674 | 0.17826 | 0.436263 | 0.161027 | 0.172053 | 0.460799 | 0.665866 | 0.928439 | -0.28498 | -0.29587 | 0.538311 | -0.20815 | -2.16243 | -0.55431 | -0.87725 | -0.06011 | -0.01758 | 0.003643 |
| Q8R516 | Q8R516 | MIB2_MO | Mib2 | E3 ubiquitin-protein ligase MIB2 | | + | | NaN | 0 | NaN | 1.083691 | -0.84429 | -3.26294 | 0.17673 | -0.65588 | -0.50165 | -2.78877 | -3.231 | -2.88628 | -2.37267 | -3.68741 | -2.67231 | -3.32577 | -3.21412 | -1.75354 | -1.81685 | -3.72888 | -1.31908 |
| Q8CJ19 | Q8CJ19 | MICA3_M | Mical3 | [F-actin]-monooxygenase MICAL3 | | | | 0.46302 | 0.412862 | 1.228065 | 0.739668 | -0.56364 | -2.0103 | 0.58371 | -0.27132 | -1.55225 | -0.78142 | -1.45201 | -0.39706 | 0.068864 | -0.33741 | -0.03516 | -1.50509 | -1.55407 | 0.243529 | 0.555552 | -0.00279 | 0.172869 |
| Q8R404 | Q8R404 | MIC13_M | Micos13 | MICOS complex subunit MIC13 | | | | 0.109286 | -0.03125 | -0.32272 | 0.3546 | 0.14277 | 0.950999 | 0.128861 | 0.137499 | 0.375683 | 0.948035 | 0.763896 | 0.615771 | 0.435562 | 1.008598 | 0.276651 | 0.857203 | 0.91723 | 0.502976 | 0.262818 | 0.500909 | 0.509342 |
| Q8VCX5 | Q8VCX5 | MICU1_M | Micu1 | Calcium uptake protein 1, mitochondrial | | | | NaN | 0 | NaN | 0.008615 | 0.034218 | 0.027783 | 0.302819 | 0.296604 | 0.820981 | -3.19164 | -3.32325 | -4.81464 | -2.42604 | -2.71298 | -3.37957 | -3.68514 | -3.55103 | -3.35377 | -3.95535 | -3.48229 | -3.20347 |
| Q9CTY5 | Q9CTY5 | MICU3_M | Micu3 | Calcium uptake protein 3, mitochondrial | | | | 0.032164 | 0.030353 | 0.101211 | 0.442854 | 0.25951 | 1.175756 | 0.255214 | 0.224419 | 0.701499 | -0.11265 | -0.57522 | 0.155982 | 0.220495 | 0.373212 | -0.18146 | -0.18338 | -0.5652 | 0.147076 | -0.28962 | -0.28801 | 0.030925 |
| P34884 | P34884 | MIF_MOU | Mif | Macrophage migration inhibitory factor | | + | + | 0.566819 | -0.10891 | -1.50554 | 1.107164 | -0.94239 | -3.36437 | 1.11904 | 0.495604 | 3.416608 | 6.6475 | 6.502822 | 5.509815 | 5.065597 | 6.742363 | 7.03239 | 6.684467 | 6.683675 | 6.400767 | 6.059425 | 6.388207 | 6.395337 |
| Q8VE19 | Q8VE19 | MIO_MOU | Mios | GATOR complex protein MIOS | | | | 0.363353 | 0.993097 | 0.973061 | 0.085316 | -0.04093 | -0.25634 | 0.539246 | -0.54605 | -1.43031 | -1.69623 | -1.68787 | 0.085047 | 0.349955 | -0.62166 | -0.23477 | -1.66456 | -3.70573 | 0.3476 | 0.169263 | -0.2113 | 0.44697 |
| A6H611 | A6H611 | MIPEP_M | Mipep | Mitochondrial intermediate peptidase | | | + | 0.039442 | -0.13783 | -0.12324 | 0.393968 | -0.74199 | -1.05056 | 2.306268 | -0.48978 | -14.1749 | -3.40783 | -1.90821 | -4.06974 | -3.09838 | -2.35867 | -2.29923 | -1.69044 | -3.34994 | -3.35486 | -2.32928 | -1.82154 | -1.85679 |
| Q8VHK5 | Q8VHK5 | MLC1_MO | Mlc1 | Membrane protein MLC1 | | | | 0.272725 | 0.455982 | 0.745509 | 0.697086 | 0.733586 | 1.87994 | 0.487089 | 0.650357 | 1.291099 | -0.49552 | -0.37252 | -0.60331 | -0.28041 | 0.254129 | -0.49439 | -1.49854 | -0.28146 | -1.53069 | -0.82019 | -0.43334 | -1.10763 |
| Q99KX1 | Q99KX1 | MLF2_MO | Mlf2 | Myeloid leukemia factor 2 | | | | 0.678818 | 1.687088 | 1.825319 | 0.689513 | 0.322031 | 1.857206 | 0.232481 | -0.5403 | -0.64415 | -0.31449 | -0.73796 | 1.51546 | 1.860198 | 0.708527 | 0.676087 | -1.31362 | -3.113 | 1.346959 | 1.384638 | 0.393986 | 2.071224 |
| A0A1Y7V | A0A1Y7V | A0A1Y7V | Mlh3 | MutL homolog 3 | | | | 0.003821 | 0.030631 | 0.012389 | NaN | 0 | NaN | 0.354933 | 2.187711 | 0.951839 | -3.55209 | -0.38114 | -3.72879 | -3.26358 | 0.824741 | -3.6818 | -3.89441 | -0.10009 | -3.01878 | -4.03702 | -4.06949 | -3.16299 |
| Q5FW52 | Q5FW52 | MLIP_MOU | Mlip | Muscular LMNA-interacting protein | | | | NaN | 0 | NaN | 0.65956 | -0.91139 | -1.76854 | 0.522523 | -1.03789 | -1.38525 | -3.50306 | -3.73841 | -2.52049 | -1.52675 | -3.48067 | -3.11233 | -3.74061 | -3.0148 | -1.24895 | -0.97551 | -2.98487 | -1.53236 |
| Q9DCJ1 | Q9DCJ1 | LST8_MO | Mlst8 | Target of rapamycin complex subunit LST8 | | + | | 0.686775 | 1.147848 | 1.84902 | 1.088393 | 0.476724 | 3.283063 | 0.791291 | -0.48546 | -2.17446 | 0.533436 | -0.0788 | 0.615027 | 0.786265 | -0.59884 | -0.16126 | -0.38047 | -1.46059 | 0.341201 | 0.106642 | 0.06099 | 0.149832 |
| Q99J39 | Q99J39 | DCMC_M | Mlycd | Malonyl-CoA decarboxylase, mitochondrial | | | | 0.335507 | 0.114371 | 0.902979 | 0.64091 | 0.521029 | 1.714306 | 0.027885 | -0.01009 | -0.08812 | 2.501973 | 2.28862 | 1.797369 | 2.120276 | 1.646131 | 1.803174 | 2.212642 | 2.34921 | 1.180295 | 1.695294 | 1.818048 | 1.651432 |
| Q99LX5 | Q99LX5 | MMTA2_M | Mmtag2 | Multiple myeloma tumor-associated protein 2 homolog | | + | | 0.431085 | 1.384909 | 1.145422 | 2.127853 | 0.812689 | 11.52098 | 0.22674 | 0.918317 | 0.629616 | -0.70139 | -1.05976 | -0.88336 | -0.74917 | -1.16354 | -1.47735 | -1.06975 | -3.46121 | -1.65074 | -1.60717 | -3.68883 | -0.78869 |
| P51949 | P51949 | MAT1_MO | Mnat1 | CDK-activating kinase assembly factor MAT1 | | | + | 0.337121 | 0.355793 | 0.907035 | 0.747994 | -0.5178 | -2.03631 | 1.674562 | -0.5011 | -6.76555 | 0.662629 | 0.961896 | 0.411275 | 0.408233 | -0.20475 | -0.10675 | 0.093872 | 0.819067 | 1.181831 | 0.673274 | 0.289817 | 0.400901 |
| D0QMC3 | D0QMC3 | MNDAL_M | Mndal | Myeloid cell nuclear differentiation antigen-like protein | | | | NaN | 0 | NaN | 0.442978 | -0.54428 | -1.17608 | NaN | 0 | NaN | -3.26761 | -2.99364 | -3.47329 | -4.34819 | -3.98705 | -3.70889 | -3.58884 | -3.54058 | -3.51752 | -3.2154 | -3.3185 | -2.95633 |
| Q8BP80 | Q921Y0;Q | MOB1B_M | Mob1b | MOB kinase activator 1B | + | | | 1.258214 | 1.144237 | 4.078788 | 0.630022 | 0.497098 | 1.682973 | 0.14578 | -0.36184 | -0.42072 | 2.479264 | 2.448693 | 1.528551 | 2.115505 | 0.005915 | 1.676578 | 1.599858 | 1.039625 | 1.358305 | 1.291556 | 0.998317 | 1.407863 |
| Q8VI63 | Q8VI63 | MOB2_MO | Mob2 | MOB kinase activator 2 | | | | 0.860865 | 0.305182 | 2.407445 | 0.125634 | -0.15199 | -0.36702 | 0.340977 | -0.10342 | -0.91672 | 2.96783 | 3.221218 | 1.789102 | 2.465665 | 1.99413 | 1.987278 | 2.785068 | 2.793617 | 2.518242 | 2.040502 | 1.981361 | 2.206882 |
| Q9JMC8 | Q9JMC8 | E41LB_M | Epb41l4b | Band 4.1-like protein 4B | + | | | 1.574955 | 0.738229 | 6.007214 | 0.112537 | 0.183939 | 0.331584 | 0.246241 | -0.34386 | -0.6789 | 0.531889 | 0.286618 | 1.225487 | 2.33243 | 0.320337 | 1.015346 | -0.33688 | -0.32107 | 1.557695 | 1.632344 | 0.643223 | 1.380171 |
| Q9D2P8 | Q9D2P8 | MOBP_M | Mobp | Myelin-associated oligodendrocyte basic protein | | | | 0.638258 | -0.45211 | -1.70665 | 0.242672 | 0.415335 | 0.669897 | 0.779146 | 0.876608 | 2.135206 | 0.929587 | 0.55409 | -0.3549 | -1.23564 | 0.493583 | 0.43769 | 1.38084 | 1.007058 | -0.77417 | -1.64704 | -0.00137 | -0.82057 |
| Q3UGC7;Q | Q66JS6;Q | EI3JA_MO | Eif3j1;Eif3j2 | Eukaryotic translation initiation factor 3 subunit J-A | + | | + | 1.498088 | 0.73271 | 5.476492 | 0.295995 | 0.195705 | 0.803876 | 1.043841 | 0.267733 | 3.096103 | 9.033266 | 9.26989 | 8.10643 | 8.567078 | 8.228838 | 8.401566 | 8.356398 | 8.481339 | 8.062181 | 8.219915 | 8.051835 | 8.043103 |
| Q80TL7 | Q80TL7 | MON2_M | Mon2 | Protein MON2 homolog | + | | | 1.251893 | 0.581598 | 4.046589 | 0.572982 | 0.245094 | 1.522528 | 0.269305 | -0.30692 | -0.73692 | 0.918412 | 1.029171 | 1.493202 | 1.801077 | 0.505743 | 1.002048 | 0.524821 | 0.259566 | 1.449132 | 1.354959 | 0.726329 | 1.395293 |
| P63030 | P63030 | MPC1_M | Mpc1 | Mitochondrial pyruvate carrier 1 | | | | 0.271805 | -0.1803 | -0.7432 | 0.483887 | -0.65813 | -1.28267 | 0.164268 | 0.136429 | 0.469263 | 2.468858 | 2.568026 | 0.557739 | 0.196527 | 1.608415 | 1.829425 | 2.461263 | 2.936224 | 1.515521 | 0.555008 | 1.851401 | 1.313581 |
| Q9D023 | Q9D023 | MPC2_M | Mpc2 | Mitochondrial pyruvate carrier 2 | | | | 0.88602 | 0.739101 | 2.495281 | 0.541087 | -0.38835 | -1.43529 | 0.687286 | 0.111047 | 1.850547 | 2.648723 | 2.564546 | 2.04323 | 1.522765 | 2.551726 | 2.586555 | 2.160728 | 1.574339 | 2.245439 | 2.09726 | 2.515519 | 2.400669 |
| Q9R0Q9 | Q9R0Q9 | MPU1_M | Mpdu1 | Mannose-P-dolichol utilization defect 1 protein | | | | 0.425653 | -0.43601 | -1.13147 | 0.059383 | -0.07361 | -0.18223 | 0.207124 | 0.163811 | 0.579747 | 0.196598 | 0.915254 | -0.27408 | -0.64283 | 0.311819 | 0.68409 | 0.85273 | 1.131147 | -0.74429 | -0.0254 | 0.546727 | 0.121559 |
| Q8VBX6 | Q8VBX6 | MPDZ_M | Mpdz | Multiple PDZ domain protein | | | | NaN | 0 | NaN | 0.344134 | -0.47455 | -0.92466 | 0.272333 | -0.26844 | -0.74452 | -3.38084 | -3.99685 | -3.22426 | -4.22479 | -3.37398 | -2.66819 | -2.787 | -3.33701 | -3.36453 | -3.13542 | -2.6787 | -2.82658 |
| Q924M7 | Q924M7 | MPI_MOU | Mpi | Mannose-6-phosphate isomerase | | | | NaN | 0 | NaN | 0.236801 | 0.828655 | 0.655073 | 0.139982 | -0.48813 | -0.40535 | -4.1629 | -3.01907 | -1.02298 | -2.64681 | -3.65491 | -1.5423 | -3.0618 | -3.76836 | -3.63359 | -1.69352 | -1.53224 | -2.68871 |
| P70290 | P70290 | EM55_M | Mpp1 | 55 kDa erythrocyte membrane protein | | | | 0.205261 | 0.107878 | 0.574993 | 0.091898 | -0.04488 | -0.27475 | 0.806752 | -0.29614 | -2.22503 | -0.55744 | -0.33681 | -0.4583 | -0.26568 | -0.67892 | -0.65298 | -0.70676 | -0.40325 | -0.44904 | -0.18519 | -0.50227 | -0.23735 |
| Q9WV34 | Q9WV34 | MPP2_M | Mpp2 | MAGUK p55 subfamily member 2 | | | | 0.409723 | 0.677133 | 1.090688 | 0.906772 | -0.48059 | -2.56927 | 0.712495 | -0.22239 | -1.92661 | -0.65516 | -0.20355 | 0.905749 | 0.975077 | 0.602778 | 0.375973 | -1.6848 | -0.52817 | 1.237188 | 1.604812 | 0.690225 | 0.733305 |
| O88910 | O88910 | MPP3_M | Mpp3 | MAGUK p55 subfamily member 3 | + | | | 1.174394 | 1.777453 | 3.668624 | 0.348491 | -0.29656 | -0.93562 | 0.593262 | -0.88658 | -1.57889 | -2.00214 | -1.11247 | -1.45191 | -1.84642 | -1.16753 | -2.13071 | -3.14276 | -3.52675 | -1.60071 | -1.10449 | -0.47379 | -1.0513 |
| Q99J99 | Q99J99 | THTM_M | Mpst | 3-mercaptopyruvate sulfurtransferase | | | | 0.129614 | 0.137962 | 0.3777 | 0.146888 | -0.35493 | -0.42365 | 0.29971 | -0.49062 | -0.81319 | -3.07197 | -3.76635 | -1.36324 | -2.49296 | -2.26695 | -1.86664 | -3.44365 | -3.6706 | -2.19192 | -0.95442 | -2.14534 | -1.00702 |
| Q61216 | Q61216 | MRE11_M | Mre11 | Double-strand break repair protein MRE11 | | | | 0.033271 | 0.061201 | 0.104579 | 0.845813 | -0.30879 | -2.35583 | 0.818123 | -1.25795 | -2.26265 | -3.72729 | -2.68677 | -1.1552 | -1.12734 | -2.79491 | -3.20099 | -3.53619 | -3.00028 | -0.96281 | -0.70215 | -2.25757 | -1.22245 |
| Q9DB15 | Q9DB15 | RM12_M | Mrpl12 | Large ribosomal subunit protein bL12m | | | | 0.21236 | -0.46225 | -0.59309 | 0.65434 | 0.31538 | 1.753286 | 0.336962 | -0.29963 | -0.90663 | -2.35026 | -0.87642 | -0.34611 | -0.20895 | -1.52633 | -0.87986 | -1.40488 | -0.89729 | -0.7592 | -0.42662 | -0.97231 | -0.83461 |
| Q9D1I6 | Q9D1I6 | RM14_M | Mrpl14 | Large ribosomal subunit protein uL14m | | + | | 0.876511 | 1.052886 | 2.461841 | 1.016411 | -0.58422 | -2.98503 | 0.244033 | 0.180223 | 0.67333 | 0.407642 | -0.28219 | -0.11728 | -0.10036 | -0.3364 | -0.00393 | -0.73728 | -1.24303 | 0.279871 | 0.670941 | -0.56017 | -0.14061 |
| Q9D1N9 | Q9D1N9 | RM21_M | Mrpl21 | Large ribosomal subunit protein bL21m | | | | NaN | 0 | NaN | 0.374173 | 0.761559 | 1.000389 | 0.283736 | 0.834425 | 0.773138 | -3.44716 | -3.5202 | -1.70433 | -1.67888 | -1.83921 | -2.31527 | -3.06439 | -3.6026 | -3.21432 | -1.69201 | -3.96436 | -1.85897 |
| Q8R035 | Q8R035 | ICT1_M | Mrpl58 | Large ribosomal subunit protein mL62 | | | | 0.021093 | -0.08914 | -0.06712 | 0.044436 | -0.07641 | -0.1382 | 0.064 | -0.20039 | -0.19562 | -3.61411 | -1.72216 | -2.02658 | -2.11375 | -2.18366 | -2.76185 | -1.64689 | -3.5111 | -1.44259 | -2.54493 | -1.28961 | -3.25511 |
| Q80WQ2 | Q80WQ2 | VAC14_M | Vac14 | Protein VAC14 homolog | + | | | 1.758147 | 0.716929 | 7.470164 | 0.256551 | 0.216013 | 0.704862 | 0.618613 | -0.29337 | -1.65039 | 0.255399 | 0.068223 | -0.06995 | 0.484196 | -0.58998 | -0.2904 | -0.53386 | -0.57638 | -0.13985 | 0.122064 | -0.24253 | -0.05112 |
| Q9CPX7 | Q9CPX7 | RT16_M | Mrps16 | Small ribosomal subunit protein bS16m | | | | 0.098049 | 0.497956 | 0.291826 | 0.762807 | -0.09792 | -2.08302 | 0.994609 | 0.649831 | 2.898844 | -0.90963 | -2.61053 | -1.15257 | -1.17964 | -0.58437 | -0.994 | -3.73734 | -0.77873 | -1.1132 | -1.02317 | -1.53014 | -1.3479 |
| P58059 | P58059 | RT21_M | Mrps21 | Small ribosomal subunit protein bS21m | | | | NaN | 0 | NaN | 0.070298 | 0.145878 | 0.213749 | 0.296806 | -0.54972 | -0.80591 | -2.8437 | -3.26863 | -4.42647 | -3.23049 | -3.05452 | -4.37883 | -3.98001 | -3.64826 | -3.64546 | -4.30326 | -3.00317 | -3.33075 |
| Q9D0G0 | Q9D0G0 | RT30_M | Mrps30 | Small ribosomal subunit protein mL65 | | | | 0.240317 | 0.753534 | 0.663953 | 0.557921 | -1.91435 | -1.48113 | 0.89637 | 0.273476 | 2.532005 | -1.73671 | -3.44997 | -1.0282 | -3.53664 | -0.30185 | -0.2208 | -2.60241 | -4.09134 | -0.6803 | -0.05584 | -0.63492 | -0.43468 |
| P59759 | P59759 | MRTFB_M | Mrtfb | Myocardin-related transcription factor B | | | | 0.233076 | -1.0836 | -0.64566 | 0.097889 | 0.180364 | 0.291385 | NaN | 0 | NaN | -3.38746 | -3.31885 | -4.09794 | -2.92561 | -3.56218 | -2.96375 | -3.94749 | -0.59161 | -3.89103 | -3.49324 | -3.24168 | -2.98517 |
| Q920Q6 | Q920Q6 | MSI2H_M | Msi2 | RNA-binding protein Musashi homolog 2 | | | | 0.46803 | 0.843514 | 1.24113 | 0.043253 | -0.043 | -0.13467 | 0.71984 | -0.28244 | -1.94906 | -0.23411 | -0.78287 | -1.23957 | -0.62591 | -1.08314 | -1.20802 | -0.73022 | -1.97379 | -0.97817 | -0.80132 | -0.73238 | -0.99391 |
| P26041 | P26041 | MOES_M | Msn | Moesin | | + | | 0.516702 | -0.45003 | -1.36967 | 1.713857 | -2.34503 | -7.08862 | 0.215002 | -0.36927 | -0.59982 | -2.55225 | -2.79518 | -3.94714 | -3.66125 | -3.1783 | -3.87222 | -1.91839 | -2.52898 | -1.16082 | -1.7575 | -3.66455 | -2.64743 |
| Q9D6Y7 | Q9D6Y7 | MSRA_M | Msra | Mitochondrial peptide methionine sulfoxide reductase | | | | 0.046387 | 0.134162 | 0.144005 | 0.815728 | 0.339182 | 2.254693 | 0.222149 | 0.755726 | 0.617975 | -1.06313 | -1.23674 | -0.88189 | -1.09711 | -1.4878 | -2.03726 | -0.3565 | -2.21169 | -1.43381 | -1.22356 | -3.70991 | -1.32661 |
| P61620 | Q2RLR1;P | S61A1_M | Sec61a1 | Protein transport protein Sec61 subunit alpha isoform 1 | + | | | 1.420643 | 0.712031 | 4.985113 | 0.687309 | 0.470134 | 1.850615 | 0.583818 | 0.268941 | 1.552551 | 3.455361 | 3.727407 | 3.011021 | 2.734606 | 3.357092 | 3.018136 | 2.835781 | 2.922925 | 2.615837 | 2.189522 | 2.882839 | 2.954507 |
| P02802 | P02802 | MT1_MOU | Mt1 | Metallothionein-1 | | | + | 0.49005 | 1.366778 | 1.298905 | 0.463858 | 0.723158 | 1.230247 | 1.219296 | 0.737011 | 3.883884 | -2.31573 | -2.35051 | -3.49784 | -2.56355 | -2.53699 | -2.40859 | -4.752 | -2.64778 | -3.39706 | -4.11064 | -3.38837 | -3.03123 |
| P02798 | P02798 | MT2_MOU | Mt2 | Metallothionein-2 | | | | 0.701923 | -0.94281 | -1.89453 | 0.496664 | -0.63889 | -1.31638 | 0.237032 | -0.41014 | -0.65566 | -3.95513 | -3.04409 | -4.87167 | -4.1042 | -3.48279 | -2.50102 | -2.35642 | -2.75718 | -4.1462 | -3.55189 | -2.96948 | -2.19405 |
| Q8K4B0 | Q8K4B0 | MTA1_M | Mta1 | Metastasis-associated protein MTA1 | | | | 0.012578 | 0.060938 | 0.040386 | 0.011581 | -0.01428 | -0.03723 | 0.753196 | -0.92133 | -2.05264 | -3.37422 | -1.44326 | -0.90108 | -0.40459 | -2.20168 | -1.58926 | -1.3101 | -3.62926 | -0.93112 | -0.34599 | -1.30232 | -0.64596 |
| Q9R190 | Q9R190 | MTA2_M | Mta2 | Metastasis-associated protein MTA2 | | | + | 0.390663 | 0.804503 | 1.042165 | 0.156216 | -0.13915 | -0.4482 | 1.271545 | -0.52347 | -4.14741 | -1.40245 | -0.62473 | -0.17475 | 0.354927 | -0.88585 | -0.64946 | -1.15124 | -2.48496 | 0.067237 | 0.39123 | -0.1999 | -0.28846 |
| Q924K8 | Q924K8 | MTA3_M | Mta3 | Metastasis-associated protein MTA3 | | | | 0.020438 | -0.0479 | -0.06508 | 0.032191 | -0.03961 | -0.10129 | 0.345113 | -0.56846 | -0.92712 | -2.62839 | -4.06199 | -2.55907 | -1.85286 | -2.38326 | -3.48277 | -3.46434 | -3.13024 | -2.3344 | -1.99831 | -2.63606 | -2.09304 |
| Q9CQ65 | Q9CQ65 | MTAP_M | Mtap | S-methyl-5'-thioadenosine phosphorylase | | + | | 0.054063 | -0.01333 | -0.16668 | 1.257234 | -0.2945 | -4.07378 | 0.013029 | -0.01407 | -0.04181 | 1.3823 | 1.242718 | -0.11457 | 0.026651 | -0.17939 | 0.473587 | 1.286738 | 1.364948 | 0.235041 | 0.266038 | 0.080079 | 0.242248 |
| Q9CSP9 | Q9CSP9 | TTC14_M | Ttc14 | Tetratricopeptide repeat protein 14 | + | | | 1.345459 | 0.702793 | 4.545975 | 0.119056 | 0.059774 | 0.349277 | 0.610955 | -0.44059 | -1.62867 | 1.764095 | 2.043711 | 1.068908 | 1.405134 | 0.396645 | 0.928207 | 1.267094 | 1.135127 | 1.209275 | 1.145219 | 1.052596 | 1.153432 |
| P07901 | P07901 | HS90A_M | Hsp90aa1 | Heat shock protein HSP 90-alpha | + | | | 1.44432 | 0.702617 | 5.130964 | 0.072872 | 0.055445 | 0.221109 | 0.164114 | 0.10212 | 0.468863 | 4.194882 | 4.165114 | 4.889988 | 5.320189 | 5.0355 | 4.703069 | 3.613506 | 3.341256 | 4.920754 | 5.178533 | 4.626412 | 4.907916 |
| P03930 | P03930 | ATP8_M | Mtatp8 | ATP synthase protein 8 | + | | | 1.235466 | 0.410032 | 3.963901 | 0.242158 | -0.1712 | -0.6686 | 0.566369 | 0.454867 | 1.504303 | 3.098276 | 3.116084 | 2.179689 | 1.841013 | 2.549407 | 2.639741 | 2.800205 | 2.59409 | 2.373609 | 1.989486 | 2.438692 | 1.840723 |
| Q791T5 | Q791T5 | MTCH1_M | Mtch1 | Mitochondrial carrier homolog 1 | | | | 0.155515 | 0.09826 | 0.446359 | 0.549746 | -0.26998 | -1.45881 | 0.197892 | 0.188096 | 0.556147 | 1.951603 | 1.577032 | 1.024038 | 0.796303 | 2.068238 | 1.435262 | 1.550366 | 1.781748 | 1.326044 | 1.034258 | 1.444394 | 1.682915 |
| Q91Z61 | Q91Z61 | DIRA1_M | Diras1 | GTP-binding protein Di-Ras1 | + | | | 1.667699 | 0.701964 | 6.710572 | 0.079518 | -0.03101 | -0.24 | 0.938858 | -0.15658 | -2.6865 | 1.595625 | 1.556993 | 1.487247 | 1.240591 | 1.455071 | 1.538808 | 0.977152 | 0.771538 | 1.433478 | 1.356382 | 1.694064 | 1.612972 |
| Q3UHU5 | Q3UHU5 | MTCL1_M | Mtcl1 | Microtubule cross-linking factor 1 | | | + | 0.032566 | -0.12885 | -0.10243 | 0.09285 | -0.1166 | -0.27741 | 1.059611 | -0.70132 | -3.16133 | -2.3145 | -1.66288 | -0.99944 | -0.22921 | -2.17494 | -1.73379 | -3.07478 | -0.6449 | -0.32928 | -0.66616 | -1.27672 | -1.22936 |
| P35980 | P35980 | RL18_M | Rpl18 | Large ribosomal subunit protein eL18 | + | + | | 1.503735 | 0.699314 | 5.513969 | 1.545828 | 0.416194 | 5.800808 | 0.59721 | 0.276225 | 1.589945 | 3.015978 | 2.968316 | 3.186738 | 3.235288 | 2.741451 | 2.485422 | 2.417399 | 2.168266 | 2.862336 | 2.727303 | 2.219758 | 2.454664 |
| Q80WJ7 | Q80WJ7 | LYRIC_M | Mtdh | Protein LYRIC | | | | 0.218386 | 0.149606 | 0.60842 | 0.993842 | 0.286923 | 2.895845 | 0.599327 | 0.16376 | 1.595885 | -0.36079 | -0.13238 | 0.387869 | 0.220791 | 0.045079 | 0.212312 | -0.17843 | -0.61395 | -0.03587 | 0.070682 | 0.024416 | -0.09454 |
| Q9CR88 | Q9CR88 | MTFP1_M | Mtfp1 | Mitochondrial fission process protein 1 | | | | 0.81138 | 0.657746 | 2.240295 | 0.034881 | 0.069513 | 0.10947 | 0.001011 | 0.000863 | 0.003288 | -2.38254 | -2.47703 | -2.22223 | -2.9256 | -1.58613 | -2.11097 | -3.3773 | -2.79776 | -3.17213 | -2.11472 | -1.83987 | -1.85896 |
| Q9CWE0 | Q9CWE0 | MFR1L_M | Mtfr1l | Mitochondrial fission regulator 1-like | | | | 0.001656 | -0.00205 | -0.00538 | 0.598803 | 0.438669 | 1.594414 | 0.35911 | 0.247426 | 0.962363 | -0.60477 | -1.35191 | 0.405643 | 0.303776 | 0.356984 | -0.03637 | -1.04746 | -0.90513 | -0.35433 | 0.186413 | 0.078465 | -0.25271 |
| Q922D8 | Q922D8 | C1TC_M | Mthfd1 | C-1-tetrahydrofolate synthase, cytoplasmic | | | | 0.538554 | -0.33371 | -1.42843 | 0.807798 | -0.68913 | -2.22847 | 0.164235 | -0.10894 | -0.46918 | -0.49538 | -0.43564 | -0.82282 | -1.4253 | -0.25388 | -0.11651 | 0.099906 | -0.3635 | -0.36503 | -0.50481 | 0.145543 | -0.29806 |
| Q3V3R1 | Q3V3R1 | C1TM_M | Mthfd1l | Monofunctional C1-tetrahydrofolate synthase, mitochondrial | | | | 0.376383 | 0.292321 | 1.005977 | 0.348789 | 0.411837 | 0.936369 | 0.365437 | -0.1729 | -0.97832 | -0.97931 | -1.07815 | -0.76286 | -1.48804 | -1.42616 | -1.34708 | -1.6074 | -1.0347 | -1.78622 | -1.28835 | -1.04146 | -1.38597 |
| Q9Z2C4 | Q9Z2C4 | MTMR1_M | Mtmr1 | Myotubularin-related protein 1 | | | | 0.264947 | -1.11434 | -0.72597 | 0.395206 | -0.14651 | -1.05371 | 0.419316 | -0.36204 | -1.11522 | -3.16777 | -0.2132 | 1.313407 | 1.510196 | 1.072673 | 1.59031 | -0.99296 | -0.15932 | 1.656548 | 1.460071 | 1.497566 | 1.889503 |
| Q9Z2D1 | Q9Z2D1 | MTMR2_M | Mtmr2 | Myotubularin-related protein 2 | | | + | 0.10857 | -0.05314 | -0.32076 | 0.119424 | 0.10233 | 0.350271 | 1.099499 | -0.23255 | -3.33098 | -0.63546 | -0.72003 | -0.00306 | 0.112534 | -0.24461 | -0.18199 | -0.46441 | -0.78479 | -0.33396 | 0.238779 | -0.04315 | 0.081653 |
| Q9Z2C9 | Q9Z2C9 | MTMR7_M | Mtmr7 | Myotubularin-related protein 7 | | | | NaN | 0 | NaN | 0.010001 | 0.007898 | 0.032202 | 0.667112 | -0.90334 | -1.79071 | -3.25755 | -3.16758 | -2.56793 | -2.29505 | -4.14682 | -3.16543 | -3.7997 | -3.09979 | -2.23557 | -2.6432 | -2.86981 | -2.63575 |
| Q9Z2D0 | Q9Z2D0 | MTMR9_M | Mtmr9 | Myotubularin-related protein 9 | | | | 0.062724 | -0.32705 | -0.19193 | 0.975429 | -0.54219 | -2.82451 | 0.311626 | -0.20665 | -0.84306 | -1.19544 | -3.79396 | -1.28428 | -0.91565 | -1.08937 | -1.47323 | -1.06519 | -3.27012 | -0.61139 | -0.50416 | -1.22711 | -0.92219 |
| Q9R0P5 | Q9R0P5 | DEST_MO | Dstn | Destrin | + | | | 2.015497 | 0.69057 | 10.10619 | 0.17469 | -0.12068 | -0.49637 | 0.388846 | 0.183628 | 1.037554 | 2.327907 | 2.462615 | 2.456625 | 2.079737 | 2.763601 | 3.071744 | 1.693177 | 1.716206 | 2.235233 | 2.542495 | 2.646956 | 2.821134 |
| P03893 | P03893 | NU2M_M | mt-Nd2 | NADH-ubiquinone oxidoreductase chain 2 | | | | 0.733886 | 0.836664 | 1.992338 | 0.8024 | 0.464337 | 2.210727 | 0.667782 | 1.161606 | 1.792683 | -1.2016 | -1.68694 | -3.14636 | -2.74031 | -2.20541 | -1.13781 | -2.62366 | -1.93821 | -3.35383 | -3.46151 | -3.20054 | -2.4659 |
| P03899 | P03899 | NU3M_M | mt-Nd3 | NADH-ubiquinone oxidoreductase chain 3 | | | | 0.774981 | 0.078522 | 2.121834 | 0.474541 | 0.613086 | 1.258151 | 0.197499 | 0.300091 | 0.555138 | 0.858159 | 0.790385 | -0.80418 | -1.77134 | 0.166114 | 0.64755 | 0.730877 | 0.760622 | -1.84082 | -1.96087 | 0.590755 | -0.37727 |
| P01837 | P01837 | IGKC_M | Igkc | Immunoglobulin kappa constant | + | | | 1.546341 | 0.686447 | 5.804381 | 0.569752 | 0.11126 | 1.513617 | 0.170448 | 0.480371 | 0.485357 | 0.198263 | -0.03202 | -1.52986 | -1.59232 | -0.52593 | -1.50852 | -0.63032 | -0.57633 | -1.73889 | -1.60581 | -2.35678 | -0.63842 |
| P03921 | P03921 | NU5M_M | mt-Nd5 | NADH-ubiquinone oxidoreductase chain 5 | | | | 0.605191 | -0.59047 | -1.61238 | 0.168163 | -0.17277 | -0.47941 | 0.133658 | 0.404178 | 0.388515 | 2.589315 | 2.420331 | 1.03661 | 1.008756 | 1.754347 | 3.290208 | 2.738963 | 3.451617 | 1.555555 | 0.835347 | 2.819908 | 1.416291 |
| P62774 | P62774 | MTPN_M | Mtpn | Myotrophin | | | | 0.667701 | -0.62454 | -1.79245 | 0.24584 | -0.10887 | -0.67789 | 0.140824 | 0.137332 | 0.407591 | -0.06758 | -0.73777 | 0.473262 | 0.612252 | 1.010206 | 1.45432 | 0.12639 | 0.317332 | 0.796409 | 0.506841 | 1.348341 | 0.841521 |
| Q9CZU3 | Q9CZU3 | MTREX_M | Mtrex | Exosome RNA helicase MTR4 | | | | 0.015097 | -0.06045 | -0.04835 | 0.548519 | -0.29034 | -1.45547 | 0.079003 | -0.10051 | -0.23854 | -2.71769 | -1.20154 | -2.20565 | -1.96227 | -2.89122 | -2.145 | -2.89345 | -0.90489 | -1.63556 | -1.95168 | -2.61334 | -2.22186 |
| Q3UHD3 | Q3UHD3 | MTUS2_M | Mtus2 | Microtubule-associated tumor suppressor candidate 2 homolog | | | | NaN | 0 | NaN | 0.011266 | -0.02272 | -0.03623 | 0.106465 | -0.16036 | -0.315 | -3.51142 | -3.72543 | -3.82023 | -2.59917 | -3.35561 | -3.28019 | -2.53484 | -3.28513 | -3.04304 | -3.33091 | -2.64986 | -3.66522 |
| P47802 | P47802 | MTX1_M | Mtx1 | Metaxin-1 | | | | 0.696078 | -1.64365 | -1.87691 | 0.352185 | 0.493772 | 0.944918 | 0.444164 | 0.994912 | 1.179143 | -3.25243 | -3.95611 | -3.00118 | -3.4264 | -1.38172 | -2.0293 | -1.15868 | -2.76255 | -4.18491 | -3.23021 | -1.92126 | -3.47958 |
| Q9CQV8 | Q9CQV8 | 1433B_M | Ywhab | 14-3-3 protein beta/alpha | + | + | | 1.769665 | 0.685713 | 7.572532 | 1.341565 | 0.477921 | 4.524185 | 0.976444 | 0.589189 | 2.828413 | 2.845707 | 2.966299 | 3.668261 | 3.759402 | 4.223638 | 3.807688 | 2.287849 | 2.152731 | 3.140608 | 3.331213 | 3.438305 | 3.414642 |
| D3YTP3 | D3YTP3 | D3YTP3_M | Mtx3 | Metaxin | + | | | 1.068629 | 0.188727 | 3.1991 | 0.001027 | -0.00163 | -0.00334 | 0.330312 | 0.345736 | 0.889936 | 0.490502 | 0.507195 | -1.10199 | -1.02344 | -0.20547 | -0.84012 | 0.368522 | 0.251721 | -0.57607 | -1.54612 | -1.09266 | -0.6444 |
| P28665 | P28665;P | MUG1_M | Mug1 | Murinoglobulin-1 | | | | 0.09328 | -0.28795 | -0.2786 | 0.234823 | -0.25404 | -0.65007 | 0.060385 | 0.073717 | 0.185144 | -2.93681 | -0.98474 | -1.48161 | -0.70974 | -1.23038 | -1.12771 | -1.33282 | -2.01282 | -0.78024 | -0.90304 | -1.6476 | -0.85792 |
| O35682 | O35682 | MYADM_M | Myadm | Myeloid-associated differentiation marker | + | + | | 1.024162 | -1.90798 | -3.01611 | 2.838715 | 0.84118 | 26.23522 | 0.95484 | 0.866576 | 2.746227 | -3.51856 | -2.43036 | -1.48755 | -1.55076 | -1.07942 | -1.01443 | -1.38918 | -0.74379 | -2.36573 | -2.35494 | -1.59963 | -2.22737 |
| Q7TPV4 | Q7TPV4 | MBB1A_M | Mybbp1a | Myb-binding protein 1A | | | | 0.731957 | 0.752777 | 1.986364 | 0.660252 | 0.294997 | 1.770568 | 0.78634 | -0.11898 | -2.15841 | -0.84844 | -1.46796 | -1.04267 | -1.01454 | -1.49822 | -1.6076 | -2.12931 | -1.69264 | -1.48962 | -1.15759 | -1.42702 | -1.44083 |
| Q7TPH6 | Q7TPH6 | MYCB2_M | Mycbp2 | E3 ubiquitin-protein ligase MYCBP2 | | | | 0.42699 | 0.882347 | 1.1349 | 0.276234 | 0.232117 | 0.754316 | 0.174052 | -0.26183 | -0.49471 | -1.96553 | -1.69888 | -1.4438 | -0.96504 | -2.11066 | -1.59489 | -1.9486 | -3.4805 | -1.62989 | -1.24318 | -2.05312 | -1.12877 |
| Q8C854 | Q8C854 | MYEF2_M | Myef2 | Myelin expression factor 2 | | | | 0.795497 | 1.196731 | 2.188153 | 0.3517 | 0.301012 | 0.943696 | 0.369638 | 0.871555 | 0.988928 | 0.497102 | 0.800364 | 1.31031 | 1.599536 | 2.230642 | 0.587139 | -0.02252 | -1.07347 | 0.869606 | 1.438217 | 0.218843 | 0.855827 |
| Q61879 | Q61879;Q | MYH10_M | Myh10 | Myosin-10 | | + | | 0.025549 | 0.019706 | 0.080929 | 2.015628 | 0.419708 | 10.10774 | 0.562281 | 0.691137 | 1.493074 | -0.40117 | -0.03613 | 0.508577 | 0.495641 | 0.745852 | 0.181595 | -0.07719 | -0.39952 | 0.041384 | 0.123417 | 0.139568 | -0.5944 |
| O08638 | O08638 | MYH11_M | Myh11 | Myosin-11 | | | | 0.152816 | 0.138829 | 0.439269 | 0.991461 | 1.141917 | 2.886548 | 0.229506 | -0.38449 | -0.63662 | -3.20677 | -3.30105 | -3.04449 | -2.45186 | -2.94056 | -4.08596 | -3.08023 | -3.70525 | -3.628 | -4.15219 | -3.32054 | -2.93701 |
| Q8VDD5 | Q8VDD5 | MYH9_M | Myh9 | Myosin-9 | | | | 0.782303 | -0.50615 | -2.14537 | 0.521787 | 0.278055 | 1.383279 | 0.903299 | 0.961932 | 2.55679 | -1.14447 | -1.42908 | -0.15657 | -0.27435 | 0.336294 | -0.24559 | -0.59245 | -0.9688 | -0.6857 | -0.30132 | -0.67804 | -1.15512 |
| Q3THE2 | Q3THE2;Q | ML12B_M | Myl12b | Myosin regulatory light chain 12B | | | | 0.038151 | -0.05018 | -0.11936 | 0.463846 | 0.122926 | 1.230217 | 0.263195 | 0.372895 | 0.721572 | 1.450735 | 1.212306 | 1.792799 | 1.640388 | 2.362459 | 1.802504 | 1.784876 | 0.978527 | 1.529037 | 1.658299 | 2.143955 | 1.275217 |
| Q60605 | Q60605 | MYL6_M | Myl6 | Myosin light polypeptide 6 | | | | 0.536206 | -0.25242 | -1.42208 | 0.014284 | 0.002047 | 0.045781 | 0.801844 | 0.605093 | 2.208902 | 0.650354 | 0.781026 | 1.503546 | 1.431458 | 2.018571 | 2.025509 | 1.133154 | 0.803073 | 1.439003 | 1.491908 | 1.690859 | 1.143035 |
| Q5DU14 | Q5DU14 | MYO16_M | Myo16 | Unconventional myosin-XVI | | | | NaN | 0 | NaN | 0.391807 | -0.25983 | -1.04507 | 0.237853 | -0.20523 | -0.65773 | -3.33242 | -2.66376 | -1.72258 | -1.54433 | -3.58541 | -4.1769 | -2.98147 | -3.66444 | -1.14152 | -1.60573 | -3.77541 | -3.57643 |
| Q9JMH9 | Q9JMH9 | MY18A_M | Myo18a | Unconventional myosin-XVIIIa | | | | 0.984731 | -0.42236 | -2.86039 | 0.448538 | 0.089378 | 1.190456 | 0.245736 | 0.35033 | 0.676717 | -0.89496 | -1.16897 | -0.42909 | -0.47379 | 0.364731 | -0.59374 | -0.66468 | -0.55454 | -0.61249 | -0.46915 | -0.26904 | -0.66063 |
| P46735 | P46735 | MYO1B_M | Myo1b | Unconventional myosin-Ib | | | | 0.388701 | 1.285521 | 1.037186 | 0.180824 | -0.67155 | -0.51224 | 0.187604 | 0.386822 | 0.529728 | -3.60486 | -1.14554 | -2.05769 | -3.16834 | -2.28147 | -1.71427 | -3.81605 | -3.50539 | -3.12903 | -0.75389 | -3.0576 | -1.71178 |
| P60469 | P60469 | LIPA3_M | Ppfia3 | Liprin-alpha-3 | + | | | 1.629631 | 0.68154 | 6.413082 | 0.167674 | -0.08407 | -0.47814 | 0.426004 | -0.47656 | -1.13237 | 0.130917 | 0.277738 | 2.457476 | 2.650353 | 1.389849 | 2.198098 | -0.40037 | -0.55406 | 2.490962 | 2.785014 | 2.153063 | 2.387998 |
| Q9D6A1 | Q9D6A1 | MYO1H_M | Myo1h | Unconventional myosin-Ih | | | | NaN | 0 | NaN | 0.342419 | 2.378926 | 0.920348 | 0.011616 | -0.13511 | -0.03734 | -2.68239 | -3.67566 | -3.51652 | 1.653102 | 1.440159 | -4.03982 | -3.16465 | -3.48558 | -3.31268 | -3.30859 | -3.52848 | 1.19904 |
| Q99104 | Q99104 | MYO5A_M | Myo5a | Unconventional myosin-Va | | + | | 0.573696 | -0.44245 | -1.5245 | 1.589978 | -0.64382 | -6.11629 | 0.051853 | -0.09459 | -0.16018 | 0.613057 | 0.474902 | 0.849277 | 0.889971 | 0.936176 | 2.003691 | 1.268307 | 0.704542 | 1.616724 | 1.410167 | 1.817204 | 1.31185 |
| Q64331 | Q64331 | MYO6_M | Myo6 | Unconventional myosin-VI | | | + | 0.431733 | -1.07739 | -1.14709 | 0.197577 | -0.79605 | -0.55534 | 1.046588 | 1.622229 | 3.107393 | -3.90467 | -2.83108 | -1.80631 | -3.81628 | -1.53009 | -1.18959 | -1.51976 | -3.06121 | -0.99311 | -3.03739 | -3.47558 | -2.48855 |
| Q8C170 | Q9QY06;Q | MYO9A_M | Myo9a | Unconventional myosin-IXa | | | | 0.184494 | 0.203254 | 0.521717 | 0.183605 | -0.04635 | -0.51942 | 0.717789 | -0.92418 | -1.94278 | 0.462861 | -0.19639 | 0.988007 | 1.132674 | -0.67127 | -0.02086 | -0.27769 | 0.137654 | 1.054438 | 1.158942 | 0.230934 | 0.925285 |
| Q8K3I4 | Q8K3I4 | MYRIP_M | Myrip | Rab effector MyRIP | | + | | 0.935684 | -1.65469 | -2.67475 | 1.0643 | 0.918432 | 3.180929 | 0.024414 | 0.087439 | 0.077422 | -3.61064 | -3.88532 | -0.55248 | -0.72749 | -2.10181 | -2.23475 | -1.4901 | -2.69649 | -1.83356 | -1.28326 | -3.38315 | -1.12829 |
| Q8BUR9 | Q8BUR9 | MZT1_MO | Mzt1 | Mitotic-spindle organizing protein 1 | | | | NaN | 0 | NaN | 0.298099 | -0.83436 | -0.80915 | 0.495548 | -0.41632 | -1.31342 | -4.27709 | -3.1002 | -3.21854 | -3.67318 | -3.27118 | -3.42434 | -2.99292 | -3.92341 | -3.61729 | -1.60572 | -3.23902 | -2.62385 |
| Q9QY36 | Q9QY36;Q | NAA10_M | Naa10 | N-alpha-acetyltransferase 10 | | | | 0.398874 | 1.355963 | 1.063036 | 0.117191 | 0.077312 | 0.344228 | 0.003545 | -0.00261 | -0.0115 | -3.318 | -1.30137 | -0.53255 | -0.4539 | -0.08568 | -0.2536 | -2.8844 | -4.4469 | -0.34941 | -0.79167 | -0.37789 | 0.043819 |
| Q80UM3 | Q80UM3 | NAA15_M | Naa15 | N-alpha-acetyltransferase 15, NatA auxiliary subunit | | + | | 0.275304 | 0.219393 | 0.751982 | 1.015706 | -0.3786 | -2.98221 | 0.085694 | 0.089813 | 0.257404 | -0.87495 | -0.80537 | -0.91498 | -1.15172 | -0.82686 | -0.32365 | -0.76988 | -1.34922 | -0.60887 | -0.70064 | -0.90681 | -0.42333 |
| Q6PEB6 | Q6PEB6 | PHOCN_M | Mob4 | MOB-like protein phocein | + | | | 1.345479 | 0.677107 | 4.546089 | 0.076706 | 0.072862 | 0.232027 | 0.587895 | -0.8618 | -1.5639 | 1.263101 | 1.40288 | 2.860849 | 3.488846 | 1.209574 | 2.255041 | 0.787411 | 0.524356 | 3.106102 | 3.097867 | 2.419705 | 2.768517 |
| Q8CES0 | Q8CES0 | NAA30_M | Naa30 | N-alpha-acetyltransferase 30 | | | + | 0.539525 | 0.630407 | 1.43106 | 0.513916 | 0.766282 | 1.362225 | 1.976219 | 0.237641 | 9.652668 | -1.96818 | -2.34377 | -1.0878 | -0.90654 | -1.13564 | -1.1497 | -3.18487 | -2.3879 | -2.31863 | -1.20828 | -1.40391 | -1.35672 |
| Q6PHQ8 | Q6PHQ8 | NAA35_M | Naa35 | N-alpha-acetyltransferase 35, NatC auxiliary subunit | | | | 0.195591 | 0.585158 | 0.550249 | 0.028001 | 0.022424 | 0.088477 | 0.450955 | -0.22222 | -1.19672 | -3.95506 | -1.85594 | -0.63036 | -0.57658 | -0.72947 | -1.06318 | -3.66194 | -3.31937 | -0.87791 | -0.37387 | -0.59261 | -0.7556 |
| P70670 | P70670 | NACAM_M | Naca | Nascent polypeptide-associated complex subunit alpha, muscle-specific form | | | | 0.782843 | 1.045028 | 2.147113 | 0.291919 | 0.739344 | 0.793657 | 0.040436 | 0.040958 | 0.126228 | -1.35631 | -1.94252 | -1.11574 | -2.06701 | -0.67309 | -1.28094 | -2.30588 | -3.083 | -3.13171 | -1.52973 | -1.13162 | -0.90432 |
| Q7TSZ8 | Q7TSZ8 | NACC1_M | Nacc1 | Nucleus accumbens-associated protein 1 | | + | | 0.513093 | 1.311606 | 1.360027 | 2.049247 | 0.568934 | 10.51237 | 0.321973 | -0.23423 | -0.86901 | 0.344106 | -0.60177 | 1.097376 | 1.164462 | -0.06342 | -0.15583 | -0.59997 | -2.28091 | 0.604457 | 0.519513 | -0.14094 | 0.390158 |
| Q8VBW6 | Q8VBW6 | ULA1_M | Nae1 | NEDD8-activating enzyme E1 regulatory subunit | | + | | 0.458887 | 1.176271 | 1.217309 | 1.965183 | 0.552696 | 9.528834 | 0.334348 | -0.4592 | -0.90007 | -0.84821 | -1.42683 | -1.12021 | -1.036 | -2.40513 | -1.39828 | -3.23575 | -1.39183 | -1.59091 | -1.6707 | -1.35971 | -1.5253 |
| Q9WTP6 | Q9WTP6 | KAD2_M | Ak2 | Adenylate kinase 2, mitochondrial | + | | | 1.435545 | 0.671676 | 5.076471 | 0.227885 | 0.072217 | 0.632519 | 0.700804 | 0.568691 | 1.891153 | 2.287349 | 2.396253 | 0.978538 | 1.188781 | 1.535101 | 1.463887 | 1.790712 | 1.549537 | 1.055996 | 0.966889 | 0.632208 | 1.229399 |
| Q8BXR5 | Q8BXR5 | NALCN_M | Nalcn | Sodium leak channel NALCN | | | | 0.717051 | 2.177293 | 1.940518 | 0.287586 | -0.19718 | -0.78279 | 0.966935 | -0.81103 | -2.79203 | 0.019065 | -1.1507 | -0.3435 | 0.15952 | -0.97407 | -0.41917 | -3.70063 | -1.7856 | 0.0913 | 0.119082 | 0.028381 | 0.200433 |
| Q99KQ4 | Q99KQ4 | NAMPT_M | Nampt | Nicotinamide phosphoribosyltransferase | | | | 0.70237 | 0.747395 | 1.895883 | 0.333385 | -0.43686 | -0.89765 | 0.067839 | -0.12242 | -0.20669 | -0.75395 | -1.44262 | -2.86313 | -2.10093 | -1.61308 | -0.7133 | -2.03763 | -1.65374 | -2.34784 | -1.7425 | -0.65552 | -1.42602 |
| Q6ZWX6 | Q6ZWX6 | IF2A_M | Eif2s1 | Eukaryotic translation initiation factor 2 subunit 1 | + | | | 1.461666 | 0.671197 | 5.240223 | 0.795405 | 0.765157 | 2.187853 | 0.288485 | 0.151705 | 0.785047 | -1.02168 | -0.76947 | -0.02119 | -0.49137 | -0.63006 | -0.8913 | -1.54432 | -1.58922 | -1.28037 | -0.76251 | -0.76997 | -1.0548 |
| Q9DB05 | Q9DB05 | SNAA_M | Napa | Alpha-soluble NSF attachment protein | | | | 0.568641 | -0.85069 | -1.51055 | 0.094652 | 0.232606 | 0.282413 | 0.604533 | 0.476713 | 1.610532 | -1.84465 | -0.74539 | -0.03662 | -1.67232 | -0.08805 | 0.127288 | -0.3216 | -0.56706 | -0.98961 | -1.18454 | -0.18137 | -0.73281 |
| Q99KC8 | Q99KC8 | VMA5A_M | Vwa5a | von Willebrand factor A domain-containing protein 5A | + | | + | 1.361675 | 0.641605 | 4.637705 | 0.312133 | -0.18357 | -0.84433 | 1.730178 | 0.602167 | 7.227029 | 0.058639 | 0.018076 | -1.20648 | -0.84462 | -0.14241 | -0.30242 | -0.4664 | -0.7401 | -0.96253 | -0.72143 | -0.84785 | -0.80131 |
| Q9CWZ7 | Q9CWZ7 | SNAG_M | Napg | Gamma-soluble NSF attachment protein | | | | 0.333703 | -0.21853 | -0.89845 | 0.326998 | -0.25142 | -0.88162 | 0.109935 | -0.12592 | -0.32449 | 1.068276 | 1.401898 | 1.40002 | 0.835926 | 1.362346 | 1.834985 | 1.276598 | 1.630643 | 1.411533 | 1.327248 | 2.032367 | 1.416797 |
| Q8BP47 | Q8BP47 | SYNC_M | Nars1 | Asparagine--tRNA ligase, cytoplasmic | | | | 0.546454 | -0.24408 | -1.44986 | 0.042525 | -0.04527 | -0.13249 | 0.493651 | 0.150971 | 1.30841 | -0.5094 | -0.38583 | -0.27008 | -0.94683 | 0.149835 | -0.00495 | -0.04692 | -0.36013 | -0.6106 | -0.51577 | 0.007054 | -0.16411 |
| Q8CH77 | Q8CH77 | NAV1_M | Nav1 | Neuron navigator 1 | | | | 0.069926 | 0.313383 | 0.212682 | 0.069829 | -0.04157 | -0.2124 | 0.551395 | -0.46335 | -1.4633 | -0.917 | -2.87959 | 0.325015 | 0.659842 | -0.36213 | 0.118089 | -1.11249 | -3.31086 | 0.635382 | 0.432618 | 0.134902 | 0.547756 |
| Q80TN7 | Q80TN7 | NAV3_M | Nav3 | Neuron navigator 3 | | | | 0.44963 | -0.97866 | -1.19329 | 0.335754 | 0.148083 | 0.903601 | 0.324428 | -0.69292 | -0.87517 | -3.32723 | -3.99676 | 0.317716 | 0.013273 | -1.8143 | -0.71573 | -3.43204 | -1.93464 | -0.0433 | 0.078122 | -1.14233 | -0.00185 |
| P67778 | P67778 | PHB1_M | Phb1 | Prohibitin 1 | + | | | 2.019432 | 0.63719 | 10.15275 | 0.0468 | -0.02521 | -0.14523 | 0.453581 | -0.19537 | -1.20353 | 5.61596 | 5.49044 | 4.931378 | 5.278532 | 5.126358 | 5.444786 | 4.915771 | 4.916248 | 5.132868 | 5.127466 | 5.449279 | 5.512607 |
| E9Q411 | E9Q411 | NBAS_M | Nbas | Neuroblastoma amplified sequence | + | | | 1.049131 | 0.364872 | 3.117872 | 0.846093 | -0.27892 | -2.35678 | 0.446882 | -0.45794 | -1.18617 | 1.912061 | 1.686534 | 1.856033 | 1.656187 | 0.969087 | 1.722849 | 1.40313 | 1.465723 | 2.098443 | 1.971613 | 1.720204 | 1.887609 |
| Q9EPN1 | Q9EPN1 | NBEA_M | Nbea | Neurobeachin | | | | 0.650664 | 0.332178 | 1.742581 | 0.117302 | -0.05329 | -0.34453 | 0.289491 | -0.17664 | -0.78757 | -0.80881 | -0.86683 | 0.486168 | 0.481087 | 0.370537 | 0.468494 | -0.9816 | -1.3584 | 0.38227 | 0.691556 | 0.377284 | 0.815022 |
| Q91X97 | Q91X97 | NCALD_M | Ncald | Neurocalcin-delta | + | | | 1.103105 | 1.032279 | 3.346655 | 0.366725 | 0.304376 | 0.981573 | 0.0254 | 0.012198 | 0.08047 | 1.484831 | 1.614283 | 2.470207 | 2.603948 | 2.127813 | 2.378812 | 0.215694 | 0.818861 | 1.929906 | 2.535495 | 2.156106 | 2.326124 |
| P13595 | P13595 | NCAM1_M | Ncam1 | Neural cell adhesion molecule 1 | | | | 0.017845 | 0.020442 | 0.056977 | 0.004208 | 0.006345 | 0.013636 | 0.866705 | 0.729983 | 2.42766 | 1.469779 | 1.548165 | 2.578204 | 1.658816 | 2.992752 | 2.456684 | 1.84516 | 1.131898 | 2.04008 | 2.18425 | 2.131023 | 1.858446 |
| O35136 | O35136 | NCAM2_M | Ncam2 | Neural cell adhesion molecule 2 | | | | 0.496996 | -0.16362 | -1.31725 | 0.236462 | -0.2055 | -0.65422 | 0.69742 | 0.796888 | 1.880947 | -0.49353 | -0.33349 | 0.207009 | -0.41902 | 0.808345 | -0.00666 | -0.15489 | -0.34489 | 0.125823 | 0.07317 | -0.28014 | -0.51194 |
| P55066 | P55066 | NCAN_M | Ncan | Neurocan core protein | | + | | 0.295364 | 0.397543 | 0.802292 | 1.106624 | 0.583272 | 3.362006 | 0.486504 | 0.292958 | 1.289561 | -0.34268 | -0.8176 | 0.889713 | 0.556173 | 0.971098 | 0.701203 | -0.54278 | -1.41259 | 0.187487 | 0.091856 | 0.725945 | 0.360441 |
| Q3UYV9 | Q3UYV9 | NCBP1_M | Ncbp1 | Nuclear cap-binding protein subunit 1 | | | | NaN | 0 | NaN | 0.435957 | 0.189623 | 1.157965 | 0.497902 | -0.64818 | -1.31965 | -2.58466 | -3.08185 | -2.37878 | -2.66183 | -3.78334 | -2.82938 | -2.67461 | -2.90619 | -2.79231 | -2.62754 | -2.7754 | -2.54097 |
| Q3X9C2 | Q3X9C2 | FBX50_M | Nccrp1 | F-box only protein 50 | | | | 0.691153 | 2.123302 | 1.86212 | 0.040673 | 0.166027 | 0.12694 | 0.044945 | 0.136167 | 0.139715 | 0.102629 | -1.19907 | -2.35217 | -2.07312 | -3.63761 | -1.77512 | -3.60779 | -1.73526 | -3.67912 | -1.07822 | -3.13001 | -2.55506 |
| Q9Z0E0 | Q9Z0E0 | NCDN_M | Ncdn | Neurochondrin | | | | 0.631636 | 0.394405 | 1.687603 | 0.511741 | 0.183668 | 1.356422 | 0.411191 | -0.1488 | -1.09444 | 2.88538 | 2.602595 | 3.228829 | 3.28153 | 4.102034 | 3.918416 | 2.535666 | 2.163499 | 2.938694 | 3.204329 | 4.058743 | 4.259308 |
| P47963 | P47963 | RL13_M | Rpl13 | Large ribosomal subunit protein eL13 | + | + | | 1.544231 | 0.634089 | 5.789678 | 1.100338 | 0.306531 | 3.334622 | 0.2523 | 0.249047 | 0.694164 | 2.840361 | 2.912035 | 3.184396 | 3.363784 | 2.896602 | 2.873477 | 2.3456 | 2.138617 | 2.947434 | 2.987684 | 2.277406 | 2.994578 |
| O55033 | O55033 | NCK2_M | Nck2 | Cytoplasmic protein NCK2 | | | | 0.944869 | 0.603296 | 2.70886 | 0.152516 | -0.51913 | -0.43848 | 0.217016 | -0.29787 | -0.60494 | 1.289396 | 0.890563 | 1.082325 | -1.25299 | -0.19409 | 0.784866 | 0.58585 | 0.387518 | 0.238173 | 0.629419 | 0.539648 | 0.646877 |
| P28660 | P28660 | NCKP1_M | Nckap1 | Nck-associated protein 1 | | + | | 0.154031 | 0.051882 | 0.442462 | 1.351941 | -0.31309 | -4.58245 | 0.063895 | -0.04053 | -0.19532 | 1.434563 | 1.509636 | 3.110176 | 3.153181 | 3.218761 | 3.522731 | 1.309131 | 1.531305 | 3.379915 | 3.509617 | 3.269988 | 3.552567 |
| Q9ESJ4 | Q9ESJ4 | SPN90_M | Nckipsd | NCK-interacting protein with SH3 domain | | + | | 0.47601 | -0.52627 | -1.262 | 1.976515 | -0.24679 | -9.65601 | 0.471581 | -0.12637 | -1.25041 | -0.48052 | -0.07037 | 0.688552 | 0.739507 | 0.795219 | 0.728134 | 0.613934 | -0.11228 | 0.962844 | 0.958787 | 0.792712 | 0.983379 |
| P09405 | P09405 | NUCL_M | Ncl | Nucleolin | + | | | 1.16008 | 0.807542 | 3.602077 | 0.744716 | 0.476932 | 2.026045 | 0.289873 | 0.301661 | 0.788526 | 2.294021 | 2.734951 | 3.039612 | 3.318436 | 2.748367 | 2.034956 | 1.747632 | 1.666255 | 2.512414 | 2.89177 | 1.95174 | 2.228261 |
| Q8VCM8 | Q8VCM8 | NCLN_M | Ncln | BOS complex subunit NCLN | | | | 0.171253 | 0.700739 | 0.487449 | 0.25819 | 0.252147 | 0.708986 | 0.029312 | 0.081763 | 0.092497 | -0.96211 | -0.48307 | 0.02782 | -0.67699 | 0.651587 | -1.11379 | -0.00586 | -2.8408 | -0.52883 | -0.62464 | -0.26551 | -0.36021 |
| Q91W39 | Q91W39 | NCOA5_M | Ncoa5 | Nuclear receptor coactivator 5 | | + | | NaN | 0 | NaN | 1.182997 | 0.993901 | 3.709096 | 0.381512 | 1.206522 | 1.01896 | -2.75012 | -2.94959 | -2.24199 | -1.71537 | -1.14169 | -3.50766 | -4.05888 | -3.5593 | -2.92286 | -3.0223 | -3.58186 | -3.48054 |
| Q8BNY6 | Q8BNY6 | NCS1_M | Ncs1 | Neuronal calcium sensor 1 | | | | 0.50286 | 0.312252 | 1.332797 | 0.710358 | 0.626988 | 1.920106 | 0.011576 | -0.01518 | -0.03721 | 1.198246 | 0.777319 | 1.042771 | 1.418402 | -0.12129 | 0.691282 | 0.778458 | 0.572603 | 0.33648 | 0.870718 | 0.264951 | 0.335395 |
| Q8VCB1 | Q8VCB1 | NDC1_M | Ndc1 | Nucleoporin NDC1 | | | | 0.307556 | 0.528992 | 0.832856 | 0.674745 | 1.239242 | 1.813244 | 0.250764 | -0.27739 | -0.6903 | -2.78111 | -1.75094 | -1.91747 | -2.78771 | -2.51984 | -2.35785 | -2.42338 | -3.16665 | -3.0648 | -4.11886 | -1.76785 | -2.55505 |
| Q9ERR1 | Q9CZA6;Q | NDEL1_M | Ndel1 | Nuclear distribution protein nudE-like 1 | | | | NaN | 0 | NaN | 0.231941 | 0.208035 | 0.642785 | 0.631984 | -0.76568 | -1.6886 | -3.62464 | -3.12366 | -0.68435 | -0.11713 | -1.51736 | -0.63484 | -3.40909 | -3.6194 | -0.7647 | -0.45285 | -0.41481 | -0.20605 |
| Q62433 | Q62433 | NDRG1_M | Ndrg1 | Protein NDRG1 | | | | 0.621574 | -0.32768 | -1.65882 | 0.230467 | 0.383951 | 0.639056 | 0.815236 | 0.419451 | 2.253061 | -0.55256 | -0.87467 | -1.44123 | -1.96136 | -1.65151 | -1.33569 | -0.50031 | -0.27155 | -1.54364 | -2.62686 | -1.81444 | -2.01166 |
| Q9QYG0 | Q9QYG0 | NDRG2_M | Ndrg2 | Protein NDRG2 | | | | 0.301929 | 0.251082 | 0.818751 | 0.410389 | 0.330412 | 1.092391 | 0.436151 | -0.26218 | -1.15846 | 3.432522 | 3.57948 | 3.117887 | 3.474744 | 2.859591 | 3.208674 | 3.55265 | 2.957188 | 2.721671 | 3.210136 | 3.440374 | 3.152248 |
| Q9QYF9 | Q9QYF9 | NDRG3_M | Ndrg3 | Protein NDRG3 | | | | 0.053065 | -0.05843 | -0.16374 | 0.024734 | -0.01922 | -0.07841 | 0.012051 | -0.01261 | -0.03872 | 0.243716 | 0.444166 | 0.909839 | 0.442773 | 0.681673 | 0.656874 | 0.744837 | 0.059903 | 0.621156 | 0.769892 | 1.007393 | 0.356379 |
| Q8BTG7 | Q8BTG7 | NDRG4_M | Ndrg4 | Protein NDRG4 | | + | | 0.159076 | -0.06594 | -0.45569 | 1.295171 | 0.584836 | 4.271418 | 0.693377 | 0.59026 | 1.86879 | 1.259998 | 1.474084 | 1.494597 | 1.508285 | 1.581039 | 1.534309 | 1.33561 | 1.530354 | 0.779857 | 1.053352 | 1.2824 | 0.652428 |

| | | | | | | | | | | | | | | | | | | | | | | | | | | | | |
|---|---|---|---|---|---|---|---|---|---|---|---|---|---|---|---|---|---|---|---|---|---|---|---|---|---|---|---|---|
| O35683 | O35683 | NDUA1_M | Ndufa1 | NADH dehydrogenase [ubiquinone] 1 alpha subcomplex subunit 1 | | | | 0.942244 | 0.658839 | 2.699083 | 0.286796 | 0.433749 | 0.780812 | 0.779523 | 0.682013 | 2.136417 | -1.14778 | -0.69218 | -0.7477 | -0.80423 | -0.37475 | 0.116623 | -1.66651 | -1.49112 | -1.76451 | -0.65492 | -1.01491 | -0.60725 |
| Q60870 | Q60870 | REEP5_MC | Reep5 | Receptor expression-enhancing protein 5 | + | | | 1.811206 | 0.628067 | 7.952913 | 0.267258 | 0.058928 | 0.731781 | 0.095771 | 0.05356 | 0.285519 | 0.95499 | 1.100927 | 0.247077 | 0.104949 | 0.674315 | 0.371602 | 0.430097 | 0.369686 | 0.154959 | 0.079211 | 0.358578 | 0.580218 |
| Q9D8B4 | Q9D8B4 | NDUAB_M | Ndufa11 | NADH dehydrogenase [ubiquinone] 1 alpha subcomplex subunit 11 | | | | 0.071829 | -0.14924 | -0.21813 | 0.078539 | -0.05863 | -0.23723 | 0.396684 | -0.62588 | -1.05746 | -0.71502 | -2.02638 | -1.21946 | -1.10603 | -1.4178 | -1.95952 | -1.02598 | -1.41693 | -1.34467 | -0.86356 | -0.53653 | -1.58904 |
| Q7TMF3 | Q7TMF3 | NDUAC_M | Ndufa12 | NADH dehydrogenase [ubiquinone] 1 alpha subcomplex subunit 12 | | | | 0.891095 | -0.17297 | -2.51325 | 0.167797 | 0.197684 | 0.478461 | 0.328409 | 0.331733 | 0.885157 | 2.206551 | 2.185211 | 1.827446 | 1.049218 | 2.157213 | 2.327254 | 2.300859 | 2.436841 | 1.101734 | 1.379562 | 2.275502 | 1.5455 |
| Q9ERS2 | Q9ERS2 | NDUAD_M | Ndufa13 | NADH dehydrogenase [ubiquinone] 1 alpha subcomplex subunit 13 | | | | 0.77245 | -0.34292 | -2.11373 | 0.247804 | -0.1109 | -0.68284 | 0.122092 | 0.15009 | 0.35748 | 3.632486 | 3.36285 | 2.817979 | 2.647523 | 3.463107 | 3.628711 | 3.930826 | 3.750343 | 2.981904 | 2.7054 | 3.80743 | 2.984207 |
| Q9CQ75 | Q9CQ75 | NDUA2_M | Ndufa2 | NADH dehydrogenase [ubiquinone] 1 alpha subcomplex subunit 2 | | | | 0.341477 | 0.148503 | 0.917981 | 0.53475 | -0.39237 | -1.41815 | 0.903828 | 0.228744 | 2.558687 | 1.179582 | 1.404031 | 0.711805 | 0.169896 | 1.396891 | 1.261205 | 1.259818 | 1.026789 | 0.777236 | 0.889208 | 1.158524 | 1.042084 |
| Q9CQ91 | Q9CQ91 | NDUA3_M | Ndufa3 | NADH dehydrogenase [ubiquinone] 1 alpha subcomplex subunit 3 | + | | + | 1.222039 | -0.64143 | -3.89736 | 0.009922 | -0.01449 | -0.03195 | 1.097476 | 0.612488 | 3.322215 | 0.4797 | 0.210387 | -0.16181 | -0.12289 | 0.263433 | 0.562942 | 0.891844 | 1.081097 | 0.325211 | -0.58093 | -0.30683 | -0.09177 |
| Q62425 | Q62425 | NDUA4_M | Ndufa4 | Cytochrome c oxidase subunit NDUFA4 | | + | | 0.903773 | 0.339133 | 2.558489 | 1.992945 | 1.023869 | 9.843329 | 0.768153 | 0.542272 | 2.100016 | 3.831081 | 3.612101 | 4.586266 | 4.789865 | 4.217709 | 3.761923 | 3.307746 | 3.457171 | 3.642834 | 3.685559 | 3.326119 | 3.568969 |
| Q9CPP6 | Q9CPP6 | NDUA5_M | Ndufa5 | NADH dehydrogenase [ubiquinone] 1 alpha subcomplex subunit 5 | | + | | 0.884545 | -0.34396 | -2.49007 | 1.329898 | -0.31197 | -4.45944 | 0.267965 | 0.255639 | 0.733557 | 1.405405 | 1.641794 | 0.795996 | 0.827969 | 1.362844 | 1.879344 | 1.939054 | 1.796072 | 1.192061 | 1.055848 | 1.599451 | 1.13146 |
| Q80XK6 | Q80XK6 | ATG2B_MC | Atg2b | Autophagy-related protein 2 homolog B | + | | | 2.378538 | 0.624431 | 15.41357 | 0.236332 | 0.168227 | 0.653889 | 0.342045 | -0.26947 | -0.91941 | -0.92554 | -0.906 | -0.07921 | 0.389141 | -0.50261 | -0.74486 | -1.57952 | -1.50089 | -0.1198 | 0.093276 | -0.62116 | -0.08737 |
| Q9Z1P6 | Q9Z1P6 | NDUA7_M | Ndufa7 | NADH dehydrogenase [ubiquinone] 1 alpha subcomplex subunit 7 | | + | | 0.07318 | -0.03069 | -0.22199 | 1.110056 | -0.64935 | -3.37703 | 0.34785 | 0.39185 | 0.934007 | 2.3951 | 2.537158 | 1.183848 | 0.984886 | 2.097164 | 2.869043 | 2.378216 | 2.615421 | 1.898273 | 1.56917 | 2.255757 | 1.92675 |
| Q9DCJ5 | Q9DCJ5 | NDUA8_M | Ndufa8 | NADH dehydrogenase [ubiquinone] 1 alpha subcomplex subunit 8 | | | | 0.078932 | -0.06272 | -0.23834 | 0.3188 | -0.14694 | -0.86105 | 0.221562 | 0.245975 | 0.616485 | 1.941718 | 1.563611 | 1.247625 | 1.071555 | 1.760708 | 1.969593 | 1.998443 | 1.632328 | 1.452724 | 1.160338 | 2.004259 | 1.234091 |
| Q9DC69 | Q9DC69 | NDUA9_M | Ndufa9 | NADH dehydrogenase [ubiquinone] 1 alpha subcomplex subunit 9, mitochondrial | | | | 0.345333 | -0.18923 | -0.92768 | 0.040354 | -0.04287 | -0.12598 | 0.468479 | 0.247168 | 1.242302 | 2.159741 | 2.199757 | 1.767089 | 1.151111 | 2.018616 | 2.302415 | 2.571978 | 2.16598 | 1.646654 | 1.357285 | 2.052809 | 1.773886 |
| Q9CR21 | Q9CR21 | ACPM_MC | Ndufab1 | Acyl carrier protein, mitochondrial | | | | 0.419412 | 0.298476 | 1.115464 | 0.29588 | 0.467496 | 0.803586 | 0.078692 | -0.08479 | -0.23766 | 1.651698 | 1.456829 | 2.017369 | 1.591591 | 1.543296 | 1.729993 | 1.504997 | 1.006578 | 0.795573 | 1.878395 | 2.065769 | 1.377097 |
| Q9CWG8 | Q9CWG8 | NDUF7_M | Ndufaf7 | Protein arginine methyltransferase NDUFAF7, mitochondrial | | | | 0.080903 | 0.068904 | 0.243914 | 0.611353 | -0.43231 | -1.62979 | 0.321474 | 0.059249 | 0.867756 | 0.364387 | 0.164959 | -0.42253 | 0.035407 | 0.280258 | 0.366582 | 0.460079 | -0.06854 | 0.372659 | 0.104832 | 0.211266 | 0.317077 |
| Q9DCS9 | Q9DCS9 | NDUBA_M | Ndufb10 | NADH dehydrogenase [ubiquinone] 1 beta subcomplex subunit 10 | | | | 0.890053 | -0.40223 | -2.50955 | 0.062003 | -0.03573 | -0.18984 | 0.137026 | 0.249808 | 0.397494 | 1.546291 | 1.856762 | 0.805292 | 0.429903 | 1.20708 | 1.55826 | 2.143656 | 2.063862 | 0.667307 | 0.639349 | 1.736291 | 0.529434 |
| Q8VDP6 | Q8VDP6 | CDIPT_MC | Cdipt | CDP-diacylglycerol--inositol 3-phosphatidyltransferase | + | | | 1.362238 | 0.611208 | 4.640919 | 0.304044 | -0.17106 | -0.82405 | 0.023011 | 0.015634 | 0.07308 | 2.523037 | 2.781424 | 2.410562 | 2.380322 | 2.785169 | 2.359729 | 2.015451 | 2.066595 | 2.773533 | 2.35947 | 2.579519 | 2.534111 |
| Q9CPU2 | Q9CPU2 | NDUB2_M | Ndufb2 | NADH dehydrogenase [ubiquinone] 1 beta subcomplex subunit 2, mitochondrial | + | | | 1.040639 | -0.26736 | -3.08298 | 0.387905 | -0.69171 | -1.03517 | 0.109242 | -0.14896 | -0.3226 | -0.11257 | 0.027561 | -1.79798 | -1.23462 | -0.90676 | -0.30084 | 0.173753 | 0.275962 | -0.21864 | -1.43053 | -0.10635 | -0.80332 |
| P03911 | P03911 | NU4M_MC | Mtnd4 | NADH-ubiquinone oxidoreductase chain 4 | + | | | 1.610492 | 0.610747 | 6.268205 | 0.099018 | -0.22221 | -0.29451 | 0.022643 | 0.07052 | 0.071937 | 1.675731 | 1.76755 | 0.284573 | -0.86597 | 0.82056 | 2.358385 | 1.024952 | 1.196836 | 0.419697 | -0.55669 | 2.127038 | 0.910867 |
| Q9CQC7 | Q9CQC7 | NDUB4_M | Ndufb4 | NADH dehydrogenase [ubiquinone] 1 beta subcomplex subunit 4 | | | | 0.164808 | 0.139527 | 0.470674 | 0.066342 | -0.08863 | -0.20238 | 0.265985 | 0.439691 | 0.728583 | 1.705968 | 1.338568 | 1.753041 | 0.948928 | 1.579446 | 2.38284 | 1.615403 | 1.150079 | 1.613204 | 1.266024 | 1.991827 | 1.091076 |
| Q9CQH3 | Q9CQH3 | NDUB5_M | Ndufb5 | NADH dehydrogenase [ubiquinone] 1 beta subcomplex subunit 5, mitochondrial | + | | | 1.198023 | -0.81032 | -3.78065 | 0.007597 | 0.007237 | 0.024528 | 0.063339 | 0.138005 | 0.193711 | 1.790258 | 1.449958 | 0.754855 | 0.539403 | 1.105625 | 1.58585 | 2.300092 | 2.56077 | 0.91456 | 0.365224 | 1.878476 | 0.53699 |
| Q3UIU2 | Q3UIU2 | NDUB6_M | Ndufb6 | NADH dehydrogenase [ubiquinone] 1 beta subcomplex subunit 6 | | | | 0.941167 | -0.47599 | -2.69508 | 0.077452 | 0.164842 | 0.234145 | 0.24051 | 0.539285 | 0.664442 | 0.120295 | -0.23293 | -2.05439 | -1.3184 | -1.43218 | -0.88101 | 0.419771 | 0.419566 | -3.20442 | -2.36306 | -0.93246 | -2.4593 |
| Q9CR61 | Q9CR61 | NDUB7_M | Ndufb7 | NADH dehydrogenase [ubiquinone] 1 beta subcomplex subunit 7 | + | | | 1.192051 | -0.55614 | -3.75208 | 0.08463 | -0.09204 | -0.25441 | 0.020447 | -0.03891 | -0.06511 | 0.014436 | 0.137371 | -0.14748 | -0.625 | -0.18634 | 0.515481 | 0.766922 | 0.497169 | -0.0224 | -0.566 | 0.687313 | -0.28034 |
| Q9D6J5 | Q9D6J5 | NDUB8_M | Ndufb8 | NADH dehydrogenase [ubiquinone] 1 beta subcomplex subunit 8, mitochondrial | | | | 0.956626 | -1.0263 | -2.75296 | 0.229196 | -0.29434 | -0.63584 | 0.127898 | 0.244309 | 0.373099 | 1.54512 | 1.094578 | 1.086982 | 0.371692 | 1.100555 | 1.593865 | 2.643182 | 2.049107 | 1.317591 | 0.729766 | 1.709479 | 0.496324 |
| P10637 | P10637 | TAU_MOU | Mapt | Microtubule-associated protein tau | + | | | 1.808703 | 0.610341 | 7.929487 | 0.148876 | -0.06473 | -0.42889 | 0.303296 | -0.18068 | -0.82218 | 2.352625 | 2.495313 | 3.300226 | 3.295881 | 2.930987 | 3.266819 | 1.784739 | 1.842517 | 3.211871 | 3.513703 | 3.421357 | 3.137814 |
| Q9CQ54 | Q9CQ54 | NDUC2_M | Ndufc2 | NADH dehydrogenase [ubiquinone] 1 subunit C2 | | | | 0.124676 | 0.11309 | 0.364442 | 0.071696 | -0.06171 | -0.21775 | 0.034708 | -0.04457 | -0.10894 | 2.450971 | 2.086083 | 1.756314 | 1.579837 | 1.833761 | 1.99618 | 2.406447 | 1.904427 | 1.999113 | 1.460464 | 2.360469 | 1.558604 |
| Q91VD9 | Q91VD9 | NDUS1_M | Ndufs1 | NADH-ubiquinone oxidoreductase 75 kDa subunit, mitochondrial | | | | 0.024207 | -0.00927 | -0.07678 | 0.377507 | -0.19552 | -1.00882 | 0.614509 | 0.265868 | 1.638735 | 3.113971 | 3.035939 | 2.757535 | 2.370814 | 3.265466 | 3.143443 | 3.198499 | 2.969954 | 2.772864 | 2.746521 | 3.088917 | 2.788255 |
| Q91WD5 | Q91WD5 | NDUS2_M | Ndufs2 | NADH dehydrogenase [ubiquinone] iron-sulfur protein 2, mitochondrial | | | | 0.133973 | 0.085139 | 0.389358 | 0.181967 | 0.140909 | 0.515195 | 0.279446 | 0.143849 | 0.762375 | 2.230961 | 2.457063 | 2.35872 | 1.858718 | 2.548182 | 2.3357 | 2.446046 | 2.071702 | 1.85688 | 2.07874 | 2.454024 | 2.142159 |
| Q9DCT2 | Q9DCT2 | NDUS3_M | Ndufs3 | NADH dehydrogenase [ubiquinone] iron-sulfur protein 3, mitochondrial | | | | 0.8238 | 0.265094 | 2.281568 | 0.083079 | -0.12361 | -0.25005 | 0.134402 | 0.121296 | 0.390501 | 2.431629 | 2.396621 | 1.805119 | 1.387467 | 2.367139 | 2.405744 | 2.034168 | 2.263895 | 1.271846 | 2.167954 | 2.575161 | 1.955131 |
| Q8R349 | Q8R349 | CDC16_M | Cdc16 | Cell division cycle protein 16 homolog | + | | | 1.343195 | 0.601912 | 4.533295 | 0.449756 | 0.376111 | 1.193612 | 0.055167 | -0.06868 | -0.16991 | -1.29572 | -1.17826 | -0.43736 | -0.20319 | -0.64548 | -0.2938 | -1.71982 | -1.95798 | -0.98893 | -0.40384 | -0.76489 | -0.03704 |
| Q99LY9 | Q99LY9 | NDUS5_M | Ndufs5 | NADH dehydrogenase [ubiquinone] iron-sulfur protein 5 | | | | 0.22435 | 0.108771 | 0.623557 | 0.388982 | -0.11777 | -1.0379 | 0.239363 | 0.306072 | 0.661545 | 2.599979 | 2.371717 | 2.116469 | 1.922159 | 2.339452 | 2.889778 | 2.508994 | 2.24516 | 2.195692 | 2.078468 | 2.680486 | 1.9366 |
| P52503 | P52503 | NDUS6_M | Ndufs6 | NADH dehydrogenase [ubiquinone] iron-sulfur protein 6, mitochondrial | | | | 0.110718 | -0.10424 | -0.32663 | 0.200321 | -0.24873 | -0.56236 | 0.260884 | 0.208504 | 0.715761 | 2.299427 | 2.271641 | 1.383366 | 1.141007 | 1.835135 | 1.877182 | 2.070924 | 2.708634 | 1.936286 | 1.085547 | 1.938199 | 1.357109 |
| Q9DC70 | Q9DC70 | NDUS7_M | Ndufs7 | NADH dehydrogenase [ubiquinone] iron-sulfur protein 7, mitochondrial | | | | 0.242557 | -0.10548 | -0.66961 | 0.39263 | -0.26066 | -1.04716 | 0.202379 | 0.148324 | 0.567629 | 1.664364 | 1.439799 | 1.232305 | 0.963955 | 1.570107 | 1.698549 | 1.768042 | 1.547079 | 1.568459 | 1.149127 | 1.739294 | 1.232714 |
| Q8K3J1 | Q8K3J1 | NDUS8_M | Ndufs8 | NADH dehydrogenase [ubiquinone] iron-sulfur protein 8, mitochondrial | | | | 0.246612 | 0.177675 | 0.679834 | 0.502045 | 0.261517 | 1.330634 | 0.286939 | 0.196409 | 0.781169 | 1.504721 | 1.522941 | 1.231892 | 0.964747 | 1.567509 | 1.811059 | 1.074965 | 1.597348 | 0.692633 | 0.980972 | 1.712847 | 1.272903 |
| Q6ZQ82 | Q6ZQ82 | RHG26_M | Arhgap26 | Rho GTPase-activating protein 26 | + | | | 1.835979 | 0.601346 | 8.188359 | 0.411369 | -0.84329 | -1.09489 | 0.527057 | -1.46117 | -1.39743 | 0.148306 | 0.289931 | 3.523098 | 2.057724 | 0.981358 | 3.02747 | -0.40169 | -0.36276 | 3.39626 | 3.871133 | 3.249565 | 3.681597 |
| Q9D6J6 | Q9D6J6 | NDUV2_M | Ndufv2 | NADH dehydrogenase [ubiquinone] flavoprotein 2, mitochondrial | | | | 0.437939 | -0.19298 | -1.16307 | 0.585822 | -0.40537 | -1.55813 | 0.071367 | -0.12765 | -0.21681 | 2.239347 | 2.025002 | 1.407186 | 1.280316 | 1.915371 | 2.417951 | 2.198488 | 2.451818 | 1.496809 | 2.001435 | 2.826773 | 1.761853 |
| Q8BK30 | Q8BK30 | NDUV3_M | Ndufv3 | NADH dehydrogenase [ubiquinone] flavoprotein 3, mitochondrial | | | | 0.39945 | 0.17945 | 1.064503 | 0.289738 | -0.57599 | -0.78819 | 0.329622 | -0.33188 | -0.8882 | 0.103753 | 0.429843 | -1.89217 | -2.49149 | -0.93147 | -1.64367 | 0.044519 | 0.130176 | -0.94933 | -2.28235 | -0.84251 | -1.06887 |
| Q91ZP9 | Q91ZP9 | NECA2_M | Necab2 | N-terminal EF-hand calcium-binding protein 2 | | | | 0.674045 | 1.591303 | 1.811172 | 0.098736 | 0.088629 | 0.293726 | 0.323968 | -0.02639 | -0.87401 | 0.320352 | -0.95519 | 0.26078 | 0.476054 | 0.686008 | 0.674633 | -1.30441 | -2.51303 | 0.561677 | -0.0021 | 0.736362 | 0.677057 |
| Q8CHG7 | Q8CHG7 | RPGF2_M | Rapgef2 | Rap guanine nucleotide exchange factor 2 | + | | | 1.368183 | 0.600265 | 4.67497 | 0.573855 | -0.24434 | -1.52494 | 0.785243 | -0.401 | -2.15486 | -0.35309 | -0.38517 | 1.165794 | 1.315269 | 1.228067 | 1.299359 | -1.09679 | -0.842 | 1.343141 | 1.626606 | 1.482069 | 1.847361 |
| P46935 | P46935 | NEDD4_M | Nedd4 | E3 ubiquitin-protein ligase NEDD4 | | | | 0.547946 | 0.355883 | 1.453912 | 0.012906 | -0.01112 | -0.04143 | 0.375737 | 0.393867 | 1.004345 | 0.174949 | -0.27376 | 0.255778 | 0.581388 | 0.857608 | 0.132581 | -0.3074 | -0.50317 | 0.216204 | 0.643206 | -0.04836 | 0.250813 |
| Q8CFI0 | Q8CFI0 | NEDD4L_M | Nedd4l | E3 ubiquitin-protein ligase NEDD4-like | | | | 0.196659 | 0.128642 | 0.552985 | 0.185392 | 0.03436 | 0.52403 | 0.26416 | 0.289175 | 0.723996 | -0.76399 | -0.56954 | 0.126133 | -0.00281 | 1.501575 | 0.70321 | -0.58406 | -1.00675 | 0.015364 | 0.039238 | 0.799561 | 0.826874 |
| P29595 | P29595 | NEDD8_M | Nedd8 | NEDD8 | | | | 0.122585 | 0.187865 | 0.358809 | 0.530646 | 0.679171 | 1.407085 | 0.164075 | -0.15098 | -0.46876 | -2.06063 | -1.13363 | -0.26888 | -1.1215 | -0.67071 | -1.31366 | -2.02852 | -1.54147 | -1.60072 | -1.14799 | -0.86113 | -0.82126 |
| P19246 | P19246 | NFH_MOU | Nefh | Neurofilament heavy polypeptide | | | + | 0.804637 | -1.78836 | -2.21807 | 0.28231 | -0.03478 | -0.76956 | 1.476288 | 0.837092 | 5.333944 | 2.146858 | 1.67964 | 0.003349 | -0.08634 | 0.855839 | 1.128763 | 4.473291 | 2.929925 | -0.00108 | -0.01235 | 0.232716 | 0.077703 |
| P08551 | P08551 | NFL_MOU | Nefl | Neurofilament light polypeptide | | | + | 0.762378 | -1.87297 | -2.08166 | 0.944271 | -0.48165 | -2.70663 | 1.812 | 0.837986 | 7.960364 | 3.582513 | 2.783618 | 1.627548 | 1.281948 | 2.9143 | 3.019559 | 5.862261 | 4.249816 | 1.978904 | 1.893889 | 2.220113 | 2.037774 |
| P08553 | P08553 | NFM_MOL | Nefm | Neurofilament medium polypeptide | | | + | 0.663428 | -1.62766 | -1.77988 | 0.890454 | -0.59712 | -2.51097 | 2.747154 | 0.939508 | 23.60443 | 2.40076 | 1.695652 | 0.398807 | -0.00224 | 1.9231 | 1.864993 | 4.519648 | 2.832078 | 0.923236 | 0.667569 | 0.927333 | 0.981743 |
| Q80Z24 | Q80Z24 | NEGR1_M | Negr1 | Neuronal growth regulator 1 | | | | 0.519593 | -1.22765 | -1.3774 | 0.464968 | 0.736349 | 1.233142 | 0.938363 | -0.79219 | -2.68467 | -3.89087 | -2.12183 | -0.63356 | -1.42764 | -2.07944 | -1.88099 | -1.88825 | -1.66916 | -2.21297 | -1.32093 | -0.91012 | -1.46592 |
| P97798 | P97798 | NEO1_MO | Neo1 | Neogenin | | | | 0.237121 | 0.743399 | 0.655883 | 0.394525 | -0.34362 | -1.05198 | 0.443938 | 0.794911 | 1.178558 | -3.18091 | -1.09621 | -1.06537 | -0.97224 | 0.297367 | -0.90628 | -2.4368 | -3.32712 | -0.35188 | -0.99849 | -1.40388 | -0.79486 |
| Q8BNJ6 | Q8BNJ6 | NETO2_M | Neto2 | Neuropilin and tolloid-like protein 2 | | | | NaN | 0 | NaN | 0.201333 | -0.2449 | -0.56495 | 0.127862 | -0.26542 | -0.373 | -3.64037 | -4.03949 | -2.59176 | -2.85222 | -2.10737 | -3.52883 | -2.99195 | -2.82883 | -2.89055 | -2.06363 | -2.51774 | -2.58761 |
| Q62414 | Q62414 | NDF2_MO | Neurod2 | Neurogenic differentiation factor 2 | | | | NaN | 0 | NaN | 0.416952 | 0.546838 | 1.109168 | 0.193478 | 0.298158 | 0.544827 | -3.04375 | -3.23684 | -3.03387 | -2.38505 | -1.61317 | -2.48639 | -3.51824 | -3.46542 | -2.88506 | -3.62754 | -2.67787 | -2.018 |
| Q04690 | Q04690 | NF1_MOU | Nf1 | Neurofibromin | + | + | | 1.141038 | 0.420168 | 3.515055 | 1.067951 | 0.197713 | 3.196251 | 0.419886 | -0.45571 | -1.11668 | 1.66109 | 1.475991 | 1.884372 | 2.006621 | 0.715438 | 1.267799 | 1.072723 | 1.224022 | 1.738287 | 1.75728 | 1.146888 | 1.747766 |
| Q810U3 | Q810U3 | NFASC_M | Nfasc | Neurofascin | | | | 0.269765 | -0.26902 | -0.73808 | 0.085429 | 0.078115 | 0.256661 | 0.502892 | 0.798836 | 1.33288 | 0.571746 | 0.745355 | 1.115602 | 0.507002 | 2.057027 | 0.858413 | 1.281573 | 0.57357 | 0.738726 | 0.727648 | 0.653537 | 0.664231 |
| P97863 | P97863 | NFIB_MOL | Nfib | Nuclear factor 1 B-type | | | | 0.692065 | 1.453068 | 1.864855 | 0.856375 | -0.33921 | -2.39197 | 0.201795 | -0.1579 | -0.56613 | -0.02597 | 0.875206 | 0.778292 | 1.03915 | 1.284039 | 1.024501 | -1.66414 | -0.39276 | 1.192264 | 1.303599 | 1.065288 | 1.559057 |
| Q9Z1J3 | Q9Z1J3 | NFS1_MOI | Nfs1 | Cysteine desulfurase | + | | | 1.297313 | -0.37789 | -4.28281 | 0.501728 | -0.33697 | -1.32979 | 0.050377 | 0.040799 | 0.155824 | -0.33901 | -0.25128 | -0.78686 | -0.48181 | -0.09836 | 0.150059 | 0.159303 | 0.006187 | -0.09501 | -0.49972 | -0.24544 | 0.215539 |
| Q9QZ23 | Q9QZ23 | NFU1_MO | Nfu1 | NFU1 iron-sulfur cluster scaffold homolog, mitochondrial | | | | 0.083555 | 0.378224 | 0.251391 | 0.218336 | 0.619461 | 0.608292 | 0.339703 | -0.2289 | -0.91352 | -3.80821 | -1.09402 | -1.46968 | -1.82023 | -2.03702 | -1.54681 | -3.47887 | -2.17981 | -3.26758 | -1.26125 | -1.511 | -1.61504 |
| B1AY10 | B1AY10 | NFX1_MOI | Nfx1 | Transcriptional repressor NF-X1 | | | | 0.215059 | 0.408763 | 0.599962 | 0.000731 | 0.001354 | 0.002378 | 0.514454 | -0.39341 | -1.36366 | -3.25166 | -1.93172 | -2.45744 | -1.43542 | -2.76977 | -2.32381 | -2.83126 | -3.16964 | -2.19864 | -1.69694 | -2.33643 | -1.97033 |
| Q8CHT1 | Q8CHT1 | NGEF_MO | Ngef | Ephexin-1 | | | | 0.489273 | 0.17146 | 1.296856 | 0.488166 | -0.1738 | -1.29394 | 0.292662 | -0.17069 | -0.79552 | -0.54 | -0.68718 | 1.001314 | 0.956329 | 0.12868 | -0.29926 | -0.67521 | -0.89489 | 1.020199 | 1.285042 | 0.069439 | 0.101361 |
| P70441 | P70441 | NHRF1_M | Nherf1 | Na(+)/H(+) exchange regulatory cofactor NHE-RF1 | | | | 0.037099 | 0.090304 | 0.11618 | 0.354958 | 0.489722 | 0.9519 | 0.121462 | 0.220747 | 0.355778 | -1.90153 | -0.53592 | 0.047896 | -0.981 | -0.65674 | -0.69981 | -1.68042 | -0.93764 | -0.95209 | -0.96046 | -0.27893 | -1.51911 |
| Q9JHL1 | Q9JHL1 | NHRF2_M | Nherf2 | Na(+)/H(+) exchange regulatory cofactor NHE-RF2 | | | | 0.38476 | -0.89321 | -1.02719 | 0.003026 | -0.00317 | -0.00982 | 0.00949 | 0.005468 | 0.030576 | -2.48933 | -2.16498 | -2.45846 | -1.92637 | -1.45113 | -1.22069 | -2.28825 | -0.57964 | -2.00667 | -2.37182 | -1.2046 | -1.47816 |
| Q9CRB2 | Q9CRB2 | NHP2_MO | Nhp2 | H/ACA ribonucleoprotein complex subunit 2 | | | | 0.601509 | -0.44876 | -1.60202 | 0.128912 | -0.48398 | -0.37582 | 0.089268 | -0.16927 | -0.26742 | -4.09078 | -3.58951 | -5.28668 | -2.71553 | -3.36169 | -3.2286 | -3.26629 | -3.51648 | -3.44148 | -3.59277 | -3.75534 | -2.49642 |
| B1AXH1 | B1AXH1 | NHSL2_M | Nhsl2 | NHS-like protein 2 | | | + | NaN | 0 | NaN | 0.064944 | -0.08859 | -0.19835 | 1.000114 | -1.10023 | -2.92043 | -3.50113 | -3.27043 | -0.56254 | 0.022545 | -0.90538 | -0.42572 | -2.79388 | -2.88472 | -0.51887 | 0.156047 | 0.144145 | 0.725213 |
| E9Q137 | E9Q137 | TX264_MC | Tex264 | Testis-expressed protein 264 homolog | + | | + | 1.476188 | 0.599964 | 5.333298 | 0.95563 | 0.399938 | 2.749202 | 2.221529 | 0.430536 | 12.847 | 2.391848 | 2.594739 | 1.526104 | 1.766867 | 1.514613 | 1.579223 | 1.844712 | 1.941947 | 1.328225 | 1.164871 | 1.107468 | 1.125298 |
| Q3U0M1 | Q3U0M1 | TPPC9_M | Trappc9 | Trafficking protein particle complex subunit 9 | + | | | 1.63429 | 0.596628 | 6.448823 | 0.0929 | 0.061927 | 0.277544 | 0.069721 | -0.06845 | -0.21209 | -0.56994 | -0.38723 | 0.117818 | 0.439552 | -0.00365 | 0.159019 | -1.0606 | -1.08983 | 0.062144 | 0.371374 | -0.1662 | 0.458477 |
| O55126 | O55126 | NIPS2_MO | Nipsnap2 | Protein NipSnap homolog 2 | | | + | 0.4643 | 0.23019 | 1.231401 | 0.017172 | -0.02662 | -0.05487 | 1.670588 | 0.400167 | 6.73367 | 1.763895 | 2.130531 | 1.304603 | 2.240354 | 1.576266 | 1.510239 | 1.753608 | 1.680439 | 1.92731 | 1.670883 | 1.093672 | 1.1925 |
| Q80TM9 | Q80TM9 | NISCH_M | Nisch | Nischarin | | | | 0.025922 | 0.069861 | 0.08208 | 0.485362 | 0.392463 | 1.286553 | 0.410913 | -0.37159 | -1.09373 | -0.80002 | -1.55575 | -0.13623 | 0.098563 | -0.60334 | -0.86639 | -0.48509 | -2.01039 | -0.69285 | -0.12974 | -0.67653 | -0.05002 |
| Q8VDK1 | Q8VDK1 | NIT1_MOU | Nit1 | Deaminated glutathione amidase | | | | 0.642363 | 0.630103 | 1.718504 | 0.031877 | -0.04948 | -0.10034 | 0.12016 | -0.2727 | -0.35226 | -0.45119 | -0.30569 | -1.58481 | -0.71145 | -1.74315 | -0.19487 | -1.36791 | -0.64918 | -1.32772 | -0.86958 | -0.69797 | -0.69465 |
| Q9D0F4 | Q9D0F4;Q | NKAP_MO | Nkap | NF-kappa-B-activating protein | | | | 0.917276 | 2.463813 | 2.607262 | 0.623241 | 0.636001 | 1.663576 | 0.709571 | 1.069772 | 1.917714 | -0.25308 | 0.621309 | -0.54335 | 0.124289 | -0.41141 | -0.63535 | -1.44193 | -3.11746 | -1.03188 | -0.65918 | -2.13963 | -1.04667 |
| E9PUQ3 | E9PUQ3 | E9PUQ3_M | Nkapd1 | NKAP domain containing 1 | | | + | 0.675851 | 1.16487 | 1.816519 | 0.592594 | 0.262854 | 1.57702 | 1.059737 | 0.539561 | 3.161861 | 0.584486 | 1.500286 | 0.657303 | 0.748639 | 1.371888 | 1.037767 | 0.326458 | -0.57143 | 0.279818 | 0.600417 | 0.630466 | 0.700067 |
| Q8CEC5 | Q8CEC5 | KBRS1_M | Nkiras1 | NF-kappa-B inhibitor-interacting Ras-like protein 1 | | | | 0.346346 | 0.47783 | 0.930223 | 0.112785 | 0.139406 | 0.332259 | 0.208305 | -0.22241 | -0.58276 | -2.38502 | -1.81334 | -0.54033 | -0.40938 | -1.06949 | -0.94488 | -2.15022 | -3.00381 | -1.02869 | -0.19983 | -1.16131 | -0.40824 |
| Q9CR56 | Q9CR56 | KBRS2_M | Nkiras2 | NF-kappa-B inhibitor-interacting Ras-like protein 2 | + | | | 1.105359 | 0.823887 | 3.356485 | 0.158538 | 0.289591 | 0.454284 | 0.590512 | -0.20312 | -1.5712 | -2.69599 | -2.21628 | -1.5018 | -0.58359 | -1.84551 | -1.85074 | -3.33219 | -3.22786 | -1.77453 | -0.89003 | -1.77425 | -1.51575 |
| P30415 | P30415 | NKTR_MOI | Nktr | NK-tumor recognition protein | | | + | 0.810807 | 2.658003 | 2.238402 | 0.856417 | -0.83905 | -2.39212 | 1.060579 | 1.041573 | 3.165373 | 0.020359 | 0.755409 | -1.22875 | -0.86187 | 0.857811 | 0.332788 | -3.39927 | -1.14097 | 0.092709 | -0.50522 | -0.24787 | -0.64468 |
| Q69ZK9;Q | Q69ZK9;Q | NLGN1_M | Nlgn1;Nlgn2 | Neuroligin-2 | | | | 0.546821 | -1.55766 | -1.45085 | 0.033662 | 0.031239 | 0.10577 | 0.437267 | 0.672528 | 1.16134 | -4.30986 | -2.4238 | -1.74492 | -1.74861 | -0.35936 | -1.45008 | -1.29601 | -2.32234 | -1.48266 | -2.07334 | -1.38247 | -1.77202 |
| Q8BYM5 | Q8BYM5 | NLGN3_M | Nlgn3 | Neuroligin-3 | + | | | 1.005091 | 1.185358 | 2.940052 | 0.204036 | -0.10043 | -0.57186 | 0.048212 | 0.082481 | 0.149421 | -1.47828 | -1.52266 | -0.72446 | -0.65695 | 0.109447 | -0.72674 | -3.08839 | -2.28326 | -0.76262 | -0.41792 | -0.0307 | -0.75155 |
| B0F2B4 | B0F2B4 | NLGN4_M | Nlgn4l | Neuroligin 4-like | | | + | NaN | 0 | NaN | 0.327902 | -0.79006 | -0.88389 | 1.314002 | 0.391346 | 4.372506 | -2.59996 | -4.50258 | -4.75977 | -2.97945 | -3.10993 | -2.97679 | -2.62811 | -3.17776 | -2.99843 | -3.16068 | -3.49453 | -3.37488 |
| Q91YP2 | Q91YP2 | NEUL_MOI | Nln | Neurolysin, mitochondrial | | | | 0.177078 | 0.251376 | 0.502557 | 0.183784 | -0.31172 | -0.51988 | 0.56999 | -0.77095 | -1.51427 | -2.94109 | -2.59122 | -1.01129 | -2.1319 | -1.87367 | -2.87818 | -2.54892 | -3.48613 | -1.0464 | -1.47335 | -1.52164 | -1.68832 |
| Q3TL44 | Q3TL44 | NLRX1_M | Nlrx1 | NLR family member X1 | | | | 0.203561 | 0.088773 | 0.57065 | 0.026563 | 0.013952 | 0.084056 | 0.124337 | -0.068 | -0.36353 | -0.99602 | -0.81418 | -0.95185 | -0.62549 | -1.21566 | -1.56691 | -1.1201 | -0.86765 | -0.83302 | -0.77223 | -1.38493 | -1.26464 |
| Q80UW2 | Q80UW2 | FBX2_MO | Fbxo2 | F-box only protein 2 | + | + | | 2.711669 | 0.59355 | 22.65693 | 1.297111 | 1.345648 | 4.281738 | 0.217695 | -0.08927 | -0.60666 | 1.122892 | 1.15477 | 1.272646 | 1.151107 | -0.04839 | 0.120396 | 0.566072 | 0.524491 | -0.44212 | 0.174573 | 0.245819 | 0.004729 |
| Q01768 | Q01768 | NDKB_MO | Nme2 | Nucleoside diphosphate kinase B | + | | | 1.063556 | -0.76213 | -3.17782 | 0.345874 | -0.65501 | -0.92904 | 0.052009 | 0.165781 | 0.160638 | -0.83183 | -0.96461 | -0.33983 | -1.63361 | -0.62812 | 0.581497 | -0.36654 | 0.094366 | -0.0513 | -0.6121 | 0.647122 | -1.02531 |
| Q9WV85 | Q9WV85 | NDK3_MO | Nme3 | Nucleoside diphosphate kinase 3 | | + | | 0.026391 | -0.10208 | -0.08353 | 1.064668 | 0.975957 | 3.182472 | 0.031598 | 0.0404 | 0.099485 | -4.09316 | -2.16906 | -1.17083 | -1.12758 | -0.42933 | -1.06546 | -3.78281 | -2.27524 | -2.43106 | -1.81926 | -1.04028 | -0.53531 |
| Q8K2T1 | Q8K2T1 | NMRL1_M | Nmral1 | NmrA-like family domain-containing protein 1 | | + | | 0.526248 | 0.216878 | 1.395252 | 1.05611 | -0.46096 | -3.14677 | 0.336951 | -0.18254 | -0.90661 | 1.712711 | 1.642809 | -0.09737 | 0.152823 | 0.214326 | 0.279142 | 1.612342 | 1.309423 | 0.412467 | 0.564894 | 0.627988 | 0.230556 |
| O70310 | O70310 | NMT1_MO | Nmt1 | Glycylpeptide N-tetradecanoyltransferase 1 | | | | 0.649023 | -1.79649 | -1.73781 | 0.388142 | -1.4272 | -1.03577 | 0.212727 | -0.1852 | -0.59403 | -4.13778 | -3.3476 | -0.88481 | -3.58182 | -1.07193 | -1.68685 | -0.99091 | -2.90149 | -0.52298 | -1.08926 | -1.14247 | -1.2459 |
| O70311 | O70311 | NMT2_MO | Nmt2 | Glycylpeptide N-tetradecanoyltransferase 2 | | | | NaN | 0 | NaN | 0.227771 | 0.505645 | 0.632228 | 0.435131 | -0.41845 | -1.15584 | -3.81722 | -3.86473 | -1.45496 | -1.59212 | -2.07028 | -2.73058 | -3.87452 | -3.03381 | -2.82602 | -1.23235 | -2.13054 | -1.83342 |
| Q61941 | Q61941 | NNTM_MC | Nnt | NAD(P) transhydrogenase, mitochondrial | | | + | 0.871216 | 4.59929 | 2.443346 | 0.717611 | 2.856946 | 1.942232 | 2.387333 | 5.055884 | 15.57141 | 2.822227 | 2.674561 | 1.569153 | 1.848178 | 2.046993 | 1.826083 | -3.73182 | 0.030029 | 0.316049 | -2.61261 | -3.42467 | -2.81402 |
| O35710 | O35710 | NOCT_MO | Noct | Nocturnin | | | | 0.793531 | 0.701626 | 2.181746 | 0.015462 | -0.01645 | -0.04949 | 0.405594 | 0.547062 | 1.080155 | -2.84217 | -2.2033 | -2.12353 | -2.77978 | -1.30029 | -2.27347 | -3.18723 | -3.26149 | -2.48814 | -2.38227 | -2.19346 | -2.47443 |
| Q8CHK3 | Q8CHK3 | MBOA7_M | Mboat7 | Lysophospholipid acyltransferase 7 | + | + | | 1.599467 | 0.586962 | 6.186136 | 1.723246 | 0.119898 | 7.167932 | 0.29985 | -0.12783 | -0.81354 | 0.747587 | 0.557944 | 0.312105 | 0.341134 | 0.299688 | 0.353807 | 0.069233 | 0.062374 | 0.215035 | 0.198408 | 0.609358 | 0.299797 |
| Q6GQT9 | Q6GQT9 | NOMO1_M | Nomo1 | BOS complex subunit NOMO1 | | | | 0.159641 | -0.46059 | -0.45717 | 0.141899 | 0.173016 | 0.410441 | 0.162731 | 0.365474 | 0.465253 | -3.06806 | -1.13725 | -1.45433 | -2.29721 | -2.23574 | -1.73088 | -1.35399 | -1.93015 | -2.03969 | -2.05789 | -1.10752 | -1.59004 |
| Q99K48 | Q99K48 | NONO_M | Nono | Non-POU domain-containing octamer-binding protein | | | | 0.29482 | 0.207543 | 0.80093 | 0.2124 | -0.0607 | -0.59319 | 0.444289 | 0.365734 | 1.179464 | -0.57486 | -0.51339 | -0.25949 | -0.14118 | 0.523433 | 0.133185 | -0.49437 | -1.00896 | -0.05614 | -0.22313 | 0.203571 | -0.27842 |
| Q9D6Z1 | Q9D6Z1 | NOP56_M | Nop56 | Nucleolar protein 56 | + | | | 1.045583 | 0.662806 | 3.103258 | 0.129915 | 0.133576 | 0.378506 | 0.519643 | 0.57551 | 1.377536 | -0.17525 | 0.030022 | -1.042 | -0.35531 | 0.016727 | -0.81793 | -0.92273 | -0.54811 | -0.9138 | -0.75067 | -0.95661 | -0.99561 |
| Q6DFW4 | Q6DFW4 | NOP58_M | Nop58 | Nucleolar protein 58 | | | + | 0.36141 | 0.585247 | 0.968161 | 0.858976 | 1.866206 | 2.400929 | 1.741798 | 1.150292 | 7.327114 | -2.93634 | -2.81407 | -2.81463 | -1.29904 | -1.84347 | -1.9901 | -4.06185 | -2.85906 | -3.75007 | -4.09601 | -3.20589 | -2.92825 |
| P33173 | P33173 | KIF1A_MO | Kif1a | Kinesin-like protein KIF1A | + | | | 1.393194 | 0.576481 | 4.820645 | 0.626358 | 0.233527 | 1.672481 | 0.02127 | 0.015556 | 0.067672 | 0.566909 | 0.762846 | 1.466929 | 1.536858 | 1.230319 | 0.850691 | 0.156975 | 0.019819 | 1.133186 | 1.403547 | 0.895282 | 1.154616 |
| Q9D3A8 | Q9D3A8 | CAPON_M | Nos1ap | Carboxyl-terminal PDZ ligand of neuronal nitric oxide synthase protein | | | | NaN | 0 | NaN | 0.475597 | -0.28438 | -1.26092 | 0.359692 | -1.46488 | -0.96383 | -3.72085 | -3.68765 | -1.31215 | -0.93926 | -3.89467 | -0.91484 | -3.4857 | -3.17527 | -0.96823 | -0.71443 | -1.24005 | -0.63971 |
| A0A1W2P | A0A1W2P | NOVA2_M | Nova2 | RNA-binding protein Nova-2 | | + | | 0.244885 | 0.358633 | 0.675479 | 1.64005 | 0.712853 | 6.493257 | 0.248992 | 0.085504 | 0.685833 | -1.81352 | -0.99867 | -0.82802 | -0.96067 | -1.49888 | -1.25977 | -1.4243 | -2.10516 | -1.69469 | -1.51971 | -1.42948 | -1.50018 |
| Q11011 | Q11011 | PSA_MOU | Npepps | Puromycin-sensitive aminopeptidase | + | | | 1.243675 | -0.77853 | -4.00504 | 0.386055 | -0.70372 | -1.03048 | 0.073342 | -0.07678 | -0.22245 | 0.356761 | 0.572031 | 0.942923 | -0.4072 | 0.582762 | 1.126679 | 1.404791 | 1.081056 | 0.868369 | 1.07479 | 1.144001 | 0.718993 |
| Q61937 | Q61937 | NPM_MOL | Npm1 | Nucleophosmin | | | | 0.303137 | 0.099466 | 0.821777 | 0.851838 | -1.0052 | -2.37641 | 0.332556 | 0.355264 | 0.895568 | 0.738677 | 0.513958 | -0.43951 | 0.1417 | 1.002588 | 0.325741 | 0.481845 | 0.571858 | 1.163652 | 0.548933 | 0.515863 | 0.101937 |
| P97300 | P97300 | NPTN_MO | Nptn | Neuroplastin | | | | 0.294779 | -0.22003 | -0.80083 | 0.339166 | -0.29889 | -0.91217 | 0.192879 | 0.135051 | 0.543289 | 0.962703 | 1.417677 | 2.168439 | 1.541507 | 1.940311 | 2.142334 | 1.256145 | 1.564294 | 2.058442 | 2.249284 | 2.106511 | 1.670032 |
| Q62443 | Q62443 | NPTX1_MC | Nptx1 | Neuronal pentraxin-1 | + | + | + | 1.039946 | -2.6959 | -3.08015 | 1.528245 | 0.885882 | 5.679371 | 1.196612 | 0.539615 | 3.773883 | -2.40126 | -3.89315 | 0.470117 | 0.387596 | 0.079374 | 0.359633 | -0.90916 | 0.006545 | -0.60745 | -0.3066 | -0.34855 | -0.29167 |
| O70340 | O70340 | NPTX2_M | Nptx2 | Neuronal pentraxin-2 | | | | NaN | 0 | NaN | 0.469519 | 0.744374 | 1.245017 | 0.028038 | 0.080557 | 0.08859 | -3.70963 | -4.18071 | -1.96323 | -2.99986 | -1.8868 | -3.6768 | -4.00246 | -3.10575 | -3.52393 | -2.92791 | -2.70163 | -3.02308 |
| Q99J85 | Q99J85 | NPTXR_M | Nptxr | Neuronal pentraxin receptor | | | + | 0.555416 | -0.7739 | -1.47428 | 0.609602 | 0.898863 | 1.624837 | 2.842263 | 0.914576 | 26.34286 | -3.09542 | -4.12704 | -0.11195 | -0.67259 | -0.20868 | -0.22866 | -2.9348 | -2.73985 | -1.76805 | -0.81421 | -1.16649 | -1.09999 |
| Q64669 | Q64669 | NQO1_MC | Nqo1 | NAD(P)H dehydrogenase [quinone] 1 | | | | 0.631093 | 0.721943 | 1.686044 | 0.154288 | -0.08523 | -0.44314 | 0.75523 | 0.987439 | 2.059052 | 2.332384 | 3.155056 | 1.071411 | 1.385964 | 1.269064 | 1.8213 | 2.140719 | 1.902833 | 1.424637 | 1.203203 | 0.16565 | 0.949836 |
| P11499 | P11499 | HS90B_MC | Hsp90ab1 | Heat shock protein HSP 90-beta | + | | | 1.945663 | 0.574586 | 9.313557 | 0.113966 | 0.074579 | 0.335473 | 0.146108 | 0.139824 | 0.421584 | 3.924801 | 3.871418 | 4.6522 | 4.859495 | 5.008596 | 4.389516 | 3.379144 | 3.267903 | 4.484597 | 4.87794 | 4.440131 | 4.678334 |
| P06537 | P06537 | GCR_MOU | Nr3c1 | Glucocorticoid receptor | | | | 0.14102 | 0.411093 | 0.40811 | 0.012355 | 0.014397 | 0.039681 | 0.261493 | -0.19656 | -0.71729 | -3.81739 | -2.10024 | -1.07946 | -1.03987 | -1.77689 | -1.53911 | -3.89671 | -2.84311 | -1.43633 | -0.71179 | -1.70834 | -1.21453 |
| Q8VII8 | Q8VII8 | MCR_MOU | Nr3c2 | Mineralocorticoid receptor | | + | + | NaN | 0 | NaN | 2.207718 | 0.449674 | 12.64249 | 1.444348 | -1.14664 | -5.13114 | -2.99361 | -3.87293 | -3.41751 | -3.3581 | -2.15193 | -2.55524 | -2.96577 | -3.6525 | -3.81792 | -3.85705 | -1.11065 | -1.30325 |
| Q9DBL1 | Q9DBL1 | ACDSB_M | Acadsb | Short/branched chain specific acyl-CoA dehydrogenase, mitochondrial | + | | | 4.04944 | 0.571604 | 105.85 | 0.433662 | -0.15854 | -1.15205 | 0.125504 | 0.101963 | 0.366669 | 0.624398 | 0.62858 | 0.133019 | -0.11325 | 0.10325 | 0.650836 | 0.059864 | 0.049906 | 0.106975 | 0.229878 | 0.22644 | 0.323718 |
| Q810U4 | Q810U4 | NRCAM_M | Nrcam | Neuronal cell adhesion molecule | | | | 0.144242 | 0.472009 | 0.41665 | 0.379144 | 0.452612 | 1.012965 | 0.783693 | 1.050461 | 2.149854 | -1.42131 | -0.72304 | 0.06854 | -0.75825 | 0.976369 | 0.104944 | -0.46646 | -2.62191 | -0.96703 | -0.62791 | -0.28866 | -0.73095 |
| P60761 | P60761 | NEUG_MO | Nrgn | Neurogranin | | | | 0.368276 | 0.661324 | 0.985488 | 0.359567 | 2.405809 | 0.963515 | 0.258038 | -0.07947 | -0.7086 | -3.70827 | -2.38443 | 1.200779 | 1.164276 | 1.188535 | 1.228621 | -3.59729 | -3.81806 | -3.72013 | 1.273562 | 1.3984 | 1.177702 |
| Q91W89 | Q91W89 | MA2C1_M | Man2c1 | Alpha-mannosidase 2C1 | + | | | 1.389809 | 0.567149 | 4.800696 | 0.629876 | -0.23455 | -1.68255 | 0.151034 | -0.17827 | -0.43458 | 0.670082 | 0.900635 | 0.441332 | 0.547891 | 0.836399 | 0.62518 | 0.192363 | 0.244056 | 0.600345 | 0.857986 | 0.512675 | 1.305443 |
| P97333 | P97333 | NRP1_MO | Nrp1 | Neuropilin-1 | | | | NaN | 0 | NaN | 0.398012 | 0.708516 | 1.060843 | 0.221785 | 0.21756 | 0.617051 | -2.88834 | -3.62571 | -2.59251 | -2.79286 | -0.39845 | -1.09611 | -2.73357 | -3.46786 | -4.06152 | -2.74088 | -0.91355 | -1.01614 |
| Q61070 | Q61070 | EI24_MOU | Ei24 | Etoposide-induced protein 2.4 | + | + | | 1.581251 | 0.566475 | 6.052708 | 1.195017 | 0.271633 | 3.766244 | 0.218093 | -0.14303 | -0.60768 | 1.152738 | 1.124399 | 0.386392 | 0.529343 | 0.638177 | 0.191515 | 0.664605 | 0.479582 | 0.176589 | 0.195879 | 0.632171 | 0.483574 |
| E9Q7X7 | E9Q7X7 | NRX2A_M | Nrxn2 | Neurexin-2 | | + | | 0.487439 | 0.682655 | 1.292023 | 1.159529 | 0.321358 | 3.599538 | 0.051656 | 0.05212 | 0.159599 | -1.33507 | -0.48496 | 0.452246 | 0.278217 | 0.063434 | -0.58784 | -1.27884 | -1.90651 | 0.0239 | 0.063848 | -0.33902 | -0.28963 |
| Q6P9K9 | Q6P9K9 | NRX3A_M | Nrxn3 | Neurexin-3 | | | | 0.155433 | 0.613413 | 0.446144 | 0.978427 | 0.646619 | 2.83604 | 0.594129 | 0.934254 | 1.581313 | -1.93296 | -1.48019 | -0.96637 | -1.2743 | -0.13625 | -1.31566 | -0.96383 | -3.67615 | -1.93512 | -1.59879 | -1.62409 | -1.69632 |
| P46460 | P46460 | NSF_MOU | Nsf | Vesicle-fusing ATPase | | | | 0.340718 | -0.20128 | -0.91607 | 0.036241 | 0.030959 | 0.113587 | 0.579254 | 0.312931 | 1.539881 | 3.736554 | 3.769594 | 4.666746 | 4.127916 | 5.549803 | 5.172628 | 4.173455 | 3.735255 | 4.325107 | 4.407637 | 5.123996 | 4.972572 |
| Q9CZ44 | Q9CZ44 | NSF1C_M | Nsfl1c | NSFL1 cofactor p47 | | | | 0.251802 | 0.41977 | 0.692911 | 0.101184 | 0.364005 | 0.300483 | 0.148129 | 0.229315 | 0.42692 | -3.32787 | -2.11714 | -1.97098 | -4.19498 | -2.62963 | -3.69312 | -3.11914 | -3.16541 | -3.92755 | -2.96641 | -3.31477 | -3.46661 |
| Q99NF2 | Q99NF2 | NSMF_MO | Nsmf | NMDA receptor synaptonuclear signaling and neuronal migration factor | | + | + | 0.39672 | 0.682803 | 1.057557 | 1.013865 | -0.48759 | -2.97487 | 1.436222 | -0.60947 | -5.08066 | -1.12046 | -2.27734 | 0.221582 | 0.378576 | 1.298719 | 1.538402 | -2.0949 | -2.66851 | 0.643787 | 0.931554 | 2.033347 | 2.022717 |
| Q3UHB1 | Q3UHB1 | NT5D3_M | Nt5dc3 | 5'-nucleotidase domain-containing protein 3 | | | | 0.95742 | -0.66234 | -2.75595 | 0.029802 | 0.024383 | 0.093997 | 0.067037 | -0.09744 | -0.20438 | -0.53555 | -0.97995 | -0.37209 | -0.75227 | 0.397816 | 1.256992 | -0.00385 | -0.18698 | -0.76307 | -0.41006 | 1.131638 | 0.718058 |
| Q99PJ0 | Q99PJ0 | NTRI_MOL | Ntm | Neurotrimin | | | | 0.632717 | -0.59602 | -1.69071 | 0.227398 | -0.63672 | -0.63128 | 0.050391 | 0.117261 | 0.155868 | -0.73994 | -0.17843 | 0.74436 | -1.23095 | -0.31759 | 0.273027 | 0.350041 | -0.07636 | 0.597924 | 0.188921 | 0.552384 | -0.83147 |
| Q9R061 | Q9R061 | NUBP2_M | Nubp2 | Cytosolic Fe-S cluster assembly factor NUBP2 | | | | 0.309751 | -0.31644 | -0.83836 | 0.646517 | 1.019894 | 1.730535 | 0.351656 | 0.315513 | 0.943586 | -3.25185 | -3.90742 | -2.48378 | -1.953 | -2.4178 | -1.75745 | -3.45035 | -3.07604 | -3.7645 | -2.71207 | -2.3503 | -2.45597 |
| Q80XU3 | Q80XU3 | NUCKS_M | Nucks1 | Nuclear ubiquitous casein and cyclin-dependent kinase substrate 1 | | | | 0.634573 | 0.735438 | 1.696039 | 0.50372 | -0.37662 | -1.33508 | 0.743764 | 0.574006 | 2.023071 | 2.026525 | 1.494102 | 2.868267 | 3.274009 | 2.951621 | 3.104132 | 1.36716 | 0.682591 | 3.643762 | 3.251745 | 2.727161 | 2.18058 |
| O35685 | O35685 | NUDC_MO | Nudc | Nuclear migration protein nudC | | | | 0.298894 | -0.99648 | -0.81114 | 0.610981 | 0.748807 | 1.628739 | 0.277347 | 0.419359 | 0.757109 | -0.3894 | -1.02609 | -0.03424 | -0.48305 | 0.367777 | -0.56581 | -0.87527 | -1.54726 | -1.40871 | -0.60619 | -0.81654 | -0.2202 |
| P0C027;P | P0C027;P | NUD10_M | Nudt10;Nudt11 | Diphosphoinositol polyphosphate phosphohydrolase 3-alpha | | | | 0.356004 | 0.144706 | 0.954534 | 0.572654 | 0.090967 | 1.521621 | 0.228367 | 0.174643 | 0.633739 | -0.16145 | -0.14658 | -0.29779 | -0.18042 | -0.21006 | 0.272322 | -0.14731 | -0.45014 | -0.34146 | -0.31868 | -0.01021 | -0.27681 |
| Q9JI46 | Q9JI46 | NUDT3_M | Nudt3 | Diphosphoinositol polyphosphate phosphohydrolase 1 | | + | | 0.292093 | 0.147171 | 0.794094 | 1.267013 | -0.3804 | -4.12397 | 0.604493 | -0.40528 | -1.61042 | 0.038867 | 0.407718 | 0.110296 | 0.045856 | 0.292976 | 0.677034 | 0.057816 | 0.094429 | 0.372046 | 0.544909 | 1.052945 | 0.727625 |
| Q9JKX6 | Q9JKX6 | NUDT5_M | Nudt5 | ADP-sugar pyrophosphatase | | | + | 0.844208 | -1.23562 | -2.35036 | 0.109724 | 0.230263 | 0.323911 | 1.182456 | -0.88649 | -3.70654 | -3.50082 | -3.87929 | -1.62496 | -2.0094 | -1.19587 | -1.62354 | -1.96395 | -2.94491 | -2.73185 | -1.36305 | -0.63034 | -0.41609 |
| O55125 | O55125 | NIPS1_MO | Nipsnap1 | Protein NipSnap homolog 1 | + | | + | 1.737735 | 0.561471 | 7.291974 | 0.029818 | 0.028443 | 0.094045 | 1.334877 | 0.402604 | 4.486969 | 3.223669 | 3.221572 | 3.237735 | 3.834308 | 3.376256 | 3.475623 | 2.738141 | 2.584158 | 3.557534 | 3.457623 | 2.948619 | 3.098052 |
| E9Q7G0 | E9Q7G0 | NUMA1_M | Numa1 | Nuclear mitotic apparatus protein 1 | | | | 0.100261 | -0.40399 | -0.29794 | 0.515624 | -0.24769 | -1.36678 | 0.546233 | -0.52776 | -1.44925 | -3.43433 | -0.72332 | -1.00756 | -0.96405 | -1.56775 | -0.85812 | -1.64064 | -1.70904 | -0.5582 | -0.91802 | -0.76715 | -0.60319 |
| Q9QZS3 | Q9QZS3 | NUMB_MC | Numb | Protein numb homolog | | | | NaN | 0 | NaN | 0.510105 | 0.226022 | 1.352062 | 0.548373 | -0.14301 | -1.45507 | -4.34869 | -3.76379 | -1.95787 | -1.89121 | -2.85659 | -3.03751 | -3.64345 | -3.11914 | -2.31438 | -1.98675 | -2.7656 | -2.84248 |
| O08919 | O08919 | NUMBL_M | Numbl | Numb-like protein | | | | 0.118939 | -0.09794 | -0.34896 | 0.084026 | 0.022291 | 0.252717 | 0.478892 | -0.36258 | -1.26956 | -0.30019 | 0.205539 | 2.261753 | 2.351371 | 1.417332 | 1.876738 | -0.07119 | 0.172423 | 2.208292 | 2.360248 | 1.839906 | 2.179317 |
| Q8R0G9 | Q8R0G9 | NU133_M | Nup133 | Nuclear pore complex protein Nup133 | | | | 0.525741 | 0.280877 | 1.393889 | 0.308051 | 0.356768 | 0.834097 | 0.292796 | -0.33284 | -0.79586 | -3.51802 | -3.11715 | -3.42073 | -2.87235 | -2.77675 | -3.58803 | -3.57771 | -3.6192 | -3.17502 | -3.8316 | -2.74775 | -2.95135 |
| Q99P88 | Q99P88 | NU155_M | Nup155 | Nuclear pore complex protein Nup155 | | | | 0.663926 | 1.204351 | 1.781344 | 0.74015 | 0.569263 | 2.011798 | 0.875611 | -0.2297 | -2.45869 | -2.2774 | -1.23644 | -1.95908 | -2.50473 | -2.29926 | -2.18964 | -2.52976 | -3.39278 | -2.7261 | -2.87623 | -1.9391 | -2.09041 |
| B9EJ54 | B9EJ54 | B9EJ54_M | Nup205 | Nucleoporin 205 | | | | 0.814222 | 0.779133 | 2.249698 | 0.177489 | 0.109977 | 0.503619 | 0.109149 | -0.12695 | -0.32234 | -1.02577 | -0.51876 | -1.18509 | -1.02965 | -0.69505 | -1.45261 | -1.31543 | -1.78736 | -1.01327 | -1.42142 | -1.05471 | -0.83906 |
| Q80U93 | Q80U93 | NU214_M | Nup214 | Nuclear pore complex protein Nup214 | | | | 0.236503 | 0.994709 | 0.654319 | 0.492073 | 0.467164 | 1.304243 | 0.456943 | -0.60324 | -1.21225 | -1.59362 | -1.19102 | -0.8408 | -0.95812 | -2.14457 | -1.23591 | -0.88019 | -3.89386 | -1.71997 | -1.01327 | -1.29001 | -0.88399 |
| Q63850 | Q63850 | NUP62_M | Nup62 | Nuclear pore glycoprotein p62 | | | | 0.498617 | -0.5177 | -1.32154 | 0.061141 | 0.08524 | 0.18734 | 0.686666 | -0.27368 | -1.84869 | -2.54787 | -2.48807 | -2.31831 | -1.71333 | -2.36469 | -2.14155 | -2.39087 | -1.60967 | -1.76117 | -2.44095 | -2.07675 | -1.88213 |
| Q8R480 | Q8R480 | NUP85_M | Nup85 | Nuclear pore complex protein Nup85 | | | | 0.196043 | 1.79112 | 0.551408 | 0.12197 | -0.30617 | -0.35715 | 0.470755 | 3.471161 | 1.248247 | 1.301514 | 2.121119 | 2.517304 | 0.827412 | 1.563591 | 2.529835 | 3.14251 | -3.30212 | 2.12333 | 1.833731 | -4.16299 | 1.314093 |
| Q8CEC0 | Q8CEC0 | NUP88_M | Nup88 | Nuclear pore complex protein Nup88 | | | | 0.31616 | -0.8426 | -0.85443 | 0.310119 | 0.341072 | 0.83928 | 0.232141 | -0.14947 | -0.64329 | -1.13745 | -3.04538 | -1.23712 | -0.55976 | -1.62953 | -1.17833 | -0.9989 | -1.49873 | -1.01491 | -1.46412 | -1.19885 | -1.31007 |
| Q8BJ71 | Q8BJ71 | NUP93_M | Nup93 | Nuclear pore complex protein Nup93 | | + | | 0.200811 | -0.71659 | -0.56362 | 1.746432 | 1.477207 | 7.367398 | 0.060794 | -0.29988 | -0.18633 | -3.58806 | -3.2673 | -1.32302 | -1.28447 | -0.10032 | -3.305 | -3.97235 | -1.44983 | -2.98053 | -2.58138 | -1.25228 | -1.55328 |
| Q6PFD9 | Q6PFD9 | NUP98_M | Nup98 | Nuclear pore complex protein Nup98-Nup96 | | | | 0.789454 | -0.98494 | -2.1685 | 0.578476 | 0.558478 | 1.537726 | 0.457198 | -1.21668 | -1.21292 | -0.73289 | 0.077948 | -0.10662 | 0.158714 | -1.77316 | 0.210635 | 0.452686 | 0.862251 | -0.19434 | -0.87052 | 0.28591 | 0.584929 |
| Q8R570 | Q8R570 | SNP47_M | Snap47 | Synaptosomal-associated protein 47 | + | | | 1.501573 | 0.558587 | 5.499593 | 0.565 | 0.196434 | 1.500539 | 0.292902 | 0.396401 | 0.79612 | 0.191371 | 0.070528 | 0.99242 | 0.803531 | 1.287308 | 0.304717 | -0.50928 | -0.346 | 0.610892 | 0.792191 | 0.318688 | 0.480535 |
| Q6P5U7 | Q6P5U7 | NWD2_M | Nwd2 | NACHT and WD repeat domain-containing protein 2 | | + | | 0.256501 | 0.822906 | 0.704737 | 1.238499 | -0.32226 | -3.97906 | 0.775803 | -0.4508 | -2.12447 | -3.74565 | -1.62488 | -0.00628 | -0.06167 | -0.24818 | -0.03009 | -3.9971 | -3.01924 | 0.21218 | 0.364396 | 0.129633 | 0.49369 |
| Q6PFX7 | Q6PFX7 | NYAP1_M | Nyap1 | Neuronal tyrosine-phosphorylated phosphoinositide-3-kinase adapter 1 | | | | NaN | 0 | NaN | 0.556435 | 0.154268 | 1.477064 | 0.031618 | 0.058818 | 0.099545 | -3.98149 | -3.90114 | 0.455905 | 0.500518 | -0.47076 | -0.07736 | -3.40885 | -2.50085 | 0.221912 | 0.425976 | -0.89004 | 0.224282 |
| Q8BM65 | Q8BM65 | NYAP2_M | Nyap2 | Neuronal tyrosine-phosphorylated phosphoinositide-3-kinase adapter 2 | | | | NaN | 0 | NaN | 0.001111 | -0.00261 | -0.00361 | 0.145046 | 0.312704 | 0.418776 | -3.21751 | -2.60991 | -1.87748 | -2.49818 | -2.3556 | -2.47553 | -3.68722 | -3.33121 | -2.83793 | -1.53251 | -1.98397 | -3.47257 |
| P29758 | P29758 | OAT_MOU | Oat | Ornithine aminotransferase, mitochondrial | | | | 0.820935 | -2.30139 | -2.27201 | 0.296912 | -0.39489 | -0.80617 | 0.181171 | -0.34785 | -0.51314 | -1.30251 | -1.27978 | -0.68483 | -1.62021 | -1.94186 | -0.96666 | 0.066598 | -0.0461 | -0.61202 | -0.90323 | -0.63549 | -1.57734 |
| O88447 | O88447 | KLC1_MOL | Klc1 | Kinesin light chain 1 | + | | | 1.747844 | 0.555926 | 7.37971 | 0.321454 | 0.140266 | 0.867706 | 0.209925 | 0.108654 | 0.586889 | -0.19672 | -0.32246 | -0.13786 | 0.074553 | 0.035903 | -0.31548 | -0.77401 | -0.85702 | -0.29379 | -0.05005 | -0.19007 | -0.30682 |
| Q6NVF0 | Q6NVF0 | OCRL_MO | Ocrl | Inositol polyphosphate 5-phosphatase OCRL | | | + | NaN | 0 | NaN | 0.172573 | 0.355615 | 0.490878 | 1.250027 | 1.361723 | 4.037124 | -2.6332 | -3.32162 | -1.93106 | -2.41968 | -1.8542 | -2.5195 | -3.33977 | -3.62388 | -3.213 | -1.84898 | -3.60437 | -3.49278 |
| Q9EQQ9 | Q9EQQ9 | OGA_MOL | Oga | Protein O-GlcNAcase | | + | | 0.511631 | -1.78692 | -1.35613 | 1.363262 | 0.326708 | 4.646767 | 0.444942 | 0.19097 | 1.181153 | -1.79983 | -3.50682 | 0.826558 | 0.848497 | 0.555028 | 0.753946 | 0.137478 | -1.87029 | 0.441372 | 0.580267 | 0.590987 | 0.336046 |
| Q60597 | Q60597 | ODO1_MC | Ogdh | 2-oxoglutarate dehydrogenase complex component E1 | + | | | 1.079906 | -0.65305 | -3.24681 | 0.163472 | -0.16451 | -0.46719 | 0.038168 | 0.048604 | 0.119407 | 1.408422 | 1.384596 | 1.507758 | 0.803596 | 0.990425 | 1.431494 | 2.250348 | 1.848779 | 1.313942 | 1.326438 | 1.504481 | 0.82023 |
| Q61249 | Q61249 | IGBP1_MC | Igbp1 | Immunoglobulin-binding protein 1 | + | | | 1.853444 | 0.554268 | 8.358337 | 0.030864 | -0.05848 | -0.09724 | 0.075453 | -0.09516 | -0.22846 | -1.2777 | -1.30967 | -1.02284 | -1.59892 | -0.79433 | -1.1959 | -1.7836 | -1.91231 | -1.78035 | -0.72444 | -1.26489 | -0.53503 |
| Q8CGY8 | Q8CGY8 | OGT1_MO | Ogt | UDP-N-acetylglucosamine--peptide N-acetylglucosaminyltransferase 110 kDa subunit | | | | 0.474994 | 0.364973 | 1.259336 | 0.444144 | -0.16685 | -1.17909 | 0.568157 | -0.96293 | -1.50922 | 1.043419 | 1.108004 | 2.831052 | 3.090117 | 1.66289 | 2.864228 | 0.998748 | 0.42273 | 3.070461 | 3.184415 | 3.011364 | 3.441607 |
| Q9CZ30 | Q9CZ30 | OLA1_MOI | Ola1 | Obg-like ATPase 1 | | | | 0.422831 | -0.68457 | -1.12423 | 0.314503 | -0.29497 | -0.85027 | 0.042562 | -0.04896 | -0.1326 | -0.64247 | -0.08848 | 0.326698 | -0.31202 | 0.039742 | 0.616832 | 0.861383 | -0.22318 | 0.166818 | 0.4378 | 0.607639 | 0.146858 |
| O88998 | O88998 | NOE1_MO | Olfm1 | Noelin | | | | 0.772977 | -0.91362 | -2.11542 | 0.86154 | 0.33421 | 2.409775 | 0.719095 | 0.576482 | 1.946773 | -2.94803 | -2.58287 | -0.24662 | -0.52358 | 0.202672 | -0.07405 | -1.46044 | -2.24323 | -0.71164 | -0.72698 | -0.25036 | -0.77398 |
| Q8CJH3 | Q8CJH3 | PLXB1_MC | Plxnb1 | Plexin-B1 | + | | | 1.707739 | 0.522208 | 7.037387 | 0.778288 | 1.358193 | 2.132447 | 0.405024 | 0.622437 | 1.078701 | -1.10966 | -0.96991 | -2.00835 | -2.32175 | -0.94339 | -1.86811 | -1.53703 | -1.58696 | -4.14058 | -2.9059 | -2.37341 | -1.68296 |
| P58281 | P58281 | OPA1_MO | Opa1 | Dynamin-like 120 kDa protein, mitochondrial | + | | | 1.262177 | 0.256771 | 4.099086 | 0.262117 | 0.034296 | 0.71886 | 0.097253 | 0.06213 | 0.289625 | 2.601533 | 2.52064 | 2.364933 | 2.380088 | 2.906435 | 2.477747 | 2.352148 | 2.256483 | 2.385317 | 2.291111 | 2.621284 | 2.638638 |
| Q505D7 | Q505D7 | OPA3_MO | Opa3 | Optic atrophy 3 protein homolog | | | | 0.422552 | 0.399606 | 1.12351 | 0.1646 | -0.14119 | -0.47013 | 0.152124 | 0.166596 | 0.437447 | 0.655472 | 0.450517 | 0.25672 | 0.348685 | 0.442739 | -0.3158 | 0.493982 | -0.18721 | 0.740662 | 0.147116 | -0.06864 | -0.13762 |
| P48722 | P48722 | HS74L_MC | Hspa4l | Heat shock 70 kDa protein 4L | + | | + | 1.823212 | 0.521486 | 8.066204 | 0.800736 | 0.552651 | 2.205274 | 1.089726 | -0.48517 | -3.28879 | 1.687589 | 1.567422 | 2.432486 | 2.708646 | 1.131394 | 1.3977 | 1.129889 | 1.082152 | 1.808782 | 2.227047 | 1.686211 | 1.813231 |
| Q8K010 | Q8K010 | OPLA_MO | Oplah | 5-oxoprolinase | | + | | 0.774759 | 0.40682 | 2.121121 | 1.740142 | -0.5496 | -7.31278 | 0.07312 | -0.02287 | -0.22182 | -1.10432 | -1.03083 | -1.55654 | -1.51385 | -1.32572 | -1.19803 | -1.28615 | -1.66264 | -1.05766 | -0.91354 | -1.31996 | -1.15806 |
| E9PZS7 | E9PZS7 | E9PZS7_M | Or10ad1 | Olfactory receptor | | | | NaN | 0 | NaN | 0.905149 | -1.03316 | -2.56343 | 0.369234 | 1.349336 | 0.987907 | -2.57094 | -3.52375 | -3.59203 | -3.86797 | -3.26837 | -0.85144 | -2.18973 | -2.70315 | -2.31816 | -3.07553 | -2.77272 | -4.04576 |
| S4R1M9 | S4R1M9;Q | OSB10_M | Osbpl10 | Oxysterol-binding protein-related protein 10 | | | | 0.754641 | 0.818706 | 2.057196 | 0.07845 | 0.091637 | 0.236976 | 0.589306 | -0.58318 | -1.56784 | -2.40855 | -1.9295 | -0.44148 | -0.40473 | -0.98855 | -1.73229 | -2.66991 | -3.30555 | -0.901 | -0.12848 | -0.76888 | -0.7856 |
| Q91XL9 | Q91XL9 | OSBL1_M | Osbpl1a | Oxysterol-binding protein-related protein 1 | | | | 0.170065 | 0.839298 | 0.484361 | 0.532523 | -0.43839 | -1.41214 | 0.781504 | -0.42886 | -2.1428 | -2.85071 | 0.277003 | -0.32449 | -0.90462 | -1.53304 | -1.24693 | -1.37987 | -2.87243 | -0.28678 | -0.06554 | -1.10109 | -0.82115 |
| Q8BXR9 | Q8BXR9 | OSBL6_M | Osbpl6 | Oxysterol-binding protein-related protein 6 | | | | 0.971631 | 1.353363 | 2.809957 | 0.188619 | -0.09026 | -0.53234 | 0.004006 | 0.002516 | 0.012987 | -0.75483 | -1.56238 | -0.91454 | -0.99489 | -0.84834 | -1.11307 | -2.77452 | -2.24941 | -1.02918 | -0.69972 | -1.12469 | -0.84175 |
| A2A716 | A2A716 | A2A716_M | Osbpl7 | Oxysterol-binding protein | + | | | 1.097662 | 0.478607 | 3.323019 | 0.294927 | 0.234649 | 0.801198 | 0.135556 | -0.14887 | -0.39358 | 0.579516 | 0.478827 | -0.5053 | 0.076958 | -0.84979 | -0.26438 | 0.185507 | -0.08438 | -0.41693 | -0.48071 | -0.64779 | -0.16864 |
| B9EJ86 | B9EJ86 | OSBL8_M | Osbpl8 | Oxysterol-binding protein-related protein 8 | | | | 0.184971 | 0.128529 | 0.522945 | 0.304483 | 2.253237 | 0.825152 | 0.071194 | 0.142256 | 0.216315 | -0.3485 | -3.19411 | -0.75306 | -1.6637 | -0.45808 | -1.77289 | -3.27013 | -3.66623 | -6.15409 | -0.76915 | -1.27527 | -1.24021 |
| A2A8Z1 | A2A8Z1 | OSBL9_M | Osbpl9 | Oxysterol-binding protein-related protein 9 | | | | 0.458324 | 0.821224 | 1.215844 | 0.083029 | 0.140405 | 0.24991 | 0.033667 | 0.070975 | 0.105786 | -2.11641 | -1.85646 | -1.48712 | -2.08063 | -2.77914 | -2.48794 | -2.14485 | -3.47047 | -2.40134 | -1.44723 | -2.04958 | -3.35945 |
| Q8BWU5 | Q8BWU5 | OSGEP_M | Osgep | tRNA N6-adenosine threonylcarbamoyltransferase | | | | 0.423903 | 0.341474 | 1.126975 | 0.057494 | 0.064428 | 0.176722 | 0.241325 | -0.34072 | -0.6665 | -1.16515 | -0.57072 | -1.61975 | -0.9219 | -0.96588 | -1.88383 | -1.26835 | -1.15047 | -1.4409 | -1.22961 | -1.30927 | -0.85899 |
| Q78XF5 | Q78XF5 | OSTC_MO | Ostc | Oligosaccharyltransferase complex subunit OSTC | + | | | 1.079572 | -0.89389 | -3.24538 | 0.199243 | 0.22906 | 0.559605 | 0.127199 | 0.160361 | 0.371223 | -2.57519 | -2.48306 | -2.40379 | -2.30639 | -1.32697 | -1.94696 | -1.90679 | -1.36368 | -2.99057 | -2.17773 | -1.49648 | -2.09818 |
| F7A4A7 | F7A4A7 | OTOGL_M | Otogl | Otogelin-like protein | + | | | 1.146296 | 1.326548 | 3.538916 | NaN | 0 | NaN | 0.414135 | -0.72112 | -1.10196 | -1.83539 | -1.76922 | -3.13483 | -4.07469 | -3.30366 | -3.68619 | -2.75547 | -3.50224 | -3.45231 | -3.03909 | -3.39963 | -2.14797 |
| Q7TQI3 | Q7TQI3 | OTUB1_M | Otub1 | Ubiquitin thioesterase OTUB1 | | | | 0.006197 | -0.00365 | -0.02004 | 0.184347 | -0.14298 | -0.52134 | 0.578371 | -0.22535 | -1.53743 | 1.879708 | 1.58274 | 2.082011 | 1.552231 | 1.96632 | 2.079415 | 1.62948 | 1.840265 | 1.889016 | 2.031193 | 2.383454 | 2.11299 |
| Q8R554 | B2RUR8;Q | OTU7A_M | Otud7a | OTU domain-containing protein 7A | | | | NaN | 0 | NaN | 0.685223 | -0.60415 | -1.84439 | 0.246634 | 0.128909 | 0.67989 | -2.70668 | -2.87873 | -1.68527 | -1.38397 | -1.27964 | -0.91633 | -3.11839 | -3.65031 | -1.22133 | -0.6396 | -1.17257 | -1.28122 |
| Q9D0K2 | Q9D0K2 | SCOT1_M | Oxct1 | Succinyl-CoA:3-ketoacid coenzyme A transferase 1, mitochondrial | | | | 0.888449 | -1.02254 | -2.50387 | 0.19959 | -0.31114 | -0.56049 | 0.010234 | 0.02309 | 0.032944 | -0.31397 | 0.482887 | 1.502002 | 0.413832 | 0.629101 | 1.924406 | 1.017381 | 1.196615 | 1.379217 | 1.158905 | 1.521585 | 0.985741 |
| Q62417 | Q62417 | SRBS1_M | Sorbs1 | Sorbin and SH3 domain-containing protein 1 | + | | + | 1.739313 | 0.501854 | 7.305603 | 0.459009 | -0.15252 | -1.21763 | 1.264601 | -0.5044 | -4.11155 | -1.30354 | -1.43722 | -1.11568 | -1.29314 | -1.10061 | -0.98768 | -1.88809 | -1.85637 | -1.1403 | -0.96348 | -0.43084 | -0.64866 |
| Q9CPV9 | Q9CPV9 | P2Y12_M | P2ry12 | P2Y purinoceptor 12 | | | | 0.609879 | 0.421301 | 1.625622 | 0.067361 | -0.10749 | -0.20532 | 0.243092 | -0.15116 | -0.67096 | -0.96412 | -0.46149 | -0.14763 | -0.57298 | -0.18189 | -0.42809 | -1.0708 | -1.19741 | 0.22558 | -0.73121 | 0.034845 | -0.34252 |
| P09103 | P09103 | PDIA1_M | P4hb | Protein disulfide-isomerase | | | | 0.051813 | -0.2248 | -0.16006 | 0.068194 | 0.088932 | 0.207711 | 0.00362 | 0.012533 | 0.01174 | -3.21654 | -1.22615 | -0.80465 | -1.64629 | -0.06413 | -2.12532 | -1.00557 | -2.98753 | -1.39331 | -1.2355 | -0.82866 | -1.38586 |
| P50580 | P50580 | PA2G4_M | Pa2g4 | Proliferation-associated protein 2G4 | | | | 0.706457 | -2.02384 | -1.90826 | 0.832996 | -0.98177 | -2.31242 | 0.191968 | -0.32194 | -0.54095 | -4.24139 | -2.20431 | -1.7894 | -2.23611 | -2.41603 | -1.22847 | -1.4946 | -0.90343 | -0.66992 | -1.39205 | -1.46022 | -1.5404 |
| P29341 | P29341 | PABP1_M | Pabpc1 | Polyadenylate-binding protein 1 | | + | | 0.47028 | 0.507229 | 1.247005 | 1.068768 | 0.306517 | 3.199685 | 0.032419 | -0.02389 | -0.10199 | 1.25303 | 1.212116 | 1.878099 | 2.063089 | 1.177386 | 0.832773 | 1.131587 | 0.319102 | 1.639147 | 1.689007 | 0.870333 | 1.187599 |
| Q6PHQ9 | Q6PHQ9;( | Q6PHQ9_I | Pabpc4 | Polyadenylate-binding protein | | | | 0.419872 | 1.324876 | 1.116642 | 0.845094 | 0.583265 | 2.353379 | 0.186603 | 0.239168 | 0.52715 | -0.68133 | -1.23897 | -0.4416 | -0.29753 | -0.56281 | -1.30202 | -1.13177 | -3.43828 | -0.71569 | -1.18997 | -1.43471 | -0.90846 |
| Q61584 | Q61584;Q | FXR1_MO | Fxr1 | RNA-binding protein FXR1 | + | | | 1.316844 | 0.495029 | 4.387941 | 0.409974 | 0.342804 | 1.09133 | 0.028715 | 0.048108 | 0.090666 | 1.044961 | 0.925401 | 1.175989 | 1.473277 | 1.239655 | 0.17844 | 0.394477 | 0.585827 | 0.705109 | 1.258548 | 0.661745 | 0.660132 |
| Q3V3Q7 | Q3V3Q7 | PACS2_M | Pacs2 | Phosphofurin acidic cluster sorting protein 2 | | | | 0.704762 | -1.06042 | -1.90312 | 0.45947 | -0.24794 | -1.21882 | 0.67811 | 0.454421 | 1.823218 | -2.08105 | -3.10195 | -1.77999 | -1.93041 | -1.39567 | -1.57319 | -1.7545 | -1.30766 | -1.41825 | -1.79627 | -2.17175 | -1.70595 |
| Q61644 | Q61644 | PACN1_M | Pacsin1 | Protein kinase C and casein kinase substrate in neurons protein 1 | + | | | 1.100621 | -0.55754 | -3.33585 | 0.09792 | 0.201263 | 0.291471 | 0.078747 | 0.094939 | 0.237817 | -0.02762 | 0.276688 | 1.664097 | 0.287834 | 0.609784 | 0.888089 | 0.612907 | 0.751239 | 0.83195 | 0.717455 | 1.02817 | 0.279826 |
| Q08642 | Q08642 | PADI2_M | Padi2 | Protein-arginine deiminase type-2 | | | | 0.608983 | -0.31308 | -1.62309 | 0.213602 | -0.10495 | -0.59626 | 0.25925 | -0.20707 | -0.71165 | 0.191239 | 0.286263 | -1.05185 | -1.17372 | -0.8357 | -0.26041 | 0.738777 | 0.364882 | -1.17297 | -0.84269 | -0.29705 | -0.38492 |
| P60229 | P60229 | EIF3E_MC | Eif3e | Eukaryotic translation initiation factor 3 subunit E | + | | | 2.161769 | 0.488711 | 11.98481 | 0.651073 | 0.23479 | 1.743768 | 0.447312 | 0.363212 | 1.187283 | 0.471527 | 0.407316 | 0.748231 | 0.611623 | 0.906384 | 0.424395 | -0.02415 | -0.07443 | 0.329104 | 0.561171 | 0.113741 | 0.490613 |
| Q61206 | Q61206 | PA1B2_M | Pafah1b2 | Platelet-activating factor acetylhydrolase IB subunit alpha2 | | | | 0.761618 | 1.445408 | 2.079246 | 0.141918 | 0.566525 | 0.410492 | 0.231581 | -0.24082 | -0.64188 | -2.26136 | -0.95849 | -0.98633 | -2.90129 | -2.41844 | -1.67089 | -2.81267 | -3.29799 | -3.50429 | -1.51638 | -1.83633 | -1.77135 |
| Q99M71 | Q99M71 | EPDR1_M | Epdr1 | Mammalian ependymin-related protein 1 | + | | | 1.693228 | 0.483714 | 6.91725 | 0.386896 | -0.07365 | -1.03261 | 0.026361 | 0.030627 | 0.083433 | 0.799656 | 0.818629 | 0.770776 | 0.62815 | 0.847044 | 1.574179 | 0.394711 | 0.256146 | 0.774558 | 0.771676 | 1.129291 | 1.230679 |
| Q8VE62 | Q8VE62 | PAIP1_MO | Paip1 | Polyadenylate-binding protein-interacting protein 1 | | | | 0.311148 | -0.41535 | -0.84186 | 0.068339 | 0.057648 | 0.208128 | 0.108236 | 0.090729 | 0.319846 | -2.23195 | -1.56198 | -1.55336 | -1.02391 | -1.57007 | -1.46529 | -1.11941 | -1.84383 | -1.42777 | -1.26479 | -1.32963 | -1.8872 |
| Q9Z0P4 | Q9Z0P4 | PALM_MO | Palm | Paralemmin-1 | | | | 0.204836 | -0.20131 | -0.57391 | 0.441231 | 0.630766 | 1.171567 | 0.054218 | 0.070126 | 0.167132 | 0.013376 | 0.567694 | 1.731946 | 0.660032 | 1.113485 | 0.503121 | 0.706836 | 0.276851 | 0.514035 | 0.616411 | 1.026125 | 0.45023 |
| Q8BR92 | Q8BR92 | PALM2_M | Palm2 | Paralemmin-2 | | | + | 0.073095 | -0.08373 | -0.22175 | 0.587187 | 0.243217 | 1.561928 | 1.045776 | -0.3089 | -3.10405 | -0.96887 | -1.2172 | -0.87384 | -0.70625 | -0.41829 | -0.58497 | -0.65273 | -1.36589 | -0.90202 | -1.16451 | -0.24712 | -0.13833 |
| Q9JLB2 | Q9JLB2 | PALS1_M | Pals1 | Protein PALS1 | | | | NaN | 0 | NaN | 0.032407 | -0.05119 | -0.10195 | 0.060519 | 0.169285 | 0.185534 | -3.15795 | -3.82951 | -1.99613 | -2.98092 | -1.74849 | -3.17101 | -3.0595 | -3.29039 | -2.53583 | -2.33883 | -3.20054 | -2.05753 |
| Q9JLB0 | Q9JLB0 | PALS2_M | Pals2 | Protein PALS2 | | | | 0.886169 | 0.241703 | 2.495807 | 0.156793 | -0.01203 | -0.44971 | 0.024043 | 0.005326 | 0.076276 | 1.054912 | 1.130874 | 1.297365 | 1.34227 | 1.423566 | 1.329395 | 0.940275 | 0.762105 | 1.317304 | 1.346391 | 1.422708 | 1.319602 |
| P97467 | P97467 | AMD_MOL | Pam | Peptidyl-glycine alpha-amidating monooxygenase | | | | 0.398547 | -0.21786 | -1.06221 | 0.777603 | 1.04425 | 2.130247 | 0.196791 | 0.515443 | 0.553325 | -1.34497 | -0.94927 | -0.79848 | 0.079709 | -0.56924 | -2.20919 | -0.98333 | -0.87518 | -1.1857 | -1.62157 | -1.46261 | -2.3467 |
| Q80YV4 | Q80YV4 | PANK4_M | Pank4 | 4'-phosphopantetheine phosphatase | | | | 0.255619 | 0.694146 | 0.702519 | 0.490014 | -0.38596 | -1.29881 | 0.520863 | -0.43415 | -1.3808 | -2.17835 | -1.47179 | -1.37678 | -1.92186 | -1.63877 | -2.14079 | -3.44198 | -1.59645 | -1.3818 | -1.14491 | -1.64497 | -1.26628 |
| Q6TCG2 | Q6TCG2 | PAQR9_M | Paqr9 | Membrane progesterone receptor epsilon | + | | | 1.213866 | 1.915243 | 3.857316 | 0.838544 | 1.039542 | 2.331148 | 0.611787 | 0.818558 | 1.631021 | -1.21817 | -0.98947 | -0.96401 | -1.11247 | -0.12606 | -0.06616 | -3.50224 | -2.53589 | -1.63806 | -2.51749 | -1.41564 | -0.41369 |
| Q99LX0 | Q99LX0 | PARK7_M | Park7 | Parkinson disease protein 7 homolog | + | | + | 1.24209 | 0.15787 | 3.997069 | 0.705658 | -0.7463 | -1.90583 | 1.75012 | 0.530951 | 7.399601 | 3.076424 | 3.095164 | 2.363874 | 1.820194 | 2.905551 | 2.861867 | 2.889555 | 2.966293 | 3.120193 | 2.556477 | 2.421107 | 2.28441 |
| Q9D0B6 | Q9D0B6 | PBDC1_M | Pbdc1 | Protein PBDC1 | | | | 0.649496 | -0.9816 | -1.73918 | 0.44641 | 0.390981 | 1.18495 | 0.017164 | 0.020268 | 0.054843 | -3.71107 | -3.66988 | -0.62251 | -0.54086 | -1.38172 | -0.96712 | -2.14485 | -3.27291 | -1.30009 | -0.64525 | -1.50064 | -0.88873 |
| Q05920 | Q05920 | PYC_MOU | Pc | Pyruvate carboxylase, mitochondrial | + | | | 1.099122 | -0.3311 | -3.32935 | 0.115071 | 0.112746 | 0.338475 | 0.358258 | 0.704711 | 0.960214 | 0.209932 | 0.332087 | 0.425374 | -0.23995 | 1.728322 | 0.320487 | 0.680588 | 0.523624 | -0.00296 | -0.03711 | 0.112029 | 0.527359 |
| P60335 | P60335 | PCBP1_M | Pcbp1 | Poly(rC)-binding protein 1 | | | | 0.372858 | 0.125252 | 0.997065 | 0.026213 | -0.01548 | -0.08297 | 0.049934 | -0.02016 | -0.15452 | 1.402061 | 1.531385 | 1.805004 | 1.828049 | 2.073568 | 1.951969 | 1.233769 | 1.449172 | 1.645749 | 2.018273 | 2.148366 | 1.917491 |

| | | | | | | | | | | | | | | | | | | | | | | | | | | | |
|---|---|---|---|---|---|---|---|---|---|---|---|---|---|---|---|---|---|---|---|---|---|---|---|---|---|---|---|
| Q61990 | Q61990;Q | PCBP2_M(Pcbp2 | Poly(rC)-binding protein 2 | | | | 0.533368 | 0.275148 | 1.414421 | 0.015708 | -0.00539 | -0.05027 | 0.010914 | -0.00796 | -0.03511 | 2.289929 | 2.415776 | 2.74094 | 2.788018 | 3.080319 | 2.630633 | 2.261777 | 1.893631 | 2.66525 | 2.874488 | 2.891847 | 2.835018 |
| P57722 | P57722 | PCBP3_M(Pcbp3 | Poly(rC)-binding protein 3 | | | | 0.211059 | -0.3346 | -0.58978 | 0.449475 | 0.490662 | 1.192884 | 0.225623 | 0.361092 | 0.626786 | -2.59668 | -1.46805 | -1.94884 | -1.62464 | -1.03978 | -2.14498 | -1.7562 | -1.63932 | -1.54291 | -2.01163 | -1.79061 | -2.11634 |
| Q91ZA3 | Q91ZA3 | PCCA_MO Pcca | Propionyl-CoA carboxylase alpha chain, mitochondrial | | | | 0.020333 | -0.01002 | -0.06475 | 0.262353 | 0.106549 | 0.719455 | 0.274495 | 0.348284 | 0.749951 | 0.116179 | 0.083666 | 0.072552 | -0.21529 | 1.023717 | 0.179727 | 0.263753 | -0.04388 | -0.21283 | -0.14301 | 0.059539 | 0.447336 |
| Q99MN9 | Q99MN9 | PCCB_MO Pccb | Propionyl-CoA carboxylase beta chain, mitochondrial | | | | 0.740427 | 0.113434 | 2.012661 | 0.039507 | -0.03027 | -0.12344 | 0.059479 | 0.045093 | 0.182507 | 0.219676 | 0.258183 | 0.158445 | -0.02195 | 0.542307 | 1.018368 | 0.072526 | 0.178465 | -0.15623 | 0.153258 | 0.801489 | 0.669 |
| Q8CFX3 | Q8CFX3 | Q8CFX3_M Pcdh1 | Protocadherin 1 | | | | 0.050287 | -0.32929 | -0.15556 | 0.093393 | 0.053687 | 0.278915 | 0.101903 | 0.034706 | 0.302464 | -4.00804 | -0.85982 | 1.029316 | 0.713445 | 1.379128 | 1.168195 | -3.51995 | -0.68933 | 0.707664 | 0.927723 | 1.193757 | 1.284155 |
| E9PX28 | E9PX28 | E9PX28_M Pcdh10 | Protocadherin 10 | | | | NaN | 0 | NaN | 0.372582 | 0.25209 | 0.996365 | 0.07524 | 0.312822 | 0.227859 | -3.82826 | -2.99962 | -2.34345 | -2.7478 | -2.79349 | -3.54232 | -3.82718 | -2.42354 | -2.94983 | -2.6456 | -2.15989 | -4.80156 |
| P70694 | P70694;Q | DHB5_MO Akr1c6 | Estradiol 17 beta-dehydrogenase 5 | + | | | 2.38285 | 0.47756 | 15.49076 | 0.419132 | -1.66179 | -1.11475 | 0.24993 | -0.28408 | -0.68819 | 5.24725 | 5.185995 | 6.773534 | 3.811769 | 5.118103 | 5.939559 | 4.742577 | 4.735548 | 7.125521 | 6.783361 | 5.771745 | 5.854072 |
| A2RS43 | A2RS43 | A2RS43_M Pcdh7 | Protocadherin 7 | | | | 0.046727 | -0.17291 | -0.14501 | 0.904996 | 0.77165 | 2.562882 | 0.172946 | 0.599648 | 0.491846 | -1.76323 | -3.42475 | -1.43729 | -1.19849 | -1.65093 | -3.24498 | -3.27638 | -1.56578 | -1.81314 | -2.36594 | -3.97018 | -2.12503 |
| F8VPK8 | F8VPK8 | F8VPK8_M Pcdh9 | Protocadherin 9 | | | + | 0.292986 | 0.91408 | 0.796333 | 0.754626 | -0.20184 | -2.05715 | 1.194414 | 0.582694 | 3.763364 | -2.07318 | -1.2472 | -0.36964 | -0.31095 | -0.2994 | -0.60907 | -1.50328 | -3.64527 | -0.04483 | -0.23208 | -1.03755 | -1.03631 |
| Q91Y18 | Q91Y12;Q | Q91Y18_M Pcdha12 | Protocadherin alpha 12 | | | | 0.049565 | -0.19908 | -0.15343 | 1.062664 | 0.60977 | 3.174082 | 0.440533 | -1.37266 | -1.16976 | -4.43534 | -1.88026 | -1.30287 | -1.01176 | -1.79531 | -1.00983 | -2.73144 | -3.18599 | -1.89247 | -1.64171 | -1.14139 | -1.91844 |
| Q6PB90;Q | Q91XZ6;Q | PCDB6_M Pcdhb14;Pcdhb6 | Protocadherin beta-14 | | | | NaN | 0 | NaN | 0.421493 | 0.495881 | 1.120796 | 0.460796 | 1.517481 | 1.222275 | -2.79774 | -3.0982 | -0.89884 | -0.03542 | -0.54305 | -0.76958 | -3.20674 | -1.39957 | -1.05985 | -0.86617 | -3.41014 | -0.93745 |
| Q91XW9 | Q91XX6;Q | Q91XW9_I Pcdhgc5 | Pcdhgc5 protein | | | | NaN | 0 | NaN | 0.574236 | 0.598021 | 1.52599 | 0.025574 | -0.03406 | -0.081 | -3.28329 | -3.70118 | -0.41003 | 0.050486 | -0.61475 | -1.42761 | -3.508 | -3.12942 | -1.0949 | -0.46068 | -0.87941 | -1.09484 |
| Q8BTQ0 | Q8BTQ0 | PCGF3_M(Pcgf3 | Polycomb group RING finger protein 3 | | | | 0.676173 | 1.921859 | 1.817475 | 0.315991 | -0.31746 | -0.854 | 0.128979 | 0.094153 | 0.375998 | -0.25448 | 0.060184 | -0.72252 | -0.23395 | -0.65907 | -0.31758 | -0.29451 | -0.29351 | -0.44097 | 0.11943 | -0.39931 | -0.76564 |
| Q3UK78 | Q3UK78 | PCGF5_M(Pcgf5 | Polycomb group RING finger protein 5 | | | | 0.948805 | 1.678766 | 2.723569 | 0.134316 | 0.232257 | 0.390273 | 0.774292 | -0.35798 | -2.11963 | -1.82387 | -0.60002 | -1.65426 | -1.01171 | -2.19221 | -2.50622 | -2.81672 | -2.96471 | -2.06618 | -1.0643 | -2.05347 | -1.929 |
| Q8BFV2 | Q8BFV2 | PCID2_MC Pcid2 | PCI domain-containing protein 2 | | | | 0.001752 | -0.00355 | -0.00569 | 0.377537 | -0.8294 | -1.0089 | 0.888614 | 0.388121 | 2.504453 | -3.29799 | -3.06484 | -3.56376 | -4.81674 | -2.97287 | -3.14784 | -2.5645 | -3.79122 | -3.89314 | -2.82857 | -3.32056 | -3.57639 |
| Q8BH04 | Q8BH04 | PCKGM_M Pck2 | Phosphoenolpyruvate carboxykinase [GTP], mitochondrial | | | | 0.385541 | -0.88211 | -1.02917 | 0.32882 | -0.94964 | -0.88619 | 0.413322 | -1.3364 | -1.09988 | -3.33184 | -1.6521 | -1.40916 | -3.55145 | -2.87262 | -0.61954 | -1.78089 | -1.43883 | -1.56197 | -1.49935 | 0.045533 | -0.86489 |
| Q9QYX7 | Q9QYX7 | PCLO_MO Pclo | Protein piccolo | | | | 0.907099 | 0.391811 | 2.570445 | 0.651543 | 0.434403 | 1.745138 | 0.172765 | -0.19524 | -0.49138 | -0.00041 | -0.14669 | 0.753723 | 1.433788 | 0.640289 | 0.539727 | -0.59864 | -0.33009 | 0.968637 | 1.350069 | 0.391113 | 1.179375 |
| P23506 | P23506 | PIMT_MOL Pcmt1 | Protein-L-isoaspartate(D-aspartate) O-methyltransferase | | | | 0.146224 | 0.085297 | 0.421891 | 0.845595 | 0.490494 | 2.355087 | 0.425755 | -0.28707 | -1.13173 | 0.559326 | 0.918547 | 1.447623 | 1.404928 | 0.724996 | 1.021981 | 0.560819 | 0.74646 | 0.728608 | 1.142954 | 1.366206 | 0.954909 |
| P59913 | P59913 | PCMD1_M Pcmtd1 | Protein-L-isoaspartate O-methyltransferase domain-containing protein 1 | | | | 0.202128 | -0.22932 | -0.56699 | 0.472916 | -0.25212 | -1.2539 | 0.292092 | 1.63654 | 0.794015 | -0.73462 | -1.48895 | 0.737086 | 1.062201 | 0.290656 | 0.840328 | -0.7382 | -1.02773 | 1.270111 | 1.033425 | -3.11374 | 0.971641 |
| Q8BHD8 | Q8BHD8 | PCMD2_M Pcmtd2 | Protein-L-isoaspartate O-methyltransferase domain-containing protein 2 | + | | | NaN | 0 | NaN | 0.158778 | -0.14953 | -0.45491 | 2.101432 | -1.49232 | -11.1719 | -3.1031 | -2.94405 | -1.8572 | -1.27005 | -3.26587 | -3.19433 | -3.61071 | -3.02681 | -1.56192 | -1.26628 | -1.86648 | -1.60909 |
| P12660 | P12660 | PCP2_MO Pcp2 | Purkinje cell protein 2 | | | | 0.349982 | 1.962156 | 0.939373 | NaN | 0 | NaN | 0.413651 | 0.290877 | 1.100724 | -3.41695 | 0.734369 | -3.45642 | -3.95138 | -3.2482 | -3.54132 | -3.06958 | -3.53731 | -3.63038 | -3.12068 | -3.46575 | -3.90553 |
| P63054 | P63054 | PCP4_MO Pcp4 | Calmodulin regulator protein PCP4 | | | | 0.018619 | 0.028276 | 0.0594 | 0.484801 | 1.532475 | 1.285077 | 0.465905 | 0.861243 | 1.235584 | -2.52769 | -0.89857 | -2.74577 | -2.21035 | -3.58504 | -2.8856 | -3.61773 | -4.10901 | -2.60026 | -3.64313 | -3.87474 |
| Q9QXV0 | Q9QXV0 | PCSK1N_M Pcsk1n | ProSAAS | | + | | 0.334893 | 0.303098 | 0.901437 | 1.041187 | 1.376774 | 3.085226 | 0.478558 | 0.426626 | 1.268679 | 0.350457 | 1.022934 | 1.425976 | 0.582195 | 0.128362 | -0.32915 | 0.383946 | 0.383248 | -0.22727 | -0.5181 | -0.28054 | -0.7735 |
| Q3UHX2 | Q3UHX2 | HAP28_M Pdap1 | 28 kDa heat- and acid-stable phosphoprotein | | + | | NaN | 0 | NaN | 1.018325 | 2.079937 | 2.992682 | 0.770892 | -1.25522 | -2.10875 | -3.94471 | -3.74869 | -1.1005 | -1.7175 | -2.68211 | -3.87254 | -3.04908 | -2.8958 | -4.11172 | -2.86615 | -2.0164 | -2.0278 |
| Q8VE70 | Q8VE70 | PDC10_M Pdcd10 | Programmed cell death protein 10 | | | | 0.75354 | 0.241007 | 2.053727 | 0.116316 | -0.04715 | -0.34185 | 0.936253 | -0.22296 | -2.67686 | 1.79102 | 1.998878 | 1.733152 | 1.98965 | 1.410677 | 1.554245 | 1.70844 | 1.599444 | 1.959278 | 1.857819 | 1.747662 | 1.663176 |
| P12815 | P12815 | PDCD6_M Pdcd6 | Programmed cell death protein 6 | | | | 0.786867 | 1.318584 | 2.160115 | 0.466515 | 0.52082 | 1.237177 | 0.380322 | 0.280756 | 1.015947 | -1.02545 | -0.54532 | -1.28066 | -0.54164 | -1.24445 | -0.80246 | -0.30805 | -2.71885 | -1.7443 | -1.64567 | -1.71248 | -0.95953 |
| Q9WU78 | Q9WU78 | PDC6I_MC Pdcd6ip | Programmed cell death 6-interacting protein | | | | 0.104456 | 0.074268 | 0.309485 | 0.801001 | -0.38492 | -2.20614 | 0.922477 | 0.296556 | 2.626214 | 1.159265 | 0.99299 | 0.44879 | 0.641119 | 1.064753 | 0.959368 | 1.22697 | 0.77675 | 0.78429 | 1.075452 | 0.61563 | 0.815378 |
| Q60598 | Q60598 | SRC8_MOI Cttn | Src substrate cortactin | + | | | 1.522792 | 0.471273 | 5.642187 | 0.899688 | -0.41787 | -2.54385 | 0.489955 | -0.35144 | -1.29865 | 1.33316 | 1.398942 | 1.758124 | 2.025591 | 2.313463 | 2.662985 | 0.818 | 0.971556 | 2.214315 | 2.405258 | 3.04628 | 2.633041 |
| P0C1Q2 | P0C1Q2 | PDE11_M Pde11a | Dual 3',5'-cyclic-AMP and -GMP phosphodiesterase 11A | | | | 0.70296 | -1.14028 | -1.39743 | 0.545743 | 0.308559 | 1.447923 | 0.017169 | -0.02435 | -0.05486 | -3.35067 | -1.72032 | -1.67429 | -1.38614 | -1.05572 | -0.27548 | -1.43152 | -1.35892 | -2.0071 | -1.68246 | -0.42939 | -0.8531 |
| Q3TIU4 | Q3TIU4 | PDE12_M Pde12 | 2',5'-phosphodiesterase 12 | | | | 0.429237 | -0.45051 | -1.14067 | 0.478786 | -0.46631 | -1.26928 | 0.33047 | -0.65017 | -0.89033 | -3.10051 | -3.75134 | -3.28006 | -2.78368 | -3.20194 | -4.03109 | -2.29424 | -3.08059 | -2.83643 | -2.29469 | -3.56751 | -2.36519 |
| Q61481 | Q61481 | PDE1A_M Pde1a | Dual specificity calcium/calmodulin-dependent 3',5'-cyclic nucleotide phosphodiesterase 1A | | | + | 0.206519 | 0.391324 | 0.578204 | 0.825485 | 0.621241 | 2.287201 | 1.083643 | 0.531776 | 3.262735 | -0.35146 | 0.204664 | 2.140619 | 2.014352 | 2.267361 | 1.945196 | -1.08176 | 0.152308 | 1.192067 | 1.720421 | 1.599331 | 1.549673 |
| Q01065 | Q01065 | PDE1B_M Pde1b | Dual specificity calcium/calmodulin-dependent 3',5'-cyclic nucleotide phosphodiesterase 1B | + | | | 0.272075 | 0.194495 | 0.743878 | 1.749473 | 0.494901 | 7.393946 | 0.476191 | 0.325234 | 1.262474 | 0.855175 | 0.53951 | 1.800308 | 1.93326 | 1.285478 | 0.77539 | 0.711296 | 0.2944 | 1.379693 | 1.364072 | 0.668882 | 0.741517 |
| Q9CR67 | Q9CR67 | TMM33_M Tmem33 | Transmembrane protein 33 | + | | | 1.771199 | 0.468737 | 7.586263 | 0.116911 | -0.08637 | -0.34347 | 0.243349 | -0.11608 | -0.67161 | 2.824056 | 2.798337 | 1.650903 | 1.486373 | 1.944553 | 1.706357 | 2.282024 | 2.402484 | 1.892643 | 1.417376 | 1.816279 | 2.066791 |
| A2AR02 | A2AR02 | PPIG_MOL Ppig | Peptidyl-prolyl cis-trans isomerase G | + | | | 1.39459 | 0.465439 | 4.82889 | 0.907079 | -0.41291 | -2.57037 | 0.818548 | 0.375941 | 2.264061 | 0.895855 | 1.049315 | -0.06989 | 0.220321 | 0.750458 | 0.499934 | 0.56548 | 0.448812 | 0.500132 | 0.44912 | 0.140255 | 0.358255 |
| B1AWC9 | B1AWC9 | B1AWC9_I Pde4b | Phosphodiesterase | | | | 0.864624 | 1.091179 | 2.420445 | 0.862117 | 0.438636 | 2.411768 | 0.292816 | -0.33425 | -0.7959 | 0.288513 | 0.188112 | 1.893855 | 2.247046 | 0.190445 | 0.632839 | -0.40485 | -1.30088 | 1.675309 | 1.58832 | 0.388905 | 1.102877 |
| Q9R0I9 | Q9R0I9 | MEP50_M Wdr77 | Methylosome protein WDR77 | | | | 2.241091 | 0.458493 | 13.14224 | 0.155891 | 0.101651 | 0.447345 | 0.644559 | 0.224358 | 1.72486 | -0.68603 | -0.61635 | -0.39764 | -0.21179 | 0.156381 | 0.388173 | -1.11148 | -1.10788 | -0.61373 | -0.19901 | -0.01113 | 0.106971 |
| Q8CG03 | Q8CG03 | PDE5A_M Pde5a | cGMP-specific 3',5'-cyclic phosphodiesterase | | | | 0.072226 | -0.32774 | -0.21927 | 0.523489 | -0.1996 | -1.38784 | 0.681702 | -1.5546 | -1.83389 | -3.53502 | -1.41526 | -1.53584 | -1.78154 | -2.1254 | -3.75592 | -1.37191 | -0.29435 | -3.20135 | -1.3843 | -1.53388 | -1.6182 |
| O55057 | O55057 | PDE6D_M Pde6d | Retinal rod rhodopsin-sensitive cGMP 3',5'-cyclic phosphodiesterase subunit delta | | | | 0.577714 | 1.275534 | 1.535612 | 0.259997 | -0.22233 | -0.71353 | 0.224727 | -0.36763 | -0.62451 | -1.12839 | -1.40951 | -1.65218 | -1.66559 | -1.39104 | -2.2095 | -3.36373 | -1.72624 | -1.74808 | -1.12504 | -1.65578 | -1.0095 |
| E9Q4S1 | E9Q4S1 | E9Q4S1_M Pde8b | High affinity cAMP-specific and IBMX-insensitive 3',5'-cyclic phosphodiesterase 8B | | | | 0.028996 | 0.077923 | 0.091528 | 0.459053 | 0.430433 | 1.217739 | 1.559183 | -0.57718 | -5.89467 | -2.07526 | -1.63801 | -1.15907 | -2.33755 | -3.14591 | -2.83699 | -1.68348 | -1.90086 | -1.63804 | -1.71142 |
| Q8BFY6 | Q8BFY6 | PEF1_MOI Pef1 | Peflin | + | | | 1.340038 | 0.454885 | 4.515663 | 0.312748 | -0.1631 | -0.84587 | 0.398591 | -0.14845 | -1.06232 | 2.056208 | 1.982831 | 1.071892 | 1.078292 | 2.026018 | 1.795674 | 1.470819 | 1.658451 | 1.045399 | 1.430995 | 2.138439 | 1.980155 |
| Q9D051 | Q9D051 | ODPB_MO Pdhb | Pyruvate dehydrogenase E1 component subunit beta, mitochondrial | + | | | 1.001476 | -0.87171 | -2.92579 | 0.271064 | 0.106094 | 0.741338 | 0.06086 | 0.081523 | 0.186523 | 1.659854 | 1.753654 | 2.520516 | 2.306494 | 2.248961 | 2.677766 | 2.872692 | 2.28424 | 2.212387 | 2.402435 | 2.762707 | 2.000975 |
| Q8BKZ9 | Q8BKZ9 | ODPX_MO Pdhx | Pyruvate dehydrogenase protein X component, mitochondrial | | | | 0.407624 | -0.40619 | -1.08533 | 0.139278 | -0.0546 | -0.40348 | 0.28455 | 0.078596 | 0.77518 | 1.355238 | 0.609622 | 1.566706 | 1.457453 | 1.411342 | 1.472606 | 1.421536 | 1.355713 | 1.690476 | 1.442878 | 1.460031 | 1.266726 |
| P27773 | P27773 | PDIA3_M(Pdia3 | Protein disulfide-isomerase A3 | | | | 0.571122 | -0.3513 | -1.51739 | 0.390969 | 0.723165 | 1.042943 | 0.403751 | 0.367756 | 1.075457 | -0.27444 | -0.12067 | 0.754879 | -0.55607 | 0.380078 | 0.381159 | 0.372119 | -0.06463 | -0.8499 | -0.39762 | 0.355216 | -0.32869 |
| P08003 | P08003 | PDIA4_M(Pdia4 | Protein disulfide-isomerase A4 | | | | 0.328448 | 1.484966 | 0.885256 | 0.679228 | 0.713715 | 1.826538 | 0.624137 | 0.343846 | 1.666135 | -1.25577 | -1.2407 | -1.39134 | -0.64899 | -0.91086 | -1.1113 | -1.41063 | -1.55778 | -1.85601 | -1.61175 | -1.53533 | -1.17452 |
| Q922R8 | Q922R8 | PDIA6_M(Pdia6 | Protein disulfide-isomerase A6 | | | | 0.848482 | 0.416419 | 2.364932 | 0.076171 | 0.163032 | 0.230508 | 0.746675 | 0.662635 | 2.032174 | -1.1619 | -1.18745 | -0.46974 | -1.69044 | -0.02048 | -0.65603 | -1.76671 | -1.41548 | -1.60048 | -0.88576 | -0.92781 | -1.07397 |
| Q8CI51 | Q8CI51 | PDLI5_MO Pdlim5 | PDZ and LIM domain protein 5 | | | | 0.152636 | 0.171125 | 0.438795 | 0.520061 | -0.42736 | -1.37865 | 0.125288 | -1.10068 | -0.36609 | -2.68724 | -2.62116 | -3.33411 | -1.71346 | -1.68934 | -1.19873 | -2.43648 | -3.21457 | -0.85125 | -1.3416 | -1.46767 | -1.71906 |
| Q3UV70 | Q3UV70 | PDP1_MO Pdp1 | [Pyruvate dehydrogenase [acetyl-transferring]]-phosphatase 1, mitochondrial | | | | 0.780655 | 1.16798 | 2.140061 | 0.386029 | 0.123877 | 1.030409 | 0.698607 | 0.527833 | 1.884522 | -2.48819 | -1.58801 | -2.05591 | -1.96099 | -2.23978 | -1.87823 | -3.51476 | -2.8974 | -2.24278 | -2.02187 | -2.80077 | -2.3729 |
| Q99K01 | Q99K01 | PDXD1_M Pdxdc1 | Pyridoxal-dependent decarboxylase domain-containing protein 1 | | | | 0.578551 | -0.47182 | -1.53793 | 0.546329 | 0.322196 | 1.449517 | 0.144171 | 0.286947 | 0.41646 | -2.20555 | -1.60151 | -0.95731 | -0.98036 | -0.1396 | -1.28835 | -1.37782 | -1.48559 | -1.51448 | -1.07069 | -1.38149 | -0.62035 |
| P62281 | P62281 | RS11_MOI Rps11 | Small ribosomal subunit protein uS17 | + | | | 1.75221 | 0.448706 | 7.417919 | 0.572354 | -0.18936 | -1.52079 | 0.290711 | -0.40754 | -0.79063 | 2.873619 | 2.932967 | 3.891065 | 3.94051 | 3.456019 | 3.473135 | 2.507298 | 2.401876 | 4.227179 | 3.983112 | 3.356724 | 4.387514 |
| P53026 | P53026 | RL10A_M(Rpl10a | Large ribosomal subunit protein uL1 | + | | | 2.443669 | 0.446746 | 16.62108 | 1.006243 | 0.632057 | 2.944606 | 0.562348 | 0.275238 | 1.493257 | 2.769838 | 2.757979 | 2.344116 | 3.415062 | 2.572284 | 2.29169 | 2.344036 | 2.500635 | 2.89443 | 2.2467 | 2.552807 |
| O35417 | O35417 | PDYN_MO Pdyn | Proenkephalin-B | + | + | + | NaN | 1.319309 | 3.194249 | 4.401368 | 1.07251 | 0.544811 | 3.215457 | -3.19213 | -4.48363 | -0.06515 | 0.237247 | -2.18637 | -2.28969 | -2.70769 | -3.44569 | -2.39839 | -3.81802 | -2.62147 | -2.94421 |
| E9Q1M1 | E9Q1M1 | E9Q1M1_I Pdzd2 | PDZ domain containing 2 | | | | NaN | 0 | NaN | 0.849303 | -0.6787 | -2.36774 | 0.085216 | -1.13883 | -0.25606 | -2.99025 | -3.3002 | -1.33438 | -0.77868 | -1.88783 | -0.83968 | -3.51642 | -3.03388 | -0.30737 | -0.44828 | -1.08593 | -1.36391 |
| B9EJ80 | B9EJ80 | PDZD8_M Pdzd8 | PDZ domain-containing protein 8 | | | | 0.12654 | 0.06034 | 0.369454 | 0.092071 | 0.274959 | 0.275235 | 0.187184 | 0.285117 | 0.528648 | -2.64945 | -2.9743 | -2.39301 | -3.0205 | -1.66766 | -2.5984 | -2.88599 | -2.85509 | -3.93017 | -2.14555 | -2.69075 |
| Q69ZS0 | Q69ZS0 | PZRN3_M Pdzrn3 | E3 ubiquitin-protein ligase PDZRN3 | | | | NaN | 0 | NaN | 0.210739 | -0.22127 | -0.58897 | 0.158562 | -0.36358 | -0.45435 | -3.2876 | -2.61942 | -1.14811 | -2.19759 | -3.26299 | -2.80103 | -2.9053 | -1.12955 | -0.43572 | -2.96386 | -1.76958 |
| E9PUZ9 | E9PUZ9 | E9PUZ9_M Pdzrn4 | PDZ domain containing RING finger 4 | + | | | 1.117496 | 0.289671 | 3.409783 | 0.02469 | 0.029784 | 0.078276 | 0.015091 | -0.02214 | -0.04833 | 1.190776 | 1.086033 | 0.79857 | 1.374161 | 0.058098 | 0.822558 | 0.915624 | 0.781845 | 1.305484 | 0.807679 | 0.209637 | 0.715102 |
| Q62048 | Q62048 | PEA15_M Pea15 | Astrocytic phosphoprotein PEA-15 | | | | NaN | 0 | NaN | 0.658223 | 1.197342 | 1.764627 | 0.30603 | 1.090491 | 0.829031 | -4.17945 | -3.13741 | 0.093367 | -1.2629 | -0.55436 | -1.87861 | -3.44038 | -3.53122 | -1.75903 | -1.80519 | -1.1704 | -3.44356 |
| P70296 | P70296 | PEBP1_M(Pebp1 | Phosphatidylethanolamine-binding protein 1 | | | | 0.13718 | -0.29627 | -0.3979 | 0.336922 | 1.103204 | 0.906534 | 0.039115 | -0.08663 | -0.12226 | -0.21442 | 1.101829 | 2.706428 | 2.272764 | 1.232064 | 0.953722 | 0.391768 | 1.088168 | 0.402472 | 0.370293 | 1.874274 | 0.404764 |
| Q80TZ3 | Q80TZ3 | AUXI_MOI Dnajc6 | Putative tyrosine-protein phosphatase auxilin | + | | | 2.35242 | 0.437719 | 14.95408 | 1.409289 | 0.499289 | 4.916485 | 0.253882 | 0.405637 | 0.698147 | 2.412275 | 2.469848 | 3.33596 | 3.177768 | 3.913408 | 2.75172 | 1.99804 | 2.008645 | 2.69388 | 2.82127 | 2.912654 | 2.9412 |
| Q80X73 | Q80X73 | PELO_MOI Pelo | Protein pelota homolog | | | | 0.078722 | 0.38417 | 0.237746 | 0.285445 | -0.34246 | -0.77742 | 0.357665 | -1.14499 | -0.95872 | -1.216 | -1.28138 | -0.66164 | -1.5258 | -0.90402 | -2.36231 | -3.24842 | -0.01771 | -0.6655 | -0.83701 | -1.11689 | -0.76346 |
| Q11136 | Q11136 | PEPD_MOI Pepd | Xaa-Pro dipeptidase | | | | 0.390771 | -0.4156 | -1.04753 | 0.614128 | -1.10789 | -1.63765 | 0.060658 | -0.0982 | -0.18594 | -1.48558 | -1.72805 | -2.02995 | -1.2511 | -2.02795 | -1.41298 | -3.56898 | -0.81435 | -1.82342 | -1.24152 | -1.19285 | -1.25168 |
| Q9EPU0 | Q9EPU0 | RENT1_M(Upf1 | Regulator of nonsense transcripts 1 | + | | | 1.40439 | 0.435502 | 4.887137 | 0.359061 | 0.220298 | 0.962238 | 0.006196 | -0.00616 | -0.02004 | -0.66989 | -0.75976 | 0.194968 | 0.360097 | 0.271358 | -0.24051 | -1.22728 | -1.07338 | -0.1563 | 0.270772 | -0.14858 | 0.191734 |
| Q9R0A0 | Q9R0A0 | PEX14_M(Pex14 | Peroxisomal membrane protein PEX14 | | | | 0.841929 | 1.992428 | 2.342619 | 0.947615 | 0.957462 | 2.719116 | 0.643796 | -2.16766 | -1.72265 | -0.61059 | -1.76063 | 0.176479 | 1.076042 | -0.28957 | -0.93677 | -1.66797 | -2.20364 | 0.021366 | 0.546112 | -0.39214 | 0.501128 |
| O09012 | O09012 | PEX5_MO Pex5 | Peroxisomal targeting signal 1 receptor | + | | | 0.707209 | 1.357801 | 1.910538 | 1.247594 | 0.837198 | 4.024808 | 0.871824 | -0.88733 | -2.44547 | -0.79683 | -1.95151 | 0.440458 | 0.610807 | -1.65001 | -1.22728 | -2.31718 | -3.14856 | -0.50133 | -0.12176 | -0.84625 | -0.25689 |
| Q8C437 | Q8C437 | PEX5R_M(Pex5l | PEX5-related protein | | | | 0.21135 | 0.115279 | 0.590521 | 0.255669 | -0.06526 | -0.70264 | 0.30009 | -0.17512 | -0.81414 | 0.672982 | 0.548695 | 0.387762 | 0.228567 | 0.502769 | 0.135685 | 0.68062 | 0.310498 | 0.325559 | 0.421296 | 0.606503 | 0.382189 |
| Q9CWM4 | Q9CWM4 | PFD1_MO Pfdn1 | Prefoldin subunit 1 | | | | 0.327608 | 0.768956 | 0.883148 | 0.190097 | 0.23525 | 0.536142 | 0.867196 | -0.94651 | -2.42936 | -2.85683 | -1.11715 | -3.73907 | -1.08316 | -2.02035 | -1.30723 | -2.79457 | -2.71732 | -1.55001 | -0.74272 | -0.56023 | -0.87432 |
| O70591 | O70591 | PFD2_MO Pfdn2 | Prefoldin subunit 2 | | | | 0.277317 | 0.170075 | 0.757035 | 0.594723 | -0.29023 | -1.58298 | 0.196708 | -0.16671 | -0.55311 | -0.08122 | 0.216751 | 0.375114 | 0.015791 | 0.085456 | 0.658939 | -0.27046 | 0.065838 | 0.449125 | 0.522233 | 0.446063 | 0.631744 |
| Q3UWL8 | Q3UWL8 | Q3UWL8_I Pfdn4 | Prefoldin subunit 4 | | | + | 0.020031 | -0.03611 | -0.06434 | 0.366941 | 0.644559 | 0.982117 | 1.255432 | -1.24694 | -4.06469 | -2.5126 | -3.58066 | -2.58268 | -2.42283 | -3.55129 | -2.96458 | -3.18305 | -2.83799 | -3.79873 | -2.49591 | -1.92124 | -2.10075 |
| Q9WU28 | Q9WU28 | PFD5_MO Pfdn5 | Prefoldin subunit 5 | + | | | 0.209251 | 0.52417 | 0.585173 | 1.538638 | 0.771783 | 5.750864 | 0.273156 | -0.27531 | -0.74659 | -1.76885 | -3.53244 | -1.44482 | -1.63491 | -1.63517 | -1.92464 | -1.01729 | -3.33234 | -2.40639 | -2.2169 | -1.8542 | -1.84377 |
| Q03958 | Q03958 | PFD6_MO Pfdn6 | Prefoldin subunit 6 | | | | 0.06078 | 0.089483 | 0.186292 | 0.29045 | 0.514973 | 0.789974 | 0.530579 | -0.44043 | -1.4069 | -1.80026 | -0.96883 | -0.30296 | -0.51458 | -0.64864 | -1.27466 | -1.71467 | -1.23339 | -1.56699 | -0.2805 | -0.51626 | -0.52617 |
| P12382 | P12382 | PFKAL_M Pfkl | ATP-dependent 6-phosphofructokinase, liver type | | | | 0.486604 | -0.66968 | -1.28982 | 0.248329 | -0.53796 | -0.93321 | 0.06557 | 0.082992 | 0.200157 | -0.38232 | -0.95484 | 0.633773 | -0.44189 | 0.965832 | 1.716839 | 0.434255 | -0.43206 | 0.429894 | 0.837899 | 1.434178 | 0.082509 |
| P47857 | P47857 | PFKAM_M Pfkm | ATP-dependent 6-phosphofructokinase, muscle type | | | | 0.575694 | -0.37551 | -1.53002 | 0.396137 | -0.53595 | -1.05607 | 0.147163 | 0.163452 | 0.424372 | 1.963215 | 2.208675 | 2.754537 | 1.758079 | 2.486818 | 3.12269 | 2.605837 | 2.117086 | 2.695747 | 2.888762 | 2.858712 | 2.423891 |
| Q9WUA3 | Q9WUA3 | PFKAP_M Pfkp | ATP-dependent 6-phosphofructokinase, platelet type | | | | 0.451055 | -0.26177 | -1.19698 | 0.188163 | -0.2502 | -0.53117 | 0.118713 | 0.086735 | 0.34835 | 1.026562 | 0.755841 | 2.185346 | 1.257781 | 1.627323 | 1.950654 | 1.324737 | 0.981205 | 1.889406 | 2.054122 | 1.891621 | 1.512885 |
| P62962 | P62962 | PROF1_M(Pfn1 | Profilin-1 | | | | 0.475573 | -1.8203 | -1.26085 | 0.01144 | -0.03661 | -0.03678 | 0.161454 | 0.354243 | 0.461916 | -2.6832 | 0.129033 | 1.29161 | -0.60095 | 0.455796 | 1.355876 | 0.8705 | 0.215932 | 0.07336 | 0.690516 | 1.17256 | -0.06937 |
| Q9JJV2 | Q9JJV2 | PROF2_M(Pfn2 | Profilin-2 | | | | 0.356154 | 0.379737 | 0.954913 | 0.879676 | -0.36893 | -2.47294 | 0.357162 | 0.139016 | 0.957457 | 5.999756 | 5.588518 | 4.594378 | 4.839727 | 5.922071 | 6.053846 | 5.074018 | 5.754781 | 5.17088 | 5.001085 | 5.978326 | 5.71956 |
| Q9D0J1 | Q9D0J1 | PGAM1_M Pgam1 | Phosphoglycerate mutase 1 | | | | 0.426238 | -0.43541 | -1.13297 | 0.082498 | 0.280806 | 0.248414 | 0.245692 | 0.501813 | 0.677513 | 0.205995 | 0.974603 | 2.316889 | 0.079534 | 0.803498 | 1.900391 | 1.027497 | 1.02392 | 1.079742 | 0.755068 | 1.347924 | 0.352339 |
| Q8BX10 | Q8BX10 | PGAM5_M Pgam5 | Serine/threonine-protein phosphatase PGAM5, mitochondrial | | | | 0.889167 | 0.817973 | 2.506053 | 0.374497 | 0.282117 | 1.001207 | 0.113387 | -0.16249 | -0.3339 | 0.752433 | 0.936053 | 1.4568 | 2.008581 | 0.823168 | 0.55934 | 0.539442 | -0.2869 | 1.393276 | 1.50787 | 1.385311 | 1.322184 |
| Q3UUQ7 | Q3UUQ7 | PGAP1_M Pgap1 | GPI inositol-deacylase | | | | NaN | 0 | NaN | 0.242748 | -0.77441 | -0.67009 | 0.264911 | -0.14784 | -0.75288 | -3.73632 | -3.50121 | -1.75172 | -3.58172 | -1.23452 | -1.55525 | -3.58677 | -2.95495 | -2.95827 | -1.18635 | -1.51242 | -2.6595 |
| Q5EBJ4 | Q5EBJ4 | ERMIN_M(Ermn | Ermin | + | | | 2.99242 | 0.421411 | 31.32408 | 0.363269 | 0.243163 | 0.97285 | 0.193262 | -0.07782 | -0.54427 | 2.836305 | 2.811881 | 0.754804 | 1.245144 | 0.695046 | 0.929061 | 2.397038 | 2.408326 | 0.708168 | 0.805455 | 0.972052 | 0.807698 |
| Q9DCD0 | Q9DCD0 | 6PGD_MO Pgd | 6-phosphogluconate dehydrogenase, decarboxylating | | | | 0.324003 | -1.48469 | -0.8741 | 0.38458 | -0.7414 | -1.02674 | 0.050779 | -0.31041 | -0.15701 | -3.13021 | -2.81693 | -1.16264 | -2.60304 | -2.85552 | -0.70038 | -3.18017 | 0.202413 | -1.08922 | -1.19367 | -0.77606 | -3.15902 |
| P09411 | P09411 | PGK1_MOI Pgk1 | Phosphoglycerate kinase 1 | | | | 0.79323 | -0.85446 | -2.18077 | 0.028172 | -0.08681 | -0.089 | 0.015477 | 0.033097 | 0.049542 | 0.895529 | 1.302468 | 2.465164 | 0.516701 | 1.181899 | 1.982036 | 2.288295 | 1.618613 | 1.530236 | 1.625252 | 2.083893 | 1.013848 |
| Q61285 | Q61285 | ABCD2_M Abcd2 | ATP-binding cassette sub-family D member 2 | + | | | 1.505196 | 0.402227 | 5.523704 | 0.053271 | 0.061293 | 0.164349 | 0.592995 | -0.22543 | 1.57814 | 0.151008 | 0.294288 | -0.68036 | -0.17743 | -0.91355 | -1.16973 | 0.19265 | -0.50814 | -0.62448 | -1.2559 | -0.75299 | -0.87943 |
| Q8CAA7 | Q8CAA7 | PGM2L_M Pgm2l1 | Glucose 1,6-bisphosphate synthase | | | | 0.741914 | -1.45411 | -2.0173 | 0.363563 | -0.61716 | -0.97359 | 0.030624 | -0.04502 | -0.09651 | -1.39798 | -2.36737 | 0.007207 | -1.14892 | -0.52567 | 0.189868 | 0.104969 | -0.96209 | -0.21383 | 0.30644 | 0.176535 | -0.42229 |
| Q9CYR6 | Q9CYR6 | AGM1_MO Pgm3 | Phosphoacetylglucosamine mutase | | | + | 0.238545 | 0.425219 | 0.65948 | 0.83411 | 0.871821 | 2.316171 | 1.394919 | 0.628354 | 4.830834 | 0.072249 | 0.343804 | 1.640334 | 2.339006 | 1.861929 | 1.741085 | 0.413129 | -0.84751 | 1.258012 | 0.977685 | 1.288339 | 1.057967 |
| Q8CHP8 | Q8CHP8 | PGP_MOU Pgp | Glycerol-3-phosphate phosphatase | | | | 0.859765 | -0.61361 | -2.40365 | 0.937047 | -0.91153 | -2.67979 | 0.002768 | 0.008642 | 0.524674 | 0.881567 | -0.14469 | -0.41166 | 0.415735 | 0.963618 | 1.499286 | 1.134196 | 0.946225 | 0.320494 | 0.853942 | 0.520674 |
| O55022 | O55022 | PGRC1_M Pgrmc1 | Membrane-associated progesterone receptor component 1 | | | | 0.739594 | -0.53853 | -2.01007 | 0.725568 | -0.76847 | -1.96665 | 0.143767 | -0.28586 | -0.41539 | 1.164013 | 1.44601 | 1.879327 | 1.104941 | 1.484281 | 1.615727 | 2.07135 | 2.313221 | 2.20799 | 2.730346 | 1.608089 |
| Q80UU9 | Q80UU9 | PGRC2_M Pgrmc2 | Membrane-associated progesterone receptor component 2 | | | | 0.377968 | -0.41105 | -1.00999 | 0.034048 | 0.024344 | 0.106943 | 0.123715 | -0.0635 | -0.36185 | -0.65934 | 0.030744 | -0.34761 | -0.76629 | 0 | -0.34709 | 0.312568 | -0.11907 | -0.49188 | -0.67071 | -0.136 | -0.0841 |
| Q8BHF7 | Q8BHF7 | PGPS1_M Pgs1 | CDP-diacylglycerol--glycerol-3-phosphate 3-phosphatidyltransferase, mitochondrial | | | | 0.192491 | 0.753435 | 0.542294 | 0.90388 | 0.461313 | 2.558874 | 0.211689 | -0.58368 | -0.59138 | -3.32896 | -0.73289 | -1.03552 | -1.21375 | -3.70729 | -1.82479 | -3.27972 | -2.28899 | -1.74266 | -1.42923 | -1.88543 | -2.47929 |
| Q2M3X8 | Q2M3X8 | PHAR1_M Phactr1 | Phosphatase and actin regulator 1 | | | | 0.538371 | 1.127026 | 1.427937 | 0.884853 | 0.428748 | 2.491161 | 0.441177 | -0.55757 | -1.17143 | 0.620915 | -2.07757 | 2.694425 | 2.816128 | 0.757078 | 1.055669 | -2.22401 | -3.48717 | 2.165537 | 2.48752 | 1.012004 | 1.91619 |
| Q922R1 | Q922R1 | PHAF1_M Phaf1 | Phagosome assembly factor 1 | + | | | 0.48716 | 0.998318 | 1.291287 | 1.554702 | -0.404 | -5.86299 | 0.098903 | -0.10032 | -0.29419 | -1.05291 | -1.64476 | -0.2951 | -0.1687 | -0.4225 | -0.55694 | -1.63291 | -3.0614 | 0.144659 | 0.199536 | -0.72371 | -0.05551 |
| Q7TMK9 | Q8VHM5;C | HNRPQ_M Syncrip | Heterogeneous nuclear ribonucleoprotein Q | + | | | 2.215485 | 0.400628 | 12.75711 | 0.716171 | 0.483299 | 1.937828 | 0.229164 | 0.397182 | 0.635758 | 0.005356 | -0.03075 | 0.810097 | 1.150061 | 0.973537 | -0.27582 | -0.38762 | -0.43902 | 0.314526 | 0.685032 | -0.03979 | -0.05686 |
| O35129 | O35129 | PHB2_MO Phb2 | Prohibitin-2 | | | | 0.926077 | 0.406615 | 2.639384 | 0.088928 | -0.07725 | -0.26646 | 0.363804 | -0.16033 | -0.9742 | 5.076535 | 5.396518 | 4.744497 | 5.074533 | 4.981342 | 5.310066 | 5.196878 | 5.057933 | 5.22515 | 4.748389 | 5.297688 | 5.314374 |
| Q80TL4 | Q80TL4 | PHF24_M Phf24 | PHD finger protein 24 | | | | 0.02108 | 0.027901 | 0.06708 | 0.032814 | -0.03273 | -0.10319 | 0.084639 | -0.08812 | -0.25444 | -0.70319 | -0.30106 | 0.523292 | -0.083 | -0.04267 | -0.64357 | -0.9159 | -0.36799 | 0.346211 | 0.159545 | -0.08275 | -0.42725 |
| P83870 | P83870 | PHF5A_M Phf5a | PHD finger-like domain-containing protein 5A | | | | 0.265611 | -0.40499 | -0.72764 | 0.759521 | -0.74333 | -2.06123 | 0.718171 | 0.328847 | 1.943943 | -0.60819 | 0.451002 | -1.60031 | -1.09296 | -0.001 | 0.293735 | 0.093961 | 0.658837 | -0.34997 | -0.85963 | -0.09942 | -0.26554 |
| Q8K2Z2 | Q8K2Z2 | PACS1_M Pacs1 | Phosphofurin acidic cluster sorting protein 1 | + | | | 1.341875 | 0.399238 | 4.529915 | 0.843315 | -0.28159 | -2.34733 | 0.014601 | -0.00852 | -0.04878 | 0.193882 | 0.177598 | 1.684221 | 1.637667 | 1.415284 | 1.350622 | -0.12566 | -0.30133 | 1.825909 | 2.055253 | 1.212266 | 1.237276 |
| P18826 | P18826 | KPB1_MO Phka1 | Phosphorylase b kinase regulatory subunit alpha, skeletal muscle isoform | | | | 0.680551 | 0.448086 | 1.830469 | 0.884963 | -0.17119 | -2.49155 | 0.002252 | 0.001603 | 0.007315 | 0.442568 | 0.280059 | -0.13973 | -0.00976 | -0.3425 | -0.2654 | 0.144142 | -0.31769 | 0.118753 | 0.074126 | -0.08951 | -0.52074 |
| Q8BWJ3 | Q8BWJ3 | KPB2_MO Phka2 | Phosphorylase b kinase regulatory subunit alpha, liver isoform | | | | 0.592017 | 0.827515 | 1.575408 | 0.722067 | -0.30641 | -1.95588 | 0.499296 | -1.15112 | -1.32335 | 0.059667 | 0.126611 | 1.746416 | 1.436593 | -0.80609 | 0.906332 | 0.028504 | -0.56026 | 1.874564 | 1.921269 | 1.047777 | 1.354707 |
| Q9DCL9 | Q9DCL9 | PUR6_MO Paics | Bifunctional phosphoribosylaminoimidazole carboxylase/phosphoribosylaminoimidazole succinocarboxamide synthetase | + | | | 1.461583 | 0.396543 | 5.239694 | 0.311816 | -0.30882 | -0.84354 | 0.691292 | 0.304267 | 1.862536 | 0.239605 | 0.160868 | 0.185972 | -0.15646 | 0.293439 | 0.345954 | -0.26339 | -0.13322 | -2.15E-05 | 0.647168 | -0.14581 | 0.176667 |
| Q9JJK2 | Q9JJK2 | LANC2_M Lancl2 | LanC-like protein 2 | + | | | 1.440602 | 0.390224 | 5.135281 | 0.031851 | 0.002135 | 0.041545 | 0.163933 | 1.716141 | 2.724004 | 2.822436 | 3.23467 | 3.262113 | 3.313854 | 3.350803 | 2.325799 | 2.266724 | 3.270476 | 3.257776 | 2.916508 | 2.933565 |
| Q8K0S0 | Q8K0S0 | PHYIP_MC Phyhip | Phytanoyl-CoA hydroxylase-interacting protein | | | | 0.276614 | 0.22991 | 0.75527 | 0.862843 | -0.25292 | -2.41428 | 0.675438 | -0.45866 | -1.8153 | 1.916313 | 2.444582 | 2.892124 | 3.003847 | 2.984355 | 3.469652 | 1.79922 | 2.101855 | 3.112284 | 3.289536 | 3.756212 | 3.615029 |
| Q8BGT8 | Q8BGT8 | PHIPL_MO Phyhipl | Phytanoyl-CoA hydroxylase-interacting protein-like | | | | 0.059342 | -0.0761 | -0.18211 | 0.41602 | 0.060086 | 1.106782 | 0.850066 | -0.33175 | -2.3724 | 0.11942 | 0.274411 | 0.269 | 0.174215 | -0.08782 | 0.189918 | 0.706944 | -0.12091 | 0.12504 | 0.188003 | 0.366364 | 0.399234 |
| Q2TBE6 | Q2TBE6 | P4K2A_M Pi4k2a | Phosphatidylinositol 4-kinase type 2-alpha | | | | 0.258309 | 0.168503 | 0.71714 | 0.178482 | 0.476111 | 0.50619 | 0.489477 | 0.588064 | 1.297393 | -3.17724 | -3.88409 | -2.09285 | -0.91212 | -2.41147 | -1.89782 | -2.25733 | -3.23932 | -3.71788 | -2.36922 | -2.93825 | -3.11619 |
| E9Q3L2 | E9Q3L2 | PI4KA_M(Pi4ka | Phosphatidylinositol 4-kinase alpha | | | | 0.847313 | 0.172931 | 2.360941 | 0.104705 | -0.03655 | -0.10894 | 0.226074 | -0.37783 | -0.62573 | 1.179414 | 1.426261 | 2.724401 | 3.920365 | 2.445091 | 2.448697 | 0.450367 | 0.442888 | 3.260783 | 3.633337 | 3.767093 |
| Q7M6Y3 | Q7M6Y3 | PICAL_MO Picalm | Phosphatidylinositol-binding clathrin assembly protein | | | | NaN | 0 | NaN | 0.022221 | 0.017878 | 0.070627 | 0.78434 | 0.486875 | 2.151944 | -3.51072 | -2.79001 | -2.82868 | -2.91117 | -2.20543 | -2.2552 | -2.99399 | -3.65154 | -2.63806 | -3.13755 | -2.49231 | -2.94207 |
| Q62083 | Q62083 | PICK1_MO Pick1 | PRKCA-binding protein | | | | 0.007199 | -0.04523 | -0.02325 | 0.387411 | -0.09721 | -1.03391 | 0.096194 | -0.12356 | -0.28669 | -0.25365 | -3.66197 | -0.27375 | -0.18459 | -0.1376 | -0.92144 | -0.97487 | -2.85029 | -0.21474 | -0.04918 | -0.58525 | -2.22667 |
| Q8K358 | Q8K358 | PIGU_MOI Pigu | Phosphatidylinositol glycan anchor biosynthesis class U protein | | | | 0.024453 | 0.016547 | 0.077543 | 0.059558 | 0.255172 | 0.182737 | 0.136387 | -0.1167 | -0.39579 | 0.036695 | 0.008841 | -0.24596 | -1.70842 | -0.06962 | -0.36864 | -0.24481 | -0.48585 | -0.36886 | -0.46083 | -0.43591 | -0.47685 |
| Q8BTP0 | Q8BTP0 | PIGZ_MOL Pigz | GPI mannosyltransferase 4 | + | | | 1.238528 | -0.60501 | -3.97921 | 0.093361 | -0.21037 | -0.28837 | 0.003294 | -0.01272 | -0.01069 | -0.49739 | -0.68176 | -2.85145 | -3.60044 | -3.68704 | -1.93949 | -0.10547 | 0.136349 | -3.67055 | -2.36601 | -1.99275 | -3.60835 |
| Q9CS84 | Q9CS84 | NRX1A_M Nrxn1 | Neurexin-1 | + | | | 2.336189 | 0.372591 | 14.67535 | 0.503517 | 0.097496 | 1.33454 | 0.154871 | 0.274991 | 0.444467 | 0.077757 | 0.127729 | 1.295549 | 1.397931 | 1.636818 | 0.526762 | -0.26534 | -0.27435 | 1.197122 | 1.301365 | 0.534068 | 1.07953 |
| O35904 | O35904 | PK3CD_M Pik3cd | Phosphatidylinositol 4,5-bisphosphate 3-kinase catalytic subunit delta isoform | | | | 0.36974 | 0.685985 | 0.989184 | 0.071883 | 0.063083 | 0.212884 | 0.304287 | -0.17262 | -0.62466 | -0.42417 | -0.72875 | -0.90869 | 0.207397 | -1.939 | -0.58589 | 0.273337 | -0.08077 | -0.50945 | -0.70266 |
| E9PVA8 | E9PVA8 | GCN1_MO Gcn1 | Stalled ribosome sensor GCN1 | + | + | | 2.42179 | 0.370078 | 16.20538 | 1.374073 | 0.382644 | 4.708925 | 0.244806 | -0.23101 | -0.67528 | -0.09046 | -0.06033 | 0.435434 | 0.593125 | -0.16283 | 0.059662 | -0.46268 | -0.43187 | 0.151293 | 0.111978 | -0.14408 | 0.502934 |
| Q68FF6 | Q68FF6 | GIT1_MOL Git1 | ARF GTPase-activating protein GIT1 | + | + | | 1.774458 | 0.3601 | 7.615517 | 1.178729 | -0.19145 | -3.2418 | 0.376479 | -0.19143 | -1.00622 | 0.027856 | 0.07953 | 1.635499 | 1.693613 | 1.498001 | 1.69362 | -0.34601 | -0.2668 | 1.911611 | 1.806594 | 1.624063 | 1.950419 |
| O08908 | O08908 | P85B_MOI Pik3r2 | Phosphatidylinositol 3-kinase regulatory subunit beta | | | | 0.597285 | 1.333835 | 1.590156 | 0.422977 | -0.33053 | -1.1246 | 0.575678 | -0.32191 | -1.52998 | -1.37619 | -3.05129 | -1.556 | -1.1494 | -1.03485 | -1.1147 | -3.59349 | -3.50166 | -0.80992 | -1.23443 | -0.95944 | -0.54628 |
| Q9Z1T6 | Q9Z1T6 | FYV1_MOI Pikfyve | 1-phosphatidylinositol 3-phosphate 5-kinase | | | | 0.234689 | 0.744891 | 0.499759 | 0.022634 | -0.01552 | -0.07377 | 0.692636 | -0.19099 | -1.86697 | -2.17708 | -1.63788 | -1.48082 | -1.32943 | -1.51161 | -1.69976 | -1.53807 | -3.86797 | -1.19325 | -1.58594 | -1.37658 | -1.45459 |
| Q9QUR7 | Q3ULQ2;C | PIN1_MOL Pin1 | Peptidyl-prolyl cis-trans isomerase NIMA-interacting 1 | | | | 0.726704 | -1.49633 | -1.97015 | 0.866515 | -0.79893 | -2.427 | 0.133693 | -0.17651 | -0.38861 | -1.20266 | -0.37327 | -2.00768 | -0.03683 | 1.167692 | -0.3304 | -0.04058 | 0.077248 | 0.243634 | -0.49421 |
| Q99J27 | Q99J27 | ACATN_M Slc33a1 | Acetyl-coenzyme A transporter 1 | + | | | 1.678679 | 0.35929 | 6.800365 | 0.445518 | 0.474356 | 1.182642 | 0.162137 | 0.319972 | 0.4637 | 0.494365 | 0.401137 | -1.04144 | -0.24218 | -0.57632 | -0.52146 | 0.06359 | 0.113331 | -1.15167 | -1.08086 | -1.55836 | -0.17936 |
| Q78J03 | Q78J03 | MSRB2_M Msrb2 | Methionine-R-sulfoxide reductase B2, mitochondrial | + | | | 1.870502 | 0.336479 | 8.573317 | 1.184725 | -0.29821 | -3.71727 | 0.037098 | -0.03978 | -0.11799 | 0.637864 | 0.68497 | 0.010789 | 0.160714 | 0.427455 | 1.058099 | 0.356437 | 0.293559 | 0.407328 | 0.365598 | 0.663243 | 0.901868 |
| Q9R0N7 | Q9R0N7 | SYT7_MOL Syt7 | Synaptotagmin-7 | + | + | + | 1.341502 | 0.332458 | 4.523834 | 1.562715 | -0.37962 | -5.91966 | 1.16857 | -0.417 | -3.64143 | -0.0871 | -0.04777 | 0.990368 | 0.915098 | 0.802751 | 1.019893 | -0.4707 | -0.32908 | 1.280047 | 1.284751 | 1.291906 | 1.364732 |
| P70182 | P70182 | PI51A_MO Pip5k1a | Phosphatidylinositol 4-phosphate 5-kinase type-1 alpha | | + | | 0.571724 | 0.890732 | 1.519055 | 1.10399 | -0.30872 | -4.3562 | 0.724003 | -0.61228 | -1.96183 | -1.23952 | -1.3883 | 0.070558 | 0.160501 | 0.080117 | 0.0135 | -1.62301 | -2.78628 | 0.369048 | 0.465944 | 0.348773 | 0.969396 |
| P70181 | P70181 | PI51B_MO Pip5k1b | Phosphatidylinositol 4-phosphate 5-kinase type-1 beta | | + | | 0.074069 | -0.44768 | -0.22452 | 0.629315 | -0.34974 | -2.97332 | 0.557684 | -0.31139 | -1.48048 | -2.41606 | 0.508525 | -1.13136 | -1.24692 | 0.146336 | 0.324826 | -0.65248 | -1.3597 | -0.76916 | -0.51247 | 0.476869 | 0.687379 |
| O70161 | O70161 | PI51C_MO Pip5k1c | Phosphatidylinositol 4-phosphate 5-kinase type-1 gamma | | | | 1.042946 | 0.64421 | 3.092434 | 0.105399 | 0.097782 | 0.312074 | 0.28044 | -0.25379 | -0.76989 | 0.327197 | 0.686247 | 2.514792 | 2.482179 | 2.248545 | 1.909256 | -0.03181 | -0.24316 | 2.087797 | 2.713069 | 2.050049 | 2.615328 |
| Q8BSF4 | Q8BSF4 | PISD_MOL Pisd | Phosphatidylserine decarboxylase proenzyme, mitochondrial | | | | 0.602245 | -2.46654 | -1.60409 | 0.116318 | 0.068936 | 0.341858 | 0.140287 | -0.11593 | -0.40617 | -3.88783 | -0.82285 | -0.02486 | 0.17228 | -0.44211 | -0.38819 | 0.237159 | -0.01477 | 0.180702 | -0.17111 | -0.58337 | -0.0156 |
| Q8BWR2 | Q8BWR2 | PITH1_MO Pithd1 | PITH domain-containing protein 1 | | | | 0.708017 | 0.623199 | 1.912992 | 0.833371 | 0.47354 | 2.313678 | 0.061597 | -0.03337 | -0.18866 | -0.26365 | -0.31041 | 0.278647 | 0.67789 | -0.16389 | -0.01079 | -0.58553 | -1.23516 | -0.04096 | 0.050715 | -0.21341 | 0.105456 |
| Q8VE97 | Q8VE97;Q | SRSF4_M Srsf4 | Serine/arginine-rich splicing factor 4 | + | | | 1.421898 | 0.327356 | 4.989705 | 1.231714 | 0.181634 | 4.642212 | 0.847396 | 0.343698 | 2.841542 | 0.387724 | 0.429134 | 0.762853 | 0.591076 | 0.468461 | 0.965799 | 0.213829 | 0.299542 | 1.024866 | 1.085051 | 0.709042 | 0.859675 |
| Q8K4R4 | Q8K4R4 | PITC1_MO Pitpnc1 | Cytoplasmic phosphatidylinositol transfer protein 1 | | | | 0.009642 | 0.008602 | 0.031058 | 0.5487 | -0.2162 | -1.45596 | 0.215988 | -0.09007 | -0.60233 | -0.79811 | -1.05459 | -0.70405 | -0.71146 | -1.01882 | -1.10356 | -1.18044 | -0.68947 | -0.64 | -0.34311 | -0.77661 | -1.03852 |
| O35954 | O35954 | PITM1_MO Pitpnm1 | Membrane-associated phosphatidylinositol transfer protein 1 | | | | 0.862422 | 0.436484 | 2.412825 | 0.538717 | -0.24673 | -1.43158 | 0.285059 | -0.12776 | -0.76467 | -0.74072 | -0.45037 | -0.18637 | -0.42536 | -0.29384 | -0.70714 | -0.9241 | -1.13996 | -0.18333 | 0.065066 | -0.41905 | -0.23641 |
| Q6ZPQ6 | Q6ZPQ6 | PITM2_MO Pitpnm2 | Membrane-associated phosphatidylinositol transfer protein 2 | | | | 0.544907 | -0.5498 | -1.45655 | 0.378722 | -0.35811 | -0.83718 | 0.774688 | -0.39849 | -2.21068 | -1.74678 | -1.46839 | 0.718064 | 0.910323 | 0.143568 | 0.466905 | -0.733 | -1.4322 | 1.074097 | 1.187311 | 0.582829 | 0.806043 |
| Q3UHE1 | Q3UHE1 | PITM3_MO Pitpnm3 | Membrane-associated phosphatidylinositol transfer protein 3 | | | | NaN | 0 | NaN | 0.029692 | -0.05695 | -0.09386 | 0.179573 | -0.6995 | -0.50901 | -3.47234 | -3.22499 | -2.0943 | -0.88744 | -1.24699 | -3.90006 | -4.33633 | -2.59057 | -1.35884 | -1.50899 | -1.51513 | -2.23293 |
| O55176 | O55176 | PJA1_MOL Pja1 | E3 ubiquitin-protein ligase Praja-1 | + | | | NaN | 0 | NaN | 1.146343 | 0.602026 | 3.539913 | 0.902422 | -0.78254 | -2.55364 | -2.69836 | -3.0389 | -1.73849 | -1.44512 | -3.41783 | -2.80628 | -3.06388 | -2.71451 | -2.1077 | -2.27997 | -2.30932 | -2.3497 |
| Q8K0U4 | Q8K0U4 | PJA2_MOL Pja2 | E3 ubiquitin-protein ligase Praja-2 | | | | 0.380816 | -1.02113 | -1.0172 | 0.43281 | 0.68113 | 1.14986 | 0.183555 | -0.4978 | -0.53186 | -0.3613 | -3.57656 | -1.75573 | -1.19589 | -3.38015 | -3.13254 | -3.73306 | -1.76254 | -2.67899 | -1.63489 | -1.80597 | -2.11111 |
| P52480 | P52480 | PKPM_MO Pkm | Pyruvate kinase PKM | | | | 1.182791 | -1.1756 | -3.70812 | 0.264376 | -0.60906 | -0.72484 | 0.015309 | -0.02839 | -0.02813 | 2.649871 | 3.111144 | 3.446852 | 4.495568 | 2.581342 | 3.640505 | 3.727791 | 4.824755 | 4.185647 | 4.176778 | 4.361411 | 4.642787 |
| P97350 | P97350 | PKP1_MOL Pkp1 | Plakophilin-1 | | | | 0.053264 | 0.082342 | 0.164331 | 0.220507 | 0.157666 | 0.613808 | 0.350939 | 0.559441 | 0.941781 | 2.078598 | 0.208684 | -0.21032 | -0.4497 | -0.37287 | -0.05724 | 0.810707 | 0.312528 | -0.71495 | -0.2604 | -0.20182 | -1.34717 |
| Q9CQ73 | Q9CQ73 | PKP2_MOL Pkp2 | Plakophilin-2 | | | | NaN | 0 | NaN | 0.578046 | 0.587572 | 1.536532 | 0.170705 | -0.28776 | -0.48496 | -2.65619 | -1.61034 | -3.21197 | -2.51171 | -1.324 | -0.91727 | -2.76285 | -3.4322 | -3.6349 | -3.17392 | -1.3903 | -2.27546 |
| O70456 | O70456 | 1433S_MC Sfn | 14-3-3 protein sigma | + | | | 2.578827 | 0.307744 | 19.43396 | 1.715495 | -1.39217 | -7.10239 | 0.375379 | 0.34303 | 1.003458 | 5.74227 | 5.713499 | 4.350762 | 4.740134 | 5.371918 | 5.541194 | 5.42676 | 5.41352 | 5.960406 | 5.914838 | 5.444737 | 4.782314 |
| P27612 | P27612 | PLAP_MOI Plaa | Phospholipase A-2-activating protein | | | | 0.477212 | 0.946725 | 1.265149 | 0.307146 | 0.369465 | 0.831829 | 0.787955 | 0.538607 | 2.163638 | -0.54473 | -1.56068 | -0.34539 | -0.81339 | -0.25578 | 0.23984 | -2.54891 | -1.44995 | -1.20363 | -0.57133 | -0.57022 | -0.52294 |
| Q9Z1B3 | Q9Z1B3 | PLCB1_M(Plcb1 | 1-phosphatidylinositol 4,5-bisphosphate phosphodiesterase beta-1 | + | | | 1.190577 | -0.33376 | -3.74505 | 0.236197 | -0.26004 | -0.65355 | 0.271158 | -0.15786 | -0.74157 | -1.2517 | -1.17388 | 0.288138 | -0.25402 | 0.412671 | -0.01303 | -0.79885 | -0.9692 | -0.01416 | 0.568348 | 0.36059 | 0.35477 |
| Q8K4S1 | Q8K4S1 | PLCE1_M(Plce1 | 1-phosphatidylinositol 4,5-bisphosphate phosphodiesterase epsilon-1 | | | | 0.278643 | 0.681212 | 0.760362 | 0.249555 | -0.87734 | -0.68726 | 0.025491 | 0.10912 | 0.08075 | -1.27764 | -2.818 | -0.45637 | -1.00582 | -1.22894 | -2.75746 | -3.18638 | -2.2715 | -0.99543 | -1.71208 | -3.21676 | -0.98788 |
| A2AP18 | A2AP18 | PLCH2_M(Plch2 | 1-phosphatidylinositol 4,5-bisphosphate phosphodiesterase eta-2 | | | | 0.079702 | 0.13215 | 0.240519 | 0.548622 | -0.43394 | -1.49622 | 0.186213 | -0.26596 | -0.50972 | -3.26503 | -1.0693 | -1.72805 | -0.98416 | -1.79011 | -0.85055 | -1.46018 | -2.43645 | -1.30066 | -1.38035 | -1.17823 | -0.90012 |
| Q3USB7 | Q3USB7 | PLCL1_M(Plcl1 | Inactive phospholipase C-like protein 1 | | | | 0.514019 | -1.09294 | -1.3625 | 0.193473 | 0.666832 | 0.544815 | 0.317286 | 0.545548 | 0.857252 | -3.15109 | -1.57407 | -1.8949 | -1.88215 | -0.82182 | -2.03321 | -1.12231 | -1.41697 | -3.7793 | -1.33141 | -2.16832 | -1.77781 |
| Q8K394 | Q8K394 | PLCL2_M(Plcl2 | Inactive phospholipase C-like protein 2 | | | | 0.874905 | 1.001441 | 2.456222 | 1.190457 | -0.43696 | -3.74448 | 1.006479 | 0.357445 | 2.945541 | -0.86816 | -1.12599 | -0.28339 | -0.08193 | 0.05689 | -0.07246 | -0.30336 | -0.63797 | 0.190913 | -0.28255 | -0.46791 |
| O35405 | O35405 | PLD3_MOI Pld3 | 5'-3' exonuclease PLD3 | | | | 0.295836 | 0.636553 | 0.803476 | 0.654435 | 0.334388 | 1.471598 | 0.076994 | -0.04269 | -0.17297 | -0.22462 | 0.409437 | -0.07314 | -0.44891 | 1.011162 | 0.025565 | 1.401997 | 0.432982 | 0.467467 | -0.04821 | -0.22587 | -0.15988 |
| Q9QXS1 | Q9QXS1 | PLEC_MO Plec | Plectin | | | | 0.321389 | -0.33084 | -0.86754 | 0.182349 | -0.02697 | -0.51618 | 0.117284 | -0.07901 | -0.34448 | 1.137024 | 1.006107 | 1.407159 | 1.31891 | 1.07588 | 1.176723 | 1.778105 | 1.026712 | 1.362015 | 1.418 | 0.981558 | 1.429069 |
| Q8BUL6 | Q8BUL6 | PKHA1_M Plekha1 | Pleckstrin homology domain-containing family A member 1 | | | | 0.363 | -0.5553 | -1.69237 | 0.715237 | -0.11253 | -1.48052 | 0.069891 | -0.00549 | -0.00866 | -1.73099 | -2.00917 | -1.54581 | -0.59617 | -0.91437 | -1.21049 | -1.79701 | -2.31302 | -1.19055 | -1.24015 | -1.09565 | -1.26584 |
| Q9ERS5 | Q9ERS5 | PKHA2_M Plekha2 | Pleckstrin homology domain-containing family A member 2 | + | | | NaN | 0 | NaN | 1.15645 | 1.140485 | 3.585358 | 0.43596 | 1.566592 | 1.157971 | -3.61783 | -3.40347 | -0.83781 | -0.76441 | -1.53642 | -1.20266 | -2.49078 | -3.3783 | -2.25757 | -1.62563 | -4.27867 | -1.59358 |
| E9Q6H8 | E9Q6H8 | E9Q6H8_I Plekha5 | Pleckstrin homology domain containing, family A member 5 | + | | | 0.097877 | 0.105254 | 0.291351 | 1.062108 | -0.47853 | -3.25618 | 0.356084 | 0.731216 | 0.954738 | -3.78394 | -2.59527 | -0.51298 | -0.39854 | -2.23596 | -1.95212 | -3.36761 | -2.57263 | -0.6735 | -0.34206 | -1.27515 | -1.06431 |
| Q6KU45 | Q6KU45 | PKHG5_M Plekhg5 | Pleckstrin homology domain-containing family G member 5 | | | | NaN | 0 | NaN | 0.052067 | -0.08219 | -0.16081 | 0.234566 | -0.33226 | -0.80662 | -3.56693 | -1.7718 | -1.24415 | -0.6993 | -0.69594 | 0.226795 | -2.92179 | -2.6567 | -1.46082 | -3.71237 | -0.85253 | -2.00113 |
| Q9D3P8 | Q9D3P8 | PLRKT_M Plgrkt | Plasminogen receptor (KT) | | | | 1.122438 | 0.346452 | 3.431672 | 0.121283 | 0.00675 | -0.03945 | 0.348859 | 0.240406 | 0.936545 | 0.43956 | 0.240853 | 0.373322 | 0.595079 | 0.929911 | 0.575785 | 0.011676 | -0.02417 | 0.621379 | 0.360529 | 0.698294 | 0.32659 |
| Q9DBG5 | Q9DBG5 | PLIN3_MO Plin3 | Perilipin-3 | | | | 0.273005 | 0.339194 | 0.742713 | 0.925544 | -1.01995 | -2.67007 | 0.326627 | 0.178855 | 0.938457 | -1.4461 | -1.57977 | -0.4658 | -0.18091 | -0.63855 | -0.6902 | -2.33145 | -3.59001 | -1.40723 | -2.66863 | -0.70643 | -1.83496 |
| Q9CXW3 | Q9CXW3 | CYBP_MO Cacybp | Calcyclin-binding protein | + | | | 1.402082 | 0.290809 | 4.873355 | 1.192587 | -0.16897 | 3.539997 | 0.087072 | 0.257778 | 0.207466 | -0.14049 | -0.25705 | -0.55763 | -0.63298 | -0.72881 | -0.05087 | -0.47674 | -0.50242 | -1.0749 | -0.4536 | 0.008699 | -0.85467 |
| Q99JY8 | Q99JY8 | PLPP3_M(Plpp3 | Phospholipid phosphatase 3 | | | | 0.17244 | -0.15036 | -0.49053 | 0.390914 | -0.59644 | -1.0428 | 0.106809 | -0.33526 | -0.31394 | -1.47575 | -0.87561 | -1.15046 | -2.2851 | -1.80398 | -0.32439 | -0.96272 | -1.08791 | -1.04868 | -1.194 | 0.031813 | -1.48966 |
| Q8BFZ2 | Q8BFZ2 | PLPR1_M(Plppr1 | Phospholipid phosphatase-related protein type 1 | | | | NaN | 0 | NaN | 0.755285 | -0.87274 | -2.05993 | 0.465351 | -0.86662 | -1.23414 | -0.30875 | -2.54558 | -0.45358 | -2.56841 | -3.09035 | -3.78543 | -3.13609 | -1.02976 | -2.31858 | -1.31506 | -2.61646 |
| Q8BVC9 | Q8BVC9 | PLPR2_M(Plppr2 | Phospholipid phosphatase-related protein type 2 | | | | NaN | 0 | NaN | 0.178453 | 0.394767 | 0.506114 | 0.448678 | -0.19877 | -1.19082 | -3.05001 | -3.73422 | -2.35501 | -2.14359 | -1.84626 | -1.83678 | -3.2265 | -3.8415 | -3.41687 | -1.87127 | -1.47591 | -1.8096 |
| Q7TPB0 | Q7TPB0 | PLPR3_M(Plppr3 | Phospholipid phosphatase-related protein type 3 | | | | 0.295382 | -1.30206 | -0.80234 | 0.66736 | -0.39446 | -1.79144 | 0.21921 | 0.517986 | 0.610515 | -3.34457 | -1.6884 | -1.8658 | -1.84282 | -1.86221 | -1.61257 | -2.46065 | -0.60073 | -3.82812 | -1.25067 | -1.69101 | -1.81972 | -2.28947 |
| Q7TME0 | Q7TME0 | PLPR4_M(Plppr4 | Phospholipid phosphatase-related protein type 4 | | | + | 0.078443 | 0.32134 | 0.236956 | 0.282047 | -0.05274 | -0.7689 | 1.769234 | -0.28016 | -7.56868 | -3.41265 | -0.97022 | -3.39558 | 1.449776 | 2.153004 | 2.188692 | -3.10243 | -1.92312 | 1.488269 | 1.406567 | 2.418576 | 2.483438 |
| Q99K51 | Q99K51 | PLST_MOL Pls3 | Plastin-3 | + | + | | 0.871635 | 0.14722 | 2.444808 | 0.132313 | -0.09147 | -0.38492 | 1.609504 | 0.154413 | 6.257445 | 0.179283 | 0.480624 | 0.448782 | 0.104733 | 0.011267 | 0.067347 | 0.540954 | 0.320063 | 0.281575 | -0.10954 | -0.07082 | -0.32149 |
| P70206 | P70206 | PLXA1_M(Plxna1 | Plexin-A1 | | | | 0.542885 | 1.062466 | 1.440165 | 0.280024 | -0.11879 | -0.77887 | 0.264015 | 0.095991 | 0.723633 | -1.24367 | -1.10837 | -0.39291 | -0.69786 | 0.206157 | 0.007099 | -1.61554 | -0.37368 | -0.40032 | -1.40562 | -0.77706 | 0.098331 |
| P70207 | P70207 | PLXA2_M(Plxna2 | Plexin-A2 | | | | 0.272552 | 0.411523 | 0.745076 | 0.489331 | 0.315045 | 1.323437 | 0.242269 | -0.11628 | -0.86888 | -0.32056 | -0.22801 | 1.224375 | 0.303381 | 0.594196 | 0.893751 | 0.549872 | 1.429108 | 0.292002 | 0.054274 | 0.322224 | 0.784569 | 0.881623 |
| Q80UG2 | Q80UG2 | PLXA4_M(Plxna4 | Plexin-A4 | | | | 0.244153 | -0.16038 | -0.67363 | 0.893992 | 0.273935 | 0.900434 | 0.161793 | -0.00564 | -0.12145 | -1.18029 | 0.011743 | 2.066175 | 1.446846 | 1.049994 | 0.825235 | 0.415731 | 1.015772 | 2.051049 | 1.880795 | 2.005345 |
| Q64332 | Q64332 | SYN2_MOI Syn2 | Synapsin-2 | + | | | 1.302761 | 0.289322 | 4.371918 | 0.837935 | 0.761897 | 2.329087 | 0.532064 | 0.601128 | 1.410904 | 2.166768 | 2.402442 | 4.140351 | 3.655636 | 4.970901 | 4.124094 | 1.791492 | 1.837245 | 2.916342 | 3.356751 | 3.993863 | 3.898876 |
| B2RXS4 | B2RXS4 | PLXB2_M(Plxnb2 | Plexin-B2 | | | | 0.378768 | -0.64517 | -1.01201 | 0.752286 | -0.24678 | -2.04987 | 0.049782 | 0.098961 | 0.422065 | 0.294349 | -0.32566 | -2.26396 | -1.65972 | -1.84687 | -1.76821 | -1.16338 | -0.67098 | -1.52908 | -2.13858 | -0.39344 | -3.64523 | -1.55998 |
| Q3UH93 | Q3UH93 | PLXD1_M(Plxnd1 | Plexin-D1 | | | | NaN | 0 | NaN | 1.493401 | -1.03966 | -5.44556 | 0.079522 | 0.323823 | 0.240009 | -3.66297 | -2.98966 | -2.09395 | -2.4382 | -1.84863 | -3.80666 | -3.15377 | -2.83013 | -1.30901 | -1.14381 | -0.47965 | -2.22308 |
| O35621 | O35621 | PMM1_MC Pmm1 | Phosphomannomutase 1 | | | | 0.197269 | 0.122141 | 0.554549 | 0.945177 | 0.026676 | 0.145489 | 0.40406 | 0.455736 | 0.244532 | 1.209121 | -3.59022 | -1.17828 | 1.126198 | 1.11828 | -0.79128 | -3.21044 | -1.65445 | -2.37044 | -0.38 | -2.02244 | -1.14288 | -1.33851 | -0.73956 |
| Q9DC61 | Q9DC61 | MPPA_MO Pmpca | Mitochondrial-processing peptidase subunit alpha | | | | 0.629222 | -0.68551 | -1.68068 | 0.417037 | -0.88795 | -1.10938 | 0.324413 | 0.874192 | 1.329273 | -0.6542 | -0.50088 | 0.020168 | 0.03066 | -0.0922 | -0.25658 | -1.23963 | -0.80037 | -0.08002 | -0.00256 | -1.9819 | -0.28992 |
| Q9CXT8 | Q9CXT8 | MPPB_MO Pmpcb | Mitochondrial-processing peptidase subunit beta | | | | 0.342657 | -0.9947 | -0.92095 | 0.256825 | -0.1844 | -0.68995 | 0.654549 | 0.194332 | 1.753895 | -1.414 | -0.30168 | -1.18291 | -1.18234 | -0.9383 | -0.73355 | -1.88826 | -0.83801 | -0.77004 | -1.25832 | -0.07263 | -0.98788 |
| G3X9N3 | G3X9N3 | G3X9N3_I Pnma8b | PNMA family member 8B | | | | 0.346883 | 1.56701 | 0.931648 | 0.373734 | -0.11226 | -0.40458 | 0.230739 | -0.06048 | -0.31747 | 0.076755 | 0.328817 | 0.543259 | -0.35422 | -0.45475 | -0.76771 | 0.592485 | 0.517364 | 0.160892 | 0.198177 |
| O55023 | O55023 | IMPA1_MC Impa1 | Inositol monophosphatase 1 | + | | | 1.65517 | 0.28861 | 6.611269 | 0.451554 | -0.25952 | -1.19827 | 0.290187 | 0.091536 | 0.789314 | 4.005661 | 3.931879 | 3.255828 | 3.69994 | 3.931879 | 3.711238 | 3.778513 | 3.671925 | 3.252106 | 3.724464 | 3.724881 |
| P23492 | P23492 | PNPH_MO Pnp | Purine nucleoside phosphorylase | | | | 0.489433 | -1.40576 | -1.29728 | 0.424199 | -0.49642 | -1.17274 | 0.017523 | 0.009844 | 0.055597 | -2.06562 | -0.48298 | -2.05934 | -2.8379 | -1.99643 | -1.66203 | -2.06453 | -1.27254 | -2.1577 | -1.7467 | -1.89361 | -1.78453 |
| Q3TRM4 | Q3TRM4 | PLPL8_M Pnpla8 | Patatin-like phospholipase domain-containing protein 8 | | | | 0.932854 | 0.807741 | 2.664297 | 0.67253 | 0.779639 | 1.806691 | 0.021387 | -0.07739 | -0.0684 | -2.414 | -2.74007 | -1.50845 | -1.42126 | -1.39471 | -3.22479 | -3.12917 | -3.64038 | -2.67382 | -1.81518 | -2.90737 | -1.55681 |
| Q6KAU4 | Q6KAU4 | POF1B_M Pof1b | Protein POF1B | | | | 0.581335 | 1.245479 | 1.556772 | 0.29349 | -0.76707 | -0.70013 | 0.265994 | 0.837522 | 1.182026 | -0.27583 | 1.210824 | -2.08054 | -1.18446 | -1.78809 | -1.89014 | -1.77607 | -2.37549 | -1.75476 | -1.91151 | -1.92297 | -2.23095 |
| Q8BG81 | Q8BG81 | PDIP3_M(Poldip3 | Polymerase delta-interacting protein 3 | | | | 0.868854 | -0.79672 | -2.43512 | 0.429244 | 0.307851 | 1.14069 | 0.135515 | 0.248531 | 0.393469 | -0.98287 | -0.34455 | -1.47961 | -1.7388 | -2.5382 | -2.29322 | -2.93809 | -0.29789 | -1.4272 | -2.07699 | -2.83389 | -2.04459 |
| Q8CFI7 | Q8CFI7 | RPB2_MOI Polr2b | DNA-directed RNA polymerase II subunit RPB2 | | | | 0.186491 | 0.554018 | 0.526863 | 0.745597 | -0.58284 | -2.0288 | 0.380266 | -0.4048 | -1.0581 | 1.692218 | -1.17836 | -1.26441 | -0.03905 | -1.11979 | -2.75708 | -0.97343 | -3.01123 | -0.36904 | -0.77564 | -1.16719 | -0.37845 |
| P97760 | P97760 | RPB3_MOI Polr2c | DNA-directed RNA polymerase II subunit RPB3 | | | | 0.248357 | 0.56701 | 0.653367 | 0.377484 | 0.134705 | 1.205849 | 0.126389 | 0.588355 | 0.011189 | -0.02126 | -0.05308 | -3.25063 | -3.41995 | -2.20931 | -1.43536 | -2.86496 | -3.51356 | -2.61733 | -1.59953 | -0.01219 | -3.53005 |
| Q8R2R1 | Q8R2R1 | POMT1_M Pomt1 | Protein O-mannosyl-transferase 1 | | | | NaN | 0 | NaN | 0.2756 | -0.41417 | -0.75273 | 0.799428 | 0.716089 | 2.200992 | -3.47584 | -3.20728 | -3.72075 | -3.78765 | -2.86498 | -3.30804 | -3.10147 | -3.00688 | -2.7052 | -2.97487 | -4.04088 | -3.56433 |
| Q9JJJ7 | Q9JJJ7 | PORCN_M Porcn | Protein-serine O-palmitoleoyltransferase porcupine | + | | | 0.716342 | 0.698617 | 1.935851 | 1.396763 | 0.490607 | 4.841755 | 0.162803 | -0.20392 | -0.46568 | 0.297345 | -0.409 | -0.15114 | -0.30426 | -0.45037 | -0.82634 | -0.68255 | -0.76653 | -0.71219 | -0.46773 | 0.33196 |
| Q9D0S9 | Q9D0S9 | IPYR_MOL Ppa1 | Inorganic pyrophosphatase | | | | 0.340211 | -0.19657 | -0.9148 | 0.05596 | -0.14575 | -0.10831 | 0.090163 | 0.10189 | 0.233524 | 1.184087 | 1.401961 | 2.012219 | 1.286148 | 1.578689 | 1.971416 | 1.669244 | 1.565637 | 1.656 | 1.633776 | 1.770143 | 1.516208 |
| Q91VM9 | Q91VM9 | IPYR2_MC Ppa2 | Inorganic pyrophosphatase 2, mitochondrial | + | | | 0.193653 | -0.86943 | -0.54528 | 0.300346 | -0.04365 | -0.01122 | 1.039075 | -0.2881 | -3.07659 | -0.39929 | -0.20992 | -0.04041 | -0.05671 | -0.09613 | -0.45051 | -0.81281 | -0.63212 | -0.07965 | -0.46834 | -0.52696 | -0.9245 |
| Q9CR37 | Q9CR37 | PPDPF_M(Ppdpf | Pancreatic progenitor cell differentiation and proliferation factor | | | | 1.160139 | 2.235396 | 3.262402 | 0.590287 | 0.210927 | 0.860545 | 0.589443 | -1.02138 | -2.03292 | -0.17458 | -0.80662 | -0.34239 | -1.09087 | -0.98223 | -0.59316 | -1.4257 | -1.08098 | -0.38987 | -0.91129 | -0.69919 | -2.03273 |
| Q8BSS9 | Q8BSS9 | LIPA2_MO Ppfia2 | Liprin-alpha-2 | | | | 0.921789 | 1.992288 | 2.623703 | 0.16262 | -0.05938 | -0.46499 | 0.036148 | 0.152136 | -0.96327 | -0.64304 | 0.573377 | 0.565333 | 0.997396 | 1.457725 | -0.25317 | -3.53771 | 0.501092 | 0.756373 | 1.123446 | 1.258315 |
| Q6GQS1 | Q6GQS1 | SCMC3_M Slc25a23 | Mitochondrial adenyl nucleotide antiporter SLC25A23 | + | | | 1.548661 | 0.279301 | 5.682051 | 0.32194 | -0.21542 | -1.09793 | 0.290134 | 0.140453 | 0.78918 | 1.771771 | 1.798263 | 1.193142 | 2.806525 | 2.468158 | 1.582074 | 1.905056 | 2.36482 | 1.974691 | 2.449353 | 2.538085 |
| B8Q136 | B8JJ96;B8 | B8JJ96_M Ppfia4 | Liprin-alpha 4 | | | | NaN | 0 | NaN | 0.542478 | -0.38335 | -1.43906 | 0.145825 | -0.13661 | -0.42083 | -3.32813 | -1.64782 | -1.21623 | -0.99068 | -1.72738 | -2.03526 | -4.66923 | -1.98986 | -1.12966 | -1.1089 | -1.21594 | -1.06655 |
| P17742 | P17742 | PPIA_MOL Ppia | Peptidyl-prolyl cis-trans isomerase A | | | | 0.562649 | -0.68109 | -1.49408 | 0.047225 | 0.160856 | 0.146496 | 0.044117 | -0.06368 | -0.13725 | 1.450044 | 2.355665 | 3.671544 | 1.476883 | 2.014537 | 2.442551 | 2.531302 | 2.636591 | 2.374257 | 2.452457 | 2.703944 | 1.880513 |
| P24369 | P24369 | PPIB_MOL Ppib | Peptidyl-prolyl cis-trans isomerase B | | | | 0.331583 | 0.578986 | 0.893127 | 0.373734 | 0.701454 | 0.999278 | 0.544537 | 0.486294 | 1.449446 | -0.3438 | -0.07357 | 0.735955 | 0.388473 | -0.05843 | -0.11009 | -1.28115 | -0.8955 | -1.18712 | -0.30951 | -0.96899 |
| P61222 | P61222 | ABCE1_M(Abce1 | ATP-binding cassette sub-family E member 1 | + | | | 1.398469 | 0.26457 | 4.851874 | 1.772954 | -0.57475 | -7.60201 | 0.320729 | -0.15071 | -0.86589 | -0.54748 | -0.54989 | -0.78299 | -0.67343 | -0.43174 | -0.23437 | -0.75874 | -0.86777 | -0.20557 | -0.10135 | 0.034759 | -0.29945 |
| A2ARP1 | A2ARP1 | VIP1_MOU Ppip5k1 | Inositol hexakisphosphate and diphosphoinositol-pentakisphosphate kinase 1 | | | | 0.381329 | 0.607009 | 1.014897 | 0.256163 | -0.36982 | -0.70389 | 0.612132 | 0.23186 | 1.631996 | -1.98096 | -3.0488 | -1.85891 | -1.16211 | -2.58534 | -2.38423 | -2.85708 | -3.3887 | -1.53396 | -1.74741 | -2.81681 | -2.61628 |
| Q9R269 | Q9R269 | PEPL_MOI Ppl | Periplakin | | | | NaN | 0 | NaN | 0.597329 | -1.41073 | -1.59048 | 0.542824 | -0.52849 | -1.44 | -3.58969 | -2.20172 | -3.07056 | -3.59711 | -3.61947 | -4.02389 | -2.89924 | -3.05289 | -3.17584 | -1.18061 | -2.96396 | -2.88196 |
| P49443 | P49443 | PPM1A_M Ppm1a | Protein phosphatase 1A | + | + | | 0.561002 | 0.543942 | 1.489565 | 2.083394 | 1.279067 | 10.93946 | 1.184685 | 0.224561 | 3.717077 | -0.32796 | -0.61841 | -0.49732 | -0.27614 | -1.45765 | -1.38009 | -0.68208 | -1.35217 | -1.62783 | -1.70377 | -1.59711 | -1.68976 |
| Q8T0L0 | Q8T0L0 | PPM1E_M Ppm1e | Protein phosphatase 1E | + | + | | 1.211536 | -0.15368 | -3.84596 | 1.206179 | -0.6512 | -3.02424 | 0.382215 | -0.14401 | -1.02074 | 1.570178 | 1.110814 | 1.954072 | 2.073781 | 2.569389 | 2.670578 | 1.205783 | 1.264347 | 2.458283 | 2.871961 | 2.895688 | 2.632294 |
| Q61074 | Q61074 | PPM1G_M Ppm1g | Protein phosphatase 1G | | | | 0.066619 | 0.144705 | 0.203179 | 0.057703 | 0.20296 | 0.17733 | 0.405247 | -0.74155 | -1.07927 | -2.32687 | -3.7413 | -2.08039 | -2.70617 | -3.78816 | -3.31145 | -3.22988 | -1.71931 | -3.53127 | -3.45267 | -2.16383 |
| Q3UYC0 | Q3UYC0 | PPM1H_M Ppm1h | Protein phosphatase 1H | | | | 0.767704 | -0.16655 | -2.09858 | 0.289368 | 0.146526 | 0.78726 | 0.76288 | 0.461 | 2.083248 | -0.74369 | -0.86897 | 0.759189 | 0.018395 | -0.11337 | 0.277564 | -0.68851 | -0.91305 | 0.130573 | -0.10314 | 0.27516 | -0.48264 |
| Q8BVQ5 | Q8BVQ5 | PPME1_M Ppme1 | Protein phosphatase methylesterase 1 | | | | 0.702041 | -0.55553 | -1.89819 | 0.191146 | -0.13783 | -0.53965 | 0.2312 | -0.15916 | -0.64091 | -0.89945 | -1.36185 | -0.2419 | -0.74436 | -0.90712 | -0.56238 | -0.39485 | -0.75599 | -0.40133 | -0.30927 | -0.63767 | -0.64375 |
| P62137 | P62137 | PP1A_MOI Ppp1ca | Serine/threonine-protein phosphatase PP1-alpha catalytic subunit | | | | 0.319425 | 1.40047 | 0.862617 | 0.43747 | 0.279369 | 1.161862 | 0.934359 | -0.28708 | -2.66985 | -0.71778 | -0.5152 | 1.226622 | 1.326183 | 0.802797 | 0.808008 | -0.39661 | -0.63731 | 0.761794 | 1.232274 | 0.984987 | 1.199977 |

| | | | | | | | | | | | | | | | | | | | | | | | | | | | | |
|---|---|---|---|---|---|---|---|---|---|---|---|---|---|---|---|---|---|---|---|---|---|---|---|---|---|---|---|---|
| P62141 | P62137;P6 | PP1B_MO | Ppp1cb | Serine/threonine-protein phosphatase PP1-beta catalytic subunit | | | | 0.688125 | 0.162755 | 1.853054 | 0.311875 | 0.140809 | 0.843685 | 0.699186 | -0.41545 | -1.88627 | 0.544618 | 0.39368 | 2.109705 | 2.134721 | 1.486774 | 1.8804 | 0.261465 | 0.351324 | 1.814976 | 2.147832 | 2.000172 | 2.197897 |
| P63087 | P63087 | PP1G_MO | Ppp1cc | Serine/threonine-protein phosphatase PP1-gamma catalytic subunit | | | | 0.040442 | -0.0753 | -0.12625 | 0.058356 | 0.021703 | 0.179235 | 0.572303 | -0.40531 | -1.52065 | -0.80018 | -0.36225 | 0.8791 | 1.117173 | 0.867417 | 1.353945 | 0.04883 | -1.06068 | 0.954259 | 0.998609 | 1.624927 | 1.407064 |
| Q9DBR7 | Q9DBR7 | MYPT1_M | Ppp1r12a | Protein phosphatase 1 regulatory subunit 12A | | | | 0.026885 | 0.023199 | 0.085045 | 0.061129 | -0.04602 | -0.1873 | 0.045564 | 0.036007 | 0.141558 | 2.353013 | 2.473852 | 3.13712 | 3.607822 | 3.281221 | 3.403451 | 2.656246 | 2.124219 | 3.347908 | 3.489078 | 3.553242 | 3.059417 |
| Q8BG95 | Q8BG95 | MYPT2_M | Ppp1r12b | Protein phosphatase 1 regulatory subunit 12B | | | | NaN | 0 | NaN | 0.256838 | -0.40418 | -0.70559 | 0.406378 | -0.31068 | -1.08215 | -3.69457 | -3.0166 | -2.51298 | -2.59558 | -3.49926 | -2.94948 | -3.08082 | -2.97113 | -1.57876 | -2.72144 | -2.99651 | -2.83086 |
| Q8BWT1 | Q8BWT1 | THIM_MO | Acaa2 | 3-ketoacyl-CoA thiolase, mitochondrial | + | | | 1.930538 | 0.258126 | 9.149983 | 0.331092 | -0.1979 | -0.89189 | 0.006901 | 0.018078 | 0.022298 | 1.678742 | 1.733963 | 1.787075 | 2.060358 | 2.581019 | 2.434424 | 1.454013 | 1.442441 | 1.946792 | 2.296447 | 1.682203 | 3.297084 |
| Q62415 | Q62415 | ASPP1_M | Ppp1r13b | Apoptosis-stimulating of p53 protein 1 | | | | 0.344771 | -1.46604 | -0.92626 | 0.451127 | -0.45224 | -1.19716 | 0.561386 | -0.48609 | -1.49062 | -3.26109 | -0.10531 | 0.59948 | -0.06073 | -0.20884 | 0.410203 | -0.09329 | -0.34104 | 0.905278 | 0.537951 | 0.689432 | 0.484117 |
| O08539 | O08539 | BIN1_MO | Bin1 | Myc box-dependent-interacting protein 1 | + | | + | 1.675957 | 0.225435 | 6.776785 | 0.110836 | -0.09023 | -0.32695 | 1.316232 | -0.32338 | -4.38462 | 1.085908 | 1.029997 | 1.962335 | 1.750039 | 1.861878 | 1.722018 | 0.850549 | 0.814488 | 1.691666 | 2.201171 | 2.138762 | 2.09189 |
| Q8CGC7 | Q8CGC7 | SYEP_MO | Eprs1 | Bifunctional glutamate/proline--tRNA ligase | + | | | 2.09509 | 0.223628 | 11.0896 | 0.360184 | 0.194101 | 0.965069 | 0.537702 | 0.35364 | 1.426129 | 0.090086 | 0.061591 | 0.700905 | 0.516855 | 0.861322 | 0.419462 | -0.13352 | -0.16206 | 0.23594 | 0.593618 | 0.174148 | 0.399357 |
| Q9JIG4 | Q9JIG4 | PPR3F_M | Ppp1r3f | Protein phosphatase 1 regulatory subunit 3F | | | | 0.051376 | 0.076556 | 0.158774 | 0.505764 | 0.338356 | 1.340509 | 0.004951 | 0.005512 | 0.016033 | -0.37256 | 0.338199 | -0.32983 | 0.119004 | -1.14374 | -0.50046 | -0.41961 | 0.232135 | -0.32823 | -0.5593 | -0.70629 | -0.94894 |
| Q3UM45 | Q3UM45 | PP1R7_M | Ppp1r7 | Protein phosphatase 1 regulatory subunit 7 | | | | 0.339002 | -1.37507 | -0.91176 | 0.078941 | 0.084438 | 0.238366 | 0.244853 | -0.22633 | -0.6754 | -3.60187 | -0.71065 | 0.310546 | -0.32884 | -0.73141 | -0.22521 | -0.35139 | -1.21098 | 0.058982 | -0.24616 | -0.03237 | -0.4716 |
| Q7TN74 | Q7TN74 | Q7TN74_ | Ppp1r9a | Neurabin I | | | | 0.748928 | 0.971552 | 2.039233 | 0.040192 | 0.060533 | 0.125497 | 0.193979 | 0.219815 | 0.546114 | -2.97499 | -2.02248 | -1.97954 | -1.45659 | -1.30453 | -1.23486 | -3.48323 | -3.45734 | -1.37327 | -2.18392 | -1.08851 | -1.89051 |
| Q6R891 | Q6R891;Q | NEB2_MO | Ppp1r9b | Neurabin-2 | | | | 0.39009 | -1.082 | -1.04071 | 0.445703 | 0.317819 | 1.183121 | 0.257933 | 0.373239 | 0.708341 | -3.99698 | -2.69836 | -1.11462 | -0.85196 | 0.054223 | -0.14728 | -1.45369 | -3.07765 | -1.53544 | -1.06678 | 0.097427 | -0.93697 |
| P63330 | P63330;P | PP2AA_M | Ppp2ca | Serine/threonine-protein phosphatase 2A catalytic subunit alpha isoform | | | | 0.913536 | 0.75869 | 2.593694 | 0.509449 | 0.540487 | 1.350315 | 0.200202 | -0.05877 | -0.56206 | 0.997928 | 1.13229 | 2.464241 | 2.618295 | 1.951586 | 1.797382 | 0.021726 | 0.591114 | 1.607995 | 2.393567 | 1.862619 | 2.003895 |
| P62715 | P62715 | PP2AB_M | Ppp2cb | Serine/threonine-protein phosphatase 2A catalytic subunit beta isoform | | + | | 0.540446 | 1.795389 | 1.433556 | 1.540121 | 0.639127 | 5.761132 | 0.058018 | 0.085338 | 0.178251 | -0.94852 | -2.40996 | -0.53 | -0.50639 | -1.39948 | -1.02968 | -2.4575 | -4.49176 | -1.26763 | -1.04701 | -0.85831 | -1.74152 |
| Q76MZ3 | Q76MZ3 | 2AAA_MO | Ppp2r1a | Serine/threonine-protein phosphatase 2A 65 kDa regulatory subunit A alpha isoform | + | | | 1.031367 | 0.824029 | 3.045221 | 0.747543 | 0.319636 | 2.034894 | 0.047865 | 0.032612 | 0.148392 | 3.012794 | 3.131323 | 4.187648 | 4.432643 | 3.852039 | 3.580009 | 2.512057 | 1.984002 | 3.892185 | 4.088834 | 3.51079 | 3.856034 |
| P54775 | P54775 | PRS6B_M | Psmc4 | 26S proteasome regulatory subunit 6B | + | + | | 1.30174 | 0.21409 | 4.306451 | 1.010576 | 0.441256 | 2.961782 | 0.234902 | 0.4796 | 0.650274 | -0.30183 | -0.39803 | 0.694396 | 0.459098 | 1.063365 | 0.051923 | -0.57657 | -0.55146 | 0.226895 | 0.044087 | -0.4588 | 0.614892 |
| Q6ZWR4 | Q6ZWR4 | 2ABB_MO | Ppp2r2b | Serine/threonine-protein phosphatase 2A 55 kDa regulatory subunit B beta isoform | | | | 0.522522 | 0.907198 | 1.38525 | 0.442372 | 0.142626 | 1.174511 | 0.833901 | -1.24775 | -2.31547 | -1.8795 | -3.18925 | -1.34827 | -1.14383 | -1.96273 | -2.96939 | -3.43591 | -3.44724 | -1.45423 | -1.32312 | -1.02583 | -1.41078 |
| Q8BG02 | Q8BG02 | 2ABG_MO | Ppp2r2c | Serine/threonine-protein phosphatase 2A 55 kDa regulatory subunit B gamma isoform | | | | 0.710531 | 1.14542 | 1.920631 | 0.336337 | 0.428363 | 0.905065 | 0.112778 | 0.146224 | 0.33224 | -0.96336 | -1.11871 | -0.88537 | -0.42617 | -0.45682 | -1.11242 | -1.59516 | -2.77775 | -1.49801 | -0.67026 | -1.22452 | -0.63716 |
| Q925E7 | Q925E7 | 2ABD_MO | Ppp2r2d | Serine/threonine-protein phosphatase 2A 55 kDa regulatory subunit B delta isoform | | + | | 0.226508 | -1.08009 | -0.62903 | 1.29149 | -0.34461 | -4.25189 | 0.023237 | 0.031151 | 0.073778 | -3.84286 | -0.41814 | -0.97959 | -0.81886 | -0.49616 | -0.83515 | -0.92325 | -1.17758 | -0.54409 | -0.56514 | -1.08352 | -0.31009 |
| Q6PD03 | Q6PD03 | 2A5A_MO | Ppp2r5a | Serine/threonine-protein phosphatase 2A 56 kDa regulatory subunit alpha isoform | | | | 0.097788 | -0.11637 | -0.29111 | 0.006561 | -0.00471 | -0.02121 | 0.087219 | -0.03593 | -0.26168 | -2.60732 | -1.85155 | -0.53124 | -0.28522 | -0.63666 | -0.75511 | -2.24347 | -1.98266 | -0.58826 | -0.21879 | -0.5361 | -0.78382 |
| P23819 | P23819;P | GRIA2_M | Gria2 | Glutamate receptor 2 | + | | | 1.3065 | 0.188803 | 4.33199 | 0.608156 | -0.47589 | -1.62075 | 0.178573 | -0.07874 | -0.50642 | -0.70564 | -0.70991 | 0.615678 | 0.029059 | 2.25338 | 2.36329 | -0.85305 | -0.94011 | 0.784743 | 0.811767 | 2.532535 | 2.241624 |
| Q60996 | Q7TNL5;Q | 2A5G_MO | Ppp2r5c | Serine/threonine-protein phosphatase 2A 56 kDa regulatory subunit gamma isoform | | | + | 0.451903 | 0.263273 | 1.199176 | 0.06553 | 0.070274 | 0.200042 | 1.136976 | 0.303274 | 3.496713 | -0.51961 | -0.76568 | 0.456143 | 0.534131 | 0.554837 | 0.412758 | -0.72409 | -1.08775 | 0.075737 | 0.773988 | 0.130767 | 0.23028 |
| Q7TNL5 | Q7TNL5 | Q7TNL5_ | Ppp2r5d | Serine/threonine-protein phosphatase 2A 56 kDa regulatory subunit | + | | | 1.173426 | 0.270839 | 3.664093 | 0.027839 | -0.02397 | -0.08798 | 0.809099 | 0.681975 | 2.232764 | -0.78078 | -0.71804 | -0.05037 | -0.38259 | 0.483547 | -0.07825 | -1.08718 | -0.95332 | -0.40847 | 0.023455 | -0.35936 | -0.59929 |
| Q61151 | Q61151 | 2A5E_MO | Ppp2r5e | Serine/threonine-protein phosphatase 2A 56 kDa regulatory subunit epsilon isoform | + | | | 1.128187 | 0.226743 | 3.457278 | 0.105763 | -0.07492 | -0.31307 | 0.564921 | 0.441497 | 1.500323 | -0.23106 | -0.26935 | 0.250026 | -0.18763 | 0.76239 | 0.275611 | -0.41422 | -0.53968 | 0.202971 | 0.009264 | 0.242898 | -0.08789 |
| P63328 | P48453;P | PP2BA_M | Ppp3ca | Protein phosphatase 3 catalytic subunit alpha | | | | 0.998486 | 0.910668 | 2.914037 | 0.370528 | -0.53076 | -0.99117 | 0.324849 | -0.44252 | -0.87622 | 2.940721 | 3.254839 | 5.759691 | 4.84393 | 4.836417 | 5.822675 | 2.457289 | 1.916935 | 5.554915 | 6.110233 | 5.663074 | 5.881057 |
| Q61102 | Q61102 | ABCB7_M | Abcb7 | Iron-sulfur clusters transporter ABCB7, mitochondrial | + | | | 1.319205 | 0.120523 | 4.4008 | 0.958208 | 0.577356 | 2.758929 | 0.110462 | 0.078661 | 0.325927 | -0.14227 | -0.13205 | -0.01401 | 0.360703 | 0.087623 | -0.38341 | -0.28459 | -0.23078 | -0.31079 | -0.49723 | -0.27929 | -0.17382 |
| Q63810 | Q63810 | CANB1_M | Ppp3r1 | Calcineurin subunit B type 1 | | | | 0.255333 | 0.194992 | 0.7018 | 0.205921 | -0.45953 | -0.57668 | 0.200015 | -0.27196 | -0.56158 | 1.781355 | 2.247424 | 4.212401 | 3.128996 | 3.874761 | 4.843306 | 1.668093 | 1.970703 | 3.545807 | 4.714655 | 4.631976 | 4.630008 |
| Q8C0Y0 | Q8C0Y0 | PP4R4_M | Ppp4r4 | Serine/threonine-protein phosphatase 4 regulatory subunit 4 | | | + | 0.104512 | 0.250724 | 0.309638 | 0.013644 | 0.022534 | 0.04376 | 1.569537 | 0.156212 | 5.968314 | -1.9974 | -2.87363 | -2.5774 | -1.63984 | -1.64313 | -1.69222 | -2.00527 | -3.36721 | -2.34427 | -1.91804 | -1.81479 | -1.83298 |
| Q60676 | Q60676 | PPP5_MO | Ppp5c | Serine/threonine-protein phosphatase 5 | + | | | 1.0367 | -0.28842 | -3.0669 | 0.192219 | -0.18815 | -0.54159 | 0.274131 | 0.135386 | 0.749038 | -1.47516 | -1.29286 | -0.66414 | -1.11124 | -0.87845 | -0.53692 | -1.07245 | -1.11873 | -0.96545 | -0.43363 | -0.78384 | -0.9023 |
| Q9CQR6 | Q9CQR6 | PPP6_MO | Ppp6c | Serine/threonine-protein phosphatase 6 catalytic subunit | | | + | 0.113785 | -0.22115 | -0.33498 | 0.155434 | 0.042287 | 0.446148 | 1.05438 | -0.36962 | -3.13959 | -1.16565 | -0.05057 | 0.777041 | 0.697104 | 0.086588 | 0.179248 | -0.03338 | -0.74052 | 0.608843 | 0.780728 | 0.39431 | 0.61077 |
| Q8R3Q2 | Q8R3Q2 | PP6R2_M | Ppp6r2 | Serine/threonine-protein phosphatase 6 regulatory subunit 2 | | | | 0.571516 | 1.523972 | 1.51848 | 0.387419 | 0.499546 | 1.033934 | 0.216228 | 0.146786 | 0.602936 | -0.65474 | -1.01162 | 0.352457 | 0.691203 | 0.091191 | -0.32813 | -1.36952 | -3.34478 | -0.43021 | 0.474774 | -0.389 | -0.14152 |
| Q922D4 | Q922D4 | PP6R3_M | Ppp6r3 | Serine/threonine-protein phosphatase 6 regulatory subunit 3 | | | | 0.226647 | 0.664849 | 0.629381 | 0.066756 | -0.0808 | -0.20357 | 0.394454 | 0.443932 | 1.051797 | -1.56437 | -1.9981 | -1.56052 | -0.80894 | -1.25822 | -1.94738 | -3.47993 | -1.41223 | -1.23165 | -0.97622 | -2.29046 | -1.803 |
| O88531 | O88531 | PPT1_MO | Ppt1 | Palmitoyl-protein thioesterase 1 | | + | | 0.664253 | 1.292069 | 1.782303 | 1.037846 | 0.715391 | 3.071572 | 0.774421 | 1.429121 | 2.120039 | -2.95374 | -1.51046 | -2.09622 | -2.0094 | -2.03488 | -1.68685 | -3.5933 | -3.45504 | -2.99703 | -2.53937 | -3.94124 | -2.63873 |
| Q6NVE9 | Q6NVE9 | PPTC7_M | Pptc7 | Protein phosphatase PTC7 homolog | | | | 0.960489 | 0.797653 | 2.767553 | 0.370223 | 0.488128 | 0.990405 | 0.31867 | 0.343035 | 0.860723 | -1.01136 | -1.00836 | -2.02201 | -1.63873 | -1.82285 | -1.26015 | -2.09572 | -1.5193 | -2.77257 | -1.86443 | -1.60226 | -2.16681 |
| Q62188 | Q62188 | DPYL3_M | Dpysl3 | Dihydropyrimidinase-related protein 3 | + | | | 1.671627 | -0.13375 | -6.742 | 0.07915 | 0.092408 | 0.238958 | 0.127277 | 0.076098 | 0.371433 | 1.337644 | 1.320251 | 2.314102 | 2.053007 | 2.140636 | 2.516023 | 1.480532 | 1.44487 | 1.727135 | 2.455158 | 2.334365 | 2.170098 |
| Q571I4 | Q571I4 | PRAG1_M | Prag1 | Inactive tyrosine-protein kinase PRAG1 | + | + | | 1.109381 | 2.161131 | 3.374075 | 1.340454 | -0.87105 | -4.51798 | 0.772459 | -0.74415 | -2.11376 | -1.35762 | -0.30071 | -0.3394 | 0.044838 | -1.04346 | -0.57577 | -3.35222 | -2.62838 | 0.739926 | 0.707609 | -0.32863 | 0.197702 |
| Q4VAD2 | Q4VAD2 | Q4VAD2_ | Pramel27 | PRAME like 27 | | | | 0.319261 | 1.476861 | 0.862206 | NaN | 0 | NaN | NaN | 0 | NaN | -0.50415 | -3.84547 | -4.25206 | -3.58014 | -3.5469 | -3.05156 | -4.02966 | -3.27368 | -3.34381 | -3.90441 | -2.74482 | -3.30144 |
| Q60636 | Q60636 | PRDM1_M | Prdm1 | PR domain zinc finger protein 1 | | | | NaN | 0 | NaN | 0.44405 | 0.455864 | 1.178848 | 0.592426 | 2.298064 | 1.576551 | -3.4659 | -3.07104 | -1.54971 | -1.31226 | -0.7595 | -0.02727 | -3.00705 | -3.11933 | -2.25487 | -1.51882 | -4.10238 | -1.28053 |
| P35700 | P35700 | PRDX1_M | Prdx1 | Peroxiredoxin-1 | | | | 0.790458 | 0.206495 | 2.171756 | 0.857899 | -0.43301 | -2.39722 | 0.010852 | -0.00208 | -0.03491 | 5.675255 | 5.509883 | 4.252669 | 4.426496 | 4.844368 | 4.920304 | 5.339132 | 5.433017 | 4.930935 | 4.614246 | 4.838408 | 4.930429 |
| Q61171 | Q61171 | PRDX2_M | Prdx2 | Peroxiredoxin-2 | | + | | 0.070133 | -0.00993 | -0.21328 | 2.037259 | -0.78582 | -10.3663 | 0.400655 | -0.23821 | -1.06757 | 3.832625 | 3.851307 | 3.57387 | 3.576112 | 3.948055 | 4.310493 | 3.806268 | 3.897533 | 4.285011 | 4.436604 | 4.497656 | 4.237303 |
| Q4KMM3 | Q4KMM3 | OXR1_MO | Oxr1 | Oxidation resistance protein 1 | + | | | 1.400239 | -0.15355 | -4.86239 | 0.401978 | 0.171949 | 1.070938 | 0.131796 | 0.18664 | 0.383541 | -0.75135 | -0.74059 | 0.265906 | 0.038876 | 1.15037 | 0.177581 | -0.62354 | -0.56131 | -0.13311 | 0.093992 | 0.492282 | 0.462389 |
| O08807 | O08807 | PRDX4_M | Prdx4 | Peroxiredoxin-4 | | + | | 0.313841 | 0.243466 | 0.848612 | 1.241329 | -0.57824 | -3.99325 | 0.10112 | 0.050366 | 0.300308 | 3.946434 | 3.690114 | 3.210704 | 3.429363 | 3.652667 | 3.704373 | 3.831491 | 3.318125 | 3.803322 | 3.993219 | 3.462443 | 3.793864 |
| P99029 | P99029 | PRDX5_M | Prdx5 | Peroxiredoxin-5, mitochondrial | | + | | 0.986477 | 0.608993 | 2.867159 | 1.435774 | -1.99077 | -5.07788 | 0.191755 | -0.14297 | -0.5404 | 5.548983 | 5.211975 | 5.097408 | 4.95662 | 6.501871 | 6.717069 | 4.900797 | 4.642175 | 6.632109 | 7.403461 | 6.510749 | 6.994128 |
| Q8BXR1 | Q8BXR1 | S7A14_M | Slc7a14 | Probable cationic amino acid transporter | + | + | | 1.320453 | -0.16393 | -4.40761 | 1.123952 | 0.763291 | 3.438399 | 0.877356 | 0.481847 | 2.464803 | 0.104534 | 0.031118 | 0.723155 | 0.548362 | 1.541884 | 1.758841 | 0.225771 | 0.237743 | -0.33159 | 0.07653 | 1.331148 | 1.005884 |
| Q9QUR6 | Q9QUR6 | PPCE_MO | Prep | Prolyl endopeptidase | | | | 0.545321 | -0.81799 | -1.44678 | 0.231844 | -0.13541 | -0.64254 | 0.18314 | -0.43417 | -0.51822 | -3.69741 | -3.32292 | -2.45836 | -2.85568 | -3.25048 | -2.35125 | -2.1587 | -3.22565 | -2.59195 | -2.45126 | -1.65975 | -3.07364 |
| Q8C167 | Q8C167 | PPCEL_M | Prepl | Prolyl endopeptidase-like | | | | 0.706789 | -1.37589 | -1.90927 | 0.464605 | -0.73939 | -1.2322 | 0.372936 | -0.49046 | -0.99726 | -1.54011 | -2.95528 | -0.74639 | -1.9347 | -1.59151 | -0.70368 | -1.00836 | -0.73526 | -0.51718 | -0.68513 | -0.44543 | -0.86883 |
| Q69ZK0 | Q69ZK0 | PREX1_M | Prex1 | Phosphatidylinositol 3,4,5-trisphosphate-dependent Rac exchanger 1 protein | | | | 0.097904 | -0.39784 | -0.29143 | 0.22635 | 0.194728 | 0.628629 | 0.237608 | -0.11501 | -0.65711 | -3.86342 | -1.69104 | -0.90077 | -0.54967 | -1.40985 | -1.75031 | -3.2063 | -1.55249 | -1.17517 | -0.66472 | -1.42437 | -1.50577 |
| Q3U5C7 | Q3U5C7 | PRIC1_M | Prickle1 | Prickle-like protein 1 | | | + | 0.137013 | 0.363979 | 0.39746 | 0.947695 | 0.271526 | 2.719413 | 2.170659 | -1.08846 | -12.1094 | -2.01419 | -3.76333 | -1.18743 | -1.24962 | -2.04682 | -2.10844 | -2.98117 | -3.5243 | -1.58493 | -1.39517 | -0.90473 | -1.07361 |
| Q80Y24 | Q80Y24 | PRIC2_M | Prickle2 | Prickle-like protein 2 | | + | | 0.013535 | -0.03994 | -0.04342 | 1.383103 | 0.360233 | 4.761398 | 0.125218 | -0.1848 | -0.3659 | -2.17683 | -0.83367 | 1.056772 | 1.135715 | 0.808208 | 0.827015 | -0.83648 | -2.09413 | 0.671466 | 0.800555 | 0.497449 | 1.507366 |
| P28661 | P28661 | SEPT4_M | Septin4 | Septin-4 | + | | | 1.469918 | -0.23197 | -5.29294 | 0.804146 | -0.2537 | -2.21646 | 0.38702 | -0.09796 | -1.03292 | 0.748101 | 0.681194 | 0.242301 | 0.018139 | 0.031968 | 0.035345 | 0.918303 | 0.974926 | 0.360702 | 0.407131 | 0.036794 | 0.22644 |
| Q8R574 | Q9D0M1;Q | KPRB_MO | Prpsap2 | Phosphoribosyl pyrophosphate synthase-associated protein 2 | + | | | 1.478925 | -0.24214 | -5.35101 | 0.060413 | 0.086265 | 0.185223 | 0.270155 | 0.231413 | 0.739057 | -1.34255 | -1.2991 | -0.59345 | -0.2453 | -0.86559 | -0.23939 | -1.11838 | -1.03899 | -0.93762 | -0.07366 | -0.78044 | -0.78737 |
| Q8BH95 | Q8BH95 | ECHM_M | Echs1 | Enoyl-CoA hydratase, mitochondrial | + | | | 3.215073 | -0.2641 | -40.4891 | 0.239685 | -0.30467 | -0.66236 | 0.07307 | -0.10991 | -0.22167 | 2.019655 | 2.015834 | 1.653301 | 0.919526 | 1.490084 | 2.403085 | 2.288082 | 2.275609 | 1.868534 | 1.313639 | 2.24994 | 1.863041 |
| Q9QUM9 | Q9QUM9 | PSA6_MO | Psma6 | Proteasome subunit alpha type-6 | + | | | 1.66516 | -0.26799 | -6.69034 | 0.505683 | -0.59091 | -1.34029 | 0.188045 | -0.27102 | -0.53086 | 0.769473 | 0.789369 | 0.854071 | 0.015154 | 0.845833 | 1.862328 | 1.008608 | 1.08621 | 0.889765 | 1.161276 | 1.673206 | 1.576985 |
| Q9DBC7 | Q9DBC7 | KAP0_MO | Prkar1a | cAMP-dependent protein kinase type I-alpha regulatory subunit | + | | | 1.179594 | 0.992601 | 3.693042 | 0.569255 | -0.22746 | -1.51225 | 0.586837 | -0.82761 | -1.56095 | 1.877015 | 1.693223 | 2.316008 | 2.557294 | 1.115063 | 2.050159 | 1.045095 | 0.539939 | 2.753938 | 2.574283 | 2.160209 | 2.660233 |
| P12849 | P12849 | KAP1_MO | Prkar1b | cAMP-dependent protein kinase type I-beta regulatory subunit | + | | | 1.281349 | 1.099437 | 4.198492 | 0.84694 | -0.1014 | -2.35967 | 0.39547 | -0.5929 | -1.05438 | 1.355148 | 0.974504 | 2.3403 | 2.426054 | 0.781349 | 1.632232 | 0.245252 | -0.11447 | 2.487408 | 2.481739 | 1.431989 | 2.167392 |
| P12367 | P12367 | KAP2_MO | Prkar2a | cAMP-dependent protein kinase type II-alpha regulatory subunit | + | | | 1.204753 | 0.524622 | 3.81306 | 0.257665 | 0.205844 | 0.707666 | 0.080853 | -0.05522 | -0.24377 | 3.178287 | 2.906672 | 3.17404 | 3.717441 | 2.973546 | 2.704638 | 2.495811 | 2.539905 | 3.343766 | 3.136027 | 2.712022 | 3.076595 |
| P31324 | P31324 | KAP3_MO | Prkar2b | cAMP-dependent protein kinase type II-beta regulatory subunit | + | | | 1.289377 | 0.851684 | 4.240716 | 0.742314 | 0.450873 | 2.018544 | 0.021859 | -0.02113 | -0.0695 | 3.147529 | 3.009219 | 4.254064 | 4.606615 | 3.383229 | 2.9528 | 2.038136 | 2.415243 | 3.842284 | 4.116649 | 2.974361 | 3.403934 |
| Q9WTX2 | Q9WTX2 | PRKRA_M | Prkra | Interferon-inducible double-stranded RNA-dependent protein kinase activator A | | | | 0.870883 | 0.559393 | 2.442187 | 0.791213 | 0.389919 | 2.174211 | 0.302636 | 1.011154 | 0.820523 | 0.481217 | 0.186123 | -0.66045 | -0.41369 | -0.9679 | -1.57294 | -0.05052 | -0.40093 | -1.05714 | -0.79684 | -3.47619 | -1.08695 |
| Q9JIF0 | Q9JIF0 | ANM1_MO | Prmt1 | Protein arginine N-methyltransferase 1 | | | | 0.615292 | -0.16831 | -1.64095 | 0.950948 | -0.17847 | -2.7316 | 0.025001 | -0.01487 | -0.07924 | -0.23806 | -0.04415 | 0.374032 | 0.350377 | 0.221424 | 0.552125 | -0.00626 | 0.060664 | 0.476422 | 0.604937 | 0.49044 | 0.312851 |
| Q922H1 | Q922H1 | ANM3_MO | Prmt3 | Protein arginine N-methyltransferase 3 | | | | 0.052355 | -0.33201 | -0.16166 | 0.210331 | 0.245469 | 0.587924 | 0.071393 | -0.38391 | -0.21688 | -3.4189 | -0.54002 | -0.5045 | 0.082831 | -0.46465 | -3.7603 | -0.18252 | -3.11239 | -0.15952 | -0.75309 | -1.08198 | -2.37515 |
| Q8CIG8 | Q8CIG8 | ANM5_MO | Prmt5 | Protein arginine N-methyltransferase 5 | | | + | 0.310682 | -0.28225 | -0.84069 | 0.316871 | -0.14487 | -0.85621 | 1.399597 | -0.42256 | -4.85857 | -0.49517 | -0.87784 | -0.26393 | 0.073185 | 0.026971 | 0.185859 | -0.68013 | -0.12837 | 0.034733 | 0.06427 | 0.493583 | 0.564376 |
| Q6PAK3 | Q6PAK3 | ANM8_MO | Prmt8 | Protein arginine N-methyltransferase 8 | | + | | 0.264226 | 0.523845 | 0.724162 | 1.427174 | -0.34444 | -5.02497 | 0.003987 | 0.002529 | 0.012925 | -1.7976 | -0.95289 | -0.07763 | -0.11675 | 0.120713 | -0.24332 | -2.48637 | -1.31181 | 0.181559 | 0.31295 | 0.007934 | -0.1356 |
| P04925 | P04925 | PRIO_MO | Prnp | Major prion protein | + | | | 1.180075 | -0.54518 | -3.69531 | 0.838976 | 0.479021 | 2.332608 | 0.262706 | 0.272722 | 0.720343 | -0.6323 | -0.904 | -0.84811 | -0.52923 | -0.28371 | 0.162921 | -0.16543 | -0.28051 | -1.03827 | -1.29711 | -0.02739 | -0.63884 |
| Q9WU79 | Q9WU79 | PROD_MO | Prodh | Proline dehydrogenase 1, mitochondrial | + | | | 1.146283 | 0.815409 | 3.538857 | 0.040281 | 0.027642 | 0.125765 | 0.893707 | -0.51271 | -2.52252 | 2.980391 | 2.591774 | 1.815652 | 2.226044 | 1.962272 | 1.858357 | 2.09451 | 1.846836 | 2.071976 | 1.914435 | 2.226527 | 2.619531 |
| Q99KP6 | Q99KP6 | PRP19_M | Prpf19 | Pre-mRNA-processing factor 19 | + | | | 1.113962 | 1.519764 | 3.394195 | 0.06353 | -0.03801 | -0.19426 | 0.164364 | 0.188939 | 0.469513 | 1.207131 | 1.660839 | 1.525816 | 1.782753 | 1.701916 | 1.089129 | 0.300253 | -0.47181 | 1.544715 | 1.839874 | 0.945698 | 1.467468 |
| Q8CCF0 | Q8CCF0 | PRP31_M | Prpf31 | U4/U6 small nuclear ribonucleoprotein Prp31 | | | | 0.501483 | 0.58906 | 1.329142 | 0.296611 | 0.20225 | 0.805419 | 0.788134 | -0.21577 | -2.16422 | -1.56811 | -0.68176 | -0.05355 | 0.043528 | 0.048556 | -0.14929 | -1.71067 | -1.71732 | -0.45364 | 0.039117 | 0.177817 | 0.152989 |
| Q80SY5 | Q80SY5 | PR38B_M | Prpf38b | Pre-mRNA-splicing factor 38B | | | | 0.132486 | 0.474507 | 0.385386 | 0.269539 | -0.28347 | -0.73751 | 0.029463 | 0.110368 | 0.09296 | -3.566 | -1.14794 | -1.19017 | -0.54608 | -1.17559 | -3.01308 | -3.06431 | -2.59864 | -0.37484 | -0.79447 | -2.95671 | -1.4527 |
| Q9R1C7 | Q9R1C7 | PR40A_M | Prpf40a | Pre-mRNA-processing factor 40 homolog A | | | | 0.056912 | -0.14367 | -0.17502 | 0.239431 | -0.23944 | -0.66172 | 0.192197 | 0.221695 | 0.541537 | -2.18526 | -0.54473 | -1.98195 | -1.31674 | -0.71493 | -0.97093 | -1.1896 | -1.25305 | -1.5524 | -1.26742 | -1.45348 | -0.67577 |
| Q99PV0 | Q99PV0 | PRP8_MO | Prpf8 | Pre-mRNA-processing-splicing factor 8 | | | | 0.0129 | -0.01003 | -0.04141 | 0.282266 | -0.22209 | -0.76945 | 0.611387 | 0.631253 | 1.629889 | -1.65918 | -1.26816 | -1.46337 | -2.01924 | -0.51326 | -1.20974 | -1.59667 | -1.3106 | -1.43567 | -1.60076 | -1.32325 | -1.66225 |
| P15331 | P15331 | PERI_MO | Prph | Peripherin | | | | 0.880723 | -1.60787 | -2.47662 | NaN | 0 | NaN | NaN | 0 | NaN | -1.36745 | -1.85633 | -3.74216 | -4.07359 | -3.33144 | -3.45061 | 0.597425 | -0.60546 | -2.54758 | -3.1419 | -2.50738 | -3.52469 |
| Q9D7G0 | Q9D7G0;Q | PRPS1_M | Prps1 | Ribose-phosphate pyrophosphokinase 1 | | | | 0.043561 | -0.03416 | -0.13559 | 0.602607 | -0.32483 | -1.60511 | 0.567631 | -0.23397 | -1.50778 | 0.537076 | 0.540519 | 1.060956 | 0.656927 | 1.029422 | 1.236567 | 0.824867 | 0.321041 | 1.195755 | 1.171777 | 1.482511 | 1.251412 |
| Q9D0M1 | Q9D0M1 | KPRA_MO | Prpsap1 | Phosphoribosyl pyrophosphate synthase-associated protein 1 | | | + | 0.062842 | 0.101347 | 0.192271 | 0.004002 | -0.00726 | -0.01297 | 1.031199 | -0.20911 | -3.04454 | -2.07979 | -2.95383 | -3.14339 | -2.7004 | -2.21986 | -2.25865 | -2.32345 | -2.91287 | -3.42835 | -2.40093 | -2.09604 | -1.96426 |
| P63001 | P63001;P | RAC1_MO | Rac1 | Ras-related C3 botulinum toxin substrate 1 | + | | | 1.887678 | -0.26958 | -8.70138 | 0.166356 | -0.27936 | -0.47471 | 0.249972 | -0.25997 | -0.6883 | 2.36741 | 2.425387 | 3.320502 | 2.143871 | 2.568308 | 3.06752 | 2.676908 | 2.655045 | 3.025887 | 2.997208 | 3.361354 | 2.794416 |
| Q3V0I2 | Q3V0I2 | PRR7_MO | Prr7 | Proline-rich protein 7 | | | | 0.605242 | -0.16687 | -1.61253 | 0.099823 | 0.21031 | 0.296729 | 0.345449 | -0.59391 | -0.92797 | -3.26013 | -3.27542 | -2.33804 | -2.85502 | -1.16487 | -2.42244 | -3.2041 | -2.9977 | -2.14689 | -3.46678 | -1.08041 | -1.31908 |
| Q7TSC1 | Q7TSC1 | PRC2A_M | Prrc2a | Protein PRRC2A | | | | 0.413982 | 1.810501 | 1.101569 | 0.096493 | 0.066534 | 0.287519 | 0.8475 | 0.403726 | 2.361578 | -3.25426 | 0.032678 | -0.31991 | -0.25978 | -0.64925 | -0.47769 | -3.40363 | -3.43895 | -0.58582 | -0.12693 | -1.11507 | -0.81932 |
| Q7TPM1 | Q7TPM1 | PRC2B_M | Prrc2b | Protein PRRC2B | | | | 0.285397 | 0.425372 | 0.777304 | 0.05517 | -0.07318 | -0.16992 | 0.267547 | -0.13187 | -0.73251 | -2.3463 | -3.00111 | -1.13327 | -0.78242 | -0.7928 | -1.15268 | -2.66059 | -3.53758 | -1.27798 | -0.49135 | -0.83545 | -0.84629 |
| Q3TLH4 | Q3TLH4 | PRC2C_M | Prrc2c | Protein PRRC2C | | | | 0.550855 | 0.299148 | 1.461833 | 0.068188 | -0.03863 | -0.20769 | 0.902033 | -0.19575 | -2.55225 | -0.49777 | -0.80584 | -0.25485 | 0.112901 | -0.2469 | -0.09403 | -0.81623 | -1.08568 | -0.0604 | -0.00428 | 0.018916 | 0.031661 |
| O35449 | O35449 | PRRT1_M | Prrt1 | Proline-rich transmembrane protein 1 | | | + | 0.366345 | -0.61719 | -0.98061 | 0.483258 | -0.28117 | -1.28102 | 1.749827 | -0.47675 | -7.39704 | -1.69529 | -1.57764 | 0.101585 | -0.14727 | 1.25943 | 1.380195 | -1.64591 | -0.39264 | 0.439138 | 0.077515 | 1.819101 | 1.774026 |
| E9PUL5 | E9PUL5 | PRRT2_M | Prrt2 | Proline-rich transmembrane protein 2 | | | | 0.029487 | -0.02028 | -0.09303 | 0.341182 | 1.152062 | 0.917239 | 0.326735 | 0.533936 | 0.880956 | -1.21541 | -1.17205 | -0.69145 | -1.84644 | -0.28799 | -1.3562 | -1.39038 | -0.95651 | -3.53638 | -1.30563 | -1.06954 | -1.64252 |
| Q6PE13 | Q6PE13 | PRRT3_M | Prrt3 | Proline-rich transmembrane protein 3 | | | + | 0.427797 | -1.03228 | -1.13697 | 0.250385 | 0.088521 | 0.689342 | 1.140573 | 0.313551 | 3.512952 | -3.52567 | -2.16116 | -0.5784 | -0.32207 | -0.70625 | -0.53194 | -2.41017 | -1.21211 | -0.53071 | -0.5468 | -0.95191 | -0.91339 |
| P07146 | P07146 | TRY2_MO | Prss2 | Anionic trypsin-2 | | | | NaN | 0 | NaN | 0.033344 | -0.14487 | -0.1048 | 0.275328 | 0.987216 | 0.752042 | -3.53964 | -3.47161 | -1.38601 | -3.23117 | -3.51342 | -0.92709 | -3.32524 | -3.04385 | -3.19308 | -1.13437 | -3.43317 | -2.98178 |
| Q9CPN9 | Q9CPN9 | Q9CPN9_ | Prss3b | RIKEN cDNA 2210010C04 gene | | + | | 0.686988 | 1.978963 | 1.849656 | 1.05253 | 0.882385 | 3.131919 | 0.208678 | 0.13315 | 0.58371 | 1.665602 | 2.496809 | 1.142806 | 0.752392 | 1.370822 | 1.060827 | -0.88365 | 1.088132 | -0.13794 | 0.268367 | 0.915312 | 1.250036 |
| P52734 | P52734 | FGD1_MO | Fgd1 | FYVE, RhoGEF and PH domain-containing protein 1 | + | | + | 1.666831 | -0.2734 | -6.70364 | 0.019182 | 0.02885 | 0.061162 | 1.253363 | -1.98783 | -4.05406 | -3.25452 | -3.32566 | -2.04516 | -2.75381 | -3.96511 | -3.55197 | -2.99673 | -3.03664 | -2.7397 | -2.11697 | -1.32602 | -2.21541 |
| Q52KR3 | Q52KR3 | PRUN2_M | Prune2 | Protein prune homolog 2 | + | | | 1.223906 | 1.121581 | 3.906557 | 0.206198 | 0.0613 | 0.577383 | 0.502961 | 0.292637 | 1.333063 | 1.03719 | 1.251508 | 1.245888 | 1.363938 | 1.365484 | 1.78846 | -0.24359 | 0.289122 | 1.331862 | 1.155365 | 1.343182 | 1.225489 |
| Q9CYH2 | Q9CYH2 | PXL2A_M | Prxl2a | Peroxiredoxin-like 2A | | | | 0.91001 | -0.39221 | -2.58094 | 0.561836 | -0.651 | -1.49185 | 0.138423 | -0.13273 | -0.40121 | 0.366074 | 0.59413 | 0.229744 | -0.35038 | 0.412148 | 0.634068 | 0.771859 | 0.972761 | 0.264671 | 0.916687 | 0.967483 | 0.344185 |
| Q9DB60 | Q9DB60 | PXL2B_M | Prxl2b | Prostamide/prostaglandin F synthase | | | | 0.348363 | 0.110592 | 0.935299 | 0.724 | -0.26653 | -1.96183 | 0.654603 | -0.34913 | -1.75405 | -0.07321 | -0.2125 | -0.33974 | -0.52893 | -0.27367 | 0.054216 | -0.349 | -0.15789 | -0.26532 | -0.07028 | 0.352275 | 0.126531 |
| Q61207 | Q61207 | SAP_MOU | Psap | Prosaposin | | | | 0.176204 | 0.284158 | 0.500291 | 0.554055 | 1.220905 | 1.47056 | 0.368258 | 0.15577 | 0.985441 | -0.5555 | -0.0877 | 1.262436 | -0.28928 | -0.55052 | -0.72913 | -1.12335 | -0.08817 | -1.02984 | -0.43882 | -0.66517 | -0.92602 |
| Q9CXZ1 | Q9CXZ1 | NDUS4_M | Ndufs4 | NADH dehydrogenase [ubiquinone] iron-sulfur protein 4, mitochondrial | + | | | 1.953515 | -0.31339 | -9.39959 | 0.267485 | -0.09551 | -0.73235 | 0.489195 | 0.233353 | 1.29665 | 1.868789 | 1.908817 | 1.355377 | 1.097383 | 1.716271 | 1.829331 | 2.228861 | 2.175529 | 1.341046 | 1.302728 | 1.710305 | 1.36859 |
| Q5DTT2 | Q5DTT2 | PSD1_MO | Psd | PH and SEC7 domain-containing protein 1 | | + | | 0.578123 | -2.14415 | -1.53675 | 1.149673 | -0.50677 | -3.55431 | 0.684135 | -0.54094 | -1.84114 | -4.00398 | -3.62354 | 0.124082 | 0.398866 | 0.252732 | 0.331335 | -0.28738 | -3.05183 | 0.806344 | 0.730135 | 0.541808 | 1.124144 |
| Q2PFD7 | Q2PFD7 | PSD3_MO | Psd3 | PH and SEC7 domain-containing protein 3 | | + | | 0.592108 | 1.44599 | 1.575662 | 1.350967 | -0.15077 | -4.57696 | 0.914788 | 0.210449 | 2.598231 | -0.54551 | -0.95531 | 0.400009 | 0.465297 | 0.871121 | 1.014663 | -1.30186 | -3.09094 | 0.579006 | 0.587849 | 0.769986 | 0.694901 |
| Q99JF8 | Q99JF8 | PSIP1_M | Psip1 | PC4 and SFRS1-interacting protein | | | | 0.736972 | 0.867319 | 2.001915 | 0.639997 | 0.612788 | 1.711669 | 0.455686 | -0.39422 | -1.20899 | 0.057149 | -0.41072 | -0.02855 | 0.598116 | -0.19352 | -0.41318 | -0.67944 | -1.40876 | -0.15482 | -0.50118 | 0.397896 | -0.21615 |
| Q9R1P4 | Q9R1P4 | PSA1_MO | Psma1 | Proteasome subunit alpha type-1 | | | | 0.239582 | 0.20177 | 0.662098 | 0.646659 | -0.56077 | -1.73095 | 0.049964 | 0.072052 | 0.154607 | 0.961103 | 1.289785 | 1.542369 | 0.896608 | 1.460838 | 2.350775 | 1.180307 | 0.66704 | 1.753744 | 1.806776 | 1.972293 | 1.695216 |
| P49722 | P49722 | PSA2_MO | Psma2 | Proteasome subunit alpha type-2 | | | | 0.10122 | -0.05694 | -0.30058 | 0.798918 | -0.58081 | -2.19932 | 0.070747 | 0.068397 | 0.215034 | 0.115338 | 0.010602 | 0.601927 | 0.090292 | 0.5165 | 1.136812 | 0.301965 | -0.06214 | 0.86136 | 0.99247 | 0.687719 | 0.828797 |
| Q8BGZ2 | Q8BGZ2 | F168A_M | Fam168a | Protein FAM168A | + | | | 1.662027 | -0.33241 | -6.66545 | 0.127926 | 0.276069 | 0.373176 | 0.401401 | 0.2197 | 1.06947 | 0.420746 | 0.327883 | 0.182495 | 0.729986 | 0.267874 | 0.54285 | 0.688526 | 0.724924 | 0.867444 | -0.5071 | 0.033026 | 0.338299 |
| Q91V76 | Q91V76 | CK054_M | N/A | Ester hydrolase C11orf54 homolog | + | | | 2.051936 | -0.34654 | -10.5454 | 0.085363 | 0.093231 | 0.256475 | 0.186336 | 0.122616 | 0.526461 | -0.92061 | -0.96031 | -1.22239 | -0.70726 | -0.87055 | -0.5151 | -0.62011 | -0.56773 | -1.31457 | -0.80154 | -0.66491 | -0.96597 |
| Q9Z2U1 | Q9Z2U1 | PSA5_MO | Psma5 | Proteasome subunit alpha type-5 | | | | 0.003572 | -0.00436 | -0.01158 | 0.276103 | -0.27351 | -0.75399 | 0.272281 | 0.158474 | 0.744394 | 0.107081 | 0.015268 | 0.296354 | -0.41838 | 0.026046 | 0.335278 | 0.439511 | -0.30843 | 0.15025 | 0.274737 | -0.12415 | 0.16853 |
| Q9WUM4 | Q9WUM4 | COR1C_M | Coro1c | Coronin-1C | + | + | | 1.988859 | -0.35242 | -9.79643 | 1.149908 | -0.40644 | -3.55538 | 0.173358 | 0.159234 | 0.492914 | -0.18005 | -0.25085 | 0.41427 | 0.209944 | 0.701628 | 1.313672 | 0.130568 | 0.143377 | 0.667253 | 0.76984 | 0.951901 | 0.74493 |
| Q9Z2U0 | Q9Z2U0 | PSA7_MO | Psma7 | Proteasome subunit alpha type-7 | | | | 0.987708 | -0.74482 | -2.87194 | 0.423368 | -0.51573 | -1.1256 | 0.168973 | -0.21964 | -0.48152 | 1.770614 | 1.909213 | 2.48204 | 1.739247 | 2.299882 | 3.19931 | 2.334819 | 2.834645 | 2.358057 | 2.894689 | 3.04551 | 2.892961 |
| O09061 | O09061 | PSB1_MO | Psmb1 | Proteasome subunit beta type-1 | | | | 0.646222 | -0.32577 | -1.72968 | 0.139572 | -0.19652 | -0.40427 | 0.493875 | 0.291555 | 1.309001 | 1.079403 | 0.99586 | 1.726537 | 0.757661 | 1.547413 | 1.961669 | 1.547049 | 1.17975 | 1.398124 | 1.479124 | 1.544882 | 1.381091 |
| Q9R1P3 | Q9R1P3 | PSB2_MO | Psmb2 | Proteasome subunit beta type-2 | | | | 0.128828 | 0.110982 | 0.375595 | 0.0454 | -0.038 | -0.14107 | 0.243436 | 0.231872 | 0.671824 | 1.028178 | 0.694609 | 0.930562 | 0.391822 | 1.099702 | 1.77723 | 0.506498 | 0.994324 | 0.703001 | 0.695391 | 1.272617 | 1.140571 |
| Q9R1P1 | Q9R1P1 | PSB3_MO | Psmb3 | Proteasome subunit beta type-3 | | | | 0.098631 | -0.03597 | -0.29344 | 0.064424 | -0.11198 | -0.19685 | 0.470928 | 0.524768 | 1.248698 | -0.96166 | -0.82478 | -0.09612 | -1.02544 | -0.37161 | 0.407685 | -0.95895 | -0.75554 | -0.77694 | -0.12066 | -0.3493 | -0.66417 |
| P99026 | P99026 | PSB4_MO | Psmb4 | Proteasome subunit beta type-4 | | | | 0.53568 | -0.24497 | -1.42066 | 0.446124 | -0.29392 | -1.18421 | 0.210443 | 0.182595 | 0.588211 | 1.301021 | 1.059635 | 1.050134 | 0.583793 | 1.08094 | 1.603707 | 1.548444 | 1.302145 | 1.02583 | 1.195937 | 1.327187 | 0.99227 |
| O55234 | O55234 | PSB5_MO | Psmb5 | Proteasome subunit beta type-5 | | | | 0.846023 | -0.45185 | -2.35654 | 0.37609 | -0.32344 | -1.00524 | 0.594939 | 0.25858 | 1.583582 | 0.873115 | 1.023423 | 1.101137 | 0.46071 | 1.397847 | 1.722011 | 1.576522 | 1.223719 | 1.135825 | 1.072902 | 1.281537 | 1.32116 |
| Q9WV80 | Q9WV80 | SNX1_MO | Snx1 | Sorting nexin-1 | + | + | | 2.36858 | -0.38428 | -15.2368 | 1.532537 | -0.21282 | -5.7088 | 0.461929 | 0.309327 | 1.225224 | -0.31066 | -0.353 | -0.12704 | -0.1765 | 0.46428 | -0.03701 | 0.038747 | 0.066156 | 0.088949 | 0.033156 | -0.12596 | -0.06543 |
| P28571 | P28571 | SC6A9_M | Slc6a9 | Sodium- and chloride-dependent glycine transporter 1 | + | | | 1.624022 | -0.38849 | -6.3703 | 0.0207 | 0.073883 | 0.065896 | NaN | 0 | NaN | 0.214341 | 0.106165 | -1.46562 | -3.52302 | -3.28683 | -3.77483 | 0.520574 | 0.576919 | -2.12223 | -3.01417 | -2.81296 | -3.49739 |
| P62192 | P62192 | PRS4_MO | Psmc1 | 26S proteasome regulatory subunit 4 | | | | 0.45049 | -0.26523 | -1.19551 | 0.363846 | -0.16671 | -0.97431 | 0.832074 | 0.431627 | 2.30931 | -0.06663 | 0.095178 | 0.994027 | 0.659323 | 1.392517 | 1.04778 | 0.486077 | 0.072927 | 0.957721 | 1.029056 | 0.860794 | 0.716249 |
| P46471 | P46471 | PRS7_MO | Psmc2 | 26S proteasome regulatory subunit 7 | + | | | 1.243653 | -0.28434 | -4.00493 | 0.082098 | -0.05579 | -0.24729 | 0.794877 | 0.62213 | 2.186131 | 0.366763 | 0.224771 | 1.243944 | 0.799097 | 1.811625 | 1.243367 | 0.579485 | 0.580738 | 1.039546 | 1.115074 | 0.889344 | 0.921388 |
| O88685 | O88685 | PRS6A_M | Psmc3 | 26S proteasome regulatory subunit 6A | + | | | 1.267608 | 0.697738 | 4.127043 | 0.058484 | 0.052472 | 0.179609 | 0.303818 | 0.331558 | 0.823486 | -0.2799 | -0.10484 | 0.852712 | 0.428283 | 1.00296 | 0.199958 | -1.03475 | -0.74546 | 0.387238 | 0.788812 | 0.239794 | 0.300008 |
| P56379 | P56379 | ATP68_M | Atp5mpl | ATP synthase subunit ATP5MPL, mitochondrial | + | | | 2.254971 | -0.39033 | -13.3558 | 0.170794 | -0.14062 | -0.48626 | 0.046609 | 0.035268 | 0.144666 | 2.611206 | 2.562781 | 2.610188 | 2.17508 | 2.407365 | 2.710907 | 2.960954 | 2.993688 | 2.723783 | 2.342726 | 2.71465 | 2.333087 |
| P62196 | P62196 | PRS8_MO | Psmc5 | 26S proteasome regulatory subunit 8 | | | | 0.922806 | 0.259841 | 2.627417 | 0.721854 | 0.243593 | 1.955232 | 0.385546 | 0.373895 | 1.029183 | 0.49348 | 0.46772 | 1.259041 | 1.198811 | 1.643565 | 1.033747 | 0.318812 | 0.122705 | 0.864442 | 1.106224 | 0.767246 | 1.162276 |
| P62334 | P62334 | PRS10_M | Psmc6 | 26S proteasome regulatory subunit 10B | | | | 0.8209 | 0.554648 | 2.271891 | 0.215959 | 0.1408 | 0.602252 | 0.608503 | 0.388951 | 1.621733 | 0.58035 | 0.110901 | 0.823265 | 0.549545 | 1.19489 | 0.728321 | -0.14189 | -0.27615 | 0.356061 | 0.735149 | 0.516979 | 0.62833 |
| Q3TXS7 | Q3TXS7 | PSMD1_M | Psmd1 | 26S proteasome non-ATPase regulatory subunit 1 | | | | 0.218654 | -0.15649 | -0.6091 | 0.090077 | 0.113158 | 0.269675 | 0.617778 | 0.527185 | 1.648016 | -1.27508 | -0.76126 | -0.22012 | -0.40372 | 0.211197 | -0.38583 | -0.86453 | -0.85882 | -0.83452 | -0.01563 | -0.49953 | -0.72948 |
| Q8BG32 | Q8BG32 | PSD11_M | Psmd11 | 26S proteasome non-ATPase regulatory subunit 11 | | | | 0.213741 | 0.22106 | 0.596608 | 0.261349 | 0.193842 | 0.71693 | 0.104874 | 0.120911 | 0.310634 | -0.81247 | -0.92172 | 0.034396 | -0.35968 | 0.103872 | -0.6709 | -0.72167 | -1.45463 | -0.54163 | -0.17134 | -0.3665 | -0.44235 |
| P28663 | P28663 | SNAB_MO | Napb | Beta-soluble NSF attachment protein | + | | | 1.708099 | -0.39097 | -7.04039 | 0.094307 | 0.165448 | 0.281455 | 0.313574 | 0.486213 | 0.847944 | -0.71456 | -0.78053 | 0.33495 | -0.81329 | 0.817336 | 0.616975 | -0.3119 | -0.40125 | -0.2784 | -0.53083 | 0.795526 | -0.33364 |
| Q9WVJ2 | Q9WVJ2 | PSD13_M | Psmd13 | 26S proteasome non-ATPase regulatory subunit 13 | + | | | 1.111597 | -0.27096 | -3.38379 | 0.134752 | 0.04064 | 0.391434 | 0.325846 | 0.294873 | 0.878726 | -0.16314 | -0.02645 | 0.59629 | 0.389728 | 0.989628 | 0.361634 | 0.217881 | 0.134443 | 0.441783 | 0.462955 | 0.262386 | 0.49913 |
| O35593 | O35593 | PSDE_MO | Psmd14 | 26S proteasome non-ATPase regulatory subunit 14 | | | | 0.486715 | -0.14461 | -1.29012 | 0.128691 | -0.04384 | -0.37523 | 0.119212 | 0.110427 | 0.349698 | 0.374107 | 0.194286 | 0.957069 | 0.742329 | 1.128468 | 0.554024 | 0.495735 | 0.361871 | 0.847461 | 0.93962 | 0.862043 | 0.599594 |
| Q8VDM4 | Q8VDM4 | PSMD2_M | Psmd2 | 26S proteasome non-ATPase regulatory subunit 2 | | | | 0.337864 | 0.258205 | 0.908902 | 0.495588 | -0.11917 | -1.31353 | 0.59854 | 0.364485 | 1.593678 | -0.54099 | -0.7774 | -0.31771 | -0.49663 | -0.0471 | -0.32365 | -0.65908 | -1.17572 | -0.30312 | -0.27288 | -0.36769 | -0.73203 |
| P14685 | P14685 | PSMD3_M | Psmd3 | 26S proteasome non-ATPase regulatory subunit 3 | | | | 0.573129 | 0.3425 | 1.522932 | 0.646115 | 0.362128 | 1.729368 | 0.449797 | 0.550204 | 1.193716 | 0.878107 | 0.793647 | 1.691734 | 1.297865 | 2.26206 | 1.34715 | 0.714272 | 0.272481 | 1.061503 | 1.203839 | 1.198019 | 1.310784 |
| O35226 | O35226 | PSMD4_M | Psmd4 | 26S proteasome non-ATPase regulatory subunit 4 | | | | 0.595066 | -0.30175 | -1.58394 | 0.549923 | 0.226045 | 1.459295 | 0.295568 | 0.217612 | 0.802806 | -1.15336 | -1.00539 | 0.51145 | 0.205424 | 0.051504 | 0.02001 | -0.95318 | -0.60207 | 0.156499 | 0.108284 | 0.088752 | -0.45246 |
| Q8BJY1 | Q8BJY1 | PSMD5_M | Psmd5 | 26S proteasome non-ATPase regulatory subunit 5 | | | | 0.355226 | 0.295959 | 0.952577 | 0.156322 | 0.099494 | 0.448478 | 0.589689 | 0.601872 | 1.568905 | 0.112251 | -0.47269 | 0.129898 | -0.29396 | 0.76716 | 0.001661 | -0.37134 | -0.58102 | -0.11593 | -0.24712 | -0.19156 | -0.24337 |
| Q99JI4 | Q99JI4 | PSMD6_M | Psmd6 | 26S proteasome non-ATPase regulatory subunit 6 | | | | 0.117638 | -0.10499 | -0.34544 | 0.180315 | 0.074019 | 0.510928 | 0.455835 | 0.336695 | 1.209379 | -0.06971 | -0.44506 | 0.389994 | 0.204788 | 0.622224 | 0.238081 | 0.086664 | -0.39146 | 0.11196 | 0.334784 | -0.10808 | 0.294993 |
| P26516 | P26516 | PSMD7_M | Psmd7 | 26S proteasome non-ATPase regulatory subunit 7 | | | + | 0.583471 | -2.22812 | -1.55159 | 0.021601 | -0.02486 | -0.0687 | 1.147817 | 0.249237 | 3.545842 | -1.20437 | -4.06893 | 0.133761 | -0.39677 | -0.29308 | -0.20427 | -0.30484 | -0.51222 | 0.139423 | -0.35272 | -0.44342 | -0.5524 |
| Q9CX56 | Q9CX56 | PSMD8_M | Psmd8 | 26S proteasome non-ATPase regulatory subunit 8 | | | | 0.21857 | -0.2817 | -0.60889 | 0.526007 | 0.189718 | 1.394606 | 0.955004 | 0.258016 | 2.746845 | -0.47053 | -0.67198 | -0.02487 | -0.23207 | -0.15987 | -0.30535 | 0.161982 | -0.7411 | -0.23002 | -0.40635 | -0.55006 | -0.43119 |
| Q9CR00 | Q9CR00 | PSMD9_M | Psmd9 | 26S proteasome non-ATPase regulatory subunit 9 | | | | 0.605363 | -0.35469 | -1.61287 | 0.464184 | -0.28209 | -1.2311 | 0.026196 | 0.050085 | 0.082925 | 0.219553 | -0.13417 | -0.02469 | -0.21335 | -0.05324 | 1.026058 | 0.266685 | 0.52808 | 0.371902 | -0.04575 | 0.707553 | 0.165093 |
| P97371 | P97371 | PSME1_M | Psme1 | Proteasome activator complex subunit 1 | | | | 0.876547 | 0.967996 | 2.461966 | 0.077163 | -0.1033 | -0.23333 | 0.80894 | -0.14692 | -2.23224 | -0.5797 | -0.93132 | -0.21521 | -0.68909 | -0.5803 | -0.49126 | -1.37182 | -2.07519 | -0.72285 | 0.025148 | -0.34039 | -0.43734 |
| P97372 | P97372 | PSME2_M | Psme2 | Proteasome activator complex subunit 2 | | | | NaN | 0 | NaN | 0.214027 | 1.255786 | 0.597337 | 0.338716 | 0.921842 | 0.911041 | -3.87638 | -2.83759 | 0.032127 | -4.15144 | -3.33296 | -1.38469 | -2.91736 | -3.12669 | -3.52555 | -3.10534 | -3.00696 | -3.55437 |
| Q8BHL8 | Q8BHL8 | PSMF1_M | Psmf1 | Proteasome inhibitor PI31 subunit | | | | 0.39532 | 1.195647 | 1.053997 | 0.116772 | -0.18403 | -0.34309 | 0.068867 | 0.272375 | 0.209645 | -3.39434 | -1.18182 | -1.4465 | -3.65432 | -2.75196 | -0.99787 | -3.23264 | -3.7348 | -1.22809 | -0.50466 | -1.18877 | -3.10582 |
| Q8R326 | Q8R326 | PSPC1_M | Pspc1 | Paraspeckle component 1 | | | | 0.62591 | 0.884797 | 1.671201 | 0.396489 | -0.47065 | -1.05697 | 0.182749 | 0.292306 | 0.517214 | -0.88293 | -1.17686 | -0.51839 | -0.71174 | 0.098385 | -0.99554 | -2.42333 | -1.40606 | 0.290241 | -0.57907 | -0.59865 | -0.88312 |
| Q99M15 | Q99M15 | PPIP2_M | Pstpip2 | Proline-serine-threonine phosphatase-interacting protein 2 | | | | NaN | 0 | NaN | 0.382929 | 0.279042 | 1.02255 | 0.339115 | -0.40752 | -0.91204 | -3.30921 | -3.07734 | 0.24223 | 0.771685 | -0.06632 | 0.337976 | -3.77822 | -2.94183 | 0.294154 | 0.161677 | 0.14487 | 0.941817 |
| Q91Z31 | Q91Z31 | PTBP2_M | Ptbp2 | Polypyrimidine tract-binding protein 2 | | + | | 0.21521 | 1.099185 | 0.600346 | 1.130619 | 0.426265 | 3.468152 | 0.198383 | 0.429862 | 0.557402 | -0.98824 | -2.69451 | -2.0311 | -1.93742 | -0.34348 | -1.85722 | -1.32055 | -4.56057 | -2.29689 | -2.52416 | -1.38229 | -1.67813 |
| Q99LH2 | Q99LH2 | PTSS1_M | Ptdss1 | Phosphatidylserine synthase 1 | | | | 0.587525 | 1.50307 | 1.56287 | 0.918532 | 0.442877 | 2.61183 | 0.601286 | -0.58798 | -1.60139 | -0.36477 | -0.62544 | -0.43695 | -0.46413 | -0.61136 | -1.32299 | -1.04531 | -2.95104 | -1.06243 | -0.72439 | -0.46977 | -0.28863 |
| O09114 | O09114 | PTGDS_M | Ptgds | Prostaglandin-H2 D-isomerase | | | + | 0.211686 | 0.169848 | 0.591377 | 0.211931 | 0.145433 | 0.592001 | 1.598225 | 0.788259 | 6.17695 | -0.85941 | -0.76401 | -0.59754 | -0.67131 | -0.75711 | -0.65229 | -1.26477 | -0.69834 | -0.53698 | -1.02274 | -1.37661 | -1.60931 |
| Q9R0Q7 | Q9R0Q7 | TEBP_MO | Ptges3 | Prostaglandin E synthase 3 | + | | | 1.187014 | -0.48601 | -3.72811 | 0.242832 | -0.32009 | -0.6703 | 0.175729 | 0.186392 | 0.499063 | 1.007083 | 0.771433 | 0.57313 | -0.11291 | 0.68676 | 1.420165 | 1.319486 | 1.43106 | 0.882432 | 0.217971 | 0.937922 | 0.796219 |
| Q9D0J8 | Q9D0J8 | PTMS_MO | Ptms | Parathymosin | | | | 0.13135 | -0.77226 | -0.38235 | 0.49405 | 1.371224 | 1.309464 | 0.038397 | 0.114644 | 0.120096 | -3.59388 | 0.410478 | 1.414175 | -0.67222 | 0.71419 | -1.02268 | -1.08553 | -0.55335 | -0.9092 | -1.0913 | 0.127452 | -0.66523 |
| Q99L04 | Q99L04 | DHRS1_M | Dhrs1 | Dehydrogenase/reductase SDR family member 1 | + | | | 1.60308 | -0.39148 | -6.21292 | 0.040401 | -0.07877 | -0.12613 | 0.292628 | 0.223886 | 0.795435 | 0.220246 | 0.346101 | -0.39606 | -0.88289 | -0.02402 | 0.369827 | 0.67787 | 0.67143 | 0.01443 | -1.13584 | 0.150118 | -0.25209 |
| P35235 | P35235 | PTN11_M | Ptpn11 | Tyrosine-protein phosphatase non-receptor type 11 | | | | 0.181091 | -0.14614 | -0.51293 | 0.870911 | 0.234793 | 2.442284 | 0.334675 | -0.2086 | -0.90089 | 0.063182 | -0.28001 | 0.316494 | 0.422693 | -0.08281 | 0.155074 | 0.265162 | -0.18971 | 0.214942 | 0.054658 | 0.443391 | 0.046067 |
| Q64512 | Q64512 | PTN13_M | Ptpn13 | Tyrosine-protein phosphatase non-receptor type 13 | | + | + | 0.083719 | 0.237783 | 0.251852 | 1.321393 | -1.19511 | -4.41274 | 1.458575 | 0.937204 | 5.220606 | -4.39671 | -2.53846 | -3.37729 | -3.91818 | -3.044 | -3.30868 | -3.53768 | -3.87305 | -2.46703 | -2.43821 | -4.23484 | -3.99224 |
| Q6PB44 | Q6PB44 | PTN23_M | Ptpn23 | Tyrosine-protein phosphatase non-receptor type 23 | | | | 0.301634 | 0.902875 | 0.81801 | 0.096994 | 0.073939 | 0.288908 | 0.680015 | 0.502259 | 1.828875 | -1.50385 | -1.63027 | -1.14436 | -0.95116 | -0.88619 | -1.21323 | -3.57187 | -1.368 | -1.35869 | -0.88471 | -1.77261 | -1.33133 |
| A2ALK8 | A2ALK8 | PTN3_MO | Ptpn3 | Tyrosine-protein phosphatase non-receptor type 3 | | + | | NaN | 0 | NaN | 1.434594 | -0.90364 | -5.0706 | 0.668377 | -0.33681 | -1.79444 | -3.47633 | -2.70692 | -1.88762 | -1.56831 | -1.38094 | -1.74081 | -3.48347 | -3.13068 | -0.9035 | -0.74514 | -1.17064 | -1.27748 |
| P54830 | P54830 | PTN5_MO | Ptpn5 | Tyrosine-protein phosphatase non-receptor type 5 | | | | 0.57203 | 0.312392 | 1.5199 | 0.532355 | 0.573692 | 1.411689 | 0.229683 | 0.351748 | 0.637071 | -0.58988 | -0.19117 | 0.808832 | 1.426171 | 0.365908 | -0.44095 | -0.75293 | -0.6529 | 0.808145 | 0.279475 | -0.01231 | -0.76623 |
| O35239 | O35239 | PTN9_MO | Ptpn9 | Tyrosine-protein phosphatase non-receptor type 9 | | | | 0.301724 | -0.23909 | -0.81824 | 0.251396 | -0.1707 | -0.69189 | 0.027916 | 0.021929 | 0.088215 | -0.59928 | -1.18074 | -0.81989 | -0.607 | -0.22178 | -0.71853 | -0.62163 | -0.68021 | -0.76532 | -0.32018 | -0.48195 | -0.50222 |
| P18052 | P18052 | PTPRA_M | Ptpra | Receptor-type tyrosine-protein phosphatase alpha | | | | 0.297128 | 0.298561 | 0.806715 | 0.291529 | 0.421329 | 0.792679 | 0.155338 | 0.098832 | 0.445893 | -1.38932 | -1.08504 | -0.28315 | -0.40431 | 0.086202 | -0.31324 | -1.87312 | -1.19837 | -1.29312 | -0.237 | -0.30848 | -0.11623 |
| Q64487 | Q64487;A | PTPRD_M | Ptprd | Receptor-type tyrosine-protein phosphatase delta | | | | 0.052259 | 0.102561 | 0.161373 | 0.175984 | 0.033672 | 0.499723 | 0.146154 | 0.269734 | 0.421707 | -1.22606 | -0.53586 | -0.14094 | -0.00766 | 0.382052 | -0.86504 | -0.44982 | -1.51722 | -0.11794 | -0.098 | -0.65374 | -0.36872 |
| Q9ES56 | Q9ES56 | TPPC4_M | Trappc4 | Trafficking protein particle complex subunit 4 | + | | | 2.768889 | -0.4117 | -24.2041 | 0.743616 | -0.28307 | -2.02261 | 0.079016 | -0.07772 | -0.23858 | -0.20341 | -0.20207 | 0.032542 | 0.196767 | -0.11977 | 0.499685 | 0.191957 | 0.22595 | 0.511052 | 0.28439 | 0.166688 | 0.368677 |
| D3Z7P3 | D3Z7P3 | GLSK_MO | Gls | Glutaminase kidney isoform, mitochondrial | + | | | 1.474558 | -0.42047 | -5.32278 | 0.059623 | 0.132083 | 0.182927 | 0.364586 | 0.645001 | 0.976174 | 0.065292 | 0.016819 | 1.328128 | -0.10809 | 1.60516 | 1.364045 | 0.386341 | 0.53671 | 0.402531 | 0.553344 | 1.489254 | 0.189948 |
| Q64455 | Q64455 | PTPRJ_M | Ptprj | Receptor-type tyrosine-protein phosphatase eta | | + | | 0.017733 | -0.03286 | -0.05663 | 1.04146 | 1.390419 | 3.086341 | 0.598936 | 0.953697 | 1.594788 | -2.90068 | -4.02885 | -2.44865 | -1.54779 | -0.99168 | -2.17395 | -3.56785 | -3.29596 | -3.39705 | -3.38023 | -2.62692 | -2.44611 |
| Q60673 | Q60673 | PTPRN_M | Ptprn | Receptor-type tyrosine-protein phosphatase-like N | | | + | 0.618737 | 1.023671 | 1.650744 | 0.510738 | 0.29543 | 1.353748 | 1.002174 | 1.664459 | 2.928542 | -1.93563 | -3.11771 | -2.47648 | -2.91025 | -2.05703 | -1.35685 | -3.73803 | -3.36265 | -3.01299 | -2.9646 | -2.92366 | -3.81913 |
| P80560 | P80560 | PTPR2_M | Ptprn2 | Receptor-type tyrosine-protein phosphatase N2 | | + | | 0.579283 | 1.261012 | 1.539962 | 1.823392 | 1.273054 | 8.067914 | 0.681251 | 0.818576 | 1.832553 | -0.98027 | -1.59205 | -0.59662 | -0.3693 | -0.74844 | -1.14292 | -1.30675 | -1.7876 | -1.86547 | -1.64656 | -2.16504 | -1.36348 |
| B0V2N1 | B0V2N1 | PTPRS_M | Ptprs | Receptor-type tyrosine-protein phosphatase S | | | | 0.350188 | 0.181739 | 0.939891 | 0.9231 | 0.154325 | 2.628491 | 0.30624 | 0.360143 | 0.829557 | 0.014073 | -0.19458 | 1.173722 | 1.102381 | 1.901423 | 1.060945 | -0.43479 | -0.10919 | 0.937092 | 1.030361 | 1.012061 | 1.23002 |
| Q99M80 | Q99M80 | PTPRT_M | Ptprt | Receptor-type tyrosine-protein phosphatase T | | | | NaN | 0 | NaN | 0.537584 | 0.555844 | 1.42581 | 0.390489 | 0.683481 | 1.041725 | -3.63004 | -3.28425 | -2.68633 | -2.42286 | -2.97291 | -3.51177 | -3.40947 | -3.21734 | -3.47735 | -2.74353 | -4.52405 | -3.32759 |
| B9EKR1 | B9EKR1 | PTPRZ_M | Ptprz1 | Receptor-type tyrosine-protein phosphatase zeta | | | | 0.413637 | 0.176864 | 1.100689 | 0.456081 | 0.364272 | 1.210018 | 0.502375 | 0.393461 | 1.331508 | 1.063346 | 0.816049 | 1.675526 | 1.20939 | 1.398557 | 0.976098 | 0.865453 | 0.660214 | 1.268734 | 0.887639 | 1.000512 | 0.587219 |
| D3Z4S3 | D3Z4S3 | PTRD1_M | Ptrhd1 | Putative peptidyl-tRNA hydrolase PTRHD1 | | + | | 0.484823 | -1.38063 | -1.28514 | 1.326446 | -0.32166 | -4.44044 | 0.156275 | 0.197579 | 0.448352 | -2.84942 | -0.89274 | -1.42189 | -1.56018 | -0.86444 | -0.13332 | -0.93427 | -0.04663 | -1.19097 | -1.14778 | -1.45036 | -0.94255 |
| Q3UEB3 | Q3UEB3 | PUF60_M | Puf60 | Poly(U)-binding-splicing factor PUF60 | | | | 0.285623 | 0.646398 | 0.77787 | 0.40235 | 0.733282 | 1.071887 | 0.475264 | 0.942414 | 1.260044 | -1.69361 | -3.16158 | -2.43782 | -2.15553 | -1.91343 | -3.40727 | -2.68438 | -3.46361 | -3.69935 | -2.36058 | -3.56412 | -3.6414 |
| Q80U78 | Q80U78 | PUM1_MO | Pum1 | Pumilio homolog 1 | | | | NaN | 0 | NaN | 0.415545 | 0.146305 | 1.105566 | 0.024284 | -0.00484 | -0.07702 | -2.93805 | -3.73379 | -0.95605 | -1.1659 | -1.47838 | -1.52832 | -3.29625 | -3.53023 | -1.12663 | -1.28792 | -1.44081 | -1.5562 |
| P42669 | P42669 | PURA_MO | Pura | Transcriptional activator protein Pur-alpha | | | | 0.589244 | 0.595631 | 1.567663 | 0.36429 | 0.114369 | 0.975426 | 0.569987 | 0.478349 | 1.514265 | 2.90425 | 3.039874 | 3.152664 | 3.355093 | 3.353669 | 2.738492 | 2.750278 | 2.002582 | 3.198698 | 3.080321 | 2.495766 | 2.639698 |
| O35295 | O35295 | PURB_MO | Purb | Transcriptional activator protein Pur-beta | | | | 0.605343 | 0.856492 | 1.612815 | 0.926133 | 0.446443 | 2.639591 | 0.663438 | 0.591776 | 1.779911 | 0.769881 | 0.996225 | 1.784144 | 2.091566 | 1.827742 | 1.182989 | 0.545416 | -0.49229 | 1.420849 | 1.561974 | 0.832268 | 0.99491 |
| Q8R4E6 | Q8R4E6 | PURG_MO | Purg | Purine-rich element-binding protein gamma | | | | 0.436148 | -1.23209 | -1.15846 | 0.32165 | 0.244105 | 0.868199 | 0.610094 | 1.992974 | 1.626228 | -3.12221 | -3.11496 | -0.16232 | -2.27438 | -0.222 | -2.67099 | -0.82293 | -2.95005 | -0.18693 | -0.73798 | -3.48968 | -3.38926 |
| P32848 | P32848 | PRVA_MO | Pvalb | Parvalbumin alpha | | | | 0.06303 | 0.317206 | 0.192815 | 0.496242 | 1.170627 | 1.315259 | NaN | 0 | NaN | -3.40377 | -0.93436 | -1.69554 | -3.46788 | -3.29905 | -3.90562 | -3.57346 | -1.39909 | -3.8352 | -3.66947 | -2.70158 | -3.39593 |
| Q69Z61 | Q69Z61 | PWP2A_M | Pwwp2a | PWWP domain-containing protein 2A | | | | NaN | 0 | NaN | 0.339258 | 1.046955 | 0.912403 | 0.808343 | -1.6578 | -2.23027 | -3.44307 | -3.14168 | -1.66222 | -1.39441 | -2.74452 | -3.29564 | -3.94862 | -3.10525 | -3.7149 | -1.43564 | -2.05263 | -0.67193 |
| Q8VI36 | Q8VI36 | PAXI_MO | Pxn | Paxillin | | | | NaN | 0 | NaN | 0.988407 | 0.63298 | 2.87466 | 0.824816 | 0.794539 | 2.284965 | -3.52328 | -3.3454 | -4.44811 | -0.1803 | -0.26421 | 0.413455 | -3.17161 | -2.58517 | -1.12198 | -0.77238 | -0.64179 | -0.79805 |
| Q922Q4 | Q922Q4;Q | P5CR2_M | Pycr2 | Pyrroline-5-carboxylate reductase 2 | | | | 0.068875 | 0.065 | 0.209668 | 0.70586 | 0.497656 | 1.906448 | 0.001414 | 0.002251 | 0.004597 | 0.122976 | -0.05859 | 0.305228 | 0.33362 | 1.129754 | 0.243833 | 0.263615 | -0.32923 | -0.43888 | 0.08242 | 0.475575 | 0.893509 |
| Q9DCC4 | Q9DCC4 | P5CR3_M | Pycr3 | Pyrroline-5-carboxylate reductase 3 | | | | 0.453182 | -0.40014 | -1.20249 | 0.295231 | -0.20661 | -0.80196 | 0.267581 | 0.080374 | 0.732593 | -0.27644 | 0.385794 | -0.04084 | -0.52496 | 0.17007 | 0.342945 | 0.487812 | 0.421816 | -0.1645 | 0.011929 | 0.243699 | 0.108568 |
| Q8CI94 | Q9WUB3;Q | PYGB_MO | Pygb | Glycogen phosphorylase, brain form | | | | 0.78613 | -1.26229 | -2.15773 | 0.264789 | -0.23226 | -0.72558 | 0.519303 | -0.42504 | -1.37662 | 2.99498 | 2.826566 | 2.517465 | 1.881477 | 2.607803 | 3.223691 | 3.594148 | 4.751981 | 2.395003 | 2.468466 | 3.318452 | 3.363113 |
| Q9WUB3 | Q9WUB3 | PYGM_MO | Pygm | Glycogen phosphorylase, muscle form | | | + | 0.54035 | -0.90346 | -1.43329 | 0.600568 | -0.48735 | -1.59937 | 2.661211 | -0.46926 | -21.3744 | 2.720407 | 2.62156 | 0.229913 | 0.124126 | 0.427044 | 0.44354 | 2.946046 | 4.202839 | 0.964461 | 0.364283 | 0.884209 | 0.924901 |
| Q61838 | Q61838 | PZP_MOU | Pzp | Pregnancy zone protein | | | | 0.514124 | -2.02324 | -1.36278 | 0.465684 | -1.49891 | -1.23501 | 0.417201 | 1.492552 | 1.109804 | -3.43728 | -0.47253 | -0.72596 | -1.11053 | 0.051326 | -1.13159 | 0.150426 | -0.01374 | -0.64622 | -0.19245 | -0.82484 | -3.24052 |
| Q8BML9 | Q8BML9 | SYQ_MOU | Qars1 | Glutamine--tRNA ligase | | + | | 0.255893 | 0.41437 | 0.703209 | 1.06999 | -0.18172 | -3.20483 | 0.339679 | -0.58548 | -0.91346 | -1.21632 | -0.40103 | -1.02788 | -1.13521 | -1.57119 | -2.84462 | -0.79755 | -1.64854 | -0.91814 | -0.88152 | -1.69599 | -1.54884 |
| Q8BVI4 | Q8BVI4 | DHPR_MO | Qdpr | Dihydropteridine reductase | + | | | 1.184878 | -1.54656 | -3.71799 | 0.516831 | 0.505301 | 1.37001 | 0.106665 | -0.25285 | -0.31555 | 0.343514 | 0.91502 | 1.006831 | 0.388237 | -0.36646 | 1.101038 | 1.873545 | 2.478103 | 0.393158 | -0.00869 | 0.942174 | 0.298106 |
| Q9QYS9 | Q9QYS9 | QKI_MOU | Qki | KH domain-containing RNA-binding protein QKI | | | | 0.197695 | 0.280584 | 0.555641 | 0.469794 | -0.39116 | -1.24574 | 0.549077 | 0.573376 | 1.45699 | 0.133149 | -0.71958 | -1.49774 | -1.24642 | -0.25239 | -0.7816 | -0.84437 | -0.30323 | -0.69317 | -1.26868 | -0.79908 | -1.38167 |
| E9Q9Q2 | E9Q9Q2 | E9Q9Q2_ | R3hdm1 | R3H domain containing 1 | | | | 0.294172 | 0.770485 | 0.799306 | 0.142031 | -0.29648 | -0.41079 | 0.571749 | -0.83305 | -1.51912 | -1.9988 | -3.83934 | -0.34744 | 0.277795 | -0.83029 | 0.261496 | -3.97641 | -3.4027 | -0.38885 | 0.912173 | 0.496571 | 0.600726 |
| Q80TM6 | Q80TM6 | R3HD2_M | R3hdm2 | R3H domain-containing protein 2 | | | | NaN | 0 | NaN | 0.740666 | -0.60769 | -2.01341 | 0.64914 | -1.25 | -1.73815 | -2.33654 | -3.07725 | 0.175904 | 0.05271 | -1.89158 | -0.97264 | -2.77042 | -2.46442 | 0.426529 | 1.017473 | -0.73535 | 0.37113 |
| P61027 | P61027 | RAB10_M | Rab10 | Ras-related protein Rab-10 | | | | 0.071652 | 0.089611 | 0.217623 | 0.021232 | -0.04717 | -0.06755 | 0.322323 | 0.30431 | 0.869886 | 0.0613 | -0.59101 | 0.127171 | -1.15191 | 0.223794 | -0.27567 | -0.10311 | -0.60582 | -0.18482 | -0.74558 | -0.08527 | -0.57522 |
| P46638 | P46638;P | RB11B_M | Rab11b | Ras-related protein Rab-11B | | | | 0.852924 | -0.35538 | -2.38013 | 0.10894 | 0.21332 | 0.321771 | 0.60457 | 0.628666 | 1.610636 | 1.070183 | 1.191332 | 1.999229 | 0.732821 | 1.835964 | 1.453658 | 1.622616 | 1.349668 | 1.349077 | 0.956335 | 1.356456 | 0.675835 |
| G3XA57 | G3XA57 | RFIP2_M | Rab11fip2 | Rab11 family-interacting protein 2 | | | | 0.632366 | 1.926964 | 1.689698 | 0.031647 | 0.024055 | 0.099634 | 0.762426 | -0.45798 | -2.08181 | -0.30558 | -0.7106 | -0.02341 | -0.34732 | -0.67479 | -0.60338 | -1.31276 | -3.55735 | -0.38848 | -0.03037 | -0.39818 | 0.035972 |
| Q8R361 | Q8R361 | RFIP5_M | Rab11fip5 | Rab11 family-interacting protein 5 | | | + | 0.019661 | 0.018837 | 0.062658 | 0.107758 | -0.12302 | -0.31854 | 1.490826 | -0.22455 | -5.42863 | -0.17184 | -0.57335 | 0.942544 | 0.969743 | 0.283729 | 0.364031 | -0.6152 | -0.16765 | 0.693195 | 1.465136 | 0.558365 | 0.538486 |
| P35283 | P35283 | RAB12_M | Rab12 | Ras-related protein Rab-12 | | | | 0.357767 | -0.42404 | -0.95898 | 0.320774 | 0.28136 | 0.866002 | 0.915644 | 0.59172 | 2.601336 | -3.13713 | -3.99658 | -2.57047 | -2.93645 | -2.68345 | -2.88353 | -3.03862 | -3.24701 | -3.30328 | -2.76636 | -3.17092 | -3.5795 |
| Q91V41 | Q91V41 | RAB14_M | Rab14 | Ras-related protein Rab-14 | | | | 0.818866 | -0.23494 | -2.26512 | 0.895613 | -0.22424 | -2.52931 | 0.618631 | 0.306987 | 1.650443 | 2.261817 | 2.384799 | 1.945081 | 1.884435 | 2.605213 | 2.778502 | 2.474722 | 2.64178 | 2.055688 | 2.222308 | 2.54946 | 2.220282 |
| Q8K386 | Q8K386 | RAB15_M | Rab15 | Ras-related protein Rab-15 | + | | | 1.084841 | 0.335224 | 3.26785 | 0.769081 | 0.404965 | 2.102973 | 0.413737 | 0.188407 | 1.100944 | 2.862893 | 3.013479 | 4.014832 | 3.631516 | 3.85264 | 3.711193 | 2.672633 | 2.53329 | 3.436903 | 3.399516 | 3.749344 | 3.437675 |
| P35293 | P35293 | RAB18_M | Rab18 | Ras-related protein Rab-18 | | + | | 0.065492 | 0.039492 | 0.199933 | 1.486948 | -0.38138 | -5.40323 | 0.577852 | 0.234307 | 1.535996 | 2.49084 | 2.622345 | 1.933918 | 1.799252 | 2.523716 | 2.505713 | 2.330837 | 2.703364 | 2.26913 | 2.226791 | 2.432686 | 2.12813 |
| P62821 | P62821 | RAB1A_M | Rab1A | Ras-related protein Rab-1A | + | | | 1.161305 | -0.45236 | -3.60774 | 0.114319 | 0.139914 | 0.336431 | 0.687558 | 0.302613 | 1.851359 | 3.919329 | 4.122823 | 4.344091 | 3.513577 | 4.19256 | 4.139435 | 4.546717 | 4.400163 | 3.76621 | 3.811629 | 4.024667 | 3.702104 |
| Q9D1G1 | Q9D1G1 | RAB1B_M | Rab1b | Ras-related protein Rab-1B | | | | 0.363633 | 1.025648 | 0.973768 | 0.276624 | 0.582648 | 0.755296 | 0.040713 | 0.058062 | 0.12706 | 0.9764 | 0.932991 | 1.677893 | 1.047558 | 0.989211 | 1.017267 | -1.12401 | 0.982101 | 0.075982 | 1.484174 | 1.401928 | 0.488425 |
| P35282 | P35282 | RAB21_M | Rab21 | Ras-related protein Rab-21 | | | + | 0.686893 | -0.38883 | -1.84937 | 0.132865 | 0.05883 | 0.386397 | 1.582688 | 0.250007 | 6.063134 | 0.815478 | 1.232846 | 1.100902 | 0.797213 | 1.263905 | 1.254669 | 1.38738 | 1.438605 | 0.879104 | 0.901351 | 0.968305 | 1.050254 |
| P35285;Q | P35285;Q | RAB31_M | Rab22a;Rab31 | Ras-related protein Rab-22A | | | | 0.088903 | -0.17886 | -0.2664 | 0.070245 | 0.226629 | 0.213596 | 0.025496 | -0.06134 | -0.08076 | -2.76481 | -3.27213 | -1.76112 | -3.84254 | -2.1048 | -3.52264 | -3.46125 | -2.21797 | -3.23506 | -2.82187 | -2.47994 | -3.02482 |
| P35288 | P35288 | RAB23_M | Rab23 | Ras-related protein Rab-23 | | | | NaN | 0 | NaN | 0.269573 | -0.63844 | -0.73759 | 0.177839 | 0.316694 | 0.504525 | -3.8375 | -3.75825 | -2.90401 | -4.08536 | -2.54389 | -1.5648 | -3.30143 | -2.80087 | -3.48895 | -2.22353 | -1.97815 | -2.76393 |
| Q504M8 | Q504M8 | RAB26_M | Rab26 | Ras-related protein Rab-26 | | | | NaN | 0 | NaN | 0.421437 | 1.242808 | 1.120653 | 0.00696 | 0.009789 | 0.022486 | -3.44452 | -3.61644 | -0.85644 | -1.10984 | -0.69115 | -0.97505 | -2.9751 | -3.16606 | -3.32769 | -1.12421 | -1.25444 | -0.43134 |
| P53994 | P59279;P | RAB2A_M | Rab2a | Ras-related protein Rab-2A | | | | 0.111411 | 0.110293 | 0.328515 | 0.233402 | 0.381803 | 0.64648 | 0.265177 | 0.157164 | 0.726551 | 1.383747 | 1.497659 | 2.346453 | 1.219419 | 1.934278 | 1.845781 | 0.999546 | 1.661274 | 1.224378 | 1.577887 | 1.944607 | 1.521124 |
| P59279 | P59279 | RAB2B_M | Rab2b | Ras-related protein Rab-2B | | + | + | 0.23571 | -0.06917 | -0.65232 | 1.012414 | 0.545832 | 2.96909 | 1.272641 | 0.315473 | 4.153092 | -1.78109 | -1.81406 | -1.88628 | -1.52021 | -1.26563 | -1.15361 | -1.62367 | -1.83315 | -2.23188 | -2.26627 | -1.47378 | -1.5764 |
| Q923S9 | Q923S9 | RAB30_M | Rab30 | Ras-related protein Rab-30 | | | | 0.590054 | -0.68157 | -1.56992 | 0.132074 | -0.13893 | -0.38429 | 0.917741 | -0.3937 | -2.60895 | -0.83516 | 0.026404 | -0.13003 | -0.83781 | -0.25132 | -0.1825 | 0.34935 | 0.209627 | -0.27109 | -0.41889 | 0.323723 | 0.029865 |
| P97950 | P97950 | RB33A_M | Rab33a | Ras-related protein Rab-33A | | | | 0.444569 | -1.01338 | -1.18019 | 0.05867 | 0.033056 | 0.180151 | 0.948122 | 0.566561 | 2.72101 | -0.76843 | -2.48426 | -0.11714 | 0.221813 | 0.204196 | -0.13106 | -0.64858 | -0.57736 | -0.05105 | 0.089612 | -0.65351 | -0.40648 |
| Q35963 | Q35963;P | RB33B_M | Rab33b | Ras-related protein Rab-33B | | + | | 0.151694 | -0.09209 | -0.43632 | 2.262061 | 0.137539 | 13.46614 | 0.596296 | 0.199574 | 1.587383 | 1.334666 | 1.062042 | 1.377675 | 1.358059 | 1.6558 | 1.420498 | 1.455568 | 1.127316 | 1.227478 | 1.233179 | 1.294245 | 1.382905 |

| | | | | | | | | | | | | | | | | | | | | | | | | | | | | |
|---|---|---|---|---|---|---|---|---|---|---|---|---|---|---|---|---|---|---|---|---|---|---|---|---|---|---|---|---|
| Q6PHN9 | Q6PHN9 | RAB35_M | Rab35 | Ras-related protein Rab-35 | | | | 0.337212 | -0.07241 | -0.90726 | 0.200205 | -0.30325 | -0.56207 | 0.530929 | 0.340222 | 1.407847 | -1.04579 | -0.90618 | -0.68632 | -1.57061 | -0.31288 | -0.74546 | -0.94227 | -0.86489 | -0.51602 | -1.1344 | -0.7616 | -0.97719 |
| Q8BHC1 | Q8BHC1; | RAB39B_M | Rab39b | Ras-related protein Rab-39B | | | | 0.131752 | -0.07882 | -0.38342 | 0.347893 | 0.42753 | 0.934116 | 0.760782 | -0.04251 | -2.0766 | -0.08807 | 0.194182 | -0.64426 | -0.55697 | -0.15573 | -0.15107 | 0.281345 | -0.0176 | -0.57254 | -1.48374 | -0.09055 | -0.13123 |
| P63011 | P35276;P | RAB3A_M | Rab3a | Ras-related protein Rab-3A | | | | 0.074149 | -0.03761 | -0.22475 | 0.415437 | 0.612988 | 1.10529 | 0.352506 | 0.424008 | 0.945726 | 2.307212 | 2.316087 | 3.95593 | 2.853508 | 3.831955 | 3.305387 | 2.516562 | 2.181965 | 2.730566 | 2.852897 | 3.507556 | 2.78177 |
| Q9CZT8 | Q9CZT8;P | RAB3B_M | Rab3b | Ras-related protein Rab-3B | | | | 0.079305 | 0.084496 | 0.239397 | 0.689716 | 0.110294 | 1.857815 | 0.034598 | -0.01245 | -0.10861 | 2.0906 | 2.598987 | 3.894168 | 3.8234 | 3.934135 | 4.017397 | 2.015428 | 2.505167 | 3.70082 | 3.796161 | 3.881403 | 4.095033 |
| P62823 | P62823 | RAB3C_M | Rab3c | Ras-related protein Rab-3C | | | + | 0.722132 | -0.29241 | -1.95609 | 0.583201 | 0.523745 | 1.550836 | 1.015583 | 0.770722 | 2.981723 | 1.089334 | 0.969782 | 2.6679 | 1.996012 | 3.114433 | 2.598945 | 1.184951 | 1.458978 | 1.773642 | 1.84278 | 2.105487 | 2.066447 |
| P35276 | P35276 | RAB3D_M | Rab3d | Ras-related protein Rab-3D | | | | 0.561318 | -0.25078 | -1.49043 | 0.007771 | 0.004537 | 0.025084 | 0.408849 | -0.03953 | -1.08846 | 1.943095 | 2.23743 | 3.88572 | 3.606568 | 3.761881 | 3.771907 | 2.259477 | 2.422604 | 3.626553 | 3.85666 | 3.842397 | 3.770454 |
| Q80UJ7 | Q80UJ7 | RB3GP_M | Rab3gap1 | Rab3 GTPase-activating protein catalytic subunit | | | | 0.284092 | 0.930544 | 0.774031 | 0.150658 | -0.05286 | -0.43359 | 0.155851 | -0.16407 | -0.44724 | -0.94511 | -1.58236 | -0.74509 | -0.62979 | -0.04845 | -0.778 | -1.03507 | -3.35349 | -0.52718 | -0.74199 | -0.28817 | -0.21014 |
| Q8BMG7 | Q8BMG7 | RBGPR_M | Rab3gap2 | Rab3 GTPase-activating protein non-catalytic subunit | | | + | 0.282692 | 0.142059 | 0.77052 | 0.906197 | -0.18652 | -2.5672 | 1.133446 | 0.191541 | 3.480829 | 0.128434 | 0.032611 | 0.273147 | 0.25259 | 0.68614 | 0.578493 | 0.116497 | -0.23957 | 0.377462 | 0.521307 | 0.452222 | 0.429329 |
| Q68EF0 | Q68EF0 | RAB3I_MO | Rab3ip | Rab-3A-interacting protein | | | | 0.181035 | 0.254344 | 0.512787 | 0.984427 | -0.46062 | -2.85921 | 0.513018 | -0.30894 | -1.35983 | -2.81407 | -3.37985 | -2.34281 | -2.20182 | -1.71734 | -2.16814 | -2.94388 | -3.75873 | -1.95655 | -1.66684 | -1.6623 | -1.6053 |
| Q9D8W5 | Q9D8W5 | PSD12_M | Psmd12 | 26S proteasome non-ATPase regulatory subunit 12 | + | | | 1.648668 | -0.4238 | -6.56028 | 0.028228 | -0.0243 | -0.08917 | 0.463567 | 0.266418 | 1.22949 | -0.75768 | -0.62998 | 0.437616 | -0.08949 | 0.197929 | 0.183549 | -0.26019 | -0.27985 | 0.128952 | 0.267778 | -0.29225 | 0.140892 |
| Q9CQD1 | Q9CQD1 | RAB5A_M | Rab5a | Ras-related protein Rab-5A | | | | NaN | 0 | NaN | 0.164697 | 0.467956 | 0.470383 | 0.821781 | 0.730171 | 2.274826 | -3.31418 | -3.7172 | -1.76381 | -2.3005 | -1.85417 | -2.28334 | -3.3526 | -3.5086 | -3.45808 | -1.54214 | -2.56022 | -3.03763 |
| P61021 | P61021 | RAB5B_M | Rab5b | Ras-related protein Rab-5B | | | | 0.347401 | -0.14533 | -0.93288 | 0.491373 | 0.384941 | 1.302394 | 0.206321 | 0.080439 | 0.577698 | 2.563996 | 2.71125 | 3.179255 | 2.647452 | 2.878943 | 2.873889 | 2.920254 | 2.645662 | 2.39936 | 2.657465 | 2.935195 | 2.65676 |
| P35278 | P35278 | RAB5C_M | Rab5c | Ras-related protein Rab-5C | | | | 0.829706 | 1.321763 | 2.30135 | 0.013514 | -0.02122 | -0.04335 | 0.876696 | 0.573592 | 2.462491 | -1.11773 | -1.22941 | -1.07942 | -2.04487 | -1.12697 | -0.98216 | -3.06696 | -1.92371 | -1.45962 | -1.62223 | -1.40676 | -1.84955 |
| P35279 | P35279 | RAB6A_M | Rab6a | Ras-related protein Rab-6A | | | | 0.064568 | -0.04558 | -0.19727 | 0.423721 | 0.437426 | 1.12651 | 0.114283 | -0.09914 | -0.33633 | -0.63736 | -1.06137 | 0.276425 | -0.29514 | -0.09503 | -0.67372 | -0.89572 | -0.71184 | -0.70967 | -0.18391 | -0.34156 | -0.2289 |
| P61294 | P61294 | RAB6B_M | Rab6b | Ras-related protein Rab-6B | | | + | 0.543762 | -0.20773 | -1.44255 | 0.251212 | 0.240206 | 0.691423 | 1.28169 | 0.311199 | 4.200279 | 1.719381 | 1.984713 | 3.031427 | 2.352593 | 2.57186 | 2.424796 | 2.003773 | 2.115783 | 2.377715 | 2.525893 | 2.178054 | 2.196203 |
| P51150 | P51150 | RAB7A_M | Rab7a | Ras-related protein Rab-7a | + | | | 1.162068 | -0.39277 | -3.61126 | 0.215387 | 0.38482 | 0.600798 | 0.13165 | 0.114774 | 0.383149 | 1.133039 | 1.092618 | 2.461736 | 1.187874 | 1.617472 | 1.482573 | 1.612473 | 1.398734 | 1.37232 | 1.50765 | 1.727111 | 1.143385 |
| Q922Q1 | Q922Q1 | MARC2_M | Mtarc2 | Mitochondrial amidoxime reducing component 2 | + | | | 1.475609 | -0.42894 | -5.32956 | 0.062978 | -0.10669 | -0.19267 | 0.195245 | -0.17566 | -0.54936 | -0.4761 | -0.33941 | -0.55875 | -0.54737 | -0.69344 | -0.17659 | -0.02132 | 0.063687 | 0.10736 | -1.0001 | -0.07103 | -0.44767 |
| P61028 | P61028 | RAB8B_M | Rab8b | Ras-related protein Rab-8B | | | | NaN | 0 | NaN | 0.172323 | 0.590738 | 0.490229 | 0.075817 | 0.078275 | 0.229502 | -3.78485 | -4.54974 | -1.87457 | -3.99679 | -3.21644 | -2.67644 | -3.63954 | -3.41421 | -4.09749 | -2.95534 | -2.81632 | -3.23311 |
| Q8BHH2 | Q8BHH2 | RAB9B_M | Rab9b | Ras-related protein Rab-9B | | | | 0.468255 | -0.52883 | -1.24172 | 0.122919 | 0.119086 | 0.35971 | 0.305459 | 0.170686 | 0.8276 | 0.228135 | -0.17788 | 0.082201 | -0.41017 | 0.16545 | 0.080319 | 0.92834 | 0.179567 | -0.50442 | -0.06172 | -0.2496 | 0.154 |
| A2AWA9 | A2AWA9 | RBGP1_M | Rabgap1 | Rab GTPase-activating protein 1 | | | | 0.061683 | 0.085111 | 0.188912 | 0.345774 | 1.447564 | 0.928786 | 0.03747 | -0.08171 | -0.1173 | -0.79171 | -1.04227 | -0.513 | -0.15812 | 0.372357 | -0.97641 | -0.56933 | -1.43486 | -3.33155 | -0.23471 | -0.39495 | -0.04566 |
| Q9JHK4 | Q9JHK4 | PGTA_MO | Rabggta | Geranylgeranyl transferase type-2 subunit alpha | | | | 0.087821 | -0.34509 | -0.26337 | 0.699959 | -0.69131 | -1.8886 | 0.069896 | -0.0641 | -0.2126 | -2.53733 | -2.6103 | -0.88298 | -1.49347 | -0.58921 | -0.81803 | -0.91896 | -3.5385 | -0.69893 | -0.29489 | -0.36058 | -0.91847 |
| P53612 | P53612 | PGTB2_M | Rabggtb | Geranylgeranyl transferase type-2 subunit beta | | | | 0.282861 | -0.24145 | -0.77094 | 0.609228 | -0.74701 | -1.62378 | 0.332559 | 0.225036 | 0.895577 | 0.968853 | 0.627059 | -1.17995 | -0.75744 | 0.249318 | 0.706397 | 0.776957 | 1.301851 | 0.186985 | -0.63036 | 0.357267 | 0.148375 |
| Q91X96 | Q91X96 | MSS4_MO | Rabif | Guanine nucleotide exchange factor MSS4 | | | + | 0.179094 | 0.210113 | 0.507772 | 0.347993 | 0.194842 | 0.934367 | 1.167407 | -0.15677 | -3.63602 | 0.887758 | 0.168949 | 0.879862 | 0.812333 | 0.827218 | 0.795038 | 0.113168 | 0.523312 | 0.44548 | 0.857032 | 1.007896 | 0.927896 |
| Q9D4V7 | Q9D4V7 | RABL3_M | Rabl3 | Rab-like protein 3 | | | | 0.099948 | 0.037491 | 0.297074 | 0.126667 | 0.169429 | 0.369794 | 0.881193 | -0.59427 | -2.47827 | 0.823516 | 1.070871 | 0.62945 | 1.373278 | -0.3188 | 0.01274 | 0.934809 | 0.884597 | 1.099516 | 0.564354 | 0.614506 | 0.267975 |
| Q5U3K5 | Q5U3K5 | RABL6_M | Rabl6 | Rab-like protein 6 | | | | 0.803613 | 0.620497 | 2.214707 | 0.087787 | -0.07092 | -0.26327 | 0.179374 | 0.070239 | 0.508496 | -0.34953 | 0.140129 | 0.654544 | 1.019094 | 0.952136 | 0.752568 | -0.86141 | -0.58898 | 0.70938 | 1.106105 | 0.686598 | 0.877628 |
| Q91V61 | Q91V61 | SFXN3_M | Sfxn3 | Sideroflexin-3 | + | | | 1.578121 | -0.43583 | -6.03005 | 0.115243 | 0.064695 | 0.338943 | 0.240499 | 0.113233 | 0.664412 | 1.879571 | 1.745312 | 2.442459 | 2.105194 | 3.142944 | 2.983771 | 2.221484 | 2.275054 | 2.119716 | 2.298547 | 3.100826 | 2.799422 |
| P68040 | P68040 | RACK1_M | Rack1 | Small ribosomal subunit protein RACK1 | | | | 0.745142 | 0.280318 | 2.027379 | 0.490988 | -0.72542 | -1.30138 | 0.070548 | -0.07215 | -0.21447 | 1.764854 | 1.496575 | 1.577739 | 0.617857 | 1.593044 | 2.254056 | 1.383924 | 1.316868 | 1.539709 | 2.106722 | 2.058481 | 1.932918 |
| P54728 | P54728 | RD23B_M | Rad23b | UV excision repair protein RAD23 homolog B | | | | 0.905826 | 0.519772 | 2.565865 | 0.055431 | 0.439416 | 0.170687 | 0.191667 | 0.712673 | 0.540176 | -0.79938 | -0.39899 | 0.28132 | -4.33898 | -0.82863 | -1.64617 | -1.14991 | -1.088 | -3.60436 | -1.33214 | -0.69566 | -3.20449 |
| P70388 | P70388 | RAD50_M | Rad50 | DNA repair protein RAD50 | | | | NaN | 0 | NaN | 0.298566 | 0.612003 | 0.810321 | 0.399271 | -0.73174 | -1.06405 | -3.68315 | -4.10912 | -1.30398 | -1.07509 | -2.83224 | -3.59773 | -3.74436 | -3.38666 | -2.54808 | -1.055 | -3.05458 | -1.91189 |
| Q99NG0 | Q99NG0 | ARIP4_MO | Rad54l2 | Helicase ARIP4 | | + | | 0.541603 | -0.36292 | -1.43669 | 1.95077 | -0.99383 | -9.36942 | 0.07472 | -0.14753 | -0.22638 | 0.080239 | 0.583992 | 1.006013 | 1.075496 | 0.350877 | 1.394058 | 0.714249 | 0.675821 | 2.134807 | 1.934365 | 1.410666 | 0.62932 |
| Q8C570 | Q8C570 | RAE1L_M | Rae1 | mRNA export factor | | | | 0.52425 | 0.339876 | 1.389887 | 0.239483 | 0.185219 | 0.661848 | 0.633385 | -0.93942 | -1.69263 | -1.92416 | -1.44924 | -1.01472 | -1.12395 | -2.12082 | -1.17086 | -1.96819 | -2.08497 | -1.52902 | -0.98008 | -0.99352 | -0.41933 |
| P63321 | Q9JIW9;P | RALA_MO | Rala | Ras-related protein Ral-A | | | | 0.716247 | -0.78609 | -1.93806 | 0.09395 | -0.27973 | -0.28046 | 0.159126 | 0.240502 | 0.455824 | -0.04256 | 0.478614 | 1.271453 | -0.69165 | 0.255034 | 0.527831 | 1.314948 | 0.693295 | 0.746684 | 0.392583 | 0.660615 | -0.35875 |
| Q62172 | Q62172 | RBP1_MO | Ralbp1 | RalA-binding protein 1 | | | | 0.236283 | 0.162888 | 0.653764 | 0.311966 | 0.123713 | 0.843912 | 0.399281 | -0.62967 | -1.06407 | 0.903523 | 0.533317 | 1.24232 | 1.512302 | -0.17698 | 0.877264 | 0.722309 | 0.388755 | 1.196436 | 1.310759 | 0.710894 | 1.24873 |
| Q6GYP7 | Q6GYP7 | RGPA1_M | Ralgapa1 | Ral GTPase-activating protein subunit alpha-1 | | | | 0.331216 | 0.256809 | 0.892204 | 0.278072 | 0.187177 | 0.758929 | 0.551062 | -0.81193 | -1.4624 | 0.14547 | 0.092659 | 0.883851 | 1.367783 | -0.4595 | 0.29804 | 0.148878 | -0.42437 | 0.890888 | 0.986392 | 0.325262 | 1.137146 |
| Q8BQZ4 | Q8BQZ4 | RLGPB_M | Ralgapb | Ral GTPase-activating protein subunit beta | | | | 0.322351 | 1.21724 | 0.869958 | 0.102676 | 0.115674 | 0.304593 | 0.377025 | -0.37562 | -1.0076 | -0.74869 | 0.280394 | 0.789887 | 1.44843 | -0.19721 | 0.183066 | -0.15024 | -2.75254 | 1.192699 | 0.81427 | 0.047897 | 0.68919 |
| O70435 | O70435 | PSA3_MO | Psma3 | Proteasome subunit alpha type-3 | + | | | 1.471702 | -0.4457 | -5.30439 | 0.572655 | -0.68063 | -1.52162 | 0.110212 | -0.15048 | -0.32524 | 0.570773 | 0.705146 | 0.981535 | 0.087136 | 0.821836 | 1.740077 | 1.033197 | 1.134112 | 1.224791 | 1.205147 | 1.488626 | 1.374246 |
| Q64012 | Q64012 | RALY_MO | Raly | RNA-binding protein Raly | | | | 0.143172 | -0.63413 | -0.41382 | 0.500112 | 0.614771 | 1.325507 | 0.009314 | 0.05548 | 0.030014 | -4.00739 | -1.38676 | -1.96633 | -1.04458 | -1.08827 | -4.26808 | -1.26837 | -2.8575 | -2.17223 | -2.06822 | -3.67655 | -1.79076 |
| P62827 | Q61820;P | RAN_MOU | Ran | GTP-binding nuclear protein Ran | | + | | 0.433166 | 0.344429 | 1.150776 | 1.790911 | 0.824177 | 7.764856 | 0.145058 | -0.05202 | -0.41881 | 2.610499 | 3.100939 | 3.496202 | 3.346697 | 2.501688 | 2.671627 | 2.682896 | 2.339685 | 2.672626 | 2.521919 | 2.54807 | 2.729292 |
| P34022 | P34022 | RANG_MO | Ranbp1 | Ran-specific GTPase-activating protein | | | | 0.51118 | 1.529289 | 1.354927 | 0.77973 | 1.47241 | 2.137082 | 0.491634 | 1.12093 | 1.303084 | -2.79518 | -1.56376 | -0.85288 | -1.18639 | -1.28291 | -1.34132 | -4.65471 | -2.7628 | -3.16055 | -1.82355 | -1.57333 | -3.29276 |
| Q9ERU9 | Q9ERU9 | RBP2_MO | Ranbp2 | E3 SUMO-protein ligase RanBP2 | + | + | | 1.159783 | 1.913642 | 3.600709 | 2.059486 | 1.515878 | 10.63869 | 0.376605 | -0.41339 | -1.00654 | 1.355528 | 2.242633 | 2.374085 | 2.659006 | 0.510909 | 0.810558 | -0.40733 | 0.178208 | 1.003428 | 0.997906 | 0.691722 | 1.456527 |
| Q9CT10 | Q9CT10 | RANB3_M | Ranbp3 | Ran-binding protein 3 | | | | 0.155076 | -0.19545 | -0.44521 | 0.113171 | -0.10694 | -0.33331 | 0.366009 | -0.15115 | -0.97977 | -3.34315 | -3.29505 | -1.35262 | -1.05783 | -1.12466 | -1.38707 | -3.562 | -2.6853 | -1.38327 | -0.81328 | -1.02357 | -1.18586 |
| Q8BIV3 | Q8BIV3 | RNBP6_M | Ranbp6 | Ran-binding protein 6 | | | | 0.445444 | 0.51947 | 1.18245 | 0.36326 | 0.344534 | 0.972828 | 0.009709 | -0.01235 | -0.03127 | -2.09726 | -2.50316 | -1.69975 | -1.98311 | -2.04979 | -1.82514 | -2.43005 | -3.20931 | -2.51055 | -1.86138 | -2.30364 | -1.54659 |
| P69566 | P69566 | RANB9_M | Ranbp9 | Ran-binding protein 9 | | | | NaN | 0 | NaN | 0.418056 | -0.46459 | -1.11199 | 0.126164 | 0.327456 | 0.368442 | -3.45371 | -2.86854 | -1.80475 | -1.71284 | -1.74639 | -2.21339 | -3.77925 | -3.24973 | -1.70947 | -0.87895 | -3.16489 | -1.44981 |
| P46061 | P46061 | RAGP1_M | Rangap1 | Ran GTPase-activating protein 1 | + | + | | 1.06077 | 1.367141 | 3.166171 | 1.568492 | 1.347943 | 5.96084 | 0.055479 | -0.06736 | -0.17083 | -0.00506 | 0.806939 | 1.254298 | 1.536386 | -0.09035 | -0.20318 | -0.8192 | -1.11321 | -0.12936 | 0.224155 | -0.46964 | 0.310819 |
| P62835 | P62835;Q | RAP1A_M | Rap1a | Ras-related protein Rap-1A | + | | | 1.161397 | -0.63674 | -3.60816 | 0.648937 | 0.58611 | 1.737561 | 0.078593 | 0.100929 | 0.23738 | 2.704838 | 2.652943 | 2.727972 | 2.065026 | 2.160278 | 2.753442 | 3.49018 | 3.141074 | 1.747864 | 1.872913 | 2.660588 | 2.051274 |
| A2ALS5 | A2ALS5 | RPGP1_M | Rap1gap | Rap1 GTPase-activating protein 1 | + | + | | 1.214007 | 0.814718 | 3.858005 | 1.086228 | 0.937572 | 3.273783 | 0.0767 | 0.10702 | 0.232012 | 0.15448 | 0.385334 | 2.45973 | 2.809605 | 1.546541 | 1.184401 | -0.36797 | -0.72165 | 1.470347 | 1.923844 | 0.834206 | 1.682696 |
| Q5SVL6 | Q5SVL6 | RPGP2_M | Rap1gap2 | Rap1 GTPase-activating protein 2 | | | | 0.194405 | 0.963171 | 0.547207 | 0.8509 | 0.569208 | 2.373195 | 0.528708 | 0.630171 | 1.401867 | -1.29187 | -0.70694 | 1.437053 | 1.283665 | 0.831537 | -0.00936 | -3.69827 | -0.22689 | 0.563895 | 1.018407 | -0.37812 | -0.06005 |
| E9Q912 | E9Q912 | GDS1_MO | Rap1gds1 | Rap1 GTPase-GDP dissociation stimulator 1 | + | + | | 1.051882 | -0.38888 | -3.12924 | 1.076171 | 0.478787 | 3.230946 | 0.719197 | 0.302019 | 1.947085 | 2.065095 | 1.929833 | 3.078727 | 2.819909 | 2.837606 | 2.649354 | 2.490601 | 2.282085 | 2.39833 | 2.542732 | 2.564751 | 2.318171 |
| Q80ZJ1 | Q80ZJ1;P | RAP2A_M | Rap2a | Ras-related protein Rap-2a | | | | 0.385806 | -0.42839 | -1.02984 | 0.251941 | 0.239548 | 0.69326 | 0.289826 | 0.173069 | 0.788409 | 0.602279 | 0.910212 | 1.950684 | 1.261433 | 1.724175 | 1.613677 | 0.798203 | 1.57107 | 1.34141 | 1.391611 | 1.283406 | 1.708307 |
| P61226 | P61226 | RAP2B_M | Rap2b | Ras-related protein Rap-2b | | | | 0.190407 | -0.54015 | -0.53694 | 0.039083 | 0.052438 | 0.122162 | 0.542016 | 0.555282 | 1.437808 | -3.38338 | -1.52977 | -0.01606 | -0.81872 | -0.08107 | -0.39858 | -1.52521 | -2.30763 | -0.62211 | -0.31754 | -0.44304 | -1.14717 |
| Q8BU31 | Q8BU31 | RAP2C_M | Rap2c | Ras-related protein Rap-2c | | | | 0.311771 | -0.21971 | -0.84342 | 0.044543 | 0.06249 | 0.138515 | 0.167938 | 0.302496 | 0.478828 | -1.46734 | -0.94654 | -1.33661 | -1.72153 | -0.89076 | -1.34453 | -0.99423 | -0.98023 | -1.18353 | -1.99958 | -2.00973 | -0.83055 |
| A0A5F8MF | A0A5F8MF | A0A5F8MF | Rapgef1 | Rap guanine nucleotide exchange factor (GEF) 1 | | | + | 0.425261 | 1.127299 | 1.130461 | 0.399917 | 0.241179 | 1.06569 | 1.21624 | -0.8953 | -3.86891 | -1.8053 | -0.88381 | 0.183773 | 0.072538 | -1.60314 | -1.19974 | -1.58747 | -3.35623 | -0.3324 | 0.106349 | -0.61958 | -0.39271 |
| P84084 | P84084 | ARF5_MO | Arf5 | ADP-ribosylation factor 5 | + | | | 1.362378 | -0.44918 | -4.64172 | 0.268138 | -0.01866 | -0.73399 | 0.208473 | 0.092495 | 0.583188 | 2.892135 | 3.066463 | 3.480011 | 3.429167 | 3.686213 | 3.983748 | 3.47052 | 3.386446 | 3.473176 | 3.473321 | 3.797466 | 3.687506 |
| Q9EQZ6 | Q8VCC8; | RPGF4_M | Rapgef4 | Rap guanine nucleotide exchange factor 4 | | + | | 0.005926 | 0.006352 | 0.019166 | 1.70782 | 0.729601 | 7.03806 | 0.180971 | 0.285625 | 0.512623 | -1.79489 | -1.5083 | 0.2613 | 0.143236 | -0.23375 | -1.01286 | -1.3591 | -1.95679 | -0.61255 | -0.44212 | -1.3073 | -0.51056 |
| Q8C0Q9 | Q8C0Q9 | RPGF5_M | Rapgef5 | Rap guanine nucleotide exchange factor 5 | | | + | NaN | 0 | NaN | 0.056838 | 0.232664 | 0.174805 | 1.073985 | 1.910001 | 3.221688 | -2.44959 | -3.64649 | -3.57914 | -1.21097 | -0.75953 | -1.93452 | -3.45641 | -3.05129 | -2.01985 | -3.23558 | -3.33658 | -3.17746 |
| Q5NCJ0 | Q5NCJ0 | Q5NCJ0_M | Rapgef6 | Rap guanine nucleotide exchange factor (GEF) 6 | | | + | NaN | 0 | NaN | 0.110261 | 0.48296 | 0.325379 | 1.362648 | -0.83207 | -4.64326 | -3.5501 | -3.65152 | -1.91599 | -1.01422 | -1.21646 | -1.57445 | -2.73986 | -3.38181 | -3.36223 | -0.5339 | -0.55482 | -0.57196 |
| F2Z3U3 | F2Z3U3 | F2Z3U3_M | Raph1 | Ras association (RalGDS/AF-6) and pleckstrin homology domains 1 | | + | | 0.787547 | -0.57262 | -2.16232 | 1.079424 | 0.48532 | 3.244756 | 0.279526 | 0.314373 | 0.762576 | -3.77035 | -3.35702 | -1.20735 | -1.22006 | -1.73248 | -2.03277 | -3.15666 | -2.82548 | -1.84846 | -1.54659 | -2.58093 | -1.81306 |
| Q9D0I9 | Q9D0I9 | SYRC_MO | Rars1 | Arginine--tRNA ligase, cytoplasmic | | | + | 0.047766 | 0.014311 | 0.1481 | 0.079101 | 0.077472 | 0.238817 | 2.496026 | 0.215678 | 17.65922 | -0.89423 | -0.96613 | 0.022672 | -0.14021 | 0.227159 | 0.204857 | -0.8548 | -1.03418 | -0.45025 | 0.177766 | 0.005313 | -0.00465 |
| Q3U186 | Q3U186 | SYRM_MO | Rars2 | Probable arginine--tRNA ligase, mitochondrial | | | | 0.080991 | -0.16615 | -0.24416 | 0.239378 | 0.063897 | 0.661582 | 0.005125 | -0.00539 | -0.01659 | -1.75873 | -0.84336 | -1.07214 | -1.25677 | -0.79308 | -1.22947 | -0.6313 | -1.63849 | -1.25674 | -1.19995 | -1.24674 | -0.76503 |
| E9PYG6 | E9PYG6 | E9PYG6_M | Rasa1 | RAS p21 protein activator 1 | | | | NaN | 0 | NaN | 0.453298 | -0.60554 | -1.20279 | 0.663158 | 0.618687 | 1.779087 | -3.61589 | -3.27274 | -3.40669 | -4.33318 | -2.34349 | -3.01864 | -3.01994 | -3.51146 | -3.06726 | -3.46153 | -3.38326 | -3.21624 |
| Q60790 | Q60790 | RASA3_M | Rasa3 | Ras GTPase-activating protein 3 | | | | 0.340824 | -0.41021 | -0.91634 | 0.225828 | -0.23582 | -0.6273 | 0.64582 | 0.302332 | 1.728514 | -1.15504 | -1.84835 | -1.20719 | -1.7233 | -0.63554 | -0.66506 | -1.37474 | -0.80823 | -1.50279 | -0.95605 | -0.77835 | -1.12691 |
| Q9Z268 | Q9Z268 | RASL1_M | Rasal1 | RasGAP-activating-like protein 1 | | | + | 0.550829 | -1.77334 | -1.46176 | 0.132267 | -0.10611 | -0.3848 | 1.537287 | -0.49269 | -5.74153 | -3.73702 | -3.57416 | 0.132618 | 0.171434 | -0.05742 | 0.11418 | -0.67184 | -3.09267 | -0.01693 | 0.533206 | 0.522445 | 0.519692 |
| E9PW37 | E9PW37 | E9PW37_M | Rasal2 | RAS protein activator like 2 | | | | 0.916198 | 3.769584 | 2.603346 | 0.680099 | 0.205161 | 1.829125 | 0.492613 | -0.69173 | -1.30567 | -0.7406 | 2.132204 | 2.28138 | 2.06004 | -0.03324 | 0.951351 | -2.89108 | -3.25649 | 1.947304 | 1.983794 | 0.955025 | 1.346541 |
| P27671 | P27671 | RGRF1_M | Rasgrf1 | Ras-specific guanine nucleotide-releasing factor 1 | | | | 0.251275 | 0.44224 | 0.691584 | 0.537292 | -0.46472 | -1.42502 | 0.76214 | -0.83871 | -2.0809 | -0.89518 | -0.76688 | -0.67608 | -0.10773 | -0.87475 | -0.55978 | -0.63704 | -1.90951 | -0.08718 | 0.23281 | -0.24956 | 0.492453 |
| P70392 | P70392 | RGRF2_M | Rasgrf2 | Ras-specific guanine nucleotide-releasing factor 2 | | + | | 0.47716 | 0.439401 | 1.265013 | 3.059153 | -0.84033 | -33.8292 | 0.268752 | -0.40666 | -0.73553 | 0.302584 | -0.29813 | 0.596117 | 0.642675 | -0.17498 | -0.39021 | -0.61164 | -0.26271 | 1.468397 | 1.451063 | -0.41824 | 0.666359 |
| Q9Z1S3 | Q9Z1S3 | GRP1_MO | Rasgrp1 | RAS guanyl-releasing protein 1 | | + | | NaN | 0 | NaN | 2.099827 | 1.279121 | 11.15098 | 0.049504 | -0.06083 | -0.15324 | -3.70738 | -3.69589 | 2.162465 | 2.121115 | 0.934323 | 1.37884 | -3.52902 | -3.54764 | 0.749838 | 0.9755 | 0.888534 | 1.546287 |
| Q9QUG9 | Q9QUG9 | GRP2_MO | Rasgrp2 | RAS guanyl-releasing protein 2 | | | | 0.081342 | 0.324461 | 0.245153 | 0.965542 | 0.782404 | 2.786729 | 0.166492 | 0.242896 | 0.475062 | -0.93246 | -3.18432 | -0.39823 | 0.133753 | -2.13191 | -1.18556 | -1.68719 | -3.07851 | -0.82478 | -1.0045 | -2.09535 | -1.70792 |
| Q5SSG5 | Q5SSG5 | RSLAB_M | Rasl10b | Ras-like protein family member 10B | | | | NaN | 0 | NaN | 0.013873 | -0.02108 | -0.04448 | 0.684123 | -0.72379 | -1.8411 | -3.02685 | -3.33078 | -3.30229 | -4.16052 | -3.92759 | -3.72768 | -4.15657 | -3.07064 | -3.91129 | -3.50936 | -3.48405 | -2.72363 |
| Q8BMS9 | Q8BMS9 | RASF2_M | Rassf2 | Ras association domain-containing protein 2 | | | | 0.677151 | 0.474854 | 1.820373 | 0.648264 | -0.49515 | -1.7356 | 0.444735 | -0.45407 | -1.18062 | 0.46116 | 0.400655 | -0.44507 | -0.13161 | -0.41008 | 0.357921 | 0.215149 | -0.30304 | 0.445191 | -0.03157 | 0.449481 | 0.406495 |
| Q5EBH1 | Q5EBH1 | RASF5_M | Rassf5 | Ras association domain-containing protein 5 | | | | 0.741675 | 0.228592 | 2.016551 | 0.269873 | 0.340051 | 0.738348 | 0.88127 | -0.82494 | -2.47854 | -0.90672 | -0.71975 | -1.21849 | -0.73953 | -0.30012 | -2.33955 | -1.10594 | -0.97772 | -1.71246 | -0.92567 | -1.88599 | -1.80379 |
| Q9ESK9 | Q9ESK9 | RBCC1_M | Rb1cc1 | RB1-inducible coiled-coil protein 1 | | | | 0.166145 | 0.219657 | 0.47416 | 0.75143 | 0.528933 | 2.04709 | 0.484336 | -0.4543 | -1.28385 | -0.47112 | -1.11529 | 0.594725 | 1.07846 | -0.85789 | -0.57703 | -0.67989 | -1.34582 | 0.216765 | 0.398554 | -0.58795 | 0.061646 |
| Q60972 | Q60972 | RBBP4_M | Rbbp4 | Histone-binding protein RBBP4 | + | | | 1.135216 | 0.73352 | 3.488787 | 0.085411 | -0.04816 | -0.25661 | 0.67027 | -0.81121 | -1.80002 | 0.262146 | 0.595702 | 1.443532 | 1.778319 | 0.05689 | 0.88125 | -0.43262 | -0.17657 | 1.574249 | 1.743914 | 1.098045 | 1.462522 |
| Q60973 | Q60973 | RBBP7_M | Rbbp7 | Histone-binding protein RBBP7 | | | | 0.070747 | 0.268646 | 0.215036 | 0.043886 | 0.028113 | 0.136557 | 0.339805 | -0.33852 | -0.91378 | -4.19401 | -1.7075 | -0.65369 | -0.25432 | -1.45494 | -1.03323 | -3.34218 | -3.09662 | -0.53219 | -0.43205 | -1.21017 | -0.60096 |
| Q8BP71;Q | Q8BIF2;Q | RFOX1_M | Rbfox1;Rbfox2 | RNA binding protein fox-1 homolog 2 | | | | 0.365 | 0.34624 | 0.977217 | 0.005131 | -0.00512 | -0.01661 | 0.020493 | -0.01485 | -0.06525 | -0.08277 | 0.622054 | 0.888451 | 1.498213 | 0.901508 | 0.761775 | -0.11324 | -0.03996 | 1.245267 | 1.151644 | 0.629978 | 1.062995 |
| Q8BIF2 | Q8BIF2 | RFOX3_M | Rbfox3 | RNA binding protein fox-1 homolog 3 | | | | 0.395399 | 1.200665 | 1.054199 | 0.555402 | 1.117066 | 1.474239 | 0.27791 | 0.616397 | 0.758523 | -3.58751 | -1.39487 | -0.9072 | -0.63127 | -0.96622 | -1.74527 | -4.00049 | -3.38322 | -2.63136 | -1.14124 | -2.68532 | -1.25895 |
| Q8C2Q3 | Q8C2Q3 | RBM14_M | Rbm14 | RNA-binding protein 14 | + | | | 1.021037 | 0.399141 | 3.003553 | 0.438449 | 0.255793 | 1.164387 | 0.10434 | -0.11326 | -0.30917 | 2.540149 | 2.631983 | 2.915819 | 3.319834 | 2.68818 | 2.710596 | 2.31163 | 2.062221 | 2.948361 | 2.775705 | 2.446472 | 3.17883 |
| B2RY56 | B2RY56 | RBM25_M | Rbm25 | RNA-binding protein 25 | | | | 0.395156 | 0.485117 | 1.05358 | 0.033907 | -0.05212 | -0.10651 | 0.602952 | 0.397262 | 1.606077 | -0.40618 | -0.10576 | -0.73065 | -0.46217 | 0.193051 | -0.23641 | -0.30583 | -1.17634 | -1.01487 | -0.07369 | -0.54171 | -0.29618 |
| Q8VH51 | Q8VH51 | RBM39_M | Rbm39 | RNA-binding protein 39 | + | | | 1.037447 | 1.214236 | 3.069944 | 0.65361 | 0.848928 | 1.75116 | 0.342943 | 0.628097 | 0.921667 | 0.360514 | 1.14431 | 0.845725 | 1.110433 | 0.535689 | -0.47742 | -0.40839 | -0.51526 | -0.33721 | 0.595514 | -1.05483 | -0.14309 |
| Q91W59 | Q8VC70;Q | RBMS1_M | Rbms1 | RNA-binding motif, single-stranded-interacting protein 1 | | | | 0.775346 | -0.39259 | -2.123 | 0.122227 | 0.29419 | 0.357844 | 0.509325 | 0.657823 | 1.349984 | 0.960348 | 0.591903 | -1.01289 | 0.22304 | -0.85958 | 0.027156 | 1.152635 | 1.184801 | -0.1469 | -1.23133 | -0.87188 | -1.27619 |
| Q91VM5 | Q91VM5;Q | RMXL1_M | Rbmxl1 | RNA binding motif protein, X-linked-like-1 | | | | 0.97446 | 0.731924 | 2.820794 | 0.521333 | 0.975291 | 1.382063 | 0.24142 | 0.443645 | 0.666738 | 0.076507 | -0.00739 | -0.73505 | 0.662763 | 0.07991 | -0.98761 | -0.44131 | -0.95343 | -0.9139 | -1.10896 | -1.2948 | -0.50019 |
| Q91YT0 | Q91YT0 | NDUV1_M | Ndufv1 | NADH dehydrogenase [ubiquinone] flavoprotein 1, mitochondrial | + | | | 2.140072 | -0.46824 | -11.686 | 0.463315 | -0.34656 | -1.22883 | 0.02405 | 0.024764 | 0.076297 | 2.35846 | 2.434056 | 1.980906 | 1.439754 | 2.109868 | 2.44714 | 2.877799 | 2.851204 | 2.136426 | 1.977354 | 2.531072 | 1.976408 |
| Q9JHG2 | Q9JHG2 | RCAN2_M | Rcan2 | Calcipressin-2 | + | + | + | 1.285474 | 2.352762 | 4.220141 | 1.382945 | 0.996096 | 4.760477 | 1.494486 | 0.32864 | 5.452705 | -1.00377 | 0.075349 | -0.14813 | 0.076715 | -1.63968 | -1.59999 | -2.67666 | -2.95729 | -1.20828 | -0.85533 | -2.00539 | -1.89157 |
| Q61753 | Q61753 | SERA_MO | Phgdh | D-3-phosphoglycerate dehydrogenase | + | | | 1.485457 | -0.48471 | -5.39348 | 0.259148 | -0.19306 | -0.7114 | 0.040444 | -0.01925 | -0.12625 | 1.828916 | 1.879317 | 1.814346 | 1.274995 | 1.846624 | 1.727356 | 2.425093 | 2.252564 | 1.768137 | 1.707326 | 1.946576 | 1.665907 |
| Q8BK67 | Q8BK67 | RCC2_MO | Rcc2 | Protein RCC2 | | | | 0.4413 | 1.526331 | 1.171744 | 0.00116 | 0.001808 | 0.003774 | 0.289069 | -0.15173 | -0.78651 | -0.59115 | -0.86165 | -0.32108 | -0.75569 | -0.41035 | -0.52841 | -3.54831 | -0.95716 | -0.96713 | -0.11326 | -0.50131 | -0.13399 |
| Q8BP92 | Q8BP92 | RCN2_MO | Rcn2 | Reticulocalbin-2 | | | | 0.399869 | 1.180846 | 1.065569 | 0.548203 | 1.747723 | 1.454612 | 0.050929 | -0.27221 | -0.15745 | -0.79464 | -1.15387 | -0.15319 | -0.60188 | -0.82224 | -3.55592 | -1.06157 | -3.24863 | -3.30563 | -0.94488 | -2.97545 | -0.85829 |
| Q9QYF1 | Q9QYF1 | RDH11_M | Rdh11 | Retinol dehydrogenase 11 | | | | 0.897727 | -0.53345 | -2.53685 | 0.146304 | 0.208592 | 0.422103 | 0.312316 | 0.27482 | 0.84479 | -0.76982 | -0.5815 | -1.23675 | -1.33889 | -1.52416 | -0.89808 | 0.045813 | -0.33023 | -1.00489 | -1.98794 | -1.39743 | -1.57444 |
| Q9ERI6 | Q9ERI6 | RDH14_M | Rdh14 | Retinol dehydrogenase 14 | | | | 0.528769 | -0.33582 | -1.40203 | 0.167855 | -0.29059 | -0.47861 | 0.106681 | -0.11355 | -0.31559 | -0.61651 | -0.84592 | -0.77687 | -1.50071 | -0.49094 | -0.03765 | -0.60567 | -0.18513 | -0.36072 | -1.33568 | 0.128726 | -0.43021 |
| Q8VCD6 | Q8VCD6 | REEP2_M | Reep2 | Receptor expression-enhancing protein 2 | | | | 0.178292 | 0.046057 | 0.505697 | 0.126985 | 0.135154 | 0.370648 | 0.761381 | 0.356364 | 2.078494 | 0.190958 | 0.191599 | 0.006228 | -0.69806 | 0.155989 | -0.08667 | 0.054148 | 0.236297 | -0.38643 | -0.57572 | -0.20056 | -0.44285 |
| P12787 | P12787 | COX5A_M | Cox5a | Cytochrome c oxidase subunit 5A, mitochondrial | + | | | 1.677009 | -0.49194 | -6.78527 | 0.09346 | -0.08144 | -0.2791 | 0.080928 | 0.144846 | 0.243986 | 4.319376 | 4.278376 | 3.516005 | 3.071854 | 3.292695 | 3.78069 | 4.860363 | 4.721277 | 3.564653 | 3.186088 | 3.933052 | 2.850641 |
| Q148V7 | Q148V7 | RELCH_M | Relch | RAB11-binding protein RELCH | | | | 0.424896 | 1.257264 | 1.129523 | 0.206458 | 0.215245 | 0.578048 | 0.05358 | 0.074517 | 0.16526 | -2.62801 | -1.47533 | -0.8528 | -1.34556 | -0.91103 | -1.38707 | -4.26119 | -2.35667 | -1.59362 | -1.03523 | -1.60653 | -0.8406 |
| Q8BRJ3 | Q8BRJ3 | RELL2_M | Rell2 | RELT-like protein 2 | | | | NaN | 0 | NaN | NaN | 0 | NaN | 0.6599 | 0.581143 | 1.769537 | -3.16273 | -2.94227 | -3.63627 | -2.83014 | -3.06446 | -2.87034 | -3.00103 | -2.343 | -3.86502 | -3.42941 | -3.86229 | -3.2348 |
| Q8VEL9 | Q8VEL9 | REM2_MO | Rem2 | GTP-binding protein REM 2 | | | | 0.427043 | -1.087 | -1.13503 | 0.281777 | 0.157037 | 0.768224 | 0.186581 | 0.060977 | 0.527095 | -3.12364 | -3.83806 | 0.675881 | 1.074028 | 1.626111 | 1.854393 | -3.28242 | -1.50528 | 0.671493 | 0.764343 | 1.660436 | 1.698114 |
| O54916 | O54916 | REPS1_M | Reps1 | RalBP1-associated Eps domain-containing protein 1 | | | | 0.05625 | 0.032833 | 0.173084 | 0.123104 | 0.085439 | 0.360209 | 0.305505 | -0.5849 | -0.82771 | 0.659029 | 0.42469 | 0.668594 | 0.911943 | -0.68792 | 0.724769 | 0.65821 | 0.359843 | 0.501223 | 0.908436 | 0.624151 | 0.58251 |
| Q80XA6 | Q80XA6 | REPS2_M | Reps2 | RalBP1-associated Eps domain-containing protein 2 | | | | 0.040983 | 0.02608 | 0.12787 | 0.134287 | 0.111208 | 0.390194 | 0.433346 | -0.57678 | -1.15124 | 0.215745 | 0.119406 | 0.74184 | 1.279531 | -0.30779 | 0.683143 | 0.339685 | -0.05669 | 0.80487 | 0.994085 | 0.690166 | 0.838741 |
| Q8C3B8 | Q8C3B8 | RFT1_MO | Rft1 | Protein RFT1 homolog | | | | 0.277028 | 0.268551 | 0.756309 | 0.415893 | 0.086166 | 1.106457 | 0.186981 | -0.09901 | -0.52812 | -0.38567 | -0.14792 | -1.04533 | -1.09456 | -0.94515 | -0.99658 | -0.86994 | -0.20076 | -1.22999 | -1.08223 | -0.68616 | -1.05755 |
| Q8CHX7 | Q8CHX7 | RFTN2_M | Rftn2 | Raftlin-2 | | + | | 0.984086 | 1.209781 | 2.857892 | 2.281561 | -0.43029 | -13.7744 | 0.319784 | -0.30208 | -0.86352 | -0.92601 | -1.13281 | -1.86476 | -1.82332 | -1.42388 | -0.96807 | -2.64968 | -1.8287 | -1.39037 | -1.43713 | -1.15929 | -0.62851 |
| P97492 | P97492 | RGS14_M | Rgs14 | Regulator of G-protein signaling 14 | | | | 0.360732 | -0.284 | -0.96645 | 0.22989 | 0.130644 | 0.637594 | 0.359232 | 0.291645 | 0.962671 | -3.5136 | -3.18682 | -0.60347 | -0.35164 | 0.63369 | 0.441997 | -2.82195 | -3.31046 | -0.76985 | -0.44655 | -0.04119 | 0.533591 |
| Q9Z2H2 | Q9Z2H2 | RGS6_MO | Rgs6 | Regulator of G-protein signaling 6 | | | | NaN | 0 | NaN | 0.785798 | -0.80018 | -2.15666 | 0.111417 | 0.215943 | 0.328532 | -2.79245 | -3.39275 | -1.5062 | -2.05815 | -2.74053 | -4.05172 | -2.95287 | -3.3548 | -1.22998 | -3.73401 | -3.65934 | -3.56479 |
| O54829 | O54829;Q | RGS7_MO | Rgs7 | Regulator of G-protein signaling 7 | | | | 0.910541 | 0.44271 | 2.582862 | 0.177248 | -0.13217 | -0.503 | 0.936038 | -0.23772 | -2.67606 | 0.017551 | -0.22492 | 1.369162 | 1.167056 | 1.032252 | 0.921453 | -0.66756 | -0.42523 | 1.157721 | 1.642838 | 1.145134 | 1.284019 |
| Q8BQP9 | Q8BQP9 | R7BP_MO | Rgs7bp | Regulator of G-protein signaling 7-binding protein | | | | NaN | 0 | NaN | 0.49918 | 0.208068 | 1.323037 | 0.183091 | 0.053807 | 0.518097 | -3.35615 | -4.29143 | -0.96181 | -0.78225 | -1.58391 | -1.64653 | -3.44663 | -2.79214 | -1.20922 | -0.95098 | -1.76805 | -1.57 |
| O54828 | O54828 | RGS9_MO | Rgs9 | Regulator of G-protein signaling 9 | | + | | NaN | 0 | NaN | 1.45612 | 1.991847 | 5.205066 | 0.111959 | -0.22436 | -0.33001 | -3.5121 | -3.76561 | 0.17939 | 0.849297 | -2.61044 | -2.86711 | -3.00125 | -2.52439 | -1.66256 | -1.29245 | -3.18205 | -1.84677 |
| Q6PIX5 | Q6PIX5 | RHDF1_M | Rhbdf1 | Inactive rhomboid protein 1 | | | | 0.458685 | -0.25163 | -1.21678 | 0.182309 | 0.089036 | 0.516079 | 0.765054 | -0.55099 | -2.09015 | 0.086093 | -0.29524 | 1.479555 | 1.823654 | 0.505281 | 1.004884 | 0.22713 | 0.066982 | 1.575344 | 1.549794 | 1.221865 | 1.390286 |
| Q921J2 | Q921J2 | RHEB_MO | Rheb | GTP-binding protein Rheb | | | | 0.582596 | -0.29969 | -1.54916 | 0.205638 | 0.173002 | 0.575956 | 0.298899 | 0.155144 | 0.811155 | -0.68946 | -0.36055 | 0.548547 | -0.00692 | 0.271821 | 0.518428 | -0.12342 | -0.3272 | 0.21221 | -0.01659 | 0.386192 | 0.093768 |
| Q9QUI0 | Q9QUI0;Q | RHOA_MO | Rhoa | Transforming protein RhoA | | | | 0.712954 | -0.34071 | -1.92801 | 0.275076 | 0.428302 | 0.751411 | 0.142866 | -0.09994 | -0.413 | 0.200469 | 0.552193 | 1.83914 | 0.731173 | 0.243925 | 0.684974 | 0.699702 | 0.734374 | 0.722701 | 0.991009 | 0.664039 | 0.464748 |
| P62746 | Q9QUI0;Q | RHOB_MO | Rhob | Rho-related GTP-binding protein RhoB | | | | 0.137974 | -0.04866 | -0.40002 | 0.531542 | 0.544295 | 1.409497 | 0.765255 | 0.312091 | 2.090792 | 0.9291 | 0.785167 | 1.963357 | 1.191161 | 1.014059 | 1.267755 | 0.807718 | 1.00387 | 1.02591 | 1.040019 | 0.750134 | 0.907498 |
| Q8BYP3 | Q8BYP3 | RHOF_MO | Rhof | Rho-related GTP-binding protein RhoF | | | | 0.554147 | 2.012257 | 1.470812 | 0.019672 | -0.01758 | -0.06269 | 0.065205 | -0.04542 | -0.1991 | 1.058083 | -0.88637 | 0.710218 | 0.292506 | 0.689705 | 0.462286 | -2.88897 | -0.96383 | 0.706139 | 0.331751 | 0.423652 | 0.819179 |
| P84096 | P84096 | RHOG_M | Rhog | Rho-related GTP-binding protein RhoG | | | | 0.135252 | -0.08981 | -0.39277 | 0.276507 | -0.18742 | -0.755 | 0.177943 | 0.13416 | 0.504794 | 1.703202 | 1.506422 | 0.368021 | 0.016376 | 0.621088 | 0.550718 | 1.901013 | 1.488222 | 0.554849 | 0.204383 | 0.18831 | 0.715176 |
| Q8BG51 | Q8BG51 | MIRO1_M | Rhot1 | Mitochondrial Rho GTPase 1 | | | | 0.563188 | 0.230312 | 1.495563 | 0.773766 | -0.2757 | -2.11794 | 0.227092 | 0.187668 | 0.63051 | 0.992383 | 0.97745 | 1.034983 | 0.787786 | 1.544621 | 0.978281 | 0.908421 | 0.60079 | 1.227939 | 1.146236 | 0.982092 | 1.165475 |
| Q8JZN7 | Q8JZN7 | MIRO2_M | Rhot2 | Mitochondrial Rho GTPase 2 | | | | 0.026012 | 0.011664 | 0.082358 | 0.367585 | -0.30595 | -0.98374 | 0.093167 | -0.12984 | -0.27829 | 0.299658 | 0.397793 | -0.74784 | -0.42291 | 0.45721 | -0.46282 | 0.469913 | 0.204212 | -0.54462 | -0.01423 | 0.049164 | 0.204897 |
| Q9D0B8 | Q9D0B8 | RIBC1_M | Ribc1 | RIB43A-like with coiled-coils protein 1 | | | | NaN | 0 | NaN | 0.672908 | 0.657976 | 1.807808 | 0.296024 | -0.79523 | -0.80395 | -3.27772 | -3.29737 | -0.46448 | 0.06984 | -3.38785 | -3.38447 | -3.60445 | -3.62459 | -0.60813 | -1.10247 | -3.58009 | -1.60178 |
| Q69ZJ7 | Q69ZJ7 | RIC1_MO | Ric1 | Guanine nucleotide exchange factor subunit RIC1 | | | | 0.273128 | 1.145837 | 0.74652 | 0.410308 | -0.36651 | -1.09218 | 0.38039 | -0.69317 | -1.01612 | -3.54613 | -0.48912 | 0.763309 | 1.189242 | -1.58615 | -0.62755 | -3.02344 | -3.30349 | 1.602129 | 1.083447 | -0.8991 | 0.071731 |
| Q3TIR3 | Q3TIR3 | RIC8A_M | Ric8a | Synembryn-A | | | | 0.366261 | -1.09107 | -0.9804 | 0.801212 | -0.54408 | -2.20683 | 0.007259 | 0.005041 | 0.023445 | -3.3635 | -1.25963 | -1.46887 | -1.92084 | -1.06653 | -1.36063 | -0.85726 | -1.58373 | -1.24934 | -1.0522 | -1.37548 | -1.06176 |
| Q6QI06 | Q6QI06 | RICTR_M | Rictor | Rapamycin-insensitive companion of mTOR | | | | 0.060614 | -0.17763 | -0.18581 | 0.333997 | 0.294375 | 0.899187 | 0.349196 | -0.37816 | -0.9374 | -2.26313 | -1.82682 | -0.64541 | -0.34573 | -1.30754 | -1.41367 | -2.79809 | -0.9366 | -1.08102 | -0.49887 | -1.38236 | -0.58253 |
| Q80U40 | Q80U40 | RIMB2_M | Rimbp2 | RIMS-binding protein 2 | | | | 0.661847 | 0.664557 | 1.775241 | 0.589992 | 0.43968 | 1.56975 | 0.391878 | 0.345283 | 1.045252 | 0.454271 | 0.098018 | 0.741264 | 1.028562 | 1.38039 | 0.759215 | -0.05916 | -0.71766 | 0.685688 | 0.204777 | 0.61202 | 0.83702 |
| Q8BR90 | Q8BR90 | RIMC1_M | Rimoc1 | RAB7A-interacting MON1-CCZ1 complex subunit 1 | | | | 0.240488 | -0.10771 | -0.66438 | 0.02239 | 0.048978 | 0.071153 | 0.328731 | -0.40331 | -0.88597 | -3.44611 | -3.53842 | -1.8315 | -2.19588 | -1.70712 | -2.56479 | -3.22914 | -3.53997 | -2.72646 | -1.39887 | -1.5799 | -1.88539 |
| Q99NE5 | Q99NE5 | RIMS1_M | Rims1 | Regulating synaptic membrane exocytosis protein 1 | | + | | 0.62 | 0.502873 | 1.654339 | 1.033031 | -0.42218 | -3.05197 | 0.726185 | -0.15678 | -1.96855 | 0.701829 | 1.302448 | 2.412465 | 2.48529 | 2.431261 | 2.45544 | 0.546313 | 0.452218 | 2.737606 | 3.004509 | 2.521413 | 2.678856 |
| Q9EQZ7 | Q9EQZ7 | RIMS2_M | Rims2 | Regulating synaptic membrane exocytosis protein 2 | + | | | 1.18016 | 0.401323 | 3.695709 | 0.749775 | -0.52948 | -2.04189 | 0.784343 | -0.24871 | -2.15196 | 0.594504 | 0.417735 | 0.350338 | 0.836643 | 0.19566 | 0.399838 | 0.167886 | 0.041707 | 1.032872 | 1.213071 | 0.492282 | 0.600629 |
| Q80U57 | Q80U57 | RIMS3_M | Rims3 | Regulating synaptic membrane exocytosis protein 3 | | + | | 0.892901 | 0.885085 | 2.519658 | 1.033268 | -0.32973 | -3.05293 | 0.121403 | 0.094108 | 0.35562 | -0.30893 | -1.01097 | -0.19696 | -0.35856 | -0.64466 | -0.18705 | -1.5583 | -1.53177 | 0.123638 | -0.01969 | -0.377 | -0.64292 |
| P60191 | P60191 | RIMS4_M | Rims4 | Regulating synaptic membrane exocytosis protein 4 | | | | NaN | 0 | NaN | 0.683309 | 0.381681 | 1.83868 | 0.035557 | 0.040476 | 0.111519 | -3.04226 | -3.68801 | -1.62187 | -1.21932 | -3.20484 | -2.70161 | -4.39327 | -3.68708 | -1.85307 | -1.75149 | -2.73212 | -3.25528 |
| Q921Q7 | Q921Q7 | RIN1_MO | Rin1 | Ras and Rab interactor 1 | | | | NaN | 0 | NaN | 0.605049 | 0.890701 | 1.611984 | 0.216601 | 0.30603 | 0.603884 | -3.99969 | -3.08076 | 1.851567 | 2.700667 | 1.019694 | 2.030764 | -3.2821 | -3.51067 | 1.031766 | 1.739066 | 1.254559 | 1.18384 |
| O35730 | O35730 | RING1_M | Ring1 | E3 ubiquitin-protein ligase RING1 | + | + | + | 1.268604 | 1.9923 | 4.132186 | 1.071275 | -0.23247 | -3.21024 | 1.2929 | -0.31187 | -4.25936 | -1.71671 | -1.01909 | -1.4628 | -1.38706 | -2.14677 | -2.03224 | -3.69305 | -3.02734 | -1.13074 | -1.25419 | -1.73201 | -1.82326 |
| Q68FE6 | Q68FE6 | RIPR1_M | Ripor1 | Rho family-interacting cell polarization regulator 1 | + | | | 1.045276 | 1.053264 | 3.101998 | 0.164258 | -0.0718 | -0.46924 | 0.068983 | 0.067481 | 0.209977 | -1.61404 | -1.87017 | -1.08659 | -1.35621 | -1.06 | -1.695 | -3.10983 | -2.4809 | -1.22199 | -1.07721 | -1.49469 | -1.39527 |
| P70425 | P70425 | RIT2_MO | Rit2 | GTP-binding protein Rit2 | + | | | 1.015435 | 1.761063 | 2.98113 | 0.304186 | -0.18486 | -0.82441 | 0.36877 | -0.21598 | -0.98674 | -0.72166 | -1.43121 | -0.76746 | -1.13943 | -1.16926 | -1.60468 | -3.30984 | -2.36516 | -0.64333 | -0.89385 | -1.19363 | -1.14835 |
| Q80YQ8 | Q80YQ8 | RMD5A_M | Rmnd5a | E3 ubiquitin-protein ligase RMND5A | | | | NaN | 0 | NaN | 0.194453 | 0.487082 | 0.547329 | 0.625551 | -0.32419 | -1.67017 | -3.55718 | -3.10036 | -2.29615 | -2.2187 | -2.55361 | -2.93 | -3.28553 | -3.39438 | -3.63359 | -1.85543 | -2.46514 | -2.37009 |
| Q99ML9 | Q99ML9 | RN111_M | Rnf111 | E3 ubiquitin-protein ligase Arkadia | | | | 0.113044 | 0.094419 | 0.332964 | 0.127885 | -0.12669 | -0.37307 | 0.110955 | 0.125073 | 0.327273 | -3.18434 | -3.34705 | -2.08756 | -2.53956 | -2.59133 | -2.29307 | -3.63177 | -3.08847 | -2.44033 | -1.93342 | -2.2154 | -2.91914 |
| Q91YL2 | Q91YL2 | RN126_M | Rnf126 | E3 ubiquitin-protein ligase RNF126 | | + | | 0.299286 | -0.86948 | -0.81212 | 1.124983 | 0.657291 | 3.442987 | 0.173124 | -0.20142 | -0.49231 | -1.11901 | -3.19531 | 0.044111 | 0.282485 | -0.75651 | -0.64064 | -1.02599 | -1.54938 | -0.64312 | -0.34486 | -0.09214 | -0.90217 |
| Q9JI90 | Q9JI90 | RNF14_M | Rnf14 | E3 ubiquitin-protein ligase RNF14 | | + | | 0.364109 | 0.336849 | 0.97497 | 1.607101 | -0.22413 | -6.24286 | 0.66842 | -0.27305 | -1.79456 | -0.75072 | -1.42644 | -1.23455 | -1.20893 | -1.24731 | -1.35492 | -1.35318 | -1.49768 | -1.03115 | -0.96407 | -0.88575 | -1.17039 |
| Q99MB7 | Q99MB7 | RN141_M | Rnf141 | RING finger protein 141 | | | | 0.25176 | -0.87344 | -0.6928 | 0.399175 | -1.54239 | -1.0638 | 0.279637 | -0.23943 | -0.76286 | -0.62294 | -2.74156 | -0.92679 | -3.81805 | -0.24134 | -0.15791 | -1.4924 | -0.12522 | -0.94097 | -0.71909 | 0.350884 | -0.27127 |
| Q9CQJ4 | Q9CQJ4 | RING2_M | Rnf2 | E3 ubiquitin-protein ligase RING2 | + | | | 1.233068 | 1.970251 | 3.951947 | 0.1042 | -0.13943 | -0.30878 | 0.524179 | -0.50854 | -1.3897 | -1.43964 | -0.62073 | -0.10177 | 0.070992 | -1.00356 | -0.86791 | -2.716 | -3.28486 | -0.31917 | 0.567247 | -0.78679 | -0.0676 |
| Q8BFU3 | Q8BFU3 | RN214_M | Rnf214 | RING finger protein 214 | | + | | 0.10815 | 0.164897 | 0.319609 | 1.570431 | -0.18695 | -5.97471 | 0.208004 | -0.2642 | -0.58199 | -0.18159 | -0.11673 | 0.441171 | 0.496223 | -0.76127 | 0.138271 | -0.82897 | 0.200857 | 0.670523 | 0.640767 | -0.10884 | 0.014245 |
| Q9WTZ1 | Q9WTZ1; | RBX2_MO | Rnf7 | RING-box protein 2 | | | | 0.374134 | -0.32071 | -1.00029 | 0.044828 | -0.02829 | -0.13937 | 0.816366 | -0.21799 | -2.25681 | -0.74869 | -0.22533 | 0.607449 | 0.964896 | 0.743969 | 0.710712 | 0.018941 | -0.35155 | 0.910708 | 0.718217 | 0.85018 | 1.040479 |
| Q91VI7 | Q91VI7 | RINI_MO | Rnh1 | Ribonuclease inhibitor | + | + | | 1.259971 | -0.34697 | -4.08778 | 1.120304 | 0.181866 | 3.422206 | 0.06416 | 0.058255 | 0.196085 | -0.06451 | -0.09788 | 0.067149 | -0.03854 | -0.19647 | 0.353973 | 0.349005 | 0.182557 | -0.1732 | -0.16192 | 0.132369 | -0.09138 |
| Q9D0L8 | Q9D0L8 | MCES_MO | Rnmt | mRNA cap guanine-N7 methyltransferase | | | | 0.612939 | -0.26093 | -1.63428 | 0.923376 | -0.4224 | -2.6295 | 0.2043 | -0.1674 | -0.57254 | -0.59095 | -0.87359 | 0.127112 | 0.190822 | 0.356771 | 0.139562 | -0.54564 | -0.39704 | 0.42392 | 0.738821 | 0.687033 | 0.144101 |
| Q8VCT3 | Q8VCT3 | AMPB_MO | Rnpep | Aminopeptidase B | + | | | 1.16492 | -1.935 | -3.62447 | 0.453608 | -1.32289 | -1.2036 | 0.074778 | -0.16891 | -0.22654 | -2.64971 | -3.55916 | -1.62623 | -3.66392 | -1.54636 | -0.8591 | -0.88971 | -1.44915 | -1.73451 | -0.90988 | -0.37211 | -1.69553 |
| Q99M28 | Q99M28 | RNPS1_M | Rnps1 | RNA-binding protein with serine-rich domain 1 | + | | + | 1.238271 | 1.069284 | 3.977916 | 0.485588 | 0.190173 | 1.287149 | 1.212764 | 0.595601 | 3.851939 | 0.885246 | 1.073534 | 0.836016 | 0.812836 | 1.542567 | 1.297968 | -0.34167 | 0.161886 | 0.486961 | 0.781545 | 0.919278 | 0.730055 |
| Q3TDK6 | Q3TDK6 | ROGDI_M | Rogdi | Protein rogdi homolog | + | | | 1.243508 | -0.17044 | -4.0042 | 0.028033 | -0.02515 | -0.08857 | 0.017988 | 0.012323 | 0.057426 | 0.589424 | 0.519892 | 1.824619 | 1.742465 | 2.037449 | 2.251183 | 0.749662 | 0.700541 | 1.527748 | 2.089634 | 1.945914 | 2.318072 |
| P97762 | P97762 | RP9_MOU | rp9 | Retinitis pigmentosa 9 protein homolog | | | | 0.634017 | 2.072044 | 1.694441 | 0.102877 | 0.125858 | 0.305146 | 0.337173 | 0.854366 | 0.907165 | -2.51482 | -0.06913 | -1.21419 | -0.38951 | -0.88402 | -1.57663 | -3.35975 | -3.36829 | -0.91812 | -0.9373 | -2.96051 | -1.20888 |
| P47708 | P47708 | RP3A_MO | Rph3a | Rabphilin-3A | | | | 0.307048 | -0.11904 | -0.83158 | 0.074271 | 0.024332 | 0.2251 | 0.625296 | 0.724577 | 1.669445 | -0.04197 | 0.125235 | 0.286937 | 0.100698 | 1.092341 | 0.235668 | 0.276874 | 0.044474 | 0.114591 | 0.224379 | 0.009451 | -0.1306 |
| Q9CQJ8 | Q9CQJ8 | NDUB9_M | Ndufb9 | NADH dehydrogenase [ubiquinone] 1 beta subcomplex subunit 9 | + | | | 1.989526 | -0.49392 | -9.80407 | 0.537921 | -0.34938 | -1.42672 | 0.032407 | 0.076781 | 0.10195 | 1.168151 | 1.103476 | 0.689826 | 0.384616 | 0.376903 | 1.840408 | 1.668361 | 1.5911 | 1.078113 | 0.695081 | 1.209986 | 0.853764 |
| O55026 | O55026 | ENTP2_M | Entpd2 | Ectonucleoside triphosphate diphosphohydrolase 2 | + | | | 1.751018 | -0.4998 | -7.40747 | 0.184843 | -0.66723 | -0.52261 | 0.136287 | 0.494943 | 0.395528 | -1.86985 | -1.8801 | -1.52895 | -3.97279 | -2.17289 | -0.80086 | -1.30791 | -1.44246 | -2.45365 | -1.71364 | -0.93527 | -3.02836 |
| Q69ZS6 | Q69ZS6 | SV2C_MO | Sv2c | Synaptic vesicle glycoprotein 2C | + | | | 1.419639 | -0.50138 | -4.97901 | 0.977281 | 1.42631 | 2.83163 | 0.361475 | 1.21146 | 0.968325 | 0.875883 | 0.697113 | 0.193799 | 0.311175 | -0.55587 | -0.2157 | 1.241506 | 1.334258 | -1.6741 | -0.67355 | -2.83672 | -0.35777 |
| P35979 | P35979 | RL12_MO | Rpl12 | Large ribosomal subunit protein uL11 | | + | | 0.750342 | 0.333145 | 2.043673 | 1.118635 | 0.323063 | 3.414817 | 0.324868 | -0.14071 | -0.87627 | 2.286808 | 2.200808 | 2.728719 | 2.817406 | 1.933839 | 1.840971 | 2.067903 | 1.753424 | 2.366429 | 2.53357 | 1.874397 | 2.181832 |
| Q9JIG8 | Q9JIG8 | PRAF2_M | Praf2 | PRA1 family protein 2 | + | | | 1.565774 | -0.50575 | -5.94144 | 0.162264 | 0.151969 | 0.464032 | 0.196968 | -0.08791 | -0.55378 | 1.516666 | 1.348385 | 0.932189 | 0.277839 | 0.661309 | 0.859616 | 1.925382 | 1.951171 | 0.438547 | 0.467543 | 0.972356 | 0.724397 |
| P19253 | P19253 | RL13A_M | Rpl13a | Large ribosomal subunit protein uL13 | | | | 0.089181 | -0.07399 | -0.26717 | 0.070747 | -0.07602 | -0.21504 | 0.076343 | 0.067026 | 0.230998 | 1.526872 | 1.63396 | 1.685439 | 1.520953 | 1.376911 | 1.039049 | 1.926116 | 1.382695 | 2.023031 | 1.335398 | 1.376865 | 0.905043 |
| Q61885 | Q61885 | MOG_MO | Mog | Myelin-oligodendrocyte glycoprotein | + | | | 1.978769 | -0.51309 | -9.68151 | 0.31275 | -0.30836 | -0.84588 | 0.317561 | 0.32663 | 0.857941 | 5.929192 | 5.916147 | 3.021592 | 2.3531 | 4.189663 | 4.015662 | 6.488348 | 6.383161 | 2.850209 | 3.141194 | 4.146672 | 3.405393 |
| Q9CZM2 | Q9CZM2 | RL15_MO | Rpl15 | Large ribosomal subunit protein eL15 | + | + | | 1.129445 | 0.972748 | 3.462899 | 1.111315 | 0.460864 | 3.382555 | 0.450154 | 0.413318 | 1.194642 | 2.697649 | 2.687391 | 3.018135 | 3.259069 | 2.836202 | 2.474281 | 2.000631 | 1.438914 | 2.741385 | 2.614091 | 1.947044 | 2.536801 |
| Q9CPR4 | Q9CPR4 | RL17_MO | Rpl17 | Large ribosomal subunit protein uL22 | | | | 0.432072 | 0.389189 | 1.147962 | 0.413832 | 0.729094 | 1.101187 | 0.492705 | 0.302021 | 1.305911 | 1.637424 | 1.737379 | 2.188726 | 2.456594 | 2.228845 | 2.11619 | 1.633534 | 0.96289 | 0.945156 | 2.241976 | 1.646189 | 2.094805 |
| Q80YN3 | Q80YN3 | BCAS1_M | Bcas1 | Breast carcinoma-amplified sequence 1 homolog | + | + | | 2.405927 | -0.5163 | -15.9104 | 1.064799 | -0.4596 | -3.18302 | 0.177859 | -0.18887 | -0.50458 | 2.805073 | 2.741319 | 1.394892 | 1.674325 | 1.656749 | 2.395174 | 3.28342 | 3.295563 | 2.030651 | 1.957765 | 2.276369 | 2.153285 |
| P62717 | P62717 | RL18A_M | Rpl18a | Large ribosomal subunit protein eL20 | | + | | 0.842083 | 1.071631 | 2.343144 | 1.868794 | 0.395795 | 8.51051 | 0.051019 | 0.035746 | 0.157719 | 0.874162 | 0.935131 | 1.356798 | 1.433425 | 0.809293 | 0.630858 | 0.289346 | -0.62332 | 1.025678 | 0.972955 | 0.475982 | 0.892676 |
| P84099 | P84099 | RL19_MO | Rpl19 | Large ribosomal subunit protein eL19 | | + | | 0.94871 | 0.766286 | 2.723211 | 1.701864 | 0.422361 | 6.988515 | 0.928307 | 0.278142 | 2.647566 | 2.904879 | 3.209501 | 3.312157 | 3.431076 | 2.951303 | 2.896891 | 2.054298 | 2.527509 | 2.960079 | 2.938433 | 2.747427 | 2.544483 |
| P70704 | P70704 | AT8A1_M | Atp8a1 | Phospholipid-transporting ATPase IA | + | | | 1.897009 | -0.52102 | -8.79721 | 0.097181 | -0.13466 | -0.28942 | 0.468028 | 0.552916 | 1.241124 | 0.169019 | 0.210672 | 0.755915 | -0.16679 | 1.366901 | 1.810491 | 0.655422 | 0.766308 | 0.368915 | 0.489534 | 1.42214 | 0.64942 |
| P67984 | P67984 | RL22_MO | Rpl22 | Large ribosomal subunit protein eL22 | | | | 0.541184 | 0.329302 | 1.435553 | 0.621099 | 0.225332 | 1.657468 | 0.435943 | 0.363151 | 1.157928 | 1.605828 | 2.021809 | 1.877443 | 1.636778 | 1.557992 | 2.039585 | 1.581263 | 1.38777 | 1.468513 | 1.595043 | 1.234701 | 1.636574 |
| Q9D7S7 | Q9D7S7 | RL22L_M | Rpl22l1 | Large ribosomal subunit protein eL22-like 1 | | + | | 0.429532 | -1.72705 | -1.14143 | 1.510581 | 0.790755 | 5.559722 | 0.049359 | 0.081257 | 0.152816 | -3.95592 | -3.23845 | -2.21484 | -2.38193 | -2.7445 | -2.82477 | -0.40021 | -3.34005 | -3.20425 | -2.97404 | -3.3961 | -2.33568 |
| P62830 | P62830 | RL23_MO | Rpl23 | Large ribosomal subunit protein uL14 | | | | 0.472108 | 0.478182 | 1.251785 | 0.694707 | 0.272391 | 1.872786 | 0.137547 | -0.20895 | -0.39888 | 2.255963 | 2.0846 | 3.753966 | 3.627091 | 3.190513 | 2.763282 | 2.064366 | 1.319832 | 3.287254 | 3.549021 | 2.707541 | 3.664152 |
| P62751 | P62751 | RL23A_M | Rpl23a | Large ribosomal subunit protein uL23 | + | | | 1.105914 | 1.248894 | 3.358907 | 0.178047 | 0.083383 | 0.505064 | 0.361921 | 0.412679 | 0.969449 | 4.039857 | 4.06835 | 4.142232 | 4.453232 | 4.446872 | 3.720128 | 3.176752 | 2.433667 | 4.158886 | 4.269811 | 3.449083 | 3.892558 |
| P70404 | P70404 | IDHG1_M | Idh3g | Isocitrate dehydrogenase [NAD] subunit gamma 1, mitochondrial | + | | | 2.132079 | -0.56585 | -11.5778 | 0.293735 | -0.45364 | -0.79821 | 0.244768 | -0.18471 | -0.67518 | 1.350118 | 1.270123 | 2.174978 | 1.139485 | 1.999901 | 2.498507 | 1.847883 | 1.904055 | 1.8765 | 2.345248 | 2.32128 | 2.546541 |
| P61255 | P61255 | RL26_MO | Rpl26 | Large ribosomal subunit protein uL24 | | | | 0.835843 | 0.770902 | 2.322016 | 0.609209 | 0.22932 | 1.623728 | 0.316651 | 0.108592 | 0.85566 | 2.271137 | 2.301892 | 2.461299 | 2.631509 | 2.117214 | 1.962683 | 1.847253 | 1.183972 | 2.429793 | 2.204376 | 1.830677 | 2.032036 |
| P61358 | P61358 | RL27_MO | Rpl27 | Large ribosomal subunit protein eL27 | | + | | 0.712134 | 0.621951 | 1.925511 | 1.070928 | 0.253651 | 3.208781 | 0.156094 | 0.105906 | 0.447877 | 2.812476 | 2.90409 | 3.130893 | 3.279895 | 2.745947 | 2.400782 | 2.556073 | 1.916591 | 2.97817 | 2.925316 | 2.305813 | 2.629105 |
| P14115 | P14115 | RL27A_M | Rpl27a | Large ribosomal subunit protein uL15 | | | | 0.510522 | 0.517866 | 1.353172 | 0.677231 | 0.075573 | 1.820611 | 0.060965 | -0.05729 | -0.18683 | 4.22759 | 4.087716 | 4.091032 | 4.170813 | 4.036842 | 4.509819 | 4.016047 | 3.263525 | 4.066829 | 4.043869 | 4.135383 | 4.525867 |
| P41105 | P41105 | RL28_MO | Rpl28 | Large ribosomal subunit protein eL28 | | | | 0.40601 | 0.510789 | 1.081216 | 0.168584 | -0.06123 | -0.48051 | 0.344652 | -0.57685 | -0.92596 | 1.957713 | 1.441221 | 3.317046 | 3.430552 | 2.582388 | 3.351913 | 1.584267 | 0.793089 | 3.549122 | 3.320937 | 3.054049 | 4.033945 |
| P47915 | P47915;W | RL29_MO | Rpl29 | Large ribosomal subunit protein eL29 | + | | + | 1.171572 | 1.07397 | 3.655428 | 0.845322 | 0.69561 | 2.354157 | 1.222554 | 0.301456 | 3.899895 | 1.281866 | 0.902982 | 1.022843 | 1.590251 | 0.999344 | 0.883705 | 0.243023 | -0.20611 | 0.528346 | 0.693529 | 0.691371 | 0.588766 |
| P27659 | P27659;E | RL3_MOU | Rpl3 | Large ribosomal subunit protein uL3 | + | | | 1.127092 | 0.995641 | 3.45239 | 0.981846 | 0.498668 | 2.849229 | 0.316527 | 0.257125 | 0.85535 | 1.728878 | 1.636513 | 1.920907 | 2.264165 | 1.429406 | 1.106775 | 0.971725 | 0.402384 | 1.628148 | 1.559589 | 0.757307 | 1.264624 |
| P27601 | P27601 | GNA13_M | Gna13 | Guanine nucleotide-binding protein subunit alpha-13 | + | | | 1.555199 | -0.56621 | -5.86649 | 0.382168 | -0.27919 | -1.02062 | 0.26799 | 0.126723 | 0.73362 | 5.193318 | 5.208932 | 5.436557 | 4.891626 | 5.393003 | 5.422143 | 5.863539 | 5.671138 | 5.418971 | 5.467592 | 5.452971 | 5.108729 |
| P62900 | P62900 | RL31_MO | Rpl31 | Large ribosomal subunit protein eL31 | | + | | 0.997149 | 0.96668 | 2.90879 | 1.376006 | 0.639739 | 4.720114 | 0.31923 | 0.213452 | 0.862128 | 3.099405 | 3.321941 | 3.859503 | 4.113034 | 3.522498 | 3.218853 | 2.557143 | 1.930843 | 3.29857 | 3.39449 | 2.961649 | 3.352798 |
| P62911 | P62911 | RL32_MO | Rpl32 | Large ribosomal subunit protein eL32 | + | | + | 1.009188 | 0.607548 | 2.956273 | 0.19357 | 0.084738 | 0.545064 | 1.216169 | 0.328404 | 3.868563 | 1.571504 | 1.590836 | 1.55249 | 1.733286 | 1.426958 | 1.544617 | 1.178906 | 0.768339 | 1.684631 | 1.431668 | 1.096184 | 1.218584 |
| Q9D1R9 | Q9D1R9 | RL34_MO | Rpl34 | Large ribosomal subunit protein eL34 | + | | | 1.15508 | 0.819492 | 3.579062 | 0.337162 | 0.255742 | 0.907138 | 0.692821 | 0.558658 | 1.867123 | 0.180863 | 0.576156 | 0.503881 | 1.067537 | 0.54226 | 0.594927 | -0.32539 | -0.55658 | 0.537227 | 0.522707 | -0.28811 | 0.307982 |
| Q6ZWV7 | Q6ZWV7 | RL35_MO | Rpl35 | Large ribosomal subunit protein uL29 | | | | 0.42572 | 0.213481 | 1.131639 | 0.155202 | -0.16606 | -0.44554 | 0.357467 | 0.21729 | 0.958222 | 3.325699 | 2.966147 | 2.322294 | 2.554994 | 2.781806 | 2.480978 | 2.989613 | 2.875271 | 2.95881 | 2.250605 | 2.5838 | 2.244405 |
| O55142 | O55142 | RL35A_M | Rpl35a | Large ribosomal subunit protein eL33 | | | | 0.59445 | 0.415965 | 1.582213 | 0.519462 | 0.16848 | 1.377049 | 0.399617 | 0.232342 | 1.064928 | 1.425339 | 1.438594 | 1.797917 | 1.822816 | 1.439288 | 1.019445 | 1.278819 | 0.753185 | 1.7636 | 1.520173 | 0.937576 | 1.056472 |
| P47964 | P47964 | RL36_MO | Rpl36 | Large ribosomal subunit protein eL36 | | + | | 0.59612 | 0.304527 | 1.586889 | 1.310801 | 0.407 | 4.355181 | 0.198667 | 0.100822 | 0.55813 | 2.206821 | 2.146537 | 2.573858 | 2.642031 | 2.00259 | 1.892514 | 2.061672 | 1.682633 | 2.11393 | 2.287958 | 1.674677 | 2.018784 |
| P83882 | P83882;A | RL36A_M | Rpl36a | Large ribosomal subunit protein eL42 | | | | 0.49803 | 1.83523 | 1.319993 | 0.092839 | 0.066891 | 0.277373 | 0.386784 | 0.262145 | 1.032323 | 1.019551 | 0.773732 | 0.745485 | 1.070193 | 0.727612 | 0.841703 | 0.446301 | -2.32348 | 1.019271 | 0.662624 | 0.275066 | 0.769959 |
| P61514 | P61514 | RL37A_M | Rpl37a | Large ribosomal subunit protein eL43 | | | | 0.317247 | 0.524093 | 0.857154 | 0.64025 | 0.393294 | 1.712399 | 0.046964 | -0.01725 | -0.14572 | 0.009991 | 0.346968 | 0.794609 | 0.698476 | 0.000314 | 0.20043 | 0.242147 | -0.93337 | 0.577837 | 0.12866 | 0.180906 | 0.054341 |
| Q9JJI8 | Q9JJI8 | RL38_MO | Rpl38 | Large ribosomal subunit protein eL38 | | | | 0.523489 | 0.244441 | 1.387844 | 0.907898 | 0.131286 | 2.573326 | 0.013767 | -0.0163 | -0.04415 | 1.993132 | 2.224869 | 2.83147 | 2.930519 | 2.721691 | 2.342084 | 1.997211 | 1.731908 | 2.761962 | 2.737455 | 2.231601 | 2.864767 |
| P62892 | P62892 | RL39_MO | Rpl39 | Large ribosomal subunit protein eL39 | | | | 0.967461 | 1.592128 | 2.794033 | 0.523427 | 0.254666 | 1.387678 | 0.238471 | 0.30616 | 0.659293 | 0.687227 | 0.936856 | 0.966099 | 0.823045 | 0.827765 | 0.786344 | -0.22409 | -1.33608 | 0.808913 | 0.4709 | 0.03698 | 0.964808 |
| Q9D8E6 | Q9D8E6 | RL4_MOU | Rpl4 | Large ribosomal subunit protein uL4 | + | + | | 1.198032 | 0.845519 | 3.780693 | 1.018008 | 0.476271 | 2.991414 | 0.239733 | 0.160302 | 0.662479 | 2.727749 | 2.655652 | 3.07221 | 3.380187 | 2.704541 | 2.298186 | 2.066898 | 1.625465 | 2.709478 | 2.790377 | 2.209647 | 2.472476 |
| P47962 | P47962 | RL5_MOU | Rpl5 | Large ribosomal subunit protein uL18 | | | | 0.502519 | 0.319962 | 1.331892 | 0.012814 | 0.012088 | 0.041134 | 0.650252 | 0.096396 | 1.741383 | -0.58892 | -0.16866 | 0.297553 | 0.076338 | 0.00656 | -0.05477 | -0.58231 | -0.81519 | -0.0974 | 0.447116 | -0.16659 | -0.07441 |
| P47911 | P47911 | RL6_MOU | Rpl6 | Large ribosomal subunit protein eL6 | | + | | 0.64724 | 0.816306 | 1.732631 | 1.066664 | 0.575003 | 3.190843 | 0.246576 | 0.318379 | 0.679741 | 2.361332 | 2.490388 | 2.904034 | 3.165153 | 2.671161 | 1.880882 | 2.076251 | 1.142857 | 2.335381 | 2.583799 | 1.706154 | 2.209131 |
| P14148 | P14148 | RL7_MOU | Rpl7 | Large ribosomal subunit protein uL30 | | + | | 0.675363 | 0.683126 | 1.815076 | 1.795468 | 0.479177 | 7.806714 | 0.276258 | 0.223683 | 0.754377 | 2.734682 | 2.757716 | 3.116223 | 3.204532 | 2.6481 | 2.179365 | 2.439259 | 1.686886 | 2.723837 | 2.638564 | 2.008411 | 2.371687 |
| Q60692 | Q60692 | PSB6_MO | Psmb6 | Proteasome subunit beta type-6 | + | | | 1.367898 | -0.57533 | -4.67333 | 0.322642 | -0.32396 | -0.87069 | 0.030599 | -0.04382 | -0.09644 | 1.156031 | 0.936151 | 1.056153 | 0.363657 | 0.922426 | 1.824582 | 1.566024 | 1.676825 | 1.170054 | 0.897668 | 1.472161 | 1.362487 |
| P62918 | P62918 | RL8_MOU | Rpl8 | Large ribosomal subunit protein uL2 | | | | 0.707777 | 0.527482 | 1.912263 | 0.420942 | 0.268465 | 1.119383 | 0.772075 | 0.243696 | 2.112532 | 2.437429 | 2.458657 | 2.402925 | 2.712572 | 2.108999 | 1.906034 | 2.196198 | 1.644924 | 2.472449 | 2.106118 | 1.708969 | 1.818671 |
| P51410 | A0A140T8 | RL9_MOU | Rpl9 | Large ribosomal subunit protein uL6 | | | | 0.496703 | -0.1545 | -1.31648 | 0.267638 | 0.241813 | 0.732734 | 0.087029 | 0.03973 | 0.261149 | 1.148588 | 0.914507 | 1.802663 | 1.60884 | 1.537453 | 1.796234 | 1.177407 | 1.194688 | 1.779403 | 1.148474 | 1.547091 | 1.707135 |
| P14869 | P14869 | RLA0_MO | Rplp0 | Large ribosomal subunit protein uL10 | | + | | 0.759551 | 0.698697 | 2.0727 | 1.194174 | 0.417663 | 3.762215 | 0.402309 | 0.727306 | 1.071783 | 2.177922 | 2.341496 | 2.766063 | 2.886509 | 3.343063 | 2.021142 | 1.888035 | 1.233989 | 2.315362 | 2.501884 | 1.801105 | 2.108488 |
| P47955 | P47955 | RLA1_MO | Rplp1 | Large ribosomal subunit protein P1 | | | | 0.889045 | 2.083224 | 2.505978 | 0.373593 | 0.761315 | 0.998921 | 0.511081 | -0.64406 | -1.35466 | -0.3966 | -0.61078 | 0.706014 | 0.406248 | -0.53526 | -1.28808 | -3.41129 | -1.76254 | -0.95244 | 0.54207 | -0.55805 | 0.02284 |
| P55012 | P55012 | S12A2_M | Slc12a2 | Solute carrier family 12 member 2 | + | | + | 1.538681 | -0.57596 | -5.75116 | 0.126716 | -0.1035 | -0.36993 | 1.257991 | 1.039201 | 4.07765 | 0.867037 | 0.691519 | -0.84126 | -1.3474 | 0.550704 | 0.068944 | 1.30699 | 1.40348 | -0.87159 | -1.11011 | -0.81261 | -0.64615 |
| Q91YQ5 | Q91YQ5 | RPN1_MO | Rpn1 | Dolichyl-diphosphooligosaccharide--protein glycosyltransferase subunit 1 | | | | 0.038863 | -0.04964 | -0.1215 | 0.056474 | 0.071951 | 0.173741 | 0.815972 | 0.514448 | 2.255503 | -0.12731 | -0.03526 | 0.26981 | -0.51855 | 0.619106 | 0.38588 | 0.374282 | -0.43758 | -0.06934 | -0.3233 | 0.184067 | -0.20798 |
| Q9DBG6 | Q9DBG6 | RPN2_MO | Rpn2 | Dolichyl-diphosphooligosaccharide--protein glycosyltransferase subunit 2 | | | | 0.011023 | 0.007486 | 0.035454 | 0.006857 | -0.00472 | -0.02216 | 0.485031 | 0.479904 | 1.285682 | 0.225786 | -0.03186 | -0.32939 | -0.72452 | 0.696138 | -0.04711 | 0.256777 | -0.07782 | -0.6021 | -0.44236 | -0.1204 | -0.19038 |
| P63325 | P63325 | RS10_MO | Rps10 | Small ribosomal subunit protein eS10 | | | | 0.261292 | 0.314376 | 0.716788 | 0.177958 | 0.350873 | 0.504834 | 0.272106 | 0.101815 | 0.743955 | -1.01777 | -0.24135 | 0.703021 | -0.66942 | -0.35356 | -0.57204 | -1.14802 | -0.73985 | -0.2238 | -0.44434 | -0.48217 | -0.64706 |
| O35633 | O35633 | VIAAT_MO | Slc32a1 | Vesicular inhibitory amino acid transporter | + | | | 1.796537 | -0.58076 | -7.81656 | 0.483552 | -0.38292 | -1.28179 | 0.224074 | 0.374424 | 0.622859 | 3.132407 | 2.996723 | 1.446836 | 0.971194 | 1.966085 | 2.546423 | 3.675624 | 3.615033 | 1.772736 | 1.411143 | 2.408298 | 1.355363 |
| P63323 | P63323 | RS12_MO | Rps12 | Small ribosomal subunit protein eS12 | | + | | 0.796306 | 0.315743 | 2.19079 | 1.009419 | 0.295487 | 2.95719 | 0.929477 | 0.112063 | 2.651865 | 1.608577 | 1.709767 | 2.076279 | 1.90898 | 2.081696 | 2.144991 | 1.478379 | 1.208478 | 1.642489 | 1.751797 | 1.973278 | 2.029284 |
| P62301 | P62301 | RS13_MO | Rps13 | Small ribosomal subunit protein uS15 | | | | 0.238645 | 0.272418 | 0.659731 | 0.244675 | 0.115378 | 0.674949 | 0.045865 | -0.04392 | -0.14245 | 2.109973 | 1.515834 | 2.099482 | 1.761979 | 1.831929 | 1.916909 | 1.827288 | 1.253684 | 1.78807 | 1.842636 | 1.612974 | 2.223702 |

| | | | | | | | | | | | | | | | | | | | | | | | | | | | | |
|---|---|---|---|---|---|---|---|---|---|---|---|---|---|---|---|---|---|---|---|---|---|---|---|---|---|---|---|---|
| P62264 | P62264 | RS14_MO | Rps14 | Small ribosomal subunit protein uS11 | | | | 0.299826 | 0.357653 | 0.813479 | 0.8094 | 0.168598 | 2.233756 | 0.182816 | 0.139483 | 0.517388 | 1.608239 | 1.425315 | 2.392904 | 2.443312 | 2.584268 | 2.32498 | 1.589164 | 0.729084 | 2.320654 | 2.178364 | 2.07877 | 2.551513 |
| Q8VEM8 | Q8VEM8 | S25A3_M | Slc25a3 | Solute carrier family 25 member 3 | + | | + | 1.837243 | -0.60014 | -8.20055 | 0.232315 | -0.20569 | -0.64373 | 1.29633 | 0.427754 | 4.277581 | 4.324747 | 4.46318 | 4.082565 | 3.459154 | 4.683622 | 4.644741 | 4.970337 | 5.017866 | 4.046798 | 3.906296 | 4.334518 | 4.138336 |
| P62245 | P62245 | RS15A_M | Rps15a | Small ribosomal subunit protein uS8 | | | | 0.604132 | 0.558975 | 1.609399 | 0.523601 | 0.205035 | 1.388143 | 0.070465 | 0.094497 | 0.214226 | 2.344378 | 2.196891 | 3.358803 | 3.120877 | 3.167669 | 2.761041 | 2.05106 | 1.37226 | 2.947258 | 3.122352 | 2.478399 | 3.261317 |
| P14131 | P14131 | RS16_MO | Rps16 | Small ribosomal subunit protein uS9 | | | | 0.689189 | 0.45832 | 1.856236 | 0.074078 | -0.02686 | -0.22455 | 0.104669 | -0.09652 | -0.31007 | 2.326593 | 2.354867 | 3.09496 | 2.858326 | 2.848294 | 2.590358 | 2.128913 | 1.635907 | 2.985773 | 3.021243 | 2.532524 | 3.099176 |
| P63276 | P63276 | RS17_MO | Rps17 | Small ribosomal subunit protein eS17 | | | | 0.900453 | 1.215268 | 2.546591 | 0.094043 | 0.064935 | 0.28072 | 0.407242 | 0.267716 | 1.084356 | -0.13647 | 0.317057 | -0.39886 | -0.83778 | -0.038 | -0.29161 | -0.70508 | -1.54487 | -0.75635 | -0.61016 | -0.64436 | -0.22069 |
| P62270 | F6YVP7;P | RS18_MO | Rps18 | Small ribosomal subunit protein uS13 | | | | 0.563606 | 0.199973 | 1.49671 | 0.171709 | 0.120719 | 0.488634 | 0.26989 | 0.185959 | 0.738392 | 1.596371 | 1.792916 | 2.157049 | 1.663 | 1.772932 | 2.218393 | 1.58519 | 1.40415 | 1.793115 | 1.785496 | 1.692163 | 1.927245 |
| Q9CZX8 | Q9CZX8 | RS19_MO | Rps19 | Small ribosomal subunit protein eS19 | | | | 0.518482 | -0.4039 | -1.37443 | 0.529896 | 0.405933 | 1.405063 | 0.030913 | -0.02433 | -0.09739 | -0.53734 | 0.002996 | 1.038334 | 0.579521 | -0.36855 | 0.13102 | 0.252347 | 0.02111 | 0.578608 | 0.227381 | -0.09206 | -0.09681 |
| P25444 | P25444 | RS2_MO | Rps2 | Small ribosomal subunit protein uS5 | | | | 0.859955 | 0.604892 | 2.404304 | 0.348382 | -0.12455 | -0.93535 | 0.080402 | -0.09782 | -0.2425 | 3.729805 | 3.465347 | 4.140865 | 4.380021 | 4.0906 | 3.920546 | 3.20672 | 2.778648 | 4.443579 | 4.326406 | 3.709064 | 4.497727 |
| P60867 | P60867 | RS20_MO | Rps20 | Small ribosomal subunit protein uS10 | | | | 0.981037 | 0.528826 | 2.846106 | 0.090546 | 0.041628 | 0.270982 | 0.280489 | 0.08499 | 0.764993 | 2.32208 | 2.518395 | 3.216954 | 2.920371 | 3.164939 | 3.150371 | 2.049175 | 1.733649 | 2.986928 | 3.067141 | 2.961804 | 3.183525 |
| Q9CQR2 | Q9CQR2 | RS21_MO | Rps21 | Small ribosomal subunit protein eS21 | | | | 0.887953 | 0.481136 | 2.502113 | 0.621933 | -0.2907 | -1.65985 | 0.012933 | -0.00947 | -0.04151 | 1.687073 | 1.515938 | 1.885114 | 1.630568 | 2.009568 | 2.454939 | 0.948166 | 1.292574 | 2.168858 | 1.928232 | 2.291777 | 2.19168 |
| P01831 | P01831 | THY1_MO | Thy1 | Thy-1 membrane glycoprotein | + | | | 2.343144 | -0.60311 | -14.7941 | 0.433716 | 1.084879 | 1.152193 | 0.185663 | 0.258477 | 0.52473 | 0.156525 | 0.192729 | 1.95375 | 0.072169 | 0.706139 | 1.003979 | 0.814262 | 0.741208 | -0.1104 | -0.03343 | 1.066123 | 0.12704 |
| P62849 | P62849 | RS24_MO | Rps24 | Small ribosomal subunit protein eS24 | + | | | 1.110082 | 0.944947 | 3.377145 | 0.411114 | 0.428481 | 1.094241 | 0.556136 | 0.472075 | 1.476245 | -0.39991 | 0.115348 | 0.84947 | 1.143064 | 0.843391 | 0.713697 | -0.97805 | -1.1964 | 0.204765 | 0.930807 | -0.00667 | 0.619606 |
| P62852 | P62852 | RS25_MO | Rps25 | Small ribosomal subunit protein eS25 | | | | 0.315663 | -0.26183 | -0.85318 | 0.162771 | 0.06173 | 0.465357 | 0.036176 | 0.035213 | 0.11339 | 2.034904 | 2.521658 | 2.412677 | 2.168987 | 1.922946 | 2.543759 | 2.727044 | 2.353173 | 2.281541 | 2.176664 | 2.207391 | 2.188889 |
| P62855 | P62855 | RS26_MO | Rps26 | Small ribosomal subunit protein eS26 | + | | | 1.194363 | 1.373159 | 3.763119 | 0.363683 | 0.295153 | 0.973895 | 0.080644 | 0.135207 | 0.243182 | 1.051284 | 1.764207 | 2.27463 | 2.163034 | 2.000885 | 1.725025 | 0.112603 | -0.04343 | 1.625796 | 2.221563 | 1.189137 | 2.266358 |
| Q6ZWU9 | Q6ZWY3;C | RS27_MO | Rps27 | Small ribosomal subunit protein eS27 | | + | | 0.520472 | 0.1541 | 1.379755 | 1.199194 | 0.352606 | 3.786272 | 0.225791 | 0.214895 | 0.627211 | 2.011958 | 1.827404 | 2.870494 | 3.034565 | 2.979628 | 2.413612 | 1.8285 | 1.702661 | 2.644002 | 2.555845 | 2.288604 | 2.674845 |
| P0CG49;P | P0CG49;P | RL40_MO | Rps27a;Uba52;Ubb;Ubc | Polyubiquitin-B | + | | | 1.004634 | 0.544589 | 2.938246 | 0.010167 | -0.00219 | -0.03273 | 0.109105 | -0.06488 | -0.32222 | 4.792181 | 4.824463 | 5.15147 | 5.068292 | 4.949592 | 5.352287 | 4.448374 | 4.079093 | 5.164572 | 5.059574 | 5.214053 | 5.217587 |
| Q6ZWY3 | Q6ZWY3 | RS27L_M | Rps27l | Small ribosomal subunit protein eS27-like | | | | 0.742598 | 2.11067 | 2.019432 | 0.417197 | -0.08864 | -1.10979 | 0.642568 | 0.469545 | 1.719096 | -0.04332 | -0.19905 | -0.66473 | -0.50831 | -0.33745 | -0.42765 | -1.18958 | -3.27413 | -0.48166 | -0.51409 | -1.12148 | -0.58271 |
| P62858 | P62858 | RS28_MO | Rps28 | Small ribosomal subunit protein eS28 | | | | 0.857412 | 0.680723 | 2.395542 | 0.312034 | 0.390855 | 0.844083 | 0.72754 | -0.29728 | -1.97272 | 0.916999 | 1.139397 | 1.565606 | 1.524918 | 1.52891 | 1.273138 | 0.085974 | 0.608977 | 0.691801 | 1.617013 | 1.778027 | 1.61859 |
| P62274 | P62274 | RS29_MO | Rps29 | Small ribosomal subunit protein uS14 | | | | 0.244606 | 0.154026 | 0.674776 | 0.027189 | -0.02361 | -0.08598 | 0.920388 | 0.207135 | 2.618592 | 0.769276 | 0.391664 | 1.092982 | 0.64151 | 0.828769 | 0.983545 | 0.554725 | 0.298164 | 1.047144 | 0.734561 | 0.682645 | 0.715398 |
| P62908 | P62908 | RS3_MO | Rps3 | Small ribosomal subunit protein uS3 | | | | 0.458048 | 0.127313 | 1.215127 | 0.006734 | 0.004133 | 0.021761 | 0.135813 | 0.123588 | 0.394264 | 2.457029 | 2.472112 | 3.40849 | 3.288 | 3.514669 | 3.182959 | 2.441759 | 2.232755 | 3.163974 | 3.524249 | 2.959231 | 3.49122 |
| P97351 | P97351 | RS3A_MO | Rps3a | Small ribosomal subunit protein eS1 | + | + | + | 1.071899 | 0.67396 | 3.212879 | 1.001119 | 0.29125 | 2.924389 | 1.1614 | 0.413458 | 3.608173 | 2.740983 | 2.934742 | 3.18283 | 3.33528 | 2.876491 | 2.647321 | 2.349959 | 1.977846 | 2.903707 | 3.031904 | 2.347432 | 2.349464 |
| P62702 | P62702 | RS4X_MO | Rps4x | Small ribosomal subunit protein eS4 | | | | 0.704172 | 0.705402 | 1.901335 | 0.160156 | 0.08103 | 0.458521 | 0.081432 | -0.10593 | -0.24541 | 2.327116 | 2.342754 | 2.839455 | 2.491925 | 2.40766 | 2.303828 | 2.000454 | 1.258612 | 2.61684 | 2.552481 | 2.033167 | 2.890177 |
| P97461 | P97461 | RS5_MO | Rps5 | Small ribosomal subunit protein uS7 | | | | 0.917464 | 0.419921 | 2.607948 | 0.302882 | 0.09037 | 0.821138 | 0.216067 | 0.356688 | 0.602526 | 1.844615 | 1.622403 | 1.675205 | 1.749099 | 2.304949 | 1.514443 | 1.197048 | 1.430128 | 1.725449 | 1.518115 | 1.1123 | 1.993716 |
| P62754 | P62754 | RS6_MO | Rps6 | Small ribosomal subunit protein eS6 | | | | 0.693903 | 0.870683 | 1.870371 | 0.090211 | 0.096686 | 0.270049 | 0.53058 | 0.632927 | 1.406906 | 2.914377 | 2.76597 | 2.660001 | 3.369579 | 3.604652 | 2.881817 | 2.429051 | 1.509928 | 2.870044 | 2.966165 | 2.342424 | 2.878192 |
| P62082 | P62082 | RS7_MO | Rps7 | Small ribosomal subunit protein eS7 | | + | | 0.12937 | 0.405419 | 0.377045 | 1.254474 | 0.34111 | 4.059708 | 0.289552 | 0.273712 | 0.787721 | 0.774219 | 1.32208 | 2.647163 | 2.490532 | 2.092643 | 1.669858 | 1.682504 | -0.39704 | 2.197297 | 2.258177 | 1.331764 | 1.883312 |
| P62242 | P62242 | RS8_MO | Rps8 | Small ribosomal subunit protein eS8 | | | | 0.973579 | 0.888743 | 2.817417 | 0.94014 | 0.659014 | 2.691259 | 0.181151 | 0.211573 | 0.513088 | 2.62446 | 2.753973 | 3.240367 | 3.605082 | 2.79376 | 2.146474 | 2.109201 | 1.491746 | 2.600284 | 2.927137 | 2.003027 | 2.514062 |
| Q9CPQ1 | Q9CPQ1 | COX6C_M | Cox6c | Cytochrome c oxidase subunit 6C | + | | | 1.797135 | -0.60487 | -7.82208 | 0.046352 | -0.07324 | -0.1439 | 0.110369 | 0.178183 | 0.325674 | 3.263336 | 3.117111 | 2.735954 | 2.020979 | 2.085953 | 2.67282 | 3.820271 | 3.769906 | 2.813927 | 2.089476 | 2.66298 | 1.739426 |
| P14206 | P14206 | RSSA_MO | Rpsa | Small ribosomal subunit protein uS2 | | | | 0.534383 | 0.107049 | 1.41716 | 0.51716 | -0.31077 | -1.37089 | 0.672082 | -0.12752 | -1.80537 | 1.475228 | 1.392656 | 1.7021 | 1.456516 | 1.950987 | 2.06089 | 1.263637 | 1.39015 | 1.699522 | 2.080631 | 2.177843 | 2.08908 |
| Q8K4Q0 | Q8K4Q0 | RPTOR_M | Rptor | Regulatory-associated protein of mTOR | | | | 0.400683 | 0.218009 | 1.067641 | 0.270454 | 0.287527 | 0.739808 | 0.036704 | -0.04096 | -0.11499 | -0.53884 | -0.75523 | 0.286044 | 0.428577 | 0.006315 | 0.15011 | -0.69187 | -1.03822 | -0.31228 | 0.451845 | -0.22974 | 0.468096 |
| Q80X95 | Q6NTA4;Q | RRAGA_M | Rraga | Ras-related GTP-binding protein A | | | | 0.264123 | 0.192676 | 0.723903 | 0.014964 | -0.00592 | -0.04793 | 0.124392 | 0.08914 | 0.363678 | -0.25279 | -0.77589 | -0.20004 | -0.23503 | -0.38675 | 0.084713 | -0.75635 | -0.65768 | -0.33382 | -0.08942 | -0.3073 | -0.17301 |
| Q99K70 | Q7TT45;Q | RRAGC_M | Rragc | Ras-related GTP-binding protein C | | | | 0.138627 | 0.137687 | 0.401753 | 0.392464 | -0.32474 | -1.04674 | 0.083539 | 0.04847 | 0.251346 | -0.84145 | -0.15794 | -0.30182 | -0.71786 | -0.39809 | -0.5069 | -0.66304 | -0.61172 | 0.045063 | -0.41526 | -0.31595 | -0.68597 |
| P62071 | P62071 | RRAS2_M | Rras2 | Ras-related protein R-Ras2 | | | | 0.250598 | 0.140502 | 0.689877 | 0.046671 | 0.026489 | 0.144848 | 0.05192 | 0.045607 | 0.160375 | 0.637511 | 0.72728 | 0.44164 | 0.102229 | 0.927874 | 0.691321 | 0.740548 | 0.343239 | 0.177316 | 0.313576 | 0.505374 | 1.022606 |
| Q99PL5 | Q99PL5 | RRBP1_M | Rrbp1 | Ribosome-binding protein 1 | | | + | 0.583702 | -1.11613 | -1.55223 | 0.366235 | -1.07192 | -0.98034 | 1.055346 | 1.523946 | 3.143591 | -3.66187 | -3.40707 | -2.84759 | -3.61857 | -1.92243 | -2.81862 | -1.71067 | -3.12602 | -1.13795 | -3.18437 | -4.07946 | -3.70948 |
| Q6P5B0 | Q6P5B0 | RRP12_M | Rrp12 | RRP12-like protein | | | | 0.835008 | 1.034443 | 2.319198 | 0.24338 | 0.227377 | 0.671684 | 0.526064 | 0.680444 | 1.394756 | -2.51944 | -1.7473 | -1.40484 | -2.0656 | -2.537 | -1.5725 | -3.39119 | -2.94444 | -2.03638 | -1.88882 | -2.80897 | -2.66142 |
| Q01730 | Q01730 | RSU1_MO | Rsu1 | Ras suppressor protein 1 | | | + | 0.504771 | 1.474529 | 1.337871 | 0.236437 | -0.21418 | -0.65415 | 1.024258 | -0.34883 | -3.0165 | -0.269 | -0.02419 | -0.46338 | -0.51342 | -0.43942 | -0.47646 | -2.71645 | -0.5258 | 0.052239 | -0.60068 | 0.005034 | -0.22326 |
| Q9D7H3 | Q9D7H3 | RTCA_MO | RtcA | RNA 3'-terminal phosphate cyclase | | | | 0.154913 | 0.446083 | 0.444779 | 0.793704 | 0.392294 | 2.182312 | 0.260191 | -0.40767 | -0.71402 | -1.85253 | -1.64924 | -0.91974 | -0.65245 | -1.46137 | -2.26766 | -3.19473 | -1.1992 | -1.05817 | -1.2986 | -1.86114 | -1.05256 |
| Q99LF4 | Q99LF4 | RTCB_MO | Rtcb | RNA-splicing ligase RtcB homolog | | + | | 0.046303 | -0.05924 | -0.14376 | 1.086735 | -0.39124 | -3.27596 | 0.305033 | -0.28103 | -0.82653 | -0.21761 | -0.4077 | 0.382421 | 0.277336 | -0.02875 | 0.632675 | 0.147525 | -0.65436 | 0.613871 | 0.82837 | 0.661954 | 0.504025 |
| A2AQ19 | A2AQ19 | RTF1_MO | Rtf1 | RNA polymerase-associated protein RTF1 homolog | | | | 0.195114 | 0.661209 | 0.549024 | 0.54665 | 0.277019 | 1.450389 | 0.033053 | -0.02462 | -0.10392 | -1.43918 | -0.92111 | -0.61554 | -0.50501 | -0.66001 | -1.09557 | -0.6652 | -3.0175 | -1.02012 | -0.65447 | -0.75991 | -0.94643 |
| Q7M732 | Q7M732 | RTL1_MO | Rtl1 | Retrotransposon-like protein 1 | | | | 0.46959 | 1.892278 | 1.245202 | 0.721731 | 2.820476 | 1.954854 | 0.563455 | 1.996871 | 1.496294 | -0.03215 | -0.00429 | 0.698582 | 1.24363 | -0.54002 | -0.31876 | -0.39091 | -3.43009 | -3.26621 | -0.43253 | -3.75621 | -1.09631 |
| Q9D1F0 | Q9D1F0 | Q9D1F0_M | Rtl8b | CAAX box 1 homolog A (Human) | | | | 0.312649 | -0.72607 | -0.84563 | 0.646798 | 0.417594 | 1.731348 | 0.362209 | 1.213636 | 0.970176 | -3.65373 | -3.69674 | -0.38553 | -0.32418 | -0.93717 | -1.83171 | -2.09082 | -3.80751 | -1.01169 | -0.53321 | -3.76633 | -1.42982 |
| Q8BLF1 | Q8BLF1 | NCEH1_M | Nceh1 | Neutral cholesterol ester hydrolase 1 | + | | | 1.979712 | -0.60512 | -9.69219 | 0.184202 | -0.23142 | -0.52096 | 0.05549 | 0.038403 | 0.17086 | -0.86415 | -0.75439 | 0.020775 | -0.77946 | -0.31467 | 0.014218 | -0.17438 | -0.23392 | -0.34087 | 0.045015 | -0.03541 | -0.34185 |
| Q9ES97 | Q9ES97 | RTN3_MO | Rtn3 | Reticulon-3 | | | | 0.501963 | -0.24561 | -1.33042 | 0.141647 | -0.05683 | -0.40977 | 0.795452 | -0.21694 | -2.18801 | 2.825398 | 2.469492 | 3.63679 | 3.770581 | 3.550462 | 3.354585 | 2.942173 | 2.843929 | 3.639022 | 3.882017 | 3.654011 | 3.684917 |
| Q99P72 | Q99P72 | RTN4_MO | Rtn4 | Reticulon-4 | | | | 0.041024 | 0.017352 | 0.127994 | 0.755873 | 0.328611 | 2.061081 | 0.59282 | 0.307197 | 1.577651 | 0.325393 | 0.495835 | 1.098044 | 0.861032 | 1.051676 | 0.69329 | 0.498694 | 0.28783 | 0.544267 | 0.757587 | 0.489096 | 0.641477 |
| Q9CQE8 | Q9CQE8 | RTRAF_M | RTRAF | RNA transcription, translation and transport factor protein | | | | 0.153402 | -0.13404 | -0.44081 | 0.43086 | -0.10493 | -1.14484 | 0.280397 | -0.24056 | -0.76476 | -0.07118 | 0.314734 | 0.276442 | 0.100492 | 0.252715 | 0.804848 | 0.490818 | 0.02081 | 0.267692 | 0.319099 | 0.920118 | 0.618567 |
| Q8R4C2 | Q8R4C2 | RUFY2_M | Rufy2 | RUN and FYVE domain-containing protein 2 | | | | 0.438736 | 2.443771 | 1.165127 | 0.891693 | 0.671889 | 2.515365 | 0.690643 | 0.967067 | 1.860592 | -3.12658 | 0.939847 | -1.15544 | -0.67393 | -1.35404 | -1.69109 | -4.05216 | -3.02212 | -1.70227 | -1.47088 | -2.98131 | -1.99794 |
| A2A8L5 | A2A8L5 | PTPRF_M | Ptprf | Receptor-type tyrosine-protein phosphatase F | + | | | 1.58465 | -0.6072 | -6.0774 | 0.091985 | 0.145472 | 0.274996 | 0.246546 | 0.658086 | 0.679667 | -3.30525 | -3.46194 | -2.16489 | -1.55302 | -1.34147 | -3.02135 | -2.8384 | -2.71439 | -2.43598 | -1.57287 | -2.35783 | -3.32117 |
| Q9CQZ5 | Q9CQZ5 | NDUA6_M | Ndufa6 | NADH dehydrogenase [ubiquinone] 1 alpha subcomplex subunit 6 | + | | | 2.411231 | -0.61401 | -16.0084 | 0.325211 | 0.249768 | 0.877132 | 0.442074 | 0.271923 | 1.173741 | 2.307114 | 2.382594 | 1.991013 | 1.516843 | 2.375835 | 1.959048 | 2.952019 | 2.965707 | 1.346438 | 1.661882 | 1.996731 | 1.794307 |
| Q6PDC0 | Q6PDC0 | RUN3B_M | Rundc3b | RUN domain-containing protein 3B | | + | + | 0.449386 | 0.724826 | 1.192653 | 1.071276 | 0.989649 | 3.210251 | 2.077985 | -0.85347 | -10.8707 | -0.23615 | -1.19879 | -0.02395 | 0.123162 | -1.87231 | -1.73086 | -1.81335 | -1.07124 | -1.23941 | -0.64067 | -0.98221 | -0.91402 |
| Q9R1P0 | Q9R1P0 | PSA4_MO | Psma4 | Proteasome subunit alpha type-4 | + | + | | 1.730783 | -0.61788 | -7.2322 | 1.033378 | -0.86132 | -3.05338 | 0.071042 | -0.08477 | -0.21588 | 1.577791 | 1.577978 | 1.559083 | 1.0071 | 1.781935 | 2.511871 | 2.110331 | 2.2812 | 2.202746 | 2.086084 | 2.376491 | 2.086845 |
| Q9WTM5 | Q9WTM5 | RUVB2_M | Ruvbl2 | RuvB-like 2 | | | | 0.825223 | 0.40339 | 2.286323 | 0.431427 | 0.308637 | 1.146303 | 0.094652 | -0.10381 | -0.28241 | -0.16776 | 0.162567 | 0.853426 | 1.304477 | 0.495342 | 0.035448 | -0.34393 | -0.46804 | 0.623237 | 0.91739 | 0.082441 | 0.65596 |
| E9Q401 | E9Q401 | RYR2_MO | Ryr2 | Ryanodine receptor 2 | | | | 0.177669 | -0.12732 | -0.50408 | 0.500222 | -0.12199 | -1.3258 | 0.460722 | -0.64692 | -1.22208 | -0.37212 | -0.26948 | 1.195249 | 1.363297 | 0.641796 | 1.486793 | -0.44079 | 0.053816 | 1.363764 | 1.438756 | 1.392286 | 2.030139 |
| A2AGL3 | A2AGL3 | RYR3_MO | Ryr3 | Ryanodine receptor 3 | | | | 0.243358 | -1.06838 | -0.67163 | 0.060721 | 0.068066 | 0.186118 | 0.729263 | -1.34989 | -1.97804 | -3.05664 | -1.16157 | -0.40139 | 0.272753 | -0.69462 | 0.497568 | -2.31846 | 0.237006 | 0.009501 | -0.27427 | 0.919106 | 1.583617 |
| Q9CQZ6 | Q9CQZ6 | NDUB3_M | Ndufb3 | NADH dehydrogenase [ubiquinone] 1 beta subcomplex subunit 3 | + | | | 1.462439 | -0.62802 | -5.24514 | 0.445464 | -0.44106 | -1.1825 | 0.07097 | 0.128857 | 0.215674 | 2.786499 | 2.566723 | 1.936805 | 1.565056 | 2.099584 | 2.768054 | 3.35218 | 3.257082 | 2.515369 | 1.868616 | 2.800185 | 1.809738 |
| P50543 | P50543 | S10AB_M | S100a11 | Protein S100-A11 | + | + | | 1.089827 | -2.2258 | -3.28922 | 1.235558 | -2.61514 | -3.96436 | 0.516138 | -0.86284 | -1.36816 | -1.40764 | -2.75139 | -2.80347 | -1.56882 | -3.55262 | -2.35451 | 0.226895 | 0.06568 | 0.661504 | 0.196487 | -1.89357 | -2.28786 |
| Q9D2Q8 | Q9D2Q8 | S10AE_M | S100a14 | Protein S100-A14 | | | | 0.213558 | 0.297706 | 0.596143 | 0.221548 | -0.30473 | -0.61645 | 0.256838 | -0.29458 | -0.70558 | 0.60101 | -0.23802 | -2.09967 | -1.23742 | -1.76661 | -2.05333 | -0.38712 | 0.154698 | -1.60567 | -1.12197 | -2.00751 | -1.22327 |
| Q9D708 | Q9D708 | S10AG_M | S100a16 | Protein S100-A16 | | | | 0.603164 | 1.08671 | 1.606675 | 0.084057 | -0.22092 | -0.2528 | 0.278534 | 0.502193 | 0.760087 | -1.50583 | -1.52906 | -2.70692 | -3.73299 | -2.03246 | -1.95701 | -3.28043 | -1.92788 | -3.70647 | -2.29161 | -3.15655 | -1.8373 |
| P14069 | P14069 | S10A6_M | S100a6 | Protein S100-A6 | + | + | | 1.287482 | 0.747832 | 4.230719 | 1.162616 | 0.719499 | 3.613798 | 0.579392 | 1.366315 | 1.540264 | 3.171446 | 2.819026 | 1.966576 | 1.819214 | 3.350319 | 2.13738 | 2.233445 | 2.261363 | 1.358358 | 0.988433 | 0.730171 | 2.024899 |
| O08530 | O08530 | S1PR1_M | S1pr1 | Sphingosine 1-phosphate receptor 1 | | | + | 0.589417 | 1.39003 | 1.568145 | 0.235886 | 0.285877 | 0.652762 | 1.675193 | -0.34506 | -6.77063 | -1.17014 | -0.82597 | -0.97299 | -1.0532 | -1.20732 | -1.1976 | -1.51853 | -3.25763 | -1.73508 | -0.86286 | -0.80667 | -0.90813 |
| Q9EP69 | Q9EP69 | SAC1_MO | Sacm1l | Phosphatidylinositol-3-phosphatase SAC1 | | | | 0.773715 | -0.58838 | -2.11778 | 0.164336 | 0.161811 | 0.469441 | 0.280725 | -0.14095 | -0.76558 | 0.166258 | 0.08783 | -0.34763 | -0.69765 | -0.23004 | -0.02163 | 0.990466 | 0.440375 | -0.38749 | -0.9814 | 0.166909 | -0.13666 |
| Q9JLC8 | Q9JLC8 | SACS_MO | Sacs | Sacsin | | | | 0.228738 | 0.368928 | 0.634678 | 0.491906 | 0.144365 | 1.303802 | 0.181591 | -0.18144 | -0.51423 | -1.1733 | -2.32651 | -1.4702 | -1.30716 | -1.86775 | -1.50522 | -2.19243 | -2.04524 | -1.45811 | -1.60798 | -1.80777 | -1.20231 |
| Q8BGH2 | Q8BGH2 | SAM50_M | Samm50 | Sorting and assembly machinery component 50 homolog | | | | 0.983299 | 0.608617 | 2.854846 | 0.287057 | 0.086433 | 0.781465 | 0.706856 | 0.411239 | 1.909469 | 2.082912 | 2.103286 | 1.555118 | 1.643252 | 2.140992 | 1.725227 | 1.697426 | 1.271538 | 1.614198 | 1.411306 | 1.465582 | 1.578158 |
| O55128 | O55128 | SAP18_M | Sap18 | Histone deacetylase complex subunit SAP18 | | | + | 0.096236 | 0.242224 | 0.286809 | 0.098486 | 0.165875 | 0.293035 | 2.027997 | 0.487742 | 10.25481 | -0.0131 | 1.654383 | 0.930228 | 2.002633 | 1.453477 | 1.525158 | 0.443723 | 0.713113 | 1.481966 | 1.119145 | 0.970309 | 1.032843 |
| Q02614 | Q02614 | S30BP_M | Sap30bp | SAP30-binding protein | | | | 0.805242 | 2.217016 | 2.220059 | 0.317595 | 0.255167 | 0.858028 | 0.319613 | -0.24218 | -0.86309 | -2.4179 | -0.45039 | 0.441755 | 0.712852 | 0.536279 | 0.210574 | -3.47942 | -3.8229 | 0.057436 | 0.586836 | 0.387102 | 0.844115 |
| P36536;Q | Q9CQC9;F | SAR1A_M | Sar1a;Sar1b | GTP-binding protein SAR1a | | | | 0.503955 | -1.87901 | -1.3357 | 0.086779 | 0.165805 | 0.260447 | 0.11555 | -0.07834 | -0.33977 | -0.5263 | -3.33804 | 0.962852 | -0.11375 | -0.10353 | 0.273452 | -0.00335 | -0.10297 | -0.08112 | 0.598613 | 0.030519 | 0.296087 |
| Q6PDS3 | Q6PDS3 | SARM1_M | Sarm1 | NAD(+) hydrolase SARM1 | | + | | 0.34561 | 0.191179 | 0.928372 | 1.168442 | 0.303512 | 3.640835 | 0.558017 | -0.20814 | -1.48139 | -0.6596 | -0.97255 | -0.23258 | -0.15612 | -0.46808 | -0.53422 | -1.14112 | -0.87338 | -0.57194 | -0.42378 | -0.42957 | -0.15646 |
| P26638 | P26638 | SYSC_MO | Sars1 | Serine--tRNA ligase, cytoplasmic | | | | 0.368074 | -0.61581 | -0.98498 | 0.880025 | 0.560366 | 2.474165 | 0.963989 | 0.182932 | 2.780824 | -0.7517 | -0.13107 | 0.879917 | 0.743658 | 0.55075 | 0.614026 | 0.717184 | -0.36833 | 0.035424 | 0.467418 | 0.34178 | 0.457131 |
| Q9JLI8 | Q9JLI8 | SART3_M | Sart3 | Squamous cell carcinoma antigen recognized by T-cells 3 | + | | | 1.168125 | -1.27299 | -3.63936 | 0.749665 | -0.40545 | -2.04155 | 0.556154 | 1.269427 | 1.476294 | -2.99742 | -2.41814 | -2.35215 | -1.95762 | -1.27424 | -1.45762 | -1.23868 | -1.6309 | -1.72648 | -1.7724 | -3.49033 | -1.78039 |
| P59808 | P59808 | SASH1_M | Sash1 | SAM and SH3 domain-containing protein 1 | | | | 0.000299 | -0.00191 | -0.00097 | 0.038398 | -0.03248 | -0.1201 | 0.028393 | -0.0628 | -0.08968 | -0.81939 | -4.24909 | -1.16673 | -0.63395 | -1.36491 | -0.66161 | -1.57549 | -3.48917 | -0.82118 | -0.91454 | -1.55605 | -0.34487 |
| Q6ZPE2 | Q6ZPE2 | MTMR5_M | Sbf1 | Myotubularin-related protein 5 | | | | 0.576091 | 0.435724 | 1.531122 | 0.242666 | -0.11032 | -0.66988 | 0.282299 | -0.25544 | -0.76953 | 0.333223 | 0.199636 | 1.74815 | 1.9876 | 1.559314 | 1.974979 | 0.107334 | -0.44592 | 1.865119 | 2.091262 | 1.763762 | 2.281408 |
| E9PXF8 | E9PXF8 | MTMRD_M | Sbf2 | Myotubularin-related protein 13 | | | | 0.8669 | 0.357642 | 2.428336 | 0.9055 | -0.59398 | -2.56469 | 0.93617 | -0.71551 | -2.67655 | -2.86206 | -3.15136 | -2.30473 | -2.55398 | -2.29574 | -2.82513 | -3.39204 | -3.33667 | -2.03059 | -1.64017 | -1.80752 | -1.88232 |
| Q8C8N2 | Q8C8N2 | SCAI_MO | Scai | Protein SCAI | | | | 0.246813 | 0.899546 | 0.680339 | 0.103408 | 0.139426 | 0.306606 | 0.378373 | 0.189487 | 1.011013 | -2.33859 | -1.87431 | -0.41745 | -1.29477 | -0.8779 | -0.80546 | -4.30766 | -1.70433 | -1.11539 | -0.87569 | -0.84728 | -1.21506 |
| Q8K021 | Q8K021 | SCAM1_M | Scamp1 | Secretory carrier-associated membrane protein 1 | | | | 0.117471 | -0.10809 | -0.34498 | 0.737669 | 1.494275 | 2.004078 | 0.70645 | 0.500662 | 1.908236 | -2.15393 | -1.64121 | -1.45953 | -1.94799 | -1.52843 | -1.01218 | -1.60935 | -1.96961 | -2.49355 | -3.90252 | -1.72396 | -1.81798 |
| O35609 | O35609 | SCAM3_M | Scamp3 | Secretory carrier-associated membrane protein 3 | | + | | 0.507663 | -0.46507 | -1.34556 | 1.199489 | -0.83237 | -3.78769 | 0.098763 | -0.14733 | -0.2938 | -1.69007 | -1.09797 | -2.13529 | -2.51602 | -1.11567 | -1.49599 | -0.10732 | -0.75058 | -1.38349 | -1.60308 | -0.69451 | -1.6225 |
| P28656 | P28656 | NP1L1_M | Nap1l1 | Nucleosome assembly protein 1-like 1 | + | | | 2.051137 | -0.629 | -10.5356 | 0.100033 | -0.12837 | -0.29731 | 0.108159 | -0.11663 | -0.31963 | -1.17683 | -1.11582 | -0.30746 | -0.91406 | -0.98824 | -1.32078 | -0.466 | -0.56865 | -0.78969 | -0.1751 | -0.71308 | -1.36267 |
| B2RXS3 | B2RXS3 | B2RXS3_M | Scaper | S phase cyclin A-associated protein in the ER | | | | 0.026021 | -0.08152 | -0.08239 | 0.334929 | 0.315508 | 0.901528 | 0.105852 | -0.17741 | -0.31332 | -1.86836 | -2.08589 | -0.41362 | -0.34006 | -1.8481 | -1.12523 | -0.91206 | -2.87915 | -1.04038 | -0.34431 | -0.87336 | -1.74515 |
| Q8R127 | Q8R127 | SCPDL_M | Sccpdh | Saccharopine dehydrogenase-like oxidoreductase | | | + | 0.140534 | 0.022728 | 0.406821 | 0.03424 | -0.03546 | -0.10753 | 1.087687 | 0.469362 | 3.280037 | 2.721704 | 2.611907 | 1.392656 | 1.390946 | 1.76038 | 1.710903 | 2.633715 | 2.65444 | 1.75705 | 1.097474 | 1.125337 | 1.407221 |
| P13011;P | P13516;P | ACOD1_M | Scd1;Scd2;Scd3 | Stearoyl-CoA desaturase 2 | | | | 0.354207 | -0.50193 | -0.95001 | 0.326863 | 0.934382 | 0.881279 | 0.511872 | 1.858748 | 1.356769 | 0.743865 | -0.27934 | -1.02984 | -1.3452 | -0.1061 | -0.68583 | 0.602248 | 0.866138 | -1.07344 | -3.17037 | -3.59367 | -0.91575 |
| Q8BRF7 | Q8BRF7 | SCFD1_M | Scfd1 | Sec1 family domain-containing protein 1 | | | | 0.300752 | 0.485597 | 0.815799 | 0.229654 | -0.23078 | -0.637 | 0.029375 | 0.066924 | 0.092691 | -3.78233 | -3.24436 | -3.43353 | -2.93013 | -3.8265 | -2.41896 | -3.46795 | -4.52994 | -3.21163 | -2.69048 | -3.02836 | -3.35095 |
| Q8BTY8 | Q8BTY8 | SCFD2_M | Scfd2 | Sec1 family domain-containing protein 2 | | | | 0.510371 | 0.316279 | 1.35277 | 0.282759 | -0.14615 | -0.77069 | 0.162221 | -0.12828 | -0.46392 | -0.47124 | -0.86272 | -1.53854 | -1.35878 | -1.02169 | -1.51921 | -0.8554 | -1.11112 | -1.13553 | -1.46949 | -1.26291 | -1.02142 |
| Q03517 | Q03517 | SCG2_MO | Scg2 | Secretogranin-2 | | + | | 0.754723 | 0.60058 | 2.057454 | 1.08483 | 1.989455 | 3.267806 | 0.565049 | 1.018439 | 1.500672 | -0.80201 | -0.38804 | 0.920592 | 0.140523 | -0.11502 | -1.03991 | -0.98977 | -1.40143 | -1.92635 | -0.99144 | -2.09261 | -1.09919 |
| Q99LC3 | Q99LC3 | NDUAA_M | Ndufa10 | NADH dehydrogenase [ubiquinone] 1 alpha subcomplex subunit 10, mitochondrial | + | | | 1.785417 | -0.63178 | -7.71469 | 0.275053 | -0.40457 | -0.75135 | 0.02606 | -0.05258 | -0.0825 | 1.460635 | 1.622103 | 1.14962 | 0.095004 | 1.620217 | 2.106499 | 2.186877 | 2.15942 | 0.917902 | 1.135854 | 2.505037 | 1.326839 |
| B1AWN6 | Q62205;A | SCN2A_M | Scn2a | Sodium channel protein type 2 subunit alpha | | + | | 0.184612 | 0.150881 | 0.522019 | 1.114587 | -0.36996 | -3.39695 | 0.213549 | 0.14712 | 0.596121 | 0.888103 | 0.769031 | 1.59294 | 1.410058 | 2.209081 | 1.721971 | 0.394851 | 0.960521 | 1.812301 | 1.930624 | 1.858273 | 1.778539 |
| Q56A07 | Q56A07 | SCN2B_M | Scn2b | Sodium channel subunit beta-2 | | | | 0.173607 | 0.255971 | 0.49356 | 0.70887 | -0.28431 | -1.91558 | 0.178504 | 0.067015 | 0.506246 | -0.61958 | -0.99451 | 0.37353 | 0.087776 | 0.297639 | 0.557821 | -1.54657 | -0.57946 | 0.555137 | 0.474783 | 0.336225 | 0.385205 |
| Q00612 | Q00612 | G6PD1_M | G6pdx | Glucose-6-phosphate 1-dehydrogenase X | + | + | | 1.792596 | -0.63368 | -7.78031 | 1.23576 | -0.9324 | -3.96537 | 0.370422 | 0.472927 | 0.990908 | -0.48387 | -0.43288 | -0.77653 | -0.98489 | -1.00279 | -0.28957 | 0.097949 | 0.252658 | 0.262479 | -0.15911 | -1.43631 | -0.80191 |
| Q9JJV9 | Q9JJV9 | SCN5A_M | Scn5a | Sodium channel protein type 5 subunit alpha | + | | | 1.26088 | -0.44999 | -4.09243 | 0.233776 | -0.222 | -0.64743 | 0.276841 | 1.360115 | 0.755839 | -3.26706 | -3.13032 | 0.053706 | 0.526383 | -0.26137 | 0.088212 | -2.66258 | -2.83482 | 0.760473 | 0.263609 | -3.23766 | 0.344274 |
| P32020 | P32020 | SCP2_MO | Scp2 | Sterol carrier protein 2 | | | | 0.003086 | -0.00972 | -0.01001 | 0.326263 | 1.241648 | 0.879772 | 0.166939 | 0.679574 | 0.476228 | -3.4238 | -1.93685 | 0.089633 | -0.50918 | -1.14107 | -0.86203 | -3.29526 | -2.04595 | -2.83063 | -0.07222 | -3.10128 | -0.26097 |
| O88745 | O88745 | SCRG1_M | Scrg1 | Scrapie-responsive protein 1 | | + | | 0.32134 | 1.140757 | 0.867422 | 1.719957 | 0.711549 | 7.140056 | 0.999208 | 0.876636 | 2.916872 | -0.69558 | -1.5105 | -0.20979 | -0.14171 | 0.207364 | -0.18203 | -0.9934 | -3.49419 | -0.98096 | -0.79363 | -1.09292 | -0.63502 |
| Q80U72 | Q80U72 | SCRIB_M | Scrib | Protein scribble homolog | | | + | NaN | 0 | NaN | 0.07866 | -0.47849 | -0.23757 | 2.117747 | -2.08892 | -11.3862 | -2.94214 | -3.7112 | -1.54742 | -4.50356 | -3.35983 | -3.24019 | -3.25176 | -3.34366 | -3.91519 | -1.17882 | -1.38452 | -1.03765 |
| Q9CZC8 | Q9CZC8 | SCRN1_M | Scrn1 | Secernin-1 | | | | 0.777089 | -0.96609 | -2.1286 | 0.237636 | -0.23815 | -0.65718 | 0.246273 | -0.35966 | -0.67898 | -1.23826 | -0.33137 | -0.06209 | -0.6873 | -1.09162 | -0.43102 | 0.161736 | 0.200818 | -0.31984 | 0.046749 | 0.012443 | -0.81577 |
| Q99L13 | Q99L13 | 3HIDH_M | Hibadh | 3-hydroxyisobutyrate dehydrogenase, mitochondrial | + | | | 1.368578 | -0.63503 | -4.67724 | 0.322822 | -0.53496 | -0.87114 | 0.795219 | 0.617535 | 2.187245 | -1.00423 | -0.73582 | -2.41087 | -2.86012 | -2.13062 | -1.7609 | -0.21445 | -0.25555 | -1.52899 | -2.67207 | -2.3499 | -2.77669 |
| P54071 | P54071 | IDHP_MO | Idh2 | Isocitrate dehydrogenase [NADP], mitochondrial | + | | | 1.605846 | -0.64021 | -6.2335 | 0.514328 | -0.85744 | -1.36332 | 0.08027 | 0.108075 | 0.242126 | -0.16525 | -0.13803 | -0.10123 | -1.3456 | 0.168765 | 0.907094 | 0.386768 | 0.590367 | 0.042169 | 0.225881 | 0.680762 | 0.178946 |
| Q8K2B3 | Q8K2B3 | SDHA_MO | Sdha | Succinate dehydrogenase [ubiquinone] flavoprotein subunit, mitochondrial | + | | | 1.03306 | 0.407258 | 3.05209 | 0.123184 | -0.12381 | -0.36042 | 0.30595 | 0.156379 | 0.82883 | 2.352461 | 2.508586 | 1.690326 | 1.004924 | 2.248373 | 1.972187 | 2.131485 | 1.915047 | 1.447793 | 1.495079 | 1.825338 | 2.082463 |
| Q9CQA3 | Q9CQA3 | SDHB_MO | Sdhb | Succinate dehydrogenase [ubiquinone] iron-sulfur subunit, mitochondrial | + | | | 1.044296 | 0.235251 | 3.09797 | 0.249952 | -0.21056 | -0.68825 | 0.958903 | 0.080487 | 2.761553 | 1.996948 | 2.017291 | 1.620798 | 1.042739 | 2.014718 | 1.966648 | 1.847121 | 1.696616 | 1.442028 | 1.642635 | 1.893711 | 1.926682 |
| Q9CZB0 | Q9CZB0 | C560_MO | Sdhc | Succinate dehydrogenase cytochrome b560 subunit, mitochondrial | | | | 0.588967 | -0.41179 | -1.56689 | 0.148643 | 0.394684 | 0.428278 | 0.320781 | 0.192993 | 0.866018 | -0.72352 | -0.19797 | -1.0077 | -2.65847 | -0.49585 | -0.88654 | -0.05319 | -0.04471 | -2.63766 | -1.81788 | -0.77694 | -0.99144 |
| Q5M8N4 | Q5M8N4 | D39U1_M | Sdr39u1 | Epimerase family protein SDR39U1 | | | | 0.516828 | -0.60212 | -1.37 | 0.126916 | 0.256359 | 0.370464 | 0.150073 | -0.23868 | -0.43205 | 0.041378 | -0.44143 | -0.7645 | -2.01182 | -2.14108 | -1.43817 | 0.034826 | 0.769371 | -1.34468 | -1.94436 | -1.12471 | -1.97718 |
| Q9D1M0 | Q9D1M0 | SEC13_M | Sec13 | Protein SEC13 homolog | | | | 0.513771 | 0.345388 | 1.361837 | 0.042562 | 0.039124 | 0.132601 | 0.465656 | -0.89088 | -1.23493 | -0.67368 | -1.16256 | 0.656899 | 1.240326 | -0.53267 | 0.710166 | -1.19591 | -1.33111 | 0.953758 | 0.865219 | 0.613217 | 1.346041 |
| E9QAT4 | E9QAT4 | SC16A_M | Sec16a | Protein transport protein Sec16A | + | | | 1.180589 | 0.741132 | 3.697732 | 0.119156 | -0.15476 | -0.34955 | 0.459545 | -0.96924 | -1.21902 | 0.001657 | -0.02731 | 1.91003 | 2.793786 | 0.757712 | 2.046295 | -0.55405 | -0.95386 | 2.534453 | 2.478889 | 1.905336 | 2.837144 |
| O08547 | O08547 | SC22B_M | Sec22b | Vesicle-trafficking protein SEC22b | | | | 0.720198 | -0.87732 | -1.95015 | 0.146445 | 0.182266 | 0.422474 | 0.253293 | 0.253402 | 0.696665 | -1.15839 | -0.59202 | -0.00546 | -0.79782 | -0.18616 | -0.73619 | 0.351669 | -0.34744 | -0.75468 | -0.41313 | -0.47652 | -0.95263 |
| Q01405 | Q01405 | SC23A_M | Sec23a | Protein transport protein Sec23A | | + | | 0.300377 | -0.12202 | -0.81486 | 1.156217 | -0.3554 | -3.58429 | 0.006708 | -0.00375 | -0.02168 | -0.74253 | -1.02281 | -0.21869 | -0.04045 | 0.026522 | 0.109934 | -0.8134 | -0.70791 | 0.18236 | 0.269298 | -0.09586 | 0.23981 |
| Q6NZC7 | Q6NZC7 | S23IP_MO | Sec23ip | SEC23-interacting protein | | | | 0.557746 | 1.276969 | 1.48065 | 0.377286 | -0.32892 | -1.00826 | 0.731762 | -0.25014 | -1.98576 | -1.17518 | -1.3515 | -0.21964 | -0.30869 | -0.29384 | -0.37925 | -1.68239 | -3.39823 | -0.25841 | 0.387935 | -0.20492 | 0.032094 |
| Q80ZX0 | Q80ZX0 | Q80ZX0_M | Sec24b | Sec24 related gene family, member B (S. cerevisiae) | | | | 0.280799 | -0.3063 | -0.76577 | 0.99412 | -0.22294 | -2.89693 | 0.267579 | 0.179089 | 0.732586 | -1.64677 | -0.87126 | -0.60678 | -0.71475 | -0.40549 | -0.88747 | -1.05087 | -0.85456 | -0.38298 | -0.49267 | -0.86664 | -0.7845 |
| G3X972 | G3X972 | G3X972_M | Sec24c | Sec24 related gene family, member C (S. cerevisiae) | | | | 0.075892 | 0.039565 | 0.229713 | 0.549173 | -0.42287 | -1.45725 | 0.109821 | -0.02266 | -0.32418 | -0.71817 | -0.55466 | -0.73756 | -0.39098 | -0.30712 | -0.1786 | -0.82758 | -0.52438 | -0.37416 | 0.091358 | -0.24774 | -0.19265 |
| Q3UPL0 | Q3UPL0 | SC31A_M | Sec31a | Protein transport protein Sec31A | | | | 0.475217 | -0.38899 | -1.25992 | 0.617414 | -0.59817 | -1.64698 | 0.713815 | 0.208323 | 1.930637 | -0.164 | -0.67325 | -0.73272 | -1.27059 | 0.001624 | -0.07083 | 0.144968 | -0.20424 | -0.64758 | -0.1594 | -0.14128 | -0.34457 |
| Q9JJR9 | Q9JJR9 | NRIP3_M | Nrip3 | Nuclear receptor-interacting protein 3 | + | | | 1.667667 | -0.6706 | -6.71031 | 0.641446 | -0.58858 | -1.71585 | 0.050085 | -0.05643 | -0.15496 | -3.7291 | -3.57677 | -3.86949 | -3.84573 | -2.21645 | -1.48823 | -3.04704 | -2.91763 | -3.61185 | -2.92621 | -1.79062 | -1.8012 |
| Q9JLR1 | Q9JLR1 | S61A2_M | Sec61a2 | Protein transport protein Sec61 subunit alpha isoform 2 | | | + | 0.672077 | 1.139321 | 1.805352 | 0.144272 | 0.225888 | 0.416727 | 2.740932 | 0.326494 | 23.43549 | 0.403159 | 0.372208 | 0.075679 | -0.32327 | -0.02833 | -0.04147 | -0.12075 | -1.38253 | 0.154335 | -0.8537 | -0.34911 | -0.37368 |
| Q9CQS8 | Q9CQS8 | SC61B_M | Sec61b | Protein transport protein Sec61 subunit beta | + | | | 1.091856 | 0.520805 | 3.29795 | 0.950155 | 0.296067 | 2.728625 | 0.272868 | -0.16483 | -0.74587 | 1.358436 | 1.129789 | 0.581131 | 0.463889 | 0.91109 | 0.51177 | 0.832249 | 0.614367 | 0.317749 | 0.135137 | 0.781542 | 0.970971 |
| Q8R2U0 | Q8R2U0 | SEH1_MO | Seh1l | Nucleoporin SEH1 | | | | 0.334896 | -0.51702 | -0.90145 | 0.833859 | 0.513342 | 2.315322 | 0.311713 | -0.45976 | -0.84328 | -3.78874 | -2.69263 | -1.48706 | -1.18413 | -2.46155 | -1.9616 | -2.55457 | -2.89275 | -2.01085 | -1.68703 | -2.23634 | -1.26729 |
| P17563;Q | P17563;Q | SBP1_MO | Selenbp1;Selenbp2 | Methanethiol oxidase | | | | 0.74347 | -0.34627 | -2.02215 | 0.694003 | -0.68766 | -1.87067 | 0.17912 | -0.32444 | -0.50784 | -1.16909 | -1.07787 | -1.26554 | -1.69228 | -1.17827 | -0.61458 | -0.94227 | -0.61216 | -1.09059 | -0.49191 | 0.00136 | -1.14532 |
| Q62178 | Q62178 | SEM4A_M | Sema4a | Semaphorin-4A | | | | 0.989107 | 1.440085 | 2.877381 | 0.950268 | 1.135182 | 2.729049 | 0.592981 | 1.25152 | 1.578103 | -0.82684 | -1.79067 | -1.01332 | -1.30848 | -1.62089 | -1.436 | -2.61378 | -2.88391 | -1.90718 | -2.68498 | -3.56762 | -1.99232 |
| Q8C1B7 | Q8CHH9;C | SEP11_M | Septin11 | Septin-11 | | | | 0.006845 | -0.00441 | -0.02212 | 0.862365 | -0.27246 | -2.41263 | 0.025412 | 0.017546 | 0.080505 | 1.156816 | 1.521382 | 2.898215 | 2.673157 | 2.589746 | 2.303114 | 1.424012 | 1.263 | 3.067657 | 3.048632 | 2.593084 | 2.264684 |
| P42208 | P42208 | SEPT2_M | Septin2 | Septin-2 | | | | 0.168199 | 0.091655 | 0.479509 | 0.240239 | -0.13901 | -0.66376 | 0.293627 | 0.192632 | 0.797938 | 0.262831 | 0.577322 | 0.422344 | 0.007882 | 0.493064 | 0.354809 | 0.21975 | 0.437093 | 0.323835 | 0.384416 | 0 | 0.462608 |
| Q9Z1S5 | Q9Z1S5 | SEPT3_M | Septin3 | Neuronal-specific septin-3 | | | | 0.00288 | -0.00245 | -0.00935 | 0.37321 | -0.11027 | -0.99795 | 0.077216 | -0.03132 | -0.23348 | 0.868845 | 1.174374 | 2.446997 | 2.270402 | 2.259467 | 2.401222 | 1.237257 | 0.810866 | 2.535403 | 2.402539 | 2.475577 | 2.247758 |
| Q3TZX3 | Q3TZX3;Q | S2533_M | Slc25a33 | Solute carrier family 25 member 33 | + | | | 1.866037 | -0.67535 | -8.48298 | 0.718182 | -0.30677 | -1.94398 | 0.157106 | 0.192287 | 0.450533 | -0.35399 | -0.32582 | -1.80424 | -1.49178 | -0.57178 | -1.23556 | 0.413807 | 0.257092 | -1.36348 | -1.319 | -0.82762 | -1.3643 |
| Q9Z2Q6 | Q9Z2Q6 | SEPT5_M | Septin5 | Septin-5 | | | | 0.388522 | -0.13728 | -1.03673 | 0.242744 | -0.12637 | -0.67008 | 0.31036 | -0.127 | -0.83989 | 1.730203 | 1.49668 | 3.651452 | 3.409671 | 3.233058 | 3.313756 | 1.813181 | 1.688264 | 3.512182 | 3.801687 | 3.254679 | 3.546139 |
| Q9R1T4 | Q9R1T4 | SEPT6_M | Septin6 | Septin-6 | | | | 0.116487 | -0.15027 | -0.34232 | 0.458895 | -0.45916 | -1.21733 | 0.246404 | 0.150214 | 0.67931 | -0.83827 | -0.09201 | 0.907157 | 0.164272 | 0.898724 | 0.590345 | -0.08362 | -0.54612 | 0.929299 | 1.060453 | 0.752823 | 0.435817 |
| O55131 | O55131 | SEPT7_M | Septin7 | Septin-7 | | | | 0.188335 | 0.222625 | 0.53161 | 0.023966 | -0.00996 | -0.07604 | 0.625783 | 0.227381 | 1.670838 | 1.933527 | 2.326849 | 3.368248 | 3.145058 | 3.145995 | 2.927862 | 2.277289 | 1.537838 | 3.198106 | 3.335113 | 2.890938 | 2.728158 |
| Q8CHH9 | Q8CHH9 | SEPT8_M | Septin8 | Septin-8 | | | | 0.975063 | -0.44469 | -2.82311 | 0.469191 | 0.283735 | 1.24416 | 0.617355 | 0.539528 | 1.646815 | -0.49221 | -0.54749 | 0.199618 | 0.433079 | 0.642946 | -0.00168 | 0.079921 | -0.23023 | -0.1633 | 0.228528 | -0.16016 | -0.27763 |
| Q80UG5 | Q80UG5 | SEPT9_M | Septin9 | Septin-9 | | | | 0.412471 | -0.29338 | -1.09771 | 0.095597 | -0.05713 | -0.28504 | 0.719793 | -0.07037 | -1.94891 | -0.40387 | -0.84382 | 0.993333 | 0.886044 | 1.355887 | 1.304821 | -0.17867 | -0.48226 | 0.803703 | 1.189932 | 1.426259 | 1.375195 |
| Q3U213 | Q3U213 | SRAC1_M | Serac1 | Protein SERAC1 | | | | 0.250384 | -1.69605 | -0.68934 | 0.089926 | 0.164834 | 0.269252 | 0.296043 | 0.164248 | 0.803994 | 1.385672 | -3.52223 | -0.1745 | 0.877897 | 0.556983 | 0.215401 | 0.449759 | 0.805784 | 0.499742 | -0.12602 | 0.334037 | 0.109852 |
| Q9CY58 | Q9CY58 | SERB1_M | Serbp1 | SERPINE1 mRNA-binding protein 1 | | | | 0.272664 | 1.225747 | 0.745355 | 0.36476 | 0.394865 | 0.976612 | 0.339445 | -0.44413 | -0.91287 | -0.78507 | -0.80601 | -0.39974 | -0.6609 | -1.01296 | -0.04005 | -3.66577 | -0.3768 | -1.30784 | -0.54253 | -0.07454 | -0.09021 |
| Q8BHJ6 | Q8BHJ6 | SERC5_M | Serinc5 | Serine incorporator 5 | | | | 0.699195 | -0.35467 | -1.8863 | 0.304217 | -0.75256 | -0.82449 | 0.65209 | -0.39843 | -1.74673 | -0.02017 | 0.233528 | -1.83435 | -2.41331 | -1.07695 | -1.27079 | 0.322559 | 0.600139 | -0.50562 | -2.23691 | -0.56895 | -0.98193 |
| P07758;P | P07758;P | A1AT1_M | Serpina1a;Serpina1b;Serpina1c;Serpina1d;Ser | Alpha-1-antitrypsin 1-1 | | | | 0.635261 | -2.44615 | -1.69802 | 0.171923 | -0.41007 | -0.48919 | 0.158771 | 0.298419 | 0.454894 | -1.04579 | -3.88698 | -0.38501 | -2.02872 | -0.91233 | 0.149717 | -0.25942 | 0.218944 | -0.63178 | -0.96181 | -1.06492 | -0.29453 |
| Q9D154 | Q9D154 | ILEUA_M | Serpinb1a | Leukocyte elastase inhibitor A | | | | 0.24566 | 0.120808 | 0.677433 | 0.920086 | -0.33837 | -2.61749 | 0.555734 | -0.21777 | -1.47515 | 2.213321 | 1.972439 | -0.39669 | -0.30446 | -0.03067 | 0.254197 | 1.840557 | 2.103588 | 0.108555 | -0.13298 | 0.36834 | 0.290725 |
| Q8VHP7 | Q8VHP7 | ILEUB_M | Serpinb1b | Leukocyte elastase inhibitor B | + | | | 1.169818 | -0.70846 | -3.64725 | NaN | 0 | | NaN | 0 | NaN | 0.333096 | 0.097851 | -3.35101 | -2.84251 | -3.05791 | -2.90195 | 0.769353 | 1.078524 | -3.59541 | -3.42882 | -2.78492 | -2.90237 |
| P70124 | P70124 | SPB5_MO | Serpinb5 | Serpin B5 | | | + | 0.915987 | -1.44912 | -2.60258 | 0.204268 | -1.20002 | -0.57246 | 1.340488 | -1.2256 | -4.51818 | -3.00809 | -4.10385 | -1.80504 | -3.71894 | -3.23712 | -3.77663 | -2.00757 | -2.20612 | -3.42706 | 0.303124 | -2.25273 | -2.30982 |
| Q60854 | Q60854 | SPB6_MO | Serpinb6 | Serpin B6 | | | | 0.454846 | 0.15166 | 1.20681 | 0.601079 | -0.34754 | -1.60081 | 0.800955 | -0.32308 | -2.20599 | 1.526462 | 1.275992 | 0.923894 | 1.098221 | 1.479092 | 1.412227 | 1.260016 | 1.239117 | 1.159762 | 1.557441 | 1.626149 | 1.911325 |
| O08797 | O08797 | O08797_M | Serpinb9 | SPI6 | | | | 0.667875 | -0.39877 | -1.79296 | 0.482154 | -0.69256 | -1.27812 | 0.102212 | -0.19416 | -0.30332 | -3.16742 | -3.55093 | -2.97487 | -1.93242 | -2.51303 | -1.2337 | -3.07309 | -2.84772 | -1.9092 | -1.61296 | -1.6545 | -1.7039 |
| P19324 | P19324 | SERPH_M | Serpinh1 | Serpin H1 | | + | | NaN | 0 | NaN | 2.618388 | 0.454481 | 20.34269 | 0.233553 | 0.564774 | 0.646864 | -3.44529 | -3.65634 | -2.91298 | -2.90318 | -1.91568 | -3.56482 | -3.55066 | -3.1132 | -3.38488 | -3.34124 | -3.59204 | -3.01801 |
| Q80UK0 | Q80UK0 | SESD1_M | Sestd1 | SEC14 domain and spectrin repeat-containing protein 1 | | | | 0.344793 | 0.436647 | 0.926318 | 0.110257 | 0.100653 | 0.325368 | 0.811453 | -0.91108 | -2.24054 | -0.58016 | -1.40662 | 0.207254 | 0.598612 | -0.97567 | -0.46663 | -1.20324 | -1.65683 | 0.062678 | 0.541881 | -0.1272 | 0.507065 |
| Q9EQU5 | Q9EQU5 | SET_MOU | Set | Protein SET | | | | 0.508651 | -0.98163 | -1.34819 | 0.150367 | -0.26073 | -0.43282 | 0.049683 | 0.039374 | 0.153774 | -3.77214 | -3.96813 | -2.55907 | -2.283 | -2.99803 | -3.28649 | -2.16702 | -3.60999 | -1.57392 | -2.74668 | -3.39321 | -2.97006 |
| Q8K4Z5 | Q8K4Z5 | SF3A1_M | Sf3a1 | Splicing factor 3A subunit 1 | | | | 0.19129 | 0.121938 | 0.539207 | 0.004393 | 0.004907 | 0.014233 | 0.220945 | 0.247893 | 0.61492 | 1.56073 | 1.174866 | 0.595518 | 0.855918 | 1.176613 | 0.402492 | 1.363836 | 1.127882 | 1.040036 | 0.401586 | 0.42897 | 0.654348 |
| Q99NB9 | Q99NB9 | SF3B1_M | Sf3b1 | Splicing factor 3B subunit 1 | | | + | 0.301233 | 0.66665 | 0.817006 | 0.209045 | 0.270283 | 0.584646 | 1.020299 | 0.66279 | 3.000591 | -2.2666 | -1.6772 | -1.58431 | -0.72724 | -0.61652 | -0.99623 | -1.87766 | -3.39944 | -1.25262 | -1.5995 | -1.58206 | -1.35627 |
| Q3UJB0 | Q3UJB0 | Q3UJB0_M | Sf3b2 | Splicing factor 3b, subunit 2 | | | | 0.010569 | 0.018973 | 0.034012 | 0.149248 | -0.05656 | -0.42987 | 0.773475 | 0.673428 | 2.117012 | -0.99849 | -1.03536 | -0.50859 | -0.42235 | 0.265132 | -0.33909 | -0.47837 | -1.59343 | -0.2846 | -0.53322 | -0.61082 | -0.81 |
| Q921M3 | Q921M3 | SF3B3_M | Sf3b3 | Splicing factor 3B subunit 3 | | | | 0.492129 | 0.912542 | 1.304391 | 0.158212 | 0.168869 | 0.453431 | 0.409526 | 0.221914 | 1.090186 | -2.09446 | -0.87989 | -0.67766 | -0.4668 | -0.63395 | -0.82255 | -2.05239 | -2.74705 | -1.09829 | -0.38391 | -1.13056 | -0.76977 |
| P59708 | P59708 | SF3B6_M | Sf3b6 | Splicing factor 3B subunit 6 | | | + | 0.012833 | -0.04221 | -0.04119 | 0.037825 | 0.076782 | 0.118372 | 1.321397 | -0.67841 | -4.41277 | -3.45872 | -2.16476 | -3.2867 | -2.03357 | -2.71297 | -2.881 | -3.56406 | -1.97501 | -2.56912 | -2.90472 | -1.98983 | -2.24732 |
| Q9JM63 | Q9JM63 | KCJ10_M | Kcnj10 | ATP-sensitive inward rectifier potassium channel 10 | + | | | 1.437608 | -0.6828 | -5.08923 | 0.384482 | -0.26499 | -1.02649 | 0.472989 | 0.272317 | 1.254089 | 1.321746 | 1.159228 | -0.25997 | 0.065289 | 0.421656 | -0.01118 | 2.03005 | 1.816532 | 0.36815 | -0.03284 | -0.08479 | -0.04937 |
| Q8VIJ6 | Q8VIJ6 | SFPQ_MO | Sfpq | Splicing factor, proline- and glutamine-rich | | | | 0.10425 | -0.03606 | -0.30892 | 0.033459 | -0.02957 | -0.10515 | 0.384298 | 0.359433 | 1.02602 | 1.711123 | 1.482213 | 1.732234 | 1.445148 | 2.522029 | 1.91363 | 1.60973 | 1.655735 | 1.860084 | 1.376439 | 2.032135 | 1.684658 |
| Q99JR1 | Q99JR1 | SFXN1_M | Sfxn1 | Sideroflexin-1 | | | | 0.287888 | -0.10584 | -0.78355 | 0.440602 | -0.47797 | -1.16994 | 0.259946 | 0.263276 | 0.713404 | -0.30436 | -0.21087 | -0.37333 | -0.97498 | 0.470089 | 0.477965 | -0.02503 | -0.27851 | 0.080236 | -0.47261 | 0.579772 | -0.15827 |
| Q9QZ08 | Q9QZ08 | NAGK_MO | Nagk | N-acetyl-D-glucosamine kinase | + | | | 1.596124 | -0.68593 | -6.16145 | 0.052449 | 0.036067 | 0.161933 | 0.186922 | 0.169572 | 0.527973 | -0.88961 | -0.83128 | -0.51867 | -0.47817 | -0.519 | 0.110607 | -0.28195 | -0.06708 | -0.75629 | -0.31268 | -0.43743 | -0.31011 |
| Q925N0 | Q925N0 | SFXN5_M | Sfxn5 | Sideroflexin-5 | | | | 0.573533 | -0.3752 | -1.52405 | 0.124532 | -0.12125 | -0.36405 | 0.616798 | 0.408594 | 1.645233 | 2.574539 | 2.537033 | 1.367201 | 0.969725 | 2.208579 | 1.820078 | 3.176455 | 2.685514 | 1.556959 | 1.02246 | 1.760475 | 1.450994 |
| Q8VD37 | Q8VD37 | SGIP1_M | Sgip1 | SH3-containing GRB2-like protein 3-interacting protein 1 | | | | 0.28822 | 0.507062 | 0.784383 | 0.017492 | -0.01801 | -0.05587 | 0.159157 | -0.07775 | -0.45591 | 0.174864 | -0.94899 | 0.078384 | -0.25717 | 0.276175 | -0.06357 | -1.2137 | -0.57455 | -0.34657 | 0.203804 | 0.169088 | 0.199007 |
| Q8BPQ7 | Q8BPQ7 | SGSM1_M | Sgsm1 | Small G protein signaling modulator 1 | + | | | 1.167925 | 0.784541 | 3.638427 | 0.244439 | 0.218927 | 0.674354 | 0.240122 | 0.103831 | 0.663461 | -0.87161 | -1.30234 | -0.12737 | 0.307798 | -0.23597 | -0.34083 | -1.86094 | -1.88209 | -0.36965 | 0.112233 | -0.53969 | -0.24477 |
| Q80U12 | Q80U12 | SGSM2_M | Sgsm2 | Small G protein signaling modulator 2 | | | | NaN | 0 | NaN | 0.114541 | 0.376146 | 0.337035 | 0.052822 | -0.10614 | -0.16303 | -3.35376 | -3.15219 | -4.16052 | -2.15027 | -2.44022 | -1.87861 | -2.72347 | -3.73941 | -4.01659 | -3.0465 | -2.64064 | -1.46591 |
| Q8BJU0 | Q8BJU0 | SGTA_MO | Sgta | Small glutamine-rich tetratricopeptide repeat-containing protein alpha | | | | 0.252459 | 0.196849 | 0.694565 | 0.056984 | 0.040268 | 0.175231 | 0.00826 | -0.01246 | -0.02665 | -0.92367 | -0.39766 | 0.0209 | -0.43853 | -0.62436 | -0.16203 | -0.96312 | -0.75192 | -0.24278 | -0.25539 | 0.02586 | -0.78732 |
| Q8VD33 | Q8VD33 | SGTB_MO | Sgtb | Small glutamine-rich tetratricopeptide repeat-containing protein beta | | | | 0.313739 | -0.20473 | -0.84836 | 0.431746 | -0.5094 | -1.14712 | 0.306178 | -0.62534 | -0.8294 | -1.27898 | -1.73909 | -1.52413 | -1.2691 | -2.18585 | -2.26215 | -1.37718 | -1.23142 | -1.03192 | -1.6425 | -0.84565 | -2.35166 |
| Q91ZM2 | Q91ZM2 | SH2B1_M | Sh2b1 | SH2B adapter protein 1 | | + | | 0.992596 | 0.963494 | 2.890976 | 1.114699 | 0.323589 | 3.397439 | 0.643633 | -0.28688 | -1.72218 | 3.055303 | 2.445067 | 1.648254 | 1.81417 | 0.60871 | 0.881193 | 1.920766 | 1.652616 | 1.454416 | 1.36083 | 0.935982 | 1.127686 |
| Q8JZW5 | Q8JZW5 | SH2D5_M | Sh2d5 | SH2 domain-containing protein 5 | | + | | 0.383364 | 1.858423 | 1.023653 | 1.253187 | 1.11126 | 4.053161 | 0.162489 | 0.145661 | 0.464619 | 0.148616 | -3.46307 | 1.808385 | 2.324234 | 0.925852 | 0.637053 | -3.70246 | -3.32884 | 0.862072 | 1.048028 | 0.357521 | 0.914063 |
| Q9JJU8 | Q9JJU8 | SH3L1_M | Sh3bgrl | Adapter Sh3bgrl | | | | 0.053132 | 0.085644 | 0.163941 | 0.724101 | -0.28678 | -1.96213 | 0.398087 | -0.46911 | -1.06103 | -0.46154 | -1.29032 | -0.61289 | -0.53423 | -0.34414 | 0.48775 | -1.27967 | -0.64348 | -0.14601 | -0.42755 | 0.690811 | 0.391026 |
| Q8BG73 | Q8BG73 | SH3L2_M | Sh3bgrl2 | SH3 domain-binding glutamic acid-rich-like protein 2 | | | | 0.123045 | 0.325906 | 0.360048 | 0.378827 | -0.55515 | -1.01216 | 0.003441 | -0.00495 | -0.01116 | 0.139946 | -0.68274 | -1.41144 | -1.96898 | -0.74737 | 0.004548 | -1.40361 | 0.20901 | -1.60741 | -0.66271 | -0.13132 | -0.60161 |
| Q91VW3 | Q91VW3 | SH3L3_M | Sh3bgrl3 | SH3 domain-binding glutamic acid-rich-like protein 3 | | | | 0.357348 | -0.99933 | -0.95792 | 0.019567 | 0.118757 | 0.062364 | 0.182853 | 0.451287 | 0.517481 | -2.96677 | -0.88609 | 0.411036 | -3.39742 | -0.69554 | 0.048007 | -0.84961 | -1.0046 | -1.60073 | -1.62316 | 0.013817 | -1.56392 |
| P55194 | P55194 | 3BP1_MO | Sh3bp1 | SH3 domain-binding protein 1 | | | | 0.277021 | 1.25315 | 0.75629 | 0.337105 | -0.27729 | -0.90699 | 0.378886 | 1.7702 | 1.012311 | -0.36556 | -0.4429 | -0.28817 | 0.25182 | -0.40517 | 0.146204 | -0.00087 | -3.3139 | 0.115687 | 0.402549 | -3.62649 | -0.17288 |
| Q62419 | Q62419 | SH3G1_M | Sh3gl1 | Endophilin-A2 | | | | 0.64445 | 0.912079 | 1.724543 | 0.273889 | -0.31409 | -0.74843 | 0.526159 | 0.202377 | 1.395012 | -1.63853 | -1.33479 | 0.325641 | 0.02231 | 0.294798 | 0.572088 | -1.89213 | -2.90535 | 0.096767 | 0.879358 | 0.273767 | 0.188364 |
| Q62420 | Q62420;Q | SH3G2_M | Sh3gl2 | Endophilin-A1 | | | | 0.008233 | -0.00819 | -0.02656 | 0.000929 | -0.00204 | -0.00302 | 0.150607 | 0.088824 | 0.433454 | 0.711286 | 1.234249 | 2.609213 | 1.261027 | 2.128617 | 2.205785 | 0.817654 | 1.144259 | 1.962255 | 1.912062 | 2.279634 | 1.877121 |
| Q9JK48 | Q9JK48 | SHLB1_M | Sh3glb1 | Endophilin-B1 | | | | 0.002113 | -0.00641 | -0.00686 | 0.522716 | -0.20076 | -1.38577 | 0.601788 | 0.410736 | 1.602803 | -1.37462 | -1.7897 | -0.90237 | -0.80274 | -0.97329 | -0.48469 | -2.48672 | -0.66477 | -0.51576 | -0.78783 | -1.06235 | -1.21709 |
| Q8R3V5 | Q8R3V5 | SHLB2_M | Sh3glb2 | Endophilin-B2 | | | | 0.642144 | -0.13739 | -1.71787 | 0.685278 | -0.27495 | -1.84455 | 0.632296 | -0.30905 | -1.6895 | 0.610593 | 0.59706 | 0.966929 | 0.688444 | 0.46927 | 0.805214 | 0.661528 | 0.820911 | 1.155829 | 1.049435 | 1.018722 | 0.873859 |
| Q8R550 | Q8R550 | SH3K1_M | Sh3kbp1 | SH3 domain-containing kinase-binding protein 1 | | + | | 0.041394 | 0.110169 | 0.129105 | 1.020964 | -0.37169 | -3.00326 | 0.584957 | -0.69274 | -1.55572 | -0.67093 | -2.15833 | 0.233163 | 0.447631 | -1.2011 | -0.77766 | -1.10637 | -1.94322 | 0.650299 | 0.773884 | -0.68838 | 0.095085 |
| P70195 | P70195 | PSB7_MO | Psmb7 | Proteasome subunit beta type-7 | + | | | 1.521152 | -0.6934 | -5.63105 | 0.513923 | -0.46107 | -1.36224 | 0.084212 | 0.092619 | 0.253239 | 1.81747 | 1.954414 | 1.834342 | 1.189695 | 1.846626 | 2.557775 | 2.476993 | 2.681683 | 1.86981 | 2.076374 | 2.02396 | 2.195203 |
| Q80Z38 | Q80Z38 | SHAN2_M | Shank2 | SH3 and multiple ankyrin repeat domains protein 2 | | + | | 0.369352 | 0.630615 | 0.988204 | 1.442769 | -0.78361 | -5.12129 | 0.289669 | -0.10887 | -0.78802 | 1.030746 | 2.273794 | 2.826358 | 3.02141 | 3.676168 | 3.853425 | 1.166342 | 0.876967 | 3.589591 | 3.825393 | 3.979639 | 3.767686 |
| Q4ACU6 | Q4ACU6 | SHAN3_M | Shank3 | SH3 and multiple ankyrin repeat domains protein 3 | + | | | 1.094122 | 1.274194 | 3.307717 | 0.153467 | 0.055172 | 0.44098 | 0.079074 | 0.095188 | 0.238742 | -0.46282 | -0.74032 | 1.553703 | 1.56234 | 1.503874 | 0.820036 | -1.5164 | -2.23513 | 1.377811 | 1.627887 | 0.861683 | 1.271851 |
| Q6PD21 | Q6PD21 | SHB_MOU | Shb | SH2 domain-containing adapter protein B | | | | NaN | 0 | NaN | 0.280884 | 0.467425 | 0.765983 | 0.477974 | -0.5133 | -1.26715 | -3.10637 | -3.88507 | -2.46174 | -1.61004 | -3.25977 | -4.06651 | -3.60179 | -4.00624 | -2.94039 | -2.06625 | -3.18709 | -3.11258 |
| P98083 | P98083;Q | SHC1_MO | Shc1 | SHC-transforming protein 1 | | | | NaN | 0 | NaN | 0.00405 | 0.007255 | 0.013127 | 0.052301 | 0.07426 | 0.161498 | -3.00707 | -2.87962 | -2.92501 | -3.49072 | -3.6716 | -2.8906 | -2.71173 | -3.32061 | -3.68988 | -2.74035 | -3.11258 | -3.59814 |
| Q61120 | Q61120 | SHC3_MO | Shc3 | SHC-transforming protein 3 | | | | 0.033794 | -0.2415 | -0.10617 | 0.109899 | -0.04075 | -0.32439 | 0.173313 | 0.140219 | 0.492797 | -0.2374 | -3.0933 | -0.54331 | -0.55362 | -1.02808 | -0.69646 | -3.19441 | 0.346709 | -0.63323 | -0.38219 | -1.23372 | -0.77125 |
| Q8CA71 | Q8CA71 | SHSA4_M | Shisa4 | Protein shisa-4 | | | | 0.034128 | -0.15302 | -0.10718 | 0.178857 | 0.65768 | 0.507158 | 0.019508 | 0.030883 | 0.062179 | -2.81187 | -1.65942 | -1.92954 | -2.33314 | -2.94515 | -2.01612 | -3.38884 | -0.7764 | -4.07002 | -1.50802 | -2.68735 | -2.33569 |
| Q3UH99 | Q3UH99 | SHSA6_M | Shisa6 | Protein shisa-6 | | | | 0.110346 | 0.299732 | 0.32561 | 0.155737 | 0.238926 | 0.446943 | 0.496436 | -0.57183 | -1.31577 | -1.00631 | 0.758385 | 0.731183 | 1.758528 | 2.94043 | 3.582883 | -0.16134 | -0.68605 | 0.857895 | 1.153964 | 4.126213 | 3.540762 |
| Q8C3Q5 | Q8C3Q5 | SHSA7_M | Shisa7 | Protein shisa-7 | | | | 0.415812 | 1.313891 | 1.10625 | 0.815472 | 0.859544 | 2.253843 | 0.235928 | -0.32267 | -0.65287 | -0.95481 | -3.32993 | 3.141972 | 3.876858 | 2.634121 | 1.771776 | -3.47426 | -3.43826 | 2.751986 | 2.547756 | 2.284041 | 2.767196 |
| A2ALU4 | A2ALU4 | SHRM2_M | Shroom2 | Protein Shroom2 | | | | 0.167715 | -0.62652 | -0.47825 | 0.987323 | -0.37794 | -2.87045 | 0.45282 | -0.13984 | -1.20155 | -1.85802 | -3.9854 | -0.38254 | -0.52178 | -0.71308 | -0.48152 | -1.53051 | -3.05987 | -0.18597 | 0.037535 | -0.46923 | -0.4457 |
| Q9CPR7 | Q9CPR7 | SIKE1_MO | Sike1 | Suppressor of IKBKE 1 | | | | 0.518577 | 1.470536 | 1.374681 | 0.21817 | 0.311964 | 0.607873 | 0.491437 | -1.26253 | -1.30256 | -2.93741 | -0.86733 | -0.98192 | 0.029544 | -3.23049 | -1.3656 | -3.64311 | -3.1027 | -0.70089 | -0.87542 | -1.30013 | -0.77091 |
| Q60520 | Q60520 | SIN3A_MO | Sin3a | Paired amphipathic helix protein Sin3a | | | | NaN | 0 | NaN | 0.504184 | 0.605882 | 1.336312 | NaN | 0 | NaN | -4.15632 | -4.42181 | -2.9555 | -3.08204 | -2.77348 | -3.5021 | -2.42449 | -3.39596 | -3.17569 | -4.07361 | -3.33813 | -3.20573 |
| Q8C0T5 | Q80TE4;Q | SI1L1_MO | Sipa1l1 | Signal-induced proliferation-associated 1-like protein 1 | | + | | 0.075533 | 0.0822 | 0.228694 | 1.404118 | -0.39593 | -4.88551 | 0.162694 | -0.33927 | -0.46516 | -1.75871 | -1.14895 | 0.596744 | 0.725069 | 0.071397 | 1.450151 | -1.34567 | -1.72639 | 1.007332 | 1.106349 | 1.338202 | 0.861876 |
| Q80TE4 | Q80TE4 | SI1L2_MO | Sipa1l2 | Signal-induced proliferation-associated 1-like protein 2 | | | | 0.301935 | -0.28661 | -0.81877 | 0.457044 | 0.154886 | 1.212519 | 0.838546 | -0.84413 | -2.33115 | -1.00584 | -0.35679 | 0.107078 | 0.212019 | -0.99934 | -0.45762 | -0.52592 | -0.2635 | -0.1118 | 0.121128 | -0.12468 | 0.355972 |
| G3X9J0 | G3X9J0 | SI1L3_MO | Sipa1l3 | Signal-induced proliferation-associated 1-like protein 3 | | | | 0.540041 | -0.38657 | -1.43246 | 0.138306 | 0.053682 | 0.400899 | 0.020233 | 0.029459 | 0.064441 | -3.61857 | -3.72222 | -2.05111 | -2.31067 | -0.95866 | -0.11839 | -3.54866 | -3.01898 | -2.2016 | -2.26754 | -0.38778 | -0.74818 |
| P97797 | P97797 | SHPS1_M | Sirpa | Tyrosine-protein phosphatase non-receptor type substrate 1 | | | | 0.26334 | 0.43514 | 0.721935 | 0.154415 | -0.42099 | -0.44347 | 0.200147 | -0.14349 | -0.56192 | -0.74868 | 0.403024 | 1.592207 | -0.19026 | 0.510448 | 0.632308 | -0.42994 | -0.78599 | 0.795001 | 1.448938 | 0.962853 | 0.466886 |
| Q9DBP5 | Q9DBP5 | KCY_MOU | Cmpk1 | UMP-CMP kinase | + | | | 1.304863 | -0.69351 | -4.3232 | 0.238402 | 0.452426 | 0.659117 | 0.048936 | 0.096424 | 0.151566 | -0.96253 | -0.80949 | 0.359637 | -1.01054 | -0.86234 | -0.37901 | -0.05152 | -0.33349 | -0.73525 | -0.82051 | -0.1286 | -1.30559 |
| Q9WTX5 | Q9WTX5 | SKP1_MO | Skp1 | S-phase kinase-associated protein 1 | | + | | 0.62516 | 0.238074 | 1.669057 | 2.028509 | 0.24764 | 10.26094 | 0.039202 | -0.02558 | -0.12252 | 3.360897 | 3.34518 | 3.598428 | 3.644158 | 3.265488 | 3.669693 | 2.972542 | 3.257388 | 3.36593 | 3.381377 | 3.440814 | 3.545525 |
| A2AQ25 | A2AQ25 | SKT_MOU | Skt | Sickle tail protein | | + | | 0.50233 | 0.420012 | 1.33139 | 1.456495 | 0.451428 | 5.207439 | 0.441098 | -0.56144 | -1.17122 | 0.337992 | -0.27266 | 3.04507 | 3.211651 | 0.882504 | 1.200123 | -0.30799 | -0.4667 | 2.700968 | 2.652897 | 1.150461 | 2.055046 |
| Q9CR95 | Q9CR95 | NECP1_M | Necap1 | Adaptin ear-binding coat-associated protein 1 | + | | | 2.260128 | -0.70496 | -13.436 | 0.31194 | 0.694029 | 0.843848 | 0.222032 | 0.308435 | 0.617678 | -1.01311 | -1.09245 | 0.67412 | -0.94831 | 0.190122 | -0.39952 | -0.31348 | -0.38216 | -0.6956 | -0.96665 | -0.01012 | -0.81616 |
| Q91V14 | Q91V14 | S12A5_M | Slc12a5 | Solute carrier family 12 member 5 | | + | | 0.059396 | 0.036446 | 0.182266 | 1.015987 | -0.73197 | -2.98334 | 0.058617 | 0.047824 | 0.179998 | 4.50816 | 4.763586 | 4.618366 | 4.130948 | 5.145844 | 4.680741 | 4.753288 | 4.445566 | 5.078267 | 5.134992 | 4.99397 | 4.736966 |
| P63005 | P63005 | LIS1_MO | Pafah1b1 | Platelet-activating factor acetylhydrolase IB subunit beta | + | | | 1.518024 | -0.71556 | -5.60985 | 0.655139 | -0.88728 | -1.75562 | 0.171115 | -0.25913 | -0.48709 | -0.21893 | -0.30014 | 0.822312 | -0.11354 | 0.213101 | 1.272974 | 0.576941 | 0.335105 | 1.050701 | 1.432622 | 1.048847 | 0.955482 |
| P53986 | P53986 | MOT1_MO | Slc16a1 | Monocarboxylate transporter 1 | | | | 0.349511 | -0.35277 | -0.93819 | 0.84722 | 0.355394 | 2.360623 | 0.427114 | 0.335469 | 1.135217 | 0.961579 | 1.678109 | 0.306625 | 0.604409 | 0.842767 | 0.580161 | 1.786782 | 1.55845 | 0.12241 | 0.077836 | 0.640733 | 0.111258 |
| O70451 | O70451 | MOT2_MO | Slc16a7 | Monocarboxylate transporter 2 | | | | 0.033552 | 0.177172 | 0.105434 | 0.039672 | 0.030239 | 0.123935 | 0.15468 | 0.245463 | 0.444166 | -3.41039 | -0.85936 | -0.08842 | -0.52724 | 0.377892 | -0.56074 | -1.21805 | -3.40604 | -0.44481 | -0.23133 | -0.62869 | -0.04508 |
| Q8BLE7 | Q8BLE7 | VGLU2_M | Slc17a6 | Vesicular glutamate transporter 2 | | | | 0.14967 | 0.163887 | 0.430985 | 0.919672 | 0.494741 | 2.615981 | 0.001556 | -0.00214 | -0.00506 | 1.444854 | 1.155406 | 0.580052 | 0.908494 | 0.866327 | 0.168793 | 1.487888 | 0.784598 | 0.15573 | 0.343334 | 0.279202 | 0.760204 |
| Q3TXX4 | Q3TXX4 | VGLU1_M | Slc17a7 | Vesicular glutamate transporter 1 | | | | 0.105999 | 0.221676 | 0.31372 | 0.244843 | -0.27349 | -0.67537 | 0.771322 | 0.632424 | 2.110127 | 0.594493 | 1.815683 | 4.027586 | 3.218119 | 4.454877 | 4.781425 | 0.627799 | 1.339026 | 3.883402 | 3.909273 | 4.237058 | 3.734397 |
| P43006 | P43006 | EAA2_MO | Slc1a2 | Excitatory amino acid transporter 2 | + | | | 1.192745 | 0.312251 | 3.755386 | 0.159127 | 0.068552 | 0.455828 | 0.770485 | 0.241313 | 2.107451 | 3.94328 | 3.849761 | 4.363657 | 4.068374 | 4.924994 | 4.820714 | 3.653023 | 3.515516 | 4.118847 | 4.17608 | 4.733486 | 4.529596 |
| P56564 | O35544;P | EAA1_MO | Slc1a3 | Excitatory amino acid transporter 1 | | | | 0.615752 | 2.265391 | 1.642262 | 0.369479 | 0.904339 | 0.988527 | 0.284303 | 0.377557 | 0.774561 | -0.03943 | 1.414916 | 2.589811 | 2.6448 | 2.448702 | 1.639459 | -2.74985 | -0.40544 | 0.798544 | 2.627388 | 1.394704 | 1.938343 |
| O35874 | O35874 | SATT_MO | Slc1a4 | Neutral amino acid transporter A | | | | 0.213521 | -0.48993 | -0.59605 | 0.039979 | 0.14826 | 0.124857 | 0.105739 | 0.268017 | 0.313007 | -2.63321 | -3.4249 | -2.11678 | -3.10347 | -1.96823 | -1.54197 | -3.25949 | -1.81876 | -3.83849 | -1.67828 | -1.1938 | -2.85243 |
| Q8BUN9 | Q8BUN9 | NCKX2_M | Slc24a2 | Sodium/potassium/calcium exchanger 2 | | | | 0.699601 | -0.26927 | -1.88752 | 0.428548 | -0.33407 | -1.1389 | 0.115357 | 0.170231 | 0.339252 | 0.516072 | 0.370988 | -0.12852 | -0.08638 | 1.075271 | 0.512382 | 0.589964 | 0.835642 | 0.519193 | -0.06595 | 1.039018 | 0.208173 |
| Q8JZU2 | Q8JZU2 | TXTP_MO | Slc25a1 | Tricarboxylate transport protein, mitochondrial | | | | 0.827287 | -0.45718 | -2.79323 | 0.188343 | 0.047169 | 0.53163 | 0.400921 | 0.270654 | 1.068247 | 2.661832 | 2.302891 | 1.230737 | 1.317029 | 2.163112 | 1.667235 | 2.54122 | 2.937788 | 1.304243 | 1.149185 | 1.592371 | 1.696667 |
| Q9QZD8 | Q9QZD8 | DIC_MOU | Slc25a10 | Mitochondrial dicarboxylate carrier | | | | 0.35342 | 0.10254 | 0.948028 | 0.225393 | 0.194788 | 0.626204 | 0.306222 | 0.325256 | 0.829511 | 1.726696 | 1.702337 | 0.542756 | 0.607067 | 1.385744 | 0.76745 | 1.71945 | 1.504504 | 0.689518 | 0.070728 | 0.510146 | 0.992536 |
| Q9CR62 | Q9CR62 | M2OM_M | Slc25a11 | Mitochondrial 2-oxoglutarate/malate carrier protein | | | + | 0.508891 | -0.12344 | -1.34883 | 0.064127 | 0.033178 | 0.195991 | 1.106978 | 0.354687 | 3.363556 | 4.104208 | 3.921252 | 3.700597 | 3.530413 | 4.559548 | 4.379199 | 4.138749 | 4.133586 | 3.728668 | 3.435987 | 4.060021 | 4.169351 |

| | | | | | | | | | | | | | | | | | | | | | | | | | | | | |
|---|---|---|---|---|---|---|---|---|---|---|---|---|---|---|---|---|---|---|---|---|---|---|---|---|---|---|---|---|
| Q8BH59 | Q8BH59 | S2512_MC | Slc25a12 | Electrogenic aspartate/glutamate antiporter SLC25A12, mitochondrial | | | | 0.217082 | -0.09995 | -0.60511 | 0.266359 | 0.048631 | 0.729521 | 0.279713 | 0.204614 | 0.763047 | 4.122764 | 3.865575 | 3.807884 | 3.674562 | 4.752741 | 4.269977 | 4.197803 | 3.990445 | 3.692227 | 3.692955 | 4.189949 | 4.423541 |
| Q9QXX4 | Q9QXX4 | S2513_MC | Slc25a13 | Electrogenic aspartate/glutamate antiporter SLC25A13, mitochondrial | | + | | 0.371007 | 0.373515 | 0.992385 | 1.133473 | -0.39787 | -3.48095 | 0.03166 | 0.093085 | 0.099676 | -1.5451 | -1.69743 | -2.20834 | -2.00295 | -0.94272 | -2.21648 | -2.36337 | -1.62618 | -1.65758 | -1.75796 | -2.3557 | -0.98966 |
| Q9DB41 | Q9DB41 | GHC2_MO | Slc25a18 | Mitochondrial glutamate carrier 2 | | | | 0.773552 | 0.438566 | 2.117256 | 0.626896 | 0.076559 | 1.674021 | 0.155998 | 0.257046 | 0.447626 | 2.71171 | 2.530552 | 2.247043 | 2.313901 | 2.536119 | 1.62991 | 2.36885 | 1.99628 | 2.235123 | 2.172703 | 1.47319 | 2.178745 |
| Q9DAM5 | Q9DAM5 | TPC_MOU | Slc25a19 | Mitochondrial thiamine pyrophosphate carrier | | | | 0.01188 | 0.003091 | 0.038175 | 0.954981 | 0.831639 | 2.746757 | 0.181149 | 0.316526 | 0.513083 | -0.35351 | -0.4994 | -0.08201 | 0.419032 | 0.163196 | -1.00041 | -0.46469 | -0.39439 | -0.83316 | -0.4931 | -0.94028 | -0.52999 |
| Q9Z2Z6 | Q9Z2Z6 | MCAT_MO | Slc25a20 | Mitochondrial carnitine/acylcarnitine carrier protein | | | | 0.166692 | -0.11279 | -0.47558 | 0.075229 | -0.08839 | -0.22783 | 0.52826 | 0.630343 | 1.400662 | 1.086187 | 0.667828 | 0.178109 | -0.12516 | 1.848338 | 0.956281 | 0.878031 | 1.101571 | 0.471984 | -0.24225 | 0.831864 | 0.712069 |
| Q9D6M3 | Q9D6M3 | GHC1_MO | Slc25a22 | Mitochondrial glutamate carrier 1 | | + | | 0.519832 | 0.420234 | 1.378041 | 1.44474 | 0.780518 | 5.133583 | 0.488821 | 0.621368 | 1.295664 | 3.853746 | 4.052734 | 5.527689 | 5.771633 | 6.268951 | 5.386871 | 3.821269 | 3.244743 | 4.959914 | 4.778371 | 5.018193 | 5.394893 |
| P13020 | P13020 | GELS_MO | Gsn | Gelsolin | + | | | 2.630276 | -0.71706 | -20.624 | 0.567241 | 0.402327 | 1.5067 | 0.32566 | 0.278624 | 0.878259 | -0.84701 | -0.90815 | -1.14426 | -0.80447 | -1.5521 | -0.94063 | -0.17709 | -0.14395 | -1.17068 | -1.5827 | -1.60967 | -1.44031 |
| A2ASZ8 | A2ASZ8 | SCMC2_M | Slc25a25 | Mitochondrial adenyl nucleotide antiporter SLC25A25 | | | | 0.266899 | 0.07545 | 0.730878 | 0.499477 | 0.116782 | 1.323823 | 0.297089 | 0.139903 | 0.806618 | 2.031274 | 2.009278 | 0.745003 | 0.779737 | 1.199385 | 1.104516 | 1.842181 | 2.047471 | 0.559098 | 0.732077 | 0.845215 | 1.178879 |
| Q9D6D0 | Q9D6D0 | Q9D6D0_ | Slc25a27 | Solute carrier family 25, member 27 | | | | 0.817375 | -0.71922 | -2.26016 | 0.029862 | -0.06248 | -0.09418 | 0.209363 | -0.1939 | -0.58546 | -0.58505 | -0.91332 | -0.02718 | -0.27554 | -0.36689 | 0.257163 | 0.242663 | -0.30258 | 0.562782 | -0.74054 | 0.250076 | 0.027999 |
| Q8BL03 | Q8BL03 | S2529_MC | Slc25a29 | Mitochondrial basic amino acids transporter | | | | 0.087367 | -0.13244 | -0.26209 | 0.014629 | -0.00492 | -0.04687 | 0.495521 | 0.28822 | 1.313353 | -2.18636 | -1.59599 | -1.95024 | -2.06738 | -1.0844 | -1.20047 | -2.16888 | -1.34858 | -1.91686 | -2.09093 | -1.6423 | -1.21901 |
| P14602 | P14602 | HSPB1_M | Hspb1 | Heat shock protein beta-1 | + | | + | 1.657551 | -0.73979 | -6.63003 | 0.47834 | -0.21515 | -1.26811 | 1.543215 | 0.739815 | 5.782612 | -0.36873 | -0.24843 | -0.56828 | -0.819 | -0.0705 | -0.31153 | 0.337234 | 0.525195 | -0.59282 | -0.36416 | -0.97377 | -0.88788 |
| Q9CX86 | Q9CX86 | ROA0_MO | Hnrnpa0 | Heterogeneous nuclear ribonucleoprotein A0 | + | | | 1.522675 | -0.74486 | -5.6414 | 0.403742 | -0.31949 | -1.07543 | 0.008328 | 0.027654 | 0.026867 | -3.8395 | -3.63166 | -2.63837 | -2.36322 | -1.29171 | -2.78586 | -2.90927 | -3.07217 | -1.918 | -2.44461 | -1.35837 | -2.77451 |
| P48962 | P48962 | ADT1_MO | Slc25a4 | ADP/ATP translocase 1 | | | | 0.363907 | 0.228359 | 0.974461 | 0.23218 | -0.14892 | -0.64339 | 0.354389 | 0.147196 | 0.950467 | 7.619748 | 7.470088 | 6.643201 | 6.305609 | 7.444523 | 7.252626 | 7.094484 | 7.538635 | 6.781693 | 6.464954 | 7.079816 | 7.322942 |
| Q8BGP6 | Q8BGP6 | S2540_MC | Slc25a40 | Probable mitochondrial glutathione transporter SLC25A40 | | | | 0.033116 | -0.04722 | -0.10411 | 0.122589 | 0.11038 | 0.35882 | 0.20006 | 0.227787 | 0.561697 | -0.8786 | -0.56057 | -1.11249 | -1.28159 | -0.88571 | -1.57832 | -0.24754 | -1.09718 | -1.60319 | -1.01165 | -1.67083 | -1.24877 |
| Q8R0Y8 | Q8R0Y8 | S2542_MC | Slc25a42 | Mitochondrial coenzyme A transporter SLC25A42 | | + | | 0.262687 | 0.254133 | 0.720294 | 1.655059 | 1.112763 | 6.610392 | 0.470618 | 0.348833 | 1.247888 | 0.034941 | 0.216385 | 1.105772 | 1.420894 | 1.111398 | 0.939286 | 0.212485 | -0.46943 | 0.091313 | 0.209828 | 0.942472 | 0.410545 |
| Q8BGF9 | Q8BGF9 | S2544_MC | Slc25a44 | Solute carrier family 25 member 44 | | + | | 0.462906 | 0.408466 | 1.227769 | 1.116662 | 1.05489 | 3.406099 | 0.821466 | 0.583901 | 2.273777 | -3.03079 | -2.61575 | -2.35215 | -2.60278 | -2.15954 | -2.53443 | -3.49177 | -2.9717 | -3.24913 | -3.81558 | -2.75536 | -3.10641 |
| Q9CQS4 | Q9CQS4 | S2546_MC | Slc25a46 | Mitochondrial outer membrane protein SLC25A46 | | | + | 0.220231 | -0.18181 | -0.61311 | 0.020487 | 0.042121 | 0.065232 | 1.060722 | 0.610379 | 3.16597 | -0.96294 | -0.43759 | -0.52424 | -1.34281 | 0.159429 | -0.22247 | -0.38085 | -0.65606 | -0.47622 | -1.47508 | -0.66851 | -0.61529 |
| P51881 | Q3V132;P | ADT2_MO | Slc25a5 | ADP/ATP translocase 2 | | | | 0.242499 | 0.0361 | 0.66946 | 0.151775 | -0.13097 | -0.43653 | 0.474957 | 0.223798 | 1.25924 | 8.757013 | 8.758144 | 7.700592 | 7.51227 | 8.577147 | 8.361355 | 8.667557 | 8.7754 | 8.02228 | 7.452529 | 8.104228 | 8.386678 |
| Q5HZI9 | Q5HZI9 | S2551_MC | Slc25a51 | Mitochondrial nicotinamide adenine dinucleotide transporter SLC25A51 | | | | 0.44554 | 0.293434 | 1.182699 | 0.377067 | 0.388326 | 1.007708 | 0.111162 | 0.093313 | 0.327838 | 1.473453 | 1.480813 | 1.122141 | 1.369715 | 1.858674 | 1.294562 | 1.431777 | 0.935621 | 1.222535 | 0.492669 | 1.445102 | 1.521508 |
| Q60714 | Q60714 | S27A1_MC | Slc27a1 | Long-chain fatty acid transport protein 1 | | | | 0.119823 | 0.081105 | 0.351352 | 0.0579 | -0.05723 | -0.17791 | 0.561188 | 0.21466 | 1.490075 | 0.88358 | 0.566629 | 0.743283 | 0.545696 | 0.799356 | 0.664829 | 0.811842 | 0.476157 | 0.39559 | 1.007844 | 0.39004 | 0.644825 |
| Q91VE0 | Q91VE0 | S27A4_MC | Slc27a4 | Long-chain fatty acid transport protein 4 | + | | | 1.270337 | -0.30161 | -4.14115 | 0.31636 | -0.2462 | -0.85493 | 0.130071 | 0.126491 | 0.378926 | -0.37768 | -0.27281 | -0.44682 | -0.9607 | 0.096694 | -0.23107 | 0.026913 | -0.07418 | -0.58761 | -0.32752 | 0.097136 | -0.4845 |
| P60202 | P60202 | MYPR_MO | Plp1 | Myelin proteolipid protein | + | | | 1.946967 | -0.77905 | -9.32779 | 0.859025 | -0.63427 | -2.4011 | 0.037395 | -0.07588 | -0.11708 | 8.827574 | 8.808819 | 6.075174 | 5.592339 | 6.181885 | 7.283048 | 9.514259 | 9.680243 | 6.575245 | 6.360801 | 7.15029 | 6.466401 |
| Q3UHK1 | Q3UHK1 | MYCT_MO | Slc2a13 | Proton myo-inositol cotransporter | | + | | 0.79581 | 0.463076 | 2.189174 | 1.29715 | 0.718226 | 4.281946 | 0.731976 | 0.403426 | 1.986423 | -0.43271 | -0.42093 | 0.310153 | 0.119615 | 0.066736 | 0.0881 | -0.67845 | -1.10135 | -0.64139 | -0.36529 | -0.1232 | -0.52882 |
| P32037 | P32037 | GTR3_MO | Slc2a3 | Solute carrier family 2, facilitated glucose transporter member 3 | | | | 0.756344 | -1.24941 | -2.06257 | 0.266644 | -0.67522 | -0.73024 | 0.10961 | 0.197279 | 0.3236 | -0.36604 | -0.27076 | 1.103323 | -0.73939 | 0.414936 | 1.346204 | 1.534885 | 0.327129 | 0.779099 | 0.935277 | 1.076794 | 0.289788 |
| Q60738 | Q60738 | ZNT1_MO | Slc30a1 | Proton-coupled zinc antiporter SLC30A1 | | | | 0.110164 | -0.34188 | -0.32511 | 0.794176 | -0.46981 | -2.18385 | 0.093193 | -0.09802 | -0.27836 | -3.35629 | -2.87891 | -1.6258 | -2.04051 | -1.68338 | -1.16401 | -1.75161 | -3.79984 | -1.42066 | -1.30603 | -1.08782 | -1.56352 |
| P97441 | P97441 | ZNT3_MO | Slc30a3 | Probable proton-coupled zinc antiporter SLC30A3 | | | | 0.447568 | -1.42002 | -1.18795 | 0.325614 | -0.62269 | -0.87814 | 0.152168 | 0.334932 | 0.437565 | -1.11785 | -3.48987 | -0.11183 | -1.50631 | -0.53277 | 0.194323 | -0.73464 | -1.03304 | -0.31553 | -0.05722 | 0.169444 | -1.17776 |
| Q6P5E4 | Q6P5E4 | UGGG1_M | Uggt1 | UDP-glucose:glycoprotein glucosyltransferase 1 | + | + | | 1.662444 | -0.81123 | -6.66876 | 1.359142 | -0.93414 | -4.62327 | 0.440831 | -0.89071 | -1.17053 | -2.05402 | -1.85805 | -1.06869 | -1.3834 | -0.8693 | -0.4924 | -1.07272 | -1.21689 | -0.41865 | -0.16516 | 0.947101 | -0.52738 |
| P19001 | P19001 | K1C19_M | Krt19 | Keratin, type I cytoskeletal 19 | + | | | 1.38175 | -0.82141 | -4.7535 | 0.418537 | 1.196991 | 1.113225 | 0.267907 | 1.378896 | 0.733411 | -3.28995 | -2.96464 | -1.5017 | -1.54782 | -1.71105 | 0.50172 | -2.24755 | -2.36423 | -3.79675 | -1.64675 | -3.50367 | -0.46345 |
| Q91ZN5 | Q91ZN5 | S35B2_MC | Slc35b2 | Adenosine 3'-phospho 5'-phosphosulfate transporter 1 | | | | 0.239526 | -1.22435 | -0.66196 | 0.996626 | -1.59974 | -2.90674 | 0.144367 | -0.67423 | -0.41698 | -2.60076 | -4.45901 | -3.06271 | -3.92851 | -3.70807 | -2.26454 | -3.90482 | -0.70624 | -2.2357 | -1.55604 | -0.86516 | -3.75898 |
| Q8CD26 | Q8CD26 | S35E1_MC | Slc35e1 | Solute carrier family 35 member E1 | | | + | 0.256134 | 0.611494 | 0.703814 | 0.552326 | 0.883861 | 1.465843 | 1.237098 | 1.208827 | 3.972049 | -3.34793 | -1.78403 | -2.25222 | -1.22067 | -2.53701 | -2.03461 | -2.79877 | -3.55618 | -2.93264 | -2.30798 | -3.32283 | -3.66644 |
| Q9DCP2 | Q9DCP2 | S38A3_MC | Slc38a3 | Sodium-coupled neutral amino acid transporter 3 | | | | 0.5523 | -0.9154 | -1.46577 | 0.011869 | 0.07611 | 0.038139 | 0.304907 | -0.32386 | -0.82622 | -1.1362 | 0.0588 | -0.56784 | -3.49329 | -1.27081 | -0.58851 | 0.558408 | 0.194998 | -0.74916 | -3.4642 | -0.41275 | -0.79884 |
| Q6P5F6 | Q6P5F6 | S39AA_MC | Slc39a10 | Zinc transporter ZIP10 | | | | 0.614791 | -0.65872 | -1.63954 | 0.295916 | -0.22271 | -0.80368 | 0.030894 | -0.02516 | -0.09734 | -2.36796 | -1.93084 | -0.68391 | -0.99543 | -1.6666 | -1.34156 | -1.15356 | -1.82781 | -0.84616 | -0.38778 | -1.67988 | -1.27798 |
| Q5FWH7 | Q5FWH7 | S39AC_MC | Slc39a12 | Zinc transporter ZIP12 | | | | 0.079023 | 0.056441 | 0.238598 | 0.109153 | -0.12033 | -0.32235 | 0.120567 | -0.17096 | -0.35336 | -0.56155 | -0.3411 | -1.46588 | -1.37422 | -1.11138 | -2.01861 | -0.29847 | -0.71707 | -0.92927 | -1.67018 | -1.22583 | -1.56225 |
| P10852 | P10852 | 4F2_MOU | Slc3a2 | 4F2 cell-surface antigen heavy chain | | | | 0.259876 | -0.10526 | -0.71323 | 0.262004 | -0.16941 | -0.71858 | 0.334575 | 0.312855 | 0.900638 | 2.313345 | 2.34293 | 2.286116 | 1.814612 | 2.836422 | 2.187961 | 2.580236 | 2.286559 | 2.221802 | 2.217751 | 2.323999 | 2.074674 |
| Q6X893 | Q6X893 | CTL1_MO | Slc44a1 | Choline transporter-like protein 1 | + | | | 1.103915 | -0.83259 | -3.35019 | 0.443149 | 0.426984 | 1.17652 | 0.12108 | -0.40371 | -0.35475 | -1.00258 | -0.56613 | -1.19101 | -1.16024 | -2.70927 | -0.67488 | 0.167149 | -0.07068 | -1.9652 | -1.24001 | -0.77804 | -1.79868 |
| P04919 | P04919 | B3AT_MO | Slc4a1 | Band 3 anion transport protein | | | | 0.126349 | 0.457024 | 0.368941 | 0.230062 | -0.62829 | -0.63803 | 0.202647 | 0.333675 | 0.568313 | -1.96908 | -1.39241 | -3.11163 | -3.18478 | -1.71688 | -1.75746 | -3.34249 | -0.93305 | -1.53585 | -3.50397 | -1.48406 | -2.65763 |
| Q5DTL9 | Q5DTL9 | S4A10_MC | Slc4a10 | Sodium-driven chloride bicarbonate exchanger | + | | | 1.12561 | 1.6727 | 3.445783 | 0.146487 | -0.12 | -0.42259 | 0.813936 | 0.632674 | 2.248751 | -1.63159 | -0.73169 | 0.006835 | -0.30935 | 0.501331 | 0.010337 | -2.67215 | -3.03652 | 0.204622 | -0.26714 | -0.51427 | -0.23941 |
| P16283 | P16283 | B3A3_MO | Slc4a3 | Anion exchange protein 3 | | | | 0.056355 | -0.11934 | -0.17339 | 0.495261 | 0.518596 | 1.312666 | 0.058263 | 0.074126 | 0.178964 | -0.19625 | -0.57186 | -0.31076 | 0.306078 | -0.20577 | -0.83866 | -0.92686 | 0.397423 | -0.76784 | -0.27404 | -0.86358 | -0.3291 |
| Q8VHL0 | Q8VHL0 | UT1_MOU | Slc14a1 | Urea transporter 1 | + | | | 1.939697 | -0.82162 | -9.24871 | 0.762912 | -0.94833 | -2.08335 | 0.370779 | 0.425825 | 0.991811 | -0.87344 | -0.72985 | -2.14261 | -3.02275 | -1.05037 | -0.19557 | -0.03234 | 0.072297 | -1.51799 | -1.75071 | -1.08953 | -1.00806 |
| Q8JZR6 | Q8JZR6 | S4A8_MO | Slc4a8 | Electroneutral sodium bicarbonate exchanger 1 | | | | NaN | 0 | NaN | 0.145005 | -0.31941 | -0.41867 | 0.021552 | 0.061202 | 0.068547 | -3.74383 | -3.52606 | -2.25982 | -3.41299 | -2.02762 | -3.71225 | -3.22114 | -3.46155 | -3.01659 | -2.01741 | -3.22726 | -2.63502 |
| P31648 | P31648 | SC6A1_M | Slc6a1 | Sodium- and chloride-dependent GABA transporter 1 | | | | 0.118213 | 0.188359 | 0.346994 | 0.379769 | -1.1636 | -1.01455 | 0.221961 | 0.507793 | 0.617499 | -0.75587 | 0.247806 | -0.87274 | -3.16564 | -1.26081 | 0.005613 | -0.64933 | -0.23545 | -0.88834 | -0.82283 | -0.61072 | -1.66007 |
| P31650 | P31650 | S6A11_MC | Slc6a11 | Sodium- and chloride-dependent GABA transporter 3 | | | | 0.164344 | -0.11223 | -0.46946 | 0.390138 | -0.30055 | -1.04083 | 0.905425 | -0.26314 | -2.56442 | 5.180999 | 4.721397 | 2.147435 | 2.234025 | 2.327788 | 2.370113 | 4.997549 | 5.129304 | 2.776783 | 2.205785 | 2.712498 | 2.511685 |
| Q8BJI1 | Q8BJI1 | S6A17_MC | Slc6a17 | Sodium-dependent neutral amino acid transporter SLC6A17 | | | + | 0.829065 | 0.541275 | 2.299198 | 0.253427 | 0.370816 | 0.697001 | 1.095699 | 0.388602 | 3.314527 | -1.0045 | -0.65798 | 0.124186 | -0.93911 | 0.168619 | 0.228826 | -1.5319 | -1.21313 | -0.79803 | -0.75853 | -0.07657 | -0.30319 |
| Q61327 | Q61327 | SC6A3_M | Slc6a3 | Sodium-dependent dopamine transporter | | + | | NaN | 0 | NaN | 1.011528 | 2.951692 | 2.965567 | NaN | 0 | NaN | -4.0196 | -3.43126 | 0.253582 | 0.484127 | -2.79565 | -3.82399 | -3.12021 | -3.79655 | -3.57146 | -1.59421 | -3.19224 | -3.40908 |
| Q761V0 | Q761V0 | SC6A5_M | Slc6a5 | Sodium- and chloride-dependent glycine transporter 2 | | + | | 0.202342 | 0.2081 | 0.567534 | 2.416364 | 2.097262 | 16.10388 | NaN | 0 | NaN | 1.33269 | 1.131254 | -1.45584 | -1.25264 | -2.89274 | -3.35237 | 1.376442 | 0.671301 | -3.37003 | -3.53297 | -3.34041 | -3.00988 |
| O35316 | O35316 | SC6A6_M | Slc6a6 | Sodium- and chloride-dependent taurine transporter | | | + | 0.059305 | 0.055136 | 0.182001 | 0.779199 | -0.76445 | -2.13538 | 1.460677 | -0.86948 | -5.23394 | 4.448862 | 3.84969 | 0.843682 | 0.915262 | 1.135295 | 0.904702 | 4.139136 | 4.049142 | 2.000126 | 1.287724 | 2.009078 | 1.769881 |
| Q9D8W7 | Q9D8W7 | OCAD2_M | Ociad2 | OCIA domain-containing protein 2 | + | | + | 1.429085 | -0.83305 | -5.03668 | 0.149531 | 0.37388 | 0.430619 | 1.416687 | 1.334718 | 4.961108 | -3.66878 | -3.53228 | -2.25794 | -2.4712 | -1.22599 | -0.73379 | -2.91814 | -2.61683 | -1.87678 | -3.60012 | -2.42331 | -2.20591 |
| Q9WTR6 | Q9WTR6 | XCT_MOU | Slc7a11 | Cystine/glutamate transporter | | | | NaN | 0 | NaN | 0.383858 | 0.631054 | 1.024904 | 0.039181 | -0.22517 | -0.12246 | -4.04472 | -3.19379 | -0.96168 | -0.71068 | -0.63518 | -4.13995 | -3.31922 | -3.53239 | -2.07002 | -0.86444 | -2.71936 | -1.60542 |
| Q64105 | Q64105 | SPRE_MO | Spr | Sepiapterin reductase | + | | | 1.512322 | -0.83724 | -5.57142 | 0.513254 | -0.71064 | -1.36046 | 0.045452 | 0.140478 | 0.141225 | -0.69718 | -0.6801 | -0.66475 | -1.69029 | -0.85682 | 0.246837 | -0.00143 | 0.298634 | -0.36729 | -0.56648 | 0.382133 | -1.27307 |
| Q9Z127 | Q9Z127 | LAT1_MO | Slc7a5 | Large neutral amino acids transporter small subunit 1 | | | | 0.61356 | 0.250701 | 1.636044 | 0.48946 | 0.382914 | 1.297347 | 0.984613 | 0.704946 | 2.859935 | 0.658062 | 0.954803 | 0.279718 | -0.17425 | 0.739706 | 0.506766 | 0.594041 | 0.517422 | -0.14152 | -0.51884 | 0.135528 | -0.29895 |
| P70414 | P70414 | NAC1_MO | Slc8a1 | Sodium/calcium exchanger 1 | | | | 0.642725 | -0.69987 | -1.71955 | 0.311327 | -0.29133 | -0.84231 | 0.311993 | 0.240633 | 0.84398 | -1.34383 | -0.99711 | -0.23258 | -0.90141 | 0.356509 | 0.54227 | -0.10236 | -0.83884 | -0.18737 | -0.36395 | 0.478321 | -0.06081 |
| Q8K596 | Q8K596 | NAC2_MO | Slc8a2 | Sodium/calcium exchanger 2 | | | | 0.360947 | 1.530362 | 0.966993 | 0.875247 | -0.47924 | -2.45742 | 0.101512 | -0.04367 | -0.30139 | -1.25713 | -0.36374 | 1.176552 | 0.787284 | 1.526141 | 1.465273 | -0.82255 | -3.85904 | 1.448915 | 1.473408 | 1.681049 | 1.397709 |
| Q9D8T7 | Q9D8T7 | SLIRP_MO | Slirp | SRA stem-loop-interacting RNA-binding protein, mitochondrial | | | | 0.133594 | 0.498268 | 0.388344 | 0.781891 | -1.5616 | -2.14404 | 0.09837 | -0.41426 | -0.29271 | -3.33545 | -1.29913 | -1.93408 | -3.38378 | -0.32177 | -3.13644 | -3.59631 | -2.03481 | -1.16859 | -1.02607 | -1.16542 | -1.46426 |
| Q3URD3 | Q3URD3 | SLMAP_M | Slmap | Sarcolemmal membrane-associated protein | | | | 0.157649 | -0.14243 | -0.45196 | 0.137656 | 0.17136 | 0.399171 | 0.66816 | -0.52091 | -1.7938 | -0.57992 | 0.007213 | 1.901597 | 2.597471 | 0.986433 | 1.422043 | -0.02934 | -0.25851 | 2.32963 | 1.826718 | 1.53308 | 1.917224 |
| Q62432 | Q62432 | SMAD2_M | Smad2 | Mothers against decapentaplegic homolog 2 | | | | 0.562815 | 0.910414 | 1.49454 | 0.12773 | 0.134093 | 0.37265 | 0.744966 | -0.65042 | -2.02683 | -1.50249 | -2.47588 | -1.64691 | -1.03234 | -2.24161 | -1.60571 | -2.53327 | -3.26594 | -1.66095 | -1.28648 | -1.31672 | -1.22976 |
| Q60771 | Q60771 | CLD11_M | Cldn11 | Claudin-11 | + | | | 1.587126 | -0.84683 | -6.09544 | 0.120965 | -0.11055 | -0.35444 | 0.331127 | 0.324065 | 0.89198 | 3.268736 | 3.148423 | 0.977205 | 0.86415 | 1.963967 | 2.467133 | 3.930184 | 4.180644 | 1.337983 | 0.724481 | 2.153592 | 1.629377 |
| Q7TN29 | Q7TN29 | SMAP2_M | Smap2 | Stromal membrane-associated protein 2 | | | | 0.275241 | 0.128756 | 0.751825 | 0.054109 | -0.03519 | -0.16681 | 0.541578 | 0.145599 | 1.436621 | 0.82648 | 0.641535 | 1.172733 | 1.025919 | 1.24972 | 1.28196 | 0.461106 | 0.749398 | 0.936748 | 1.332282 | 1.220299 | 1.020183 |
| Q6DIC0 | Q6DIC0 | SMCA2_M | Smarca2 | Probable global transcription activator SNF2L2 | + | | | 1.267487 | 2.172828 | 4.126418 | 0.553301 | -0.36692 | -1.4685 | 0.75229 | -0.33165 | -2.04979 | -1.66458 | -0.71827 | -1.45426 | -0.98331 | -1.10388 | -1.27718 | -3.13317 | -3.59533 | -0.93542 | -0.76831 | -0.99552 | -0.72225 |
| Q3TKT4 | Q6DIC0;Q | SMCA4_M | Smarca4 | Transcription activator BRG1 | + | | + | 1.179433 | 1.426715 | 3.692285 | 0.168502 | 0.070556 | 0.480298 | 1.281583 | 0.703317 | 4.199713 | -1.2474 | -0.72067 | -0.93545 | -1.10581 | -0.32767 | -0.65476 | -2.6935 | -2.128 | -0.97151 | -1.21087 | -1.23056 | -1.1585 |
| Q9Z0H3 | Q9Z0H3 | SNF5_MO | Smarcb1 | SWI/SNF-related matrix-associated actin-dependent regulator of chromatin subfamily B member 1 | | | | 0.148678 | 0.333029 | 0.428369 | 0.19074 | 0.720259 | 0.537794 | 0.172022 | -0.38366 | -0.48945 | -3.58971 | -2.06652 | -2.55907 | -1.6394 | -3.26096 | -3.17688 | -3.31726 | -3.00503 | -4.07736 | -1.56163 | -3.61799 | -2.05254 |
| Q6PDG5 | Q6PDG5 | SMRC2_M | Smarcc2 | SWI/SNF complex subunit SMARCC2 | | | | 0.154224 | 0.703074 | 0.442968 | 0.299336 | 0.172847 | 0.81225 | 0.270209 | -0.35326 | -0.73919 | -1.44804 | -0.69724 | -0.44794 | -0.28452 | -1.27321 | -0.43671 | -3.31787 | -0.23356 | -0.73557 | -0.34259 | -0.73291 | -0.27048 |
| Q61466 | Q61466 | SMRD1_M | Smarcd1 | SWI/SNF-related matrix-associated actin-dependent regulator of chromatin subfamily D member 1 | | | | NaN | 0 | NaN | 0.355006 | 0.261509 | 0.95202 | 0.2982 | -0.41772 | -0.8094 | -3.63828 | -2.53863 | -1.04141 | -0.74168 | -2.06354 | -1.46799 | -3.76023 | -3.31349 | -0.92285 | -1.38326 | -1.76956 | -0.92653 |
| O54941 | O54941 | SMCE1_M | Smarce1 | SWI/SNF-related matrix-associated actin-dependent regulator of chromatin subfamily E member 1 | | | | 0.056971 | -0.08432 | -0.17519 | 0.066279 | -0.0886 | -0.2022 | 0.741774 | -0.4046 | -2.01686 | -3.36056 | -2.40919 | -1.76919 | -1.3046 | -2.21774 | -2.02508 | -2.8737 | -2.72741 | -1.81984 | -1.07673 | -1.89277 | -1.54083 |
| Q9CW03 | Q9CW03 | SMC3_MO | Smc3 | Structural maintenance of chromosomes protein 3 | | + | | 0.094062 | 0.187743 | 0.280774 | 1.207118 | 1.378875 | 3.824505 | 0.651319 | 0.82143 | 1.744487 | -2.94196 | -3.70337 | -2.01475 | -2.30934 | -1.51659 | -2.24094 | -2.96071 | -4.06011 | -3.87 | -3.21184 | -2.39928 | -3.00111 |
| Q6P5D8 | Q6P5D8 | SMHD1_M | Smchd1 | Structural maintenance of chromosomes flexible hinge domain-containing protein 1 | | | | 0.432569 | 2.112892 | 1.14924 | 0.628198 | -0.42311 | -1.67775 | 0.014103 | 0.010773 | 0.045209 | 1.52391 | 1.049114 | 2.299291 | 1.815967 | 3.135349 | 2.673989 | -2.6495 | 0.996741 | 2.55285 | 2.408633 | 2.953651 | 2.834141 |
| Q3UMB5 | Q3UMB5 | SMCR8_M | Smcr8 | Guanine nucleotide exchange protein SMCR8 | | | | 0.768396 | 1.293598 | 2.100789 | 0.099736 | 0.091935 | 0.29649 | 0.950676 | -0.31208 | -2.73058 | -1.26203 | -1.73168 | -0.6236 | -1.0416 | -1.02307 | -1.24145 | -2.22122 | -3.35968 | -1.15359 | -0.69548 | -0.85395 | -0.78641 |
| P61406 | P61406 | EST1A_MC | Smg6 | Telomerase-binding protein EST1A | | | | 0.080047 | -0.07854 | -0.24149 | 0.209981 | -0.21287 | -0.58703 | 0.764222 | -0.89128 | -2.08751 | -3.45904 | -3.56168 | -1.19591 | -0.51106 | -1.41829 | -1.1901 | -3.11067 | -3.75297 | -0.75995 | -0.52128 | -0.82435 | -0.00148 |
| Q5RJH6 | Q5RJH6 | SMG7_MO | Smg7 | Nonsense-mediated mRNA decay factor SMG7 | | | + | 0.209243 | -0.51582 | -0.58515 | 0.225678 | -0.16057 | -0.62693 | 1.056668 | -0.68936 | -3.14908 | -3.40161 | -3.34697 | -1.25107 | -1.69496 | -2.16914 | -2.18015 | -1.97739 | -3.73956 | -1.44026 | -1.18463 | -1.70412 | -1.26644 |
| Q8BGT7 | Q8BGT7 | SPF30_MC | Smndc1 | Survival of motor neuron-related-splicing factor 30 | | | | NaN | 0 | NaN | 0.51 | -1.77231 | -1.35178 | 0.607983 | -0.11928 | -1.62026 | -2.40479 | -3.51839 | -3.86361 | -1.24162 | -1.43058 | -1.28335 | -2.493 | -2.86905 | -0.76453 | -0.7961 | -1.23657 | -1.23879 |
| Q6ZPR5 | Q6ZPR5 | NSMA3_M | Smpd4 | Sphingomyelin phosphodiesterase 4 | | | + | 0.487943 | 0.841868 | 1.293349 | 0.798069 | 0.759545 | 2.196547 | 2.413507 | 0.338974 | 16.05069 | -2.97519 | -1.77292 | -2.71996 | -2.65433 | -2.66693 | -2.68062 | -2.96625 | -3.46559 | -3.79092 | -3.10246 | -2.99277 | -3.03273 |
| Q9CUN6 | Q9CUN6 | SMUF1_M | Smurf1 | E3 ubiquitin-protein ligase SMURF1 | | | | 0.204766 | 0.781687 | 0.573728 | 0.254551 | 1.759861 | 0.699831 | 0.492651 | 0.481249 | 1.305768 | -1.00932 | -3.73394 | 0.141356 | 0.599709 | 1.171647 | 0.435184 | -3.17409 | -3.13255 | -3.89356 | 1.114902 | 0.306707 | 0.337626 |
| P60879 | P60879 | SNP25_M | Snap25 | Synaptosomal-associated protein 25 | | | + | 0.575034 | 0.663759 | 1.528198 | 0.754683 | 0.390191 | 2.057327 | 2.172445 | 0.11563 | 12.13456 | 2.317218 | 2.566766 | 3.307652 | 3.108295 | 2.485014 | 2.473661 | 2.194266 | 1.3622 | 2.65643 | 2.979136 | 2.356053 | 2.371361 |
| P21278 | P21278 | GNA11_M | Gna11 | Guanine nucleotide-binding protein subunit alpha-11 | + | | | 1.576961 | -0.84902 | -6.02167 | 0.154795 | 0.141349 | 0.444468 | 0.227974 | 0.178046 | 0.632744 | -1.09927 | -1.29181 | -0.82591 | -0.25247 | -0.35565 | 0.007323 | -0.44953 | -0.24351 | -0.81811 | -0.54297 | -0.13717 | -0.56725 |
| Q61548 | Q61548 | AP180_M | Snap91 | Clathrin coat assembly protein AP180 | | | | 0.136477 | 0.092591 | 0.396033 | 0.503615 | 0.314276 | 1.3348 | 0.559382 | 0.493054 | 1.485126 | 0.951688 | 1.40229 | 2.38627 | 1.951938 | 2.738293 | 2.191341 | 1.021947 | 1.146849 | 1.763863 | 1.945793 | 2.159995 | 1.78353 |
| O55042 | O55042 | SYUA_MO | Snca | Alpha-synuclein | | | | 0.943212 | 0.472252 | 2.702683 | 0.491012 | 1.341122 | 1.301441 | 0.071564 | 0.085522 | 0.217372 | 1.509778 | 1.622232 | 3.799696 | 1.738991 | 2.370643 | 1.904266 | 1.259194 | 0.928313 | 1.411401 | 1.445042 | 2.368816 | 1.735048 |
| Q91ZZ3 | Q91ZZ3 | SYUB_MO | Sncb | Beta-synuclein | | | | 0.101256 | 0.204737 | 0.300682 | 0.488222 | 1.159667 | 1.294085 | 0.249206 | -0.61757 | -0.68637 | 1.295676 | 2.48753 | 3.16563 | 1.387667 | 1.722227 | 0.259944 | 1.357464 | 2.016268 | 1.229934 | 1.004029 | 2.133074 | 1.084244 |
| Q9Z0F7 | Q9Z0F7 | SYUG_MO | Sncg | Gamma-synuclein | | | | 0.287927 | 1.400919 | 0.783647 | 0.066957 | 0.179402 | 0.204153 | NaN | 0 | NaN | -0.99498 | -0.91224 | -2.46611 | -4.19396 | -3.19703 | -3.51092 | -4.14174 | -0.56732 | -3.67023 | -3.34864 | -3.49108 | -2.35307 |
| Q78PY7 | Q78PY7 | SND1_MO | Snd1 | Staphylococcal nuclease domain-containing protein 1 | | | | 0.196046 | -0.39541 | -0.55141 | 0.020212 | 0.028751 | 0.064376 | 0.381974 | 0.904794 | 1.020131 | -3.27133 | -2.31108 | -1.47172 | -2.35762 | 0.111363 | -1.59846 | -2.92842 | -1.86316 | -2.0005 | -1.88634 | -1.88453 | -1.41216 |
| Q80U23 | Q8BHS8;Q | SNPH_MO | Snph | Syntaphilin | | | | 0.933894 | 0.797276 | 2.668136 | 0.751631 | 0.556487 | 2.047721 | 0.508527 | 0.460093 | 1.347858 | 0.976591 | 0.741905 | 0.867079 | 1.410596 | 1.003672 | 0.672859 | 0.336782 | -0.21284 | 0.581688 | 0.583013 | 0.079574 | 0.676771 |
| Q9D1G5 | Q9D1G5 | LRC57_M | Lrrc57 | Leucine-rich repeat-containing protein 57 | + | | | 1.346308 | -0.85382 | -4.55074 | 0.127067 | 0.146133 | 0.370869 | 0.267548 | -0.14147 | -0.73251 | -1.68744 | -2.06212 | -0.55402 | -0.90116 | -0.65369 | -0.37814 | -1.01068 | -1.03123 | -1.22747 | -0.51998 | -0.23911 | -0.50978 |
| Q62376 | Q62376 | RU17_MO | Snrnp70 | U1 small nuclear ribonucleoprotein 70 kDa | | | | 0.635694 | 1.186886 | 1.699264 | 0.340652 | -0.21469 | -0.91591 | 0.260124 | 0.183132 | 0.713852 | -1.84503 | -1.47072 | -1.58251 | -1.85698 | -1.34936 | -1.09455 | -2.17183 | -3.51769 | -1.69508 | -1.31502 | -1.18242 | -1.62776 |
| P27048;P | P27048;P | RSMB_MO | Snrpb;Snrpn | Small nuclear ribonucleoprotein-associated protein B | | | | 0.068424 | -0.08654 | -0.20837 | 0.040239 | -0.03008 | -0.12564 | 0.1418 | 0.149931 | 0.410178 | 0.226216 | -0.38794 | 0.556195 | 1.03047 | 0.596316 | 1.220454 | 0.285275 | -0.27393 | 0.856471 | 0.790357 | 0.56813 | 0.948777 |
| P62317 | P62317 | SMD2_MO | Snrpd2 | Small nuclear ribonucleoprotein Sm D2 | | | | 0.058451 | 0.074657 | 0.179512 | 0.79611 | 0.323619 | 2.190152 | 0.32991 | 0.090026 | 0.888926 | -1.14711 | -0.42497 | -0.66359 | -0.42025 | -0.43006 | -0.23129 | -1.06707 | -0.65432 | -0.94938 | -0.78169 | -0.40121 | -0.44018 |
| P62320 | P62320 | SMD3_MO | Snrpd3 | Small nuclear ribonucleoprotein Sm D3 | | | | 0.355417 | 0.449357 | 0.953055 | 0.340026 | -0.26537 | -0.91433 | 0.360925 | 0.238455 | 0.966937 | -0.30366 | 0.586451 | 0.536321 | 0.194793 | 0.773952 | 1.127961 | -0.46362 | -0.1523 | 0.396247 | 0.865604 | 0.54079 | 0.884214 |
| P62305 | P62305 | RUXE_MO | Snrpe | Small nuclear ribonucleoprotein E | | | | 0.398328 | 1.158053 | 1.061647 | 0.278678 | -0.0654 | -0.76045 | 0.250136 | -0.112 | -0.68871 | -1.27764 | -1.15752 | -0.79796 | -0.63107 | -0.7737 | -0.98369 | -1.28648 | -3.46478 | -0.66995 | -0.62828 | -0.89088 | -0.64251 |
| P62307 | P62307 | RUXF_MO | Snrpf | Small nuclear ribonucleoprotein F | | | + | 0.475498 | -1.27495 | -1.26066 | 0.047587 | 0.090975 | 0.147568 | 1.278748 | 0.589756 | 4.184884 | -3.50569 | -3.86662 | -1.44102 | -2.17632 | -1.7055 | -1.43895 | -1.4161 | -3.40631 | -1.40477 | -2.39452 | -2.20778 | -2.11618 |
| P62309 | P62309 | RUXG_MO | Snrpg | Small nuclear ribonucleoprotein G | | | | 0.428397 | 2.069934 | 1.138514 | 0.933176 | -0.48252 | -2.66549 | 0.146076 | -0.22021 | -0.4215 | 0.509838 | 1.252178 | 2.163482 | 1.921901 | 2.056741 | 2.413176 | 0.590884 | -2.96874 | 2.660043 | 2.390379 | 1.964056 | 2.946288 |
| Q61234 | Q61234 | SNTA1_M | Snta1 | Alpha-1-syntrophin | | | | 0.856334 | -0.5233 | -2.39183 | 0.369554 | 0.178369 | 0.988716 | 0.202493 | 0.256416 | 0.567919 | -0.85368 | -1.09776 | -0.36999 | -0.07866 | 0.491321 | -0.40534 | -0.63401 | -0.27084 | -0.50913 | -0.29626 | -0.16001 | -0.26684 |
| Q61235 | Q61235 | SNTB2_M | Sntb2 | Beta-2-syntrophin | | | | NaN | 0 | NaN | 0.355355 | 1.090563 | 0.952901 | 0.18432 | 0.388537 | 0.521268 | -3.81864 | -3.72212 | -1.91032 | -1.59412 | -2.02656 | -0.83649 | -3.85444 | -3.16817 | -3.97627 | -1.70929 | -2.26896 | -1.37117 |
| Q9CQ69 | Q9CQ69 | QCR8_MO | Uqcrq | Cytochrome b-c1 complex subunit 8 | + | | | 1.571809 | -0.85545 | -5.9846 | 0.468262 | -0.60089 | -1.24174 | 0.03372 | 0.033058 | 0.105946 | 2.565495 | 2.851354 | 2.754191 | 1.807868 | 2.790893 | 3.351658 | 3.565779 | 3.561973 | 2.983361 | 2.780482 | 3.175135 | 2.9013 |
| Q9D855 | Q9D855 | QCR7_MO | Uqcrb | Cytochrome b-c1 complex subunit 7 | + | | | 2.639602 | -0.86532 | -20.8474 | 0.382703 | 0.492453 | 1.021978 | 0.059553 | -0.1342 | -0.18272 | -0.47482 | -0.53684 | -0.47106 | -0.82846 | -1.63901 | -0.82821 | 0.3319 | 0.387076 | -0.69472 | -1.58972 | -0.48699 | -1.71183 |
| O70493 | O70493 | SNX12_M | Snx12 | Sorting nexin-12 | | | | 0.334441 | -1.14654 | -0.9003 | 0.136249 | 0.063112 | 0.395426 | 0.864787 | 1.289019 | 2.421009 | -3.26975 | -3.20572 | -1.27654 | -1.00047 | -0.30638 | -1.15691 | -3.3643 | -0.8181 | -1.12149 | -1.28175 | -1.7003 | -2.34103 |
| Q8C080 | Q8C080 | SNX16_M | Snx16 | Sorting nexin-16 | | | + | 0.406605 | 0.845614 | 1.082732 | 0.116333 | 0.156504 | 0.3419 | 2.581668 | 2.179217 | 19.49751 | -3.54922 | -2.04958 | -1.85517 | -1.63186 | -1.54121 | -1.64948 | -3.42653 | -3.86349 | -2.34394 | -1.45609 | -3.67678 | -3.87235 |
| Q9CWK8 | Q9CWK8 | SNX2_MO | Snx2 | Sorting nexin-2 | | | | 0.039787 | 0.043341 | 0.124281 | 0.003603 | 0.014834 | 0.011684 | 0.138853 | 0.225022 | 0.402355 | -2.87695 | -2.31279 | -1.56918 | -3.14275 | -0.48981 | -1.58056 | -2.84327 | -2.43316 | -3.36718 | -1.37441 | -1.38405 | -1.13636 |
| Q3UHD6 | Q3UHD6 | SNX27_M | Snx27 | Sorting nexin-27 | | | + | 0.238551 | 0.181741 | 0.659493 | 0.665763 | -0.32444 | -1.78674 | 1.692839 | -0.50758 | -6.91406 | -0.59295 | -0.1024 | 0.428591 | 0.098654 | 0.051906 | 0.153265 | -0.40378 | -0.65505 | 0.663944 | 0.512185 | 0.66328 | 0.557053 |
| O70492 | O70492 | SNX3_MO | Snx3 | Sorting nexin-3 | | | | 0.347504 | 0.826613 | 0.933138 | 0.377603 | 0.941842 | 1.009064 | 0.039644 | 0.15557 | 0.12385 | -3.48752 | -2.06653 | -0.7904 | -2.1932 | -1.31956 | -3.25169 | -4.13271 | -3.07457 | -1.81782 | -3.04947 | -1.68366 | -3.24402 |
| Q8CE50 | Q8CE50 | SNX30_M | Snx30 | Sorting nexin-30 | | | | 0.160229 | -0.16216 | -0.45871 | 0.066469 | -0.13528 | -0.20275 | 0.435155 | 0.660775 | 1.155898 | -1.94592 | -2.32324 | -1.44432 | -2.76062 | -0.81705 | -1.79523 | -2.27138 | -1.67347 | -2.07684 | -1.85754 | -2.26285 | -1.67097 |
| Q80ZJ7 | Q80ZJ7 | SNX32_M | Snx32 | Sorting nexin-32 | | | + | 0.006566 | -0.00662 | -0.02122 | 0.322259 | 0.72848 | 0.869726 | 1.575399 | -1.52763 | -6.01041 | -3.77512 | -3.27539 | -2.62619 | -1.79801 | -3.42416 | -3.88393 | -3.70544 | -3.33183 | -3.66866 | -2.2125 | -2.23483 | -2.01801 |
| Q91YJ2 | Q91YJ2 | SNX4_MO | Snx4 | Sorting nexin-4 | | | | 0.727722 | 1.154184 | 1.973285 | 0.771591 | -0.74086 | -2.11099 | 0.116703 | 0.076924 | 0.342904 | -2.47237 | -1.61906 | -1.46942 | -2.06811 | -1.65003 | -1.44522 | -3.6 | -2.7998 | -1.2111 | -0.84471 | -1.82414 | -1.42496 |
| Q6P8X1 | Q6P8X1 | SNX6_MO | Snx6 | Sorting nexin-6 | | | | 0.034693 | -0.17829 | -0.1089 | 0.046877 | -0.05282 | -0.14546 | 0.207806 | 0.174724 | 0.581488 | -1.80582 | -3.33827 | -1.53161 | -1.85245 | -1.32275 | -1.12524 | -0.94695 | -3.84056 | -1.96495 | -1.31348 | -1.6825 | -1.11493 |
| O09111 | O09111 | NDUBB_M | Ndufb11 | NADH dehydrogenase [ubiquinone] 1 beta subcomplex subunit 11, mitochondrial | + | | | 1.865034 | -0.87109 | -8.473 | 0.24527 | -0.22075 | -0.67645 | 0.091804 | 0.228138 | 0.274491 | 3.414422 | 3.306601 | 2.20367 | 1.946781 | 2.4778 | 3.386525 | 4.144066 | 4.319146 | 2.595973 | 1.99598 | 3.399966 | 2.008083 |
| P08228 | P08228 | SODC_MO | Sod1 | Superoxide dismutase [Cu-Zn] | | | | 0.237219 | -0.55319 | -0.65613 | 0.450868 | 0.867032 | 1.196492 | 0.001186 | 0.003814 | 0.003856 | -0.95371 | 0.652067 | 1.027539 | -0.42132 | -0.15006 | 0.16771 | 0.145071 | 0.659661 | -0.54636 | -0.58149 | 0.981229 | -0.97121 |
| P09671 | P09671 | SODM_MO | Sod2 | Superoxide dismutase [Mn], mitochondrial | + | | | 1.141676 | -1.96615 | -3.51794 | 0.389144 | -1.0854 | -1.03831 | 0.043998 | -0.17284 | -0.13689 | 0.13072 | 1.030342 | 1.296238 | -0.77924 | 0.369549 | 2.436219 | 2.878385 | 2.214971 | 1.21801 | 1.469777 | 2.301239 | 0.850205 |
| E1U8D0 | E1U8D0 | SOGA1_M | Soga1 | Protein SOGA1 | | | + | 0.27939 | -0.51662 | -0.76223 | 0.538988 | 0.988422 | 1.429607 | 1.498393 | -0.5031 | -5.47851 | -3.8956 | -2.54019 | -2.51121 | -1.54212 | -2.5891 | -2.50345 | -2.71109 | -2.69146 | -3.50829 | -2.52189 | -2.12441 | -1.96194 |
| Q6NZL0 | Q6NZL0 | SOGA3_M | Soga3 | Protein SOGA3 | | | | 0.346364 | -0.12098 | -0.93027 | 0.106518 | 0.076778 | 0.315142 | 0.089743 | -0.01982 | -0.26874 | 0.504727 | 0.254 | 1.102097 | 1.424224 | 1.034894 | 0.948013 | 0.534933 | 0.465753 | 1.003589 | 1.369176 | 0.951669 | 1.070881 |
| Q61598 | Q61598 | GDIB_MO | Gdi2 | Rab GDP dissociation inhibitor beta | + | | | 3.273789 | -0.87257 | -43.3233 | 0.014346 | 0.02843 | 0.045977 | 0.118666 | -0.20584 | -0.34822 | -0.52478 | -0.53856 | 0.525394 | -0.60332 | -0.55982 | 0.371449 | 0.359825 | 0.321973 | -0.32012 | 0.185336 | 0.475813 | -0.2525 |
| Q3UTJ2 | Q3UTJ2 | SRBS2_MC | Sorbs2 | Sorbin and SH3 domain-containing protein 2 | | | | 0.123449 | 0.255149 | 0.361139 | 0.106804 | -0.09864 | -0.31592 | 0.347642 | 0.103933 | 0.933484 | -3.5341 | -2.12128 | -0.34744 | -0.47578 | -0.51447 | -0.35486 | -3.07099 | -3.09469 | -0.61852 | -0.00743 | -0.61624 | -0.46096 |
| Q9EPR5 | Q9EPR5 | SORC2_M | Sorcs2 | VPS10 domain-containing receptor SorCS2 | | | | 0.647421 | 1.301067 | 1.733157 | 0.063809 | 0.086546 | 0.19507 | 0.925233 | 0.706801 | 2.636291 | -1.28228 | -1.94219 | -1.07654 | -1.77179 | -0.45075 | -0.97979 | -2.62227 | -4.10433 | -1.78638 | -1.23504 | -1.37838 | -1.46576 |
| Q99LC5 | Q99LC5 | ETFA_MO | Etfa | Electron transfer flavoprotein subunit alpha, mitochondrial | + | | | 2.775172 | -0.8776 | -24.3803 | 0.002796 | 0.002873 | 0.009076 | 0.380854 | -0.18242 | -1.0173 | 0.220671 | 0.287368 | 0.549026 | -0.0835 | 0.388175 | 0.694234 | 1.145169 | 1.118069 | 0.216992 | 0.242787 | 0.817083 | 0.630157 |
| O88307 | O88307 | SORL_MO | Sorl1 | Sortilin-related receptor | | | | 0.503129 | 1.474073 | 1.33351 | 0.362207 | 0.208526 | 0.970171 | 0.066692 | -0.06849 | -0.20339 | -0.52921 | -1.195 | -0.09004 | -0.35718 | -0.39468 | -0.97403 | -1.28209 | -3.39027 | -0.60053 | -0.26374 | -0.78759 | -0.44413 |
| Q6PHU5 | Q6PHU5 | SORT_MO | Sort1 | Sortilin | | | + | 0.335961 | -1.30626 | -0.90412 | 0.270116 | 0.253486 | 0.738958 | 2.016447 | 0.72663 | 10.11741 | -3.65799 | -0.77799 | -0.19833 | -0.55148 | 0.083299 | -0.05301 | -1.02923 | -0.79423 | -0.92249 | -0.3343 | -0.73413 | -0.68884 |
| Q58A65 | Q58A65 | JIP4_MOU | Spag9 | C-Jun-amino-terminal kinase-interacting protein 4 | | | | 0.048677 | -0.17603 | -0.1508 | 0.54569 | 0.508735 | 1.447781 | 0.81572 | -0.23368 | -2.25467 | -1.82691 | -3.06516 | -0.77601 | -1.18911 | -1.81181 | -2.00566 | -1.28042 | -3.25959 | -1.77557 | -1.20702 | -1.63835 | -1.71177 |
| Q02053 | P31254;Q | UBA1_MO | Uba1 | Ubiquitin-like modifier-activating enzyme 1 | + | | | 1.30635 | -0.89662 | -4.33119 | 0.263026 | -0.45097 | -0.72115 | 0.050771 | -0.06746 | -0.15699 | 0.251625 | 0.372516 | 1.47457 | 0.306541 | 1.062351 | 1.692985 | 1.406689 | 1.0107 | 1.117933 | 1.56512 | 1.737081 | 1.153179 |
| Q8K004 | Q8K004 | SPAT2_MC | Spata2 | Spermatogenesis-associated protein 2 | | | | NaN | 0 | NaN | 0.560274 | 0.242419 | 1.48757 | 0.6826 | -0.8655 | -1.83657 | -3.38552 | -3.63842 | -0.78289 | -0.64661 | -1.96115 | -1.91614 | -3.32804 | -3.31635 | -1.10521 | -0.80913 | -1.54387 | -0.60242 |
| Q8BNN1 | Q8BNN1 | SPA2L_MC | Spata2l | Spermatogenesis-associated protein 2-like protein | | + | | NaN | 0 | NaN | 1.62965 | 0.421825 | 6.413227 | 0.439473 | -0.36228 | -1.16703 | -3.56601 | -3.42307 | 1.276091 | 1.378433 | -0.04175 | -0.31897 | -3.70591 | -3.07474 | 0.864112 | 0.946763 | -0.09584 | 0.459688 |
| Q91WJ7 | Q91WJ7 | SPS2L_MC | Spats2l | SPATS2-like protein | | | | NaN | 0 | NaN | 0.276043 | 0.264073 | 0.753837 | 0.29391 | -0.70209 | -0.79865 | -2.62382 | -3.37278 | -3.0511 | -2.98566 | -1.90007 | -3.52685 | -3.60598 | -3.45586 | -3.63123 | -2.93368 | -1.6779 | -2.34485 |
| Q5SXY1 | Q5SXY1 | CYTSB_MC | Specc1 | Cytospin-B | | | | 0.226507 | -0.25663 | -0.62903 | 0.094874 | -0.15563 | -0.28303 | 0.483278 | -0.46017 | -1.28107 | -1.54687 | -1.08312 | -0.66847 | -0.255 | -0.49221 | 0.197334 | -0.72269 | -1.39404 | -0.81564 | 0.203439 | 0.21193 | 0.413529 |
| Q6NSW3 | Q6NSW3 | SPKAP_MC | Sphkap | A-kinase anchor protein SPHKAP | | | | 0.350478 | 0.575188 | 0.940621 | 0.426664 | -0.15407 | -1.13406 | 0.683338 | -0.63474 | -1.83877 | 0.324276 | 0.04696 | 0.529927 | 0.400626 | 0.652639 | 0.732507 | -0.98514 | 0.206001 | 0.499858 | 0.738837 | 0.984431 | 1.670193 |
| Q52KF3 | Q52KF3 | SPIR1_MO | Spire1 | Protein spire homolog 1 | | | | 0.722526 | 0.765413 | 1.957295 | 0.421676 | -0.43684 | -1.12127 | 0.915087 | -0.87762 | -2.59932 | -0.42323 | -0.22875 | 0.082415 | 0.770603 | -0.01703 | 0.384485 | -0.71263 | -1.47018 | 0.680632 | 1.046075 | 0.789883 | 1.332819 |
| Q62288 | Q62288 | TICN1_MC | Spock1 | Testican-1 | | | | 0.168163 | -0.1981 | -0.47941 | 0.439532 | 0.423359 | 1.167181 | 0.462745 | -1.29017 | -1.22735 | -1.2587 | -1.25511 | 0.044523 | -0.57952 | -3.62347 | -1.63911 | -0.64559 | -1.47202 | -0.5059 | -0.87581 | -1.68835 | -0.99389 |
| Q9ER58 | Q9ER58 | TICN2_MC | Spock2 | Testican-2 | | | + | 0.363977 | 0.709633 | 0.974637 | 0.598625 | 0.591839 | 1.593914 | 1.793712 | 0.657452 | 7.79056 | -2.47355 | -1.65843 | -2.24979 | -1.54016 | -1.67458 | -1.50628 | -2.17228 | -3.37896 | -2.59626 | -2.37737 | -2.25425 | -2.24151 |
| Q8VCC9 | Q8VCC9 | SPON1_M | Spon1 | Spondin-1 | | | | 0.005311 | -0.00568 | -0.01719 | 0.167726 | 0.174743 | 0.478278 | 0.193312 | 0.194939 | 0.5444 | -0.67115 | -1.24353 | -0.3721 | 0.32408 | -0.63442 | -0.27956 | -1.11674 | -0.78659 | -0.30975 | -0.08776 | -0.96296 | -0.34089 |
| P14824 | P14824 | ANXA6_M | Anxa6 | Annexin A6 | + | | | 1.683353 | -0.90214 | -6.83661 | 0.052364 | -0.184 | -0.16168 | 0.123406 | 0.245703 | 0.361021 | -0.10821 | -0.17052 | 0.170214 | -2.09943 | 0.34808 | -0.72123 | 0.891003 | 0.63455 | -0.69557 | -0.86565 | -0.01117 | -0.85338 |
| Q924S7 | Q924S7 | SPRE2_MC | Spred2 | Sprouty-related, EVH1 domain-containing protein 2 | | | | 0.20287 | 0.174143 | 0.568883 | 0.566194 | 0.092866 | 1.503822 | 0.706295 | -0.55502 | -1.90777 | -0.67638 | -0.47858 | -0.15729 | -0.28077 | -1.13389 | -0.93243 | -0.46193 | -1.04132 | -0.31061 | -0.31319 | -0.2052 | -0.75107 |
| Q62266 | Q62266 | SPR1A_MC | Sprr1a | Cornifin-A | | | | 0.008876 | -0.0189 | -0.02862 | 0.047172 | -0.14635 | -0.14634 | 0.098652 | -0.15733 | -0.29349 | 2.470465 | 1.275613 | 0.84057 | 1.040049 | 0.495836 | 1.324567 | 1.610435 | 2.173443 | 0.091555 | 2.081768 | 1.407627 | 0.727439 |
| Q62267 | Q62267 | SPR1B_MC | Sprr1b | Cornifin-B | | | | 0.263344 | 0.565874 | 0.721946 | 0.148115 | 0.789019 | 0.426885 | 0.231093 | -0.54034 | -0.64064 | 1.177824 | 0.018001 | -0.34913 | -0.37885 | -1.20134 | 0.193468 | -0.49529 | 0.559365 | -3.00127 | 0.695251 | 0.510766 | -0.43795 |
| O70554;O | O70555;O | SPR2B_MC | Sprr2b;Sprr2d;Sprr2g;Sprr2h;Sprr2k | Small proline-rich protein 2B | | + | | 0.083485 | -0.43 | -0.2512 | 1.057369 | -2.00347 | -3.152 | 0.097351 | 0.205136 | 0.289897 | -0.55931 | -1.78032 | -2.69659 | -3.95971 | -2.20819 | -0.99787 | -2.33908 | 0.859447 | -1.25298 | -1.39639 | -1.44142 | -2.17491 |
| Q3TFQ1 | Q3TFQ1 | SPRY7_MC | Spryd7 | SPRY domain-containing protein 7 | | | | 0.042716 | 0.15634 | 0.133063 | 0.503076 | 0.123129 | 1.33337 | 0.281379 | -0.15901 | -0.76723 | -3.23222 | -1.45616 | -2.01852 | -1.90096 | -2.43379 | -2.17983 | -1.73119 | -3.26986 | -2.15409 | -2.01164 | -1.98399 | -2.3116 |
| P16546 | P16546 | SPTN1_MC | Sptan1 | Spectrin alpha chain, non-erythrocytic 1 | | | | 0.076657 | -0.12493 | -0.23189 | 0.753694 | -0.16444 | -2.05421 | 0.606143 | -0.12874 | -1.61507 | 1.890108 | 2.030818 | 2.569499 | 2.454817 | 2.491185 | 2.557703 | 2.619543 | 1.551247 | 2.62074 | 2.73245 | 2.580742 | 2.725621 |
| Q62261 | P15508;Q | SPTB2_MC | Sptbn1 | Spectrin beta chain, non-erythrocytic 1 | | | | 0.020745 | -0.02817 | -0.06604 | 0.497401 | -0.08107 | -1.31833 | 0.244542 | 0.05741 | 0.674615 | 2.341467 | 2.461282 | 2.93572 | 2.853105 | 2.867958 | 2.846363 | 2.851906 | 2.007185 | 2.929927 | 3.02103 | 2.715338 | 2.884163 |
| Q68FG2 | Q68FG2 | Q68FG2_ | Sptbn2 | Spectrin beta chain | | + | | 0.570243 | 0.654298 | 1.51497 | 1.40856 | -0.50364 | -4.91211 | 0.819078 | -0.25674 | -2.26582 | -0.37125 | 0.069906 | 0.461648 | 0.27077 | 0.700103 | 0.816356 | -0.43366 | -1.17628 | 0.832384 | 0.907324 | 0.917704 | 1.11223 |
| E9PX29 | E9PX29 | E9PX29_M | Sptbn4 | Spectrin beta chain | | | | 0.168821 | 0.104868 | 0.481126 | 0.398772 | 0.532197 | 1.062777 | 0.289294 | 0.07194 | 0.787075 | -0.84169 | -0.41479 | -0.39987 | -0.07613 | -1.25526 | -1.23332 | -0.68898 | -0.77724 | -1.24407 | -0.29632 | -1.40697 | -1.22549 |
| Q91Z53 | Q91Z53 | GRHPR_M | Grhpr | Glyoxylate reductase/hydroxypyruvate reductase | + | | | 2.422116 | -0.90428 | -16.2115 | 0.97723 | -0.70278 | -2.83143 | 0.014978 | -0.02605 | -0.04797 | 1.055401 | 1.161312 | -0.21461 | -0.6974 | 0.172114 | 0.840567 | 1.995111 | 2.030165 | 0.189037 | 0.304513 | 0.960303 | 0.104469 |
| Q64337 | Q64337 | SQSTM_M | Sqstm1 | Sequestosome-1 | | | | 0.238293 | 0.453073 | 0.658843 | 0.23796 | 0.82486 | 0.658 | 0.31124 | 0.553166 | 0.842091 | -3.40214 | -2.28684 | -1.75169 | -1.34221 | -2.79185 | -1.73111 | -3.69997 | -2.89516 | -3.60856 | -1.13506 | -2.42707 | -3.20222 |
| Q9QWI6 | Q9QWI6 | SRCN1_M | Srcin1 | SRC kinase signaling inhibitor 1 | | | | 0.722831 | 0.523265 | 1.958232 | 0.944565 | 0.381466 | 2.707725 | 0.31232 | -0.45394 | -0.8448 | 0.606075 | 0.268114 | 3.805774 | 4.084637 | 2.069243 | 2.525503 | 0.120828 | -0.29317 | 3.543582 | 3.583896 | 2.264811 | 3.237813 |
| Q91Z67 | Q91Z69;Q | SRGP2_M | Srgap2 | SLIT-ROBO Rho GTPase-activating protein 2 | | | | 0.748391 | 0.493729 | 2.037549 | 0.241169 | 0.04654 | 0.666103 | 0.090041 | 0.073987 | 0.269575 | -0.49076 | -0.91884 | -0.31924 | -0.12318 | | -0.20703 | -1.31212 | -1.08494 | -0.30051 | -0.2916 | -0.51033 | 0.032142 |
| Q812A2 | Q812A2 | SRGP3_M | Srgap3 | SLIT-ROBO Rho GTPase-activating protein 3 | + | | | 1.02893 | 0.473665 | 3.035352 | 0.021874 | 0.007678 | 0.069549 | 0.473196 | -0.16005 | -1.25463 | -0.56055 | -0.80524 | 0.407833 | 0.589615 | 0.62402 | 0.435741 | -1.25343 | -1.0597 | 0.428391 | 0.553701 | 0.603841 | 0.77603 |
| Q6P069 | Q6P069 | SORCN_M | Sri | Sorcin | | | | 0.560039 | -0.86547 | -1.48693 | 0.350879 | 1.019588 | 0.941629 | 0.15938 | -0.26664 | -0.45649 | -1.14206 | -0.59845 | -0.85779 | -2.35048 | -1.12093 | -0.16614 | -0.51948 | 0.509911 | -3.4082 | -1.83924 | -0.04034 | -0.71346 |
| P16254 | P16254 | SRP14_MC | Srp14 | Signal recognition particle 14 kDa protein | | | | 0.335656 | -0.44745 | -0.90335 | 0.032806 | -0.0951 | -0.10317 | 0.674929 | 0.677544 | 1.81379 | -3.89543 | -3.00021 | -2.77057 | -3.65985 | -2.57191 | -3.04792 | -3.21247 | -2.78827 | -3.92757 | -2.31265 | -3.77537 | -3.19954 |
| Q8BMA6 | Q8BMA6 | SRP68_MC | Srp68 | Signal recognition particle subunit SRP68 | | | | 0.173858 | -0.21118 | -0.49421 | 0.354262 | 0.028127 | 0.950147 | 0.779882 | 0.835458 | 2.137572 | -1.25826 | -1.07549 | -1.30047 | -1.33743 | -0.4395 | -1.18383 | -1.37311 | -0.53828 | -1.37021 | -1.32395 | -1.52774 | -1.76651 |
| F8VQC1 | F8VQC1 | F8VQC1_ | Srp72 | Signal recognition particle subunit SRP72 | | | | 0.144137 | 0.347945 | 0.41637 | 0.283338 | -0.07594 | -0.77214 | 0.311548 | 0.353743 | 0.842865 | -2.29812 | -3.60257 | -3.48281 | -3.67317 | -1.79141 | -2.6223 | -3.82072 | -2.77586 | -3.5268 | -3.47731 | -2.50106 | -2.62014 |
| Q9DBG7 | Q9DBG7 | SRPRA_M | Srpra | Signal recognition particle receptor subunit alpha | | | | 0.524303 | -1.15441 | -1.39003 | 0.289905 | -0.41468 | -0.78861 | 0.291106 | -0.73811 | -0.79162 | -3.48985 | -3.92077 | -2.21669 | -1.27437 | -1.41623 | -3.2796 | -3.35296 | -1.74884 | -1.56433 | -1.09738 | -1.57334 | -1.64628 |
| P47758 | P47758 | SRPRB_M | Srprb | Signal recognition particle receptor subunit beta | | | | 0.462883 | 0.393942 | 1.227709 | 0.2716 | 0.522314 | 0.742684 | 0.028083 | 0.056258 | 0.088728 | -1.09576 | -1.68091 | -1.67062 | -1.56096 | -1.15032 | -1.78384 | -1.65051 | -1.91405 | -2.83924 | -1.43697 | -0.97408 | -2.07259 |
| Q9QZX7 | Q9QZX7 | SRR_MOU | Srr | Serine racemase | | | | 0.461862 | 0.381805 | 1.22505 | 0.911089 | -0.21094 | -2.58484 | 0.021851 | -0.01243 | -0.06948 | -0.72657 | -0.59817 | 0.821274 | 0.6592 | 1.423711 | 1.302938 | -0.73919 | -1.34916 | 0.960803 | 0.941553 | 1.207303 | 1.544211 |
| Q52KI8 | Q52KI8 | SRRM1_M | Srrm1 | Serine/arginine repetitive matrix protein 1 | | | | 0.689213 | 0.622225 | 1.85631 | 0.302971 | 0.543123 | 0.821363 | 0.410232 | 1.082532 | 1.091988 | -0.73415 | -0.25283 | -1.43589 | -0.46164 | -0.99115 | -1.51372 | -0.8824 | -1.34903 | -1.93905 | -1.04472 | -3.29125 | -1.37868 |
| Q9CZ42 | Q9CZ42 | NNRD_MO | Naxd | ATP-dependent (S)-NAD(P)H-hydrate dehydratase | + | | | 3.651178 | -0.91089 | -66.9139 | 0.232526 | 0.357936 | 0.644264 | 0.825088 | 0.347723 | 2.285872 | -1.28623 | -1.30995 | -0.20758 | -1.06693 | -0.08385 | -0.08788 | -0.38051 | -0.39387 | -1.34739 | -0.643 | -0.28148 | -0.58569 |
| Q80WV7 | Q80WV7 | SRRM3_M | Srrm3 | Serine/arginine repetitive matrix protein 3 | | | | 0.635069 | 2.108526 | 1.697466 | 0.345469 | 0.348321 | 0.928017 | 0.465251 | 1.438588 | 1.233878 | -0.15968 | 0.604142 | 0.219893 | 0.83287 | -0.44639 | -1.28075 | -0.7043 | -3.06829 | -0.03861 | 0.394726 | -3.39087 | -1.21344 |
| Q62093 | Q62093 | SRSF2_MC | Srsf2 | Serine/arginine-rich splicing factor 2 | | | | 0.666488 | 0.417199 | 1.788875 | 0.504529 | 0.41957 | 1.337228 | 0.472021 | 0.486031 | 1.251558 | 0.959924 | 1.425647 | 0.773537 | 0.966958 | 0.393758 | 0.905044 | 0.762687 | 0.788487 | 0.749162 | 0.152193 | -0.12896 | 0.455697 |
| P84104 | P84104 | SRSF3_MC | Srsf3 | Serine/arginine-rich splicing factor 3 | | | | 0.383325 | 1.68147 | 1.023553 | 0.150286 | 0.07921 | 0.432608 | 0.665913 | 0.671321 | 1.787184 | -0.69558 | 0.303438 | -0.45627 | -0.25436 | -0.39965 | 0.276663 | -0.31254 | -3.44253 | -0.58727 | -0.28177 | -0.89636 | -0.56927 |
| P20108 | P20108 | PRDX3_M | Prdx3 | Thioredoxin-dependent peroxide reductase, mitochondrial | + | | | 1.796421 | -0.92787 | -7.81549 | 0.715674 | -0.69174 | -1.93631 | 0.358329 | -0.4807 | -0.96039 | 1.710344 | 1.824549 | 2.706927 | 3.117076 | 2.237785 | 3.234312 | 2.59123 | 2.799406 | 3.311219 | 3.896266 | 3.264268 | 3.16923 |
| P32067 | P32067 | LA_MOUS | Ssb | Lupus La protein homolog | | | | 0.581488 | -0.36777 | -1.54608 | 0.496485 | 1.033503 | 1.315902 | 0.016325 | 0.055444 | 0.052207 | -3.48654 | -3.53655 | -1.89019 | -0.84994 | -1.18963 | -3.30558 | -2.90722 | -3.38033 | -2.99206 | -1.81508 | -2.21062 | -2.39548 |
| Q9CYZ8;Q | Q9D032;Q | SSBP2_M | Ssbp2;Ssbp3 | Single-stranded DNA-binding protein 2 | | | | NaN | 0 | NaN | 0.11582 | 0.132256 | 0.34051 | 0.346962 | -1.31665 | -0.93177 | -3.12778 | -4.09169 | -1.17303 | -0.56531 | -1.28436 | -3.93524 | -3.14663 | -3.10667 | -1.24335 | -0.7595 | -1.78299 | -0.80331 |
| Q62186 | Q62186 | SSRD_MO | Ssr4 | Translocon-associated protein subunit delta | | | | 0.458101 | 0.880236 | 1.215266 | 0.336683 | 0.450565 | 0.905934 | 0.153188 | -0.12333 | -0.44025 | -1.66972 | -2.55275 | -1.1469 | -1.96579 | -1.46942 | -1.93625 | -3.56567 | -2.41728 | -2.28923 | -1.72458 | -1.73442 | -1.42458 |
| Q08943 | Q08943 | SSRP1_MC | Ssrp1 | FACT complex subunit SSRP1 | | | | 0.190636 | 0.820777 | 0.537528 | 0.330636 | -0.36854 | -0.89075 | 0.204491 | -0.26357 | -0.57302 | -1.13488 | -1.88661 | -1.55301 | -0.73254 | -0.58649 | -1.47412 | -0.85156 | -3.81149 | -0.72045 | -0.82801 | -0.88757 | -0.6459 |
| P60041 | P60041 | SMS_MOU | Sst | Somatostatin | | + | | NaN | 0 | NaN | 1.378309 | 1.626755 | 4.733474 | 0.6195 | 0.903687 | 1.652915 | -3.68289 | -3.19632 | -1.57321 | -2.22345 | -1.00119 | -1.79073 | -3.03404 | -3.50522 | -3.41371 | -3.63646 | -2.67788 | -1.92141 |
| Q99L47 | Q99L47 | F10A1_MC | St13 | Hsc70-interacting protein | | | | 0.181567 | -0.33905 | -0.51416 | 0.430653 | 0.981555 | 1.144312 | 0.181151 | -0.20411 | -0.51309 | -2.91713 | -1.66473 | -0.73124 | -2.2436 | -1.4212 | -2.08536 | -2.15852 | -1.74524 | -2.06406 | -2.87389 | -1.33015 | -1.76819 |
| P70297 | P70297 | STAM1_M | Stam | Signal transducing adapter molecule 1 | | + | | 0.308118 | 0.715861 | 0.834265 | 1.045796 | 0.585122 | 3.104135 | 0.036749 | -0.06287 | -0.11512 | -1.06338 | -1.93045 | -0.22278 | -0.17141 | -0.3742 | -1.45891 | -2.95327 | -1.47228 | -0.96896 | -0.59548 | -0.78946 | -0.9179 |
| P42225 | P42225 | STAT1_MC | Stat1 | Signal transducer and activator of transcription 1 | | | | 0.23894 | 0.566977 | 0.660476 | 0.227635 | -0.63932 | -0.63188 | 0.264533 | -0.74265 | -0.72493 | -1.88298 | -3.57658 | -2.76517 | -3.71503 | -2.09206 | -4.06411 | -3.15591 | -3.43761 | -3.49415 | -1.70742 | -2.61333 | -2.05754 |
| Q8CJ67 | Q8CJ67 | STAU2_M | Stau2 | Double-stranded RNA-binding protein Staufen homolog 2 | | | | 0.407768 | 0.655564 | 1.085699 | 0.378894 | 0.961408 | 1.012333 | 0.027455 | -0.05485 | -0.0868 | -2.37062 | -3.29294 | -1.61534 | -1.79062 | -2.82649 | -1.59169 | -3.87712 | -3.09756 | -3.61003 | -1.71874 | -2.0196 | -2.28888 |
| Q5SYD0 | Q5SYD0 | MYO1D_M | Myo1d | Unconventional myosin-Id | + | | | 1.376489 | -0.95234 | -4.72291 | 0.003614 | 0.008549 | 0.01172 | 0.697053 | 0.887127 | 1.879843 | -0.95265 | -0.81007 | -2.53278 | -2.97741 | -1.12195 | -1.03258 | 0.259602 | -0.11764 | -3.45837 | -2.06891 | -1.4946 | -2.43419 |
| P70302 | P70302 | STIM1_M | Stim1 | Stromal interaction molecule 1 | | | | 0.515019 | 0.599504 | 1.365169 | 0.375305 | 0.292974 | 1.003251 | 0.304022 | -0.40621 | -0.824 | -0.96581 | -0.88628 | 0.003356 | 0.36448 | -0.80634 | -0.5519 | -1.96289 | -1.08821 | -0.33857 | 0.120456 | -0.74919 | 0.203371 |
| P83093 | P83093 | STIM2_M | Stim2 | Stromal interaction molecule 2 | | | + | 0.953143 | 1.240747 | 2.739843 | 1.406548 | 0.683131 | 4.900047 | 0.366893 | -0.56005 | -0.982 | -0.05732 | 0.634302 | 1.450151 | 1.652942 | 0.878576 | 1.429815 | -1.24465 | -0.65987 | 0.772734 | 0.964097 | 1.21495 | 2.213541 |
| Q60864 | Q60864 | STIP1_MO | Stip1 | Stress-induced-phosphoprotein 1 | | | | 0.416774 | -0.30423 | -1.10871 | 0.437447 | 0.875408 | 1.161803 | 0.065431 | -0.11557 | -0.19976 | -1.03291 | -0.48953 | 0.560829 | -0.85512 | -0.55311 | -0.41295 | -0.41852 | -0.49545 | -1.28047 | -0.76463 | 0.206826 | -0.94175 |
| P54227 | P54227 | STMN1_M | Stmn1 | Stathmin | | | | 0.812426 | -0.46997 | -2.24375 | 0.250487 | 0.58567 | 0.689599 | 0.021156 | 0.065835 | 0.067318 | -0.11604 | 0.177389 | 1.605202 | -0.01206 | 0.84421 | -0.9996 | 0.650135 | 0.351156 | 0.470535 | -0.04873 | 0.182858 | -0.46992 |
| P55821 | P55821 | STMN2_M | Stmn2 | Stathmin-2 | | | + | 0.776898 | -1.98255 | -2.12798 | 2.211867 | 0.291049 | 12.70359 | 0.095857 | 0.167153 | 0.285758 | -3.95593 | -2.14239 | -0.08281 | -0.03855 | -0.67641 | -1.76194 | -1.28052 | -0.85269 | -0.34579 | -0.35766 | -1.60444 | -1.16822 |
| O70166 | O70166 | STMN3_M | Stmn3 | Stathmin-3 | | | | 0.928683 | 0.721716 | 2.648947 | 0.350864 | 0.338545 | 0.941592 | 0.416305 | 0.276646 | 1.107511 | -0.70322 | -1.23304 | -0.47688 | -0.97324 | -0.94482 | -1.4434 | -1.75352 | -1.62617 | -0.80345 | -1.32375 | -1.45499 | -1.48652 |
| B2RXT3 | B2RXT3 | B2RXT3_M | Ogdhl | oxoglutarate dehydrogenase (succinyl-transferring) | + | | | 1.604791 | -0.96124 | -6.22564 | 0.286827 | -0.19364 | -0.78089 | 0.009317 | -0.01031 | -0.03002 | -0.06119 | -0.13139 | 0.063339 | -0.39771 | -0.18665 | 0.00845 | 1.015304 | 0.71459 | -0.06493 | 0.117839 | 0.2506 | -0.40817 |
| Q8BZ60 | Q8BZ60 | STON2_M | Ston2 | Stonin-2 | | | + | 0.78543 | 0.536157 | 2.155467 | 1.51578 | 0.932651 | 5.594701 | 0.240906 | 0.357868 | 0.665441 | -0.79266 | -0.64249 | -1.07292 | -0.80472 | -1.25515 | -0.63753 | -1.01659 | -1.49087 | -1.9705 | -1.77244 | -1.7445 | -0.86392 |
| Q499E5 | Q499E5 | STOX2_M | Stox2 | Storkhead-box protein 2 | | | | NaN | 0 | NaN | 0.265449 | 0.817356 | 0.727235 | 0.337699 | -0.35851 | -0.90849 | -3.87554 | -3.91081 | -2.33772 | -1.9385 | -2.30364 | -2.49623 | -3.02556 | -3.39072 | -4.06152 | -1.84941 | -2.42412 | -1.65873 |
| Q9Z1Z2 | Q9Z1Z2 | STRAP_MC | Strap | Serine-threonine kinase receptor-associated protein | + | | | 1.121769 | 0.524246 | 3.428702 | 0.312753 | -0.03675 | -0.84589 | 0.620841 | 0.394305 | 1.656733 | 0.206612 | 0.468603 | 0.449832 | 0.36844 | 0.664772 | 0.218265 | -0.10778 | -0.26549 | 0.430685 | 0.461081 | 0.129691 | -0.03526 |
| Q91WM1 | Q91WM1 | STRBP_M | Strbp | Spermatid perinuclear RNA-binding protein | | | | NaN | 0 | NaN | 0.402334 | 0.513751 | 1.071846 | 0.116974 | -0.1952 | -0.34364 | -2.85304 | -3.1882 | -3.11712 | -2.34453 | -2.58148 | -3.70862 | -3.51628 | -3.33081 | -3.52834 | -2.96081 | -3.02102 | -2.87868 |
| Q8C079 | Q8C079 | STRP1_MC | Strip1 | Striatin-interacting protein 1 | | | | 0.464605 | 0.058269 | 1.232195 | 0.617618 | -0.6815 | -1.64756 | 0.937298 | -0.7904 | -2.68072 | -1.24157 | -1.14965 | -0.24079 | 0.354015 | -0.38389 | 0.198824 | -1.265 | -1.24276 | 0.450622 | 1.025608 | 0.743091 | 0.652647 |
| Q8C9H6 | Q8C9H6 | STRP2_MC | Strip2 | Striatin-interacting proteins 2 | + | | | 1.101425 | 0.988875 | 3.339347 | 0.657405 | 0.798131 | 1.762237 | 0.397132 | -0.2761 | -1.0586 | 2.003271 | 2.568055 | 3.621904 | 4.51445 | 2.55225 | 2.511414 | 1.385932 | 1.207644 | 3.192806 | 3.347286 | 2.547917 | 3.067947 |
| O55106 | O55106 | STRN_MO | Strn | Striatin | | | | 0.309882 | 0.20285 | 0.838687 | 0.176874 | 0.207101 | 0.502026 | 0.589979 | -0.86607 | -1.56971 | 0.600388 | 0.631676 | 2.808965 | 3.618791 | 0.895644 | 1.94662 | 0.654542 | 0.171822 | 2.927871 | 3.085683 | 2.119042 | 2.455369 |
| Q9ERG2 | Q9ERG2 | STRN3_M | Strn3 | Striatin-3 | + | | | 1.139387 | 0.96069 | 3.507592 | 0.116143 | -0.11108 | -0.34139 | 0.553619 | -0.82389 | -1.46937 | 0.398637 | 0.862773 | 1.650718 | 2.294321 | 0.291247 | 1.374679 | -0.18452 | -0.47545 | 2.131694 | 2.035502 | 1.51215 | 1.801557 |
| P58404 | P58404 | STRN4_M | Strn4 | Striatin-4 | | | | 0.696041 | 0.903917 | 1.876796 | 0.327571 | -0.30621 | -0.88305 | 0.494498 | -0.82442 | -1.31065 | -0.64276 | 0.292994 | 2.082238 | 2.77431 | 0.736808 | 1.992731 | -0.96455 | -1.19306 | 2.75683 | 2.712129 | 2.15272 | 2.225663 |
| P46978 | P46978 | STT3A_MC | Stt3a | Dolichyl-diphosphooligosaccharide--protein glycosyltransferase subunit STT3A | | | | 0.798509 | 0.516622 | 2.197985 | 0.014117 | -0.0101 | -0.04525 | 0.507043 | 0.450523 | 1.34391 | 1.74252 | 1.470517 | 0.390669 | 0.010988 | 1.769447 | 1.15429 | 0.898196 | 1.281597 | 0.328318 | 0.093541 | 0.878018 | 1.144673 |
| P35486 | P35486 | ODPA_MO | Pdha1 | Pyruvate dehydrogenase E1 component subunit alpha, somatic form, mitochondrial | + | | | 1.609474 | -0.96634 | -6.26059 | 0.883019 | -0.25663 | -2.48469 | 0.112833 | 0.207924 | 0.332389 | 2.211291 | 2.311676 | 2.502275 | 2.397338 | 2.579706 | 3.433427 | 3.373792 | 3.081862 | 2.795402 | 2.617472 | 3.255911 | 2.341375 |
| Q0VBF8 | Q0VBF8 | STUM_MO | Stum | Protein stum homolog | | | | 0.186025 | -0.38584 | -0.52566 | 0.159397 | 0.439055 | 0.456535 | 0.904796 | 1.234907 | 2.562163 | -3.558 | -2.71859 | -0.94627 | -2.69999 | -0.86822 | 0.091882 | -3.35464 | -2.15027 | -1.8672 | -2.65717 | -1.66614 | -1.58001 |
| Q9ER00 | Q9ER00 | STX12_MC | Stx12 | Syntaxin-12 | | | | 0.26675 | 0.571727 | 0.730503 | 0.259624 | 1.198759 | 0.712593 | 0.17361 | -0.26367 | -0.49357 | -2.24618 | -3.7632 | -0.95781 | -1.68459 | -0.71491 | -1.67025 | -3.76927 | -3.38356 | -4.16249 | -0.87743 | -0.6897 | -1.16811 |
| Q9D0I4 | Q9D0I4 | STX17_MC | Stx17 | Syntaxin-17 | | | | NaN | 0 | NaN | 0.825548 | 1.112305 | 2.287411 | 0.155461 | -0.56697 | -0.44622 | -3.64749 | -3.52902 | -1.17213 | -1.72734 | -0.61683 | -3.15508 | -3.35349 | -4.04081 | -2.96129 | -2.1628 | -1.25755 | -1.38042 |
| Q8R1Q0 | Q8R1Q0 | STX19_MC | Stx19 | Syntaxin-19 | | + | | 0.034642 | 0.294553 | 0.108744 | 2.028382 | -3.84073 | -10.2594 | 0.335355 | -1.82057 | -0.9026 | -3.15594 | 0.343977 | -3.7743 | -3.80206 | 0.074182 | -3.88697 | -3.76806 | 0.366987 | 0.426657 | -0.32155 | 0.295947 | -0.46759 |
| O35526 | O35526 | STX1A_MC | Stx1a | Syntaxin-1A | | | | 0.343543 | -1.22561 | -0.92318 | 0.146671 | -0.32377 | -0.42307 | 0.14859 | 0.213365 | 0.428138 | -0.6316 | -3.24533 | 1.899365 | 0.396935 | 0.990779 | 1.21055 | -0.47912 | -0.94659 | 1.32579 | 1.61806 | 1.373389 | 0.40121 |
| P61264 | P61264 | STX1B_MC | Stx1b | Syntaxin-1B | | | | 0.919876 | -0.42403 | -2.61673 | 0.040469 | 0.081627 | 0.126329 | 0.104686 | 0.170271 | 0.310117 | 2.126929 | 2.421531 | 3.015992 | 1.726814 | 2.117689 | 2.800529 | 2.630723 | 2.765798 | 2.244965 | 2.334588 | 2.718829 | 1.858847 |
| Q9JKK1 | Q9JKK1 | STX6_MO | Stx6 | Syntaxin-6 | | | | 0.051254 | 0.116961 | 0.158414 | 0.708505 | 0.598246 | 1.914474 | 0.195715 | -0.3992 | -0.55057 | -3.92259 | -2.48912 | -1.49607 | -1.6046 | -3.73196 | -2.38808 | -3.14556 | -3.50007 | -1.84084 | -2.45632 | -2.38837 | -2.93326 |
| O08599 | O08599 | STXB1_MC | Stxbp1 | Syntaxin-binding protein 1 | | | | 0.896479 | -0.63435 | -2.53239 | 0.6463 | -1.0468 | -1.72991 | 0.014697 | -0.02409 | -0.04708 | 3.548096 | 3.83832 | 4.697381 | 3.500582 | 4.261242 | 5.111724 | 4.531736 | 4.123376 | 5.055857 | 5.2357 | 4.995257 | 4.425898 |
| Q8K400 | Q8K400 | STXB5_MC | Stxbp5 | Syntaxin-binding protein 5 | | | | 0.008366 | -0.0154 | -0.02699 | 0.544646 | -0.39533 | -1.44494 | 0.599298 | 0.309054 | 1.595805 | -1.68559 | -0.54623 | 0.346211 | -0.20089 | 0.697566 | 0.720692 | -1.0674 | -1.13362 | 0.472908 | 0.46307 | 0.593396 | 0.206755 |
| Q5DQR4 | Q8K400;Q | STB5L_MC | Stxbp5l | Syntaxin-binding protein 5-like | | | | 0.470763 | 1.057808 | 1.248269 | 0.707868 | -0.16605 | -1.91254 | 0.585614 | 0.133734 | 1.557549 | -0.78752 | 0.57885 | -0.02021 | -0.12232 | 0.384487 | 0.55516 | -0.66077 | -1.66352 | 0.024561 | 0.165002 | 0.326606 | 0.345573 |
| Q8R3T5 | Q8R3T5 | STXB6_MC | Stxbp6 | Syntaxin-binding protein 6 | + | + | | 1.028439 | -1.12405 | -3.03337 | 1.379091 | -0.88625 | -4.73802 | 0.247652 | -0.21901 | -0.68245 | -0.31722 | -0.40952 | -0.294 | -0.65432 | -0.28219 | 0.102783 | 1.128353 | 0.393002 | 0.462389 | 0.361779 | 0.38609 | -0.12748 |

| | | | | | | | | | | | | | | | | | | | | | | | | | | | | |
|---|---|---|---|---|---|---|---|---|---|---|---|---|---|---|---|---|---|---|---|---|---|---|---|---|---|---|---|---|
| P11031 | P11031 | TCP4_MOI | Sub1 | Activated RNA polymerase II transcriptional coactivator p15 | | + | | 0.092312 | 0.131207 | 0.275906 | 1.007575 | 0.580796 | 2.949879 | 0.207932 | -0.3398 | -0.58181 | 1.116928 | 1.075615 | 2.81603 | 2.885363 | 2.402117 | 2.204392 | 1.440166 | 0.489963 | 2.463712 | 2.076087 | 2.067439 | 3.218676 |
| Q9Z2I9 | Q9Z2I9 | SUCB1_M( | Sucla2 | Succinate--CoA ligase [ADP-forming] subunit beta, mitochondrial | | + | | 0.613145 | -0.41834 | -1.63487 | 1.952264 | 0.473715 | 9.38583 | 0.597133 | 0.553437 | 1.589729 | 2.198768 | 2.059899 | 2.42136 | 2.405813 | 2.897693 | 2.293801 | 2.793957 | 2.301387 | 1.989741 | 1.890003 | 2.215588 | 1.869031 |
| Q9WUM5 | Q9WUM5 | SUCA_MO | Suclg1 | Succinate--CoA ligase [ADP/GDP-forming] subunit alpha, mitochondrial | | | | 0.347443 | -0.33122 | -0.93298 | 0.502295 | -0.53687 | -1.3313 | 0.022395 | -0.03825 | -0.07117 | 1.048339 | 1.577691 | 1.914082 | 1.155462 | 1.564104 | 2.634243 | 1.880834 | 1.407634 | 2.20858 | 1.93471 | 2.187732 | 2.08711 |
| Q9Z2I8 | Q9Z2I8 | SUCB2_M( | Suclg2 | Succinate--CoA ligase [GDP-forming] subunit beta, mitochondrial | | | | 0.202955 | 0.198119 | 0.5691 | 0.89852 | 0.902004 | 2.539679 | 0.126495 | -0.20536 | -0.36933 | -1.47977 | -2.0449 | -1.72762 | -2.28133 | -1.68705 | -2.69124 | -1.7571 | -2.1638 | -3.12895 | -2.68401 | -2.22266 | -1.74491 |
| Q8CH09 | Q8CH09 | SUGP2_M( | Sugp2 | SURP and G-patch domain-containing protein 2 | | | | 0.574753 | 1.727741 | 1.527421 | 0.016563 | 0.011987 | 0.052957 | 0.10638 | 0.255799 | 0.314764 | -0.29523 | -1.47231 | -0.35608 | -0.00833 | 0.531463 | -1.06072 | -1.64553 | -3.57749 | -0.33914 | -0.04925 | -0.68374 | -0.35711 |
| Q9CX34 | Q9CX34 | SGT1_MOI | Sugt1 | Protein SGT1 homolog | | | | 0.019285 | 0.054327 | 0.061481 | 0.396487 | 0.28492 | 1.056965 | 0.477828 | 0.76995 | 1.266765 | -4.12595 | -2.74896 | -1.78506 | -2.31876 | -1.13639 | -2.32086 | -4.04567 | -2.9379 | -2.37499 | -2.29868 | -2.36186 | -2.63529 |
| P63046 | P63046 | ST4A1_MC | Sult4a1 | Sulfotransferase 4A1 | | | | 0.467184 | 1.086455 | 1.238921 | 0.08982 | -0.21932 | -0.26896 | 0.248589 | 0.192298 | 0.684817 | -1.36096 | -1.24183 | -0.98826 | -2.53223 | -1.64817 | -1.60568 | -1.51294 | -3.26276 | -1.27832 | -1.80354 | -2.09922 | -1.53923 |
| Q8BHK1 | Q8BHK1 | NIPA1_MC | Nipa1 | Magnesium transporter NIPA1 | + | | | 1.350788 | -0.97442 | -4.57594 | 0.194155 | -0.11234 | -0.54657 | 0.777116 | 1.060712 | 2.128681 | -0.90292 | -1.07787 | -1.73841 | -2.13451 | -1.19179 | -1.48278 | 0.178179 | -0.21012 | -1.87908 | -1.76916 | -2.87458 | -1.92141 |
| O55201 | O55201 | SPT5H_MC | Supt5h | Transcription elongation factor SPT5 | | | | NaN | 0 | NaN | 0.020941 | 0.039135 | 0.066646 | 0.448526 | 1.069968 | 1.190424 | -2.83447 | -3.8822 | -3.11506 | -4.06782 | -1.4828 | -3.26659 | -3.56222 | -2.98995 | -3.28725 | -3.9739 | -3.55597 | -3.33336 |
| Q80YD1 | Q80YD1 | SUV3_MOI | Supv3l1 | ATP-dependent RNA helicase SUPV3L1, mitochondrial | | | | 0.001917 | 0.004198 | 0.00623 | 0.435824 | 0.79676 | 1.15762 | 0.259406 | 0.952232 | 0.712046 | -2.97909 | -2.96368 | -2.16587 | -3.50989 | -0.97639 | -3.64156 | -2.30175 | -3.64942 | -3.78339 | -3.48589 | -3.37366 | -3.14875 |
| Q61016 | Q61016 | GBG7_MO | Gng7 | Guanine nucleotide-binding protein G(I)/G(S)/G(O) subunit gamma-7 | + | | | 2.001404 | -0.97901 | -9.94114 | 0.123535 | -0.10176 | -0.36137 | 0.207704 | -0.75737 | -0.58123 | -0.40358 | -0.36672 | 1.558403 | 0.995578 | 0.179893 | 2.425163 | 0.690603 | 0.497121 | 1.368566 | 1.388935 | 2.721435 | 1.398357 |
| Q8BI08 | Q8BI08 | MAL2_MOI | Mal2 | Protein MAL2 | + | | | 1.402378 | -0.99307 | -4.87513 | 0.698986 | -0.91242 | -1.88567 | 0.081836 | -0.31037 | -0.24655 | -0.06936 | -0.41095 | 1.253953 | 0.291757 | 0.276751 | 2.065779 | 0.641906 | 0.863923 | 1.633553 | 1.736994 | 2.367374 | 0.595889 |
| Q8BG39 | Q8BG39 | SV2B_MOI | Sv2b | Synaptic vesicle glycoprotein 2B | | | | 0.259737 | 0.332562 | 0.712877 | 0.628174 | 0.322244 | 1.677676 | 0.217223 | 0.067818 | 0.605464 | -0.3521 | 0.486027 | 2.532533 | 2.185913 | 2.469046 | 2.624346 | -0.06062 | -0.47057 | 2.119789 | 1.95417 | 2.559604 | 2.398152 |
| P17809 | P17809 | GTR1_MOI | Slc2a1 | Solute carrier family 2, facilitated glucose transporter member 1 | + | | | 1.444156 | -0.99764 | -5.12994 | 0.841874 | -0.49509 | -2.34243 | 0.08909 | 0.132708 | 0.266916 | 1.343417 | 1.103211 | 0.659923 | 0.321644 | 1.025542 | 1.417722 | 2.373905 | 2.067996 | 0.85913 | 1.112611 | 1.545813 | 0.632035 |
| Q3UZP4 | Q3UZP4 | SVIP_MOU | Svip | Small VCP/p97-interacting protein | | + | | 0.757712 | -0.35852 | -2.06689 | 1.474966 | -0.31436 | -5.32541 | 0.015648 | 0.022282 | 0.050078 | 0.136664 | -0.13245 | -1.27416 | -1.38349 | -0.77064 | -1.44875 | 0.470086 | 0.251163 | -0.9922 | -1.03674 | -0.84384 | -1.42012 |
| Q8BHS8 | Q8BHS8 | SYBU_MOI | Sybu | Syntabulin | | | | 0.072229 | 0.357907 | 0.219272 | 0.324535 | 0.200386 | 0.875436 | 0.450779 | -0.31397 | -1.19626 | -1.00422 | -3.91923 | -0.77838 | -0.4315 | -1.09387 | -1.43767 | -2.08486 | -3.55441 | -0.65595 | -0.9547 | -1.15013 | -0.75347 |
| O88935 | O88935 | SYN1_MOI | Syn1 | Synapsin-1 | | | | 0.239374 | 0.267102 | 0.661573 | 0.180138 | 0.24632 | 0.510471 | 0.553918 | 1.031181 | 1.470188 | 1.679404 | 2.014296 | 3.579626 | 2.643657 | 4.658062 | 3.297599 | 1.947125 | 1.212369 | 2.747713 | 2.982929 | 3.117643 | 2.775656 |
| P47791 | P47791 | GSHR_MO | Gsr | Glutathione reductase, mitochondrial | + | | | 1.390293 | -1.00794 | -4.80355 | 0.031872 | 0.073496 | 0.100321 | 0.069969 | -0.15862 | -0.21281 | -1.35509 | -1.08363 | -0.78268 | -1.61533 | -1.6316 | -0.96811 | -0.37144 | -0.0514 | -0.66969 | -1.87531 | -0.47377 | -1.80871 |
| Q8JZP2 | Q8JZP2 | SYN3_MOI | Syn3 | Synapsin-3 | | | | 0.238364 | 0.577661 | 0.659023 | 0.342017 | 0.353332 | 0.919337 | 0.349189 | 0.358342 | 0.937376 | -1.38087 | -2.17365 | -0.94858 | -1.10328 | -0.59501 | -1.3455 | -1.57313 | -3.13671 | -1.75573 | -1.00279 | -1.25559 | -1.40161 |
| P58389 | P58389 | PTPA_MOI | Ptpa | Serine/threonine-protein phosphatase 2A activator | + | | | 1.532864 | -1.03566 | -5.71104 | 0.683909 | -0.97338 | -1.84047 | 0.377646 | -0.83536 | -1.00917 | -1.31248 | -1.17653 | -0.7044 | -1.57085 | -1.76954 | -0.29006 | -0.04072 | -0.37697 | -0.4676 | 0.139119 | 0.177019 | -0.5659 |
| Q6ZWR6 | Q6ZWR6 | SYNE1_MC | Syne1 | Nesprin-1 | | + | | 0.188675 | -0.68566 | -0.53248 | 1.068047 | -0.32926 | -3.19665 | 0.448867 | 0.317463 | 1.191309 | -3.57951 | -2.2255 | -1.41016 | -1.46458 | -1.06399 | -0.92543 | -1.1215 | -3.31218 | -1.00876 | -1.20745 | -1.56949 | -1.05485 |
| F6SEU4 | F6SEU4 | SYGP1_MC | Syngap1 | Ras/Rap GTPase-activating protein SynGAP | | | | 0.590974 | 0.632532 | 1.572492 | 0.348886 | -0.21842 | -0.93661 | 0.206283 | -0.25944 | -0.5776 | -0.57256 | -0.29272 | 3.225646 | 3.62541 | 4.189816 | 3.702012 | -0.68805 | -1.4423 | 3.523818 | 3.764087 | 3.828171 | 4.582545 |
| O55100 | O55100 | SNG1_MO | Syngr1 | Synaptogyrin-1 | | | | 0.090342 | -0.07183 | -0.27041 | 0.317575 | 0.472961 | 0.857978 | 0.055341 | 0.069717 | 0.170423 | -0.19858 | 0.270154 | 1.784831 | 0.684565 | 0.50631 | 1.078761 | 0.23268 | -0.01744 | 0.726655 | 0.796818 | 1.015088 | 0.43055 |
| Q8R191 | Q8R191 | SNG3_MO | Syngr3 | Synaptogyrin-3 | | | | 0.547637 | 0.251396 | 1.453074 | 0.163376 | -0.07671 | -0.46694 | 0.276245 | 0.134808 | 0.754344 | 3.401274 | 3.44542 | 3.861278 | 3.653122 | 4.128174 | 4.138364 | 3.343547 | 3.000354 | 3.961002 | 3.706809 | 4.177097 | 3.819825 |
| Q8CHC4 | Q8CHC4 | SYNJ1_MC | Synj1 | Synaptojanin-1 | | | | 0.886464 | 0.093594 | 2.496848 | 0.01264 | -0.00552 | -0.04058 | 0.225807 | -0.05789 | -0.62725 | 2.803964 | 2.759082 | 4.15821 | 4.131084 | 4.244804 | 4.064781 | 2.717955 | 2.657904 | 4.014738 | 4.285605 | 4.192317 | 4.233042 |
| Q9D6K5 | Q9D6K5 | SYJ2B_MO | Synj2bp | Synaptojanin-2-binding protein | | + | | 0.009685 | 0.012164 | 0.031196 | 1.003472 | -0.82557 | -2.93366 | 0.512607 | -0.40282 | -1.35873 | -0.31193 | -0.99875 | -1.54627 | -1.99221 | -1.23327 | -0.65156 | -0.8522 | -0.4828 | -0.77198 | -1.11537 | -0.59701 | -0.48219 |
| Q8CC35 | Q8CC35 | SYNPO_M( | Synpo | Synaptopodin | | + | | 0.157184 | -0.17533 | -0.45074 | 1.596325 | -1.61563 | -6.16293 | 0.26791 | 0.248173 | 0.733419 | -3.61489 | -3.06216 | -0.20063 | -0.52285 | 1.241837 | 1.458418 | -2.88944 | -3.43695 | 1.460691 | 1.047088 | 1.422537 | 0.781371 |
| Q9CR68 | Q9CR68 | UCRI_MOI | Uqcrfs1 | Cytochrome b-c1 complex subunit Rieske, mitochondrial | + | | | 3.339723 | -1.04507 | -46.7425 | 0.706637 | -0.41986 | -1.9088 | 0.104454 | 0.104483 | 0.309479 | 2.887186 | 2.931878 | 2.991453 | 2.557416 | 3.626329 | 4.068594 | 3.95387 | 3.955334 | 3.158442 | 3.230148 | 3.998089 | 3.487867 |
| Q5SV85 | Q5SV85 | SYNRG_M( | Synrg | Synergin gamma | | | | NaN | 0 | NaN | 0.161386 | 0.150009 | 0.461738 | 0.914069 | 0.89568 | 2.595624 | -3.12278 | -3.46005 | -2.42498 | -2.23459 | -2.53354 | -2.62754 | -3.01052 | -3.11904 | -2.79041 | -2.16917 | -3.13436 | -3.81808 |
| Q62277 | Q62277 | SYPH_MOI | Syp | Synaptophysin | + | | | 1.287633 | -0.43268 | -4.23151 | 0.277087 | -0.57555 | -0.75646 | 0.040679 | 0.099908 | 0.12696 | 3.41865 | 3.568734 | 4.604733 | 3.125793 | 4.14185 | 5.151397 | 3.995827 | 3.856915 | 4.619923 | 4.261706 | 5.150416 | 3.943016 |
| P24527 | P24527 | LKHA4_MC | Lta4h | Leukotriene A-4 hydrolase | + | | | 1.936755 | -1.05753 | -9.21688 | 0.924956 | -0.75267 | -2.63528 | 0.133587 | -0.13795 | -0.38833 | -1.97713 | -1.89533 | -1.24807 | -1.75191 | -1.26379 | -0.73159 | -0.7715 | -0.9859 | -0.8819 | -0.61273 | -0.62439 | -1.09509 |
| P46096 | Q9R0N5;P | SYT1_MOI | Syt1 | Synaptotagmin-1 | | | | 0.674145 | 0.292015 | 1.811467 | 0.010268 | 0.020777 | 0.033053 | 0.77117 | 0.595197 | 2.10964 | 2.057185 | 1.893975 | 3.644533 | 2.414864 | 3.555181 | 3.334283 | 1.822587 | 1.544542 | 2.878101 | 3.139741 | 3.109149 | 2.58992 |
| Q9R0P9 | Q9R0P9 | UCHL1_M( | Uchl1 | Ubiquitin carboxyl-terminal hydrolase isozyme L1 | + | | | 1.318403 | -1.06068 | -4.39643 | 0.038053 | 0.060647 | 0.11906 | 0.151719 | 0.21628 | 0.436382 | 2.306405 | 1.954727 | 3.005689 | 1.986921 | 2.232933 | 2.987369 | 3.356436 | 3.02606 | 2.43496 | 2.436356 | 2.715347 | 2.072394 |
| Q9R0N3 | Q9R0N3 | SYT11_MC | Syt11 | Synaptotagmin-11 | | | | NaN | 0 | NaN | 0.604989 | 1.149089 | 1.611815 | NaN | 0 | NaN | -3.55456 | -4.3389 | -2.22968 | -3.35944 | -3.46725 | -3.98608 | -2.50632 | -3.20697 | -4.37857 | -3.50872 | -2.98375 | -3.44548 |
| Q920N7 | Q920N7 | SYT12_MC | Syt12 | Synaptotagmin-12 | | | | 0.878393 | 0.810985 | 2.468437 | 0.12384 | 0.207054 | 0.362192 | 0.539082 | 1.104264 | 1.429863 | -2.50436 | -2.16038 | -2.16833 | -2.79833 | -0.87686 | -2.40696 | -2.86343 | -3.42329 | -2.21333 | -3.16744 | -2.64069 | -2.85165 |
| P46097 | P46097 | SYT2_MOI | Syt2 | Synaptotagmin-2 | | | | 0.102773 | -0.05539 | -0.30486 | 0.488258 | -0.77079 | -1.29418 | 0.464629 | 0.31606 | 1.232257 | 2.320259 | 2.623199 | -0.25983 | -1.44224 | -0.37163 | -0.83432 | 2.426801 | 2.627428 | -0.15229 | -0.0082 | -1.02979 | -0.80828 |
| O35681 | O35681 | SYT3_MOI | Syt3 | Synaptotagmin-3 | | | | 0.00266 | -0.00908 | -0.00863 | 0.89011 | -1.80181 | -2.50975 | 0.141288 | -0.71262 | -0.40882 | -1.4792 | -1.75814 | -2.91698 | -1.49924 | 0.110212 | -3.36684 | -0.56718 | -2.652 | -0.29268 | -0.51993 | -0.78937 | -1.04202 |
| Q9R0N5 | Q9R0N5 | SYT5_MOI | Syt5 | Synaptotagmin-5 | | | | 0.094648 | 0.238086 | 0.282401 | 0.304671 | 0.307842 | 0.825624 | 0.473749 | 0.252443 | 1.256078 | -2.09674 | -3.39273 | -0.13288 | -0.68085 | -0.3425 | -0.58704 | -3.52216 | -2.44349 | -0.46181 | -0.96761 | -0.5577 | -0.87672 |
| Q9R0N8 | Q9R0N8 | SYT6_MOI | Syt6 | Synaptotagmin-6 | + | | | 1.198302 | -0.51574 | -3.78199 | 0.060361 | 0.081692 | 0.185073 | 0.130069 | 0.2618 | 0.378919 | 0.172278 | 0.200441 | 1.50241 | 2.044893 | -0.18513 | 1.045166 | 0.837736 | 0.56646 | 1.343725 | 2.040194 | 0.482793 | -0.14635 |
| Q9D0F9 | Q9D0F9 | PGM1_MO | Pgm1 | Phosphoglucomutase-1 | + | | | 2.075021 | -1.06325 | -10.8332 | 0.177454 | -0.46825 | -0.50353 | 0.410669 | -0.48137 | -1.0931 | -0.12949 | -0.32523 | -0.07075 | -1.88236 | -0.86635 | -0.273 | 0.843284 | 0.82851 | -0.2978 | -0.71881 | 0.237142 | -0.41374 |
| Q9DBY1 | Q9DBY1 | SYVN1_MC | Syvn1 | E3 ubiquitin-protein ligase synoviolin | | | | 0.423657 | -0.1867 | -1.12635 | 0.153519 | 0.107904 | 0.441115 | 0.506258 | -0.83966 | -1.34182 | 1.054759 | 0.863351 | 0.992046 | 0.747698 | -0.61194 | 0.584856 | 1.281087 | 1.010417 | 0.973889 | 0.550047 | 0.643093 | 1.009146 |
| Q5QD07 | Q5QD07 | TAA8A_MC | Taar8a | Trace amine-associated receptor 8a | | | | 0.217859 | 0.514722 | 0.607081 | 0.636014 | 1.497584 | 1.700186 | 0.447223 | 0.814662 | 1.187052 | -3.69783 | -2.08015 | -1.88189 | -1.09201 | -1.29745 | -3.21204 | -3.14945 | -3.65797 | -3.77187 | -2.1972 | -2.15719 | -3.08163 |
| Q8CF89 | Q8CF89 | TAB1_MOI | Tab1 | TGF-beta-activated kinase 1 and MAP3K7-binding protein 1 | | | | NaN | 0 | NaN | 0.427227 | -0.73071 | -1.13551 | 0.810664 | -0.82129 | -2.23793 | -2.81417 | -3.39759 | -2.67444 | -1.39684 | -2.10494 | -2.21878 | -3.46402 | -2.57586 | -1.22719 | -1.38266 | -1.70312 | -0.97803 |
| Q6Y685 | Q6Y685 | TACC1_M( | Tacc1 | Transforming acidic coiled-coil-containing protein 1 | | | | 0.063045 | -0.29092 | -0.19286 | 0.00258 | 0.005009 | 0.008375 | 0.26262 | -0.27809 | -0.72013 | -1.33267 | -3.58272 | -1.35027 | -0.97929 | -1.31729 | -1.85243 | -1.16195 | -3.17161 | -0.60116 | -1.73842 | -1.02833 | -1.58522 |
| Q9WVA4 | Q9WVA4 | TAGL2_MC | Tagln2 | Transgelin-2 | | | + | 0.376889 | -0.68646 | -1.00726 | 0.335115 | -0.59234 | -0.902 | 1.420161 | 0.912705 | 4.982178 | -1.78403 | -2.89223 | -1.66184 | -2.84851 | -2.27097 | -1.97699 | -2.04845 | -1.25489 | -1.3814 | -1.94426 | -3.14602 | -2.92735 |
| Q9R1Q8 | Q9R1Q8 | TAGL3_MC | Tagln3 | Transgelin-3 | | | | 0.735017 | -0.44331 | -1.99585 | 0.001622 | 0.005685 | 0.005271 | 0.140535 | 0.127187 | 0.406824 | -0.17674 | 0.132937 | 0.494449 | -1.66195 | -0.31084 | -0.50719 | 0.262159 | 0.580671 | -0.55849 | -0.62039 | -0.23938 | -0.83302 |
| Q93092 | Q93092 | TALDO_M( | Taldo1 | Transaldolase | | | | 0.582681 | -1.79679 | -1.54939 | 0.557825 | 0.414768 | 1.480864 | 0.385739 | 0.54845 | 1.029672 | -3.11314 | -0.84703 | -0.98614 | -1.4691 | -0.98254 | -0.42301 | 0.063735 | -0.43033 | -1.78428 | -1.5005 | -0.79797 | -1.70448 |
| Q9JJA9 | Q9JJA9 | GRASP_M( | Tamalin | General receptor for phosphoinositides 1-associated scaffold protein | | | | NaN | 0 | NaN | 0.184377 | 0.095502 | 0.521413 | 0.016709 | -0.02551 | -0.05342 | -3.46929 | -3.67004 | 0.631682 | 0.994975 | 0.711282 | 0.96851 | -2.9651 | -2.87758 | 0.741317 | 0.694336 | 0.405517 | 1.325292 |
| Q3TUH1 | Q3TUH1 | TAM41_M( | Tamm41 | Phosphatidate cytidylyltransferase, mitochondrial | | | | 0.02037 | -0.01506 | -0.06487 | 0.159071 | -0.13822 | -0.45568 | 0.315729 | -0.35583 | -0.85335 | -0.45447 | 0.009844 | -0.52065 | -0.72362 | -0.33461 | -0.62804 | -0.2024 | -0.2121 | -0.76976 | -0.19807 | -0.51581 | 0.264819 |
| Q0VGY8 | Q0VGY8 | TANC1_M( | Tanc1 | Protein TANC1 | | | | 0.65408 | 1.301936 | 1.752529 | 0.874826 | 0.608541 | 2.455947 | 0.055885 | -0.05287 | -0.17202 | -0.69716 | -0.9709 | -0.36952 | 0.014926 | 0.381885 | 0.581384 | -1.40579 | -2.86614 | -0.94219 | -0.62948 | 0.243769 | 0.825249 |
| A2A690 | A2A690 | TANC2_M( | Tanc2 | Protein TANC2 | | + | | 0.010035 | -0.01031 | -0.03231 | 2.17786 | -0.5475 | -12.2112 | 0.626813 | -0.80274 | -1.67378 | -0.39306 | -0.74789 | 1.478249 | 1.506672 | 0.375356 | 1.105104 | -0.29489 | -0.82544 | 1.997441 | 2.082489 | 1.231714 | 1.854217 |
| P15532 | P15532;Q( | NDKA_MO | Nme1 | Nucleoside diphosphate kinase A | + | | | 1.764398 | -1.08891 | -7.52556 | 0.172637 | -0.31468 | -0.49104 | 0.062749 | 0.198666 | 0.192003 | 2.34731 | 2.501102 | 2.87526 | 1.609735 | 2.65695 | 4.035222 | 3.390548 | 3.63569 | 2.65855 | 2.455798 | 3.919234 | 2.375606 |
| Q4VBD2 | Q4VBD2 | TAPT1_M( | Tapt1 | Transmembrane anterior posterior transformation protein 1 | | | | 0.432544 | 0.224606 | 1.149176 | 0.695283 | 0.375423 | 1.874518 | 0.047921 | -0.0791 | -0.14856 | -0.66063 | -0.26973 | -1.02241 | -0.65762 | -1.42705 | -0.40646 | -0.68988 | -0.68969 | -1.29816 | -1.13271 | -0.68571 | -0.98959 |
| Q9DB77 | Q9DB77 | QCR2_MO | Uqcrc2 | Cytochrome b-c1 complex subunit 2, mitochondrial | + | | | 2.499853 | -1.1049 | -17.7376 | 0.21207 | -0.37255 | -0.59235 | 0.032902 | -0.06777 | -0.10346 | 1.343845 | 1.422916 | 1.788287 | 0.535466 | 1.454827 | 2.620312 | 2.536416 | 2.440142 | 1.478204 | 1.590644 | 2.404495 | 1.806182 |
| Q9D0R2 | Q9D0R2 | SYTC_MOI | Tars1 | Threonine--tRNA ligase 1, cytoplasmic | | | | 0.161841 | 0.621438 | 0.462926 | 0.281098 | -0.18389 | -0.76652 | 0.221983 | 0.21474 | 0.617553 | -1.66428 | -1.67706 | -0.82005 | -1.29969 | -0.59046 | -1.21699 | -3.63451 | -0.94971 | -0.86976 | -0.88221 | -0.96754 | -1.26939 |
| Q8BLY2 | Q8BLY2 | SYTC2_MC | Tars3 | Threonine--tRNA ligase 2, cytoplasmic | | | | 0.478706 | -1.29541 | -1.26907 | 0.535681 | -0.36423 | -1.42067 | 0.440833 | 0.223091 | 1.170537 | -0.96031 | -0.87515 | -0.31649 | -0.63691 | -0.26384 | -0.61578 | -0.64219 | 1.397556 | -0.31262 | 0.08769 | -0.58969 | -0.73611 |
| Q3UKC1 | Q3UKC1 | TAXB1_MC | Tax1bp1 | Tax1-binding protein 1 homolog | | | | 0.208972 | 0.706517 | 0.584461 | 0.387244 | 0.3799 | 1.03349 | 0.180021 | -0.26637 | -0.51017 | -1.70264 | -3.21198 | -0.67261 | -0.01309 | -2.34634 | -1.5971 | -2.2195 | -4.10816 | -0.56032 | -0.88518 | -2.06904 | -1.34165 |
| Q8BHL3 | Q8BHL3 | TB10B_MC | Tbc1d10b | TBC1 domain family member 10B | | | | 0.74324 | 0.584692 | 2.021433 | 0.578153 | -0.3688 | -1.53683 | 0.445965 | -0.13809 | -1.1838 | 0.609006 | 0.531417 | 1.293188 | 1.630916 | 1.876346 | 1.839798 | 0.272153 | -0.30111 | 1.660345 | 2.001361 | 1.880953 | 2.111377 |
| Q8BYH7 | Q8BYH7 | TBC17_MC | Tbc1d17 | TBC1 domain family member 17 | | + | | 0.254665 | -0.72603 | -0.70012 | 1.286428 | -0.94403 | -4.22516 | 0.015155 | -0.014 | -0.04853 | -3.35135 | -2.77768 | -1.16023 | -1.60329 | -1.14521 | -0.77209 | -1.34193 | -3.33504 | -0.4668 | -0.40866 | -1.1648 | -0.7245 |
| Q3UUG6 | Q3UUG6 | TBC24_MC | Tbc1d24 | TBC1 domain family member 24 | | | | 0.534666 | -0.24795 | -1.41793 | 0.85341 | -0.63122 | -2.38179 | 0.698612 | 0.363949 | 1.884539 | -1.26199 | -0.98811 | -0.8113 | -1.06692 | 0.388916 | 0.343178 | -0.76836 | -0.98585 | -0.07572 | -0.54005 | 0.193863 | -0.18967 |
| Q8BYJ6 | Q8BYJ6 | TBCD4_M( | Tbc1d4 | TBC1 domain family member 4 | | | | 0.821072 | 1.429291 | 2.272465 | 0.92558 | -1.25185 | -2.63756 | 0.194893 | 0.317628 | 0.548459 | -1.6669 | -1.29382 | -3.52318 | -4.38502 | -3.26708 | -2.55716 | -3.51031 | -2.30899 | -2.50332 | -2.90119 | -3.68734 | -2.77215 |
| Q9D1E6 | Q9D1E6 | TBCB_MOI | Tbcb | Tubulin-folding cofactor B | | | | 0.541818 | -0.23736 | -1.43727 | 0.865745 | -0.55973 | -2.42433 | 0.220679 | -0.17081 | -0.61424 | -0.43341 | -0.45643 | -1.11341 | -1.12192 | -1.08203 | -1.01335 | -0.37231 | -0.04281 | -0.32709 | -0.78877 | -0.60093 | -1.15283 |
| Q3TC72 | Q3TC72 | FAHD2_M( | Fahd2 | Fumarylacetoacetate hydrolase domain-containing protein 2A | + | | | 2.778309 | -1.13375 | -24.4687 | 0.740818 | -1.08064 | -2.01388 | 0.055171 | -0.20028 | -0.16993 | -0.64442 | -0.73166 | -0.85439 | -1.90553 | -1.26339 | 0.164781 | 0.461344 | 0.430079 | -0.40756 | -0.19108 | 0.588676 | -1.28672 |
| Q8BYA0 | Q8BYA0 | TBCD_MO | Tbcd | Tubulin-specific chaperone D | | | | 0.30451 | -1.1863 | -0.82522 | 0.470106 | 1.164789 | 1.246551 | 0.046874 | -0.03825 | -0.14545 | -3.95701 | -1.19991 | -1.84221 | -1.63245 | -1.27562 | -1.78781 | -0.98454 | -1.79978 | -3.83063 | -1.97362 | -1.43384 | -1.55309 |
| Q8CIV8 | Q8CIV8 | TBCE_MOI | Tbce | Tubulin-specific chaperone E | | | | 0.344602 | -1.14738 | -0.92584 | 0.309964 | 0.487557 | 0.838894 | 0.023229 | -0.03858 | -0.07375 | -4.02608 | -1.5959 | -0.94088 | -0.9546 | -0.9524 | -1.44403 | -1.41993 | -1.9073 | -2.01645 | -0.85415 | -1.62135 | -0.69791 |
| Q8QZT1 | Q8QZT1 | THIL_MOU | Acat1 | Acetyl-CoA acetyltransferase, mitochondrial | + | | | 1.681262 | -1.16561 | -6.81965 | 0.371165 | -0.75417 | -0.99279 | 0.129254 | -0.41511 | -0.37674 | 0.162529 | 0.413188 | 1.467334 | -0.03162 | 0.418461 | 2.131162 | 1.569688 | 1.337256 | 1.348116 | 1.59594 | 2.383285 | 0.996556 |
| Q8BHJ5;Q | Q8BHJ5;Q | TBL1R_MC | Tbl1x;Tbl1xr1 | F-box-like/WD repeat-containing protein TBL1XR1 | | | | NaN | 0 | NaN | 0.646172 | -0.77578 | -1.72953 | 0.713933 | -1.50244 | -1.93099 | -3.00763 | -4.27662 | -1.70565 | -1.57239 | -3.60677 | -3.38818 | -3.8112 | -3.36815 | -0.41966 | -1.30681 | -2.76539 | -1.22468 |
| Q64336 | Q64336 | TBR1_MOI | Tbr1 | T-box brain protein 1 | | | | NaN | 0 | NaN | 0.189945 | 0.387953 | 0.535751 | 0.276766 | -0.37756 | -0.75565 | -3.01475 | -2.69818 | -1.83482 | -2.58956 | -3.65514 | -4.18821 | -3.52257 | -3.32473 | -1.98212 | -3.21817 | -3.96674 | -3.12149 |
| Q8BNE1 | Q8BNE1 | TCAF1_MC | Tcaf1 | TRPM8 channel-associated factor 1 | | | | NaN | 0 | NaN | 0.259248 | -0.40593 | -0.71165 | 0.003691 | 0.00535 | 0.011968 | -3.46846 | -2.8544 | -2.80073 | -1.69614 | -2.1729 | -2.29604 | -3.40585 | -2.29619 | -1.69991 | -1.9851 | -2.68262 | -1.79702 |
| Q8CCT4 | Q8R0A5;Q | TCAL5_MC | Tceal5 | Transcription elongation factor A protein-like 5 | | | | 0.462776 | 1.093926 | 1.22743 | 0.23751 | 0.140078 | 0.656865 | 0.783118 | -0.3339 | -2.148 | -1.05799 | -0.86219 | -0.10481 | -0.52723 | -0.42879 | -0.11977 | -2.93986 | -1.16818 | -0.42664 | -0.48554 | 0.0767 | 0.042551 |
| O08784 | O08784 | TCOF_MO | Tcof1 | Treacle protein | | | | 0.555022 | -0.13249 | -1.4732 | 0.694053 | -0.21403 | -1.87082 | 0.508044 | 0.183462 | 1.346572 | -0.37646 | -0.23249 | -0.37712 | -0.16091 | -0.227 | -0.24294 | -0.11808 | -0.2259 | -0.01756 | -0.09241 | -0.28242 | -0.55444 |
| Q9JLZ3 | Q9JLZ3 | AUHM_MC | Auh | Methylglutaconyl-CoA hydratase, mitochondrial | + | | | 1.434982 | -1.176 | -5.07299 | 0.473023 | -0.81203 | -1.25418 | 0.080096 | -0.12487 | -0.24163 | 1.194692 | 1.657213 | 1.198149 | -0.0958 | 1.01931 | 1.942994 | 2.585913 | 2.617994 | 1.338203 | 1.3882 | 1.837838 | 1.374197 |
| Q8BTG3 | Q8BTG3 | T11L1_MO | Tcp11l1 | T-complex protein 11-like protein 1 | + | | | 1.168314 | 1.297229 | 3.64024 | 0.127553 | 0.252877 | 0.372173 | 0.300987 | -0.46043 | -0.81639 | -0.28122 | -0.56179 | -0.50101 | 0.125933 | -0.70557 | -0.7912 | -1.39115 | -2.04632 | -1.04325 | 0.162415 | -0.85031 | 0.274398 |
| Q80VP0 | Q80VP0 | TCPR1_MC | Tecpr1 | Tectonin beta-propeller repeat-containing protein 1 | + | | | 1.070499 | 0.188339 | 3.206975 | 0.365575 | 0.096713 | 0.978668 | 0.064467 | 0.023779 | 0.196972 | -0.72924 | -0.84637 | -0.37799 | -0.57469 | 0.060169 | -0.07923 | -0.98054 | -0.97175 | -0.58266 | -0.56345 | 0.065257 | -0.13188 |
| Q3UH45 | Q3UH45 | Q3UH45_I | Tecpr2 | Tectonin beta-propeller repeat containing 2 | | | | 0.77707 | 1.016024 | 2.128533 | 0.529519 | -0.43183 | -1.40405 | 0.186808 | -0.21671 | -0.52768 | -2.29176 | -2.83173 | -0.64827 | -0.04482 | -0.60543 | -1.04842 | -3.18412 | -3.97141 | 0.025657 | 0.14492 | -0.95606 | -0.26437 |
| Q9CY27 | Q9CY27 | TECR_MOI | Tecr | Very-long-chain enoyl-CoA reductase | + | | | 1.241065 | 0.686939 | 3.991922 | 0.682613 | 0.303525 | 1.836607 | 0.016809 | -0.02153 | -0.05373 | 5.302781 | 5.318485 | 4.176313 | 4.180658 | 4.494694 | 4.111736 | 4.795597 | 4.451791 | 4.04021 | 3.709711 | 3.972661 | 4.676835 |
| Q9WTS4 | Q9WTS4 | TEN1_MOI | Tenm1 | Teneurin-1 | | | | 0.282806 | 0.391021 | 0.770804 | 0.331696 | 0.045945 | 0.89341 | 0.350022 | -0.08425 | -0.93947 | -2.18371 | -3.19756 | -1.03538 | -0.96779 | -1.28112 | -1.45673 | -3.06241 | -3.10091 | -1.00876 | -1.0863 | -1.26646 | -1.30289 |
| Q9WTS5 | Q9WTS5 | TEN2_MOI | Tenm2 | Teneurin-2 | | | | 0.147385 | 0.48745 | 0.424957 | 0.355342 | -0.27414 | -0.95287 | 0.452421 | -0.459 | -1.20052 | -3.7406 | -1.70779 | -1.84181 | -1.30949 | -1.11673 | -1.2252 | -3.74331 | -2.67998 | -1.41074 | -1.19227 | -1.09044 | -0.3335 |
| Q9WTS6 | Q9WTS6 | TEN3_MOI | Tenm3 | Teneurin-3 | | | | 0.735201 | -0.5914 | -1.99641 | 0.312497 | 0.368333 | 0.845243 | 0.110234 | -0.12906 | -0.32531 | -3.81427 | -3.52307 | -2.61641 | -2.5151 | -2.88241 | -2.17687 | -3.33525 | -2.81929 | -3.3669 | -2.50127 | -2.58212 | -2.21904 |
| Q3UHK6 | Q3UHK6 | TEN4_MOI | Tenm4 | Teneurin-4 | | | | 0.069866 | 0.029043 | 0.212511 | 0.032178 | 0.017709 | 0.101253 | 0.34439 | 0.17529 | 0.925305 | -0.94326 | -0.90501 | 0.005142 | 0.336554 | 0.055682 | -0.04524 | -0.81785 | -1.0885 | 0.209108 | 0.097168 | -0.35267 | 0.012525 |
| P19536 | P19536 | COX5B_M( | Cox5b | Cytochrome c oxidase subunit 5B, mitochondrial | + | | | 2.182541 | -1.18292 | -12.2779 | 0.858419 | -1.07382 | -2.39901 | 0.014677 | -0.06387 | -0.04702 | 4.662649 | 4.608963 | 4.092057 | 3.367459 | 3.729264 | 6.119688 | 5.726196 | 5.911258 | 5.066441 | 4.540721 | 5.633862 | 4.342837 |
| P40630 | P40630 | TFAM_MO | Tfam | Transcription factor A, mitochondrial | | | | NaN | 0 | NaN | 0.184996 | 0.295398 | 0.52301 | 0.395844 | 0.713066 | 1.055329 | -3.04887 | -3.71271 | -0.90215 | -2.03015 | -3.16015 | -1.83301 | -3.7226 | -3.37927 | -1.7314 | -1.7917 | -3.33701 | -3.08229 |
| Q9Z1A1 | Q9Z1A1 | Q9Z1A1_M | Tfg | TFG protein | | | | 0.712547 | -0.33534 | -1.92677 | 0.402671 | 0.500784 | 1.072704 | 0.278625 | -0.20413 | -0.76032 | 0.02394 | 0.371828 | 1.507045 | 0.919083 | -0.14956 | -0.08182 | 0.527393 | 0.539051 | 0.349627 | 1.074933 | 0.354789 | -0.17789 |
| Q62351 | Q62351 | TFR1_MOI | Tfrc | Transferrin receptor protein 1 | | | | 0.29457 | -0.2166 | -0.8003 | 0.579261 | -0.31196 | -1.5399 | 0.494112 | 0.215416 | 1.309628 | -0.19453 | -0.44491 | -0.32711 | -0.43155 | 0.167002 | 0.063311 | -0.34308 | 0.136838 | 0.12837 | -0.26311 | 0.055842 | -0.25636 |
| Q62219 | Q62219 | TGFI1_MO | Tgfb1i1 | Transforming growth factor beta-1-induced transcript 1 protein | | | | 0.660684 | 0.67847 | 1.771833 | 0.078633 | -0.14798 | -0.23749 | 0.305683 | -0.7489 | -0.82816 | -2.5327 | -2.13013 | -0.00137 | -1.24148 | -1.90736 | -0.11477 | -3.33563 | -2.68414 | -0.53484 | -0.41205 | -0.14217 | -0.38216 |
| Q9JLF6 | Q9JLF6 | TGM1_MO | Tgm1 | Protein-glutamine gamma-glutamyltransferase K | + | | | 1.137982 | 1.229623 | 3.501249 | 0.057652 | 0.096298 | 0.177183 | 0.019565 | 0.019087 | 0.062356 | 0.795422 | 0.146718 | -0.92374 | -0.77724 | -1.03876 | -0.46276 | -0.62389 | -0.89322 | -1.48532 | -0.40825 | -0.87356 | -0.66614 |
| Q08189 | Q08189 | TGM3_MO | Tgm3 | Protein-glutamine gamma-glutamyltransferase E | | | | NaN | 0 | NaN | 0.290161 | 0.896391 | 0.78925 | NaN | 0 | NaN | -3.43549 | -3.24571 | -3.75456 | -1.48315 | -3.25086 | -4.0718 | -3.15186 | -3.27294 | -3.52536 | -3.50513 | -3.4544 | -3.75289 |
| Q923W1 | Q923W1 | TGS1_MOI | Tgs1 | Trimethylguanosine synthase | | | | NaN | 0 | NaN | 0.482965 | 0.350308 | 1.280251 | 0.515926 | -0.64972 | -1.36759 | -3.76097 | -3.28221 | -1.42894 | -1.47727 | -2.73436 | -1.90779 | -2.77874 | -2.95457 | -2.07597 | -1.53086 | -1.90566 | -1.43706 |
| Q3UUI3 | Q3UUI3 | THEM4_M( | Them4 | Acyl-coenzyme A thioesterase THEM4 | | | | 0.530167 | -1.0225 | -1.40579 | 0.22638 | -0.50573 | -0.62871 | 0.010486 | 0.048049 | 0.033747 | -3.9726 | -2.99653 | -2.09116 | -3.48456 | -3.38493 | -2.16521 | -3.00137 | -1.92275 | -2.6842 | -1.88007 | -1.53654 | -4.1097 |
| Q8BH55 | Q8BH55 | THNS1_M( | Thnsl1 | Threonine synthase-like 1 | | | | 0.998324 | -1.0642 | -2.9134 | 0.79339 | 0.490813 | 2.181289 | 0.12054 | 0.114212 | 0.35329 | -2.67054 | -3.36142 | -1.5567 | -1.75683 | -1.6094 | -1.38803 | -2.07052 | -1.83303 | -1.94604 | -2.34911 | -1.91667 | -1.30919 |
| Q8C1A5 | Q8C1A5 | THOP1_M( | Thop1 | Thimet oligopeptidase | | | | 0.013391 | 0.009375 | 0.04296 | 0.501319 | -0.34201 | -1.32871 | 0.084614 | 0.103643 | 0.254369 | -0.77086 | -0.84529 | -0.26739 | -0.77623 | -0.48673 | 0.154245 | -0.60241 | -1.03249 | -0.14075 | -0.21886 | -0.01828 | -0.52149 |
| P62843 | P62843 | RS15_MOI | Rps15 | Small ribosomal subunit protein uS19 | + | | | 1.847083 | -1.19129 | -8.29604 | 0.909371 | -1.51482 | -2.57864 | 0.069692 | -0.22462 | -0.21201 | -1.84877 | -2.03575 | -1.42199 | -2.24471 | -2.00173 | -3.31906 | -0.85997 | -0.64197 | 0.100847 | -0.73791 | -0.3256 | -1.60596 |
| O08749 | O08749 | DLDH_MO | Dld | Dihydrolipoyl dehydrogenase, mitochondrial | + | | | 1.912053 | -1.24134 | -8.95384 | 0.636068 | -0.94949 | -1.70034 | 0.029368 | -0.0771 | -0.09267 | 0.675807 | 0.903708 | 1.182267 | 0.070556 | 1.016393 | 2.49084 | 2.110067 | 1.952134 | 1.629281 | 1.522525 | 2.216395 | 1.445044 |
| P60487 | P60487 | PLPP_MOI | Pdxp | Chronophin | + | | | 1.526254 | -1.2545 | -5.66577 | 0.288378 | -0.84902 | -0.78478 | 0.093194 | 0.187559 | 0.278361 | -1.2513 | -1.04419 | 0.642961 | -1.42815 | 0.208795 | 0.69126 | -0.08895 | 0.302464 | 0.769554 | 0.143301 | 0.891604 | -0.36667 |
| P70318 | P70318;P! | TIAR_MOU | Tial1 | Nucleolysin TIAR | | | | 0.177944 | -0.10451 | -0.5048 | 0.077381 | 0.067636 | 0.233943 | 0.380993 | 0.435829 | 1.017647 | 2.210879 | 2.563613 | 1.267664 | 1.842127 | 2.169309 | 2.751155 | 2.600176 | 2.383331 | 1.520183 | 1.454337 | 2.338696 | 1.710111 |
| Q60610 | Q60610 | TIAM1_MC | Tiam1 | Rho guanine nucleotide exchange factor TIAM1 | | | | 0.025439 | -0.10644 | -0.08059 | 0.979494 | -0.64626 | -2.84015 | 0.436204 | -0.54102 | -1.1586 | -3.51419 | -1.17442 | 0.153603 | 0.607566 | -0.39853 | 0.513121 | -1.62466 | -2.85106 | 1.04285 | 1.010843 | 0.699673 | 0.496956 |
| Q6ZPF3 | Q6ZPF3 | TIAM2_MC | Tiam2 | Rho guanine nucleotide exchange factor TIAM2 | | | | NaN | 0 | NaN | 0.618182 | 0.707937 | 1.649167 | 0.046364 | 0.226577 | 0.143938 | -2.87417 | -3.51987 | -1.10253 | -1.27659 | -1.43924 | -3.20258 | -3.48065 | -3.09858 | -2.31785 | -1.47714 | -3.85154 | -1.24343 |
| O08709 | O08709 | PRDX6_M( | Prdx6 | Peroxiredoxin-6 | + | | | 1.664434 | -1.28871 | -6.68456 | 0.569782 | -0.80898 | -1.5137 | 0.000103 | 0.000317 | 0.000335 | 1.716814 | 2.098013 | 2.641909 | 1.605034 | 2.08952 | 3.597004 | 3.225097 | 3.167153 | 2.802656 | 3.062245 | 3.413034 | 2.272856 |
| Q9CQ85 | Q9CQ85 | TIM22_MO | Timm22 | Mitochondrial import inner membrane translocase subunit Tim22 | | | | 0.700426 | 1.028799 | 1.890012 | 0.752907 | 1.275027 | 2.051735 | 0.099902 | -0.11364 | -0.29695 | -1.20161 | -1.85646 | -1.24937 | -2.46495 | -1.34953 | -1.85964 | -2.12298 | -2.99269 | -3.00266 | -3.26171 | -1.20564 | -1.77626 |
| Q9WTQ8 | Q9WTQ8 | TIM23_MO | Timm23 | Mitochondrial import inner membrane translocase subunit Tim23 | | | | 0.523088 | 0.463888 | 1.386769 | 0.151688 | -0.11945 | -0.4363 | 0.319947 | 0.234104 | 0.863928 | -1.03986 | -0.37832 | -0.66987 | -1.05963 | 0.080795 | -0.40552 | -1.22284 | -1.12312 | -0.55301 | -0.93759 | -0.51606 | -0.27688 |
| Q8BGX2 | Q8BGX2 | TIM29_MO | Timm29 | Mitochondrial import inner membrane translocase subunit Tim29 | | | | 0.176192 | 0.1525 | 0.500261 | 0.040409 | 0.042561 | 0.126148 | 0.481814 | 0.408849 | 1.277226 | -0.39236 | -0.4371 | -0.71686 | -1.23794 | 0.234072 | -0.30082 | -0.26321 | -0.87125 | -0.8056 | -1.23432 | -0.26632 | -0.61812 |
| O35857 | O35857 | TIM44_MO | Timm44 | Mitochondrial import inner membrane translocase subunit TIM44 | | | | NaN | 0 | NaN | 0.34484 | -0.58012 | -0.92644 | 0.750745 | 1.08909 | 2.044938 | -3.22461 | -3.43345 | -4.46013 | -3.54258 | -2.1125 | -3.17584 | -4.17952 | -3.75651 | -2.99505 | -3.84742 | -3.76433 | -3.70218 |
| Q9D880 | Q9D880 | TIM50_MO | Timm50 | Mitochondrial import inner membrane translocase subunit TIM50 | | | | 0.052666 | 0.031754 | 0.162571 | 0.64596 | 0.843752 | 1.72892 | 0.503286 | 0.560663 | 1.333927 | 0.232542 | -0.15791 | 0.560205 | 0.943382 | 0.879775 | 0.04811 | -0.00055 | 0.011676 | -0.5408 | 0.356884 | -0.1579 | -0.03554 |
| Q9D172 | Q9D172 | GAL3A_M( | Gatd3 | Glutamine amidotransferase-like class 1 domain-containing protein 3, mitochondrial | + | | | 1.462294 | -1.29748 | -5.24422 | 0.82595 | -0.31586 | -2.28876 | 0.830314 | -0.23746 | -2.30339 | 0.923671 | 1.377441 | 1.249466 | 1.144807 | 1.595189 | 1.762784 | 2.546712 | 2.349369 | 1.640694 | 1.385297 | 1.856398 | 1.976487 |
| P11679 | P11679 | K2C8_MOI | Krt8 | Keratin, type II cytoskeletal 8 | + | + | | 1.62348 | -1.35059 | -6.36619 | 1.027166 | -1.7467 | -3.02822 | 0.066718 | 0.094175 | 0.203466 | -2.10535 | -2.06533 | -2.12251 | -1.73681 | -2.0661 | -1.41153 | -0.94595 | -0.52354 | 0.358623 | -0.72753 | -2.16028 | -1.5057 |
| Q9WV98 | Q9WV98 | TIM9_MOL | Timm9 | Mitochondrial import inner membrane translocase subunit Tim9 | | | | 0.373284 | 1.381502 | 0.99814 | 0.253377 | 0.422247 | 0.696875 | 0.373088 | 0.250849 | 0.997646 | -1.06572 | -0.41698 | -0.06769 | -1.24753 | -0.42475 | -0.61188 | -0.77732 | -3.46838 | -0.94156 | -1.21816 | -0.53578 | -1.00255 |
| Q8BH58 | Q8BH58 | TIPRL_MO | Tiprl | TIP41-like protein | | | | 0.081034 | -0.09986 | -0.24428 | 0.34223 | -0.1266 | -0.91987 | 0.025784 | -0.03078 | -0.08165 | -0.82387 | -0.03922 | -0.60388 | -0.37953 | -1.02049 | -0.26649 | -0.21679 | -0.44657 | -0.28536 | -0.44484 | -0.6135 | -0.61191 |
| P39447 | P39447 | ZO1_MOU | Tjp1 | Tight junction protein ZO-1 | | | | 0.003648 | 0.015775 | 0.01183 | 0.034215 | 0.046092 | 0.107449 | 0.265331 | 0.097962 | 0.72694 | -2.99853 | -2.05924 | -1.83069 | -1.37888 | -1.32034 | -1.53512 | -1.29672 | -3.7926 | -1.28622 | -2.01554 | -1.44428 | -1.6071 |
| Q7M729 | Q7M729 | SCN4B_M( | Scn4b | Sodium channel subunit beta-4 | + | + | | 2.285105 | -1.38322 | -13.8311 | 2.140425 | -0.30049 | -11.6908 | 0.32803 | -0.31644 | -0.88421 | -0.3888 | -0.51821 | -2.93409 | -2.98547 | -3.49122 | -2.92216 | 1.005972 | 0.853459 | -2.6584 | -2.66018 | -2.67319 | -3.10732 |
| Q7TNV1 | Q7TNV1 | TLC3B_MC | Tlcd3b | Ceramide synthase | | | | 0.671167 | 1.972897 | 1.802667 | 0.013156 | 0.041329 | 0.042217 | 0.19871 | 0.75955 | 0.558242 | 0.030926 | -0.00346 | -1.72126 | -3.67654 | -1.24294 | -3.60603 | -3.05346 | -0.86487 | -2.68941 | -2.79104 | -3.85872 | -2.50934 |
| Q71LX4 | Q71LX4 | TLN2_MOL | Tln2 | Talin-2 | | | | 0.415292 | -0.37816 | -1.10492 | 0.333528 | -0.1361 | -0.89801 | 0.221742 | 0.27031 | 0.616944 | -1.30087 | -1.25931 | -0.78033 | -1.02053 | -0.1973 | -1.04586 | -1.24355 | -0.56031 | -0.67189 | -0.85677 | -1.00125 | -0.78254 |
| P58021 | P58021 | TM9S2_MC | Tm9sf2 | Transmembrane 9 superfamily member 2 | | | | 0.028549 | 0.10422 | 0.090158 | 0.42956 | -0.8134 | -1.1415 | 0.316663 | 0.680506 | 0.855691 | -2.85229 | -2.00094 | -2.35976 | -3.55983 | -2.08356 | -0.62662 | -3.60557 | -1.4561 | -2.53074 | -1.76205 | -1.71656 | -2.35463 |
| Q8K003 | Q8K003 | TMA7_MO | Tma7 | Translation machinery-associated protein 7 | | + | | 0.288581 | 0.634699 | 0.785288 | 1.257077 | 1.050052 | 4.072981 | 0.435386 | 0.594784 | 1.156493 | -1.16641 | 0.41614 | -0.56379 | -1.0794 | -1.13063 | -0.48551 | -0.84513 | -1.17454 | -1.87018 | -1.87311 | -1.80343 | -1.00228 |
| Q8C428 | Q8C428 | TMC7_MO | Tmc7 | Transmembrane channel-like protein 7 | | | | 0.195173 | 0.328397 | 0.549175 | 0.243839 | 0.354253 | 0.67284 | 0.046392 | 0.034281 | 0.14402 | 4.578033 | 4.366555 | 2.220563 | 2.91075 | 2.207748 | 2.564573 | 4.732456 | 3.555337 | 2.609043 | 1.813766 | 2.194319 | 2.509441 |
| Q69ZZ6 | Q69ZZ6 | TMCC1_M | Tmcc1 | Transmembrane and coiled-coil domains protein 1 | | | | NaN | 0 | NaN | 0.058694 | 0.063666 | 0.180223 | 0.426162 | -1.04581 | -1.13277 | -3.42696 | -3.55376 | -0.64408 | -1.30597 | -2.58893 | -1.01693 | -3.16607 | -2.78201 | -1.16226 | -0.91512 | -0.95627 | -0.82797 |
| Q8R310 | Q8R310 | TMCC3_M | Tmcc3 | Transmembrane and coiled-coil domains protein 3 | | | | NaN | 0 | NaN | 0.361432 | 0.19717 | 0.968218 | NaN | 0 | NaN | -3.8066 | -3.38003 | -2.92802 | -3.29097 | -3.56196 | -2.64858 | -3.78547 | -3.35791 | -3.21427 | -3.39906 | -2.69249 | -3.78258 |
| Q9D1D4 | Q9D1D4 | TMEDA_M | Tmed10 | Transmembrane emp24 domain-containing protein 10 | | | | 0.041644 | -0.11378 | -0.12985 | 0.105874 | -0.33509 | -0.31338 | 0.432817 | 0.482523 | 1.149878 | -3.37107 | -2.30328 | -1.50147 | -3.5121 | -2.28511 | -1.54563 | -3.41821 | -2.02856 | -2.53598 | -1.80743 | -2.19944 | -2.59634 |
| P16125 | P06151;P( | LDHB_MO | Ldhb | L-lactate dehydrogenase B chain | + | | | 1.82545 | -1.40463 | -8.08749 | 0.636011 | -0.70125 | -1.70018 | 0.127918 | -0.18952 | -0.37315 | 3.494625 | 3.826632 | 3.929656 | 3.147372 | 3.530338 | 4.353766 | 5.116314 | 5.014194 | 4.370646 | 4.108885 | 4.428941 | 3.834195 |
| Q9QYM9 | Q9QYM9 | TEFF2_MC | Tmeff2 | Tomoregulin-2 | + | | | 1.055382 | 1.052104 | 3.143742 | 0.311363 | 0.100792 | 0.842401 | 0.169911 | 0.19999 | 0.483961 | 0.443268 | 0.037752 | 1.666718 | 1.850198 | 1.52312 | 1.137106 | -0.54534 | -1.07785 | 1.580857 | 1.734475 | 0.764729 | 1.495517 |
| Q8BK08 | Q8BK08 | TMM11_M | Tmem11 | Transmembrane protein 11, mitochondrial | | | | 0.70091 | -2.59012 | -1.89147 | 0.26373 | -0.11644 | -0.72292 | 0.388311 | 0.313521 | 1.036196 | -1.03683 | -3.72706 | -0.52849 | -0.80301 | -0.12366 | 0.142683 | 0.464728 | -0.04839 | -0.46504 | -0.63359 | -0.03232 | -0.5757 |
| Q9D8Y1 | Q9D8Y1 | T126A_MC | Tmem126a | Transmembrane protein 126A | + | | | 1.287133 | -0.38532 | -4.22888 | 0.49332 | -0.59216 | -1.30753 | 0.09421 | 0.145955 | 0.281186 | -0.79384 | -0.8023 | -1.49216 | -1.99324 | -1.41408 | -0.55388 | -0.50377 | -0.32173 | -1.52781 | -0.77327 | -0.1156 | -1.14428 |
| Q9D1R1 | Q9D1R1 | T126B_MC | Tmem126b | Complex I assembly factor TMEM126B, mitochondrial | | | | 0.762588 | -0.89455 | -2.08232 | 0.630872 | 0.48121 | 1.685409 | 0.205519 | 0.50245 | 0.575652 | -0.62748 | -1.38931 | -0.77319 | -0.2218 | -0.0657 | -1.1935 | 0.084779 | -0.31248 | -0.90446 | -1.05294 | -1.79828 | -0.46582 |
| F7BAB2 | F7BAB2 | F7BAB2_M | Tmem132b | Transmembrane protein 132B | | | | NaN | 0 | NaN | 0.458385 | 0.43775 | 1.216002 | 0.419485 | 0.39825 | 1.115652 | -4.03302 | -3.29674 | -0.09825 | -0.41049 | -0.18803 | -0.87077 | -3.42825 | -3.42574 | -1.0165 | -0.36774 | -1.03202 | -0.82328 |
| Q9CQG6 | Q9CQG6 | TM147_MC | Tmem147 | BOS complex subunit TMEM147 | + | | | 1.040501 | 1.833834 | 3.08242 | 0.497625 | 1.800709 | 1.31892 | 0.001713 | 0.003006 | 0.005568 | 0.293757 | -0.13009 | -0.62805 | -0.68175 | -0.53098 | -1.5047 | -2.30791 | -1.19609 | -1.09058 | -3.82064 | -1.25404 | -0.78765 |
| Q8C817 | Q8C817 | T200A_MC | Tmem200a | Transmembrane protein 200A | | | | 0.002683 | 0.013545 | 0.00871 | 0.237465 | -0.26593 | -0.65675 | 0.353475 | -0.40784 | -0.94817 | -1.39016 | -3.14713 | 0.28848 | 0.981889 | 0.328125 | 1.146945 | -0.99908 | -3.5653 | 0.691929 | 1.110308 | 1.277277 | 1.013469 |
| J3QK46 | J3QK46 | J3QK46_M | Tmem200c | Transmembrane protein 200C | | | | 0.070586 | 0.068536 | 0.214575 | 0.127745 | -0.11057 | -0.37269 | 0.431053 | -0.92259 | -1.14534 | -2.96378 | -3.48911 | -2.15051 | -2.08089 | -2.55784 | -3.86922 | -3.11325 | -3.47671 | -2.29976 | -1.7105 | -2.75884 | -1.82304 |
| Q8BM55 | Q8BM55 | TM214_MC | Tmem214 | Transmembrane protein 214 | | | | 0.583095 | 1.485267 | 1.550541 | 0.379655 | 0.58131 | 1.014259 | 0.119966 | -0.43239 | -0.35174 | -0.7823 | -1.15669 | -1.30101 | -1.60514 | -1.8147 | -3.62748 | -1.51533 | -3.3942 | -1.48179 | -2.58698 | -3.11914 | -1.45826 |
| Q9DAM7 | Q9DAM7 | TM263_MC | Tmem263 | Transmembrane protein 263 | | | | 0.455312 | 0.229584 | 1.20802 | 0.639577 | 0.58613 | 1.710455 | 0.471676 | 0.405399 | 1.250656 | 1.230459 | 1.284276 | 2.142137 | 2.822745 | 1.991308 | 2.272223 | 1.215919 | 0.839649 | 1.85607 | 1.936552 | 1.43423 | 2.018504 |
| Q8BWF0 | Q8BWF0 | SSDH_MO | Aldh5a1 | Succinate-semialdehyde dehydrogenase, mitochondrial | + | | | 1.90514 | -1.40831 | -8.88153 | 0.687813 | -1.00707 | -1.85212 | 0.079684 | -0.23503 | -0.24047 | 1.287943 | 1.605072 | 0.944539 | -0.13135 | 0.823847 | 2.505589 | 2.855388 | 2.854239 | 1.492822 | 1.334506 | 2.39797 | 1.401529 |
| Q4VAE3 | Q4VAE3 | TMM65_M | Tmem65 | Transmembrane protein 65 | | + | | NaN | 0 | NaN | 2.139359 | 1.579525 | 11.67633 | 0.449784 | 0.662381 | 1.193683 | -3.76203 | -3.40904 | -1.67358 | -1.5777 | -2.30773 | -2.19163 | -3.42089 | -3.18034 | -3.07867 | -3.33166 | -3.46392 | -2.3602 |
| P16460 | P16460 | ASSY_MOI | Ass1 | Argininosuccinate synthase | + | | | 1.479052 | -1.41765 | -5.35183 | 0.713661 | -0.83313 | -1.93016 | 0.192081 | -0.48631 | -0.54124 | -0.14072 | -0.67025 | 0.27963 | -0.47421 | -0.37268 | 1.2966 | 1.020304 | 1.004025 | 0.94618 | 0.525504 | 1.280985 | 0.615553 |
| Q9JKK7 | Q9JKK7 | TMOD2_M | Tmod2 | Tropomodulin-2 | | | | 0.836527 | -0.44783 | -2.32433 | 0.146917 | 0.027472 | 0.423722 | 0.21914 | -0.13462 | -0.61034 | -0.27884 | -0.40143 | 0.443047 | 0.52337 | 0.392999 | 0.38443 | -0.07496 | 0.290358 | 0.506634 | 0.404839 | 0.743866 | 0.302808 |
| Q61033 | Q61033 | LAP2A_MC | Tmpo | Lamina-associated polypeptide 2, isoforms alpha/zeta | | | | 0.780856 | 0.951938 | 2.14071 | 0.093365 | 0.102143 | 0.278837 | 0.090411 | 0.157945 | 0.270607 | -0.62842 | -0.23816 | -1.20723 | -1.51415 | -1.64218 | -0.53978 | -0.98565 | -1.78482 | -1.79546 | -1.13021 | -1.44089 | -1.05696 |
| Q8VBT0 | Q8VBT0 | TMX1_MOI | Tmx1 | Thioredoxin-related transmembrane protein 1 | | | | 0.251757 | 0.040475 | 0.692796 | 0.18638 | -0.07399 | -0.52658 | 0.274395 | 0.16213 | 0.7497 | -1.21598 | -0.10623 | -0.36872 | -0.34271 | 0.295955 | 0.066419 | -0.22163 | -0.18154 | -0.42164 | -0.14181 | 0.202351 | -0.16424 |
| Q9D710 | Q9D710 | TMX2_MOI | Tmx2 | Thioredoxin-related transmembrane protein 2 | | | | 0.534476 | -1.74017 | -1.41741 | 0.334206 | -0.41375 | -0.89971 | 0.101555 | 0.354252 | 0.301506 | -4.14706 | -1.76187 | -3.28283 | -3.37266 | -2.50964 | -2.46834 | -1.50584 | -0.92274 | -3.37167 | -2.45632 | -1.66849 | -4.018 |
| Q8BXZ1 | Q8BXZ1 | TMX3_MOI | Tmx3 | Protein disulfide-isomerase TMX3 | | + | | 0.49286 | 0.431531 | 1.30632 | 1.599393 | 0.310919 | 6.185589 | 0.594098 | 0.665261 | 1.581228 | 0.361789 | 0.216641 | 0.592792 | 0.67679 | 0.906628 | 0.076459 | 0.179955 | -0.46459 | 0.351489 | 0.296257 | -0.24238 | -0.10506 |
| Q8C0L0 | Q8C0L0 | TMX4_MOI | Tmx4 | Thioredoxin-related transmembrane protein 4 | | | | 0.274864 | -0.43259 | -0.75088 | 0.511143 | -0.39077 | -1.35483 | 0.213057 | 0.223982 | 0.594867 | -1.01412 | 0.098881 | 0.21819 | -0.352 | 0.274794 | -0.17395 | -0.17405 | 0.123981 | 0.367602 | 0.280133 | 0.128808 | -0.47593 |
| Q80YX1 | Q80YX1 | TENA_MOI | Tnc | Tenascin | | | | NaN | 0 | NaN | 0.520867 | 1.399925 | 1.380812 | 0.198278 | 0.229182 | 0.557135 | -3.35302 | -3.45207 | -1.25061 | -1.3143 | -0.38735 | -1.20727 | -3.48228 | -3.11364 | -1.66904 | -3.69572 | -0.9926 | -1.06039 |
| Q6PFX9 | Q6PFX9 | TNKS1_MC | Tnks | Poly [ADP-ribose] polymerase tankyrase-1 | | | | 0.036842 | 0.051154 | 0.115405 | 0.646983 | 0.796413 | 1.731888 | 0.204213 | 0.149457 | 0.572314 | -1.55036 | -1.82085 | -0.83913 | -0.15357 | -1.46657 | -1.3735 | -1.31463 | -2.15888 | -1.5993 | -0.98622 | -1.82646 | -1.31253 |
| P58871 | P58871 | TB182_MC | Tnks1bp1 | 182 kDa tankyrase-1-binding protein | | | | 0.223956 | 0.751003 | 0.62256 | 0.482237 | -0.36858 | -1.27834 | 0.765265 | -0.67931 | -2.09082 | -3.46605 | -1.471 | 0.044989 | 0.258302 | -0.28486 | 0.250398 | -2.54119 | -3.89787 | 0.25235 | 0.78811 | 0.477864 | 0.846297 |
| Q8BFY9 | Q8BFY9 | TNPO1_M( | Tnpo1 | Transportin-1 | | | | 0.611486 | 1.393735 | 1.630169 | 0.23208 | -0.05279 | -0.64314 | 0.241433 | -0.26797 | -0.66677 | -1.59938 | -1.09792 | -2.58365 | -2.55589 | -2.12356 | -2.89844 | -3.55975 | -1.92501 | -2.43607 | -2.59788 | -2.34983 | -2.13623 |
| Q99LG2 | Q99LG2 | TNPO2_M( | Tnpo2 | Transportin-2 | + | | | 1.05942 | 0.7162 | 3.16054 | 0.014271 | 0.00617 | 0.045742 | 0.059937 | -0.02774 | -0.18384 | 1.47087 | 1.17437 | 1.289309 | 1.076497 | 1.270907 | 1.007232 | 0.435036 | 0.777805 | 1.259635 | 1.093831 | 1.093436 | 1.240179 |
| Q6P2B1 | Q6P2B1 | TNPO3_M( | Tnpo3 | Transportin-3 | | + | | 0.119122 | 0.162536 | 0.349455 | 1.014824 | 0.336967 | 2.978692 | 0.295544 | -0.17706 | -0.80274 | -0.03201 | -0.9413 | -0.21594 | -0.33768 | -0.33322 | -0.68771 | -0.55105 | -0.74733 | -0.51842 | -0.70913 | -0.46565 | -0.19517 |
| Q8BYI9 | Q8BYI9 | TENR_MOI | Tnr | Tenascin-R | | + | | 0.448107 | 0.510788 | 1.189341 | 1.477278 | 0.370412 | 5.340344 | 0.205227 | 0.286688 | 0.574905 | 1.774368 | 1.987298 | 1.945798 | 1.872468 | 2.342969 | 1.457692 | 1.786111 | 0.953979 | 1.479844 | 1.5976 | 1.383984 | 1.843301 |
| Q5SSZ5 | Q5SSZ5 | TENS3_MC | Tns3 | Tensin-3 | | | | 0.617407 | 0.97878 | 1.646963 | 0.249333 | -0.31928 | -0.68669 | 0.77076 | 0.477463 | 2.108329 | -0.26236 | -1.40866 | -2.23773 | -1.31585 | -1.67235 | -1.62396 | -1.65717 | -1.97141 | -1.51844 | -1.3966 | -2.35079 | -1.90045 |
| Q9QZ06 | Q9QZ06 | TOLIP_MO | Tollip | Toll-interacting protein | | | | 0.718746 | 0.283503 | 1.945705 | 0.121129 | 0.054514 | 0.35488 | 0.035003 | 0.025956 | 0.109839 | -0.59066 | -0.3556 | 0.211712 | 0.192275 | -0.05377 | -0.37486 | -0.84275 | -0.67051 | -0.00583 | 0.300785 | -0.06687 | -0.41367 |
| Q5SRX1 | Q5SRX1 | TM1L2_MC | Tom1l2 | TOM1-like protein 2 | | | | 0.036162 | -0.04568 | -0.11335 | 0.085639 | -0.05728 | -0.25725 | 0.376843 | -0.12773 | -1.00714 | 1.138971 | 0.407405 | 1.39759 | 1.347839 | 1.212674 | 0.978371 | 0.98799 | 0.649742 | 1.208734 | 1.65125 | 1.27182 | 1.174679 |
| Q9DCC8 | Q9DCC8 | TOM20_M( | Tomm20 | Mitochondrial import receptor subunit TOM20 homolog | | | | 0.405198 | -0.9545 | -1.07914 | 0.594775 | 0.884941 | 1.583121 | 0.471946 | -0.3853 | -1.25136 | -1.7209 | -3.46515 | -1.54277 | -2.26861 | -1.53526 | -1.19607 | -1.78595 | -1.49111 | -3.21578 | -2.36548 | -0.72338 | -1.23736 |
| Q9CPQ3 | Q9CPQ3 | TOM22_M( | Tomm22 | Mitochondrial import receptor subunit TOM22 homolog | | | | 0.284991 | -0.33909 | -0.77629 | 0.07532 | -0.2272 | -0.22809 | 0.523817 | 1.202838 | 1.388725 | -3.82386 | -3.40314 | -2.88912 | -4.64577 | -2.46935 | -2.32449 | -2.89159 | -3.65723 | -4.01015 | -3.07032 | -2.73665 | -4.46287 |
| Q9CYG7 | Q9CYG7 | TOM34_M( | Tomm34 | Mitochondrial import receptor subunit TOM34 | + | | | 1.168113 | -1.17883 | -3.63931 | 0.493818 | 1.407018 | 1.308851 | 0.22929 | 0.599537 | 0.636075 | -3.36437 | -2.78028 | -1.4133 | -3.54953 | -2.02035 | -2.59192 | -2.03361 | -1.75338 | -4.00989 | -3.76697 | -2.00749 | -3.80386 |
| Q9QYA2 | Q9QYA2 | TOM40_M( | Tomm40 | Mitochondrial import receptor subunit TOM40 homolog | | | + | 0.066133 | 0.091295 | 0.201781 | 0.113217 | -0.29106 | -0.33344 | 1.635728 | 0.858558 | 6.459889 | 0.298976 | -0.59807 | -0.52121 | -1.18157 | 0.059213 | 0.186267 | -0.1814 | -0.30028 | 0.247733 | -1.36839 | -0.61908 | -0.85256 |
| Q9CZR3 | Q9CZR3 | TM40L_MC | Tomm40l | Mitochondrial import receptor subunit TOM40B | | | | 0.726679 | 0.360441 | 1.970069 | 0.368708 | -0.33041 | -0.98658 | 0.644591 | 0.450873 | 1.724953 | -0.75034 | -0.52979 | -1.53053 | -1.86297 | -0.62149 | -0.27255 | -1.14649 | -0.85452 | -1.0756 | -1.65708 | -0.70326 | -1.09252 |
| Q9CZW5 | Q9CZW5 | TOM70_M( | Tomm70 | Mitochondrial import receptor subunit TOM70 | | | | 0.140248 | -0.03184 | -0.40606 | 0.085912 | 0.074886 | 0.258017 | 0.537298 | 0.395212 | 1.425036 | 0.212238 | 0.199437 | 0.269916 | -0.26107 | 0.959746 | 0.612457 | 0.159532 | 0.315816 | 0.046801 | -0.18773 | 0.607135 | 0.174644 |
| Q04750 | Q04750 | TOP1_MOI | Top1 | DNA topoisomerase 1 | | | | 0.912619 | 1.772764 | 2.590372 | 0.248058 | 0.17422 | 0.683478 | 0.177339 | 0.252733 | 0.503232 | -0.75456 | -1.13566 | -1.24407 | -0.73811 | -0.66301 | -1.37323 | -2.06057 | -3.37518 | -1.13405 | -1.19656 | -1.62599 | -0.91572 |
| Q64511 | Q64511 | TOP2B_M( | Top2b | DNA topoisomerase 2-beta | | | | 0.312837 | 0.86576 | 0.846097 | 0.45494 | 0.534791 | 1.207055 | 0.163919 | 0.424591 | 0.468353 | -1.87582 | -1.56247 | -1.5611 | -0.67502 | -0.48638 | -2.28906 | -1.57373 | -3.59608 | -1.65678 | -1.64892 | -1.90945 | -1.71518 |
| Q9Z321 | Q9Z321 | TOP3B_M( | Top3b | DNA topoisomerase 3-beta-1 | | | | NaN | 0 | NaN | 0.265421 | 0.187412 | 0.727165 | 0.308222 | 0.79358 | 0.834526 | -3.41764 | -2.74986 | -3.18209 | -3.16061 | -3.89173 | -2.00791 | -2.74562 | -3.21224 | -3.61627 | -3.10126 | -3.61272 | -3.87408 |
| P97807 | P97807 | FUMH_MC | Fh | Fumarate hydratase, mitochondrial | + | | | 1.35985 | -1.42879 | -4.6273 | 0.03999 | -0.1151 | -0.12489 | 0.062188 | 0.129124 | 0.190376 | 0.126326 | 0.735016 | 1.496192 | -0.34704 | 0.458686 | 1.42462 | 1.807353 | 1.911571 | 0.692523 | 0.686834 | 1.288739 | 0.336319 |
| Q62393 | Q62393 | TPD52_MC | Tpd52 | Tumor protein D52 | | | | 0.011407 | 0.018547 | 0.036675 | 0.47636 | 0.594064 | 1.262916 | 0.251827 | -0.24215 | -0.69297 | -3.31601 | -2.43717 | 0.137808 | -0.79847 | -1.44982 | -2.14772 | -3.14543 | -2.64484 | -0.97037 | -0.87842 | -1.57488 | -1.53837 |
| Q9CYZ2 | Q9CYZ2 | TPD54_MC | Tpd52l2 | Tumor protein D54 | | | | 0.284017 | 0.167848 | 0.773842 | 0.701511 | 0.424275 | 1.893288 | 0.097078 | -0.10458 | -0.28914 | -0.77799 | -1.16641 | -0.23695 | -0.67562 | -1.13312 | -1.267 | -1.04346 | -1.23664 | -0.92652 | -0.8346 | -1.45092 | -0.74005 |
| Q99MS8 | Q99MS8 | TPGS1_MC | Tpgs1 | Tubulin polyglutamylase complex subunit 1 | | | | NaN | 0 | NaN | 0.304595 | -0.50802 | -0.82543 | 0.270948 | -0.5907 | -0.74105 | -3.49343 | -4.04116 | -2.97723 | -2.70386 | -3.96389 | -3.30381 | -3.28854 | -2.97387 | -2.93262 | -1.73243 | -3.76872 | -2.31758 |
| Q8CAY6 | Q8CAY6;Q | THIC_MOI | Acat2 | Acetyl-CoA acetyltransferase, cytosolic | + | | | 1.367559 | -1.4389 | -4.67139 | 0.53206 | -1.42332 | -1.41089 | 0.377182 | 1.299937 | 1.007998 | -0.66567 | -1.07307 | -1.36734 | -3.33521 | -0.78073 | -0.32817 | 0.800587 | 0.338481 | -0.70533 | -1.15058 | -0.58477 | -3.12401 |
| P17751 | P17751 | TPIS_MOU | Tpi1 | Triosephosphate isomerase | | | | 0.978473 | 0.328732 | 2.836217 | 0.016145 | 0.012139 | 0.051642 | 0.017471 | -0.01377 | -0.05581 | 3.352666 | 3.260701 | 3.692285 | 3.228915 | 3.478456 | 3.926543 | 3.084345 | 2.871558 | 3.488173 | 3.408749 | 3.819728 | 3.612814 |
| P58771 | P58771 | TPM1_MOI | Tpm1 | Tropomyosin alpha-1 chain | | | | 0.412205 | -0.66971 | -1.09703 | 0.380001 | 0.5445 | 1.015135 | 0.157822 | -0.18154 | -0.45241 | -1.60013 | -0.68078 | 0.221801 | -0.6565 | -0.38217 | -0.07611 | -0.87247 | -0.06903 | -1.06983 | -0.45387 | 0.323362 | -0.41855 |
| P21107 | P58771;P2 | TPM3_MOI | Tpm3 | Tropomyosin alpha-3 chain | | | | 0.343281 | 0.142169 | 0.922515 | 0.315725 | 0.60307 | 0.853339 | 0.343111 | -0.24938 | -0.92209 | -0.06708 | 0.223507 | 0.911888 | 0.115023 | 0.408134 | 0.378499 | -0.11533 | -0.01258 | -0.67331 | 0.494082 | 0.912743 | 0.372651 |
| D3Z2H9 | D3Z2H9 | D3Z2H9_M | Tpm3-rs7 | Tropomyosin 3, related sequence 7 | | | + | 0.486245 | 1.910792 | 1.288877 | 0.632821 | 1.051901 | 1.691005 | 1.331693 | -0.67418 | -4.46934 | -0.29893 | -0.7297 | -0.13848 | -0.29196 | -1.10992 | -0.81839 | -3.89191 | -0.95832 | -1.88443 | -0.64982 | -0.32878 | -0.25118 |
| Q64514 | Q64514 | TPP2_MOI | Tpp2 | Tripeptidyl-peptidase 2 | | | | 0.398224 | 0.139889 | 1.061384 | 0.086685 | -0.0524 | -0.26019 | 0.384485 | 0.302536 | 1.026494 | -0.42113 | -0.46452 | 0.01399 | -0.38872 | 0.365605 | -0.18687 | -0.45271 | -0.71272 | -0.13081 | -0.13913 | -0.11043 | -0.31591 |
| Q7TQD2 | Q7TQD2 | TPPP_MOI | Tppp | Tubulin polymerization-promoting protein | | | | 0.035228 | 0.023236 | 0.110521 | 0.037192 | 0.030581 | 0.116461 | 0.215999 | -0.26695 | -0.60235 | 1.944044 | 2.279262 | 2.766448 | 2.274645 | 2.242801 | 2.850915 | 1.961506 | 2.215329 | 2.397855 | 2.582076 | 3.136236 | 2.491383 |
| P61922 | P61922 | GABT_MOI | Abat | 4-aminobutyrate aminotransferase, mitochondrial | + | | | 1.311643 | -1.57116 | -4.35973 | 0.023051 | -0.09465 | -0.0732 | 0.221422 | -0.59056 | -0.61613 | -0.0062 | 0.613208 | 1.24305 | -1.27097 | -0.99049 | 0.583457 | 2.058938 | 1.690392 | 0.383427 | -0.22205 | 0.934229 | -0.16013 |
| P63028 | P63028 | TCTP_MOI | Tpt1 | Translationally-controlled tumor protein | | | | NaN | 0 | NaN | 0.190174 | 0.553897 | 0.53634 | 0.787174 | -0.55461 | -2.16111 | -2.86898 | -3.14171 | -1.31017 | -3.31234 | -3.07946 | -2.95451 | -3.81601 | -2.88498 | -2.61145 | -3.11887 | -2.71128 | -2.21346 |
| Q922B6 | Q922B6 | TRAF7_MC | Traf7 | E3 ubiquitin-protein ligase TRAF7 | | | | 0.32503 | 0.578041 | 0.876679 | 0.152594 | 0.040365 | 0.438685 | 0.917846 | -1.14169 | -2.60934 | -3.40627 | -2.13615 | -1.06513 | -0.93551 | -2.25867 | -1.40448 | -1.17193 | -3.52658 | -0.97537 | -1.106 | -0.59484 | -0.78493 |
| Q6PD31 | Q6PD31 | TRAK1_MC | Trak1 | Trafficking kinesin-binding protein 1 | | | | NaN | 0 | NaN | 0.18366 | 0.405023 | 0.519564 | 0.563154 | 0.277488 | 1.495468 | -3.73932 | -2.0613 | -2.34406 | -2.93013 | -3.21466 | -3.05366 | -3.6136 | -3.5058 | -3.76449 | -2.31975 | -3.57883 | -3.24446 |
| Q6P9N8 | Q6P9N8 | Q6P9N8_I | Trak2 | Trafficking protein, kinesin binding 2 | | | | 0.553433 | -2.00051 | -1.46886 | 0.771972 | 0.490998 | 2.112202 | 0.720106 | 0.788959 | 1.949871 | -0.93934 | -3.66323 | -0.23614 | 0.038377 | -1.38232 | -0.93073 | -0.30175 | -0.29981 | -0.77749 | -0.40227 | -2.28125 | -1.60972 |
| Q9CQN1 | Q9CQN1 | TRAP1_MC | Trap1 | Heat shock protein 75 kDa, mitochondrial | + | + | | 1.098228 | 0.622928 | 3.325472 | 3.551075 | 0.291038 | 59.62743 | 0.091018 | 0.093345 | 0.272301 | 4.743912 | 4.544411 | 4.892565 | 4.884459 | 5.423044 | 4.751908 | 3.862681 | 4.179785 | 4.594755 | 4.600194 | 5.064185 | 4.924076 |
| Q5NCF2 | Q5NCF2 | TPPC1_MC | Trappc1 | Trafficking protein particle complex subunit 1 | | | | 0.259152 | 0.076537 | 0.711407 | 0.162617 | -0.11768 | -0.46495 | 0.083022 | -0.04712 | -0.24989 | 0.131785 | -0.03089 | -0.20357 | -0.48345 | -0.18795 | 0.019331 | -0.09651 | 0.044326 | -0.01492 | -0.43673 | -0.19473 | 0.120357 |
| Q3TLI0 | Q3TLI0 | TPC10_MC | Trappc10 | Trafficking protein particle complex subunit 10 | | | | 0.326899 | 0.325437 | 0.881368 | 0.251436 | -0.15269 | -0.69199 | 0.081095 | -0.07617 | -0.24446 | -1.4766 | -0.74405 | -0.28988 | 0.142084 | -0.23572 | -0.16761 | -1.38907 | -1.48246 | 0.033601 | 0.123992 | -0.43523 | 0.184245 |
| B2RXC1 | B2RXC1 | TPC11_MC | Trappc11 | Trafficking protein particle complex subunit 11 | | | | 0.41047 | -0.7872 | -1.0926 | 0.490776 | -0.27968 | -1.30082 | 0.416531 | -0.19686 | -1.10809 | -3.22556 | -2.12491 | -1.42334 | -1.31416 | -1.3272 | -1.52754 | -1.42302 | -2.35305 | -1.29703 | -0.88111 | -1.37724 | -1.08379 |
| Q8K2L8 | Q8K2L8 | TPC12_MC | Trappc12 | Trafficking protein particle complex subunit 12 | | + | | 0.529569 | -1.40958 | -1.40419 | 1.044711 | -0.97087 | -3.09968 | 0.19051 | 0.097285 | 0.537203 | -3.62842 | -3.37993 | -1.28541 | -1.78247 | -1.20338 | -1.40672 | -1.09847 | -3.09072 | -0.37245 | -0.75369 | -1.25247 | -1.5522 |
| Q3UTZ3 | Q3UTZ3 | TPC14_MC | Trappc14 | Trafficking protein particle complex subunit 14 | | | | 0.14468 | -0.5477 | -0.41781 | 0.83 | -0.40059 | -2.30234 | 0.152914 | 0.237589 | 0.439527 | -0.85736 | -3.47203 | -0.39114 | -0.58154 | 0.154768 | -0.60719 | -1.52059 | -1.71341 | 0.059888 | -0.23139 | -0.84728 | -0.08032 |
| P55258 | P55258 | RAB8A_MC | Rab8a | Ras-related protein Rab-8A | + | | | 1.927194 | -1.62363 | -9.1142 | 0.281253 | 0.639786 | 0.766909 | 0.80842 | 1.172625 | 2.230523 | -3.49376 | -3.41206 | -1.99138 | -2.64942 | -2.05044 | -2.51551 | -2.00268 | -1.65588 | -3.7268 | -2.19357 | -2.9841 | -3.92709 |
| O55013 | O55013 | TPPC3_MC | Trappc3 | Trafficking protein particle complex subunit 3 | | | | 0.597382 | 0.28876 | 1.590426 | 0.701377 | -0.17931 | -1.89288 | 0.838593 | -0.64383 | -2.33131 | 0.62531 | 0.342259 | 1.197964 | 1.382992 | 0.517349 | 0.986094 | 0.308757 | 0.081292 | 1.449426 | 1.490147 | 1.249479 | 1.541631 |
| Q8JZV7 | Q8JZV7 | NAGA_MO | Amdhd2 | N-acetylglucosamine-6-phosphate deacetylase | + | | | 1.818573 | -1.64024 | -8.02225 | 0.387121 | -0.6039 | -1.03318 | 0.444888 | 0.639741 | 1.181014 | -3.09457 | -2.75829 | -3.40801 | -2.60278 | -1.49448 | -1.76185 | -1.16985 | -1.40253 | -1.97776 | -2.82522 | -1.74297 | -2.79283 |
| Q9CQA1 | Q9CQA1 | TPPC5_MC | Trappc5 | Trafficking protein particle complex subunit 5 | + | | | 1.008819 | 0.420803 | 2.95481 | 0.910742 | 0.362032 | 2.583586 | 0.31742 | -0.2332 | -0.85759 | -0.07632 | -0.34833 | 0.114252 | -0.00157 | 0.139538 | -0.34502 | -0.5909 | -0.67536 | -0.43329 | -0.17809 | 0.006991 | 0.253928 |
| Q9D289 | Q9D289 | TPC6B_MC | Trappc6b | Trafficking protein particle complex subunit 6B | | | | 0.205553 | 0.826674 | 0.575738 | 0.241786 | 0.305374 | 0.667662 | 0.184184 | -0.12087 | -0.52092 | -3.40193 | -0.85105 | 0.028 | 0.121636 | -0.22887 | -0.07207 | -3.61265 | -2.29368 | -0.68553 | 0.22442 | -0.24799 | 0.188802 |
| A0A286YC | A0A286YC | A0A286YC | Trappc8 | Trafficking protein particle complex 8 | | | | 0.496955 | 1.860667 | 1.317146 | 0.317803 | -0.14828 | -0.85855 | 0.610797 | 0.515225 | 1.628218 | -0.27478 | -0.56415 | -0.97826 | -0.69444 | -0.63632 | -1.01268 | -0.87491 | -3.68535 | -0.78651 | -0.58963 | -1.08533 | -1.59413 |
| Q9R0N4 | Q9R0N4 | SYT10_MC | Syt10 | Synaptotagmin-10 | + | | | 1.307233 | -1.65964 | -4.33594 | 0.084446 | 0.256783 | 0.253897 | 0.009689 | 0.045545 | 0.031208 | -3.52795 | -3.35694 | -2.22781 | -1.94381 | -1.37862 | -3.82223 | -2.1559 | -1.40971 | -3.34394 | -1.34125 | -1.84781 | -3.44414 |
| Q9ESN6 | Q9ESN6 | TRIM2_MC | Trim2 | Tripartite motif-containing protein 2 | | | | 0.27767 | 0.326833 | 0.75792 | 0.390598 | -0.40596 | -1.042 | 0.800371 | 0.601439 | 2.204079 | -0.83517 | -1.0465 | -0.45877 | -1.03343 | 0.676132 | 0.131123 | -0.84959 | -1.68575 | -0.60325 | -0.07703 | -0.21204 | -0.18359 |
| Q9R1R2 | Q9R1R2 | TRIM3_MC | Trim3 | Tripartite motif-containing protein 3 | | | | 0.288771 | 1.137552 | 0.785763 | 0.185995 | -0.07234 | -0.52558 | 0.867069 | 0.175497 | 2.428923 | -0.93645 | -0.79781 | 0.18119 | -0.04445 | 0.459749 | 0.324443 | -0.55864 | -3.45073 | 0.061862 | 0.219569 | 0.241968 | 0.19123 |
| Q8CH72 | Q8CH72 | TRI32_MOI | Trim32 | E3 ubiquitin-protein ligase TRIM32 | | | | 0.148361 | -0.60743 | -0.42753 | 0.223001 | -0.19865 | -0.62014 | 0.176302 | -0.18245 | -0.50055 | -1.86488 | -3.58196 | -0.66204 | -0.72573 | -0.54519 | -1.18822 | -0.98393 | -3.24804 | -0.81398 | -0.17648 | -0.85598 | -0.51253 |
| Q6PCX9 | Q6PCX9 | TRI37_MOI | Trim37 | E3 ubiquitin-protein ligase TRIM37 | | | | 0.077395 | 0.088853 | 0.233982 | 0.421527 | 0.338321 | 1.120885 | 0.674337 | 1.111548 | 1.812036 | -3.37236 | -3.24315 | -1.51949 | -2.0055 | -2.0613 | -1.94523 | -3.0224 | -3.77082 | -2.27985 | -1.92179 | -3.72549 | -2.50414 |

| | | | | | | | | | | | | | | | | | | | | | | | | | | | | |
|---|---|---|---|---|---|---|---|---|---|---|---|---|---|---|---|---|---|---|---|---|---|---|---|---|---|---|---|---|
| Q7TNM2 | Q7TNM2 | TRI46_MO | Trim46 | Tripartite motif-containing protein 46 | | | | 0.048482 | 0.289512 | 0.150222 | 0.67386 | 0.459897 | 1.810626 | 0.154067 | -0.16977 | -0.44256 | -0.39857 | -3.26099 | -0.27032 | -0.32043 | -0.46925 | -0.92332 | -0.82861 | -3.40997 | -1.00803 | -0.50251 | -0.83573 | -0.21729 |
| Q8JZK6 | Q8JZK6 | Q8JZK6_M | Trim61 | RING finger protein 35 | | | | NaN | 0 | NaN | 0.833693 | 0.889805 | 2.314764 | 0.240422 | -0.40128 | -0.66422 | -3.08636 | -0.35502 | 0.221964 | 0.325433 | -0.01491 | -0.48366 | -2.79923 | -3.73285 | -0.99701 | -0.2352 | -0.40483 | 0.70883 |
| Q8C7M3 | Q8C7M3 | TRIM9_MO | Trim9 | E3 ubiquitin-protein ligase TRIM9 | | | | 0.181961 | 0.339538 | 0.515181 | 0.138913 | -0.13896 | -0.40251 | 0.688727 | -0.50791 | -1.85485 | 0.323995 | -0.75853 | 1.650825 | 1.849211 | 1.095988 | 1.547617 | -0.18077 | -0.93284 | 1.558309 | 2.219642 | 1.674829 | 1.984602 |
| G5E870 | G5E870 | TRIPC_MO | Trip12 | E3 ubiquitin-protein ligase TRIP12 | | | | 0.12259 | 0.055345 | 0.358822 | 0.286672 | 0.500241 | 0.780501 | 0.247775 | -0.20663 | -0.68276 | -2.8971 | -2.59029 | -1.94807 | -1.63186 | -2.34848 | -2.809 | -2.81508 | -2.783 | -2.91132 | -1.66909 | -2.5685 | -2.17574 |
| Q9D735 | Q9D735 | TRIR_MOU | Trir | Telomerase RNA component interacting RNase | | | | NaN | 0 | NaN | 0.592984 | -0.64337 | -1.57811 | 0.235104 | -0.24043 | -0.65079 | -3.36491 | -3.13023 | -1.62375 | -0.87999 | -1.54913 | -0.84212 | -2.67991 | -3.23337 | -0.77558 | -0.44142 | -0.84782 | -1.06257 |
| Q4QQM4 | Q4QQM4 | P5I11_MO | Trp53i11 | Tumor protein p53-inducible protein 11 | | | | 0.53301 | 0.970384 | 1.413456 | 0.00968 | -0.01979 | -0.03118 | 0.301067 | 0.20974 | 0.816589 | -0.26185 | -0.46151 | 0.094739 | -1.03345 | 0.096342 | 0.494269 | -0.65282 | -2.01129 | -0.74022 | -0.15892 | 0.248 | -0.07687 |
| Q9QUQ5 | Q9QUQ5 | TRPC4_MO | Trpc4 | Short transient receptor potential channel 4 | | | + | NaN | 0 | NaN | NaN | 0 | NaN | 1.47557 | -1.4833 | -5.32931 | -2.85293 | -3.97791 | -3.33909 | -3.29225 | -3.53011 | -2.97984 | -2.91058 | -3.73581 | -3.94998 | -3.23692 | -1.81374 | -1.72962 |
| Q5F4S8 | Q5F4S8 | Q5F4S8_M | Trpm3 | Transient receptor potential cation channel, subfamily M, member 3 | | + | + | 0.402766 | -0.70419 | -1.07295 | 1.076645 | -1.24117 | -3.23295 | 1.514309 | -0.98318 | -5.58478 | -0.67873 | -1.28594 | -1.19643 | -0.42973 | -0.44397 | -0.51973 | 0.303721 | -0.86002 | 0.448873 | 0.407301 | 0.329403 | 0.673245 |
| Q9EP53 | Q9EP53 | TSC1_MO | Tsc1 | Hamartin | | | | 0.263347 | 1.169741 | 0.721954 | 0.195875 | -0.12946 | -0.55098 | 0.442657 | -0.48732 | -1.17525 | -1.05605 | -1.91843 | -0.6198 | -0.39007 | -1.24284 | -0.85296 | -1.09517 | -4.2188 | -0.58045 | -0.1705 | -0.92655 | -0.1946 |
| Q61037 | Q61037 | TSC2_MO | Tsc2 | Tuberin | | | | 0.281715 | 0.384096 | 0.768069 | 0.146182 | 0.05034 | 0.421779 | 0.087443 | -0.13961 | -0.26231 | -0.91528 | -1.42336 | -0.37954 | -0.38366 | -0.87574 | -0.65782 | -1.12267 | -1.98416 | -0.55127 | -0.31261 | -1.14814 | -0.1062 |
| P62500 | P62500 | T22D1_MO | Tsc22d1 | TSC22 domain family protein 1 | | | | NaN | 0 | NaN | 0.132447 | 0.495962 | 0.38528 | 0.596501 | -0.2451 | -1.58796 | -3.94418 | -3.80278 | -1.97099 | -4.34821 | -2.54045 | -2.6806 | -2.46281 | -2.19955 | -3.16131 | -4.14982 | -2.22791 | -2.50295 |
| Q61187 | Q61187 | TS101_MO | Tsg101 | Tumor susceptibility gene 101 protein | | | | 0.903696 | 0.306988 | 2.558215 | 0.001287 | -0.00082 | -0.00419 | 0.144301 | -0.03044 | -0.41681 | 0.157923 | 0.3949 | 1.006612 | 1.200072 | 0.862303 | 0.966487 | -0.04957 | -0.01158 | 0.932929 | 1.275403 | 0.89364 | 0.996038 |
| Q62348 | Q62348 | TSN_MOU | Tsn | Translin | + | | | 1.273696 | -0.74576 | -4.15857 | 0.152224 | -0.1422 | -0.43771 | 0.134718 | -0.05336 | -0.39134 | -0.27344 | 0.049126 | -0.33596 | -0.89866 | -0.1175 | -0.05723 | 0.712018 | 0.555199 | -0.63756 | -0.31265 | 0.098971 | -0.16699 |
| P18242 | P18242 | CATD_MO | Ctsd | Cathepsin D | + | | | 2.094804 | -1.67708 | -11.0859 | 0.335175 | -0.65468 | -0.90215 | 0.028084 | -0.06467 | -0.08873 | -0.50276 | -0.38849 | 0.723751 | -0.70016 | -0.26242 | 0.94687 | 1.091374 | 1.37153 | 0.525939 | 0.807018 | 0.813787 | 0 |
| Q7TQI8 | Q7TQI8 | TSYL2_MO | Tspyl2 | Testis-specific Y-encoded-like protein 2 | | | | 0.564206 | 0.694906 | 1.498359 | 0.489975 | 0.81517 | 1.298706 | 0.341799 | -0.88027 | -0.91879 | -1.8059 | -2.73288 | -2.54277 | -1.32565 | -4.18906 | -2.54196 | -2.98064 | -2.94795 | -2.90312 | -2.59564 | -1.99569 | -2.9748 |
| Q8VD63 | Q8VD63 | TSYL4_MO | Tspyl4 | Testis-specific Y-encoded-like protein 4 | | | | 0.648612 | 2.09311 | 1.736616 | 0.275013 | 0.211631 | 0.751251 | 0.1476 | -0.16528 | -0.42552 | 0.139562 | -0.32037 | -0.46236 | -0.42713 | -0.49864 | -1.04589 | -1.00037 | -3.36665 | -0.93753 | -0.37522 | -0.88267 | -0.3313 |
| Q8C8T8 | Q8C8T8 | TSR2_MO | Tsr2 | Pre-rRNA-processing protein TSR2 homolog | | + | + | 0.384642 | 0.545712 | 1.026893 | 1.033995 | -0.19011 | -3.05589 | 1.245401 | -0.92978 | -4.01374 | -3.11904 | -2.06653 | -0.26268 | -0.22069 | -1.27714 | -1.18868 | -3.06456 | -3.21243 | 0.006984 | -0.11013 | -0.07575 | -0.53052 |
| Q8R0F8 | Q8R0F8 | FAHD1_M | Fahd1 | Acylpyruvase FAHD1, mitochondrial | + | | | 1.501136 | -1.69122 | -5.49669 | 0.017482 | 0.036915 | 0.055841 | 0.106605 | -0.39599 | -0.31538 | -3.88272 | -3.39249 | -2.81463 | -2.12408 | -2.12466 | -0.93825 | -1.76042 | -2.13236 | -3.07002 | -1.94252 | -1.01789 | -2.25305 |
| O88196 | O88196 | TTC3_MO | Ttc3 | E3 ubiquitin-protein ligase TTC3 | | | | 0.659165 | 0.56864 | 1.767383 | 0.223899 | 0.145794 | 0.622415 | 0.247011 | -0.22302 | -0.68084 | -0.73024 | -1.20984 | 0.193359 | 0.658371 | -0.92723 | -0.84007 | -1.32417 | -1.75318 | 0.30851 | 0.251632 | -0.98528 | -0.33598 |
| Q8VE09 | Q8VE09 | TT39C_MC | Ttc39c | Tetratricopeptide repeat protein 39C | | | + | NaN | 0 | NaN | NaN | 0 | NaN | 1.047264 | -0.9678 | -3.11018 | -3.0848 | -2.86767 | -3.77236 | -3.64035 | -3.38637 | -3.59583 | -3.25607 | -3.17772 | -4.03114 | -3.44837 | -2.81632 | -2.23029 |
| E9Q6P5 | E9Q6P5 | TTC7B_MC | Ttc7b | Tetratricopeptide repeat protein 7B | | | | 0.559918 | 0.274208 | 1.486595 | 0.121056 | -0.12074 | -0.35468 | 0.338774 | -0.422 | -0.91119 | 1.734052 | 1.765358 | 2.911149 | 3.558537 | 2.042336 | 2.968512 | 1.659286 | 1.291708 | 3.250231 | 3.460927 | 2.921301 | 2.93354 |
| Q3V038 | Q3V038 | TTC9A_MC | Ttc9 | Tetratricopeptide repeat protein 9A | | | | 0.480037 | -0.18811 | -1.27256 | 0.06895 | 0.048821 | 0.209882 | 0.223718 | 0.251175 | 0.621956 | -0.7111 | -1.00672 | -1.77874 | -1.44861 | -1.00833 | -1.09842 | -0.67246 | -0.66915 | -1.82639 | -1.4986 | -0.90322 | -1.70587 |
| A4Q9F0 | A4Q9F0 | TTLL7_MO | Ttll7 | Tubulin polyglutamylase TTLL7 | | | | NaN | 0 | NaN | 0.405425 | 0.993935 | 1.079722 | 0.398839 | 0.543402 | 1.062947 | -3.51772 | -3.38475 | -2.0137 | -1.21513 | -3.35751 | -3.03726 | -2.89003 | -3.30335 | -3.43779 | -1.7789 | -3.25529 | -4.22628 |
| P07309 | P07309 | TTHY_MO | Ttr | Transthyretin | | | | 0.593532 | 1.723866 | 1.579644 | 0.256239 | -0.65394 | -0.70408 | 0.184222 | 0.286369 | 0.521014 | -0.44477 | -0.72302 | -4.0903 | -2.64657 | -1.28292 | -0.29418 | -3.39016 | -1.22537 | -2.13007 | -3.29893 | -1.31513 | -0.83471 |
| Q9D3A9 | Q9D3A9 | TTYH1_MC | Ttyh1 | Protein tweety homolog 1 | | | | 0.111719 | -0.07513 | -0.32936 | 0.335246 | -1.66071 | -0.90232 | 0.293028 | -0.35116 | -0.79644 | -0.04415 | 0.246377 | -0.35347 | -3.90118 | -0.8657 | -0.24334 | 0.352118 | 0.000368 | -0.95733 | 0.024106 | 0.10899 | -0.51572 |
| Q6P5F7 | Q6P5F7 | TTYH3_MC | Ttyh3 | Protein tweety homolog 3 | | | | 0.615058 | -0.64296 | -1.64029 | 0.380116 | -0.69009 | -1.01543 | 0.159903 | 0.536875 | 0.457858 | -3.98269 | -4.27538 | -3.37877 | -3.31821 | -4.00592 | -2.1307 | -3.12244 | -3.84971 | -3.33733 | -1.97946 | -4.30933 | -2.90104 |
| P50586 | Q9Z273;P | TUB_MOU | Tub | Tubby protein | | | | 0.282067 | 1.223587 | 0.768952 | 0.686006 | 0.656305 | 1.846724 | 0.545954 | -0.83647 | -1.4485 | -0.97118 | -1.03192 | 1.092487 | 1.768897 | -1.41444 | -0.904 | -3.81609 | -0.63419 | 0.665216 | 0.883559 | -0.84077 | 0.195255 |
| P68369 | P68373;Q | TBA1A_MC | Tuba1a | Tubulin alpha-1A chain | + | | | 1.234125 | 1.931238 | 3.957212 | 0.521651 | 0.643224 | 1.382915 | 0.063337 | 0.09097 | 0.193704 | 7.039282 | 7.03667 | 8.272749 | 8.40024 | 8.253571 | 7.931606 | 4.61871 | 5.594766 | 7.232537 | 8.154003 | 7.560439 | 8.442798 |
| P68368 | Q3UX10;P | TBA4A_MC | Tuba4a | Tubulin alpha-4A chain | + | | | 1.012333 | 0.969053 | 2.968768 | 0.412202 | 0.343291 | 1.09702 | 0.04348 | 0.047895 | 0.135346 | 7.342696 | 7.297714 | 8.448709 | 8.618155 | 8.253969 | 8.130205 | 6.676792 | 6.025512 | 7.888897 | 8.491385 | 7.79577 | 8.492614 |
| Q9JJZ2 | P68373;Q | TBA8_MO | Tuba8 | Tubulin alpha-8 chain | | | | 0.797988 | 1.203404 | 2.196283 | 0.758783 | 0.626305 | 2.070272 | 0.123539 | -0.12951 | -0.36138 | 0.138226 | 1.220694 | 1.045058 | 1.335501 | 0.693971 | 0.636275 | -0.43856 | -0.60933 | 0.298585 | 0.829362 | 0.43742 | 1.151845 |
| Q3UX10 | Q3UX10 | TBAL3_MC | Tubal3 | Tubulin alpha chain-like 3 | | | | 0.545916 | 1.225928 | 1.448394 | 0.553145 | 0.915798 | 1.468076 | 0.383342 | -0.48622 | -1.0236 | -1.5681 | -3.25095 | -0.77193 | -0.53135 | -1.34498 | -1.87145 | -3.54379 | -3.72712 | -2.17954 | -0.95534 | -1.51739 | -0.7266 |
| Q7TMM9 | Q7TMM9; | TBB2A_MC | Tubb2a | Tubulin beta-2A chain | + | | | 1.050056 | 0.911071 | 3.12169 | 0.440255 | 0.394866 | 1.169048 | 0.002196 | 0.001708 | 0.007134 | 5.481621 | 5.49625 | 6.725803 | 6.836355 | 6.815413 | 6.785404 | 4.286104 | 4.869625 | 6.053 | 6.719427 | 6.559772 | 7.037629 |
| Q8VDQ8 | Q8VDQ8 | SIR2_MOU | Sirt2 | NAD-dependent protein deacetylase sirtuin-2 | + | | | 2.091222 | -1.69672 | -11.0397 | 0.032337 | -0.06135 | -0.10174 | 0.070886 | 0.150144 | 0.215432 | 1.584359 | 1.694897 | 1.536526 | 0.340514 | 0.837909 | 1.931083 | 3.479756 | 3.192935 | 0.922384 | 1.077352 | 1.666755 | 0.80195 |
| Q9ERD7 | Q922F4;Q | TBB3_MO | Tubb3 | Tubulin beta-3 chain | | | | 0.689638 | 0.665287 | 1.85758 | 0.541585 | 0.546983 | 1.436641 | 0.061778 | -0.06365 | -0.18919 | 7.505541 | 7.548797 | 8.286066 | 8.301382 | 7.631947 | 7.667652 | 7.219376 | 6.504388 | 7.36608 | 8.127401 | 7.37749 | 8.049407 |
| Q9D6F9 | Q9D6F9 | TBB4A_MC | Tubb4a | Tubulin beta-4A chain | + | | | 1.099106 | 2.04228 | 3.329276 | 0.592647 | 0.932787 | 1.577168 | 0.21733 | 0.303695 | 0.605737 | 4.449893 | 4.827845 | 5.60025 | 5.788122 | 5.077372 | 4.867288 | 2.012992 | 3.180186 | 4.177474 | 5.345323 | 4.178398 | 5.158872 |
| P97384 | P97384 | ANX11_MC | Anxa11 | Annexin A11 | + | | | 2.354439 | -1.71909 | -14.9891 | 0.018309 | 0.054485 | 0.058432 | 0.303643 | 0.407182 | 0.823046 | -4.10177 | -3.98218 | -2.35019 | -3.09805 | -0.73511 | -1.62973 | -2.22501 | -2.42075 | -1.92441 | -3.6328 | -1.37825 | -1.80095 |
| P99024 | P99024 | TBB5_MO | Tubb5 | Tubulin beta-5 chain | + | | | 1.081335 | 1.634629 | 3.252888 | 0.450905 | 0.680188 | 1.196589 | 0.313783 | 0.153832 | 0.848468 | 4.680751 | 4.423638 | 6.364458 | 6.680124 | 5.856361 | 5.579181 | 2.431772 | 3.40336 | 5.296015 | 6.388191 | 5.447043 | 5.680835 |
| Q922F4 | Q922F4 | TBB6_MO | Tubb6 | Tubulin beta-6 chain | | | | 0.638266 | 2.158154 | 1.706675 | 0.406394 | 1.166414 | 1.082193 | 0.332693 | 0.952891 | 0.895912 | -1.33728 | 1.015955 | 2.589364 | 3.172655 | 2.431945 | 2.444181 | -2.78209 | -1.85554 | 0.676979 | 2.752212 | 0.421592 | 2.548753 |
| P83887;Q | P83887;Q | TBG1_MO | Tubg1;Tubg2 | Tubulin gamma-1 chain | | | | 0.059072 | -0.08087 | -0.18132 | 0.306667 | -0.32628 | -0.83063 | 0.74971 | -0.43764 | -2.04169 | 0.699174 | -0.03647 | 1.257997 | 2.017732 | 0.851686 | 0.859546 | 0.160014 | 0.664426 | 1.864144 | 2.06414 | 1.078938 | 1.507566 |
| Q921G8 | Q921G8 | GCP2_MO | Tubgcp2 | Gamma-tubulin complex component 2 | | | | 0.068021 | -0.12247 | -0.20722 | 0.769995 | -0.43226 | -2.10589 | 0.560985 | -0.58014 | -1.48952 | -1.66857 | -1.60994 | -0.15475 | -0.09806 | -1.08252 | -1.60313 | -2.10709 | -0.92648 | 0.10256 | 0.509155 | -1.0524 | -0.47296 |
| P58854 | P58854 | GCP3_MO | Tubgcp3 | Gamma-tubulin complex component 3 | | | + | 0.226267 | 0.434975 | 0.628417 | 0.065339 | -0.0295 | -0.19949 | 1.045899 | -0.25527 | -3.10456 | -0.47531 | -0.72304 | -0.03256 | 0.236759 | -0.58498 | -0.57017 | -0.35315 | -1.71515 | 0.192646 | 0.070544 | -0.40419 | -0.24041 |
| Q9D4F8 | Q9D4F8 | GCP4_MO | Tubgcp4 | Gamma-tubulin complex component 4 | + | | | 1.154957 | 1.360357 | 3.578498 | 0.316123 | 0.314378 | 0.854335 | 0.530685 | -0.58724 | -1.40719 | -1.7567 | -1.14311 | -1.23634 | -0.68781 | -1.79265 | -1.98995 | -2.58579 | -3.03474 | -1.52179 | -1.03113 | -1.70955 | -0.89857 |
| Q8BFR5 | Q8BFR5 | EFTU_MO | Tufm | Elongation factor Tu, mitochondrial | + | + | | 1.180195 | 0.622809 | 3.695872 | 1.044757 | 0.428693 | 3.099866 | 0.09798 | -0.10954 | -0.29164 | 3.838647 | 3.577471 | 4.50181 | 4.749551 | 4.434021 | 4.247962 | 2.978743 | 3.191759 | 4.135495 | 4.25848 | 4.086642 | 4.814413 |
| Q9WVF8 | Q9WVF8 | TUSC2_MC | Tusc2 | Tumor suppressor candidate 2 | | | | 0.595178 | -1.58803 | -1.58425 | 0.544169 | -0.14695 | -1.44365 | 0.676272 | -0.77497 | -1.81777 | -2.93989 | -1.03608 | 1.024518 | 1.227791 | -0.39543 | 0.457143 | -0.71404 | -0.08587 | 1.267469 | 1.278746 | 0.8118 | 0.799849 |
| Q91YR1 | Q91YR1 | TWF1_MO | Twf1 | Twinfilin-1 | | | | 0.20356 | -0.30904 | -0.57065 | 0.554922 | -0.43067 | -1.47293 | 0.654181 | 0.338245 | 1.752822 | -0.89429 | -1.22401 | -0.68355 | -0.97036 | 0.409528 | 0.150445 | -0.23425 | -1.26597 | -0.14147 | -0.65109 | 0.08477 | -0.20129 |
| P10639 | P10639 | THIO_MO | Txn | Thioredoxin | | | | 0.985607 | 0.416102 | 2.863788 | 0.286728 | -0.15948 | -0.78064 | 0.390527 | -0.14731 | -1.04182 | 3.400639 | 3.288546 | 3.037001 | 3.427673 | 3.153543 | 3.415137 | 3.062544 | 2.794438 | 3.451638 | 3.331993 | 3.377941 | 3.485353 |
| Q9CQM5 | Q9CQM5 | TXD17_MC | Txndc17 | Thioredoxin domain-containing protein 17 | + | | | 1.00328 | -2.0029 | -2.9329 | 0.312705 | 0.986818 | 0.845765 | 0.025192 | 0.066427 | 0.079826 | -3.73276 | -2.40095 | -0.36286 | -2.69615 | -1.71689 | -2.79127 | -1.21538 | -0.91252 | -2.49874 | -2.5339 | -1.68499 | -2.95602 |
| Q91W90 | Q91W90 | TXND5_MC | Txndc5 | Thioredoxin domain-containing protein 5 | | | | 0.305405 | -0.61744 | -0.82746 | 0.447195 | -0.60683 | -1.18698 | 0.016565 | 0.034836 | 0.052961 | -2.96615 | -4.24679 | -2.77901 | -1.7672 | -1.38501 | -2.5256 | -3.37215 | -2.60591 | -1.73993 | -1.59262 | -2.31789 | -1.66239 |
| P83877 | P83877 | TXN4A_MC | Txnl4a | Thioredoxin-like protein 4A | + | | | 1.266032 | 1.964595 | 4.118914 | 0.895299 | 0.858266 | 2.528189 | 0.803392 | -1.03786 | -2.21398 | -1.95513 | -1.01542 | 0.10043 | 0.321594 | -1.29745 | -0.84561 | -3.53196 | -3.36778 | -0.96822 | -0.32629 | -0.44441 | 0.377084 |
| Q9JMH6 | Q9JMH6 | TRXR1_MC | Txnrd1 | Thioredoxin reductase 1, cytoplasmic | | + | | 0.476538 | -0.79998 | -1.26338 | 1.242683 | -0.72712 | -4.00005 | 0.438726 | 0.44605 | 1.165103 | -0.59461 | 0.670932 | -0.56663 | -0.85378 | 0.140449 | 0.670172 | 0.861558 | 0.814723 | -0.09457 | 0.128401 | 0.235695 | -0.31717 |
| P26369 | P26369 | U2AF2_MC | U2af2 | Splicing factor U2AF 65 kDa subunit | | | | 0.477452 | 0.948935 | 1.265777 | 0.427303 | 0.198684 | 1.135703 | 0.203354 | -0.13539 | -0.57012 | -2.98277 | -1.53913 | -0.74694 | -1.05123 | -0.71349 | -1.18427 | -3.41238 | -3.00739 | -1.18413 | -1.01141 | -0.7821 | -0.84488 |
| Q6NV83 | Q6NV83 | SR140_MC | U2surp | U2 snRNP-associated SURP motif-containing protein | | | | 0.57453 | 2.38314 | 1.526804 | 0.312857 | -1.87682 | -0.84615 | 0.253675 | 0.453225 | 0.697625 | 0.022304 | -0.25444 | -0.36547 | 1.070648 | 0.946751 | -0.32904 | -0.45393 | -0.94449 | 0.719981 | 0.738833 | -0.2675 | -0.02125 |
| Q91YM2 | Q91YM2 | RHG35_MC | Arhgap35 | Rho GTPase-activating protein 35 | + | | + | 2.675935 | -1.769 | -21.741 | 0.767732 | 0.333968 | 2.098673 | 1.979792 | 1.351328 | 9.693103 | -3.45142 | -3.2995 | -1.19515 | -1.50991 | -1.35244 | -1.13929 | -1.63562 | -1.57731 | -1.71006 | -1.66294 | -2.50732 | -2.68706 |
| Q9Z1F9 | Q9Z1F9 | SAE2_MO | Uba2 | SUMO-activating enzyme subunit 2 | | | | 0.465663 | -0.79708 | -1.23495 | 0.237456 | 0.466462 | 0.656727 | 0.449209 | 0.686293 | 1.192196 | -3.38097 | -2.42803 | -1.42056 | -1.79473 | -1.54758 | -1.38726 | -1.67203 | -2.5428 | -2.75931 | -1.3889 | -1.58367 | -2.72376 |
| Q8C878 | Q8C878 | UBA3_MO | Uba3 | NEDD8-activating enzyme E1 catalytic subunit | | | | 0.258607 | 0.720913 | 0.710035 | 0.019617 | 0.019393 | 0.06252 | 0.241131 | -0.38655 | -0.66601 | -2.12233 | -1.32172 | -1.82023 | -1.97656 | -2.1697 | -1.28256 | -1.50987 | -3.37602 | -2.21796 | -1.61761 | -0.96528 | -1.71389 |
| Q8VE47 | Q8VE47 | UBA5_MO | Uba5 | Ubiquitin-like modifier-activating enzyme 5 | | | | 0.151415 | -0.42584 | -0.43558 | 0.672154 | -0.19551 | -1.80558 | 0.612785 | -0.43658 | -1.63385 | -2.48788 | -0.96973 | -1.13756 | -1.26846 | -1.24895 | -1.00424 | -0.6869 | -1.91905 | -1.09376 | -0.92124 | -0.45246 | -0.92757 |
| Q8C7R4 | Q8C7R4 | UBA6_MO | Uba6 | Ubiquitin-like modifier-activating enzyme 6 | | | | 0.24996 | -1.04852 | -0.68827 | 0.269534 | 0.437001 | 0.737496 | 0.162642 | -0.0998 | -0.46502 | -4.16762 | -1.14678 | -1.18051 | -0.14743 | -0.84103 | -1.27006 | -1.80722 | -1.41014 | -1.39132 | -0.81063 | -0.96265 | -0.94883 |
| Q8R1K1 | Q8R1K1 | UBAC2_M | Ubac2 | Ubiquitin-associated domain-containing protein 2 | | | | 0.921406 | 0.382756 | 2.622306 | 0.225187 | 0.297196 | 0.625681 | 0.196775 | 0.169113 | 0.553285 | -0.13361 | -0.3927 | -0.71537 | -0.39621 | 0.12863 | -0.35152 | -0.57866 | -0.71317 | -1.30038 | -0.4056 | -0.46974 | -0.09138 |
| Q80X50 | Q80X50 | UBP2L_MC | Ubap2l | Ubiquitin-associated protein 2-like | | | | 0.719851 | 0.522811 | 1.949088 | 0.315826 | 0.500054 | 0.853591 | 0.271893 | 0.148096 | 0.74342 | -1.75762 | -1.34904 | -1.43994 | -1.33274 | -1.59126 | -1.24032 | -2.24996 | -1.90232 | -2.46976 | -1.30303 | -1.6582 | -1.46957 |
| Q8BGG7 | Q8BGG7 | UBS3B_MC | Ubash3b | Ubiquitin-associated and SH3 domain-containing protein B | | | | 0.804103 | -0.81706 | -2.21632 | 0.286905 | -0.26047 | -0.78108 | 0.335943 | 0.484512 | 0.904074 | -3.52758 | -2.93271 | -2.78629 | -2.68078 | -1.94514 | -1.59387 | -2.19527 | -2.63089 | -2.80234 | -2.14379 | -2.76034 | -1.74769 |
| P61079;P | P62838;Q | U2D2B_MC | Ube2d2;Ube2d2b;Ube2d3 | Ubiquitin-conjugating enzyme E2 D3 | + | | | 1.04867 | 0.457538 | 3.115969 | 0.551494 | -0.68259 | -1.46358 | 0.072752 | -0.03069 | -0.22077 | 1.957901 | 1.667614 | 1.46069 | 0.534632 | 1.940054 | 1.854653 | 1.332987 | 1.377453 | 1.736119 | 1.624388 | 2.060316 | 1.795763 |
| Q922J6 | Q922J6 | TSN2_MO | Tspan2 | Tetraspanin-2 | + | | | 2.05832 | -1.78893 | -10.6242 | 0.106616 | 0.46595 | 0.315409 | 0.188935 | 0.509528 | 0.533154 | -0.6537 | -0.50282 | -1.61398 | -3.59247 | -1.94515 | -0.83401 | 1.060132 | 1.361205 | -4.16634 | -1.97201 | -1.12149 | -2.67672 |
| P68037 | P68037 | UB2L3_MC | Ube2l3 | Ubiquitin-conjugating enzyme E2 L3 | | | | 0.01672 | -0.01994 | -0.05345 | 0.069699 | -0.04783 | -0.21203 | 0.028972 | 0.016229 | 0.091454 | -1.1522 | -1.33494 | -1.07031 | -1.49836 | -1.15417 | -1.25598 | -0.86203 | -1.58524 | -1.1653 | -1.30772 | -1.05131 | -1.39131 |
| P61082 | P61082 | UBC12_MC | Ube2m | NEDD8-conjugating enzyme Ubc12 | | | | 0.277935 | -0.26618 | -0.75858 | 0.341078 | -0.33655 | -0.91698 | 0.141085 | 0.192654 | 0.408281 | -0.21187 | -0.67493 | -0.47031 | -1.19648 | 0.016896 | 0.144346 | 0.086451 | -0.44088 | -0.44326 | -0.55043 | 0.35551 | -0.57958 |
| P61089 | P61089 | UBE2N_MC | Ube2n | Ubiquitin-conjugating enzyme E2 N | | | | 0.317953 | -0.2332 | -0.85892 | 0.12758 | -0.29016 | -0.37225 | 0.537856 | 0.411288 | 1.426546 | 1.872273 | 1.633383 | 2.163369 | 0.607316 | 2.348168 | 1.84055 | 2.22985 | 1.742211 | 1.627859 | 1.723147 | 1.819833 | 1.54631 |
| Q6ZPJ3 | Q6ZPJ3 | UBE2O_MC | Ube2o | (E3-independent) E2 ubiquitin-conjugating enzyme UBE2O | | | | 0.45007 | 0.680887 | 1.194424 | 0.972812 | 0.573138 | 2.814477 | 0.414048 | 0.319854 | 1.10174 | 0.035004 | -0.43993 | 0.520629 | 0.698279 | 0.287666 | -0.2494 | -0.36511 | -1.40159 | -0.14693 | 0.219562 | -0.41106 | -0.19038 |
| Q9CZY3;Q | Q9D2M8;( | UB2V1_MC | Ube2v1;Ube2v2 | Ubiquitin-conjugating enzyme E2 variant 1 | | | | 0.826016 | -0.77159 | -2.28898 | 0.164714 | -0.36711 | -0.47043 | 0.193095 | -0.16096 | -0.54384 | 0.141678 | 0.808184 | 1.539776 | 0.005385 | 0.878951 | 1.383897 | 1.195802 | 1.29725 | 1.282445 | 0.996927 | 1.446852 | 1.137925 |
| O08759 | O08759 | UBE3A_MC | Ube3a | Ubiquitin-protein ligase E3A | | | | 0.84748 | 0.481677 | 2.361511 | 0.377242 | -0.10767 | -1.00815 | 0.86782 | 0.405877 | 2.431533 | -0.03599 | -0.43601 | -0.12335 | -0.33651 | 0.39213 | 0.069166 | -0.75766 | -0.67769 | -0.11539 | -0.12912 | -0.13296 | -0.2175 |
| Q9ES34 | Q9ES34 | UBE3B_MC | Ube3b | Ubiquitin-protein ligase E3B | | | | 0.829813 | 0.945411 | 2.30171 | 0.261682 | 0.278525 | 0.717768 | 0.384814 | -0.44869 | -1.02733 | -0.30978 | -0.70894 | -0.68177 | -0.13468 | -1.43805 | -0.80115 | -1.09577 | -1.81376 | -0.41152 | -0.96197 | -0.96982 | -0.372 |
| Q80U95 | Q80U95 | UBE3C_MC | Ube3c | Ubiquitin-protein ligase E3C | | | | 0.34782 | 0.293407 | 0.933933 | 0.973393 | 0.627884 | 2.816702 | 0.052115 | -0.05068 | -0.16095 | -0.2602 | -0.87031 | 0.27231 | 0.470724 | -0.47009 | -0.1338 | -0.93375 | -0.78357 | -0.45599 | -0.05675 | -0.51748 | 0.014948 |
| E9Q735 | E9Q735 | UBE4A_MC | Ube4a | Ubiquitin conjugation factor E4 A | | | + | 0.670805 | -0.86697 | -1.8016 | 0.937715 | -0.41613 | -2.68227 | 1.66024 | 0.616178 | 6.651287 | -1.81589 | -2.71381 | -2.19554 | -2.3561 | -1.41583 | -1.23096 | -1.22463 | -1.57113 | -1.72693 | -1.99244 | -1.94576 | -1.93339 |
| Q9ES00 | Q9ES00 | UBE4B_MC | Ube4b | Ubiquitin conjugation factor E4 B | | | | NaN | 0 | NaN | 0.128488 | -0.25294 | -0.37468 | 0.100407 | -0.31744 | -0.29834 | -3.79965 | -3.11528 | -1.70693 | -2.93097 | -1.61682 | -3.21671 | -3.59104 | -3.75132 | -1.78113 | -2.35089 | -2.8009 | -1.39775 |
| P21126 | P21126 | UBL4A_MC | Ubl4a | Ubiquitin-like protein 4A | | + | | 0.255575 | 0.204585 | 0.702407 | 1.36572 | -0.4964 | -4.66084 | 0.291553 | -0.24047 | -0.79274 | -0.75664 | -0.17437 | 0.68507 | 0.563135 | -0.07908 | 0.524821 | -0.66143 | -0.67874 | 1.033175 | 1.207832 | 0.434393 | 0.492277 |
| Q9QZM0 | Q9QZM0 | UBQL2_MC | Ubqln2 | Ubiquilin-2 | | | | 0.061392 | -0.18228 | -0.18807 | 0.124835 | -0.52 | -0.36487 | 0.849252 | -0.91715 | -2.36756 | -2.78585 | -3.52296 | -0.97136 | -3.80269 | -2.91555 | -3.65746 | -3.86853 | -2.07572 | -2.03132 | -1.70272 | -2.48096 | -2.25773 |
| Q5U430 | Q5U430 | UBR3_MO | Ubr3 | E3 ubiquitin-protein ligase UBR3 | | | | 0.007343 | 0.015865 | 0.023714 | 0.225956 | 0.337386 | 0.62763 | 0.11794 | -0.32029 | -0.34626 | -2.98788 | -2.9493 | -2.06764 | -1.66182 | -1.76457 | -3.55716 | -3.65321 | -2.3157 | -2.69991 | -1.70433 | -2.1119 | -2.56926 |
| A2AN08 | A2AN08 | UBR4_MO | Ubr4 | E3 ubiquitin-protein ligase UBR4 | | | | 0.778561 | 0.507466 | 2.133325 | 0.75955 | 0.168619 | 2.072696 | 0.153964 | -0.107 | -0.44229 | 0.215827 | 0.070334 | 0.831906 | 0.990301 | 0.080227 | 0.282837 | -0.13791 | -0.59086 | 0.723883 | 0.761085 | 0.068839 | 0.508226 |
| Q9JHI5 | Q9JHI5 | IVD_MOUS | Ivd | Isovaleryl-CoA dehydrogenase, mitochondrial | + | | | 1.460333 | -1.8083 | -5.23175 | 0.374996 | -1.44985 | -1.00247 | 0.130828 | -0.27425 | -0.38095 | -1.16976 | -0.51378 | -0.72931 | -3.60918 | -0.79799 | 0.402795 | 0.857482 | 1.07558 | -0.85476 | -0.58402 | 0.473919 | -0.3206 |
| P58321;Q | P58321;Q | UCHL3_MC | Uchl3;Uchl4 | Ubiquitin carboxyl-terminal hydrolase isozyme L4 | | | | NaN | 0 | NaN | 0.125763 | 0.263787 | 0.367364 | 0.040946 | 0.040466 | 0.127759 | -3.83837 | -3.51114 | -1.72651 | -3.0134 | -2.83347 | -2.24902 | -3.4138 | -2.85568 | -2.95245 | -2.31502 | -2.45953 | -2.7039 |
| P52623 | P52623;Q | UCK1_MO | Uck1 | Uridine-cytidine kinase 1 | | | + | 0.39963 | 1.390959 | 1.064961 | 0.574958 | -0.55429 | -1.52799 | 1.927092 | -0.44826 | -9.11311 | -1.19288 | -1.23801 | -1.12699 | -1.39999 | -1.33864 | -1.38557 | -1.30049 | -3.91232 | -1.0453 | -0.3731 | -0.87061 | -0.95707 |
| Q91YL3 | Q91YL3 | UCKL1_MC | Uckl1 | Uridine-cytidine kinase-like 1 | | + | | 0.504058 | 1.561881 | 1.335977 | 1.002971 | -0.31427 | -2.93168 | 0.951703 | -0.35716 | -2.73443 | -3.01218 | -0.68601 | -0.34313 | -0.13359 | -0.5026 | -0.76371 | -3.52933 | -3.29262 | 0.098605 | 0.053218 | -0.27986 | -0.27214 |
| Q9CR09 | Q9CR09 | UFC1_MO | Ufc1 | Ubiquitin-fold modifier-conjugating enzyme 1 | | + | | 0.584644 | -0.49665 | -1.55485 | 1.235023 | -0.54332 | -3.96169 | 0.710992 | -0.52938 | -1.92203 | 0.773981 | 0.136614 | -0.22459 | 0.030746 | 0.40349 | -0.05173 | 0.930268 | 0.973626 | 0.396307 | 0.496485 | 0.860353 | 0.550167 |
| P70362 | P70362 | UFD1_MO | Ufd1 | Ubiquitin recognition factor in ER-associated degradation protein 1 | | + | | NaN | 0 | NaN | 1.029111 | 1.102719 | 3.036083 | 0.064574 | 0.269781 | 0.197283 | -3.20224 | -3.76009 | -2.68375 | -1.95763 | -0.97406 | -3.22369 | -3.49386 | -3.11456 | -3.43359 | -3.41323 | -1.59097 | -3.14634 |
| P61961 | P61961 | UFM1_MO | Ufm1 | Ubiquitin-fold modifier 1 | | | | 0.286445 | -0.75405 | -0.77993 | 0.339668 | 1.3355 | 0.913435 | 0.015688 | -0.04304 | -0.0502 | -1.83113 | -3.67738 | -0.68311 | -1.53738 | -0.82018 | -0.50284 | -2.28755 | -1.71287 | -3.84402 | -1.04747 | 0.223982 | -1.46093 |
| O09117 | O09117 | SYPL1_MC | Sypl1 | Synaptophysin-like protein 1 | + | | | 1.519537 | -1.82166 | -5.6201 | 0.224612 | -0.99121 | -0.62422 | 0.094671 | -0.60446 | -0.28247 | -0.54336 | -0.0585 | -0.8582 | -3.95219 | -0.70541 | -0.13557 | 1.735877 | 1.305574 | -1.05585 | -1.77213 | -0.13564 | -2.49643 |
| Q91ZJ5 | Q91ZJ5 | UGPA_MO | Ugp2 | UTP--glucose-1-phosphate uridylyltransferase | | | | 0.095065 | 0.143386 | 0.28356 | 0.434387 | 0.330061 | 1.153919 | 0.415656 | 0.825199 | 1.105852 | -0.22343 | 0.188671 | 0.117708 | -0.44634 | 1.531229 | 0.047504 | 0.301013 | -0.62254 | -0.54211 | -0.44664 | 0.044615 | -0.11628 |
| Q4KUS2 | Q4KUS2 | UN13A_MC | Unc13a | Protein unc-13 homolog A | | | | 0.60643 | 0.441148 | 1.615878 | 0.989656 | -0.28269 | -2.87952 | 0.52889 | -0.36666 | -1.40236 | 2.07119 | 2.224206 | 3.619676 | 3.809813 | 3.127439 | 3.454875 | 1.968619 | 1.444482 | 3.972942 | 4.021924 | 3.45396 | 3.861672 |
| Q8K1S4 | Q8K1S4 | UNC5A_M | Unc5a | Netrin receptor UNC5A | | | | 0.632815 | -1.864 | -1.69099 | 0.547367 | -0.50428 | -1.45234 | 0.932892 | -0.62739 | -2.66444 | -3.1304 | -1.22431 | -0.97829 | -0.28394 | -0.40339 | -0.12133 | 0.240539 | -0.86725 | -0.12142 | -0.13225 | 0.176468 | 0.553595 |
| Q0KK59 | Q0KK59 | UNC79_M | Unc79 | Protein unc-79 homolog | | | | 0.537216 | 0.43602 | 1.424815 | 0.039057 | 0.031978 | 0.122084 | 0.658065 | -0.79313 | -1.76416 | 0.082663 | -0.00107 | 0.592172 | 1.112063 | -0.40979 | 0.398861 | -0.09208 | -0.69837 | 0.852349 | 0.787931 | 0.591093 | 0.98424 |
| Q8BLN6 | Q8BLN6 | UNC80_M | Unc80 | Protein unc-80 homolog | | | | 0.505627 | 0.435929 | 1.340146 | 0.053061 | -0.04537 | -0.16373 | 0.894339 | -1.09826 | -2.52477 | -0.10355 | -0.49425 | 0.356798 | 0.907223 | -0.86775 | -0.13497 | -0.47474 | -0.99492 | 0.709519 | 0.645236 | 0.362427 | 0.831379 |
| P40142 | P40142 | TKT_MOUS | Tkt | Transketolase | + | | | 1.340642 | -1.82243 | -4.51903 | 0.283617 | -0.56046 | -0.77284 | 0.062844 | -0.1807 | -0.19228 | 0.528846 | 1.198971 | 1.629911 | 0.189885 | 0.592993 | 1.936573 | 2.91076 | 2.461911 | 1.556893 | 1.38382 | 2.102668 | 0.788298 |
| A2AT37 | A2AT37 | RENT2_MC | Upf2 | Regulator of nonsense transcripts 2 | | | | 0.370524 | 0.790208 | 0.991166 | 0.019703 | 0.043401 | 0.062787 | 0.342417 | -0.30792 | -0.92034 | -3.1934 | -1.9802 | -1.16861 | -0.77635 | -1.66759 | -1.18235 | -3.89436 | -2.85966 | -1.67872 | -0.35304 | -1.34742 | -0.88667 |
| Q3ULL6 | Q3ULL6 | Q3ULL6_M | Upf3b | UPF3 regulator of nonsense transcripts homolog B (yeast) | | | | NaN | 0 | NaN | 0.635166 | -1.66743 | -1.69775 | 0.089125 | -0.29848 | -0.26702 | -3.49655 | -3.59252 | -4.83718 | -3.06812 | -3.76997 | -2.28969 | -3.41257 | -3.26521 | -1.85837 | -2.71208 | -1.89363 | -3.56907 |
| B1AVZ0 | B1AVZ0 | UPP_MOU | Uprt | Uracil phosphoribosyltransferase homolog | | | | 0.382738 | 0.524649 | 1.022066 | 0.118584 | -0.15903 | -0.348 | 0.403906 | 0.536301 | 1.075851 | -2.25286 | -1.22747 | -1.39923 | -1.94701 | -1.16879 | -2.07564 | -2.23951 | -2.29012 | -1.8799 | -1.14828 | -1.9514 | -2.36563 |
| Q9CWU6 | Q9CWU6 | UQCC1_M | Uqcc1 | Ubiquinol-cytochrome-c reductase complex assembly factor 1 | | | | 0.534133 | 0.347275 | 1.416485 | 0.00551 | -0.01193 | -0.01783 | 0.256735 | 1.064978 | 0.705326 | -2.72577 | -2.50601 | -3.17113 | -3.06117 | -1.15477 | -1.67082 | -3.18233 | -2.744 | -2.43771 | -3.77074 | -0.99008 | -3.96547 |
| Q8R1I1 | Q8R1I1 | QCR9_MO | Uqcr10 | Cytochrome b-c1 complex subunit 9 | | | | 0.204031 | -0.21016 | -0.57185 | 0.005092 | -0.01359 | -0.01649 | 0.11114 | 0.234989 | 0.327775 | 0.237885 | 0.963946 | 0.78874 | -0.82085 | 0.509624 | 1.256054 | 0.868283 | 0.753869 | -0.18072 | 0.175793 | 1.259967 | 0.035732 |
| Q61137 | Q61137 | ASTN1_MC | Astn1 | Astrotactin-1 | + | | | 1.452303 | -1.8328 | -5.18099 | 0.370465 | -0.09828 | -0.99102 | 0.011825 | -0.01312 | -0.038 | -3.72587 | -4.33243 | -2.24154 | -3.43848 | 0.063682 | -0.61708 | -2.01424 | -2.37846 | -0.22999 | -0.25348 | -0.32141 | -0.20575 |
| Q9CZ13 | Q9CZ13 | QCR1_MO | Uqcrc1 | Cytochrome b-c1 complex subunit 1, mitochondrial | + | | | 1.298195 | -0.5301 | -4.28751 | 0.356652 | -0.6266 | -0.95617 | 0.051323 | 0.093315 | 0.158617 | 2.793523 | 3.03478 | 2.0301 | 0.767816 | 2.461813 | 3.213772 | 3.471365 | 3.417138 | 2.201959 | 1.849166 | 3.196963 | 2.291991 |
| Q9CR11 | Q9CR11 | YETS4_MC | Yeats4 | YEATS domain-containing protein 4 | + | | | 2.482176 | -1.84016 | -17.385 | 0.183049 | -0.29398 | -0.51799 | 0.494218 | 1.593436 | 1.309907 | -2.87101 | -2.90728 | -0.28042 | -1.1864 | -0.07883 | -0.60667 | -0.94466 | -1.1533 | -0.09751 | -0.78135 | -3.12366 | -0.74871 |
| Q9CZU6 | Q80X68;Q | CISY_MOL | Cs | Citrate synthase, mitochondrial | + | | | 1.963148 | -1.86669 | -9.50618 | 0.25483 | -0.72675 | -0.70053 | 0.057433 | -0.23598 | -0.17654 | 3.554266 | 3.935469 | 4.246431 | 2.254507 | 3.346101 | 5.476266 | 5.564329 | 5.658794 | 4.267555 | 3.686875 | 5.45482 | 3.839507 |
| P99028 | P99028 | QCR6_MO | Uqcrh | Cytochrome b-c1 complex subunit 6, mitochondrial | + | | | 1.132855 | -1.29288 | -3.47818 | 0.039668 | -0.07656 | -0.12392 | 0.090602 | 0.178345 | 0.271141 | 0.893956 | 1.4341 | 1.654846 | 0.425544 | 1.304061 | 1.873927 | 2.201505 | 2.712312 | 1.17955 | 1.053969 | 2.003489 | 0.817808 |
| P00405 | P00405 | COX2_MO | Mtco2 | Cytochrome c oxidase subunit 2 | + | | | 1.919877 | -1.89643 | -9.03636 | 0.477228 | -0.55018 | -1.26519 | 0.026147 | 0.093691 | 0.082774 | 4.478471 | 4.297213 | 4.447646 | 3.709146 | 4.017063 | 5.596855 | 6.473564 | 6.094985 | 4.858254 | 4.398889 | 5.523967 | 3.90257 |
| Q5NCI0 | Q5NCI0 | URGCP_M | Urgcp | Up-regulator of cell proliferation | | | | NaN | 0 | NaN | 0.059226 | -0.13438 | -0.18177 | 0.071281 | 0.061738 | 0.216562 | -3.74827 | -3.6453 | -3.03146 | -3.09582 | -2.36171 | -1.94697 | -3.02171 | -3.96713 | -3.66784 | -2.19068 | -2.41171 | -2.02045 |
| Q9Z1Z0 | Q9Z1Z0 | USO1_MO | Uso1 | General vesicular transport factor p115 | | | | 0.249193 | 0.207852 | 0.686339 | 0.03906 | -0.02154 | -0.12209 | 0.503634 | 0.582524 | 1.334851 | -0.86022 | -0.45721 | -0.36227 | -0.70589 | 0.512025 | -0.29843 | -1.09264 | -0.6405 | -0.55261 | -0.47248 | -0.31375 | -0.63769 |
| P52479 | P52479 | UBP10_MC | Usp10 | Ubiquitin carboxyl-terminal hydrolase 10 | + | | | 1.228772 | 0.503899 | 3.930609 | 0.895617 | -1.03442 | -2.52932 | 0.011074 | 0.013327 | 0.035616 | -2.87422 | -2.64922 | -3.63311 | -2.84215 | -1.6707 | -2.30561 | -3.20414 | -3.32709 | -2.3101 | -2.09432 | -1.80341 | -2.19956 |
| Q99K46 | Q99K46 | UBP11_MC | Usp11 | Ubiquitin carboxyl-terminal hydrolase 11 | | | | 0.55777 | 0.401739 | 1.480713 | 0.659337 | -0.31086 | -1.76789 | 0.690644 | -0.47457 | -1.86059 | -0.82758 | -0.91014 | 0.04004 | 0.180424 | -0.67787 | -0.60961 | -1.53876 | -1.00245 | 0.259871 | 0.582305 | -0.42194 | 0.083604 |
| Q9JMA1 | Q9JMA1 | UBP14_MC | Usp14 | Ubiquitin carboxyl-terminal hydrolase 14 | | | | 0.816643 | -0.8553 | -2.25773 | 0.204449 | 0.175043 | 0.572918 | 0.025072 | -0.03323 | -0.07946 | -1.75436 | -1.05051 | -0.60173 | -0.86947 | -0.98272 | -0.60928 | -0.40692 | -0.68735 | -0.636 | -1.18528 | -0.38859 | -1.13696 |
| Q8R5H1 | Q8R5H1 | UBP15_MC | Usp15 | Ubiquitin carboxyl-terminal hydrolase 15 | | | | 0.296021 | 0.274646 | 0.80394 | 0.149777 | 0.036332 | 0.431267 | 0.175114 | -0.09358 | -0.49747 | -1.07942 | -0.6818 | 0.053098 | 0.026999 | -0.4293 | -0.67514 | -0.87744 | -1.43307 | -0.07951 | 0.086944 | -0.60104 | -0.31623 |
| Q3UJD6 | Q3UJD6 | UBP19_MC | Usp19 | Ubiquitin carboxyl-terminal hydrolase 19 | | | | 0.154305 | -0.4321 | -0.44318 | 0.767719 | 0.436237 | 2.098632 | 0.124887 | -0.07864 | -0.36501 | -3.24302 | -3.64247 | -0.84779 | -0.99644 | -1.29214 | -1.61005 | -3.96497 | -2.05631 | -1.16422 | -1.55248 | -1.51789 | -1.22703 |
| B1AY13 | B1AY13 | UBP24_MC | Usp24 | Ubiquitin carboxyl-terminal hydrolase 24 | | | | 0.493339 | 0.807575 | 1.307586 | 0.07782 | 0.016739 | 0.23519 | 0.509315 | -0.16179 | -1.34996 | -1.06935 | -0.52061 | -0.38666 | -0.39683 | -0.75595 | -0.5219 | -2.15587 | -1.04924 | -0.47948 | -0.33749 | -0.503 | -0.45128 |
| Q9JL62 | Q9JL62 | GLTP_MO | Gltp | Glycolipid transfer protein | + | | + | 2.133568 | -1.89837 | -11.5979 | 0.032686 | -0.15101 | -0.1028 | 1.169273 | -1.85268 | -3.6447 | -0.97048 | -0.88919 | -1.39169 | -3.78835 | -0.3809 | -2.82713 | 0.809978 | 1.127088 | -3.28869 | -1.58932 | -1.3957 | -1.55698 |
| P35123 | P35123 | UBP4_MO | Usp4 | Ubiquitin carboxyl-terminal hydrolase 4 | | | + | 0.417321 | 1.113757 | 1.110111 | 0.069221 | -0.08828 | -0.21066 | 1.154389 | -0.64445 | -3.57589 | -1.08678 | -1.60091 | -0.55235 | -0.48466 | -1.01284 | -1.1501 | -1.3474 | -1.48781 | -0.84793 | -0.01251 | -0.60366 | -0.27037 |
| P62069 | Q9D9M2;F | UBP46_MC | Usp46 | Ubiquitin carboxyl-terminal hydrolase 46 | | | | 0.406906 | 1.625615 | 1.083499 | 0.241359 | -0.23728 | -0.66658 | 0.030771 | 0.011232 | 0.096962 | -0.18295 | -0.25275 | 0.85099 | 0.81268 | 0.916642 | 0.721444 | -0.34353 | -0.3434 | 0.713667 | 1.424559 | 0.745414 | 0.870208 |
| Q9WUC3 | Q9WUC3 | LY6H_MO | Ly6h | Lymphocyte antigen 6H | + | | | 1.369037 | -1.97932 | -4.67988 | 0.663695 | 0.681085 | 1.780666 | 0.057574 | 0.07192 | 0.176954 | -3.21248 | -3.39091 | -0.86628 | -1.35133 | -0.65657 | -0.91592 | -1.7358 | -0.90895 | -1.49412 | -2.08566 | -0.47297 | -1.24335 |
| P56399 | P56399 | UBP5_MO | Usp5 | Ubiquitin carboxyl-terminal hydrolase 5 | | | | 0.004375 | -0.00175 | -0.01417 | 0.011934 | 0.008106 | 0.038348 | 0.024268 | -0.00497 | -0.07697 | 2.329586 | 2.135191 | 2.851355 | 2.761344 | 2.977869 | 2.864866 | 2.310619 | 2.157664 | 2.591721 | 3.004767 | 2.895004 | 2.957677 |
| Q6A4J8 | Q6A4J8 | UBP7_MO | Usp7 | Ubiquitin carboxyl-terminal hydrolase 7 | | | | 0.952776 | 0.343833 | 2.738464 | 0.359655 | -0.07931 | -0.96374 | 0.565934 | -0.25603 | -1.50311 | 0.175155 | 0.193285 | 0.790374 | 0.95474 | 0.743324 | 0.402689 | -0.03438 | -0.28484 | 0.947567 | 0.956168 | 0.826656 | 0.831418 |
| P70398 | F8VPU6;P | USP9X_MC | Usp9x | Probable ubiquitin carboxyl-terminal hydrolase FAF-X | + | | | 1.171455 | 0.524223 | 3.65488 | 0.68504 | 0.155572 | 1.843839 | 0.092472 | -0.07464 | -0.27635 | 0.813865 | 0.884627 | 1.573891 | 1.662883 | 0.886709 | 1.135919 | 0.464022 | 0.186024 | 1.391128 | 1.534503 | 0.846326 | 1.325581 |
| E9Q6R7 | E9Q6R7 | E9Q6R7_M | Utrn | Utrophin | | | | 0.670865 | 0.636466 | 1.801774 | 0.705323 | 0.211424 | 1.90482 | 0.144209 | 0.075672 | 0.416561 | -1.40305 | -1.31291 | -1.14136 | -1.10478 | -1.9248 | -1.60927 | -1.64409 | -2.3448 | -1.44397 | -1.22501 | -1.93277 | -1.75265 |
| P05202 | P05202 | AATM_MO | Got2 | Aspartate aminotransferase, mitochondrial | + | | | 2.064422 | -2.07857 | -10.7001 | 0.305338 | -0.70773 | -0.8273 | 0.057911 | -0.19573 | -0.17794 | 2.642913 | 3.003307 | 3.702104 | 1.999832 | 2.815234 | 4.688999 | 4.829124 | 4.974245 | 3.644745 | 3.472652 | 4.524204 | 3.371483 |
| G5E8G3 | G5E8G3 | G5E8G3_M | Opcml | Opioid binding protein/cell adhesion molecule-like | + | | | 1.790132 | -2.15152 | -7.75773 | 0.01483 | 0.070876 | 0.047502 | 0.098295 | 0.336531 | 0.292507 | -3.82504 | -3.36117 | -0.72323 | -3.44861 | -1.36018 | 0.120203 | -1.28953 | -1.59364 | -2.76452 | -1.54908 | -0.07574 | -1.8373 |
| P63044 | Q62442;P | VAMP2_M | Vamp2 | Vesicle-associated membrane protein 2 | | | | 0.084656 | -0.02307 | -0.25449 | 0.450031 | 1.014796 | 1.194324 | 0.194732 | 0.377364 | 0.548046 | 0.113116 | 0.221014 | 1.939208 | 0.24272 | 1.343278 | 0.655811 | 0.262984 | 0.117284 | 0.026753 | 0.125583 | 1.21881 | 0.02555 |
| Q9Z2P8 | Q9Z2P8 | VAMP5_M | Vamp5 | Vesicle-associated membrane protein 5 | | | | 0.358689 | -3.06047 | -0.9613 | 0.236366 | -2.22016 | -0.65397 | 0.408367 | 2.797921 | 1.087227 | -4.18271 | -3.18989 | -2.66561 | -3.9561 | 2.085849 | -3.01248 | 2.5189 | -3.77056 | -4.42369 | 2.242302 | -2.90855 | -3.61392 |
| Q9WV55 | Q9WV55 | VAPA_MO | Vapa | Vesicle-associated membrane protein-associated protein A | | + | | 0.751205 | 0.431904 | 2.046385 | 1.527847 | -0.39705 | -5.67665 | 0.015507 | -0.00877 | -0.04964 | 0.27844 | 0.270148 | 0.681184 | 0.621575 | 0.499654 | 0.84319 | -0.36863 | 0.053406 | 1.111708 | 0.985155 | 0.638178 | 0.722413 |
| Q9QY76 | Q9QY76 | VAPB_MO | Vapb | Vesicle-associated membrane protein-associated protein B | | | | 0.423552 | 0.353111 | 1.126075 | 0.025305 | 0.010536 | 0.080176 | 0.848418 | -0.20571 | -2.36471 | 1.413188 | 1.472432 | 1.70306 | 1.451142 | 1.25985 | 1.432685 | 1.401874 | 0.777524 | 1.529087 | 1.604043 | 1.56195 | 1.542005 |
| Q9Z1Q9 | Q9Z1Q9 | SYVC_MO | Vars1 | Valine--tRNA ligase | | | | 0.863795 | 0.198137 | 2.417573 | 0.299733 | 0.198222 | 0.813244 | 0.524544 | 0.278136 | 1.390675 | -0.66912 | -0.72091 | -0.43857 | -0.30742 | -0.30481 | -0.29695 | -0.81539 | -0.80597 | -0.33646 | -0.77898 | -0.37905 |
| Q62465 | Q62465 | VAT1_MO | Vat1 | Synaptic vesicle membrane protein VAT-1 homolog | + | | | 1.210418 | -0.68222 | -3.84052 | 0.183493 | -0.37822 | -0.51913 | 0.570213 | 0.295216 | 1.514888 | 0.709083 | 0.847388 | 0.442677 | -0.96285 | 0.250856 | 0.539606 | 1.624076 | 1.296829 | -0.07403 | 0.310298 | 0.230907 | -0.03088 |
| Q80TB8 | Q80TB8 | VAT1L_MC | Vat1l | Synaptic vesicle membrane protein VAT-1 homolog-like | | | | 0.807124 | -0.54835 | -2.22625 | 0.30261 | -0.23042 | -0.82046 | 0.219052 | 0.171868 | 0.610114 | 0.102123 | 0.345554 | -0.49558 | -0.90425 | -0.99889 | -0.45271 | 0.986318 | 0.55805 | -0.27682 | -0.66216 | -0.96677 | -0.82857 |
| Q99K85 | Q99K85 | SERC_MO | Psat1 | Phosphoserine aminotransferase | + | | | 1.394047 | -2.28144 | -4.82568 | 0.35422 | -0.9595 | -0.95004 | 0.085229 | 0.185228 | 0.256098 | -1.81553 | -0.87137 | -0.25784 | -2.26709 | -0.73682 | 0.334849 | 0.912395 | 0.963589 | -0.19929 | -0.40662 | 0.099589 | -0.87201 |
| P61759 | P61759 | PFD3_MO | Vbp1 | Prefoldin subunit 3 | | | | 0.066479 | -0.05852 | -0.20278 | 0.420809 | -0.16838 | -1.11904 | 0.189634 | -0.18775 | -0.53495 | -0.89815 | -0.68217 | -0.30942 | -0.49053 | -0.65821 | 0.040938 | -0.46399 | -0.99928 | -0.35177 | -0.11143 | -0.15207 | -0.08971 |
| P29533 | P29533 | VCAM1_M | Vcam1 | Vascular cell adhesion protein 1 | | | | 0.12795 | 0.252728 | 0.373238 | 0.013249 | 0.053273 | 0.042512 | 0.30732 | 0.404305 | 0.832263 | -2.80588 | -2.85511 | -1.91146 | -3.21511 | -1.26277 | -2.2337 | -3.7599 | -2.40655 | -3.68682 | -1.54629 | -2.17038 | -2.13471 |
| Q62059 | Q62059 | CSPG2_M | Vcan | Versican core protein | | | | 0.733217 | 0.833089 | 1.990268 | 0.382099 | -0.21317 | -1.02045 | 0.860279 | 0.670618 | 2.405423 | -0.18114 | 0.136854 | -0.72796 | -1.08784 | 0.057927 | -0.20503 | -0.46803 | -1.24244 | -0.80084 | -0.58862 | -0.49832 | -0.99001 |
| Q64727 | Q64727 | VINC_MO | Vcl | Vinculin | | | | 0.005089 | 0.030247 | 0.016477 | 0.407633 | 0.384717 | 1.085356 | 0.412241 | 0.336408 | 1.097121 | -4.03524 | -1.22497 | -2.84881 | -2.80885 | -2.64974 | -2.64849 | -1.47903 | -3.84167 | -2.85965 | -3.56744 | -2.67889 | -3.29215 |
| Q01853 | Q01853 | TERA_MO | Vcp | Transitional endoplasmic reticulum ATPase | | | | 0.763547 | -0.44391 | -2.08536 | 2.32E-05 | 4.51E-05 | 7.56E-05 | 0.618887 | 0.551122 | 1.65117 | 0.229308 | 0.383722 | 1.332101 | 0.304984 | 1.431403 | 0.832377 | 0.948798 | 0.55205 | 0.516101 | 1.120894 | 0.728073 | 0.433462 |
| Q8CDG3 | Q8CDG3 | VCIP1_MC | Vcpip1 | Deubiquitinating protein VCPIP1 | | | | 0.130074 | -0.47117 | -0.37893 | 0.037424 | 0.033623 | 0.117164 | 0.863134 | 0.266634 | 2.415287 | -3.02679 | -2.88077 | -1.10607 | -0.77699 | -0.50518 | -0.67553 | -1.24134 | -3.72387 | -1.21027 | -0.74003 | -0.78676 | -0.92722 |
| Q8VE37 | Q8VE37 | RCC1_MO | Rcc1 | Regulator of chromosome condensation | + | + | | 2.457722 | -2.41853 | -16.8937 | 1.420645 | 1.231607 | 4.985121 | 0.311482 | -0.60955 | -0.8427 | -3.54144 | -3.72507 | -0.40581 | -0.02127 | -3.1935 | -1.82756 | -1.10488 | -1.32457 | -1.29 | -1.60029 | -1.66273 | -2.13923 |
| Q80TF3 | Q80TF3 | PCD19_M | Pcdh19 | Protocadherin-19 | + | | | 2.366286 | -2.71648 | -15.1963 | 0.519936 | -0.83351 | -1.37832 | 0.570365 | -0.59049 | -1.51531 | -2.90568 | -3.09984 | -1.63001 | -0.42207 | -0.33743 | -1.07779 | -0.43639 | -0.13618 | -0.22288 | -0.16217 | 0.004616 | -0.23885 |
| Q9CQI3 | Q9CQI3 | GMFB_MO | Gmfb | Glia maturation factor beta | + | + | | 2.15694 | -2.79153 | -11.9177 | 1.158968 | 0.923347 | 3.596951 | 0.012802 | 0.025334 | 0.041095 | -3.28632 | -3.29136 | 0.261494 | -0.16922 | -0.40339 | 0.080073 | -0.73153 | -0.26309 | -1.01691 | -0.73751 | 0.380107 | -0.7541 |
| Q0VGU4 | Q0VGU4 | VGF_MOU | Vgf | Neurosecretory protein VGF | + | + | | 1.274902 | 1.087767 | 4.164839 | 2.257427 | 2.259737 | 13.3939 | 0.98668 | 1.35723 | 2.86795 | 0.315428 | 0.241857 | 1.813269 | 1.793259 | 1.347486 | 0.425899 | -0.55055 | -1.0677 | -0.28806 | -0.62489 | -0.57836 | -0.36271 |
| P20152 | P20152 | VIME_MO | Vim | Vimentin | | | | 0.062885 | 0.139682 | 0.192397 | 0.272927 | -0.1936 | -0.74601 | 0.673415 | 0.564007 | 1.809307 | 0.766737 | 1.297798 | 1.533186 | 1.052109 | 2.753258 | 3.120199 | 1.568293 | 0.216878 | 1.583667 | 1.388834 | 2.120708 | 2.624736 |
| Q8BGQ1 | Q8BGQ1 | SPE39_MC | Vipas39 | Spermatogenesis-defective protein 39 homolog | | | | 0.106863 | -0.1493 | -0.31609 | 0.437821 | -0.62473 | -1.16277 | 0.27591 | 0.051359 | 0.753503 | -1.68281 | -1.64384 | -0.72121 | -1.78113 | -0.20062 | -0.31479 | -1.0421 | -1.98596 | -0.71481 | -0.53807 | -0.27182 | -0.34631 |
| Q9CRC0 | Q9CRC0 | VKOR1_MC | Vkorc1 | Vitamin K epoxide reductase complex subunit 1 | + | | | 1.121215 | 0.455104 | 3.426247 | 0.214746 | 0.266208 | 0.599165 | 0.223888 | 0.230496 | 0.622388 | 1.248816 | 1.316946 | -0.31559 | -0.66466 | 0.298683 | -0.11236 | 0.956163 | 0.699391 | -0.34775 | -1.16491 | -0.44541 | 0.170742 |
| Q6TEK5 | Q6TEK5 | VKORL_MC | Vkorc1l1 | Vitamin K epoxide reductase complex subunit 1-like protein 1 | | | | 0.547634 | 0.626708 | 1.453063 | 0.070195 | -0.10192 | -0.21345 | 0.252986 | -0.19427 | -0.69589 | -0.17311 | -0.5558 | -0.79865 | -1.72536 | -0.80661 | -1.2081 | -0.60463 | -1.3777 | -1.04472 | -1.27544 | -0.61899 | -1.00709 |
| Q78T54 | Q78T54 | VMA21_M | Vma21 | Vacuolar ATPase assembly integral membrane protein Vma21 | | | | 0.594484 | 0.800434 | 1.582307 | 0.711249 | 0.141796 | 1.922818 | 0.168071 | -0.12939 | -0.47917 | 0.510169 | 1.428649 | -0.23201 | -0.33089 | -0.28146 | -0.2261 | 0.381102 | -0.04315 | -0.36853 | -0.47796 | 0.144219 | -0.39299 |
| Q99KU0 | Q99KU0 | VMP1_MO | Vmp1 | Vacuole membrane protein 1 | | | | 0.019065 | 0.036037 | 0.057097 | 0.286775 | 0.190906 | 0.780758 | 0.516973 | 0.190434 | 1.37039 | -0.51263 | 0.606879 | -0.68503 | -1.04704 | -0.72373 | -0.52321 | -0.18392 | 0.206094 | -0.89255 | -1.22133 | -0.91012 | -0.71769 |
| Q91W86 | Q91W86 | VPS11_MC | Vps11 | Vacuolar protein sorting-associated protein 11 homolog | | | | 0.250954 | -0.79179 | -0.69077 | 0.744275 | -0.33005 | -2.02467 | 0.6676 | -0.36398 | -1.79215 | -1.09557 | -1.37643 | -0.68324 | -1.00301 | -0.79474 | -0.99187 | -1.32892 | -1.55949 | -0.54489 | -0.48126 | -0.7069 | -0.35175 |
| Q5H8C4 | Q5H8C4 | VP13A_MC | Vps13a | Intermembrane lipid transfer protein VPS13A | | | | 0.667163 | 0.945047 | 1.790862 | 0.376286 | 0.007509 | 1.005732 | 0.106116 | -0.10477 | -0.31404 | -0.77308 | -1.18421 | -0.30426 | -0.29099 | -0.57786 | -0.74802 | -1.43767 | -2.40971 | -0.30171 | -0.30856 | -0.88076 | -0.23557 |
| Q8BX70 | Q8BX70 | VP13C_MC | Vps13c | Intermembrane lipid transfer protein VPS13C | | | | 0.778755 | 0.615001 | 2.133949 | 0.008324 | -0.00621 | -0.02685 | 0.388688 | -0.39575 | -1.03715 | -0.53387 | -0.31522 | -0.12901 | -0.17922 | -0.25939 | 0.213472 | -0.77289 | -1.3062 | -0.37794 | 0.082134 | 0.073295 | 0.67228 |
| B1ART2 | B1ART2 | B1ART2_M | Vps13d | Vacuolar protein sorting 13D | | | | 0.416964 | 1.339314 | 1.109197 | 0.111816 | 0.145758 | 0.32962 | 0.649515 | -0.57478 | -1.73924 | -0.79328 | -1.09631 | -0.41279 | -0.11801 | -1.54374 | -0.93883 | -1.08619 | -3.48203 | -0.82807 | 0.005758 | -0.79969 | -0.53334 |
| Q920Q4 | Q920Q4 | VPS16_MC | Vps16 | Vacuolar protein sorting-associated protein 16 homolog | | | | 0.104206 | 0.094974 | 0.308798 | 0.582808 | -0.45414 | -1.54974 | 0.417477 | -0.35382 | -1.11051 | -0.73671 | -0.86381 | -0.65254 | -0.24596 | -0.35869 | -0.56234 | -0.59431 | -1.19616 | -0.20617 | 0.215946 | -0.4086 | 0.1952 |
| Q8R307 | Q8R307 | VPS18_MC | Vps18 | Vacuolar protein sorting-associated protein 18 homolog | | | | 0.798944 | 0.990446 | 2.199407 | 0.06483 | 0.043213 | 0.19802 | 0.192071 | 0.233516 | 0.541215 | -2.50337 | -1.74367 | -1.64873 | -1.21785 | -1.16859 | -1.51233 | -2.87209 | -3.35585 | -1.51124 | -1.44176 | -1.96974 | -1.17822 |
| P40336 | P40336 | VP26A_MC | Vps26a | Vacuolar protein sorting-associated protein 26A | | | | 0.754817 | 1.984149 | 2.05775 | 0.23012 | -0.16183 | -0.63818 | 0.174955 | 0.137801 | 0.497056 | -0.47271 | -0.67596 | -0.18211 | -0.63222 | 0.131058 | -0.37064 | -1.59962 | -3.51735 | -0.36217 | -0.12851 | -0.37563 | -0.13955 |
| Q8C0E2 | Q8C0E2 | VP26B_MC | Vps26b | Vacuolar protein sorting-associated protein 26B | | | + | NaN | 0 | NaN | 0.421736 | -0.41888 | -1.12142 | 1.025572 | 0.944076 | 3.021792 | -3.22602 | -3.55094 | -2.53192 | -3.27229 | -1.85766 | -2.44695 | -3.78116 | -3.12044 | -2.53311 | -2.43333 | -3.20027 | -2.99249 |
| Q9D1C8 | Q9D1C8 | VPS28_MC | Vps28 | Vacuolar protein sorting-associated protein 28 homolog | | | | 0.021236 | -0.04158 | -0.06757 | 0.819609 | -0.57826 | -2.26759 | 0.285297 | -0.15906 | -0.77705 | -3.55062 | -2.34068 | -1.39723 | -1.90715 | -1.67562 | -1.35274 | -3.01725 | -2.79087 | -1.06895 | -1.07892 | -1.48097 | -1.22926 |
| Q9QZ88 | Q9QZ88 | VPS29_MC | Vps29 | Vacuolar protein sorting-associated protein 29 | | | | 0.234772 | 0.622435 | 0.649945 | 0.011978 | 0.020711 | 0.038486 | 0.095032 | 0.067344 | 0.283469 | -1.13712 | -1.48301 | -1.09882 | -1.77728 | -1.02404 | -1.04576 | -0.99057 | -2.87443 | -1.041 | -1.87651 | -0.86492 | -1.33957 |
| Q9D2N9 | Q9D2N9 | VP33A_MC | Vps33a | Vacuolar protein sorting-associated protein 33A | | | | 0.248157 | -0.20964 | -0.68373 | 0.479746 | -0.31472 | -1.2718 | 0.194423 | -0.09239 | -0.54725 | 0.049695 | -0.27832 | 0.2084 | 0.328348 | 0.313766 | 0.495383 | 0.354384 | -0.16373 | 0.343011 | 0.823185 | 0.354644 | 0.639281 |
| P59016 | P59016 | VP33B_MC | Vps33b | Vacuolar protein sorting-associated protein 33B | | + | | 0.341077 | -0.77735 | -0.91698 | 1.337417 | -0.92679 | -4.50107 | 0.107596 | -0.19289 | -0.31809 | -3.40669 | -1.75953 | -1.83871 | -2.26231 | -2.17978 | -1.13851 | -2.0067 | -1.60482 | -1.03616 | -1.17527 | -1.15536 | -1.77715 |
| Q9EQH3 | Q9EQH3 | VPS35_MC | Vps35 | Vacuolar protein sorting-associated protein 35 | | | | 0.285071 | -0.1708 | -0.77649 | 0.421915 | -0.29735 | -1.12188 | 0.482399 | 0.532078 | 1.278762 | 1.308617 | 1.740241 | 2.229412 | 1.755691 | 3.30002 | 2.473609 | 1.652678 | 1.737781 | 2.170957 | 2.408853 | 2.40363 | 2.305844 |
| Q8BWQ6 | Q8BWQ6 | VPS35L_MC | Vps35l | VPS35 endosomal protein-sorting factor-like | | | | 0.060208 | -0.14814 | -0.18463 | 0.258931 | -0.68553 | -0.71085 | 0.249359 | 0.190862 | 0.686755 | -3.80061 | -2.23928 | -1.51753 | -3.43604 | -1.07957 | -1.62755 | -2.68642 | -3.05718 | -1.69192 | -1.89059 | -1.59098 | -1.49786 |
| Q8R105 | Q8R105 | VP37C_MC | Vps37c | Vacuolar protein sorting-associated protein 37C | | | | 0.418919 | -0.55595 | -1.1142 | 0.261415 | 0.190529 | 0.717098 | 0.889691 | -0.62284 | -2.50827 | -3.68014 | -3.63052 | -2.31607 | -2.81257 | -3.08952 | -3.21852 | -2.60102 | -3.59773 | -2.84955 | -2.66015 | -2.29139 | -2.77097 |
| Q8R5L3 | Q8R5L3 | VPS39_MC | Vps39 | Vam6/Vps39-like protein | | | | 0.238937 | 0.714451 | 0.66047 | 0.375053 | -0.32166 | -1.00261 | 0.201876 | -0.28365 | -0.56634 | -3.36438 | -1.46339 | -2.28562 | -1.87055 | -1.8063 | -2.57783 | -3.64477 | -2.61191 | -1.51178 | -2.00108 | -2.22784 | -1.58898 |
| P97390 | P97390 | VPS45_MC | Vps45 | Vacuolar protein sorting-associated protein 45 | | | | 0.505549 | -0.86888 | -1.33994 | 0.661573 | 0.129936 | 1.774439 | 0.084763 | 0.07892 | 0.25479 | -4.40065 | -3.20403 | -1.27112 | -1.20275 | -0.94297 | -0.84562 | -2.68343 | -3.18349 | -1.43162 | -1.30211 | -0.66732 | -1.27911 |
| Q8VEJ9 | Q8VEJ9 | VPS4A_MC | Vps4a | Vacuolar protein sorting-associated protein 4A | | + | | 0.487153 | 0.59347 | 1.29127 | 1.758681 | 0.378549 | 7.474888 | 0.067591 | 0.062373 | 0.205978 | 0.838325 | -0.01291 | 0.774829 | 0.676277 | 0.59997 | 0.576942 | -0.00732 | -0.35421 | 0.335318 | 0.35869 | 0.223486 | 0.828679 |
| Q8CI71 | Q8CI71 | VPS50_MC | Vps50 | Syndetin | | | | 0.575086 | 1.35277 | 1.528342 | 0.653621 | -0.40674 | -1.75119 | 0.198286 | 0.243972 | 0.557156 | -1.07405 | -1.71164 | -0.82437 | -1.00522 | -0.5362 | -0.70503 | -1.9199 | -3.57133 | -0.722 | -0.29412 | -1.29426 | -0.43491 |
| Q3UVL4 | Q3UVL4 | VPS51_MC | Vps51 | Vacuolar protein sorting-associated protein 51 homolog | | | | 0.118177 | 0.199662 | 0.346898 | 0.612423 | -0.34259 | -1.63282 | 0.309464 | 0.334049 | 0.83764 | -0.91004 | -0.24297 | 0.546925 | 0.129731 | 1.013061 | 0.375903 | -0.3071 | -1.24524 | 0.658359 | 0.703472 | 0.120543 | 0.600323 |
| Q8C754 | Q8C754 | VPS52_MC | Vps52 | Vacuolar protein sorting-associated protein 52 homolog | | | | 0.424453 | 0.722525 | 1.128388 | 0.084836 | -0.02064 | -0.25499 | 0.275805 | 0.233455 | 0.75324 | 1.122862 | 0.643959 | 1.487236 | 1.440168 | 2.06568 | 1.543565 | 0.754744 | -0.43297 | 1.561792 | 1.406894 | 1.404109 | 1.738226 |
| Q8CCB4 | Q8CCB4 | VPS53_MC | Vps53 | Vacuolar protein sorting-associated protein 53 homolog | | | | 0.456276 | -1.59708 | -1.21052 | 0.180887 | -0.13502 | -0.51241 | 0.081443 | 0.061937 | 0.245438 | -1.65492 | -3.49438 | -0.1746 | -0.48297 | -0.20423 | -0.20628 | -0.03168 | -1.92348 | -0.40745 | 0.019929 | -0.51954 | -0.01484 |
| Q0P5W1 | Q0P5W1 | VPS8_MO | Vps8 | Vacuolar protein sorting-associated protein 8 homolog | | | | 0.078245 | -0.3812 | -0.2364 | 0.653894 | 0.624322 | 1.751986 | 0.039231 | -0.0495 | -0.12261 | -3.83681 | -2.29949 | -0.88649 | -1.03424 | -1.27487 | -1.95887 | -4.10453 | -1.26937 | -1.9333 | -1.23608 | -1.78192 | -1.35283 |
| P62761 | P62761 | VISL1_MO | Vsnl1 | Visinin-like protein 1 | | | | 0.289728 | -0.1347 | -0.78816 | 0.56554 | -0.48778 | -1.50202 | 0.259931 | 0.069294 | 0.713364 | 3.532835 | 3.452729 | 3.55047 | 2.90149 | 4.152034 | 4.324344 | 3.793626 | 3.461338 | 3.726795 | 3.700733 | 4.213759 | 4.124031 |
| Q9CR26 | Q9CR26 | VTA1_MO | Vta1 | Vacuolar protein sorting-associated protein VTA1 homolog | | | | 0.168648 | 0.733557 | 0.480676 | 0.357272 | -1.3639 | -0.95773 | 0.385941 | 2.036146 | 1.030185 | -1.24729 | -0.32003 | -0.65446 | -3.41 | 0.644708 | -0.92657 | -0.06325 | -2.97118 | -0.30803 | -1.02862 | -0.36344 | -3.99071 |
| Q8BGN8 | Q8BGN8 | SYNPR_M | Synpr | Synaptoporin | + | | | 2.462742 | -2.80832 | -16.9921 | 0.22363 | -1.05798 | -0.62173 | 0.09368 | 0.305234 | 0.279711 | -3.35175 | -3.68176 | 0.056928 | -3.33987 | -0.15114 | 1.678396 | -0.69904 | -0.71783 | -0.68895 | -0.47803 | 1.05339 | -0.1366 |
| Q8CC88 | Q8CC88 | VWA8_MO | Vwa8 | von Willebrand factor A domain-containing protein 8 | | | | 0.148216 | 0.063146 | 0.427151 | 0.248966 | -0.14025 | -0.68577 | 0.218297 | 0.332214 | 0.608194 | -0.61441 | -0.46075 | -0.79128 | -0.52238 | -0.23367 | -1.05394 | -0.72702 | -0.47443 | -0.36247 | -0.6707 | -1.33679 | -0.61525 |
| P32921 | P32921 | SYWC_MO | Wars1 | Tryptophan--tRNA ligase, cytoplasmic | | | | 0.90619 | 0.22787 | 2.567174 | 0.339446 | 0.273203 | 0.912876 | 0.311199 | -0.07629 | -0.84199 | -0.0824 | 0.065073 | 0.051132 | -0.22349 | -0.11497 | -0.28236 | -0.28595 | -0.18712 | -0.6253 | -0.09346 | -0.0877 | -0.15706 |
| Q8R5H6 | Q8R5H6 | WASF1_M | Wasf1 | Actin-binding protein WASF1 | | | | 0.325545 | 1.135331 | 0.87797 | 0.4467 | -0.30235 | -1.1857 | 0.295024 | -0.23123 | -0.80144 | -0.79528 | -0.58657 | 1.631033 | 1.780894 | 1.4888 | 2.060875 | -0.53734 | -0.31517 | 1.764575 | 2.252054 | 1.968349 | 2.043778 |
| Q8VHI6 | Q8VHI6 | WASF3_M | Wasf3 | Actin-binding protein WASF3 | | + | | 0.142218 | 0.14418 | 0.411288 | 1.289537 | -0.27265 | -4.24156 | 0.400216 | -0.40831 | -1.06645 | -0.813 | -0.20064 | 0.435518 | 0.36655 | -0.18486 | 0.393953 | -0.48029 | -0.82171 | 0.619435 | 0.727931 | 0.262196 | 0.763508 |
| Q8C2E7 | Q8C2E7 | WASC5_M | Washc5 | WASH complex subunit 5 | | | | 0.083462 | 0.188964 | 0.25113 | 0.121199 | -0.3007 | -0.35507 | 0.483005 | 0.41426 | 1.280356 | -1.87457 | -2.60835 | -3.03577 | -2.19366 | -1.43566 | -1.95773 | -3.08736 | -1.77348 | -3.04881 | -1.57922 | -1.91978 | -2.30213 |
| Q91YD9 | Q91YD9 | WASL_MO | Wasl | Actin nucleation-promoting factor WASL | | | | NaN | 0 | NaN | 0.108558 | 0.198928 | 0.320725 | 0.962698 | 0.542582 | 2.775923 | -3.97083 | -3.99322 | -0.7424 | -1.53741 | -0.24154 | -0.42331 | -3.37881 | -3.08462 | -1.81495 | -0.86271 | -1.04806 | -0.70196 |
| P97765 | P97765 | WBP2_MO | Wbp2 | WW domain-binding protein 2 | | | | 0.206671 | -0.21164 | -0.57859 | 0.214769 | -0.12377 | -0.59922 | 0.8954 | -0.25703 | -2.52855 | -0.1081 | 0.609221 | 0.491118 | 0.354403 | 0.658859 | 0.769519 | 0.53409 | 0.390322 | 0.741428 | 0.351623 | 1.056494 | 0.885946 |
| Q6VNB8 | Q6VNB8 | WDFY3_M | Wdfy3 | WD repeat and FYVE domain-containing protein 3 | | | | 0.647565 | 0.38769 | 1.733576 | 0.550356 | 0.357016 | 1.460473 | 0.512516 | -0.41693 | -1.35849 | -0.70303 | -0.34098 | 0.463221 | 0.799568 | -0.29541 | -0.02996 | -0.77838 | -1.041 | 0.096967 | 0.451789 | -0.02248 | 0.530967 |
| O88342 | O88342 | WDR1_MC | Wdr1 | WD repeat-containing protein 1 | | | | 0.949791 | -1.12394 | -2.72726 | 0.839488 | -1.16964 | -2.33434 | 0.05835 | -0.11976 | -0.17922 | -0.28859 | 0.422109 | 0.914189 | -0.08505 | 1.048044 | 2.274035 | 1.399431 | 0.981981 | 1.62219 | 1.546238 | 2.046813 | 1.514785 |
| Q8K1X1 | Q8K1X1 | WDR11_M | Wdr11 | WD repeat-containing protein 11 | | | | 0.031687 | -0.06133 | -0.09976 | 0.0906 | 0.058688 | 0.271134 | 0.645451 | 0.408488 | 1.727445 | -1.83554 | -1.3819 | -1.09478 | -1.50677 | -0.82238 | -1.23039 | -2.11885 | -0.97593 | -1.42593 | -1.293 | -1.55445 | -1.3153 |
| Q91V09 | Q91V09 | WDR13_M | Wdr13 | WD repeat-containing protein 13 | | | | 0.129565 | 0.516571 | 0.377568 | 0.02926 | 0.047582 | 0.092339 | 0.399456 | 0.234071 | 1.064518 | -1.27765 | -0.78878 | 0.770399 | 0.677023 | 0.122227 | 0.528344 | -2.89593 | -0.20365 | 0.162953 | 1.189305 | 0.175572 | 0.006857 |
| Q4VBE8 | Q4VBE8 | WDR18_M | Wdr18 | WD repeat-containing protein 18 | | | | 0.021882 | 0.05667 | 0.069573 | 0.710663 | 1.085249 | 1.921032 | 0.316264 | 0.258642 | 0.854689 | -3.10049 | -3.02352 | -0.63215 | -0.5068 | -0.97046 | -1.53321 | -3.9323 | -2.30505 | -2.21617 | -1.09328 | -1.62186 | -1.39909 |
| Q3UWE6 | Q3UWE6 | Q3UWE6_ | Wdr20 | WD repeat domain 20 | | | | NaN | 0 | NaN | 0.294877 | -0.4015 | -0.80107 | 0.124604 | 0.093062 | 0.364247 | -3.5859 | -3.60893 | -1.23528 | -2.22344 | -1.45156 | -1.10817 | -3.32105 | -3.38026 | -1.41208 | -1.24363 | -1.18373 | -1.56213 |
| Q9D5R2 | Q9D5R2;Q | WDR20_M | Wdr20 | WD repeat-containing protein 20 | | | | NaN | 0 | NaN | 0.314742 | -0.28665 | -0.85087 | 0.277211 | 0.244209 | 0.756767 | -2.54716 | -3.77774 | -2.38084 | -1.74517 | -1.67259 | -1.82247 | -3.1855 | -3.36676 | -1.88804 | -1.66466 | -1.67786 | -2.30562 |
| Q8CFJ9 | Q8CFJ9 | WDR24_M | Wdr24 | GATOR complex protein WDR24 | | | | 0.065149 | 0.090706 | 0.198942 | 0.09259 | -0.05886 | -0.27668 | 0.469936 | -0.58869 | -1.24611 | -3.1548 | -3.11863 | -0.77368 | -1.15053 | -1.76323 | -0.98694 | -3.68301 | -2.77184 | -1.002 | -0.80449 | -1.0557 | -0.5171 |
| Q8C6G8 | Q8C6G8 | WDR26_M | Wdr26 | WD repeat-containing protein 26 | | | | 0.919796 | 0.659791 | 2.616434 | 0.560143 | -0.21623 | -1.48721 | 0.786185 | -0.52207 | -2.15791 | -0.67774 | -0.52872 | 0.282695 | 0.533082 | -0.07818 | -0.22887 | -1.02211 | -1.50393 | 0.550189 | 0.698046 | 0.138644 | 0.598444 |
| Q8CBE3 | Q8CBE3 | WDR37_M | Wdr37 | WD repeat-containing protein 37 | | + | | 0.039369 | -0.02551 | -0.12302 | 2.505949 | -0.30913 | -17.8631 | 0.054179 | 0.032776 | 0.167017 | 0.49206 | 0.251465 | 1.858824 | 1.884219 | 1.743851 | 1.681099 | 0.56613 | 0.228408 | 2.16889 | 2.192405 | 1.485979 | 1.873419 |
| Q80VQ0 | Q80VQ0 | AL3B1_MC | Aldh3b1 | Aldehyde dehydrogenase family 3 member B1 | + | | | 1.68501 | -2.82226 | -6.85008 | 0.490918 | -0.89945 | -1.30119 | 0.316037 | 0.780171 | 0.85412 | -3.19913 | -3.24489 | -3.78929 | -4.33607 | -1.98493 | -1.91047 | 0.01162 | -0.81112 | -3.79812 | -2.52834 | -1.81521 | -3.64053 |
| Q6NVE8 | Q6NVE8 | WDR44_M | Wdr44 | WD repeat-containing protein 44 | | | | NaN | 0 | NaN | 0.099691 | -0.27937 | -0.29637 | 0.043044 | 0.111611 | 0.134041 | -2.90686 | -4.16207 | -2.00806 | -3.54148 | -2.29768 | -2.60383 | -3.64811 | -3.53587 | -3.04383 | -1.94696 | -1.7439 | -3.38084 |
| Q91VM3 | Q91VM3 | WIPI4_MO | Wdr45 | WD repeat domain phosphoinositide-interacting protein 4 | | | | 0.663916 | 1.575626 | 1.781312 | 0.426396 | -0.27374 | -1.13337 | 0.485289 | -0.30809 | -1.28636 | -1.05311 | -0.41408 | -0.27267 | 0.054547 | -0.76048 | -0.75619 | -1.48441 | -3.13403 | -0.01299 | 0.342362 | -0.68974 | -0.21075 |

| | | | | | | | | | | | | | | | | | | | | | | | | | | | | |
|---|---|---|---|---|---|---|---|---|---|---|---|---|---|---|---|---|---|---|---|---|---|---|---|---|---|---|---|---|
| Q8CGF6 | Q8CGF6 | WDR47_M | Wdr47 | WD repeat-containing protein 47 | + | | | 1.191496 | 1.178598 | 3.749431 | 0.146718 | -0.05751 | -0.4232 | 0.44832 | -0.32185 | -1.18989 | -0.05522 | 0.501904 | 1.479417 | 1.646844 | 0.881629 | 1.177068 | -1.1009 | -0.80962 | 1.513584 | 1.727706 | 1.124611 | 1.577779 |
| Q61647 | Q61647 | HYAS1_MC | Has1 | Hyaluronan synthase 1 | + | + | | 4.748422 | -2.85329 | -236.704 | 2.773511 | -4.62837 | -24.3336 | 0.392449 | 2.037631 | 1.046703 | -2.824 | -2.84771 | -3.5243 | -3.60848 | 0.450424 | -3.4174 | 0.015238 | 0.019629 | 1.247468 | 0.876489 | -3.29822 | -3.74401 |
| Q8C0M0 | Q8C0M0 | WDR59_M | Wdr59 | GATOR complex protein WDR59 | | | | NaN | 0 | NaN | 0.575144 | 0.374786 | 1.528501 | 0.0097 | -0.02247 | -0.03124 | -3.51916 | -3.49682 | -1.23746 | -1.30234 | -2.15237 | -1.4762 | -3.15121 | -4.06239 | -1.88773 | -1.40165 | -2.42645 | -1.15718 |
| Q99ME2 | Q99ME2 | WDR6_MC | Wdr6 | WD repeat-containing protein 6 | | | | 0.574408 | 1.076253 | 1.526467 | 0.029514 | 0.020388 | 0.093115 | 0.07022 | -0.08065 | -0.21352 | -0.98451 | -1.26041 | -0.60503 | -0.24542 | -0.7231 | -1.29376 | -1.50728 | -2.89015 | -0.57056 | -0.32066 | -1.17528 | -0.68027 |
| Q920I9 | Q920I9 | WDR7_MC | Wdr7 | WD repeat-containing protein 7 | | | | 0.594395 | 0.267052 | 1.582058 | 0.562055 | 0.203757 | 1.492452 | 0.059655 | 0.04759 | 0.183018 | 1.969511 | 2.152851 | 3.809284 | 3.92396 | 3.919982 | 4.024333 | 1.935868 | 1.652388 | 3.538964 | 3.786766 | 3.669827 | 4.179308 |
| Q920E5 | Q920E5 | FPPS_MOI | Fdps | Farnesyl pyrophosphate synthase | + | | | 2.136826 | -2.95091 | -11.642 | NaN | 0 | NaN | 0.410002 | 0.961461 | 1.091402 | -3.96649 | -4.11381 | -3.00325 | -3.88044 | -3.01919 | -1.6241 | -1.33177 | -0.84671 | -3.03 | -3.3371 | -2.74507 | -3.82115 |
| Q5ND34 | Q5ND34 | WDR81_M | Wdr81 | WD repeat-containing protein 81 | | | + | 0.223159 | 0.234644 | 0.620537 | 0.037235 | 0.066962 | 0.116592 | 1.574243 | -0.6671 | -6.00209 | -3.62565 | -2.8892 | -2.36068 | -1.58648 | -2.38308 | -2.24111 | -3.57805 | -3.40608 | -2.46481 | -1.61627 | -1.73052 | -1.55947 |
| Q8BFQ4 | Q8BFQ4 | WDR82_M | Wdr82 | WD repeat-containing protein 82 | | | | NaN | 0 | NaN | 0.477387 | -0.2725 | -1.26561 | 0.618871 | -0.33215 | -1.65113 | -3.1573 | -4.05019 | -0.72662 | -0.88901 | -1.1594 | -1.15447 | -3.10745 | -3.56078 | -0.73473 | -0.3359 | -1.02594 | -0.62364 |
| Q7TMQ7 | Q7TMQ7 | WDR91_M | Wdr91 | WD repeat-containing protein 91 | | | | 0.515039 | 0.442528 | 1.365222 | 0.346175 | -0.28857 | -0.92979 | 0.400962 | -0.70716 | -1.06835 | -1.02244 | -1.33371 | -0.82118 | -0.54012 | -1.95122 | -0.73438 | -1.33627 | -1.90494 | -0.6688 | -0.11537 | -0.89633 | -0.37495 |
| O88291 | O88291 | ZN326_MC | Znf326 | DBIRD complex subunit ZNF326 | + | + | | 2.731775 | -2.95779 | -23.189 | 1.208764 | -0.33008 | -3.83249 | 0.107166 | -0.26032 | -0.31692 | -3.17005 | -3.34392 | -0.04806 | 0.118647 | 1.249424 | -0.00405 | -0.39253 | -0.20586 | 0.387058 | 0.343692 | 0.352031 | 1.413986 |
| P56695 | P56695 | WFS1_MO | Wfs1 | Wolframin | + | + | | 1.253067 | 1.253995 | 4.05255 | 1.626563 | 1.388537 | 6.389651 | 0.058838 | 0.088384 | 0.180642 | 0.546787 | 0.778744 | 2.249811 | 2.659775 | 2.154391 | 1.180528 | -0.87811 | -0.30435 | 1.138409 | 0.994102 | 1.626934 | 1.531218 |
| P0C7L0 | P0C7L0 | WIPF3_MC | Wipf3 | WAS/WASL-interacting protein family member 3 | | + | | NaN | 0 | NaN | 1.270106 | 1.228593 | 4.139955 | 0.119799 | -0.14416 | -0.35129 | -3.17454 | -3.36473 | -1.60183 | -1.66825 | -1.10079 | -0.34288 | -3.70291 | -3.2259 | -3.15854 | -2.56873 | -0.42018 | -0.73517 |
| Q80W47 | Q80W47 | WIPI2_MO | Wipi2 | WD repeat domain phosphoinositide-interacting protein 2 | | + | | 0.066401 | 0.050572 | 0.202551 | 1.352987 | -0.45963 | -4.58836 | 0.397137 | 0.101409 | 1.058617 | -0.03076 | -0.45643 | -0.20307 | -0.13088 | 0.552833 | 0.689948 | -0.16364 | -0.42471 | 0.199214 | 0.386103 | 0.453076 | 0.586886 |
| Q5SXA9 | Q5SXA9 | KIBRA_MO | Wwc1 | Protein KIBRA | | | | 0.928592 | 0.708364 | 2.648611 | 0.781556 | -0.82406 | -2.14296 | 0.639581 | -1.65678 | -1.71047 | -2.50731 | -2.5329 | -2.06314 | -1.97733 | -3.88426 | -2.09701 | -2.96133 | -3.49561 | -1.57832 | -0.81404 | -1.70753 | -0.96019 |
| Q8BZZ3 | Q8C863;Q | WWP1_MC | Wwp1 | NEDD4-like E3 ubiquitin-protein ligase WWP1 | | | | 0.171131 | 0.243044 | 0.487133 | 0.171661 | 0.29397 | 0.488508 | 0.393953 | 0.272533 | 1.050523 | -2.09588 | -1.56376 | -2.21853 | -1.99273 | -1.88241 | -1.51392 | -1.6508 | -2.49493 | -2.99069 | -1.80852 | -1.78806 | -2.15333 |
| Q6P1B1 | Q6P1B1 | XPP1_MOI | Xpnpep1 | Xaa-Pro aminopeptidase 1 | | | | 0.087029 | -0.19955 | -0.26115 | 0.594024 | -0.87556 | -1.58102 | 0.300228 | 0.857796 | 0.814486 | -2.23689 | -3.765 | -2.11252 | -3.21297 | -2.83643 | -1.5518 | -2.81251 | -2.79027 | -1.84996 | -1.72441 | -2.21728 | -3.88654 |
| Q6P5F9 | Q6P5F9 | XPO1_MOI | Xpo1 | Exportin-1 | + | + | | 1.032984 | 0.596508 | 3.051782 | 1.111741 | 0.394667 | 3.384426 | 0.430898 | 0.251946 | 1.144942 | 0.775275 | 0.878356 | 0.93949 | 0.750414 | 1.202981 | 0.824347 | 0.418852 | 0.041763 | 0.382013 | 0.518557 | 0.649549 | 0.873886 |
| Q924C1 | Q924C1 | XPO5_MOI | Xpo5 | Exportin-5 | + | + | | 1.084538 | 0.27027 | 3.266556 | 1.456292 | 0.80239 | 5.206154 | 0.075437 | -0.07209 | -0.22842 | -0.08172 | -0.03662 | 0.384549 | 0.513394 | 0.194353 | -0.23272 | -0.40905 | -0.24984 | -0.49343 | -0.21341 | -0.17948 | 0.285286 |
| Q924Z6 | Q924Z6 | XPO6_MOI | Xpo6 | Exportin-6 | | | + | 0.400047 | 1.88813 | 1.066021 | 0.702451 | 0.606228 | 1.896127 | 1.26241 | -0.41807 | -4.10028 | -0.71455 | -0.25009 | -0.14671 | 0.02874 | -0.63511 | -0.66138 | -4.12635 | -0.61454 | -0.35777 | -0.97266 | -0.33129 | -0.12907 |
| Q9EPK7 | Q9EPK7 | XPO7_MOI | Xpo7 | Exportin-7 | + | | | 1.23597 | 0.569621 | 3.966416 | 0.23077 | 0.163228 | 0.639823 | 0.024378 | -0.02687 | -0.07731 | 1.05199 | 0.877447 | 0.778384 | 0.862494 | 0.684837 | 0.658779 | 0.281046 | 0.509149 | 0.908835 | 0.405588 | 0.351373 | 1.045982 |
| Q9CRT8 | Q9CRT8 | XPOT_MOI | Xpot | Exportin-T | | + | | 0.018518 | 0.08891 | 0.059085 | 1.019278 | 0.442628 | 2.996498 | 0.224109 | 0.297194 | 0.622948 | -2.00923 | -1.83359 | -1.03964 | -0.7446 | -1.08817 | -1.14298 | -0.50811 | -3.51252 | -1.32713 | -1.34237 | -1.88906 | -0.93648 |
| Q9Z0U0 | Q9Z0U0 | S53A1_MC | Xpr1 | Solute carrier family 53 member 1 | | | | NaN | 0 | NaN | 0.420308 | -1.10999 | -1.11776 | 0.418197 | -1.05874 | -1.11235 | -3.12477 | -4.01786 | -3.24565 | -3.56318 | -3.33725 | -3.61873 | -3.5588 | -3.18146 | -1.31414 | -3.2747 | -1.4779 | -3.36059 |
| Q9DBR1 | Q9DBR1 | XRN2_MOI | Xrn2 | 5'-3' exoribonuclease 2 | | | | 0.014611 | -0.06742 | -0.04681 | 0.230905 | 0.155479 | 0.640165 | 0.285704 | -0.1435 | -0.77807 | -1.63712 | -4.04916 | -1.34193 | -0.87803 | -1.60754 | -1.66977 | -1.98846 | -3.56298 | -1.19343 | -1.33749 | -1.31336 | -1.67694 |
| Q91WQ3 | Q91WQ3 | SYYC_MOI | Yars1 | Tyrosine--tRNA ligase, cytoplasmic | | | | 0.005089 | -0.00173 | -0.01648 | 0.207676 | 0.101031 | 0.581155 | 0.497872 | -0.17374 | -1.31957 | -0.70584 | -0.60715 | -0.11911 | -0.46212 | -0.39183 | -0.64187 | -0.56193 | -0.74759 | -0.3632 | -0.42009 | -0.38441 | -0.3018 |
| P61971 | P61971 | NTF2_MOI | Nutf2 | Nuclear transport factor 2 | + | | | 2.542068 | -3.45112 | -18.625 | 0.243804 | -0.93026 | -0.67275 | 0.213981 | -0.44029 | -0.59722 | -3.6962 | -3.41861 | -0.76654 | -3.33655 | -1.4338 | -0.76296 | 0.016469 | -0.22905 | -0.6106 | -1.63196 | -0.00159 | -1.31459 |
| Q9CX30 | Q9CX30 | YIF1B_MO | Yif1b | Protein YIF1B | | | | 0.025781 | 0.030952 | 0.081646 | 0.176103 | -0.23254 | -0.50003 | 0.178269 | -0.13352 | -0.50564 | -1.67067 | -1.62904 | -2.42073 | -1.62648 | -1.79314 | -1.30722 | -2.05933 | -1.30228 | -2.03306 | -1.54908 | -1.31321 | -1.52011 |
| Q9EQQ2 | Q9EQQ2 | YIPF5_MO | Yipf5 | Protein YIPF5 | | | | 0.408921 | 0.193981 | 1.088642 | 0.667255 | -0.38322 | -1.79113 | 0.007363 | 0.010531 | 0.023779 | 0.319032 | 0.208706 | -0.52593 | -0.46595 | 0.344002 | -0.30306 | 0.239321 | -0.09955 | 0.099119 | -0.32457 | -0.29246 | 0.312336 |
| Q9CQW1 | Q9CQW1 | YKT6_MOI | Ykt6 | Synaptobrevin homolog YKT6 | | | | 0.531772 | -0.58776 | -1.41012 | 0.728556 | 1.596111 | 1.975858 | 0.609376 | 0.877355 | 1.6242 | -4.03353 | -3.20588 | -1.97736 | -2.84651 | -2.26424 | -3.31656 | -2.98214 | -3.08175 | -4.689 | -3.32709 | -3.5455 | -3.79001 |
| Q3U5F4 | Q3U5F4 | YRDC_MO | Yrdc | Threonylcarbamoyl-AMP synthase | | | | 0.706519 | 1.212998 | 1.908447 | 0.150289 | 0.266298 | 0.432616 | 0.019407 | 0.072768 | 0.061863 | -2.72447 | -1.48005 | -2.98351 | -2.45839 | -2.47617 | -1.48096 | -3.44502 | -3.18549 | -3.54399 | -2.4305 | -3.11717 | -0.9855 |
| P59326;Q | Q91YT7;Q | YTHD1_MC | Ythdf1;Ythdf2;Ythdf3 | YTH domain-containing family protein 1 | | | | NaN | 0 | NaN | 0.801373 | 0.609875 | 2.207359 | 0.009361 | -0.03791 | -0.03016 | -3.58382 | -2.51877 | -1.48937 | -0.99392 | -1.73657 | -3.08599 | -3.96794 | -3.97497 | -1.72917 | -1.97388 | -3.43365 | -1.31309 |
| Q9R0C8 | Q9R0C8 | VAV3_MOI | Vav3 | Guanine nucleotide exchange factor VAV3 | + | | | 2.45611 | -3.45641 | -16.8622 | NaN | 0 | NaN | 0.044766 | -0.30058 | -0.13918 | -2.7845 | -2.93585 | -3.42455 | -3.56615 | -3.17289 | -1.02533 | 0.405737 | 0.786739 | -3.24707 | -3.07603 | 0.07524 | -3.6723 |
| P62259 | P62259 | 1433E_MC | Ywhae | 14-3-3 protein epsilon | + | | + | 1.111826 | 0.71323 | 3.384801 | 0.671207 | 0.245115 | 1.802782 | 1.2089 | 0.161224 | 3.833146 | 5.958031 | 6.362936 | 6.866111 | 7.130009 | 6.740646 | 6.798633 | 5.505684 | 5.388822 | 6.720143 | 6.785747 | 6.638886 | 6.577945 |
| P61982 | P61982 | 1433G_MC | Ywhag | 14-3-3 protein gamma | + | | + | 1.24663 | 0.731882 | 4.019942 | 0.764696 | 0.602174 | 2.089013 | 1.873218 | 0.45053 | 8.554865 | 5.674971 | 5.960681 | 6.25266 | 6.550268 | 6.116112 | 6.101722 | 5.198812 | 4.973075 | 5.552411 | 6.046171 | 5.606217 | 5.710557 |
| P68510 | P68510 | 1433F_MC | Ywhah | 14-3-3 protein eta | | | + | 0.922261 | 1.630398 | 2.625425 | 0.826158 | 0.423282 | 2.289453 | 1.052952 | 0.460891 | 3.133665 | 3.502396 | 4.17751 | 4.781377 | 5.04244 | 4.809151 | 4.741322 | 2.730804 | 1.688306 | 4.357694 | 4.61956 | 4.171232 | 4.457459 |
| P68254 | P68254 | 1433T_MC | Ywhaq | 14-3-3 protein theta | + | | | 1.03648 | 0.889467 | 3.066002 | 0.321501 | 0.181209 | 0.867824 | 0.586894 | 0.162502 | 1.561111 | 3.708059 | 4.283019 | 4.20154 | 4.459696 | 4.113266 | 4.320182 | 3.14502 | 3.067124 | 3.985276 | 4.313543 | 4.065706 | 4.042739 |
| P63101 | P63101 | 1433Z_MC | Ywhaz | 14-3-3 protein zeta/delta | | | + | 0.827305 | 0.578183 | 2.293295 | 0.811828 | 0.565691 | 2.241775 | 1.050119 | 0.387172 | 3.121951 | 4.238013 | 4.688871 | 5.937232 | 5.825848 | 5.891869 | 5.729915 | 3.998156 | 3.772362 | 5.069731 | 5.561967 | 5.51765 | 5.329791 |
| Q8BJH1 | Q8BJH1 | ZC21A_MC | Zc2hc1a | Zinc finger C2HC domain-containing protein 1A | | | | 0.842948 | 0.866442 | 2.346081 | 0.470027 | 0.089789 | 1.246345 | 0.324646 | -0.3412 | -0.87572 | 0.725199 | 0.465424 | 0.421979 | 0.51696 | 0.082331 | 0.768181 | 0.07459 | -0.61685 | 0.433854 | 0.325508 | 0.951419 | 0.581496 |
| G3X9I7 | G3X9I7 | G3X9I7_MC | Zc3h12b | Zinc finger CCCH-type containing 12B | | | | 0.008911 | 0.0566 | 0.028727 | 0.107712 | 0.068941 | 0.318413 | 0.697177 | -0.77571 | -1.88022 | -1.14284 | -3.04213 | 0.278311 | 0.363938 | -0.78097 | -0.36368 | -0.42273 | -3.87544 | 0.039945 | 0.464423 | -0.15253 | 0.5593 |
| Q6ZPZ3 | Q6ZPZ3 | ZC3H4_MC | Zc3h4 | Zinc finger CCCH domain-containing protein 4 | | | | 0.619896 | 0.647474 | 1.654042 | 0.997479 | -0.23058 | -2.91009 | 0.355441 | 0.219921 | 0.953116 | -0.76253 | 0.020361 | -0.42606 | -0.30113 | -0.2076 | -0.65767 | -1.01952 | -1.0176 | -0.08426 | -0.18176 | -0.60156 | -0.70354 |
| Q9ESX4 | Q9ESX4 | ZCC17_MC | Zcchc17 | Zinc finger CCHC domain-containing protein 17 | + | | + | 1.19764 | 0.585875 | 3.778811 | 0.917019 | 0.573094 | 2.606328 | 1.027002 | 0.499427 | 3.02756 | 1.223261 | 1.238949 | 0.753964 | 1.141379 | 1.260132 | 1.496618 | 0.490387 | 0.800074 | 0.478631 | 0.270525 | 0.763925 | 0.993972 |
| Q80ZJ6 | Q80ZJ6 | ZER1_MOI | Zer1 | Protein zer-1 homolog | | | | 0.448166 | 0.498205 | 1.189494 | 0.067286 | 0.07544 | 0.205101 | 0.223833 | -0.14005 | -0.62225 | -0.88372 | -0.84491 | -0.25996 | -0.02708 | -0.24188 | -0.19174 | -1.78091 | -0.94413 | -0.56786 | 0.129943 | -0.30043 | 0.146903 |
| O88532 | O88532 | ZFR_MOUS | Zfr | Zinc finger RNA-binding protein | | | | 0.515108 | 1.70541 | 1.365408 | 0.39304 | 0.835692 | 1.048203 | 0.602111 | 1.141816 | 1.60371 | -3.02262 | -0.5503 | -1.37984 | -1.14436 | -0.69395 | -1.43846 | -3.67056 | -3.31318 | -2.88631 | -1.30927 | -1.6011 | -2.81493 |
| Q9JMD0 | Q9JMD0 | ZN207_MC | Znf207 | BUB3-interacting and GLEBS motif-containing protein ZNF207 | | | + | 0.235787 | 0.441325 | 0.65251 | 0.807947 | -0.54428 | -2.22896 | 1.600791 | -0.83524 | -6.19594 | -3.05684 | -1.75234 | -2.17256 | -2.34267 | -1.91397 | -2.15638 | -2.66697 | -3.02487 | -1.48444 | -1.94223 | -1.14094 | -1.25894 |
| Q63912 | Q63912 | OMGP_MC | Omg | Oligodendrocyte-myelin glycoprotein | + | | | 1.565227 | -3.84339 | -5.93753 | 0.293638 | 1.104894 | 0.797966 | 0.023644 | -0.09875 | -0.07504 | -3.90726 | -3.29796 | -0.84746 | -2.10118 | -2.61827 | -2.00627 | 0.811904 | -0.33036 | -3.81383 | -1.3446 | -0.93361 | -3.49342 |
| Q8BXJ8 | Q8BXJ8 | Z385B_MC | Znf385b | Zinc finger protein 385B | | | | 0.686413 | 2.399891 | 1.847937 | 0.701861 | 1.224562 | 1.894343 | 0.116404 | 0.217061 | 0.342093 | -0.37635 | 0.104928 | -2.22409 | -2.2041 | -0.53183 | -1.65586 | -1.25941 | -3.8118 | -2.7923 | -4.08501 | -1.0164 | -1.60541 |
| Q69Z99 | Q69Z99 | ZN512_MC | Znf512 | Zinc finger protein 512 | | | | 0.094465 | -0.26779 | -0.2819 | 0.198316 | -0.2152 | -0.55723 | 0.333445 | 0.470572 | 0.8978 | -2.06491 | -3.75884 | -1.93258 | -1.20231 | -1.75873 | -1.51663 | -2.2139 | -3.07427 | -1.22645 | -1.47803 | -2.61822 | -1.59828 |
| Q9D2D7 | Q9D2D7 | ZN687_MC | Znf687 | Zinc finger protein 687 | + | | | 1.184879 | 1.596346 | 3.717996 | 0.232097 | 0.213433 | 0.643179 | 0.09646 | -0.3341 | -0.28743 | -1.64063 | -0.78402 | -1.65095 | -1.57948 | -1.65752 | -3.87848 | -2.77864 | -2.8387 | -1.49873 | -2.15856 | -2.77732 | -2.09049 |
| Q9D115 | Q9D115 | ZN706_MC | Znf706 | Zinc finger protein 706 | | + | | 0.211615 | 0.986172 | 0.591197 | 1.009184 | 1.970177 | 2.956259 | 0.037207 | 0.063557 | 0.116508 | -3.43447 | -0.38675 | -0.67484 | -0.42925 | -1.89562 | -0.80879 | -2.21827 | -3.5753 | -1.86719 | -3.17725 | -1.46359 | -1.36793 |
| P56873 | P56873 | ZNRD2_MC | Znrd2 | Protein ZNRD2 | + | + | | 1.050466 | 1.216398 | 3.123382 | 1.235698 | 1.298518 | 3.965059 | 0.337403 | -0.62639 | -0.90774 | 0.718677 | -0.05999 | 1.296561 | 1.890836 | -1.30244 | -0.01032 | -0.87753 | -0.89658 | 0.157487 | 0.432872 | -0.27243 | 0.212461 |
| Q9R020 | Q9R020 | ZRAB2_MC | Zranb2 | Zinc finger Ran-binding domain-containing protein 2 | | | | 0.240406 | 0.314742 | 0.664178 | 0.343979 | 0.172908 | 0.924272 | 0.309178 | -0.31837 | -0.83692 | 0.095272 | 0.440071 | 0.334378 | 0.609934 | -0.52134 | -0.82442 | -0.48848 | 0.394339 | 0.425795 | 0.1727 | -0.70343 | -0.0056 |
| Q3UHH1 | Q3UHH1 | ZSWM8_M | Zswim8 | Zinc finger SWIM domain-containing protein 8 | | | | 0.292287 | 0.700315 | 0.79458 | 0.315439 | 0.414632 | 0.852621 | 0.334955 | -0.64667 | -0.90159 | -3.25076 | -1.58584 | -0.46073 | 0.119866 | -1.60313 | -1.20626 | -3.40812 | -2.82911 | -0.97521 | -0.19491 | -1.44728 | -0.06876 |
| Q9CQU5 | Q9CQU5 | ZWINT_MC | Zwint | ZW10 interactor | | | | 0.001256 | -0.00683 | -0.00408 | 0.315391 | -0.24423 | -0.8525 | 0.200652 | 0.871024 | 0.563213 | -1.34763 | -4.6847 | -0.30246 | -0.87258 | -0.86322 | -1.30169 | -2.89383 | -3.12484 | -0.31477 | -0.37181 | -3.48439 | -0.42257 |
| Q3UFS0 | Q3UFS0 | ZY11B_MC | Zyg11b | Protein zyg-11 homolog B | + | | | 1.185055 | 1.498059 | 3.718827 | 0.503434 | 0.478633 | 1.33432 | 0.067718 | -0.09394 | -0.20634 | -1.81843 | -1.82318 | -1.3803 | -1.0622 | -0.97071 | -1.7976 | -3.72169 | -2.91604 | -2.02141 | -1.37836 | -1.48085 | -1.09957 |
| Q5SSH7 | Q5SSH7 | ZZEF1_MO | Zzef1 | Zinc finger ZZ-type and EF-hand domain-containing protein 1 | | | | 0.092187 | -0.09528 | -0.27556 | 0.02766 | 0.188844 | 0.087428 | 0.301446 | 1.156523 | 0.81754 | -3.2386 | -2.54844 | -0.17353 | -3.15492 | -0.18649 | -1.2028 | -2.77636 | -2.82012 | -3.4162 | -0.28994 | -0.53095 | -3.17139 |
| Q3UE31 | Q3UE31 | K0930_MC | N/A | Uncharacterized protein KIAA0930 homolog | | | | 0.420851 | -0.5988 | -1.11915 | 0.077244 | -0.11616 | -0.23355 | 0.391676 | -0.5481 | -1.04474 | -3.35878 | -2.29118 | -1.55791 | -1.6046 | -2.38638 | -1.38809 | -2.18959 | -2.26276 | -1.96189 | -0.96831 | -1.50065 | -1.17762 |
| Q6PIU9 | Q6PIU9 | YJ005_MO | N/A | Uncharacterized protein FLJ45252 homolog | + | | | 1.172829 | 0.983335 | 3.661301 | 0.691613 | 0.714402 | 1.863497 | 0.022404 | -0.03478 | -0.07119 | 5.174616 | 5.111645 | 4.449445 | 4.908706 | 3.811373 | 4.768854 | 3.893072 | 4.426519 | 3.657689 | 4.271658 | 4.227828 | 4.421952 |
| Q8C3W1 | Q8C3W1 | CA198_MC | N/A | Uncharacterized protein C1orf198 homolog | | | | 0.14825 | 0.089377 | 0.42724 | 0.013399 | -0.01872 | -0.04298 | 0.963704 | -0.82819 | -2.77974 | -1.05576 | -1.32773 | -0.92221 | -0.8567 | -1.83847 | -1.38563 | -1.44009 | -1.12215 | -0.43655 | -1.30492 | -0.97751 | -0.59021 |
| Q8C708 | Q8C708 | CP054_MC | N/A | Transmembrane protein C16orf54 homolog | | | | 0.945117 | 1.61537 | 2.709787 | 0.236597 | 1.312311 | 0.654557 | 0.478278 | 1.938202 | 1.267945 | -2.0402 | -0.99591 | 0.234163 | -3.77509 | -0.69649 | -0.02399 | -3.42105 | -2.8458 | -3.0505 | -3.11505 | -0.80727 | -3.78962 |
| Q8R092 | Q8R092 | CA043_MC | N/A | Protein C1orf43 homolog | | | + | 0.259058 | 0.745674 | 0.71117 | 0.449812 | 0.562016 | 1.193757 | 1.189308 | -0.97676 | -3.73901 | -2.6915 | -1.98554 | -0.09108 | 0.312428 | -2.1088 | -1.61094 | -2.09688 | -4.07151 | -0.87672 | -0.02596 | -0.96234 | -0.80387 |
| Q8VC23 | Q8VC23 | CK086_MC | N/A | Uncharacterized protein C11orf86 homolog | | | | 0.31164 | -1.50168 | -0.84309 | 0.393968 | -2.12318 | -1.05056 | 0.324215 | -1.65062 | -0.87463 | -3.20291 | -3.85957 | -3.49038 | -3.45754 | -3.54242 | 0.211498 | -0.27893 | -3.78019 | 0.670159 | -3.37171 | 0.181708 | -0.21138 |
| P08207 | P08207 | S10AA_MC | S100a10 | Protein S100-A10 | + | | | 1.910117 | -3.85341 | -8.93353 | 0.180604 | -0.76392 | -0.51167 | NaN | 0 | NaN | -3.51893 | -3.29023 | -3.17884 | -2.64308 | -2.91359 | -2.71632 | 0.864738 | 0.03292 | -3.61579 | -0.6783 | -3.73816 | -2.64969 |
| Q9DCT1 | Q9DCT1 | AKCL2_MC | Akr1e2 | 1,5-anhydro-D-fructose reductase | + | | | 2.209677 | -4.16361 | -12.6713 | 0.234849 | -0.95302 | -0.65014 | 0.213524 | -0.84551 | -0.59606 | -4.05047 | -3.45312 | -0.63791 | -3.31384 | -2.9977 | -0.33888 | 0.548786 | 0.274848 | -0.424 | -1.62171 | -0.32796 | -1.31759 |
| Q9CYI0 | Q9CYI0 | NJMU_MO | N/A | Protein Njmu-R1 | | | | NaN | 0 | NaN | 0.068096 | -0.23525 | -0.20743 | 0.403754 | 1.661976 | 1.075464 | -3.67046 | -3.44455 | -1.06765 | -3.0943 | -0.91233 | -1.76186 | -3.43629 | -3.74244 | -2.355 | -1.33646 | -4.4849 | -1.51323 |
